# Supplementary material for: Safety, efficacy, and distal nerve Schwann cell biodistribution in mice and NHPs to support translation of AAV9 RNAi therapy for CMT1A
Source: Mol Ther Nucleic Acids. 2026 Feb 27;37(2):102881. doi: 10.1016/j.omtn.2026.102881 (PMC13051718; doi:10.1016/j.omtn.2026.102881)
Supplement: Document S1. Figures S1–S17, S19–S22, S24–S26, S28, and Tables S1–S4 [file mmc1.pdf]

OMTN, Volume *37*

Supplemental information

Safety, efficacy, and distal nerve Schwann cell biodistribution in mice and NHPs to support translation of AAV9 RNAi therapy for CMT1A

Marina Stavrou, Lindsay M. Wallace, Merlin P. Thangaraj, Noah K. Taylor, Alexia Kagiava, Revekka Papacharalambous, Cynthia McAllister, Gloria Zender, Nizar Y. Saad, M. Bilal Bayazit, Amanda Heslegrave, Christina Tryfonos, Jan Richter, Henrik Zetterberg, Brian Price, Rachel Salzman, Kleopas A. Kleopa, and Scott Q. Harper

Table S1. Decreased transcripts in miR871-treated human cells.

≥2-fold change; FDR<0.05	≥1.75-fold change; FDR<0.05	
**Neurons**	**Neurons**	
PMP22[a]	PMP22[a]	
**Hepatocytes**	**Hepatocytes**	
ACTC1	ACTC1	ACTN2
ANKRD18B	ANKRD18B	ALPK3
MYBPC3	MYBPC3	ENSG00000223401[b]
MYH7	MYH7	ENSG00000268230
MYL2	MYL2	LDB3
MYL4	MYL4	MYH6
MYL7	MYL7	MYOM1
NEFM	NEFM	NOXA1
NPPA	NPPA	PMP22[a]
TNNI1	TNNI1	TNNC1
TNNT2	TNNT2	ZMYND10[b]
**Schwann cells**	**Schwann cells**	
RGS5	RGS5	ENSG00000268592
STMN2	STMN2	PMP22[a]

FDR, False Discovery Rate. [a]*PMP22* was the only significantly reduced transcript in every cell line treated with miR871, demonstrating on-target specificity. [b]*ENSG000000223401* and *ZMYND10* were detected in Schwann cells and hepatocytes only, while all other transcripts listed were present in each cell line.

Table S2. Relative average reductions of human PMP22 and mouse Pmp22 in CMT1A mice injected with 5E11 dose of AAV9.U6.miR871

	**Human PMP22**		**Mouse PMP22**	
**PNS tissue**	**RNA**	**Protein**	**RNA**	**Protein**
Lumbar roots	54%	76%	48%	61%
Sciatic nerve	67%	73%	63%	40%
Femoral nerve	66%	68%	66%	42%

Table S3. First-in-Human Dose Calculations for lumbar intrathecal delivery of AAV9.miR871

Dose		Human equivalent dose[c]	Expected volume and rate for human IT delivery	Dose description
Mouse[a]	NHP[b]			
1E11	––	3.7E14	---	––
2E11	6E13	7.4E14	10 – 15 mL / delivered in ~30 min	MED, planned clinical starting dose
5E11	1.2E14	1.9E15	20 – 30 mL / delivered in ~1 hr	FED, planned clinical high dose
1E12	––	3.7E15	---	––

NHP, non-human primate; IT, intrathecal; MED, minimum effective dose; FED, fully efficacious dose. [a] all mouse doses delivered in 20 microliters volume; [b] NHP doses delivered in 3 milliliters volume over a 6 hour period. [c] calculated based on cerebrospinal fluid volume

Table S4. 12-week GLP single dose biodistribution and safety study groups

Group	Target Dose (Total vg)	Target Dose Volume (mL)	Number of Animals			
			6-Week Cohort[a]		12-Week Cohort[b]	
			Male	Female	Male	Female
1 – Vehicle	0	3	1	1	1	1
2 – Low	6×10^{13}	3	2	2	2	2
3 – High	1.2×10^{14}	3	2	2	2	2

Abbreviation: vg = vector genomes.
[a] Necropsy Day 43.
[b] Necropsy Day 85.

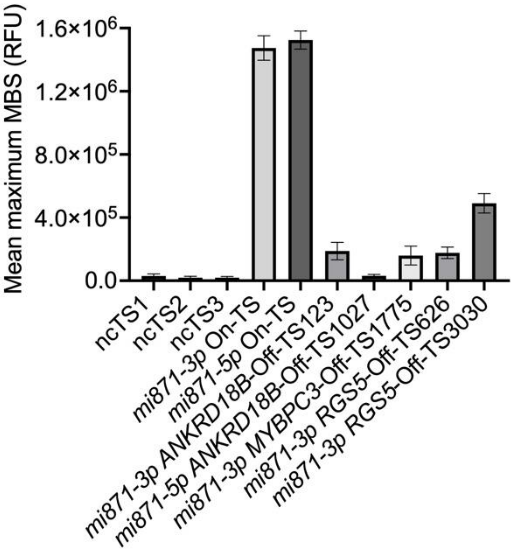

<table>
<tr><th></th><th>K_d (µM)</th><th>B_{max} (RFU)</th><th>Base pairing</th></tr>
<tr><td>mi871-3p On-TS</td><td>0.18</td><td>1495838</td><td>On-TS 5′ GGGGATTGCTGTTGATTGAAGA 3′
||||||||||||||||||||||
mi871-3p 3′ CCCCTAACGACAACTAACTTCT 5′</td></tr>
<tr><td>mi871-5p On-TS</td><td>0.06</td><td>1533775</td><td>On-TS 5′ AGTCTTCAATCAACAGCAACCC 3′
||||||||||||||||||||||
mi871-5p 3′ TCAGAAGTTAGTTGTCGTTGGG 5′</td></tr>
<tr><td>mi871-3p ANKRD18B -Off-TS123</td><td>14</td><td>281946</td><td>Off-TS 5′ G U C GG GGAG U 3′
GG GGAU GCUG UGA GAAGA
|| |||| |||| ||| |||||
CC CCUA CGAC ACU CUUCU
mi871-3p 3′ A A AA 5′</td></tr>
<tr><td>mi871-5p ANKRD18B -Off-TS1027</td><td>12.2</td><td>45347</td><td>Off-TS 5′ A AAUGACAA AGAA A 3′
UCUUCAA UCA ACAGCAACC
||||||| ||| |||||||||
AGAAGUU AGU UGUCGUUGG
mi871-5p 3′ UC G 5′</td></tr>
<tr><td>mi871-3p MYBPC3 -Off-TS1775</td><td>31.7</td><td>318579</td><td>Off-TS 5′ C G C A 3′
GGGG UGU UGA UGAAGA
|||| ||| ||| ||||||
CCCC ACA ACU ACUUCU
mi871-3p 3′ UA ACA A 5′</td></tr>
<tr><td>mi871-3p RGS5 -Off-TS626</td><td>5.2</td><td>215960</td><td>Off-TS 5′ U C GAG ACAA U 3′
GGAUUGC UGU GAUU GAAGA
||||||| ||| |||| |||||
CCUAACG ACA CUAA CUUCU
mi871-3p 3′ CC A 5′</td></tr>
<tr><td>mi871-3p RGS5 -Off-TS3030</td><td>1.5</td><td>531202</td><td>Off-TS 5′ A A A C 3′
GGAUUG UGUU UGAAGA
|||||| |||| ||||||
CCUAAC ACAA ACUUCU
mi871-3p 3′ CC G CUA 5′</td></tr>
</table>

Figure S1. Molecular Beacon-Binding Assay off-target gene assessment. (Top) The graph shows binding data for mi871-3p and mi871-5p target sites within the *PMP22* on-target transcript and RNA-seq identified off-target transcripts (*ANKRD18B*, *MYBPC3* and *RGS5*) that showed binding above background. Data are displayed as means with error bars representing SD. (Bottom) The molecular beacon signal expressed in relative fluorescence units (RFU) was subtracted from background fluorescent signal and fit to a one site-specific binding equation used to determine the binding affinity (K_d), which is the binding site concentration (μM) required to reach half of maximum fluorescence. The K_d represents the binding affinity of miRNA to its target site. The smaller the K_d value, the greater the binding affinity of the miRNA molecular beacon is for its target site. In the base pairing of the single stranded (ss) mi871 DNA sequence with the ssTS DNA sequence, the guanosine nucleotides (G) that were facing a thymidine (T) were replaced by adenosines (A) (indicated in gray) to mimic the G:U base pairing that occurs in RNA:RNA duplexes. miRNA:off-target site pairs are represented as two annealing strands. The top strand represents the target site, and the bottom strand represents the mature mi871 sequence. ncTS1: MB871-3pWT vs ncTS; ncTS2: MB871-3p-MYBPC3-1775 vs ncTS; ncTS3: MB871-5pWT vs ncTS. MBS: Molecular Beacon Signal.

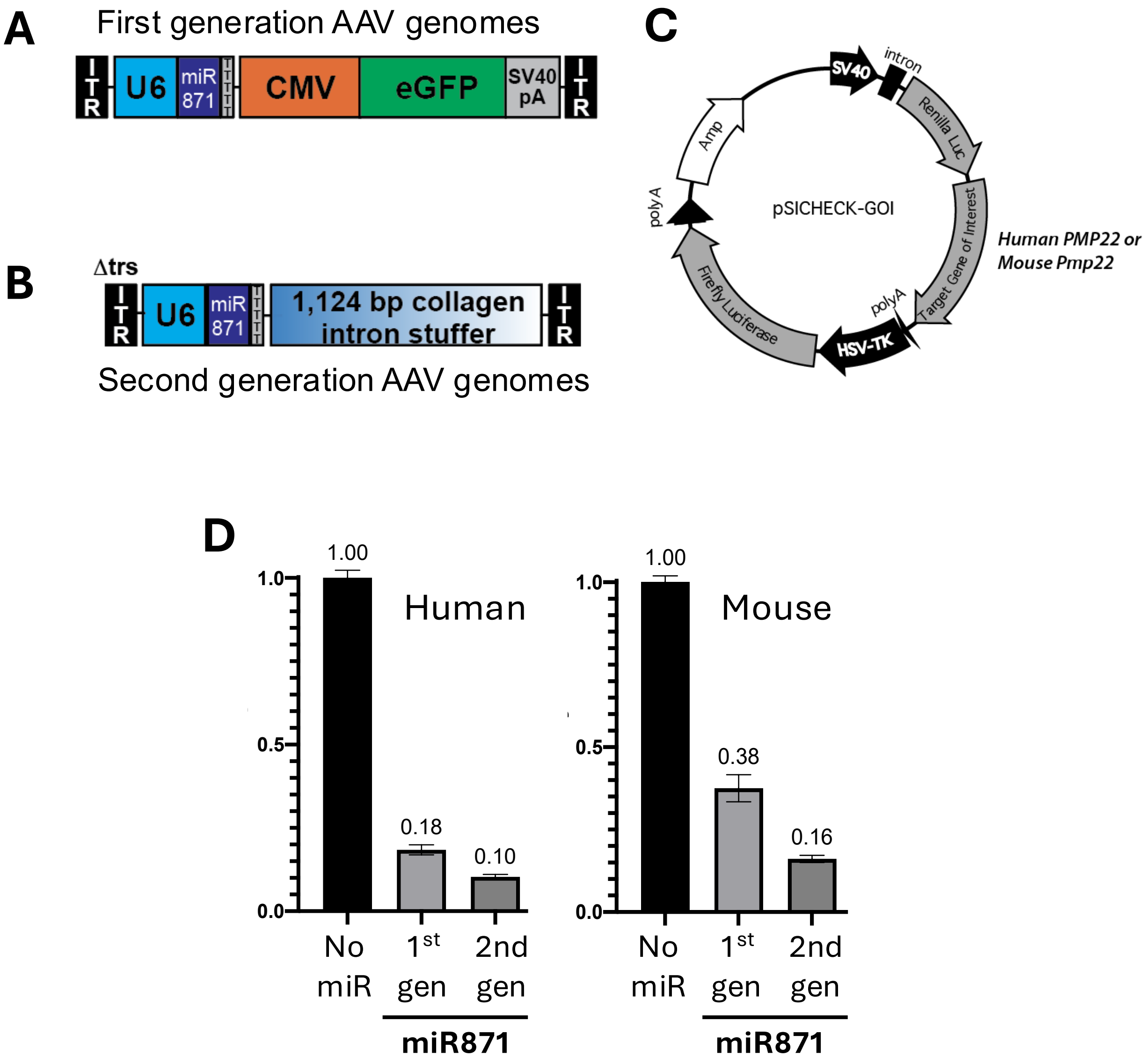

Figure S2. In vitro potency comparison of first- and second-generation miR871 vector genomes. (A) First-generation self-complementary adeno-associated viral (AAV) vector contained a U6 promoter-driven miR871 with T6 terminator and a separate cassette expressing eGFP from the cytomegalovirus (CMV) promoter. Black boxes, AAV inverted terminal repeats (ITRs); gray "T" box, miRNA terminator; SV40 (pA) polyadenylation signal. (B) In the second-generation self-complementary vectors eGFP was replaced with a 1,124 bp segment from the human collagen intron serving as a stuffer sequence to aid in packaging. Black boxes, AAV inverted terminal repeats; Δtrs, self-complementary AAV inverted terminal repeat containing a deletion in the terminal resolution site (trs); gray "T" box, miR terminator. (C) Dual luciferase reporter plasmid (pSICHECK) used for *in vitro* screening of miR871 potency targeting the human *PMP22 and* mouse *Pmp22* cDNAs as the target gene of interest (GOI). The SV40 promoter drives expression of a *Renilla* luciferase (*Renilla* Luc) with respective GOIs cloned as the 3' UTR. Firefly luciferase is driven by the thymidine kinase promoter (TK) and serves as an internal transfection control. (D) Second generation (2nd gen) miR871 vector showed increased gene silencing compared to the first generation (1st gen) vector determined by measuring the ratio of Renilla to firefly luciferase. Data are displayed as means with error bars representing SD (N=3 independent experiments performed in triplicate).

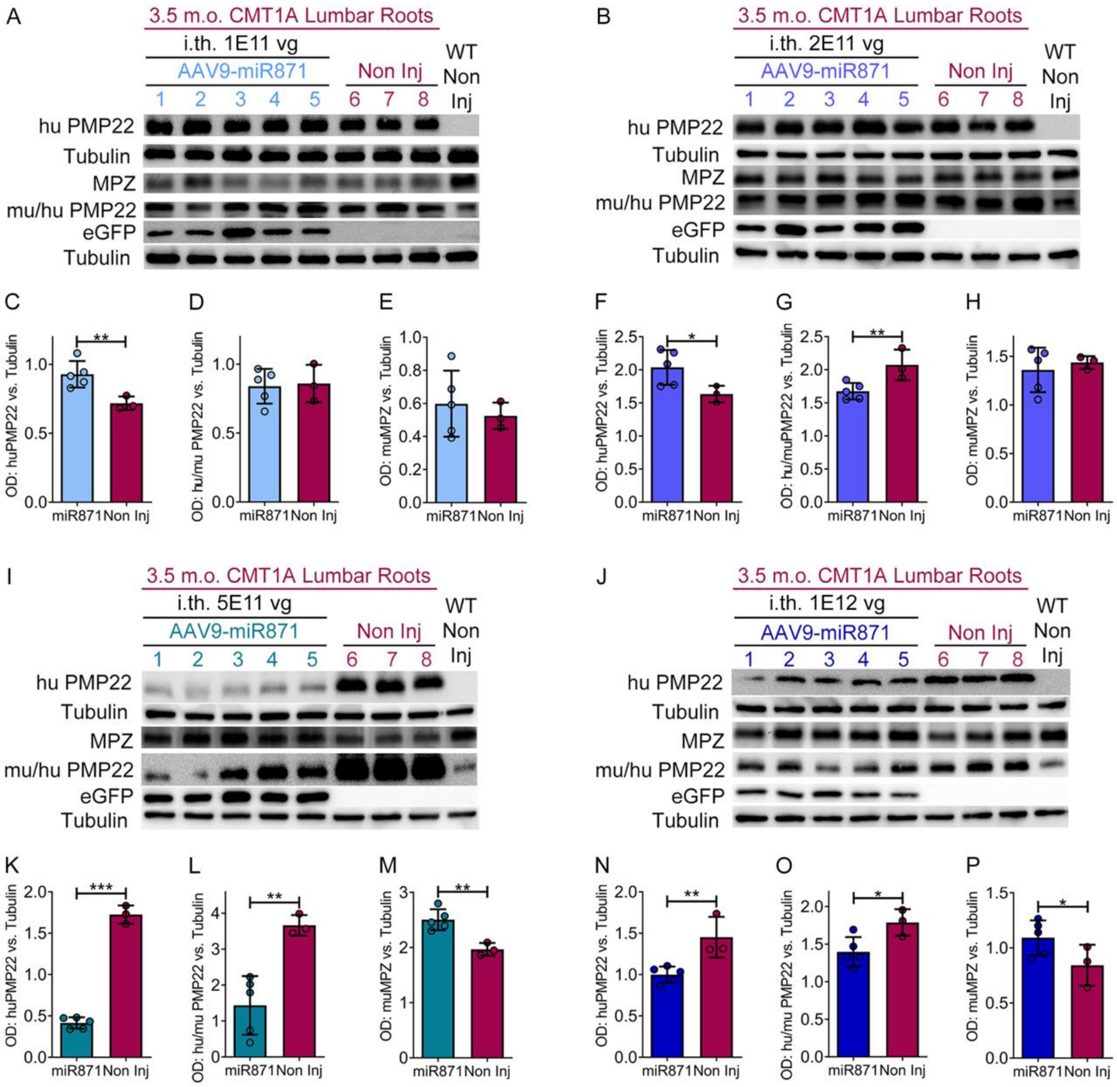

Figure S3. Western blot analysis of spinal nerve root protein lysates from CMT1A mice treated with different AAV9.U6.miR871 doses.

Western blot analysis of lumbar root lysates from CMT1A mice injected at 2 months of age with AAV9.U6.miR871 at doses of 1E11, 2E11, 5E11, or 1E12 vg/animal (n=5/group) and analysed 6 weeks later (3.5 months of age), compared to age-matched non-injected CMT1A controls (n=3). Representative western blot images showing huPMP22, muPMP22, muTubulin, EGFP and muMPZ protein expression and quantification in lumbar roots of CMT1A animals treated with different AAV9.U6.miR871 doses; 1e11 (A,C-E), 2e11 (B, F-H), 5e11 (I, K-M) or 1e12 (J, N-P) vg/animal. Values are presented as mean ± SD. *P < 0.05, **P < 0.01, ***P < 0.001, by Student's t-test.

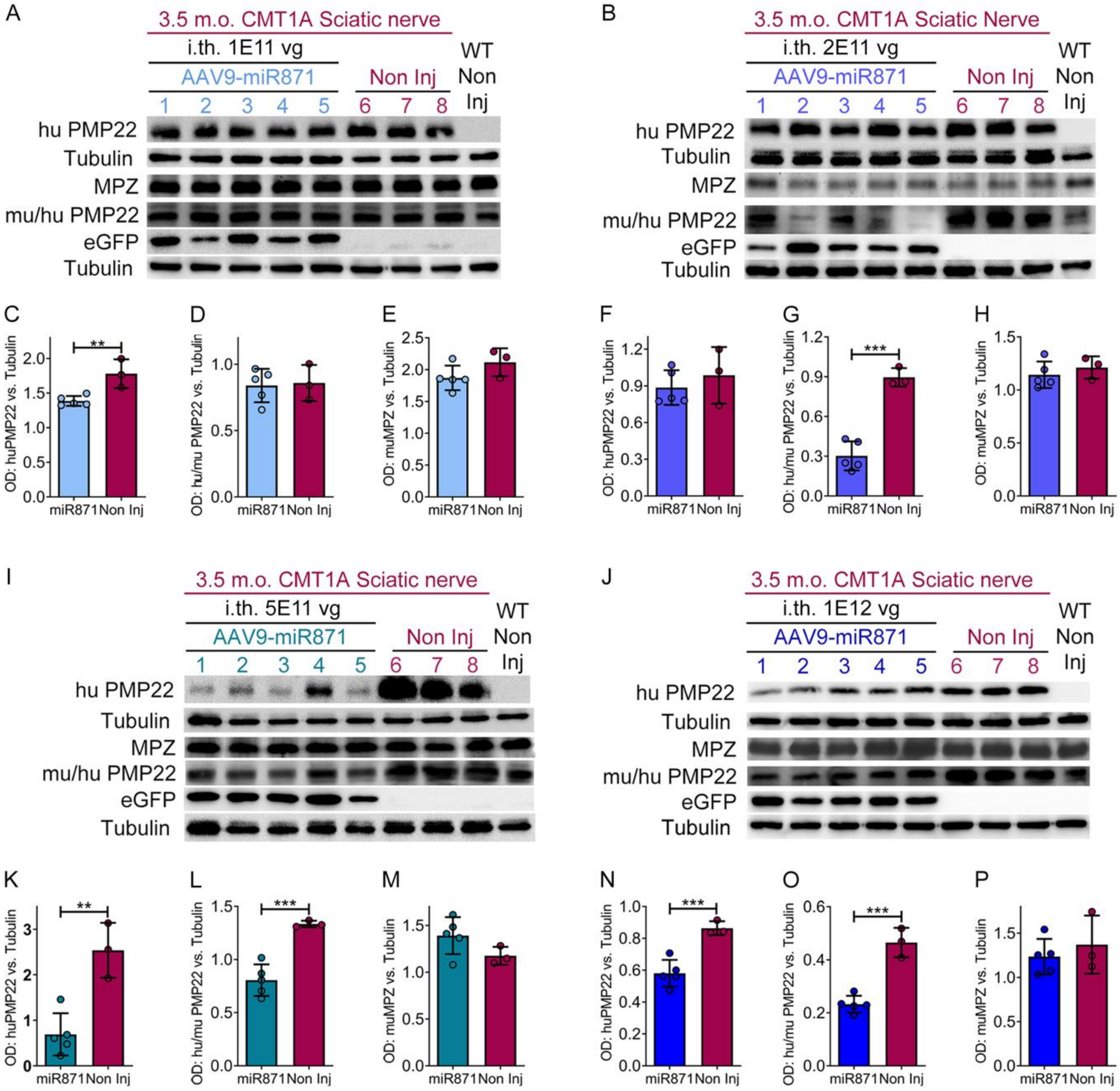

Figure S4. Western blot analysis of sciatic nerve protein lysates from CMT1A mice treated with different AAV9.U6.miR871 doses.

Western blot analysis of sciatic nerve lysates from CMT1A mice injected at 2 months of age with AAV9.U6.miR871 at doses of 1E11, 2E11, 5E11, or 1E12 vg/animal (n=5/group) and analysed 6 weeks post-injection (3.5 months of age), compared to age-matched non-injected CMT1A controls (n=3). Representative western blot images showing huPMP22, muPMP22, muTubulin, EGFP and muMPZ protein expression and quantification in sciatic nerves of CMT1A animals treated with different AAV9.U6.miR871 doses; 1e11 (**A,C-E**), 2e11 (**B, F-H**), 5e11 (**I, K-M**) or 1e12 (**J, N-P**) vg/animal. Values are presented as mean ± SD. *P < 0.05, **P < 0.01, ***P < 0.001, by Student’s test.

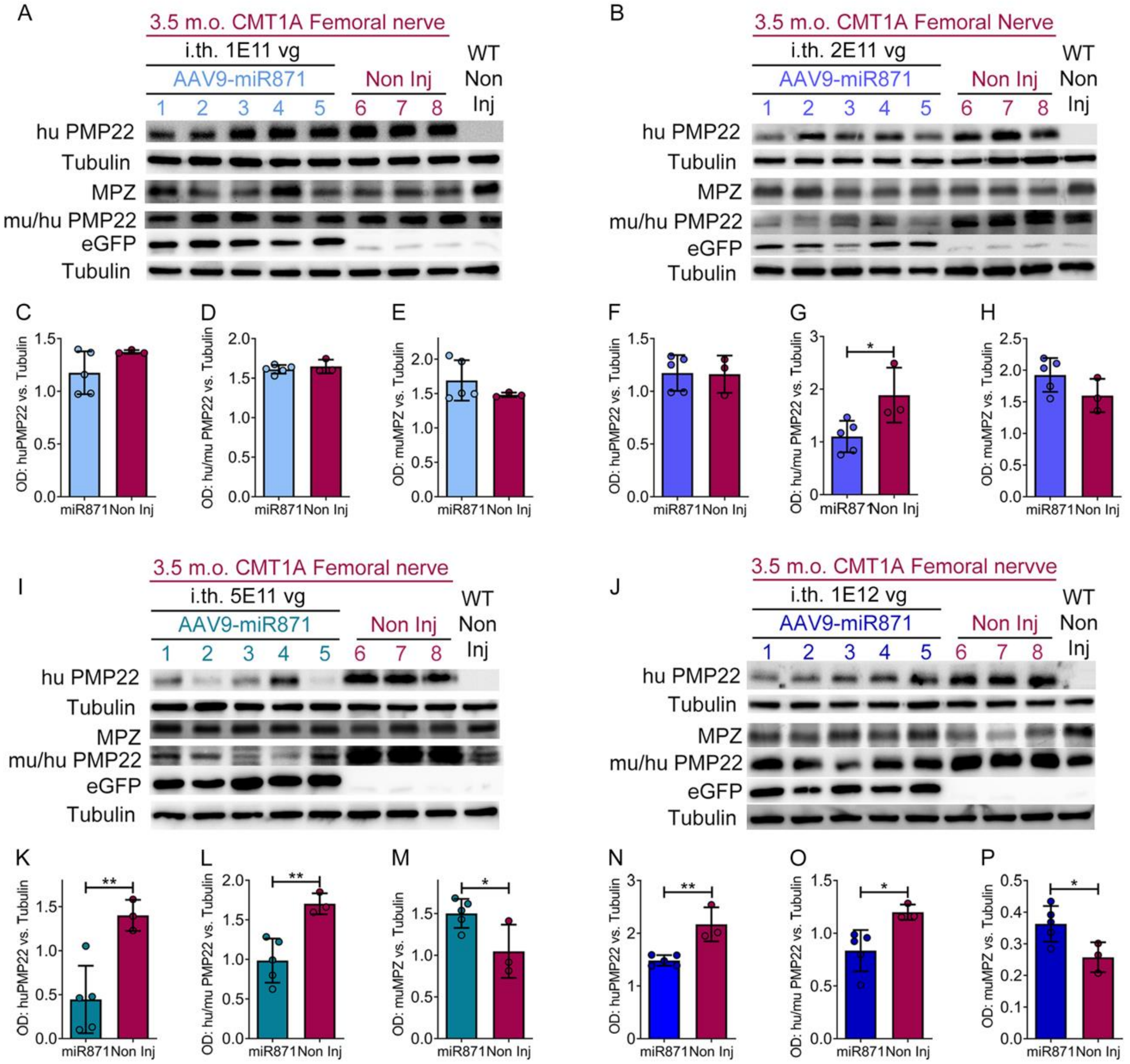

Figure S5. Western blot analysis of femoral nerves lysates in CMT1A mice treated with different AAV9.U6.miR871 doses.

Western blot analysis of femoral nerve lysates from CMT1A mice injected at 2 months of age with AAV9.U6.miR871 at doses of 1E11, 2E11, 5E11, or 1E12 vg/animal (n=5/group) and analysed 6 weeks post-injection (3.5 months of age), compared to age-matched non-injected CMT1A controls (n=3). Representative western blot images showing huPMP22, muPMP22, muTubulin, EGFP and muMPZ protein expression and quantification in femoral nerves of CMT1A animals treated with different AAV9.U6.miR871 doses; 1e11 (**A,C-E**), 2e11 (**B, F-H**), 5e11 (**I, K-M**) or 1e12 (**J, N-P**) vg/animal. Values are presented as mean ± SD. *P < 0.05, **P < 0.01, by Student's test.

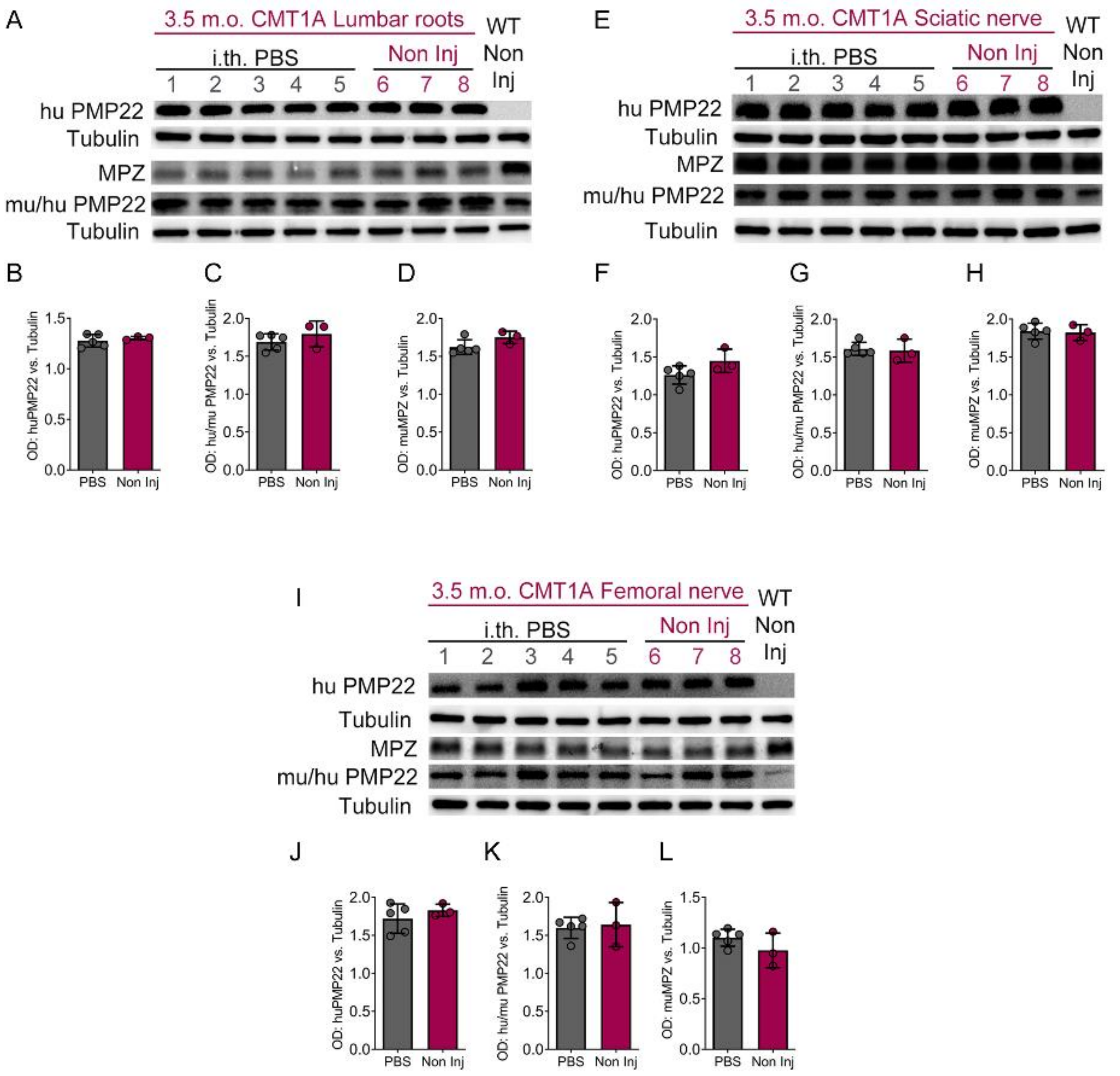

Figure S6. Western blot analysis of PNS tissues lysates in CMT1A mice injected with PBS. Western blot analysis was performed on lumbar roots (**A**), sciatic nerves (**E**) and femoral nerves (**I**) lysates from CMT1A mice injected with PBS at 2 months of age (n=5/group) and analysed 6 weeks post-injection (3.5 months of age), compared to age-matched non-injected CMT1A controls (n=3). Representative western blot images and quantification of huPMP22, muPMP22, muTubulin, EGFP and muMPZ protein expression in lumbar roots (**B-D**), sciatic nerves (**F-H**) and femoral nerves (**J-L**). Values are presented as mean ± SD and compared by unpaired Student's test.

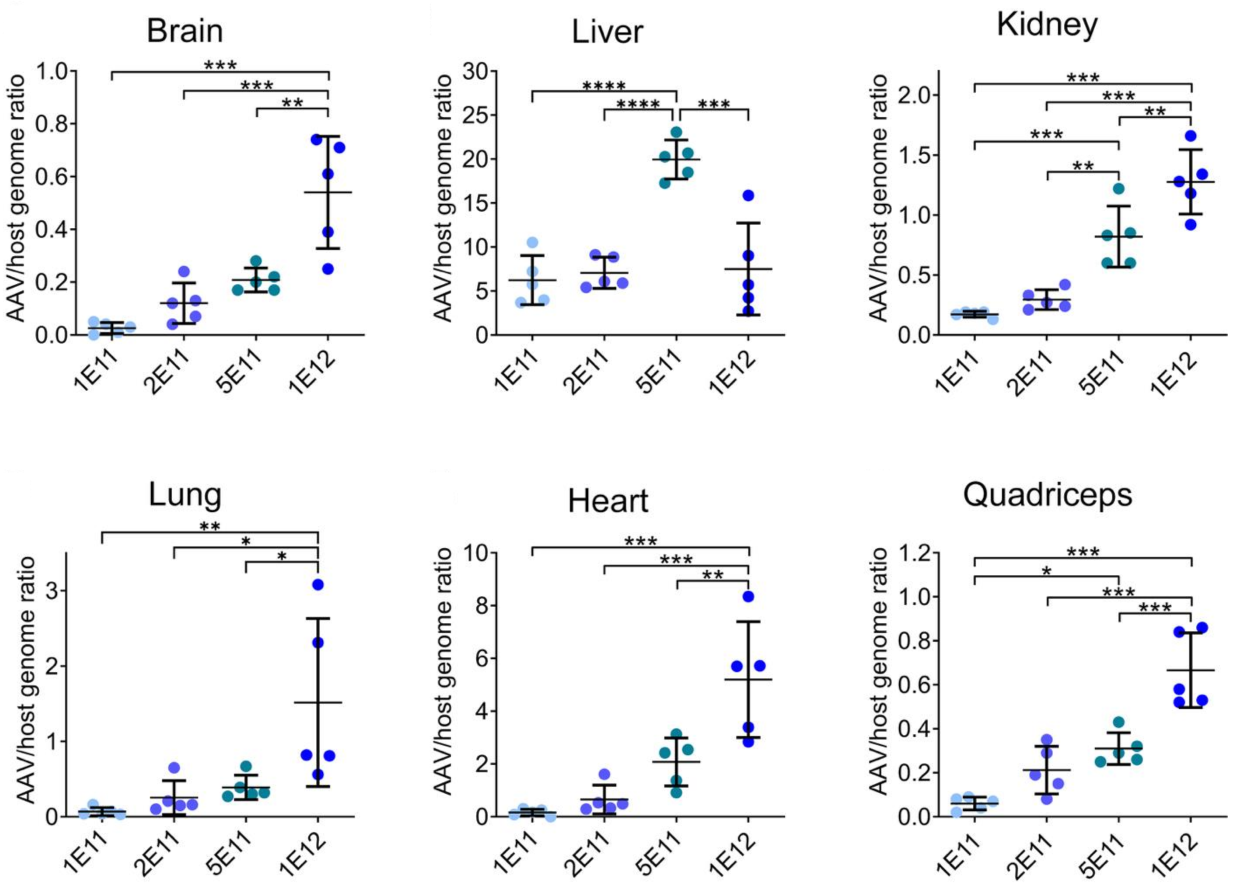

Figure S7. Vector genome copy number (VGCN) analysis in non-PNS tissues of CMT1A mice treated with different AAV9.U6.miR871 doses.

VGCN was quantified in DNA extracted from the brain, liver, kidney, lung, heart and quadriceps of CMT1A mice injected at 2-months of age with AAV9.U6.miR871 at doses of 1E11, 2E11, 5E11, or 1E12 vg/animal and analysed 6-weeks later (n=5/group). Values are presented as mean ± SD. *P < 0.05, **P < 0.01, ***P < 0.001, ****P ≤ 0.0001 by 1-way ANOVA with Tukey's multiple-comparison test.

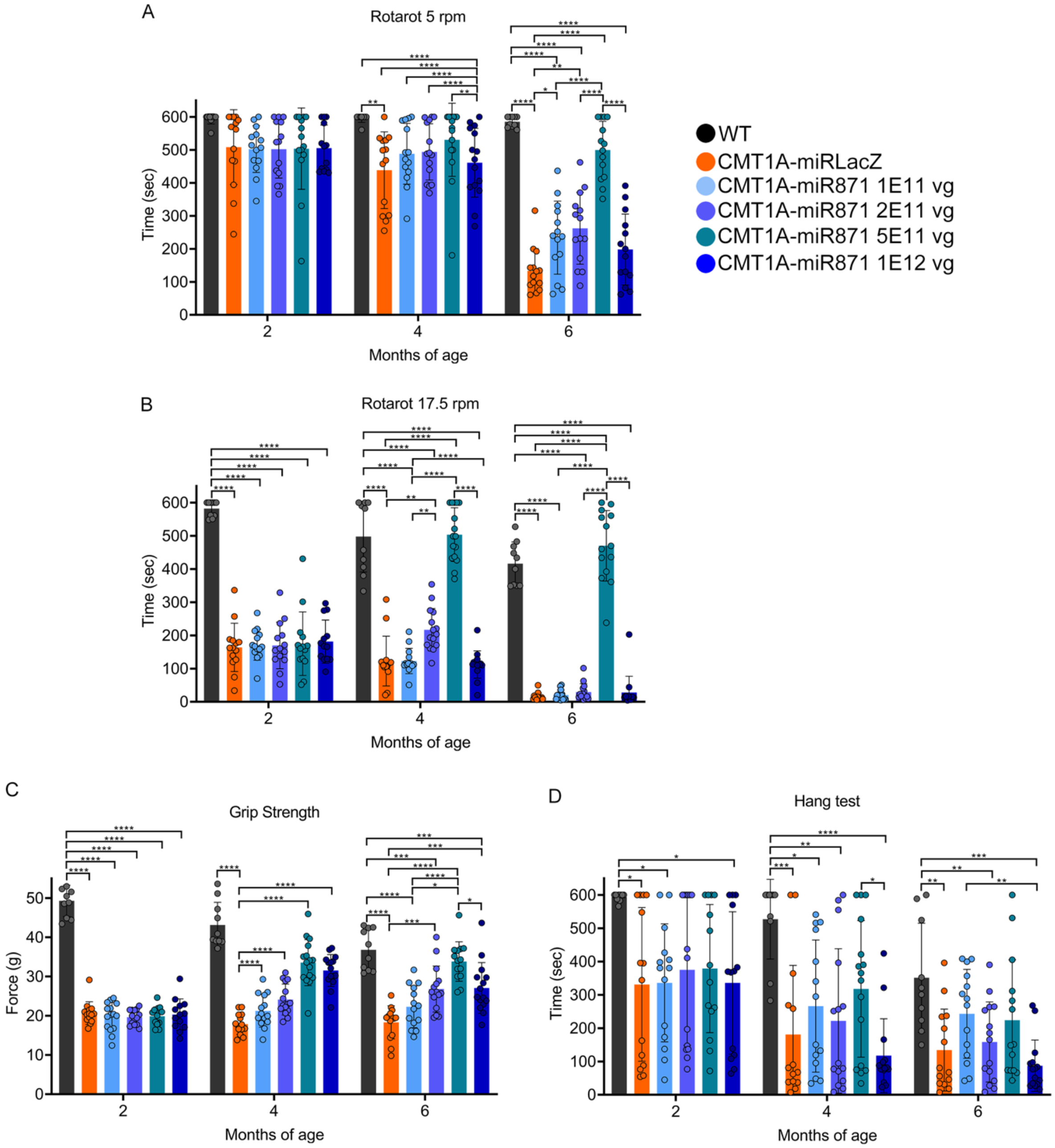

Figure S8. Behavioral analysis of CMT1A mice following treatment with different AAV9.U6.miR871 doses. (A-D) Behavioral assessments, including rotarod at 5 and 17.5 rpm, grip strength and hang test, were conducted in WT mice (rotarod, grip strength, and hang test: n = 10; hind limb abduction: n = 6) and CMT1A mice treated with different doses of AAV9.U6.miR871 (1E11, 2E11, 5E11, or 1E12 vg/animal) or AAV9.U6.miRLacZ (n = 14/group). Behavioral tests were performed before treatment (2 months of age) and monitored every 2 months until 6 months of age. Values are presented as mean ± SD. Statistical significance was determined using one-way ANOVA with Tukey's multiple-comparison test (*P < 0.05, **P < 0.01, ***P < 0.001, ****P ≤ 0.0001), with comparisons made within the same age group.

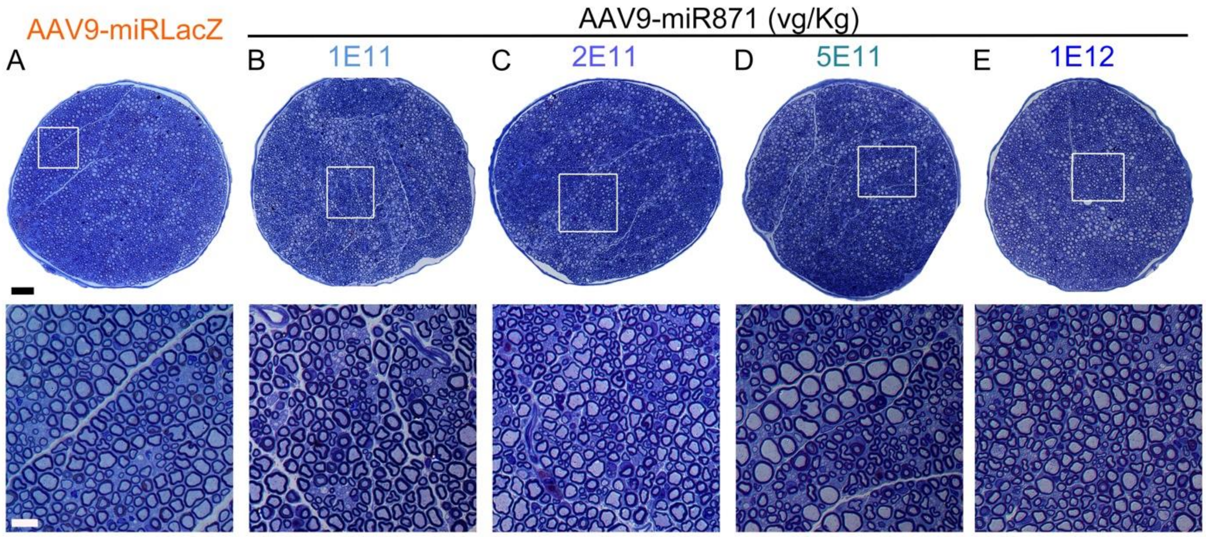

Figure S9. Representative images of toluidine blue-stained semithin sections of sciatic nerves from CMT1A mice following treatment with different AAV9.U6.miR871 doses.

(A-E) Toluidine blue-stained semithin sections of mid-sciatic nerves from CMT1A animals treated with AAV9.U6.miRLacZ or AAV9.U6.miR871 at doses of 1E11, 2E11, 5E11, or 1E12 vg/animal are shown at low (upper panels) and high magnifications (lower panels; n=5/group). Scale bars: 40 μm and 10 μm (enlarged inserts).

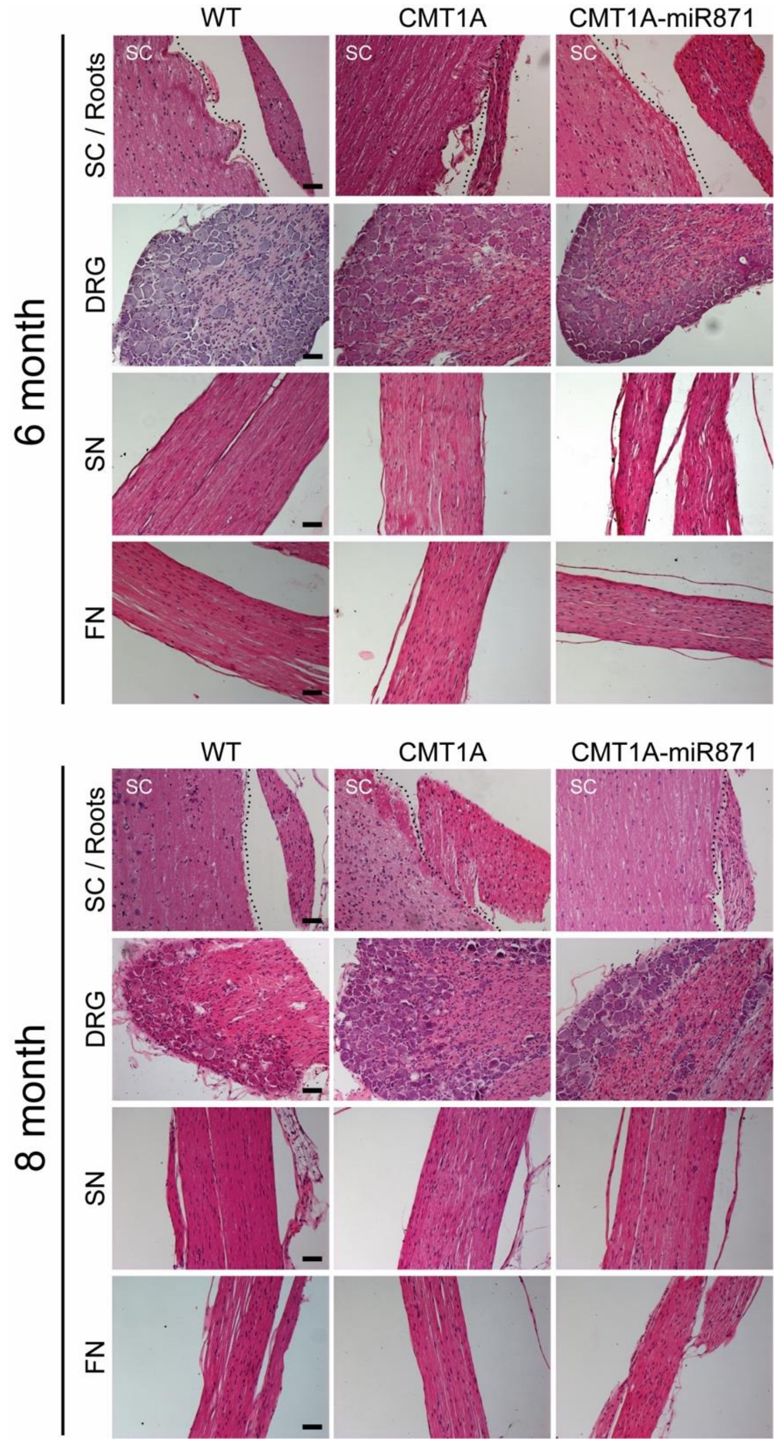

Figure S10. Representative H&E-stained images of nerve tissues from CMT1A mice following treatment with different AAV9.U6.miR871 doses. H&E staining of spinal cord (SC) and adjoining roots, DRGs, sciatic nerves, and femoral nerves from non-injected wild-type (WT), CMT1A mice, as well as CMT1A mice treated at 2 months of age with AAV9.U6.miR871. Animals were dosed at 1E11, 2E11, 5E11, or 1E12 vg/animal with the 1e12 vg/animal dose pictured here (n=4/group). Tissues were collected and analysed at 6 months (top panels) and 8 months (bottom panels) of age. Scale bar: 50 µm.

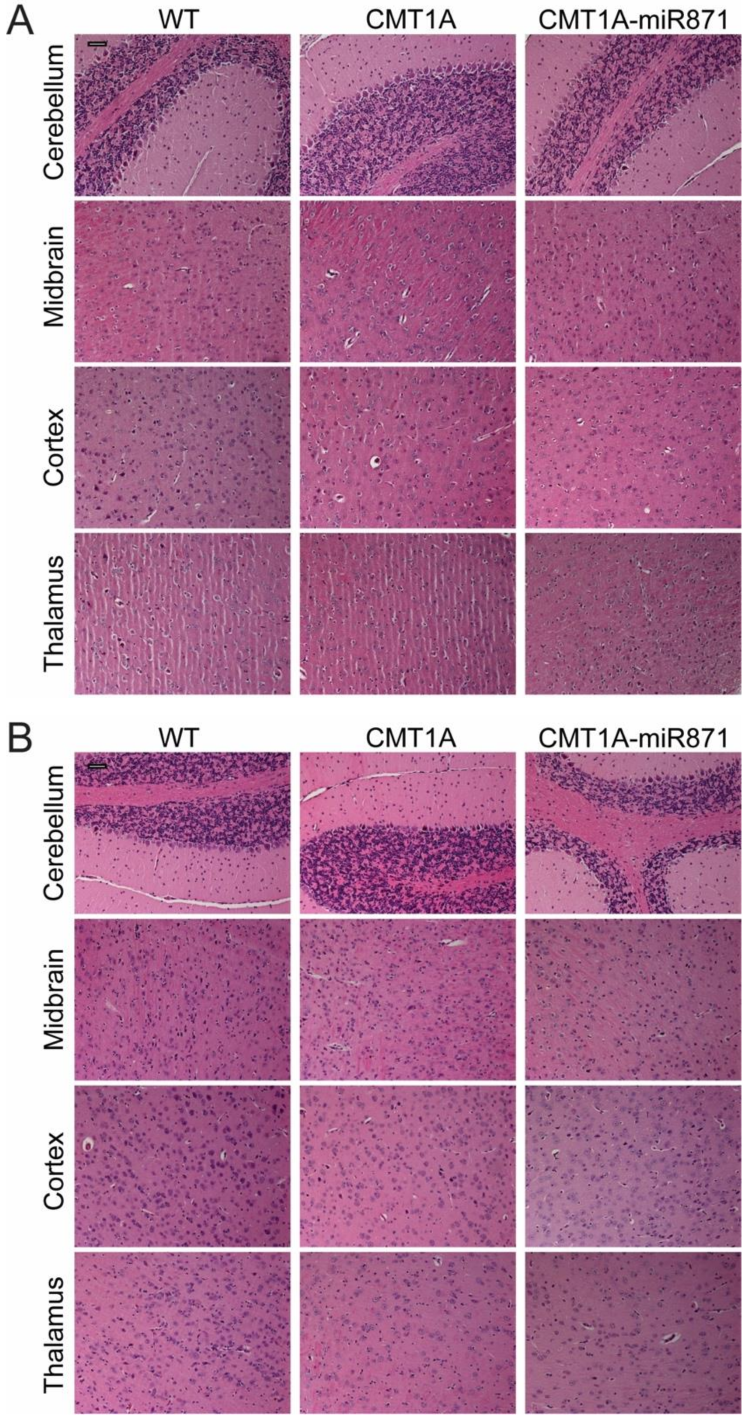

Figure S11. Representative H&E-stained images of brain areas from CMT1A mice following treatment with AAV9.U6.miR871.

H&E staining of cerebellum, midbrain brain, cortex and thalamus areas from un-injected wild-type (WT), CMT1A mice, as well as high dose (1E12 vg) representative images from CMT1A mice treated at 2 months of age as part of the AAV9.U6.miR871 dose response. Doses of 1E11, 2E11, and 5E11 vg/animal not shown (n=4/group). Tissues were collected and analysed at 6 months (A) and 8 months (B) of age. Scale bar: 50 µm.

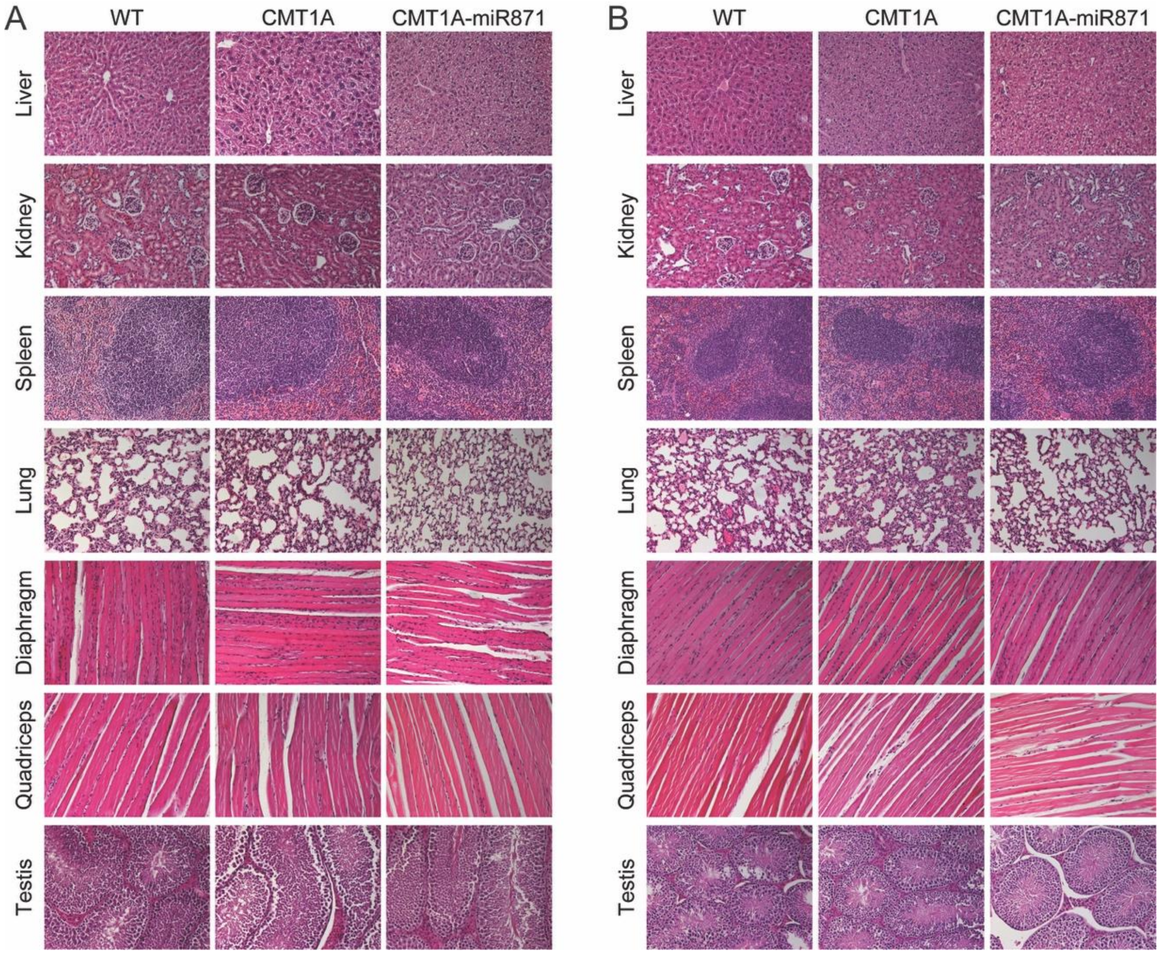

Figure S12. Representative H&E-stained images of off-target tissues from CMT1A mice following treatment with different AAV9.U6.miR871 doses.

H&E staining of organs from non-injected wild-type (WT), CMT1A mice, and CMT1A mice treated at 2 months of age with AAV9.U6.miR871. Dose response (1E11, 2E11, 5E11, or 1E12 vg/animal) represented here with the 1E12 vg dose represented (n=4/group). Tissues were collected and analysed at 6 months (A) and 8 months (B) of age. Scale bar: 50 μm.

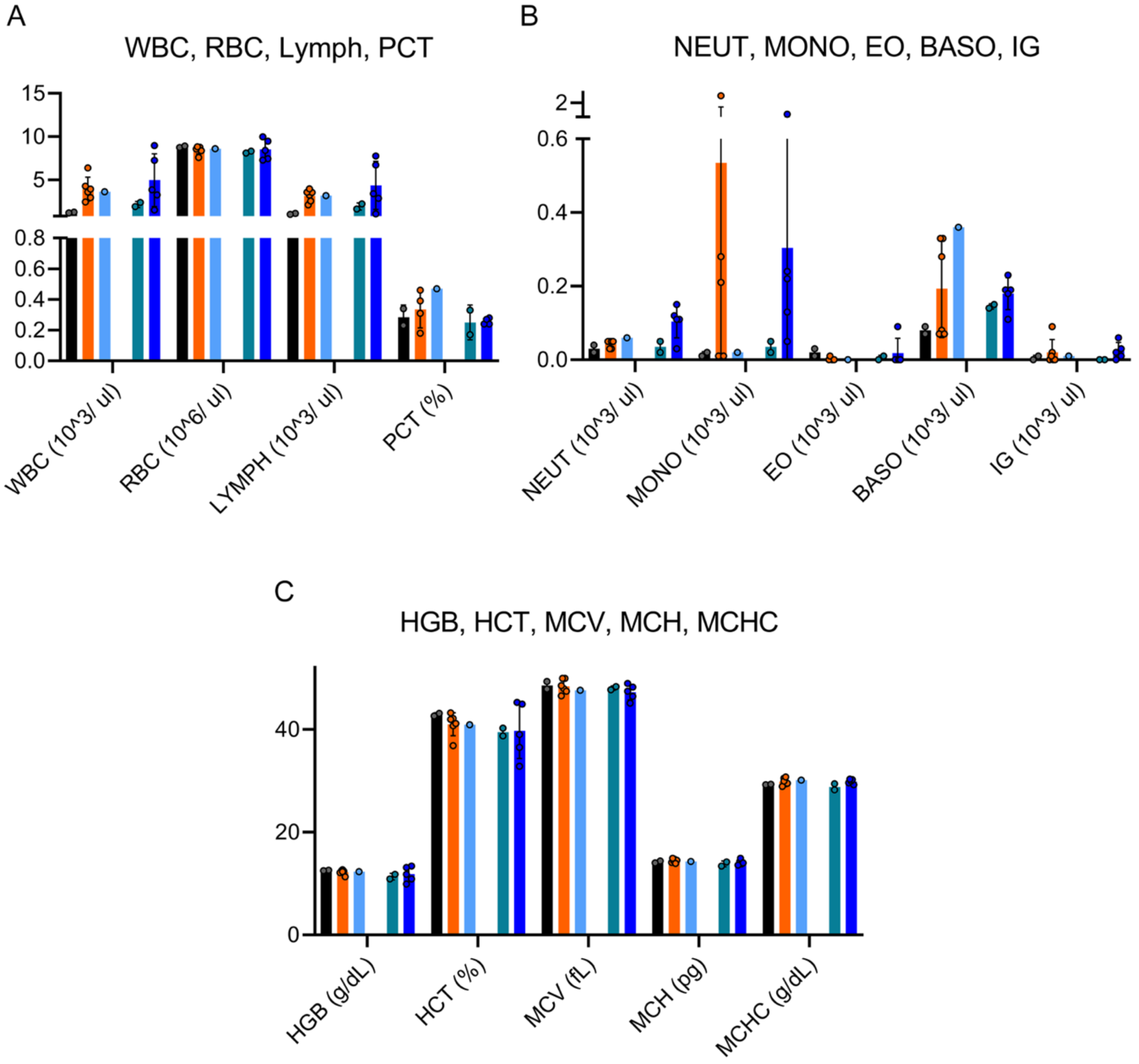

Figure S13. Comprehensive blood analysis of 6-month-old CMT1A mice following treatment with different AAV9.U6.miR871 doses.

Haematological and biochemical parameters were assessed in WT and CMT1A mice treated at 2 months of age with AAV9.U6.miRLacZ or AAV9.U6.miR871 at doses of 1E11, 2E11, 5E11, or 1E12 vg/animal. Blood samples were collected and analysed at 6-months of age (n=6/group). Parameters were analysed following our priority list (See Material and Methods). Blood analysis included (**A**) white blood cell (WBC), red blood cell (RBC), lymphocyte (Lymph) counts, and plateletcrit (PCT); (**B**) neutrophils (NEUT), monocytes (MONO), eosinophils (EO), basophils (BASO), and immature granulocytes (IG); (**C**) hemoglobin (HGB), hematocrit (HCT), mean corpuscular volume (MCV), mean corpuscular hemoglobin (MCH), and mean corpuscular hemoglobin concentration (MCHC).

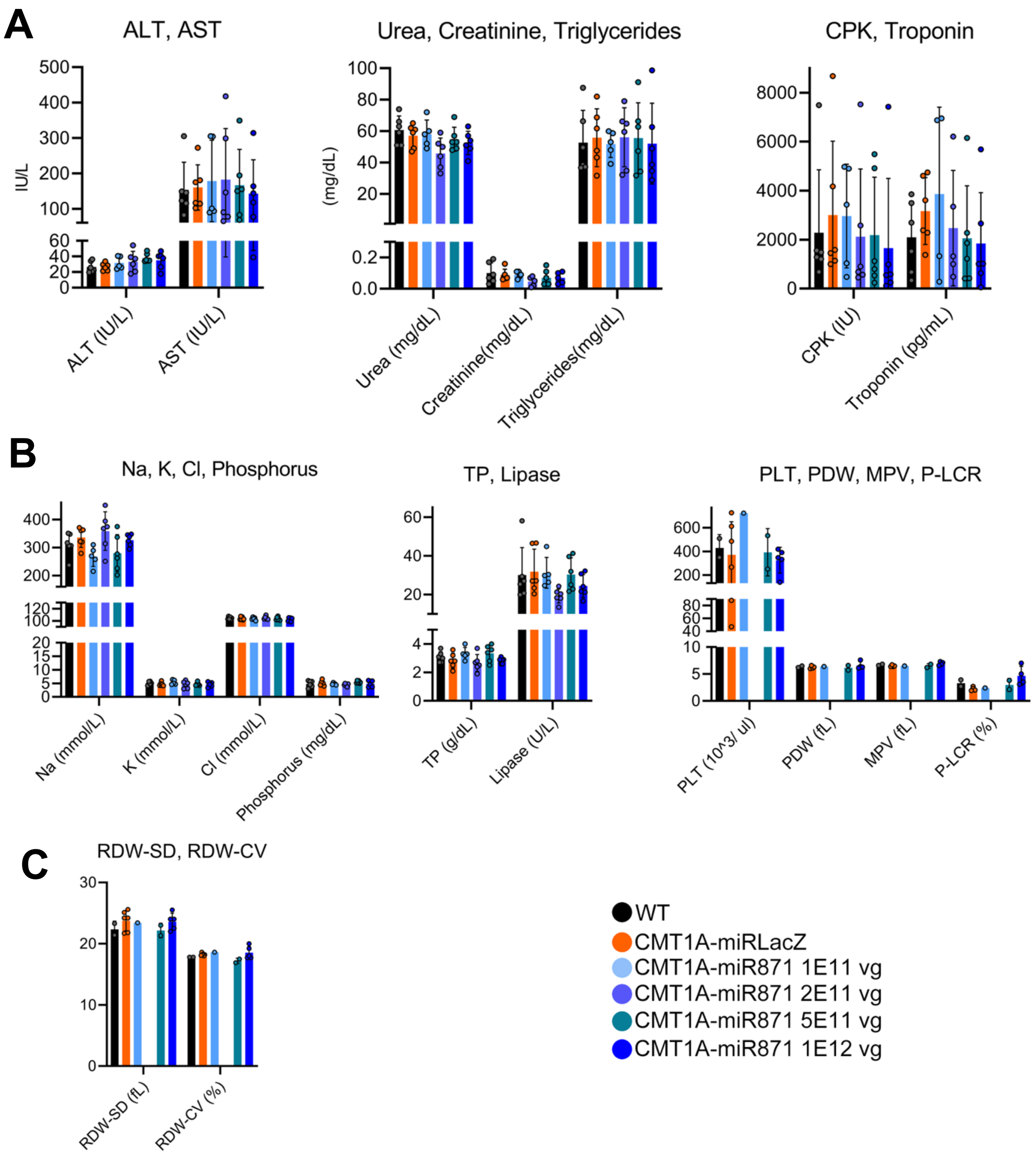

Figure S14. Comprehensive blood analysis of 6-month-old CMT1A mice following treatment with different AAV9.U6.miR871 doses.

(**A**) alanine aminotransferase (ALT) and aspartate aminotransferase (AST); urea, creatinine, and triglyceride levels; creatine phosphokinase (CPK) and troponin; (**B**) sodium (Na), potassium (K), chloride (Cl), and phosphorus (P); total protein (TP) and lipase; platelet count (PLT), platelet distribution width (PDW), mean platelet volume (MPV), and platelet large cell ratio (P-LCR); and (**C**) red cell distribution width-standard deviation (RDW-SD) and red cell distribution width-coefficient of variation (RDW-CV). Values are presented as mean ± SD. Statistical analysis was performed using one-way ANOVA with Tukey's multiple-comparison test, comparisons made within the same age group.

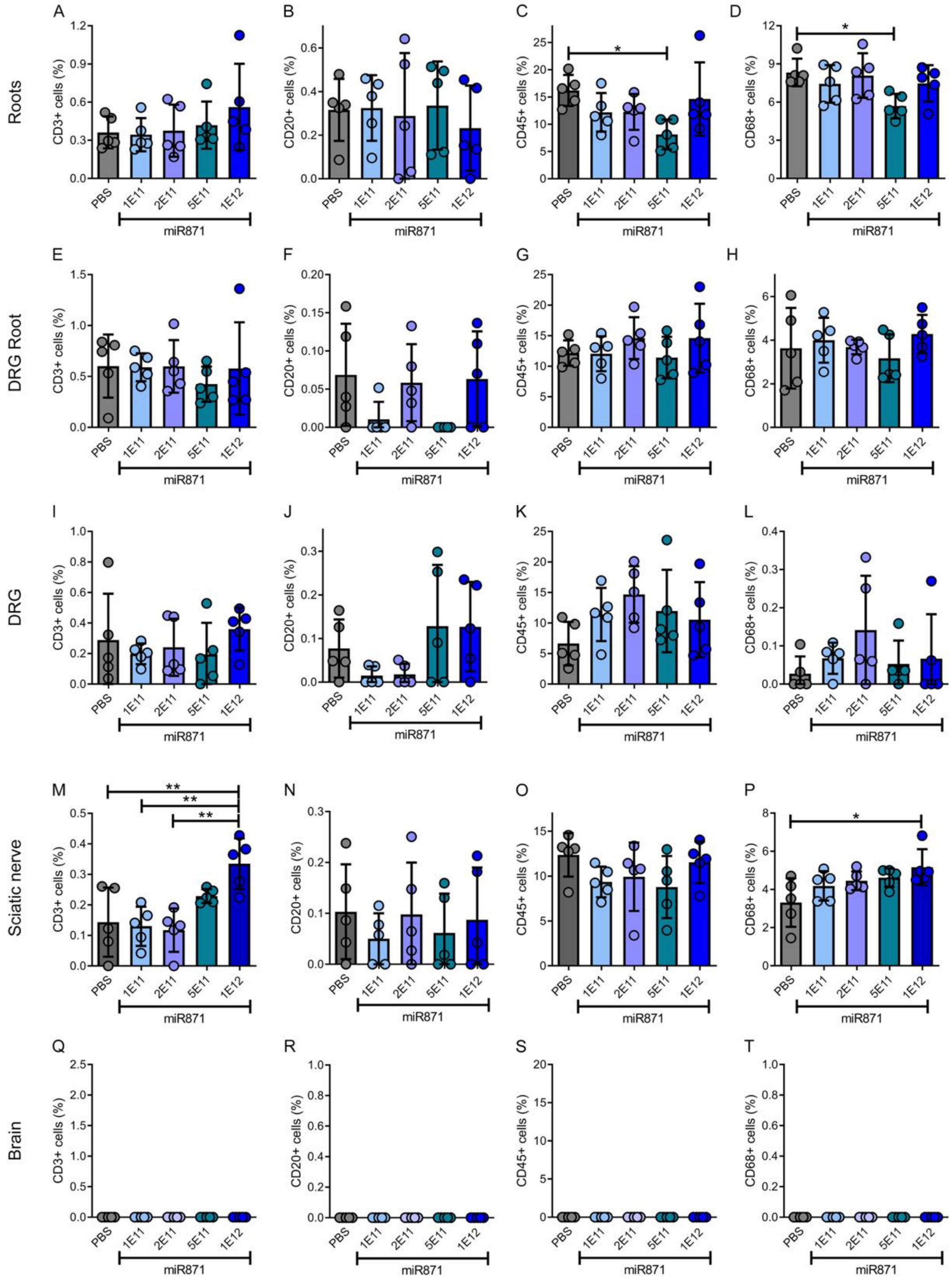

Figure S15. Inflammatory response in neural tissues of CMT1A mice following treatment with different AAV9.U6.miR871 doses.

Quantification of the percentage of cells positive for the CD3 T-cell marker, CD20 B-cell marker, CD45 leukocyte marker, and CD68 macrophage marker in immunostained (**A-D**) spinal roots, (**E-H**) DRG sensory roots, **(I-L)** DRGs, (**M-P**) sciatic nerves, and (**Q-T**) femoral nerves of CMT1A mice treated with indicated AAV9.U6.miR871 doses or PBS (n=5/group). Values are presented as mean ± SD. *P < 0.05, **P < 0.01, by 1-way ANOVA with Tukey's multiple-comparison test.

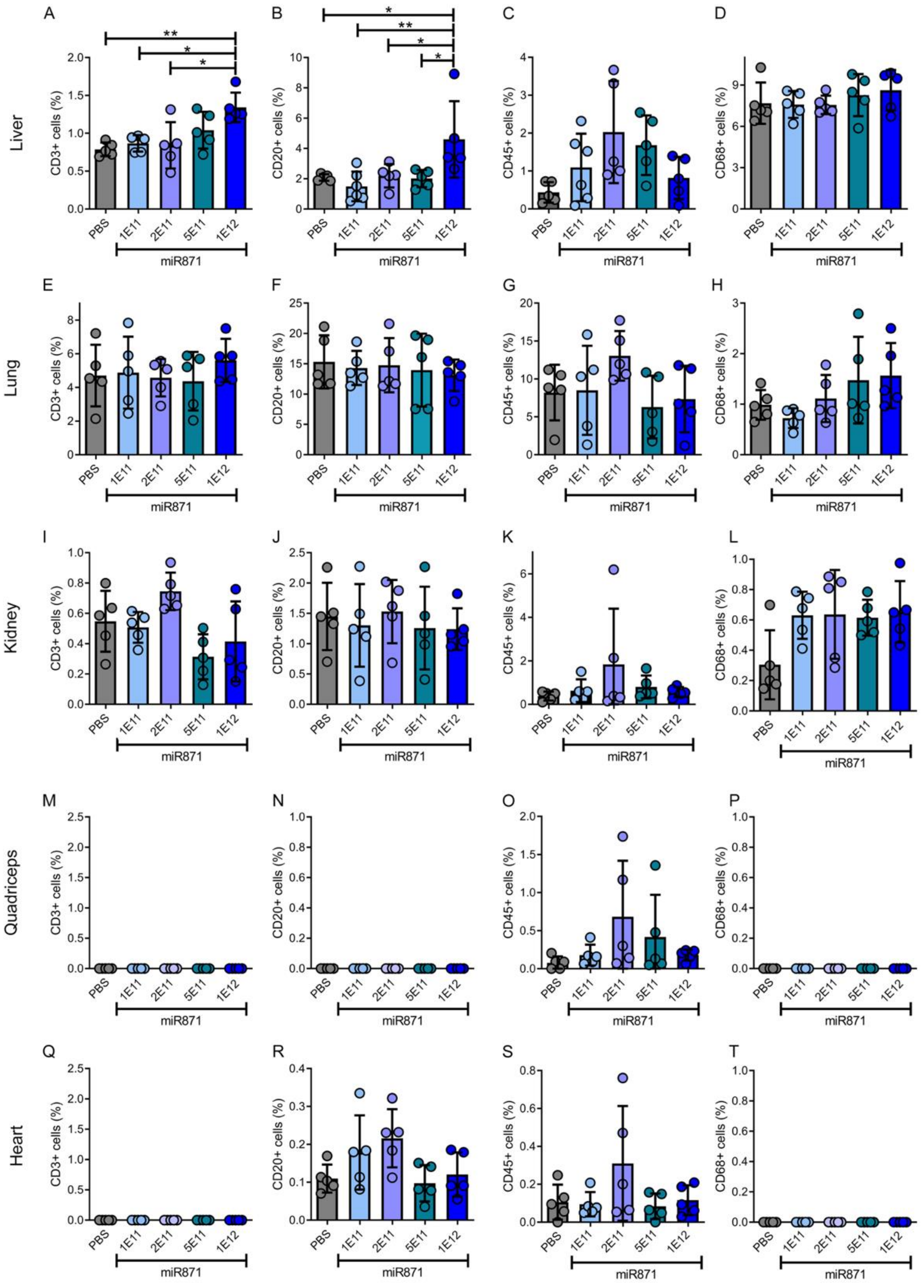

Figure S16. Inflammatory response in peripheral tissues of CMT1A mice following treatment with different AAV9.U6.miR871 doses.

Quantification of the percentage of cells positive for the CD3 T-cell marker, CD20 B-cell marker, CD45 leukocyte marker, and CD68 macrophage marker in immunostained (**A-D**) liver, (**E-H**) lung, (**I-L**) kidneys, (**M-P**) quadriceps and (**Q-T**) heart of CMT1A mice treated with indicated AAV9.U6.miR871 doses or PBS (n=5/group). Values are presented as mean ± SD. *P < 0.05, **P < 0.01, by 1-way ANOVA with Tukey's multiple-comparison test.

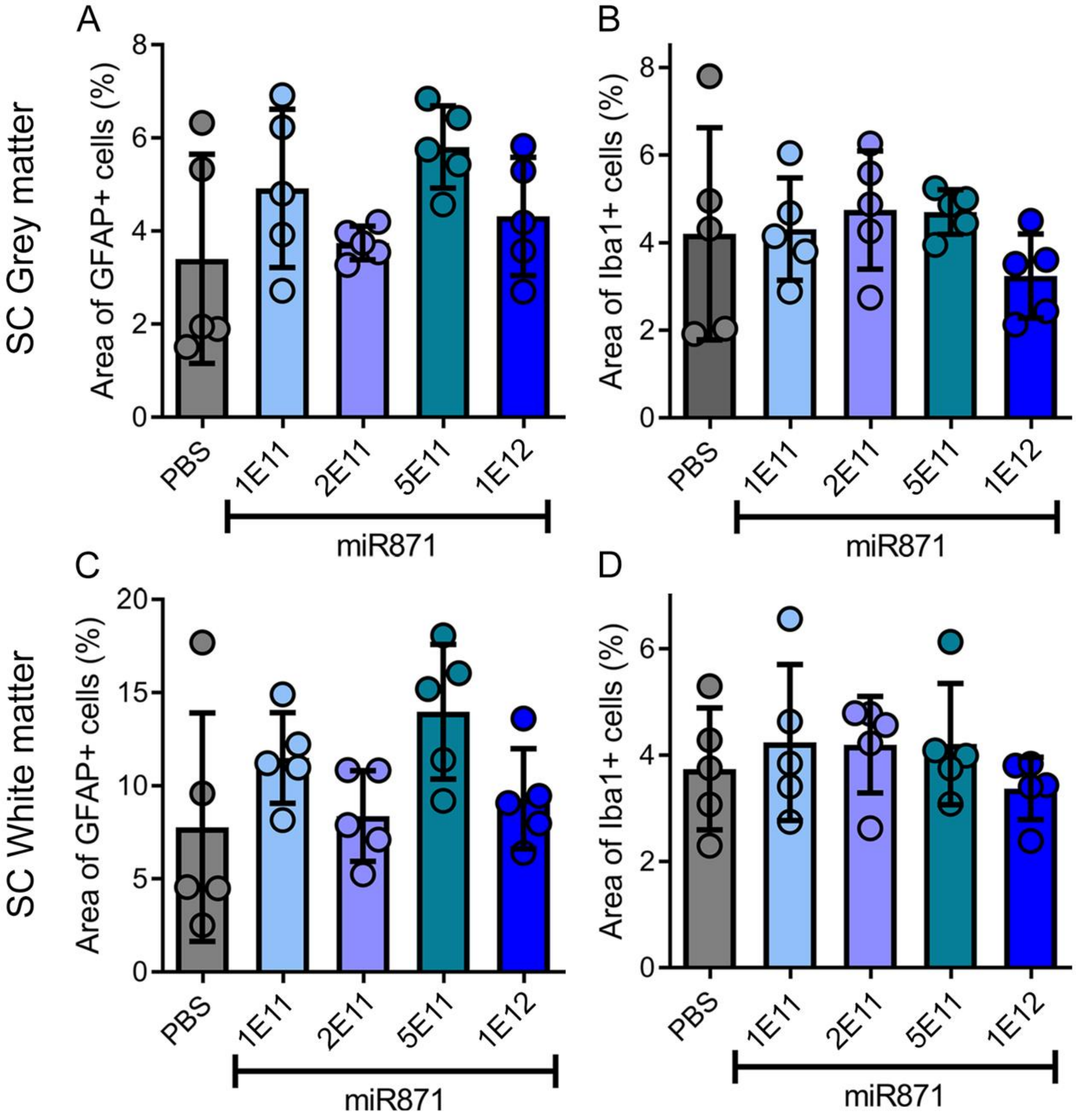

Figure S17. Lack of inflammatory side effects in spinal cord of CMT1A mice following treatment with different AAV9.U6.miR871 doses.

Quantification of the percentage of cells positive for GFAP astrocyte marker and Iba1 microglia marker in the (**A,B**) grey and (**C,D**) white matter of immunostained spinal cord (SC) of CMT1A mice treated with indicated AAV9.U6.miR871 doses or PBS (n=5/group). Values are presented as mean ± SD and compared by 1-way ANOVA with Tukey's multiple-comparison test.

Figure S18. Mouse toxicology report from Transcendent Bio. 4 page pathology report in PDF form, uploaded as a separate supplemental file.

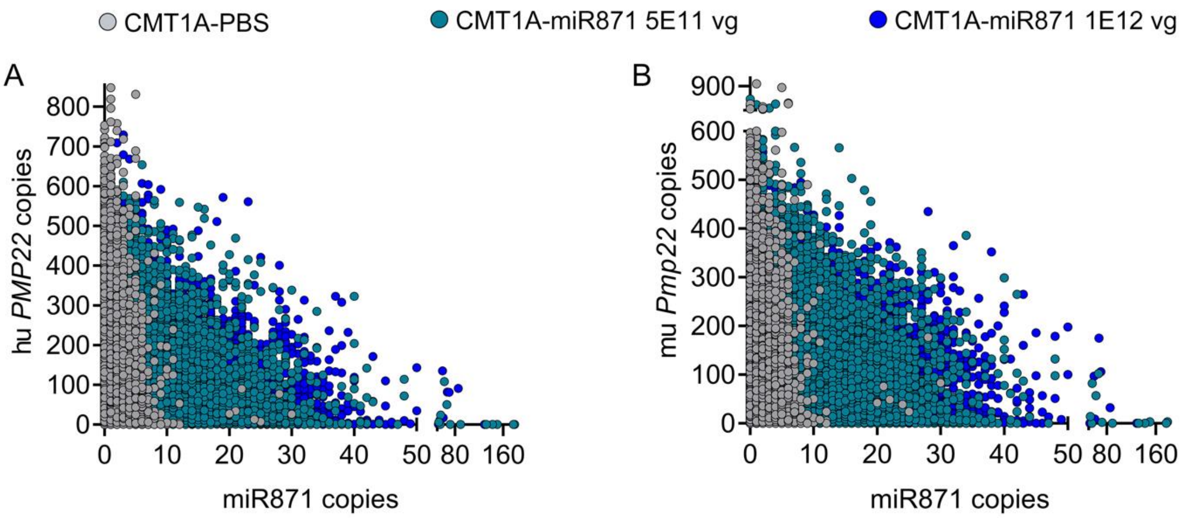

Figure S19. Single cell resolution RNAscope Plus™ analysis of miR871 co-detection with huPMP22 or muPmp22 in the sciatic nerves of CMT1A mice injected with 5E11 or 1E12 vg/animal of AAV9.U6.miR871 or PBS. RNAscope Plus™ analysis was performed to assess the co-localization of miR871 with (**A**) human *PMP22* or (**B**) murine *Pmp22* in the sciatic nerves of CMT1A mice treated with AAV9.U6.miR871 (5E11 or 1E12 vg/animal) or PBS (n=6 animals/group, n≥147,000 cells/group). Quantitative image analysis was conducted to evaluate transcript expression at single-cell resolution. Data points represent individual cells, with PBS-treated animals shown in grey, 5E11 vg/animal in turquoise, and 1E12 vg/animal in blue. Due to overlapping signals, PBS-treated cells appear on top, followed by those receiving 5E11 vg/animal, with 1E12 vg/animal-treated cells at the bottom.

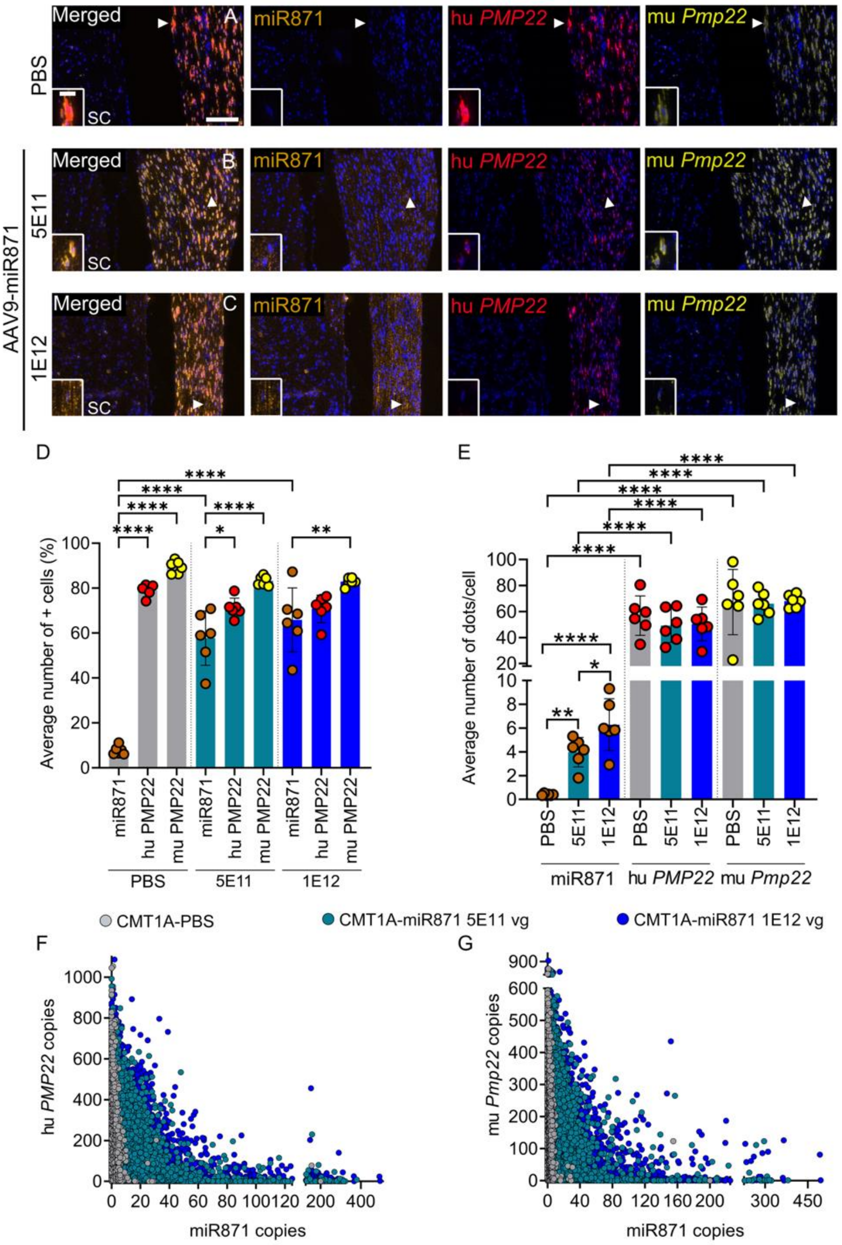

Figure S20. RNAscope Plus™ analysis for the co-detection of miR871, hu*PMP22*, and mu*Pmp22* in the spinal roots of CMT1A mice injected with 5E11 or 1E12 vg/animal of AAV9.U6.miR871 or PBS. (**A-C**) Representative images of miR871, hu*PMP22*, and mu*Pmp22* staining in spinal roots adjoining the spinal cord (SC) of CMT1A mice treated with 5E11 or 1E12 vg/animal AAV9.U6.miR871 (n=6 animals/group, n≥139,000 cells/group). **(D-E)** Quantitative image analysis was performed using HALO® software and whole-tissues scanning used to determine average numbers of miR871-, hu*PMP22*-, and mu*Pmp22*-positive cells as well the average number of corresponding miR871, hu*PMP22*, and mu*Pmp22* dots/cells in CMT1A animals treated with AAV9.U6.miR871 (5E11 or 1E12 vg/animal) or PBS. (**F-G**) Single-cell resolution data of individual cells. Due to overlapping signals, PBS-treated cells appear on top, followed by those receiving 5E11 vg/animal, with 1E12 vg/animal-treated cells at the bottom. For D and E, values are presented as mean ± SD. **P* < 0.05, ***P* < 0.01, ****P* < 0.001, *****P* ≤ 0.0001 by 1-way ANOVA with Tukey's multiple-comparison test. Scale bar: 50 μm and 15 μm (enlarged inserts).

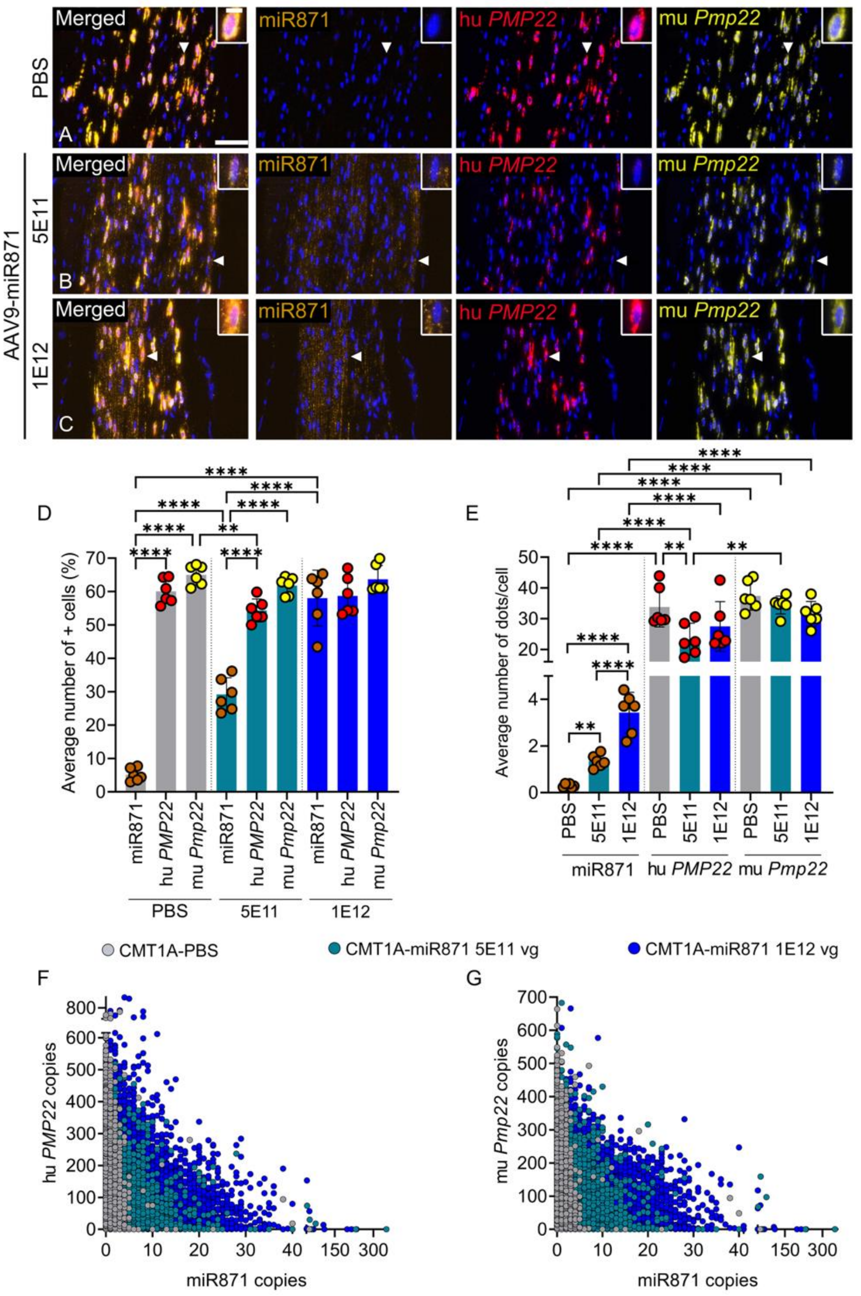

Figure S21. RNAscope Plus^{TM} analysis for the co-detection of miR871, hu*PMP22*, and mu*Pmp22* in the femoral nerves of CMT1A mice injected with 5E11 or 1E12 vg/animal of AAV9.U6.miR871 or PBS. (**A-C**) Representative images of miR871, hu*PMP22*, and mu*Pmp22* staining in femoral nerves of CMT1A mice treated with 5E11 or 1E12 vg/animal of AAV9.U6.miR871 (n=6 animals/group, n≥42,900 cells/group). (**D-E**), Quantitative image analysis was performed using HALO® software and whole-tissues scanning used to determine average number of miR871-, hu*PMP22*-, and mu*Pmp22*-positive cells as well the average number of corresponding miR871, hu*PMP22*, and mu*Pmp22* dots/cells in CMT1A animals treated with AAV9.U6.miR871 (5E11 or 1E12 vg/animal) or PBS. (**F-G**) Single-cell resolution data of individual cells. Due to overlapping signals, PBS-treated cells appear on top, followed by those receiving 5E11 vg/animal, with 1E12 vg/animal-treated cells at the bottom. For D and E, values are presented as mean ± SD. **P* < 0.05, ***P* < 0.01, ****P* < 0.001, *****P* ≤ 0.0001 by 1-way ANOVA with Tukey's multiple-comparison test. Scale bar: 50 μm and 15 μm (enlarged inserts).

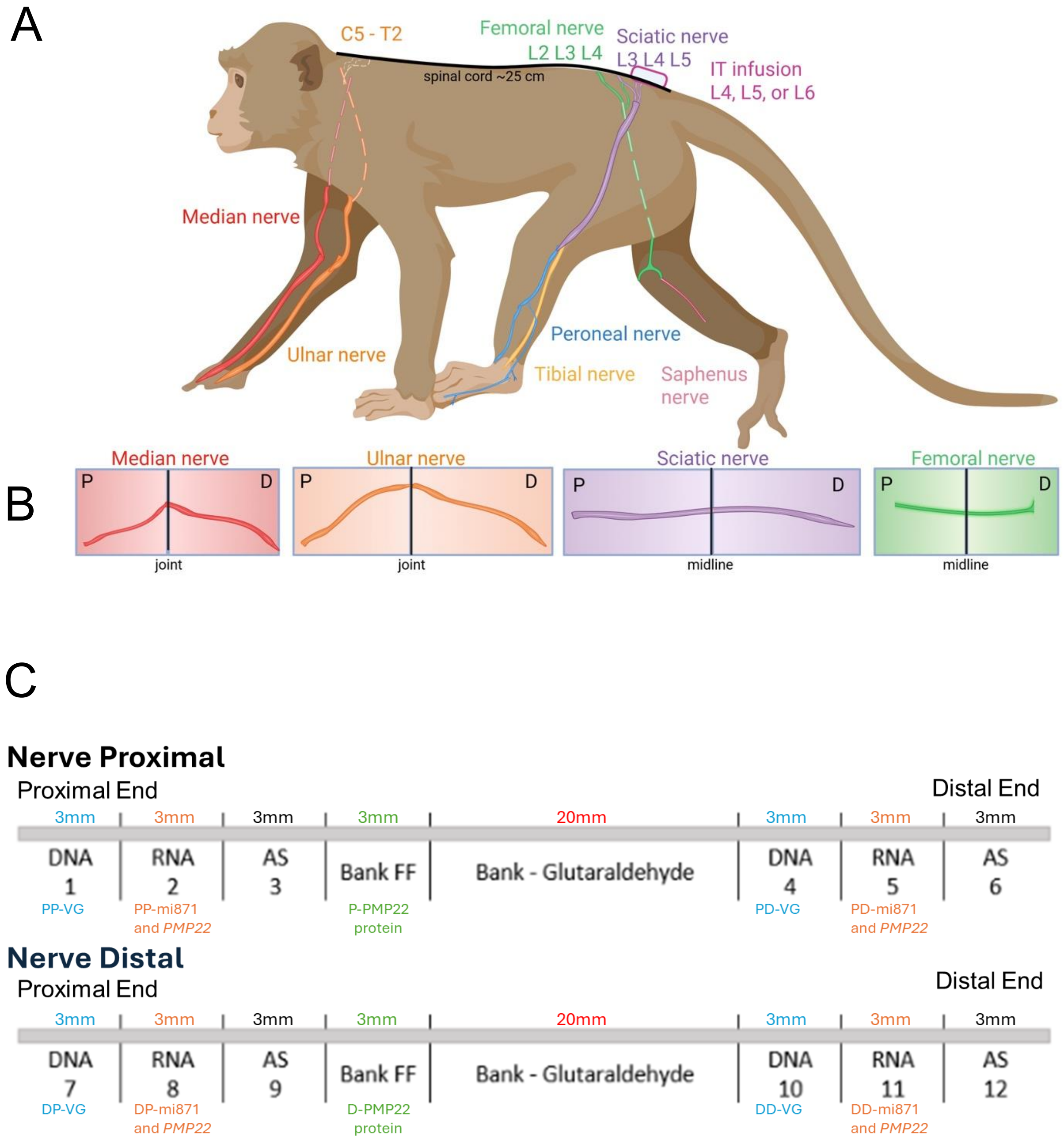

Figure S22. NHP nerve analysis design (A) Schematic of intrathecal (IT) infusion site relative to collected nerves and their innervation points. (B) Proximal and distal nerve designations were determined by the joint for the upper limb nerves (median nerve and ulnar nerve) and the midline of the fully dissected hindlimb nerves (sciatic nerve and femoral nerve). (C) Schematic of nerve dissection with minimal length per segment and assay designation; ≥3mm for each fresh fozen segment and ≥20mm for each fixed segment. Total nerve designations are ≥41mm for each individual proximal or distal segment. Vector genome analysis (VG); Probes for rt-dPCR gene of interest assays (mi871 and *PMP22);* alternative samples collected for as needed molecular assays (AS); segment for protein analysis, bank fresh frozen (Bank FF); proximal nerve proximal section (PP); proximal nerve distal section (PD); proximal (P), distal nerve proximal section (DP); distal nerve distal section (DD); distal (D).

Figure S23. Non-human primate pathology report from Amplify Bio. PDF file containing 1868 pages, uploaded as a separate supplemental file.

A

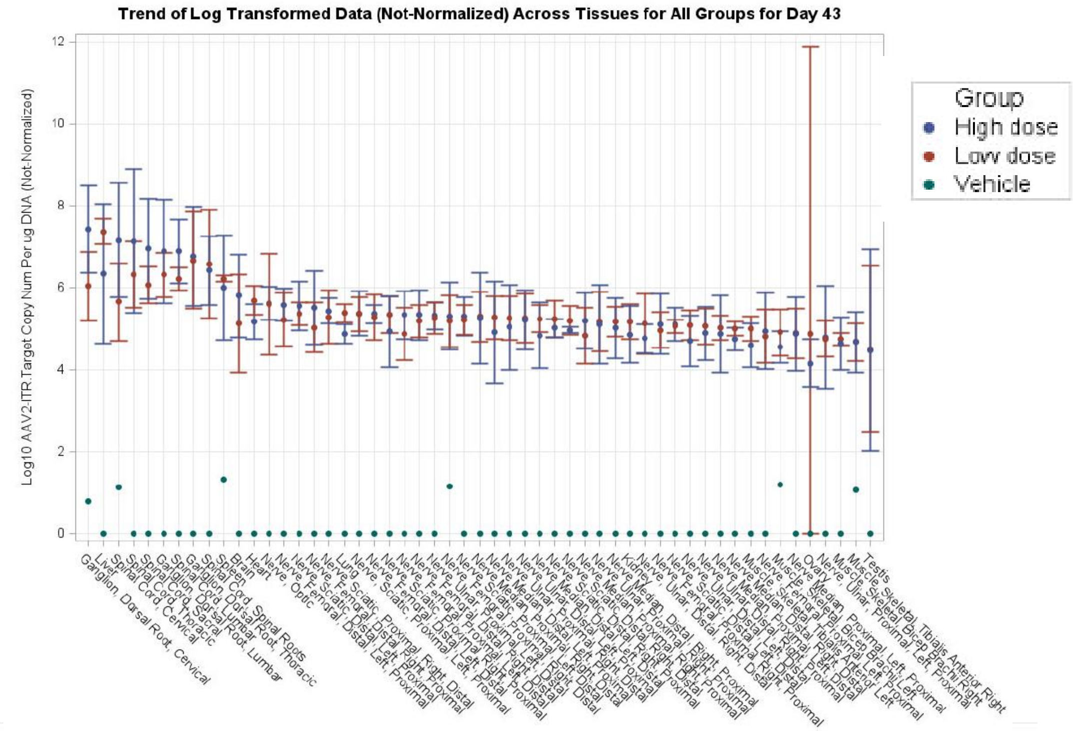

B

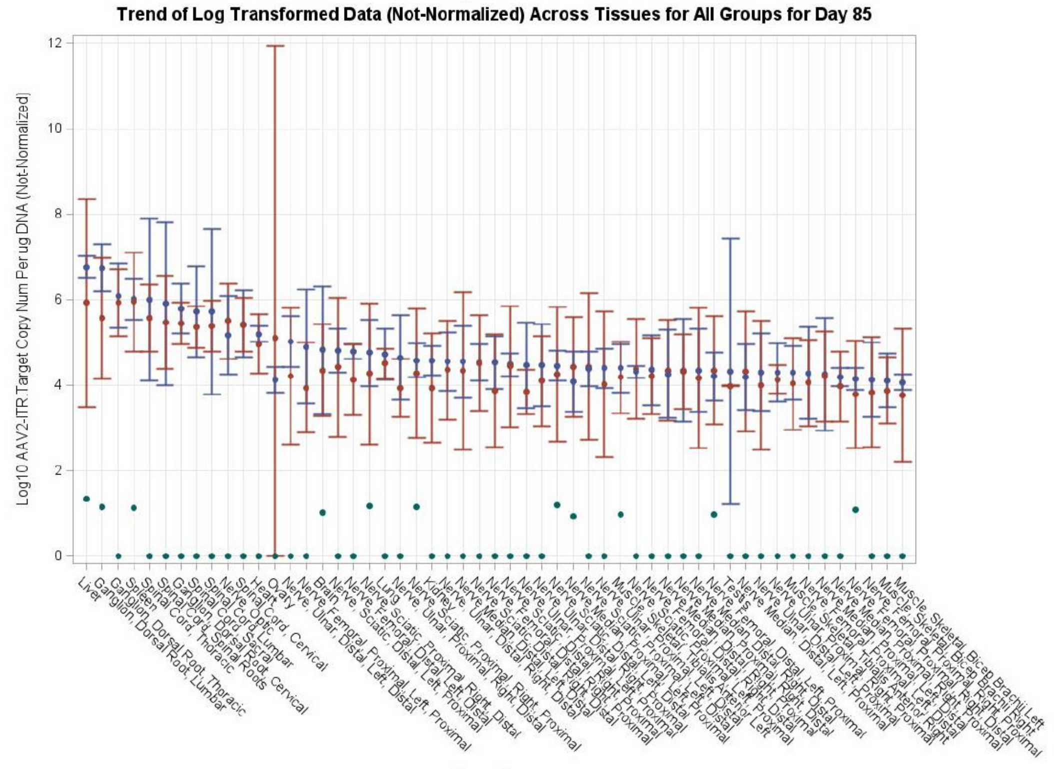

Figure S24. AAV biodistribution in all NHP tissues collected. Data show trend of log transformed AAV2-ITR mean target copy number per µg DNA. (A) 6 week and (B) 12 week, across all indicated tissues for all groups. Error bars for the low dose and high dose groups are calculated based on the 95% confidence intervals for the mean of each of these groups. Error bars have been omitted for the control group.

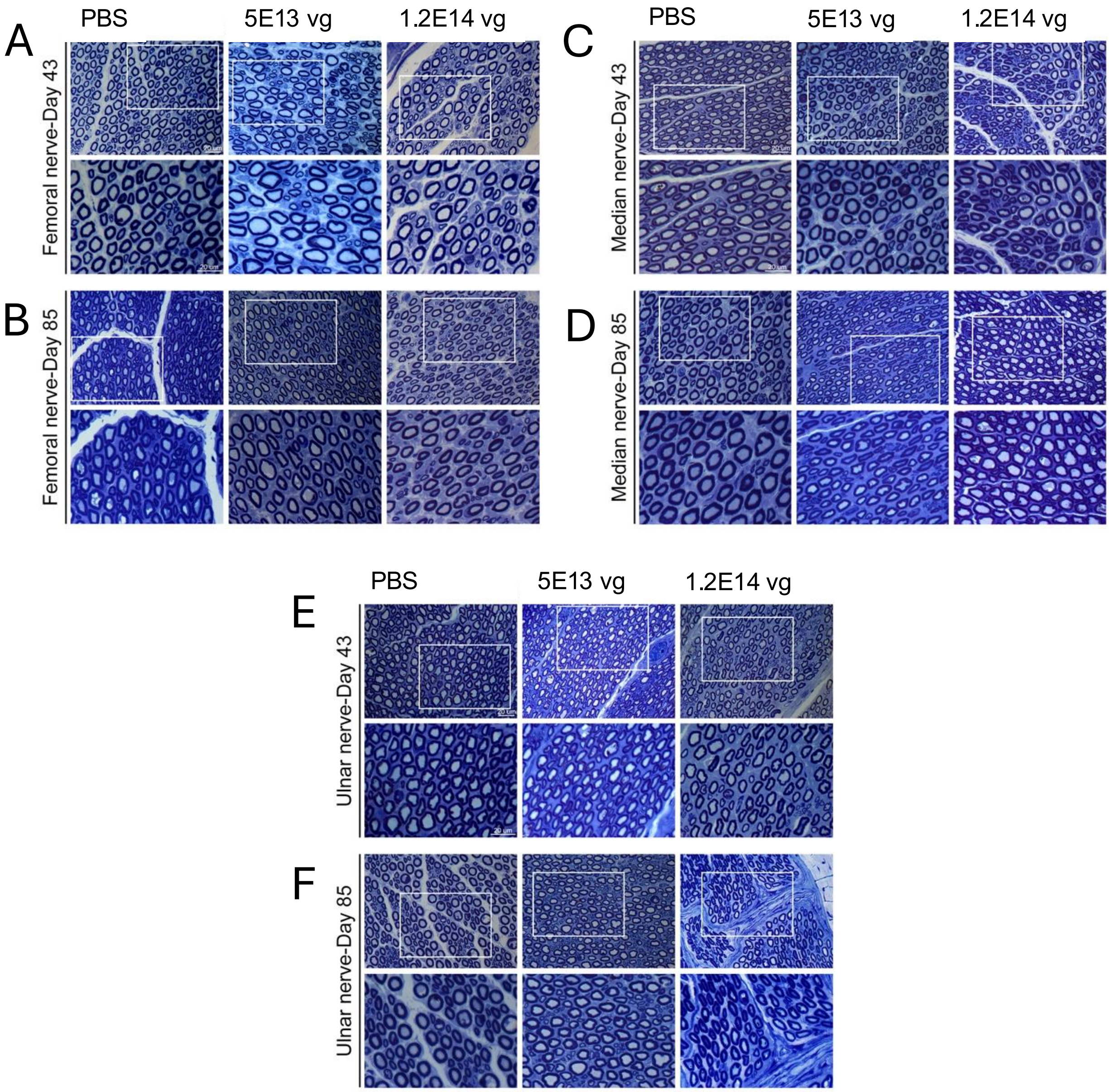

Figure S25. Representative images of NHP nerve sections stained with toluidine blue following treatment with AAV9.U6.miR871. Toluidine blue-stained semithin sections of (A-B) femoral nerves, (C-D) median nerves or (E-F) ulnar nerves 6-weeks or 12-weeks after lumbar intrathecal delivery to wild type Cynomolgus macaques. Paired images represent low (upper panels) and high magnifications (lower panels). Scale bars: 20 µm.

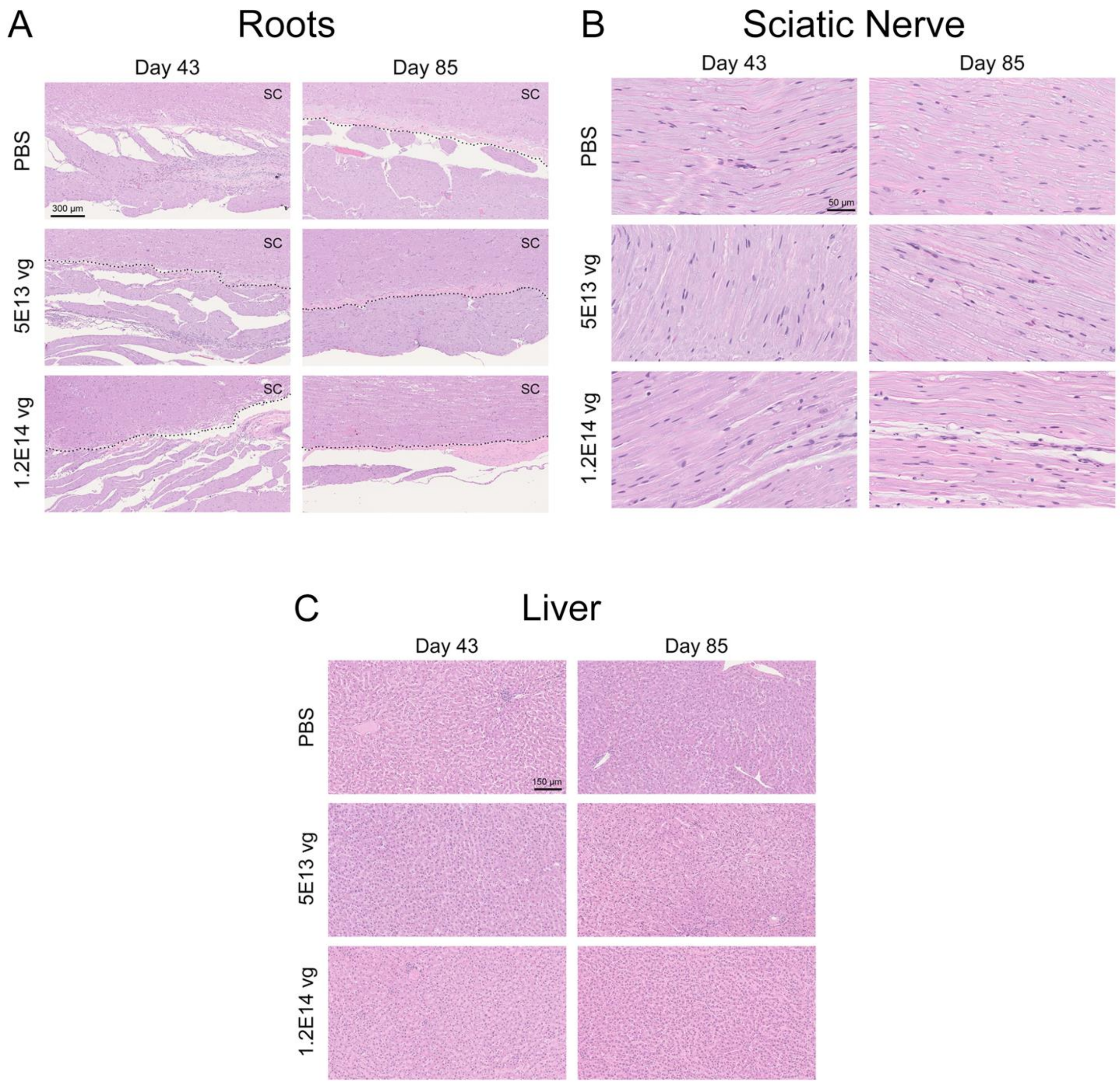

Figure S26. Representative H&E-stained images of spinal nerve roots, sciatic nerve, and liver following treatment of indicated AAV9.U6.miR871 doses.

H&E staining of spinal roots (A) attached to spinal cord, sciatic nerve (B), and liver (C) 6- and 12-weeks after lumbar intrathecal delivery in NHPs. No significant histopathological findings were noted in treated animals. Scale bar: 50 µm.

Figure S27. All raw western blots from NHP target engagement studies. PDF file containing 128 pages of images, uploaded as a separate file.

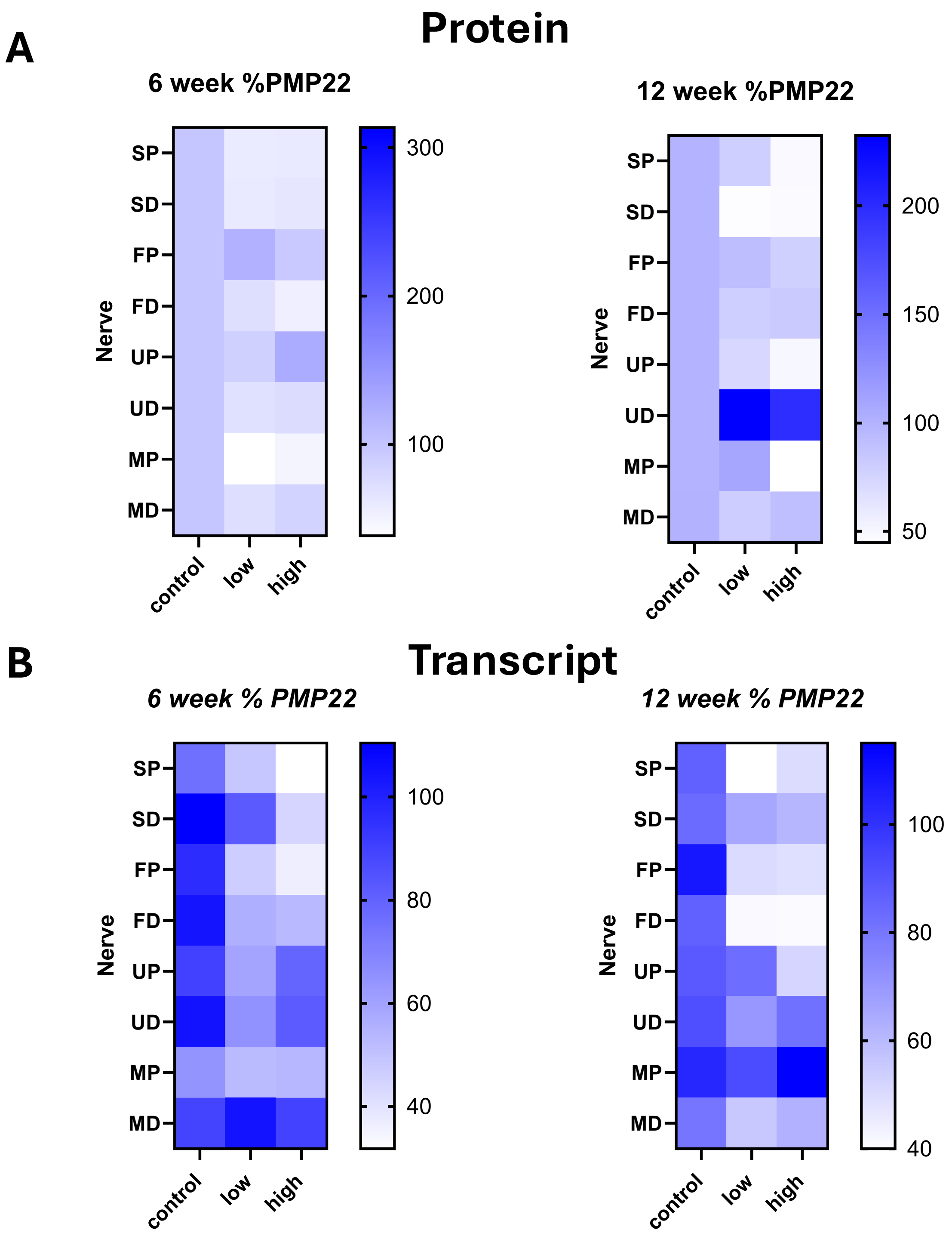

Figure S28. Heatmaps summarizing *PMP22* mRNA and PMP22 protein reductions in NHP nerves. (A) Heatmaps of average PMP22 protein level normalized to control nerve at 6- and 12-wk timepoints. (B) Heatmaps of average *PMP22* transcript normalized to total control average. Overall, reduction of protein and/or transcript indicates evidence for gene silencing in all samples tested.
