# Supplementary material for: Safety, efficacy, and distal nerve Schwann cell biodistribution in mice and NHPs to support translation of AAV9 RNAi therapy for CMT1A
Source: Mol Ther Nucleic Acids. 2026 Feb 27;37(2):102881. doi: 10.1016/j.omtn.2026.102881 (PMC13051718; doi:10.1016/j.omtn.2026.102881)
Supplement: Figure S18. Mouse toxicology report from Transcendent Bio [file mmc2.pdf]

ANATOMIC PATHOLOGY REPORT

Off-Target Effects Of AAV-Mediated RNAi Gene Silencing Of PMP22 In The CMT1A C61 Heterozygote Model Of Charcot Marie Tooth Type 1 (CMT1A) In C61 And Wild-Type Mice

SUMMARY

The purpose of this study was to evaluate the off-target effects of AAV-mediated RNAi gene silencing of PMP22 in the CMT1A C61 heterozygote model of Charcot Marie Tooth type 1 (CMT1A). Through gross and microscopic evaluation, the safety/toxicology in C61 and wild-type mice in vivo was examined, and dose-response treatment studies in C61het mice were conducted. Varying degrees of hepatocellular degeneration and necrosis was often observed with oval cell hyperplasia. The percentage of mice with oval cell hyperplasia increased in groups C, D, and E (20%, 60%, and 100%, respectively). This change was likely the result of the increased dose between the groups, if an infectious cause is ruled out. The percentage of mice with EMH increased in groups C, D, and E (80%, 60%, and 100%, respectively). EMH can occur in response to increased hematopoietic demand. Precipitating factors include xenobiotic toxicity, infection, and stress. The presence of EMH in this study was likely related to the test article, if other causes can be ruled out. The lesions observed in the heart, kidney, skeletal muscle, and DRG were generally mild and not likely related to the test article. There were no significant lesions observed in the brain, spinal cord, sciatic nerve, or femoral nerve in any group.

Methods

1. Mice were on a C57BL/6 background. There were no abnormalities observed during gross examination of all mice of treatment and control groups [per contributor]. The following tissues were evaluated for microscopic examination: Spinal cord with roots attached, quadriceps muscle (bilateral), brain, liver, kidney, heart, lungs, dorsal root ganglia (DRG) (lumbar), sciatic nerve, femoral nerve.

2. Microscopic lesions were graded on a scale of 0-3.
0=none; 0% of the tissue examined affected.
1=minimal-mild; 1-33% of the tissue examined affected.
2=moderate; 33-66% of the tissue examined affected.
3=severe; >66% of the tissue examined affected.

Regarding hepatocellular vacuolation, grading was determined by the amount of vacuolation, and presence or absence of cellular swelling.

This report discusses the macroscopic and microscopic findings of previously listed tissues occurring in association with administration of the test or control articles.

Results and Discussion

Within the liver, there was centrilobular to diffuse, hepatocellular vacuolation, consistent with intracellular glycogen accumulation, observed in animals in all groups. This was the only change observed in animals in groups A and B, except animal 1364 (group B) where there was also minimal, single-cell, hepatocyte necrosis. In groups C, D, and E, there was also oval cell hyperplasia, mild extramedullary hematopoiesis (EMH), and occasional hepatocellular necrosis observed. Oval cell

hyperplasia is often observed in livers as a result of toxic changes or infection, as well as mice treated with certain hepatocarcinogens. Varying degrees of hepatocellular degeneration and necrosis is often observed with oval cell hyperplasia. The percentage of mice with oval cell hyperplasia increased in groups C, D, and E (20%, 60%, and 100%, respectively.) This change is likely the result of the increased dose between the groups if an infectious cause is ruled out.

Within the lung, there was a mild to moderate, interstitial, and often perivascular, lymphohistiocytic infiltrate observed in all groups (including group A). This change is likely not related to the test article or model.

Within the heart, in animal 1446 (group A), there was a mild, lymphohistiocytic, epicardial infiltrate. In animal 1376 (group E), there was a mild focal, lymphocytic, endocardial infiltrate. This change is likely not related to the test article or model.

Changes in the kidney were observed in mice of all groups, including group A. Lesions consist of a focal to multifocal, minimal to mild, lymphoplasmacytic infiltrate within the interstitium, surrounding blood vessels, and surrounding the pelvis. There was also focal, mild to minimal, tubular proteinosis in 1-2 mice in groups A, B, D, and E. There was mild, multifocal, tubular degeneration and regeneration in animal 1328 (group E). Renal changes are likely not related to the test article or model.

Within the skeletal muscle (quadriceps femoris muscle), there are lesions observed in groups A, B, C, and E. Lesions consist of minimal to mild, myocyte degeneration characterized by swollen, hypereosinophilic or vacuolated cytoplasm, and regeneration characterized by centralized nuclei. There is a mild, interstitial, lymphocytic infiltrate in 3 mice from group A. Skeletal muscle changes are likely not related to the test article or model.

There are few lymphocytes, plasma cells, and neutrophils in the soft issue surrounding the DRG in mice in all groups, except group D. The cells are not infiltrating the DRG. The cells may have been displaced from the bone marrow during sample collection. The presence of these cells are likely not related to the test article or model.

There were no significant lesions observed in the brain, spinal cord, sciatic nerve, or femoral nerve in any group.

Summaries of microscopic lesions are reflected in Text Tables 1-5.

Text Table 1
Incidence Summary (with percentages) of Liver Microscopic Observations

Group	A PBS	B AAV9-miR871 1e11 vg	C AAV9-miR871 2e11 vg	D AAV9-miR871 5e11 vg	E AAV9-miR871 1e12 vg
Number Examined	4	5	5	5	5
Hepatocellular vacuolation	4<br>100%	5<br>100%	4<br>80%	3<br>60%	3<br>60%
Oval cell hyperplasia	0<br>0%	0<br>0%	1<br>20%	3<br>60%	5<br>100%

Hepatocellular necrosis	0<br>0%	1<br>20%	0<br>0%	1<br>20%	4<br>80%
Extramedullary hematopoiesis	0<br>0%	0<br>0%	4<br>80%	3<br>60%	5<br>100%

Text Table 2
Incidence Summary (with percentages) of Lung Microscopic Observations

Group	A<br>PBS	B<br>AAV9-<br>miR871<br>1e11 vg	C<br>AAV9-<br>miR871<br>2e11 vg	D<br>AAV9-<br>miR871<br>5e11 vg	E<br>AAV9-<br>miR871<br>1e12 vg
Number Examined	5	5	5	5	5
Interstitial infiltrate	5<br>100%	5<br>100%	5<br>100%	5<br>100%	5<br>100%
Perivascular infiltrate	1<br>20%	2<br>40%	5<br>100%	2<br>40%	4<br>80%
Eosinophilic crystalline pneumonia	0<br>0%	0<br>0%	1<br>20%	0<br>0%	0<br>0%

Text Table 3
Incidence Summary (with percentages) of Heart Microscopic Observations

Group	A<br>PBS	B<br>AAV9-<br>miR871<br>1e11 vg	C<br>AAV9-<br>miR871<br>2e11 vg	D<br>AAV9-<br>miR871<br>5e11 vg	E<br>AAV9-<br>miR871<br>1e12 vg
Number Examined	5	5	5	5	5
Epicardial infiltrate	1<br>20%	0<br>0%	0<br>0%	0<br>0%	0<br>0%
Endocardial infiltrate	0<br>0%	0<br>0%	0<br>0%	0<br>0%	1<br>20%

Text Table 4
Incidence Summary (with percentages) of Kidney Microscopic Observations

Group	A<br>PBS	B<br>AAV9-<br>miR871<br>1e11 vg	C<br>AAV9-<br>miR871<br>2e11 vg	D<br>AAV9-<br>miR871<br>5e11 vg	E<br>AAV9-<br>miR871<br>1e12 vg
Number Examined	5	5	5	5	5
Tubular proteinosis	2<br>40%	1<br>20%	0<br>0%	2<br>40%	1<br>20%
Renal tubular epithelial cell degeneration, and regeneration	0<br>0%	0<br>0%	0<br>0%	0<br>0%	1<br>20%
Interstitial infiltrate	0<br>0%	1<br>20%	0<br>0%	2<br>40%	1<br>20%
Peripelvic infiltrate	1<br>20%	2<br>40%	1<br>20%	2<br>40%	2<br>40%

Text Table 5
Incidence Summary (with percentages) of Quadriceps Femoris Muscle Microscopic Observations

Group	A PBS	B AAV9-miR871 1e11 vg	C AAV9-miR871 2e11 vg	D AAV9-miR871 5e11 vg	E AAV9-miR871 1e12 vg
Number Examined	5	5	5	5	5
Interstitial infiltrate	4<br>80%	0<br>0%	0<br>0%	0<br>0%	5<br>100%
Myocyte degeneration, regeneration	2<br>40%	1<br>20%	1<br>20%	0<br>0%	1<br>20%

Comments

Within the liver, there was centrilobular to diffuse, hepatocellular vacuolation, consistent with intracellular glycogen accumulation. Hepatocellular glycogen accumulation was observed in animals in all groups and likely not related to the test article. Hepatocellular glycogen accumulation, to the degree observed in this study, is considered a background change, and is associated with food consumption. This was the only change observed in animals in groups A and B, except animal 1364 (group B) where there was also minimal, single-cell, hepatocyte necrosis. In groups C, D, and E, there was also oval cell hyperplasia, mild EMH, and occasional hepatocellular necrosis observed. Oval cell hyperplasia is often observed in livers as a result of toxic changes or infection, as well as mice treated with certain hepatocarcinogens. Varying degrees of hepatocellular degeneration and necrosis is often observed with oval cell hyperplasia. The percentage of mice with oval cell hyperplasia increased in groups C, D, and E (20%, 60%, and 100%, respectively). This change is likely the result of the increased dose between the groups, if an infectious cause is ruled out. The percentage of mice with EMH increased in groups C, D, and E (80%, 60%, and 100%, respectively). EMH can occur in response to increased hematopoietic demand. Precipitating factors include xenobiotic toxicity, infection, and stress. The presence of EMH in this study is likely related to the test article, if other causes can be ruled out.

The lesions observed in the heart, kidney, skeletal muscle, and DRG were generally mild and not likely related to the test article.

There were no significant lesions observed in the brain, spinal cord, sciatic nerve, or femoral nerve in any group.

REGULATORY COMPLIANCE
The anatomic pathology evaluation and report for this study were completed as a Non-GLP study.

Angela K Brice, DVM PhD, DACVP
Pathologist

June 6, 2023
Date
