# Supplementary material for: Safety, efficacy, and distal nerve Schwann cell biodistribution in mice and NHPs to support translation of AAV9 RNAi therapy for CMT1A
Source: Mol Ther Nucleic Acids. 2026 Feb 27;37(2):102881. doi: 10.1016/j.omtn.2026.102881 (PMC13051718; doi:10.1016/j.omtn.2026.102881)
Supplement: Figure S27. All raw western blots from NHP target engagement studies [file mmc4.pdf]

Western Blot Imaging Form

**Study**	ARM101-CMT1A-NHP-001
**Timepoint**	6 week
**Tissue**	Median nerve - Right
**Anatomical Location** (Highlight/Circle one)	Distal / Proximal (circled) / N/A<br>Other:

Stain Free Gel

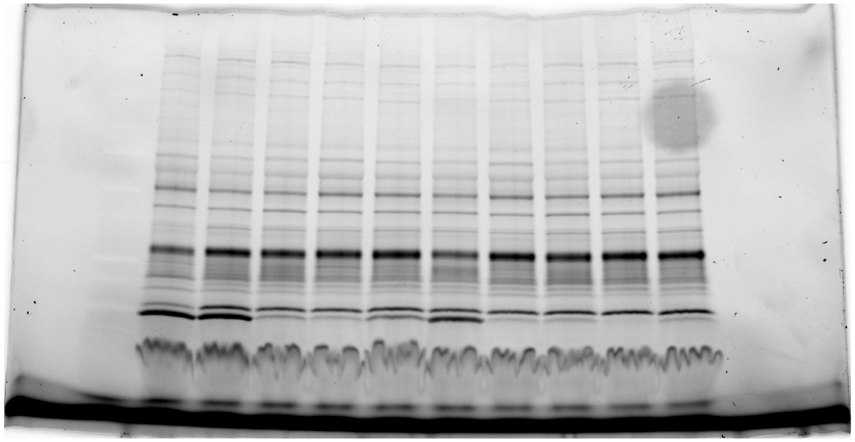

Date: 04 Jun 2025 MPT

Stain Free Membrane

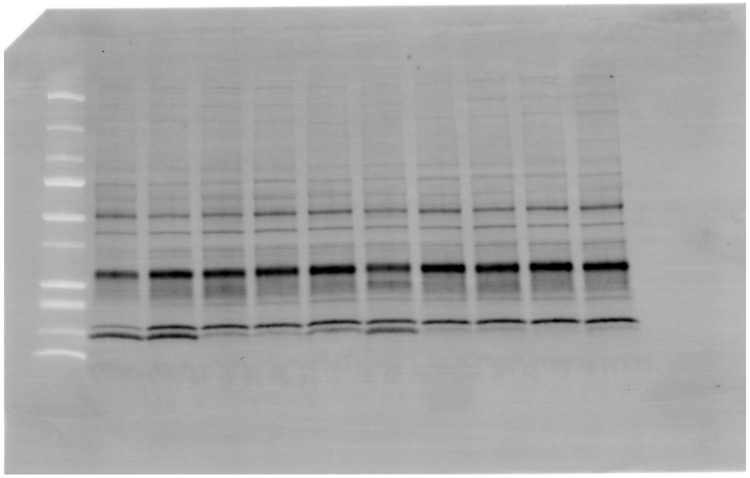

Date: 04 Jun 2025 MPT

1° PMP22

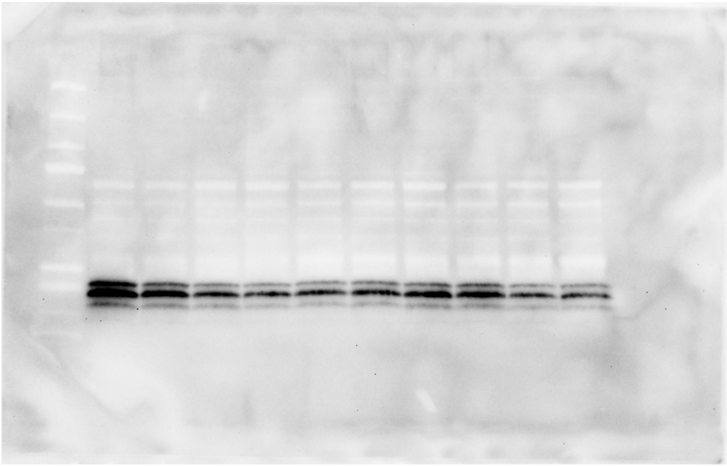

Date: 05 Jun 2025 MPT

1° β-Actin

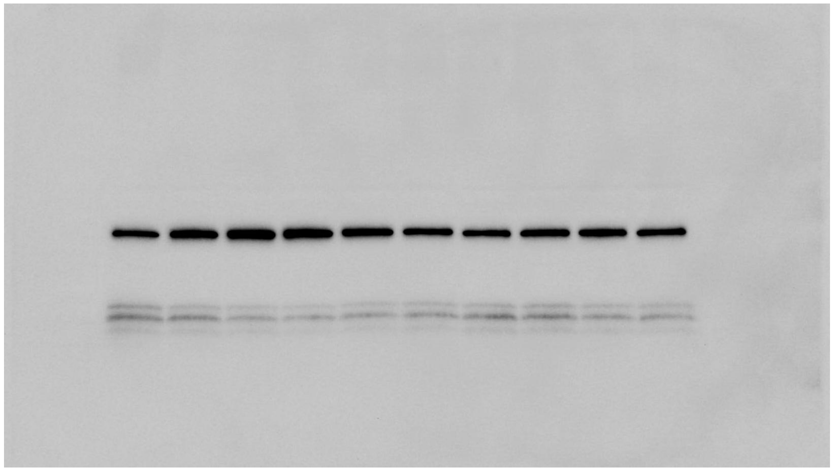

Date: 05 Jun 2025 MPT

Western Blot Imaging Form

Strip Check

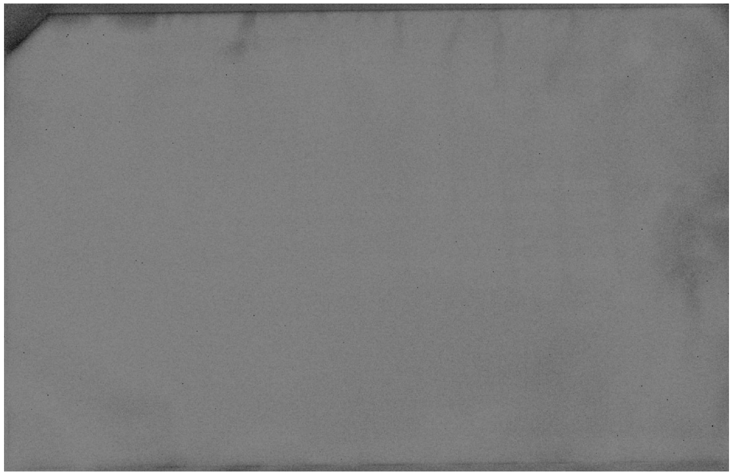

Date: 09 Jun 2025 MPT

1° MPZ

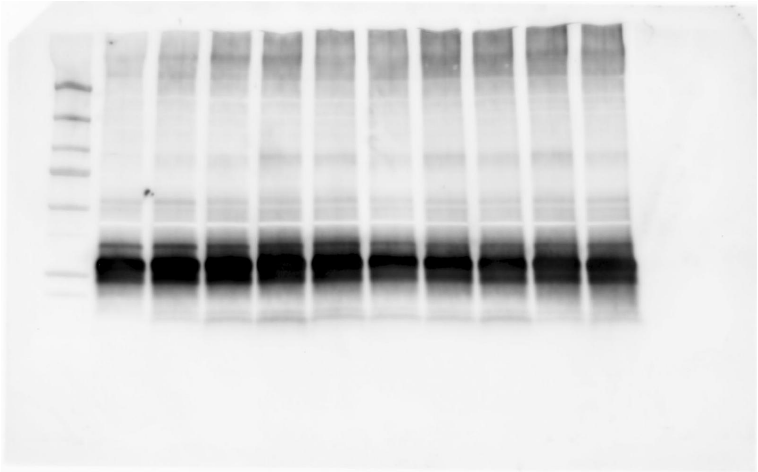

Date: 10 Jun 2025 MPT

ImageJ Quantification Box – PMP22

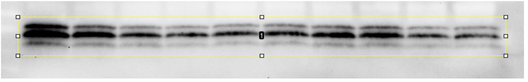

Date: 18 Jun 2025 LMW

ImageJ Quantification Box – B-Actin

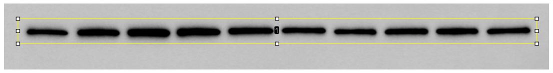

Date: 18 Jun 2025 LMW

ImageJ Quantification Box - MPZ

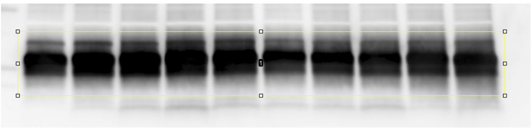

Date: 18 Jun 2025 LMW

Plot 1 Values PMP22

	Area
1	105118.7
2	62580.65
3	33277.24
4	23495.95
5	28349.97
6	37890.02
7	53472.65
8	57270.14
9	26743.24
10	26639.09

Plot 1-PMP22

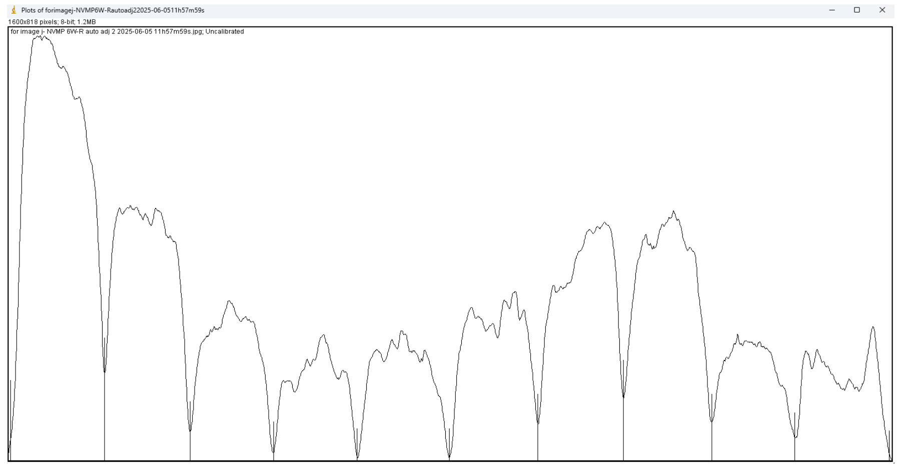

Date: 18 Jun 2025 LMW

Plot 2 Values B-Actin

	Area
1	37211.02
2	47901.32
3	53924.71
4	52745.61
5	47407.9
6	39438.9
7	36322.78
8	41384.78
9	41444.49
10	38763.32

Plot 2-B-Actin

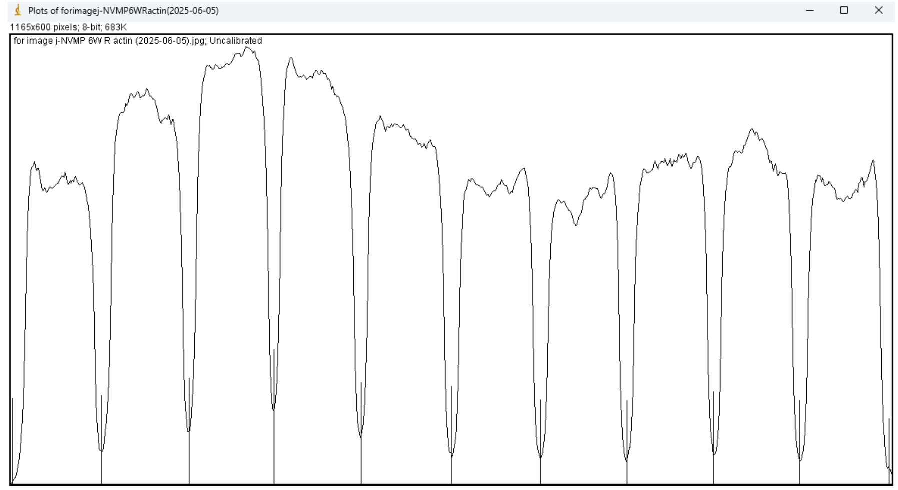

Date: 18 Jun 2025 LMW

Plot 3 Values-MPZ

	Area
1	46642.13
2	55817.74
3	57029.52
4	59123.4
5	60808.88
6	48587.42
7	52577.35
8	51451.76
9	53015.88
10	55948.42

Plot 3-MPZ

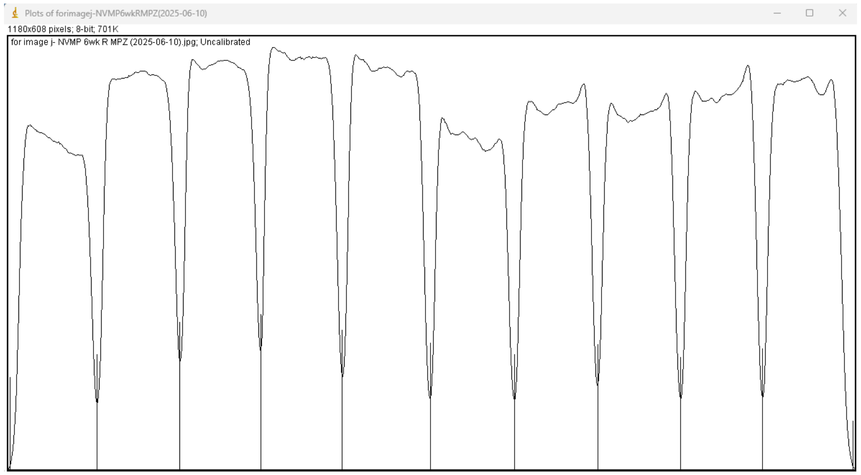

Date: 18 Jun 2025 LMW

Operator Information

Performed By:	Name:	Signature:
	Position/Lab:	Date:

Performed By:	Name: Lindsay Wallace PhD	Signature: [signature]
	Position/Lab: Sr Research Scientist / Harper Lab	Date: 18 Jun 2025

Western Blot Imaging Form

**Study**	ARM101-CMT1A-NHP-001
**Timepoint**	6 week
**Tissue**	Median nerve
**Anatomical Location** (Highlight/Circle one)	Distal (circled) Proximal N/A Other:

Stain Free Gel

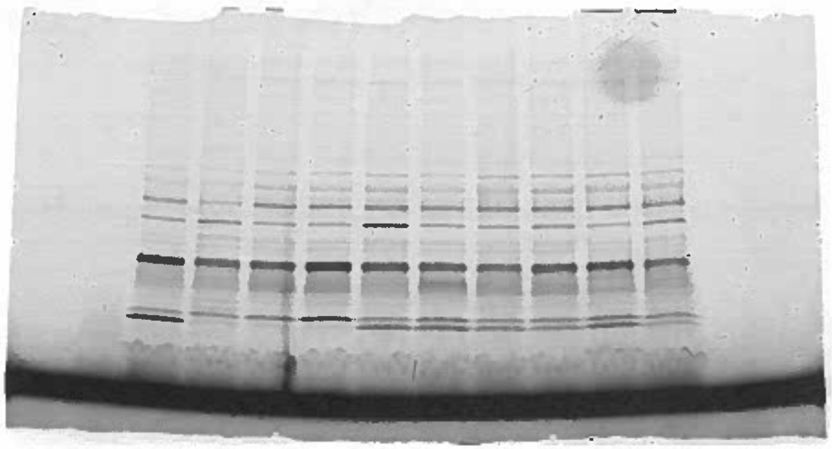

Date: 06 Jun 2024 MPT

Stain Free Membrane

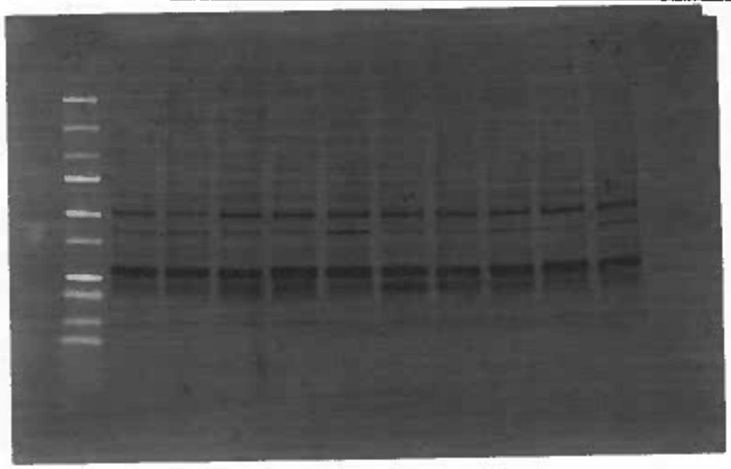

Date: 07 Jun 2024 MPT

1° PMP22

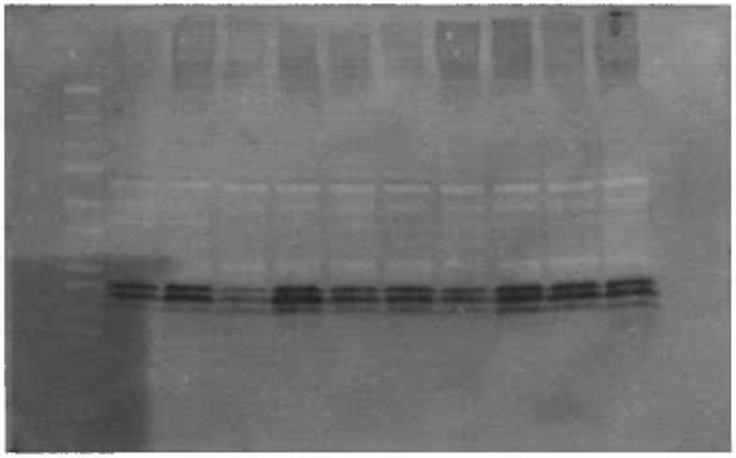

Date: 07 Jun 2024 MPT

1° β-Actin

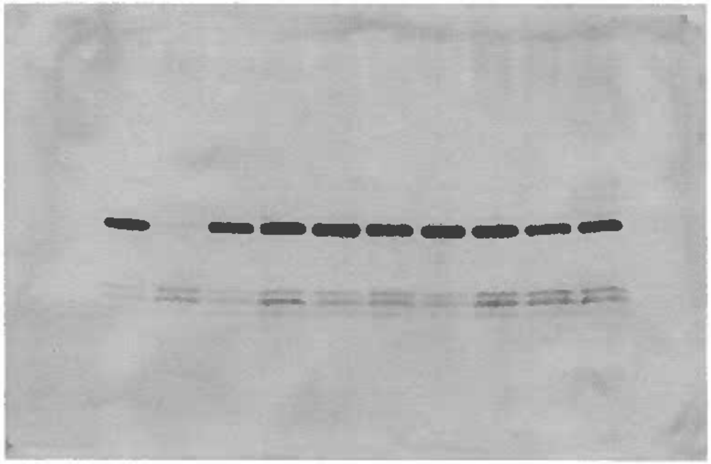

Date: 07 Jun 2024 MPT

Western Blot Imaging Form

Strip Check

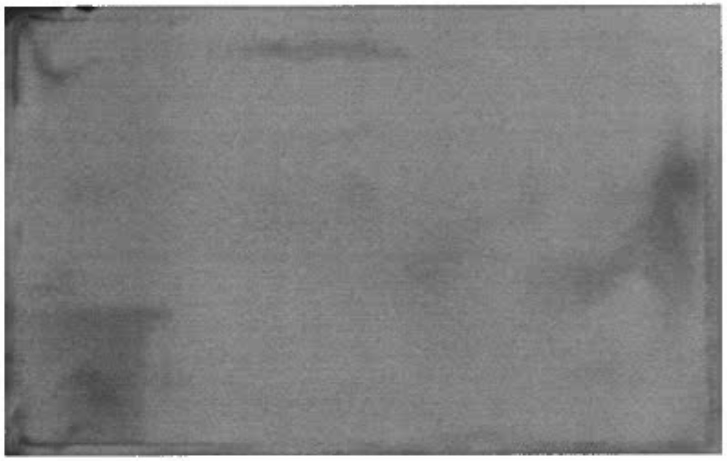

Date: 14 Jun 2024 MPT

1° MPZ

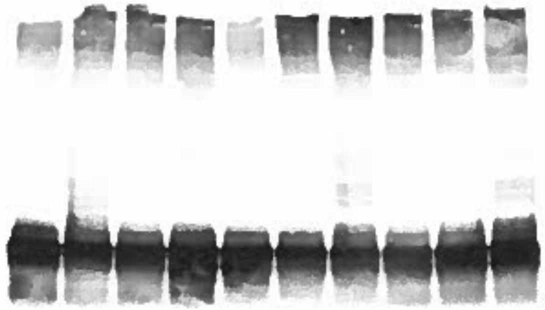

Date: 14 Jun 2024 MPT

ImageJ Quantification Box – PMP22

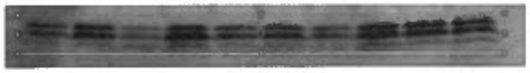

Date: 20 Jun 2024 LMW

ImageJ Quantification Box – B-Actin

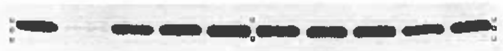

Date: 20 Jun 2024 LMW

ImageJ Quantification Box - MPZ

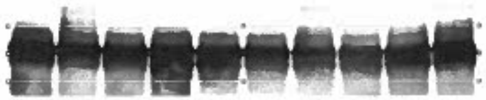

Date: 20 Jun 2024 LMW

Plot 1 Values PMP22

	Area
1	41171.49
2	35945.37
3	18432
4	45821.78
5	30343.54
6	34382.95
7	26812
8	44967.37
9	35528.25
10	36269.15

Plot 1-PMP22

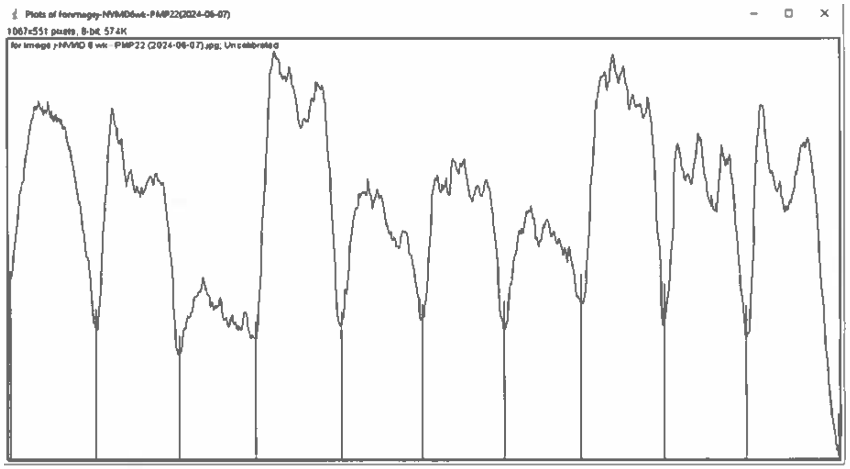

Date: 20 Jun 2024 LMW

Plot 2 Values B-Actin

	Area
1	35092.13
2	2598.761
3	37245.08
4	41875.66
5	46315.08
6	40839.78
7	39475.54
8	40853.13
9	34267.37
10	36433.71

Plot 2-B-Actin

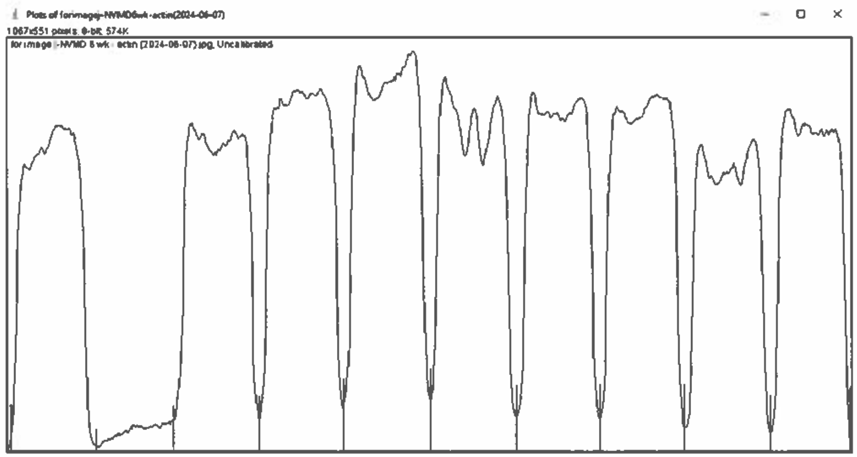

Date: 20 Jun 2024 LMW

Plot 3 Values-MPZ

	Area
1	46789.18
2	38409.83
3	38382.05
4	46092.83
5	40905.95
6	33902.93
7	32443.47
8	29805.88
9	39613.83
10	43590.71

Plot 3-MPZ

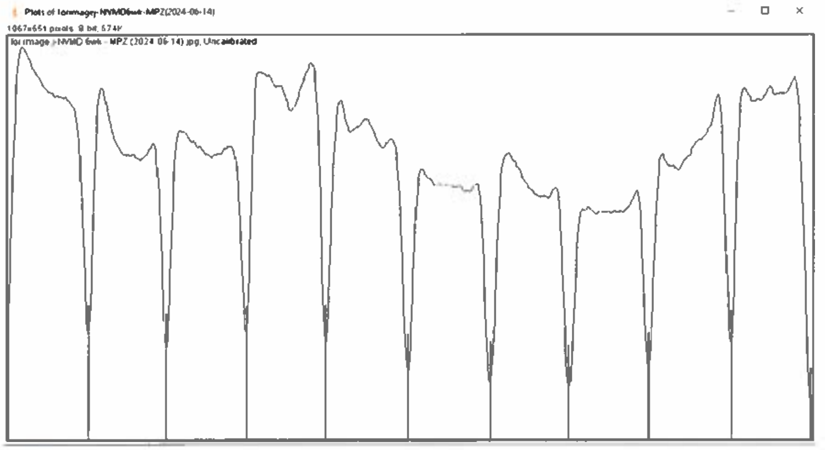

Date: 20 Jun 2024 LMW

Operator Information

**Performed By:**	Name: Merlin P Thangaraj	Signature:
	Position/Lab: Postdoctoral Scientist/ Harper Lab	Date: 27 June 2024

**Performed By:**	Name: Lindsay Wallace	Signature:
	Position/Lab: Sr Research Scientist / Harper Lab	Date: 27 Jun 2024

Western Blot Imaging Form

**Study**	ARM101-CMT1A-NHP-001
**Timepoint**	6 week
**Tissue**	Femoral nerve
**Anatomical Location** (Highlight/Circle one)	Distal (circled) Proximal N/A Other:

Stain Free Gel

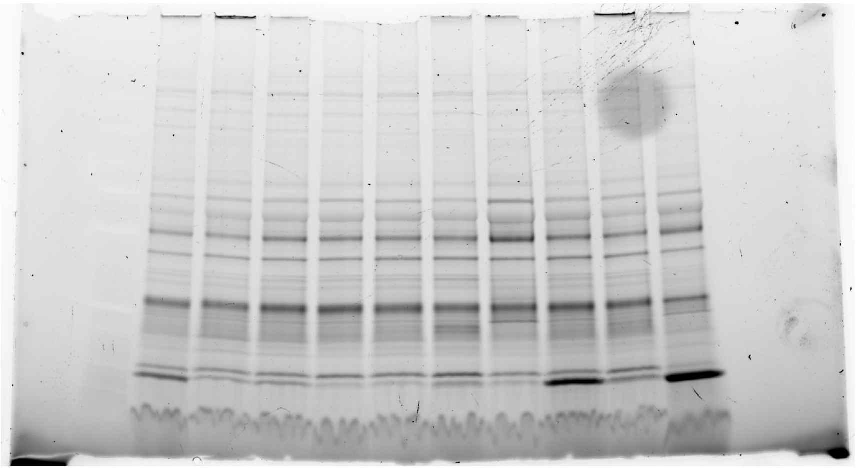

Date: 13 Jun 2024 MPT

Stain Free Membrane

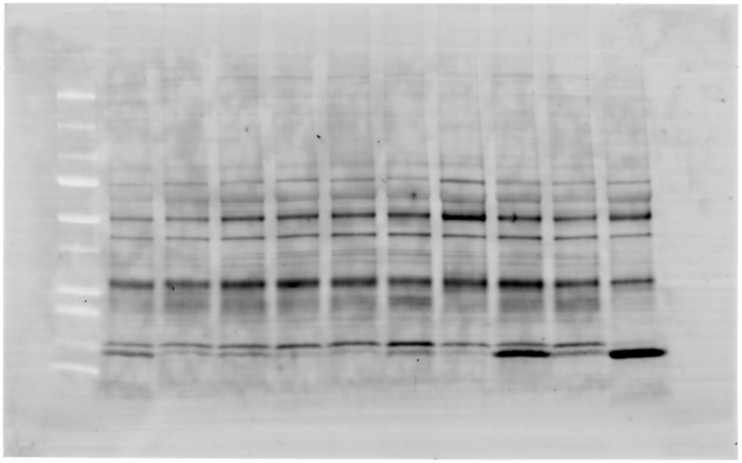

Date: 13 Jun 2024 MPT

1° PMP22

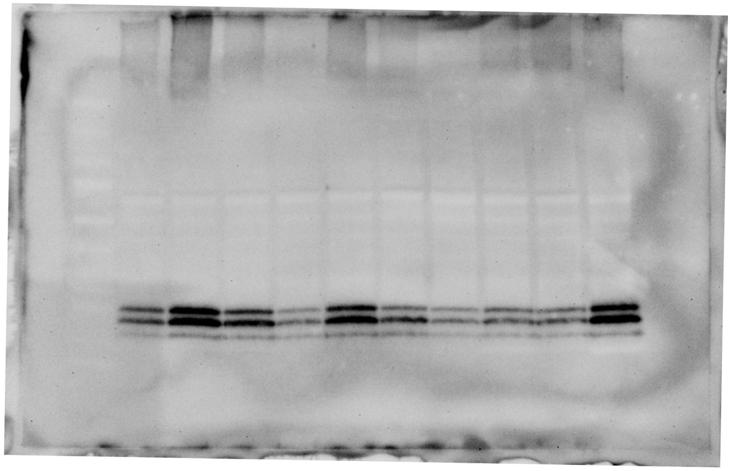

Date: 14 Jun 2024 MPT

1° β-Actin

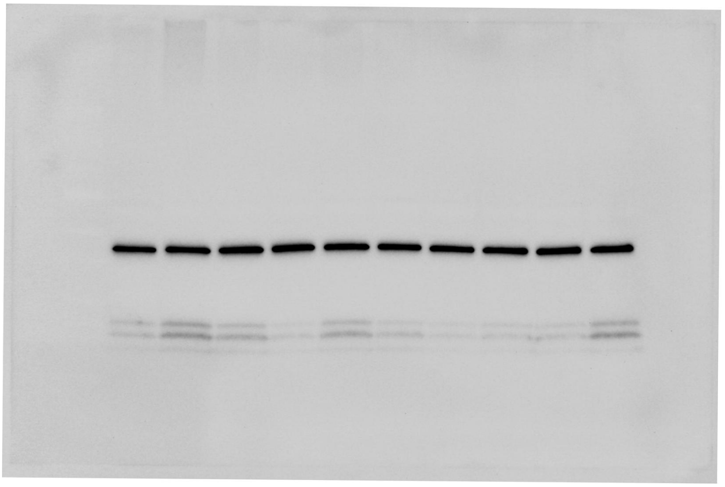

Date: 14 Jun 2024 MPT

Western Blot Imaging Form

Strip Check

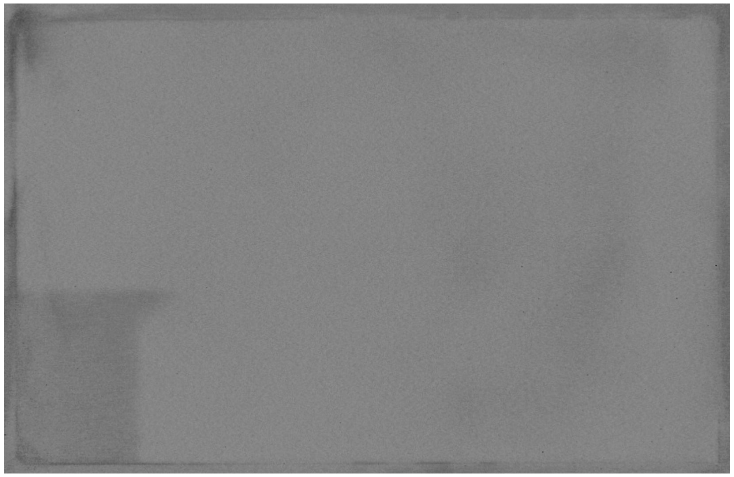

Date: 17 Jun 2024 MPT

1° MPZ

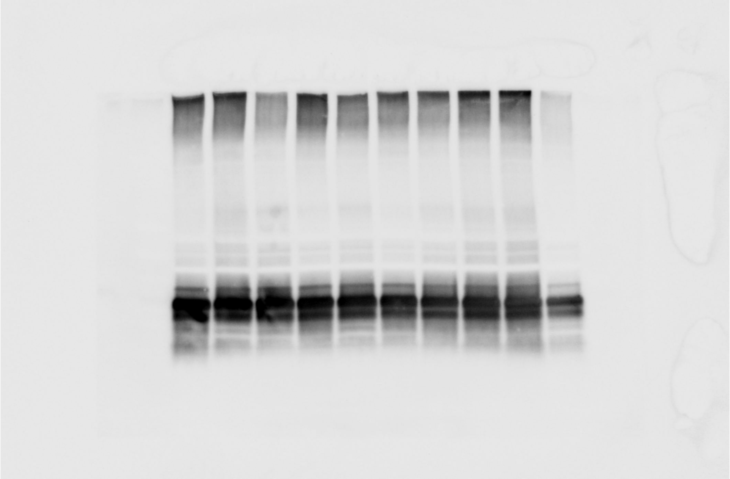

Date: 18 Jun 2024 MPT

ImageJ Quantification Box – PMP22

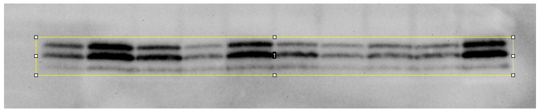

Date: 19 Jun 2024 LMW

ImageJ Quantification Box – B-Actin

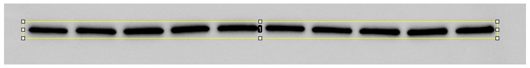

Date: 19 Jun 2024 LMW

ImageJ Quantification Box - MPZ

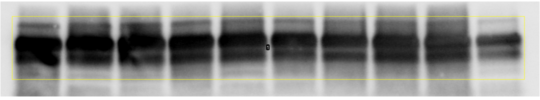

Date: 26 Jun 2024 LMW

Western Blot Imaging Form

Plot 1 Values PMP22

	Area
1	34126.15
2	71250.92
3	47957.1
4	15899.13
5	67547.87
6	34669.73
7	13834.32
8	18116.13
9	23319.51
10	68304.07

Plot 1-PMP22

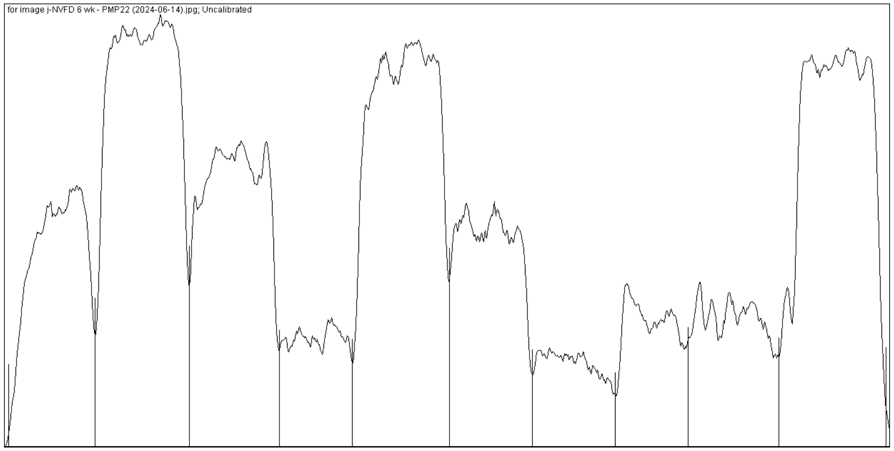

Date: 19 Jun 2024 LMW

Plot 2 Values B-Actin

	Area
1	50891.44
2	54585.97
3	58284.15
4	55913.9
5	53975.02
6	50714.49
7	51681.56
8	53526.27
9	55740.44
10	53066.61

Plot 2-B-Actin

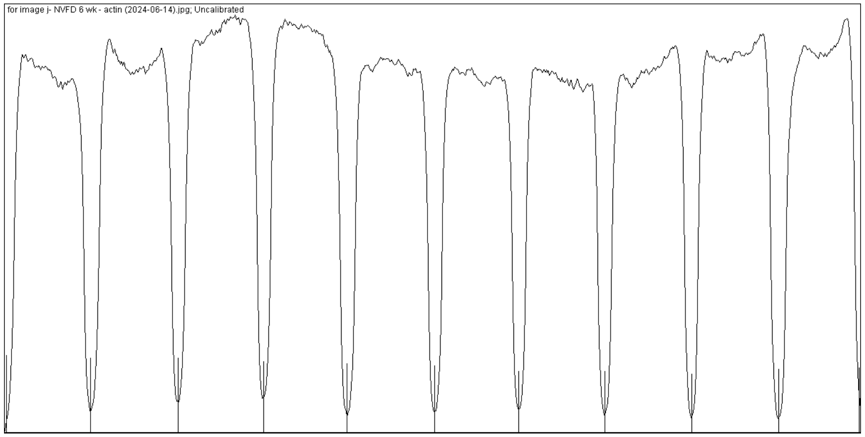

Date: 19 Jun 2024 LMW

Western Blot Imaging Form

Plot 3 Values-MPZ

	Area
1	60221.95
2	61672.32
3	61484.32
4	56440.83
5	56479.97
6	50066.95
7	52460.9
8	56632.66
9	53048.49
10	27374.78

Plot 3-MPZ

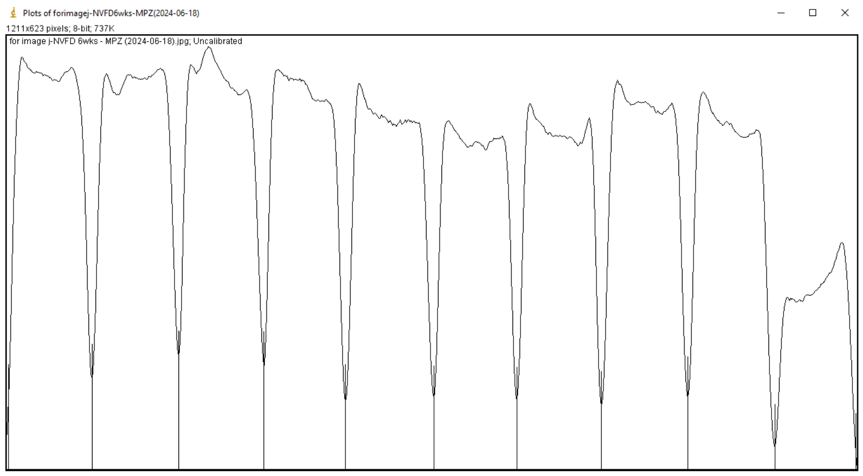

Date: 26 Jun 2024

Operator Information

Performed By:		
	**Name:** Merlin P Thangaraj	**Signature:**
	**Position/Lab:** Postdoctoral Scientist / Harper Lab	**Date:** 27 Jun 2024

Performed By:		
	**Name:** Lindsay Wallace	**Signature:**
	**Position/Lab:** Senior Research Scientist/Harper Lab	**Date:** 26 Jun 2024

Western Blot Imaging Form

**Study**	ARM101-CMT1A-NHP-001
**Timepoint**	12 week
**Tissue**	Femoral Nerve
**Anatomical Location** (Highlight/Circle one)	Distal (circled) Proximal N/A Other:

Stain Free Gel

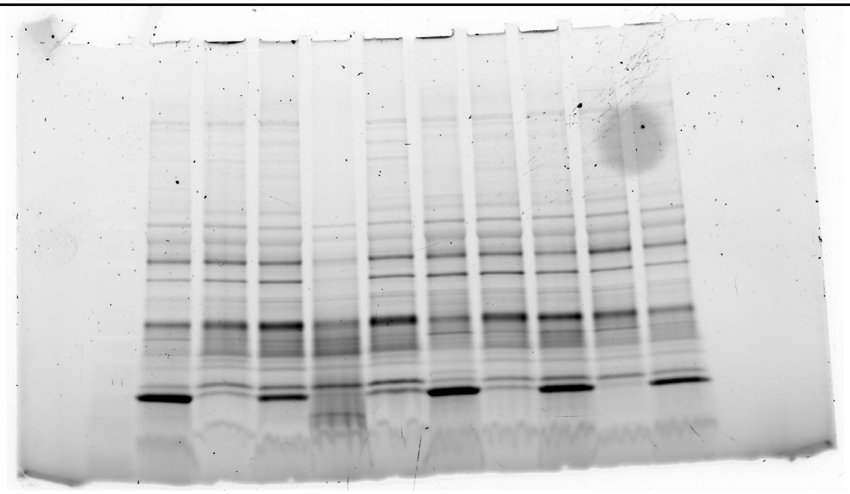

Date: 29 Feb 2024 MPT

Stain Free Membrane

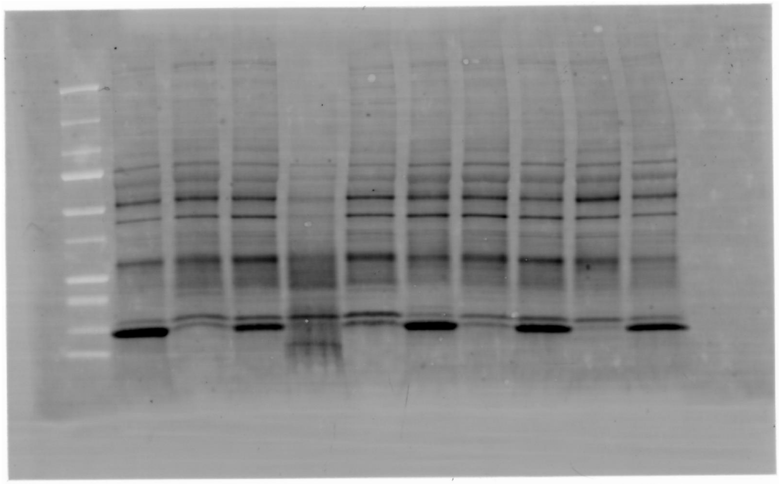

Date: 29 Feb 2024 MPT

1° PMP22

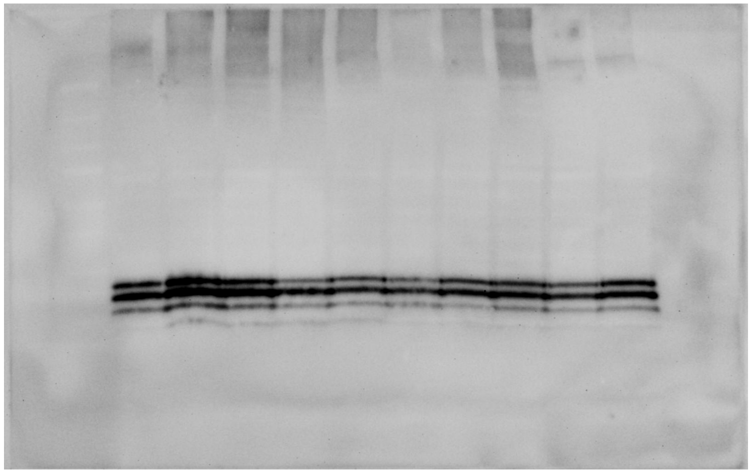

Date: 01 Mar 2024 MPT

1° β-Actin

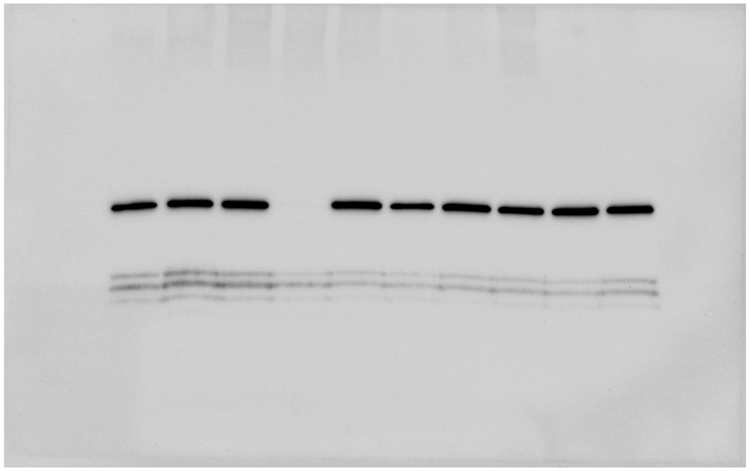

Date: 01 Mar 2024 MPT

Western Blot Imaging Form

Strip Check

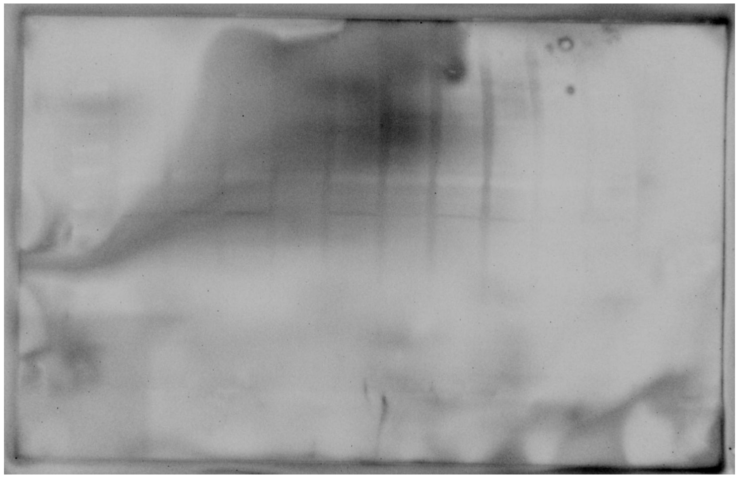

Date: 04 Mar 2024 MPT

1° MPZ

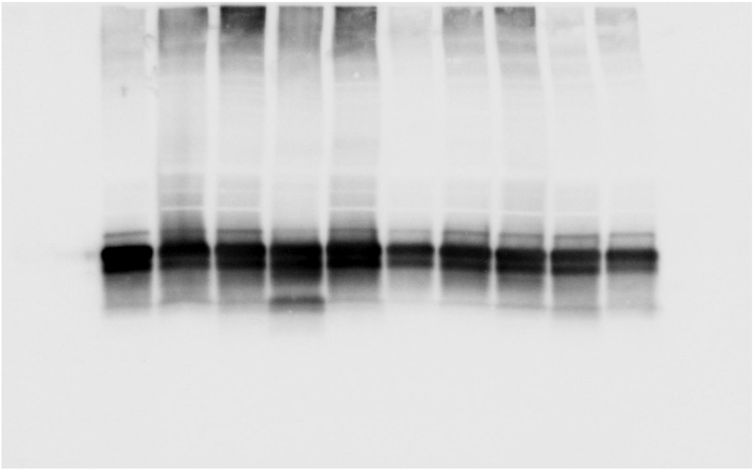

Date: 05 Mar 2024 MPT

ImageJ Quantification Box – PMP22

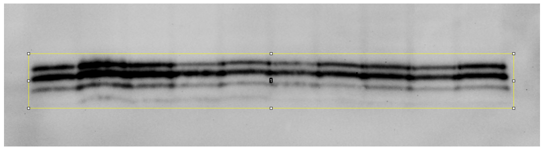

Date: 06 Mar 2024 LMW

ImageJ Quantification Box – B-Actin

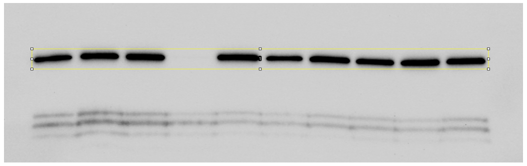

Date: 05 Mar 2024 LMW

ImageJ Quantification Box - MPZ

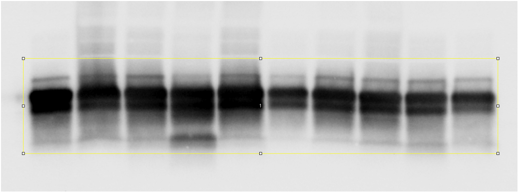

Date: 06 Mar 2024 LMW

Western Blot Imaging Form

Plot 1 Values PMP22

	Area
1	44579.2
2	69730.92
3	51129.37
4	36694.39
5	32977.73
6	31739.27
7	37399.32
8	41108.37
9	30420.27
10	41431.8

Plot 1-PMP22

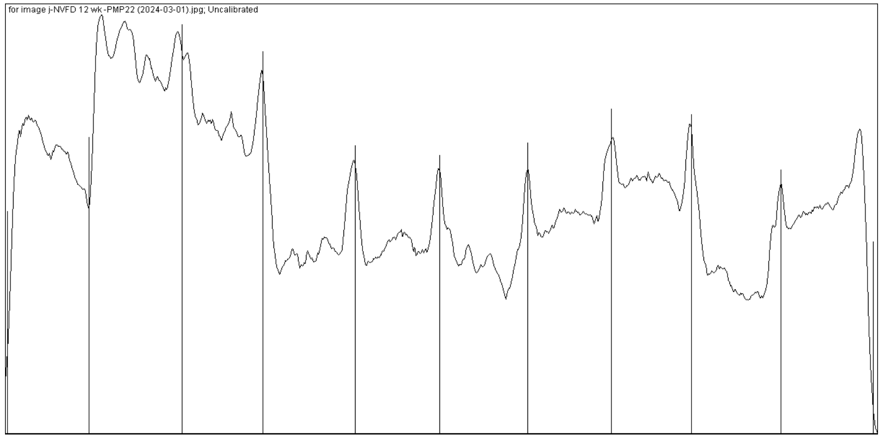

Date: 06 Mar 2024 LMW

Plot 2 Values B-Actin

	Area
1	48863.08
2	55185.49
3	58844.44
4	449.426
5	59628.39
6	41087.08
7	56358.44
8	49481.66
9	55667.85
10	54272.9

Plot 2-B-Actin

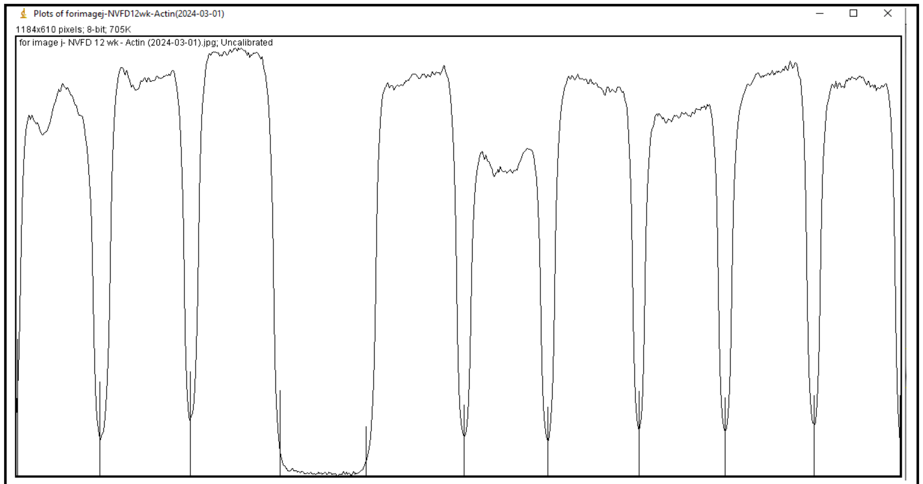

Date: 05 Mar 2024

Western Blot Imaging Form

Plot 3 Values-MPZ

	Area
1	51844.2
2	57833.66
3	55494.83
4	64191.37
5	61822.83
6	30420.76
7	46377.25
8	45783.18
9	41419.47
10	30700.13

Plot 3-MPZ

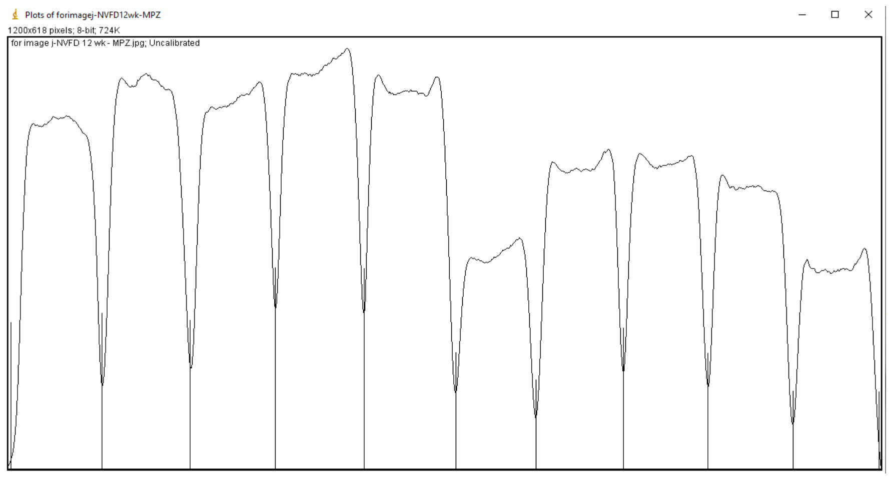

Date: 06 Mar 2024

Operator Information

**Performed By:**	**Name:** Merlin P Thangaraj	**Signature:**
	**Position/Lab:** Postdoctoral Scientist / Harper Lab	**Date:** 08 Mar 2024

**Performed By:**	**Name:** Lindsay Wallace	**Signature:**
	**Position/Lab:** Sr Research Scientist / Harper Lab	**Date:** 06 Mar 2024

Western Blot Imaging Form

**Study**	ARM101-CMT1A-NHP-001
**Timepoint**	6 week
**Tissue**	Femoral nerve - Right
**Anatomical Location** (Highlight/Circle one)	Distal (circled) Proximal N/A Other:

Stain Free Gel

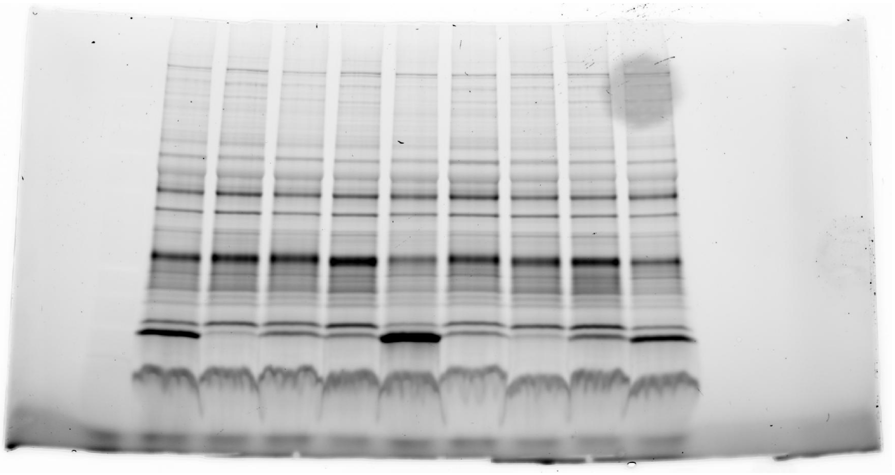

Date: 30 Apr 2025 MPT

Stain Free Membrane

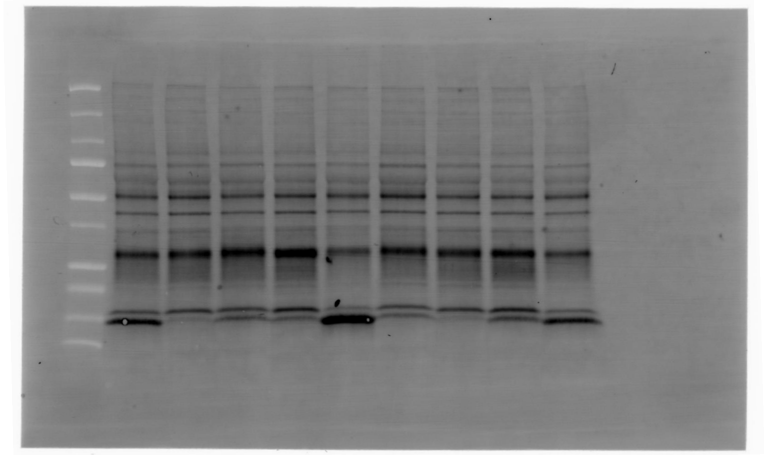

Date: 30 Apr 2025 MPT

1° PMP22

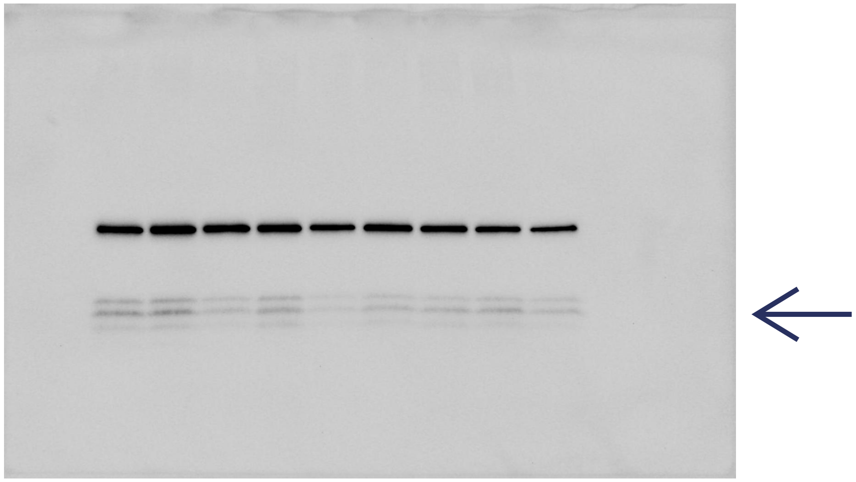

Date: 01 May 2025 MPT

1° β-Actin

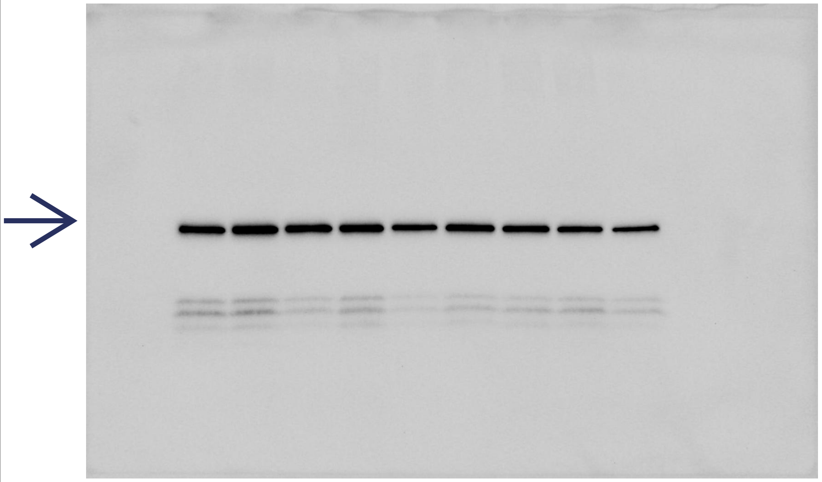

Date: 01 May 2025 MPT

Western Blot Imaging Form

Strip Check

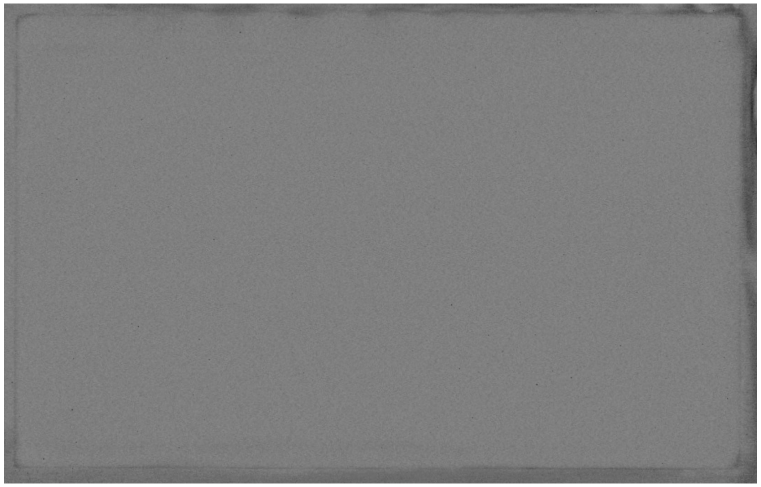

Date: 01 May 2025 MPT

1° MPZ

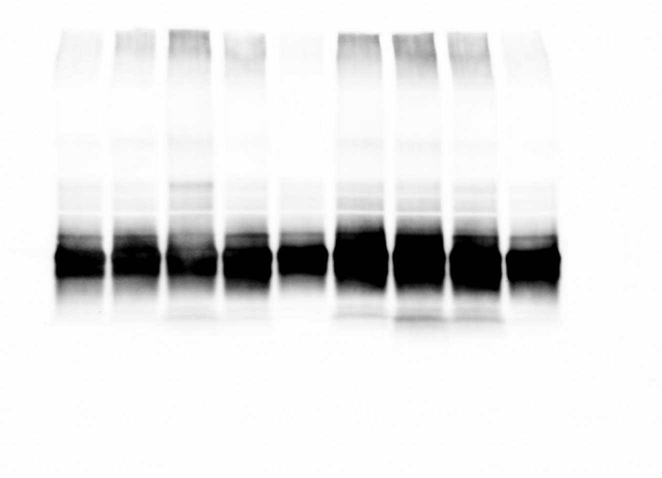

Date: 02 May 2025 MPT

ImageJ Quantification Box – PMP22

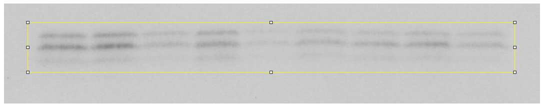

Date: 07 May 2025 LMW

ImageJ Quantification Box – B-Actin

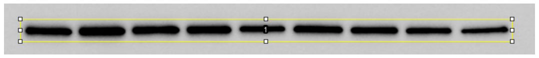

Date: 06 May 2025 LMW

ImageJ Quantification Box - MPZ

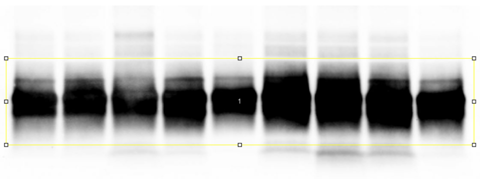

Date: 06 May 2025 LMW

Plot 1 Values PMP22

	Area
1	50159.58
2	51852.85
3	23747.78
4	37630.15
5	14114.78
6	27210.68
7	26904.85
8	32241.85
9	20904.46

Plot 1-PMP22

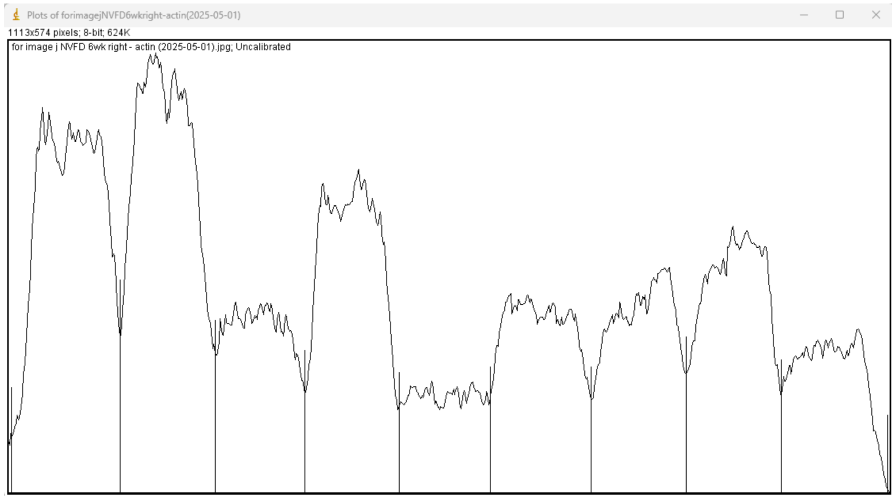

Date: 07 May 2025 LMW

Plot 2 Values B-Actin

	Area
1	45292.08
2	52222.9
3	46092.02
4	44314.42
5	35953.25
6	44509.15
7	39639.37
8	34518.42
9	29737.32

Plot 2-B-Actin

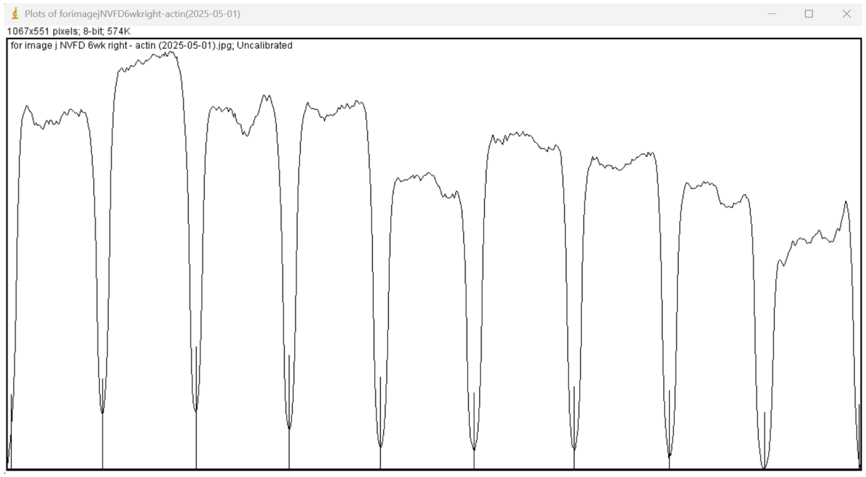

Date: 06 May 2025 LMW

Western Blot Imaging Form

Plot 3 Values-MPZ

	Area
1	35893.54
2	37435.69
3	36327.37
4	41838.83
5	35088.35
6	54079.13
7	52317.4
8	50063.76
9	42769.08

Plot 3-MPZ

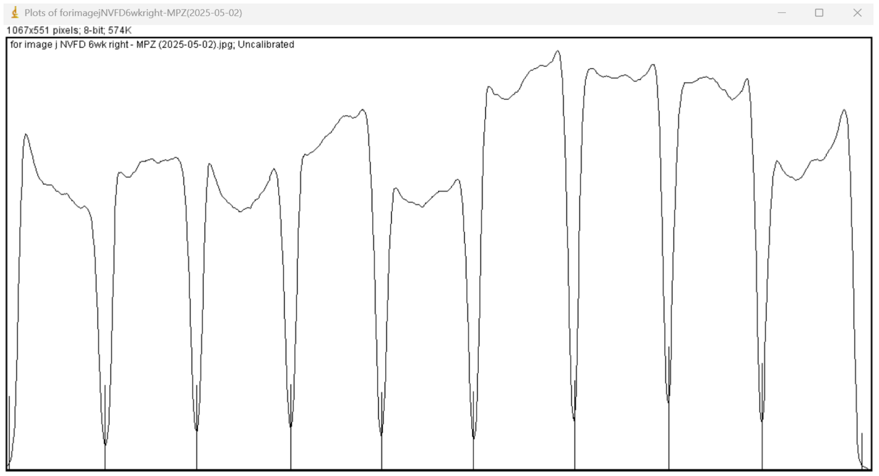

Date: 06 May 2025 LMW

Operator Information

**Performed By:**	**Name:** Merlin P Thangaraj	**Signature:**
	**Position/Lab:** Postdoctoral Scientist/Harper Lab	**Date:** 8-1-2025

**Performed By:**	**Name:** Lindsay Wallace	**Signature:**
	**Position/Lab:** Sr Research Scientist/Harper Lab	**Date:** 07 May 2025

Western Blot Imaging Form

**Study**	ARM101-CMT1A-NHP-001
**Timepoint**	12 week
**Tissue**	Femoral Nerve - Right
**Anatomical Location** (Highlight/Circle one)	Distal (circled) Proximal N/A Other:

Stain Free Gel

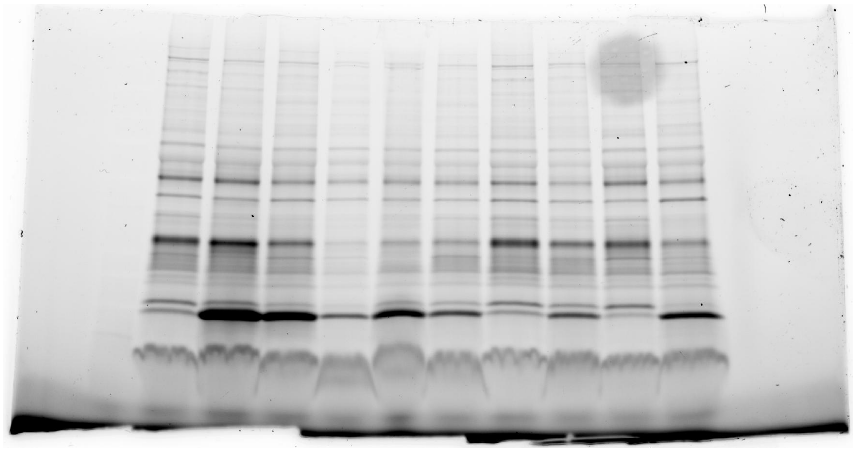

Date: 13 May 2025 MPT

Stain Free Membrane

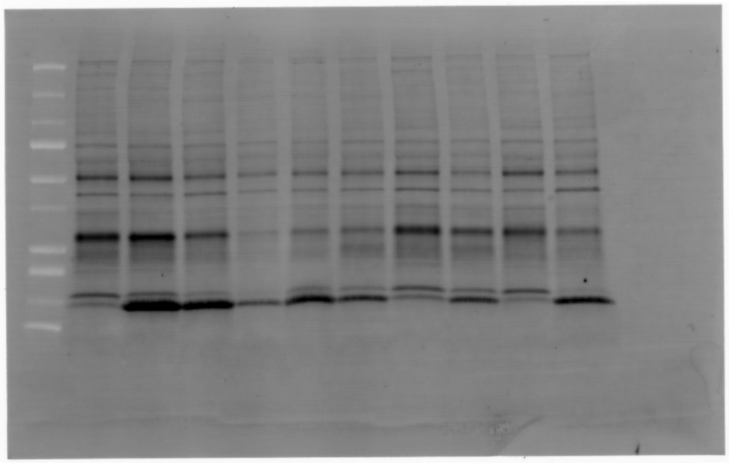

Date: 13 May 2025 MPT

1° PMP22

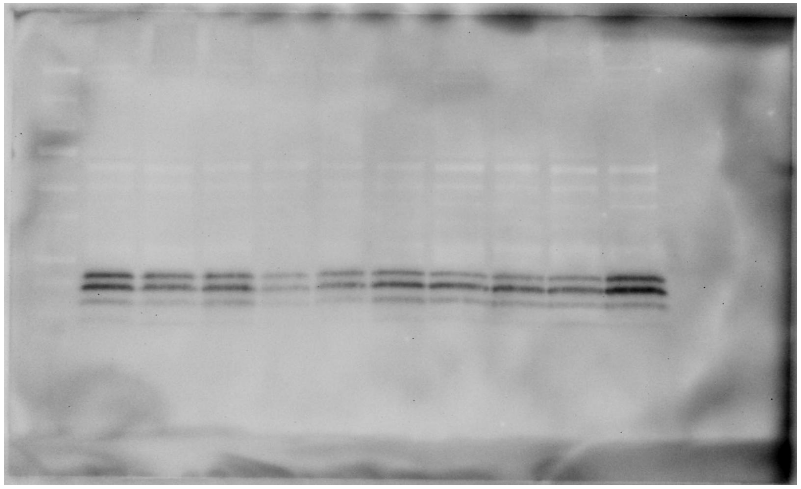

Date: 14 May 2025 MPT

1° β-Actin

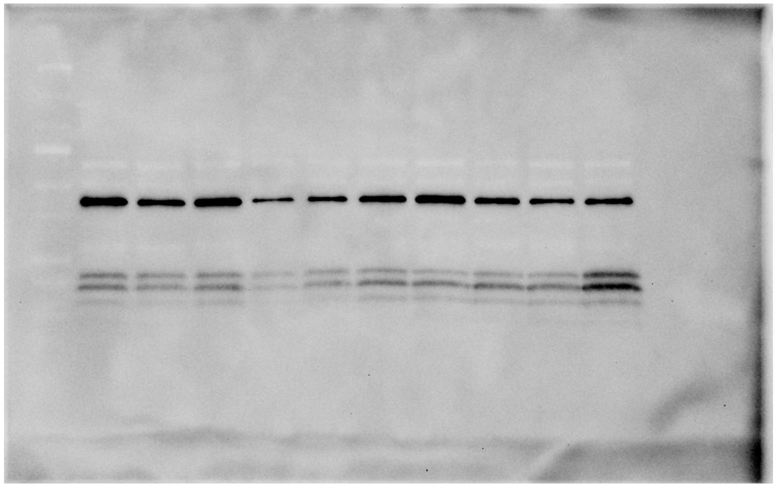

Date: 14 May 2025 MPT

Western Blot Imaging Form

Strip Check

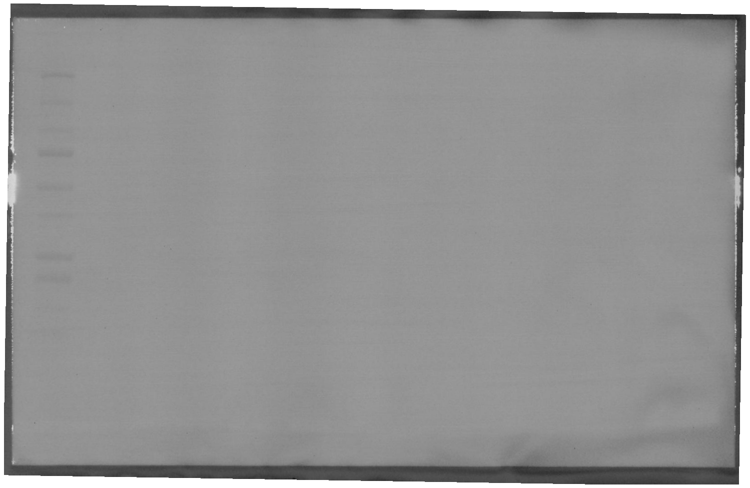

Date: 16 May 2025 MPT

1° MPZ

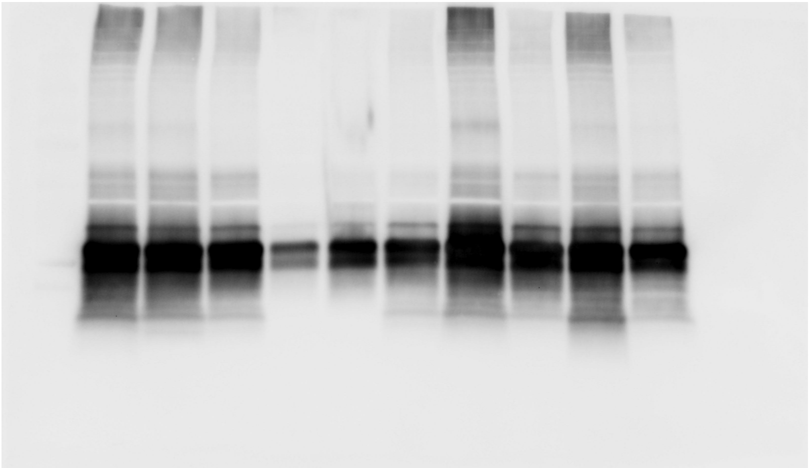

Date: 16 May 2025 MPT

ImageJ Quantification Box – PMP22

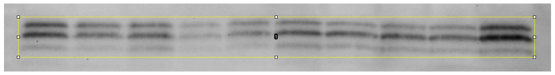

Date: 22 May 2025 LMW

ImageJ Quantification Box – B-Actin

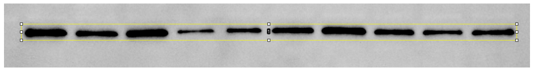

Date: 22 May 2025 LMW

ImageJ Quantification Box - MPZ

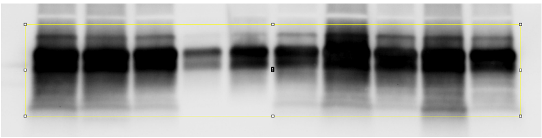

Date: 22 May 2025 LMW

Western Blot Imaging Form

Plot 1 Values PMP22

	Area
1	37004.32
2	20971.15
3	26153.27
4	6827.125
5	16727.08
6	32730.15
7	27250.97
8	31793.61
9	28699.61
10	69052.63

Plot 1-PMP22

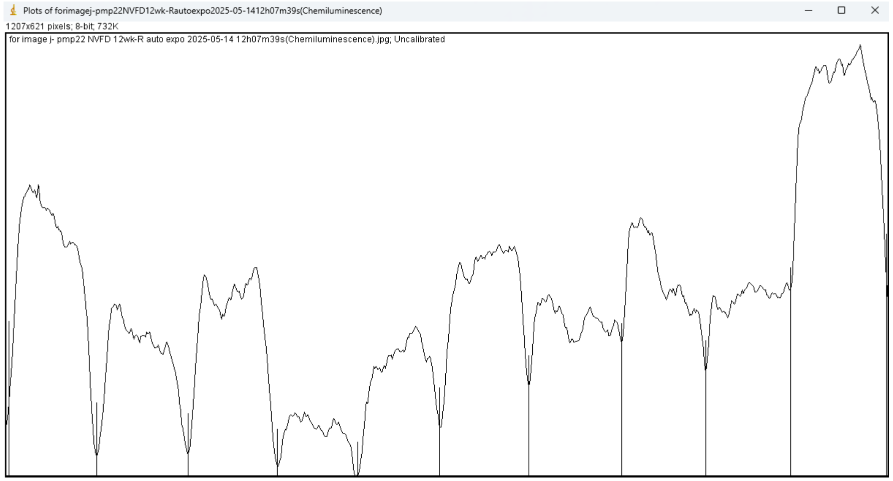

Date: 22 May 2025 LMW

Plot 2 Values B-Actin

	Area
1	53151.73
2	41018.56
3	52450.97
4	20500.08
5	27001.78
6	40132.97
7	50414.39
8	36557.32
9	29361.32
10	39096.02

Plot 2-B-Actin

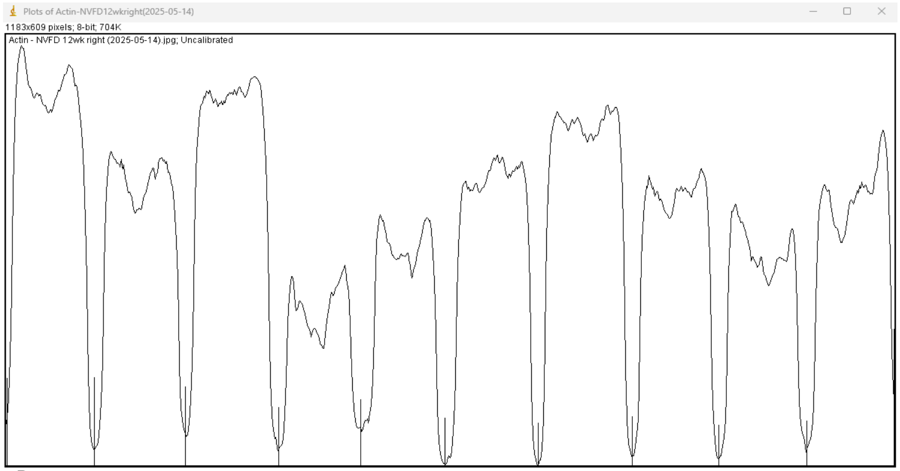

Date: 22 May 2025 LMW

Western Blot Imaging Form

Plot 3 Values-MPZ

	Area
1	111452.7
2	103110.4
3	79983.51
4	20003.69
5	46727.44
6	57422.51
7	112089
8	78540.56
9	105607.2
10	74065

Plot 3-MPZ

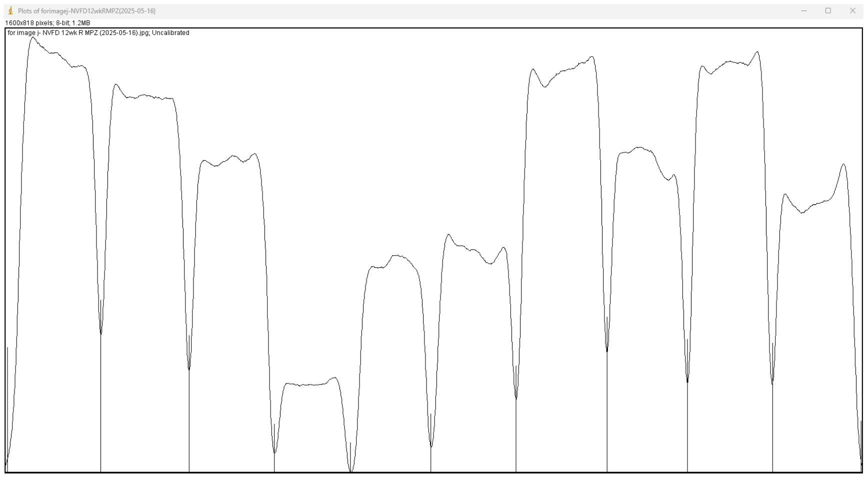

Date: 22 May 2025 LMW

Operator Information

Performed By:	Name: Merlin P Thangaraj	Signature: [signature]
	Position/Lab: Postdoctoral Scientist / Harper Lab	Date: 8-1-2025

Performed By:	Name: Lindsay Wallace	Signature: [signature]
	Position/Lab: Sr Research Scientist / Harper Lab	Date: 22 May 2025

Western Blot Imaging Form

**Study**	ARM101-CMT1A-NHP-001
**Timepoint**	6 week
**Tissue**	Femoral nerve
**Anatomical Location** (Highlight/Circle one)	Distal / Proximal (circled) / N/A<br>Other:

Stain Free Gel

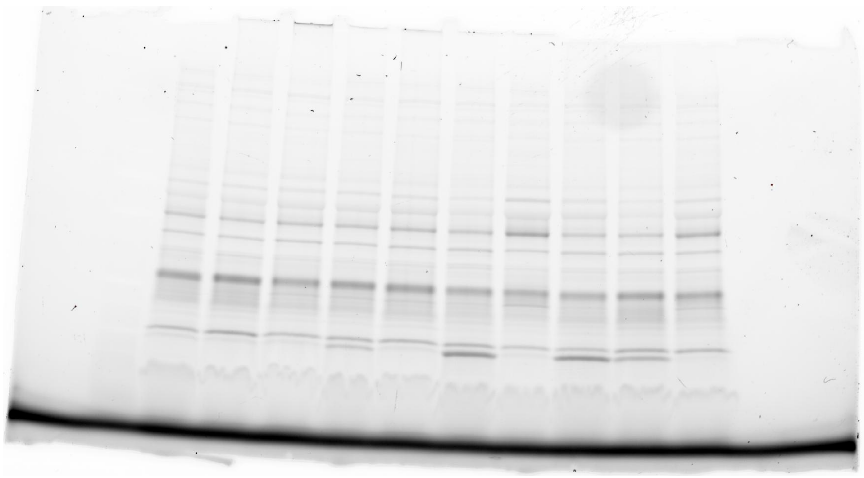

Date: 29 May 2024 MPT

Stain Free Membrane

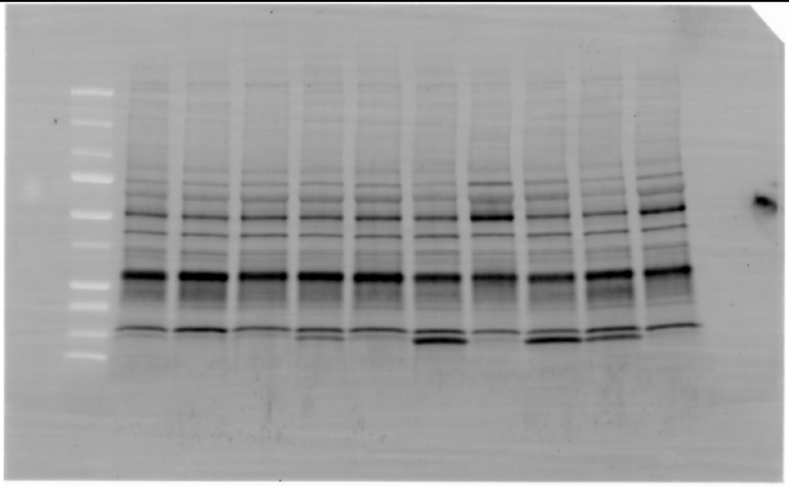

Date: 29 May 2024 MPT

1° PMP22

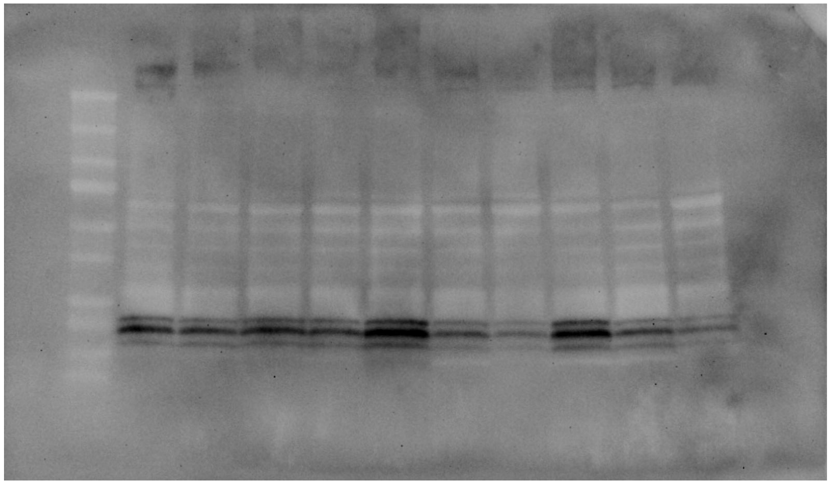

Date: 30 May 2024 MPT

1° β-Actin

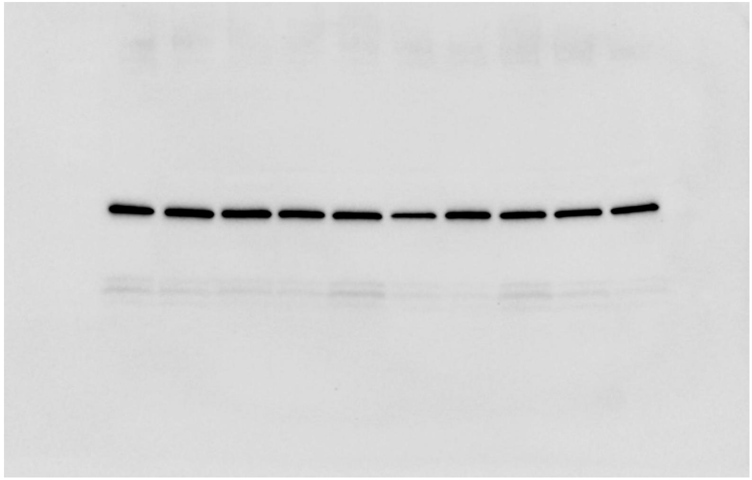

Date: 30 May 2024 MPT

Western Blot Imaging Form

Strip Check

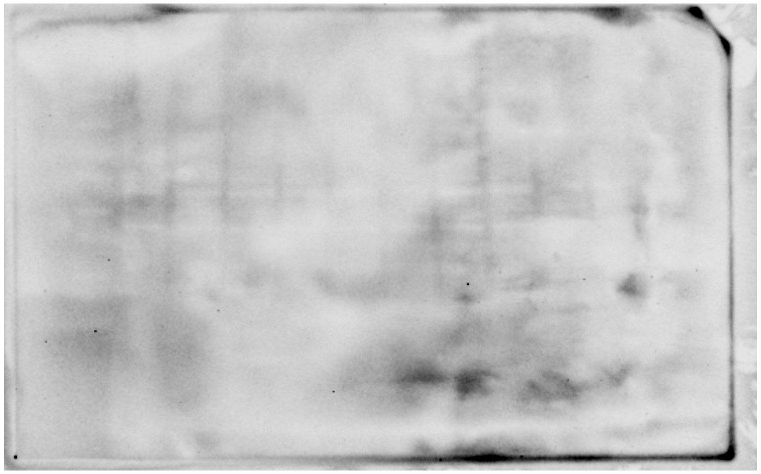

Date: 14 Jun 2024 MPT

1° MPZ

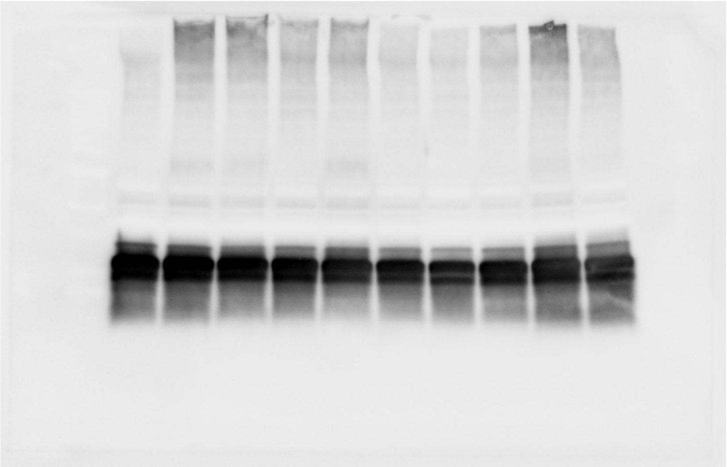

Date: 14 Jun 2024 MPT

ImageJ Quantification Box – PMP22

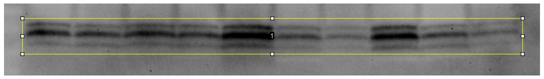

Date: 19 Jun 2024 LMW

ImageJ Quantification Box – B-Actin

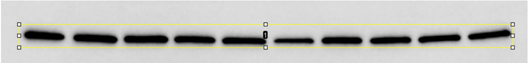

Date: 19 Jun 2024 LMW

ImageJ Quantification Box - MPZ

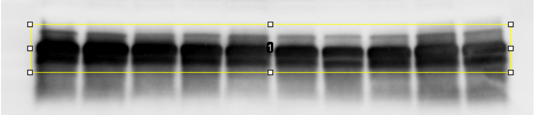

Date: 19 Jun 2024 LMW

Western Blot Imaging Form

Plot 1 Values PMP22

	Area
1	31442.44
2	32219.85
3	45600.78
4	39618.9
5	78440.48
6	27318.15
7	12735.59
8	50989.7
9	23657.49
10	23896.97

Plot 1-PMP22

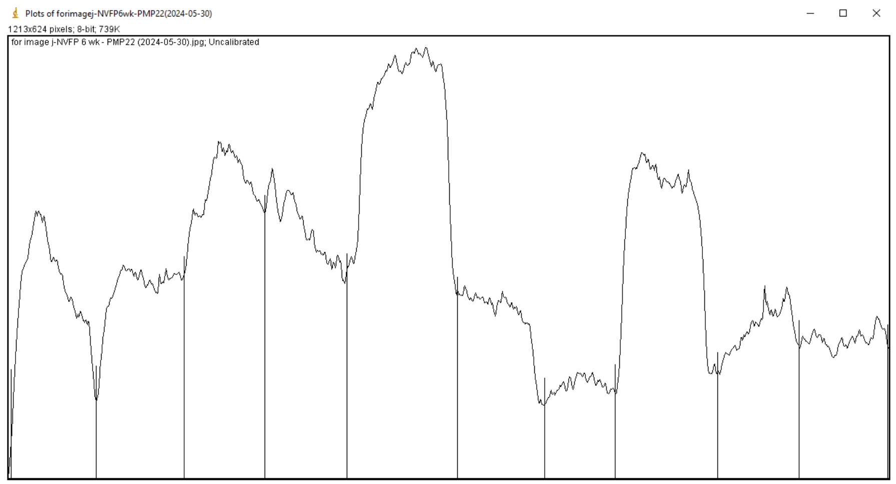

Date: 19 Jun 2024 LMW

Plot 2 Values B-Actin

	Area
1	51642.78
2	57341.15
3	55814.78
4	48271.47
5	47777.85
6	32298.9
7	45686.61
8	43840.9
9	43161.2
10	44380.37

Plot 2-B-Actin

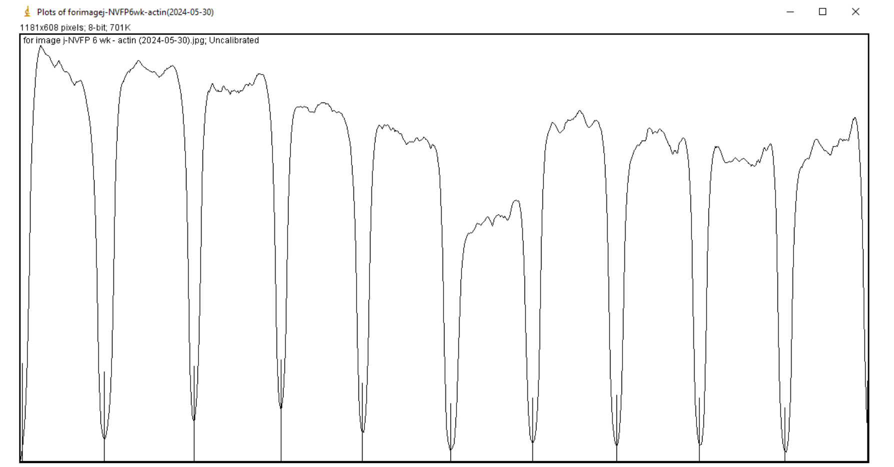

Date: 19 Jun 2024 LMW

Plot 3 Values-MPZ

	Area
1	58342.66
2	57970.18
3	53946.05
4	47351.76
5	48443.49
6	42366.47
7	36444
8	42781.28
9	49833.76
10	46782.42

Plot 3-MPZ

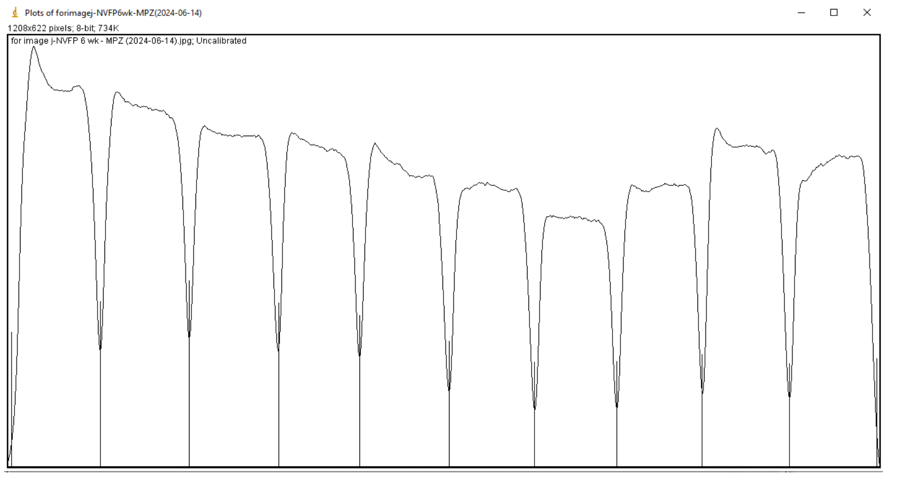

Date: 19 Jun 2024 LMW

Operator Information

Performed By:	**Name:** Merlin P Thangaraj	**Signature:** [signature]
	**Position/Lab:** Postdoctoral Scientist / Harper Lab	**Date:** 27 Jun 2024

Performed By:	**Name:** Lindsay Wallace	**Signature:** [signature]
	**Position/Lab:** Senior Research Scientist/Harper Lab	**Date:** 19 Jun 2024 LMW

Western Blot Imaging Form

**Study**	ARM101-CMT1A-NHP-001
**Timepoint**	12 week
**Tissue**	Femoral nerve
**Anatomical Location** (Highlight/Circle one)	Distal ~~~ Proximal (circled) ~~~ N/A<br>Other:

Stain Free Gel

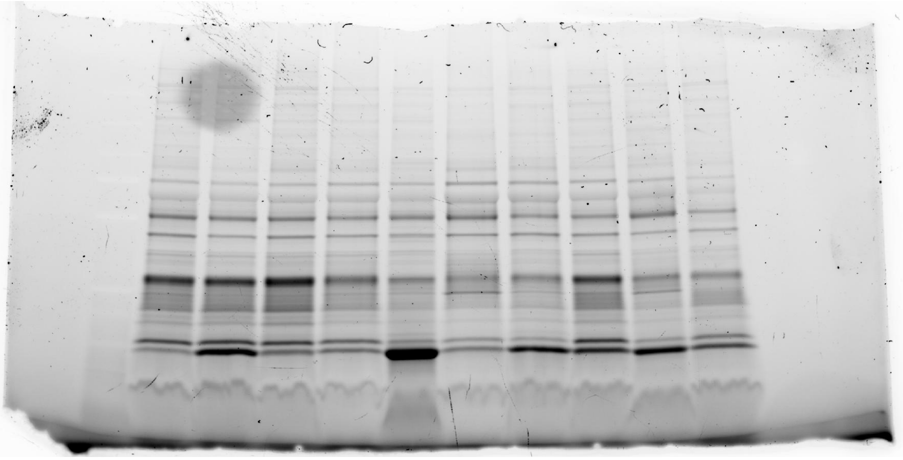

Date: 29 Feb 2024 MPT

Stain Free Membrane

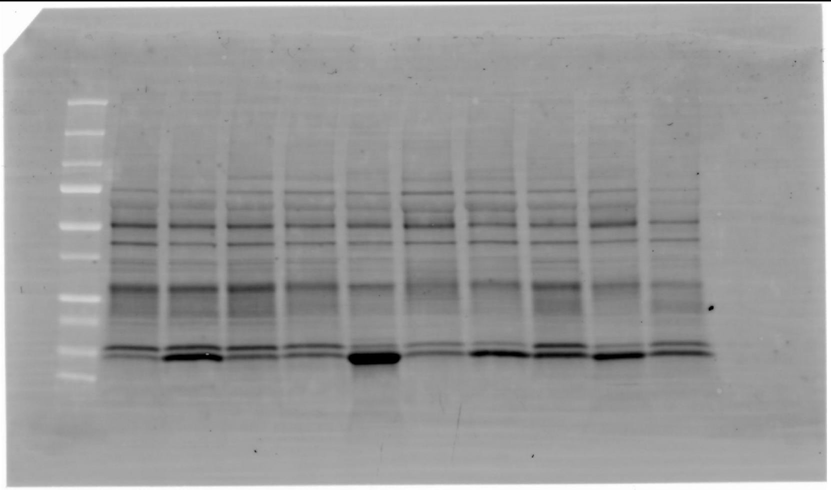

Date: 29 Feb 2024 MPT

1° PMP22

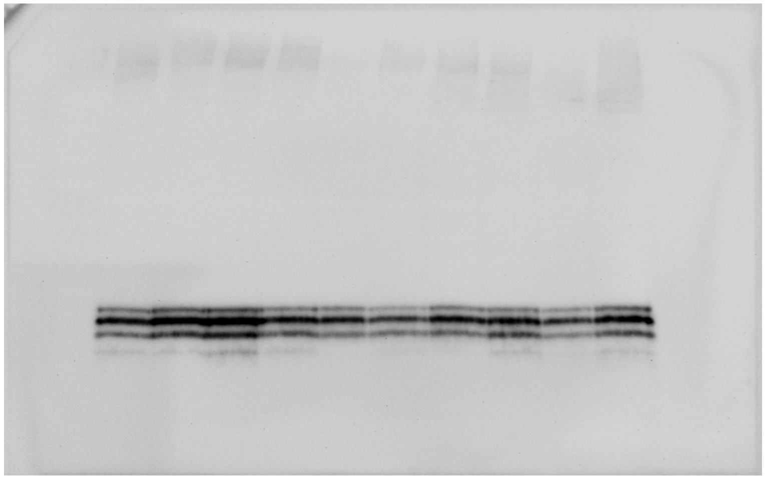

Date: 01 Mar 2024 MPT

1° β-Actin

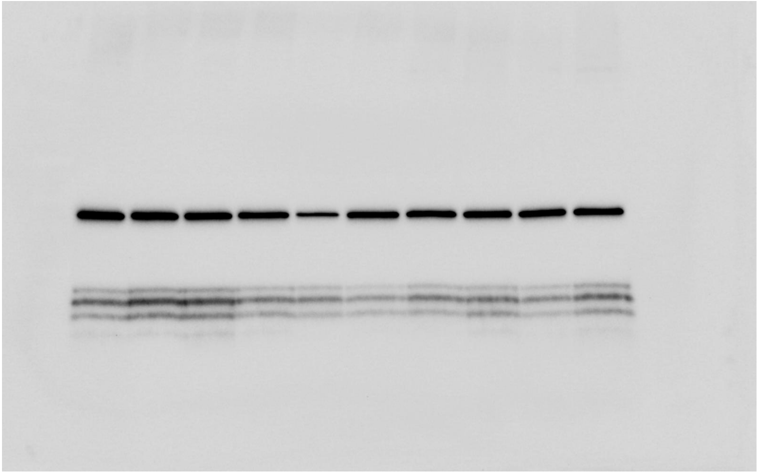

Date: 01 Mar 2024 MPT

Western Blot Imaging Form

Strip Check

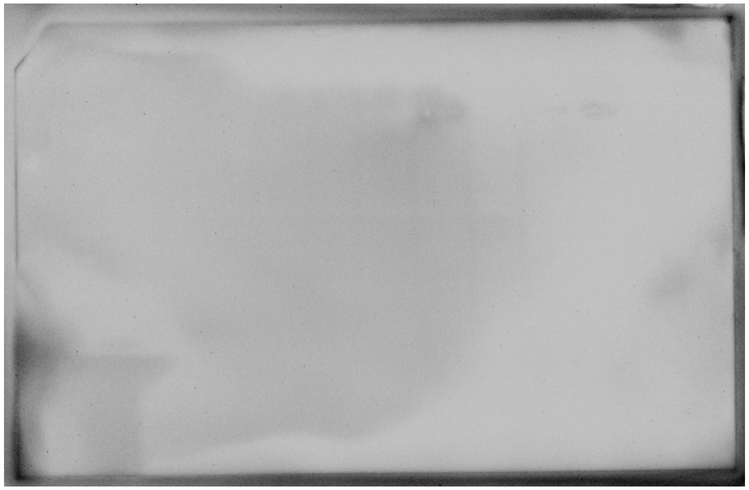

Date: 04 Mar 2024 MPT

1° MPZ

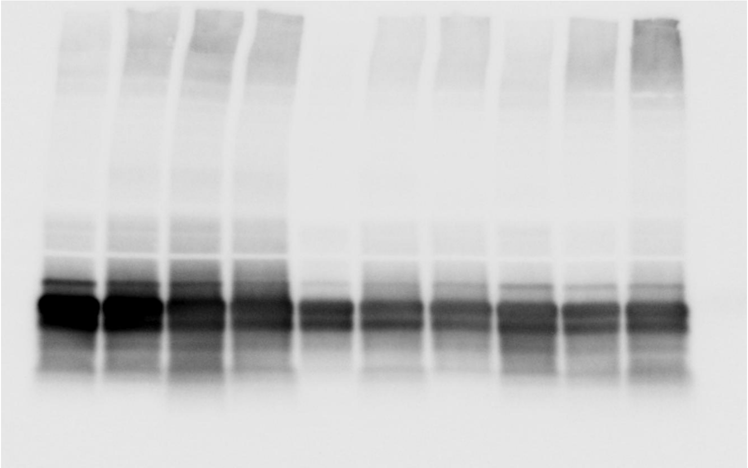

Date: 05 Mar 2024 MPT

ImageJ Quantification Box – PMP22

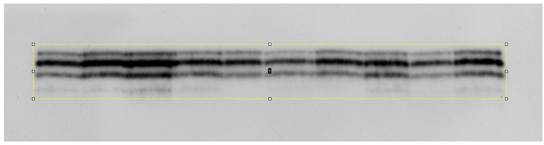

Date: 06 Mar 2024 LMW

ImageJ Quantification Box – B-Actin

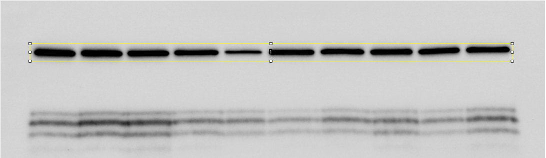

Date: 06 Mar 2024 LMW

ImageJ Quantification Box - MPZ

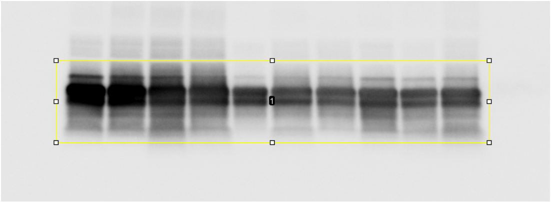

Date: 06 Mar 2024 LMW

Plot 1 Values PMP22

	Area
1	39248.32
2	55859.15
3	66453.27
4	44095.85
5	30645.3
6	31379.97
7	40018.85
8	44937.85
9	24571.25
10	48527.92

Plot 1-PMP22

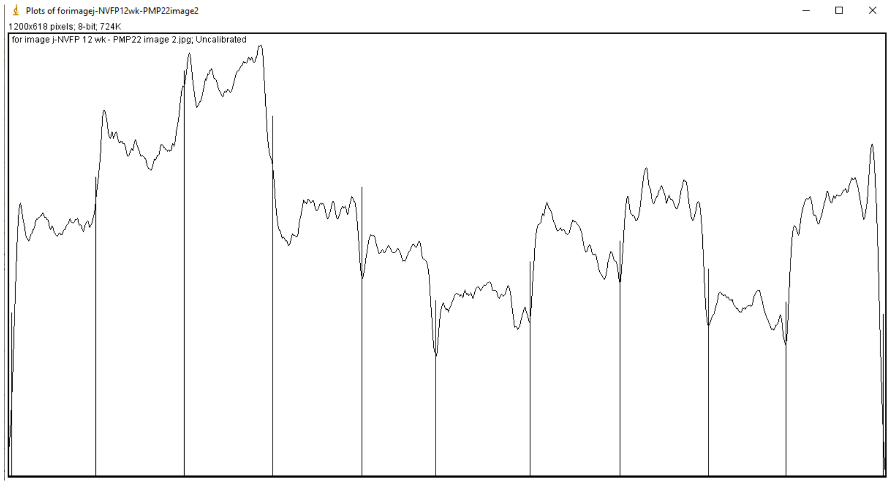

Date: 06 Mar 2024 LMW

Plot 2 Values B-Actin

	Area
1	55570.08
2	53512.61
3	49067.49
4	46377.44
5	23602.83
6	47226.85
7	45516.73
8	46224.37
9	44920.49
10	45409.2

Plot 2-B-Actin

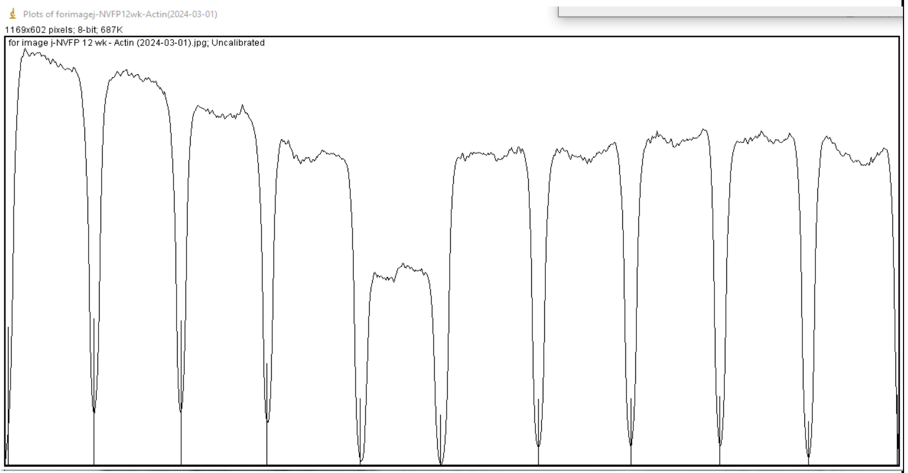

Date: 06 Mar 2024 LMW

Plot 3 Values-MPZ

	Area
1	60041.2
2	62057.3
3	61082.47
4	53557.78
5	28055.1
6	36652.25
7	32360.88
8	40963.35
9	31626.28
10	40230.97

Plot 3-MPZ

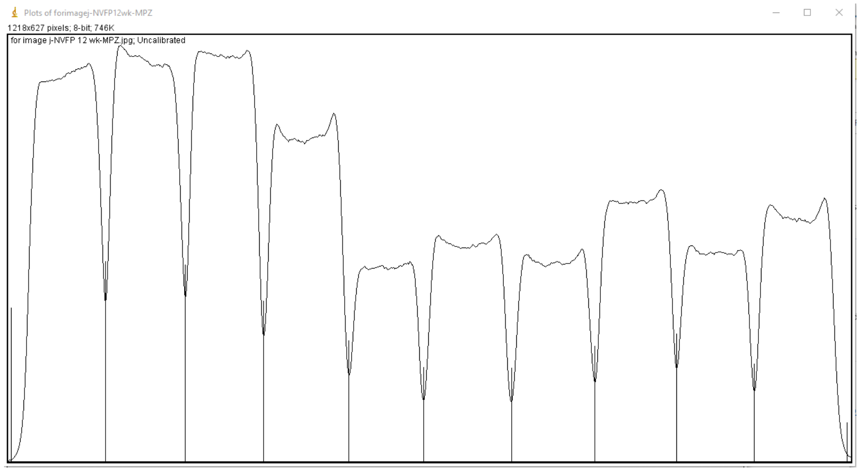

Date: 06 Mar 2024 LMW

Operator Information

Performed By:	Name: Merlin P Thangaraj	Signature: T. Merlin Pramila [signature]
	Position/Lab: Postdoctoral Scientist / Harper Lab	Date: 08 Mar 2024

Performed By:	Name: Lindsay Wallace	Signature: [signature]
	Position/Lab: Sr Research Scientist / Harper Lab	Date: 06 Mar 2024

Western Blot Imaging Form

Study	ARM101-CMT1A-NHP-001
Timepoint	6 week
Tissue	Femoral nerve - Right
Anatomical Location (Highlight/Circle one)	Distal Proximal (circled) N/A Other:

Stain Free Gel

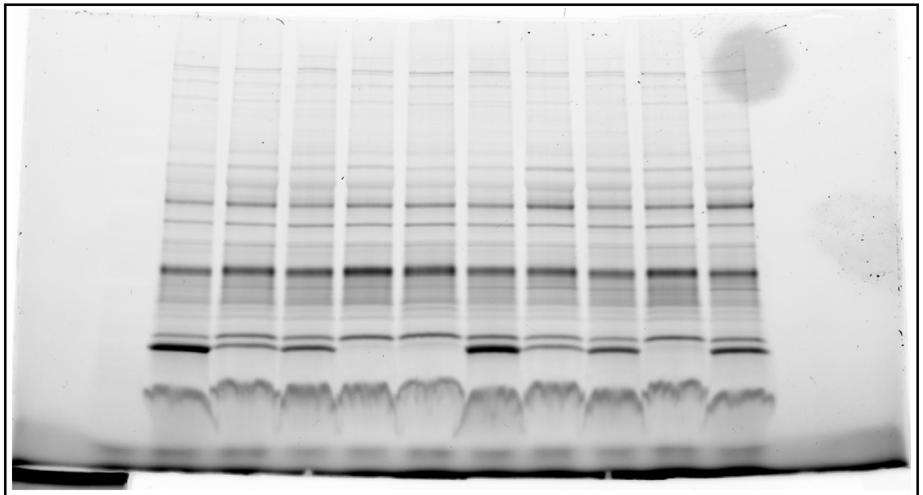

Date: 30 Apr 2025 MPT

Stain Free Membrane

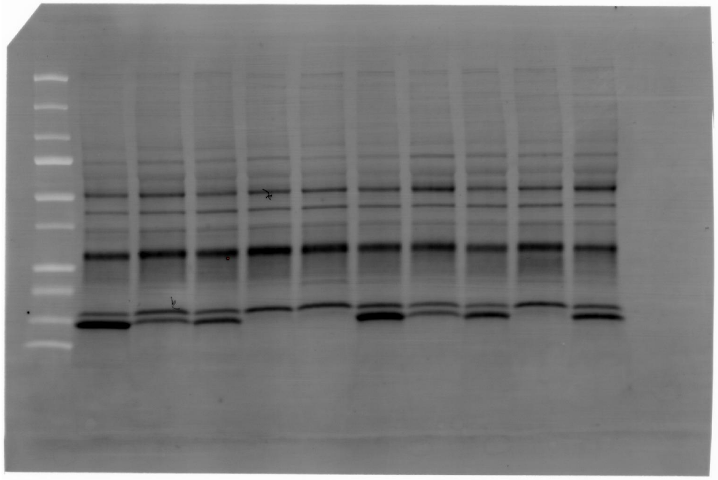

Date: 30 Apr 2025 MPT

1° PMP22

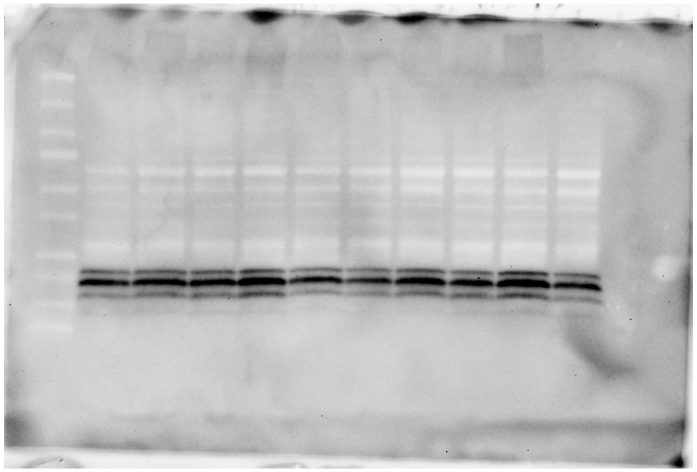

Date: 01 May 2025 MPT

1° β-Actin

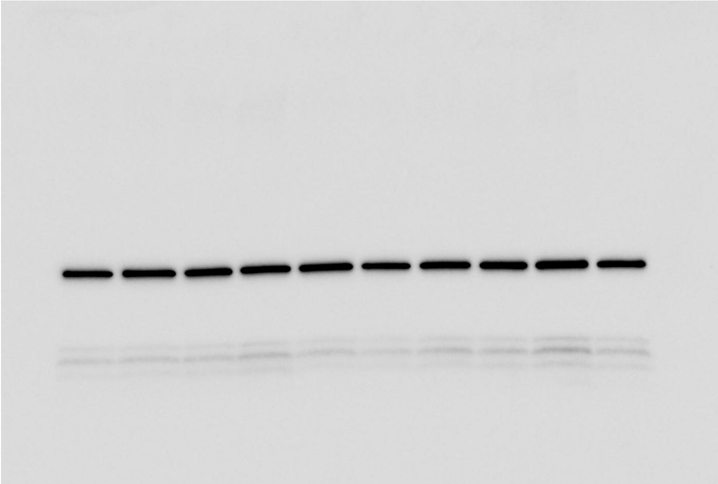

Date: 01 May 2025 MPT

Western Blot Imaging Form

Strip Check

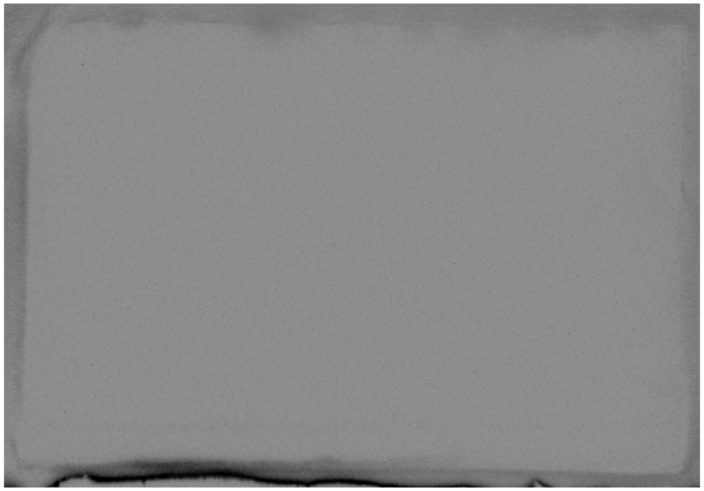

Date: 01 May 2025 MPT

1° MPZ

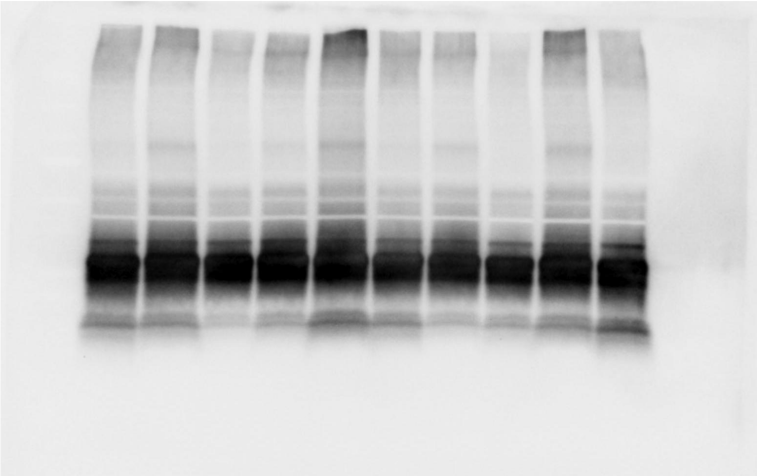

Date: 02 May 2025 MPT

ImageJ Quantification Box – PMP22

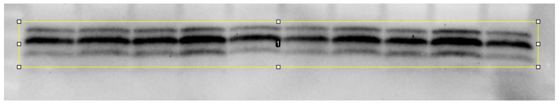

Date: 06 May 2025 LMW

ImageJ Quantification Box – B-Actin

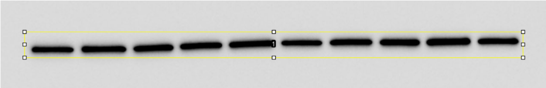

Date: 06 May 2025 LMW

ImageJ Quantification Box - MPZ

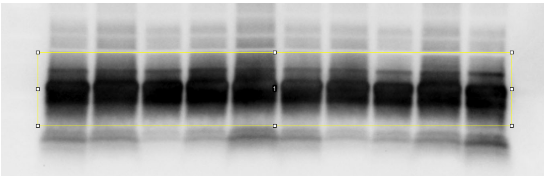

Date: 06 May 2025 LMW

Plot 1 Values PMP22

	Area
1	37308.49
2	47484.49
3	40165.18
4	45115.25
5	31311.49
6	30994
7	43713.83
8	36304.47
9	52438.37
10	37470.66

Plot 1-PMP22

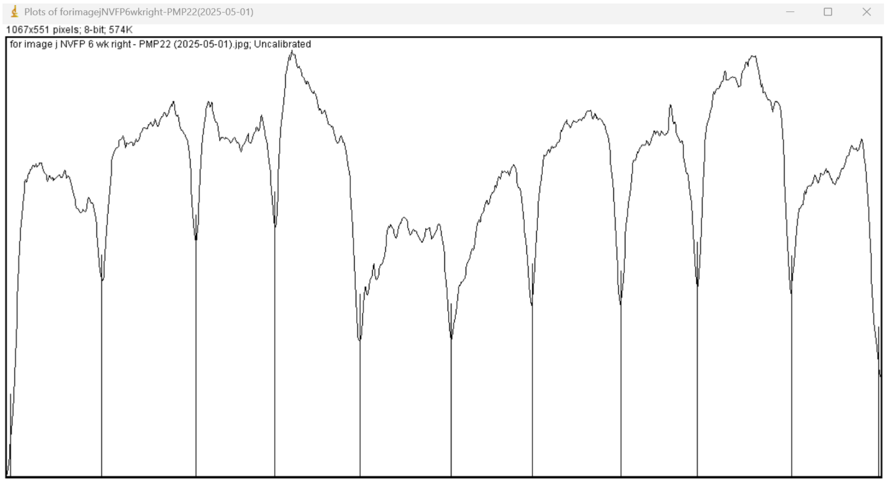

Date: 06 May 2025 LMW

Plot 2 Values B-Actin

	Area
1	39507.25
2	45703.54
3	42533.3
4	43924.42
5	47083.37
6	37466.88
7	42474.71
8	42078.47
9	48799
10	38617.18

Plot 2-B-Actin

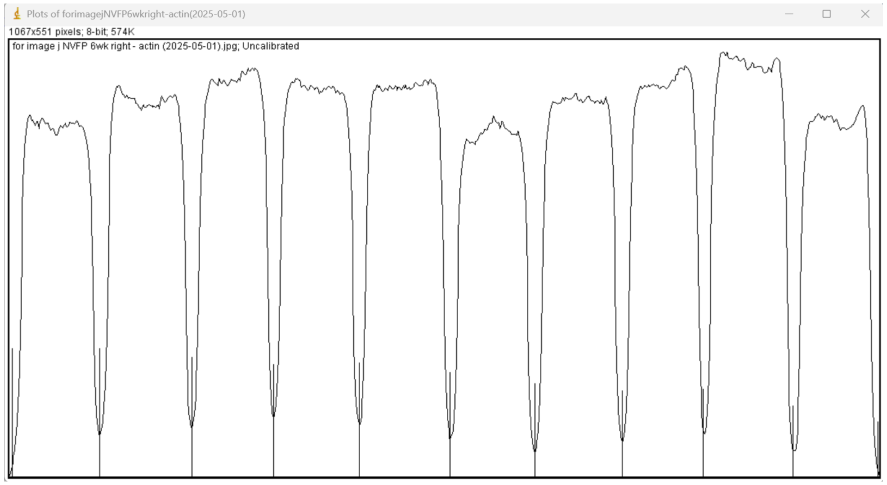

Date: 06 May 2025 LMW

Plot 3 Values-MPZ

	Area
1	48180.23
2	50309.35
3	41308.28
4	47872.03
5	51439.81
6	39783.45
7	42408.93
8	36635.28
9	48573.35
10	45793.3

Plot 3-MPZ

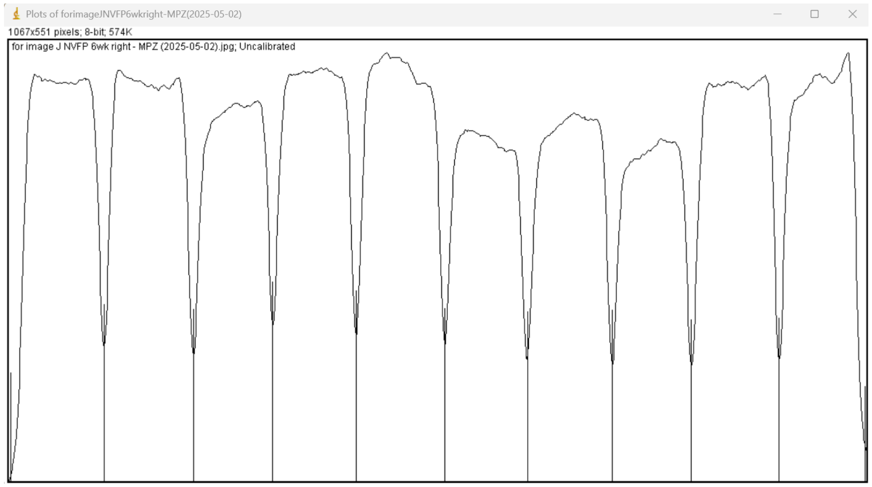

Date: 06 May 2025 LMW

Operator Information

**Performed By:**	**Name:** Merlin P Thangaraj	**Signature:** [signature]
	**Position/Lab:** Postdoctoral Scientist/Harper Lab	**Date:** 8-1-2025

**Performed By:**	**Name:** Lindsay Wallace	**Signature:** [signature]
	**Position/Lab:** Sr Research Scientist/Harper Lab	**Date:** 06 May 2025

Western Blot Imaging Form

**Study**	ARM101-CMT1A-NHP-001
**Timepoint**	12 week
**Tissue**	Femoral nerve Right
**Anatomical Location** (Highlight/Circle one)	Distal / Proximal (circled) / N/A<br>Other:

Stain Free Gel

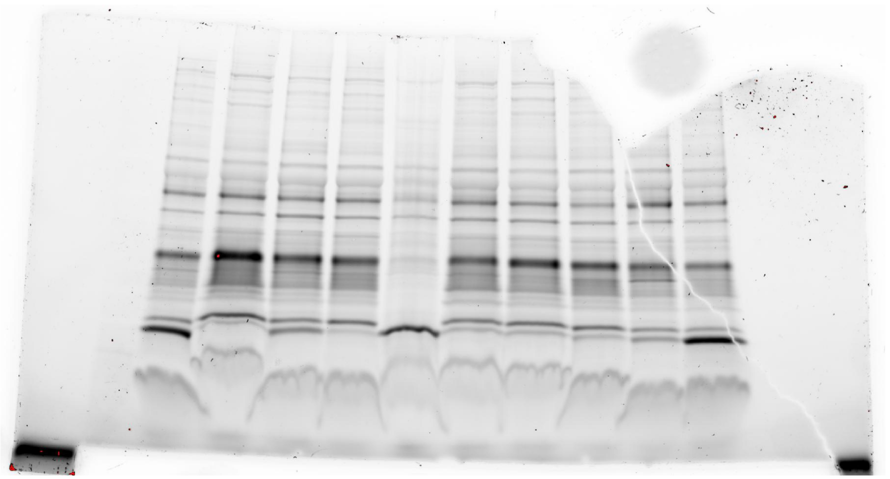

Date: 13 May 2025 MPT

Stain Free Membrane

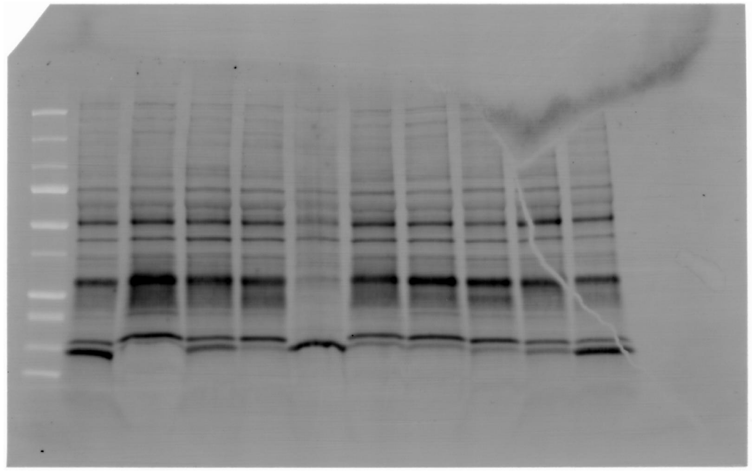

Date: 13 May 2025 MPT

1° PMP22

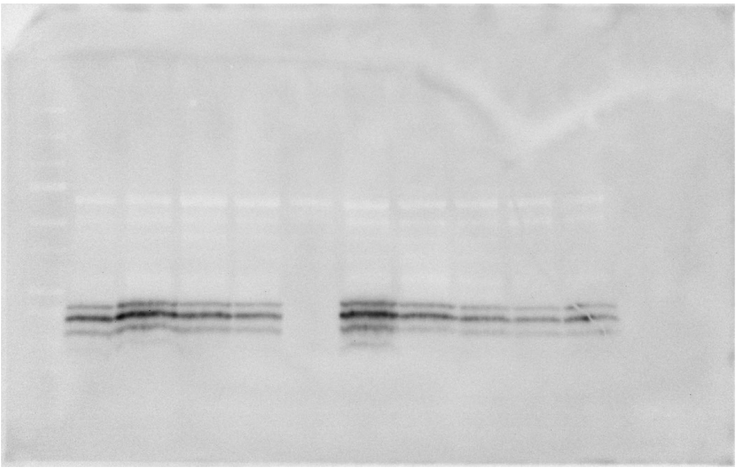

Date: 14 May 2025 MPT

1° β-Actin

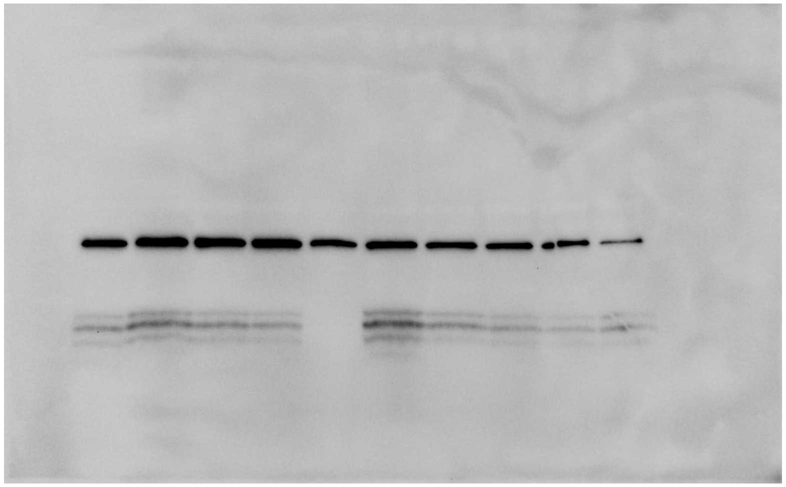

Date: 14 May 2025 MPT

Western Blot Imaging Form

Strip Check

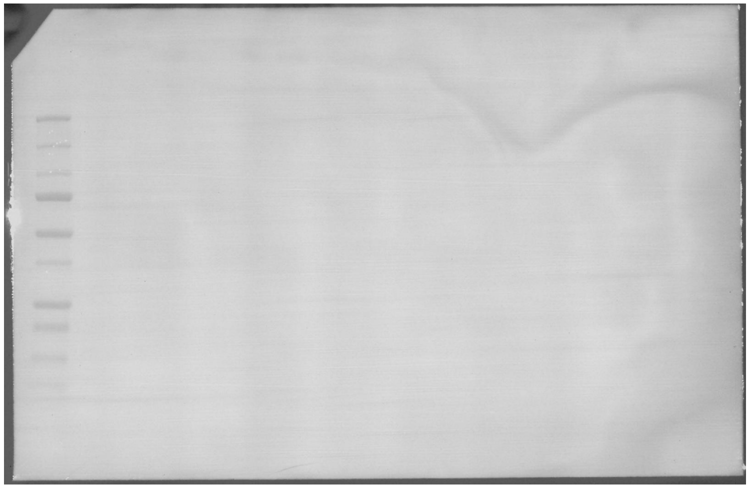

Date: 16 May 2025 MPT

1° MPZ

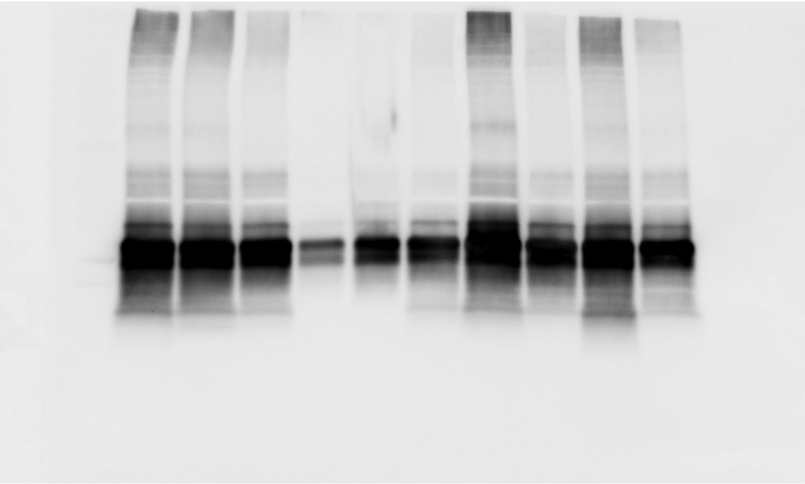

Date: 16 May 2025 MPT

ImageJ Quantification Box – PMP22

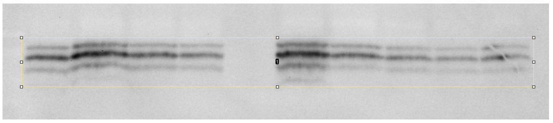

Date: 22 May 2025 LMW

ImageJ Quantification Box – B-Actin

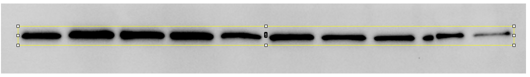

Date: 22 May 2025 LMW

ImageJ Quantification Box - MPZ

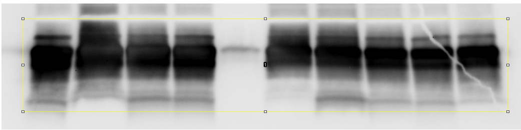

Date: 22 May 2025 LMW

Plot 1 Values PMP22

	Area
1	32084.2
2	66322.53
3	33095.32
4	20174.78
5	3740.782
6	71291.53
7	30295.68
8	23005.73
9	18473.66
10	29302.51

Plot 1-PMP22

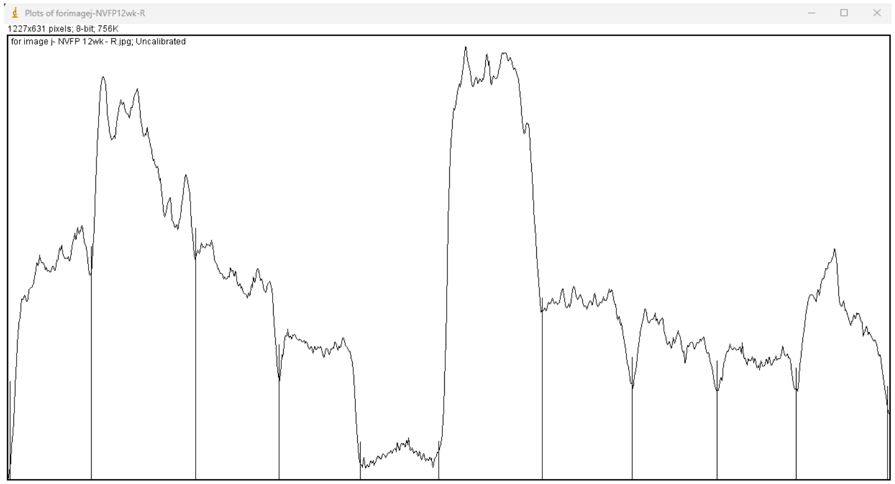

Date: 22 May 2025 LMW

Plot 2 Values B-Actin

	Area
1	40814.25
2	59989.68
3	57094.9
4	58030.15
5	38541.49
6	48500.85
7	40713.56
8	36795.32
9	31460.85
10	12106.13

Plot 2-B-Actin

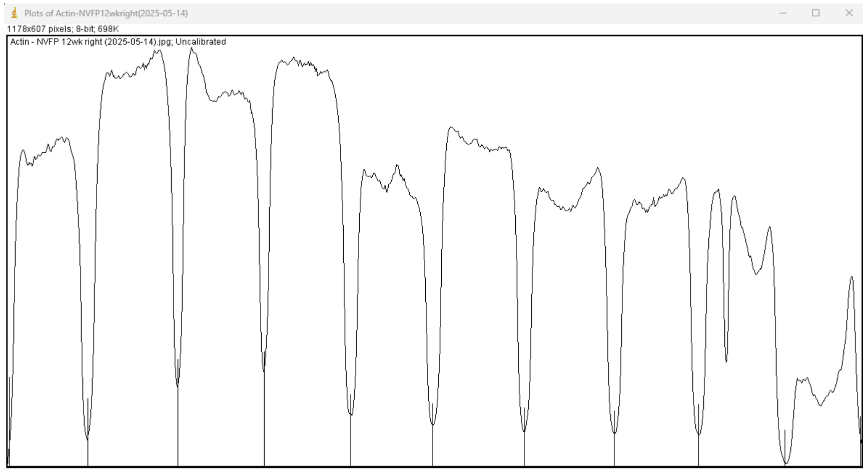

Date: 22 May 2025 LMW

Plot 3 Values-MPZ

	Area
1	103014.2
2	109597.6
3	93005.75
4	94633.39
5	2729.598
6	76848.7
7	100880.8
8	85568.9
9	78902.56
10	78835.46

Plot 3-MPZ

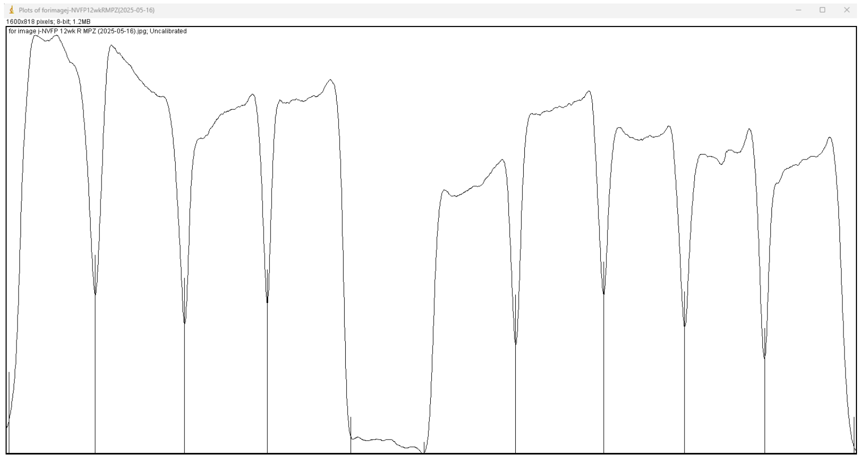

Date: 06 Mar 2024 LMW

Operator Information

Performed By:	Name: Merlin P Thangaraj	Signature: [signature]
	Position/Lab: Postdoctoral Scientist / Harper Lab	Date: 8-1-2025

Performed By:	Name: Lindsay Wallace	Signature: [signature]
	Position/Lab: Sr Research Scientist / Harper Lab	Date: 22 May 2025

Western Blot Imaging Form

**Study**	ARM101-CMT1A-NHP-001
**Timepoint**	12 week
**Tissue**	Median Nerve
**Anatomical Location** (Highlight/Circle one)	Distal (circled) Proximal N/A<br>Other:

Stain Free Gel

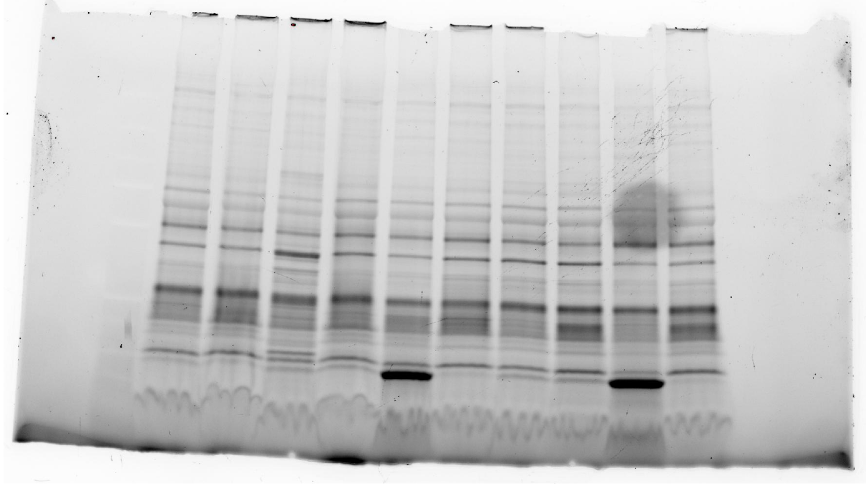

Date: MPT 14 Mar 2024

Stain Free Membrane

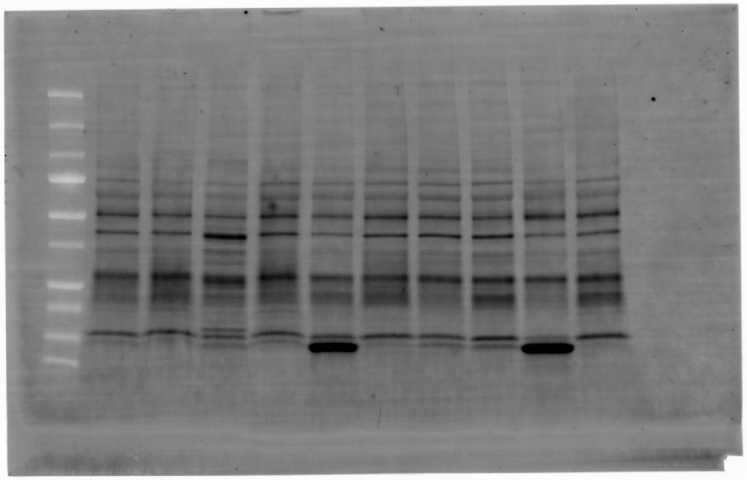

Date: MPT 14 Mar 2024

1° PMP22

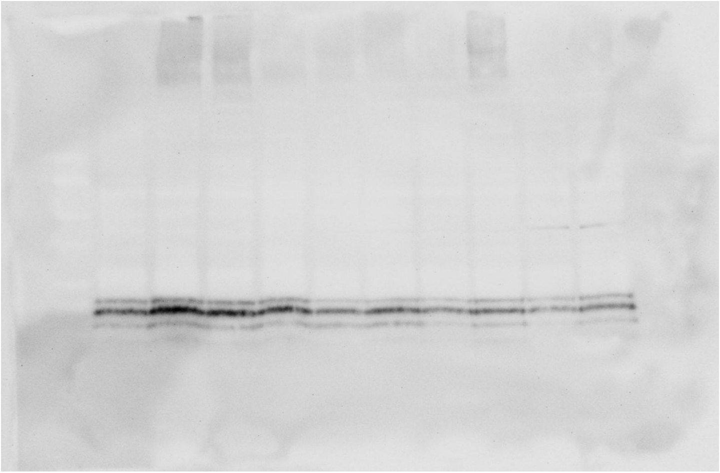

Date: MPT 20 Mar 2024

1° β-Actin

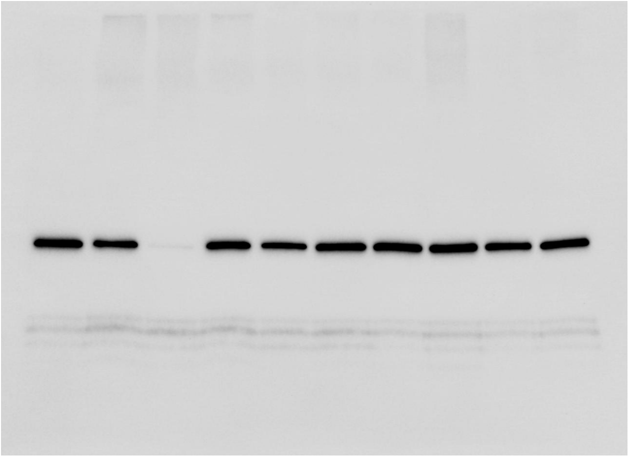

Date: MPT 15 Mar 2024

Western Blot Imaging Form

Strip Check

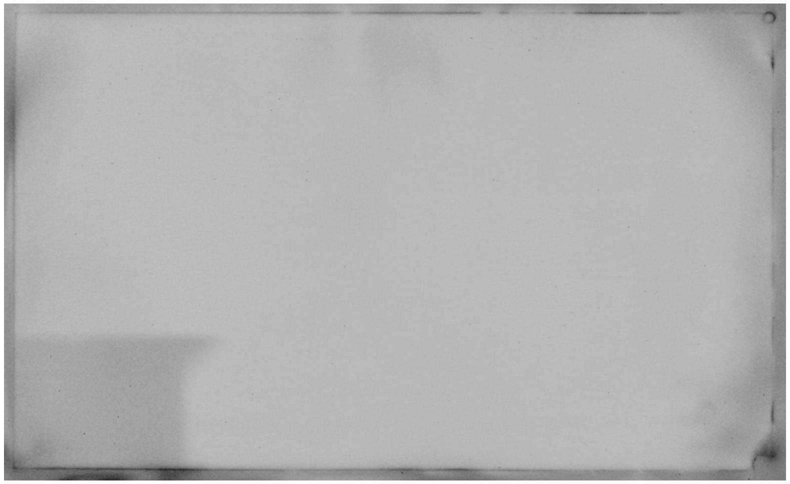

Date: MPT 19 Mar 2024

1° MPZ

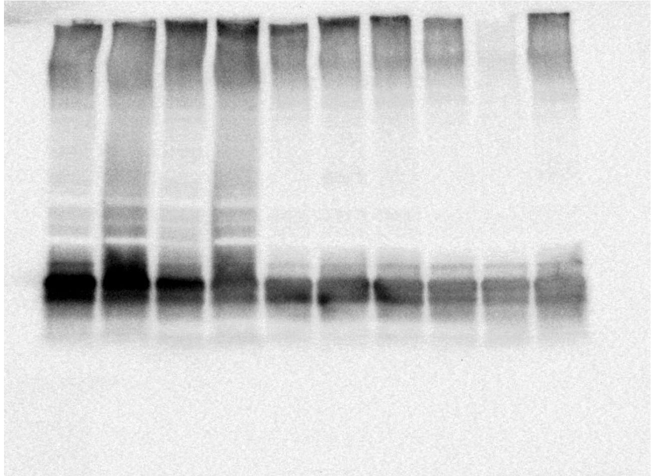

Date: MPT 19 Mar 2024

ImageJ Quantification Box – PMP22

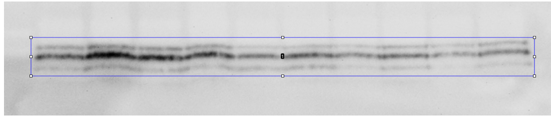

Date: LMW 27 Mar 2024

ImageJ Quantification Box – B-Actin

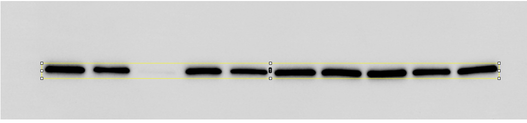

Date: LMW 22 Mar 2024

ImageJ Quantification Box - MPZ

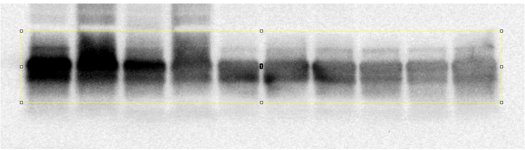

Date:

Plot 1 Values PMP22

	Area
1	36061.68
2	66070.22
3	39654.2
4	35094.68
5	14046.54
6	22675.1
7	10075.08
8	15366.61
9	6472.56
10	17643.8

Plot 1-PMP22

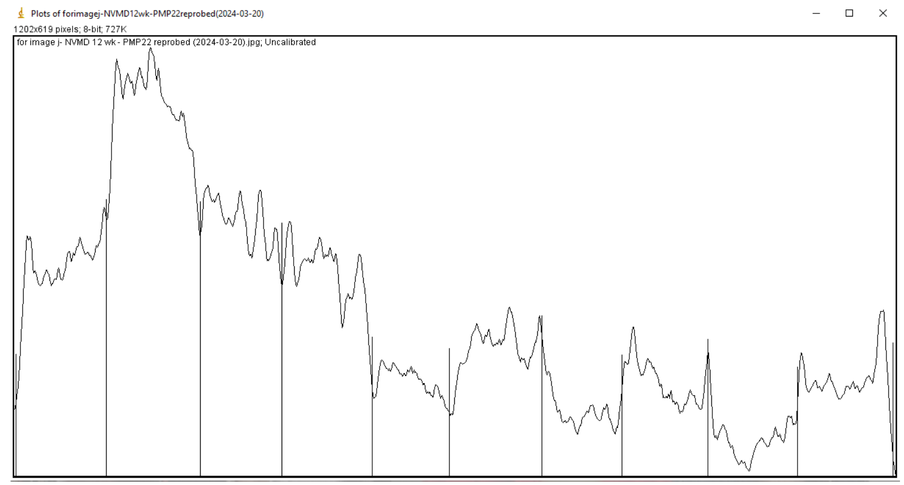

Date: LMW 27 Mar 2024

Plot 2 Values B-Actin

	Area
1	56521.32
2	44086.9
3	587.548
4	47482.25
5	40785.2
6	53800.73
7	53887.95
8	52611.66
9	45898.66
10	51154.54

Plot 2-B-Actin

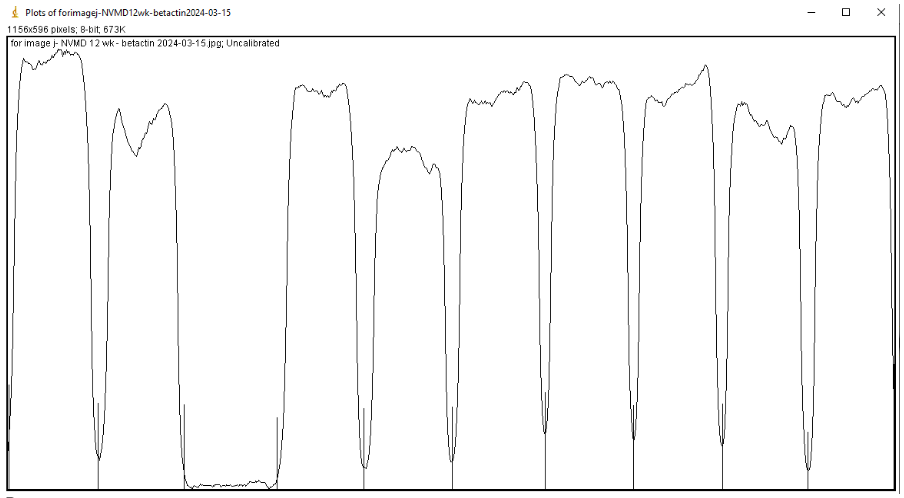

Date: LMW 22 Mar 2024

Western Blot Imaging Form

Plot 3 Values-MPZ

	Area
1	54228.97
2	57649.44
3	40949.32
4	38414.73
5	25169.66
6	31021.97
7	28096.61
8	21867.25
9	17224.78
10	24729.85

Plot 3-MPZ

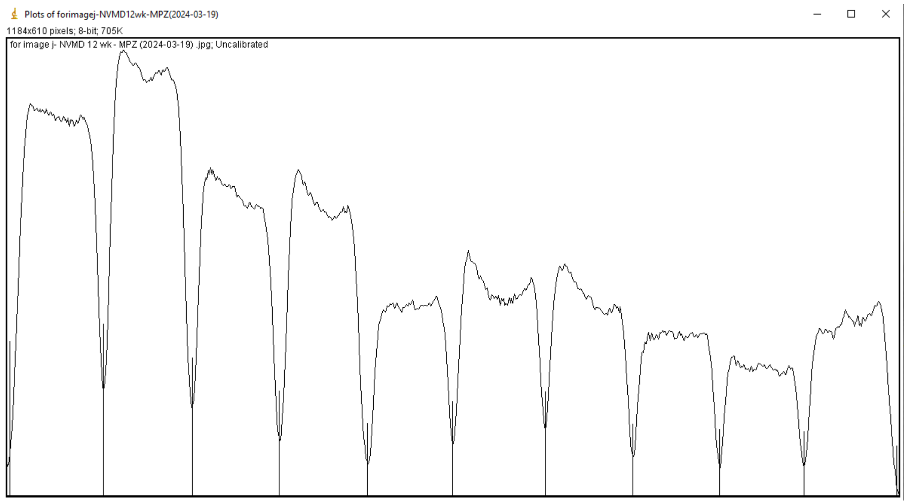

Date: 27 Mar 2024 LMW

Operator Information

Performed By:	Name: Merlin P Thangaraj	Signature: [signature]
	Position/Lab: Postdoctoral Scientist / Harper Lab	Date: 27 Jun 2024

Performed By:	Name: Lindsay Wallace	Signature: [signature]
	Position/Lab: Sr Research Scientist / Harper Lab	Date: 27 Mar 2024

Western Blot Imaging Form

**Study**	ARM101-CMT1A-NHP-001
**Timepoint**	6 week
**Tissue**	Median nerve - Right
**Anatomical Location** (Highlight/Circle one)	Distal (circled) Proximal N/A Other:

Stain Free Gel

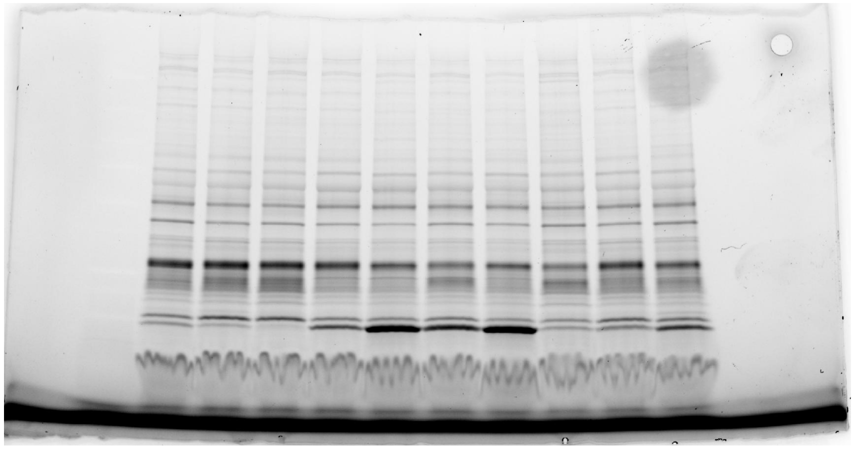

Date: 04 Jun 2025 MPT

Stain Free Membrane

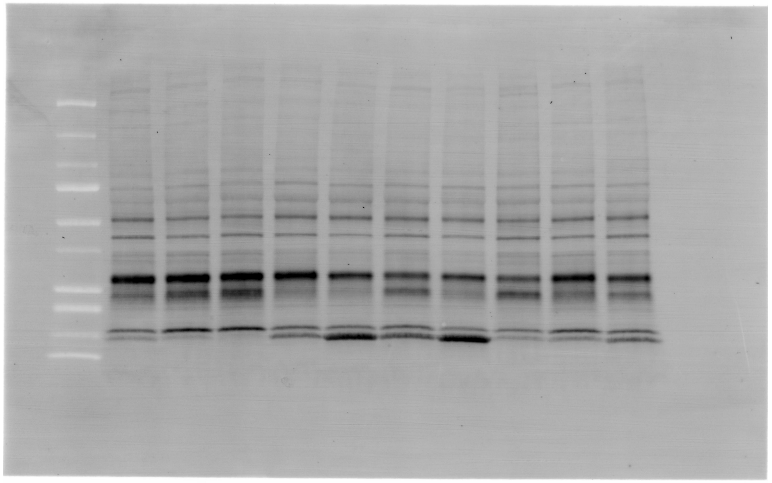

Date: 04 Jun 2025 MPT

1° PMP22

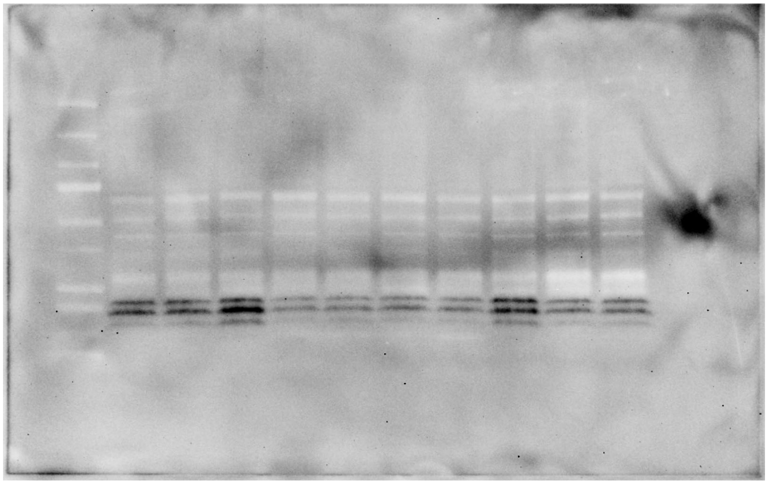

Date: 05 Jun 2025 MPT

1° β-Actin

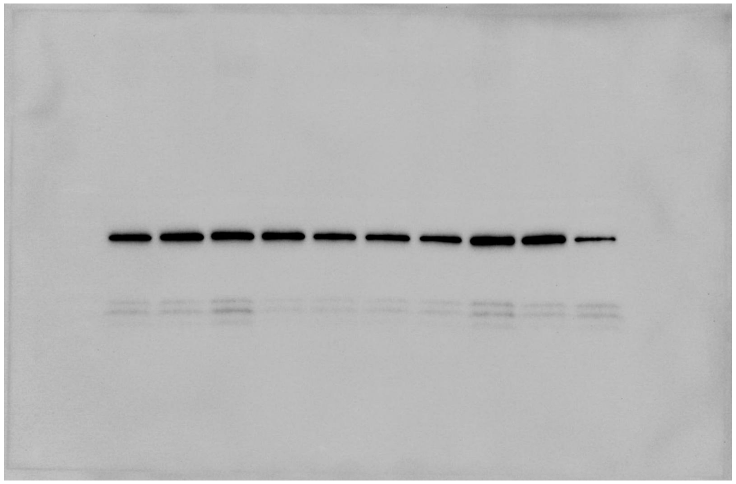

Date: 05 Jun 2025 MPT

Western Blot Imaging Form

Strip Check

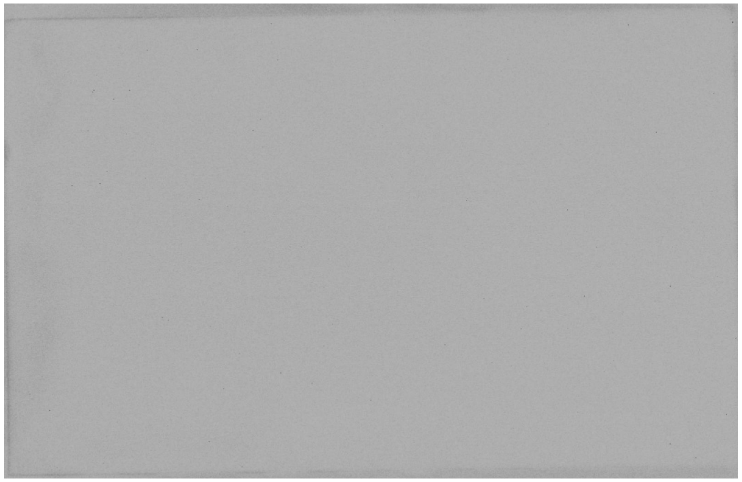

Date: 09 Jun 2025 MPT

1° MPZ

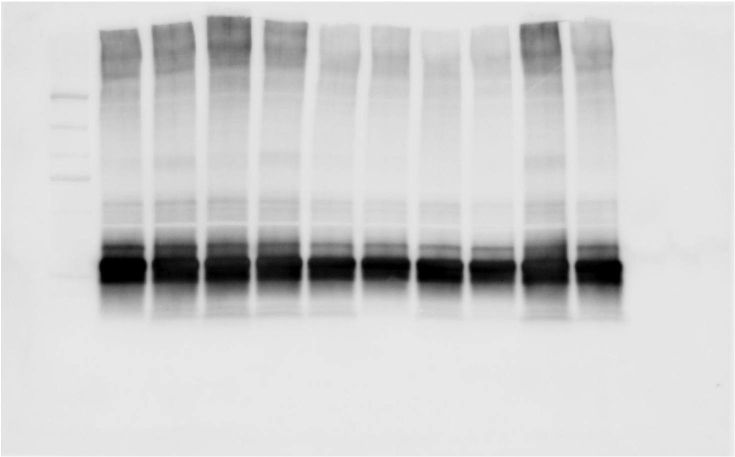

Date: 10 Jun 2025 MPT

ImageJ Quantification Box – PMP22

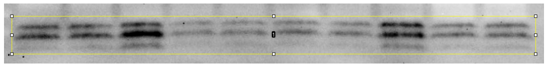

Date: 09 Jun 2025 LMW

ImageJ Quantification Box – B-Actin

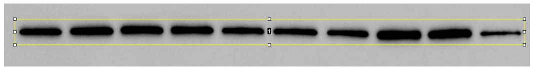

Date: 09 Jun 2025 LMW

ImageJ Quantification Box - MPZ

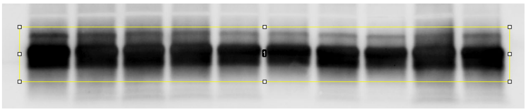

Date: 18 Jun 2025 LMW

Plot 1 Values PMP22

	Area
1	37551.49
2	36244.02
3	51290.73
4	19976.2
5	24150.61
6	22625.27
7	18176.49
8	47509.68
9	13594.61
10	16860.15

Plot 1-PMP22

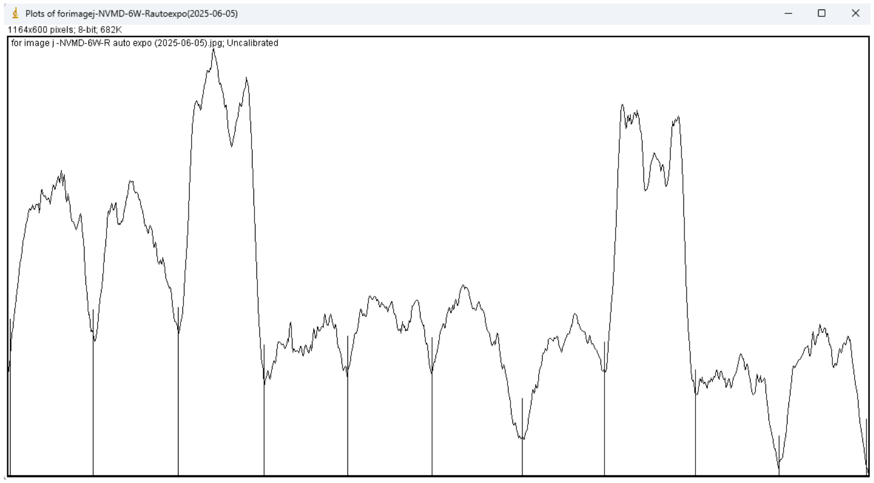

Date: 09 Jun 2025 LMW

Plot 2 Values B-Actin

	Area
1	43966.08
2	50181.02
3	48696.49
4	46735.02
5	38788.32
6	38518.2
7	36376.78
8	54230.2
9	52132.9
10	20577.54

Plot 2-B-Actin

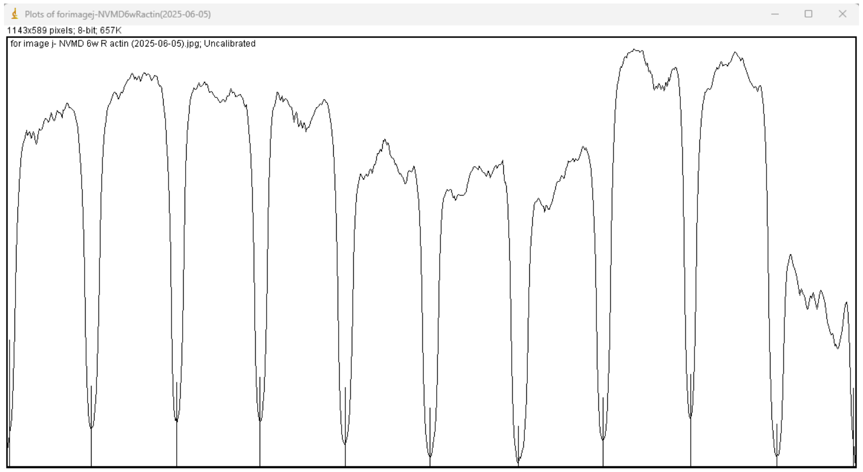

Date: 09 Jun 2025 LMW

Western Blot Imaging Form

Plot 3 Values-MPZ

	Area
1	58052.93
2	55872.88
3	53049.45
4	50239.59
5	45072.66
6	45060.78
7	45760.76
8	41196.64
9	53477.2
10	51580.25

Plot 3-MPZ

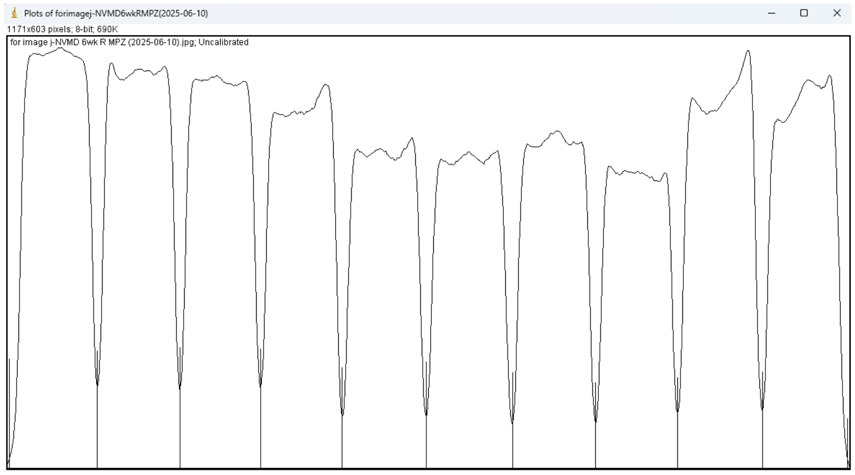

Date: 18 Jun 2025 LMW

Operator Information

**Performed By:**	**Name:** Merlin P Thangaraj	**Signature:**
	**Position/Lab:** Postdoctoral Scientist/Harper Lab	**Date:** 8-1-2025

**Performed By:**	**Name:** Lindsay Wallace	**Signature:**
	**Position/Lab:** Sr Research Scientist / Harper Lab	**Date:** 18 Jun 2025

Western Blot Imaging Form

**Study**	ARM101-CMT1A-NHP-001
**Timepoint**	12 week
**Tissue**	Median Nerve-Right
**Anatomical Location** (Highlight/Circle one)	Distal (circled) Proximal N/A Other:

Stain Free Gel

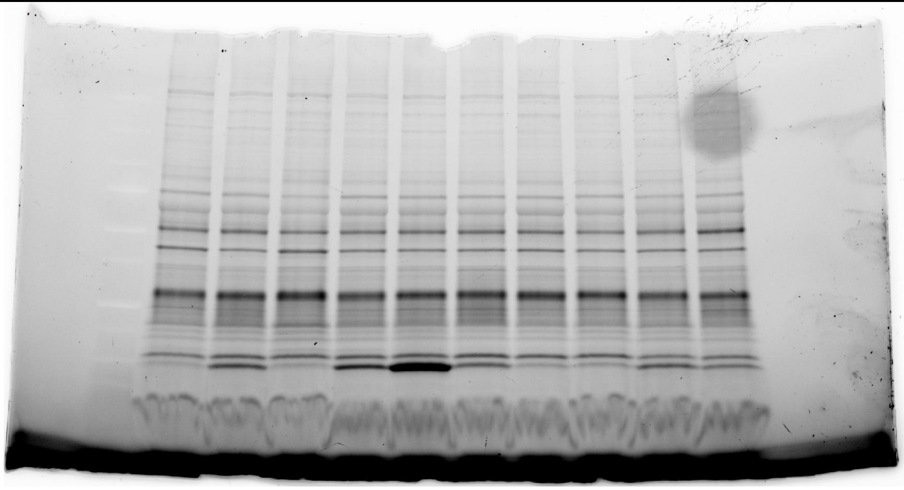

Date: MPT 25 Sep 2024

Stain Free Membrane

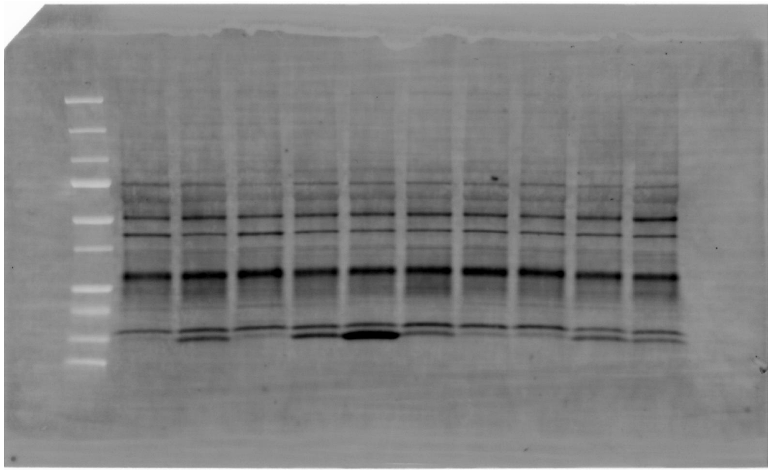

Date: MPT 25 Sep 2024

1° PMP22

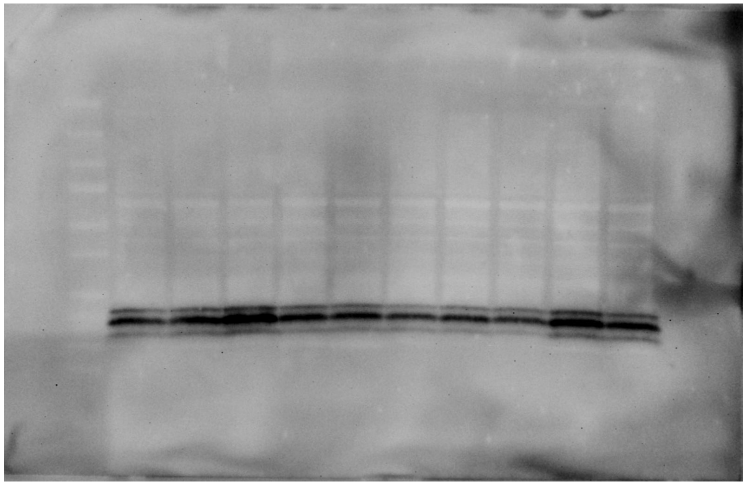

Date: MPT 26 Sep 2024

1° β-Actin

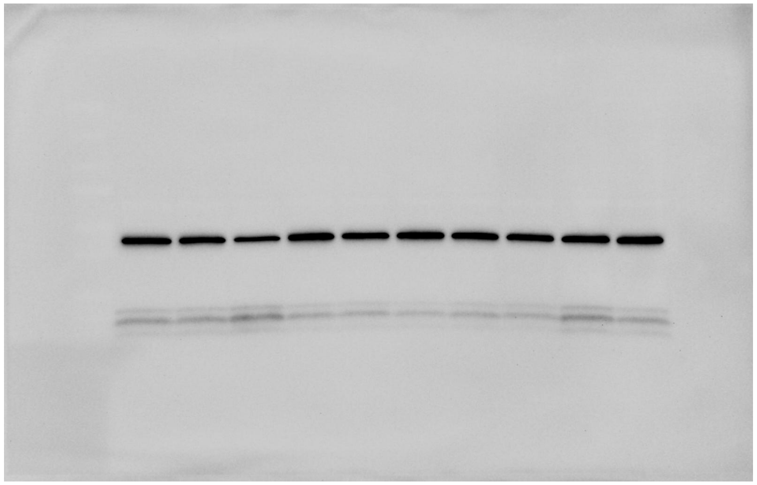

Date: MPT 26 Sep 2024

Western Blot Imaging Form

Strip Check

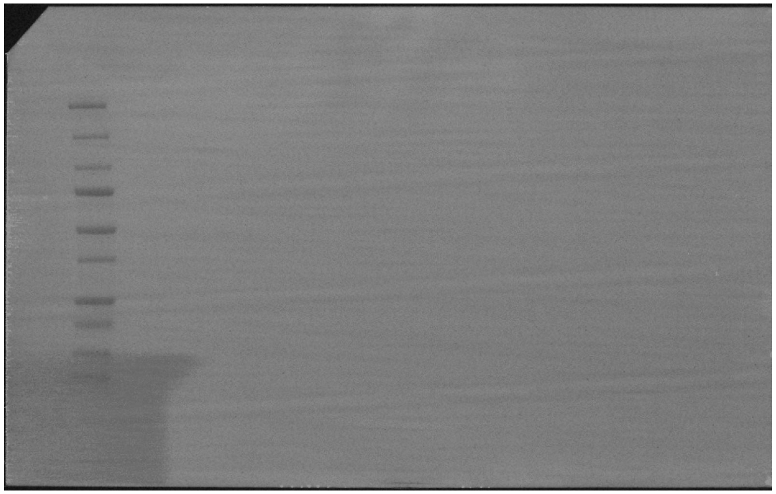

Date: MPT 26 Sep 2024

1° MPZ

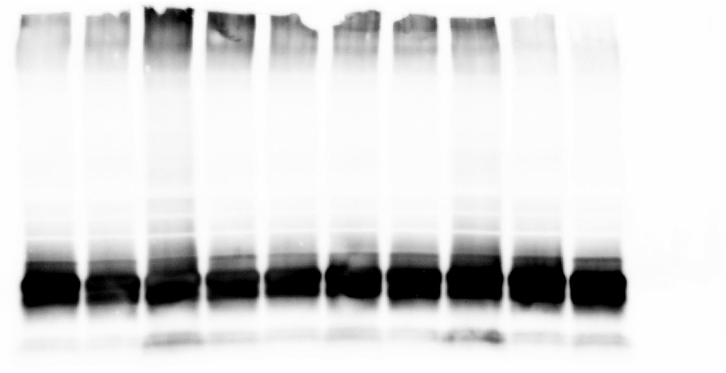

Date: MPT 27 Sep 2024

ImageJ Quantification Box – PMP22

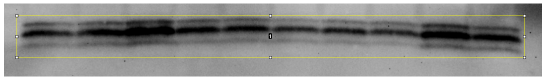

Date: LMW 29 Oct 2024

ImageJ Quantification Box – B-Actin

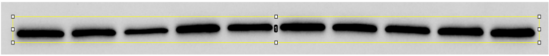

Date: LMW 29 Oct 2024

ImageJ Quantification Box - MPZ

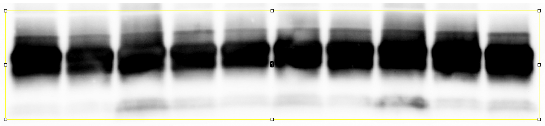

Date: LMW 29 Oct 2024

Plot 1 Values PMP22

	Area
1	41985.34
2	47806.02
3	67613.27
4	44153.37
5	44141.73
6	26703.9
7	28256.61
8	27905.32
9	63806.39
10	56281.85

Plot 1-PMP22

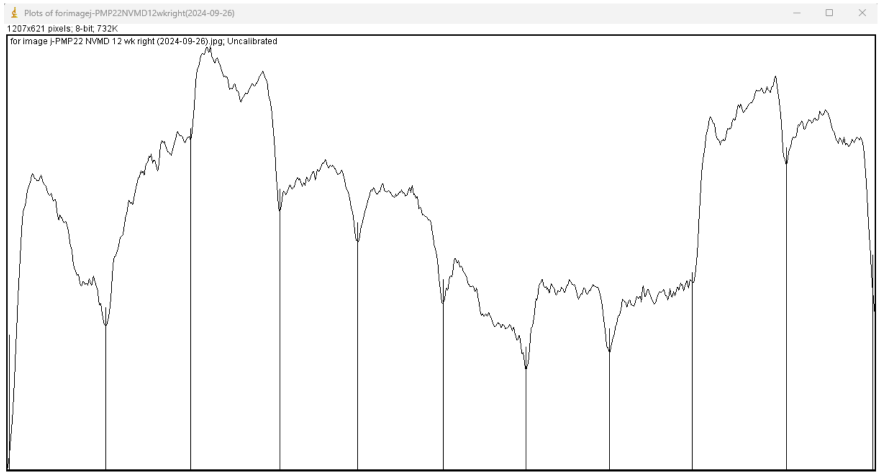

Date: LMW 29 Oct 2024

Plot 2-B-Actin

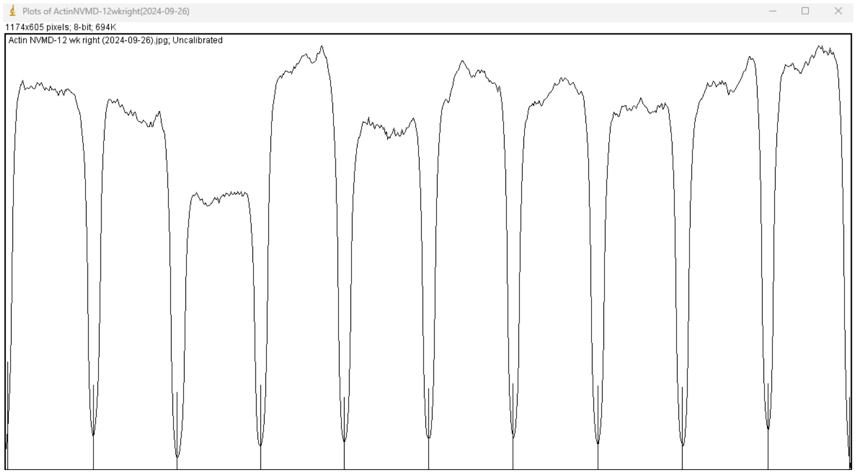

Date: LMW 29 OCT 2024

Plot 2 Values B-Actin

	Area
1	54868.73
2	48303.61
3	36685.61
4	54078.49
5	47125.02
6	53829.73
7	51545.15
8	49639.02
9	54149.44
10	54887.49

Western Blot Imaging Form

Plot 3 Values-MPZ

	Area
1	51580.49
2	40341.13
3	50938.66
4	39960.71
5	40597.3
6	45766.61
7	46328.88
8	57212.78
9	53284.25
10	51040.59

Plot 3-MPZ

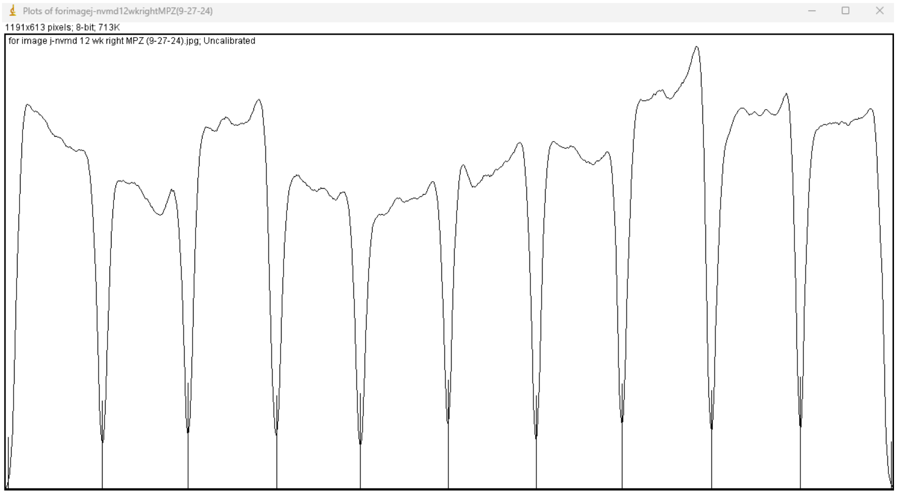

Date: 27 Mar 2024 LMW

Operator Information

**Performed By:**	**Name:** Merlin P Thangaraj	**Signature:** T. Merlin Thangaraj
	**Position/Lab:** Postdoctoral Scientist / Harper Lab	**Date:** 30 Oct 2024

**Performed By:**	**Name:** Lindsay Wallace	**Signature:** [signature]
	**Position/Lab:** Sr Research Scientist / Harper Lab	**Date:** 29 Oct 2024

Western Blot Imaging Form

**Study**	ARM101-CMT1A-NHP-001
**Timepoint**	6 week
**Tissue**	Median nerve
**Anatomical Location** (Highlight/Circle one)	Distal Proximal (circled) N/A<br>Other:

Stain Free Gel

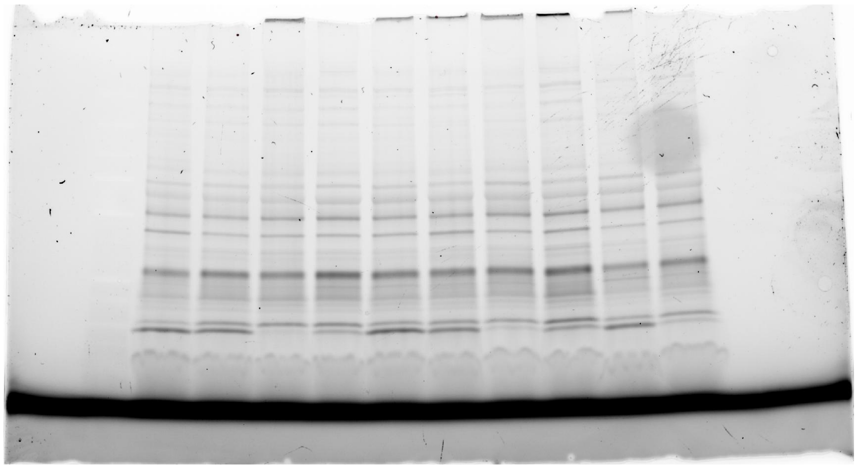

Date: 06 Jun 2024 MPT

Stain Free Membrane

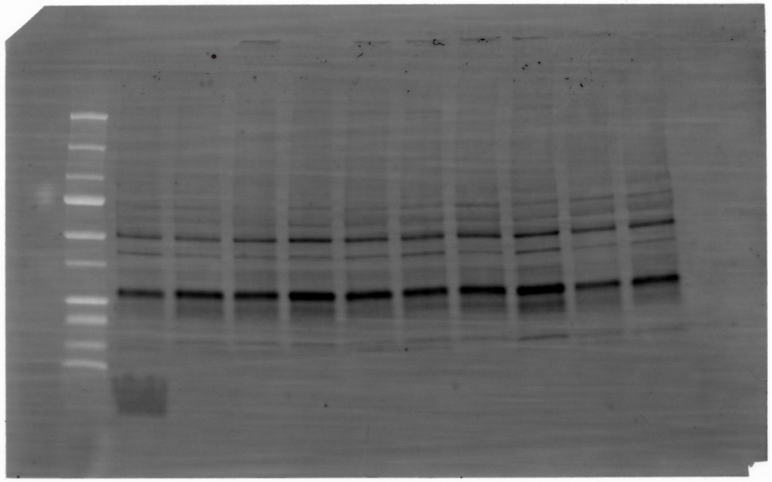

Date: 07 Jun 2024 MPT

1° PMP22

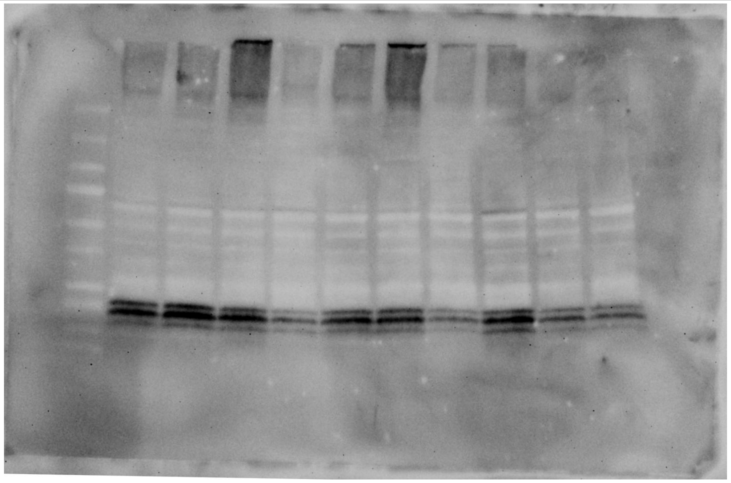

Date: 07 Jun 2024 MPT

1° β-Actin

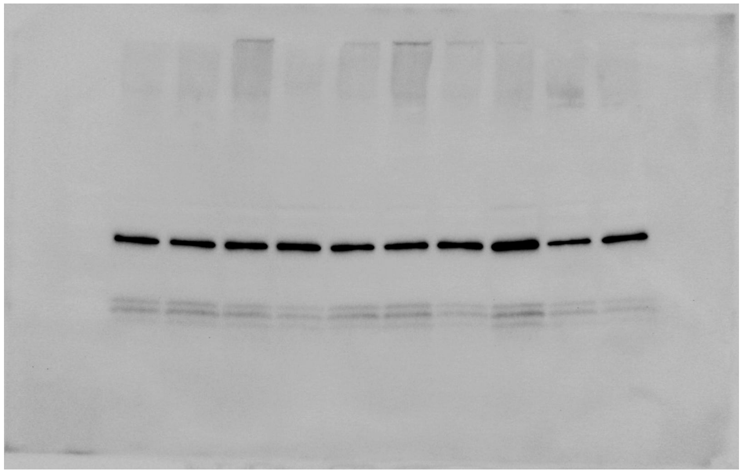

Date: 07 Jun 2024 MPT

Western Blot Imaging Form

Strip Check

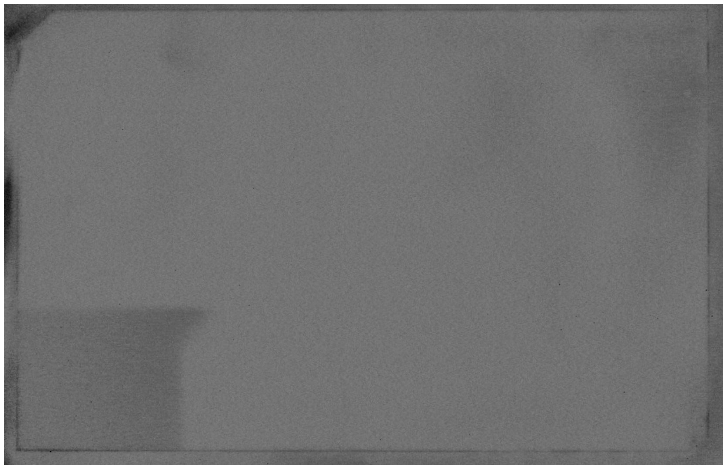

Date: 14 Jun 2024 MPT

1° MPZ

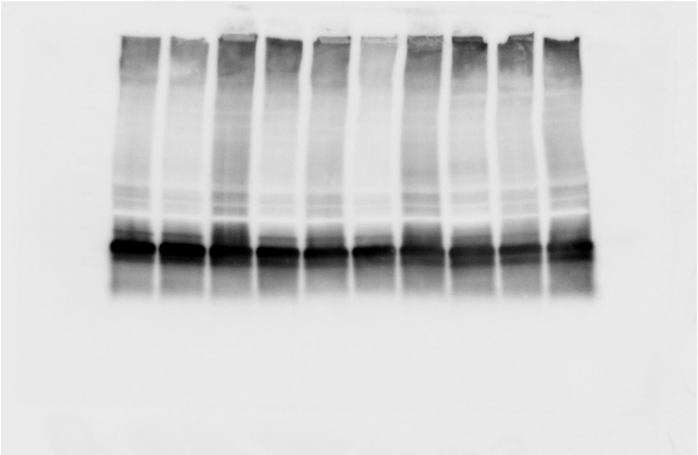

Date: 14 Jun 2024 MPT

ImageJ Quantification Box – PMP22

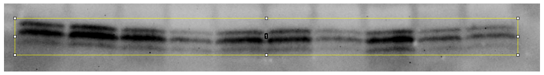

Date: 25 Jun 2024 LMW

ImageJ Quantification Box – B-Actin

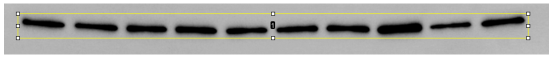

Date: 25 Jun 2024 LMW

ImageJ Quantification Box - MPZ

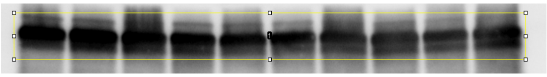

Date: 25 Jun 2024 LMW

Western Blot Imaging Form

Plot 1 Values PMP22

	Area
1	63554.27
2	63005.51
3	44559.56
4	7233.075
5	37941.39
6	41624.44
7	11772.61
8	50640.92
9	26852.9
10	26781.22

Plot 1-PMP22

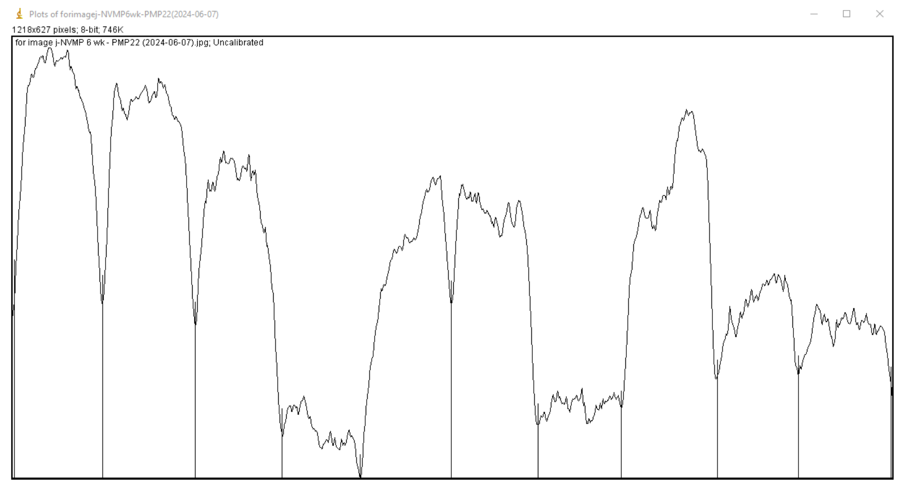

Date: 25 Jun 2024 LMW

Plot 2 Values B-Actin

	Area
1	41310.73
2	40233.27
3	42159.32
4	43896.44
5	36732.44
6	37282.15
7	41379.44
8	58589.56
9	28144.02
10	39804.44

Plot 2-B-Actin

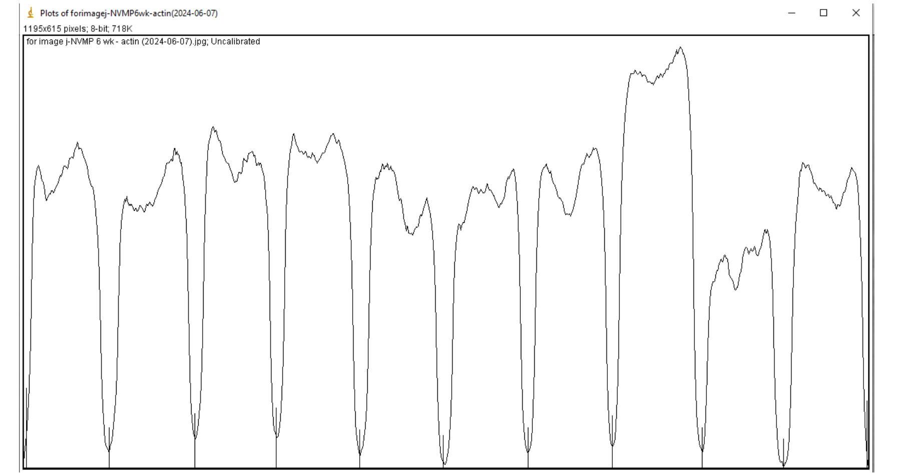

Date: 25 Jun 2024 LMW

Western Blot Imaging Form

Plot 3 Values-MPZ

	Area
1	55955.9
2	58944.37
3	58481.37
4	48045.25
5	49359.13
6	43438.9
7	51726.37
8	47000.02
9	41501.47
10	55629.22

Plot 3-MPZ

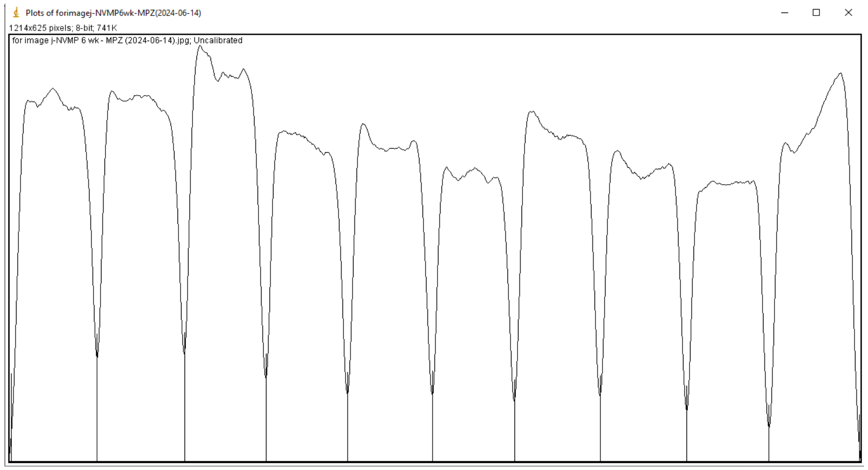

Date: 25 Jun 2024 LMW

Operator Information

**Performed By:**	**Name:** Merlin P Thangaraj	**Signature:** T. Merlin Pramelatha
	**Position/Lab:** Postdoctoral Scientist / Harper Lab	**Date:** 27 Jun 2024

**Performed By:**	**Name:** Lindsay Wallace	**Signature:**
	**Position/Lab:** Sr Research Scientist/Harper Lab	**Date:** 25 Jun 2024

Western Blot Imaging Form

**Study**	ARM101-CMT1A-NHP-001		
**Timepoint**	12 weeks		
**Tissue**	Median Nerve		
**Anatomical Location** (Highlight/Circle one)	Distal	Proximal (circled)	N/A
	Other:		

Stain Free Gel

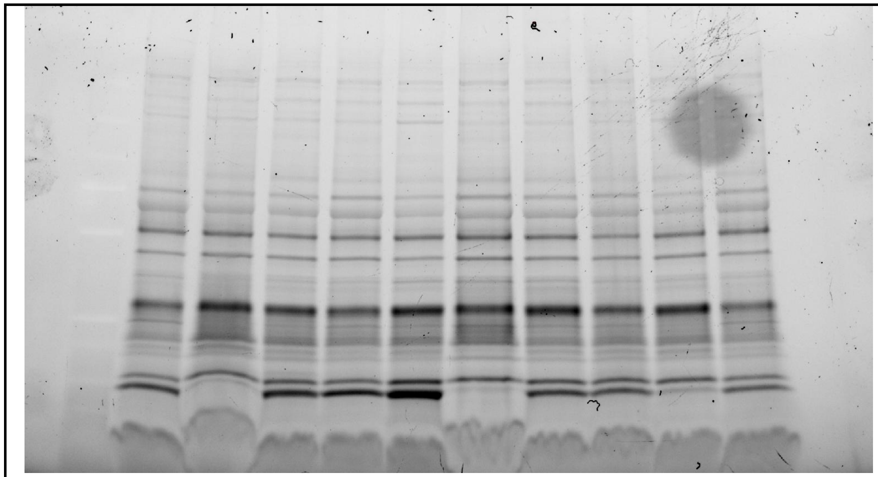

Date: 14 Mar 2024 MPT

Stain Free Membrane

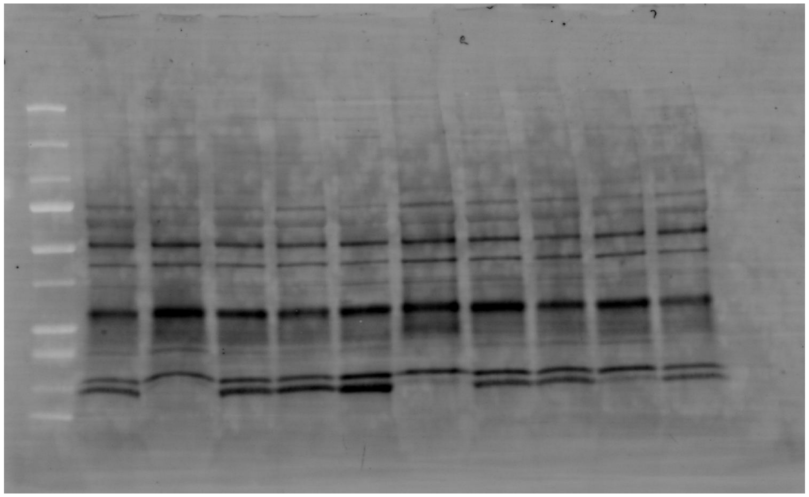

Date: 14 Mar 2024 MPT

1° PMP22

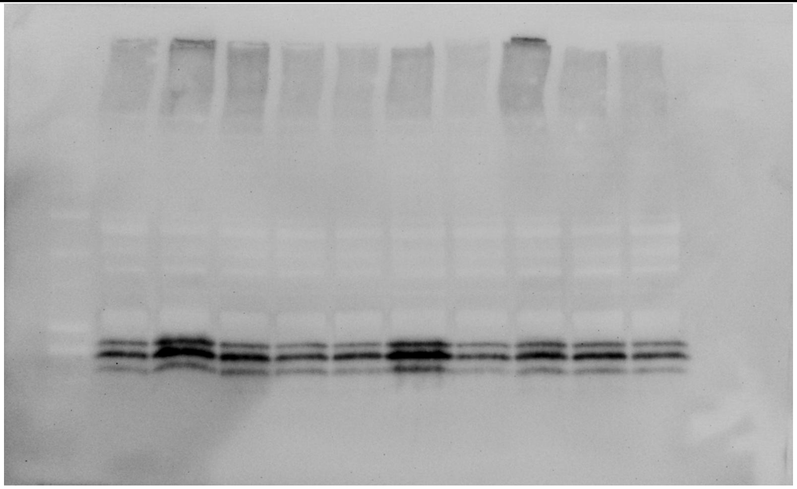

Date: 20 mar 2024 MPT

1° β-Actin

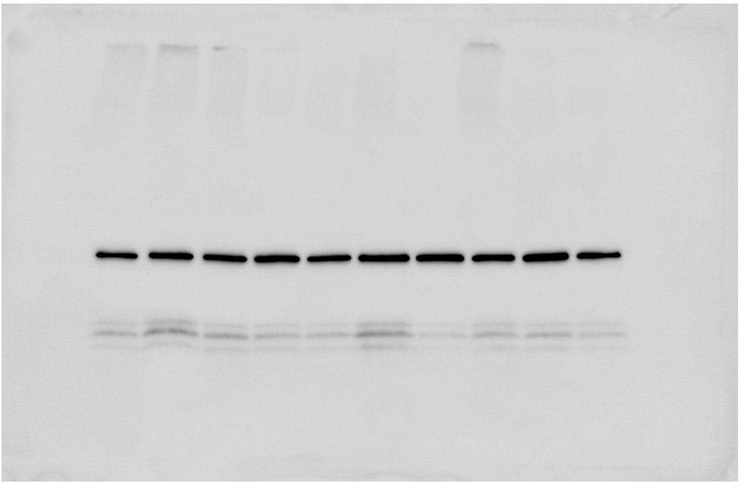

Date: 15 Mar 2024 MPT

Western Blot Imaging Form

Strip Check

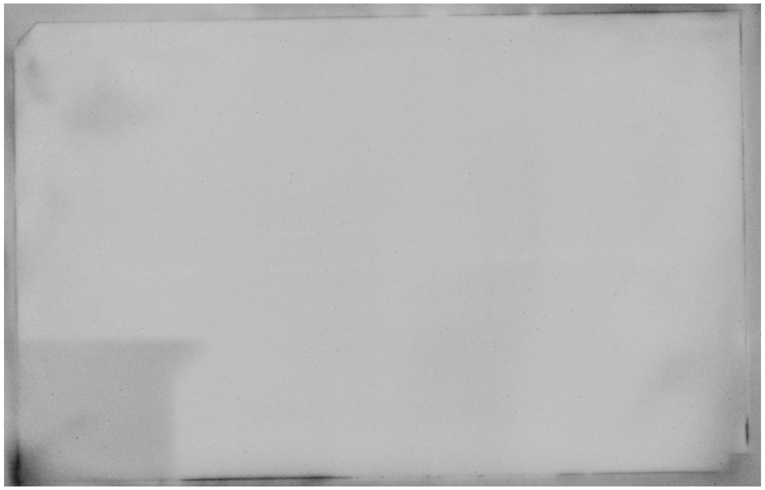

Date: 19 Mar 2024 MPT

1° MPZ

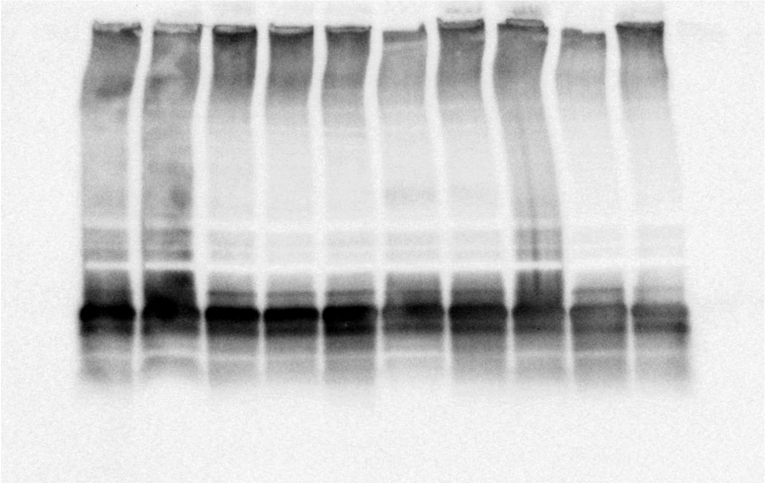

Date: 19 Mar 2024 MPT

ImageJ Quantification Box – PMP22

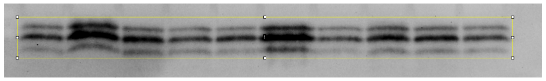

Date: 27 Mar 2024 LMW

ImageJ Quantification Box – B-Actin

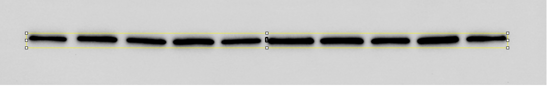

Date: 27 Mar 2024 LMW

ImageJ Quantification Box - MPZ

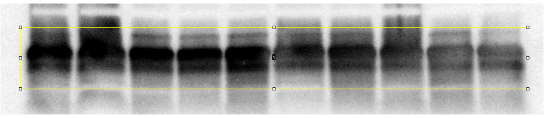

Date: 27 Mar 2024 LMW

Plot 1 Values PMP22

	Area
1	34161.2
2	69273.46
3	43738.37
4	28385.32
5	25673.95
6	65826.82
7	21544.2
8	35078.02
9	31906.44
10	20862.27

Plot 1-PMP22

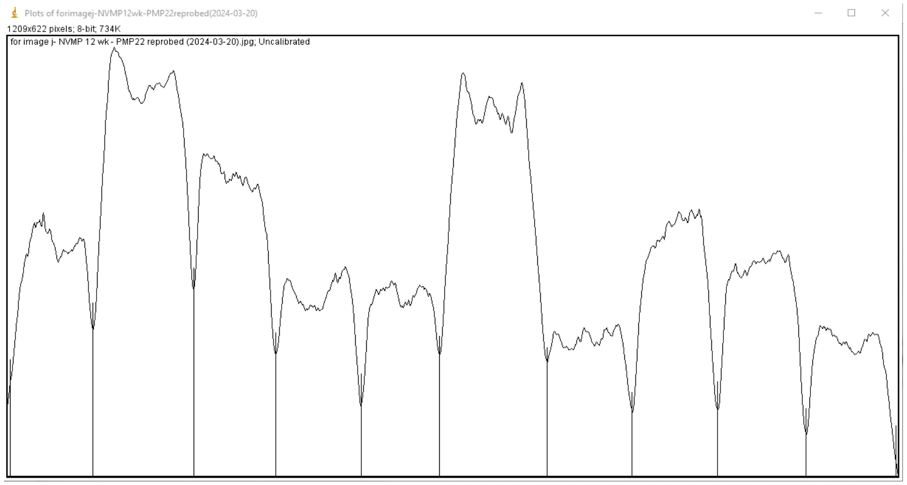

Date: 27 Mar 2024 LMW

Plot 2 Values B-Actin

	Area
1	37795.83
2	47663.02
3	46161.9
4	51781.73
5	43255.61
6	58156.51
7	55945.44
8	46799.2
9	57367.02
10	41841.54

Plot 2-B-Actin

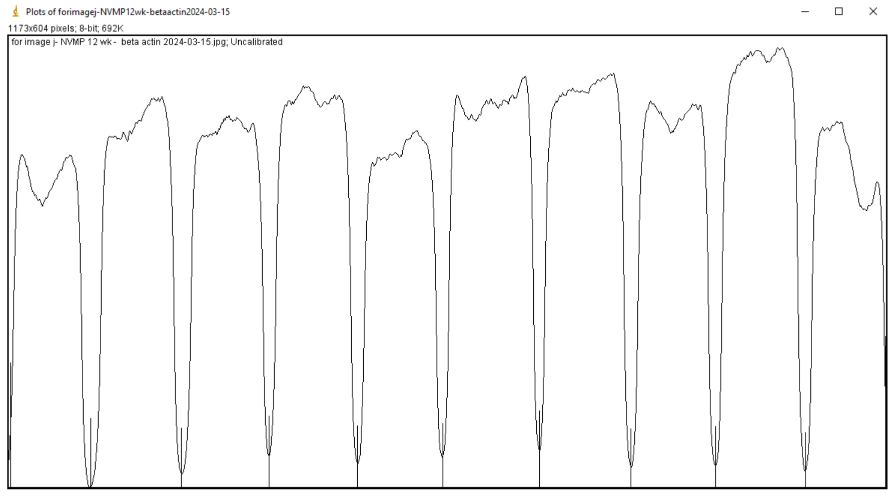

Date: 27 Mar 2024 LMW

Plot 3 Values-MPZ

	Area
1	58762.39
2	62722.39
3	53565.32
4	51850.02
5	57037.02
6	54739.92
7	58726.15
8	49398.49
9	43397.44
10	36298.27

Plot 3-MPZ

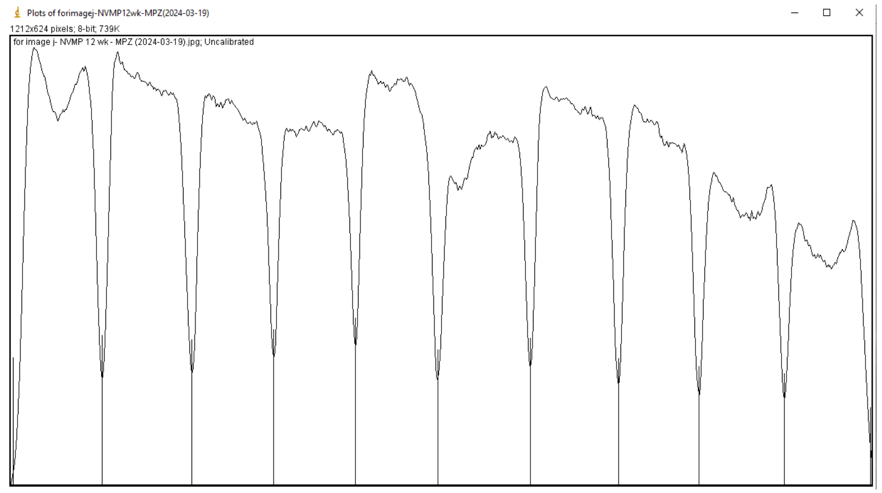

Date: 27 Mar 2024 LMW

Operator Information

Performed By:	Name: Merlin P Thangaraj	Signature:
	Position/Lab: Postdoctoral Scientist / Harper Lab	Date: 27 Jun 2024

Performed By:	Name: Lindsay Wallace	Signature:
	Position/Lab: Sr Research Scientist / Harper Lab	Date: 27 Mar 2024

Western Blot Imaging Form

Study	ARM101-CMT1A-NHP-001
Timepoint	12 weeks
Tissue	Median Nerve Right
Anatomical Location (Highlight/Circle one)	Distal Proximal (circled) N/A<br>Other:

Stain Free Gel

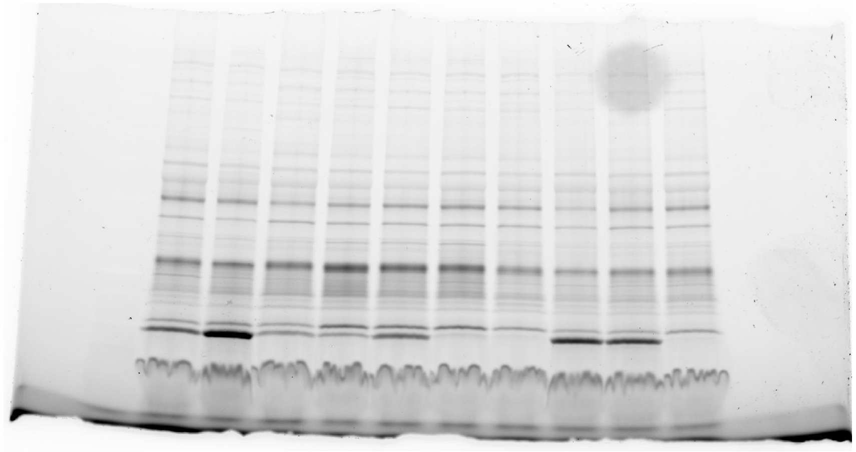

Date: 11 Jun 2025 MPT

Stain Free Membrane

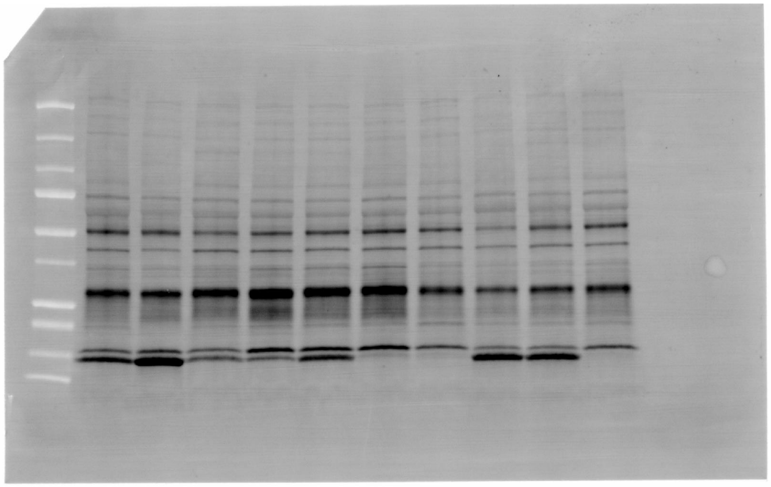

Date: 11 Jun 2025 MPT

1° PMP22

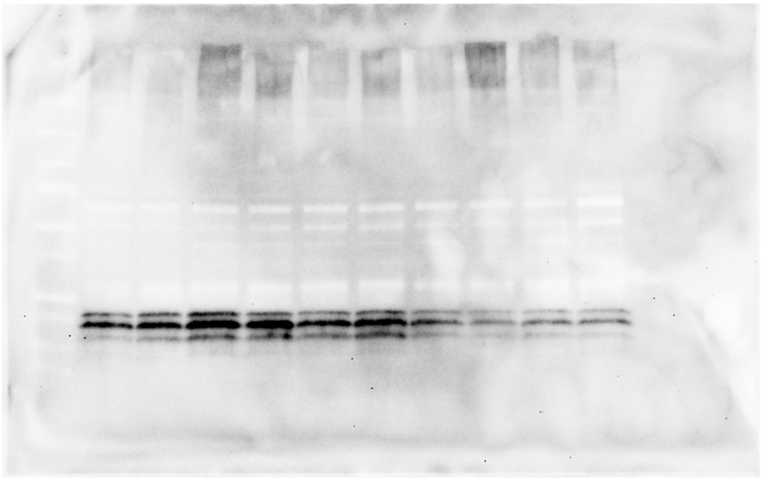

Date: 12 Jun 2025 MPT

1° β-Actin

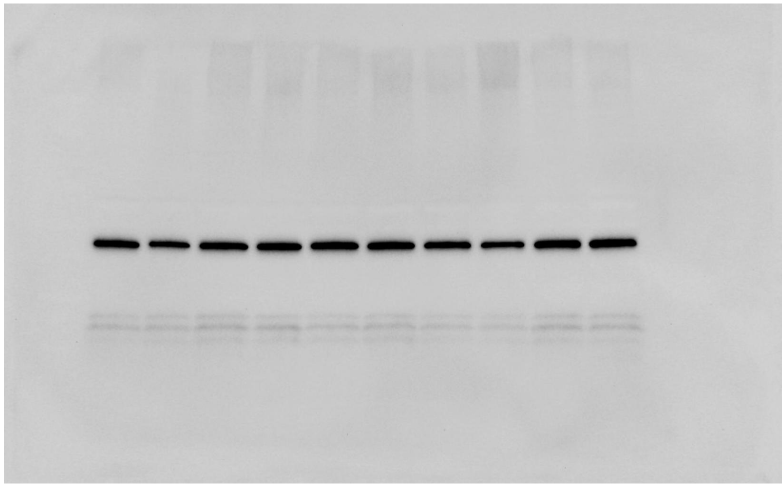

Date: 12 Jun 2025 MPT

Western Blot Imaging Form

Strip Check

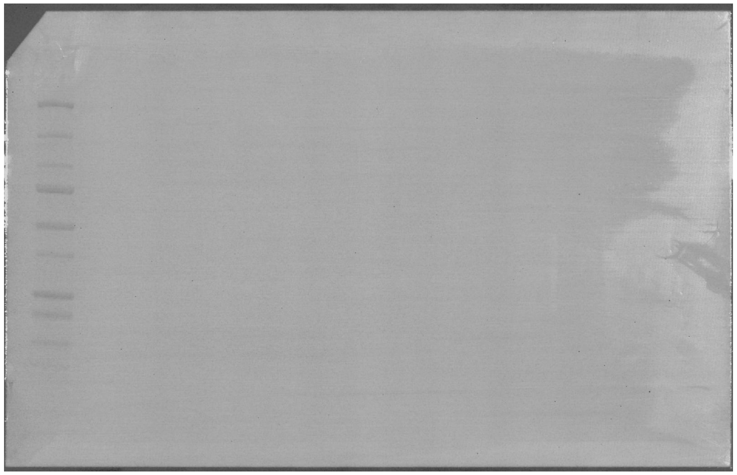

Date: 12 Jun 2025 MPT

1° MPZ

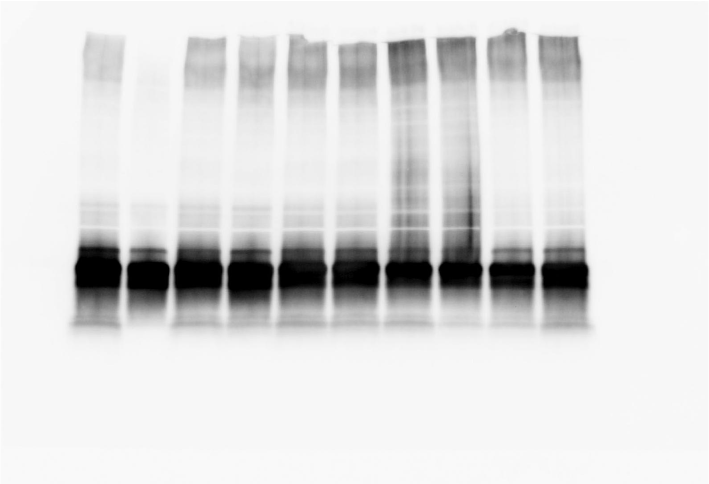

Date: 13 Jun 2025 MPT

ImageJ Quantification Box – PMP22

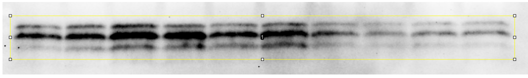

Date: 18 Jun 2025 LMW

ImageJ Quantification Box – B-Actin

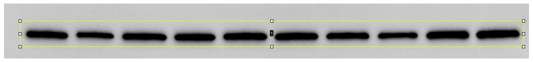

Date: 18 Jun 2025 LMW

ImageJ Quantification Box - MPZ

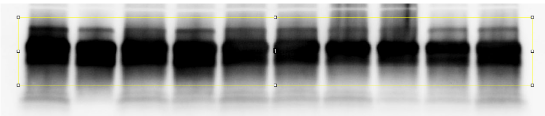

Date: 18 Jun 2025 LMW

Western Blot Imaging Form

Plot 1 Values PMP22

	Area
1	52585.55
2	56713.29
3	102152.5
4	90827.36
5	83908.72
6	108682.1
7	65569.02
8	33569.41
9	22322.65
10	18594.67

Plot 1-PMP22

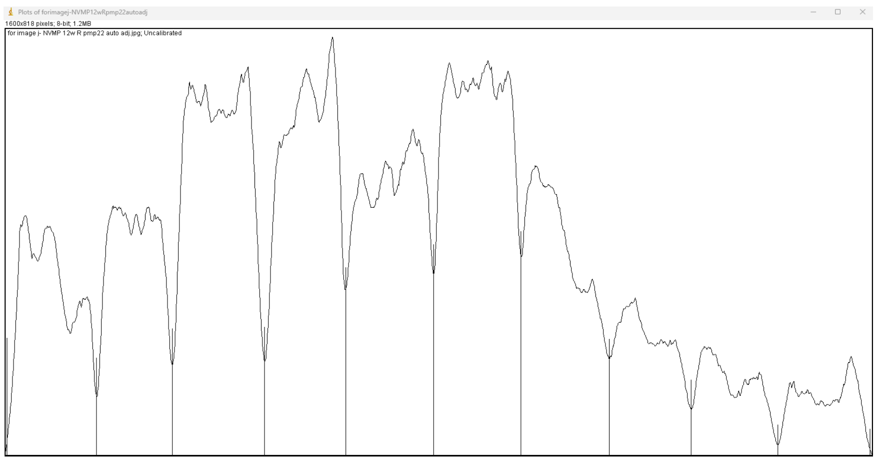

Date: 18 Jun 2025 LMW

Plot 2 Values B-Actin

	Area
1	50912.73
2	38993.83
3	52379.15
4	50468.08
5	54840.9
6	55126.15
7	48149.73
8	38491.25
9	53550.73
10	54374.32

Plot 2-B-Actin

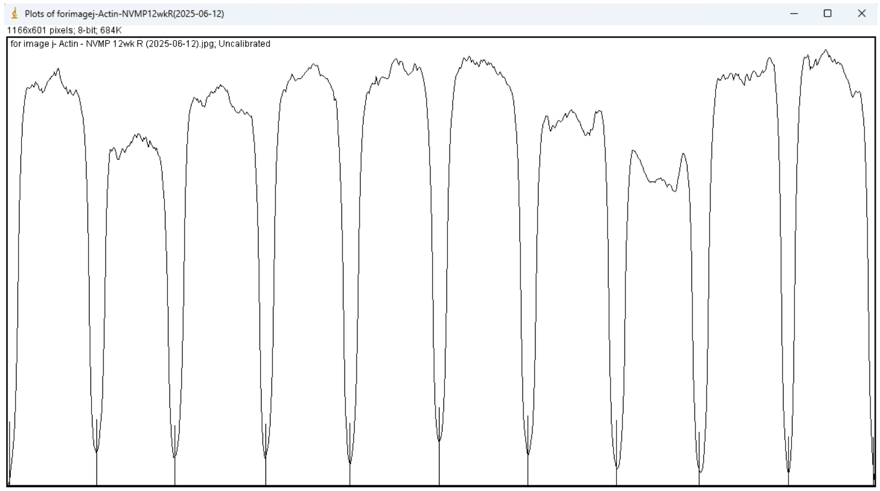

Date: 18 Jun 2025 LMW

Plot 3 Values-MPZ

	Area
1	57802.13
2	47480.25
3	57628.2
4	54622.13
5	54842.71
6	52939.42
7	50705.2
8	47551.78
9	44223.9
10	50378.61

Plot 3-MPZ

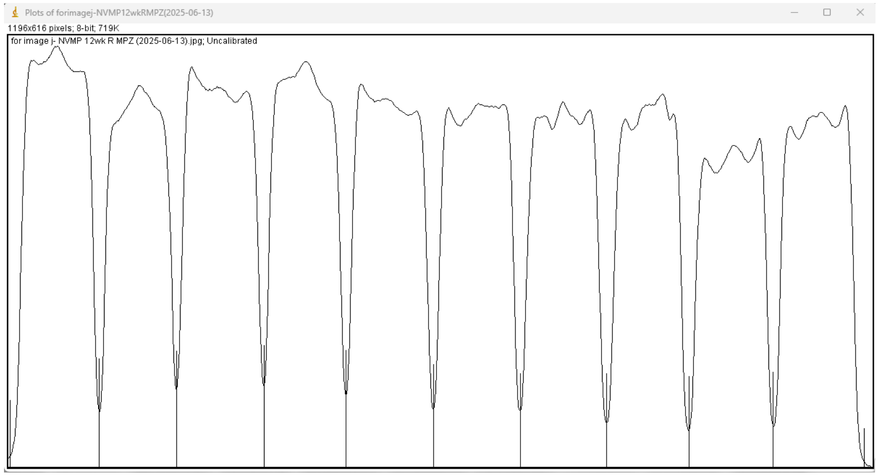

Date: 18 Jun 2025 LMW

Operator Information

**Performed By:**	**Name:** Merlin P Thangaraj	**Signature:** [signature]
	**Position/Lab:** Postdoctoral Scientist / Harper Lab	**Date:** 8-1-2025

**Performed By:**	**Name:** Lindsay Wallace	**Signature:** [signature]
	**Position/Lab:** Sr Research Scientist / Harper Lab	**Date:** 27 Mar 2024

Western Blot Imaging Form

**Study**	ARM101-CMT1A-NHP-001
**Timepoint**	6 week
**Tissue**	Sciatic nerve
**Anatomical Location** (Highlight/Circle one)	Distal (circled) Proximal N/A Other:

Stain Free Gel

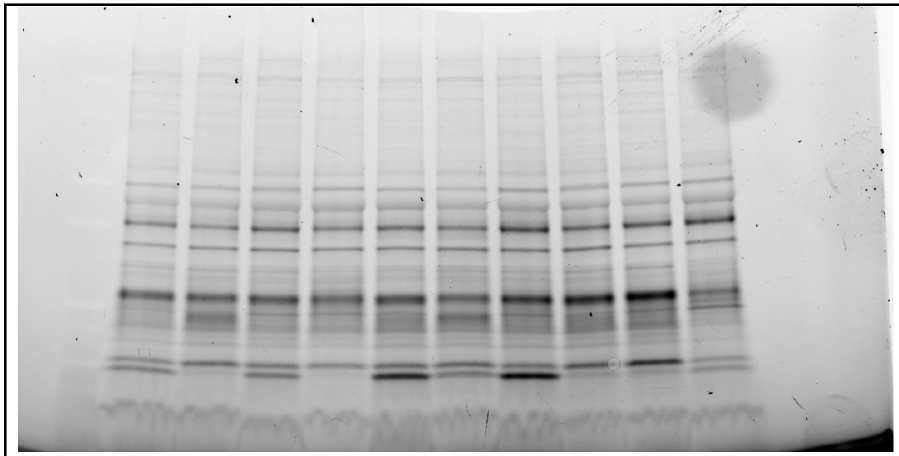

Date: 08 Apr 2024 MPT

Stain Free Membrane

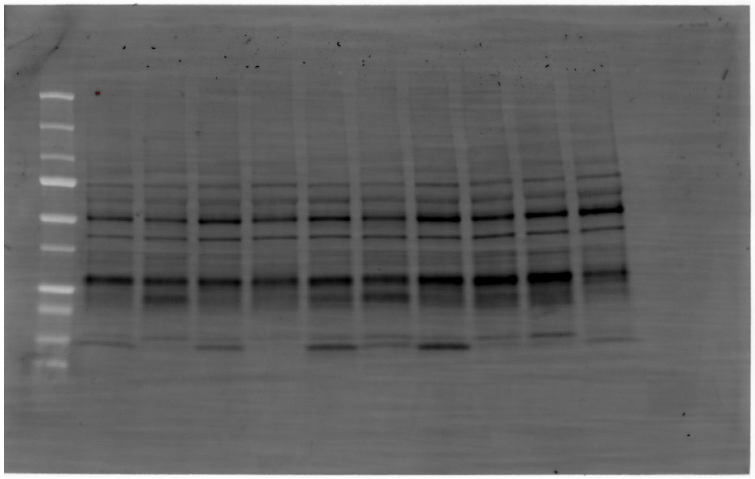

Date: 08 Apr 2024 MPT

1° PMP22

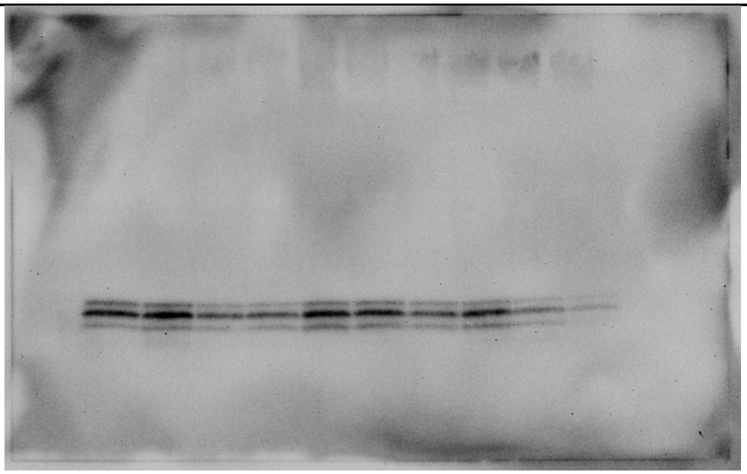

Date: 09 Apr 2024 MPT

1° β-Actin

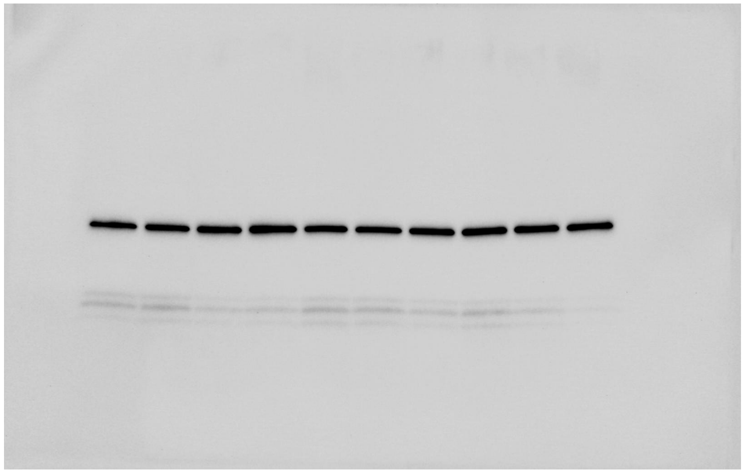

Date: 09 Apr 2024 MPT

Western Blot Imaging Form

Strip Check

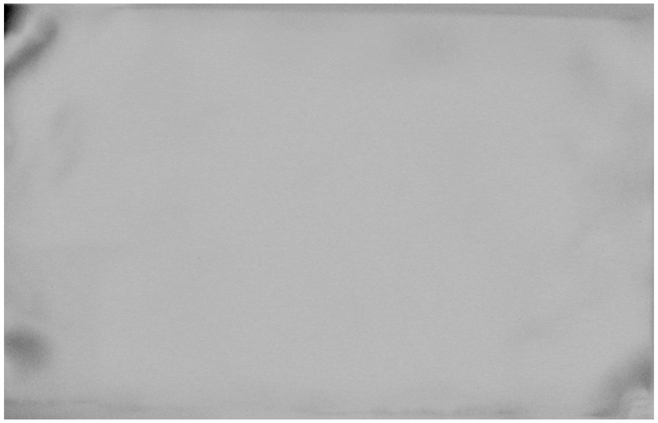

Date: 10 Apr 2024 MPT

1° MPZ

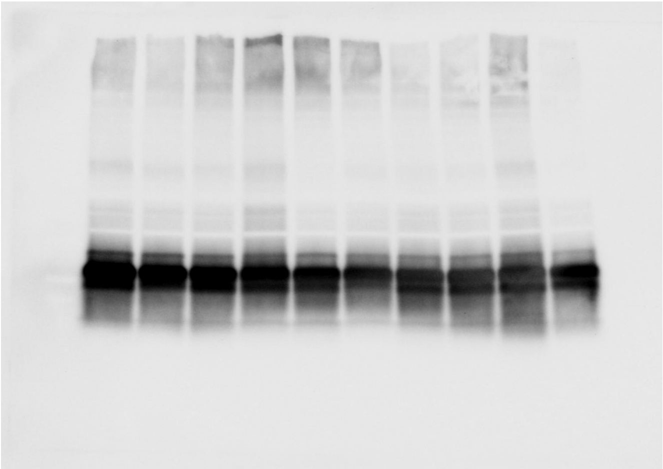

Date: 11 Apr 2024 MPT

ImageJ Quantification Box – PMP22

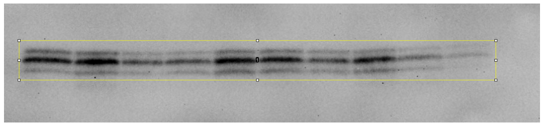

Date: 23 Apr 2024 LMW

ImageJ Quantification Box – B-Actin

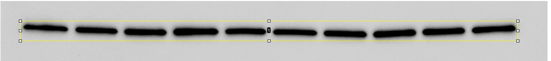

Date: 23 Apr 2024 LMW

ImageJ Quantification Box - MPZ

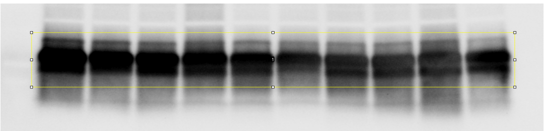

Date: 23 Apr 2024 LMW

Plot 1 Values PMP22

	Area
1	54972.34
2	58779.39
3	28883.78
4	31234.15
5	53658.44
6	49959.39
7	32238.49
8	47398.27
9	21124.32
10	12439.68

Plot 1-PMP22

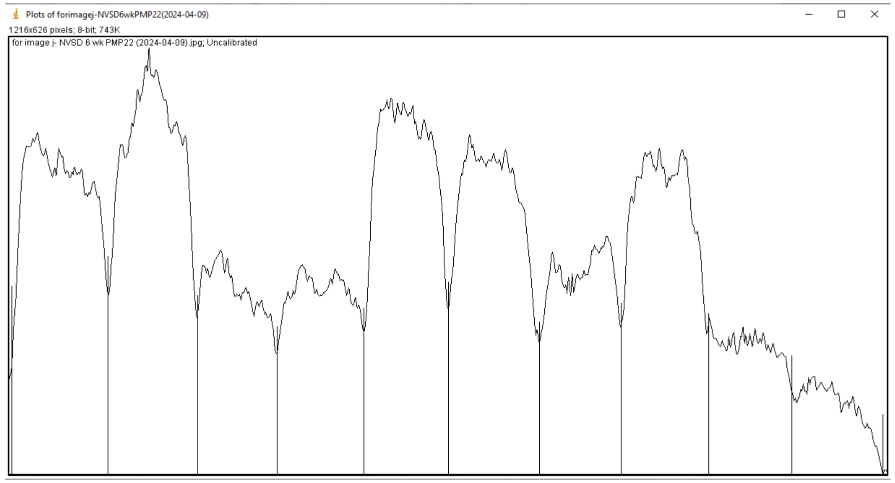

Date: 23 Apr 2024 LMW

Plot 2 Values B-Actin

	Area
1	43314.73
2	44546.49
3	47999.61
4	55235.85
5	42497.49
6	43198.49
7	45119.32
8	48115.32
9	47334.02
10	46429.2

Plot 2-B-Actin

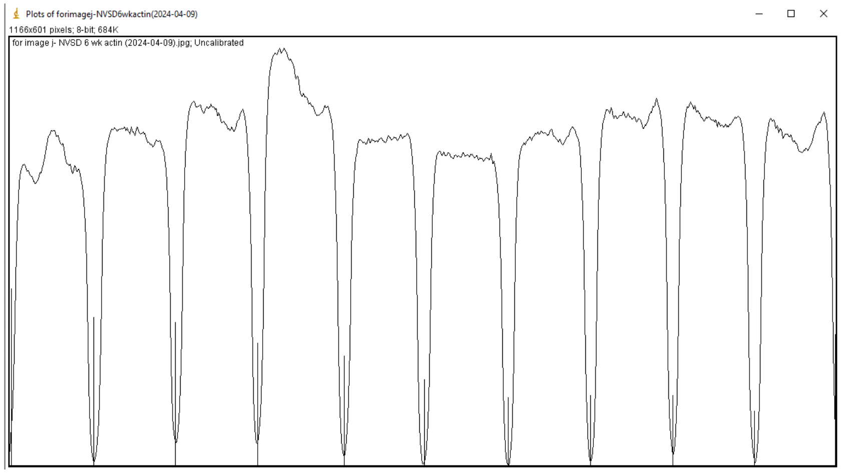

Date: 23 Apr 2024 LMW

Western Blot Imaging Form

Plot 3 Values-MPZ

	Area
1	67710.49
2	54425.05
3	53419.05
4	57118.13
5	49019.81
6	45405.88
7	46895.76
8	44442.59
9	49801.95
10	46560.73

Plot 3-MPZ

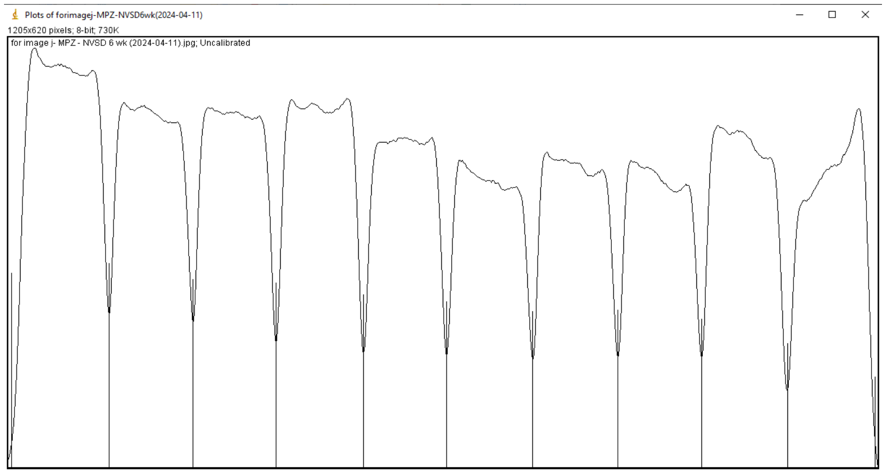

Date: 23 Apr 2024 LMW

Operator Information

**Performed By:**	**Name:** Merlin P Thangaraj	**Signature:**
	**Position/Lab:** Postdoctoral Scientist / Harper Lab	**Date:** 27 Jun 2024

**Performed By:**	**Name:** Lindsay Wallace	**Signature:**
	**Position/Lab:** Senior Research Scientist/Harper Lab	**Date:** 23 Apr 2024

Western Blot Imaging Form

**Study**	ARM101-CMT1A-NHP-001
**Timepoint**	12 wk
**Tissue**	Sciatic Nerve
**Anatomical Location** (Circle/Highlight one)	Distal Proximal N/A Other:

Stain Free Gel

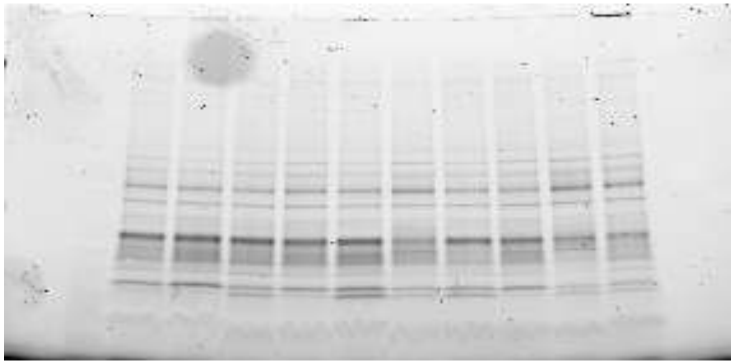

Date: 22 Feb 2024 MPT

Stain Free Membrane

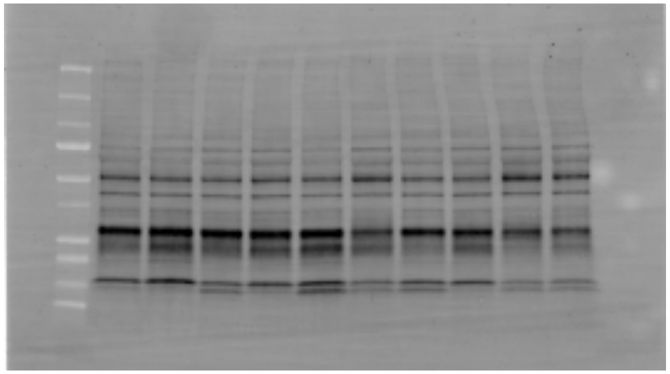

Date: 22 Feb 2024 MPT

1° PMP22

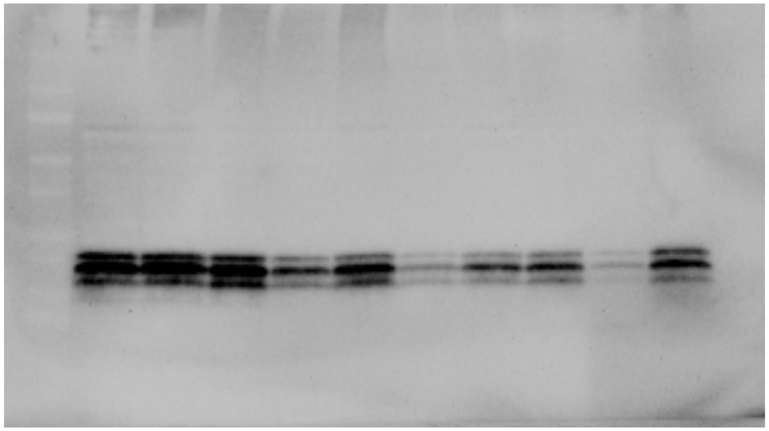

Date: 23 Feb 2024 MPT

1° β-Actin

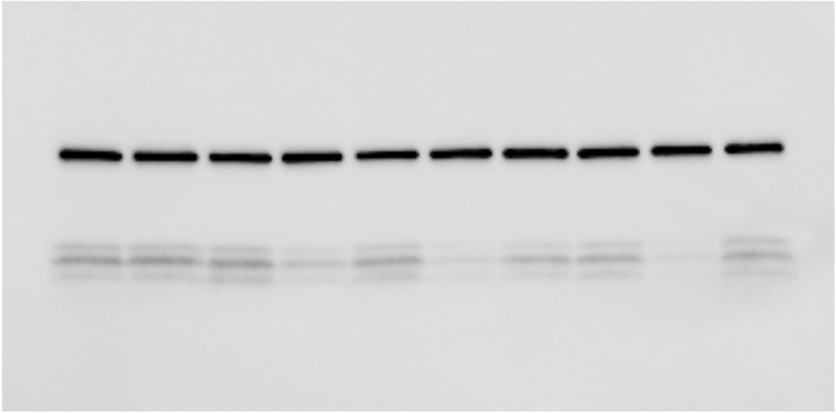

Date: 23 Feb 2024 MPT

Western Blot Imaging Form

Strip Check

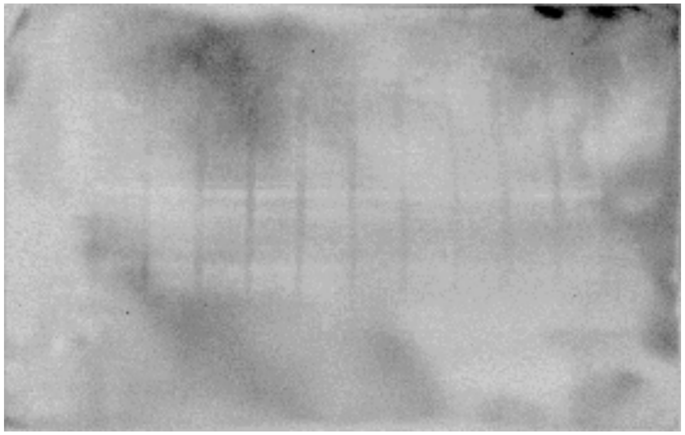

Date: 27 Feb 2024 MPT

1° MPZ

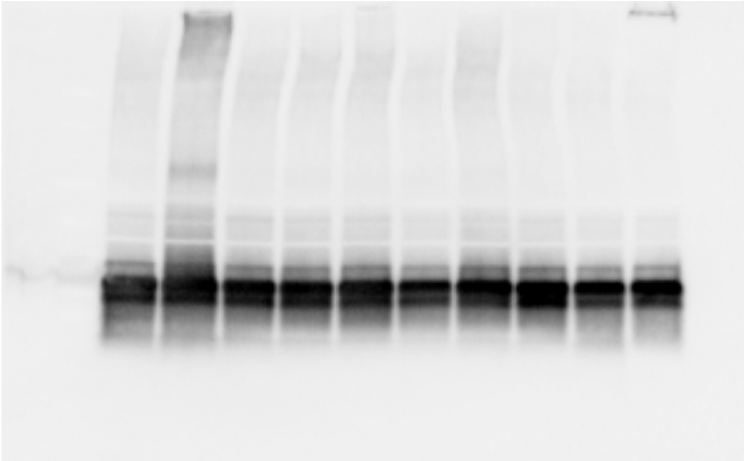

Date: 28 Feb 2024 MPT

ImageJ Quantification Box – PMP22

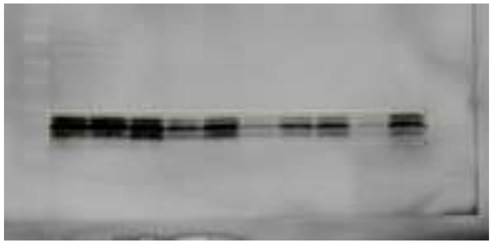

Date: 01 Mar 2024 LMW

ImageJ Quantification Box – B-Actin

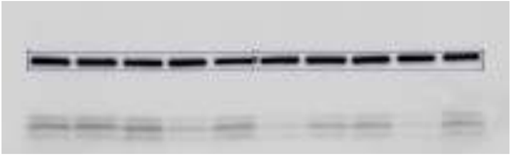

Date: 29 FEB 2024- LMW

ImageJ Quantification Box - MPZ

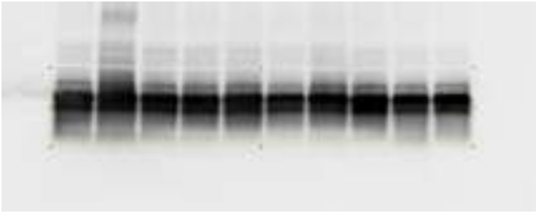

Date:01 Mar 2024 LMW

Plot 1 Values PMP22

#	Area
1	64393.51
2	62907.63
3	58943.56
4	27803.02
5	45923.34
6	4759.075
7	20086.39
8	27521.68
9	4649.539
10	46654.92

Plot 1-PMP22

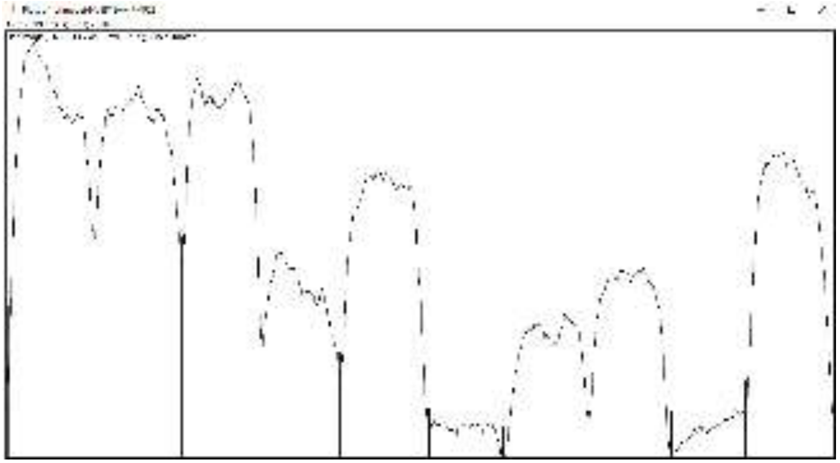

Date:01 Mar 2024 LMW

Plot 2 Values B-Actin

#	Area
1	56785.610
2	55089.974
3	49932.731
4	51428.731
5	45158.024
6	50118.267
7	55212.681
8	52368.024
9	49558.439
10	45802.196

Plot 2-B-Actin

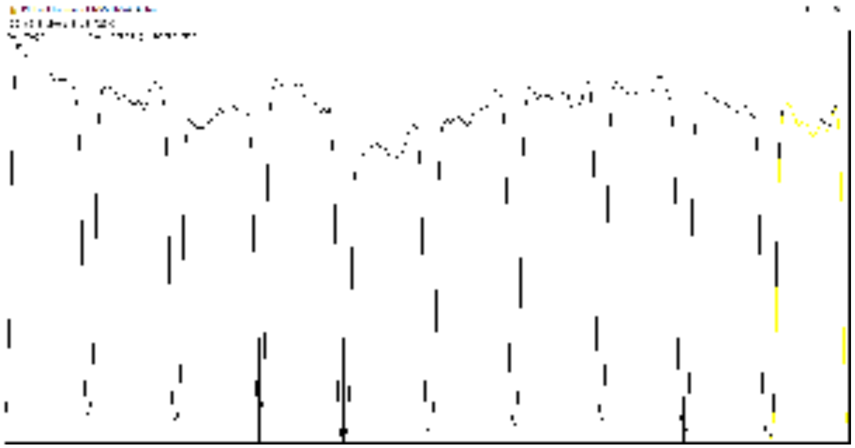

Date:29 FEB 2024 - LMW

Plot 3 Values-MPZ

#	Area
1	53082
2	66128.78
3	50528.64
4	50028
5	53045.42
6	41805.47
7	52624.2
8	50275.71
9	42346.54
10	41393.71

Plot 3-MPZ

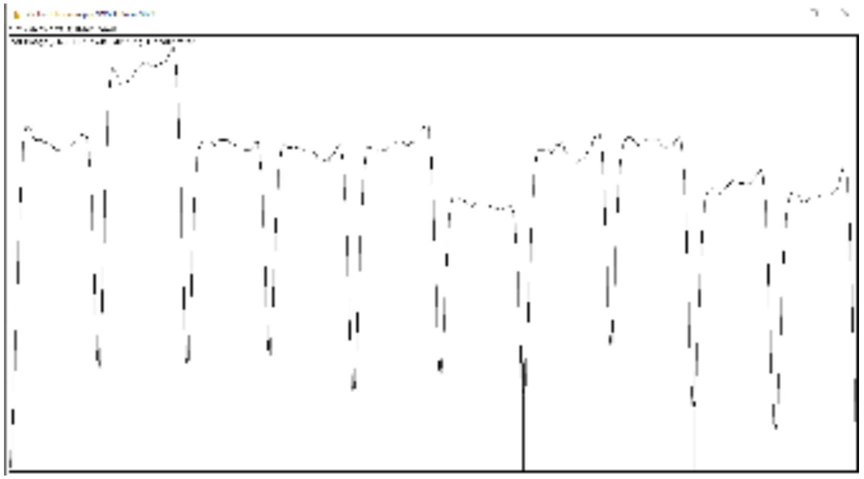

Date:

Operator Information

**Performed By:**	**Name: Merlin P Thangaraj**	**Signature:**
	**Position/Lab: Postdoctoral Scientist / Harper Lab**	**Date: 12 Mar 2024**

**Performed By:**	**Name: Lindsay Wallace**	**Signature:**
	**Position/Lab: Sr Research Sci/Harper Lab**	**Date: 01 Mar 2024**

Western Blot Imaging Form

**Study**	ARM101-CMT1A-NHP-001
**Timepoint**	6 week
**Tissue**	Sciatic nerve - right
**Anatomical Location** (Highlight/Circle one)	Distal (circled) Proximal N/A Other:

Stain Free Gel

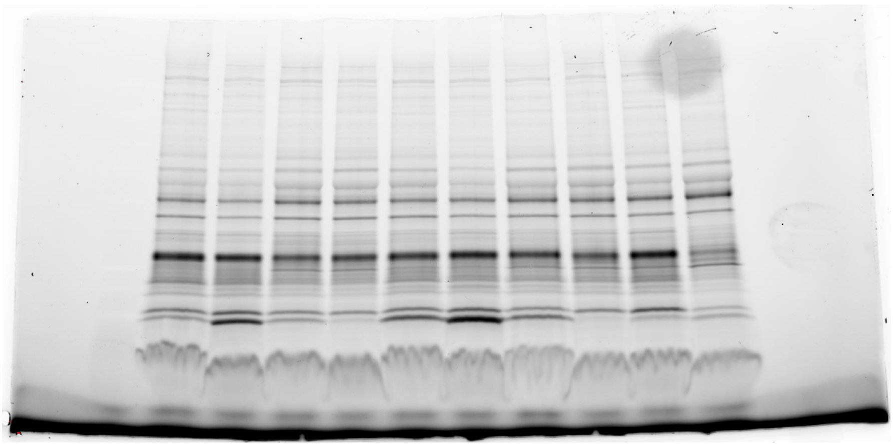

Date: 19 May 2025 MPT

Stain Free Membrane

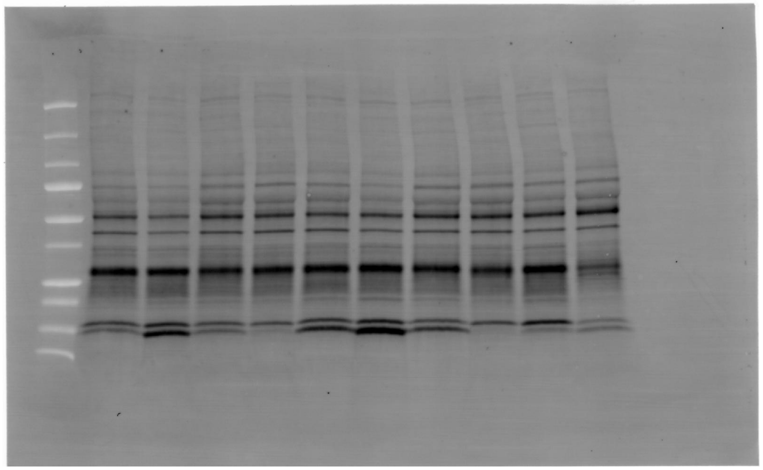

Date: 19 May 2025 MPT

1° PMP22

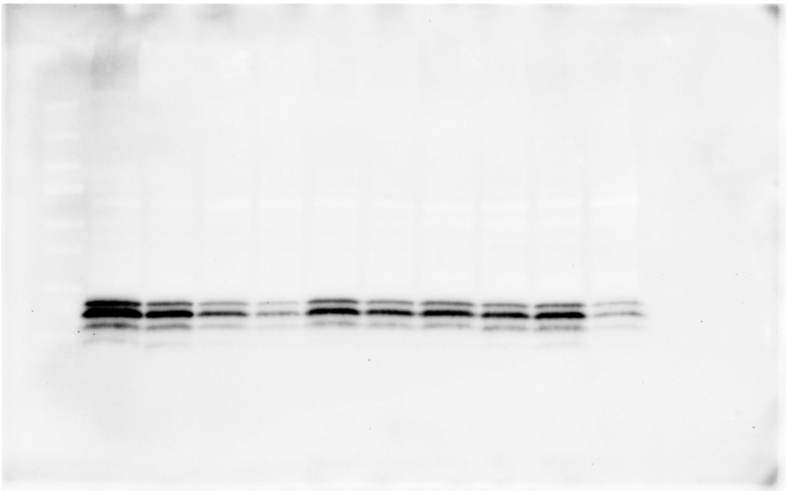

Date: 20 May 2025 MPT

1° β-Actin

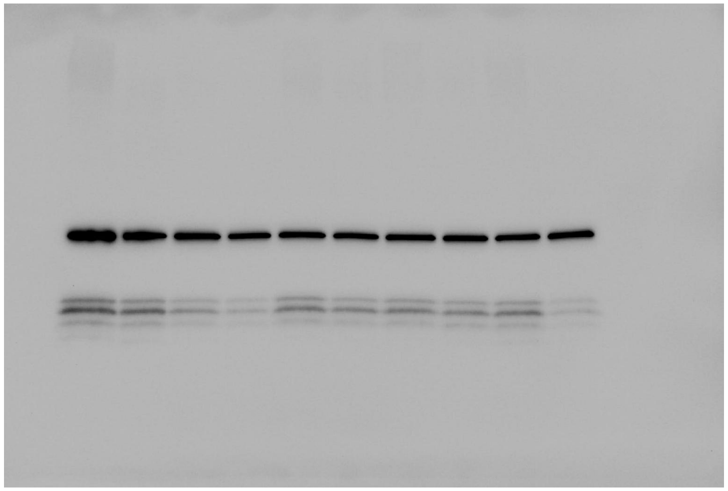

Date: 20 May 2025 MPT

Western Blot Imaging Form

Strip Check

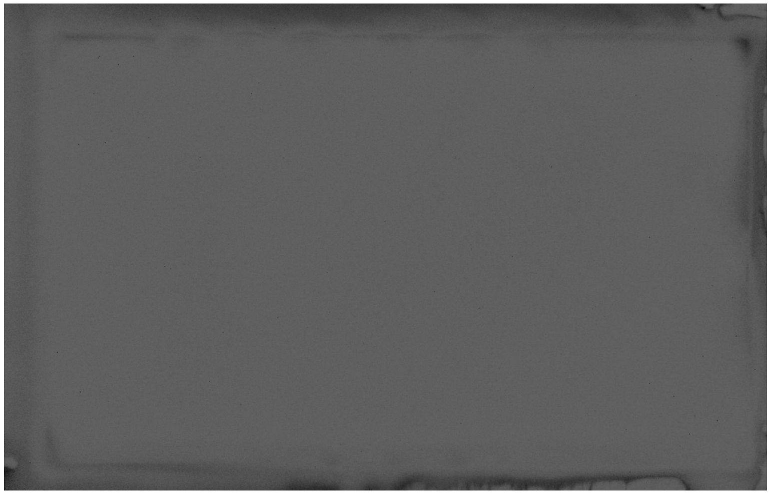

Date: 22 May 2025 MPT

1° MPZ

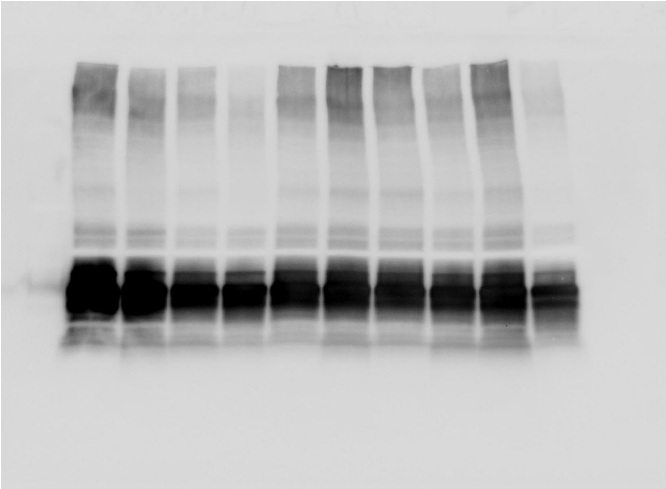

Date: 22 May 2025 MPT

ImageJ Quantification Box – PMP22

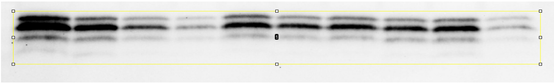

Date: 23 May 2025 LMW

ImageJ Quantification Box – B-Actin

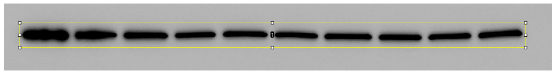

Date: 23 May 2025 LMW

ImageJ Quantification Box - MPZ

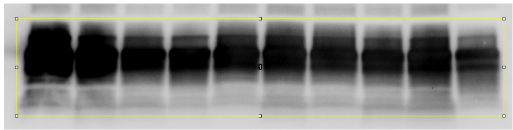

Date: 23 May 2025 LMW

<u>Plot 1 Values PMP22</u>

	Area
1	117737
2	65568.75
3	34540.95
4	13317.85
5	57873.97
6	40475.29
7	51986.31
8	42359.53
9	54494.14
10	11144

Plot 1-PMP22

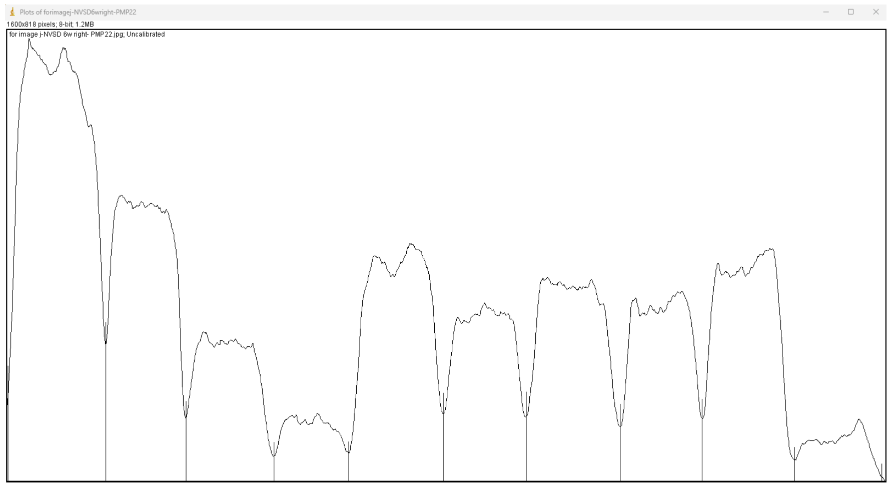

Date: 23 May 2025 LMW

<u>Plot 2 Values B-Actin</u>

	Area
1	55313.9
2	39501.95
3	36631.78
4	28466.23
5	31623.54
6	29172.13
7	33287.1
8	30360.47
9	29068
10	32016.49

Plot 2-B-Actin

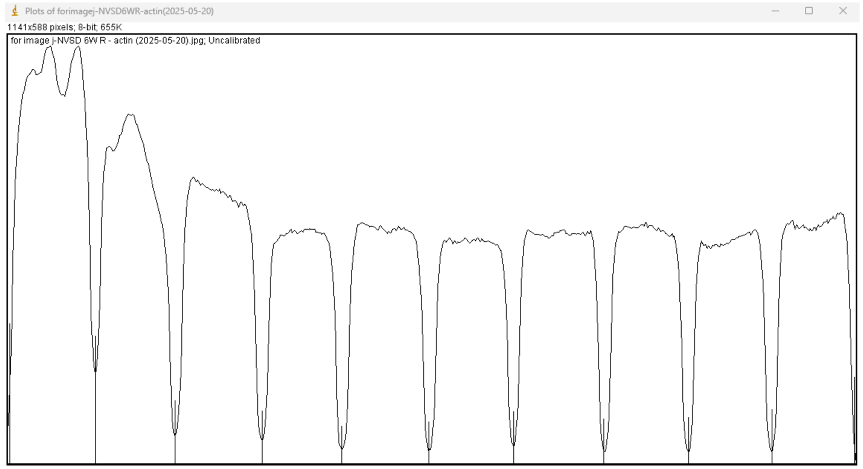

Date: 23 May 2025 LMW

Western Blot Imaging Form

Plot 3 Values-MPZ

	Area
1	65724.85
2	53448.42
3	45245.15
4	40729.91
5	45905.93
6	47326.47
7	45219.93
8	41571.47
9	52490
10	36960.1

Plot 3-MPZ

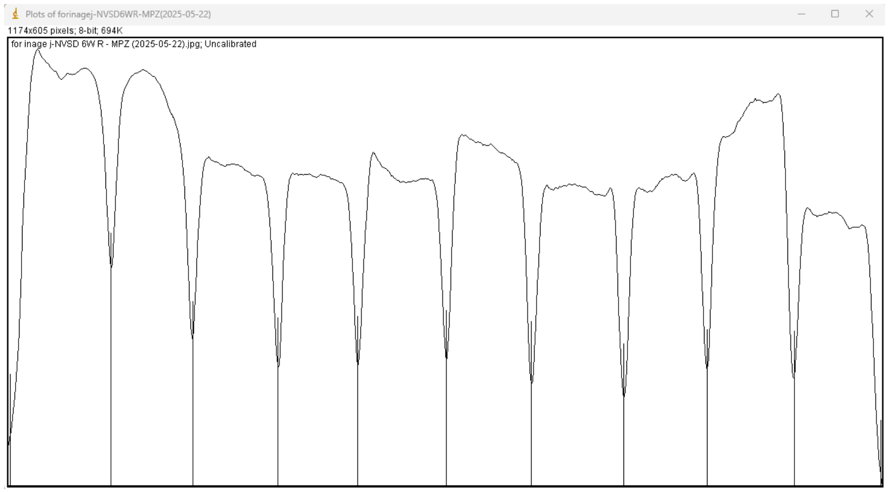

Date: 23 Apr 2024 LMW

Operator Information

**Performed By:**	**Name:** Merlin P Thangaraj	**Signature:** [signature]
	**Position/Lab:** Postdoctoral Scientist/Harper Lab	**Date:** 8-1-2025

**Performed By:**	**Name:** Lindsay Wallace	**Signature:** [signature]
	**Position/Lab:** Senior Research Scientist/Harper Lab	**Date:** 27 May 2025

Western Blot Imaging Form

Study	ARM101-CMT1A-NHP-001
Timepoint	12 week
Tissue	Sciatic nerve - right
Anatomical Location (Highlight/Circle one)	Distal (circled) Proximal N/A Other:

Stain Free Gel

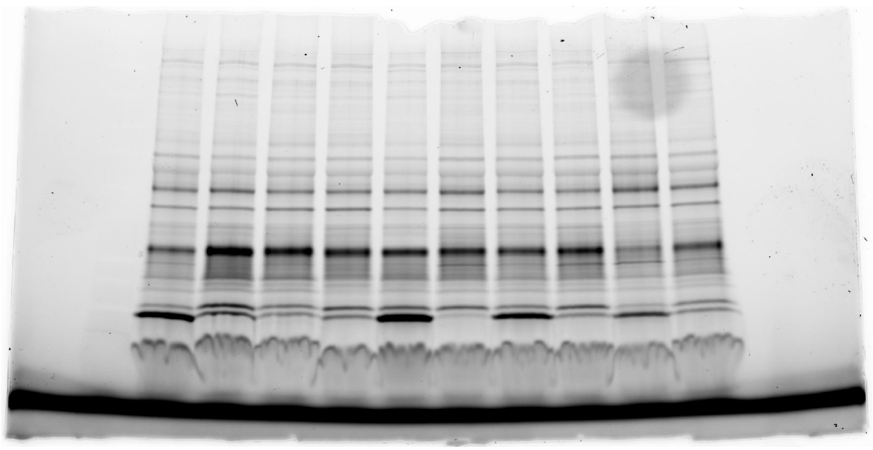

Date: 27 May 2025 MPT

Stain Free Membrane

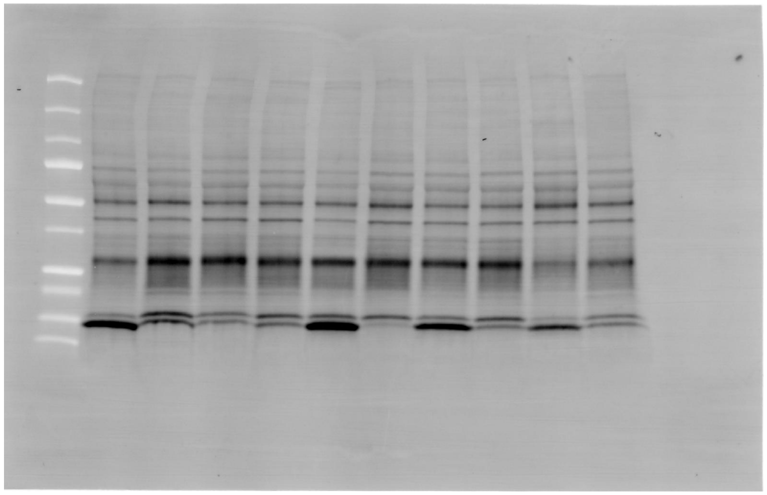

Date: 27 May 2025 MPT

1° PMP22

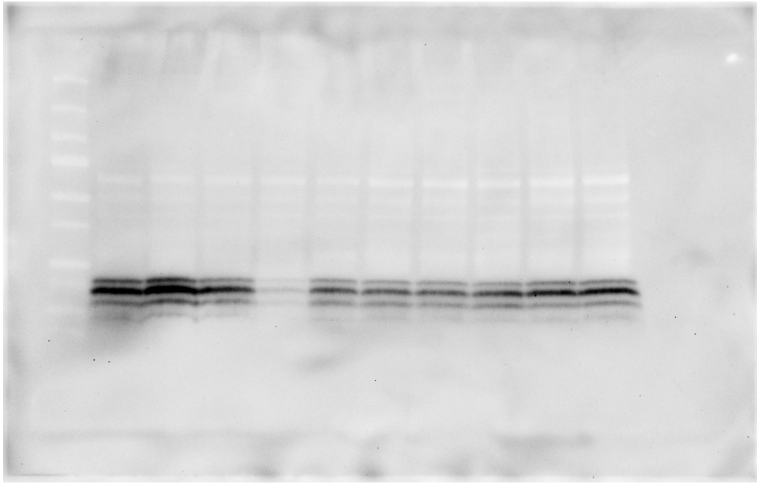

Date: 28 May 2025 MPT

1° β-Actin

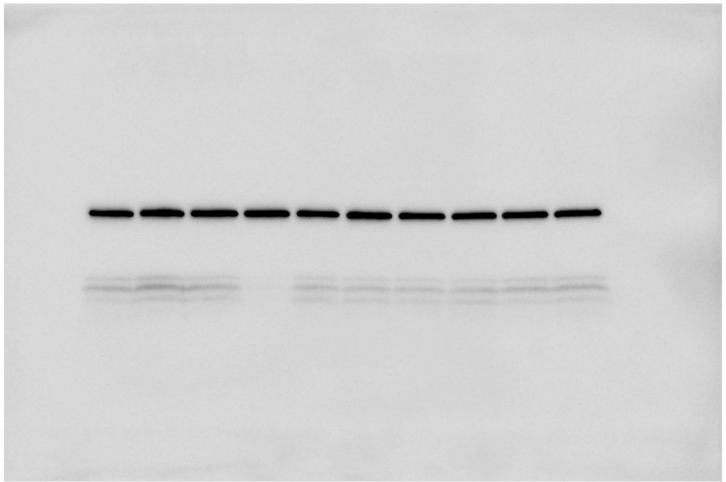

Date: 28 May 2025 MPT

Western Blot Imaging Form

Strip Check

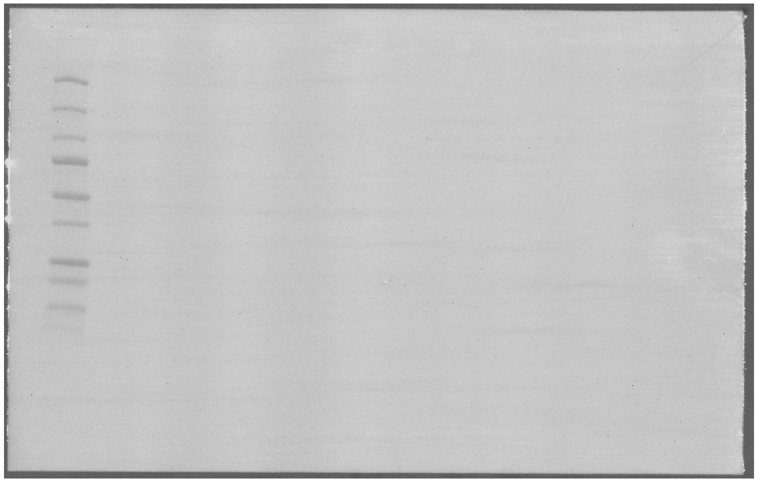

Date: 28 May 2025 MPT

1° MPZ

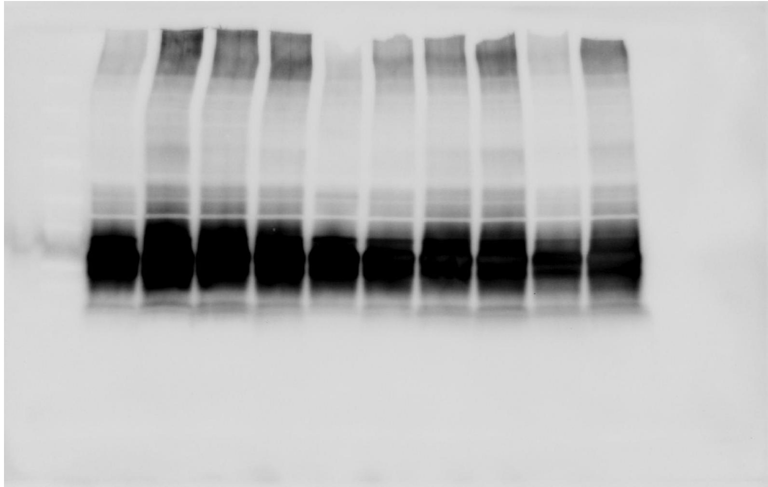

Date: 29 May 2025 MPT

ImageJ Quantification Box – PMP22

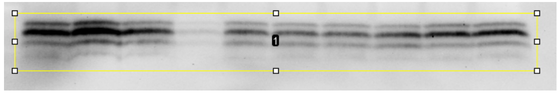

Date: 30 May 2025 LMW

ImageJ Quantification Box – B-Actin

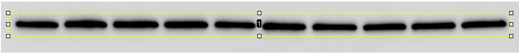

Date: 30 May 2025 LMW

ImageJ Quantification Box - MPZ

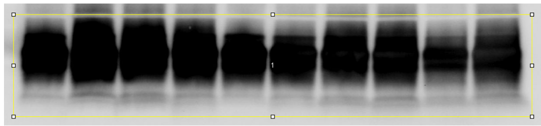

Date: 30 May 2025 LMW

Western Blot Imaging Form

Plot 1 Values PMP22

	Area
1	111084.3
2	100799.8
3	56110.43
4	6776.66
5	43222.41
6	43643.82
7	42722.95
8	49235.77
9	54075.95
10	59161.92

Plot 1-PMP22

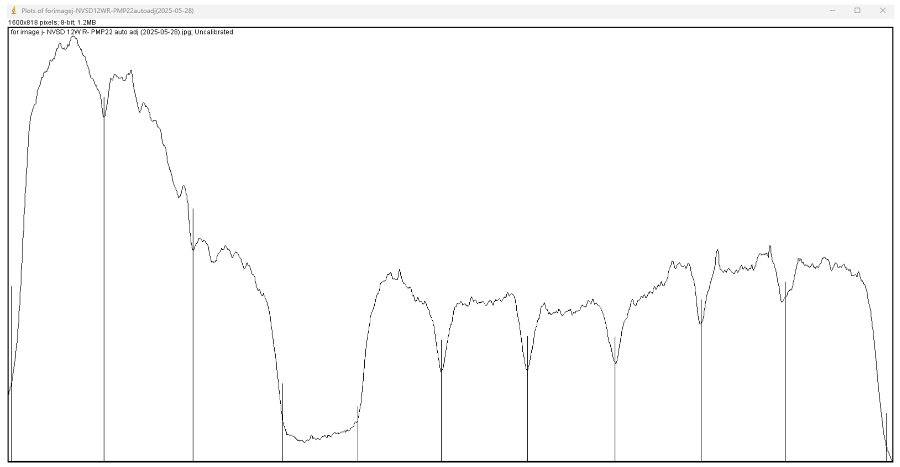

Date: 30 May 2025 LMW

Plot 2 Values B-Actin

	Area
1	48160.66
2	52807.2
3	54838.44
4	53502.95
5	45893.49
6	52786.78
7	48062.2
8	47335.37
9	48349
10	48802.32

Plot 2-B-Actin

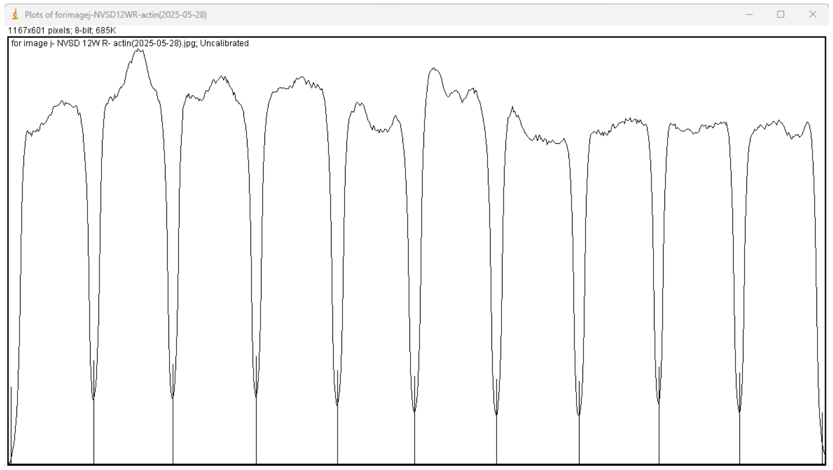

Date: 30 May 2025 LMW

Western Blot Imaging Form

Plot 3 Values-MPZ

	Area
1	53275.71
2	62736.91
3	62361.4
4	57336.69
5	49448.18
6	49705.93
7	55096
8	55762.69
9	41532.76
10	54459.15

Plot 3-MPZ

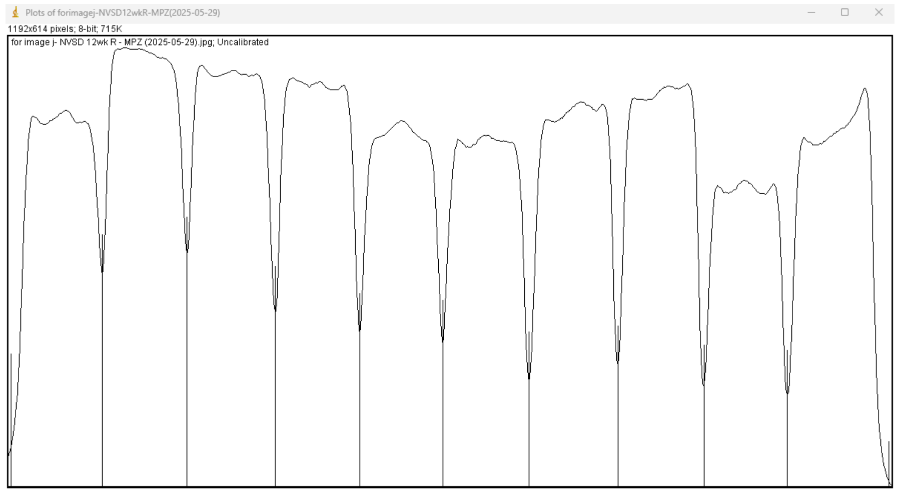

Date: 30 May 2025 LMW

Operator Information

Performed By:	Name: Merlin P Thangaraj	Signature: [signature]
	Position/Lab: Postdoctoral Scientist/Harper Lab	Date: 8-1-2025

Performed By:	Name: Lindsay Wallace	Signature: [signature]
	Position/Lab: Senior Research Scientist/Harper Lab	Date: 30 May 2025

Western Blot Imaging Form

**Study**	ARM101-CMT1A-NHP-001
**Timepoint**	6 week
**Tissue**	Sciatic nerve
**Anatomical Location** (Highlight/Circle one)	Distal ~~~ Proximal (circled) ~~~ N/A<br>Other:

Stain Free Gel

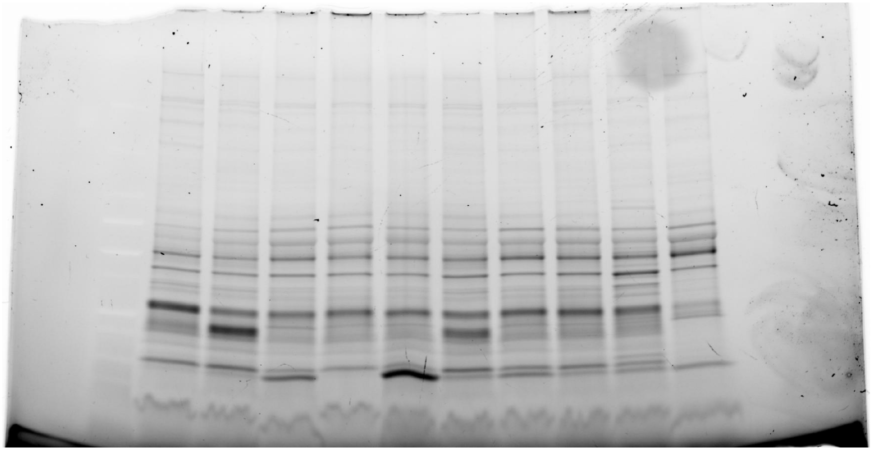

Date: 08 Arp 2024 MPT

Stain Free Membrane

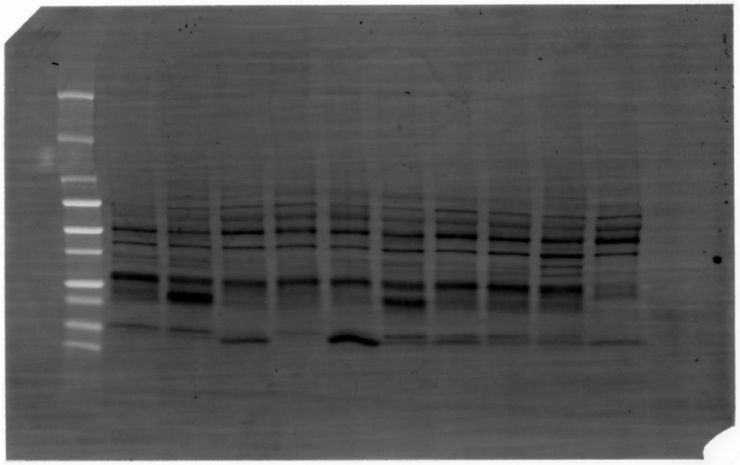

Date: 08 Arp 2024 MPT

1° PMP22

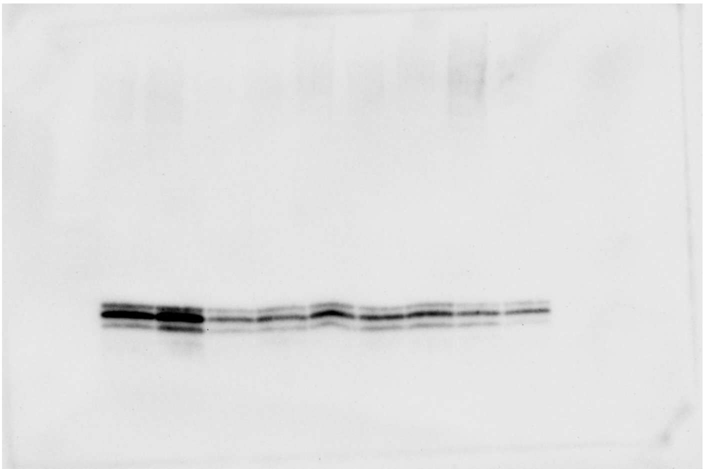

Date: 09 Apr 2024 MPT

1° β-Actin

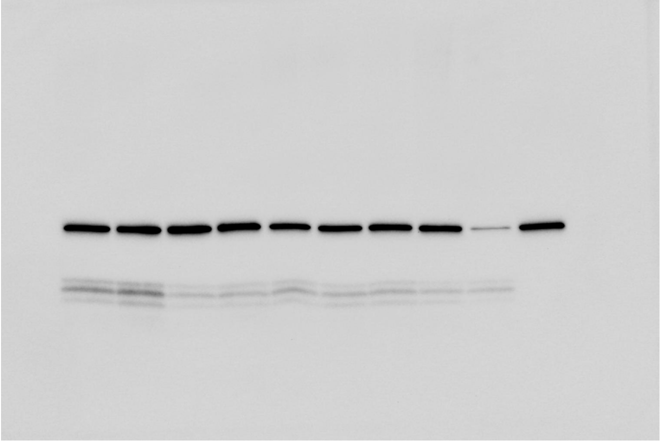

09 Apr 2024 MPT
Date:

Western Blot Imaging Form

Strip Check

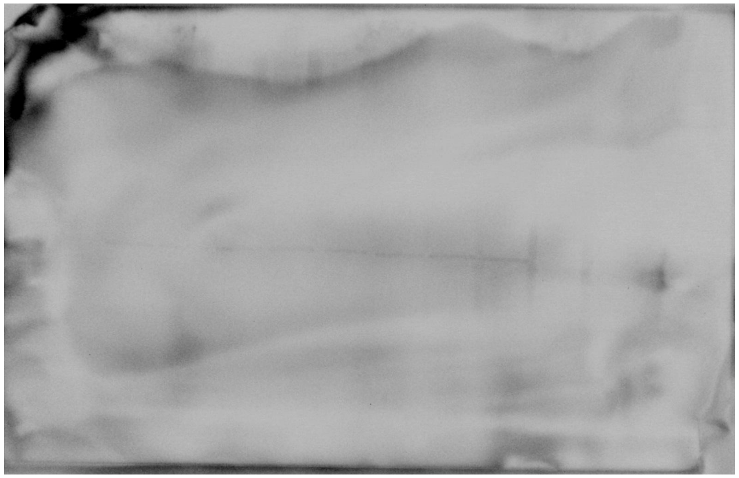

Date: 10 Apr 2024 MPT

1° MPZ

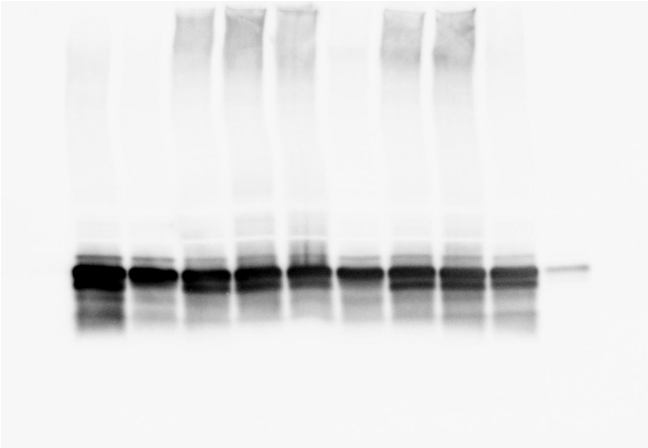

Date: 11 Apr 2024 MPT

ImageJ Quantification Box – PMP22

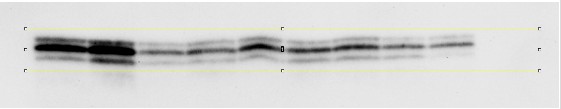

Date: 23 Apr 2024 LMW

ImageJ Quantification Box – B-Actin

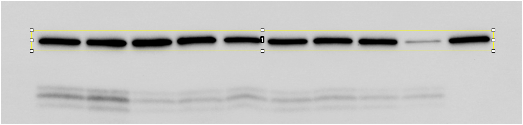

Date: 23 Apr 2024 LMW

ImageJ Quantification Box - MPZ

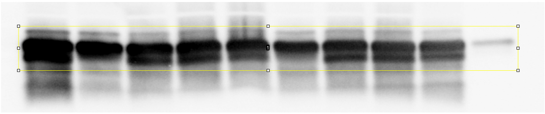

Date: 23 Apr 2024 LMW

Plot 1 Values PMP22

	Area
1	54016.17
2	65048.97
3	19153.71
4	25499.32
5	31497.9
6	25131.78
7	27152.44
8	14621.25
9	11559.78
10	787.598

Plot 1-PMP22

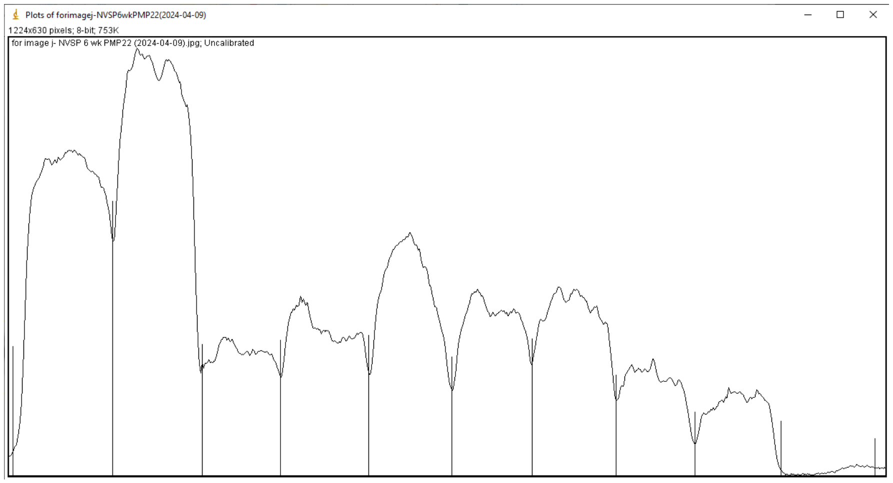

Date: 23 Apr 2024 LMW

Plot 2 Values B-Actin

	Area
1	53967.1
2	54898.49
3	57459.32
4	53175.73
5	42142.2
6	45769.37
7	47237.49
8	45521.15
9	6392.832
10	49248.68

Plot 2-B-Actin

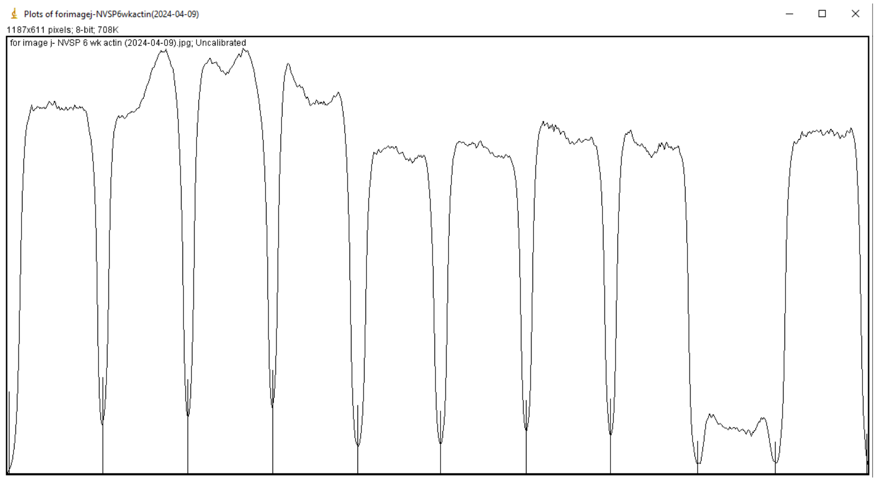

Date: 23 Apr 2024 LMW

Western Blot Imaging Form

Plot 3 Values-MPZ

	Area
1	67047.95
2	46203.78
3	49887.37
4	51286.61
5	44120.78
6	35890.71
7	44859.88
8	41375.54
9	33193.54
10	2440.184

Plot 3-MPZ

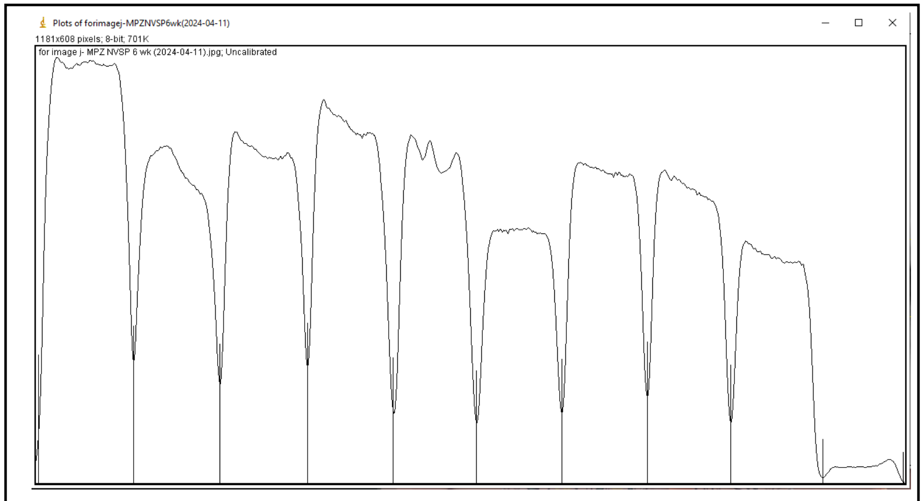

Date: 23 Apr 2024 LMW

Operator Information

**Performed By:**	**Name:** Merlin P Thangaraj	**Signature:** [signature]
	**Position/Lab:** Postdoctoral Scientist / Harper Lab	**Date:** 27 Jun 2024

**Performed By:**	**Name:** Lindsay Wallace	**Signature:** [signature]
	**Position/Lab:** Senior Research Scientist/Harper Lab	**Date:** 23 Apr 2024

Western Blot Imaging Form

**Study**	ARM101-CMT1A-NHP-001
**Timepoint**	12 week
**Tissue**	Sciatic Nerve
**Anatomical Location** (Circle one)	Distal Proximal (circled) N/A Other:

Stain Free Gel

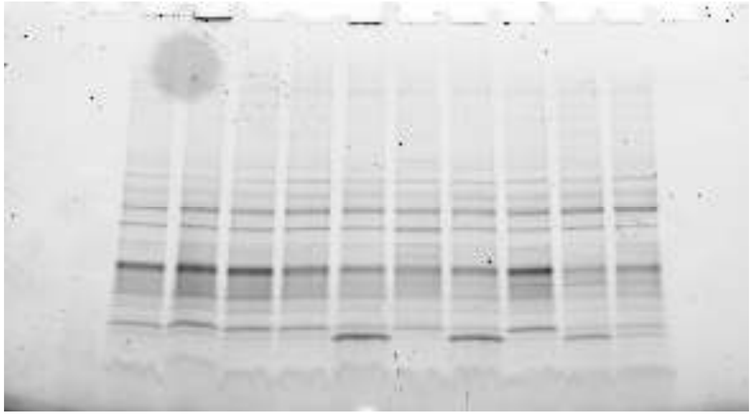

Date: 22 Feb 2024 MPT

Stain Free Membrane

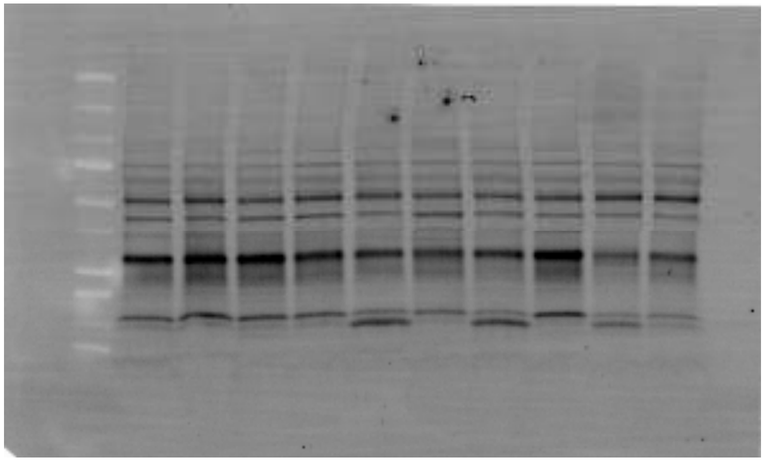

Date: 22 Feb 2024 MPT

1° PMP22

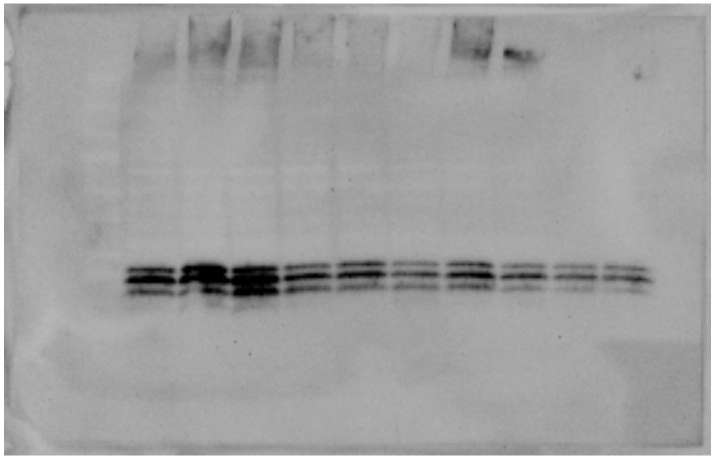

Date: 23 Feb 2024 MPT

1° β-Actin

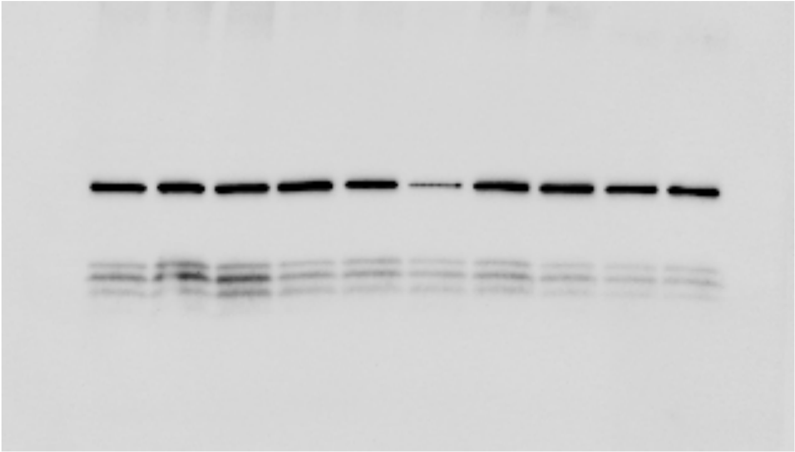

Date: 23 Feb 2024 MPT

Western Blot Imaging Form

Strip Check

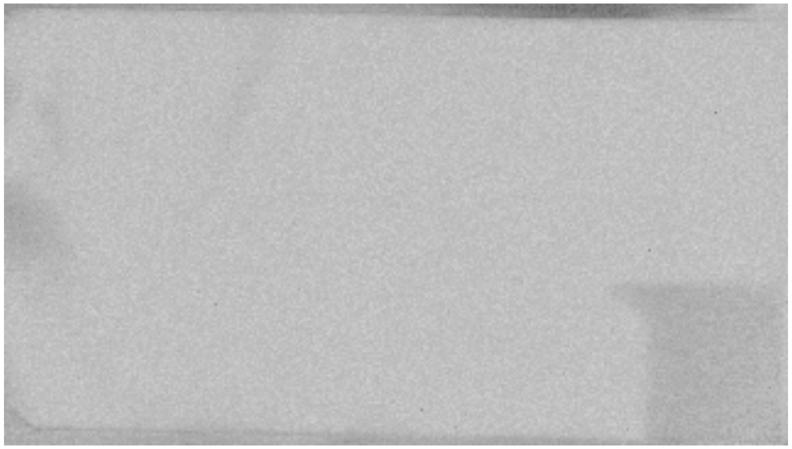

Date: 27 Feb 2024 MPT

1° MPZ

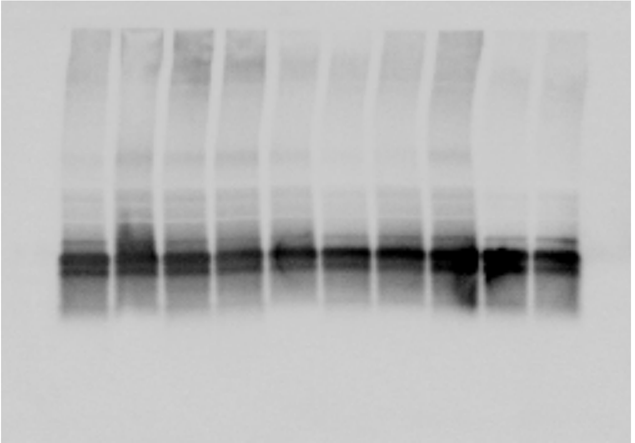

Date: 28 Feb 2024 MPT

ImageJ Quantification Box – PMP22

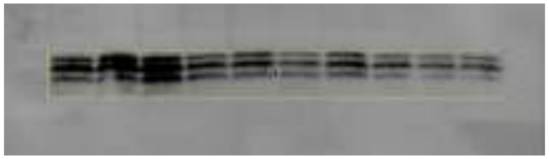

Date: 04 Mar 2024 LMW

ImageJ Quantification Box – B-Actin

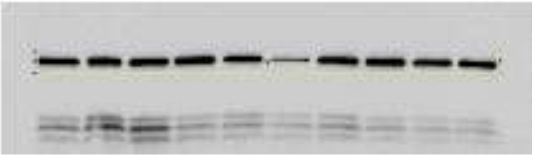

Date: 04 Mar 2024 LMW

ImageJ Quantification Box - MPZ

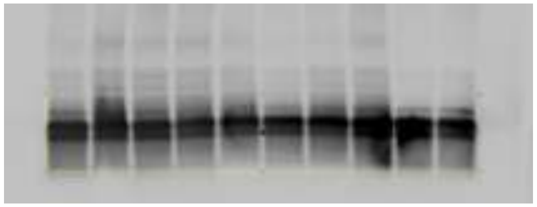

Date: 04 Mar 2024 LMW

Western Blot Imaging Form

Plot 1-PMP22

Plot 1 Values PMP22

#	Area
1	42885.8
2	54569.56
3	63819.56
4	35991.56
5	35523.22
6	23626.97
7	32098.51
8	18964.27
9	14146.2
10	15938.39

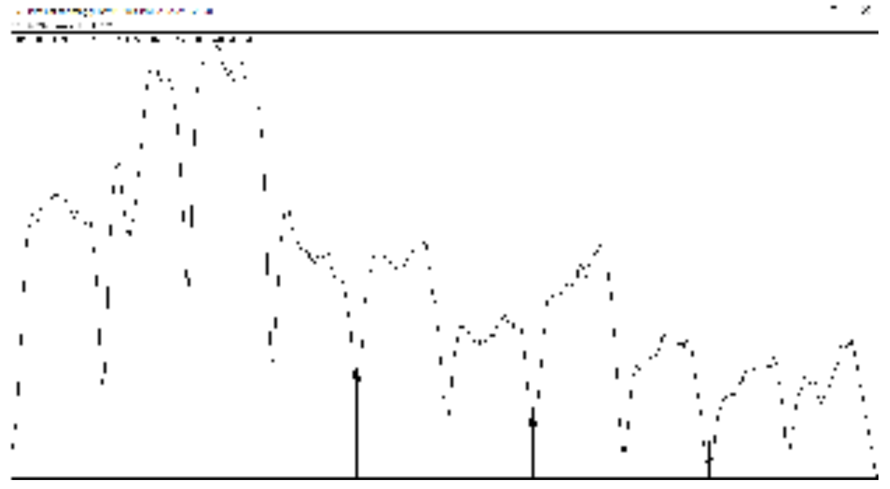

Date: 04 Mar 2024 LMW

Plot 2-B-Actin

Plot 2 Values B-Actin

#	Area
1	54434.974
2	52560.489
3	57011.974
4	61612.974
5	49064.853
6	17101.560
7	58529.510
8	59547.095
9	47553.439
10	49183.317

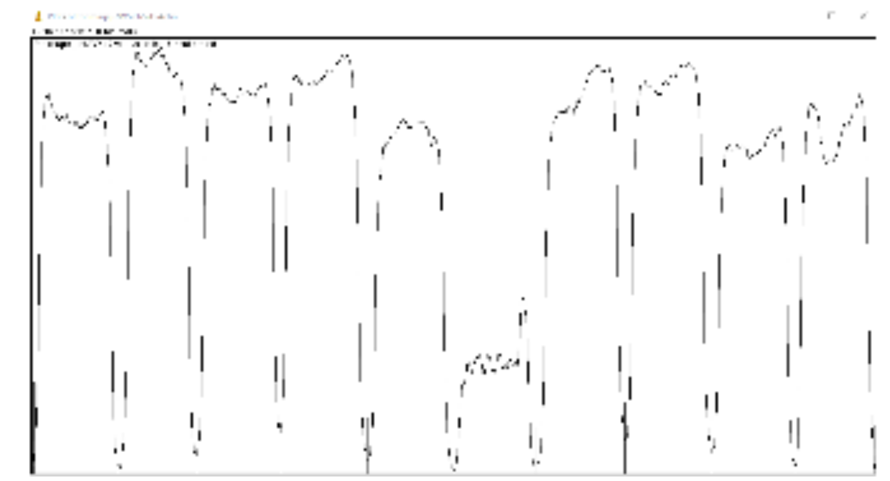

Date: 04 Mar 2024 LMW

Plot 3 Values-MPZ

#	Area
1	41609.32
2	43727.66
3	41125.13
4	39464.32
5	37480.44
6	36114.02
7	42963.15
8	58374.39
9	43933.08
10	40263.56

Plot 3-MPZ

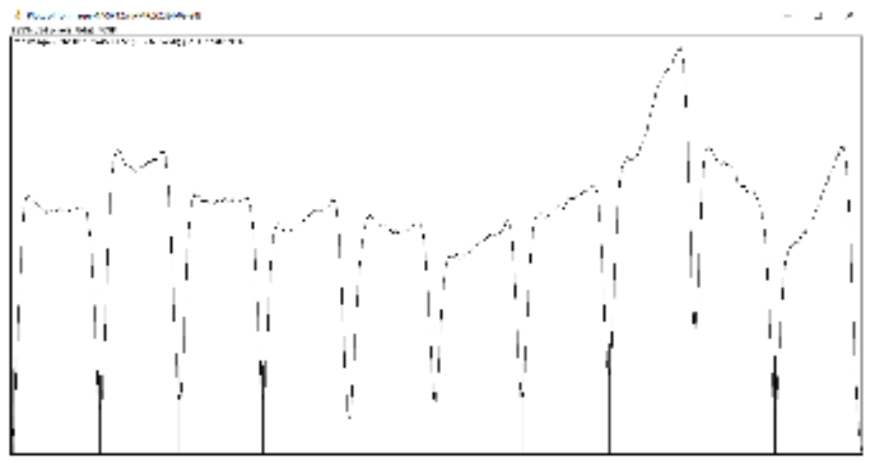

Date: 04 Mar 2024 LMW

Operator Information

**Performed By:**	**Name: Merlin P Thangaraj**	**Signature:**
	**Position/Lab: Postdoctoral Scientist / Harper Lab**	**Date: 12 Mar 2024**

**Performed By:**	**Name: Lindsay Wallace**	**Signature:**
	**Position/Lab: Sr Research Scientist**	**Date: 04 Mar 2024**

Western Blot Imaging Form

**Study**	ARM101-CMT1A-NHP-001
**Timepoint**	6 week
**Tissue**	Sciatic nerve Right
**Anatomical Location** (Highlight/Circle one)	Distal **(Proximal)** N/A Other:

Stain Free Gel

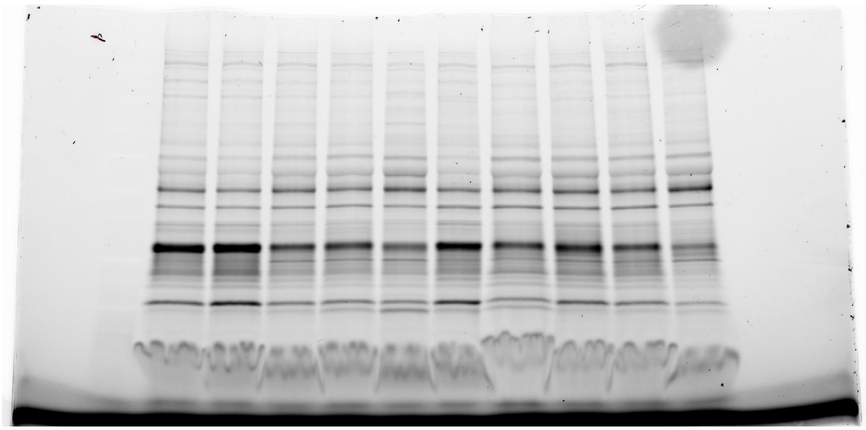

Date: 19 May 2025 MPT

Stain Free Membrane

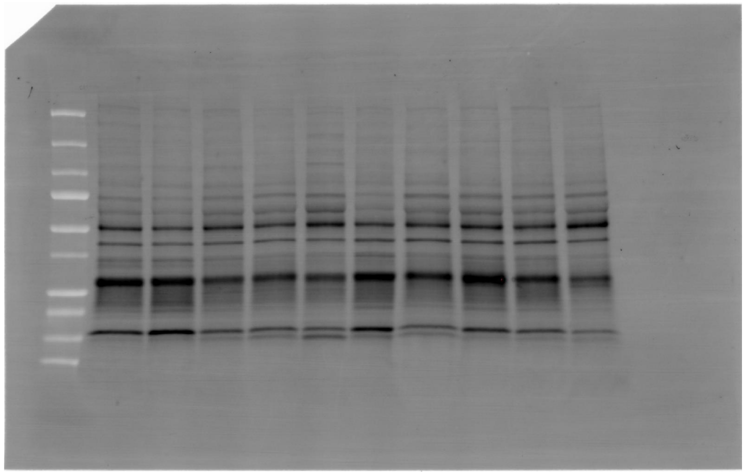

Date: 19 May 2025 MPT

1° PMP22

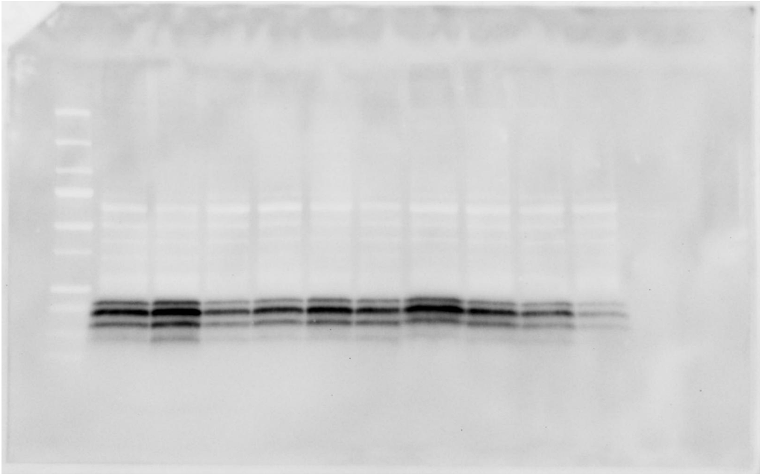

Date: 20 May 2025 MPT

1° β-Actin

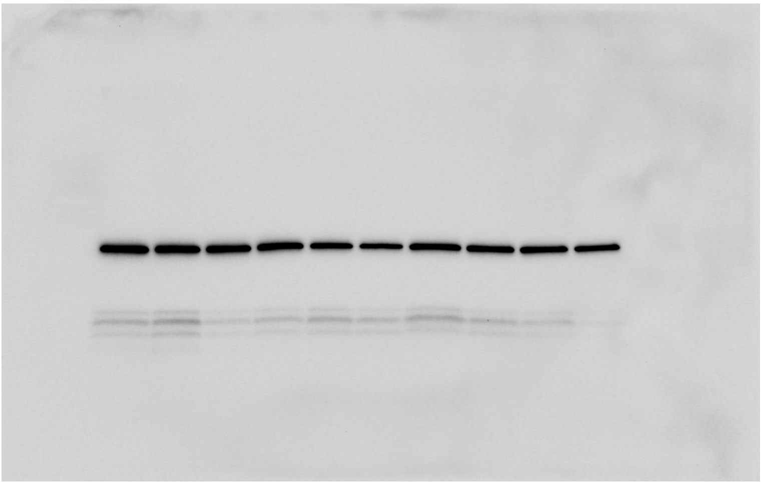

Date: 20 May 2025 MPT

Strip Check

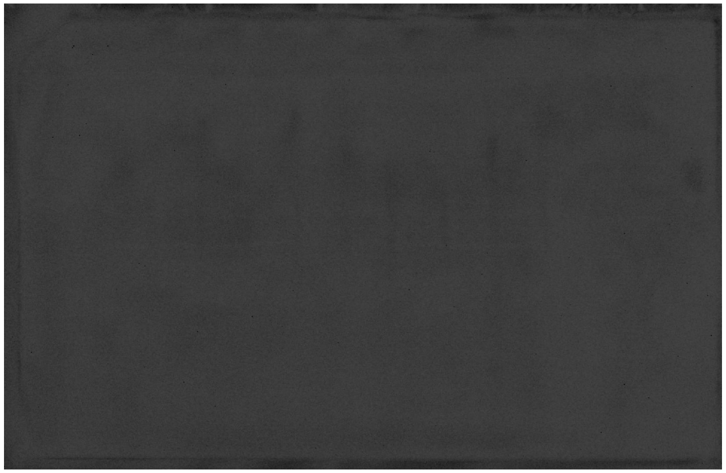

Date: 22 May 2025 MPT

1° MPZ

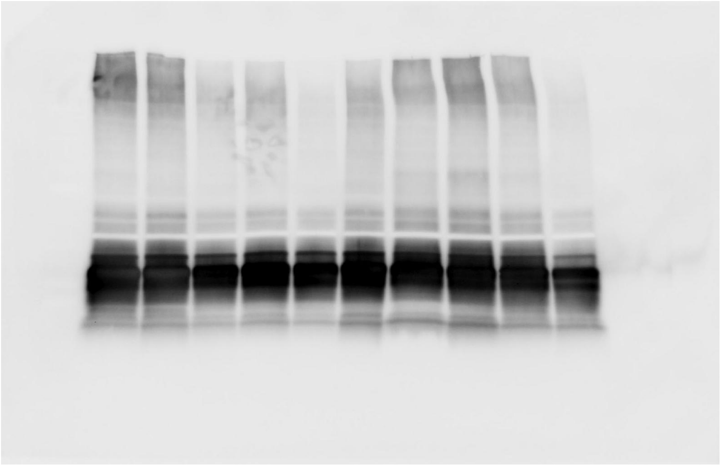

Date: 22 May 2025 MPT

ImageJ Quantification Box – PMP22

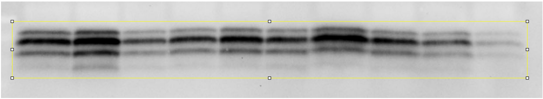

Date: 23 May 2025 LMW

ImageJ Quantification Box – B-Actin

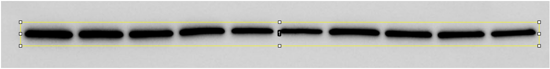

Date: 23 May 2025 LMW

ImageJ Quantification Box - MPZ

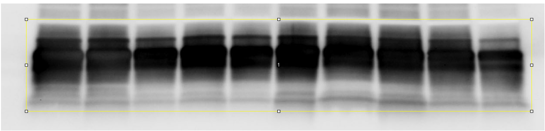

Date: 23 May 2025 LMW

Plot 1 Values PMP22

	Area
1	50102.85
2	60541.66
3	23762.64
4	32575.49
5	40920.83
6	35917.88
7	62851.8
8	41485.49
9	29396.49
10	7882.761

Plot 1-PMP22

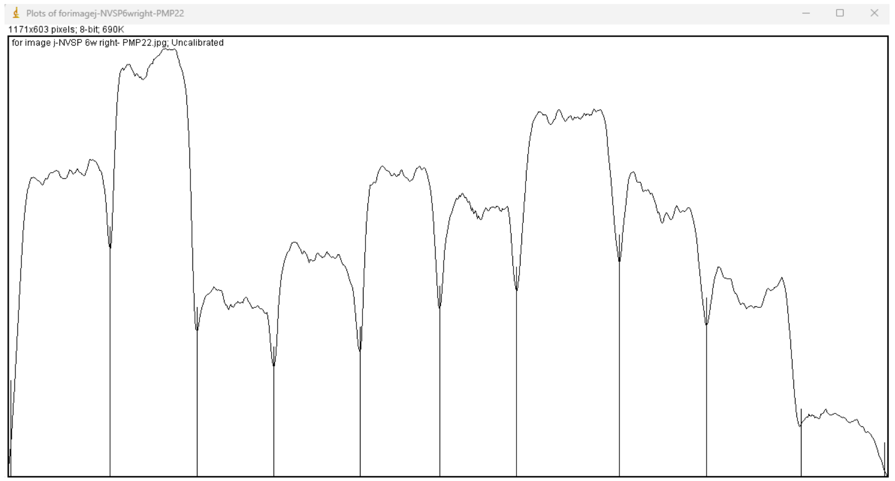

Date: 23 May 2025 LMW

Plot 2 Values B-Actin

	Area
1	55582.49
2	50702.25
3	47367.66
4	45427.54
5	34896.05
6	30395.59
7	45313.61
8	41999.54
9	40889.66
10	34724.3

Plot 2-B-Actin

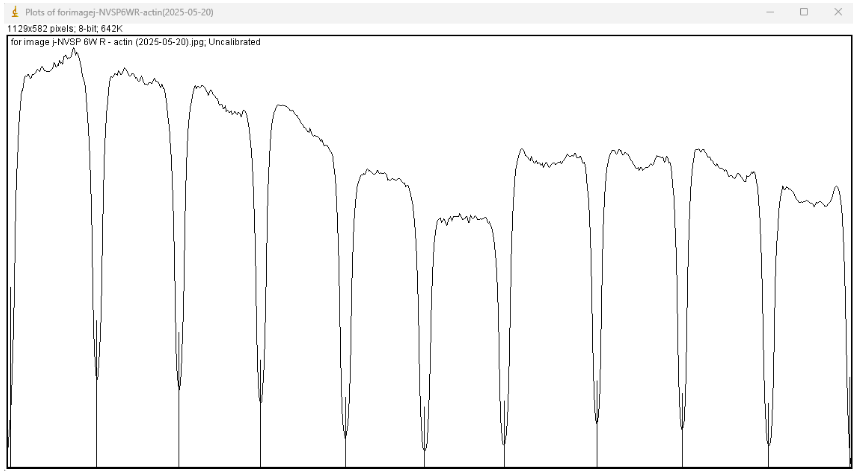

Date: 23 May 2025 LMW

Plot 3 Values-MPZ

	Area
1	60223.73
2	50011.69
3	43015.08
4	52015.45
5	43571.15
6	54724.28
7	56532.05
8	57975.98
9	52653.57
10	42818.9

Plot 3-MPZ

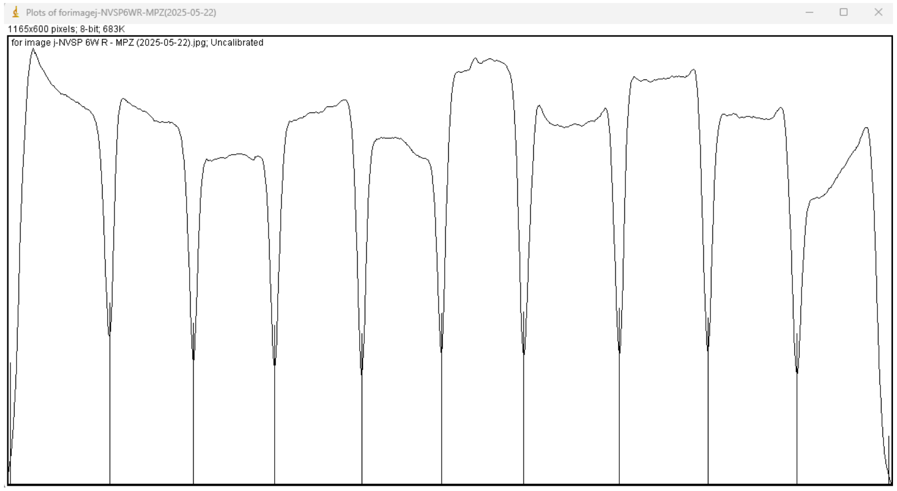

Date: 23 May 2025 LMW

Operator Information

**Performed By:**	**Name:** Merlin P Thangaraj	**Signature:**
	**Position/Lab:** Postdoctoral Scientist/Harper Lab	**Date:** 8-1-2025

**Performed By:**	**Name:** Lindsay Wallace	**Signature:**
	**Position/Lab:** Senior Research Scientist/Harper Lab	**Date:** 23 May 2025

Western Blot Imaging Form

**Study**	ARM101-CMT1A-NHP-001		
**Timepoint**	12 week		
**Tissue**	Sciatic nerve - right		
**Anatomical Location** (Highlight/Circle one)	Distal	Proximal (circled)	N/A
	Other:		

Stain Free Gel

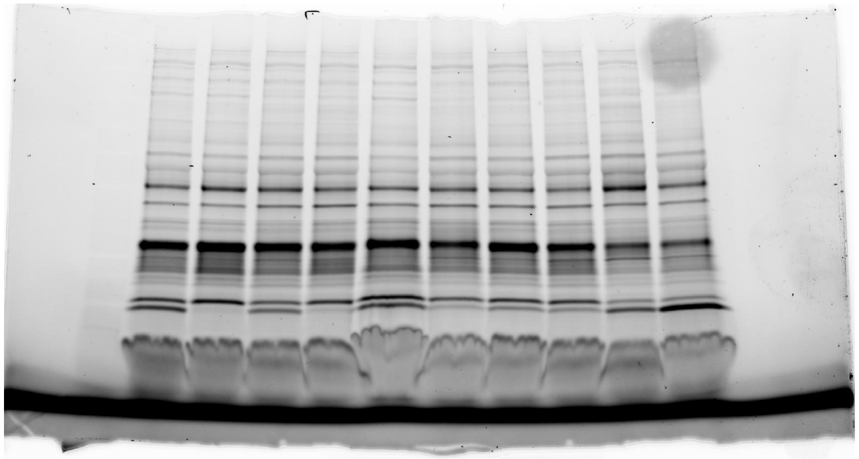

Date: 27 May 2025 MPT

Stain Free Membrane

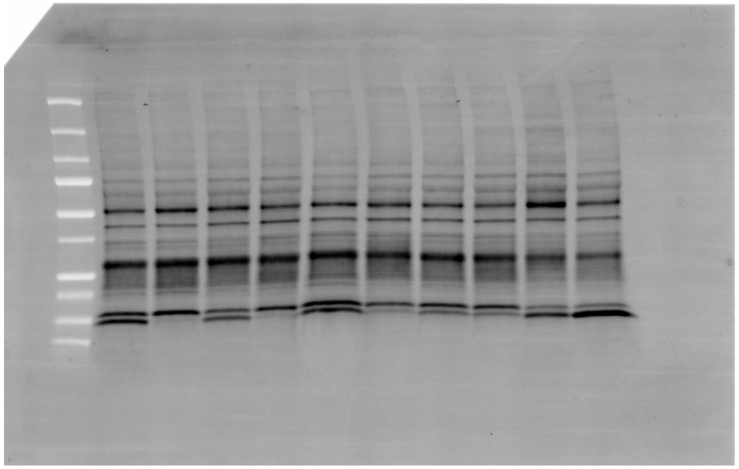

Date: 27 May 2025 MPT

1° PMP22

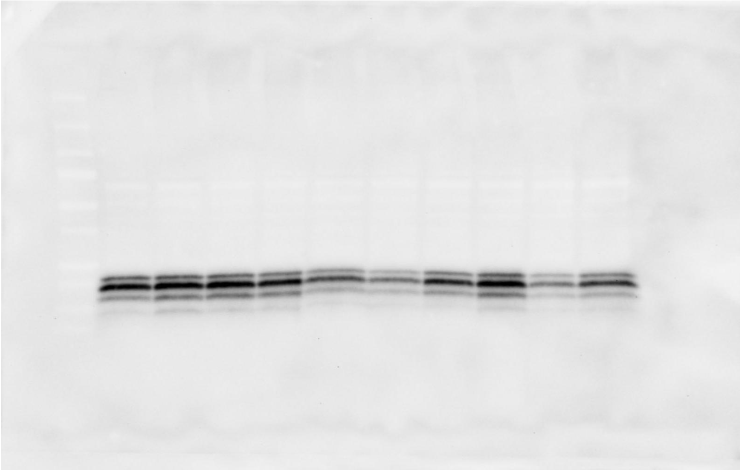

Date: 28 May 2025 MPT

1° β-Actin

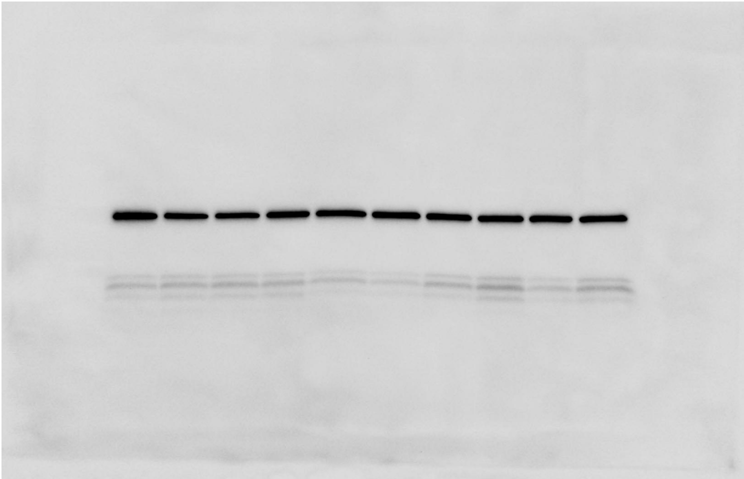

Date: 28 May 2025 MPT

Western Blot Imaging Form

Strip Check

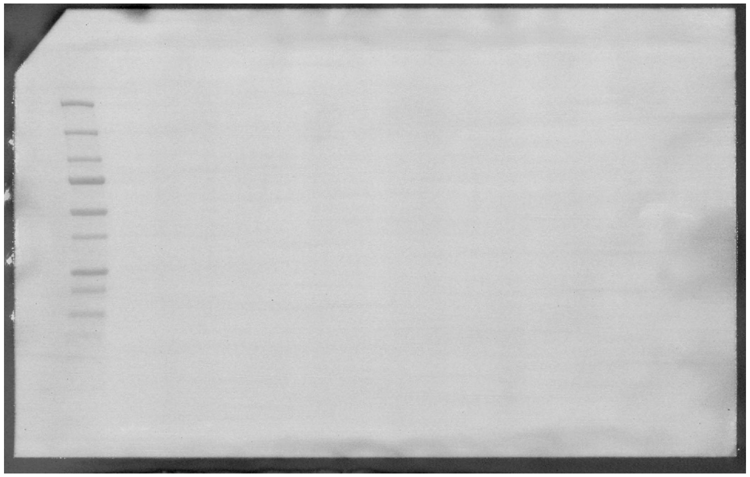

Date: 28 May 2025 MPT

1° MPZ

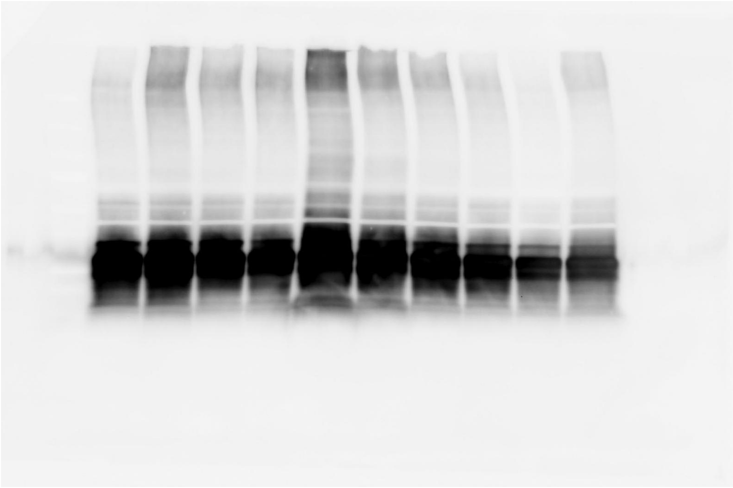

Date: 29 May 2025 MPT

ImageJ Quantification Box – PMP22

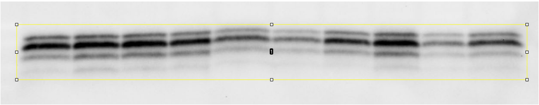

Date: 30 May 2025 LMW

ImageJ Quantification Box – B-Actin

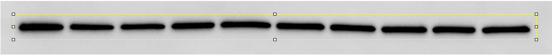

Date: 30 May 2025 LMW

ImageJ Quantification Box - MPZ

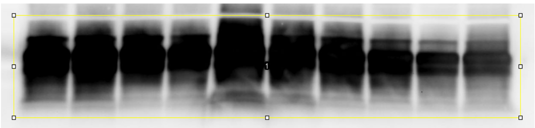

Date: 30 May 2025 LMW

Plot 1 Values PMP22

	Area
1	51418.85
2	58362.49
3	57128.37
4	46151.18
5	39872.8
6	24632.08
7	41225.95
8	58111.66
9	18347.35
10	44272.34

Plot 1-PMP22

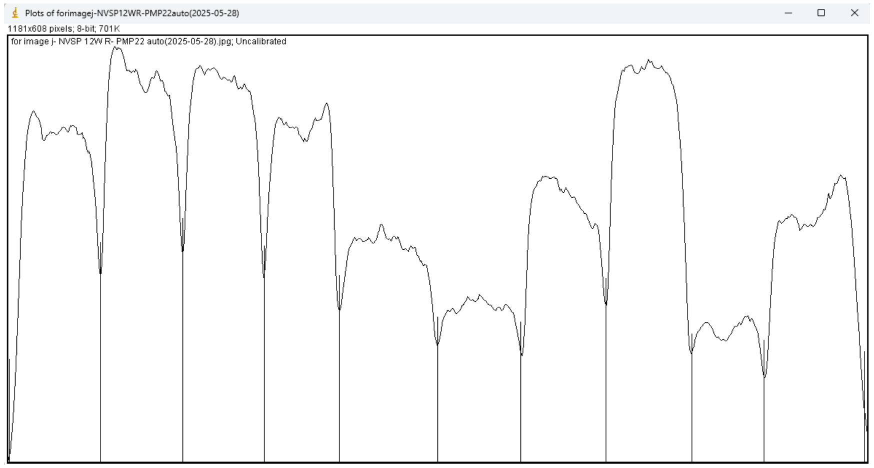

Date: 30 May 2025 LMW

Plot 2 Values B-Actin

	Area
1	53924.08
2	42731.54
3	40926.83
4	44440.59
5	49678.78
6	48130.61
7	44283.66
8	44766.08
9	42811
10	45493.9

Plot 2-B-Actin

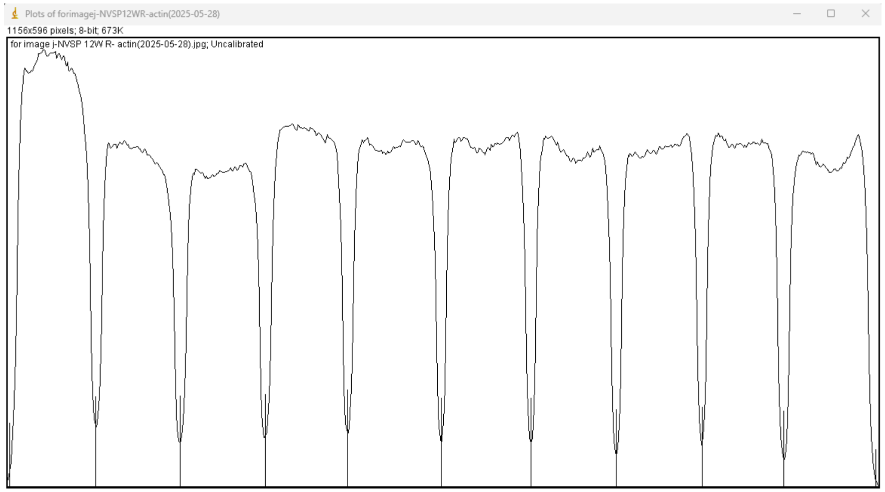

Date: 30 May 2025 LMW

Western Blot Imaging Form

Plot 3 Values-MPZ

	Area
1	55334
2	57474.45
3	50741.74
4	47660
5	69194.81
6	57125.88
7	51810.59
8	45093.69
9	33254.21
10	37609.42

Plot 3-MPZ

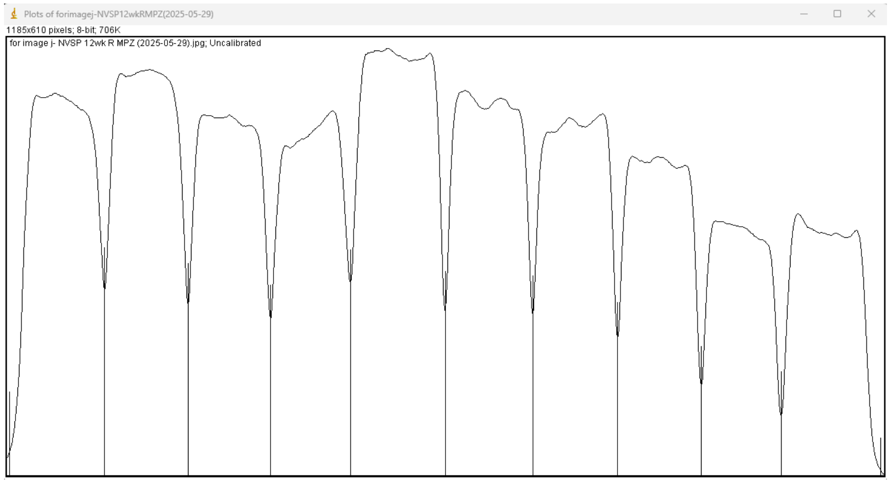

Date: 30 May 2025 LMW

Operator Information

**Performed By:**	**Name:** Merlin P Thangaraj	**Signature:** [signature]
	**Position/Lab:** Postdoctoral Scientist/Harper Lab	**Date:** 8-1-2025

**Performed By:**	**Name:** Lindsay Wallace	**Signature:** [signature]
	**Position/Lab:** Senior Research Scientist/Harper Lab	**Date:** 30 May 2025

Western Blot Imaging Form

Study	ARM101-CMT1A-NHP-001
Timepoint	6 week
Tissue	Ulnar nerve
Anatomical Location (Highlight/Circle one)	Distal (circled) Proximal N/A Other:

Stain Free Gel

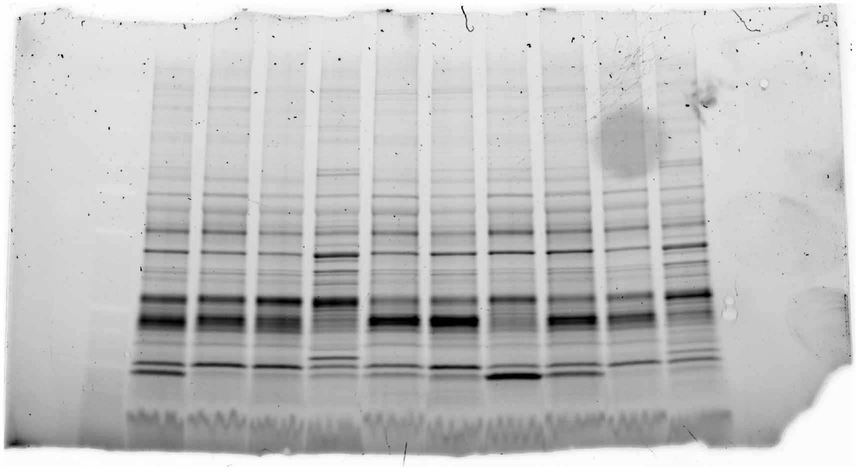

Date: 02 Apr 2024 MPT

Stain Free Membrane

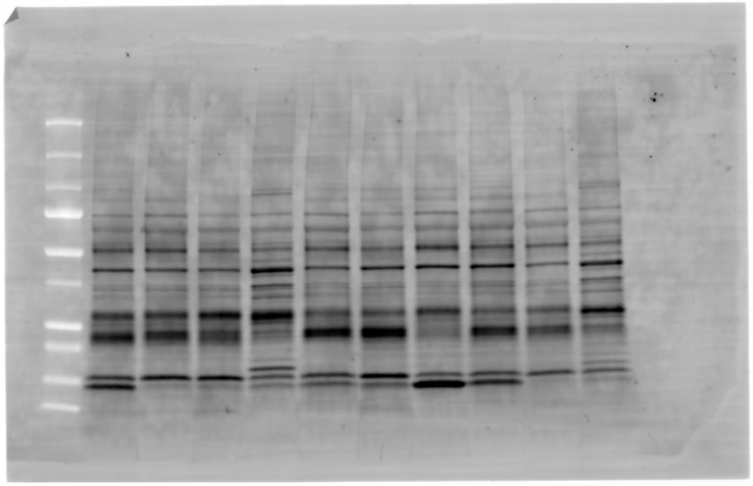

Date: 02 Apr 2024 MPT

1° PMP22

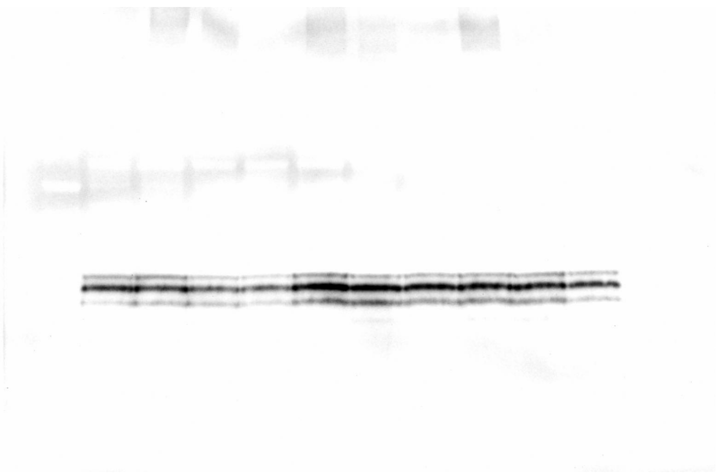

Date: 03 Apr 2024 MPT

1° β-Actin

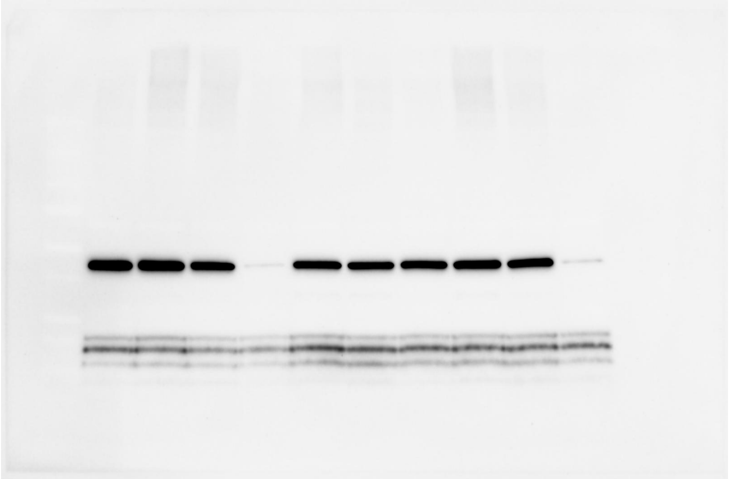

Date: 03 Apr 2024 MPT

Western Blot Imaging Form

Strip Check

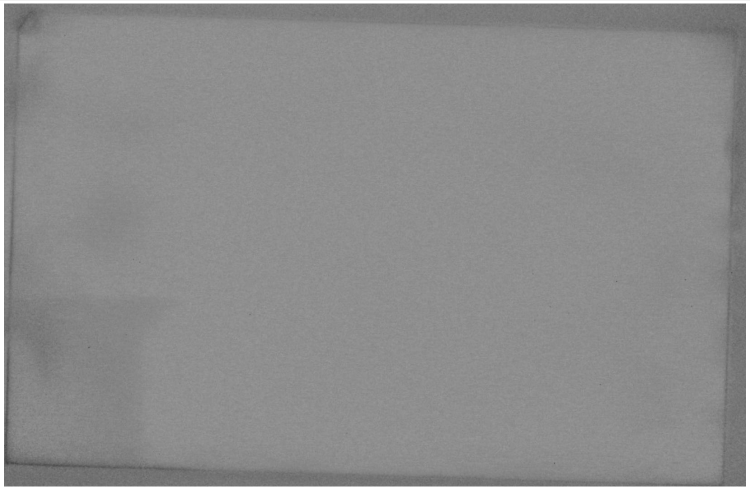

Date: 10 Apr 2024 MPT

1° MPZ

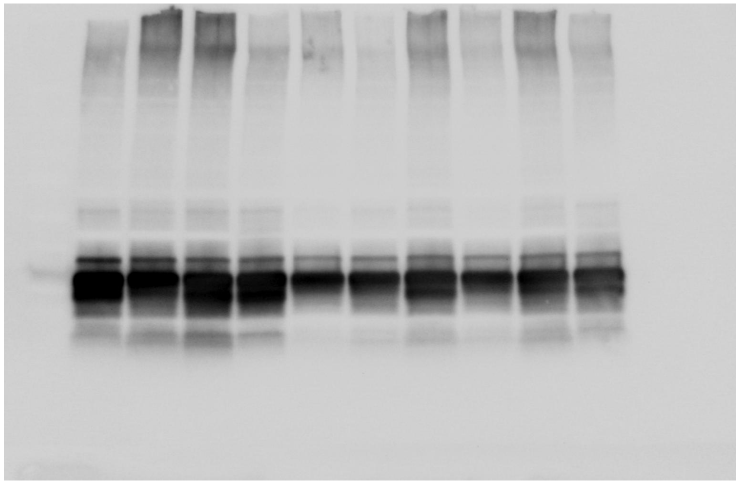

Date: 11 Apr 2024 MPT

ImageJ Quantification Box – PMP22

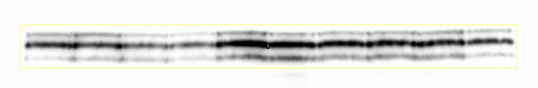

Date: 29 Apr 2024 LMW

ImageJ Quantification Box – B-Actin

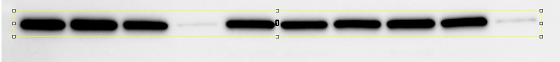

Date: 23 Apr 2024 LMW

ImageJ Quantification Box - MPZ

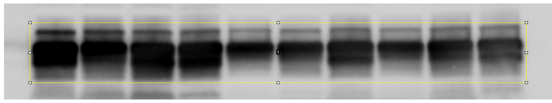

Date: 29 Apr 2024 LMW

Plot 1 Values PMP22

	Area
1	29624.2
2	37412.97
3	21137.95
4	16367.37
5	61651.75
6	52130.32
7	40986.08
8	39896.66
9	48416
10	23055.32

Plot 1-PMP22

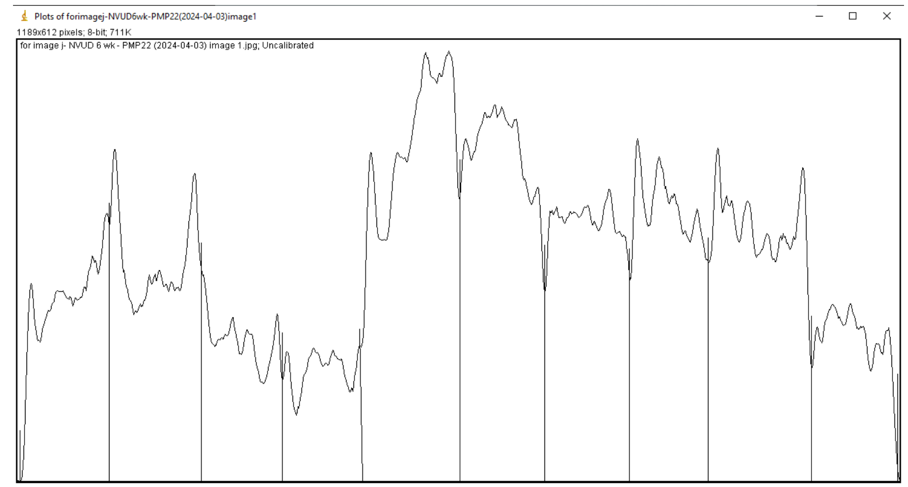

Date: 29 Apr 2024 LMW

Plot 2 Values B-Actin

	Area
1	57110.76
2	58715.88
3	46100.42
4	2393.205
5	45008.71
6	42258.18
7	43930.23
8	47847.69
9	45019.59
10	2030.154

Plot 2-B-Actin

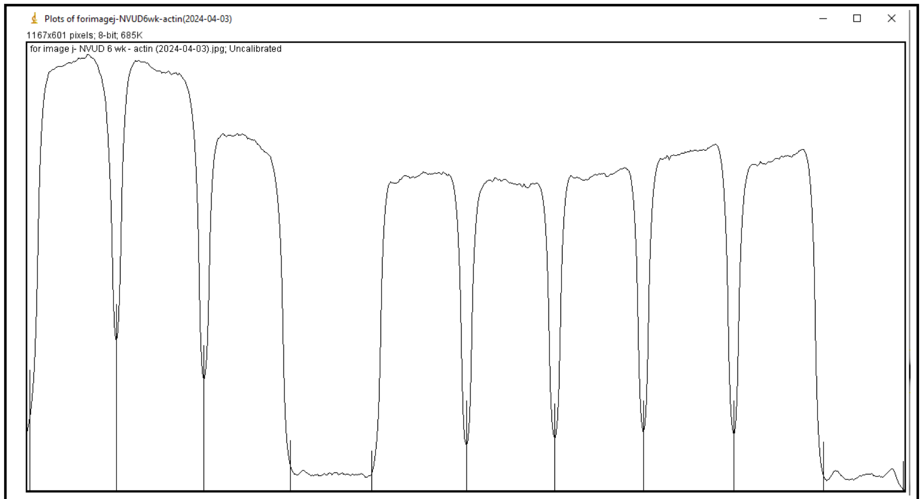

Date: 23 Apr 2024 LMW

Western Blot Imaging Form

Plot 3 Values-MPZ

	Area
1	59387.71
2	55945.52
3	57998.42
4	53728.71
5	36359.54
6	36622.13
7	45713.88
8	34213.93
9	42873.49
10	37885.42

Plot 3-MPZ

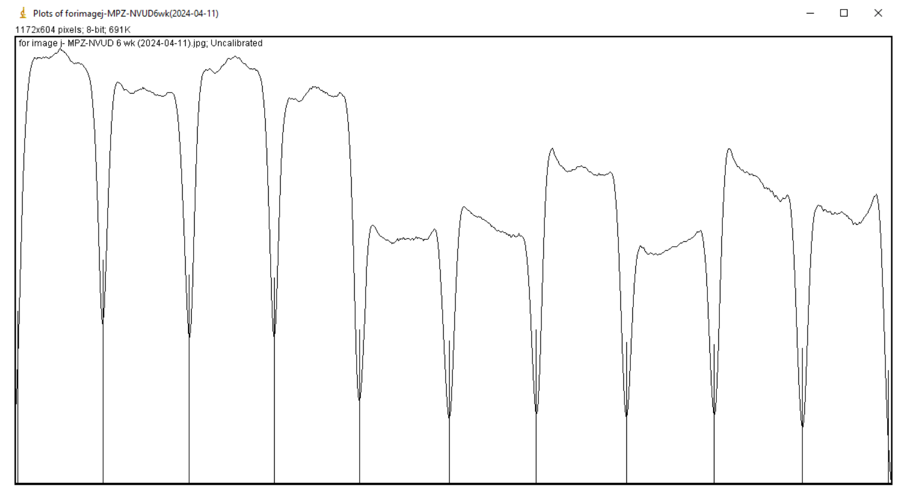

Date: 29 Apr 2024 LMW

Operator Information

**Performed By:**	**Name:** Merlin P Thangaraj	**Signature:**
	**Position/Lab:** Postdoctoral Scientist / Harper Lab	**Date:** 27 Jun 2024

**Performed By:**	**Name:** Lindsay Wallace	**Signature:**
	**Position/Lab:** Sr Research Scientist/Harper Lab	**Date:** 29 Apr 2024

Western Blot Imaging Form

**Study**	ARM101-CMT1A-NHP-001
**Timepoint**	12 week
**Tissue**	Ulnar Nerve
**Anatomical Location** (Highlight/Circle one)	(Distal) Proximal N/A Other:

Stain Free Gel

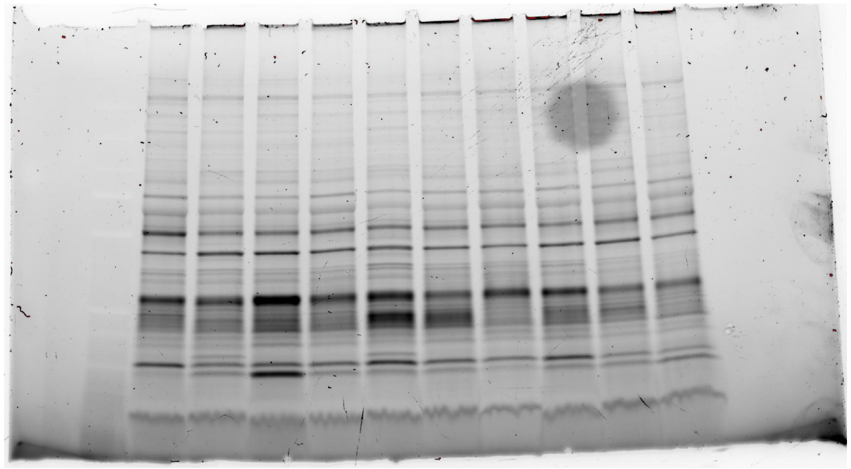

Date: 13 Mar 2024 MPT

Stain Free Membrane

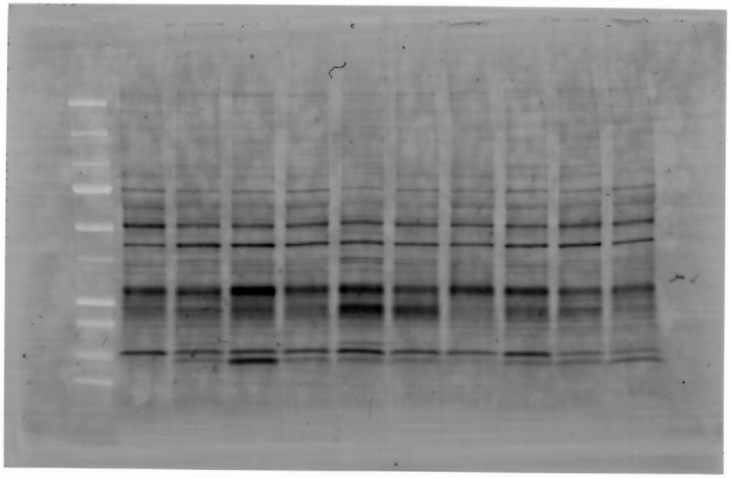

Date: 13 Mar 2024 MPT

1° PMP22

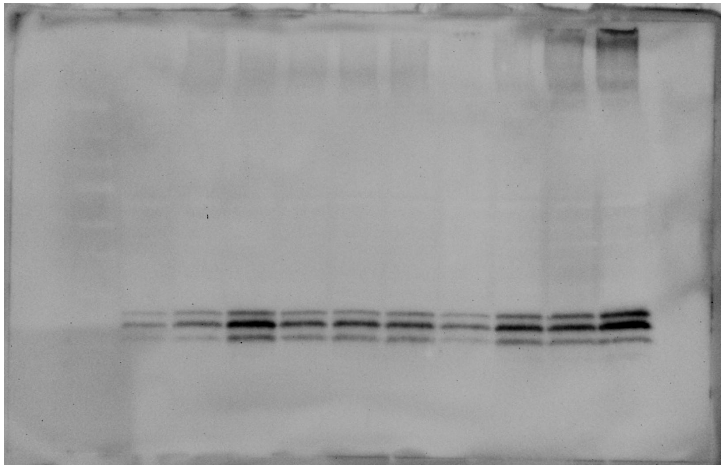

Date: 14 Mar 2024 MPT

1° β-Actin

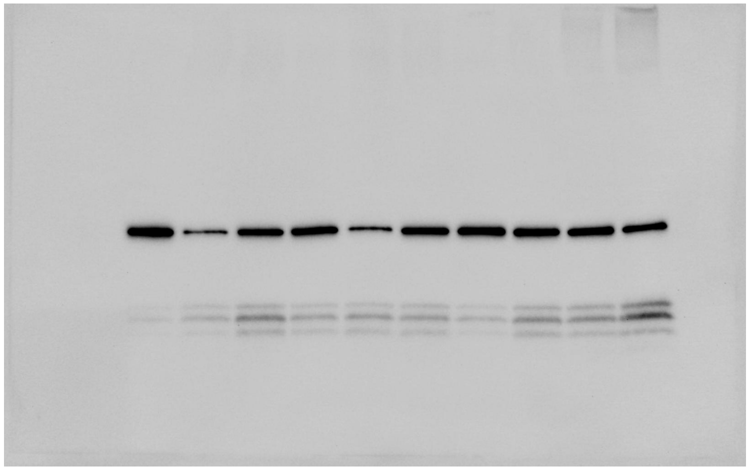

Date: 14 Mar 2024 MPT

Western Blot Imaging Form

Strip Check

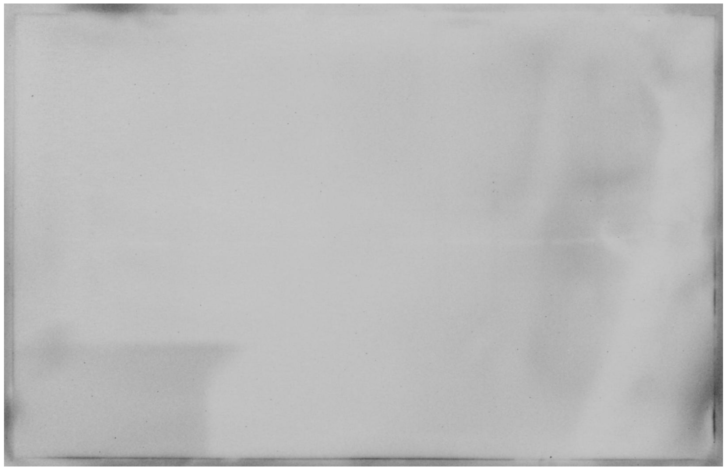

Date: 19 Mar 2024 MPT

1° MPZ

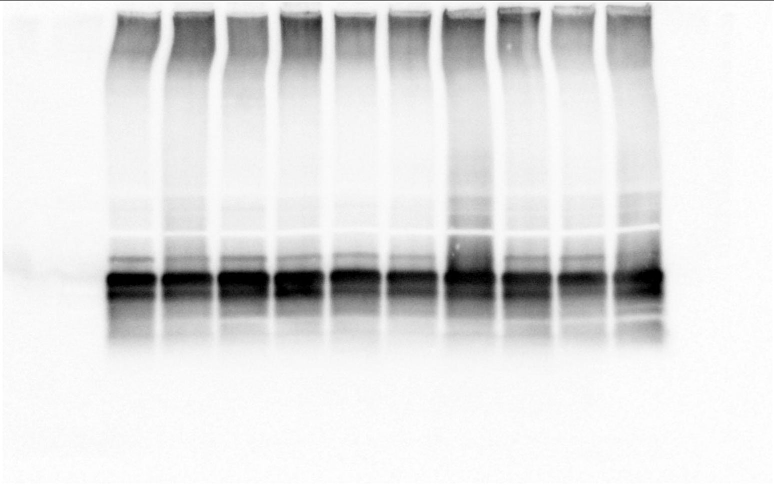

Date: 19 Mar 2024 MPT

ImageJ Quantification Box – PMP22

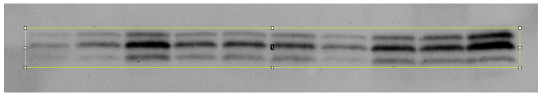

Date: 28 Mar 2024 LMW

ImageJ Quantification Box – B-Actin

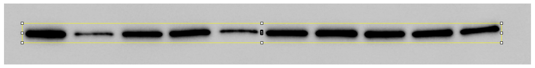

Date: 28 Mar 2024 LMW

ImageJ Quantification Box - MPZ

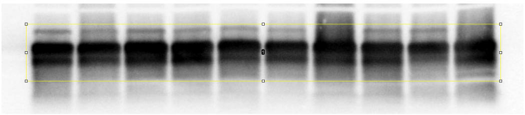

Date: 28 Mar 2024 LMW

Plot 1 Values PMP22

	Area
1	15512.9
2	19604.85
3	54915.8
4	28961.02
5	32187.44
6	33314.8
7	14223.44
8	41290.97
9	42242.73
10	65200.63

Plot 1-PMP22

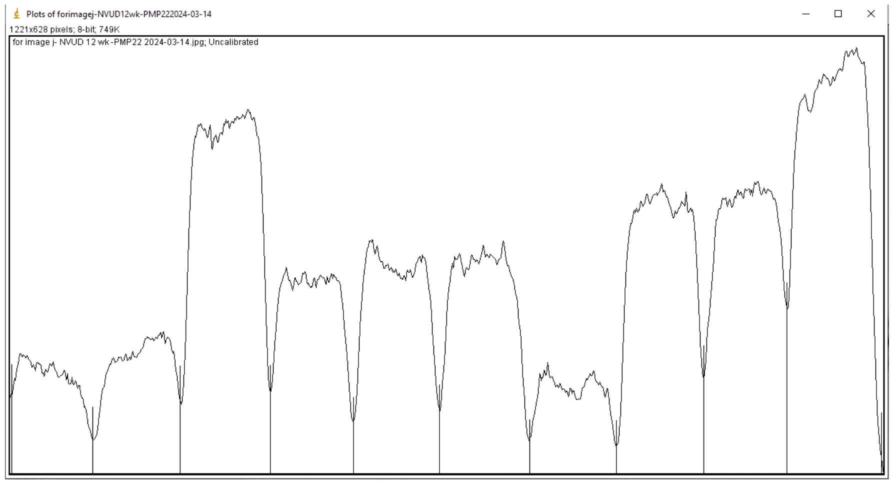

Date: 28 Mar 2024 LMW

Plot 2 Values B-Actin

	Area
1	55608.61
2	18824.32
3	44112.85
4	47958.44
5	20786.61
6	48133.27
7	52813.73
8	48548.44
9	47849.02
10	38590.66

Plot 2-B-Actin

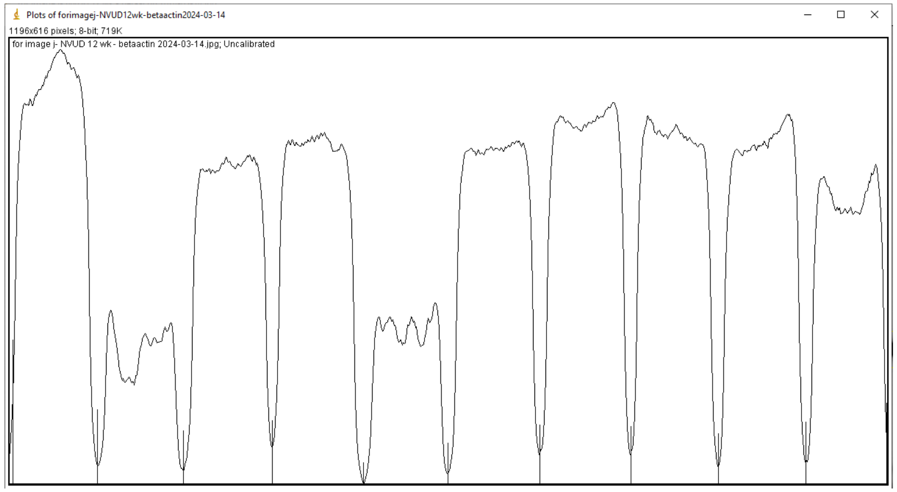

Date: 28 Mar 2024 LMW

Western Blot Imaging Form

Plot 3 Values-MPZ

	Area
1	51124.61
2	49782.15
3	54751.83
4	52203.71
5	48456.78
6	45678.2
7	62345.15
8	50922.73
9	40202.08
10	53377.44

Plot 3-MPZ

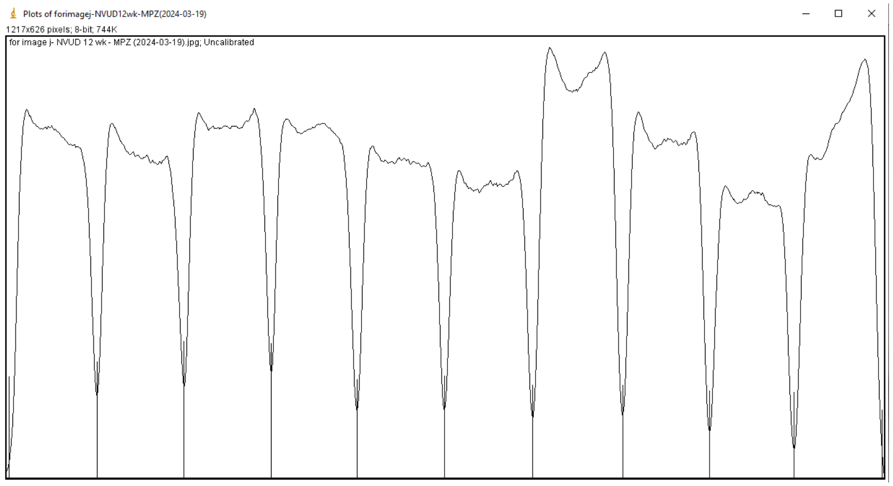

Date: 28 Mar 2024 LMW

Operator Information

**Performed By:**	**Name:** Merlin P Thangaraj	**Signature:** [signature]
	**Position/Lab:** Postdoctoral Scientist / Harper Lab	**Date:** 27 Jun 2024

**Performed By:**	**Name:** Lindsay Wallace	**Signature:** [signature]
	**Position/Lab:** Sr Research Scientist / Harper Lab	**Date:** 28 Mar 2024 LMW

Western Blot Imaging Form

**Study**	ARM101-CMT1A-NHP-001
**Timepoint**	6 week
**Tissue**	Ulnar nerve- right
**Anatomical Location** (Highlight/Circle one)	Distal (circled) Proximal N/A<br>Other:

Stain Free Gel

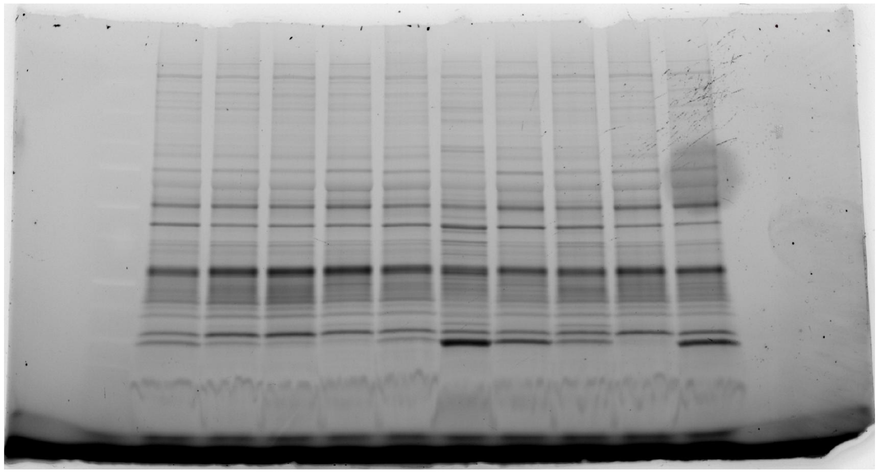

Date: 09 Oct 2024 MPT

Stain Free Membrane

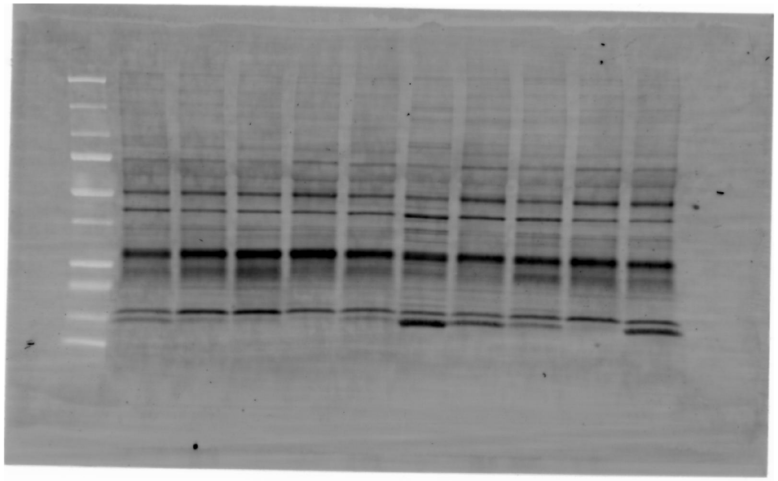

Date: 09 Oct 2024 MPT

1° PMP22

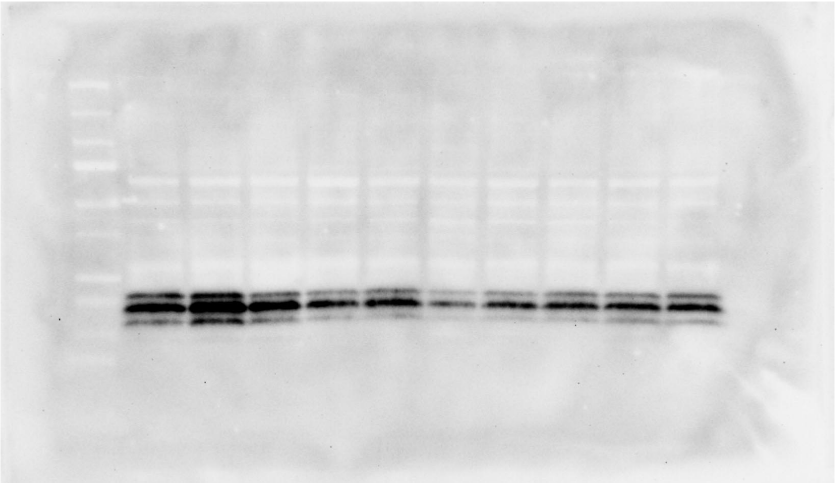

Date: 10 Oct 2024 MPT

1° β-Actin

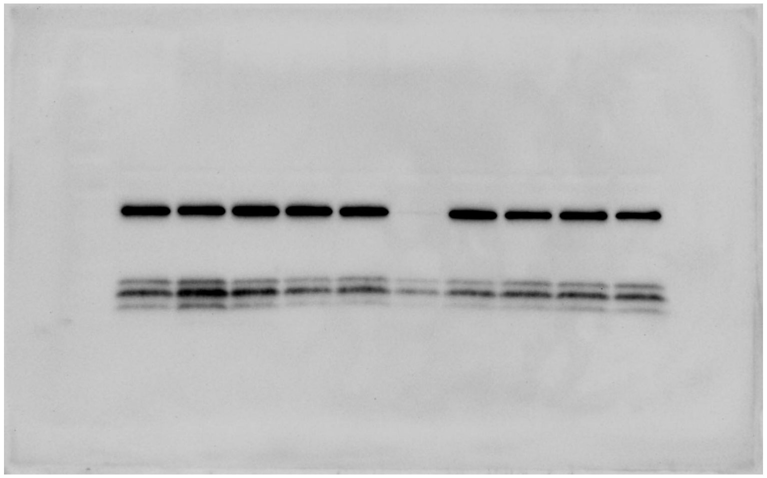

Date: 10 Oct 2024 MPT

Western Blot Imaging Form

Strip Check

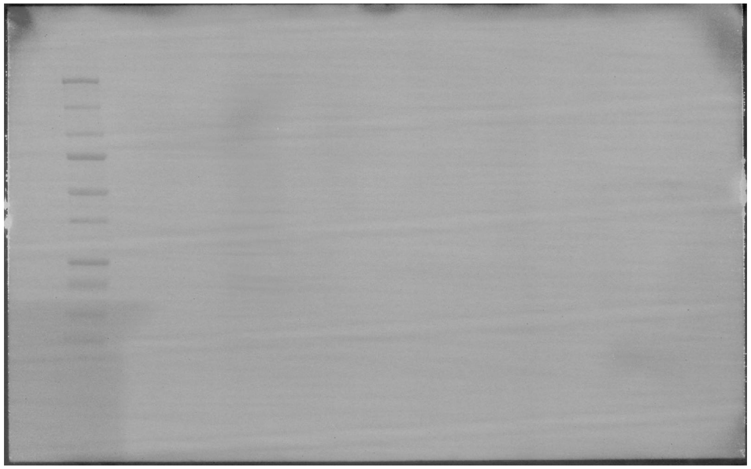

Date: 11 Oct 2024 MPT

1° MPZ

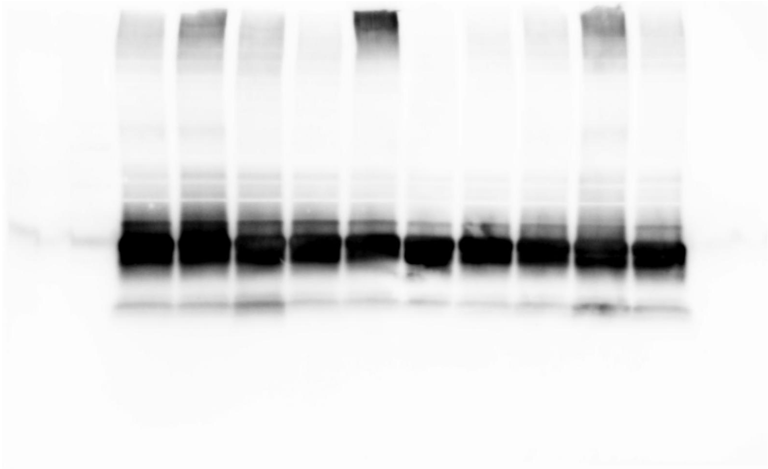

Date: 11 Oct 2024 MPT

ImageJ Quantification Box – PMP22

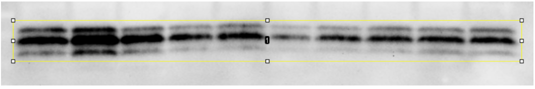

Date: 07 Jan 2025 LMW

ImageJ Quantification Box – B-Actin

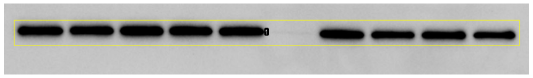

Date: 07 Jan 2025 LMW

ImageJ Quantification Box - MPZ

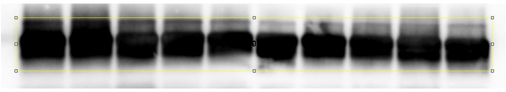

Date: 08 Jan 2025 LMW

Plot 1-PMP22

Plot 1 Values PMP22

	Area
1	40164.9
2	49721.25
3	30355
4	20508.59
5	27962.9
6	10611.23
7	18017.83
8	21818.66
9	24672.13
10	24516.49

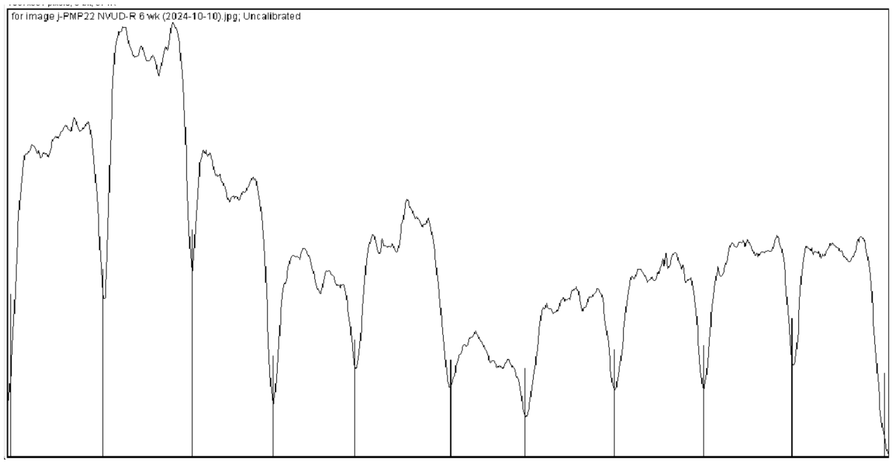

Date: 07 Jan 2025 LMW

Plot 2-B-Actin

Plot 2 Values B-Actin

	Area
1	45428.88
2	43758.13
3	48037.3
4	45515
5	45109
6	1655.376
7	44635.83
8	36787.42
9	39826.59
10	32122.59

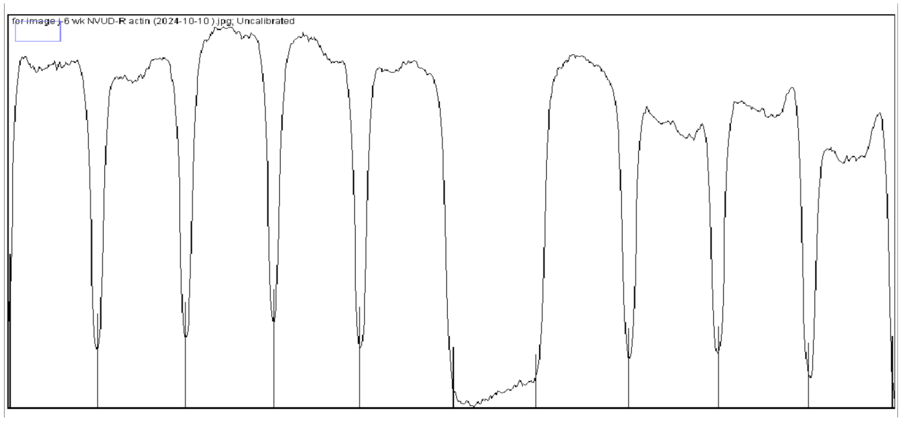

Date: 08 Jan 2025 LMW

Plot 3 Values-MPZ

	Area
1	63374.71
2	60827.69
3	52500.69
4	50852.74
5	46047.88
6	42892.47
7	47940.37
8	46252.42
9	50365.78
10	46303.76

Plot 3-MPZ

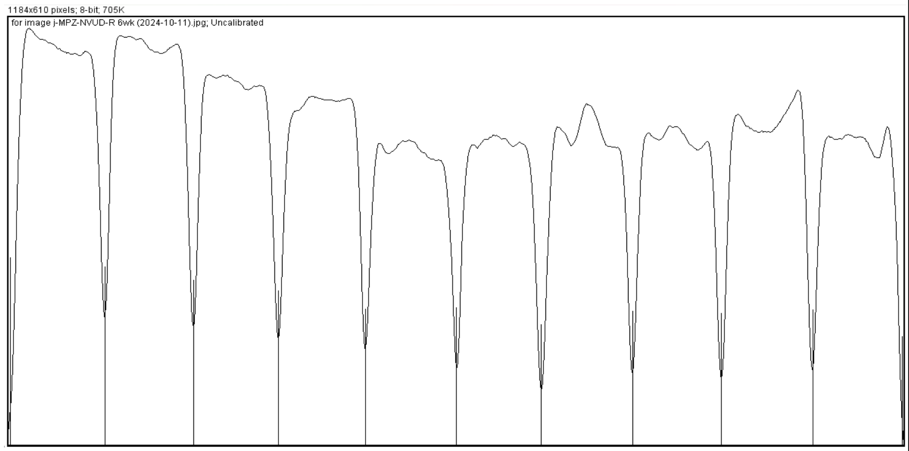

Date: 07 Jan 2025 LMW

Operator Information

Performed By:	**Name:** Merlin P Thangaraj	**Signature:**
	**Position/Lab:** Postdoctoral Scientist / Harper Lab	**Date:** 08 Jan 2025

Performed By:	**Name:** Lindsay Wallace	**Signature:**
	**Position/Lab:** Sr Research Scientist/Harper Lab	**Date:** 08 Jan 2025

Western Blot Imaging Form

**Study**	ARM101-CMT1A-NHP-001
**Timepoint**	12 week
**Tissue**	Ulnar Nerve - Right
**Anatomical Location** (Highlight/Circle one)	Distal (circled) Proximal N/A Other:

Stain Free Gel

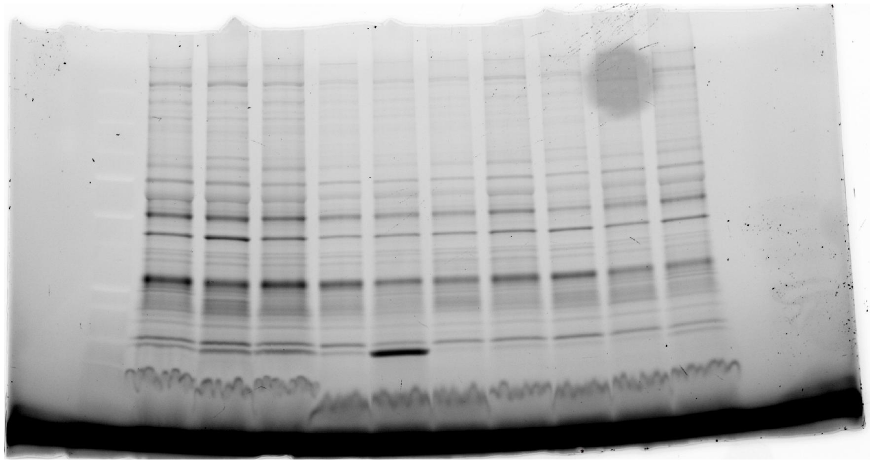

Date: 17 Sep 2024 MPT

Stain Free Membrane

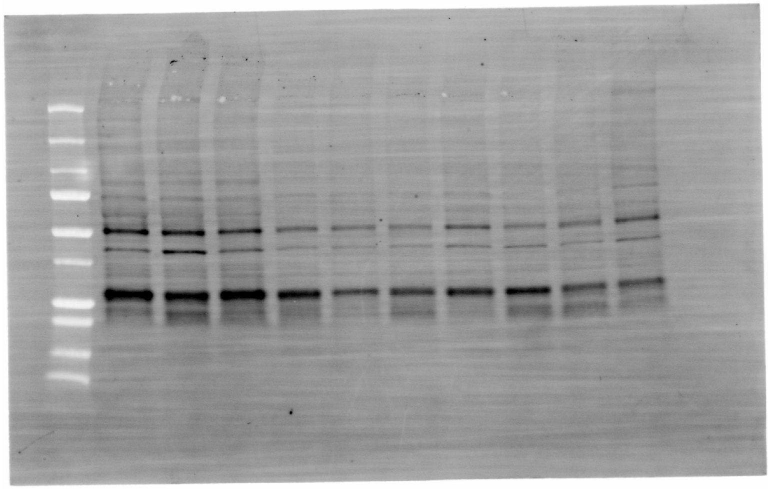

Date: 17 Sep 2024 MPT

1° PMP22

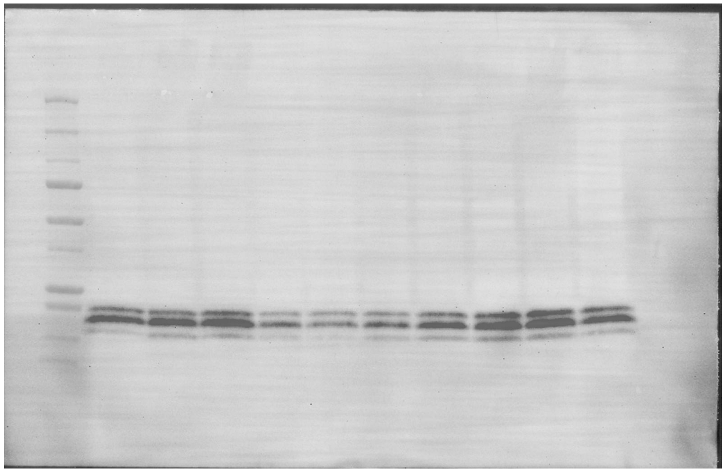

Date: 18 Sep 2024 MPT

1° β-Actin

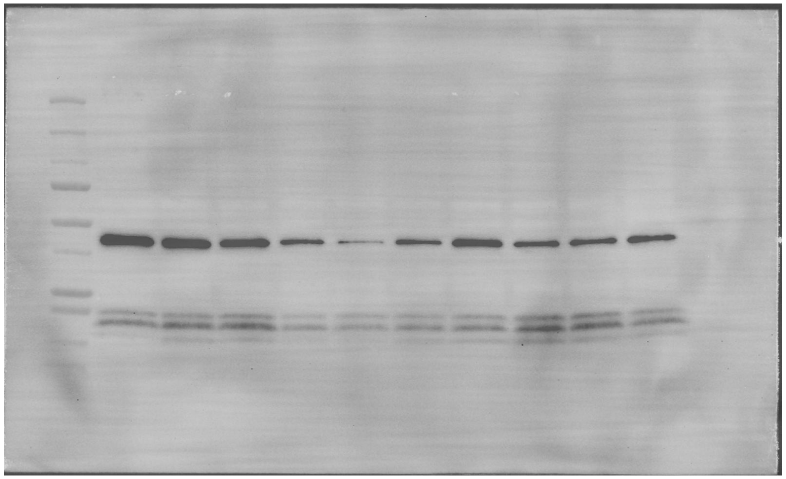

Date: 18 Sep 2024 MPT

Western Blot Imaging Form

Strip Check

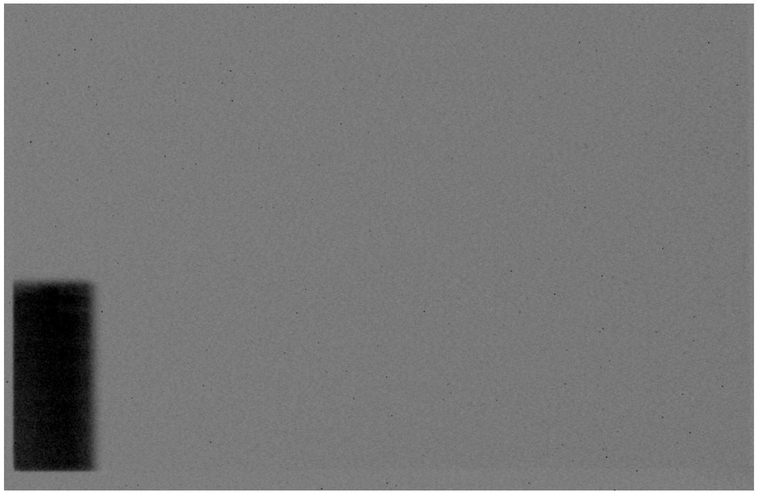

Date: 23 Sep 2024 MPT

1° MPZ

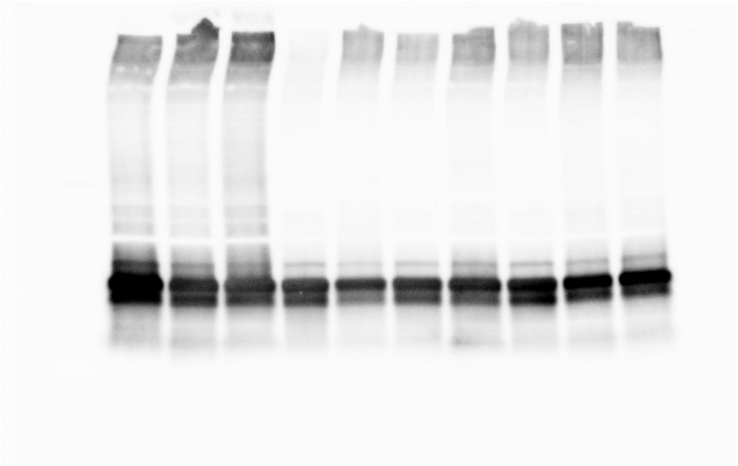

Date: 24 Sep 2024 MPT

ImageJ Quantification Box – PMP22

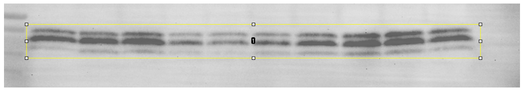

Date: 25 Sep 2024 LMW

ImageJ Quantification Box – B-Actin

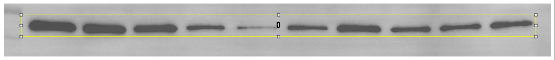

Date: 25 Sep 2024 LMW

ImageJ Quantification Box - MPZ

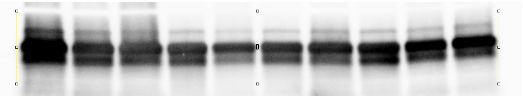

Date: 25 Sep 2024 LMW

Western Blot Imaging Form

Plot 1 Values PMP22

	Area
1	51480.58
2	46771.15
3	53368.63
4	18394.54
5	17717.68
6	28507.85
7	41159.22
8	54078.15
9	55001.85
10	43337.7

Plot 1-PMP22

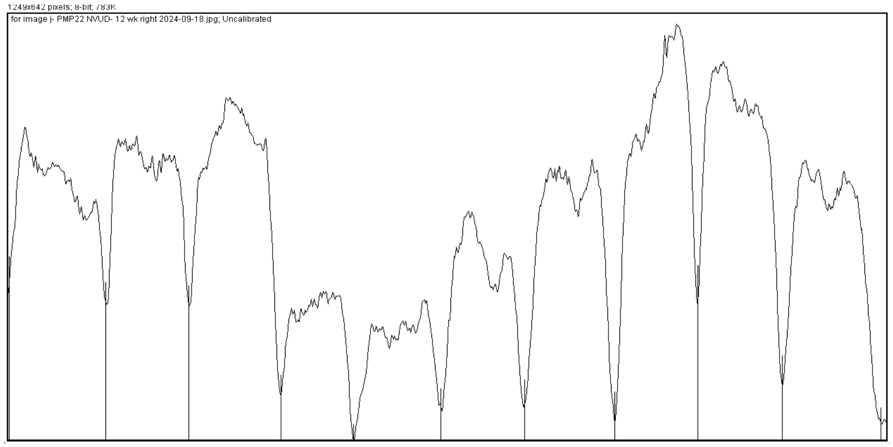

Date: 25 Sep 2024 LMW

Plot 2 Values B-Actin

	Area
1	63517.34
2	55903.02
3	48671.97
4	27305.49
5	15619.27
6	31109.56
7	44394.39
8	33232.02
9	33364.73
10	30669.9

Plot 2-B-Actin

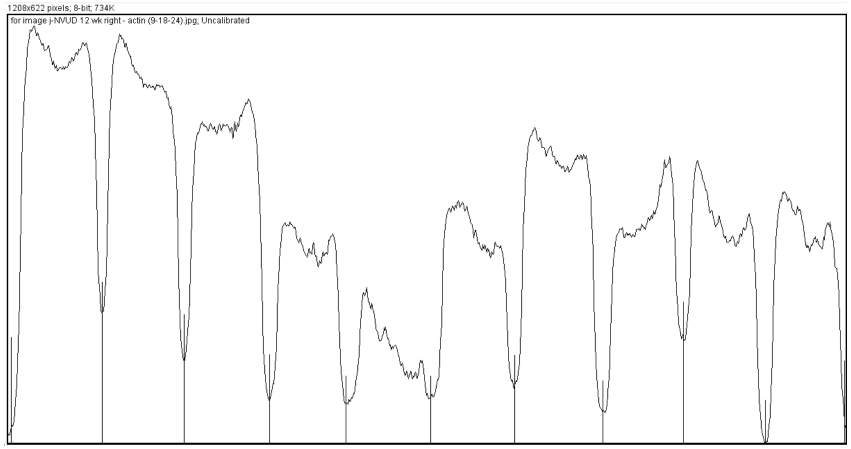

Date: 25 Sep 2024 LMW

Plot 3 Values-MPZ

	Area
1	60098.1
2	38183.32
3	38017.61
4	27568.42
5	22706.88
6	26400.42
7	33833.02
8	33948.37
9	33016.71
10	36998.37

Plot 3-MPZ

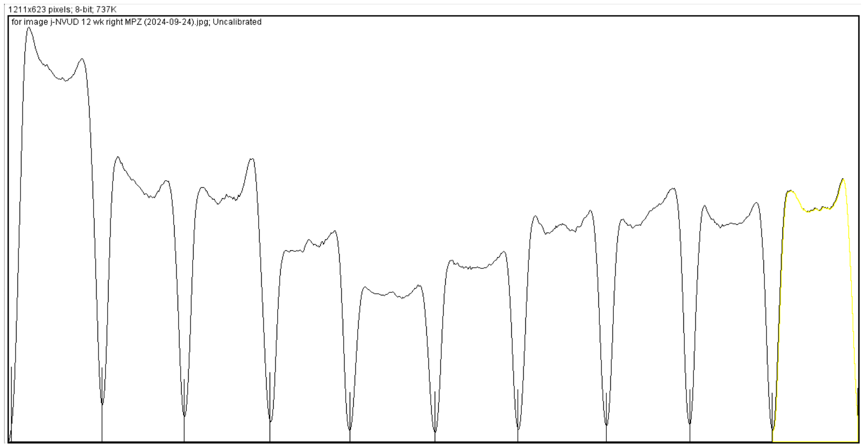

Date: 25 Sep 2024 LMW

Operator Information

**Performed By:**	Name: Merlin P Thangaraj	Signature: [signature]
	Position/Lab: Postdoctoral Scientist / Harper Lab	Date: 8-1-2025

**Performed By:**	Name: Lindsay Wallace	Signature: [signature]
	Position/Lab: Sr Research Scientist / Harper Lab	Date: 29 Oct 2024

Western Blot Imaging Form

**Study**	ARM101-CMT1A-NHP-001
**Timepoint**	6 week
**Tissue**	Ulnar nerve
**Anatomical Location** (Highlight/Circle one)	Distal / Proximal (circled) / N/A<br>Other:

Stain Free Gel

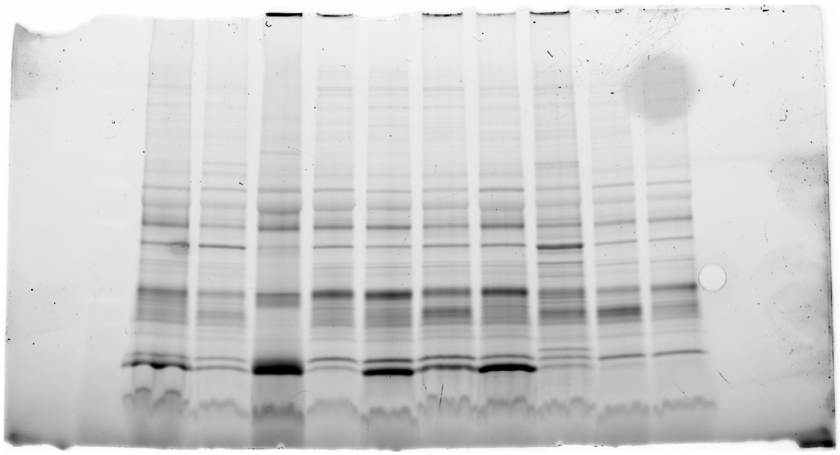

Date: 02 Apr 2024 MPT

Stain Free Membrane

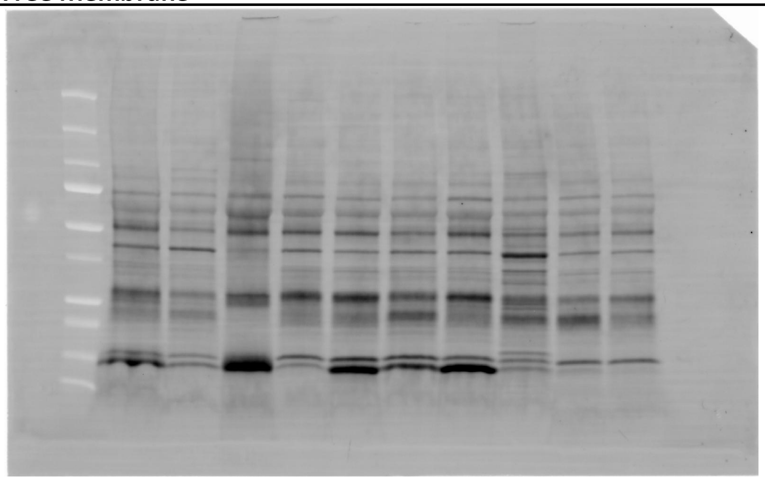

Date: 02 Apr 2024 MPT

1° PMP22

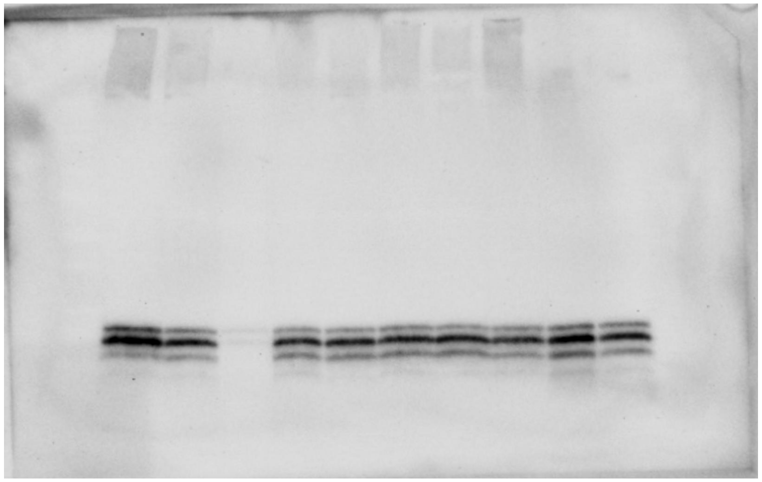

Date: 03 Apr 2024 MPT

1° β-Actin

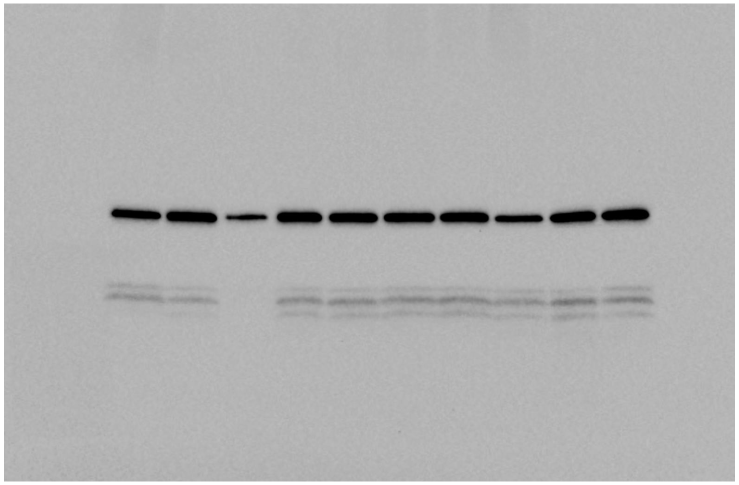

Date: 03 Apr 2024 MPT

Western Blot Imaging Form

Strip Check

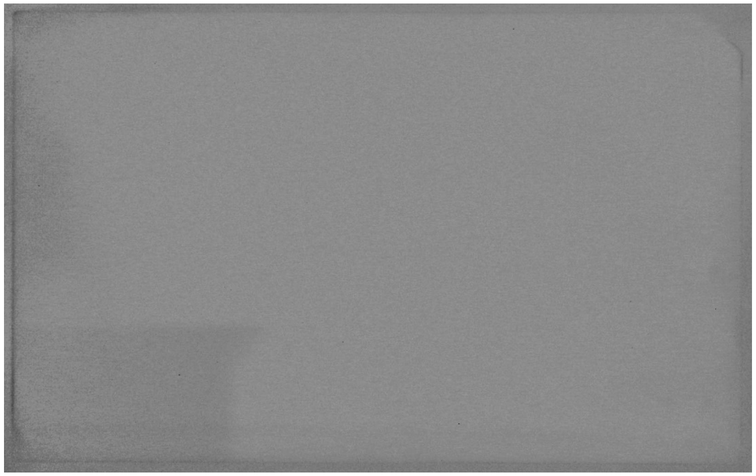

Date: 10 Apr 2024 MPT

1° MPZ

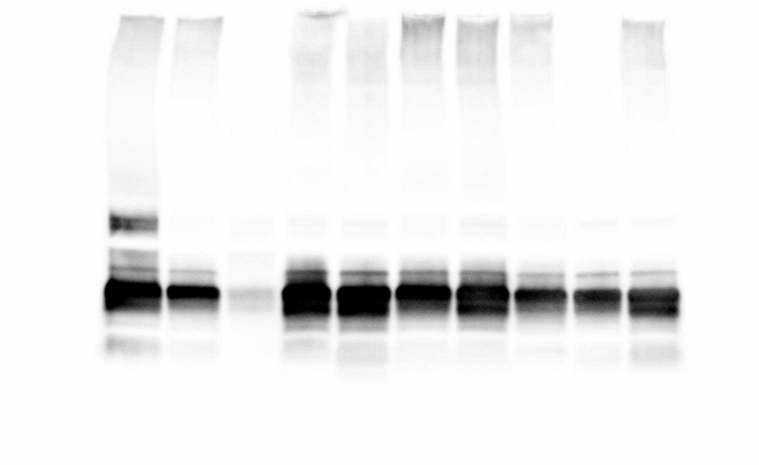

Date: 11 Apr 2024 MPT

ImageJ Quantification Box – PMP22

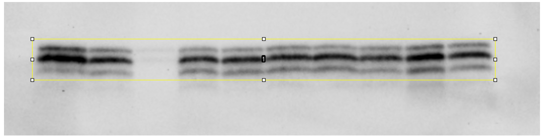

Date: 29 Apr 2024 LMW

ImageJ Quantification Box – B-Actin

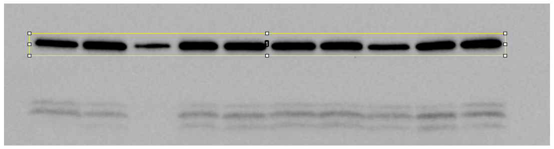

Date: 29 Apr 2024 LMW

ImageJ Quantification Box - MPZ

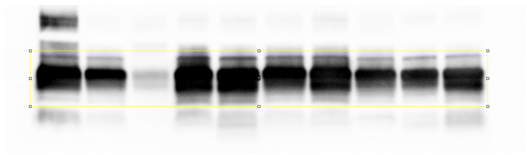

Date: 29 Apr 2024 LMW

Western Blot Imaging Form

Plot 1 Values PMP22

	Area
1	72310.7
2	41548.27
3	644.205
4	38523.44
5	43019.32
6	50080.15
7	50511.73
8	44656.2
9	57774.37
10	44048.85

Plot 1-PMP22

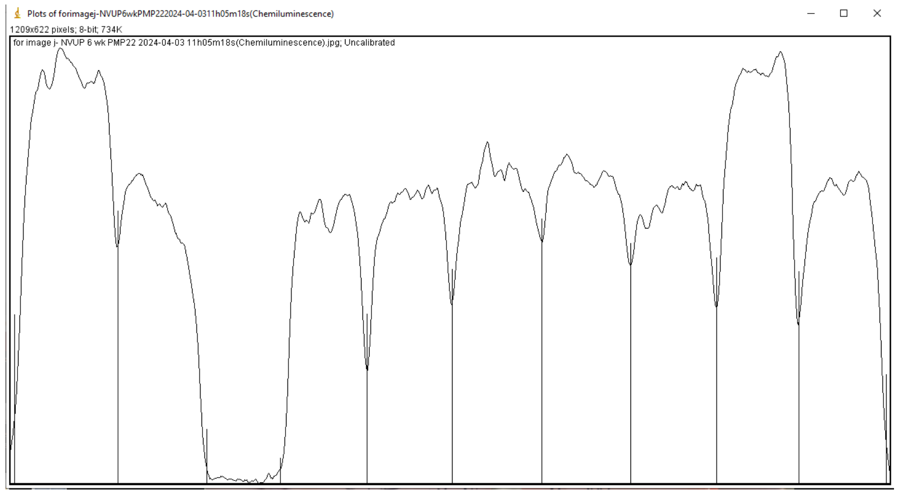

Date: 29 Apr 2024 LMW

Plot 2 Values B-Actin

	Area
1	46347.9
2	55661.73
3	23332.95
4	50347.73
5	52176.32
6	53997.32
7	52462.02
8	36700.9
9	50372.08
10	54158.32

Plot 2-B-Actin

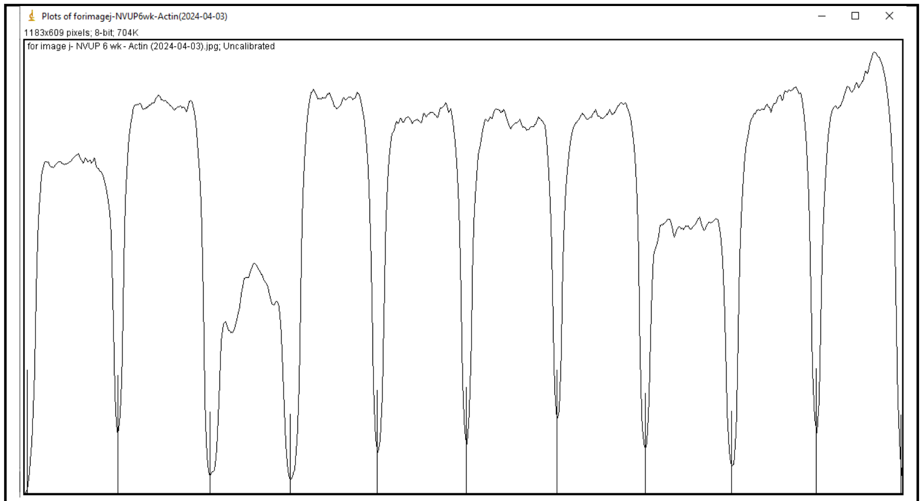

Date: 29 Apr 2024 LMW

Plot 3 Values-MPZ

	Area
1	52724.27
2	33350.42
3	6336.841
4	56036.83
5	56619.95
6	48681.71
7	52606.49
8	35208.71
9	28889.54
10	37771.13

Plot 3-MPZ

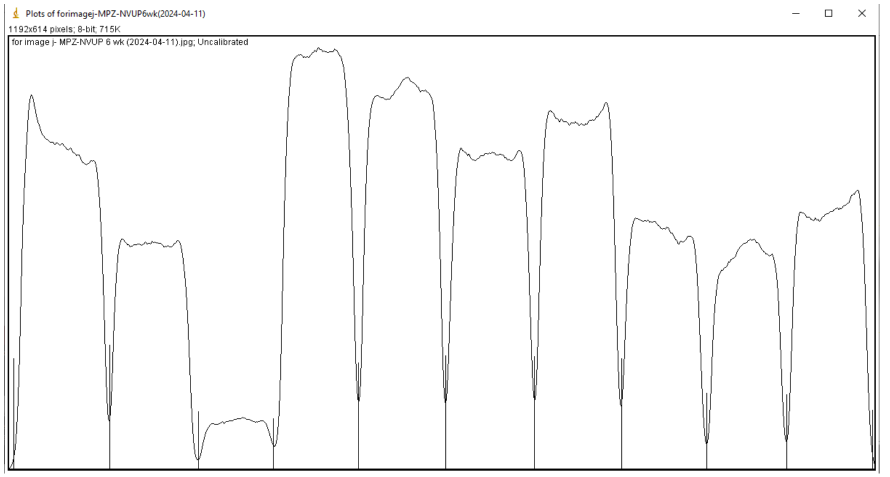

Date: 29 Apr 2024 LMW

Operator Information

**Performed By:**	**Name:** Merlin P Thangaraj	**Signature:**
	**Position/Lab:** Postdoctoral Scientist / Harper Lab	**Date:** 27 Jun 2024

**Performed By:**	**Name:** Lindsay Wallace	**Signature:**
	**Position/Lab:** Sr Research Scientist/Harper Lab	**Date:** 29 Apr 2024

Western Blot Imaging Form

**Study**	ARM101-CMT1A-NHP-001
**Timepoint**	12 week
**Tissue**	Ulnar Nerve
**Anatomical Location** (Highlight/Circle one)	Distal Proximal (circled) N/A<br>Other:

Stain Free Gel

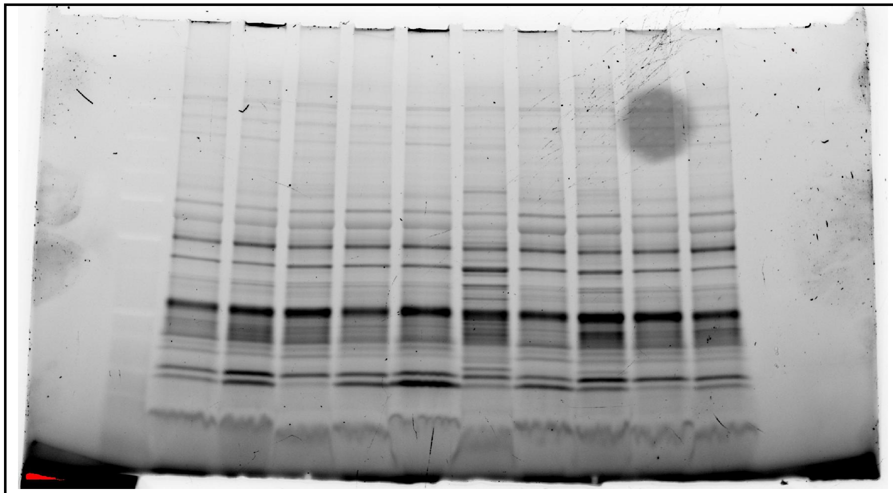

Date: 13 Mar 2024 MPT

Stain Free Membrane

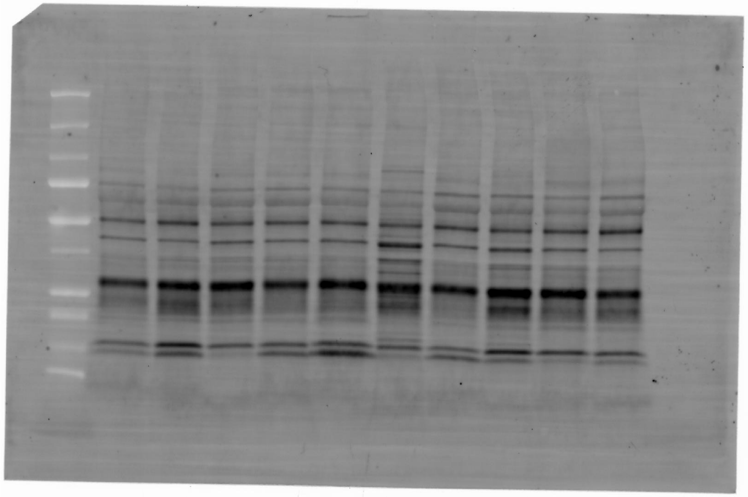

Date: 13 Mar 2024 MPT

1° PMP22

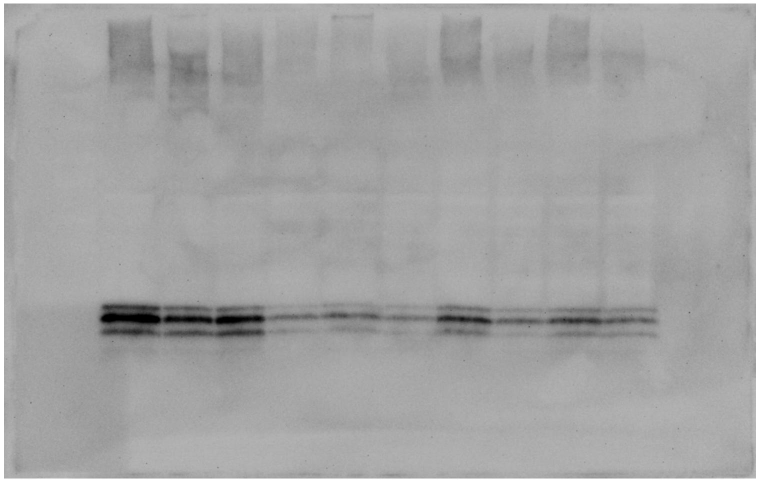

Date: 14 Mar 2024 MPT

1° β-Actin

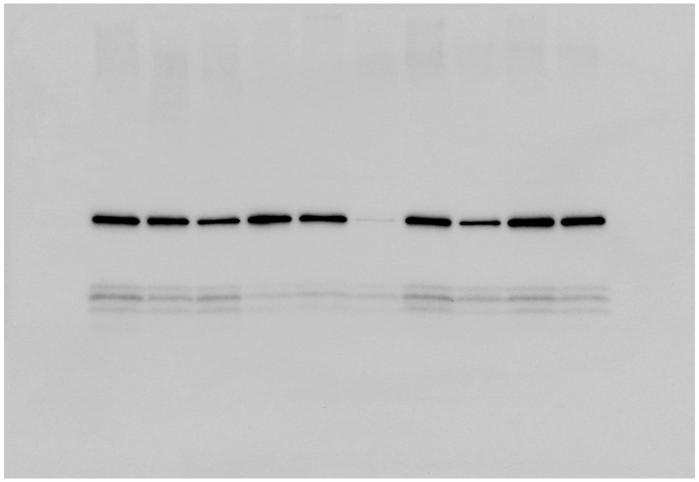

Date: 14 Mar 2024 MPT

Western Blot Imaging Form

Strip Check

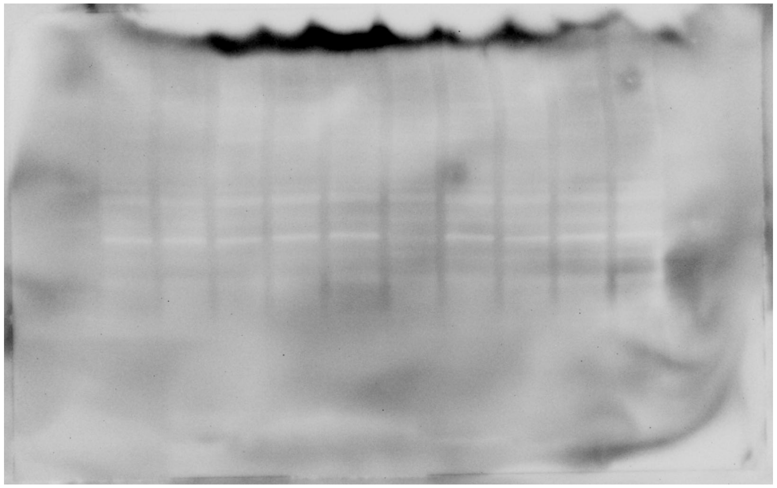

Date: 19 Mar 2024 MPT

1° MPZ

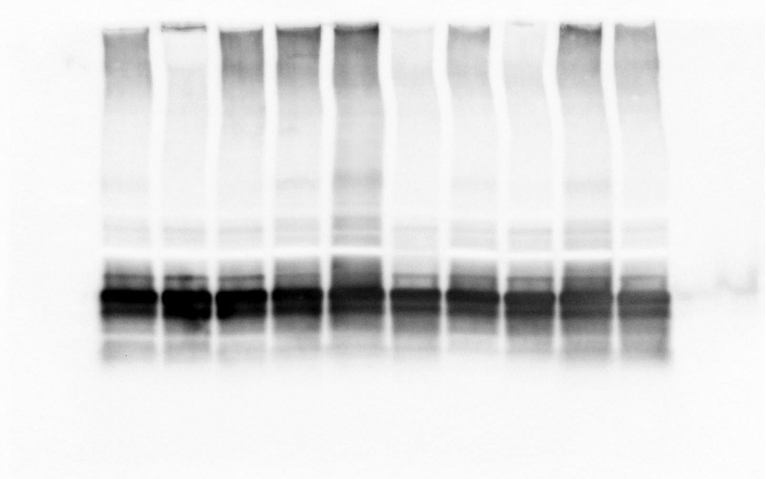

Date: 19 Mar 2024 MPT

ImageJ Quantification Box – PMP22

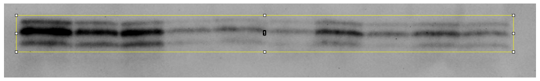

Date: 27 Mar 2024 LMW

ImageJ Quantification Box – B-Actin

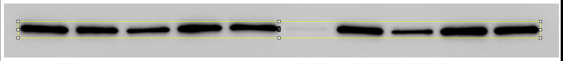

Date: 27 Mar 2024 LMW

ImageJ Quantification Box - MPZ

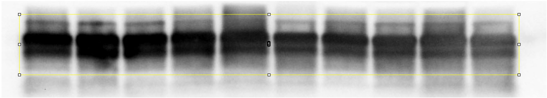

Date: 27 Mar 2024 LMW

Plot 1 Values PMP22

	Area
1	73480.75
2	44661.2
3	48884.85
4	12275.66
5	19969.41
6	10926.25
7	34069.34
8	12825.95
9	29684.29
10	16054.9

Plot 1-PMP22

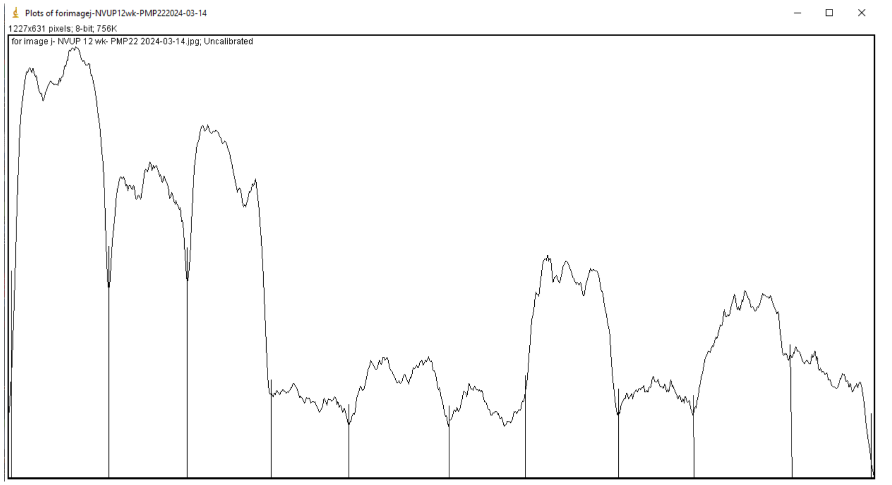

Date: 27 Mar 2024 LMW

Plot 2 Values B-Actin

	Area
1	57896.15
2	47870.32
3	38202.32
4	54296.56
5	49403.05
6	826.69
7	54950.68
8	29762.78
9	60570.68
10	54391.37

Plot 2-B-Actin

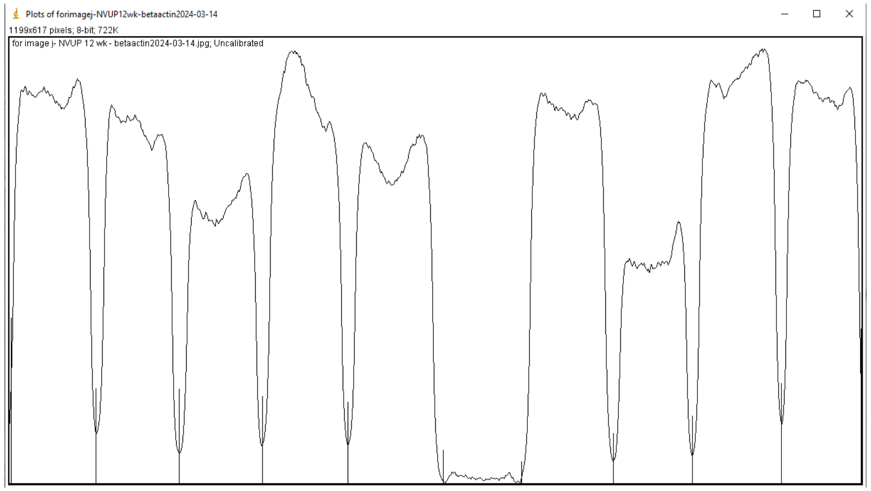

Date: 27 Mar 2024 LMW

Plot 3 Values-MPZ

	Area
1	59094.68
2	57805.49
3	54784.61
4	56389.95
5	62119.15
6	45562.42
7	49870.15
8	47419.95
9	51902.61
10	38285.95

Plot 3-MPZ

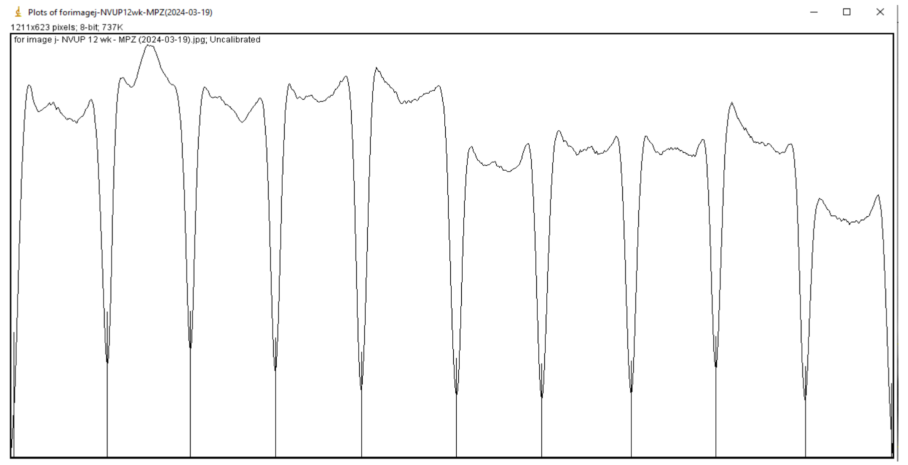

Date: 27 Mar 2024 LMW

Operator Information

**Performed By:**	**Name:** Merlin P Thangaraj	**Signature:**
	**Position/Lab:** Postdoctoral Scientist / Harper Lab	**Date:** 27 Jun 2024

**Performed By:**	**Name:** Lindsay Wallace	**Signature:**
	**Position/Lab:** Sr Research Scientist / Harper Lab	**Date:** 27 Mar 2024

Western Blot Imaging Form

**Study**	ARM101-CMT1A-NHP-001
**Timepoint**	6 week
**Tissue**	Ulnar nerve - Right
**Anatomical Location** (Highlight/Circle one)	Distal / Proximal (circled) / N/A<br>Other:

Stain Free Gel

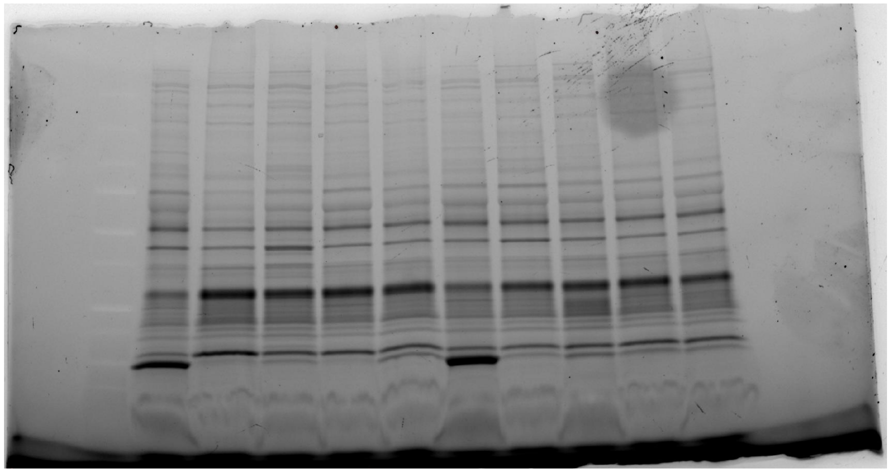

Date: 09 Oct 2024 MPT

Stain Free Membrane

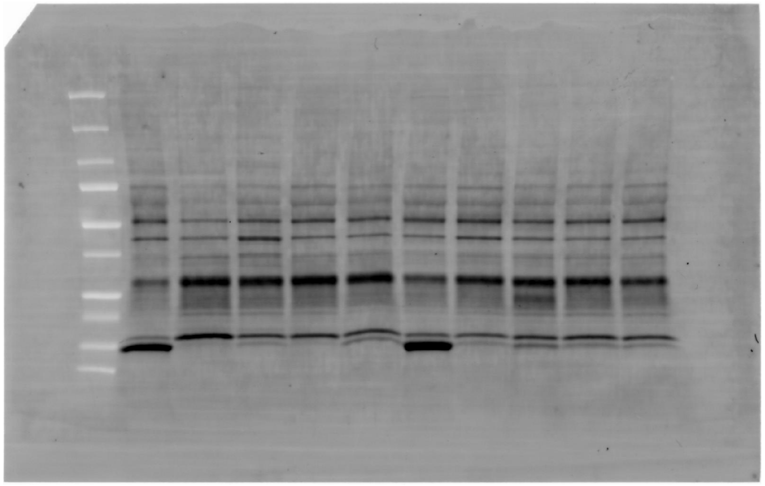

Date: 09 Oct 2024 MPT

1° PMP22

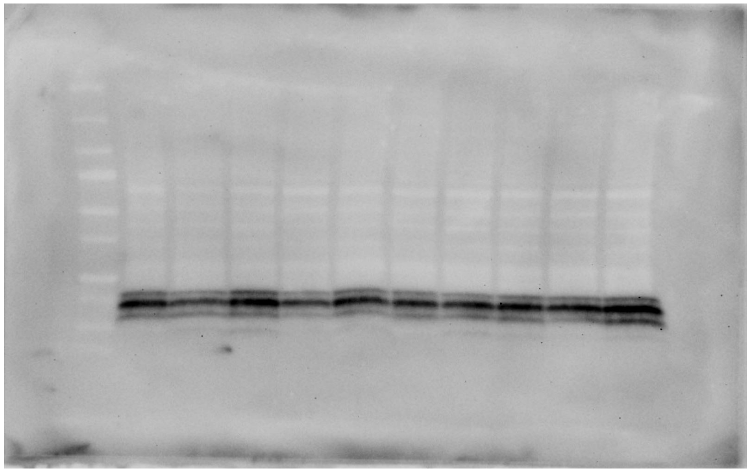

Date: 10 Oct 2024 MPT

1° β-Actin

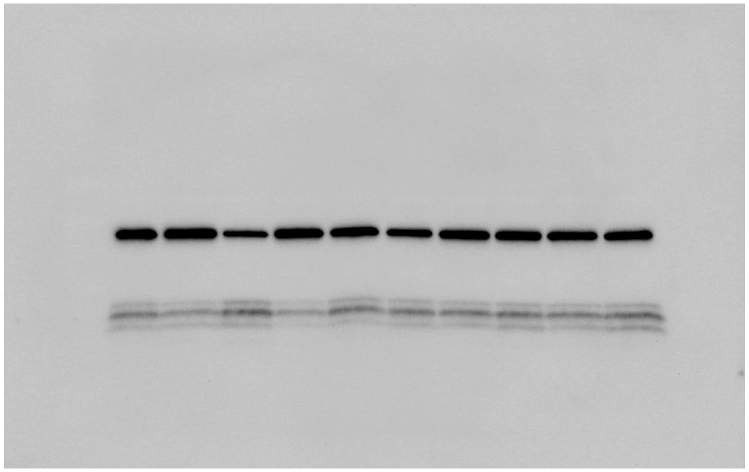

Date: 10 Oct 2024 MPT

Western Blot Imaging Form

Strip Check

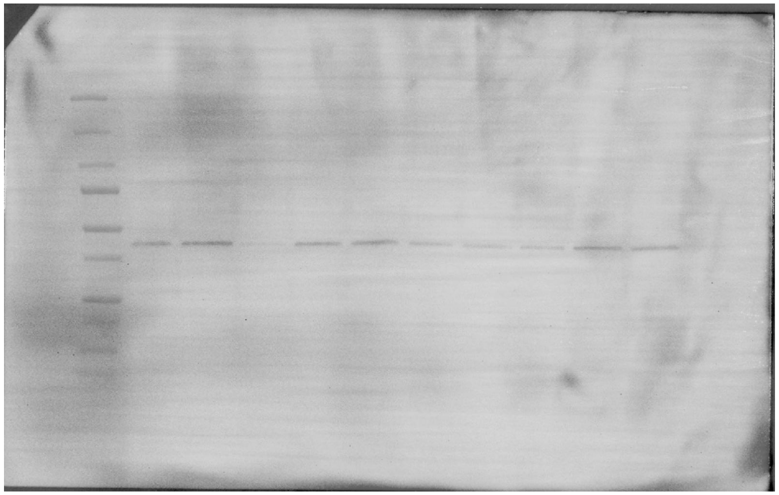

Date: 11 Oct 2024 MPT

1° MPZ

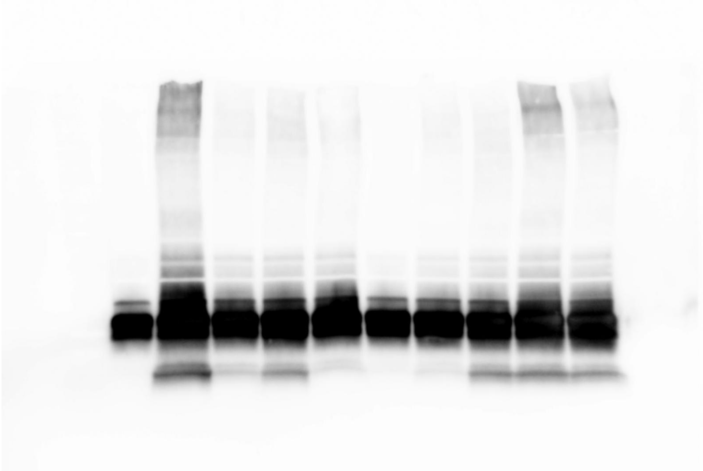

Date: 11 Oct 2024 MPT

ImageJ Quantification Box – PMP22

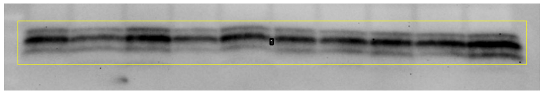

Date: 19 Dec 2024 LMW

ImageJ Quantification Box – B-Actin

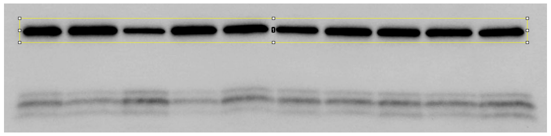

Date: 19 Dec 2024 LMW

ImageJ Quantification Box - MPZ

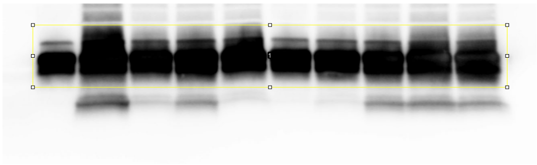

Date: 19 Dec 2024 LMW

Plot 1 Values PMP22

	Area
1	50889.27
2	37419.2
3	64464.22
4	29086.42
5	49360.92
6	37790.95
7	40951.15
8	45789.37
9	44969.85
10	72482

Plot 1-PMP22

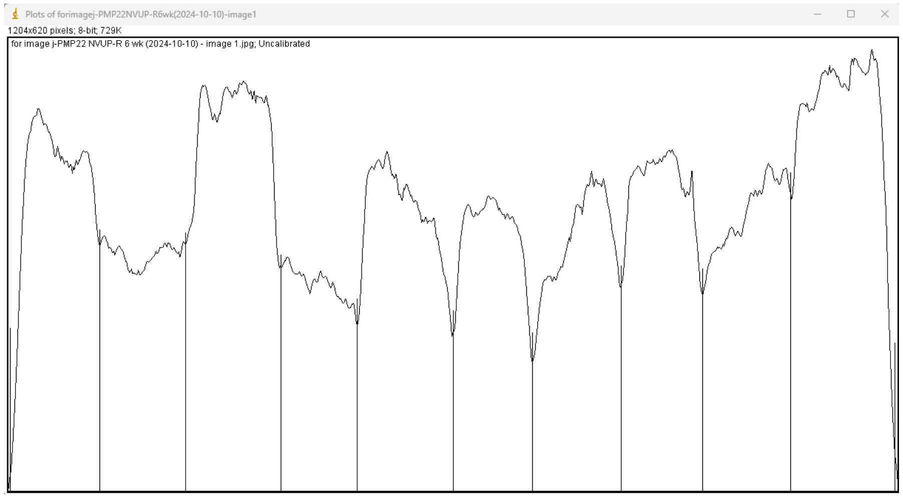

Date: 19 Dec 2024 LMW

Plot 2 Values B-Actin

	Area
1	46459.76
2	60552.85
3	32401.13
4	53305.49
5	49341.73
6	39012.25
7	51837.44
8	47421
9	48670.73
10	46819.61

Plot 2-B-Actin

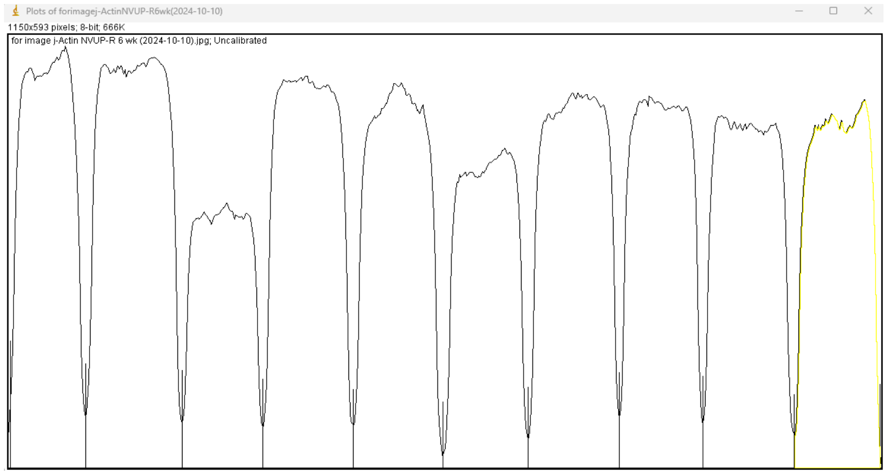

Date: 19 Dec 2024 LMW

Western Blot Imaging Form

Plot 3 Values-MPZ

	Area
1	33394.33
2	66907.86
3	45792.28
4	51936.52
5	54098.13
6	39953.4
7	46984.47
8	44838.93
9	53040.98
10	50862.78

Plot 3-MPZ

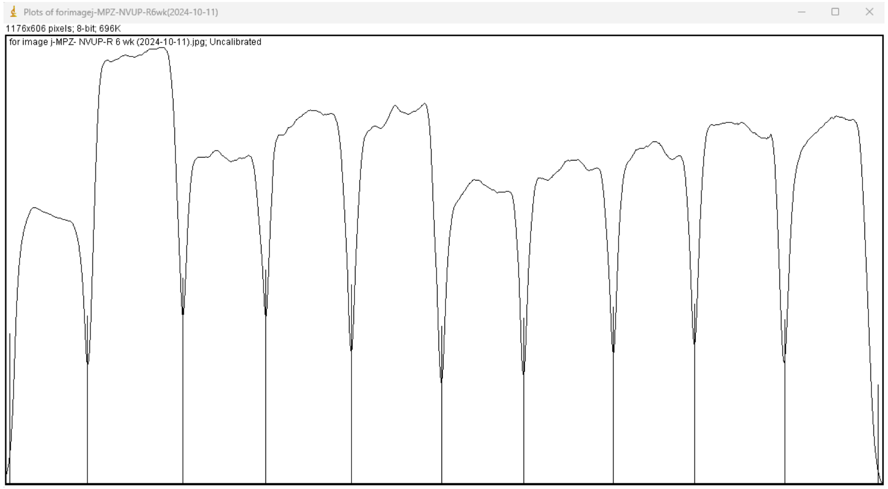

Date: 19 Dec 2024 LMW

Operator Information

Performed By:	Name: Merlin P Thangaraj	Signature: [signature]
	Position/Lab: Postdoctoral Scientist/Harper Lab	Date: 8-1-2025

Performed By:	Name: Lindsay Wallace	Signature: [signature]
	Position/Lab: Sr Research Scientist/Harper Lab	Date: 19 Dec 2024

Western Blot Imaging Form

Version 01
Page **1** of **4**

Study	ARM101-CMT1A-NHP-001
Timepoint	12 week
Tissue	Ulnar Nerve - Right
Anatomical Location (Highlight/Circle one)	Distal; Proximal (circled); N/A; Other:

Stain Free Gel

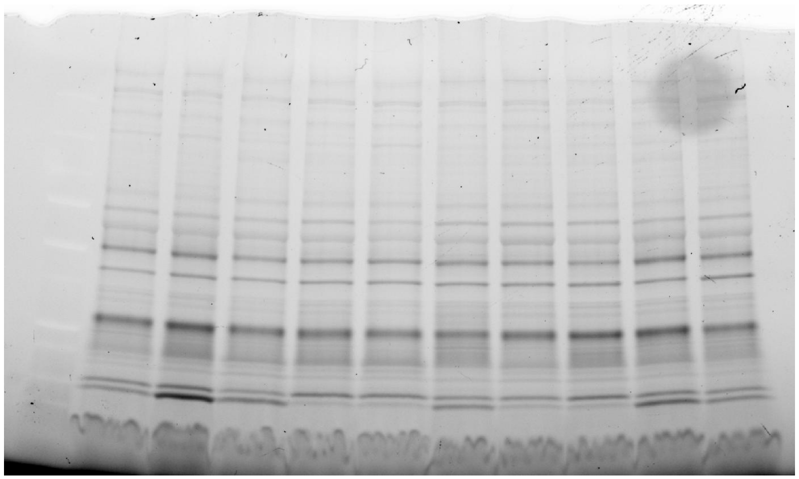

Date: 17 Sep 2024 MPT

Stain Free Membrane

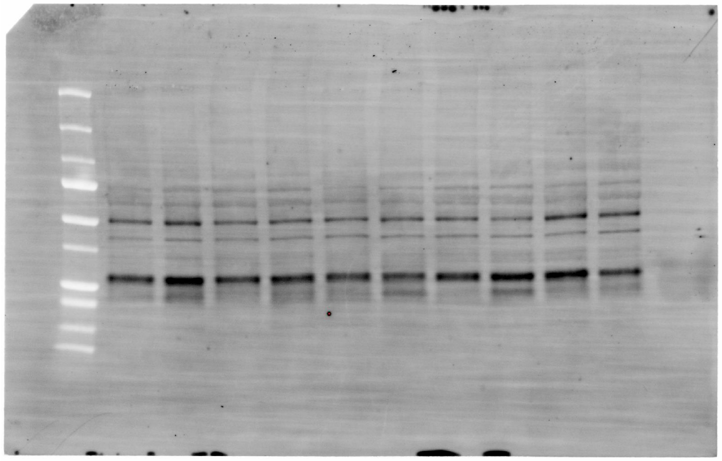

Date: 17 Sep 2024 MPT

1° PMP22

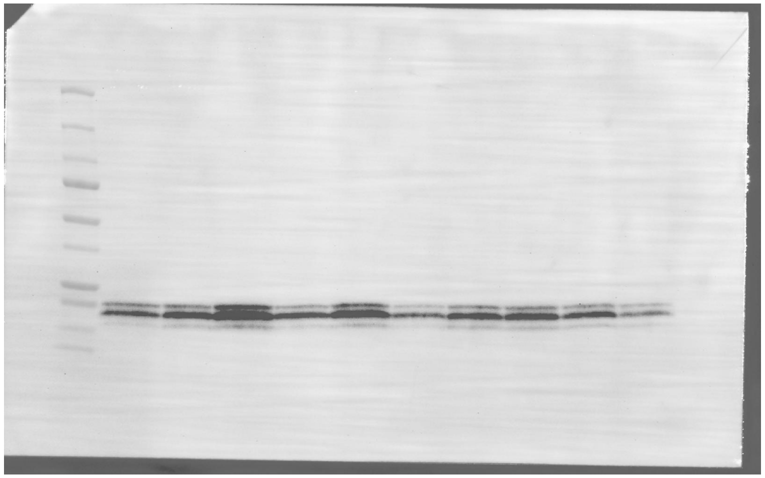

Date: 18 Sep 2024 MPT

1° β-Actin

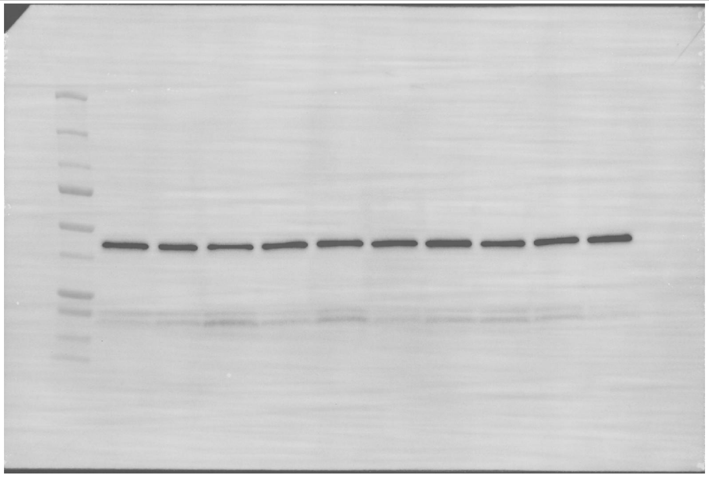

Date: 18 Sep 2024 MPT

Western Blot Imaging Form

Strip Check

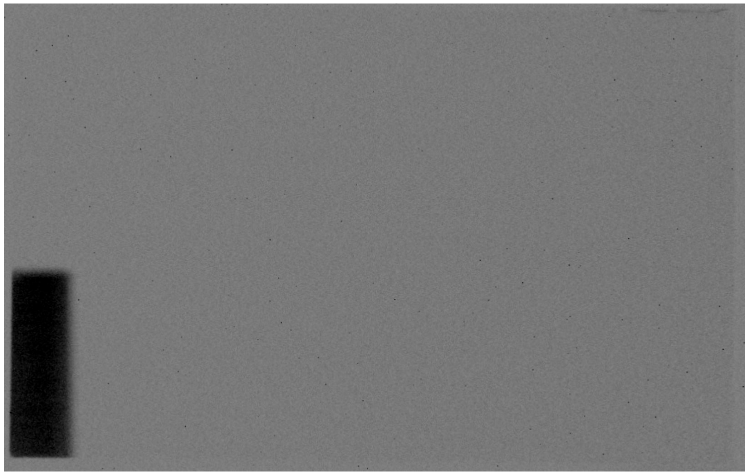

Date: 23 Sep 2024 MPT

1° MPZ

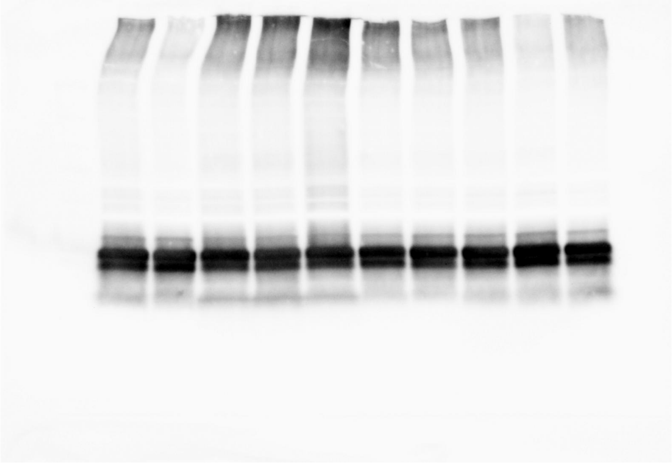

Date: 24 Sep 2024 MPT

ImageJ Quantification Box – PMP22

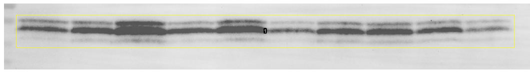

Date: 25 Sep 2024 LMW

ImageJ Quantification Box – B-Actin

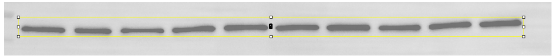

Date: 25 Sep 2024 LMW

ImageJ Quantification Box - MPZ

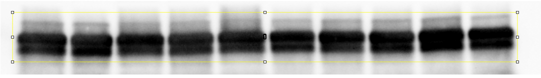

Date: 25 Sep 2024 LMW

Plot 1 Values PMP22

	Area
1	43103.63
2	40869.95
3	73020.46
4	31708.61
5	55989.46
6	22677.61
7	43880.97
8	46331.27
9	36763.97
10	18149.44

Plot 1-PMP22

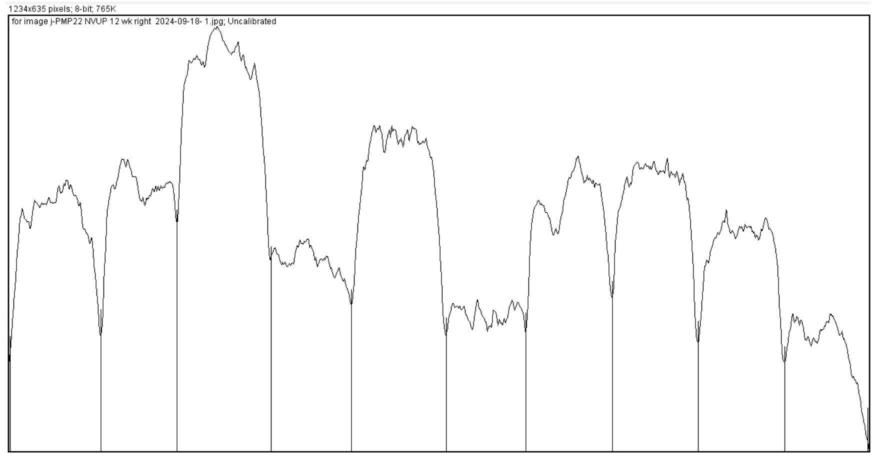

Date: 25 Sep 2024 LMW

Plot 2 Values B-Actin

	Area
1	55813.73
2	46073.08
3	43958.39
4	51878.68
5	55655.39
6	57875.27
7	60721.97
8	49692.56
9	52327.73
10	53129.2

Plot 2-B-Actin

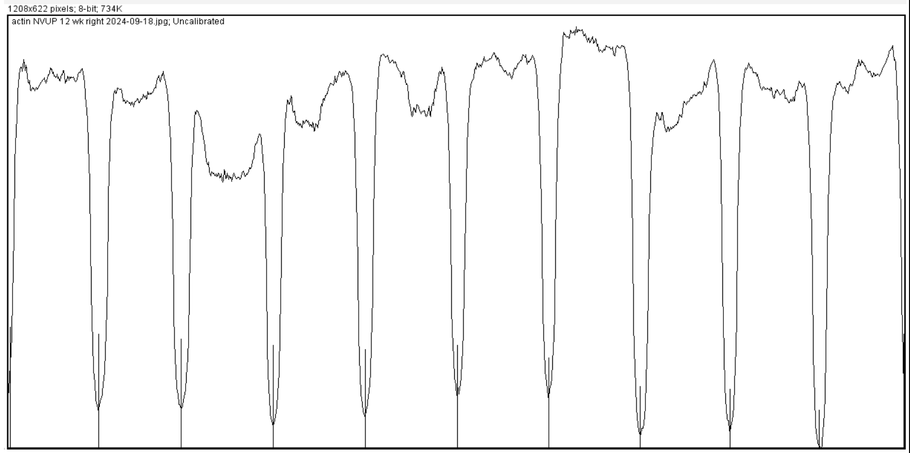

Date: 25 Sep 2024 LMW

Plot 3 Values-MPZ

	Area
1	62166.92
2	53353.25
3	60514.2
4	56141.73
5	61022.68
6	54768.66
7	57006.15
8	58396.78
9	64010.32
10	58094.44

Plot 3-MPZ

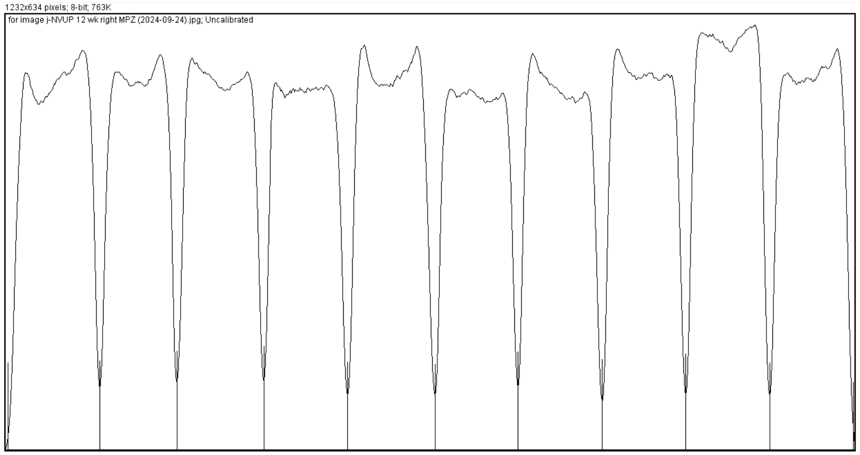

Date: 25 Sep 2024 LMW

Operator Information

**Performed By:**	**Name:** Merlin P Thangaraj	**Signature:** [signature]
	**Position/Lab:** Postdoctoral Scientist / Harper Lab	**Date:** 8-1-2025

**Performed By:**	**Name:** Lindsay Wallace	**Signature:** [signature]
	**Position/Lab:** Sr Research Scientist / Harper Lab	**Date:** 29 Oct 2024
