# Supplementary material for: Safety, efficacy, and distal nerve Schwann cell biodistribution in mice and NHPs to support translation of AAV9 RNAi therapy for CMT1A
Source: Mol Ther Nucleic Acids. 2026 Feb 27;37(2):102881. doi: 10.1016/j.omtn.2026.102881 (PMC13051718; doi:10.1016/j.omtn.2026.102881)
Supplement: Document S2. Article plus supplemental information [file mmc5.pdf]

Molecular Therapy
Nucleic Acids
Original Article

Safety, efficacy, and distal nerve Schwann cell biodistribution in mice and NHPs to support translation of AAV9 RNAi therapy for CMT1A

Marina Stavrou,[1,15] Lindsay M. Wallace,[2,15] Merlin P. Thangaraj,[2] Noah K. Taylor,[2] Alexia Kagiava,[1] Revekka Papacharalambous,[3] Cynthia McAllister,[4] Gloria Zender,[2] Nizar Y. Saad,[2,14] M. Bilal Bayazit,[2] Amanda Heslegrave,[5,6] Christina Tryfonos,[7] Jan Richter,[7] Henrik Zetterberg,[5,6,8,9,10,11] Brian Price,[12] Rachel Salzman,[12] Kleopas A. Kleopa,[1,13,16] and Scott Q. Harper[2,12,14,16]

[1]Neuroscience Department, The Cyprus Institute of Neurology and Genetics, Nicosia, Cyprus; [2]Jerry R Mendell Center for Gene Therapy, The Abigail Wexner Research Institute at Nationwide Children's Hospital, Columbus, OH 43210, USA; [3]Neuropathology Laboratory, The Cyprus Institute of Neurology and Genetics, Nicosia, Cyprus; [4]Histopathology Core at Nationwide Children's Hospital, Columbus, OH 43210, USA; [5]Department of Neurodegenerative Disease, UCL Queen Square Institute of Neurology, London, UK; [6]Dementia Research Institute, UCL, London, UK; [7]Virology Department, The Cyprus Institute of Neurology and Genetics, Nicosia, Cyprus; [8]Department of Psychiatry and Neurochemistry, Institute of Neuroscience and Physiology, The Sahlgrenska Academy, University of Gothenburg, Gothenburg, Sweden; [9]Clinical Neurochemistry Laboratory, Sahlgrenska University Hospital, Gothenburg, Sweden; [10]Hong Kong Center for Neurodegenerative Diseases, Hong Kong Science Park, Shatin, Hong Kong; [11]Wisconsin Alzheimer's Disease Research Center, University of Wisconsin School of Medicine and Public Health, University of Wisconsin-Madison, Madison, WI, USA; [12]ArmatusBio Inc, Columbus, OH 43210, USA; [13]Center for Neuromuscular Disorders, The Cyprus Institute of Neurology and Genetics, Nicosia, Cyprus; [14]Department of Pediatrics, The Ohio State University College of Medicine, Columbus, OH 43210, USA

Charcot-Marie-Tooth (CMT) type 1A, the most common inherited demyelinating peripheral neuropathy, is caused by PMP22 gene duplication, leading to overproduction of PMP22 protein in Schwann cells. To treat CMT1A, we developed a PMP22 gene silencing therapy using adeno-associated viral vectors (AAV9) to deliver a therapeutic miRNA expression cassette (U6.miR871) via lumbar intrathecal administration. A single injection produced long-term miR871 expression, triggered selective RNA interference against the PMP22 mRNA, and subsequently lowered protein levels and improved disease manifestations in a humanized CMT1A model. To support clinical translation, we confirmed on-target specificity of miR871 for PMP22 *in vitro*, identified a safe and effective dosing range in mice, demonstrated absence of significant toxicity in rodents and non-human primates (NHPs), and performed a detailed AAV biodistribution study in a large animal model. We found vector biodistribution and miR871 expression in distal peripheral nerves, PMP22 target engagement in mice and NHPs, and silencing to levels expected to support normal myelination in humans. We identified the minimally efficacious to maximum tolerated dose range of AAV9.U6.miR871 in mice and confirmed safety range in NHPs for extrapolation to anticipated clinical trials. Our study supports the scale-up potential of gene therapy to treat CMT1A and other demyelinating peripheral neuropathies.

INTRODUCTION

The peripheral nervous system (PNS) links the brain and spinal cord to the rest of the body through transmission of sensory, motor, and autonomic information. Peripheral neuropathy can arise when the PNS is damaged by physical injury or disease, such as diabetes, autoimmunity, certain viral infections, and relevant to this study, gene mutations. Indeed, inherited peripheral neuropathies can arise from single mutations in more than 100 different genes, most of which are classified as Charcot-Marie-Tooth (CMT) diseases.[1–3] Although CMTs are often grouped together, each known CMT form represents a unique disorder with distinct genetic mechanisms that will likely require gene- and disease-specific therapies.[1–3] Among the different CMT forms, CMT type 1A (CMT1A) predominates, accounting for roughly 50% of all cases (1 in 5,000 prevalence).[2] CMT1A is caused by duplication of a normal *peripheral myelin protein 22* (*PMP22*) gene, which encodes a structural protein (PMP22) used by Schwann cells to correctly deposit a protective myelin sheath around peripheral axons.[4–6] Thus, humans with CMT1A have three *PMP22* gene copies that produce ~1.5× increased wild-type PMP22 protein in Schwann cells, leading to protein misfolding, aggregation, and ultimately myelin sheath destabilization and dysfunction.[4–6] The lack of functional protective myelin on peripheral axons eventually causes progressive motor and sensory axonopathy and neurodegeneration, manifesting in people with

Received 29 September 2025; accepted 24 February 2026;
https://doi.org/10.1016/j.omtn.2026.102881.

[15]These authors contributed equally

[16]Senior author

Correspondence: Scott Q. Harper, Jerry R Mendell Center for Gene Therapy, The Abigail Wexner Research Institute at Nationwide Children's Hospital, Columbus, OH 43210, USA.
E-mail: scott.harper@nationwidechildrens.org

 © 2026 The Author(s).
Published by Elsevier Inc. on behalf of The American Society of Gene and Cell Therapy.
This is an open access article under the CC BY license (http://creativecommons.org/licenses/by/4.0/).

CMT1A as distal muscle weakness and atrophy, sensory loss in extremities, slowed nerve conduction, and significant disability.[3]

No approved disease-modifying therapies currently exist for CMT1A, leaving affected families few options to fight the relentless disorder. Since CMT1A is caused by overproduction of an otherwise normal PMP22 protein in Schwann cells, the most direct approach to CMT1A therapy would involve reducing PMP22 to normal levels. Several experimental strategies to lower *PMP22* are being or have been tested in pre-clinical and clinical studies, including approaches that will require repeated, lifelong dosing of drug cocktails or non-viral nucleic acid therapies such as small interfering RNAs (siRNAs) or antisense oligonucleotides (ASOs) conjugated within liposomes or analogous chemical delivery systems.[1,3,7–11] The success of each approach requires efficient product delivery to Schwann cells, which can be challenging therapeutic targets, well-protected by several anatomical structures that impede entry of foreign materials. For example, a prospective RNA- or DNA-based product, such as an siRNA, ASO, or viral vector, would have to enter Schwann cells by first bypassing the blood-nerve barrier and traverse two layers of connective tissue surrounding nerve fascicles (perineurium) and nerve fibers (endoneurium), before crossing the Schwann cell membrane or nuclear membrane to elicit its mechanism of action. While non-viral nucleic acid approaches typically require chemical conjugation to facilitate delivery, adeno-associated viral vectors (AAVs) have natural tropism for Schwann cells and can therefore efficiently deliver a therapeutic DNA payload to Schwann cell nuclei and possibly offer years-long protection after only one administration.[10,12,13]

We therefore developed a gene therapy for CMT1A that relies upon self-complementary AAV9 vectors to deliver a therapeutic, *PMP22*-lowering DNA payload to Schwann cells *in vivo*.[10] Specifically, our therapeutic DNA payload contains a U6 promoter to direct transcription of an engineered microRNA, called miR871 (U6.miR871), that we designed to trigger RNA interference (RNAi) against the *PMP22/Pmp22* mRNA and ultimately reduce toxic PMP22/Pmp22 protein levels in C61 heterozygote mice, a model of CMT1A.[10] Importantly, we built miR871 with intentional conservation, so that the mature, guide strand sequence would base pair with mouse, monkey, and human *PMP22* mRNAs and could be efficiently translated from animal models to humans. In our first proof-of-principle study, we demonstrated that lumbar intrathecal (IT) delivery of 5E11 vg of AAV9.U6.miR871 per adult CMT1A mouse significantly reduced *PMP22/Pmp22* mRNA and PMP22/Pmp22 protein levels and rescued numerous molecular, cellular, and functional neuropathic phenotypes.[10] These promising results encouraged us, in this study, to address several issues required to translate AAV9.U6.miR871 toward clinical trials, including (1) optimizing the DNA payload for use in humans; (2) assessing potential miR871-sequence-specific off-target effects in human cells; (3) identifying minimal efficacious dose and maximum tolerated dose of optimized AAV9.U6.miR871 following intrathecal delivery in mice; (4) assessing AAV9.U6.miR871 safety and toxicology in small and large animal models; and (5) determining the safety and feasibility of delivering AAV9 to distal peripheral nerve Schwann cells following lumbar intrathecal injection in large animals (non-human primates [NHPs]), which was unknown prior to this study. This last question was particularly important, as the success of the therapy hinges upon efficient delivery to Schwann cells. While we previously showed that IT-delivered AAV9 vectors transduce distal nerves in mice, the vector in mouse models only must travel ∼22 millimeters through the cerebrospinal and endoneurial fluid to reach the distal peripheral nerves. In contrast, the same injection in humans would have to travel perhaps up to a meter or more to reach Schwann cells at the distal sciatic nerve. To approximate the anatomical distance an AAV9 vector could travel from the site of injection following lumbar intrathecal delivery, we performed an AAV9.U6.miR871 biodistribution and target engagement study in wild-type NHPs. Lumbar IT delivery produced broad biodistribution to peripheral NHP nerves, leading to significant *PMP22* mRNA and PMP22 protein silencing at two different doses and time points, with no evidence of toxicity. *In vitro*, murine, and NHP data generated in this study support the feasibility of translating AAV-delivered RNAi-based gene therapy for CMT1A into human trials.

RESULTS

In vitro on-/off-target assessment in human cells

RNAi is a conserved process of sequence-specific gene silencing mediated by small, non-coding microRNAs (miRNAs).[14–16] Natural miRNAs arise from eukaryotic genomes as primary, non-coding transcripts and are processed to mature, 22-nucleotide (nt) forms that then associate with proteins of the RNA-induced silencing complex (RISC).[17–19] Functionally, the miRNA provides specificity to RISC, guiding it through base-pairing to certain cellular mRNAs, triggering mRNA degradation or sequestration from the ribosome. Importantly, natural miRNAs can be modified to direct RNAi against any sequence of interest, including disease genes.[10,20–26] We previously designed an artificial miRNA, called miR871, to bind and trigger silencing of human, mouse, and monkey *PMP22* mRNAs, thereby offering a path to translate this prospective gene silencing treatment for CMT1A.[10] We demonstrated that intrathecal delivery of AAV9.U6.miR871 normalized *PMP22/Pmp22* levels in peripheral nerve Schwann cells of C61-het CMT1A mice, leading to improved myelination and reduction or elimination of several molecular, histopathological, and functional deficits.[10] These results supported translation of the approach to humans, and as part of this process, we sought to assess the safety of miR871 overexpression in human pre-clinical models.

RNAi is considered a precision gene silencing approach, but RNAi-based therapies could theoretically cause side effects or toxicity related to unintended off-target silencing of important cellular transcripts. We used RNA sequencing (RNA-seq) to assess the on-target specificity or off-target effects of miR871 in human hepatocytes, neurons, and Schwann cells, as these cell lines represent tissues highly transduced following intrathecal AAV9 delivery. Because these cell lines are not efficiently transfected or transduced by AAV vectors *in vitro*, we cloned U6.miR871 into lentiviral vectors co-expressing

Table 1. RNA-seq data show few gene expression changes with miR871 expression in human cells

		# of reduced transcripts		# of increased transcripts		
	Total transcripts	FDR <0.05 and >2 FC	FDR <0.05 and >1.75 FC	FDR <0.05 and >2 FC	FDR <0.05 and >1.75 FC	*PMP22* expression in miR871-treated cells
miR871 + Puro vs. Puro						
Neuron	18,175	1	1	0	0	3.6-fold reduction; FDR 3.5e−33
Hepatocyte	17,394	14	22	2	6	1.9-fold reduction; FDR 1.1e−33
Schwann cell	15,462	2	4	0	0	1.8-fold reduction; FDR 0.0026
Puro vs. no Puro						
Schwann cell	15,901	313	480	562	795	no significant change

FDR, false discovery rate; FC, fold-change; Puro, puromycin.

a *puromycin resistance (PuroR)* gene, transduced cells at <1 multiplicity of infection, and added puromycin to culture media. Following selection of triplicate samples per condition, we harvested RNA and performed RNA-seq to assess gene expression changes associated with miR871 expression (Table 1).

From an average of ∼16,000 transcripts detected per cell line, only 2 were increased and 14 significantly reduced (using ≥2-fold change; false discovery rate [FDR] padj <0.05 as cutoff), including 1 in neurons, 11 in hepatocytes, and 2 in Schwann cells (Tables 1 and S1). Notably, *PMP22* was the lone downregulated transcript in neurons, and we also measured *PMP22* reductions in hepatocytes and Schwann cells although *PMP22* levels did not reach the initial 2-fold cutoff (1.9- and 1.8-fold reductions, respectively). Since *PMP22* was our target gene, we adjusted the fold-change cutoff to 1.75 in all three cell lines, resulting in a total of 25 significantly downregulated transcripts. As noted, *PMP22* was the only transcript significantly reduced in all three cell lines, while in contrast, the 24 other downregulated transcripts in miR871-treated cells were not universally reduced despite being expressed in the other cells lines tested (Tables 1 and S1). For example, *STMN2* and *RGS5* were significantly reduced in miR871-treated Schwann cells but unchanged in miR871-treated hepatocytes and neurons despite being detected by RNA-seq in both cell lines. Thus, the lack of uniform knockdown across all cell lines suggested that miR871 was not triggering RNAi against *STMN2*, *RGS5*, and all other putatively off-target transcripts in this experiment (Table S1). As an additional control, we compared RNA-seq signatures between untreated and Puromycin-treated Schwann cells without miR871 and found that Puromycin treatment alone caused 1,275 significant gene expression changes at the 1.75-fold cutoff, including 480 reductions, representing 3% of total Schwann cell transcripts detected (Table 1). Together, these results suggested high on-target specificity of miR871 for *PMP22* sequences and low likelihood of off-target silencing. Nevertheless, to determine if mature miR871 sense or antisense sequences contained sufficient base pairing to bind and possibly silence any empirically measured downregulated transcripts, we performed *in vitro* binding assays using an approach we previously described but adapted to miR871 sequences.[26] Briefly, we used bioinformatics software (RNAhybrid)[27] to identify putative miR871 binding sites on representative downregulated transcripts identified in RNA-seq and synthesized 47 different candidate binding sites as oligonucleotides (23 predicted to bind the miR8781 antisense strand; 24 predicted to bind the miR871 sense strand), along with controls containing perfect 22-nt miR871 sense or antisense on-target binding sites (Figure S1). We then performed binding assays using molecular beacon oligonucleotides containing a quencher (zenBHQ) at one end and fluorophore (6FAM) at the other. Unbound molecular beacons form intramolecular hairpins and do not emit fluorescence, while binding of a target site separates the molecular beacon ends, enabling fluorescence emission. The perfect, 22-nt miR871 antisense or sense target sites on *PMP22* caused fluorescence emission yielding a binding curve with K_d in the nanomolar range, consistent with previous studies (Figure S1).[24,26] Among the 47 putative off-target binding sites tested, only five showed minimal fluorescence emission in the binding assay, all with K_d in the micromolar range (*ANKRD18B*, two sites; *MYBPC3*, one site; *RGS5*, two sites). Among the putative off-targets tested, a site on *RGS5* had the strongest binding affinity (K_d 1.5 micromolar), which was 25× weaker than the perfect on-target site located on *PMP22* (Figure S1). Together, these results support that miR871 has high specificity for *PMP22* without significant sequence-specific off-target effects.

Second-generation AAV9.U6.miR871 vector optimization for *in vivo* translational use

The first-generation vectors used in our proof-of-principle studies contained the U6.miR871 expression cassette adjacent to a separate CMV.eGFP reporter gene.[10] To translate this approach, we replaced CMV.eGFP with a stuffer DNA comprised of a human collagen intron sequence. The resultant 2,053 base pair (bp) genome was sufficiently small to package into AAV9 as a self-complementary vector (AAV9.U6.miR871). Prior to performing *in vivo* studies, we confirmed human *PMP22* and mouse *Pmp22* silencing from the new vector compared to the original (Figure S2). To do this, we co-transfected HEK293 cells with the original and new U6.miR871 AAV proviral plasmids along with a reporter plasmid containing human *PMP22* or mouse *Pmp22* sequences inserted as the 3′ untranslated region (3′ UTR) of *Renilla* luciferase (Figure S2). As expected,

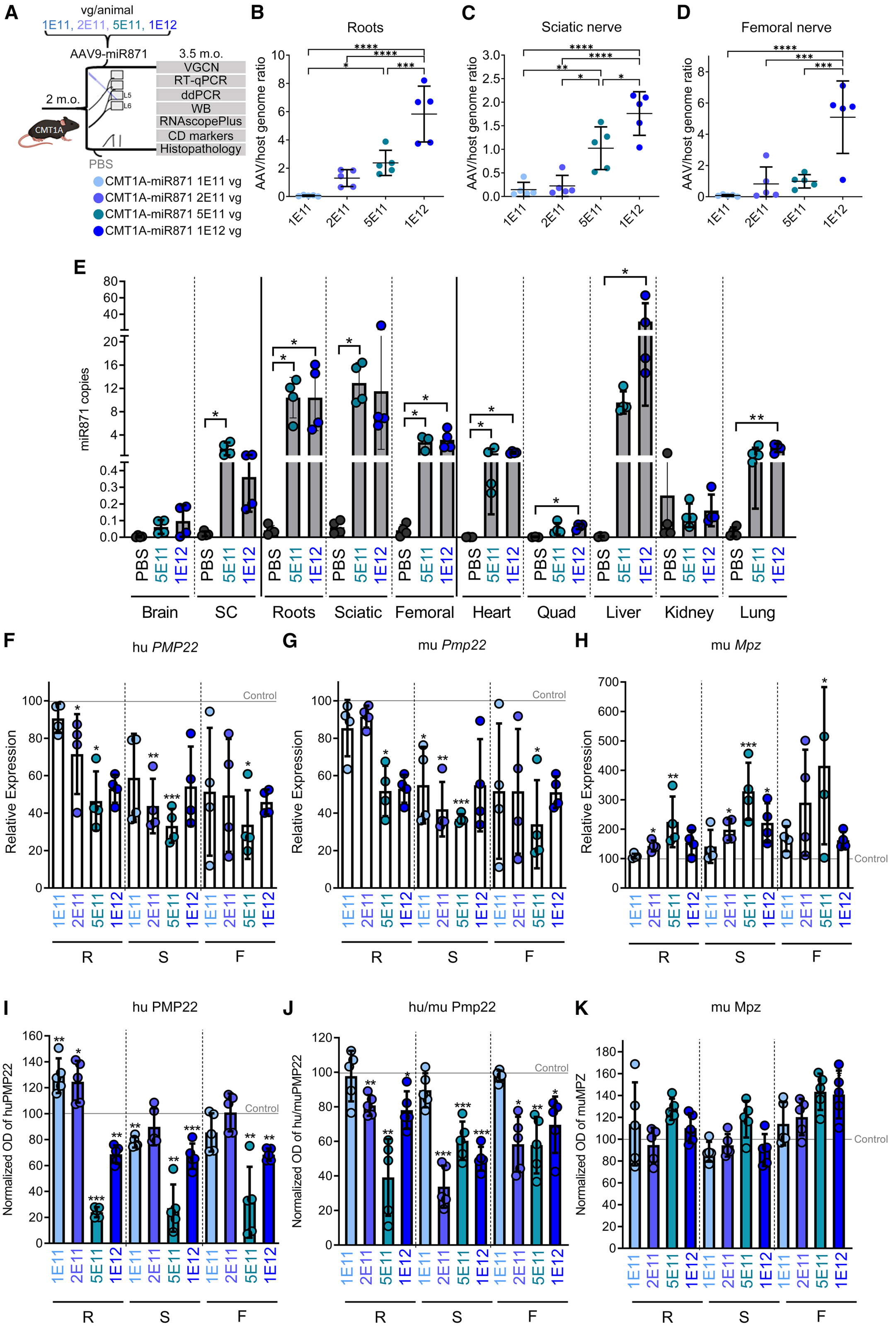

(legend on next page)

both constructs triggered *Renilla* luciferase-*PMP22/Pmp22* silencing, with the 2nd generation vector performing better in both assays (Figure S2).

AAV9.U6.miR871 dose finding and *in vivo* efficacy studies in CMT1A mice

We next generated AAV9.U6.miR871 vectors and performed dose-escalation, biodistribution, and *in vivo* efficacy studies in 2-month-old C61-het CMT1A mice. To do this, we injected the lumbar intrathecal (IT) space with AAV9.U6.miR871 using doses of 1E11, 2E11, 5E11, and 1E12 vector genomes (vg)/animal or saline controls (Figure 1A). Six weeks later, when mice were 3.5 months old, we harvested tissues to assess (1) AAV DNA biodistribution (AAV vector genome copy number [VGCN]); (2) expression of mature miR871 antisense RNA, human *PMP22*, mouse *Pmp22*, and mouse *Mpz* RNAs by droplet digital PCR (ddPCR) or real-time qPCR (RT-qPCR); and (3) levels of human PMP22, mouse Pmp22, and mouse Mpz protein by western blot (Figures 1 and S3–S6). We used Mpz (P0) expression here as an indirect indicator of nerve health. Mpz, the most abundant Schwann cell protein, is reduced in nerves of CMT1A mice and increased with improved myelination. All mouse experiments used male and female animals, and overall, we saw no sex-related differences in biodistribution, behavioral performance, or nerve pathology. VGCN analysis revealed dose-dependent transduction in both PNS (Figures 1B–1D) and non-PNS tissues (Figure S7), except in liver, where the penultimate dose (5E11) showed ∼4× more VGCN than the lowest (1E11, 2E11) and highest (1E12) doses. Using a custom ddPCR assay, we found miR871 expressed above background (saline-injected animals) in all tissues examined (Figure 1E). In addition, among all tissues, miR871 expression was highest in the spinal roots, sciatic nerves, and femoral nerves and comparable to levels produced in the liver, despite the liver showing 10–20× more VGCN than peripheral nerves. We also noted that miR871 expression plateaued at 5E11 and did not increase at 1E12 except in the liver (Figure 1E). The discordance between vector genomes and miR871 expression at this highest dose could be related to Schwann cell dysfunction impacting gene expression at the 1E12 dose, which we document throughout the mouse portion of this study (Figures 1, 2, and 3). Importantly, peak expression of miR871 at 5E11 correlated with consistent and robust knockdown of human *PMP22* and mouse *Pmp22* mRNA and PMP22/Pmp22 protein compared to other doses tested, with one exception (mouse Pmp22 protein showed greater reduction in sciatic nerve at 2E11 dose than other doses) (Table S2; Figures 1F, 1G, and 1I–1K). Thus, our molecular and biochemical analyses suggested 5E11 vg/animal as the most effective dose for consistent silencing of hu*PMP22*/PMP22 and mu*Pmp22*/Pmp22 mRNA and protein across PNS tissues. In addition, this dose was associated with increased levels of mouse *Mpz* mRNA and Mpz protein, indicating enhanced myelination and improved health of PNS tissues (Table S2; Figures 1H, 1K, and S3–S5). As an exception, Mpz protein levels remain unchanged in the sciatic nerves, aligning with prior observations showing that C61 het mice do not present prominent myelination deficits in this tissue.[10]

To perform *in vivo* efficacy studies, we injected a second cohort of animals. Specifically, we injected 2-month-old CMT1A mice with AAV9.U6.miR871 at doses of 1E11, 2E11, 5E11, or 1E12 vg/animal and performed several behavioral outcome measures 2 and 4 months later, when mice were 4 or 6 months old (Figure 2A). Controls included age-matched CMT1A mice injected with AAV9.U6.miRLacZ (2E11 vg/animal), which does not target *PMP22/Pmp22*, as well as non-injected WT and CMT1A mice. We then assessed the ability of AAV9.U6.miR871 to improve known deficits on several outcomes, including rotarod, grip strength, hang test, and hindlimb clasping. Consistent with our biodistribution, miR871 expression, and *PMP22/Pmp22* target engagement experiments, our behavioral data supported that 5E11 vg/animal was the maximum effective dose for improving functional performance of CMT1A animals (Figures 2B–2H and S8). Specifically, C61-het mice injected with 5E11 AAV9.U6.miR871 showed significantly improved rotarod performance compared to all other groups except wild-type, from which 5E11-treated CMT1A mice were indistinguishable. We also measured significantly improved grip strength and grid-hang performance (another test of strength and endurance) in CMT1A mice injected with 5E11 AAV9.U6.miR871 particles compared to other treatment groups, including AAV9.U6.miRLacZ-treated or uninjected CMT1A animals. Finally, CMT1A mice display hindlimb clasping phenotypes often seen in other mouse models of neurological disease but not in wild-type animals, which can be quantified by measuring the angle of hindlimb abduction (Figures 2G and 2H). Treatment with the 5E11 dose of AAV9.U6.miR871 normalized this clasping phenotype, while the 1E11, 2E11, and 1E12 doses only provided partial improvements (Figure 2G). Notably, for all behavioral outcome measures, mice in the 1E12 dose group performed worse than those that received 5E11 particles of same vector. These data, coupled with our AAV biodistribution data showing that mice injected with the 1E12 dose had the highest neural tissue

Figure 1. Biodistribution, transduction efficiency, and molecular effects of AAV9.U6.miR871 dose response

(A) Experimental design for *in vivo* testing of four different AAV9.U6.miR871 doses. (B–D) Vector genome copy number (VGCN) measurements showed dose-dependent transduction of PNS tissues ($n = 5$/group). (E) ddPCR detected mature miR871 sequence in the PNS and peripheral tissues. (F–H) Human (hu) *PMP22*, murine (mu) *Pmp22*, and mu *Mpz* gene expression detected in spinal nerve roots, sciatic nerve, and femoral nerve ($n = 4$/group). Fold changes in relative mRNA expression of CMT1A animals treated with different AAV9.U6.miR871 doses were calculated in comparison with expression levels of PBS-injected CMT1A mice. All samples were normalized to endogenous mouse *Gapdh*. (I–K) Western blot analysis showing protein optical densities (ODs) of human PMP22, murine Pmp22, and murine Mpz from CMT1A animals treated with different AAV9.U6.miR871 doses normalized to tubulin and compared to expression levels in age-matched PBS-injected CMT1A mice. Western blot images and unnormalized optical density calculations are shown in Figures S3–S6. Values are presented as mean ± SD. *$p < 0.05$, **$p < 0.01$, ***$p < 0.001$, and ****$p \leq 0.0001$ by one-way ANOVA with Tukey's multiple-comparison test. R, lumbar roots; S, sciatic nerves; F, femoral nerves.

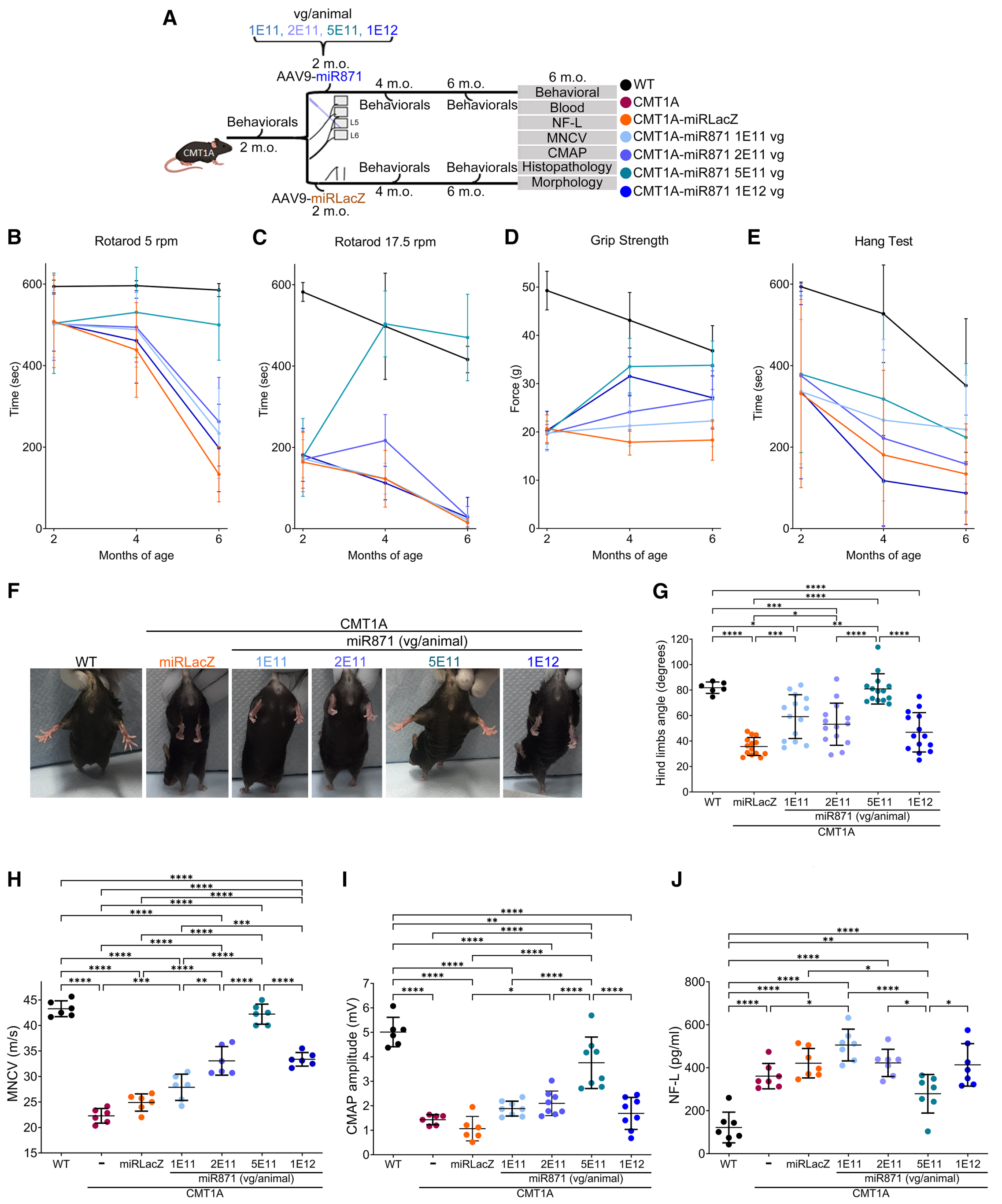

Figure 2. Effects of AAV9.U6.miR871 dosing on motor behavioral, electrophysiological, and blood biomarker phenotypes in CMT1A mice

(A) Study design for the early treatment trial. (B) Lack of significant liver enzyme elevations support safety in mice. AST, aspartate aminotransferase; ALT, alanine aminotransferase. (C–F) Behavioral assessments, including rotarod, grip strength, and hang test, were conducted in WT mice (rotarod, grip strength, and hang test: *n* = 10; hindlimb abduction: *n* = 6), and CMT1A mice were treated with different doses of AAV9.U6.miR871 or AAV9.U6.miRLacZ (*n* = 14/group). Behavioral tests were performed before treatment (2 months of age) and monitored every 2 months until 6 months of age, while (G and H) hindlimb abduction angle was assessed only at the final time point. Raw behavioral scores and statistical analyses are provided in Figure S8. (I and J) Electrophysiological analyses, including motor nerve conduction velocity (MNCV) and compound muscle action potential (CMAP), along with (K) serum NF-L levels analyses, were performed at 6 months of age. Non-injected WT and CMT1A mice (electrophysiology: *n* = 6/group; NF-L: *n* = 7/group) were compared to CMT1A mice treated with indicated doses of AAV9.U6.miR871 (electrophysiology: *n* = 8/group; NF-L: *n* = 7/group) and AAV9.U6.miRLacZ (electrophysiology: *n* = 6/group; NF-L: *n* = 7/group). Values are presented as mean ± SD. *$p < 0.05$, **$p < 0.01$, ***$p < 0.001$, and ****$p \leq 0.0001$ by one-way ANOVA with Tukey's multiple-comparison test.

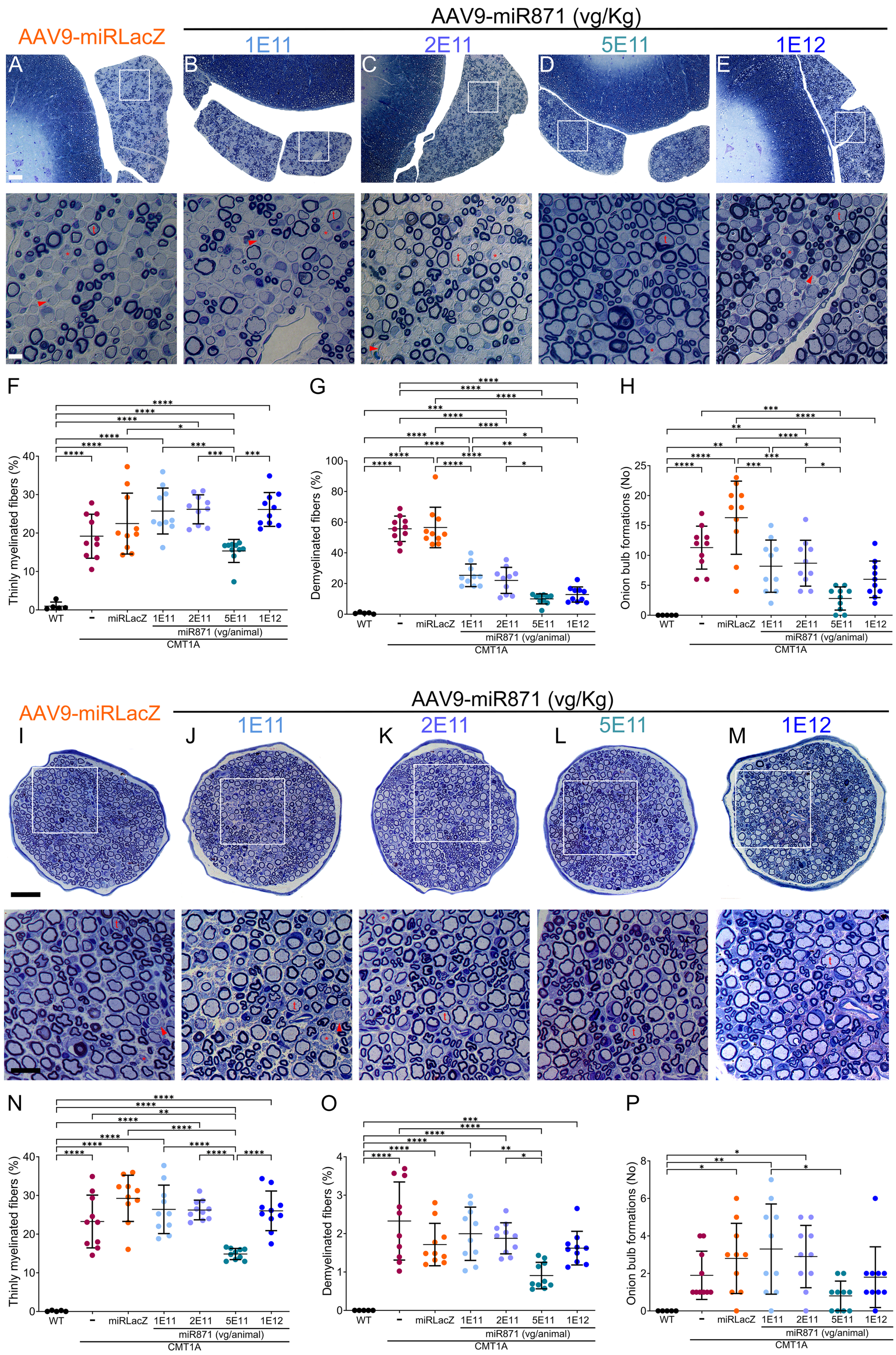

(legend on next page)

transduction but ∼4× fewer AAV genomes in the liver than the 5E11 dose, suggested that 1E12 was less efficacious and possibly deleterious.

In addition to the behavioral assays described above, prior to study termination we also performed electrophysiology and serum biomarker analysis as additional outcome measures with direct translational implications for human studies. Specifically, similar to humans with CMT1A, C61-het mice show reduced motor nerve conduction velocities (MNCV) and compound muscle action potentials (CMAP), which indicate nerve damage and subsequent impairment of muscle function.[10] In humans and mice, nerve damage is associated with presence of serum neurofilament light chain (NF-L), which is now being explored as a biomarker of CMT1A disease progression. From doses 1E11 to 5E11 vg/animal, we measured statistically significant, dose-dependent improvement of MNCV and CMAP scores (Figures 2I and 2J). Importantly, CMT1A animals treated with the 5E11 dose had MNCV scores indistinguishable from wild-type (WT: 43.28 ± 1.56 m/s, CMT1A-miR871 5E11vg/animal: 42.22 ± 1.97 m/s; Figure 2I) and significantly improved CMAP scores (WT: 5.01 ± 0.60 mV, CMT1A-miR871 5E11vg/animal: 3.76 ± 1.05 mV; Figure 2J). Notably, similar to the behavioral data described above, animals treated with the highest dose, 1E12 vg/animal, showed significantly lower electrophysiology readouts than the 5E11 vg/animals, and in fact, 1E12-treated CMT1A mice showed no improvements in CMAP scores compared to animals injected with saline or AAV9.U6.miRLacZ. In line with the electrophysiology results, 5E11 was the only AAV9.U6.miR871 dose that significantly lowered serum NF-L levels (WT: 121.95 ± 71.56 pg/mL, CMT1A-miR871 5E11vg/animal: 278.96 ± 89.66 pg/mL; CMT1A untreated = 360.83 ± 59.07 pg/mL; CMT1A miRLacZ-treated = 421.12 ± 68.46 pg/mL; Figure 2K).

As a final *in vivo* efficacy measure, we assessed myelination of anterior lumbar roots and femoral motor nerves of 6-month-old CMT1A mice that had been injected at 2 months of age (4-month, terminal time point) with 1E11, 2E11, 5E11, or 1E12 vg/animal of AAV9.U6.miR871 (Figures 2A and 3). Controls included age-matched CMT1A mice injected with AAV9.U6.miRLacZ, as well as non-injected WT and CMT1A mice. We morphometrically examined multiple roots and bilateral femoral motor nerves from each mouse and calculated the percentage of thinly myelinated and demyelinated fibers, as well as the number of onion bulb formations, all of which indicate CMT1A-related neuropathy. Specifically, demyelinated axons are likely to degenerate over time, while thinly myelinated fibers already underwent demyelination and remyelination, but with insufficient Schwann cell function leading to inappropriately thin myelin sheaths. Onion bulbs reflect defective Schwann cells that are unable to myelinate due to disease or damage. Each of these phenotypes indicate Schwann cell pathology that leads to axonal dysfunction and loss in mice and humans. In the lumbar roots, only the 5E11 dose of AAV9.U6.miR871 significantly reduced the percentage of thinly myelinated fibers compared to the miRLacZ control group (CMT1A-miRLacZ: 22.47% ± 7.92%, CMT1A-miR871 5E11vg/animal: 15.35% ± 2.99%; Figures 3A–3F). Additionally, there was a dose-dependent reduction in the proportion of demyelinated fibers, which plateaued at 5E11 vg/animal (CMT1A-miRLacZ: 56.55% ± 13.19%, CMT1A-miR871 5E11vg/animal: 9.99% ± 3.25%; Figures 3A–3E and 3G). The number of onion bulb formations was decreased at all treatment groups, with only 5E11 vg/animal preserving WT levels (WT: 0, CMT1A-miR871 5E11vg/animal: 2.80 ± 1.93; Figures 3A–3E and 3H). Similarly, in femoral motor nerves (Figures 3I–3P), 5E11 was the only dose that improved the percentage of thinly myelinated (CMT1A-miRLacZ: 29.26% ± 5.98%, CMT1A-miR871 5E11vg/animal: 14.86% ± 1.43%; Figure 3N) and demyelinated fibers (CMT1A-miRLacZ: 1.71% ± 0.55%, CMT1A-miR871 5E11vg/animal: 0.91% ± 0.35%; Figure 3O) compared to the miRLacZ control group. Onion bulb formations in femoral nerves were already low at baseline and did not significantly improve after treatment, although we saw a non-significant trend of reduced onion bulbs at the 5E11 dose (Figure 3P).

Pre-clinical safety and toxicology studies in CMT1A mice

The 5E11 vg dose improved all outcomes in CMT1A mice and showed no evidence of treatment-related pathology (Figures 1, 2, and 3). Indeed, our histopathological analysis of neural and peripheral tissues (Figures S9–S12) as well as blood counts and serum analysis (Figures S13 and S14) revealed no toxicity related to vector delivery across all treatment groups. In contrast, CMT1A mice treated with the highest AAV9.U6.miR871 dose (1E12) lacked significant correction of several neuropathic phenotypes and in fact showed some evidence of potential liver toxicity (Figures S13 and S14).

To evaluate the potential toxicity of AAV9.U6.miR871, we administered 1E11, 2E11, 5E11, and 1E12 vg/animal doses to 2-month-old CMT1A mice and evaluated immune responses 6 weeks later, when mice were 3.5 months old, via immunohistochemical analysis of cluster of differentiation (CD) markers. We were particularly focused on presence of inflammatory cells near the spinal cord, as we previously demonstrated that C61-het mice show disease-related inflammatory infiltrates in nerve roots and we did not want to

Figure 3. Effects of AAV9.U6.miR871 dosing PNS tissue morphology in CMT1A mice

(A–E) Toluidine-blue-stained semithin sections of anterior lumbar spinal roots (I–M) and femoral motor nerves from CMT1A animals treated with AAV9.U6.miRLacZ or different doses of AAV9.U6.miR871, shown at low (upper panels) and higher magnifications (lower panels). Thinly myelinated (t) or demyelinated (red asterisk) fibers and onion bulb formations (red arrowhead) are indicated. (F–H) Quantification of abnormally myelinated fibers in lumbar motor roots and (N–P) femoral motor nerves was performed in 6-month-old non-injected WT (n = 5) and CMT1A mice (n = 10), as well as CMT1A mice treated with AAV9.U6.miRLacZ or indicated doses of AAV9.U6.miR871 (n = 10/group). Values are presented as mean ± SD. *$p < 0.05$, **$p < 0.01$, ***$p < 0.001$, and ****$p \leq 0.0001$ by one-way ANOVA with Tukey's multiple-comparison test. Scale bars: (A–E) 50 μm and 10 μm (enlarged inserts); (I–M) 40 μm and 25 μm (enlarged inserts).

exacerbate this inflammatory phenotype with gene therapy, since AAV9 had been previously suggested to cause sub-clinical inflammation of dorsal root ganglia.[28–31] Importantly, we found that the 5E11 vg/animal dose reduced the number of CD45+ (pan-leukocyte marker) and CD68+ (macrophage and monocyte marker) cells in CMT1A mouse spinal roots (Figure S15). Thus, instead of exacerbating inflammation, the 5E11 dose of AAV9.U6.miR871 reduced it. In contrast, administration of 1E12 vg/animal increased inflammation, with animals showing increased CD3+ (T cell marker) and CD68+ cells in the sciatic nerves, as well as elevated CD3+ and CD20+ (B cell marker) cells counts in the liver. Importantly, none of the tested doses induced inflammation in the lumbar spinal roots, DRGs, brain, lung, kidneys, quadriceps, or heart (Figures S15–S17). Histopathological analysis from blinded third-party pathologists revealed dose-dependent hepatic alterations, including oval cell hyperplasia, hepatocellular necrosis, and extramedullary hematopoiesis 6 weeks after injection but were absent at later time points (4 and 6 months post-injection) (Figures S10–S12). These findings align with previous reports demonstrating a transient inflammatory response in the liver 6 weeks post-vector injection, which resolves by 4 months post-injection.[10] The same third-party pathologists detected no significant pathological lesions in the brain, spinal cord, or peripheral nerves (Figure S18). Collectively, these results suggest 5E11vg/animal is the optimal dose and maximum tolerated dose for efficient PNS transduction and *PMP22/Pmp22* silencing in CMT1A mice, achieving therapeutic efficacy with minimal immune activation.

Single-cell analysis of miR871 and *PMP22/Pmp22* transcript levels in PNS tissues of treated CMT1A mice

To this point in the mouse studies, we assessed gene knockdown using various molecular methods that relied upon bulk tissue collection. Thus, gene silencing values represent the average among all Schwann cells in a nerve, without consideration for differential AAV transduction, or lack thereof. We therefore sought to assess the potential that differential AAV transduction could produce different types of treated Schwann cells: those with too much PMP22 silencing (>50% remaining PMP22); those with little or no silencing (∼150%); and normalized cells with PMP22 levels hovering around 100%. To assess the uniformity of gene silencing and *PMP22* expression on a cell-to-cell basis, we developed a customized RNAscope Plus small-RNA-RNA assay in collaboration with an independent contract research organization, to simultaneously detect the mature miR871 alongside human *PMP22* and mouse *Pmp22* mRNAs in the PNS tissues of CMT1A animals treated with 5E11 or 1E12 vg/animal of AAV9.U6.miR871. PBS-injected animals served as controls (Figures 4A–4E and S19–S21). CMT1A animals were treated at 2 months of age and analyzed 6 weeks later. Single-cell resolution quantitative analysis was conducted using HALO software. miR871 was detected in 60.38% ± 13.68% of sciatic nerve cells in CMT1A mice treated with 5E11 vg/animal and in 69.28% ± 9.07% of cells treated with 1E12 vg/animal (Figure 4D). These numbers correspond to an average of 3.48 ± 1.31 and 4.56 ± 1.44 dots/cell for the 5E11 and 1E12 vg/animal treatments, respectively (Figure 4E). Minimal miR871 background staining was observed in PBS-treated controls (Figures 4A–4E).

Target transcripts in the sciatic nerve were measured at 49.07 ± 10.40 human *PMP22* and 59.53 ± 8.77 mouse *Pmp22* dots/cell in PBS-treated animals (Figure 4E). These scores significantly decreased to 34.77 ± 5.99 hu*PMP22* and 53.70 ± 7.48 mu*Pmp22* dots/cell following treatment with AAV9.U6.miR871 at 5E11 vg/animal and 27.47 ± 4.75 hu*PMP22* and 45.30 ± 5.31 mu*Pmp22* dots/cell after treatment AAV9.U6.miR871 at 1E12 vg/animal (Figures 4D and 4E). Overall, the total percentage of positive cells for both human and mouse *PMP22/Pmp22* did not significantly change in response to treatment (Figure 4D); however, the abundance (dots/cell) of PMP22/Pmp22 was significantly reduced (Figure 4E). This suggests relatively uniform reduction without total elimination of PMP22 at the single-cell level.

Similar effects were observed in spinal roots and femoral nerves (Figures S19–S21). These findings at the single-cell resolution level confirm that AAV9-mediated miR871 delivery effectively reduces PMP22 expression in the large majority of PNS cells without complete gene knockdown, reinforcing its therapeutic potential for CMT1A while avoiding producing HNPP-like phenotypes (Figure 4E).

Intrathecal delivery of AAV9.U6.miR871 to non-human primates

CMT1A therapies will require normalizing PMP22 levels in distal nerve Schwann cells. To accomplish this in mice, we used lumbar intrathecal (IT) administration to deposit AAV into the cerebrospinal fluid (CSF), which then flows to the PNS, carrying AAV particles with it.[32,33] Importantly, IT delivery of AAV9.U6.miR871 normalized PMP22 levels and significantly improved several neuropathic outcomes in C61 mice.[10] However, humans and mice have obvious size differences, and no published data had yet assessed if IT-delivered AAV9 vectors could travel the greater distances required to reach distal nerves in humans. For example, the sciatic nerve may exceed 1 meter in humans but is only ∼22 mm long in mice.[34] To approximate the ability of AAV9 to transduce Schwann cells of the PNS in humans, we delivered AAV9.U6.miR871 vectors to cynomolgus macaques using lumbar intrathecal administration and assessed biodistribution, safety/toxicology, and *PMP22* mRNA and PMP22 protein target engagement in four different CMT1A-relevant nerves (two upper limb, two lower limb) using two different vector doses and two time points. All NHP injections, in-life assessments, tissue collection, biodistribution, and *PMP22* mRNA levels were performed at a contract research organization (CRO, Amplify Bio, Columbus, OH, USA) while protein target engagement assessments and semithin morphological analysis of peripheral nerves were conducted by scientists in the sponsor laboratories. To perform this experiment, 20 wild-type monkeys (10 males, 10 females) were randomly assigned to one of three dose groups (control, formulation buffer, N = 4; low-dose [6E13 total vg] and high-dose [1.2E14 total vg] AAV9.U6.miR871) (N = 8 animals per group). We chose low and high doses in NHPs based on the minimally effective dose (MED)

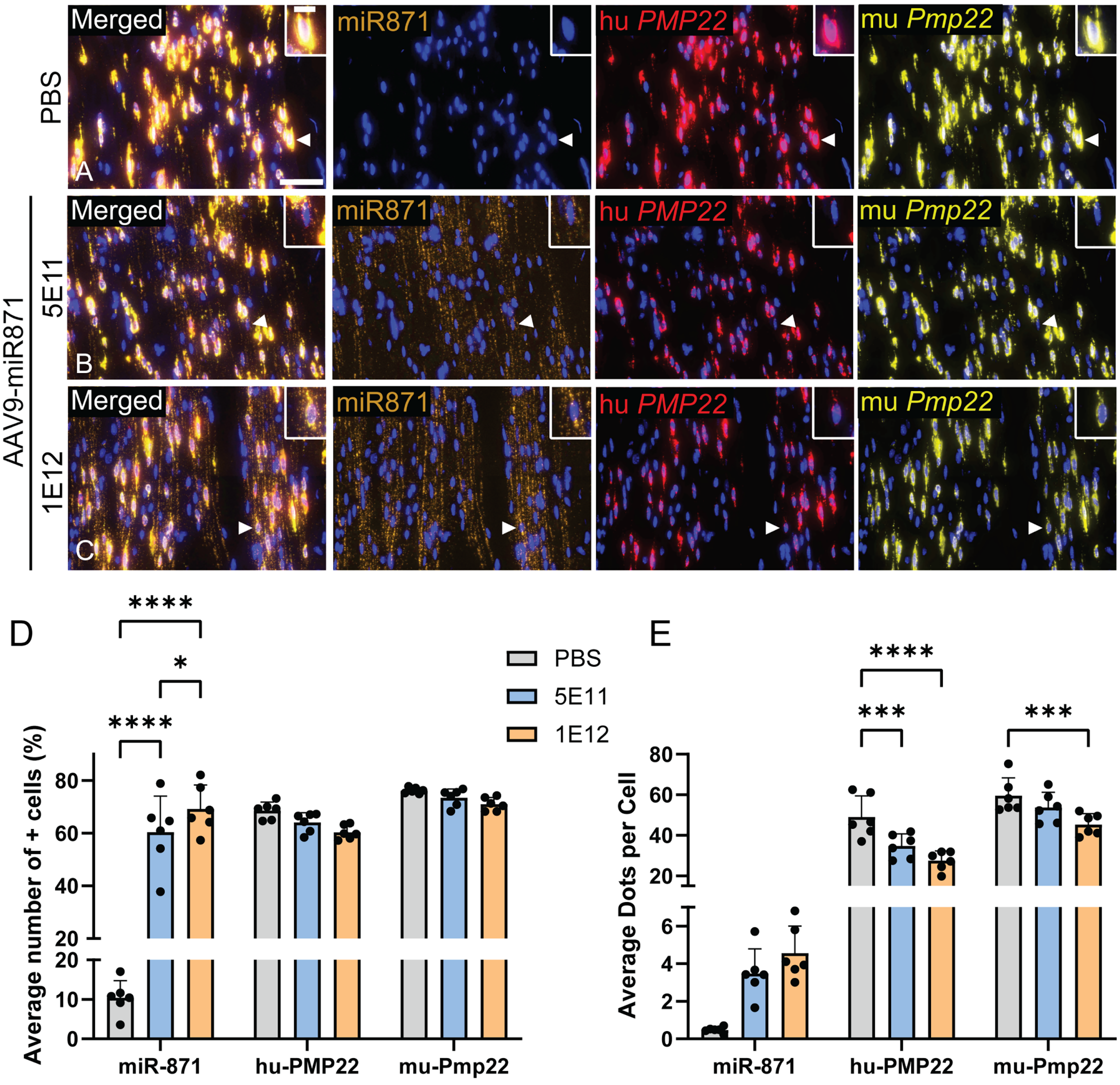

Figure 4. RNAscope Plus analysis for the co-detection of miR871, hu*PMP22*, and mu*Pmp22* in the sciatic nerves of CMT1A mice treated with AAV9.U6.miR871

(A–C) Representative images of miR871, hu*PMP22*, and mu*Pmp22* staining in the sciatic nerves of CMT1A mice treated with 5E11 or 1E12 vg/animal of AAV9.U6.miR871 (n = 6 animals/group). (D and E) Quantitative image analysis was performed using HALO software and the average number of miR871-, hu*PMP22*-, and mu*Pmp22*-positive cells ($n \geq$ 147,000 cells/group), as well the average number of corresponding miR871, hu*PMP22*, and mu*Pmp22* dots/cell, reflecting expression at the single cell level, in CMT1A animals treated with AAV9.U6.miR871 (5E11 or 1E12 vg/animal) or PBS. Results in (E) demonstrate that PMP22 reductions do not lead to oversilencing on a cell-by-cell basis. Values are presented as mean ± SD. *$p < 0.05$, **$p < 0.01$, ***$p < 0.001$, ****$p \leq 0.0001$ by one-way ANOVA with Tukey's multiple-comparison test. Scale bars: 50 μm and 15 μm (enlarged inserts).

and fully efficacious doses (FED) identified in CMT1A mice (2E11 total vg and 5E11 total vg, respectively) and scaled 371-fold for the increased CSF volume in monkeys compared to mice (Figures 2 and 3; Table S3). Vector or formulation buffers were administered via catheter, implanted pre-study, as a single 4-mL intrathecal infusion over a 6-h period to avoid increasing intracranial pressure. Animals were divided into 6-week and 12-week cohorts for bilateral tissue collection and outcome measure assessment (Table S4; Figure S22).

IT of AAV9.U6.miR871 shows a strong safety profile in NHPs

Clinical assessments were performed by an independent, third-party veterinary team at Amplify Bio. Endpoints evaluated either in-life or at 6- or 12-week necropsies included clinical observations, body weights, food consumption, neurological observations, nerve conduction velocity (NCV), ophthalmic examinations, electrocardiogram (ECG) evaluations, clinical pathology (hematology, coagulation, serum chemistry, and urinalysis), troponin I analysis, cytokine analysis (ELISpot), and anatomic pathology, including

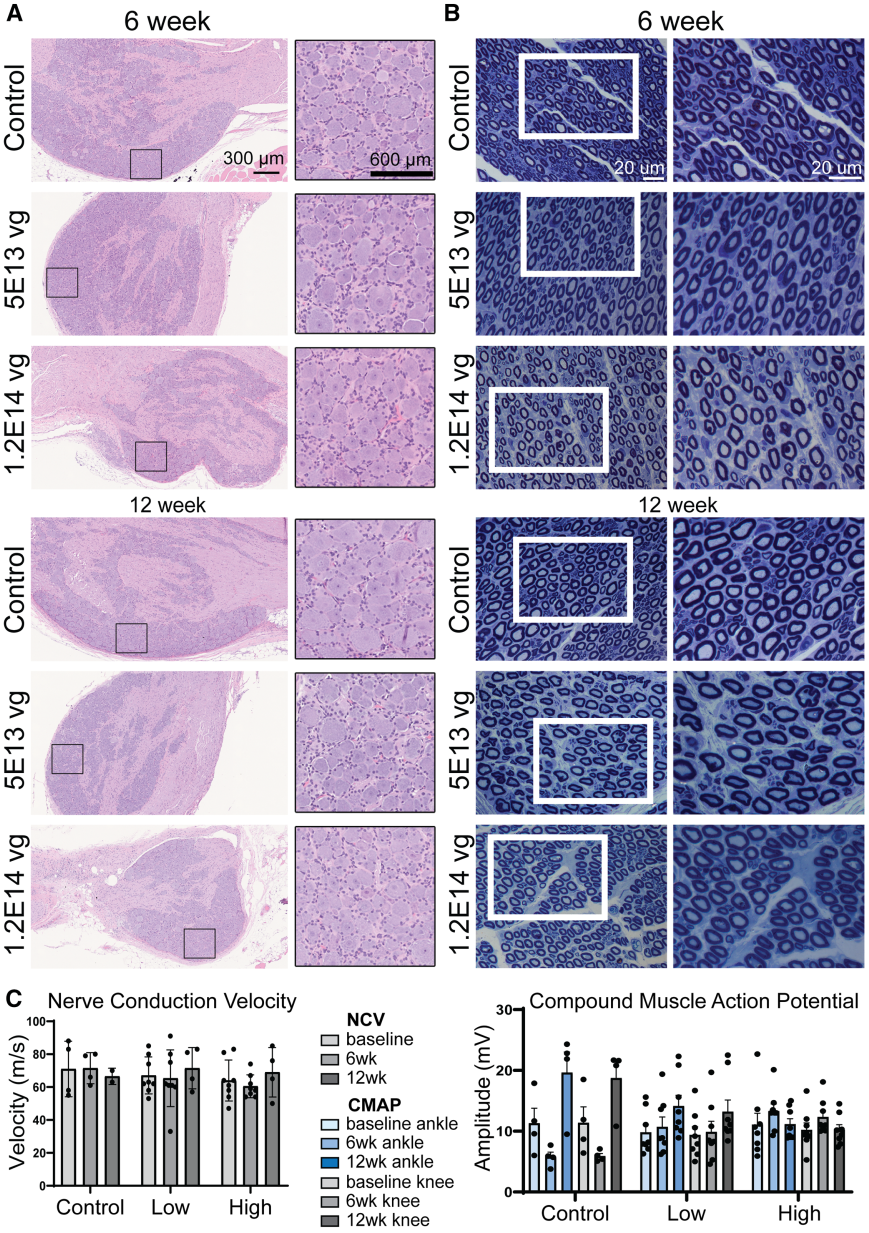

Figure 5. Safety of AAV9.U6.miR871 delivery in NHPs

Histology and electrophysiology support safety of lumbar intrathecal AAV9.U6.miR871 delivery in non-human primates. (A) H&E staining of representative dorsal root ganglia (DRG) from miR871-treated animals show no significant histological abnormalities compared to control. Black boxes in left panel are shown at high power in the right panels. Scale bars: 300 mm and 600 mm. (B) Toluidine-blue-stained semithin sections of sciatic nerves from animals treated with AAV9.U6.miR871 are shown at low (left panels) and high magnifications (right panels). White boxes indicate the area pictured in the high magnification panel. Scale bars: 20 μm. (C) Electrophysiological analyses, including nerve conduction velocity (NCV, left) and compound muscle action potential (CMAP, right), were analyzed at baseline and again at 6 and 12 weeks post-treatment. No significant changes in NCV or CMAP were detected post-treatment. Data analyzed using two-way ANOVA with Tukey's multiple comparisons test. Values are presented as means ± SD.

organ weights, gross observations at necropsy, and microscopic evaluation of selected tissues (Figures 5 and S23). All animals completed the in-life study portion without incident, and there were no adverse clinical observations attributed to the test article. Specifically, no animals showed abnormalities in body weight, food consumption, echocardiogram waveforms, neurobehavioral assessments, hematology, coagulation, serum chemistry, or urinalysis metrics. Moreover, none showed cardiac troponin I (cTnI), indicating absence of myocardial injury. No nerve dysmyelination or gross lesions in any organ were noted at 6 weeks; however, one high-dose female showed enlarged lymph nodes with increased cellularity at 12 weeks, the relevance of which was unclear (Figures 5, S23, and S24–S26). Forty percent of animals showed subclinical, minimal-to-mild lesions in the dorsal root ganglia, which were also found in two saline-treated animals, suggesting these were unrelated to test article.

AAV9.U6.miR871 biodistribution in NHPs following lumbar IT delivery

We used dPCR to assess AAV biodistribution as a function of time post-injection, vector dose administered, and distance from the delivery site. We detected AAV genomes above background in all peripheral nerves both 6- and 12-weeks after injection, with no AAV DNA in saline-treated nerves (Figure 6). Our data support durability of AAV transduction, although AAV genomes did decrease roughly 3× to 5× between 6 and 12 weeks (average 1.1 vector genome copies per cell vs. average 0.3 copies per cell). While 5 of 16 high-dose samples showed more AAV genomes than low-dose counterparts, differences were not statistically significant and overall, we found no evidence of a uniform dose-response. Finally, we hypothesized that nerve tissues closer to the injection site would contain greater levels of AAV genomes than distal sections, and to assess this possibility, we measured AAV genomes in proximal and distal samples from the same monkey nerves (Figure S22 shows nerve tissue collection). We were pleased to find AAV genomes in distal nerve sections as far as ~14 cm from the injection site (the furthest section taken in the distal sciatic nerve), which is 630% farther than the distal end of an average mouse sciatic nerve. Moreover, we found no evidence

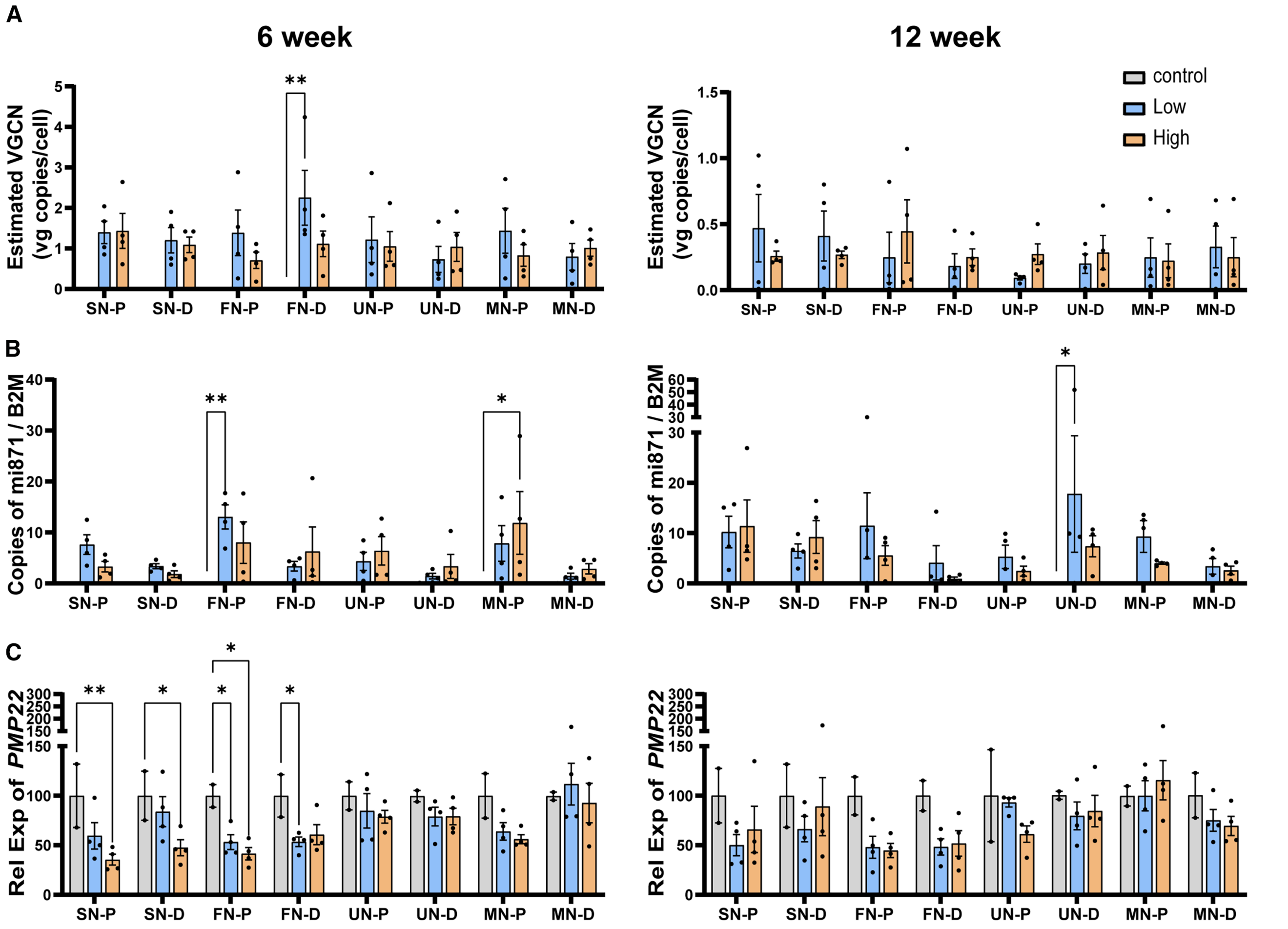

Figure 6. Widespread AAV biodistribution in NHP nerves following lumbar intrathecal delivery leads to PMP22 silencing in NHPs

(A) AAV vector genomes were detected in both the proximal and distal portions of all NHP nerves at both time points tested. Vector genomes were assessed by dPCR using a probe to the AAV2 ITR. Data were analyzed as vector copies normalized to host diploid genomes. While all AAV-treated samples showed detectable AAV genomes above PBS-treated controls, there were no significant differences between low- and high-dose groups except in the distal femoral nerve (FN-D). Together, these results indicate a lack of dose-response on AAV biodistribution to peripheral nerves in NHPs. (B) RT-dPCR detection of mature miR871 transcript at 6 weeks (left) and 12 weeks (right) indicated biodistribution and miR871 RNA accumulation in all nerve tissues at low (6E13) and high (1.2E14) doses. Absolute copies of mature miR871 were normalized to the housekeeping gene, *beta-2-mocroglobulin* (*B2M*). (C) The *PMP22* transcript was reduced at 6 (left) and 12 weeks (right) in most AAV9.U6.miR871-treated nerve segments, with significant reductions indicated by asterisks. Monkey PMP22 mRNA was quantified by RT-dPCR, normalized to B2M, and then values plotted as relative to saline controls for each nerve segment, which we considered to represent normal PMP22 levels (100%). Data are displayed as means with error bars representing SEM. $n = 2$ control or 4 treated animals per dosing group. Each point represents a single animal's averaged samples per tissue designation including two segments from the right nerve and two from the left nerve. Two-way ANOVA shows a significant treatment effect ($p < 0.0001$) at both time points. Tukey's multiple comparison tests represented as $^{*}p < 0.05$, $^{**}p < 0.01$, $^{***}p < 0.001$, and $^{****}p < 0.0001$. SN, sciatic nerve; FN, femoral nerve; UN, ulnar nerve; MN, median nerve; P, proximal; D, distal.

of nerve length impacting transduction, as there were no significant differences in AAV genomes present at the proximal and distal ends of the same nerves at either time point (Figure 6). We also found AAV9 genomes present in non-nerve tissue, with the highest levels detected in the liver, suggesting the vector escapes the CSF and enters the circulatory or lymphatic systems (Figure S23). Overall, our data support that AAV9 vectors transduce proximal and distal ends of CMT1A-relevant peripheral nerves in the upper and lower limbs of monkeys and suggest a similar approach could be translated to humans.

PMP22 target engagement in AAV9.U6.miR871-treated NHPs

Our biodistribution data demonstrated distal nerve transduction by AAV9 in four different monkey nerves (sciatic, femoral, ulnar, and median); however, these results were produced from a total lysate of each nerve portion and do not indicate whether AAV9 entered Schwann cells, axons, or both. However, PMP22 is primarily expressed in Schwann cells, so we reasoned that reduction of *PMP22* mRNA and PMP22 protein levels would confirm that AAV9.U6.miR871 indeed transduced Schwann cells and demonstrate *in vivo* efficacy in a large animal model. To do this at the

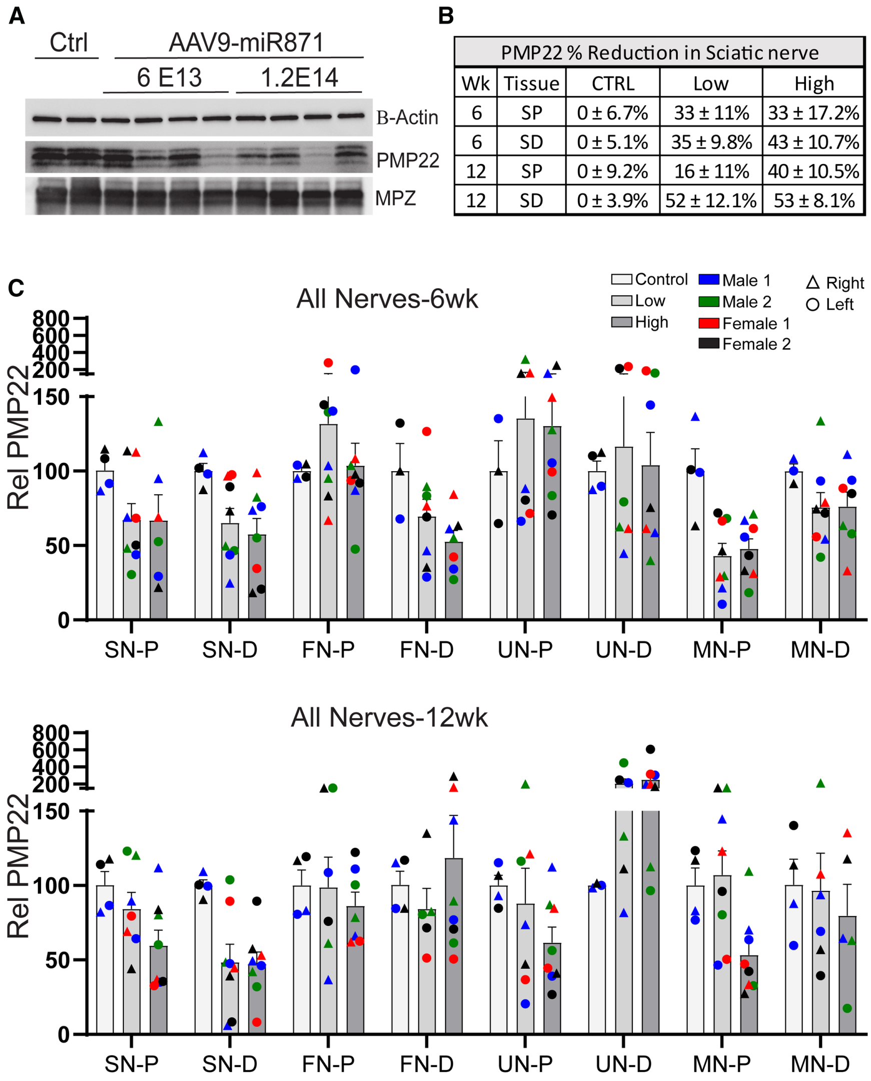

B

PMP22 % Reduction in Sciatic nerve				
Wk	Tissue	CTRL	Low	High
6	SP	0 ± 6.7%	33 ± 11%	33 ± 17.2%
6	SD	0 ± 5.1%	35 ± 9.8%	43 ± 10.7%
12	SP	0 ± 9.2%	16 ± 11%	40 ± 10.5%
12	SD	0 ± 3.9%	52 ± 12.1%	53 ± 8.1%

Figure 7. Reduction of PMP22 protein demonstrates target engagement of miR871 in NHPs

(A) Representative western blots using extracts from one side of the 12-week sciatic distal nerves showed PMP22 protein reduction in treated NHP nerves while MPZ was unaffected. Beta actin served as the loading control. (B) Quantification of all sciatic tissues demonstrated an average reduction of 16%–53% from normal PMP22 levels. (C) Similar PMP22 reduction is evident across all time points in both the upper and lower extremity nerves. Data are displayed as means with error bars representing SEM. n = 4–8 nerve segments per tissue designation from either two control or four treated animals per dosing group. One segment from the right nerve and one from the left nerve were analyzed per animal. SN = sciatic nerve, FN = femoral nerve, UN = ulnar nerve, MN = median nerve, P = proximal, D = distal. * No sig except 12wk UN-D *.Two-way ANOVA with Tukey's multiple comparison tests represented as *p < 0.05, **p < 0.01, ***p < 0.001, and ****p < 0.0001.

transcript level, we collected bilateral, proximal, and distal portions of each sciatic, femoral, ulnar, and median nerve, generated cDNA by reverse transcription, and performed dPCR to quantify levels of mature miR871 and monkey *PMP22* mRNA compared to controls. Similar to our AAV DNA biodistribution results, we measured miR871 expression in every peripheral nerve, while saline-treated animals showed none (Figure 6). In addition, average *PMP22* mRNA expression was significantly lower or trended lower in all miR871-treated nerves except the median distal at 6 weeks and median proximal at 12 weeks. Collectively, these data support successful *PMP22* target engagement by miR871 up to 3 months after intrathecal AAV9.U6.miR871 delivery (Figure 6).

We designed miR871 to base pair with mouse, monkey, and human *PMP22/Pmp22* mRNA across a conserved 22 nucleotide stretch of sequence, favoring a RNAi mechanism involving *PMP22* mRNA degradation, which is detectable using the dPCR approach we used here (Figure 6). Nevertheless, mechanistically RNAi can also silence transcripts by a translational inhibition mechanism that does not result in detectable loss of *PMP22* mRNA. Thus, to assess PMP22 gene silencing using an additional method, we measured PMP22 protein expression by western blot using protein lysates from proximal and distal portions of the indicated nerves. We measured reduced PMP22 protein levels in five of eight nerve samples at both 6- and 12-weeks post-injection (Figures 7 and S27), including significant average reductions of up to 53% in the distal sciatic nerves (Figure 7). While we noted some variability in *PMP22* RNA and PMP22 protein levels in different nerves, overall our biodistribution, RNA, and protein data suggest that AAV9-delivered miR871 can broadly silence PMP22 in multiple peripheral nerves of a large animal model (Figure S28).

DISCUSSION

Our study demonstrates for the first time the safety and scale up potential of lumbar intrathecal AAV vector gene therapy to treat demyelinating peripheral neuropathies. In particular, we show both in rodents and NHPs efficient vector biodistribution to the PNS and target engagement in myelinating Schwann cells with selective silencing of *PMP22*, the gene associated with the most common form of CMT neuropathies. Our overall goal is to translate this approach to human clinical trials. While clinical use of AAV-based gene therapies in humans is no longer unprecedented, with 6 AAV-based gene therapies approved for use in humans by the United States (US) Food and Drug Administration (FDA) since

2017, each approved therapy is unique and almost completely distinct from the approach we are using for CMT1A. Specifically, the current group of approved AAV-based therapies is diverse, utilizing unique combinations of AAV serotype (AAV2, AAV5, AAV9, and AAVRh74), genetic payload, dose, route of administration, and intended tissue target (liver, eye, brain, muscle/heart, and motor neuron). Two require local, surgical delivery to the eye (Luxturna) or brain (Kebilidi), while the others are delivered intravenously to treat liver (Roctavian, Hemgenix), striated muscle (Elevidys), or spinal motor neurons of the central nervous system (Zolgensma). Nevertheless, each approach shares the common feature of using AAV vectors to treat recessive disorders with gene replacement therapy.

In contrast, our CMT1A gene therapy program is distinct from each approved therapy in that it involves gene knockdown instead of gene replacement and is intended to treat a dominant disease affecting Schwann cells of the peripheral nervous system. In addition, we intend to use lumbar intrathecal administration to the cerebrospinal fluid as route of administration, which although not yet utilized in an approved AAV therapy, has been pioneered in ongoing clinical trials.[35] Nevertheless, Schwann cells are an anatomically difficult target for genetic therapy, as they are abundant throughout the body, forming myelin along the entire length of peripheral nerve myelinated fibers, and well protected by blood-nerve barrier as well as two layers of connective tissue. Importantly, AAV9 vectors can transduce Schwann cells of the peripheral nervous system in rodents following lumbar intrathecal delivery, which is a technique we previously used to demonstrate proof of principle for CMT1A RNAi therapy in mice.[10,13] Moreover, AAV9 vectors show a solid clinical safety profile, having been safely delivered to thousands of children with spinal muscular atrophy (SMA) in Zolgensma and are now FDA-approved for delivery via lumbar intrathecal adiministration.[36]

In this study, we generated several new pieces of data to support our goal of translating CMT1A RNAi-based gene therapy to humans. First, we optimized the U6.miR871 AAV genome (Figure S2), and performed a four-step dose-finding study in CMT1A mice (Figures 1, 2, and 3). We found dose-responsive AAV biodistribution and mature miR871 expression in all nerves tested (sciatic, femoral, and spinal roots), with combined mouse *Pmp22* and human *PMP22* mRNA reductions in all treated nerves. Importantly, in CMT1A mice, among the three lowest doses, we also measured dose-responsive improvements in several molecular (decreased neurofilament, increased *Mpz*), histological (improved myelination), and behavioral-functional outcomes, including rotarod performance, hindlimb clasping phenotypes, CMAP scores, and motor nerve conduction velocity (MNCV). In particular, the 5E11 vg dose of AAV.U6.miR871 significantly improved all outcome measures, including completely normalized rotarod performance, clasping, and MNCV deficits in CMT1A mice. However, mice injected with the highest dose (1E12 vg) performed worse than the 5E11-vg-treated animals in all outcome measures, despite showing the highest transduction. While no animals showed morbidity at any AAV9.U6.miR871 dose, these *in vivo* efficacy results suggested potential toxicity at the 1E12 vg dose. We therefore selected 5E11 vg as the fully efficacious dose (FED) and 2E11 vg as the minimally efficacious dose in mice. Importantly, the calculated human equivalent doses (7.4E14 vg and 1.9E15 vg total dose) are in range but still 2–6× higher than doses used in previous intrathecal gene therapy trials, including the recently approved Itvisma, an IT-delivered version of Zolgensma, which is given at a flat dose of 1.2E14 vg per SMA patient (Table S3).[35] Assuming an average 84 kg body weight for an American adult (combined males and females), our equivalent systemic AAV9.U6.miR871 dose would be 8.8E12–2.3E13 vg/kg, which is approximately 10× lower than doses delivered in the FDA-approved products Zolgensma or Elevidys. We do not anticipate manufacturing this quantity of vector will be a barrier to translation, as a typical 500 L suspension process produces 1E17 total vector genomes per batch, equivalent to ∼52–135 doses per manufacturing run.

We also assessed the safety of AAV9.U6.miR871 gene therapy using five different studies, and our results support a strong safety profile molecularly and in small and large animal models. First, in human Schwann cells, neurons, and hepatocytes, we found no significant sequence-specific off-targets directly caused by miR871 binding in over 48,000 transcripts sampled. In contrast, all cells treated with miR871 had significantly reduced *PMP22* expression. Together, these results support high on-target specificity of miR871 in human cells (Table 1). In mouse and monkey safety and toxicology studies using MED and FED, all animals completed the in-life portions of each study, and independent contract pathologists concluded that overall there were no significant adverse events related to the AAV9.U6.miR871 vector in either species, except some subclinical, dose-dependent, focal liver inflammation in mice that did not result in AST or ALT elevations (Figures S13, S18, and S23). We do note that we did not measure AST/ALT elevations immediately after vector delivery and could have missed transient increases in mice. We also assessed potential AAV9-mediated DRG inflammation in mice and monkeys. In CMT1A mice, the lowest three doses of AAV9.U6.miR871 (1E11, 2E11, and 5E11 vg) reduced pre-existing DRG inflammation rather than trigger it, except at the highest dose (1E12 vg), which we believe is toxic. Indeed, the 1E12 vg dose also caused inflammation in sciatic nerves and liver. In monkeys, some vector-treated animals showed subclinical minimal-to-mild DRG inflammation, as did saline-treated animals, suggesting the lesions were unrelated to the AAV9.U6.miR871 product. We therefore conclude that at doses representing the FED and below, AAV9.U6.miR871 causes no DRG toxicity and, extrapolating mouse data, could instead reduce CMT1A-related inflammation should it exist in humans with CMT1A.

A final issue related to safety pertains to the so-called “Goldilocks zone” of *PMP22* expression required to support normal myelination in Schwann cells. Specifically, while *PMP22* gene duplication causes CMT1A, heterozygous *PMP22* gene deletion leads to another, albeit less severe, neuropathy called hereditary neuropathy with liability to pressure palsies (HNPP). Our molecular and biochemical assessment of *PMP22* knockdown using various AAV9.miR871 doses in mice

and monkeys suggests our approach would serve to normalize PMP22 to healthy levels in most nerves, with little to no risk of *PMP22* oversilencing that would convert a CMT1A disorder to HNPP. For example, low-dose AAV9.miR871 injection in normal monkeys (i.e., starting at 100% PMP22 levels, not 150% as would be the case in humans with CMT1A) reduced PMP22 protein by ∼38% on average in sciatic nerves (Figure 7). Extrapolating this value to a human CMT1A sciatic nerve would produce PMP22 protein levels at 93% of normal, which we predict would improve myelination overall. Nevertheless, humans with CMT1A do not always express 1.5× PMP22 levels, but may exceed PMP22 levels by more than 2-fold.[37] PMP22 levels can be assessed in nerve endings from skin biopsies, and it may be possible to assess PMP22 overexpression in CMT1A patients prior to treatment to better understand starting levels and inform dosing. We note that our molecular and biochemical assessments of *PMP22* knockdown (e.g., ddPCR and western blot) utilized materials harvested from bulk tissue collections, and we were concerned that these methods were insufficient to understand the cell-to-cell uniformity of *PMP22* silencing. Specifically, it is likely that some Schwann cells may be more highly transduced by AAV than others, so it was theoretically possible that a 38% average PMP22 reduction from bulk materials could at least partly arise from *PMP22* oversilencing in some cells, and no silencing or diminished silencing in others, thereby creating a mixed population of Schwann cells with CMT1A, HNPP, or normal phenotypes. We therefore performed RNAscope on injected mouse nerves to assess the uniformity of miR871 and PMP22 expression on a cell-to-cell basis *in vivo*. We found no evidence of *PMP22/Pmp22* oversilencing in individual mouse Schwann cells *in vivo*. The total number of *PMP22/Pmp22*-expressing cells did not change with miR871 expression, while the total abundance of transcripts per cell was reduced in a dose-responsive fashion. Our data therefore do not support potentially deleterious oversilencing in individual Schwann cells, thereby confirming the relevance for treating CMT1A (Figure 4). At the same time, both the RNAscope data as well as lack of focal myelin pathology or nerve conduction disturbance in mice and NHPs (Figures 2, 3, 4B, and 4C) provide reassurance that this treatment is unlikely to cause HNPP-like phenotypes. Indeed, in our previous work, we intentionally tried to produce HNPP-like phenotypes by knocking down Pmp22 in wild-type mice containing 100% Pmp22 levels (i.e., normal) instead of ∼150%+ seen in CMT1A models.[10] Up to 80% knockdown of Pmp22 in wild-type mice treated with AAV.miR871 produced no nerve conduction velocity deficits or focal myelin thickening (tomacula) characteristic of HNPP, although some animals had partially impaired motor performance.[10] Nevertheless, RNAi therapy would never be delivered to a human without elevated PMP22, and we note that AAV.miR871 injection improved or normalized all phenotypes tested in CMT1A mice. Our data therefore support the conclusion that *PMP22* oversilencing is unlikely, and even if it occurred, does not cause HNPP-like pathology in mice or monkeys.

Our promising results from mouse dose-finding and safety studies support further translation of the approach, but application in humans with CMT1A would pose an additional anatomical challenge due to nerve length. Specifically, although intrathecal gene therapy has been previously tested in humans, no one had yet assessed the ability of an AAV9 vector to transduce Schwann cells of the longest distal nerves in humans. This is a critical question for translation of this approach, because if the vector cannot reach and transduce Schwann cells of the distal nerves, the therapy will simply not work. We tested biodistribution in monkeys as a best approximation of human nerves and indeed measured AAV vector genomes, as well as miR871 expression, in the proximal and distal ends of four different monkey nerves both 6 and 12 weeks after lumbar IT injection. Saline-treated animals showed neither vector nor mir871 expression. Overall, we found relatively uniform vector genomes at proximal and distal ends of all four nerves at the 6-week time point, with ∼3–5× reductions in AAV genomes at the 12-week time point. Despite the loss of vector genomes, average miR871 expression remained relatively constant with time (low dose, 5.7 copies miR871 vs. 8.4 copies between 6 and 12 weeks; high dose, 5.9 copies miR871 vs. 5.4 copies between 6 and 12 weeks). Overall, we found no evidence for a dose-response in monkey nerves, with no significant differences in vector genomes or miR871 based on dose (Figure 6). Upon initial consideration, the loss of vector genomes over time and maintenance of gene expression we saw here seem discordant but are consistent with previous data in NHPs. For example, in a study delivering β-choriogonadotropic (β-hCG) hormone to NHP livers using AAV8 and AAV.Rh10, AAV genomes and β-hCG mRNA significantly decreased over time with no liver enzyme elevations (suggesting lack of toxicity), but lower RNA levels still produced consistent β-hCG expression after an initial drop and remained stable between 3 and 6 months after gene delivery.[38] The authors of this study suggested a two-phase expression profile arising from AAV delivery, with short-lived expression from episomal genomes followed by lower stable expression, possibly from integrated vectors.[38] We did not explore integration in this study, but we do note that we saw no evidence of vector toxicity at the FED and below in mice and monkeys and that non-diseased, mature Schwann cells are post-mitotic while hepatocytes are not. Thus, it is likely that AAV genome loss was not related to any toxic effects of vector and that AAV genome stability and miR871 expression in monkey nerves could still arise from episomal DNAs because transduced Schwann cells are not dividing. Nevertheless, we propose that inefficient AAV trafficking could explain the disparity between stable miR871 expression and reduction in AAV genomes between 6 and 12 weeks. Specifically, AAV vectors enter cells through receptor-mediated endocytosis and need to be shuttled to the nucleus to enable payload transcription. Following cell entry, most AAV genomes (∼90%–99%) remain stuck in endosomes or Golgi bodies and never reach the nucleus to direct therapeutic gene expression. Thus, it is possible that the small percentage of nuclear-resident genomes remain relatively constant, while the AAV genomes detected at earlier time points represent genomes that were unable to escape the endosomal compartments and are eventually degraded over time by the lysosomes. Our data suggest AAV9.miR871 would produce stable expression in Schwann cells over time, which is important for the

long-term prospects of this therapy, since AAV vector re-dosing is not currently possible.

Finally on this topic, we noted unexpectedly disparate results in the NHP distal ulnar nerve samples, where we measured more ∼2× miR871 in low-dose-treated animals compared to high, despite having ∼2× fewer AAV genomes in the low-dose tissues. We cannot rule out that some variability seen in our results could be attributed to sample collection error and/or gene delivery rather than intrinsic differences between animals, similar to previous reports using intrathecal AAV delivery to monkey CSF.[39,40] Surprisingly, we found no evidence for dose response in vector biodistribution or PMP22 knockdown in monkeys for unclear reasons. One possibility is the PNS has a limited capacity or size restriction to allow free flow of AAV vectors. A recent study suggested the mouse PNS has a size restriction of <15 nm for free flow of particles through the CSF,[32] and yet 25 nm AAV vectors were still able to transduce distal nerves and significantly improve disease phenotypes. Although the aperture of CSF flow in the monkey PNS is not currently known, it is possible that AAV flow to the distal nerves occurs through the endoneurial fluid, but also systemically, as our data are consistent with previously published reports showing AAV vector escape to the peripheral organs following CSF delivery, as indicated by high amounts of liver transduction, for example (Figure S24).[38,41] Once AAV9 leaves the IT space and reaches the circulation, some portion of the dose administered would likely have additional opportunities to recirculate and transduce Schwann cells systemically. Nevertheless, our biodistribution data in monkeys showed AAV vector biodistribution, miR871 expression, and *PMP22* transcript or PMP22 protein knockdown in distal and proximal ends of all four nerves tested. Collectively, our results demonstrate that AAV vectors can deliver a disease-modifying effect (PMP22 reduction) to Schwann cells of the distal PNS in a primate model and will hopefully set the stage to test the approach in clinical trials.

MATERIALS AND METHODS

Second-generation miR871 construct

The U6.miR871 cassette was subcloned into a self-complementary AAV backbone containing a portion of a human collagen intron as stuffer sequence. *In vitro* potency comparing first- and second-generation constructs was assessed using the dual luciferase reporter plasmid modified from Psicheck2 (Promega) as previously described.[26] Self-complementary viral vectors for mouse and NHP studies were produced by Andelyn Biosciences (Columbus, Ohio, USA) using triple transfection into HEK293 cells of pro-viral plasmids, pHELPER (containing adenoviral helper genes), and a plasmid containing AAV2 rep and AAV9 cap genes. Vectors were isolated by iodixanol gradient ultracentrifugation followed by FPLC purification and buffer exchange with TMN200. All vectors were then titrated by ddPCR using primer/probes to detect the AAV2 ITR.

On-/off-target RNA-seq analysis

Three different lentiviral vectors produced at Genewiz (Azenta Life Sciences) were used to transduce four different human cell lines. Specifically, VSV-G pseudotyped lentiviral particles carrying either (1) U6.miR871 and a Puromycin resistance gene ($Puro^R$); (2) U6.miLacZ and $Puro^R$; or (3) $Puro^R$ alone were used to transduce human hepatocytes (THLE3; ATCC CRL-3585), neurons (SH-SY5Y; ATCC CRL-2266), or Schwann cells (hTERT ipn02.3 2l; ATCC CRL-3392). Each cell line was handled and cultured following manufacturer's recommended conditions and transduced in triplicate. Transductions were performed in 12-well plates using TransDux Max Lentivirus Transduction Reagent (System Biosciences, LV860A-1) following the manufacturer's protocol (day 1). The following MOIs were used for each cell line: Schwann cells MOI = 100, THLE-3 MOI = 20, and SH-SY5Y MOI = 5. On day 2, the virus and media were removed and replaced with fresh culture media. Puromycin selection began on day 3 at a concentration of 2.0 μg/mL (except for SH-SY5Y cells wherein puromycin concentration was 3.0 μg/mL). Selection media was replaced on day 5 and cells harvested for RNA isolation on day 7. RNA was extracted using MirVana miRNA Isolation Kit (Ambion: AM1561) and DNAse-treated using DNA-Free DNA Removal Kit (Ambion: AM1906), before submitting for RNA-seq. Samples were analyzed using an Illumina HiSeq platform, with paired end reads of 150 base pairs (HiSeq 2× 150 bp). A total of 36 samples were analyzed, with an average depth of 19.5 million reads and mean data quality score of 35.2, which indicates very high-quality sequencing reads. Differential gene expression analysis was performed using the Wald test as implemented in the DESeq2 R package (R version: 4.4.2). Genes with an absolute log2 fold change ≥1, a p value <0.05, and an FDR < 0.05 were considered statistically significant. RNA-seq data will be available following publication.

Molecular beacon-binding assay

The molecular beacon-binding assay was performed following the previously described procedures with some modifications based on target sequence (Figure S1).[26] In this assay, the binding was performed using the QuantStudio 6 Flex (Applied Biosystems, Thermo Fisher Scientific). Mi405 negative control target site (neg. ctrl TS) was used as the non-specific negative control target site (ncTS) sequence.

CMT1A model mouse study design

The goal of this study was to conduct a dose escalation analysis to evaluate the off-target effects, biodistribution, safety, and therapeutic efficacy of AAV9.U6.miR871.CMV.EGFP/AAV9.U6.miR871 vectors in improving neuropathy in C61-het mice, a model of CMT1A.[4] The first stage of *in vivo* dose escalation testing was performed in CMT1A mice. For this purpose, 2-month-old (2 mo) CMT1A mice were intrathecally injected with different doses of the AAV9.U6.miR871.CMV.EGFP vector (1E11, 2E11, 5E11, and 1E12 vg/animal) and analyzed 6 weeks post-injection using VGCN analysis (n = 5/group), RT-qPCR (n = 4/group), western blot (n = 4/group), and immunohistochemistry (n = 5/group). Using a second-generation vector exclusively expressing miR871 without CMV.EGFP (AAV9.U6.miR871), 2 mo CMT1A mice were intrathecally injected with 5E11 or 1E12 vg/animal of AAV9.U6.miR871. Six weeks post-injection, tissues were collected and analyzed with

ddPCR (n = 4/group) and RNAscope Plus (n = 6/group). For therapeutic efficacy trials, 2 mo CMT1A mice were intrathecally injected with different doses of AAV9.U6.miR871 (1E11, 2E11, 5E11, and 1E12 vg/animal) or 2E11 vg/animal of a control vector (AAV9.U6.miRLacZ). Motor performance was assessed every 2 months via behavioral testing, up to 4 months post-injection. At the final time point of each treatment group, mice underwent nerve electrophysiology testing measuring MNCV and CMAP, quantification of NF-L as a circulating biomarker of axonal degeneration, and morphological evaluation of PNS tissues using semithin section analysis. For safety assessments, 2-month-old CMT1A mice were intrathecally injected with different doses of AAV9.U6.miR871 (1E11, 2E11, 5E11, and 1E12 vg/animal) and analyzed 6 weeks, 4 months, or 6 months later with hematology tests and hematoxylin and eosin (H&E) staining.

Experimental mice handling

All experimental procedures in this study were conducted in accordance with animal care protocols approved by the Cyprus Government's Chief Veterinary Officer (project license CY/EXP/PR.L11/2022) according to national law, which is harmonized with EU guidelines (EC Directive 86/609/EEC). All inoculations were performed under anesthesia, and all efforts were made to minimize animal suffering. The protocols were approved by Cyprus Government's Chief Veterinary Officer. In this study, we used adult WT C57BL/6 or C61 Het mice. The C61 Het colony was established from two breeding pairs gifted by Prof R. Martini (Universitäts-Klinikum Würzburg, Germany). C61 Het mice carry four copies of human *PMP22* along with normal endogenous murine *Pmp22*.[4] Gene therapy trials were conducted on 2-month-old C61 Het mice and analyzed 6 weeks, 4 months, or 6 months post-injection. Mice were housed in open-top system cages in a specific pathogen-free animal facility. Up to five mice were housed in cages lined with high-absorbency wood bedding for laboratory mice, dried by high-temperature treatment, sieved, and de-dusted prior to use. Mice received a standard mouse diet and filtered and UV-sterilized potable tap water. Mice were kept in a 12-h dark/12-h light cycle at a temperature of 22°C. Both male and female mice were used and showed no sex-related differences in behavioral performance or nerve pathology.

Lumbar intrathecal injection in mice

Intrathecal injection was performed as described before.[42] Briefly, a small skin incision was made along the lower lumbar spine level of anesthetized mice to visualize the spine, and the AAV vector was delivered into the L5-L6 intervertebral space. A 50-μL Hamilton syringe (Hamilton, 80530/00) connected to a 26-gauge needle (Hamilton, 7758-02/00) was used to inject AAV stocks containing an estimated 1E11, 2E11, 5E11, or 1E12 vector genomes (vg), at a maximum rate of 1 μL/15 sec. A flick of the tail was considered indicative of successful intrathecal administration.

Vector genome copy number determination in treated mice

VGCN was determined as described before.[10] Briefly, genomic DNA was extracted using the MagMAX DNA Multi-Sample Ultra 2.0 Kit (Applied Biosystems, A36570) from mouse PNS, CNS and peripheral tissues (i.e., lumbar roots, sciatic nerve, femoral nerve, brain, liver, kidney, lung, heart, and quadriceps). DNA (5 μL) was used as a template for a duplex droplet digital PCR assay targeting the EGFP gene of the transgene cassette while *transferrin receptor protein 1* (*TFRC*) served as a reference gene. Following droplet generation on a Bio-Rad QX200 AutoDG ddPCR system (Bio-Rad, France), the emulsion was transferred to a PCR plate and cycled using the following thermal cycler conditions: pre-denaturation at 95°C for 5 min, 40 cycles at 95°C for 30 s, 60°C for 1 min with ramp-rate set at 50%, and a final step at 60°C for 10 min. Data acquisition and analysis were performed on a QX200 Droplet Reader and QuantaSoft Software (Bio-Rad). The VGCN was calculated from absolute ddPCR quantification as a ratio of the number of target copies to half the number of reference gene copies.

Droplet digital PCR for miR871 detection in treated mice

The miR871 ddPCR protocol was designed based on methods previously described.[26] Briefly, tissues were lysed using a 5-mm stainless steel bead (Qiagen: 69989) for all nerves or 1.0-mm Zirconia Beads (BioSpec Products: 11079110ZX) for all other tissues, followed by homogenization cycles on a TissueLyser II (Qiagen). RNA was extracted using the total RNA protocol for the mirVana miRNA Isolation Kit (Ambion: AM1560). After DNase treatment (Ambion: AM1906), cDNA was generated using the High-Capacity cDNA Reverse Transcription Kit (Applied Biosystems: 4368813) using a mix of random hexamer primers and 200 nM of the stem-loop forming primer (5' - GTC GTATCCAGTGCAGGGTCCGAGGTATTCGCACTGGATA CGACGGGGAT - 3′). A custom TaqMan assay (Applied Biosystems) including 1.5 mM of forward primer (5' – GCGGCTCTTCAAT CAACAGCA - 3′), 0.7 mM of reverse primer (5' -GTGCAGGGTCC-GAGGT - 3′), and 0.2 mM of miR871 probe (5' - 6FAM- ATACG ACGGGGATTG - 3') was then run using the Bio-Rad AutoDG Droplet Digital PCR System (Bio-Rad: 1864100). *Rpl13* (Mm02526700_g1; Applied Biosystems) served as the reference gene. All data were analyzed using QuantaSoft software (Bio-Rad).

Mouse RNA isolation and quantification using RT-qPCR

RNA was extracted from mouse lumbar roots, sciatic nerves, and femoral nerves using the Qiagen RNeasy Lipid Tissue Mini Kit (Qiagen, 74804) following the manufacturer's protocol from snap-frozen tissues. After DNase treatment (Qiagen, 79254), RNA was quantified by spectrophotometry, and 0.3 μg of RNA was used to synthesize cDNA using TaqMan reverse-transcription reagents (Applied Biosystems, N8080234). Expression of hu*PMP22* (Hs00991884_m1), mu*Pmp22* (Mm01333393_m1), and mu*Mpz* (Mm00485141_g1) mRNA were quantified using TaqMan gene expression assays (Applied Biosystems) and mu*Gapdh* assay (Mm99999915_g1) as an endogenous control.

Immunoblot analysis in treated mice

Fresh lumbar spinal roots, sciatic nerves, and femoral nerves were collected and lysed in ice-cold RIPA buffer (10 mM sodium phosphate, pH 7.0, 150 mM NaCl, 2 mM EDTA, 50 mM sodium fluoride, 1% Nonidet P-40, 1% sodium deoxycholate, and 0.1% SDS) containing a

mixture of protease inhibitors (Roche, 11836170001). Proteins (150 μg) from the lysates were fractionated by 12% SDS/PAGE and then transferred to a PVDF membrane (GE Healthcare Life Sciences, 10600021) using a semidry transfer unit. Nonspecific sites on the membrane were blocked with 5% non-fat milk in PBS with Tween 20 (Sigma, P1379) (PBST) for 1 h at room temperature. Immunoblots were incubated with rabbit antisera hu PMP22 (1:500; Abcam, ab90782), mu/hu PMP22 (1:500; Novus, NBP2-67068), against EGFP (1:1,000; Abcam, ab6556), and mouse β-tubulin (1:4,000; Developmental Studies Hybridoma Bank, ε-7) at 4°C overnight. After washing, the immunoblots were incubated with an anti-mouse (1:3,000; Jackson ImmunoResearch, 115-036-068) or anti-rabbit HRP-conjugated secondary antiserum (1:3,000; Jackson ImmunoResearch, 111-036-003) in 5% milk-PBST for 1 h. The bound antibody was visualized by an enhanced chemiluminescence system (Cytiva-Amersham; RPN2232). The Mpz band was quantified on Coomassie-blue-stained gel (Merk; 17-0518-01). For quantification, optical density ratios were calculated using ImageJ software.

RNAscope plus small-RNA-RNA assay in treated mice

Fresh spinal cords with roots attached, as well as sciatic and femoral nerves, were collected and flash-frozen in liquid nitrogen with isopentane. All tissues were shipped to Advanced Cell Diagnostics (ACD) for embedding, sectioning, and analysis using RNAscope Plus smRNA-RNA assay (ACD Bio: 322770). This assay was used to co-detect and evaluate biodistribution of miR871 (ACD Bio: #1177468-S1), human *PMP22* (ACD Bio: 505168-C3), and murine *Pmp22* (ACD Bio: 505178-C2). Human and mouse probes against *PMP22*/*Pmp22* were designed to avoid cross-reactivity between species. ACD positive and negative control panels were used for sample quality control (QC) and to evaluate RNA quality in tissue samples. Optimization was performed to establish the best signal-to-noise ratio, which included sample pretreatment conditions (epitope retrieval: 15 min at 88°C and protease III: 15 min at 40°C). All samples passed QC with moderate *Ppib* positive control staining and little to no *dapB* background staining. Quantitative image analysis was conducted using HALO software to determine the percentage of positive cells and quantify miR871 and transcript abundance, reported as the number of dots (i.e., copies) per cell.

Behavioral testing in treated mice

Rotarod

Animals were trained on an accelerating rotarod apparatus (Ugo Basile, 7650) for three consecutive days in three trials per day with 15-min rest periods between trials. The mice were placed on the rod, and the speed was gradually increased from 2.5 to 25 rpm. Trials were considered complete when a mouse remained on the rod for 600 s or fell from the rod prior to 600 s. Testing was performed on the fourth day using two different speeds, 5 and 17.5 rpm. Latency to fall was calculated for each speed and reported in seconds.

Hindlimb grip strength

Mice were held by the tail and allowed to grasp the grip strength apparatus grid only with hindlimb paws (Ugo Basile, 57107). Mice were gently pulled back until they released the grid. Each session consisted of six consecutive trials. Measurements of the force (reported in units of g) were indicated on the equipment. Hindlimb force was calculated by averaging the scores of each trial for each animal.

Wire hang

Animals were placed atop a wire, which was then inverted, causing the mice to hang from the paws. Latency to fall was then recorded and reported in seconds. This test was performed once a day for 3 days with data representing average performance.

Hindlimb clasping evaluation

Mice were suspended by the base of the tail and three pictures captured every 5 s. The average angle of each mouse hindlimb opening was calculated using ImageJ software.

Electrophysiological analysis in treated mice

For MNCV and CMAP measurements, the bilateral sciatic nerves were stimulated in anesthetized animals at the sciatic notch and distally at the knee via bipolar electrodes with supramaximal square-wave pulses (5 V) of 0.05 ms. MNCV was calculated by dividing the distance between the stimulating and recording electrodes by the result of subtracting the distal latency from the proximal latency. The latencies of CMAP were recorded by a bipolar electrode inserted between digits 2 and 3 of the hind paw and measured from the stimulus apparatus to the onset of the negative M-wave deflection. A fixed distance was used between the distal stimulation and recording sites to avoid errors arising from variations in knee-paw distance in each mouse.

Plasma neurofilament light levels in treated mice

Blood samples were collected from the retro-orbital sinus as previously described and processed within 1 hour.[43] Blood samples were collected in EDTA-containing tubes and centrifuged at 20°C at 3,500 rpm for 10 min. Centrifugation separated blood samples in two phases, and the top plasma phase was collected and stored at −80°C until testing. Plasma NF-L concentration was measured at University College London (UCL) using a commercially available NF-Light kit on a single molecule array (Simoa) HD-X instrument (Quanterix).[44,45]

Morphometric analysis of myelination in lumbar roots and peripheral nerves

Morphometric analysis was performed as described before.[10] Mice were transcardially perfused with 2.5% glutaraldehyde (Agar, R1010) in 0.1M PB buffer. The lumbar spinal cord with multiple spinal roots attached, as well as the femoral and sciatic nerves, were dissected and fixed overnight at 4°C, then osmicated (SPI, 02601-AB), dehydrated, and embedded in resin (mixture of 17% Araldite resin (Agar, R1040), 25.5% Agar 100 (Agar, R1043), 55.5% dodecenylsuccinic anhydride (Agar, R1051), and 2% 2,4,6-tri(dimethylaminomethyl)phenol (Agar, R1064). Transverse semi-thin sections (1 μm) of the lumbar spinal cord with roots and the

middle portion of the femoral motor and sciatic nerves were obtained and stained with alkaline toluidine blue (SPI, 02576-AB). Nikon Eclipse Ni microscope with a digital camera (DS-Fi3) using NIS Elements software was used to capture series of partially overlapping fields covering the entire cross-sectional area. Sciatic-nerves-detailed pictures were obtained at 1,000× final magnification. In brief, all demyelinated, thinly myelinated, and normally myelinated axons were counted using the following criteria: axons larger than 1 μm without a myelin sheath were considered demyelinated; axons with myelin sheaths <10% of the axonal diameter were considered thinly myelinated; axons surrounded by circumferentially arranged SC processes and extracellular matrix were considered as "onion bulbs"; all other myelinated axons were considered normally myelinated.

Hematology analysis in mice

Mice were anesthetized, and blood was collected directly from the heart. For complete blood count, blood was collected in MiniCollect EDTA-treated tubes (Greiner, 450530) while for biochemical and other immunological testing blood was collected in serum MiniCollect tubes (Greiner, 450533). Blood samples were delivered to City Clinical Laboratories (Nicosia, Cyprus) within 2 h of collection. Complete blood count testing was performed using the Sysmex XN-550 hematological analyzer. Samples were checked visually for clots, inverted 6–10 times, and then analyzed following Sysmex XN-550 manufacturers protocol. All biochemistry tests and other immunological testing blood samples were centrifuged for 20 min at 3,000 rpm and 20°C and then analyzed following Roche c311 analyzer and Roche e411 for the immunology testing using the manufacturer's protocol. Samples were processed for serum chemistry and hematology parameters. In priority order, serum chemistry parameters included aspartate aminotransferase (AST), alanine aminotransferase (ALT), alkaline phosphatase (ALP), creatine kinase (CK), creatinine, blood urea nitrogen (BUN), total protein, sodium, potassium, chloride, phosphorus, magnesium, triglycerides, gamma glutamyl transferase (GGT), lipase, and troponin I. In prior order, hematology parameters included white blood cell count, red blood cell count, hemoglobin, hematocrit, mean corpuscular volume, mean corpuscular hemoglobin, mean corpuscular hemoglobin concentration, platelet count, absolute neutrophils, absolute lymphocytes, absolute monocytes, absolute eosinophils, and basophils.

Immunofluorescence staining

For immunostaining, mice were anesthetized and then transcardially perfused with normal saline followed by fresh 4% paraformaldehyde (PFA, Merck, P6148) in 0.1M PB buffer. Neural and peripheral tissues were dissected, post-fixed in 4% PFA, and frozen for cryosections. Sections were permeabilized in cold acetone and incubated at room temperature (RT) with a blocking solution of 5% BSA (Sigma-Aldrich, A79061) containing 0.5% Triton X-(Sigma-Aldrich, T8787) for 1 h. Primary antibodies used were mouse monoclonal antibody against CD3 (1:100; Abcam, ab5690), rat CD68 (1:50; Bio-Rad, MCA1957A488), CD45 (1:100; Abcam, ab25386), goat CD20 (1:100; Santa Cruz, sc-7735), mouse GFAP (1:200, Sigma, G3893), and rabbit Iba1 (1:250, Biocare medical, CP290A), all diluted in blocking solution and incubated overnight at 4°C. Slides were then washed in PBS and incubated with mouse cross-affinity fluorescein-conjugated (1:1,000; Invitrogen, A21202), rat cross-affinity purified rhodamine-conjugated (1:2,000; Invitrogen, A21434), goat cross-affinity fluorescein-conjugated (1:700; Abcam, ab150129), and rabbit cross-affinity fluorescein-conjugated (1:1500, Jackson ImmunoResearch, 111-166-003) secondary antibodies for 1 h at RT. Cell nuclei were visualized with DAPI (1 μg/mL; Sigma, MBD0015). Slides were mounted with fluorescent mounting medium (Agilent-DAKO, S3023) and images photographed under a fluorescence microscope with a digital camera using a fluorescence microscope (Nikon Eclipse Ni) with a digital camera (DS-Qi2) using NIS-Elements software. EGFP was shown as green autofluorescence. Total cell numbers and number of positive cells were counted.

H&E staining

For H&E histological assessment, mice were anesthetized and then transcardially perfused with normal saline followed by 10% neutral buffered formalin solution. Neural (brain, spinal cord, spinal roots, DRGs, femoral nerves, and sciatic nerves) and peripheral (liver, lung, kidneys, spleen, quadriceps, diaphragm, and sex organs) tissues were isolated, fixed in 10% neutral buffered formalin solution overnight at room temperature, and then embedded in paraffin. To evaluate tissue microanatomy, 8-μm-thick paraffin sections were stained with H&E and visualized under the light microscope. Whole tissue sections were assessed, and representative images were captured at 20× magnification using Nikon Eclipse Ni microscope and a digital high-definition color camera (DS-Fi2) using NIS-Elements software.

NHP regulatory compliance statements

Cynomolgus monkeys were used to assess biodistribution, safety, and therapeutic target engagement of scAAV9.U6miR871. The NHP study was conducted at the contract research organization, Amplify Bio (Columbus Oh) in compliance with the current version of the USFDA Good Laboratory Practice (GLP) Regulations, 21 CFR Part 58 for the conduct of non-clinical laboratory studies. A full list of exceptions can be found in Figure S23. Animal care, housing, and environmental conditions met current AAALAC international recommendations stated in the "Guide for Care and Use of Laboratory Animals" (National Research Council, Current Edition) and was approved by the Amplify Bio Institutional Animal Care and Use Committee. Animals were socially housed in a temperature and humidity-controlled facility with a 12-h light-dark cycle. Animals were provided fresh water *ad libitum* and fed PMI Certified Primate Diet (LabDiet 5048) twice daily and supplemented with fruits, vegetables, or other supplemental enrichment. Alternative feeding schedules were implemented on the day of vector administration, during specified fasting periods, or when removed from the home cage for study-related tests.

NHP study design

A full study protocol can be found in Figure S23. Briefly, 20 *Macaca facicularis* (10 male, 10 female) were randomly assigned to one of three dosing groups. Four animals received administration of the control article (Formulation Buffer), 8 animals received administration of

6E13 vg (low dose) of scAAV9.U6miR871 in 0.9% saline, and 8 animals received administration of 1.2E14 vg (high dose) of scAAV9.U6-miR871 in 0.9% saline. Animals were mildly sedated with 10 mg/mL acepromazine, and test article was administered to each animal as a 3-mL single-dose intrathecal slow infusion over approximately 6 h via a pre-study implanted catheter. All in-life observations, assessments, and sample collections can be found in Figure S23.

Necropsies were performed in two cohorts with equivalent males and females pre-dosing group at day 43 (6 week) and day 85 (12 week). The tissues indicated for flash freezing (FF) at necropsy were collected under aseptic conditions. Following collection, tissue samples were sectioned into three ∼3 mm^3 aliquots (DNA, RNA, and additional sample [AS]), measured visually without the use of a measuring aid, placed into vials, and flash-frozen using liquid nitrogen. Samples were transferred to the Amplify Bio analytics department for processing and biodistribution analysis via digital PCR (dPCR) for DNA analysis of AAV2-ITR vector genomes (Bio-Rad: 12003909) or RNA analysis for miR871, *PMP22* (Thermo Fisher Scientific: 4351372, Mf02827937_m1), and *B2M* (Integrated DNA Technologies [IDT]: forward primer 5' – GCTATCCAGCGTACTCCAAAG - 3′; reverse primer 5' – TCCAGACACATAGCAATTCAGG - 3′; probe 5' – HEX-CAGGTTTAC/ZEN/TCACGCCATCCACCA-Iowa Black – 3′) gene expression with RT-dPCR. Tissue samples from peripheral nerves were collected from distal and proximal ends of each nerve. One of the two designations of distal and proximal ends of the nerve was placed in 4% glutaraldehyde in 0.1 M phosphate buffer pH 7.4 and shipped refrigerated to Nationwide Children's Hospital for morphometric analysis of myelination, as described above. One of the two designations of distal and proximal ends of the nerve was sectioned into one ∼3 mm^3 sample andflash-frozen using liquid nitrogen. Samples were then shipped frozen to Nationwide Children's Hospital for protein analysis.

Western blotting of NHP tissues

For protein extraction, flash-frozen NHP sciatic, femoral, median, and ulnar nerve tissues were weighed and suspended in ice-cold RIPA buffer (Pierce: PI89900) containing complete protease inhibitor cocktail (Roche: 4693116001) at a ratio of 20 μL per mg tissue (w/V). Protein lysate was prepared using a 5-mm stainless steel bead and a Qiagen TissueLyser II, with tissues lysed twice and supernatants pooled. Total protein was quantified using a DC protein assay kit (Bio-Rad: 5000111). For western blot analysis, 20 μg of protein per sample was loaded onto a 4%–20% Criterion TGX stain-free protein gel (Bio-Rad: 5678093) and transferred to a PVDF membrane (Bio-Rad: 1704275) using a semi-dry transfer system (Bio-Rad: 1704150). Membranes were blocked with 5% milk in tris-buffered saline with Tween 20 (TBS-T; 200 mM Tris, 1,500 mM NaCl, pH 7.6) for 1 h at room temperature, then incubated with a mouse anti-PMP22 antibody (1:500; Abcam: ab90782) overnight at 4°C. After three washes with TBS-T, membranes were incubated with goat anti-mouse IgG HRP (1:100,000; Jackson ImmunoResearch: 115-035-003) for 1 h at room temperature. PMP22 was visualized using a chemiluminescent HRP substrate (Millipore: WBKLS0500). Mouse anti-beta actin antibody (1:5,000; Abcam: ab6276) was used for loading normalization. Membranes were then treated with stripping buffer (Thermo Fisher Scientific: 21059) and subsequently blocked and incubated overnight at 4°C with rabbit anti-MPZ antibody (1:5,000; Abcam: ab183868). This was followed by incubation with goat anti-rabbit IgG HRP (1:100,000; Jackson ImmunoResearch: 111-035-003) for 1 h at room temperature. All band intensities were quantified using ImageJ. Each nerve grouping (level and side) was analyzed from a single blot per time point. PMP22 was normalized to beta-actin, then to the averaged control set to 100%. Individual samples were excluded from analysis for the following criteria: (1) beta-actin optical density below 50% of the average of all samples run on the same blot and (2) MPZ optical density below 50% of the average beta-actin-normalized MPZ density of control samples run on the same blot.

DATA AND CODE AVAILABILITY

All data are in the paper and supplementary materials. Transcriptomics data will be deposited to a public database.

ACKNOWLEDGMENTS

The authors wish to thank Dr. Steven Zelenkofske and Mary Newman at Armatus Bio for helpful advice and discussions. We also thank colleagues at Transcendent Pathology, Amplify Bio (especially Dr. Sapna Varia), and Bio-Techne for their contract work supporting these studies. Finally, we thank Natalie Rohan and Allison Lowery at Nationwide Children's Hospital for logistical support. This work was funded by Armatus Bio, through sponsored research agreements to the Harper and Kleopa laboratories at Nationwide Children's Hospital and the Cyprus Institute of Neurology and Genetics.

AUTHOR CONTRIBUTIONS

Conceptualization, M.S., L.M.W., R.S., B.P., K.A.K., and S.Q.H.; methodology, M.S., L.M.W., M.T., N.Y.S., M.B.B., K.A.K., H.Z., and S.Q.H.; investigation, M.S., L.M.W., M.T., N.K.T., A.K., R.P., C.McA., G.Z., N.Y.S., M.B.B., A.H., C.T., J.R., B.P., K.A.K., and S.Q.H.; writing—original draft, M.S., L.M.W., K.A.K., and S.Q.H.; writing—review and editing, all authors; visualization, M.S., L.M.W., M.T., N.Y.S., and M.B.B.; project administration, M.S., L.M.W., B.P., K.A.K., and S.Q.H.; funding acquisition, R.S., B.P., K.A.K., and S.Q.H.

DECLARATION OF INTERESTS

M.S., K.A.K., and S.Q.H. are listed inventors on US patent application #US 2024/0318171, entitled "Products and methods for inhibition of expression of peripheral myelin protein 22", published September 26, 2024. This invention was licensed for development and commercialization by Armatus Bio, a private startup company for which S.Q.H. is co-founder and serves as Chief Scientific Advisor. B.P. is also a co-founder of Armatus Bio and serves as Chief Technology Officer. R.S. serves as Chief Executive Officer at Armatus Bio. L.M.W. serves as a paid consultant for Armatus Bio. S.Q.H., R.S., B.P., and K.A.K. all have equity in Armatus Bio.

SUPPLEMENTAL INFORMATION

Supplemental information can be found online at https://doi.org/10.1016/j.omtn.2026.102881.

REFERENCES

1. De Grado, A., Serio, M., Saveri, P., Pisciotta, C., and Pareyson, D. (2025). Charcot-Marie-Tooth disease: a review of clinical developments and its management - What's new in 2025? Expert Rev. Neurother. *25*, 427–442.

2. Deenen, J.C., Verbeek, A.L., Verschuuren, J.J., van Engelen, B.G., and Voermans, N.C. (2025). Prevalence and incidence rates of 17 neuromuscular disorders: An updated review of the literature. J. Neuromuscul. Dis. *12*, 713–722.

3. Stavrou, M., Kagiava, A., Sargiannidou, I., Georgiou, E., and Kleopa, K.A. (2023). Charcot-Marie-Tooth neuropathies: Current gene therapy advances and the route toward translation. J. Peripher. Nerv. Syst. *28*, 150–168.
4. Huxley, C., Passage, E., Robertson, A.M., Youl, B., Huston, S., Manson, A., Sabéran-Djoniedi, D., Figarella-Branger, D., Pellissier, J.F., Thomas, P.K., and Fontés, M. (1998). Correlation between varying levels of PMP22 expression and the degree of demyelination and reduction in nerve conduction velocity in transgenic mice. Hum. Mol. Genet. *7*, 449–458.
5. Valentijn, L.J., Bolhuis, P.A., Zorn, I., Hoogendijk, J.E., van den Bosch, N., Hensels, G.W., Stanton, V.P., Housman, D.E., Fischbeck, K.H., Ross, D.A., et al. (1992). The peripheral myelin gene PMP-22/GAS-3 is duplicated in Charcot-Marie-Tooth disease type 1A. Nat. Genet. *1*, 166–170.
6. Yoshikawa, H., Nishimura, T., Nakatsuji, Y., Fujimura, H., Himoro, M., Hayasaka, K., Sakoda, S., and Yanagihara, T. (1994). Elevated expression of messenger RNA for peripheral myelin protein 22 in biopsied peripheral nerves of patients with Charcot-Marie-Tooth disease type 1A. Ann. Neurol. *35*, 445–450.
7. Boutary, S., Caillaud, M., El Madani, M., Vallat, J.M., Loisel-Duwattez, J., Rouyer, A., Richard, L., Gracia, C., Urbinati, G., Desmaële, D., et al. (2021). Squalenoyl siRNA PMP22 nanoparticles are effective in treating mouse models of Charcot-Marie-Tooth disease type 1 A. Commun. Biol. *4*, 317.
8. Lee, J.S., Kwak, G., Kim, H.J., Park, H.T., Choi, B.O., and Hong, Y.B. (2019). miR-381 Attenuates Peripheral Neuropathic Phenotype Caused by Overexpression of PMP22. Exp. Neurobiol. *28*, 279–288.
9. Lee, J.S., Lee, J.Y., Song, D.W., Bae, H.S., Doo, H.M., Yu, H.S., Lee, K.J., Kim, H.K., Hwang, H., Kwak, G., et al. (2020). Targeted PMP22 TATA-box editing by CRISPR/Cas9 reduces demyelinating neuropathy of Charcot-Marie-Tooth disease type 1A in mice. Nucleic Acids Res. *48*, 130–140.
10. Stavrou, M., Kagiava, A., Choudury, S.G., Jennings, M.J., Wallace, L.M., Fowler, A.M., Heslegrave, A., Richter, J., Tryfonos, C., Christodoulou, C., et al. (2022). A translatable RNAi-driven gene therapy silences PMP22/Pmp22 genes and improves neuropathy in CMT1A mice. J. Clin. Investig. *132*, e159814.
11. Zhao, H.T., Damle, S., Ikeda-Lee, K., Kuntz, S., Li, J., Mohan, A., Kim, A., Hung, G., Scheideler, M.A., Scherer, S.S., et al. (2018). PMP22 antisense oligonucleotides reverse Charcot-Marie-Tooth disease type 1A features in rodent models. J. Clin. Investig. *128*, 359–368.
12. Gautier, B., Hajjar, H., Soares, S., Berthelot, J., Deck, M., Abbou, S., Campbell, G., Ceprian, M., Gonzalez, S., Fovet, C.M., et al. (2021). AAV2/9-mediated silencing of PMP22 prevents the development of pathological features in a rat model of Charcot-Marie-Tooth disease 1 A. Nat. Commun. *12*, 2356.
13. Kagiava, A., Richter, J., Tryfonos, C., Leal-Julià, M., Sargiannidou, I., Christodoulou, C., Bosch, A., and Kleopa, K.A. (2021). Efficacy of AAV serotypes to target Schwann cells after intrathecal and intravenous delivery. Sci. Rep. *11*, 23358.
14. Elbashir, S.M., Harborth, J., Lendeckel, W., Yalcin, A., Weber, K., and Tuschl, T. (2001). Duplexes of 21-nucleotide RNAs mediate RNA interference in cultured mammalian cells. Nature *411*, 494–498.
15. Elbashir, S.M., Lendeckel, W., and Tuschl, T. (2001). RNA interference is mediated by 21- and 22-nucleotide RNAs. Genes Dev. *15*, 188–200.
16. Fire, A., Xu, S., Montgomery, M.K., Kostas, S.A., Driver, S.E., and Mello, C.C. (1998). Potent and specific genetic interference by double-stranded RNA in Caenorhabditis elegans. Nature *391*, 806–811.
17. Carmell, M.A., Xuan, Z., Zhang, M.Q., and Hannon, G.J. (2002). The Argonaute family: tentacles that reach into RNAi, developmental control, stem cell maintenance, and tumorigenesis. Genes Dev. *16*, 2733–2742.
18. Irvine, D.V., Zaratiegui, M., Tolia, N.H., Goto, D.B., Chitwood, D.H., Vaughn, M.W., Joshua-Tor, L., and Martienssen, R.A. (2006). Argonaute slicing is required for heterochromatic silencing and spreading. Science *313*, 1134–1137.
19. Song, J.J., and Joshua-Tor, L. (2006). Argonaute and RNA–getting into the groove. Curr. Opin. Struct. Biol. *16*, 5–11.
20. Aimiuwu, O.V., Fowler, A.M., Sah, M., Teoh, J.J., Kanber, A., Pyne, N.K., Petri, S., Rosenthal-Weiss, C., Yang, M., Harper, S.Q., and Frankel, W.N. (2020). RNAi-Based Gene Therapy Rescues Developmental and Epileptic Encephalopathy in a Genetic Mouse Model. Mol. Ther. *28*, 1706–1716.
21. Harper, S.Q., Staber, P.D., He, X., Eliason, S.L., Martins, I.H., Mao, Q., Yang, L., Kotin, R.M., Paulson, H.L., and Davidson, B.L. (2005). RNA interference improves motor and neuropathological abnormalities in a Huntington's disease mouse model. Proc. Natl. Acad. Sci. USA *102*, 5820–5825.
22. Liu, J., Wallace, L.M., Garwick-Coppens, S.E., Sloboda, D.D., Davis, C.S., Hakim, C.H., Hauser, M.A., Brooks, S.V., Mendell, J.R., and Harper, S.Q. (2014). RNAi-mediated Gene Silencing of Mutant Myotilin Improves Myopathy in LGMD1A Mice. Mol. Ther. Nucleic Acids *3*, e160.
23. Morelli, K.H., Griffin, L.B., Pyne, N.K., Wallace, L.M., Fowler, A.M., Oprescu, S.N., Takase, R., Wei, N., Meyer-Schuman, R., Mellacheruvu, D., et al. (2019). Allele-specific RNA interference prevents neuropathy in Charcot-Marie-Tooth disease type 2D mouse models. J. Clin. Investig. *129*, 5568–5583.
24. Saad, N.Y., Al-Kharsan, M., Garwick-Coppens, S.E., Chermahini, G.A., Harper, M.A., Palo, A., Boudreau, R.L., and Harper, S.Q. (2021). Human miRNA miR-675 inhibits DUX4 expression and may be exploited as a potential treatment for Facioscapulohumeral muscular dystrophy. Nat. Commun. *12*, 7128.
25. Wallace, L.M., Liu, J., Domire, J.S., Garwick-Coppens, S.E., Guckes, S.M., Mendell, J.R., Flanigan, K.M., and Harper, S.Q. (2012). RNA interference inhibits DUX4-induced muscle toxicity in vivo: implications for a targeted FSHD therapy. Mol. Ther. *20*, 1417–1423.
26. Wallace, L.M., Saad, N.Y., Pyne, N.K., Fowler, A.M., Eidahl, J.O., Domire, J.S., Griffin, D.A., Herman, A.C., Sahenk, Z., Rodino-Klapac, L.R., and Harper, S.Q. (2018). Pre-clinical Safety and Off-Target Studies to Support Translation of AAV-Mediated RNAi Therapy for FSHD. Mol. Ther. Methods Clin. Dev. *8*, 121–130.
27. Krüger, J., and Rehmsmeier, M. (2006). RNAhybrid: microRNA target prediction easy, fast and flexible. Nucleic Acids Res. *34*, W451–W454.
28. Buss, N., Lanigan, L., Zeller, J., Cissell, D., Metea, M., Adams, E., Higgins, M., Kim, K.H., Budzynski, E., Yang, L., et al. (2022). Characterization of AAV-mediated dorsal root ganglionopathy. Mol. Ther. Methods Clin. Dev. *24*, 342–354.
29. Hawley, Z.C.E., Pardo, I.D., Cao, S., Zavodszky, M.I., Casey, F., Ferber, K., Luo, Y., Hana, S., Chen, S.K., Doherty, J., et al. (2025). Dorsal root ganglion toxicity after AAV intra-CSF delivery of a RNAi expression construct into non-human primates and mice. Mol. Ther. *33*, 215–234.
30. Hordeaux, J., Buza, E.L., Dyer, C., Goode, T., Mitchell, T.W., Richman, L., Denton, N., Hinderer, C., Katz, N., Schmid, R., et al. (2020). Adeno-Associated Virus-Induced Dorsal Root Ganglion Pathology. Hum. Gene Ther. *31*, 808–818.
31. Hordeaux, J., Hinderer, C., Buza, E.L., Louboutin, J.P., Jahan, T., Bell, P., Chichester, J.A., Tarantal, A.F., and Wilson, J.M. (2019). Safe and Sustained Expression of Human Iduronidase After Intrathecal Administration of Adeno-Associated Virus Serotype 9 in Infant Rhesus Monkeys. Hum. Gene Ther. *30*, 957–966.
32. Ligocki, A.P., Vinson, A.V., Yachnis, A.T., Dunn, W.A., Jr., Smith, D.E., Scott, E.A., Alvarez-Castanon, J.V., Baez Montalvo, D.E., Frisone, O.G., Brown, G.A.J., et al. (2024). Cerebrospinal fluid flow extends to peripheral nerves further unifying the nervous system. Sci. Adv. *10*, eadn3259.
33. Mizisin, A.P., and Weerasuriya, A. (2011). Homeostatic regulation of the endoneurial microenvironment during development, aging and in response to trauma, disease and toxic insult. Acta Neuropathol. *121*, 291–312.
34. Bala, U., Tan, K.L., Ling, K.H., and Cheah, P.S. (2014). Harvesting the maximum length of sciatic nerve from adult mice: a step-by-step approach. BMC Res. Notes *7*, 714.
35. Bharucha-Goebel, D.X., Todd, J.J., Saade, D., Norato, G., Jain, M., Lehky, T., Bailey, R.M., Chichester, J.A., Calcedo, R., Armao, D., et al. (2024). Intrathecal Gene Therapy for Giant Axonal Neuropathy. N. Engl. J. Med. *390*, 1092–1104.
36. Waldrop, M.A. (2024). Clinical decision making around commercial use of gene and genetic therapies for spinal muscular atrophy. Neurotherapeutics *21*, e00437.
37. Katona, I., Wu, X., Feely, S.M.E., Sottile, S., Siskind, C.E., Miller, L.J., Shy, M.E., and Li, J. (2009). PMP22 expression in dermal nerve myelin from patients with CMT1A. Brain *132*, 1734–1740.
38. Greig, J.A., Martins, K.M., Breton, C., Lamontagne, R.J., Zhu, Y., He, Z., White, J., Zhu, J.X., Chichester, J.A., Zheng, Q., et al. (2024). Integrated vector genomes may contribute to long-term expression in primate liver after AAV administration. Nat. Biotechnol. *42*, 1232–1242.

39. Meseck, E.K., Guibinga, G., Wang, S., McElroy, C., Hudry, E., and Mansfield, K. (2022). Intrathecal sc-AAV9-CB-GFP: Systemic Distribution Predominates Following Single-Dose Administration in Cynomolgus Macaques. Toxicol. Pathol. *50*, 415–431.

40. Ohno, K., Samaranch, L., Hadaczek, P., Bringas, J.R., Allen, P.C., Sudhakar, V., Stockinger, D.E., Snieckus, C., Campagna, M.V., San Sebastian, W., et al. (2019). Kinetics and MR-Based Monitoring of AAV9 Vector Delivery into Cerebrospinal Fluid of Nonhuman Primates. Mol. Ther. Methods Clin. Dev. *13*, 47–54.

41. Bey, K., Deniaud, J., Dubreil, L., Joussemet, B., Cristini, J., Ciron, C., Hordeaux, J., Le Boulch, M., Marche, K., Maquigneau, M., et al. (2020). Intra-CSF AAV9 and AAVrh10 Administration in Nonhuman Primates: Promising Routes and Vectors for Which Neurological Diseases? Mol. Ther. Methods Clin. Dev. *17*, 771–784.

42. Stavrou, M., Georgiou, E., and Kleopa, K.A. (2024). Lumbar Intrathecal Injection in Adult and Neonatal Mice. Curr. Protoc. *4*, e1091.

43. Kagiava, A., Karaiskos, C., Richter, J., Tryfonos, C., Jennings, M.J., Heslegrave, A.J., Sargiannidou, I., Stavrou, M., Zetterberg, H., Reilly, M.M., et al. (2021). AAV9-mediated Schwann cell-targeted gene therapy rescues a model of demyelinating neuropathy. Gene Ther. *28*, 659–675.

44. Rohrer, J.D., Woollacott, I.O.C., Dick, K.M., Brotherhood, E., Gordon, E., Fellows, A., Toombs, J., Druyeh, R., Cardoso, M.J., Ourselin, S., et al. (2016). Serum neurofilament light chain protein is a measure of disease intensity in frontotemporal dementia. Neurology *87*, 1329–1336.

45. Sandelius, Å., Zetterberg, H., Blennow, K., Adiutori, R., Malaspina, A., Laura, M., Reilly, M.M., and Rossor, A.M. (2018). Plasma neurofilament light chain concentration in the inherited peripheral neuropathies. Neurology *90*, e518–e524.

OMTN, Volume *37*

Supplemental information

Safety, efficacy, and distal nerve Schwann cell biodistribution in mice and NHPs to support translation of AAV9 RNAi therapy for CMT1A

Marina Stavrou, Lindsay M. Wallace, Merlin P. Thangaraj, Noah K. Taylor, Alexia Kagiava, Revekka Papacharalambous, Cynthia McAllister, Gloria Zender, Nizar Y. Saad, M. Bilal Bayazit, Amanda Heslegrave, Christina Tryfonos, Jan Richter, Henrik Zetterberg, Brian Price, Rachel Salzman, Kleopas A. Kleopa, and Scott Q. Harper

Table S1. Decreased transcripts in miR871-treated human cells.

≥2-fold change; FDR<0.05	≥1.75-fold change; FDR<0.05	
**Neurons**	**Neurons**	
PMP22[a]	PMP22[a]	
**Hepatocytes**	**Hepatocytes**	
ACTC1	ACTC1	ACTN2
ANKRD18B	ANKRD18B	ALPK3
MYBPC3	MYBPC3	ENSG00000223401[b]
MYH7	MYH7	ENSG00000268230
MYL2	MYL2	LDB3
MYL4	MYL4	MYH6
MYL7	MYL7	MYOM1
NEFM	NEFM	NOXA1
NPPA	NPPA	PMP22[a]
TNNI1	TNNI1	TNNC1
TNNT2	TNNT2	ZMYND10[b]
**Schwann cells**	**Schwann cells**	
RGS5	RGS5	ENSG00000268592
STMN2	STMN2	PMP22[a]

FDR, False Discovery Rate. [a]*PMP22* was the only significantly reduced transcript in every cell line treated with miR871, demonstrating on-target specificity. [b]*ENSG000000223401* and *ZMYND10* were detected in Schwann cells and hepatocytes only, while all other transcripts listed were present in each cell line.

Table S2. Relative average reductions of human PMP22 and mouse Pmp22 in CMT1A mice injected with 5E11 dose of AAV9.U6.miR871

	Human PMP22		Mouse PMP22	
**PNS tissue**	**RNA**	**Protein**	**RNA**	**Protein**
Lumbar roots	54%	76%	48%	61%
Sciatic nerve	67%	73%	63%	40%
Femoral nerve	66%	68%	66%	42%

Table S3. First-in-Human Dose Calculations for lumbar intrathecal delivery of AAV9.miR871

Dose		Human equivalent dose[c]	Expected volume and rate for human IT delivery	Dose description
Mouse[a]	NHP[b]			
1E11	-–	3.7E14	---	-–
2E11	6E13	7.4E14	10 – 15 mL / delivered in ~30 min	MED, planned clinical starting dose
5E11	1.2E14	1.9E15	20 – 30 mL / delivered in ~1 hr	FED, planned clinical high dose
1E12	-–	3.7E15	---	-–

NHP, non-human primate; IT, intrathecal; MED, minimum effective dose; FED, fully efficacious dose. [a] all mouse doses delivered in 20 microliters volume; [b] NHP doses delivered in 3 milliliters volume over a 6 hour period. [c] calculated based on cerebrospinal fluid volume

Table S4. 12-week GLP single dose biodistribution and safety study groups

Group	Target Dose (Total vg)	Target Dose Volume (mL)	Number of Animals			
			6-Week Cohort[a]		12-Week Cohort[b]	
			Male	Female	Male	Female
1 – Vehicle	0	3	1	1	1	1
2 – Low	6×10^{13}	3	2	2	2	2
3 – High	1.2×10^{14}	3	2	2	2	2

Abbreviation: vg = vector genomes.
[a] Necropsy Day 43.
[b] Necropsy Day 85.

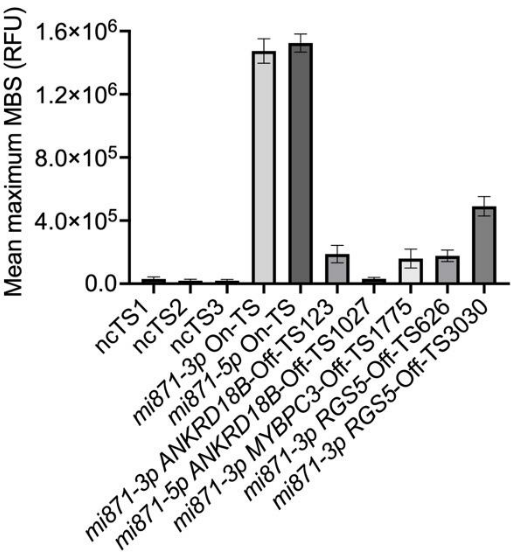

<table>
<tr><th></th><th>K_d (μM)</th><th>B_{max} (RFU)</th><th>Base pairing</th></tr>
<tr><td>mi871-3p On-TS</td><td>0.18</td><td>1495838</td><td>On-TS 5′ GGGGATTGCTGTTGATTGAAGA 3′
||||||||||||||||||||||
mi871-3p 3′ CCCCTAACGACAACTAACTTCT 5′</td></tr>
<tr><td>mi871-5p On-TS</td><td>0.06</td><td>1533775</td><td>On-TS 5′ AGTCTTCAATCAACAGCAACCC 3′
||||||||||||||||||||||
mi871-5p 3′ TCAGAAGTTAGTTGTCGTTGGG 5′</td></tr>
<tr><td>mi871-3p ANKRD18B -Off-TS123</td><td>14</td><td>281946</td><td>Off-TS 5′ G U C GG GGAG U 3′
GG GGAU GCUG UGA GAAGA
|| |||| |||| ||| |||||
CC CCUA CGAC ACU CUUCU
mi871-3p 3′ A A AA 5′</td></tr>
<tr><td>mi871-5p ANKRD18B -Off-TS1027</td><td>12.2</td><td>45347</td><td>Off-TS 5′ A AAUGACAA AGAA A 3′
UCUUCAA UCA ACAGCAACC
||||||| ||| |||||||||
AGAAGUU AGU UGUCGUUGG
mi871-5p 3′ UC G 5′</td></tr>
<tr><td>mi871-3p MYBPC3 -Off-TS1775</td><td>31.7</td><td>318579</td><td>Off-TS 5′ C G C A 3′
GGGG UGU UGA UGAAGA
|||| ||| ||| ||||||
CCCC ACA ACU ACUUCU
mi871-3p 3′ UA ACA A 5′</td></tr>
<tr><td>mi871-3p RGS5 -Off-TS626</td><td>5.2</td><td>215960</td><td>Off-TS 5′ U C GAG ACAA U 3′
GGAUUGC UGU GAUU GAAGA
||||||| ||| |||| |||||
CCUAACG ACA CUAA CUUCU
mi871-3p 3′ CC A 5′</td></tr>
<tr><td>mi871-3p RGS5 -Off-TS3030</td><td>1.5</td><td>531202</td><td>Off-TS 5′ A A A C 3′
GGAUUG UGUU UGAAGA
|||||| |||| ||||||
CCUAAC ACAA ACUUCU
mi871-3p 3′ CC G CUA 5′</td></tr>
</table>

Figure S1. Molecular Beacon-Binding Assay off-target gene assessment. (Top) The graph shows binding data for mi871-3p and mi871-5p target sites within the *PMP22* on-target transcript and RNA-seq identified off-target transcripts (*ANKRD18B*, *MYBPC3* and *RGS5*) that showed binding above background. Data are displayed as means with error bars representing SD. (Bottom) The molecular beacon signal expressed in relative fluorescence units (RFU) was subtracted from background fluorescent signal and fit to a one site-specific binding equation used to determine the binding affinity (K_d), which is the binding site concentration (μM) required to reach half of maximum fluorescence. The K_d represents the binding affinity of miRNA to its target site. The smaller the K_d value, the greater the binding affinity of the miRNA molecular beacon is for its target site. In the base pairing of the single stranded (ss) mi871 DNA sequence with the ssTS DNA sequence, the guanosine nucleotides (G) that were facing a thymidine (T) were replaced by adenosines (A) (indicated in gray) to mimic the G:U base pairing that occurs in RNA:RNA duplexes. miRNA:off-target site pairs are represented as two annealing strands. The top strand represents the target site, and the bottom strand represents the mature mi871 sequence. ncTS1: MB871-3pWT vs ncTS; ncTS2: MB871-3p-MYBPC3-1775 vs ncTS; ncTS3: MB871-5pWT vs ncTS. MBS: Molecular Beacon Signal.

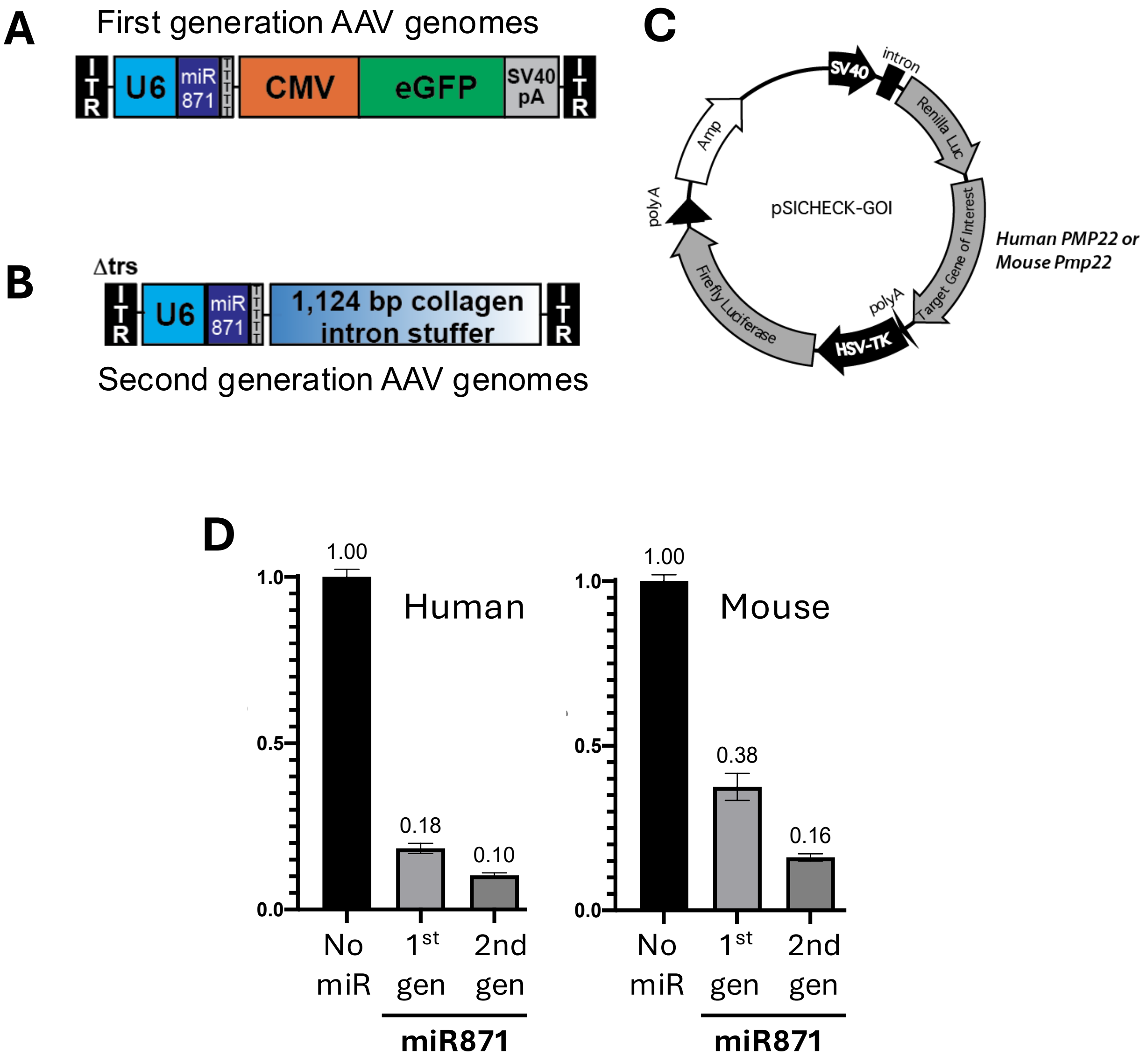

Figure S2. In vitro potency comparison of first- and second-generation miR871 vector genomes. (A) First-generation self-complementary adeno-associated viral (AAV) vector contained a U6 promoter-driven miR871 with T6 terminator and a separate cassette expressing eGFP from the cytomegalovirus (CMV) promoter. Black boxes, AAV inverted terminal repeats (ITRs); gray "T" box, miRNA terminator; SV40 (pA) polyadenylation signal. (B) In the second-generation self-complementary vectors eGFP was replaced with a 1,124 bp segment from the human collagen intron serving as a stuffer sequence to aid in packaging. Black boxes, AAV inverted terminal repeats; Δtrs, self-complementary AAV inverted terminal repeat containing a deletion in the terminal resolution site (trs); gray "T" box, miR terminator. (C) Dual luciferase reporter plasmid (pSICHECK) used for *in vitro* screening of miR871 potency targeting the human *PMP22 and* mouse *Pmp22* cDNAs as the target gene of interest (GOI). The SV40 promoter drives expression of a *Renilla* luciferase (*Renilla* Luc) with respective GOIs cloned as the 3' UTR. Firefly luciferase is driven by the thymidine kinase promoter (TK) and serves as an internal transfection control. (D) Second generation (2nd gen) miR871 vector showed increased gene silencing compared to the first generation (1st gen) vector determined by measuring the ratio of Renilla to firefly luciferase. Data are displayed as means with error bars representing SD (N=3 independent experiments performed in triplicate).

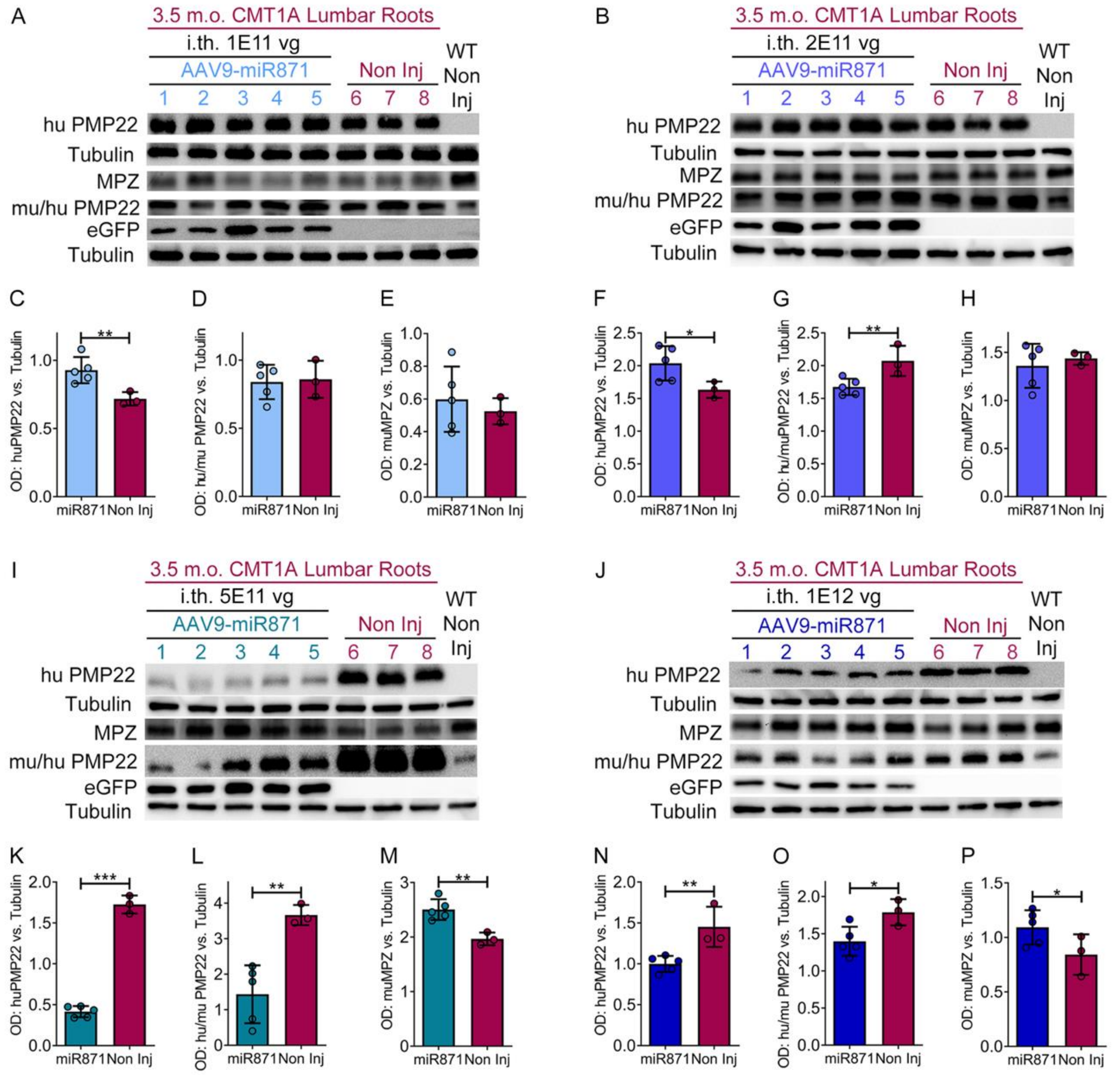

Figure S3. Western blot analysis of spinal nerve root protein lysates from CMT1A mice treated with different AAV9.U6.miR871 doses.

Western blot analysis of lumbar root lysates from CMT1A mice injected at 2 months of age with AAV9.U6.miR871 at doses of 1E11, 2E11, 5E11, or 1E12 vg/animal (n=5/group) and analysed 6 weeks later (3.5 months of age), compared to age-matched non-injected CMT1A controls (n=3). Representative western blot images showing huPMP22, muPMP22, muTubulin, EGFP and muMPZ protein expression and quantification in lumbar roots of CMT1A animals treated with different AAV9.U6.miR871 doses; 1e11 (A,C-E), 2e11 (B, F-H), 5e11 (I, K-M) or 1e12 (J, N-P) vg/animal. Values are presented as mean ± SD. *P < 0.05, **P < 0.01, ***P < 0.001, by Student's t-test.

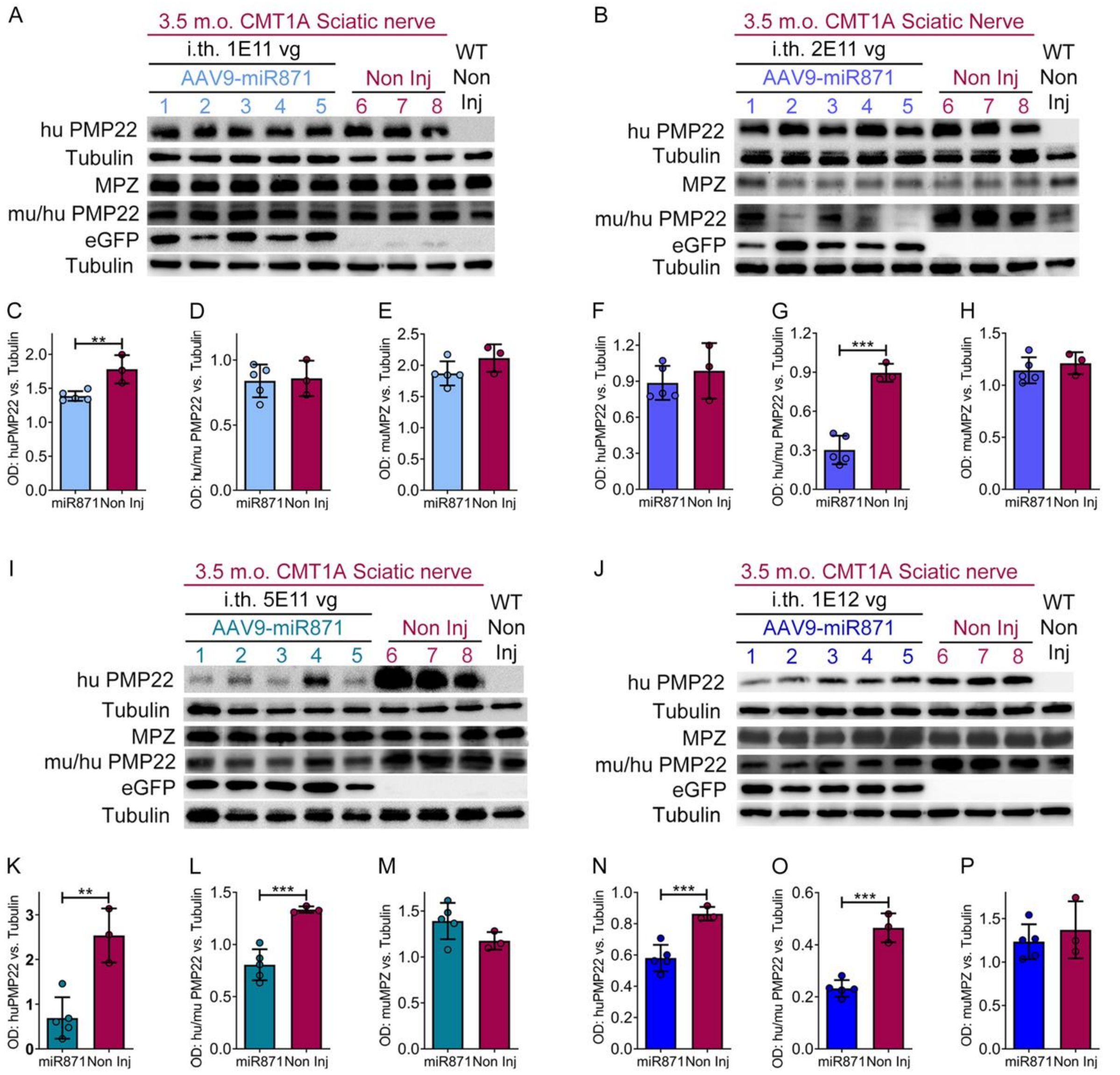

Figure S4. Western blot analysis of sciatic nerve protein lysates from CMT1A mice treated with different AAV9.U6.miR871 doses.

Western blot analysis of sciatic nerve lysates from CMT1A mice injected at 2 months of age with AAV9.U6.miR871 at doses of 1E11, 2E11, 5E11, or 1E12 vg/animal (n=5/group) and analysed 6 weeks post-injection (3.5 months of age), compared to age-matched non-injected CMT1A controls (n=3). Representative western blot images showing huPMP22, muPMP22, muTubulin, EGFP and muMPZ protein expression and quantification in sciatic nerves of CMT1A animals treated with different AAV9.U6.miR871 doses; 1e11 (**A,C-E**), 2e11 (**B, F-H**), 5e11 (**I, K-M**) or 1e12 (**J, N-P**) vg/animal. Values are presented as mean ± SD. *P < 0.05, **P < 0.01, ***P < 0.001, by Student's test.

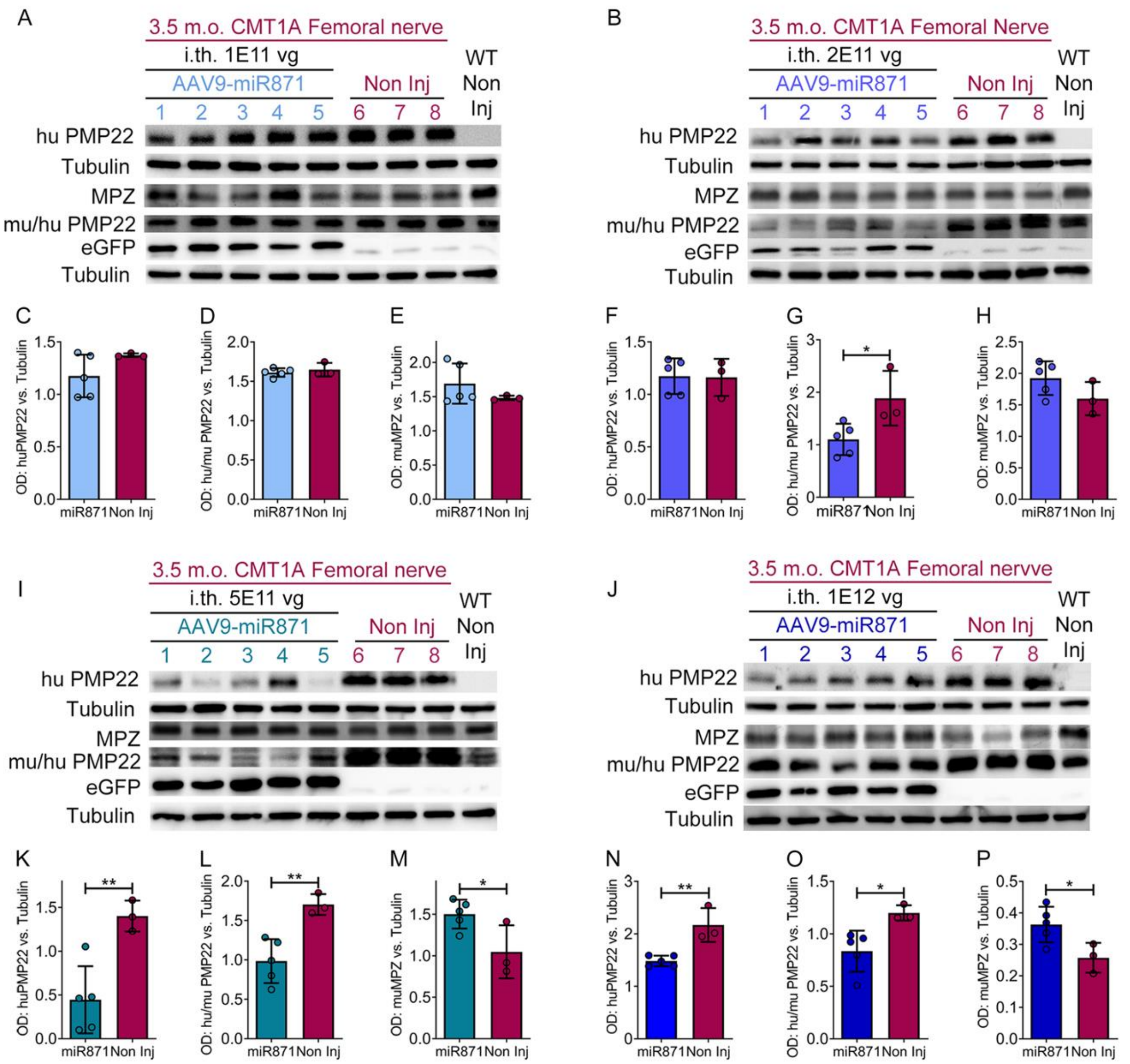

Figure S5. Western blot analysis of femoral nerves lysates in CMT1A mice treated with different AAV9.U6.miR871 doses.

Western blot analysis of femoral nerve lysates from CMT1A mice injected at 2 months of age with AAV9.U6.miR871 at doses of 1E11, 2E11, 5E11, or 1E12 vg/animal (n=5/group) and analysed 6 weeks post-injection (3.5 months of age), compared to age-matched non-injected CMT1A controls (n=3). Representative western blot images showing huPMP22, muPMP22, muTubulin, EGFP and muMPZ protein expression and quantification in femoral nerves of CMT1A animals treated with different AAV9.U6.miR871 doses; 1e11 (**A,C-E**), 2e11 (**B, F-H**), 5e11 (**I, K-M**) or 1e12 (**J, N-P**) vg/animal. Values are presented as mean ± SD. *P < 0.05, **P < 0.01, by Student's test.

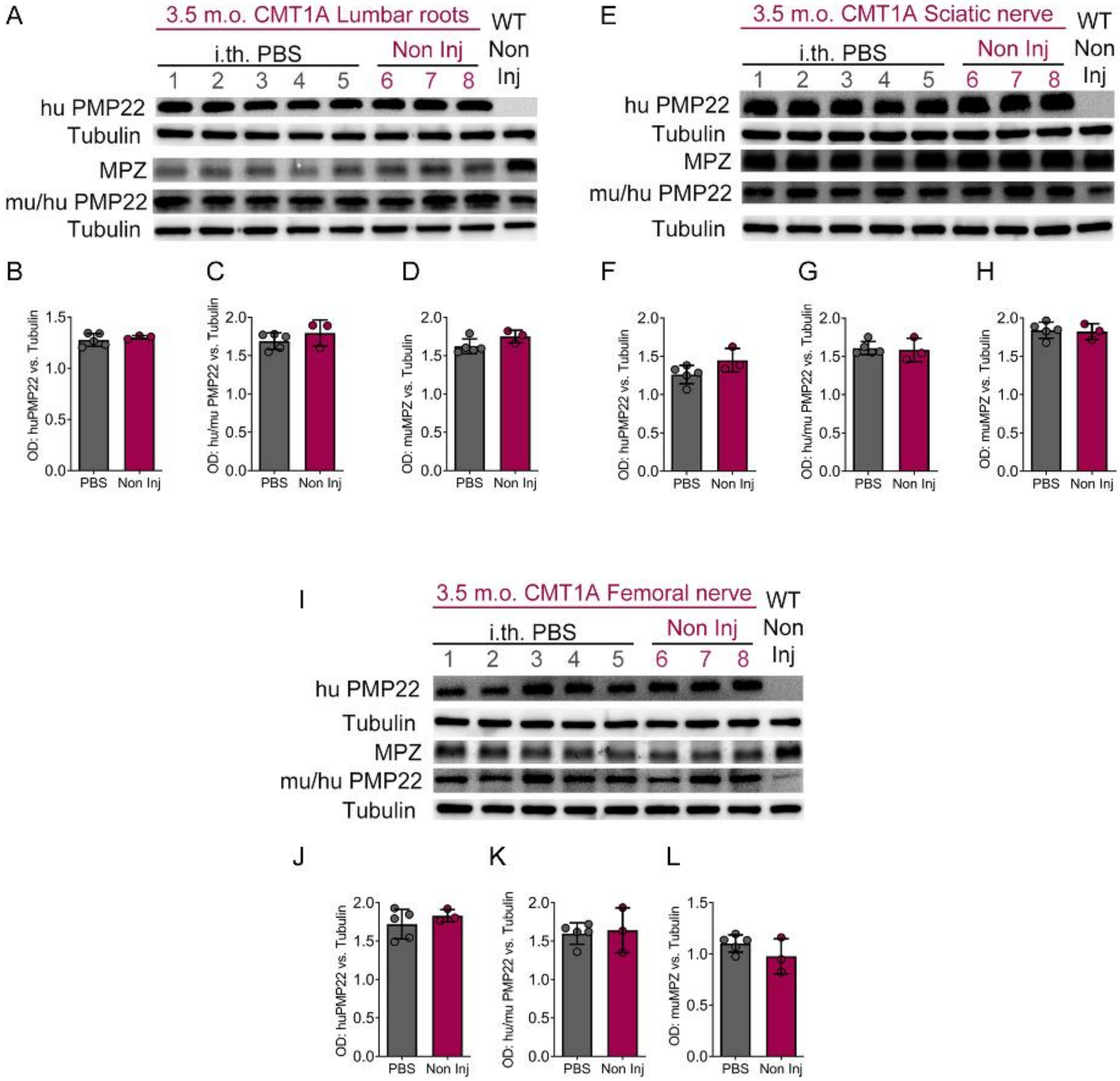

Figure S6. Western blot analysis of PNS tissues lysates in CMT1A mice injected with PBS. Western blot analysis was performed on lumbar roots (**A**), sciatic nerves (**E**) and femoral nerves (**I**) lysates from CMT1A mice injected with PBS at 2 months of age (n=5/group) and analysed 6 weeks post-injection (3.5 months of age), compared to age-matched non-injected CMT1A controls (n=3). Representative western blot images and quantification of huPMP22, muPMP22, muTubulin, EGFP and muMPZ protein expression in lumbar roots (**B-D**), sciatic nerves (**F-H**) and femoral nerves (**J-L**). Values are presented as mean ± SD and compared by unpaired Student's test.

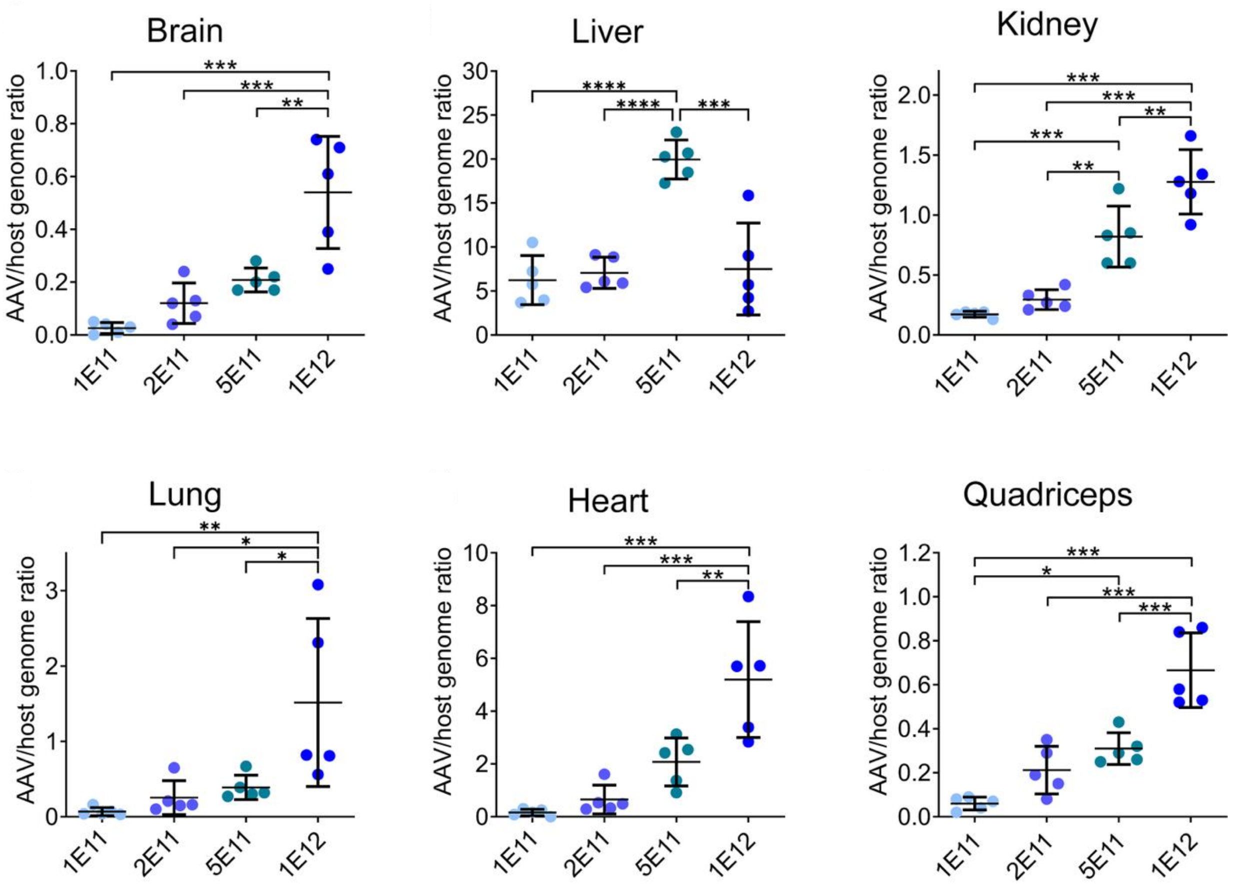

Figure S7. Vector genome copy number (VGCN) analysis in non-PNS tissues of CMT1A mice treated with different AAV9.U6.miR871 doses.

VGCN was quantified in DNA extracted from the brain, liver, kidney, lung, heart and quadriceps of CMT1A mice injected at 2-months of age with AAV9.U6.miR871 at doses of 1E11, 2E11, 5E11, or 1E12 vg/animal and analysed 6-weeks later (n=5/group). Values are presented as mean ± SD. *P < 0.05, **P < 0.01, ***P < 0.001, ****P ≤ 0.0001 by 1-way ANOVA with Tukey's multiple-comparison test.

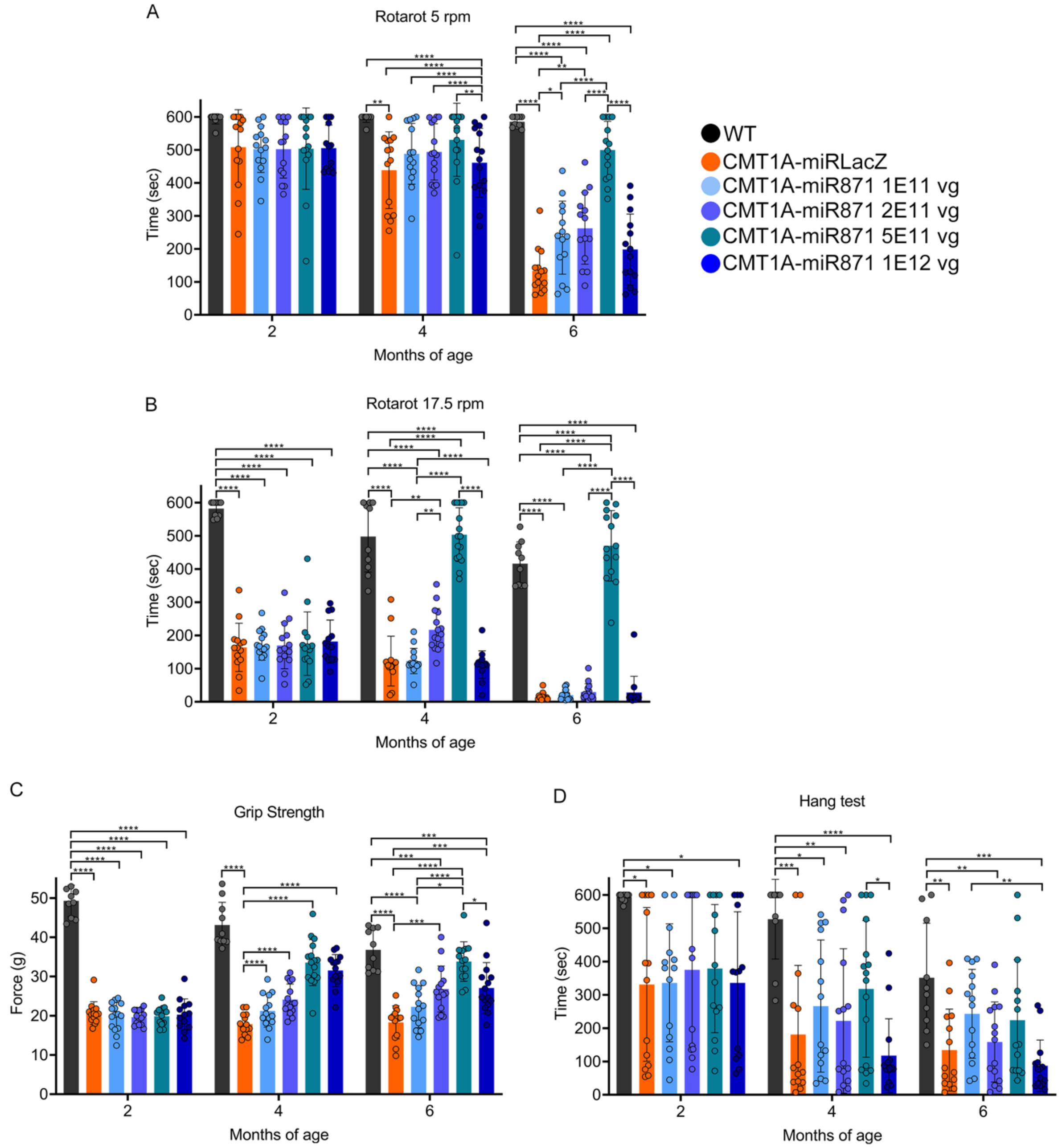

Figure S8. Behavioral analysis of CMT1A mice following treatment with different AAV9.U6.miR871 doses. (A-D) Behavioral assessments, including rotarod at 5 and 17.5 rpm, grip strength and hang test, were conducted in WT mice (rotarod, grip strength, and hang test: n = 10; hind limb abduction: n = 6) and CMT1A mice treated with different doses of AAV9.U6.miR871 (1E11, 2E11, 5E11, or 1E12 vg/animal) or AAV9.U6.miRLacZ (n = 14/group). Behavioral tests were performed before treatment (2 months of age) and monitored every 2 months until 6 months of age. Values are presented as mean ± SD. Statistical significance was determined using one-way ANOVA with Tukey's multiple-comparison test (*$P < 0.05$, **$P < 0.01$, ***$P < 0.001$, ****$P \leq 0.0001$), with comparisons made within the same age group.

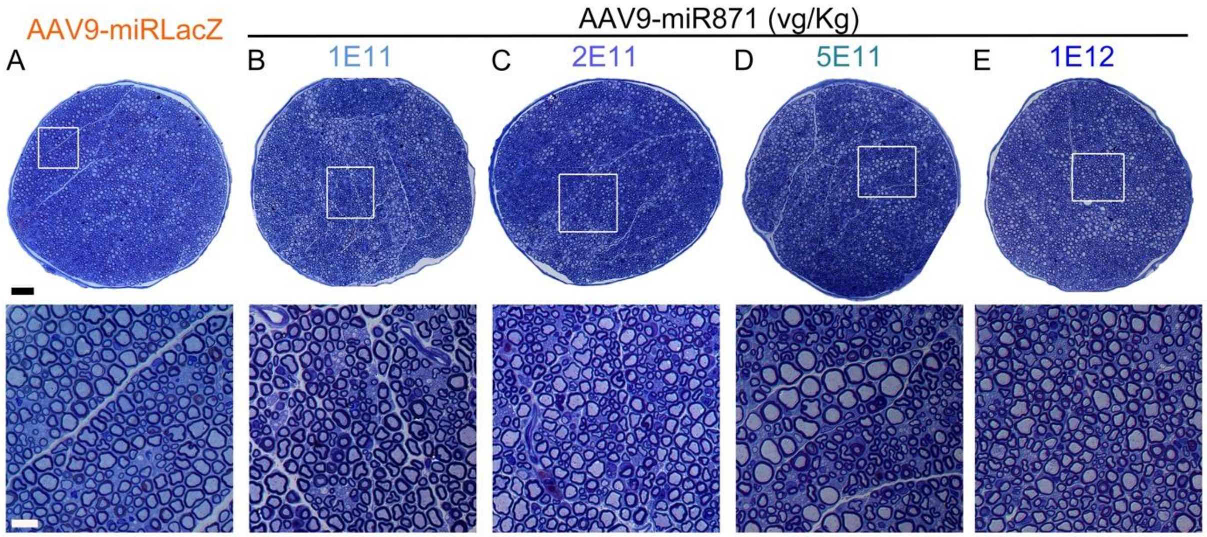

Figure S9. Representative images of toluidine blue-stained semithin sections of sciatic nerves from CMT1A mice following treatment with different AAV9.U6.miR871 doses.

(A-E) Toluidine blue-stained semithin sections of mid-sciatic nerves from CMT1A animals treated with AAV9.U6.miRLacZ or AAV9.U6.miR871 at doses of 1E11, 2E11, 5E11, or 1E12 vg/animal are shown at low (upper panels) and high magnifications (lower panels; n=5/group). Scale bars: 40 μm and 10 μm (enlarged inserts).

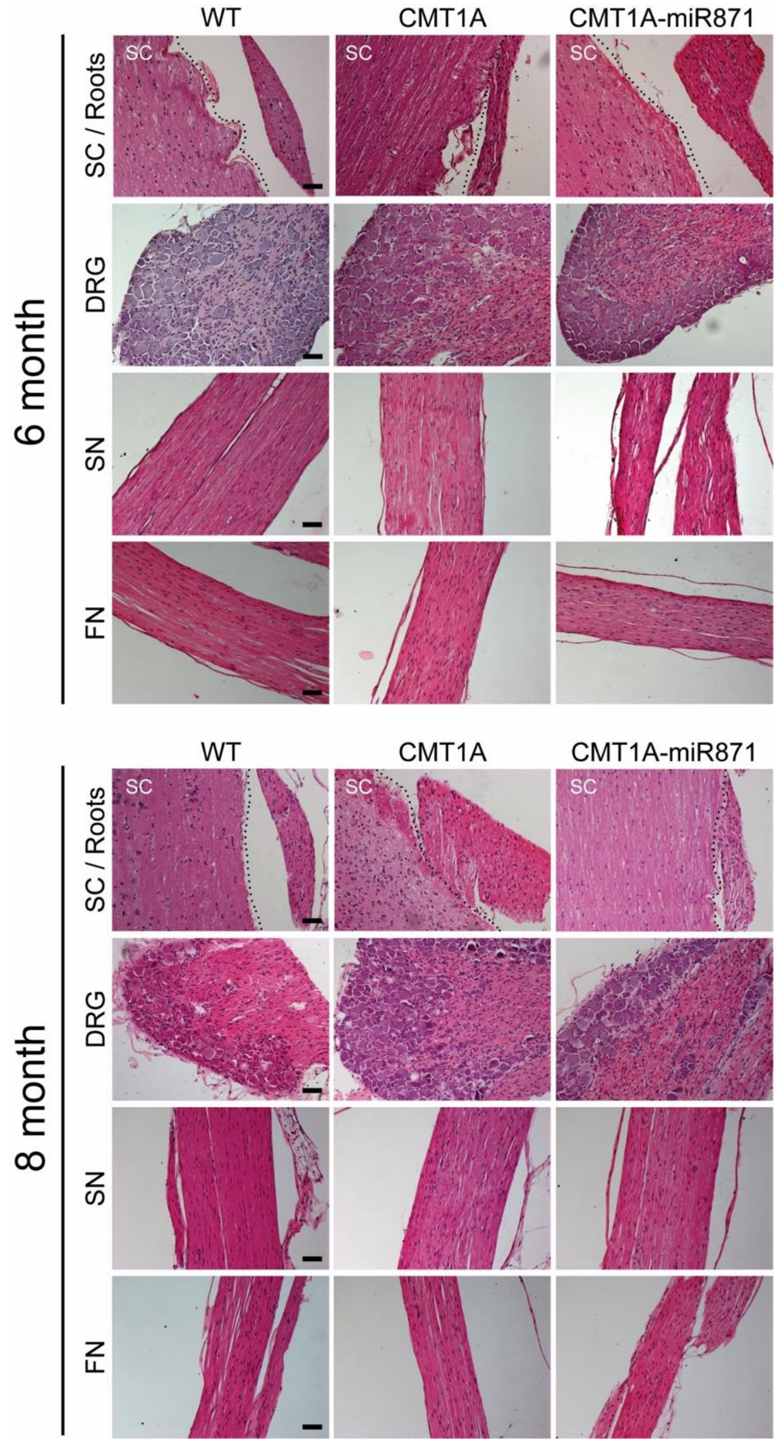

Figure S10. Representative H&E-stained images of nerve tissues from CMT1A mice following treatment with different AAV9.U6.miR871 doses. H&E staining of spinal cord (SC) and adjoining roots, DRGs, sciatic nerves, and femoral nerves from non-injected wild-type (WT), CMT1A mice, as well as CMT1A mice treated at 2 months of age with AAV9.U6.miR871. Animals were dosed at 1E11, 2E11, 5E11, or 1E12 vg/animal with the 1e12 vg/animal dose pictured here (n=4/group). Tissues were collected and analysed at 6 months (top panels) and 8 months (bottom panels) of age. Scale bar: 50 µm.

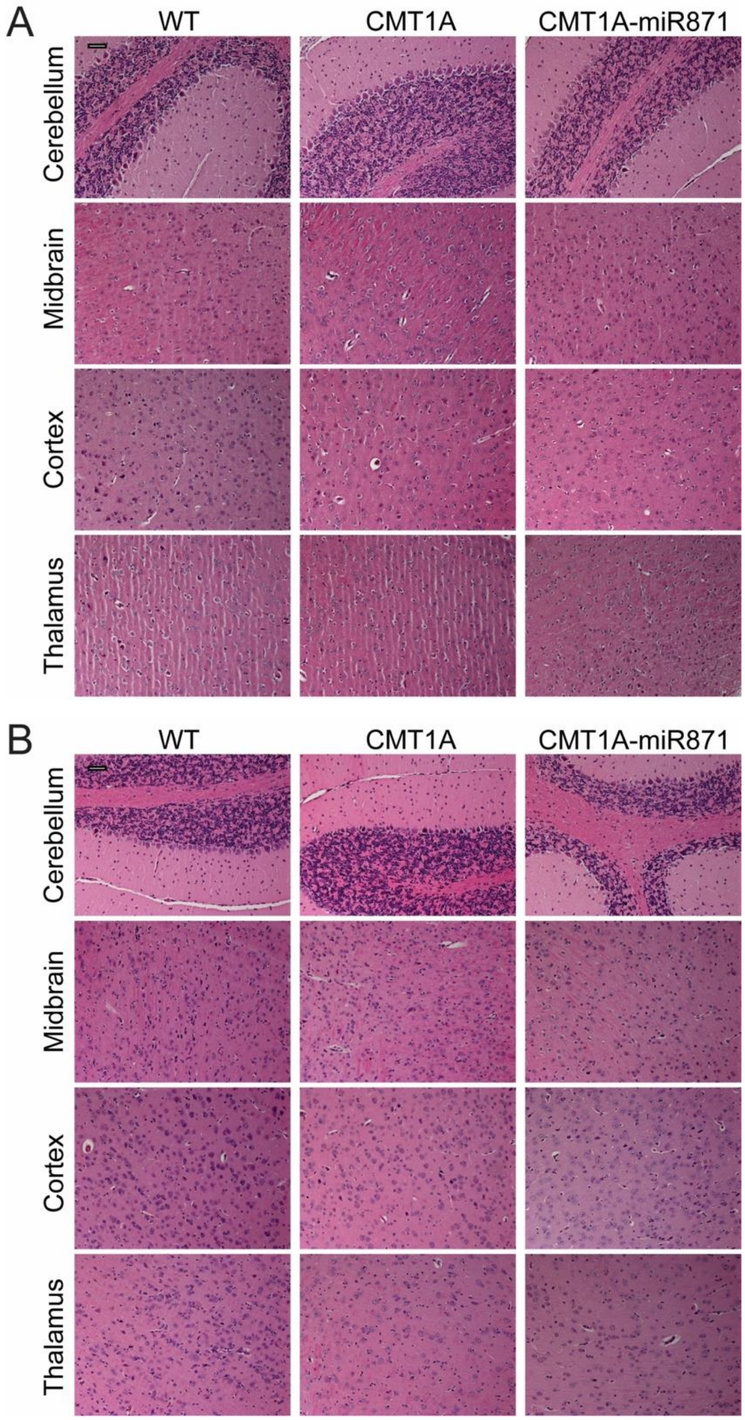

Figure S11. Representative H&E-stained images of brain areas from CMT1A mice following treatment with AAV9.U6.miR871.

H&E staining of cerebellum, midbrain brain, cortex and thalamus areas from un-injected wild-type (WT), CMT1A mice, as well as high dose (1E12 vg) representative images from CMT1A mice treated at 2 months of age as part of the AAV9.U6.miR871 dose response. Doses of 1E11, 2E11, and 5E11 vg/animal not shown (n=4/group). Tissues were collected and analysed at 6 months (A) and 8 months (B) of age. Scale bar: 50 µm.

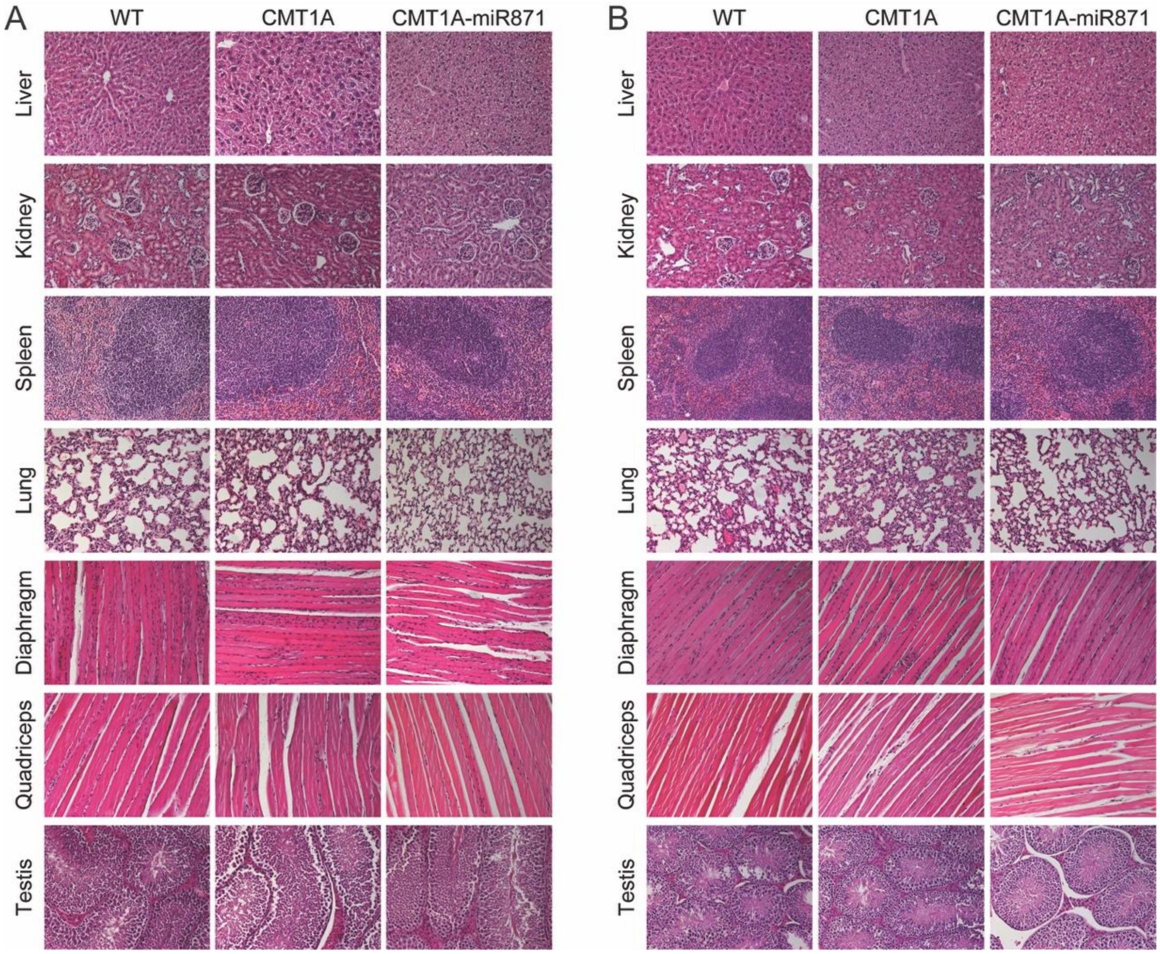

Figure S12. Representative H&E-stained images of off-target tissues from CMT1A mice following treatment with different AAV9.U6.miR871 doses.

H&E staining of organs from non-injected wild-type (WT), CMT1A mice, and CMT1A mice treated at 2 months of age with AAV9.U6.miR871. Dose response (1E11, 2E11, 5E11, or 1E12 vg/animal) represented here with the 1E12 vg dose represented (n=4/group). Tissues were collected and analysed at 6 months (A) and 8 months (B) of age. Scale bar: 50 μm.

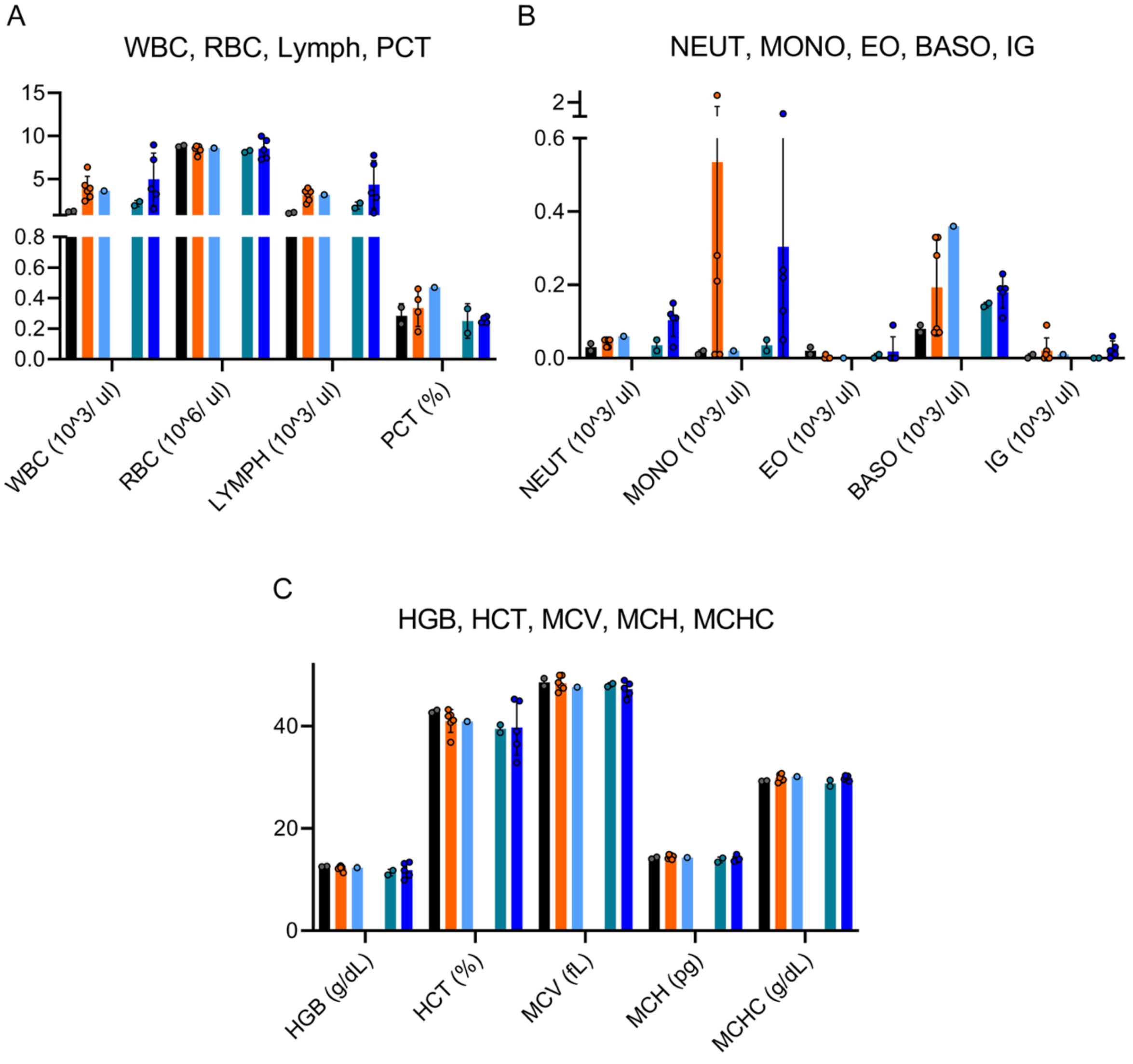

Figure S13. Comprehensive blood analysis of 6-month-old CMT1A mice following treatment with different AAV9.U6.miR871 doses.

Haematological and biochemical parameters were assessed in WT and CMT1A mice treated at 2 months of age with AAV9.U6.miRLacZ or AAV9.U6.miR871 at doses of 1E11, 2E11, 5E11, or 1E12 vg/animal. Blood samples were collected and analysed at 6-months of age (n=6/group). Parameters were analysed following our priority list (See Material and Methods). Blood analysis included (**A**) white blood cell (WBC), red blood cell (RBC), lymphocyte (Lymph) counts, and plateletcrit (PCT); (**B**) neutrophils (NEUT), monocytes (MONO), eosinophils (EO), basophils (BASO), and immature granulocytes (IG); (**C**) hemoglobin (HGB), hematocrit (HCT), mean corpuscular volume (MCV), mean corpuscular hemoglobin (MCH), and mean corpuscular hemoglobin concentration (MCHC).

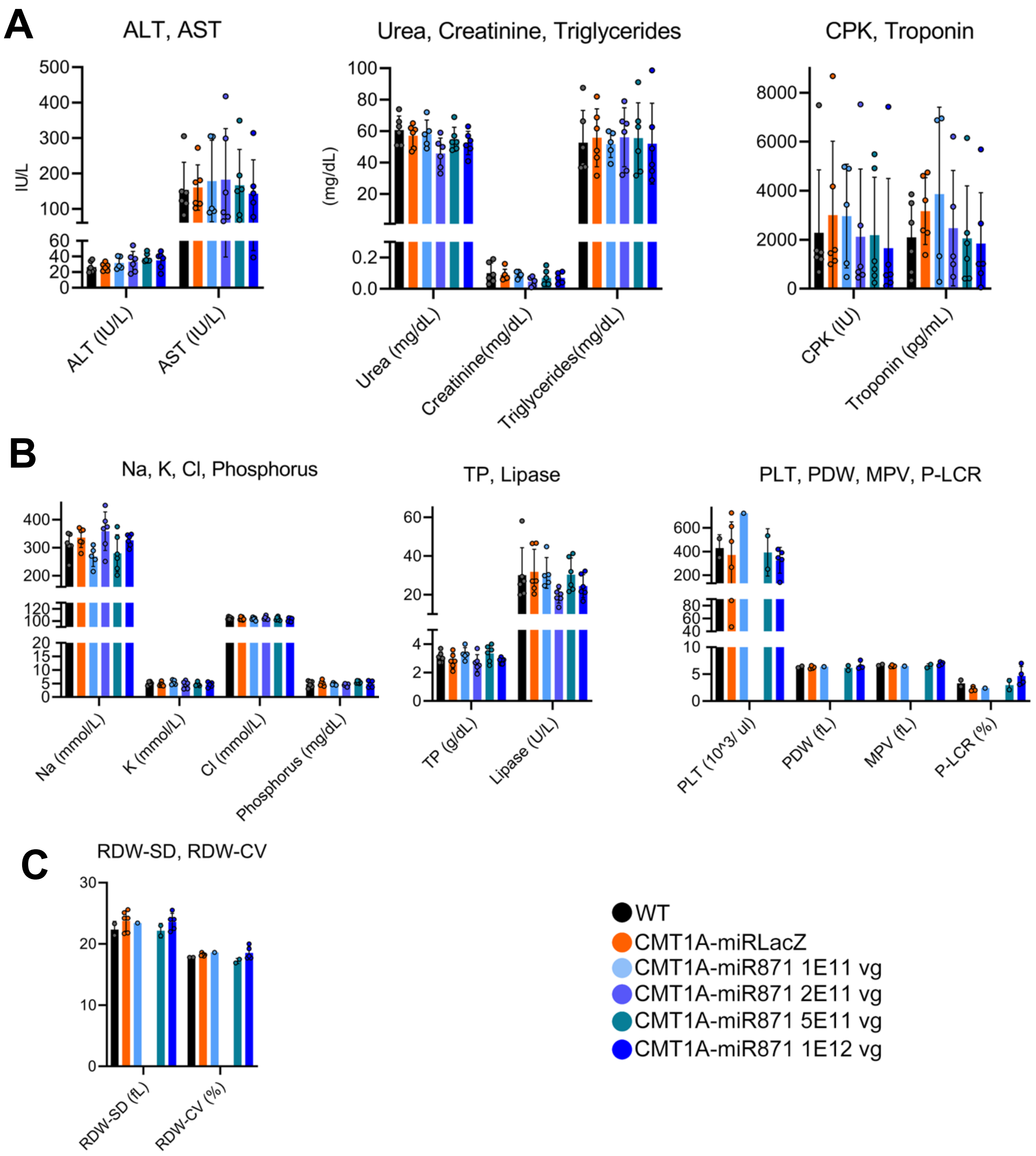

Figure S14. Comprehensive blood analysis of 6-month-old CMT1A mice following treatment with different AAV9.U6.miR871 doses.

(**A**) alanine aminotransferase (ALT) and aspartate aminotransferase (AST); urea, creatinine, and triglyceride levels; creatine phosphokinase (CPK) and troponin; (**B**) sodium (Na), potassium (K), chloride (Cl), and phosphorus (P); total protein (TP) and lipase; platelet count (PLT), platelet distribution width (PDW), mean platelet volume (MPV), and platelet large cell ratio (P-LCR); and (**C**) red cell distribution width-standard deviation (RDW-SD) and red cell distribution width-coefficient of variation (RDW-CV). Values are presented as mean ± SD. Statistical analysis was performed using one-way ANOVA with Tukey's multiple-comparison test, comparisons made within the same age group.

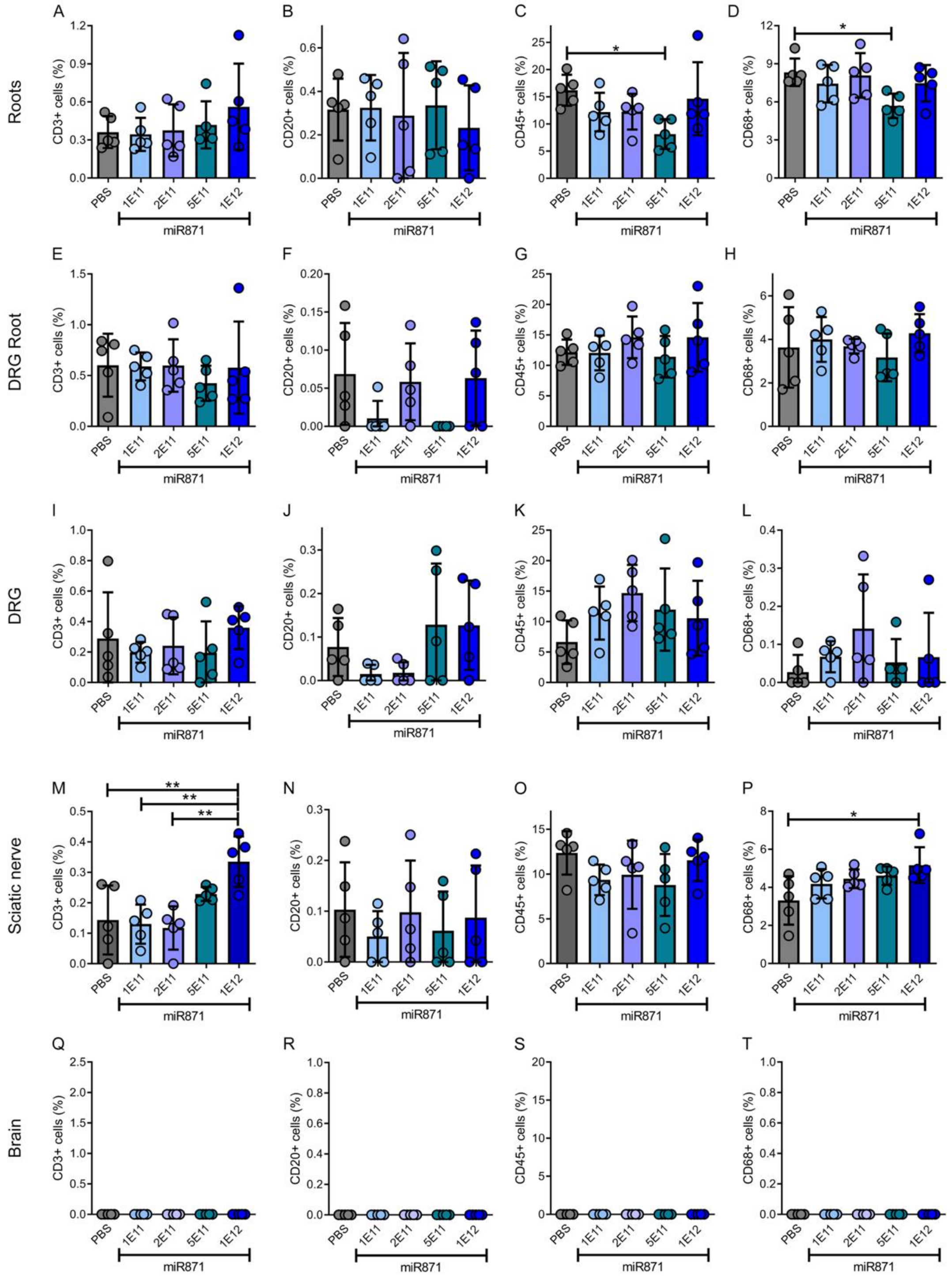

Figure S15. Inflammatory response in neural tissues of CMT1A mice following treatment with different AAV9.U6.miR871 doses.

Quantification of the percentage of cells positive for the CD3 T-cell marker, CD20 B-cell marker, CD45 leukocyte marker, and CD68 macrophage marker in immunostained (**A-D**) spinal roots, (**E-H**) DRG sensory roots, **(I-L)** DRGs, (**M-P**) sciatic nerves, and (**Q-T**) femoral nerves of CMT1A mice treated with indicated AAV9.U6.miR871 doses or PBS (n=5/group). Values are presented as mean ± SD. *P < 0.05, **P < 0.01, by 1-way ANOVA with Tukey's multiple-comparison test.

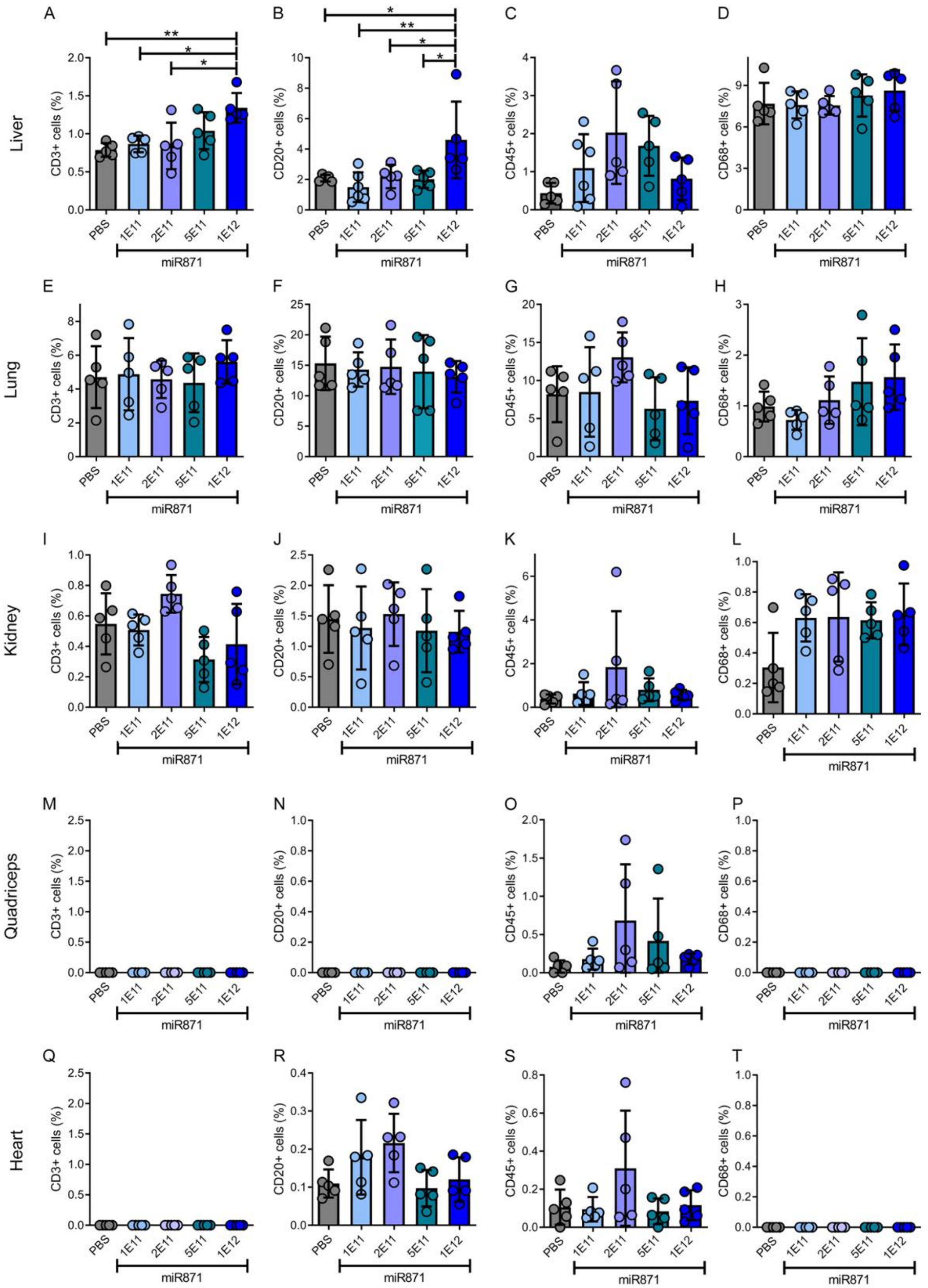

Figure S16. Inflammatory response in peripheral tissues of CMT1A mice following treatment with different AAV9.U6.miR871 doses.

Quantification of the percentage of cells positive for the CD3 T-cell marker, CD20 B-cell marker, CD45 leukocyte marker, and CD68 macrophage marker in immunostained (**A-D**) liver, (**E-H**) lung, (**I-L**) kidneys, (**M-P**) quadriceps and (**Q-T**) heart of CMT1A mice treated with indicated AAV9.U6.miR871 doses or PBS (n=5/group). Values are presented as mean ± SD. *$P < 0.05$, **$P < 0.01$, by 1-way ANOVA with Tukey's multiple-comparison test.

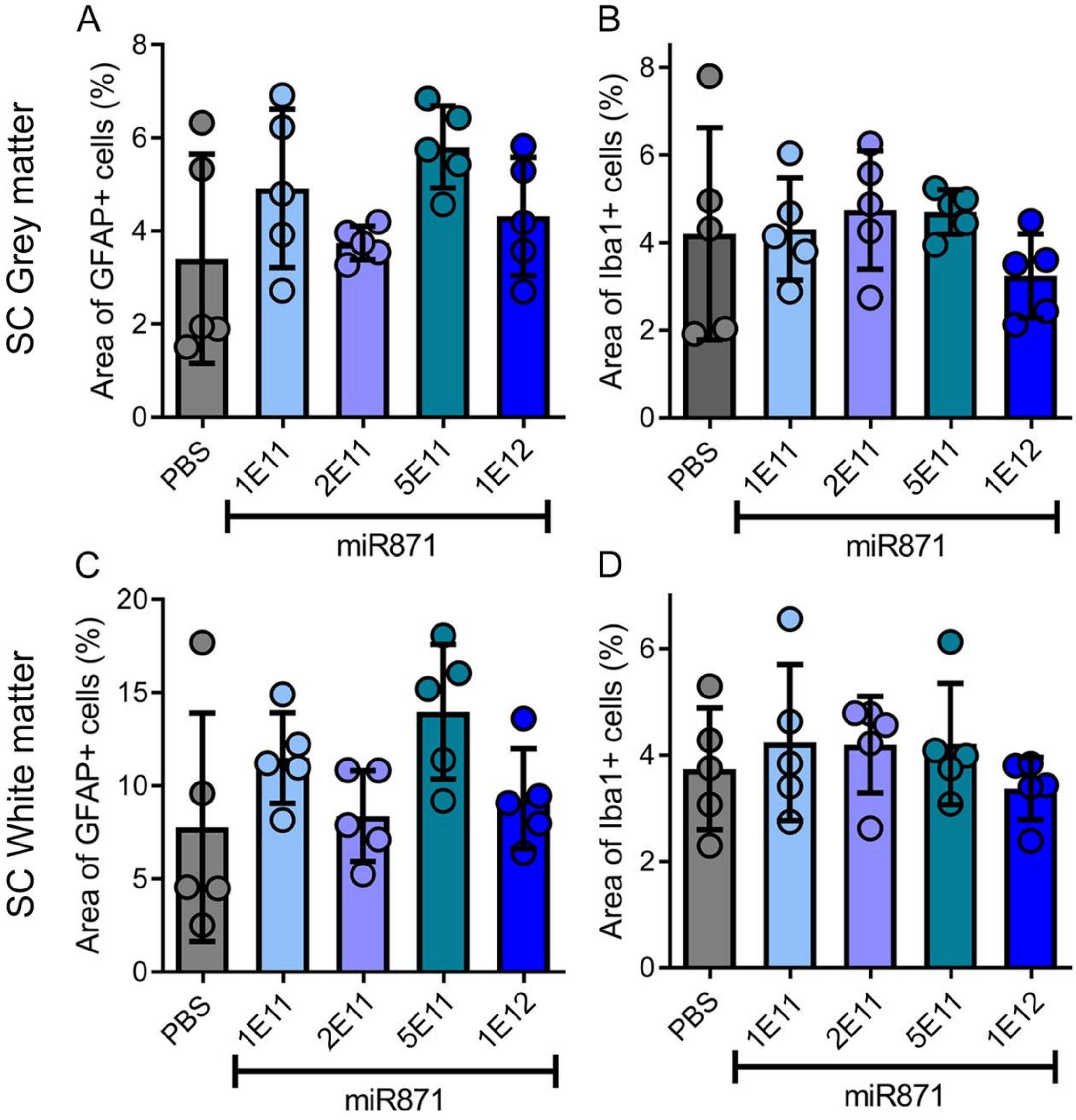

Figure S17. Lack of inflammatory side effects in spinal cord of CMT1A mice following treatment with different AAV9.U6.miR871 doses.

Quantification of the percentage of cells positive for GFAP astrocyte marker and Iba1 microglia marker in the (**A,B**) grey and (**C,D**) white matter of immunostained spinal cord (SC) of CMT1A mice treated with indicated AAV9.U6.miR871 doses or PBS (n=5/group). Values are presented as mean ± SD and compared by 1-way ANOVA with Tukey's multiple-comparison test.

Figure S18. Mouse toxicology report from Transcendent Bio. 4 page pathology report in PDF form, uploaded as a separate supplemental file.

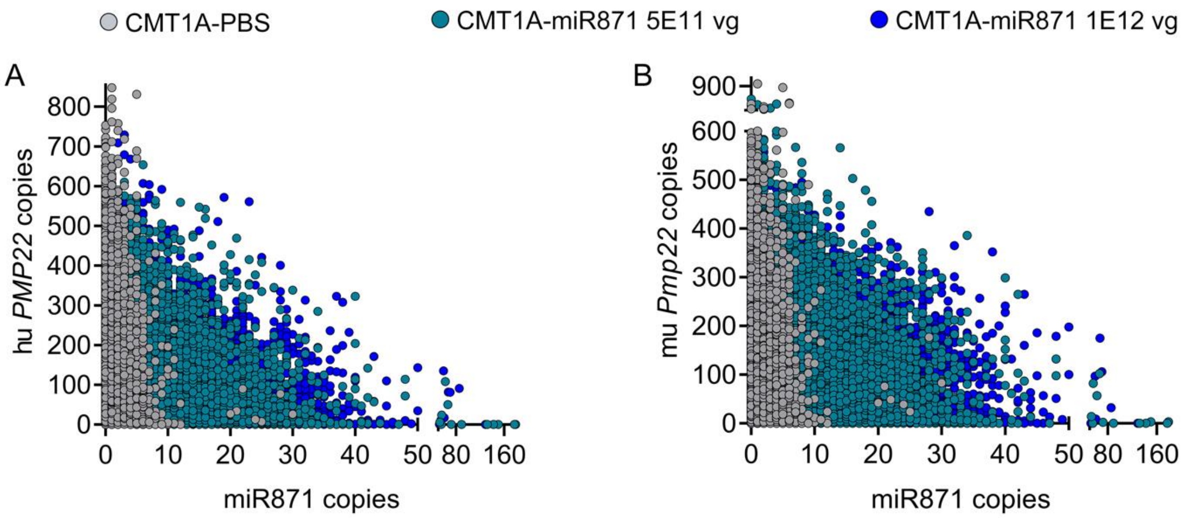

Figure S19. Single cell resolution RNAscope Plus™ analysis of miR871 co-detection with huPMP22 or muPmp22 in the sciatic nerves of CMT1A mice injected with 5E11 or 1E12 vg/animal of AAV9.U6.miR871 or PBS. RNAscope Plus™ analysis was performed to assess the co-localization of miR871 with (**A**) human *PMP22* or (**B**) murine *Pmp22* in the sciatic nerves of CMT1A mice treated with AAV9.U6.miR871 (5E11 or 1E12 vg/animal) or PBS (n=6 animals/group, n≥147,000 cells/group). Quantitative image analysis was conducted to evaluate transcript expression at single-cell resolution. Data points represent individual cells, with PBS-treated animals shown in grey, 5E11 vg/animal in turquoise, and 1E12 vg/animal in blue. Due to overlapping signals, PBS-treated cells appear on top, followed by those receiving 5E11 vg/animal, with 1E12 vg/animal-treated cells at the bottom.

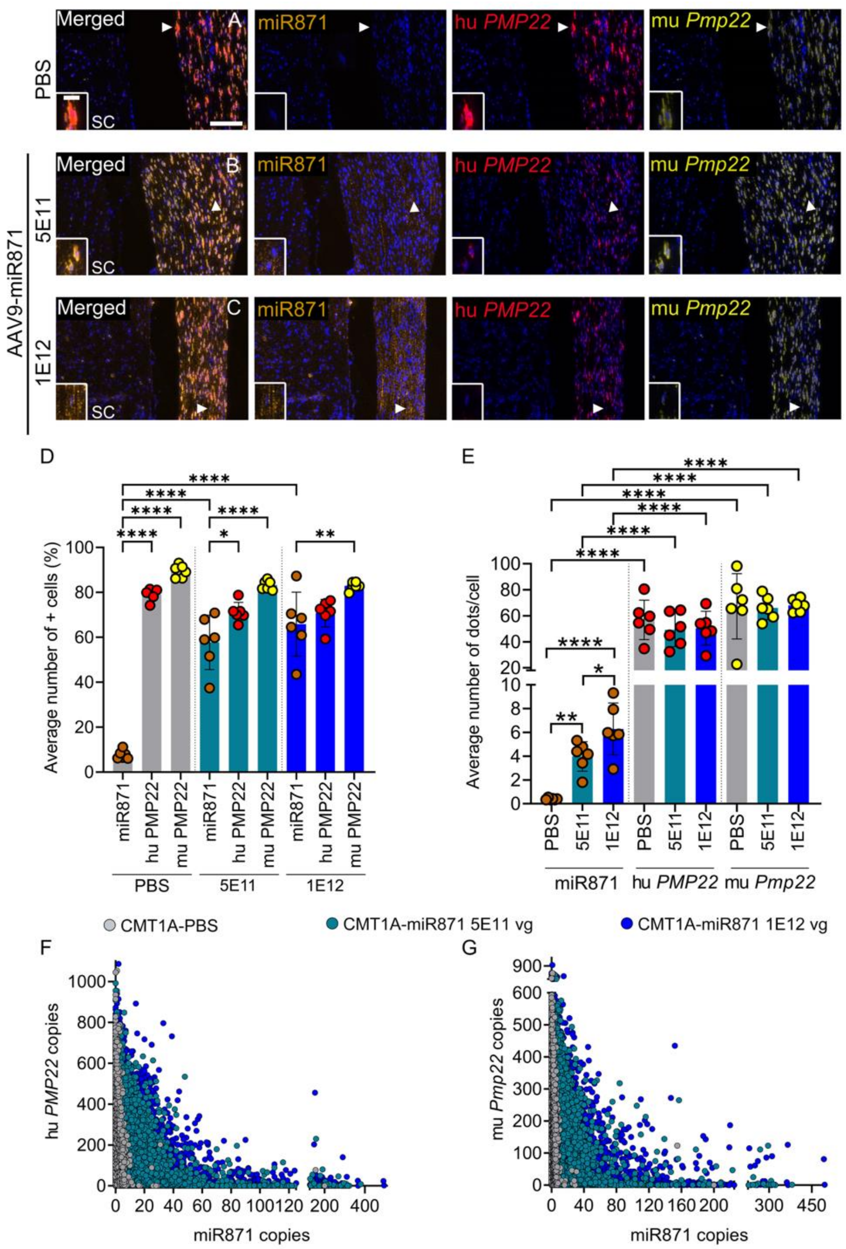

Figure S20. RNAscope Plus™ analysis for the co-detection of miR871, hu*PMP22*, and mu*Pmp22* in the spinal roots of CMT1A mice injected with 5E11 or 1E12 vg/animal of AAV9.U6.miR871 or PBS. (**A-C**) Representative images of miR871, hu*PMP22*, and mu*Pmp22* staining in spinal roots adjoining the spinal cord (SC) of CMT1A mice treated with 5E11 or 1E12 vg/animal AAV9.U6.miR871 (n=6 animals/group, n≥139,000 cells/group). **(D-E)** Quantitative image analysis was performed using HALO® software and whole-tissues scanning used to determine average numbers of miR871-, hu*PMP22*-, and mu*Pmp22*-positive cells as well the average number of corresponding miR871, hu*PMP22*, and mu*Pmp22* dots/cells in CMT1A animals treated with AAV9.U6.miR871 (5E11 or 1E12 vg/animal) or PBS. (**F-G**) Single-cell resolution data of individual cells. Due to overlapping signals, PBS-treated cells appear on top, followed by those receiving 5E11 vg/animal, with 1E12 vg/animal-treated cells at the bottom. For D and E, values are presented as mean ± SD. *P < 0.05, **P < 0.01, ***P < 0.001, ****P ≤ 0.0001 by 1-way ANOVA with Tukey's multiple-comparison test. Scale bar: 50 μm and 15 μm (enlarged inserts).

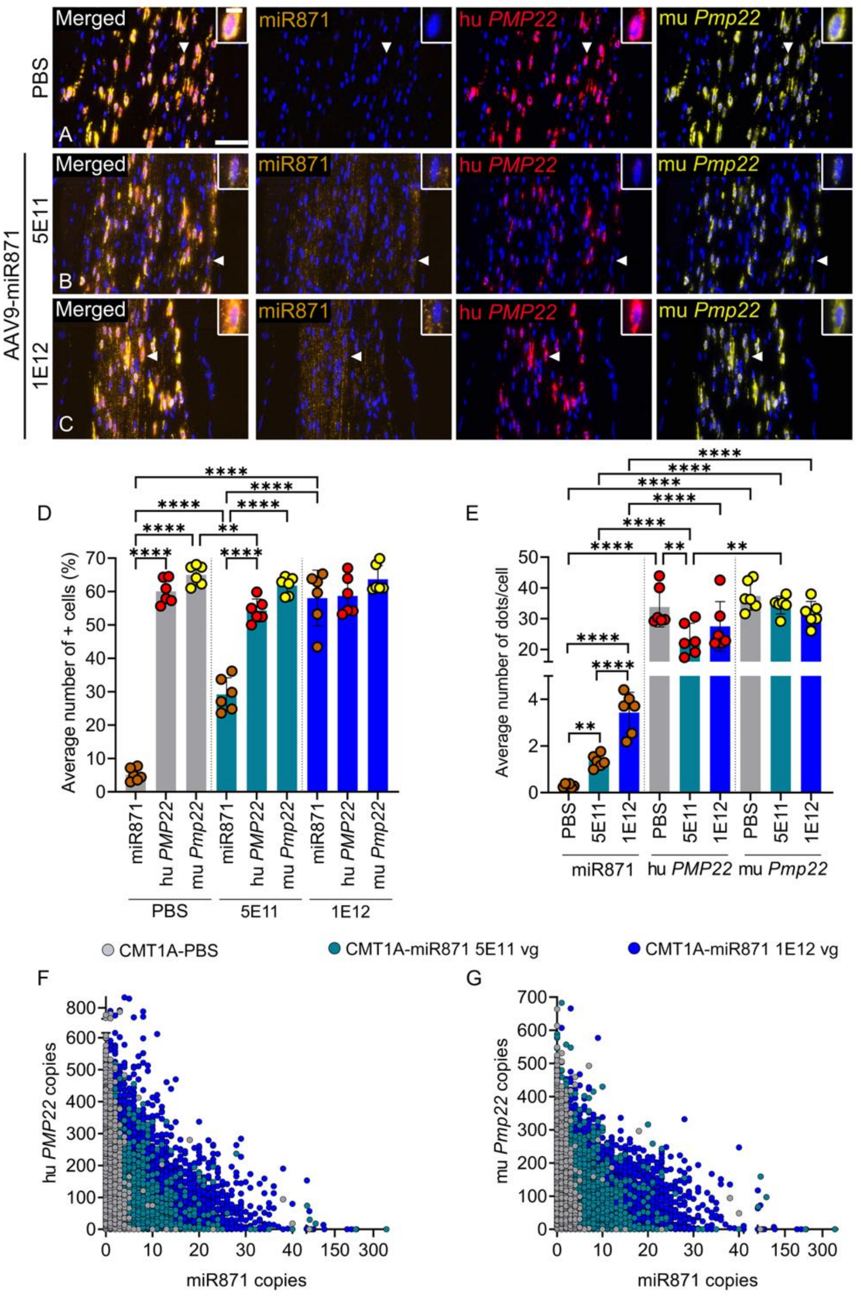

Figure S21. RNAscope Plus[TM] analysis for the co-detection of miR871, hu*PMP22*, and mu*Pmp22* in the femoral nerves of CMT1A mice injected with 5E11 or 1E12 vg/animal of AAV9.U6.miR871 or PBS. (**A-C**) Representative images of miR871, hu*PMP22*, and mu*Pmp22* staining in femoral nerves of CMT1A mice treated with 5E11 or 1E12 vg/animal of AAV9.U6.miR871 (n=6 animals/group, n≥42,900 cells/group). (**D-E**), Quantitative image analysis was performed using HALO® software and whole-tissues scanning used to determine average number of miR871-, hu*PMP22*-, and mu*Pmp22*-positive cells as well the average number of corresponding miR871, hu*PMP22*, and mu*Pmp22* dots/cells in CMT1A animals treated with AAV9.U6.miR871 (5E11 or 1E12 vg/animal) or PBS. (**F-G**) Single-cell resolution data of individual cells. Due to overlapping signals, PBS-treated cells appear on top, followed by those receiving 5E11 vg/animal, with 1E12 vg/animal-treated cells at the bottom. For D and E, values are presented as mean ± SD. $*P < 0.05$, $**P < 0.01$, $***P < 0.001$, $****P \leq 0.0001$ by 1-way ANOVA with Tukey's multiple-comparison test. Scale bar: 50 µm and 15 µm (enlarged inserts).

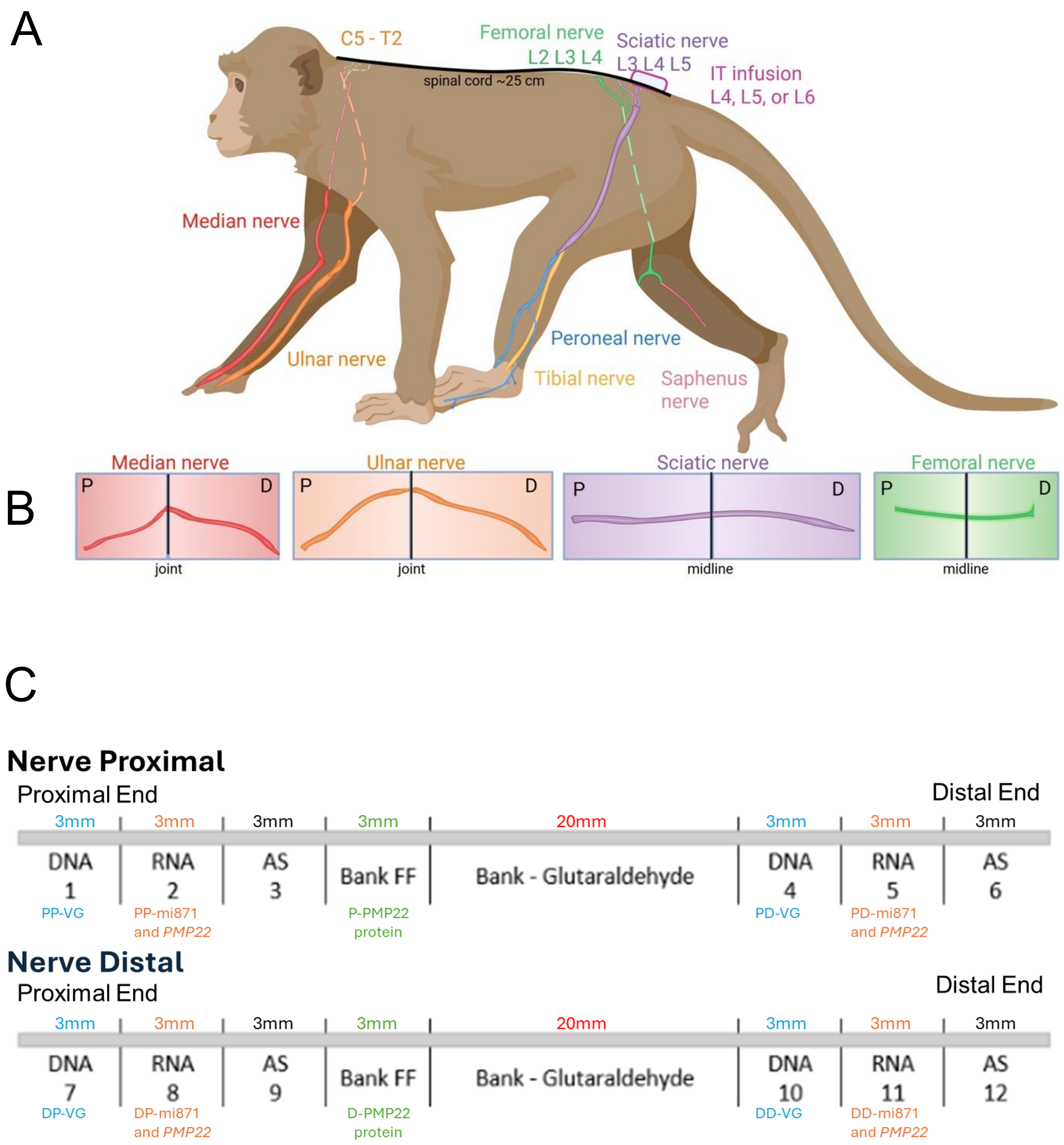

Figure S22. NHP nerve analysis design (A) Schematic of intrathecal (IT) infusion site relative to collected nerves and their innervation points. (B) Proximal and distal nerve designations were determined by the joint for the upper limb nerves (median nerve and ulnar nerve) and the midline of the fully dissected hindlimb nerves (sciatic nerve and femoral nerve). (C) Schematic of nerve dissection with minimal length per segment and assay designation; ≥3mm for each fresh fozen segment and ≥20mm for each fixed segment. Total nerve designations are ≥41mm for each individual proximal or distal segment. Vector genome analysis (VG); Probes for rt-dPCR gene of interest assays (mi871 and *PMP22);* alternative samples collected for as needed molecular assays (AS); segment for protein analysis, bank fresh frozen (Bank FF); proximal nerve proximal section (PP); proximal nerve distal section (PD); proximal (P), distal nerve proximal section (DP); distal nerve distal section (DD); distal (D).

Figure S23. Non-human primate pathology report from Amplify Bio. PDF file containing 1868 pages, uploaded as a separate supplemental file.

A

B

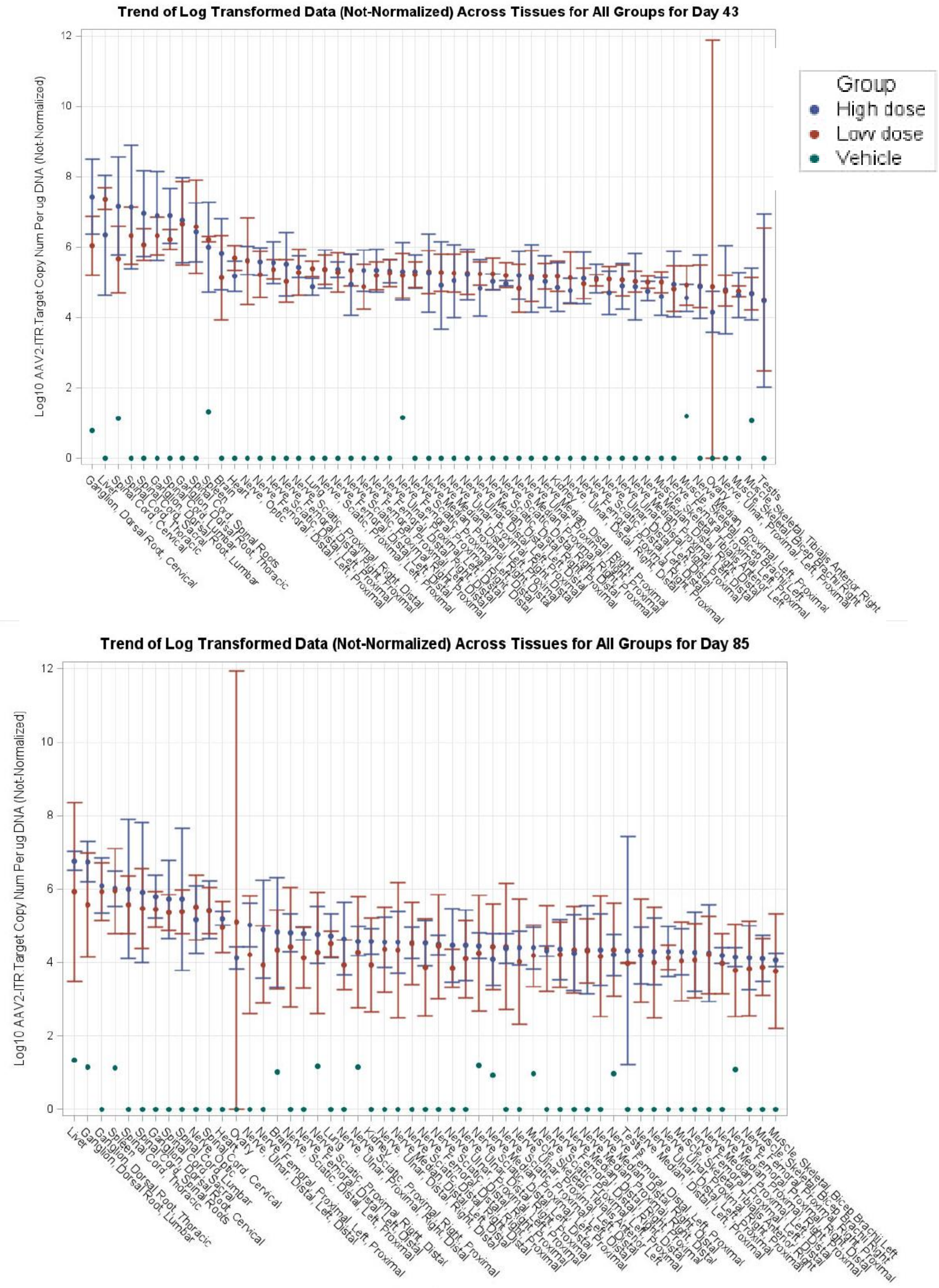

Figure S24. AAV biodistribution in all NHP tissues collected. Data show trend of log transformed AAV2-ITR mean target copy number per µg DNA. (A) 6 week and (B) 12 week, across all indicated tissues for all groups. Error bars for the low dose and high dose groups are calculated based on the 95% confidence intervals for the mean of each of these groups. Error bars have been omitted for the control group.

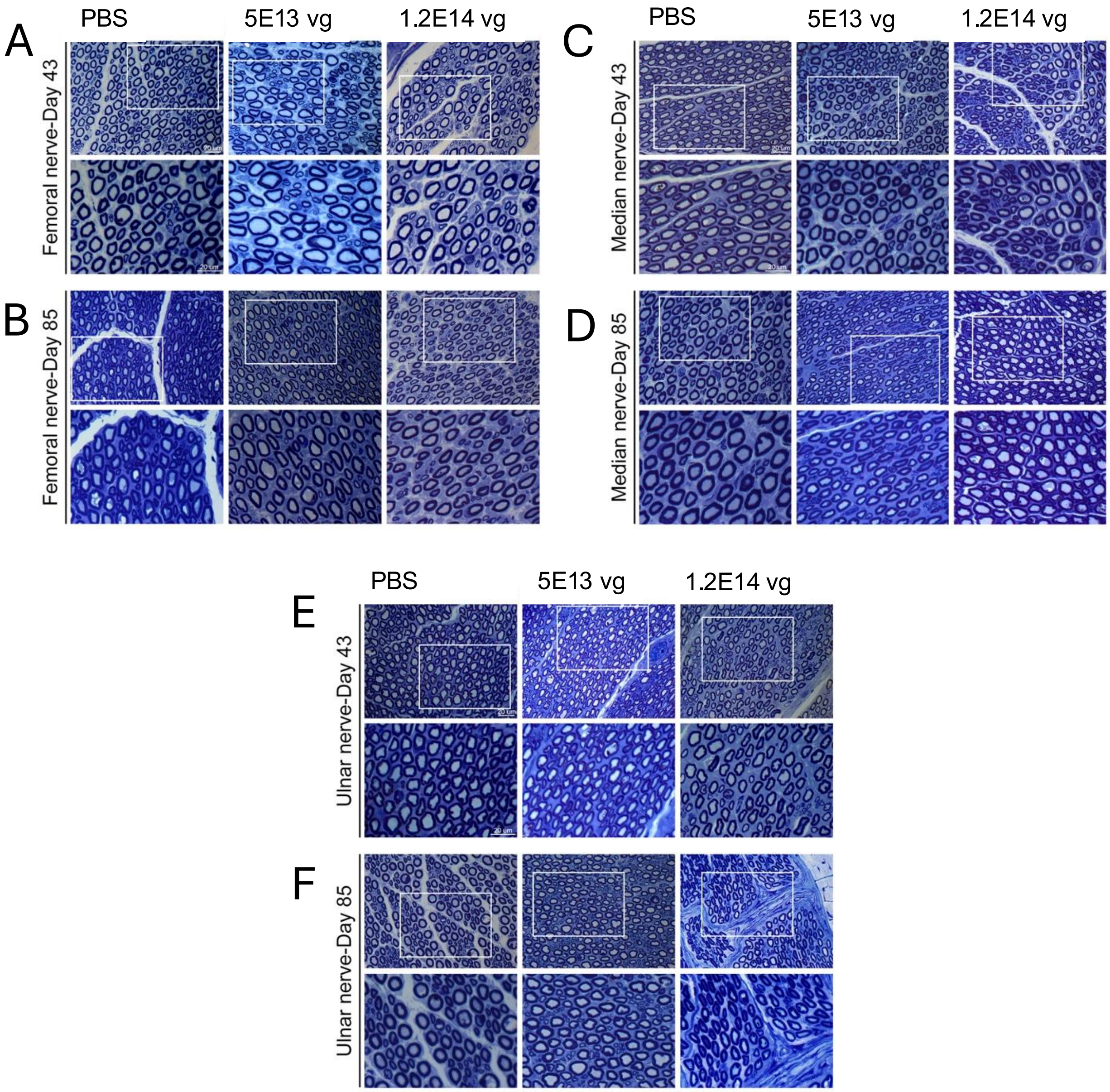

Figure S25. Representative images of NHP nerve sections stained with toluidine blue following treatment with AAV9.U6.miR871. Toluidine blue-stained semithin sections of (A-B) femoral nerves, (C-D) median nerves or (E-F) ulnar nerves 6-weeks or 12-weeks after lumbar intrathecal delivery to wild type Cynomolgus macaques. Paired images represent low (upper panels) and high magnifications (lower panels). Scale bars: 20 µm.

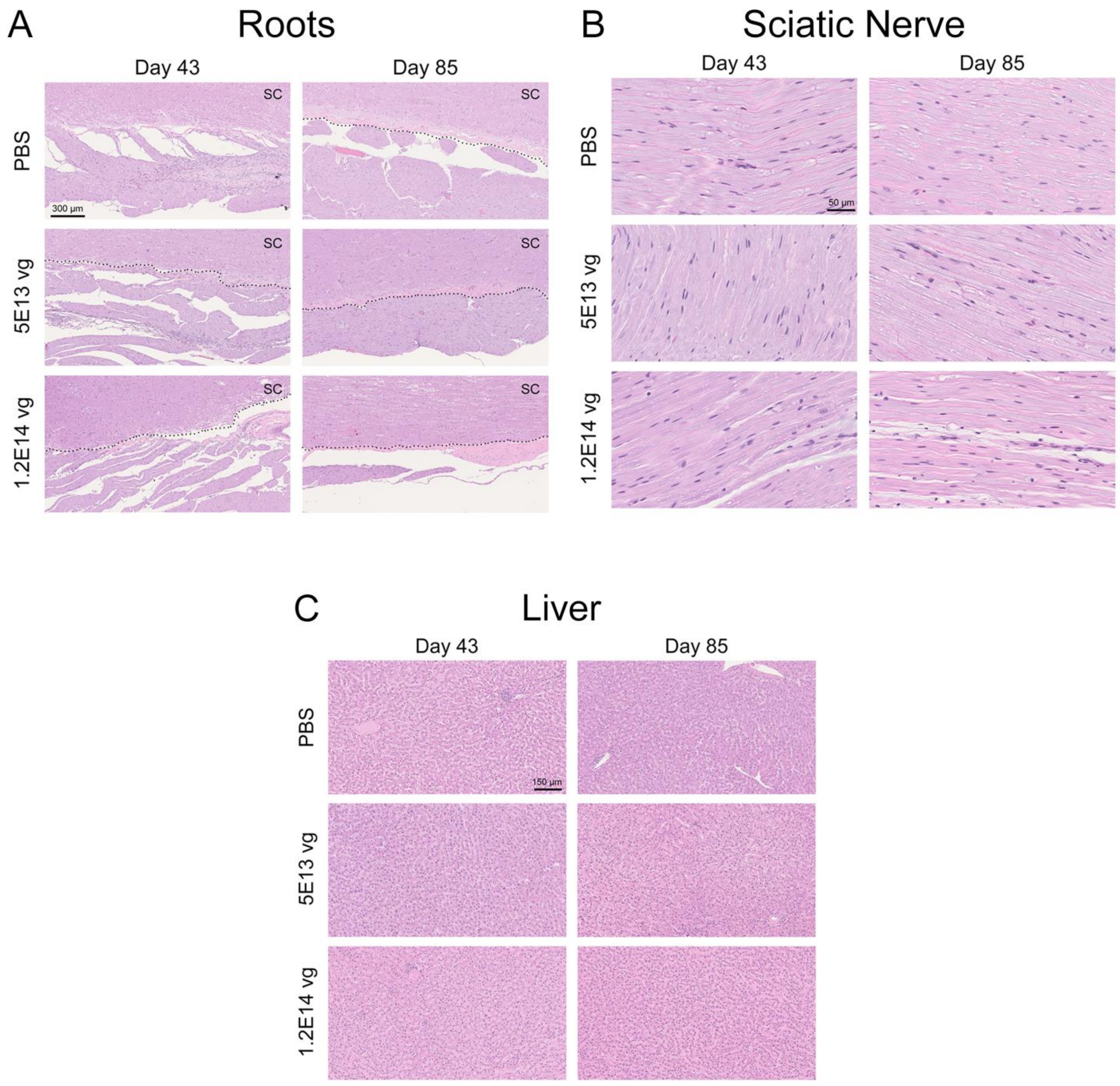

Figure S26. Representative H&E-stained images of spinal nerve roots, sciatic nerve, and liver following treatment of indicated AAV9.U6.miR871 doses.

H&E staining of spinal roots (A) attached to spinal cord, sciatic nerve (B), and liver (C) 6- and 12-weeks after lumbar intrathecal delivery in NHPs. No significant histopathological findings were noted in treated animals. Scale bar: 50 µm.

Figure S27. All raw western blots from NHP target engagement studies. PDF file containing 128 pages of images, uploaded as a separate file.

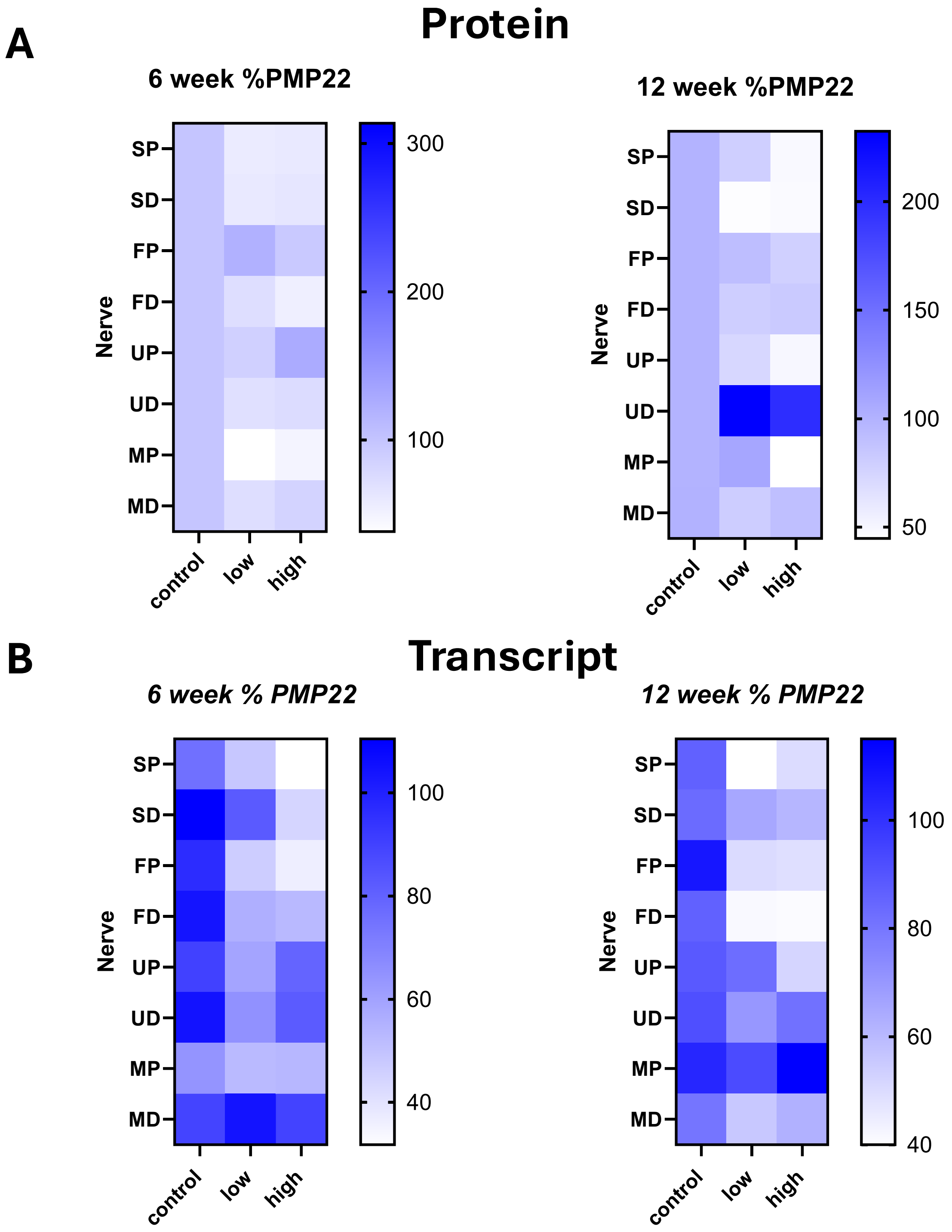

Figure S28. Heatmaps summarizing *PMP22* mRNA and PMP22 protein reductions in NHP nerves. (A) Heatmaps of average PMP22 protein level normalized to control nerve at 6- and 12-wk timepoints. (B) Heatmaps of average *PMP22* transcript normalized to total control average. Overall, reduction of protein and/or transcript indicates evidence for gene silencing in all samples tested.
