# Supplementary material for: Safety, efficacy, and distal nerve Schwann cell biodistribution in mice and NHPs to support translation of AAV9 RNAi therapy for CMT1A
Source: Mol Ther Nucleic Acids. 2026 Feb 27;37(2):102881. doi: 10.1016/j.omtn.2026.102881 (PMC13051718; doi:10.1016/j.omtn.2026.102881)
Supplement: Figure S23. Non-human primate pathology report from Amplify Bio [file mmc3.pdf]

A 12-Week Single Dose Biodistribution and Safety Study in Cynomolgus Monkeys

Final Report

November 1, 2024

Study Number A-579-01

Armatus Bio, Inc.
1275 Kinnear Road, Columbus, OH 43212

GLP Compliance Status: GLP

AmplifyBio
1425 State Route 142 (Plain City-Georgesville Road)
West Jefferson, OH 43162

SIGNATURES

Signed by:
Sapna Varia
Signer Name: Sapna Varia
Signing Reason: I approve this document
Signing Time: 11/1/2024 | 9:35:01 AM EDT
5D64C6F0F9AF4A7B9EBF31D648E66020

11/1/2024

Sapna Varia, PhD<br>Study Director	Date

Signed by:
Dean Kobs
Signer Name: Dean Kobs
Signing Reason: I approve this document
Signing Time: 11/1/2024 | 9:25:59 AM EDT
1260DA197A6046E19CA90C7D22002FCC

11/1/2024

Dean J. Kobs, MS<br>VP of CRO Operations<br>Testing Facility Management	Date

SUMMARY

The purpose of this study was to evaluate the potential biodistribution and safety of scAAV9.U6miR871 (ARM-101) when administered once via intrathecal infusion to Cynomolgus monkeys for 12 weeks.

Twenty Cynomolgus monkeys (10 males and 10 females) were randomly assigned to one of three dose groups. Four animals in Group 1 received administration of the control article (Formulation Buffer), whereas 8 animals in Group 2 received administration of 6×10^{13} vg (Low Dose) of scAAV9.U6miR871 (ARM-101) in 0.9% Saline and 8 animals in Group 3 received administration of 1.2×10^{14} vg (High Dose) of scAAV9.U6miR871 (ARM-101) in 0.9% Saline. Dose was administered to each animal (dose volume of 3 mL/animal) as a single dose via intrathecal slow infusion over approximately 6 hours via a pre-study implanted catheter.

Following a single dose administration, 10 animals from the 6-week cohort were terminated on Day 43 and the remaining 10 animals from the 12-week cohort were terminated on Day 85.

Endpoints evaluated were clinical observations, body weights, food consumption, non-invasive neurological observations, nerve conduction velocity (NCV), ophthalmic examinations, electrocardiogram (ECG) evaluations, clinical pathology (hematology, coagulation, serum chemistry, and urinalysis), troponin I analysis, cytokine analysis (ELISpot), biodistribution analysis (d-PCR and RT-dPCR), and anatomic pathology, including organ weights, gross observations at necropsy, and microscopic evaluation of selected tissues.

All animals survived until scheduled termination.

There were no test article-related body weight, food consumption, or ocular changes observed in any of the groups during the study. There were no adverse clinical observations attributed to the test article dose administration.

There were no test article-related nerve conduction findings observed in the tibial nerve post dose administration. There were no apparent test article-related effects observed in the qualitative assessment of ECG waveforms when comparing dose groups or comparing pre-dose recordings of each cohort to subsequent Week 6 (Day 43) and/or Week 12 (Day 85) recordings.

There were no test article-related neurobehavioral findings observed in any dose group during the study. Results from the study indicate the changes in the score were ambiguous and merely a change of a score by a factor of 1 in animals both in the control group as well as in the those receiving scAAV9.U6miR871 (ARM-101).

There were no test article-related findings noted in the hematology, coagulation, serum chemistry, or urinalysis data.

Cardiac Troponin I (cTnI) analysis on all available samples from control and each dose group of 6-week and 12-week cohort animals demonstrated minimal detectable values of troponin I indicating there were no myocardial injuries.

In the 6-week cohort (Day 43), no test article-associated gross lesions were identified. In the 12-week cohort (Day 85), one female had enlarged inguinal and mesenteric lymph nodes which corresponded to the microscopic finding of increased cellularity, lymphocyte. Remaining gross lesions were common background lesions observed in nonhuman primates unrelated to treatment.

The 6-week or 12-week cohort animals had minimal test article-related microscopic findings which were limited to mild lesions within the spinal cord and dorsal root ganglion. Lesions were present in the 6-week and 12-week cohorts to the same or similar degree. None of the animals receiving the test article displayed any clinical signs. Autophagy noted in several dorsal root ganglions represents programed cell death of neurons and is a common background finding in nonhuman primates but may also follow lethal injury to sensory cells. Intracytoplasmic vacuolization of neurons as seen in this study is consistent with fixation artifact.

There were no identifiable test article-related trends in any group for IFNë ELISpot evaluation. While responses to positive control mitogen conditions were consistent across all study animals and test plates, none of the study animals showed detectable responses to the test antigen AAV9 at the Pretest, 6-week, or 12-week time points.

Biodistribution of the vector AAV2-ITR of ARM-101 test article upon intrathecal slow infusion showed greater prevalence of vector on Day 43 compared to Control (mean log copies higher than the Day 85 mean) in the Central Nervous System (CNS) (spinal cord, dorsal root ganglia, and brain), liver, and heart. All the nerve tissues (Femoral, Median, Sciatic, and Ulnar) and spleen showed high vector copies per µg DNA at Day 43 and persisted until Day 85. There was no dose response observed in dosed groups but showed very high vector copies when compared to the Control group. There were no sex-related differences observed in the mean copies of the vector genome in males and females.

Biodistribution of the miR871 transgene of ARM-101 test article showed greater prevalence of the transgene on Day 43 for both low dose and high dose compared to Control in the CNS (spinal cord, dorsal root ganglia, femoral nerve, median nerve, sciatic nerve, ulnar nerve, and brain) and liver. This high expression was observed to be persistent until Day 85. There was minimal dose response observed in dosed groups but showed significantly high miR871 transgene expression when compared to the Control group. Examining the differences between miR871 and PMP22 for nerve tissues for log-transformed data normalized to B2M across different tissues showed correlation between high miR871 expression to reduced endogenous PMP22 expression.

In conclusion, a single intrathecal dose administration of scAAV9.U6miR871 (ARM-101) via a single approximate 6-hour slow infusion at 6×10^{13} vg/animal (low dose) or 1.2×10^{14} vg/animal (high dose) to male and female Cynomolgus monkeys was well tolerated and deemed safe over 43 days (6-week cohort) and 85 days (12-week cohort). Additionally, the highest scAAV9.U6miR871 vector accumulation was observed in the spinal cord, dorsal root ganglion, liver, and heart. All the nerve tissues (Femoral, Median, Sciatic, and Ulnar) also showed high concentration of the vector at Day 43 for high dose animal tissues which was observed to persist through Day 85. ARM-101 vector administration showed reduction in

endogenous PMP22 gene expression at both low (6×10^{13} vg) and high (1.2×10^{14} vg) doses compared to control group in nerve tissues and the reduced expression persisted until Day 85.

COMPLIANCE STATEMENT

This study was conducted in compliance with the current version of the United States Food and Drug Administration's (FDA) Good Laboratory Practice (GLP) Regulations, 21 CFR Part 58 for the conduct of non-clinical laboratory studies with the following exceptions:

- The test/control article were research grade products and were not manufactured and characterized according to GLP compliance. A Certificate of Analysis (CoA) was provided.
- Test article characterization was not performed under GLP compliance.
- Dose formulation analysis was not performed as the test article was received as ready to dose and diluted to the required concentration with the vehicle (0.9% saline).
- Stability of the bulk or diluted test article was not performed or reported.
- The control article formulations were not analyzed for homogeneity or concentration of test article. Control article formulations were not subjected to stability testing.
- The results from Cerebrospinal fluid (CSF) analysis or Bank tissues were not reported under this study. The Cerebrospinal fluid specimens, retained PBMC aliquot(s), residual/retained biodistribution samples and Bank tissue samples for potential analysis were shipped to the Sponsor-designee on dry ice or LN2 shipper for potential future evaluation by the Sponsor. This additional work did not have an impact on the conclusions of this study because the Cerebrospinal fluid specimens, retained PBMC aliquot(s), residual/retained biodistribution samples and Bank tissue (shipped to the Sponsor-designee) analyses did not impact the assessment of the safety and biodistribution evaluation of the test article. This analysis and evaluation did not interfere with the conduct of this study and did not jeopardize the GLP compliance of this study. The analysis performed on these samples were not considered to be part of this study and the results were not presented in this study report. The samples and the results from analysis of the samples are under the responsibility of the Sponsor.
- Nicolet Viking EDX system and associated software used for nerve conduction study was not validated.
- The method used for biodistribution analysis by Digital PCR (RT-dPCR) was not qualified.
- Statistical analysis for dPCR and RT-dPCR was not performed according to GLP compliance.
- On 23Dec2023, there were statistical methods performed using the dPCR data for study A-579-01, and these methods were not described in the protocol or other guiding document prior to use. The statistical analysis was performed as part of module 2 of the dPCR data pipeline. The statistical methods included ANOVA and estimated marginal means. There is no impact to the study due to no guiding document for the statistical analysis effective prior to protocol amendment as the data will be re-analyzed and hence there is no impact to the integrity of the data (DEV-002537 in Appendix A).

The study was listed on AmplifyBio's master list of regulated studies. All portions of this study performed at AmplifyBio adhered to the study protocol, amendments, and applicable standard operating procedures (SOP). Deviations from GLP, the study protocol, or selected SOP and study specific methods are summarized in in Appendix A. All other SOP and study specific method deviations are maintained in the study file.

Signed by:
Sapna Varia
Signer Name: Sapna Varia
Signing Reason: I approve this document
Signing Time: 11/1/2024 | 9:35:10 AM EDT
5D64C6F0F9AF4A7B9EBF31D648E66020

11/1/2024

Sapna Varia, PhD<br>Study Director	Date

QUALITY ASSURANCE STATEMENT

This study was inspected by the Quality Assurance Unit (QAU) at AmplifyBio and reports were submitted to the Study Director and Management as follows:

Audit	Phase Inspected	Inspection Start Date	Inspection End Date	Date Reported to Study Director and Management
AUD-000313	A-579-01 Draft Protocol Audit	5/11/2023	5/12/2023	5/12/2023
AUD-000322	A-579-01 Critical Phase Inspection – Room Inspection, Group Assessment, and Animal Identification	5/16/2023	5/17/2023	5/17/2023
AUD-000330	A-579-01 Critical Phase Inspection – Surgery	5/17/2023	5/18/2023	5/22/2023
AUD-000353	A-579-01 Critical Phase Inspection – Electrocardiogram Evaluations, Nerve Conducting Velocity Testing, and Cerebrospinal Fluid Collection	6/9/2023	6/9/2023	6/9/2023
AUD-000358	A-579-01 Protocol Amendment 1	6/13/2023	6/13/2023	6/13/2023
AUD-000362	A-579-01 Critical Phase Inspection – Ophthalmic Evaluations	6/15/2023	6/15/2023	6/15/2023
AUD-000363	A-579-01 Protocol Amendment 2	6/16/2023	6/16/2023	6/16/2023
AUD-000366	A-579-01 Critical Phase Inspection- Formulation Preparation and Dose Administration	6/20/2023	6/20/2023	6/20/2023
AUD-000368	A-579-01 – Draft Amended Protocol 3 Audit	6/20/2023	6/20/2023	6/20/2023
AUD-000370	A-579-01 Critical Phase Inspection – Urine Collection, Body Weights, Detailed Clinical Observation, Clinical Pathology Analysis, and Form ulation Preparation	6/21/2023	6/21/2023	6/21/2023
AUD-000371	A-579-01 Critical Phase Inspection – PBMC Processing and PBMC Cryopreservation, and Storage	6/16/2023	6/21/2023	6/21/2023
AUD-000380	A-579-01 Critical Phase Inspection- Blood Collection	6/27/2023	6/27/2023	6/27/2023
AUD-000403	A-579-01 Critical Phase Inspection – Food Consumption and Cage Side Clinical Observations	7/19/2023	7/19/2023	7/19/2023
AUD-000417	A-579-01 Draft Amended Protocol 4	7/31/2023	7/31/2023	7/31/2023

Audit	Phase Inspected	Inspection Start Date	Inspection End Date	Date Reported to Study Director and Management
AUD-000427	A-579-01 Test Site QAU Records	8/1/2023	8/1/2023	8/1/2023
AUD-000430	A-579-01 Critical Phase Inspection – PBMC Isolation	8/2/2023	8/3/2023	8/3/2023
AUD-000431	A-579-01 Critical Phase Inspection – Functional Neurological Observation	8/1/2023	8/3/2023	8/3/2023
AUD-000432	A-579-01 Critical Phase Inspection – Necropsy, Tissue Collection, Bank Tissue, and Organ Weights	8/3/2023	8/3/2023	8/3/2023
AUD-000472	A-579-01 Draft Amended Protocol 5	9/7/2023	9/7/2023	9/7/2023
AUD-000477	A-579-01 Test Site QAU Records	9/8/2023	9/8/2023	9/8/2023
AUD-000489	A-579-01 – Critical Phase Inspection – Tissue Processing	9/19/2023	9/19/2023	9/19/2023
AUD-000495	A-579-01 Ophthalmic Data	9/21/2023	9/22/2023	9/22/2023
AUD-000498	A-579-01 Electrocardiogram Evaluations Data	9/22/2023	9/25/2023	9/25/2023
AUD-000500	A-579-01 Ophthalmic Contributing Scientist Report and Tables	9/25/2023	9/25/2023	9/25/2023
AUD-000501	A-579-01 Safety Pharmacology Contributing Scientist Report Electrocardiograph Evaluation	9/25/2023	9/25/2023	9/25/2023
AUD-000509	A-579-01 PBMC Processing Data Audit	9/21/2023	9/26/2023	9/26/2023
AUD-000523	A-579-01 Clinical Pathology Data	9/26/2023	9/29/2023	9/29/2023
AUD-000524	A-579-01 Non-Invasive Functional Neurological Observation Data, Observation Tables, and Draft Contributing Scientist Report	10/2/2023	10/2/2023	10/2/2023
AUD-000525	A-579-01 Nerve Conduction Velocity Data, Velocity Table, and Draft Contributing Scientist Report	10/2/2023	10/2/2023	10/2/2023
AUD-000531	A-579-01 Formulation Data	10/3/2023	10/5/2023	10/5/2023
AUD-000534	A-579-01 Critical Phase Inspection – Tissue Homogenization and DNA Extraction	10/6/2023	10/6/2023	10/6/2023
AUD-000538	A-579-01 In-Life Data	9/27/2023	10/10/2023	10/10/2023
AUD-000551	A-579-01 Critical Phase Inspection – dPCR (DNA)	10/16/2023	10/18/2023	10/18/2023

Audit	Phase Inspected	Inspection Start Date	Inspection End Date	Date Reported to Study Director and Management
AUD-000568	A-579-01 Anatomic Pathology Data Audit	10/23/2023	10/24/2023	10/24/2023
AUD-000592	A-579-01 Critical Phase Inspection for IFN-y Peptide ELISpot	11/1/2023	11/1/2023	11/1/2023
AUD-000594	A-579-01 Draft Clinical Pathology Contributing Scientist Report	11/1/2023	11/1/2023	11/1/2023
AUD-000598	A-579-01 Draft Anatomic Pathology Critical Scientist Report	11/2/2023	11/2/2023	11/2/2023
AUD-000631	A-579-01 Day 43 dPCR Data Audit	11/8/2023	11/17/2023	11/17/2023
AUD-000648	A-579-01 ELiSpot Data	11/22/2023	11/29/2023	11/29/2023
AUD-000659	A-579-01 Day 85 DNA dPCR Data Audit	11/16/2023	12/1/2023	12/1/2023
AUD-000689	A-579-01 Critical Phase Inspection – RNA Homogenization and Manual Extraction	12/4/2023	12/6/2023	12/6/2023
AUD-000717	A-579-01 In-Life Provantis Table	12/28/2023	12/28/2023	12/28/2023
AUD-000718	A-579-01 dPCRDAP Module 1	12/20/2023	1/2/2024	1/2/2024
AUD-000720	A-579-01 Daft Amended Protocol 6	1/3/2024	1/3/2024	1/3/2024
AUD-000723	A-579-01 DNA dPCR Repeat Batches – Data	12/20/2023	1/4/2024	1/4/2024
AUD-000735	A-579-01 Draft IFN-γ ELISpot Analysis Contributing Scientist Report	1/4/2024	1/10/2024	1/10/2024
AUD-000750	A-579-01 Draft Amended Protocol 7	1/25/2024	1/25/2024	1/25/2024
AUD-000814	A-579-01 RT-dPCR Critical Phase Inspection	2/13/2024	2/13/2024	2/13/2024
AUD-000825	A-579-01 dPCR Contributing Scientist Report	2/16/2024	2/27/2024	2/27/2024
AUD-000843	A-579-01 RT-dPCR SSMs, Input Files, and Design Files	3/1/2024	3/8/2024	3/8/2024
AUD-000851	Draft Final Report	3/14/2024	3/14/2024	3/14/2024
AUD-000860	A-579-01 RT-dPCR Repeat Batches 106-108	3/20/2024	3/20/2024	3/20/2024
AUD-000899	A-579-01 RT-dPCR repeats and Final Table	4/9/2024	4/9/2024	4/9/2024
AUD-000930	A-579-01 RT-dPCR updated results Table	4/24/2024	4/24/2024	4/24/2024
AUD-001002	A-579-01 RT-dPCR Contributing Scientist Report	5/23/2024	5/23/2024	5/23/2024
AUD-001004	A-579-01 Updated dPCR Contributing Scientist Report	5/24/2024	5/24/2024	5/24/2024

Audit	Phase Inspected	Inspection Start Date	Inspection End Date	Date Reported to Study Director and Management
AUD-001005	Updated Draft Final Report	5/24/2024	5/24/2024	5/24/2024
AUD-001011	A-579-01 RT- Final Anatomic Pathology Contributing Scientist Report	5/30/2024	5/30/2024	5/30/2024
AUD-001385	A-579-01 Analytics Sample Disposition Logbooks	10/15/2024	10/15/2024	10/15/2024
AUD-001432	Final Report	11/1/2024	11/1/2024	11/1/2024

Signed by:
Lisa Gray
Signer Name: Lisa Gray
Signing Reason: I am the author of this document
Signing Time: 11/1/2024 | 9:32:32 AM EDT
401DDFD6BCCD49DC98807004E3A3E609

11/1/2024

Quality Assurance Unit

Date

TABLE OF CONTENTS

Signatures 2

Summary 3

Compliance Statement 6

Quality Assurance Statement 8

1 Introduction 16

2 Materials and Methods 16

2.1 Test Article, Control Article, and Vehicle 16

2.1.1 Test Article 16
2.1.2 Control Article 16
2.1.3 Vehicle 17

2.2 Test and Control Article Preparation 17

2.2.1 Test Article 17
2.2.2 Control Article 17

2.3 Formulation Analysis 17

2.4 Reserve Samples 17

2.5 Disposition of Test, Control Articles, and Vehicle 17

2.6 Experimental Animals 18

2.6.1 Housing and Environmental Conditions 18
2.6.2 Feed and Water 18
2.6.3 Animal Identification and Group Assignment 19

3 Experiental Design 19

3.1 Dose Administration 19

3.2 Clinical Observations 20

3.3 Body Weights 20

3.4 Food Consumption 20

3.5 Cerebrospinal Fluid 20

3.6 Ophthalmic Examinations 21

3.7 Neurological Observations 21

3.8 Nerve Conduction Velocity Testing 21

3.9 Electrocardiogram (ECG) Evaluations 21

3.10 Clinical Pathology Collection and evaluation 22

3.10.1 Clinical Pathology Blood Specimen Collection 22

3.11 PBMC Blood Collection and ELISpot Analysis 24
3.11.1 PBMC Blood Specimen Collection 24
3.11.2 IFN-γ ELISpot Analysis 25
3.12 Necropsy 25
3.12.1 Scheduled Necropsy 25
3.12.2 Tissue Collection 25
3.12.3 Organ Weights 27
3.12.4 Flash Frozen (FF) Samples 27
3.12.5 Bank Tissue Samples 27
3.12.6 Tissue Processing 28
3.12.7 Histopathologic Evaluation 28
3.13 Biodistribution Analysis By Digital PCR 28
4 Computer Systems for Data Management 29
5 Statistical Analysis 29
5.1 Provantis-Collected Data 29
5.2 Biodistribution statistical analysis 29
5.3 Data Reported by Descriptive summaries or incidence 30
6 Storage of Study Materials and Records Retention 30
7 Results 31
7.1 Mortality 31
7.2 Clinical Observations 31
7.3 Body Weights 31
7.4 Food Consumption 31
7.5 Ophthalmic Evaluations 31
7.6 Neurobehavioral Evaluation 32
7.7 Nerve Conductance Velocity Evaluation 33
7.8 ECG Evaluation 34
7.9 Clinical Pathology Evaluation 34
7.10 ELISpot Analysis 34
7.11 Anatomic Pathology Evaluation 35
7.11.1 Terminal Body Weights and Organ Weights 35
7.11.2 Gross Pathology 35
7.11.3 Histopathology 36
7.12 Biodistribution Analysis by dPCR 39
7.13 Biodistribution Analysis by RT-dPCR 42

8 Discussion **53**
9 Acknowledgements **55**
10 References **55**

LIST OF TABLES

Table 1. Experimental Design 19
Table 2. Hematology Parameters 23
Table 3. Coagulation Parameters 23
Table 4. Serum Chemistry Parameters 23
Table 5. Urinalysis Parameters 24
Table 6. Tissue Collection 26
Table 7. Computer Systems 29
Table 8. Disposition of Study Materials 30
Table 9. Summary of Neurobehavioral Assessment Findings 33
Table 10. Summary of Test Article-Related Microscopic Findings in the 6-Week Cohort (Day 43) Male and Female Cynomolgus Macaques 37
Table 11. Summary of Test Article-Related Microscopic Findings in the 12-Week Cohort (Day 85) Male and Female Cynomolgus Macaques 38
Table 12. Principal Contributors 55

LIST OF FIGURES

Figure 1. Time course of AAV-9 specific T cell responses by IFN-γ ELISpot 35
Figure 2. Trend of Log Transformed AAV2-ITR Mean Target Copy Number per µg DNA (Not Normalized to the housekeeping Gene B2M) Across Tissues for All Groups for Day 43 40
Figure 3. Trend of Log Transformed AAV2-ITR Mean Target Copy Number per µg DNA (Not Normalized to Housekeeping Gene B2M) Across Tissues for All Groups for Day 85 41
Figure 4. Assessment of Trends in Mean Log-Transformed miR871 Target Copy Number Per µg cDNA by Group Across Tissues for Day 43 43
Figure 5. Assessment of Trends in Mean Log-Transformed miR871 Target Copy Number Per µg cDNA by Group Across Tissues for Day 85 44
Figure 6. Assessment of Trends in Mean Log-Transformed PMP22 Target Copy Number Per µg cDNA by Group Across Tissues for Day 43 46
Figure 7. Assessment of Trends in Mean Log-Transformed PMP22 Target Copy Number Per µg cDNA by Group Across Tissues for Day 85 47
Figure 8. Examining Differences between miR871 and PMP22 for the Nerve Tissues for Log-Transformed Data (Normalized to B2M) across Nerve Tissues for Day 43 and Low Dose of the Vector 49
Figure 9. Examining Differences between miR871 and PMP22 for the Nerve Tissues for Log-Transformed Data (Normalized to B2M) across Nerve Tissues for Day 43 and High Dose of the Vector 50

Figure 10. Examining Differences between miR871 and PMP22 for the Nerve Tissues for Log-Transformed Data (Normalized to B2M) across Nerve Tissues for Day 85 and Low Dose of the Vector 51
Figure 11. Examining differences between miR871 and PMP22 for the Nerve Tissues for Log-Transformed Data (Normalized to B2M) across Nerve Tissues for Day 85 and High Dose of the Vector........ 52

LIST OF APPENDICES

Appendix A Protocol, Amendments, and Summary of Deviations........ A-1
Appendix B Test and Control Article Information B-1
Appendix C Intrathecal Catheter Surgery Method........ C-1
Appendix D In-Life Animal Data........ D-1
Appendix E Ophthalmic Examination Contributing Scientist Report E-1
Appendix F Neurobehavioral Contributing Scientist Report F-1
Appendix G Nerve Conduction Evaluation Contributing Scientist Report........ G-1
Appendix H Electrocardiograph Evaluation Contributing Scientist Report H-1
Appendix I Clinical Pathology Contributing Scientist Report I-1
Appendix J IFN-γ ELISpot Analysis Contributing Scientist Report J-1
Appendix K Anatomic Pathology Contributing Scientist Report K-1
Appendix L dPCR Contributing Scientist Report........L-1
Appendix M RT-dPCR Contributing Scientist Report M-1

1 INTRODUCTION

The purpose of this study was to evaluate the potential biodistribution and safety of scAAV9.U6miR871 (ARM-101) when administered once via intrathecal infusion to Cynomolgus monkeys for up to 12 weeks.

This study was conducted at AmplifyBio under the direction of Sapna Varia, PhD. The study was initiated on May 15, 2023, with the signing of the protocol. The in-life portion of the study began on June 20, 2023, with initiation of dose administration (Group 1). The final necropsy was performed on September 14, 2023.

Endpoints evaluated were clinical observations, body weights, food consumption, non-invasive neurological observations, nerve conduction velocity, ophthalmic examinations, electrocardiogram evaluations, troponin I analysis, clinical pathology (hematology, coagulation, serum chemistry, and urinalysis), ELISpot evaluation, biodistribution analysis (dPCR and RT-dPCR), and anatomic pathology, including organ weights, gross observations at necropsy, and microscopic evaluation of selected tissues.

Armatus Bio, Inc. was the Sponsor of the study. Brian Price, PhD was designated as the Sponsor Representative.

The study protocol, protocol amendments, and a summary of deviations from GLP, the protocol, and selected SOP and study specific methods are presented in Appendix A.

2 MATERIALS AND METHODS

2.1 TEST ARTICLE, CONTROL ARTICLE, AND VEHICLE

2.1.1 Test Article

The test article was identified as follows:

Name:	ARM-101 (scAAV9.U6miR871 vector)
Batch/Lot Number:	TT970-1
Storage Conditions:	-85 to -60°C
Expiry/Retest Date:	Not Provided

A Certificate of Analysis (CoA) for the test article is included in Appendix B. Documentation of synthesis was maintained by the manufacturer.

2.1.2 Control Article

The control article was identified as follows:

Control Article Name:	Formulation Buffer (TMN200P)
Lot Number:	TMNP0291-B
Storage Conditions:	-85 to -60°C
Supplier:	Andelyn Biosciences

A Certificate of Analysis (CoA) for the control article is included in Appendix B. Documentation of synthesis was maintained by the manufacturer.

2.1.3 Vehicle

The vehicle was identified as follows:

Vehicle Name:	0.9% Saline
Lot Number:	FM4935
Storage Conditions:	13 to 25°C
Expiry Date:	November 1, 2023
Supplier:	Hospira

The vehicle is a marketed product and was characterized by its labeling according to 21 CFR 58.105. Documentation of synthesis was maintained by the manufacturer.

2.2 Test and Control Article Preparation

2.2.1 Test Article

Formulations of test article were diluted at concentrations of 6×10^{13} and 1.2×10^{14} vg with the vehicle. Bulk test article used for formulations were thawed at 2 to 8°C. When not in use, the formulations were stored on wet ice until dosing was complete. Residual formulations were stored in a freezer set to maintain -85 to -60°C. The details of formulation preparation are included in Appendix B.

2.2.2 Control Article

The control article (formulation buffer) was received ready for use on study. The control article dispensed for dose administration was thawed at 2 to 8°C and stored on wet ice until dosing was complete. Residual dispensed control article was stored in a freezer set to maintain -85 to -60°C. Remaining thawed control article not dispensed for dose administration was stored at 2 to 8°C.

2.3 Formulation Analysis

Formulation analysis was not performed.

2.4 Reserve Samples

An approximate 3 mL sample from the single lot of bulk test article and control article used during the study were retained as a reserve sample and stored in a freezer set to maintain -85 to -60°C.

2.5 Disposition of Test, Control Articles, and Vehicle

Unused bulk test and remaining thawed bulk test article were shipped (frozen on dry ice [unused bulk] or on wet ice [remaining thawed bulk]) to the Sponsor-designee. Residual

formulations were discarded. Empty vials of the test article following formulations were discarded.

Unused bulk control article was shipped frozen (on dry ice) to the Sponsor-designee. Remaining thawed control article was shipped (on wet ice) to the Sponsor-designee. Residual dispensed control article was discarded.

Remaining vehicle following test article dilution was discarded.

2.6 Experimental Animals

A total of 20 Cynomolgus monkeys (10 males and 10 females) were received from Envigo, (Alice, Texas), quarantined, and acclimated under the testing facility holding colony. The animals were transferred from the testing facility holding pool and assigned to study on May 16, 2023.

All animals used for study were conditioned to restraint in chairs under the testing facility holding colony. To facilitate intrathecal (IT) infusions and blood collections, animals were progressively acclimated to accept up to 6 hours of chair restraint. After transfer to study and prior to Day 1, all animals were given an additional 6-hour chair acclimation session.

Prior to Day 1, all study animals were surgically implanted with a lumbar intrathecal catheter See Appendix C for details regarding the surgical procedure.

On Day 1, animals were between approximately 2 to 5 years of age with body weights ranging from 2.25 to 3.06 kg for males and 2.47 to 5.20 kg for females (see DEV-002293 in Appendix A for exceptions).

2.6.1 Housing and Environmental Conditions

General procedures for animal care and housing met current AAALAC International recommendations, current requirements stated in the "Guide for Care and Use of Laboratory Animals" (National Research Council, Current Edition), and conformed to Testing Facility SOP. Prior to study initiation, the study design was reviewed and approved by the Institutional Animal Care and Use Committee (IACUC).

Animals were socially housed during the duration of the study. Following the Day 43 termination for the 6-week cohort animals, Animals 102 and 152 were individually housed due to only one control animal per sex remaining on study. Individually housed animals received enhanced enrichment as determined by the veterinary staff.

The light/dark cycle was controlled by electronic timers set to maintain 12 hours of light and 12 hours of dark each day using fluorescent lighting set to start at 6:00 AM each day. Room temperature and relative humidity controls were set to 74°F and 50%, respectively. Actual room temperature and relative humidity ranges during the study were 72.01 to 76.82°F and 38.25 to 81.53%, respectively.

2.6.2 Feed and Water

Animals were provided PMI Certified Primate Diet (LabDiet 5048) twice daily, except on the day of study transfer when feed was only be offered once, during specified fasting periods, or

when the animal was removed from the home cage for study events (e.g., when placed in restraint chairs for dose administrations and blood collections). The diet was also supplemented with fruits, vegetables, or other supplemental enrichment.

Fresh water from the Village of West Jefferson municipal water supply was provided *ad libitum* to the animals by an automatic watering system except when removed from the home cage.

2.6.3 Animal Identification and Group Assignment

Prior to study assignment, animals were identified by the tattoo present at receipt. Animals were assigned to dose groups using a stratified randomization program (Provantis) designed to maintain similar group mean body weights by sex.

After assignment to dose groups, each animal received an animal identification number unique within the study and was identified with a cage card. Each cage card contained information including, but not limited to, study number, group assignment, and animal identification number. The tattoo was used to match the study animal number on the cage cards.

Prior to Day 1, Animal 352 was reassigned to Group 1 as Animal 152 on Day -6 and Animal 152 was reassigned to Group 3 as Animal 352 on Day -8. These animals were re-assigned due to the maximum allowable dose volume of the test article based on the animal body weights.

3 EXPERIENTAL DESIGN

Twenty (20) Cynomolgus monkey (10 male and 10 female) were assigned to one of three dose groups as presented in the table below.

Table 1. Experimental Design

Group	Target Dose[c] (Total vg)	Target Dose Volume (mL)	Animal Identification Numbers			
			6-Week Cohort[a]		12-Week Cohort[b]	
			Male	Female	Male	Female
1 – Control	0	3	101	151	102	152
2 – Low	6×10^{13}	3	201-202	251-252	203-204	253-254
3 – High	1.2×10^{14}	3	301-302	351-352	303-304	353-354

vg = vector genome
a. Necropsy Day 43.
b. Necropsy Day 85.
c. See QE-001542 in Appendix A for exceptions.

3.1 Dose Administration

On Day 1, the test (~3 mL of target dose) or control article (~3 mL formulation buffer) was administered to each animal via intrathecal slow infusion over approximately 6 hours via a surgically-implanted lumbar catheter (see QE-001542 in Appendix A for exceptions). For dose administration, animals were mildly sedated, as deemed appropriate by veterinary staff, with acepromazine (10 mg/mL).

3.2 Clinical Observations

Observations for moribundity and mortality were performed twice daily during the study period, except on the day of study transfer and on the day of scheduled necropsy when animals were only observed once.

Prior to Day 1, cage-side observations were performed on all animals according to facility SOPs. Beginning on Day 1, cage-side clinical observations were performed on all animals assigned to study at least once daily for the duration of the study. On the day of dose administration, cage-side clinical observations were recorded between 30 minutes and 2.5 hours post-dose administration. An observation was recorded on the day of scheduled necropsy.

Detailed clinical observations were performed on all animals assigned to study at least once during the pretest period, prior to dosing on the day of dose administration, and at least once weekly thereafter.

When detailed observations and cage-side observations coincided, only the detailed observation was performed.

Additional observations were recorded as clinical signs warranted.

3.3 Body Weights

Individual body weights were recorded for all animals at least once prior to Day 1 for group assignment and for all animals assigned to study prior to dosing on Day 1, and weekly thereafter (see DEV-002290 in Appendix A for exceptions). Terminal body weights (fasted) for calculation of organ-to-body weight ratios were recorded on the day of scheduled necropsy.

3.4 Food Consumption

Food consumption was visually assessed once daily beginning on Day -7 according to the following scale (see QE-001496 in Appendix A for exceptions):

0 = no observable food consumed
1 = up to and including ¼ consumed
2 = greater than ¼ up to and including ½ consumed
3 = greater than ½ up to and including ¾ consumed
4 = greater than ¾ up to and including the entire amount consumed

Food consumption was not assessed during times of scheduled fasting.

3.5 Cerebrospinal Fluid

Cerebrospinal fluid (CSF) was collected from all surviving animals via passive collection once prior to Day 1 and once during Week 2, Week 6, and Week 12 (see DEV-002294 in Appendix A).

Animals were sedated with ketamine and the skin overlying the cisterna magna or lumbar cisterns was clipped (see DEV-002739 in Appendix A) and aseptically prepared. CSF was

collected from each animal (up to 1.0 mL), dispensed into one properly labeled vial, and stored in a freezer set to maintain -85 to -60°C.

All CSF specimens were shipped to the Sponsor-designated test site (on dry ice) for potential analysis prior to report finalization. Any analyses performed or results obtained with these specimens were not reported under this study.

3.6 Ophthalmic Examinations

Ophthalmic examinations were performed for all animals assigned to the study once prior to Day 1, once during Week 6, and once for all surviving animals during Week 12 by a board-certified veterinary ophthalmologist. Animals were sedated with ketamine (10 mg/kg, intramuscular) for examinations.

The pupils of each assigned animal were dilated by instillation of drops of tropicamide ophthalmic solution prior to examination. The anterior segment of the eye, posterior segment of the eye, and adnexal structures were examined using a Kowa SL-17 portable slit lamp and a Heine 180 indirect ophthalmoscope with Volk 30 Diopter condensing lens. Each eye examined was recorded as either "no abnormal findings (NADO)" or "cataract (Cat)" (see QE-002014 in Appendix A).

3.7 Neurological Observations

Non-invasive functional neurological observations (cage-side) were performed on all animals once prior to Day 1 and on all surviving animals assigned to study once per study week thereafter. Details of the neurological observation assessments can be found in Appendix F. Observations were not evaluated on the day of ophthalmic examinations. Observations were conducted by staff trained in performing neurological observations in monkeys.

3.8 Nerve Conduction Velocity Testing

Nerve conduction velocity testing was performed on all animals assigned to study, while sedated with ketamine for ECG collections, once during the pretest period, once during Week 6, and for all surviving animals once during Week 12 (see DEV-002401 in Appendix A). Once properly sedated, electrodes were briefly placed over the endplate zone of abductor hallucis muscle of each hindlimb. The tibial nerve conduction was evaluated with recorded Compound Muscle Action Potential (CMAP) traces. The CMAP recordings were used to measure latency, amplitude, and duration, and to calculate nerve conduction velocity. CMAPs were recorded bilaterally and reported as separate values. Details of the nerve conduction assessment procedure can be found in Appendix G.

3.9 Electrocardiogram (ECG) Evaluations

ECG (non-telemetered) waveform tracing assessments were performed on all animals assigned to study once during the pretest period, once during Week 6, and for all surviving animals once during Week 12. Animals were sedated with ketamine (10 to 16 mg/kg, intramuscular) for all ECG evaluations.

Once properly sedated, ECG waveform tracings and blood pressure measurements were collected for at least one minute using surface leads emulating a standard Lead II configuration (see QE-002111 in Appendix A). A board-certified veterinarian qualitatively assessed the ECG waveform strips for evidence of cardiac dysfunction by examining rate, rhythm, and morphology alterations.

3.10 CLINICAL PATHOLOGY COLLECTION AND EVALUATION

3.10.1 Clinical Pathology Blood Specimen Collection

Blood specimens for clinical pathology evaluation, including Troponin I, were collected from all study animals once prior to Day 1, and on Days 2, 8, 22, and 43 and from all surviving animals (12-week cohort animals only) on Day 50 and Day 85. Pretest blood collections for serum chemistry, hematology, and coagulation were performed while the animals were under the testing facility holding colony (TOXBC HOLD). Blood collection data were transferred to study and reported. Animals were fasted overnight prior to scheduled blood collection. Ketamine (10-15 mg/kg, intramuscular) was used for blood collections, as needed, and all specimens were collected from the femoral vessel.

Blood specimens for hematology (2.0 mL) were collected in a tube containing K_3EDTA. Blood specimens for serum chemistry (2.5 mL) were collected in a serum separator tube (SST) and allowed to clot at room temperature for at least 30 minutes before processing to serum (see DEV-002206 and DEV-002563 in Appendix A for exceptions). Separation of the blood into serum was performed at a relative centrifugal force of 2,000 g for 10 minutes (see DEV-002206 in Appendix A for exceptions). Blood specimens for coagulation (1.8 mL) were collected in a sodium citrate tube and processed immediately upon clinical pathology receipt (see DEV-002565 in Appendix A for exceptions).

Blood specimens for Troponin I (1.0 mL) were collected in a serum separator tube (SST) (see DEV-002735 in Appendix A for Week 1 exceptions). The collected blood was allowed to clot at room temperature for at least 30 minutes before processing to serum (see DEV-002752, DEV-002206, and DEV-002563 in Appendix A for exceptions). Separation of the blood into serum was performed at room temperature using 10 minutes of centrifugation at a relative centrifugal force of 2,000 g (see DEV-002206 in Appendix A for exceptions). Troponin serum samples were maintained on dry ice and transferred to a freezer set to maintain -85 to -60°C.

The blood specimens collected for clinical pathology evaluation were submitted to the testing facility's clinical pathology laboratory for processing and analysis.

The Troponin I serum specimens were shipped frozen (on dry ice) to the designated test site for Troponin I analysis. The test site provided a principal investigator report (method report) which also included evidence of a QA critical phase inspection and a GLP statement. The Troponin I analysis principal investigator method report was included in the clinical pathology contributing scientist report and the results were evaluated by the testing facility.

3.10.1.1 Hematology

Hematology parameters evaluated included:

Table 2. Hematology Parameters

Cell morphology[a,b]	Mean corpuscular volume (MCV)
Erythrocyte count (RBC)	Mean corpuscular hemoglobin (MCH)
Hemoglobin (HGB)	Mean corpuscular hemoglobin concentration (MCHC)
Hematocrit (HCT)	Mean platelet volume (MPV)[b]
Leukocyte count, total (WBC)	Platelet count (PLT)
Leukocyte differential (absolute and relative)	Reticulocyte count (absolute and relative) (RETIC and %RETIC)[b]

a. Slides were prepared from un-clotted samples.
b. See deviation DEV-003039 in Appendix A for exceptions.

Hematology specimens (residual blood) were discarded following analysis.

3.10.1.2 Coagulation

Coagulation parameters evaluated included:

Table 3. Coagulation Parameters

Activated partial thromboplastin time (APTT)	Prothrombin time (PT)
Fibrinogen (FIB)	

Coagulation specimens (residual plasma) were discarded following analysis.

3.10.1.3 Serum Chemistry

Serum chemistry parameters evaluated included:

Table 4. Serum Chemistry Parameters

Alanine aminotransferase (ALT)	Creatine kinase (CK)
Albumin (ALB)	Creatinine (CREA)
Albumin/globulin ratio (A/G)	Gamma glutamyl transferase (GGT)
Alkaline phosphatase (ALP)	Globulin (GLOB)
Aspartate aminotransferase (AST)	Glucose (GLU)
Bilirubin, direct (DBILI)	Lactate dehydrogenase (LDH)
Bilirubin, total (TBILI)	Phosphorus (PHOS)
Blood urea nitrogen (BUN)	Potassium (K)
Calcium (CA)	Sodium (NA)
Cholesterol (CHOL)	Total protein (TP)
Chloride (CL)	Triglycerides (TRIG)

Residual serum following analysis were discarded prior to clinical pathology report finalization.

3.10.1.4 Urinalysis

Urine was collected overnight from all animals prior to group assignment and during Weeks 1, 2, 4 and 7 from all animals assigned to the 6-week cohort and Weeks 1, 2, 4, 7, 8, and 12 from all animals assigned to the 12-week cohort (see DEV-002185 in Appendix A for exceptions). The total urine output collected overnight (via cage pan) was provided to the clinical pathology laboratory. The actual time period of collection was documented. The following parameters were assessed:

Table 5. Urinalysis Parameters

Appearance (clarity and color)	pH[a]
Bilirubin[a]	Protein[a]
Glucose[a]	Specific gravity[b]
Ketones[a]	Total volume
Leukocytes[a]	Urobilinogen[a]
Nitrite[a]	Microscopic examination of sediment[c]
Occult blood[a]	

a. Qualitative results were obtained using reagent test strips (urine dip parameters).
b. Results were obtained using the SPER Scientific Digital Refractometer.
c. Sediment were evaluated microscopically for white blood cells, red blood cells, casts, epithelial cells, mucus, sperm, bacteria, yeast, amorphous sediment, and crystals.

Residual urine was discarded following analysis (see DEV-002205 in Appendix A).

3.10.1.5 Troponin I

Troponin I (cTnI) was analyzed at the test site (Antech Diagnostics GLP) and a principal investigator method's report was provided. The Troponin I evaluation was performed by the testing facility. The Troponin I analysis principal investigator method report is included in Appendix I.

Residual Troponin I samples were discarded following analysis by the test site.

3.11 PBMC Blood Collection and ELISpot Analysis

3.11.1 PBMC Blood Specimen Collection

Blood specimens (one 6.0 mL aliquot) for PBMC isolation and ELISpot analysis were collected in a tube containing Na-Heparin once prior to Day 1 (all animals), on Day 43 (all animals), and on Day 85 (animals assigned to the 12-week cohort) (see QE-001541 and DEV-002199 in Appendix A for exceptions). Animals were fasted overnight prior to scheduled blood collection. Ketamine (10-15 mg/kg, intramuscular) was used for blood collections, as needed, and all specimens were collected from the femoral vessel.

Blood specimens were submitted to the testing facility's analytics department for PBMC processing and ELISpot analysis.

3.11.2 IFN-γ ELISpot Analysis

Blood specimens were processed for peripheral blood mononuclear cells (PBMCs) within six hours of collection using 50 ml SepMate™ tubes (see deviation DEV-002201 in Appendix A for exceptions). Freshly isolated PBMCs were resuspended in cold Cryostor CS10 into at least two sterile conical tubes at a final concentration of 1×10^7 cells/mL (5×10^6 viable cells/vial) (see QE-001798 in Appendix A for exceptions). Residual samples were either combined with a previous vial (≤ 0.250 mL) or aliquoted into a separate vial (> 0.250 mL). Vials of isolated PBMCs were cryopreserved at < -145°C in a liquid nitrogen (LN2) vapor-phase storage tank. PBMC isolation, cryopreservation, and storage were executed according to a study-specific method (see Appendix J). Cryopreserved PBMCs were then tested for the presence of AAV9-specific T cell responses by a qualified IFN-γ ELISpot assay according to a study-specific method as described in Appendix J. ELISpot plates following analysis and retained PBMC aliquots were discarded following study director and Sponsor approval.

3.12 NECROPSY

3.12.1 Scheduled Necropsy

All animals at scheduled termination (Day 43 for 6-week cohort or Day 85 for 12-week cohort) were euthanized by an administration of a barbiturate overdose followed by exsanguination, and a gross necropsy with select tissue harvest. Carcass and remaining tissues were discarded after collection.

All scheduled necropsies were conducted with a board-certified veterinary pathologist available for consultation.

3.12.2 Tissue Collection

The following tissues, when present, were collected from all animals undergoing necropsy (see QE-001785 in Appendix A for Day 43 exception). Tissues were placed in 10% neutral buffered formalin (NBF), unless otherwise indicated.

Table 6. Tissue Collection

Tissues to be Collected	OW	HE	FF	Bank[b]	Tissues to be Collected	OW	HE	FF	Bank[b]
Animal identification[a]					Nerve, sciatic (proximal)[g]		X	X	X[l]
Adrenal glands (paired)	X	X			Nerve, Optic			X	X[k]
Aorta		X			Nerve, Ulnar (distal)[g]			X	X[l]
Bone marrow (sternum)		X			Nerve, Ulnar (proximal)[g]			X	X[l]
Bone marrow smear (rib)[c]					Ovaries (paired)	X	X	X	
Brain[d]	X	X	X	X[k]	Pancreas		X		
Cecum		X			Parathyroid gland[h]		X		
Cervix		X			Pituitary gland	X	X		
Colon		X			Prostate		X		
DRG, cervical		X	X	X[k]	Rectum		X		
DRG, thoracic		X	X	X[k]	Salivary glands (mandibular)		X		
DRG, lumbar		X	X	X[k]	Seminal Vesicle		X		
Duodenum		X			Skeletal Muscle (Biceps brachii, L)			X	X[k]
Epididymides (paired)[e]	X	X			Skeletal Muscle (Biceps brachii, R)			X	X[k]
Esophagus		X			Skeletal Muscle (Tibialis anterior, L)			X	X[k]
Eyes (paired)[f]		X			Skeletal Muscle (Tibialis anterior, R)			X	X[k]
Femur		X			Skeletal muscle (thigh)		X		
Gallbladder		X			Skin		X		
Gross lesions (if applicable)		X	X		Spinal cord (cervical)		X	X	X[k]
Heart	X	X	X	X[k]	Spinal cord (thoracic)		X	X	X[k]
Ileum		X			Spinal cord (lumbar)		X	X	X[k,m]
Jejunum		X			Spinal cord (sacral)			X	X[k]
Kidneys (paired)	X	X	X	X[k]	Spinal roots (lumbar)			X	X[k,m]
Liver	X	X	X	X[k]	Spleen	X	X	X	X[k]
Lungs	X	X	X		Stomach (cardiac, fundus, pyloris)		X		
Lymph node, mandibular		X			Testes (paired)[e]	X	X	X	
Lymph node, mesenteric		X			Thymus	X	X		
Mammary gland		X			Thyroid glands (paired)[i]	X	X		
Nerve, Femoral (distal)[g]			X	X[l]	Trachea		X		
Nerve, Femoral (proximal)[g]			X	X[l]	Urinary bladder		X		
Nerve, Median (distal)[g]			X	X[l]	Uterus[j]	X	X		
Nerve, Median (proximal)[g]			X	X[l]	Vagina		X		
Nerve, sciatic (distal)[g]		X	X	X[l]					

OW = Organ Weights; HE = Histopathologic Evaluation; FF = Flash Frozen; DRG = Dorsal Root Ganglion.
Priority: HE > FF; DNA > RNA >Additional Sample (AS) > Bank

a. Collected but not processed.
b. Only if available.
c. Bone marrow cytology smears were prepared for all animals at scheduled necropsy.
d. Corpus callosum collected for FF and Bank.
e. Preserved in Modified Davidson's fixative, then transferred to 10% NBF.
f. Preserved in Davidson's fixative, then transferred to 10% NBF.
g. From two different designations of each nerve, left and right.
h. Examined only if in routine section.
i. Weighed with parathyroids embedded.
j. Weighed with cervix
k. Flash frozen using liquid nitrogen.
l. One bank section from each designation was collected. One was saved in 4% glutaraldehyde in 0.1M Phosphate Buffer pH 7.4 and the other flash frozen.
m. Collected attached to spinal cord to include a full transverse section of the spinal cord with spinal roots attached.

3.12.3 Organ Weights

The organs indicated in Table 6, when present, were weighed at all scheduled necropsies. Paired organs were weighed together.

3.12.4 Flash Frozen (FF) Samples

The tissues indicated in Table 6 designated for Flash Frozen were collected under aseptic conditions for all animals undergoing necropsy. Following collection, tissue samples designated for FF were sectioned into three 3 mm^3 aliquots (DNA, RNA, and additional sample [AS]), measured visually without the use of a measuring aid, placed into vials, and flash frozen using liquid nitrogen. Samples were transferred to a cold storage unit set to maintain -85 to -60°C (see DEV-002774 in Appendix A for exceptions). Flash frozen tissue samples were submitted to the testing facility's analytics department for processing and biodistribution analysis.

3.12.5 Bank Tissue Samples

The tissues indicated in Table 6 designated for Bank were collected for all animals undergoing necropsy if available.

3.12.5.1 Peripheral Nerves Bank Tissue Samples

Following collection, tissue samples designated for Bank from peripheral nerves were collected from distal and proximal ends of each nerve.

One of the two designations of distal and proximal ends of the nerve were placed in 4% glutaraldehyde in 0.1M Phosphate Buffer pH 7.4 in individually labeled cassettes and stored in a cold storage unit set to maintain 2 to 8°C. Samples were shipped refrigerated (on wet ice) to the Sponsor-designee for potential analysis prior to report finalization. Any analyses performed or results obtained with these tissues following shipment were not reported under this study.

One of the two designations of distal and proximal ends of the nerve were sectioned into one 3 mm^3 sample measured visually without the use of a measuring aid and placed into vials to be flash frozen using liquid nitrogen. Samples were maintained on dry ice until transferred to a cold storage unit set to maintain -85 to -60°C (See DEV-002774 in Appendix A for exceptions). Samples were shipped frozen (on dry ice) to the Sponsor-designee for potential analysis prior to report finalization. Any analyses performed or results obtained with these tissues following shipment were not reported under this study.

3.12.5.2 Non-Peripheral Nerve Bank Tissue Samples

Remaining tissue samples designated for Bank were sectioned into one 3 mm^3 sample measured visually without the use of a measuring aid and placed into vials to be flash frozen using liquid nitrogen. Samples were maintained on dry ice until transferred to a cold storage unit set to maintain -85 to -60°C until shipment to the Sponsor-designee (See DEV-002774 in Appendix A for exceptions).

3.12.6 Tissue Processing

All fixed tissues from all animals, if present, were processed to slides and stained with hematoxylin and eosin for histopathologic examination.

3.12.7 Histopathologic Evaluation

The above tissues and slides were examined microscopically by a board-certified veterinary pathologist. An internal peer review was not performed.

3.13 BIODISTRIBUTION ANALYSIS BY DIGITAL PCR

Flash frozen (FF) tissue aliquots 1 and 2 were collected as indicated in Table 6, from both the Day 43 and Day 85 necropsy and processed to DNA and RNA according to a study specific method (see Appendix L and Appendix M, respectively). The additional flash frozen aliquot 3 (AS) sample was used for either DNA or RNA, as needed, with Sponsor approval at the direction of the Study Director and any remaining additional aliquot 3 samples were shipped to the Sponsor. Any analyses performed on the remaining AS tissues shipped to the Sponsor will not interfere with the conduct of this study and will not jeopardize the GLP compliance of this study. The analysis performed on these samples are not considered to be part of this study and the results will not be presented in this study report. The final disposition of the samples is under the responsibility of the Sponsor.

DNA was analyzed for AAV2-ITR vector genome and house-keeping gene Beta-2-microglobulin (B2M) quantification by dPCR according to a study specific method (see Appendix L for details). RNA was analyzed for miR871, PMP22, and B2M gene expression by RT-dPCR according to a study specific method (see Appendix M for details). Control (Group 1) animals are labeled in Appendix L and Appendix M as either vehicle or vehicle control group, throughout the raw data analysis and graphs for CSR reports; however, Control (Group 1) results are represented as control and not “vehicle” throughout this report.

Prepared dPCR and RT-dPCR nanoplates were discarded following analysis. Residual/retained biodistribution samples (DNA/RNA/tissue homogenates/cDNA) were shipped to the Sponsor-designee for potential analysis. Any analyses performed or results obtained will not interfere with the conduct of this study and will not jeopardize the GLP compliance of this study. The analysis performed on these samples are not considered to be part of this study and the results

will not be presented in this study report. The final disposition of the samples is under the responsibility of the Sponsor.

4 COMPUTER SYSTEMS FOR DATA MANAGEMENT

The following computer systems were used for toxicology data management:

Table 7. Computer Systems

Computer System	Manufacturer	Data Type
BMRAM	Blue Mountain Quality Resources	Asset Management
Elemental Machines	Elemental Machines	Environmental Storage and Animal Facility Environment
EMCS	Siemens	Animal Facility Environment
Nicolet Viking EDX	Natus	Nerve Conduction Velocity Testing
Provantis	Instem	Animal Toxicology, Clinical Pathology, and Post-Mortem Data Management
EMKA IOX	Emka Technologies	ECG Data Collection
EMKA ecgAUTO	Emka Technologies	ECG Waveform Analysis
ImmunoSpot	CTL	ELISpot Data Analysis
QIAcube, QIAxpert	QIAGEN	DNA/RNA Isolation
QIAcuity	QIAGEN	dPCR Analysis

5 STATISTICAL ANALYSIS

5.1 PROVANTIS-COLLECTED DATA

The quantitative in-life data collected at AmplifyBio using the Provantis system were not appropriate for statistical analysis due to the small sample sizes in the control group.

5.2 BIODISTRIBUTION STATISTICAL ANALYSIS

To determine the quantification of the target genome and the target housekeeping gene (Mean and % CV) at the sacrificial time-points (Day 43 or Day 85), an internally-validated dPCR data analysis pipeline system was used.

A suitable transformation data method was determined by examining the distribution of the data using histograms, normal P-P plots, and Shapiro-Wilk tests across gender, group, and time point. Based on the results of these descriptive statistics, log transformation or square root transformation was applied. See DEV-002537 in Appendix A for GLP exception.

Biodistribution statistical analysis was performed by the test site (Precision Consulting, LLC) and results were provided in the form of a Principal Investigator report for dPCR and RT-dPCR analysis and are included in Appendix L and Appendix M, respectively.

5.3 Data Reported by Descriptive summaries or incidence

Survival, clinical observations, and gross lesions were reported as incidences.

Histopathological findings and severity were reported as incidence and mean severity.

6 STORAGE OF STUDY MATERIALS AND RECORDS RETENTION

All records generated at the testing facility required to reconstruct the study and the final report are archived at the testing facility and described in the table below.

Table 8. Disposition of Study Materials

Study Material	Location or Disposition
Raw data (paper data and electronic data)	AmplifyBio Archives
Final report	AmplifyBio Archives
Clinical pathology slides	AmplifyBio Archives
Wet tissues	AmplifyBio Archives
Embedded tissues (tissue blocks)	AmplifyBio Archives
Histopathology slides	AmplifyBio Archives
Raw data from Test Site	Archived at the Test Site
Principal Investigator Method Report from Test Site	AmplifyBio Archives

7 RESULTS

7.1 Mortality

All animals survived until scheduled removal from study.

7.2 Clinical Observations

Summary Data: Table D-1
Individual Data: Table D-3

There were no clinical observations attributed to the test article following dose administration.

Animals had sporadic observations including skin abrasions, discolored skin, scabbing, diarrhea, shivering, soft feces, skin alopecia, red vaginal discharge, nose discharge, limb weakness, and low food consumption. Overall, these clinical observations were observed in all groups, sporadic in incidence, and/or are common in this species and thus not considered adverse or related to the administration of the test article.

7.3 Body Weights

Summary Data: Table D-2
Individual Data: Table D-4

There were no test article-related changes to body weight observed during the study.

7.4 Food Consumption

Individual Data: Table D-5

There were no test article-related changes to food consumption observed during the study.

7.5 Ophthalmic Evaluations

Contributing Report: Appendix E

Ocular examinations performed during this study revealed no clinical evidence of a test article-related ocular effect in any exposure group.

Cataracts were observed in one animal (Animal 151) during the pre-test examination (Day -5) and on the week of scheduled necropsy (Day 41). This observation was found in a control animal and is a common finding seen in Cynomolgus monkeys of this age, hence considered non-test article related.

7.6 NEUROBEHAVIORAL EVALUATION

Contributing Report: Appendix F

The parameters that were evaluated had findings mainly related to general activity, repetitive movements, and stargazing, each of which was seen in males and females administered control (Formulation Buffer) or the test article scAAV9.U6miR871 (ARM-101). Other findings such as piloerection and self-mutilation were observed in one Group 2 female (Table 9).

Repetitive motions or movements are considered a stereotypic behavior observed in a subpopulation of male and female cynomolgus macaques in a research setting. There are a number of factors that may influence an unpredictable stereotypic behavior, such as number of sedations and blood collections. Events of routine husbandry activities such as feeding, and sedation may also be stressful and have an impact on the levels of stereotypic behaviors.

Stargazing is a complex behavior that involves an interaction of both cognitive and social behaviors and was observed only in one male each of both low and high dose during the study period. Similarly, changes in mentation/behavior and general activity have a number of factors that can influence the variability of these parameters that include the contents of the cage, cage location, cage size, environmental enrichment, and social interactions that routinely vary.

Results from the study indicates the changes in the scores were ambiguous and merely a change of a score by a factor of 1 in animals both in the control group as well as in those receiving scAAV9.U6miR871 (ARM-101).

Table 9. Summary of Neurobehavioral Assessment Findings

Animal Number	Parameter – Observation	Score	Timepoint Following Post-Dose Administration (Pretest or Week Number)
102	General Activity – Minimal Activity	-1	Pretest, 9, and 11
	Repetitive Motions or Movements – Present	1	2, 4, and 5
152	General Activity – Minimal Activity	-1	1, 5, 7, 8, 9, 10, 11 and 12
204	Repetitive Motions or Movements – Present	1	Pretest, 1, 2, 4, 6, 7, 8, 9, 10, 11, and 12
	Stargazing – Present	1	Pretest, 2, 3, 4, 5, 6, 7, 8, 10, and 12
251	General Activity – Minimal Activity	-1	Pretest
	General Activity – Heightened	1	6
	Repetitive Motions or Movements – Present	1	6
253	Self-mutilation – Present	1	4
	Piloerection – Present	1	2
254	General Activity – Minimal Activity	-1	11
302	Mentation/Behavior – Anxious, enhanced arousal	1	Pretest
303	Vocalizations – Moderate, distressed	1	Pretest
	Repetitive Motions or Movements – Present	1	3
304	Repetitive Motions or Movements – Present	1	1
	Stargazing – Present	1	1, 2, 3, 4, 5, 6, 7, 8, 10, and 12
351	General Activity – Heightened	1	Pretest
	Repetitive Motions or Movements – Present	1	Pretest
353	General Activity – Minimal Activity	-1	2, 3, 5, 6, and 10
	Repetitive Motions or Movements – Present	1	7
354	Mentation/Behavior – Anxious, enhanced arousal	1	Pretest

7.7 NERVE CONDUCTANCE VELOCITY EVALUATION

Contributing Report: Appendix G

There were no abnormal nerve conduction findings observed in the tibial nerve prior to the initiation of dosing (Pretest Assessment).

Following the administration of ARM-101 in male and female Cynomolgus monkeys, there were no apparent test article related effects observed in the Nerve Conduction assessment of the tibial nerve when comparing dose groups or comparing pre-dose recordings to subsequent Week 6 and/or Week 12 recordings.

7.8 ECG Evaluation

Contributing Report: Appendix H

There were no abnormal ECG findings observed prior to the initiation of dosing that precluded the placement of any animal on study.

Following the administration of ARM-101 in male and female Cynomolgus monkeys, there were no apparent test article related effects observed in the qualitative assessment of ECG waveforms when comparing dose groups or comparing pre-dose recordings to subsequent Week 6 and/or Week 12 recordings. A single premature ventricular complex was observed in Animal 301 during Week 6, but this abnormality is not considered dose related as it is considered a background arrythmia commonly observed in Cynomolgus monkeys.

7.9 Clinical Pathology Evaluation

Contributing Report: Appendix I

Principal Investigator Report (PIR): Appendix I

There were no test article-related findings noted in the hematology, coagulation, serum chemistry, or urinalysis data.

Cardiac Troponin I (cTnI) analysis on all available samples from the control group and each dose group of the 6-week and 12-week cohort animals demonstrated minimal detectable values of troponin I in these samples indicating there were no myocardial injuries.

7.10 ELISpot Analysis

Contributing Report: Appendix J

All of the study animals in treatment Group 1 (Control), Group 2 (6×10^{13} vg), and Group 3 (1.2×10^{14} vg) showed high levels of responsivity to positive control mitogen conditions ranging from 292.5 to 3735 SFC/ 1×10^{6} PBMCs while showing minimal background responsivity to unstimulated conditions ranging from 0 to 7.5 SFC/ 1×10^{6} PBMCs. A single study animal, Animal 152 (Group 1), also showed a consistent response to the CMV peptide pool.

There were no identifiable trends associated with the dose in any group. While responses to positive control mitogen conditions were consistent across all study animals and test plates, none of the study animals showed detectable responses to the test antigen AAV9 at the Pre-dose, 6-week, or 12-week time points (Figure 1).

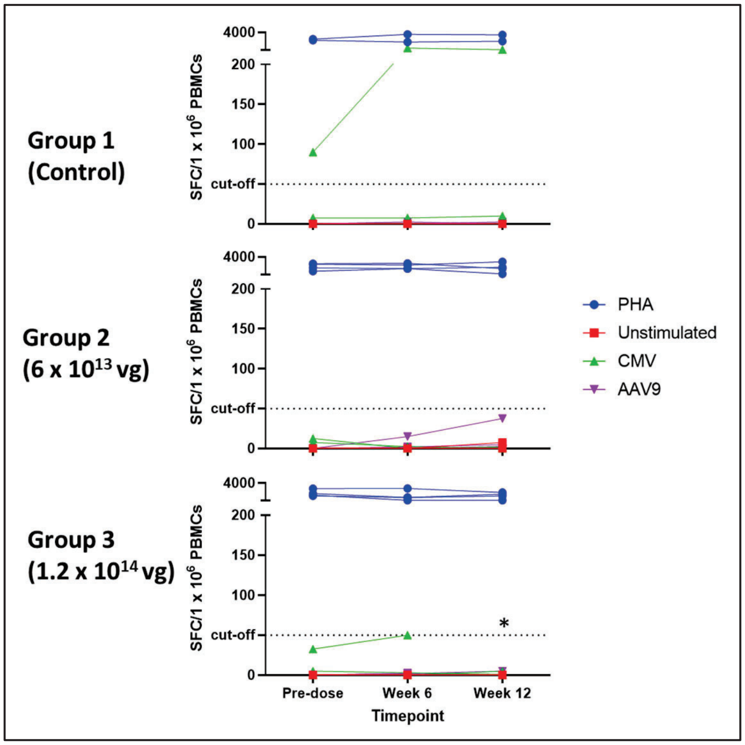

Figure 1. Time course of AAV-9 specific T cell responses by IFN-γ ELISpot

Line graph showing individual responses by animals belonging to Group 1 (Control, Upper), Group 2 (6×10^{13} vg, Center) and Group 3 (1.2×10^{14} vg, Lower) study cohorts. Individual responses to PHA, Unstimulated, CMV and AAV9 antigen stimulation conditions are shown at the Pre-Dose, Week 6, and Week 12 timepoints. The number of SFCs per 1×10^6 PBMCs plated is shown on the y-axis, for each study timepoint (x-axis). The cut-off value of 50 SFC/ 1×10^6 PBMCs for identifying positive responses is depicted by a dotted line. * = CMV antigen stimulation was not performed for Animal 353 at the Week 12 timepoint due to limited sample availability.

7.11 ANATOMIC PATHOLOGY EVALUATION

Contributing Report: Appendix K

7.11.1 Terminal Body Weights and Organ Weights

There were not enough animals assigned to each cohort to statistically compare treatment groups (Groups 2 and 3) to controls (Group 1).

7.11.2 Gross Pathology

In the 6-week cohort, no test article-associated gross lesions were identified.

In the 12-week cohort, female Animal 353 had enlarged inguinal and mesenteric lymph nodes which corresponded to the microscopic finding of increased cellularity, lymphocyte.

Remaining gross lesions are common background lesions in nonhuman primates unrelated to treatment.

7.11.3 Histopathology

Histopathologic grades were assigned according to the following scale: minimal (1), mild (2), moderate (3), and marked (4), based on increasing extent and/or complexity of change.

Test article-related microscopic findings were limited to dorsal root ganglion and spinal cord and were present in both 6-week and 12-week cohorts. The dorsal root ganglion lesions included degeneration and necrosis of neurons and autophagy of neurons. Spinal cord lesions consisted of Wallerian degeneration of axons. Intracellular vacuolization of dorsal root ganglion neurons was noted but was a fixation artifact. See Table 10 and Table 11 for a summary of the test article-related microscopic findings.

In the 6-week cohort, Group 2 male Animal 202 had a mild cellular infiltrate of the liver consisting of mononuclear cells admixed with eosinophils. The infiltrate was primarily portal but extended into the hepatic parenchyma.

In the 12-week cohort, Group 3 male Animal 304 had mild, focal, mononuclear, perivascular infiltrate of the cerebrum. Additionally, Group 3 female Animal 353 had increased cellularity, lymphocyte, in inguinal and mesenteric lymph nodes.

Remaining lesions are common background lesions in nonhuman primates unrelated to treatment.

Table 10. Summary of Test Article-Related Microscopic Findings in the 6-Week Cohort (Day 43) Male and Female Cynomolgus Macaques

Sex	Males			Females		
**Group**	**1**	**2**	**3**	**1**	**2**	**3**
**Dose (Total vg)**	**0**	**6×10^{13}**	**1.2×10^{14}**	**0**	**6×10^{13}**	**1.2×10^{14}**
**No. of animals examined**	**1**	**2**	**2**	**1**	**2**	**2**
**Spinal cord (no. examined)**	**1**	**2**	**2**	**1**	**2**	**2**
Lumbar, axon degeneration, Wallerian	0	0	0	0	0	1 (1)
**Dorsal root ganglion (no. examined)**	**1**	**2**	**2**	**1**	**2**	**2**
Cervical (neck), necrosis, neuron	0	0	1 (1)	0	0	0
Cervical (neck), degeneration, neuron	0	0	1 (2)	0	0	0
Cervical (neck), autophagy, neuron	0	0	0	0	0	1 (1)
Thoracic, degeneration, neuron	0	1 (1)	1 (1)	0	0	0
Thoracic, autophagy, neuron	0	0	1 (1)	0	0	1 (1)
Thoracic, increased cellularity, mononuclear cell	0	1 (2)	0	0	0	0
Lumbar, degeneration, neuron	0	1 (1)	0	0	0	0
Lumbar, autophagy, neuron	0	0	0	0	0	1 (1)
Lumbar, increased cellularity, mononuclear cell	0	1 (2)	0	0	0	0

Average severity (in parentheses) was calculated by adding all the grades of affected animals in the group and diving by the number affected.

Table 11. Summary of Test Article-Related Microscopic Findings in the 12-Week Cohort (Day 85) Male and Female Cynomolgus Macaques

Sex	Males			Females		
Group	1	2	3	1	2	3
Dose (Total vg)	0	6×10^{13}	1.2×10^{14}	0	6×10^{13}	1.2×10^{14}
No. of animals examined	1	2	2	1	2	2
**Spinal cord (no. examined)**	**1**	**2**	**2**	**1**	**2**	**2**
Cervical (neck), axon degeneration, Wallerian	0	0	0	0	1 (1)	0
Thoracic, axon degeneration, Wallerian	0	0	1 (1)	0	1 (1)	0
Lumbar, axon degeneration, Wallerian	0	1 (1)	0	0	1 (1)	0
**Dorsal root ganglion (no. examined)**	**1**	**2**	**2**	**1**	**2**	**2**
Cervical (neck), necrosis, neuron	0	1 (1)	0	0	1 (1)	0
Cervical (neck), degeneration, neuron	0	1 (1)	1 (1)	0	1 (1)	0
Cervical (neck), increased cellularity, mononuclear cell	0	0	0	0	1 (2)	0
Thoracic, necrosis, neuron	0	0	1 (1)	0	0	0
Thoracic, degeneration, neuron	0	0	1 (1)	0	0	1 (1)
Thoracic, autophagy, neuron	0	0	0	1 (1)	0	0
Lumbar, necrosis, neuron	0	0	1 (1)	0	0	0
Lumbar, degeneration, neuron	0	1 (1)	0	0	1 (1)	0
Lumbar, autophagy, neuron	0	0	0	0	0	1 (1)
Lumbar, increased cellularity, mononuclear cell	0	0	0	0	1 (2)	1 (2)

The average severity (in parathesis) was calculated by adding all the grades of affected animals in the group and dividing by the number affected.

7.12 Biodistribution Analysis by dPCR

Contributing Report: Appendix L

Precision Principal Investigator Report (PIR): Appendix L

Biodistribution pattern of AAV2-ITR encoded vector copies per µg DNA:

AAV2-ITR vector genome copy number (copies per µg of DNA) were detected in all of the low dose (Group 2; 6×10^{13} vg) and high dose (Group 3; 1.2×10^{14} vg) animal tissues harvested at both the Day 43 (Figure 2) and Day 85 (Figure 3) time points evaluated. The data represented are not normalized to the housekeeping gene B2M. The estimated mean log vector genome copies in the low dose group and in the high dose group were 4.95 and 5.06, respectively, when compared to the control group (0.18 mean log; Group 1) indicating no dose response within dosed groups, but a significant dose response between the control and dosed groups. There were no significant sex-related differences observed in the mean log copies of the vector genome in males or females at Day 43 or Day 85 (see Appendix L for details).

Group 1 (Control; 0 vg per animal)

Vector genomes were either ≤ 0 copies/µg DNA, Indeterminate (IND) or below quantification Limit (BQL) in any of the control (Group 1) animal tissues at both Day 43 and Day 85 time points.

Group 2 (Low Dose; 6×10^{13} vg per animal)

Highest vector genome copies were generally observed in the liver tissue, spleen, and CNS tissues, such as dorsal root ganglia, spinal cord, and brain, compared to other organs at Day 43 (Figure 2). All the nerve tissues (Femoral, Median, Sciatic, and Ulnar) showed high mean log vector copies per µg DNA at Day 43 (Figure 2) for low dose animal tissues which persisted until Day 85 (Figure 3).

Group 3 (High Dose; 1.2×10^{14} vg per animal)

High dose (Group 3) showed a similar level of vector copy number than low dose Group 2 in all tissues tested indicating minimal dose response except for DRG cervical and spinal cord cervical. Highest mean vector copies per µg DNA (6-8 mean log) was generally observed in the liver tissues, spleen and CNS organs, such as dorsal root ganglia and spinal cord, as compared to other organs at Day 43 (Figure 2). The same tissues had lower mean vector copies of 4-6 logs at Day 85 (Figure 3) compared to Day 43 except DRG lumbar and liver. All the nerve tissues (femoral, median, sciatic, and ulnar) showed high accumulation of the vector (4-6 mean log vector copies per µg DNA) at Day 43 for high dose animal tissues and persisted until Day 85.

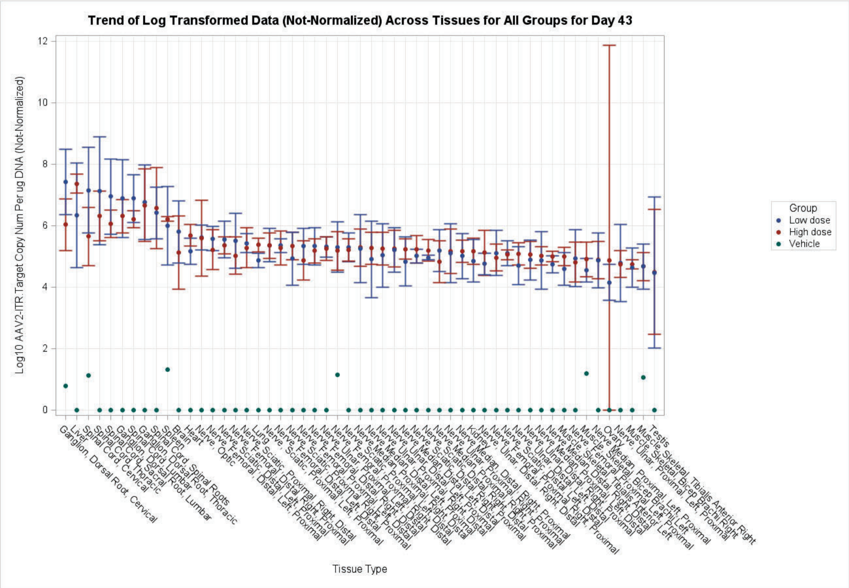

Figure 2. Trend of Log Transformed AAV2-ITR Mean Target Copy Number per µg DNA (Not Normalized to the housekeeping Gene B2M) Across Tissues for All Groups for Day 43

Error bars for the low dose and high dose groups are calculated based on the 95% confidence intervals for the mean of each of these groups. The error bars have been omitted for the control group.

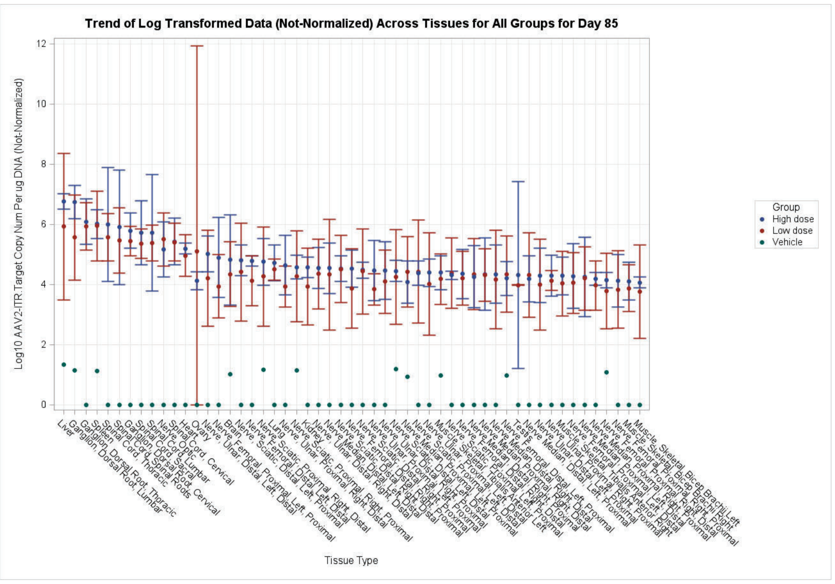

Figure 3. Trend of Log Transformed AAV2-ITR Mean Target Copy Number per µg DNA (Not Normalized to Housekeeping Gene B2M) Across Tissues for All Groups for Day 85

Error bars for the low dose and high dose groups are calculated based on the 95% confidence intervals for the mean of each of these groups. The error bars have been omitted for the control group.

7.13 BIODISTRIBUTION ANALYSIS BY RT-dPCR

Contributing Report: Appendix M

Precision Principal Investigator Report (PIR): Appendix M

Biodistribution pattern of AAV-encoded transgene miR871 expression

miR871 expression (copies per µg of cDNA) was detected in most of the low dose (Group 2; 6×10^{13} vg) and high dose (Group 3; 1.2×10^{14} vg) tissues harvested at both Day 43 (Figure 4) and Day 85 (Figure 5) time points tested, except ovary, testis, kidney, muscles, lungs, and spleen. The data represented are log-transformed and normalized to the housekeeping gene B2M. Non-normalized data is presented in the PIR (see Appendix M) but is not used for comparison and data analysis here. miR871 expression was none to minimally detected in any of the control (Group 1) animal tissues at any time point.

Assessment of trends in mean log-transformed miR871 copies per µg of cDNA at Day 43 (Figure 4) showed very high expression of miR871 (approximately ≥0.25-$\log_{10}$ or 2-fold) in most low dose group animal tissues such as spinal roots, spinal cord, femoral nerve, median nerve, and sciatic nerve. There was no significant dose response detected between the low and high dosed groups except for spinal roots, spinal cord sacral, and femoral proximal nerve at Day 43. Very low miR871 expression at Day 43 was observed in lungs, ovary, testis, kidney, optic nerve, and spleen. At Day 85 (Figure 5), a reduction in miR871 expression was observed for most of the tissues as compared to Day 43. Very low miR871 expression at Day 85 was observed in testis, ovary, optic nerve, skeletal muscle, kidney, lung, and spleen as expected (for details see Appendix M).

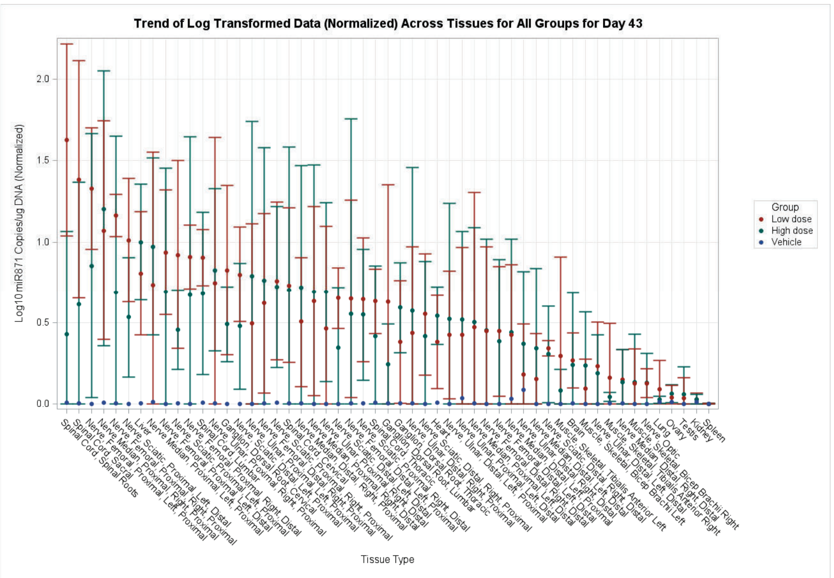

Figure 4. Assessment of Trends in Mean Log-Transformed miR871 Target Copy Number Per µg cDNA by Group Across Tissues for Day 43

Error bars are calculated based on the 95% confidence intervals for the mean of each dose level. The error bars have been omitted for the control group. Note: DNA represents cDNA.

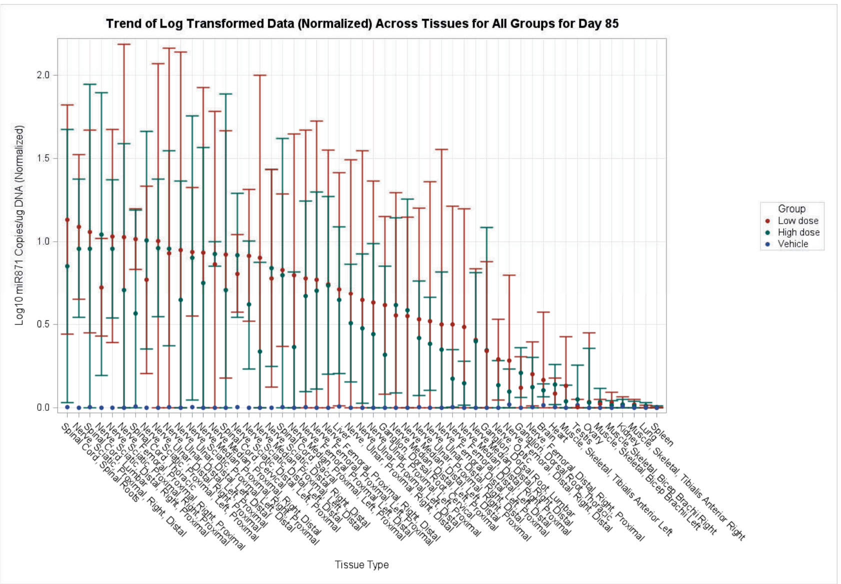

Figure 5. Assessment of Trends in Mean Log-Transformed miR871 Target Copy Number Per µg cDNA by Group Across Tissues for Day 85

Error bars are calculated based on the 95% confidence intervals for the mean of each dose level. The error bars have been omitted for the control group. Note: DNA represents cDNA.

Biodistribution pattern of endogenous PMP22 expression

Endogenous PMP22 expression (copies per µg of cDNA) was detected in most of the control, low dose (Group 2; 6×10^{13} vg), and high dose (Group 3; 1.2×10^{14} vg) tissues harvested at both Day 43 (Figure 6) and Day 85 (Figure 7) time points tested, except muscles, ovary, testis, heart, lung, liver, kidney, and spleen. The data represented are log-transformed and normalized to the housekeeping gene B2M. Non-normalized data is presented in the PIR (see Appendix M) but is not used for comparison and data analysis here.

Assessment of trends in mean log-transformed PMP22 copies per µg of cDNA at Day 43 (Figure 6) showed very high expression of PMP22 (approximately $\geq$0.1-$\log_{10}$) in control group tissues such as spinal roots, spinal cord, all nerve tissues, dorsal root ganglion, and brain. In general, there was significantly reduced PMP22 expression detected in the low and high dosed groups as compared to control in the median nerve, femoral nerve, sciatic nerve, spinal cord, ulnar nerve, dorsal root ganglion, and optic nerve tissue (for details see Appendix M).

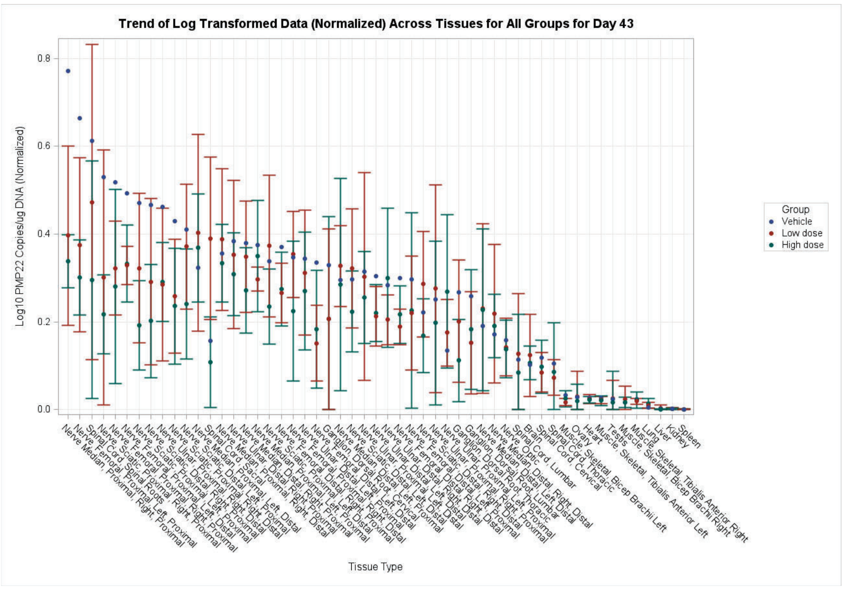

Figure 6. Assessment of Trends in Mean Log-Transformed PMP22 Target Copy Number Per µg cDNA by Group Across Tissues for Day 43

Error bars are calculated based on the 95% confidence intervals for the mean of each dose level. The error bars have been omitted for the control group. Note: DNA represents cDNA.

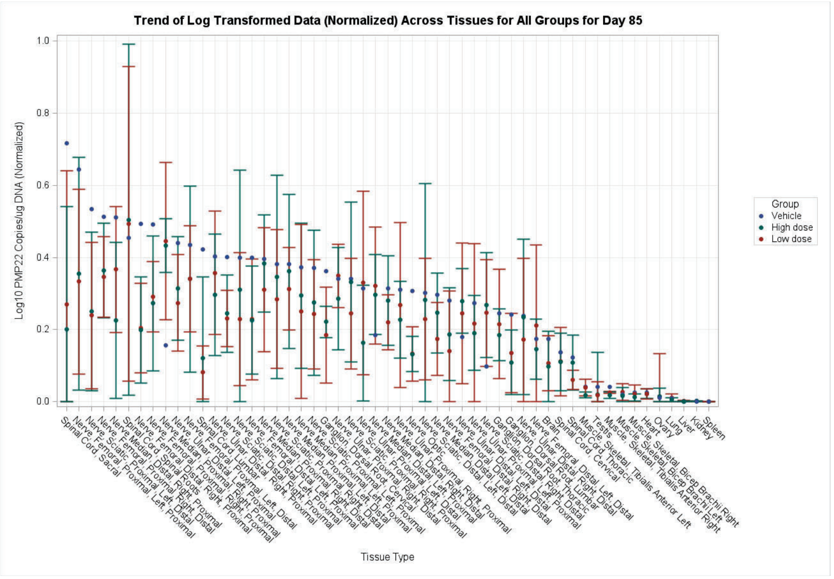

Figure 7. Assessment of Trends in Mean Log-Transformed PMP22 Target Copy Number Per µg cDNA by Group Across Tissues for Day 85

Error bars are calculated based on the 95% confidence intervals for the mean of each dose level. The error bars have been omitted for the control group. Note: DNA represents cDNA.

Comparison of miR871 and PMP22 expression in nerve tissues

Examining the differences between miR871 and PMP22 for nerve tissues for log-transformed data normalized to B2M across different tissues showed correlation between high miR871 expression to reduced endogenous PMP22 expression. As compared to the control group (Group 1), the PMP22 expression was significantly lower in tissues of the low dose and high dose groups at Day 43 (Figure 8 and Figure 9) and persisted until Day 85 (Figure 10 and Figure 11).

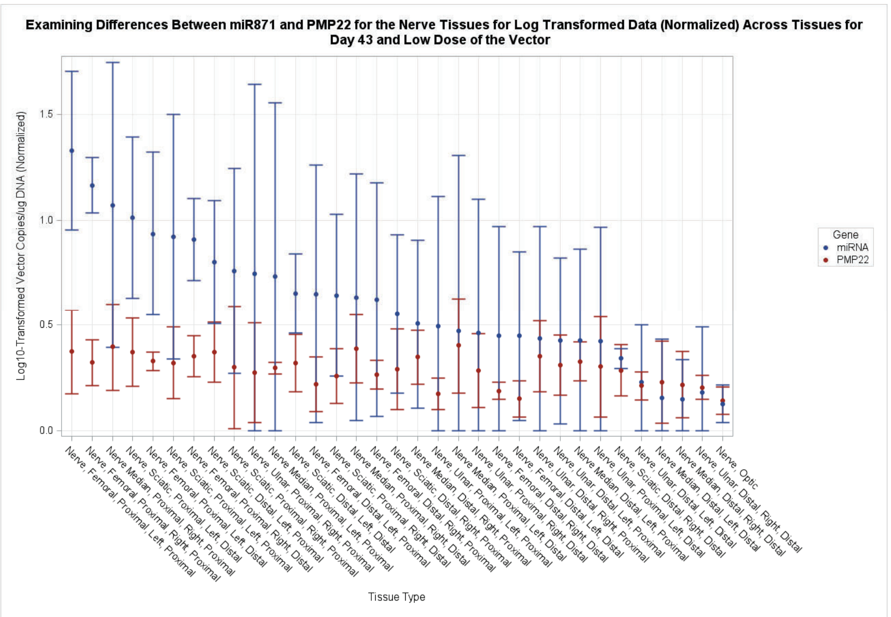

Figure 8. **Examining Differences between miR871 and PMP22 for the Nerve Tissues for Log-Transformed Data (Normalized to B2M) across Nerve Tissues for Day 43 and Low Dose of the Vector**

Error bars are calculated based on the 95% confidence intervals for the mean of each dose level. Note: DNA represents cDNA.

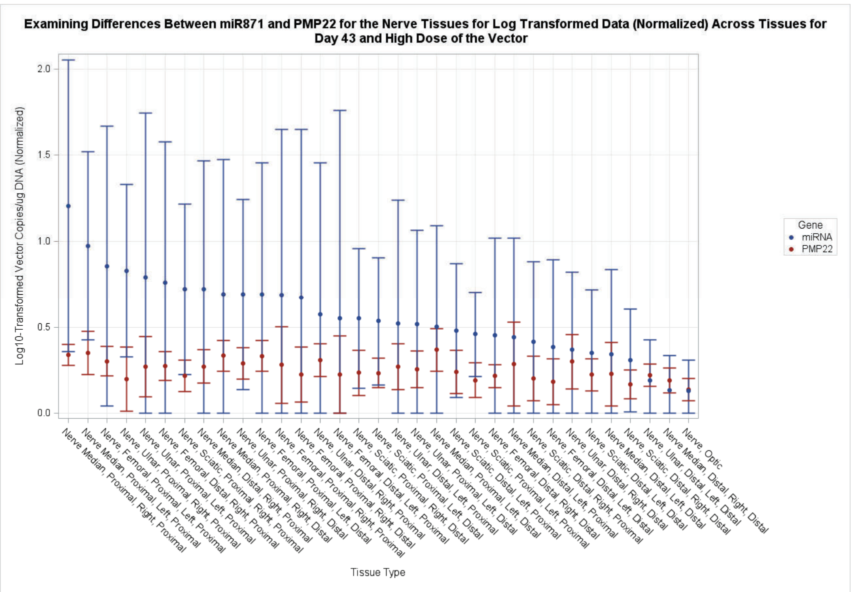

Figure 9. Examining Differences between miR871 and PMP22 for the Nerve Tissues for Log-Transformed Data (Normalized to B2M) across Nerve Tissues for Day 43 and High Dose of the Vector

Error bars are calculated based on the 95% confidence intervals for the mean of each dose level. Note: DNA represents cDNA.

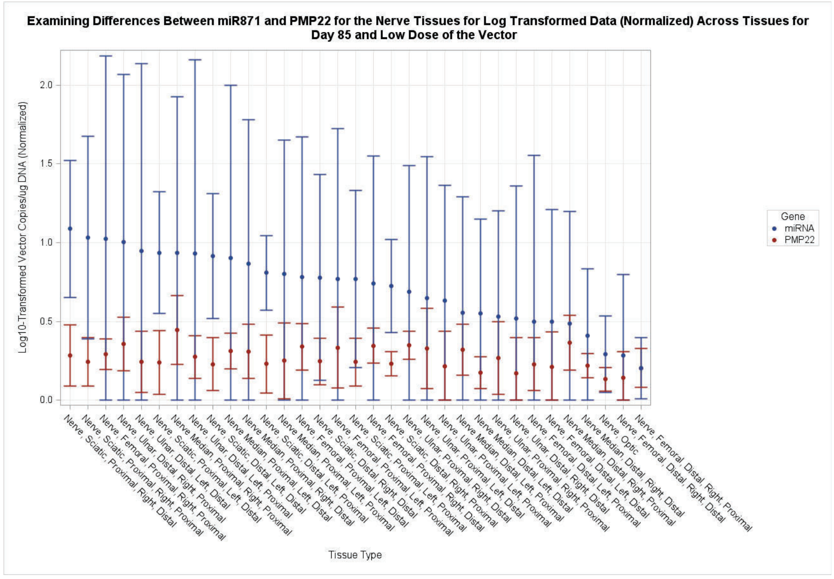

Figure 10. Examining Differences between miR871 and PMP22 for the Nerve Tissues for Log-Transformed Data (Normalized to B2M) across Nerve Tissues for Day 85 and Low Dose of the Vector

Error bars are calculated based on the 95% confidence intervals for the mean of each dose level. Note: DNA represents cDNA.

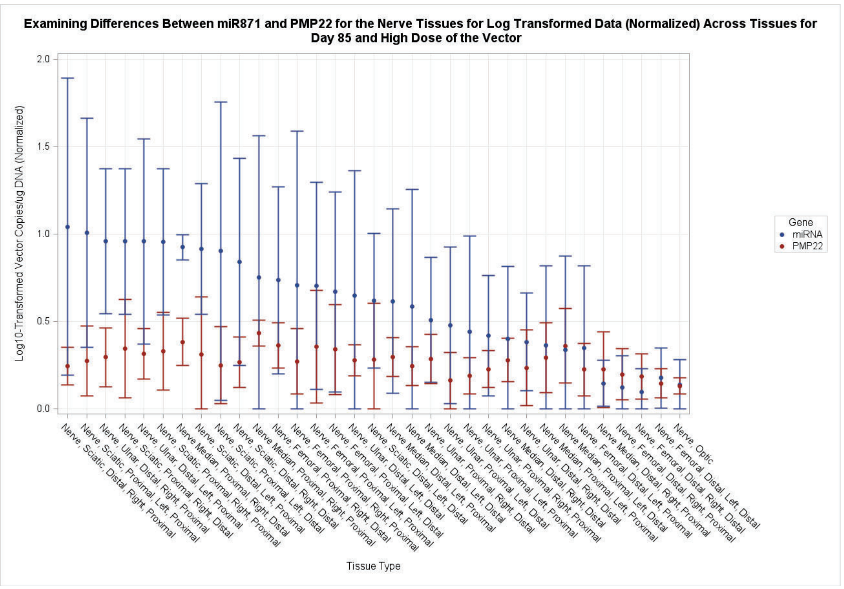

Figure 11. Examining differences between miR871 and PMP22 for the Nerve Tissues for Log-Transformed Data (Normalized to B2M) across Nerve Tissues for Day 85 and High Dose of the Vector

Error bars are calculated based on the 95% confidence intervals for the mean of each dose level. Note: DNA represents cDNA.

8 DISCUSSION

Twenty Cynomolgus monkeys (10 males and 10 females) were successfully dosed with either the control article (Formulation Buffer) or the test article (scAAV9.U6miR871 [ARM-101]). Four (4) animals in Group 1 received administration of the control article (Formulation Buffer), whereas 8 animals in Group 2 received administration of up to 6 × 10^{13} vg (Low Dose) and 8 animals in Group 3 received administration of up to 1.2 × 10^{14} vg (High Dose) of ARM-101. A dose volume of approximately 3 mL per animal was administered to each animal as a single dose via intrathecal slow infusion over approximately 6 hours via a surgically implanted catheter. Following a single dose administration, 10 animals from the 6-week cohort were terminated on Day 43 and the remaining 10 animals from the 12-week cohort were terminated on Day 85.

All animals survived until scheduled termination. There were no test article-related body weight, food consumption or ocular changes observed in any of the groups during the study. There were no adverse clinical observations attributed to the test article dose administration.

There were no test article-related nerve conduction findings observed in the tibial nerve post dose administration. There were no apparent test article related effects observed in the qualitative assessment of ECG waveforms when comparing dose groups or comparing pre-dose recordings to subsequent 6-week (Day 43) and/or 12-week (Day 85) cohorts.

There were no test article-related neurobehavioral findings observed in any dose group during the study. Results from the study indicates the changes in the score were ambiguous and merely a change of a score by a factor of 1 in animals both in the control group as well as in the those receiving scAAV9.U6miR871 (ARM-101).

There were no test article-related findings noted in the hematology, coagulation, serum chemistry, or urinalysis data.

Cardiac Troponin I (cTnI) analysis on all available samples from control and each dose group of 6-week and 12-week cohort animals demonstrated minimal detectable values of troponin I in these samples indicating there were no myocardial injuries.

In the 6-week cohort (Day 43), no test article-associated gross lesions were identified. In the 12-week cohort (Day 85), one female had enlarged inguinal and mesenteric lymph nodes which corresponded to the microscopic finding of increased cellularity, lymphocyte. Remaining gross lesions were common background lesions observed in nonhuman primates unrelated to treatment.

The 6-week or 12-week cohort animals had minimal to mild lesions within the spinal cord and dorsal root ganglion. Lesions were present in the 6-week and 12-week cohorts to the same or similar degree. Within the spinal cord, there was minimal Wallerian degeneration of axons. In dorsal root ganglions, there was minimal degeneration and necrosis of neurons. Occasionally, there was increased mononuclear cells within the ganglions. None of the animals receiving test article displayed any clinical signs. Autophagy noted in several dorsal root ganglions represents programed cell death of neurons and is a common background finding in nonhuman primates but may also follow lethal injury to sensory cells. Intracytoplasmic vacuolization of neurons as seen in this study is consistent with fixation

artifact. Enlarged inguinal and mesenteric lymph nodes from Animal 353 corresponded to microscopic finding of increased cellularity, lymphocyte and is indicative of nonspecific immune stimulation which may or may not be associated with test article. The cause of focal, perivascular, mononuclear infiltrate in Animal 304 is unknown but may be induced by mild non-specific trauma.

There were no identifiable test article-related trends in any group for IFNë ELISpot evaluation. While responses to positive control mitogen conditions were consistent across all study animals and test plates, none of the study animals showed detectable responses to the test antigen AAV9 at the Pretest, 6-week, or 12-week time points.

Biodistribution of the vector AAV2-ITR of ARM-101 test article upon intrathecal slow infusion showed greater prevalence of vector on Day 43 compared to Control (mean log copies higher than the Day 85 mean) in the CNS (spinal cord, dorsal root ganglia, and brain), liver and heart. All the nerve tissues (Femoral, Median, Sciatic, Ulnar) and spleen showed high vector copies per µg DNA at Day 43 and persisted until Day 85. There was no dose response observed in dosed groups but showed very high vector copies when compared to the Control group. There were no sex-related differences observed in the mean copies of the vector genome in males and females.

Biodistribution of the miR871 transgene of ARM-101 test article showed greater prevalence of the transgene on Day 43 for both low dose and high dose compared to Control in the CNS (spinal cord, dorsal root ganglia, femoral nerve, median nerve, sciatic nerve, ulnar nerve and brain), and liver. This high expression was observed to be persistent until Day 85. There was minimal dose response observed in dosed groups (Low 6×10^{13} vg or High 1.2×10^{14}vg per animal Dose) but showed significantly high miR871 transgene expression when compared to the Control group. Examining the differences between miR871 and PMP22 for nerve tissues for log-transformed data normalized to B2M across different tissues showed correlation between high miR871 expression to reduced endogenous PMP22 expression.

In conclusion, dose administration of scAAV9.U6miR871 (ARM-101) via a single approximate 6 hour slow infusion at 6×10^{13} vg/animal (low dose) and 1.2×10^{14} vg/animal (high dose) to male and female Cynomolgus monkeys was well tolerated and deemed safe over 43 days (6-week cohort) and 85 days (12-week cohort). Additionally, the highest vector accumulation was observed in the spinal cord, dorsal root ganglion, liver and heart. All the nerve tissues (Femoral, Median, Sciatic, Ulnar) also showed high concentration of the vector at Day 43 for high dose animal tissues which was observed to persist through Day 85. ARM-101 vector showed reduction in endogenous PMP22 gene expression at both low (6×10^{13} vg) and high (1.2×10^{14} vg) dose as compared to control group in nerve tissues and the reduced expression persisted until Day 85.

9 ACKNOWLEDGEMENTS

Acknowledgements of the principal contributors participating in the performance of this study are presented below.

Table 12. Principal Contributors

Role	Name
Study Director	Sapna Varia, PhD
Director of Laboratory Animal Resources	Christine M. Commons, BS
Director of Veterinary Sciences and Inhalation Technology	Roy Barnewall, PhD, DVM
Attending Veterinarian	Sarah Young, DMV, MS
Director of Pathology	Sabrina N. McGraw, DVM, MS, PhD, DACVP
Director of Analytical Sciences	Sapna Varia, PhD
Sr Director of Scientific Strategy and Innovation	Victoria Best, PhD
Director of Quality Operations	Shawnda V. Armentrout, BSQSM
VP of CRO Operations	Dean J. Kobs, MS
Testing Facility Management	Dean J. Kobs, MS
Associate Director of Study Management	Tessa R. Moir-Savitz, MS<br>Karen E Elsass, BS, DABT
Clinical Pathologist	Eric Snook, DVM, PhD, DACVP
Anatomic Pathologist	Joseph Novak, DVM, DACVP
Principal Investigator, Troponin Analysis	Katelin Leonesio, BS
Principal Investigator, Biostatistical Analysis (dPCR and RT-dPCR)	Zachary Cole (See DEV-003002 in Appendix A)

10 REFERENCES

National Research Council (NRC). 2011. *Guide for the Care and Use of Laboratory Animals*. 8th Ed. National Academies Press, Washington D.C.

U.S. Food and Drug Administration (1987). Guidance for Industry. 21 CFR Part 58 Good Laboratory Practice Regulations, Final Rule.

Appendix A Protocol, Amendments, and Summary of Deviations

A 12-Week Single Dose Biodistribution and Safety Study in Cynomolgus Monkeys

Amended Protocol 7

Study Number A-579-01

Armatus Bio, Inc.
1275 Kinnear Road, Columbus, OH 43212

GLP Compliance Status: GLP

AmplifyBio
1425 State Route 142 (Plain City-Georgesville Road)
West Jefferson, OH 43162

SIGNATURES

APPROVED, TESTING FACILITY:

DocuSigned by:
Sapna Varia
Signer Name: Sapna Varia
Signing Reason: I approve this document
Signing Time: 5/15/2023 | 6:15:30 PM EDT
5D64C6F0F9AF4A7B9EBF31D648E66020

5/15/2023

Sapna Varia, PhD
Study Director

Date

APPROVED, SPONSOR:

DocuSigned by:
Brian Price
Signer Name: Brian Price
Signing Reason: I approve this document
Signing Time: 5/15/2023 | 2:10:47 PM PDT
1974D3F004604A9E9D9FAF8A98283D0A

5/15/2023

Brian Price, PhD
Sponsors Representative

Date

TABLE OF CONTENTS

1.0 Purpose 4
2.0 Background 4
3.0 Regulatory Compliance 4
4.0 Testing Facility and Study Director 5
5.0 Sponsor and Sponsor's Representative 6
6.0 Proposed Study Schedule 6
7.0 Route and Duration of Administration 6
8.0 Test System 6
9.0 Animal Care, Housing, and Environmental Conditions 7
10.0 Test and Control Articles 8
11.0 Experimental Design .... a
12.0 Computer Systems for Data Management 20
13.0 Statistical Analysis 20
14.0 Reporting 21
15.0 Storage of Study Materials and Records Retention 21
16.0 References 21

1.0 PURPOSE

The purpose of this study is to evaluate the potential biodistribution and safety of scAAV9.U6miR871 [***(ARM-101)***][3] when administered once via intrathecal infusion to Cynomolgus monkeys for 12 weeks.

2.0 BACKGROUND

Charcot-Marie-Tooth disease type 1A (CMT1A) is the peripheral neurological disorder caused by Peripheral Myelin Protein (PMP22) gene duplication. Overexpression of WT PMP22 in Schwann cells destabilizes the myelin sheath, leading to demyelination and ultimately to secondary axonal loss and disability. The most direct route to CMT1A therapy will involve reducing PMP22 to normal levels. To accomplish this, Armatus Bio has developed a gene therapy strategy to reduce PMP22 using artificial miRNAs (miR871) packaged into an adeno-associated virus (AAV9) targeting human PMP22.

3.0 REGULATORY COMPLIANCE

This study will be conducted in compliance with the current version of the United States Food and Drug Administration's (FDA) Good Laboratory Practice (GLP) Regulations, 21 CFR Part 58 for the conduct of non-clinical laboratory studies with the following exceptions:

- The test/control article are research grade products and were not manufactured and characterized according to GLP compliance. A Certificate of Analysis (CoA) will be provided.
- Test article characterization will not be performed under GLP compliance.
- There will not be any formulation dose analysis as the test article will be received as ready to dose [***or may be diluted to required concentration with the*** [~~***formulation buffer***~~][1] ***vehicle (0.9% saline)***][6].
- Stability of the bulk test article and prepared formulation will not be tested or reported during this study.
- [~~In this study, the vehicle for test article formulation is used as a control article.~~][3] The control article formulations will [***not***][6] be analyzed only for concentration of test article and not for homogeneity. Control article formulations will not be subjected to stability testing.
- The [~~final disposition and~~][6] results from Cerebrospinal fluid (CSF) analysis or Bank tissues will not be reported under this study. Cerebrospinal fluid [***, PBMC aliquot(s)***[***, residual/retained biodistribution samples*** ~~***shipped to Sponsor***~~][6] ***or Bank tissue***][1] samples for potential analysis will be shipped to the Sponsor [***or Sponsor-designee***][6] on dry ice [***or LN2 shipper***][6] for potential future evaluation by the Sponsor. This additional work is not expected to have an impact on the conclusions of

this study because the Cerebrospinal fluid [***, PBMC aliquot(s)***[***, residual/retained biodistribution samples*** ~~shipped to Sponsor~~]6 ***or Bank tissue*** [***(shipped to Sponsor or Sponsor-designee)***]6]1 analysis will not impact the assessment of the safety and biodistribution evaluation of the test article. This analysis and evaluation will not interfere with the conduct of this study and will not jeopardize the GLP compliance of this study. The analysis performed on these samples are not considered to be part of this study and the results will not be presented in this study report. The samples and the results from analysis of the samples are under the responsibility of the Sponsor.

- Nicolet Viking EDX system and associated software used for nerve conduction study will not be validated.

[• ***The method used for biodistribution analysis by Digital PCR (RT-dPCR) will not be qualified.***]6

[• ***Statistical analysis for dPCR and RT-dPCR will not be performed in accordance to GLP compliance.***]7

Any deviations from GLP or the study protocol will be documented in the study file and presented in the final report. Deviations from SOP will be documented in the study file and presented in the final report as warranted.

Portions of this study to be performed at designated test sites will adhere to the applicable SOPs of the test site.

4.0 TESTING FACILITY AND STUDY DIRECTOR

4.1 TESTING FACILITY

AmplifyBio
1425 State Route 142 (Plain City-Georgesville Road)
West Jefferson, OH 43162

4.2 STUDY DIRECTOR

Sapna Varia, PhD
Telephone: (614) 642-5325
Email: svaria@amplify-bio.com

[*TEST* [~~*SITE*~~ *SITES*][7] *AND PRINCIPAL* [~~*INVESTIGATOR*~~ *INVESTIGATORS*][7]

ANTECH DIAGNOSTICS GLP

Test Site for:	*Troponin Analysis*
Address:	*600 Airport Blvd.*<br>*Suite 500*<br>*Morrisville, NC 27560*
Principal Investigator:	*Katelin Leonesio*<br>*Telephone: (919) 277-0822*<br>*Email: Katelin.Leonesio@antechmail.com*][1]

[**PRECISION** *CONSULTING, LLC*

Test Site for:	*Statistical Analysis for dPCR and RT-dPCR*
Address:	*1980 Festival Plaza Drive,*<br>*Suite 300*<br>*Las Vegas, Nevada 89135*
Principal Investigator:	*Ryan Kotowski*<br>*Telephone: (646) 553-4730*<br>*Email:*<br>*institutionalclients@precisionconsultingcompany.com*][7]

5.0 SPONSOR AND SPONSOR'S REPRESENTATIVE

5.1 SPONSOR

Armatus Bio, Inc.
1275 Kinnear Road,
Columbus, OH 43212

5.2 SPONSOR'S REPRESENTATIVE

Brian Price, PhD
Co-Founder and Chief Technical Officer
Telephone: (614) 507-2695
Email: bprice@armatusbio.com

6.0 PROPOSED STUDY SCHEDULE

Proposed dates for selected study events are listed below.

Animal transfer:	May 16, 2023
Lumbar IT catheter implantation surgery:	May 17, 2023 – May 19, 2023 ([~~10~~ ***11***][1] NHPs)<br>May 22, 2023 – May [~~24~~ ***23***][1], 2023 ([~~10~~ ***9***][1] NHPs)
First day of dosing:	June 20, 2023 – June 22, 2023
6-week necropsy (Day 43):	August 01, 2023 – August 03, 2023
12-week necropsy (Day 85):	September 12, 2023 – September 14, 2023
Draft report due:	To be determined

7.0 ROUTE AND DURATION OF ADMINISTRATION

The test article will be administered one time as an approximate 6-hour intrathecal (IT) slow infusion as this is the intended route of clinical administration.

8.0 TEST SYSTEM

Species:	*Macaca fascicularis*
Strain and Substrain:	Cynomolgus monkey
Source:	Testing facility holding pool origin: [~~Primate Products, Inc., Immokalee, FL~~ ***Envigo, Alice, TX***][1]
Age of Animals on Day 1:	1.5 to 3 years
Weight of Animals on Day 1:	[~~1.5~~ ***2***][1] to [~~5~~ ***6***][1] kg
Number of Animals Required for Study:	20

Additional animals (2/sex) will be present to ensure the appropriate number of healthy animals are available for study. Disposition of animals not used for study will be documented in the study file.

8.1 Test System Justification

The cynomolgus monkey is an accepted species that is frequently used in efficacy evaluations of drugs and vaccines intended for human use and is, therefore, an appropriate model for this study. At this time, studies in laboratory animals provide the best available basis for extrapolation to humans and are required to support regulatory submissions. Acceptable models which do not use live animals currently do not exist. The number of animals to be used is the minimum number necessary to yield meaningful results.

9.0 ANIMAL CARE, HOUSING, AND ENVIRONMENTAL CONDITIONS

General procedures for animal care and housing will meet AAALAC International recommendations, current requirements stated in the "Guide for the Care and Use of Laboratory Animals" (National Research Council, Current Edition), current requirements as stated by the U.S. Department of Agriculture through the Animal Welfare Act, as amended, and will conform to applicable testing facility SOP. Prior to study initiation, the study design was reviewed and approved by the Institutional Animal Care and Use Committee (IACUC).

9.1 Quarantine and Acclimation

Animals were quarantined and acclimated as part of the testing facility's holding pool (TOXBC HOLD). [~~Animals will be rescreened once during quarantine for negative AAV9 nAb status~~][1].

Prior to the start of dosing, animals will be conditioned to restraint in chairs. To facilitate the intrathecal (IT) infusions and blood collections, animals will be progressively acclimated to accept up to 6 hours of chair restraint.

A gross necropsy will be performed on any animals that die or are terminated as moribund prior to the first dose administration, with tissue collection, as needed. Moribund animals will be humanely euthanized.

9.2 Lumbar Intrathecal Catheter Surgical Implantation

Animals on the study (20 NHPs) will be surgically implanted with lumbar intrathecal catheters according to a study-specific method prior to Day 1. If needed, additional animals will be surgically implanted with lumbar intrathecal catheters before being placed on the study.

9.3 Animal Housing and Environmental Conditions

Animals will be socially housed during the study to the extent possible. Animals may be individually housed during study events and if events (overt clinical signs, significant aggression with cage mate, etc.) warrant separation. Any animal that is single housed will receive enhanced enrichment as determined by the veterinary staff and testing facility SOP.

Temperature and humidity of the study room will be set to 74°F and 50%, respectively. Temperature and humidity ranges will be maintained per testing facility SOP, and actual ranges will be reported. The light cycle will be set to maintain 12 hours on/12 hours off.

9.4 Feed

Animals will be provided PMI Certified Primate Diet (LabDiet 5048) twice daily, except on the day of receipt when feed may only be offered once, during specified fasting periods, or when the animal is away from its home cage for study events (e.g., when placed in restraint chairs for dose administrations and blood collections). The diet will also be supplemented with fruits, vegetables, or other supplemental enrichment.

Analytical reports of each lot of feed will be provided by the manufacturer. Reports will be reviewed to ensure acceptable standards and freedom from levels of contaminants that may interfere with the purpose or conduct of the study. There are no known contaminants in the feed capable of interfering with the study. Results will be retained in testing facility records.

9.5 Water

Animals will be provided fresh water *ad libitum* from the Village of West Jefferson municipal water supply by an automatic watering system except when removed from the home cage or during study activities when water may not be available.

The water supply is analyzed periodically to ensure acceptable standards and freedom from levels of contaminants that may interfere with the purpose or conduct of the study. There are no known contaminants in the water capable of interfering with the study. Results will be retained in testing facility records.

10.0 TEST AND CONTROL ARTICLES

Records of receipt, use, and distribution of the test and control articles will be maintained in the study file.

10.1 TEST ARTICLE

Name:	[***ARM-101 (***]3scAAV9.U6miR871 vector, AAV CMT1A[***)***]3
Batch/Lot Number:	[~~To be documented in the study file~~ ***TT970-1***]3
Storage Conditions:	-85 to -60°C

A Certificate of Analysis (CoA) and/or equivalent documentation of test article or component's identity, strength, purity, composition and other defining characteristics will be provided. Documentation of synthesis will be maintained by the manufacturer.

10.2 CONTROL ARTICLE/VEHICLE

[***Control Article***]3 Name:	Formulation Buffer (TMN200P)
Batch/Lot Number:	To be documented in the study file
Storage Conditions:	Stored according to the manufacture instructions

A Certificate of Analysis (CoA) and/or equivalent documentation of [~~test~~ ***control***]3 article [***or component's***]6 identity, strength, purity, composition and other defining characteristics will be provided. Documentation of synthesis will be maintained by the manufacturer.

[***Vehicle Name:***	***0.9% Saline***
Batch/Lot Number:	***To be documented in the study file***
Storage Conditions:	***Stored according to the manufacture instructions***

The vehicle is a marketed product and will be characterized by its labeling according to 21 CFR 58.105. A Certificate of Analysis (CoA) and/or equivalent documentation for the identity, concentration/titer, purity, composition and other defining characteristics may also be included in study file and final report. Documentation of synthesis will be maintained by the manufacturer.]3

10.3 RESERVE SAMPLES

A sample (approximate 3 mL) from each lot of bulk test article and control article used during the study will be retained as a reserve sample and stored under the appropriate storage conditions.

AmplifyBio Study Number: A-579-01

10.4 Formulation Preparation, Handling, and Storage

Formulations of test article will be diluted at concentrations of [~~2.3×10^{13} and 4.5×10^{13}~~ ***6×10^{13} and 1.2×10^{14}***][2] vg[~~/kg~~][6] [***with vehicle***][3] [~~at intervals not to exceed established stability~~][6]. When not in use, the formulations will be stored on wet ice until dosing is complete. Residual formulations will be stored in a freezer set to maintain temperature at -60 to -85°C until appropriately disposed of on or after the expiration date or shipped to the Sponsor. Details regarding formulation preparation will be maintained in the study file and included in the final report.

10.5 Formulation Analysis

Formulation analysis will not be performed.

10.6 Disposition of Test and Control Articles

Any unused bulk test and control article may be retained for use on future studies, disposed of, or shipped to a Sponsor-designated facility. Disposition of control article components (from control articles prepared per study) will be documented in the study file.

11.0 EXPERIMENTAL DESIGN

Animals will be assigned to one of three dose groups as presented in the following table.

Table 1. Experimental Design

Group	Target Dose ([~~vg/kg~~ ***Total vg***][2])	Target [~~Formulation Concentration~~ ***Dose Volume***][2] ([~~vg/mL~~ ***mL***][2]) [[a]][2]	Number of Animals			
			6-Week Cohort[b]		12-Week Cohort[c]	
			Male	Female	Male	Female
1 – [~~Vehicle~~ ***Control***][6]	0	[~~0~~ *3*][2]	1	1	1	1
2 – Low	Low ([~~2.3 × 10^{13}~~ ***6 × 10^{13}***][2])	[~~TBD~~ *3*][2]	2	2	2	2
3 – High	High ([~~4.5 × 10^{13}~~ ***1.2 × 10^{14}***][2])	[~~TBD~~ *3*][2]	2	2	2	2

[~~a. The target dose volume is [10 mL/kg~~ ***~~up to 3 mL per animal~~***~~][1]. TBD = To be determined [~~***~~and documented in the study file~~***~~][1]~~][2]
b. Necropsy Day 43.
c. Necropsy Day 85.

11.1 Group Assignment and Identification

Prior to group assignment, animals will be identified by the tattoo present at receipt. Animals will be assigned to dose groups using a stratified randomization program (Provantis) designed to maintain similar group mean body weights by sex.

After assignment to dose groups, each animal will receive an animal identification number unique within the study and will be identified with a cage card. Each cage card will contain information including, but not limited to, study number, group assignment, and animal identification number. The tattoo will be used to match the study animal number on the cage cards.

Table 2. Animal Identification

**Group**	**Target Dose ([~~vg/kg~~ *Total vg*][2])**	**Cage Card Color Scheme**	**Animal Identification Numbers**			
			**6-Week Cohort / Day 43**		**12-Week Cohort / Day 85**	
			**Male**	**Female**	**Male**	**Female**
1	0	White	101	151	102	152
2	Low ([~~2.3×10^{13}~~ ***6×10^{13}***][2])	Blue	201-202	251-252	203-204	253-254
3	High ([~~4.5×10^{13}~~ ***1.2×10^{14}***][2])	Green	301-302	351-352	303-304	353-354

11.2 DOSE ADMINISTRATION

On Day 1, the appropriate test [***(3 mL of Target Dose)***][2] or control article [***(3 mL Formulation buffer)***][2] will be administered to each animal via intrathecal slow infusion over 6 hours via [~~pre-study~~][6] implanted catheter. [~~Dose volumes will be based on each animal's most recent individual body weight.~~][2]

11.3 CLINICAL OBSERVATIONS

Observations for moribundity and mortality will be performed twice daily following transfer to study and during the study period, except on the day of necropsy when animals may only be observed once.

Cage-side clinical observations will be performed on all animals assigned to study at least once daily for the duration of the study, starting on Day 1. On days of dose administration, cage-side clinical observations will be recorded at a target time of 1 to 2 hours post-dose administration. An observation will be recorded on the day of scheduled necropsy.

Detailed clinical observations will be performed on all animals assigned to study at least once during the pretest period, prior to dosing on the day of dose administration, and at least once weekly thereafter.

When detailed observations and cage-side observations coincide, only the detailed observation will be performed.

Observations may be recorded more often as clinical signs warrant.

11.4 NON-INVASIVE NEUROLOGICAL OBSERVATIONS

Non-invasive functional neurological observations (cage-side) will be performed on all animals once during the pretest period and on all animals assigned to study once weekly for the duration of the study, starting on Day 1. Details of the methodology will be documented in the study file. Pupillary assessments will not be evaluated on the day of ophthalmic examinations. Evaluations will be conducted by staff trained in performing neurological observations in monkeys.

Results of the neurological observations will be provided to the Study Director for inclusion in the final report.

11.5 NERVE CONDUCTION VELOCITY TESTING

Once during the pretest period and once during the week of scheduled necropsy, while sedated for blood collection or [~~ICV~~][6] dose administration, each animal will undergo nerve conduction

AmplifyBio Study Number: A-579-01

velocity testing [***according to the study specific method***][1]. [***Briefly,***][1]Electrodes will be placed over the endplate zone of a muscle innervated by

AmplifyBio Study Number: A-579-01

the [~~Ulnar~~ ***Tibial***][1] Nerve. Compound Muscle Action Potential (CMAP) and Nerve Conduction Velocity will be recorded [***bilaterally and reported as separate values***][1].

Results of the nerve conductivity velocity testing will be provided to the Study Director for inclusion in the final report.

11.6 Body Weights

Individual body weights for all animals were recorded once prior to transfer from TOXBC HOLD protocol. Individual body weights for animals assigned to study will be recorded, prior to dosing on Day 1, and weekly thereafter. Terminal body weights (fasted) for calculation of organ-to-body weight ratios will be recorded on the day of scheduled necropsy.

Additional body weights may be recorded if clinical signs warrant.

11.7 Food Consumption

Food consumption will be visually assessed once daily starting Day -7 according to the following scale:

0 = no observable food consumed
1 = up to and including ¼ consumed
2 = greater than ¼ up to and including ½ consumed
3 = greater than ½ up to and including ¾ consumed
4 = greater than ¾ up to and including the entire amount consumed

Food consumption will not be assessed during times of scheduled fasting.

11.8 Ophthalmic Examinations

Ophthalmic examinations will be performed for all animals assigned to the study once during the pretest period, once during Week 6, and once for all surviving animals during Week 12 by a board-certified veterinary ophthalmologist. Animals will be sedated with ketamine (target 10-20 mg/kg, intramuscular [IM]) for examinations.

The pupils of each assigned animal will be dilated by instillation of tropicamide ophthalmic solution prior to examination. The anterior segment of the eye, posterior segment of the eye, and adnexal structures will be examined using a hand slit lamp and indirect ophthalmoscope. Each eye examined will be recorded as normal, abnormal, or not examined.

Results of the ophthalmic examination will be provided to the Study Director for inclusion in the final report.

11.9 ELECTROCARDIOGRAM (ECG) EVALUATIONS

ECG (non-telemetered) waveform tracing assessments will be performed on all animals assigned to study once during the pretest period, once during Week 6, and for all surviving animals once during Week 12. Animals will be sedated with ketamine (target 10-20 mg/kg, IM) for all ECG evaluations.

ECG waveform tracings using surface leads and a standard Lead II emulation will be collected for at least one minute and reviewed for morphologic evaluation. A board-certified [~~veterinary cardiologist~~ ***veterinarian***][1] will qualitatively evaluate the ECG tracings for evidence of cardiac dysfunction by examination of rate, rhythm, and morphology.

Results of the ECG evaluation will be provided to the Study Director for inclusion in the final report.

11.10 BIOSPECIMEN COLLECTION

Whole blood specimens will be collected according to the following schedule.

Table 3. Blood Collection Schedule

**Tube:**	**Na-Heparin**	**Serum Separator**	**K_3EDTA**	**Sodium Citrate**	[***Serum Separator***][1]
**Assay:**	**Blood for PBMC/ELISpot**	**Serum Chemistry**	**Hematology**	**Coagulation**	[***Troponin I***][1]
**Timepoint**	**Target Blood Volume (mL)**				
Pretest	6[d]	2.5	2	1.8[a]	[***1.0***][1]
Week 1	-	2.5	2	1.8[a]	[***1.0***][1]
Week 2	-	2.5	2	1.8[a]	[***1.0***][1]
Week 4	-	2.5	2	1.8[a]	[***1.0***][1]
[~~Week 6~~[e] ***Day 43***][1]	6[d]	2.5	2	1.8[a]	[***1.0***][1]
Week 8[b]	-	2.5	2	1.8[a]	[***1.0***][1]
[~~Week 12~~ ***Day 85***][1 b]	6[d]	2.5	2	1.8[a]	[***1.0***][1]

a. Volume may vary depending on the available tube size.
b. For 12-week cohort only
[~~c. For PBMC blood collection only~~][1]
d. [~~Two aliquots~~ ***One aliquot***][1] of approximately [~~3~~ ***6***][1] mL

All animals will be fasted overnight prior to scheduled blood collection for hematology and serum chemistry evaluations. Ketamine (target 5-20 mg/kg, intramuscular ([IM]) may be used for blood collections, if needed, and specimens will be collected from the femoral vessel or

other suitable peripheral vessel. Blood specimens not collected (although an attempt was made) will be documented in the study records.

Blood specimens for hematology will be collected in a tube containing K_3EDTA. Blood specimens for serum chemistry [***and Troponin I***][1] will be collected in a serum separator tube (SST) and allowed to clot at room temperature for at least 30 minutes before processing to serum. Separation of the blood into serum will be performed [***at room temperature***][1] using at least 10 minutes of centrifugation at a relative centrifugal force of at least 2,000 × g. [***Troponin serum samples will be stored on dry ice upon collection and transferred to a freezer set to maintain -85 to -60°C until analysis or shipped to the test site for Troponin I analysis.***][1] Blood specimen for coagulation will be collected in sodium citrate tubes and processed immediately upon receipt. The tubes collected for clinical pathology will be submitted to the testing facility's clinical pathology laboratory for analysis.

Blood specimens collected for PBMC/[~~ELISpot~~ ~~***Cytokine***~~][1] ***ELISpot***][6] analysis will be submitted to the testing facility's analytics laboratory for processing immediately upon receipt as described in Section [~~12.10.5~~ ~~***11***~~][1] ***11.10.5***][6].

Blood from moribund animals may be collected for clinical pathology evaluation, if needed, at the discretion of the Study Director or the Sponsor.

[~~Results of the clinical~~ ***Clinical***][1] pathology evaluation [~~will be~~ [~~***except***~~ ***including***][6] ***Troponin I will be performed at the testing facility and results will be included in a contributing scientist report***][1] provided to the Study Director for inclusion in the final report. [***A principal investigator report will be provided by the test site for Troponin I analysis*** [~~***to the Study Director for inclusion in the final report***~~][6]***. The test site will also provide evidence of a QA critical phase inspection and data/methods report audit.***][1] [***Results of the Troponin I analysis will be included in the clinical pathology contributing scientist report for inclusion in the final report.***][6]

11.10.1 Hematology

Hematology parameters to be evaluated are:

Table 4. Hematology Parameters

Cell morphology[a]	Mean corpuscular volume
Erythrocyte count	Mean corpuscular hemoglobin
Hemoglobin	Mean corpuscular hemoglobin concentration
Hematocrit	Mean platelet volume
Leukocyte count, total	Platelet count
Leukocyte differential (absolute and relative)	Reticulocyte count (absolute and relative)

a. Slides will be prepared from un-clotted samples for potential morphological assessment.

Hematology specimens (residual blood) will be disposed of following analysis.

AmplifyBio Study Number: A-579-01

11.10.2 Coagulation

Coagulation parameters to be evaluated are:

Table 5. Coagulation Parameters

Activated partial thromboplastin time<br>Fibrinogen	Prothrombin time

AmplifyBio Study Number: A-579-01

Coagulation specimens (residual plasma) will be discarded following analysis.

11.10.3 Serum Chemistry

Serum chemistry parameters to be evaluated will include but are not limited to:

Table 6. Serum Chemistry Parameters

Alanine aminotransferase	Creatine kinase
Albumin	Creatinine
Albumin/globulin ratio	Gamma glutamyltransferase
Alkaline phosphatase	Globulin
Aspartate aminotransferase	Glucose
Bilirubin, direct	Lactate dehydrogenase
Bilirubin, total	Phosphorus
Blood urea nitrogen	Potassium
Calcium	Sodium
Cholesterol	Total protein
Chloride	Triglycerides

Residual serum following analysis will be disposed of prior to clinical pathology report finalization.

11.10.4 Urinalysis

Urine will be collected overnight from all the animals prior to group assignment and during Weeks 1, 2, 4 and 6 from all animals assigned to the 6-week cohort and Weeks 1, 2, 4, 6, 8, and 12 from all animals assigned to the 12-week cohort. [~~Urine will be collected via free catch or from the cage pan~~ ***The total urine output collected overnight (via free catch or from the cage pan) will be provided to the clinical pathology laboratory***][1]. The actual time period of collection will be documented. The following parameters will be assessed:

Table 7. Urinalysis Parameters

Appearance (clarity and color)	pH[a]
Bilirubin[a]	Protein[a]
Glucose[a]	Specific gravity[b]
Ketones[a]	Total volume
Leukocytes[a]	Urobilinogen[a]
Nitrite[a]	Microscopic examination of sediment[c]
Occult blood[a]	

a. Qualitative results will be obtained using reagent test strips [(***urine dip parameters)***][1].
b. Results will be obtained using the SPER Scientific Digital Refractometer and/or the American Optical (AO) Total Solids Meter.
c. Sediment will be evaluated for white blood cells, red blood cells, casts, epithelial cells, mucus, sperm, bacteria, yeast, amorphous sediment, and crystals. Without machine/by hand.

Residual urine following analysis will be discarded.

11.10.5 [~~ELISpot Evaluation~~ [~~*Cytokine*~~ ***IFN-γ ELISpot***][6] ***Analysis***][1]

Blood (~ 6 mL) will be collected in Na-Heparin tubes from all animals assigned to the study once during the pretest period (all animals) and [~~during Week 6~~ ***on Day 43***][1] (all animals) and [~~Week 12~~ ***Day 85***][1] (for animals assigned to the 12-week cohort only). Blood will be processed for peripheral blood mononuclear cells (PBMC) isolation within approximately [4 ***6***][1] hours of collection and cryopreserved according to facility SOPs and study specific method. The PBMC will be aliquoted into at least two aliquots [~~with the first aliquot to~~ ***until the sample is exhausted depending upon the cell counts (target 10 × 10⁶ cells/mL) and cryopreserved according to the study specific method.***][1] [***2-3 aliquots may***][1] be used to analyze for cytokine concentration [~~using a qualified study specific ELISpot method.~~ ***using a qualified*** [[***IFN-γ***][6] ***ELISpot assay according to facility SOPs.***][5] The details of [~~cytokine~~ ***ELISpot***][6] analysis will be documented in the study file. [~~The second aliquot~~ ***1-2 aliquots***[***, if available,***][6]][1] will be [~~stored in a freezer set to maintain -85 to -60°C prior to shipment~~ ***shipped in*** [***an LN2***][6] ***shipper***][1] to Sponsor or [~~Sponsor-designated test site~~ ***Sponsor-designee***][6] for potential future analysis [***prior to report finalization*** ~~***and the final disposition will not be determined or reported under this study.***~~]~~[1]~~ ***and the results will not be reported under this study.***][6].

Results of the [~~ELISpot~~ ~~***cytokine***~~]~~[1]~~ ***IFN-γ ELISpot***][6] analysis [~~***by ELISpot***~~]~~[1]~~][6] will be provided to the Study Director in the form of a Contributing Scientist Report (CSR) for inclusion in the final report.

Any residual/retained [~~bioanalytical samples~~ ***PBMC aliquots***][1] will be discarded prior to report finalization with approval from the Sponsor and Study Director.

11.11 Cerebrospinal Fluid

Cerebrospinal fluid (CSF) will be collected from all surviving animals via passive collection from cisterna magna once during the pretest period, Week 2, Week 6, and Week 12.

The animals will be sedated with ketamine, or another anesthetic as determined by the veterinarian. The skin overlying the cisterna magna or lumbar cisterns will be clipped and aseptically prepped. A target of 1 mL of CSF will be collected from each animal, dispensed into one or more properly labeled vials, and stored in a freezer set to maintain a temperature of -85 to -60°C. The time of collection will be documented in the study file.

All CSF specimens will be shipped to the [~~Sponsor-designated test site~~ ***Sponsor or Sponsor-designee***][6] (on dry ice) for potential analysis prior to report finalization [~~and the final disposition will not be determined or reported under this study~~][6].

Results of the CSF analysis will not be provided to the Study Director.

11.12 NECROPSY

11.12.1 Unscheduled Necropsy

A gross necropsy with select tissue harvest will be performed on all study animals that die or are terminated at an unscheduled interval. Moribund animals will be humanely terminated by

AmplifyBio Study Number: A-579-01

administration of barbiturate overdose followed by exsanguination. Any animals found dead during study may be refrigerated until necropsy, if necessary. Tissues, if present, will be collected as listed for scheduled necropsies. Carcass and remaining tissues will be discarded after collection.

Organ weight data will not be collected at unscheduled necropsies.

11.12.2 Scheduled Necropsy

All animals that survive to the scheduled termination time point (at Day 43 for 6-week cohort or Day 85 for 12-week cohort) will be euthanized by an administration of a barbiturate overdose followed by exsanguination, and a gross necropsy with select tissue harvest will be performed. Carcass and remaining tissues will be discarded after collection.

All scheduled necropsies will be conducted with a board-certified veterinary pathologist available for consultation.

11.12.3 Tissue Collection

The following tissues, when present, will be collected from all animals undergoing necropsy. Tissues will be placed in 10% neutral buffered formalin (NBF), unless otherwise indicated.

Table 8. Tissue to be Collected

Tissue to be Collected	OW	HE	FF	Bank[b]	Tissue to be Collected	OW	HE	FF	Bank[b]
Animal identification[a]					Nerve, Median (distal)[g]			X	X[*l*][4]
Adrenal glands (paired)	X	X			Nerve, Median (proximal)[g]			X	X[*l*][4]
Aorta		X			Nerve, sciatic (distal)[g]		X	X	X[*l*][4]
Bone marrow (sternum)		X			Nerve, sciatic (proximal)[g]		X	X	X[*l*][4]
Bone marrow smear (rib)[c]					Nerve, Optic			X	X[*k*][4]
Brain[d]	X	X	X	X[*k*][4]	Nerve, Ulnar (distal)[g]			X	X[*l*][4]
[~~Biceps brachii, tibialis anterior (2x: L&R)~~][1]			[~~X~~][1]	[~~X~~][1]	Nerve, Ulnar (proximal)[g]			X	X[*l*][4]
Cecum		X			Ovaries (paired)	X	X	X	[~~X~~][4]
Cervix		X			Pancreas		X		
Colon		X			Parathyroid gland[h]		X		
DRG, cervical		X	X	X[*k*][4]	Pituitary gland	X	X		
DRG, thoracic		X	X	X[*k*][4]	Prostate		X		
DRG, lumbar		X	X	X[*k*][4]	Rectum		X		

Tissue to be Collected	OW	HE	FF	Bank[b]	Tissue to be Collected	OW	HE	FF	Bank[b]
Duodenum		X			Salivary glands (mandibular, [~~sublingual, parotid~~]~~[1]~~)		X		
Epididymides (paired)[e]	X	X			Seminal Vesicle		X		
Esophagus		X			Skeletal muscle (thigh)		X		
Eyes (paired)[f]		X			Skin		X		
Femur		X			Spinal cord (cervical)		X	X	X[*k*][4]
Gallbladder		X			Spinal cord (thoracic)		X	X	X[*k*][4]
Gross lesions (if applicable)		X	X		Spinal cord (lumbar)		X	X	X[*k,m*][4]
Heart	X	X	X	X[*k*][4]	Spinal cord (sacral)			X	X[*k*][4]
Ileum		X			Spinal roots (lumbar)			X	X[*k,m*][4]
Jejunum		X			Spleen	X	X	X	X[*k*][4]
Kidneys (paired)	X	X	X	X[*k*][4]	[***Stomach (cardiac, fundus, pyloris)***][5]		[*X*][5]		
Liver	X	X	X	X[*k*][4]	Testes (paired)[e]	X	X	X	
Lungs	X	X	X		Thymus	X	X		
Lymph node, mandibular		X			Thyroid glands (paired)[i]	X	X		
Lymph node, mesenteric		X			Trachea		X		
Mammary gland		X			Urinary bladder		X		
Nerve, Femoral (distal)[g]			X	X[*l*][4]	Uterus[j]	X	X		
Nerve, Femoral (proximal)[g]			X	X[*l*][4]	Vagina		X		
[***Skeletal Muscle (Biceps brachii, L)***][1]			[*X*][1]	[*X*][1][*k*][4]	[***Skeletal Muscle (Tibialis anterior, L)***][1]			[*X*][1]	[*X*][1][*k*][4]
[***Skeletal Muscle (Biceps brachii, R)***][1]			[*X*][1]	[*X*][1][*k*][4]	[***Skeletal Muscle (Tibialis anterior, R)***][1]			[*X*][1]	[*X*][1][*k*][4]

OW = Organ Weights; HE = Histopathologic Evaluation; FF = Flash Frozen; DRG = Dorsal Root Ganglion. Priority: HE > FF [***DNA > RNA >Additional Sample; AS***][1] > Bank

a. Collected but not processed.
b. Only if available.
c. Bone marrow cytology smears will be prepared for all animals at scheduled necropsy but will be evaluated at the discretion of the clinical pathologist only if warranted by changes in peripheral blood.
d. Corpus callosum collected for FF and Bank.
e. Preserved in Modified Davidson's fixative, then transferred to 10% NBF.
f. Preserved in Davidson's fixative, then transferred to 10% NBF.
g. From [~~3~~ *2*][4] different designations (if available) [***of each nerve, left and right.***][4]
h. To be examined only if in routine section.
i. Weighed with parathyroids embedded.
j. Weighed with cervix

AmplifyBio Study Number: A-579-01

[*k. Flash frozen using liquid nitrogen.*
l. One bank section from each designation will be collected. One will be saved in 4% glutaraldehyde in 0.1M Phosphate Buffer pH 7.4, the other flash frozen.
m. Collected attached to spinal cord to include a full transverse section of the spinal cord with spinal roots attached.][4]

11.12.4 Organ Weights

The organs indicated in Table 8, when present, will be weighed at all scheduled necropsies. Paired organs will be weighed together. Organ weight data will not be collected for animals found dead or terminated prior to scheduled necropsy. Absolute weight, organ-to-final body weight, and organ-to-brain weight will be reported.

11.12.5 Flash Frozen Samples

The [~~above~~][1] tissues [***indicated in Table 8***][1] designated for Flash Frozen will be collected [***under aseptic conditions***][1] for all animals undergoing necropsy. Following collection, tissue samples designated for FF will be sectioned into [~~one~~ ***up to three***][1] 3 mm^3 [~~sample~~ ***aliquots, if available,***][1] measured visually without the use of a measuring aid and placed into vials to be flash frozen using liquid nitrogen. Samples will be maintained on dry ice until transfer to a cold storage unit set to maintain -85 to -60°C until analyzed.

11.12.6 Bank Tissue Samples

[~~The above tissues designated for Bank will be collected for all animals undergoing necropsy if available. Following collection, tissue samples designated for Bank will be sectioned into one 3 mm³ sample measured visually without the use of a measuring aid and placed into vials to be flash frozen using liquid nitrogen. Samples will be maintained on dry ice until transfer to a cold storage unit set to maintain -85 to -60°C and shipped to the Sponsor or Sponsor-designee prior to report finalization. Final disposition and any analyses performed, or results obtained with these tissues will not be determined or reported under this study.~~

The above tissues designated for Bank will be collected for all animals undergoing necropsy if available. [~~***Final disposition and any analyses performed,***~~ ***Any analyses performed***][6] ***or results obtained with these tissues will not be determined or reported under this study.*** [***Bank tissues will be shipped to the Sponsor or Sponsor-designee for potential future analysis prior to report finalization.***][6]

11.12.6.1 Peripheral Nerves Bank Tissue Samples

Following collection, tissue samples designated for Bank from peripheral nerves will be collected from distal and proximal ends of the nerve.

One of the two designations of distal and proximal ends of the nerve will be placed in 4% glutaraldehyde in 0.1M Phosphate Buffer pH 7.4 in individually labeled cassettes and stored in a cold storage unit set to maintain 2°C to 8°C until shipped to the Sponsor or Sponsor-designee prior to report finalization.][4]

[***One of the two designations of distal and proximal ends of the nerve will be sectioned into one 3 mm^3 sample measured visually without the use of a measuring aid and placed into vials to be flash frozen using liquid nitrogen. Samples will be maintained on dry ice until transferred to a cold storage unit set to maintain -85 to -60°C and shipped to the Sponsor or Sponsor-designee prior to report finalization.***

11.12.6.2 Non-Peripheral Nerve Bank Tissue Samples

Remaining tissue samples designated for Bank will be sectioned into one 3 mm^3 sample measured visually without the use of a measuring aid and placed into vials to be flash frozen using liquid nitrogen. Samples will be maintained on dry ice until transferred to a cold storage unit set to maintain -85 to -60°C and shipped to the Sponsor or Sponsor-designee prior to report finalization.][4]

11.12.7 Tissue Processing

All fixed tissues from all animals[***, if present,***][1] will be processed to slides and stained with hematoxylin and eosin for histopathologic examination.

11.12.8 Histopathologic Evaluation

The above tissues and slides will be examined microscopically by a board-certified veterinary pathologist. Results of the examination will be provided to the Study Director for inclusion in the final report. An internal peer review will not be performed.

11.13 Biodistribution Analysis By Digital PCR (dPCR)

Flash frozen (FF) tissues [***aliquot 1 and 2***][1] collected as indicated in Table 8, from both the Day 43 and Day 85 necropsy will be processed to DNA and RNA[, ***respectively***][1]. [***Flash frozen aliquot 3 or additional sample (AS) may be used for either DNA or RNA if needed with Sponsor approval at the direction of the Study Director***[~~.~~]~~[1]~~ ***and the remaining samples will be shipped to the Sponsor for protein analysis.***][4] DNA will be analyzed for [~~AAV2~~ ***AAV2-ITR***][6] vector genome [***and house-keeping gene Beta-2-microglobulin (B2M)***][6] quantification by dPCR according to the study specific qualified method. RNA will be analyzed for miR871 [***, PMP22, and B2M gene***][6] expression [***by RT-dPCR***][6] according to the study specific [~~qualified~~][6] method. Details of the study specific [~~qualified~~][6] methods will be included in the study file. [[~~***Final disposition and any***~~ ***Any***][6] ***analyses performed on AS tissues, or results obtained with these tissues will not be determined or reported under this study.***][4]

AmplifyBio Study Number: A-579-01

Results of the dPCR and RT-dPCR will be provided to the Study Director for inclusion in the final report.

Any residual/retained [~~bioanalytical~~ ***biodistribution***][6] samples will be shipped to the Sponsor or [~~Sponsor-designated test site~~ ***Sponsor-designee***][6] for potential analysis prior to report finalization [~~and the final disposition will not be determined or~~ ***and the results will not be***][6] reported under this study.

12.0 COMPUTER SYSTEMS FOR DATA MANAGEMENT

The following computer systems are used for toxicology data management:

Table 9. Computer Systems

Computer System	Manufacturer	Data Type
BMRAM	Blue Mountain Quality Resources	Light Monitoring
Elemental Machines	Elemental Machines	Environmental Storage and Animal Facility Environment
EMCS	Siemens	Animal Facility Environment
Nicolet Viking EDX	Natus	Nerve Conduction Velocity Testing
Provantis	Instem	Test Article Inventory, Animal Toxicology, Clinical Pathology, and Post-Mortem Data Management
EMKA IOX	Emka Technologies	ECG Data Collection
EMKA ecgAUTO	Emka Technologies	ECG Waveform Analysis
ImmunoSpot	[~~ImmunoSpot~~ ***CTL***][6]	ELISpot Data [***Analysis***][6]
QIAcube, QIAxpert	QIAGEN	DNA[***/RNA***][1] Isolation
QIAcuity	QIAGEN	dPCR Analysis

Systems used at test sites will be included in the final report.

13.0 STATISTICAL ANALYSIS

13.1 PROVANTIS-COLLECTED DATA

All appropriate quantitative in-life data collected at AmplifyBio using the Provantis system will be analyzed for test article effects by parametric or nonparametric analysis of variance (ANOVA). For all data, normality will be determined by the Shapiro-Wilks test and homogeneity of variances will be determined by Levene's test. Data may be log-transformed to meet parametric assumptions. For parametric data determined to be normally distributed and homogeneous among groups, an ANOVA F-test will be used to determine whether there are differences among the group means. If the ANOVA F-test is significant, then tests for differences between the control and each of the comparison groups will be conducted using

Dunnett's test, which adjusts for multiple comparisons. For nonparametric data that are not normally-distributed and/or nonhomogeneous, a Kruskal-Wallis test will be used to determine whether there are differences among the group means. If the Kruskal-Wallis test is significant, then tests for differences between the control and each of the comparison groups will be conducted using Wilcoxon tests and the Bonferroni-Holm method to correct for multiple comparisons. All statistical tests will be performed at the 0.05 level of significance ($p < 0.05$), after accounting for multiple comparisons where indicated.

13.2 DATA REPORTED BY DESCRIPTIVE SUMMARIES OR INCIDENCE

[~~ECG, bioanalytical sample analysis, and toxicokinetic~~ ***ECG evaluations, non-invasive neurological observations, nerve conduction velocity, ophthalmic evaluations, troponin I analysis, and ELISpot analysis***][6] reports will include descriptive statistical analyses as appropriate.

Survival, clinical observations, and gross lesions will be reported as incidences.

Histopathological findings and severity will be reported as incidence and mean severity.

[***13.3 BIODISTRIBUTION STATISTICAL ANALYSIS***

To determine the quantification of the target genome and the target housekeeping gene [***(Mean and %CV)***][7] ***at the sacrificial time-points (Day 43 or Day 85), an internally-validated dPCR data analysis pipeline system*** [***will be used.*** ~~***statistical comparisons will be used for statistical comparisons to generate estimates of, and evaluate differences in, biodistribution of a target genome/gene between tissues and/or the accumulation within a medium and evaluate differences in biodistribution/accumulation of a target genome/gene between groups.***~~

~~***Tissue biodistribution statistical modeling, and figures produced, will be conducted on a log-transformed scale. In addition, estimated marginal means (EMMS) will also be conducted on a non-log transformed scale. Non-detectable values will be imputed using the minimum value observed divided by two. This is required for the log-transformation and given that it is unknown whether non-detectable values are below the limit of detection or truly not present. A linear mixed effects model with a random intercept for each animal (accounting for the correlation in measurements within each animal) will be estimated using Restricted Maximum Likelihood. Satterthwaite method will be utilized to determine the denominator degrees of freedom. Estimated marginal means (EMMs) will be produced to estimate the average amount of test article along with a 95% confidence interval for sex, dose, and each tissue. An analysis of variance (ANOVA) will be used to determine if there are main effects for sex, dose, tissue, and between dose and tissues. For dose (each sex and each tissue averaged together per dose group), tissue (each sex and each dose group averaged together per tissue) and dose within tissue comparisons, the p-values will be adjusted using Tukey's test, which***~~][6]][7]

[~~***adjusts for multiple comparisons.***~~

~~***All statistical tests will be performed at either a 0.0001, 0.001, 0.01, or 0.05, as applicable, level of significance.***~~][6]

A suitable transformation data method will be determined by examining the distribution of the data using histograms, normal P-P plots, and Shapiro-Wilk tests across gender, group, and time point. Based on the results of these descriptive statistics, log-transformation or square root transformation will be applied.

Results of the biodistribution statistical analysis will be provided to the Study Director in the form of a PIR for inclusion in the final report.][7]

14.0 REPORTING

A draft report will be prepared and submitted to the Sponsor. The Sponsor shall submit final comments on the draft report, if any. A final report will be submitted to the Sponsor following receipt of the Sponsor's final comments.

AmplifyBio Study Number: A-579-01

15.0 STORAGE OF STUDY MATERIALS AND RECORDS RETENTION

The disposition of study materials (data, documentation, samples, and/or specimens) is outlined in the following table.

Table 10. Disposition of Study Materials

Study Material	Location or Disposition
Raw data (paper data and electronic data)	AmplifyBio Archives
Bulk, unused test article	Disposition details will be included in the study records and described in the final report.
Remaining (unused) control article, and formulated test article	
Residual samples	Disposition determined prior to finalization
Final report	AmplifyBio Archives
Reserve samples (Test and Control Article)	AmplifyBio Archives
Wet tissues	AmplifyBio Archives
Embedded tissues (tissue blocks)	AmplifyBio Archives
Clinical pathology slides	AmplifyBio Archives
Serum from clinical pathology evaluation	Disposition determined prior to finalization
Histopathology slides	AmplifyBio Archives
Carcasses	Disposed following examination
Flash Frozen Tissues	Disposition determined prior to finalization
Bank Tissues [***for potential future analysis***][6]	Shipped to the Sponsor before finalization
[***PBMC***][5] [***for potential future analysis***][6]	[***Shipped to the Sponsor*** [***or Sponsor-designee***][6] ***before finalization***][5]
[***Residual DNA/RNA samples***][5]	[***Shipped to the Sponsor*** [***or Sponsor-designee***][6] ***before finalization***][5]
[***Cerebrospinal fluid for potential future analysis***][6]	[***Shipped to the Sponsor or Sponsor-designee before finalization***][6]
Raw data from Test Site(s)	Disposition details will be included in the study records and described in the final report.

16.0 REFERENCES

National Research Council (NRC). 2011. *The Guide for the Care and Use of Laboratory Animals*. 8th Ed. National Academies Press, Washington D.C.

U.S. Food and Drug Administration. 1987, *Guidance for Industry*. 21 CFR Part 58 Good Laboratory Practice Regulations, Final Rule.

Attachment 1. Protocol Amendments

List of Changes in Amended Protocol 1

The following items have been changed/revised from the original protocol signed on May 15, 2023:

1. a. Pages 4 and 5, Section **3.0 REGULATORY COMPLIANCE**. This section was updated throughout.

   b. The reasons for these changes were to add PBMC aliquot 2 or Bank tissues to the list of GLP exceptions and provide clarification for dose formulation analysis.

2. a. Page 5, Section 4.2. Immediately after this section, the following unnumbered section has been added:

   "**TEST SITE AND PRINCIPAL INVESTIGATOR**

   ANTECH DIAGNOSTICS GLP

Test Site for:	Troponin Analysis
Address:	600 Airport Blvd. Suite 500 Morrisville, NC 27560
Principal Investigator:	Katelin Leonesio Telephone: (919) 277-0822 Email: Katelin.Leonesio@antechmail.com"

   b. The reason for this change was to include Troponin Analysis on the study.

3. a. Page 6, Section **6.0 PROPOSED STUDY SCHEDULE**. The Lumbar IT catheter implantation surgery dates and number of animals was changed from:

   "May 17, 2023 – May 19, 2023 (10 NHPs)
   May 22, 2023 – May 24, 2023 (10 NHPs)"

   to:

   "May 17, 2023 – May 19, 2023 (11 NHPs)
   May 22, 2023 – May 23, 2023 (9 NHPs)"

   b. The reason for this change was to correct the surgery dates and number of animals.

4. a. Page 6, Section **8.0 TEST SYSTEM**. The source of the animals was changed from:

   "Testing facility holding pool origin: Primate Products, Inc., Immokalee, FL"

to:

"Testing facility holding pool origin: Envigo, Alice, TX"

b. The Weight of Animals on Day 1 has been changed from:

"1.5 to 5 kg"

to:

"2 to 6 kg"

c. The reason for this change was to change the source of the NHPs and to update their body weights on Day 1.

5. a. Page 7, Section **9.1 QUARANTINE AND ACCLIMATION**. The second sentence of the first paragraph in this section was removed.

b. The reason for this change was to remove the requirement for additional AAV9 testing during the quarantine period, as these animals were previously quarantined as part of the testing facility's holding pool.

6. a. Page 10, Section **11.0 EXPERIMENTAL DESIGN**. Footnote 'a' of Table 1. Experimental Design was changed from:

"a. The target dose volume is 10 mL/kg. TBD = To be determined"

to:

"a. The target dose volume is up to 3 mL per animal. TBD = To be determined and documented in the study file"

b. The reason for this change was to provide clarification of the target dose volume and documentation.

7. a. Pages 11 and 12, Section **11.5 NERVE CONDUCTION VELOCITY TESTING**. The first paragraph in this section was changed from:

"Once during the pretest period and once during the week of scheduled necropsy, while sedated for blood collection or ICV dose administration, each animal will undergo nerve conduction velocity testing. Electrodes will be placed over the endplate zone of a muscle innervated by the Ulnar Nerve. Compound Muscle Action Potential (CMAP) and Nerve Conduction Velocity will be recorded."

to:

"Once during the pretest period and once during the week of scheduled necropsy, while sedated for blood collection or ICV dose administration, each animal will undergo nerve conduction velocity testing. Electrodes will be placed over the

endplate zone of a muscle innervated by the Tibial Nerve. Compound Muscle Action Potential (CMAP) and Nerve Conduction Velocity will be recorded bilaterally and reported as separate values."

b. The reason for this change was to add the reference to the study specific method and change in the nerve evaluated.

8. a. Page 13, Section **11.9 ELECTROCARDIOGRAM (ECG) EVALUATION**. The second sentence of the second paragraph in this section was changed from:

"A board-certified veterinary cardiologist will qualitatively evaluate the ECG tracings for evidence of cardiac dysfunction by examination of rate, rhythm, and morphology."

to:

"A board-certified veterinarian will qualitatively evaluate the ECG tracings for evidence of cardiac dysfunction by examination of rate, rhythm, and morphology."

b. The reason for this change was to update the personnel for ECG evaluations.

9. a. Pages 13-14, Section **11.10 BIOSPECIMEN COLLECTION**. Updates have been made to Table 3. Blood Collection Schedule in this section.

b. In the third paragraph in this section, the second and third sentences have been changed from:

"Blood specimens for serum chemistry will be collected in a serum separator tube (SST) and allowed to clot at room temperature for at least 30 minutes before processing to serum. Separation of the blood into serum will be performed using at least 10 minutes of centrifugation at a relative centrifugal force of at least 2,000 × g. "

to:

"Blood specimens for serum chemistry and Troponin I will be collected in a serum separator tube (SST) and allowed to clot at room temperature for at least 30 minutes before processing to serum. Separation of the blood into serum will be performed at room temperature using at least 10 minutes of centrifugation at a relative centrifugal force of at least 2,000 × g."

c. In the third paragraph in this section, the following sentence was added immediately after the third sentence:

AmplifyBio Study Number: A-579-01

"Troponin serum samples will be stored on dry ice upon collection and transferred to a freezer set to maintain -85 to -60°C until analysis or shipped to the test site for Troponin I analysis."

d. The fourth paragraph in this section was changed from:

"Blood specimens collected for PBMC/ELISpot analysis will be submitted to the testing facility's analytics laboratory for processing immediately upon receipt as described in Section 12.10.5."

to:

"Blood specimens collected for PBMC/Cytokine analysis will be submitted to the testing facility's analytics laboratory for processing immediately upon receipt as described in Section 11."

e. The final paragraph in this section was changed from:

"Results of the clinical pathology evaluation will be provided to the Study Director for inclusion in the final report."

to:

"Clinical pathology evaluation except Troponin I will be performed at the testing facility and results will be included in a contributing scientist report provided to the Study Director for inclusion in the final report. A principal investigator report will be provided by the test site for Troponin I analysis to the Study Director for inclusion in the final report. The test site will also provide evidence of a QA critical phase inspection and data/methods report audit."

f. The reasons for these changes were to specify the day of clinical pathology collection for Week 6 (Day 43) and Week 12 (Day 85), to include Troponin I in the blood collection schedule, to update the number of aliquots collected and the collected volume, and to correct a typographical error.

10. a. Page 15, Section **11.10.4 Urinalysis**. The second sentence in the first paragraph of this section has been changed from:

"Urine will be collected via free catch or from the cage pan."

to:

"The total urine output collected overnight (via free catch or from the cage pan) will be provided to the clinical pathology laboratory."

b. The following was added to the end of footnote "a" in Table 7: "urine dip parameters".

c. The reason for these changes was to clarify the urine collection process and method of evaluation.

11. a. Page 16, Section **11.10.5 Cytokine Analysis**. The title of this section was changed and the section was updated throughout.

    b. The reason for this change was to correct the analysis method and add details of PBMC isolation and aliquoting.

12. a. Pages 17 and 18, Section **11.12.3 Tissue Collection**. Updates were made to Table 8. Tissue to be Collected.

    b. The reason for these changes was to clarify skeletal muscle collections and collection priority.

13. a. Page 19, Section **11.12.5 Flash Frozen Samples**. This section has been changed from:

    "The above tissues designated for Flash Frozen will be collected for all animals undergoing necropsy. Following collection, tissue samples designated for FF will be sectioned into one 3 mm^3 sample measured visually without the use of a measuring aid and placed into vials to be flash frozen using liquid nitrogen. Samples will be maintained on dry ice until transfer to a cold storage unit set to maintain -85 to -60°C until analyzed."

    to:

    "The tissues indicated in Table 8 designated for Flash Frozen will be collected under aseptic conditions for all animals undergoing necropsy. Following collection, tissue samples designated for FF will be sectioned into up to three 3 mm^3 aliquots, if available, measured visually without the use of a measuring aid and placed into vials to be flash frozen using liquid nitrogen. Samples will be maintained on dry ice until transfer to a cold storage unit set to maintain -85 to -60°C until analyzed."

    b. The reason for this change was to update the collection process for flash frozen samples.

14. a. Page 19, Section **11.13 BIODISTRIBUTION ANALYSIS BY DIGITAL PCR (DPCR)**. The first sentence in this section have been changed from:

    "Flash frozen (FF) tissues collected as indicated in Table 8, from both the Day 43 and Day 85 necropsy will be processed to DNA and RNA."

    to:

"Flash frozen (FF) tissues aliquot 1 and 2 collected as indicated in Table 8, from both the Day 43 and Day 85 necropsy will be processed to DNA and RNA, respectively."

b. The following was added after the first sentence in this section:

"Flash frozen aliquot 3 or additional sample (AS) may be used for either DNA or RNA if needed with Sponsor approval at the direction of the Study Director."

c. The reasons for these changes were to update the number of FF aliquots to account for DNA and RNA evaluations.

15. a. Page 20, Section **12.0 COMPUTER SYSTEMS FOR DATA MANAGEMENT**. The QIAcube, QIAxpert Date Type in Table 9. Computer Systems was changed from:

"DNA Isolation"

to:

"DNA/RNA Isolation"

b. The reason for these changes was to incorporate RNA Isolation.

In addition to the changes listed above (referenced through the protocol by "Amended"), the Title Page has been updated to reflect this amendment.

AMENDED PROTOCOL 1 SIGNATURES

APPROVED, TESTING FACILITY:

DocuSigned by:

Sapna Varia

Signer Name: Sapna Varia
Signing Reason: I approve this document
Signing Time: 6/13/2023 | 5:52:39 PM EDT
5D64C6F0F9AF4A7B9EBF31D648E66020

6/13/2023

Sapna Varia, PhD
Study Director

Date

APPROVED, SPONSOR:

DocuSigned by:

Brian Price

Signer Name: Brian Price
Signing Reason: I have reviewed this document
Signing Time: 6/13/2023 | 2:47:34 PM PDT
1974D3F004604A9E9D9FAF8A98283D0A

6/13/2023

Brian Price, PhD
Sponsors Representative

Date

List of Changes in Amended Protocol 2

The following items have been changed/revised from Amended Protocol 1 signed on June 13, 2023:

1. a. Page 9, Section **10.4 Formulation Preparation, Handling, and Storage**. The first sentence in this section has been changed from:

   "Formulations of test article will be diluted at concentrations of 2.3×10^{13} and 4.5×10^{13} vg/kg at intervals not to exceed established stability."

   to:

   "Formulations of test article will be diluted at concentrations of 6×10^{13} and 1.2×10^{14} vg/kg at intervals not to exceed established stability."

   b. The reason for this change was to provide accurate dosing information at Sponsor's request.

2. a. Page 10, Section **11.0 EXPERIMENTAL DESIGN**. Table 1. Experimental Design has been updated.

   b. The reason for this change was to provide accurate dosing information at Sponsor's request.

3. a. Page 10, Section **11.1 GROUP ASSIGNMENT AND IDENTIFICATION**. Table 2. Animal Identification has been updated.

   b. The reason for this change was to provide accurate dosing information at Sponsor's request.

4. a. Page 11, Section **11.2 DOSE ADMINISTRATION**. This section has been changed from:

   "On Day 1, the appropriate test or control article will be administered to each animal via intrathecal slow infusion over 6 hours via pre-study implanted catheter. Dose volumes will be based on each animal's most recent individual body weight."

   to:

   "On Day 1, the appropriate test (3 mL of Target Dose) or control article (3 mL Formulation buffer) will be administered to each animal via intrathecal slow infusion over 6 hours via pre-study implanted catheter."

   b. The reason for these changes was to clarify dose volumes.

In addition to the changes listed above (referenced through the protocol by "Amended"), the Title Page has been updated to reflect this amendment.

AMENDED PROTOCOL 2 SIGNATURES

APPROVED, TESTING FACILITY:

DocuSigned by:
Sapna Varia
Signer Name: Sapna Varia
Signing Reason: I approve this document
Signing Time: 6/16/2023 | 4:36:40 PM EDT
5D64C6F0F9AF4A7B9EBF31D648E66020

6/16/2023

Sapna Varia, PhD<br>Study Director	Date

APPROVED, SPONSOR:

DocuSigned by:
Brian Price
Signer Name: Brian Price
Signing Reason: I approve this document
Signing Time: 6/16/2023 | 1:12:30 PM PDT
1974D3F004604A9E9D9FAF8A98283D0A

6/16/2023

Brian Price, PhD<br>Sponsors Representative	Date

List of Changes in Amended Protocol 3

The following items have been changed/revised from Amended Protocol 2 signed on June 16, 2023:

1. a. Page 4, Section **1.0 PURPOSE**. This section has been changed from:

   "The purpose of this study is to evaluate the potential biodistribution and safety of scAAV9.U6miR871 when administered once via intrathecal infusion to Cynomolgus monkeys for 12 weeks."

   to:

   "The purpose of this study is to evaluate the potential biodistribution and safety of scAAV9.U6miR871 (ARM-101) when administered once via intrathecal infusion to Cynomolgus monkeys for 12 weeks."

   b. The reason for this change was to clarify the test article.

2. a. Page 4, Section **3.0 REGULATORY COMPLIANCE**. The fifth bullet in this section has been changed from:

   "In this study, the vehicle for test article formulation is used as a control article. The control article formulations will be analyzed only for concentration of test article and not for homogeneity. Control article formulations will not be subjected to stability testing."

   to:

   "The control article formulations will be analyzed only for concentration of test article and not for homogeneity. Control article formulations will not be subjected to stability testing."

   b. The reason for this change was to update the exception sentence as the vehicle is not the control article.

3. a. Page 9, Section **10.1 TEST ARTICLE**. The name and batch/lot number of the test article was changed from:

   "Name: scAAV9.U6miR871 vector, AAV CMT1A

   Batch/Lot Number: To be documented in the study file"

   to:

   "Name: ARM-101 (scAAV9.U6miR871 vector, AAV CMT1A)

   Batch/Lot Number: TT970-1"

AmplifyBio Study Number: A-579-01

b. The reasons for these changes were to clarify the test article name and include the batch/lot number.

4. a. Page 9, Section **10.2 CONTROL ARTICLE/VEHICLE**. This section was updated throughout

   b. The reason for this change was to include vehicle information and correct a typographical error.

5. a. Page 9, Section **10.4 FORMULATION PREPARATION, HANDLING, AND STORAGE**. The first sentence of this section was changed from:

   "Formulations of test article will be diluted at concentrations of 6×10^{13} and 1.2×10^{14} vg/kg at intervals not to exceed established stability."

   to:

   "Formulations of test article will be diluted at concentrations of 6×10^{13} and 1.2×10^{14} vg/kg with vehicle at intervals not to exceed established stability."

   b. The reason for this change was to clarify the formulation preparation method.

In addition to the changes listed above (referenced through the protocol by "Amended"), the Title Page, header information, and pagination has been updated to reflect this amendment.

AMENDED PROTOCOL 3 SIGNATURES

APPROVED, TESTING FACILITY:

DocuSigned by:
Sapna Varia
Signer Name: Sapna Varia
Signing Reason: I approve this document
Signing Time: 6/20/2023 | 9:07:34 PM EDT
5D64C6F0F9AF4A7B9EBF31D648E66020

6/20/2023

Sapna Varia, PhD
Study Director

Date

APPROVED, SPONSOR:

DocuSigned by:
Brian Price
Signer Name: Brian Price
Signing Reason: I approve this document
Signing Time: 6/20/2023 | 6:06:07 PM PDT
1974D3F004604A9E9D9FAF8A98283D0A

6/20/2023

Brian Price, PhD
Sponsors Representative

Date

AmplifyBio Study Number: A-579-01

List of Changes in Amended Protocol 4

The following items have been changed/revised from Amended Protocol 3 signed on June 20, 2023:

1. a. Pages 17 and 18, Section **11.12.3 Tissue Collection**. Table 8. Tissue to be Collected has been updated throughout.

   b. The reason for this change was to clarify the tissue collection procedure.

2. a. Page 19, Section **11.12.6 Bank Tissue Samples**. This section has been replaced in its entirety.

   b. The reasons for this change were to clarify collection, disposition, and reporting of the bank tissue samples.

3. a. Page 19, Section **11.13 BIODISTRIBUTION ANALYSIS BY DIGITAL PCR (DPCR).** The second sentence of the first paragraph in this section was changed from:

   "Flash frozen aliquot 3 or additional sample (AS) may be used for either DNA or RNA if needed with Sponsor approval at the direction of the Study Director."

   to:

   "Flash frozen aliquot 3 or additional sample (AS) may be used for either DNA or RNA if needed with Sponsor approval at the direction of the Study Director and the remaining samples will be shipped to the Sponsor for protein analysis."

   b. The following was added at the end of the first paragraph in this section:

   "Final disposition and any analyses performed on AS tissues, or results obtained with these tissues will not be determined or reported under this study."

   c. The reasons for these changes were to clarify the analysis, disposition, and reporting of the biodistribution samples.

In addition to the changes listed above (referenced through the protocol by "Amended"), the Title Page, header information, and pagination has been updated to reflect this amendment.

AMENDED PROTOCOL 4 SIGNATURES

APPROVED, TESTING FACILITY:

DocuSigned by:

Sapna Varia

Signer Name: Sapna Varia
Signing Reason: I approve this document
Signing Time: 7/31/2023 | 6:58:02 PM EDT
5D64C6F0F9AF4A7B9EBF31D648E66020

7/31/2023

Sapna Varia, PhD
Study Director

Date

APPROVED, SPONSOR:

DocuSigned by:

Brian Price

Signer Name: Brian Price
Signing Reason: I approve this document
Signing Time: 7/31/2023 | 3:31:02 PM PDT
1974D3F004604A9E9D9FAF8A98283D0A

7/31/2023

Brian Price, PhD
Sponsors Representative

Date

List of Changes in Amended Protocol 5

The following items have been changed/revised from Amended Protocol 4 signed on July 31, 2023:

1. a. Page 16, Section **11.10.5 Cytokine Analysis**. The fourth sentence in this section was changed from:

   "2-3 aliquots may be used to analyze for cytokine concentration using a qualified study specific ELISpot method."

   to:

   "2-3 aliquots may be used to analyze for cytokine concentration using a qualified ELISpot assay according to facility SOPs."

   b. The reason for this change was to correct the method of analysis.

2. a. Page 18, Section **11.12.3 Tissue Collection**. Table 8. Tissue to be Collected has been updated to include Stomach (cardiac, fundus, pyloris) with Histopathologic Evaluation.

   b. The reason for this change was to add an additional tissue to be collected.

3. a. Page 22, Section **15.0 STORAGE OF STUDY MATERIALS AND RECORDS RETENTION**. Table 10. Disposition of Study Materials has been updated to include PBMC and Residual DNA/RNA samples that are to be shipped to the Sponsor before finalization.

   b. The reason for this change was to include relevant study materials and their disposition.

In addition to the changes listed above (referenced through the protocol by "Amended"), the Title Page, header information, and pagination has been updated to reflect this amendment.

AMENDED PROTOCOL 5 SIGNATURES

APPROVED, TESTING FACILITY:

DocuSigned by:
Sapna Varia
Signer Name: Sapna Varia
Signing Reason: I approve this document
Signing Time: 9/8/2023 | 4:54:46 PM EDT
5D64C6F0F9AF4A7B9EBF31D648E66020

9/8/2023

Sapna Varia, PhD
Study Director

Date

APPROVED, SPONSOR:

DocuSigned by:
Brian Price
Signer Name: Brian Price
Signing Reason: I approve this document
Signing Time: 9/8/2023 | 11:44:58 AM PDT
1974D3F004604A9E9D9FAF8A98283D0A

9/8/2023

Brian Price, PhD
Sponsors Representative

Date

List of Changes in Amended Protocol 6

The following items have been changed/revised from Amended Protocol 5 signed on September 8, 2023:

1. a. Pages 4 and 5, Section **3.0 REGULATORY COMPLIANCE**. The third bullet in this section was changed from:

   "• There will not be any formulation dose analysis as the test article will be received as ready to dose or may be diluted to required concentration with the formulation buffer."

   to:

   "• There will not be any formulation dose analysis as the test article will be received as ready to dose or may be diluted to required concentration with the vehicle (0.9% saline)."

   b. The first sentence of the fifth bullet in this section was changed from:

   "The control article formulations will be analyzed only for concentration of test article and not for homogeneity."

   to:

   "The control article formulations will not be analyzed only for concentration of test article and not for homogeneity."

   c. The first, second, and third sentences of the sixth bullet in this section was changed from:

   "The final disposition and results from Cerebrospinal fluid (CSF) analysis or Bank tissues will not be reported under this study Cerebrospinal fluid, PBMC aliquot(s) shipped to Sponsor or Bank tissue samples for potential analysis will be shipped to the Sponsor on dry ice for potential future evaluation by the Sponsor. This additional work is not expected to have an impact on the conclusions of this study because the Cerebrospinal fluid, PBMC aliquot(s), shipped to Sponsor or Bank tissue analysis will not impact the assessment of the safety and biodistribution evaluation of the test article."

   to:

   "The results from Cerebrospinal fluid (CSF) analysis or Bank tissues will not be reported under this study. Cerebrospinal fluid, PBMC aliquot(s), residual/retained biodistribution samples or Bank tissue samples for potential analysis will be shipped to the Sponsor or Sponsor-designee on dry ice or LN2 shipper for potential

AmplifyBio Study Number: A-579-01

future evaluation by the Sponsor. This additional work is not expected to have an impact on the conclusions of this study because the Cerebrospinal fluid, PBMC aliquot(s), residual/retained biodistribution samples or Bank tissue (shipped to Sponsor or Sponsor-designee) analysis will not impact the assessment of the safety and biodistribution evaluation of the test article. ”

d. The following was added as a new bullet in this section:

"• The method used for biodistribution analysis by Digital PCR (RT-dPCR) will not be qualified."

e. The reason for this change was to add and update exceptions.

2. a. Page 9, Section **10.2 CONTROL ARTICLE/VEHICLE**. The first sentence in this section was changed from:

"A Certificate of Analysis (CoA) and/or equivalent documentation of control article identity, strength, purity, composition and other defining characteristics will be provided."

to:

"A Certificate of Analysis (CoA) and/or equivalent documentation of control article or component's identity, strength, purity, composition and other defining characteristics will be provided."

b. The reason for this change was to update the required documentation of the control article.

3. a. Page 9, Section **10.4 FORMULATION PREPARATION, HANDLING, AND STORAGE**. The first sentence in this section was changed from:

"Formulations of test article will be diluted at concentrations of 6×10^{13} and 1.2×10^{14} vg/kg with vehicle at intervals not to exceed established stability."

to:

"Formulations of test article will be diluted at concentrations of 6×10^{13} and 1.2×10^{14} vg with vehicle."

b. The reason for this change was to correct the dosage of the diluted test article.

4. a. Page 10, Section **11.0 EXPERIMENTAL DESIGN**. '1 – Vehicle' was changed to '1 – Control' in Table 1. Experimental Design.

b. The reason for this change was to update Group 1 designation.

AmplifyBio Study Number: A-579-01

5. a. Page 11, Section **11.2 Dose Administration**. The first sentence in this section was changed from:

   "On Day 1, the appropriate test (3 mL of Target Dose) or control article (3 mL Formulation buffer) will be administered to each animal via intrathecal slow infusion over 6 hours via pre-study implanted catheter."

   to:

   "On Day 1, the appropriate test (3 mL of Target Dose) or control article (3 mL Formulation buffer) will be administered to each animal via intrathecal slow infusion over 6 hours via implanted catheter."

   b. The reason for this change was to correct a clerical error.

6. a. Page 11, Section **11.5 Nerve Conduction Velocity Testing**. The first sentence in this section was changed from:

   "Once during the pretest period and once during the week of scheduled necropsy, while sedated for blood collection or ICV dose administration, each animal will undergo nerve conduction velocity testing according to the study specific method."

   to:

   "Once during the pretest period and once during the week of scheduled necropsy, while sedated for blood collection or dose administration, each animal will undergo nerve conduction velocity testing according to the study specific method."

   b. The reason for this change was to correct a clerical route of dose administration error.

7. a. Page 14, Section **11.10 Biospecimen Collection**. The fourth paragraph in this section was changed from:

   "Blood specimens collected for PBMC/Cytokine analysis will be submitted to the testing facility's analytics laboratory for processing immediately upon receipt as described in Section 11."

   to:

   "Blood specimens collected for PBMC/ELISpot analysis will be submitted to the testing facility's analytics laboratory for processing immediately upon receipt as described in Section 11.10.5."

AmplifyBio Study Number: A-579-01

b. The sixth paragraph in this section was changed from:

"Clinical pathology evaluation except Troponin I will be performed at the testing facility and results will be included in a contributing scientist report provided to the Study Director for inclusion in the final report. A principal investigator report will be provided by the test site for Troponin I analysis to the Study Director for inclusion in the final report. The test site will also provide evidence of a QA critical phase inspection and data/methods report audit."

to:

"Clinical pathology evaluation including Troponin I will be performed at the testing facility and results will be included in a contributing scientist report][1] provided to the Study Director for inclusion in the final report. [A principal investigator report will be provided by the test site for Troponin I analysis. The test site will also provide evidence of a QA critical phase inspection and data/methods report audit. Results of the Troponin I analysis will be included in the clinical pathology contributing scientist report for inclusion in the final report."

c. The reasons for these changes were to update the type of analysis, correct the section number, and to clarify Troponin I analysis reporting.

8. a. Page 16, Section **11.10.5 IFN-γ ELISpot Analysis**. This section was renamed and updated throughout.

b. The reasons for these changes were to update the type of analysis to be performed and disposition details.

9. a. Page 16, Section **11.11 CEREBROSPINAL FLUID**. The third paragraph in this section was changed from:

"All CSF specimens will be shipped to the Sponsor-designated test site (on dry ice) for potential analysis prior to report finalization and the final disposition will not be determined or reported under this study."

to:

"All CSF specimens will be shipped to the Sponsor or Sponsor-designee (on dry ice) for potential analysis prior to report finalization."

b. The reason for this change was to clarify disposition details.

10. a. Page 19, Section **11.12.6 Bank Tissue Samples**. This section was changed from:

"The above tissues designated for Bank will be collected for all animals undergoing necropsy if available. Final disposition and any analyses performed, or results obtained with these tissues will not be determined or reported under this study."

to:

"The above tissues designated for Bank will be collected for all animals undergoing necropsy if available. Any analyses performed or results obtained with these tissues will not be determined or reported under this study. Bank tissues will be shipped to the Sponsor or Sponsor-designee for potential future analysis prior to report finalization."

b. The reason for this change was to clarify disposition details.

11. a. Pages 19 and 20, Section **11.13 Biodistribution Analysis By Digital PCR (dPCR)**. This section was updated throughout.

b. The reason for this change was to update DNA and RNA analyses to be performed and disposition details.

12. a. Page 20, Section **12.0 COMPUTER SYSTEMS FOR DATA MANAGEMENT**. The ImmunoSpot information in Table 9. Computer Systems was updated.

b. The reason for this change was to update DNA and RNA analyses, disposition details, and to correct the manufacturer and data type for ImmunoSpot.

13. a. Page 21, Section **13.2 Data Required by Descriptive Summaries or Incidence**. The first paragraph in this section was changed from:

"ECG, bioanalytical sample analysis, and toxicokinetic reports will include descriptive statistical analyses as appropriate."

to:

"ECG evaluations, non-invasive neurological observations, nerve conduction velocity, ophthalmic evaluations, troponin I analysis, and ELISpot analysis reports will include descriptive statistical analyses as appropriate."

b. The reason for this change was to update the data to be reported by descriptive statistical analysis.

14. a. Page 21, Section **13.3 BIODISTRIBUTION STATISTICAL ANALYSIS**. This section was added.

    b. The reason for this change was to add biodistribution comparative statistical analysis.

15. a. Page 22, Section **15.0 STORAGE OF STUDY MATERIALS AND RECORDS RETENTION**. Table 10. Disposition of Study Materials has been updated throughout.

    b. The reason for this change was to add or clarify disposition of study materials.

In addition to the changes listed above (referenced through the protocol by "Amended"), the Title Page, header information, and pagination has been updated to reflect this amendment.

AmplifyBio Study Number: A-579-01

AMENDED PROTOCOL 6 SIGNATURES

APPROVED, TESTING FACILITY:

DocuSigned by:
Sapna Varia
Signer Name: Sapna Varia
Signing Reason: I approve this document
Signing Time: 1/16/2024 | 11:59:58 AM EST
5D64C6F0F9AF4A7B9EBF31D648E66020

1/16/2024

Sapna Varia, PhD
Study Director

Date

APPROVED, SPONSOR:

DocuSigned by:
Brian Price
Signer Name: Brian Price
Signing Reason: I approve this document
Signing Time: 1/16/2024 | 8:41:03 AM PST
1974D3F004604A9E9D9FAF8A98283D0A

1/16/2024

Brian Price, PhD
Sponsors Representative

Date

List of Changes in Amended Protocol 7

The following items have been changed/revised from Amended Protocol 6 signed on January 16, 2024:

1. a. Page 5, Section **3.0 REGULATORY COMPLIANCE**. The following was added as a new bullet in this section:

   "• Statistical analysis for dPCR and RT-dPCR will not be performed in accordance to GLP compliance."

   b. The reason for this change was to add this exception for statistical analysis.

2. a. Page 5, Section **TEST SITES AND PRINCIPAL INVESTIGATORS**. This section has been renamed and updated throughout.

   b. The reason for this change was to include principal investigator information for statistical analysis for dPCR and RT-dPCR.

3. a. Page 21, Section **13.3 BIODISTRIBUTION STATISTICAL ANALYSIS**. This section has been updated throughout.

   b. The reason for this change was to update methods and reporting for statistical analysis.

In addition to the changes listed above (referenced through the protocol by "Amended"), the Title Page, header information, and pagination has been updated to reflect this amendment.

AMENDED PROTOCOL 7 SIGNATURES

APPROVED, TESTING FACILITY:

DocuSigned by:
Sapna Varia
Signer Name: Sapna Varia
Signing Reason: I approve this document
Signing Time: 1/25/2024 | 6:37:36 PM EST
5D64C6F0F9AF4A7B9EBF31D648E66020

1/25/2024

Sapna Varia, PhD
Study Director

Date

APPROVED, SPONSOR:

DocuSigned by:
Brian Price
Signer Name: Brian Price
Signing Reason: I approve this document
Signing Time: 1/25/2024 | 3:35:58 PM PST
1974D3F004604A9E9D9FAF8A98283D0A

1/25/2024

Brian Price, PhD
Sponsors Representative

Date

SUMMARY OF DEVIATIONS

Deviations from GLP, the study protocol, or selected SOP and study specific methods are summarized below. All other SOP and study specific method deviations are maintained in the study file.

Deviation Number	Date Occurred	Description	Impact
DEV-002537	12/23/2023	21 CFR - Part 58.120 Protocol section (12) states that study protocols must include "A statement of the proposed statistical methods to be used."<br>On 23Dec2023, there were statistical methods performed using the dPCR data for study A-579-01, and these methods were not described in the protocol or other guiding document prior to use. The statistical analysis was performed as part of module 2 of the dPCR data pipeline. The statistical methods included ANOVA and estimated marginal means.	There is no impact to the study due to no guiding document for the statistical analysis effective prior to protocol amendment as the data will be re-analyzed and hence there is no impact to the integrity of the data.
QE-001496	6/13/2023 – 6/15/2023	Protocol section 11.7 (Food Consumption) states, "Food consumption will be visually assessed once daily starting Day -7..."<br>Food consumption was recorded beginning Day -9 for Animal 352 and Day -5 for Animal 152.	There was no study impact due to the food consumption being taken early (for the animal 352) or later (for the animal 152) than planned. All the animals remained healthy with increasing body weight measurements and there was no concern on their daily food intake. Thus, there was no impact to the integrity of the data.

Deviation Number	Date Occurred	Description	Impact
QE-001541	6/16/2023	Protocol Table 3 Blood Collection Schedule has a footnote on Blood for PBMC/ELISpot collection "One aliquot of approximately 6 mL"<br><br>For the pretest PBMC/ELISpot collection on 16Jun2023 for animal 201, the 6mL of blood was collected into 2 separate tubes and was transferred to the Analytics Laboratory. Analytics combined the blood into one tube for PBMC isolation and the total usable blood volume was 7.1mL.	There was no study impact due to blood collection into two Na-Heparin tubes instead of one resulting into additional 1.1 mL blood when pooled together as described in this deviation. PBMC isolation did not have any impact due to this extra blood. The animal was healthy and additional blood withdrawn was under IACUC approval as it was within allowable blood volumes. Thus, there was no impact to the integrity of the data and animal welfare.

Deviation Number	Date Occurred	Description	Impact
QE-001542	6/20/2023 – 6/22/2023	A-579-01 Protocol section 11.2 Dose Administration states "On Day 1, the appropriate test (3 mL of Target Dose) or control article (3 mL Formulation buffer) will be administered to each animal via intrathecal slow infusion over 6 hours via pre-study implanted catheter."<br><br>On Day 1, the dose administration was not exactly 6 hours.<br><br>For group 1 on 20Jun2023, animal 101 had dose administered starting at 0957 and the huber needle was detached several times during infusion. The final comment states the huber needle detached from port and the pump was paused at 1414 and there is no documentation of when the pump was restarted following this pause. It is unclear how long the dose was administered with the pump stop time documented at 1632. For animal 102, the dose was administered for 5hrs 58 mins and for animal 151, the dose was administered for 5hrs 56 mins. For animal 152, the dose was administered for 5hrs 3 mins.<br><br>For group 2 on 21Jun2023, for animal 201 the dose was administered for 5hrs 56 mins. For animal 202, the dose was administered for 5hrs 56 mins and for animal 203, the dose was administered for 5 hrs 57 mins. For animal 204, the dose was administered for 5hrs 55 mins and for animal 251, the dose was administered for 5hrs 56 mins. For animal 252, the dose was administered for 5hrs 54 mins and for animal 253, the dose was administered for 5hrs 56 mins. For animal 254, the dose was administered for 5hrs 55 mins.<br><br>For group 3 on 21Jun2023, for animal 301 the dose was administered for 5hrs 55 mins. For animal 302, the dose was administered for 5hrs 55 mins and for animal 303, the dose was administered for 5 hrs 51 mins. For animal 304, the dose was administered for 6hrs 2 mins and for animal 351, the dose was administered for 5 hrs 56 mins. For animal 352, the dose was administered for 5hrs 56 mins and for animal 353, the dose was administered for 5 hrs 57 mins. For animal 354, the dose was administered for 5hrs 58 mins.	There was no study impact due to the duration of dosing not being exactly 6 hours. The dose was administered to all animals in all groups approximately 6 bours (-10 minutes). The animals were having issues with the needle staying in place in the catheter during infusion and hence multiple attempts were made to secure the needle in place during dosing which caused the pump to be stopped intermittently and continued once the needle was in place. Hence, the extra ~10 minutes would account for those attempts. For Group 1 control animal 152, the dose was administered for 5hrs 3 mins, which was significantly less than the required dose duration. After multiple attempts to secure the needle in place, this animal showed signs of discomfort and hence the dosing was stopped for the welfare of the animal. The Sponsor was notified, and they approved the decision too.

Deviation Number	Date Occurred	Description	Impact
DEV-002735 (Addendum to QE-001593)	6/26/2023 – 6/28/2023	According to Protocol section 11.10 Biospecimen Collection, Table 3 Blood Collection Schedule, 1 mL of Blood was to be collected for Troponin evaluation during Week 1.<br>For Week 1 Troponin blood collections, 1.0 mL of blood was not collected from any study animal. As a result, the Week 1 residual frozen serum following serum chemistry analysis was thawed and one aliquot of ~0.5mL of serum per animal was harvested, subsequently frozen, and shipped to the test site for analysis.	There was no study impact due to the missing 1 mL of blood collection for Troponin evaluation as described in this deviation. To avoid collecting additional blood from all animals during week 1 and scheduled week 2 blood collections, Troponin was evaluated from the residual serum from week 1 serum chemistry evaluation for all animals. There was no impact to the integrity of the data
QE-001785	8/1/2023 – 8/3/2023	Protocol section 11.12.3 (Tissue Collection) lists tissues to be collected at necropsy.<br>The stomach was collected from all animals at necropsy on Day 43 for histopathologic evaluation, although not required by protocol.	There was no study impact due to the collection of stomach from all animals as it was part of the standard list for tissue collection. The protocol amendment was completed to add stomach to the list.
QE-001798	8/1/2023 – 8/3/2023	Protocol section 11.10.5 Cytokine Analysis states "The PBMC will be aliquoted into at least two aliquots until the sample is exhausted depending upon the cell counts (target 10 × 10^6 cells/mL) until the sample is exhausted depending upon the cell counts (target 10 × 10^6 cells/mL) and cryopreserved according to the study specific method."<br>For Day 43 PBMC processing there was only one PBMC aliquot frozen for several samples. On 01Aug2023, for animal 151 there was only one aliquot of PBMC sample frozen. Then on 02Aug2023, animals 201, 251 and 252 had only one aliquot of PBMC sample frozen. Then on 03Aug2023, animal 353 had only one aliquot of PBMC sample frozen.	The impact to study is minimal due to less than two aliquots of PBMC frozen as described in this deviation. The second PBMC aliquot was to be used as a bank aliquot for the Sponsor in case of need to repeat the analysis. The target concentration was more important than obtaining multiple vials. Total available cell count is subject to the animal's blood composition at the time of collection. Hence there was no impact to the data integrity.

Deviation Number	Date Occurred	Description	Impact
QE-002014	6/15/2023 – 8/11/2023	Protocol Section 11.8 "Ophthalmic Examinations" states "Each eye examined will be recorded as normal, abnormal, or not examined."<br><br>Ophthalmic examinations were recorded as "no abnormal findings" or " Cataract" on Days -7, -6, -5, 39, 40, 41, 80, 81, and 82 instead of "normal, abnormal, or not examined" as required by the protocol.	There was no study impact due to this documentation error as the animals were examined as intended.
QE-002111	6/8/2023 – 9/7/2023	Protocol Section 11.9 Electrocardiogram (ECG) Evaluations states ECG (non-telemetered) waveform tracing assessments will be performed on all animals assigned to study once during the pretest period, once during Week 6, and for all surviving animals once during Week 12. Animals will be sedated with ketamine (target 10-20 mg/kg, IM) for all ECG evaluations. ECG waveform tracings using surface leads and a standard Lead II emulation will be collected for at least one minute and reviewed for morphologic evaluation. A board-certified veterinary cardiologist veterinarian will qualitatively evaluate the ECG tracings for evidence of cardiac dysfunction by examination of rate, rhythm, and morphology.<br><br>In addition to ECG evaluations, blood pressure monitoring was also performed on all animals at each timepoint. (Pre-study- 20 animals, Week 6- 20 animals, Week- 12 10 animals) The protocol did not require blood pressure monitoring at any time point.	There was no impact to the study due to the additional blood pressure monitoring with ECG waveform analysis as outlined in this deviation. Blood pressure was performed as an additional support data to monitor the animals during ECG evaluations. The blood pressure results were not compiled or analyzed. There was no impact to the integrity of the data.
DEV-002185	8/1/2023 – 8/3/2023	Protocol section 11.10.4 states “Urinalysis Urine will be collected overnight from all the animals prior to group assignment and during Weeks 1, 2, 4 and 6 from all animals assigned to the 6-week and 12-week cohort.”<br><br>Data shows that urine was collected on week 7 (Day 43) instead of week 6.	There was no study impact due to the urine collection on Day 43 one day after week 6. The urine collection was performed as intended, hence there was no impact to the integrity of the data.

Deviation Number	Date Occurred	Description	Impact
DEV-002199	6/16/2023 – 6/22/2023	Protocol section 11.10.5 ELISpot Evaluation: Blood (~6 mL) will be collected in Na-Heparin tubes from all animals assigned to the study once during the pretest period (all animals) and during Week 6 (all animals) and Week 12 (for animals assigned to the 12-week cohort only).<br><br>On 16Jun2023, blood collected and processed to PBMC did not have enough cell count and only one aliquot of PBMC was collected for Animals 201, 204, 251, 353 and 354 thus, not meeting the protocol requirement of at least 2 aliquots of PBMCs per sample. To account for this, a secondary blood collection was scheduled for 21Jun2023 (Group 2) and 22Jun2023 (Group 3) with IACUC approval. This allowed for a second pre-study PBMC aliquot to be collected for these animals but was done in two separate blood collections, therefore not meeting the protocol requirement of blood being collected once for all animals at the pre-study timepoint.	There is minimal impact on the study based on events described in this deviation. In order to collect 2 aliquots of PBMCs per sample during the pre-study time point a second blood collection was scheduled before Day 1. Although the second blood draw was not laid out in the protocol the decision was made in order to meet the other protocol requirement of the second aliquot. Based on cell count and viability data, samples were processed appropriately, and samples can now be used in downstream processing.
DEV-002201	6/16/2023	Protocol section 11.10.5 states "Blood will be processed for peripheral blood mononuclear cells (PBMC) isolation within approximately 6 hours of collection and cryopreserved according to facility SOPs and study specific methods."<br><br>Data shows that 10 samples (Sample IDs 152, 204, 253, 254, 301, 302, 303, 304, 353, and 354) processed on 16JUN2023 were processed > 8 hours.	There is minimal impact to the study for these samples being processed outside of the 8-hour window. Based on cell count and viability data, processing time did not negatively affect samples.
DEV-003039 (Addendum to DEV-002204)	5/10/2023 – 5/11/2023	According to Protocol Section 11.10.1, Hematology Table 4, reticulocyte count (absolute and relative) and Mean platelet volume will be evaluated, and slides will be prepared from un-clotted samples for potential morphological assessment.<br><br>For the pretest hematology collection, relative reticulocyte count (% reticulocytes) and Mean Platelet Volume were not evaluated. In addition, slides were only prepared for Animals 152, 253, 352, and 353.	There is no impact to the study for the reticulocyte counte, mean platelet volume orslide preparation for the animals listed in the deviation was not needed as there were no abnormal findings to confirm manually.

Deviation Number	Date Occurred	Description	Impact
DEV-002205	5/10/2023	Per protocol, section 11.10.4 Urinalysis, "Residual urine following analysis will be discarded".<br>The 'Specimen Storage and Disposal' log did not include Animal 253. This section of the protocol also states that a 'Microscopic examination of sediment' will be performed, but there is no documentation for centrifugation or a urine sample for Animal 253 as required for this analysis.	There is no study impact due to this documentation error. There was no abnormality identified in the urine analysis which shows that the urine samples were properly processed and hence there was no impact to the data integrity.
DEV-002206	5/10/2023 – 9/14/2023	According to protocol section 11.10, "Blood specimens for serum chemistry will be collected in a serum separator tube (SST) and allowed to clot at room temperature for at least 30 minutes before processing to serum. Separation of the blood into serum will be performed at room temperature using at least 10 minutes of centrifugation at a relative centrifugal force of at least 2,000 × g."<br>Documentation of clot and separation on the centrifuge log does not indicate the temperature these tasks were performed for all serum chemistry collections.	There was no impact to the study as the separation of serum from blood samples was performed as intended. This was a documentation error and hence there was no impact to the integrity of the data

Deviation Number	Date Occurred	Description	Impact
DEV-002224	10/6/2023-10/10/2023	Batch A-579-01.006 tissues samples from animal 252 were being extracted for DNA on 10Oct2023 according to the DNA Extraction SSM (METHOD-00108) when the QIAcube disconnected from the computer and the run was stopped. Instrument disconnection occurred when the controlling laptop went into sleep mode. Since the run was disconnected right before the final step where DNA is eluted from the filter plate, the elution was performed manually in concurrence with the Study Director. However, following manual elution via centrifugation there were 33 samples which had an "NA" reading on the QIAxpert, indicating no DNA was present and the extraction was potentially unsuccessful for those samples. Since the filter plate was already disposed, the samples could not have elution attempted a second time, so they were re-extracted in Batch A-679-01.201 using retention homogenate from the TissueLyser step.	The QIAcube disconnection during the run for Batch A-579-01.006 (animal 252 samples) was minimally impactful. Due to the disconnection, manual elution was performed and 33 samples had “NA” results on the QIAxpert, leading to re-extraction of the leftover homogenate (Batch A-579-01.201). There were 6 samples that still didn’t have sufficient DNA to obtain a useable measurement on the QIAxpert, and those samples were eluted a second time from the Batch A-579-01.201 filter plate. Although measurements were obtained for all samples from Animal 252, 11 nerve and one spinal cord samples had low concentrations with high %CV (>20% per SOP recommended cutoff for acceptable precision between triplicate readings on the QIAxpert). Samples were used for dPCR analysis and results were obtained. The final impact is minor.
DEV-002290	11/8/2021	Protocol section 11.6, Body Weights, states "Individual body weights for all animals were recorded once prior to transfer from TOXBC HOLD protocol."<br><br>Data shows that body weights were recorded multiple times prior to transfer to study on May 16th, 2023.	There is no study impact. The body weights were recorded while the animals were under the TOXBC HOLD protocol. When animals transfer onto study, all Provantis history transfers with them.

Deviation Number	Date Occurred	Description	Impact
DEV-002293	6/20/2023 – 6/22/2023	Protocol section 8.0 Test System states, "Age of Animals on Day 1; 1.5 to 3 years."<br><br>All animals but 101 and 102 were over 3 years of age. The actual age for animals over the protocol required age range were between 3 years and 23 days and 4 years 9 months and 3 days.	There is no study impact due to the events described in this deviation. The minimum animal age range was important to confirm the availability of test article at correct dose concentration. The maximum age range did not have any impact on the study.
DEV-002294	6/8/2023 – 9/7/2023	Per Protocol section 11.11 Cerebrospinal Fluid States, "Cerebrospinal Fluid (CSF) will be collected from all surviving animals via passive collection from cisterna magna once during the pretest period, Week 2, Week 6, and Week 12."<br><br>Data does not indicate if CSF was collected from the cisterna magna.	There is no study impact due to this missing documentation of CSF being collected via cisterna magna or by lumbar cisterna. Both the sites were scientifically acceptable for CSF collection, and hence there is no impact to the study or data integrity.
DEV-002298	10/16/2023-10/17/2023	The nucleic acid extraction in METHOD-00108 indicated that for dilution calculations the target concentration for DNA was 25 ng/µL, if possible, based on the starting average QIAxpert concentration obtained. Due to a transcription error, two samples were inadvertently diluted when they should have been left undiluted (concentrations were < 25 ng/µl).<br>Sample A-579-01.252.NVSP.02Aug2023.04 had an average concentration of 0.75 ng/µl (entered as 51 ng/µl) and sample A-579-01.252.NVUD.02Aug2023.11 had an average concentration of 4.7 ng/µl (entered as 34.9 ng/µl).	There was no impact to the study due to the error in dilution of DNA for the animal 252 nerve samples (Nerve, ulnar, distal [right, distal]; nerve, sciatic, proximal [left, distal]) as described in the deviation. The reportable value of copies/µg of DNA input could be back calculated from the actual DNA concentration once diluted. Thus, there was no impact to the data integrity.

Deviation Number	Date Occurred	Description	Impact
DEV-002401	7/26/2023 – 7/28/2023	Per Protocol Section 11.5 Nerve Conduction Velocity Testing should occur, "Once during the pretest period and once during the week of scheduled necropsy"..."each animal will undergo nerve conduction velocity testing according to the study specific method. Only those animals scheduled for Week 6 necropsy were supposed to have Nerve Conduction Velocity Testing done, however all animals had the testing done for Week 6.	There is no study impact due to the inadvertently NCV testing for all animals instead of protocol specified week 6 animals only. It would be atypical for these kinds of evaluations to be performed only on one group at any timepoint and would normally be performed on all animals. This additional data does not impact the data integrity but gives a supportive information regarding animal welfare
DEV-002425	10/13/2023	The nucleic acid extraction in METHOD-00108 indicated that for dilution calculations the target concentration for DNA was 25 ng/µL, if possible, based on the starting average QIAxpert concentration obtained. The QIAxpert readings for the average DNA concentration calculation were not copied correctly for some Animal 252 tissue samples (brain, skeletal muscle [tibialis anterior, right], skeletal muscle [tibialis anterior, left], dorsal root ganglion [cervical, thoracic, and lumbar sections], nerve median distal, nerve median proximal). This led to the incorrect concentration of DNA to be calculated and copied to the dilution file.	There is no study impact based on the events described in the deviation. The difference in concentration after the dilution is negligible and did not affect downstream sample processing and data analysis. These concentrations were used in downstream calculations but were able to reconstruct and recalculate based on the original concentration and hence there was no impact to the data.

<table>
<tr><th>Deviation Number</th><th>Date Occurred</th><th>Description</th><th>Impact</th></tr>
<tr><td>DEV-002563</td><td>6/21/2023 – 8/3/2023</td><td>According to Protocol Section 11.10, Blood Collection, “Blood specimens for serum chemistry will be collected in a serum separator tube and allowed to clot at room temperature for at least 30 minutes before processing to serum.”

For the following animals, blood collected for serum chemistry were not allowed to clot for at least 30 minutes prior to processing to serum.
<table>
<tr><th>Timepoint</th><th>Animal ID</th><th>Elapsed Time (Minutes)</th></tr>
<tr><td rowspan="4">Week 1</td><td>301</td><td>29</td></tr>
<tr><td>302</td><td>22</td></tr>
<tr><td>303</td><td>17</td></tr>
<tr><td>304</td><td>12</td></tr>
<tr><td rowspan="2">Week 4</td><td>203</td><td>28</td></tr>
<tr><td>204</td><td>15</td></tr>
<tr><td>Day 43</td><td>304</td><td>28</td></tr>
</table></td><td>There was minimal study impact due to the blood specimens for serum chemistry not allowed to clot for at least 30 minutes prior to processing. According to the manufacturer's instructions, the blood in SST tubes can be allowed to clot for a range of 20-30 minutes and all samples except 303, 304 and 204 were within that range. The serum chemistry from these animals at the specified timepoints did not have any erroneous results. Hence, there was minimal impact to the integrity of the samples.</td></tr>
</table>

<table>
<tr><th>Deviation Number</th><th>Date Occurred</th><th>Description</th><th>Impact</th></tr>
<tr><td>DEV-002565</td><td>5/10/2023</td><td>According to Protocol Section 11.10, Blood Collection, “Blood specimens for coagulation will be collected in sodium citrate tubes and processed immediately upon receipt.”

For the following animals, coagulation blood specimens were not processed immediately upon receipt:
<table>
<tr><th>Timepoint</th><th>Animal ID</th><th>Elapsed Time (hhmm)</th></tr>
<tr><td>Pretest</td><td>All Animals</td><td>0:14</td></tr>
<tr><td rowspan="6">Week 2</td><td>101</td><td>0:33</td></tr>
<tr><td>102</td><td>0:33</td></tr>
<tr><td>151</td><td>0:33</td></tr>
<tr><td>152</td><td>0:33</td></tr>
<tr><td>301-304</td><td>1:23</td></tr>
<tr><td>351-354</td><td>0:22</td></tr>
<tr><td rowspan="6">Week 4</td><td>101</td><td>0:49</td></tr>
<tr><td>102</td><td>0:49</td></tr>
<tr><td>151</td><td>0:49</td></tr>
<tr><td>152</td><td>0:49</td></tr>
<tr><td>301-304</td><td>0:27</td></tr>
<tr><td>351-354</td><td>0:27</td></tr>
<tr><td>Day 43</td><td>All Animals</td><td>Between 0:22 and 1:42</td></tr>
<tr><td>Week 8</td><td>All Animals</td><td>Between 0:16 and 0:42</td></tr>
<tr><td>Day 85</td><td>All Animals</td><td>Between 0:13 and 1:02</td></tr>
</table></td><td>There is no study impact due to delayed processing According to SOP-00197 samples have a 4-hour window after centrifugation to be processed. Even though there was a deviation from protocol the sample integrity was not affected, and the data could be evaluated.</td></tr>
<tr><td>DEV-002739</td><td>6/8/2023 - 9/7/2023</td><td>SOP-00059 deviation. Section 3.1 Documentation and Recording of Data states " Records must include evidence of performance of the activity as described by the SOP, method, protocol, or other guiding document, using sufficient detail such that an individual not involved in the conduct of the study, is able to independently perform or reconstruct the activity as it was originally conducted, based on the records alone. The protocol requirement for the “skin overlaying the cisterna magna or lumbar cisterns will be clipped”, however the collection form indicates either or was clipped but it is unclear which location was clipped for each animal.</td><td>There is likely minimal impact to the study due to this documentation error of not recording which skin was clipped. Skin overlaying either site was acceptable as per the protocol for intrathecal dose administration.</td></tr>
</table>

Deviation Number	Date Occurred	Description	Impact
DEV-002752	6/16/2023 – 8/2/2023	According to Protocol Section 11.10, Blood specimens for Troponin I will be collected in a serum separator tube and allowed to clot at room temperature for at least 30 minutes before processing to serum.<br>For the following animals, Troponin I blood specimens were not allowed to clot for at least 30 minutes prior to processing to serum:<br>**Pretest:** Animal 304 had an elapsed time of only 24 minutes prior to processing<br>**Day 43:** Animal 254 had an elapsed time of only 25 minutes prior to processing.<br>**Week 1:** As a result of using the blood specimens and resulting frozen serum following Serum Chemistry for Troponin I analysis (as outlined in DEV-002735), the blood specimens for serum chemistry, in which the resulting serum was later used for Troponin I analysis, were not allowed to clot at room temperature prior to processing for all animals (see DEV-002206) and the specimens for Animals 301 – 304 were not allowed to clot for at least 30 minutes prior to processing to serum (see DEV-002563).	There was minimal study impact due to the blood specimens for Troponin I not allowed to clot for at least 30 minutes prior to processing. According to the manufacturer's instructions, the blood in SST tubes can be allowed to clot for a range of 20-30 minutes and all samples were within that range. The Troponin I value for each of these animals at the specified timepoints did not have any erroneous results. Hence, there was minimal impact to the integrity of the samples.

Deviation Number	Date Occurred	Description	Impact
DEV-002774 (Addendum to DEV-002331)	11/6/2023	According to Protocol Section 11.12.5, 11.12.6.1, and 11.12.6.2, Flash Frozen Samples and Peripheral and Non-Peripheral Nerve Bank Tissue Samples, “Samples will be maintained on dry ice until transfer to a cold storage unit set to maintain -85 to -60°C until analyzed.”<br>It is unclear in the data if the frozen samples for all animals were maintained on dry ice until transferred to a cold storage unit.	There was potential minimal impact to the study due to the documentation error of samples not being maintained on dry ice before storage. The samples were flash frozen and transferred frozen to the Analytics department. Due to the aberrant data analysis from RT-dPCR, there was a possibility of RNA degradation in case the tissues were not maintained on dry ice. The quality and integrity of RNA extracted from these flash frozen tissues was assessed and determined to be good for downstream dPCR analysis. Thus, there was no impact to the integrity of the frozen tissue samples.
DEV-002235	10/5/2023	For Batch A-579-01.003 during execution of METHOD-00108, Sample A 579-01.201.BRAN.02Aug2023.03 had 540 µL Buffer ATL, 50 µL Proteinase K and 10 µL RNase A added during the tissue homogenization procedure. Based on tissue weight and the indicated buffer volumes in the SSM, the incorrect volumes of Proteinase K and RNase A were added to the tissue sample (10µL less for Proteinase K and 2µL less for the RNase A).	There is minimal impact on the study based on the events described in the deviation. An acceptable amount of quality DNA was extracted for downstream PCR reactions. The difference in the volume of the Proteinase K and RNase A that should have been added was small enough that the difference in extraction efficiency was negligible.

Deviation Number	Date Occurred	Description	Impact
DEV-002236	10/6/2023	The nucleic acid extraction in METHOD-00108 indicated to store DNA samples at -85°C to -60°C after QIAxpert readings were complete. The DNA samples from Batch A-579-01.002 for animal 151 were inadvertently left at room temperature over the weekend instead of being stored at -85°C to -60°C.	There was no study impact due to the samples inadvertently left at room temperature instead of being stored at -85°C to -60°C as per the method. There was no impact to the concentration and purity of the DNA samples analyzed after they were returned to the correct storage conditions. Hence there was no impact to the integrity of the data acquired from these samples.
DEV-002312	11/16/2023	The dPCR SSM METHOD-00112 indicated that positive controls were to be labeled "Pos Con" and that the gBlock (AAV2-ITR) would be the positive control for the AAV2-ITR target and AB-gDNA (NHP DNA) would be the positive control for the B2M target. For Batch A-579-01.004 dPCR, there was a comment on the dPCR plate loading tab that the incorrect positive control was added for plates A-579-01.004.CLC.P05.02 and A 579-01.004.CLC.P06.03. Due to this error the positive control was not positive for these plates (result was less than four positive partitions).	There is minimal impact to the study due to the wrong positive control being added to the dPCR plates. The positive control was added as a control to determine if dPCR worked and these plates showed positive copies/ µL read out for all the samples on the plates proving that the dPCR worked. Hence, missing the positive control on the plates did not impact the integrity of the data.

Deviation Number	Date Occurred	Description	Impact
DEV-002848 (Addendum to DEV-002415)	6/16/2023	METHOD-00108 A-579-01 Study Specific Method for DNA Extraction specified to determine appropriate volumes for TissueLyser homogenization and QIAcube extraction based on tissue starting weight (volumes recorded in Table 3). Incorrect buffer volumes were inadvertently used for two samples based on their weight during homogenization and QIAcube extraction. Sample A-579-01.102.SCSR.12Sep2023.01 was recorded for DNA extraction on 23Oct2023 instead of sample A-579-01.102.SCSR.12Sep2023.03 that was used. Sample A-579-01.102.SCSR.12Sep2023.01 weighed 0.02722g whereas, sample A-579-01.102.SCSR.12Sep2023.03 weighed 0.06271g. Similarly, Sample A-579-01.354.DRGL.14Sep2023.01 was recorded for DNA extraction instead of A-579-01.354.DRGL.14Sep2023.02. Sample A-579-01.354.DRGL.14Sep2023.01 weighed 0.03626g instead of sample A-579-01.354.DRGL.14Sep2023.02 that weighed 0.02907g. Sample A-579-01.102.LVER.12Sep2023.02 was recorded for DNA extraction on 23Oct2023 instead of sample A-579-01.102.LVER.12Sep2023.01 that was used.	There is no study impact due to the incorrect buffer volumes inadvertently used for Animal 102 (spinal roots lumbar and liver) and Animal 354 (dorsal root ganglion lumbar) tissue samples during homogenization and QIAcube extraction. The differences in weight and subsequent buffer amounts used did not have any impact on the DNA extraction. Samples were processed and results showed an ample amount of quality DNA was extracted from the samples to continue processing downstream for dPCR. Hence, there was no impact to the integrity of the data

Deviation Number	Date Occurred	Description	Impact
DEV-002956	2/1/2024	During RNA extraction per METHOD-00107 sample A-579-01.353.NVMD.14Sep2023.05 and A-579-01.253.NVMP.13Sep2023.04 had low RNA recovery and hence no RT-dPCR result could be obtained for these samples for reporting purposes.	There was minimal study impact due to low RNA recovery affecting downstream RT-dPCR process result for two samples. Although no final result was available to include in the data table for statistical analyses, the other available data points for the nerve sections in question (median distal nerve - left distal from animal 353; median proximal nerve - left distal from animal 253) were sufficient to complete statistical analysis and form meaningful conclusions regarding the data. Therefore, the impact to study data integrity is none.
DEV-002957 (Addendum to DEV-002631)	2/1/2024	During RNA extraction per METHOD-00107, sample A-579-01.353.NVMD.14Sep2023.06 was pulled out of the tube for extraction but was so small it was lost in the biosafety cabinet due to its airflow before it could be processed. The additional sample A-579-01.353.NVMD.14Sep2023.05 was selected as a replacement for RNA extraction as per the directive from the Study Director. The RNA extraction from this additional sample was unsuccessful and hence there is no reportable result for this sample.	There was no study impact due to the loss of this nerve sample from animal 353 during RNA extraction. RNA extraction from the backup tissue sample was unsuccessful and therefore no final result was available to include in the data table or statistical analyses. However, the available data points for the nerve section in question (median distal nerve - left, distal) were sufficient to complete statistical analysis and form meaningful conclusions regarding the data. Therefore, the impact to study data integrity is none.

Deviation Number	Date Occurred	Description	Impact
DEV-002728	2/8/2024	According to METHOD-00183, cDNA was to be diluted to 0.208 ng/µL for loading to the B2M+PMP22 master mix and diluted further to 0.00417 ng/µL for loading to the miR871 master mix. Sample A-579-01.354.NVMD.14Sep2023.04 was used at 0.1932 ng/µL for the PMP22/B2M reaction instead of the desired 0.208 ng/µL. Due to this error, subsequently for miRNA sample preparation it was used at 0.003864 ng/µL instead of 0.00417 ng/µL.	The impact to the study is minimal. Although the dilution was not performed correctly, the difference was negligible and can be recalculated to the correct concentration to calculate the input concentrations for data analysis
DEV-003003 (Addendum to DEV-002738)	3/4/2024	According to SOP-00355 for Identifying and Reporting Out of Specification and Aberrant Data Section 3.3, if the cause of aberrant data is not apparent or has severe impact, the study director will initiate an investigation. The investigation was completed in a stepwise fashion using data review and process verification, collaborative discussions both internally and with other departments, Sponsor review and discussion, and a series of complex laboratory experiments to identify a root cause (see attached for a thorough summary of all steps performed and data acquired). The aberrant data (i.e. low B2M copy numbers <150 copies/uL) represented a random sampling of the total study sample population, and laboratory testing ruled out any process or handling errors. There was no singular variable or trend identified as a root cause of the low B2M values in tissue samples.	The final study impact is minimal following completion of the study investigation given that no procedural error was identified. At the conclusion of investigation, reportable values were obtained for the samples with previous aberrant values and the data was sufficient to provide meaningful conclusions.
DEV-002763	2/28/2024	According to SOP-00465, samples run in triplicate may have one well censored, and any samples that cannot be censored to pass acceptance criteria will be repeated unless otherwise determined. The following were identified as samples that were inadvertently rerun but had met acceptance criteria during the initial RT-dPCR analysis and so did not require to be re-processed:<br>• A-579-01.354.NVMD.14Sep2023.07 (A-579-01.354.NMDRP.14Sep2023.07)<br>• A-579-01.302.NVFD.03Aug2023.02 (A-579-01.302.NFDRP.03Aug2023.02)	There is no study impact due to inadvertently retesting the samples. The repeat results are included in the study files but will not be reported since the original result was passing. There is a potential in the case of inadvertent reruns to consume all of the sample or aliquots. In this case more sample is available, and there should be no further reruns of these samples as they were not flagged for rerun.

Deviation Number	Date Occurred	Description	Impact
DEV-002767	2/15/2024	According to METHOD-00183, cDNA was to be diluted to 0.208 ng/µL for loading to the B2M+PMP22 master mix and diluted further to 0.00417 ng/µL for loading to the miR871 master mix. Sample A-579-01.202.NVFP.02Aug2023.02 did not have the stock nucleic acid, desired concentration, volume of diluted nucleic acid desired, volume of nucleic acid desired, or volume of diluent recorded. There is no documentation of sample A-579-01.202.NVFP.02Aug2023.02 being diluted to 0.208 ng/µL in preparation for PMP22 and B2M RT-dPCR analysis.	There is no study impact for the missing documentation of sample dilution. The dPCR results indicate that this nerve sample from animal 202 had the housekeeping gene B2M copies at the expected copies/µL value showing consistency between samples and hence there was no impact to the integrity of the data.
DEV-002816	3/25/2024	According to METHOD-00183, RNA was to be diluted to 5 ng/µL for<br>cDNA synthesis. cDNA concentration was assumed to be half of the input RNA amount (2.5 ng/µL) and diluted to 0.208 ng/µL for loading to the B2M+PMP22 master mix and diluted further to 0.00417 ng/µL for loading to the miR871 master mix. Sample<br>A-579-01.152.NVFP.12Sep2023.12 was diluted to 3.14 ng/µL instead of 5 ng/µL. Downstream dilutions were also impacted - cDNA was actually prepared at 0.132 ng/µL for B2M+PMP22 and 0.00263 ng/µL for miR871.	There is no study impact due to the inadvertent dilution of the RNA to a value lower than the intended 5 ng/uL target value. Although downstream dilutions were impacted, correct concentrations were used to generate reportable values and the RT-dPCR results were within range and passing acceptance criteria.
DEV-003002	4/2/2024 – 5/11/2024	According to the signed Protocol, Ryan Kotowski (Precision Consulting LLC) was listed as the Principal Investigator for dPCR and RT-dPCR statistical analysis.<br>For the dPCR and RT-dPCR stats analysis PIRs, Zachary Cole was the signing Principal Investigator.	No study impact. The assigned PI was involved with the entire phase of statistical analysis of the data but was unavailable to sign the report. There was no impact to the integrity of the data.

Appendix B Test and Control Article Information

Preclinical Manufacturing

Andelyn Biosciences
575 Children's Crossroads
Columbus, OH 43215
1-844-AAV-CDMO

Friday, June 9, 2023

Brian Price, PhD
CTO
Armatus Bio
1275 Kinnear Road
Columbus, OH 43212

Dear Dr. Brian Price,

Please find enclosed the listed rAAV vector. The Certificate of Analysis (CofA) for each vector included in the shipment are on the following pages. Please do not hesitate to contact me if you have any additional questions.

Best Regards,

Laura Kerepesi, Ph.D.
Associate Director - Preclinical Manufacturing
Andelyn Biosciences
5185 Blazer Pkwy, Suite 102
Dublin, OH 43017
Email: Laura.Kerepesi@andelynbiosciences.com
Telephone: 380.226.5141
Fax: 614.355.6832

AAV Viral Vector Certificate of Analysis

Andelyn BIOSCIENCES Reliably Expert. Purposefully Driven.

Lot #: TT970-1
Vector: ARM-101

Order Specifications and Results for Vector:	Minimum	Actual
Yield (total vg)	5E+15	9.3E+15
Physical Titer (vg/mL)	5E+13	4.6E+13
Volume (mL)	N/A	203*

QC Specifications and Results:	Assay Requested	Assay Pass / Value
Endotoxin (EU/mL)	Yes	0.167
Sterile? (Bioburden)	Yes	YES

* There were 203 vials filled at 1mL each. Vials 204, 205, and 206 do not contain exactly 1mL but contain approximately 3 mL of additional vector across the three vials and will be included in the shipment.

-The Physical Titers listed above were generated using a ddPCR method targeting the AAV2 ITRs.

-The vector was analyzed by SDS-PAGE and it was determined that the banding pattern was consistent with rAAV. A scan of the gel is shown below.

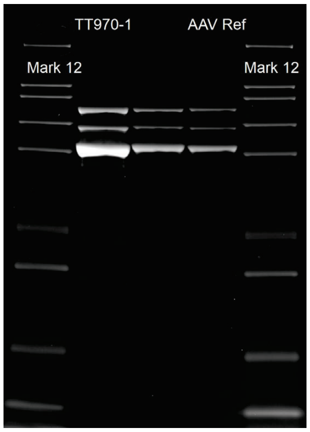

The vector is formulated in 20 mM Tris pH 8.0, 200 mM NaCl, and 1 mM $MgCl_2$ + 0.001% Pluronic F68. The vector may be stored at 4°C for up to a few weeks with no loss of titer. Upon receipt, we recommend storing the vector at ≤-60°C for long-term storage.

I, ___________________________________ acknowledge receipt/shipment on: _____________________.

We respectfully request that you acknowledge the VVC in all publication derived from use of this material

Andelyn BIOSCIENCES
Reliably Expert. Purposefully Driven

CERTIFICATE OF ANALYSIS
VERSION 1

Page 1 of 1

**Product Name:**	TMN200P
**Product Description**	Formulation Buffer [20mM Tris (pH 8.0), 1 mM $MgCl_2$ and 200 mM NaCl containing 0.001% (w/v) Poloxamer 188]
**Lot Number:**	TMNP0921-B
**Date of Manufacture:**	102221 (Vialing Date)
**Expiration Date:**	Pending Stability Testing
**Storage Conditions:**	≤ -60 °C
**Yield (for Research Use):**	114 Vials at 10.0 mL per vial

Release Testing[1]

Test Description	Testing Facility & Method ID	Acceptance Criteria	Result
**Bioburden** – Membrane Filtration with modifications for quantitative analysis.	Andelyn MD-BIOBURDEN-018-05	No Growth Observed	Vial 01: No Growth Observed TAMC: <0.20 CFU/mL TYMC: <0.20 CFU/mL<br><br>Vial 02: No Growth Observed TAMC: <0.20 CFU/mL TYMC: <0.20 CFU/mL<br><br>Vial 03: No Growth Observed TAMC: <0.21 CFU/mL TYMC: <0.21 CFU/mL<br><br>Vial 04: No Growth Observed TAMC: <0.20 CFU/mL TYMC: <0.20 CFU/mL
**Endotoxin** – Kinetic Chromogenic (USP/FDA I/E Assay).	Andelyn MD-ENDOPTS-036-04	≤0.2 EU/mL	0.055 EU/mL
**Osmolality** – Freezing Point Depression.	Andelyn MD-OSMO-041-01	382 – 423 mOsm/kg	406 mOsm/kg
**pH** – pH assay utilizing a micro volume pH probe.	Andelyn MD-PH-042-02	pH = 8.0 ± 0.5	pH = 8.08
**Appearance** – Visual observation of Color, Appearance & Clarity.	BioReliance TS408803GMP.R04	Transparent/Clear to Translucent/Opalescent, Colorless to Faint White Solution	Clarity: Transparent/Clear Color: Colorless Phase: Liquid No Particulates Observed

[1] Release testing was performed on filled vials of product.

APPROVALS

PREPARED BY - FUNCTION	PRINTED NAME	SIGNATURE	DATE
**Analytical Development**	Marissa Evans	*Marissa Evans*	Jan 25, 2022
**APPROVED BY - FUNCTION**	**PRINTED NAME**	**SIGNATURE**	**DATE**
**Quality Control**	Justine Main	[signature]	Jan 26, 2022

Confidential and Proprietary
THE USER OF THIS DOCUMENT IS RESPONSIBLE FOR ENSURING IT IS THE CURRENT VERSION

TMNP0921-B

Final Audit Report 2022-01-26

Created:	2022-01-25
By:	Marissa Evans (marissa.evans@andelynbiosciences.com)
Status:	Signed
Transaction ID:	CBJCHBCAABAAWUTgOiXTZ5AAIGjsZeP-U2CEVIiTLjVk

"TMNP0921-B" History

Document created by Marissa Evans (marissa.evans@andelynbiosciences.com)
2022-01-25 - 9:07:53 PM GMT- IP address: 69.24.144.19

Document e-signed by Marissa Evans (marissa.evans@andelynbiosciences.com)
Signature Date: 2022-01-25 - 9:08:58 PM GMT - Time Source: server- IP address: 69.24.144.19

Document emailed to Justine Main (justine.main@andelynbiosciences.com) for signature
2022-01-25 - 9:09:00 PM GMT

Email viewed by Justine Main (justine.main@andelynbiosciences.com)
2022-01-26 - 12:49:24 PM GMT

Document e-signed by Justine Main (justine.main@andelynbiosciences.com)
Signature Date: 2022-01-26 - 1:07:17 PM GMT - Time Source: server

Agreement completed.
2022-01-26 - 1:07:17 PM GMT

Adobe Sign

Page	Page 1 of 7	
Study Number	A-579-01	
Title	**Dilution of AAV CMT1A in Saline for Group 2**	
Initial/Date:		

1.0 SIGNATURES

Performed by:		Date:	
Steps Verified by:		Date:	
Technical/QC Review:		Date:	

2.0 PURPOSE

This documentation form describes the procedure to dilute scAAV9.U6miR871 vector, AAV CMT1A in Saline for Group 2 IT administration based on the protocol.

3.0 PROCEDURES

3.1 General Instructions

Initial and date the top of the page on the day that the work for that page was begun. Other entries made by the technician on a later date or entries made by another person will be initialed and dated near the data entry. The technicians' initials and dates on each page signify that they have followed the instructions as written, verified that the materials and equipment remained the same as those listed in the tables, and all equipment has been properly calibrated. The materials and equipment documented in the tables are used throughout the procedure by all technicians performing the procedure unless otherwise specified. Any additional information or observations documented during the use of the documentation form or any changes or corrections made to the form will be approved by the study director by writing "approved" and initialing and dating the entry or change. Procedure steps will be verified by a second person and documented in section 1.0.

3.2 Pre-Formulation Activities

Transfer any required materials and equipment to the Formulation Lab.

The formulation supervisor or a designee will assign a unique batch number for each formulation on the form. The batch number will increase in numerical order for the

Page	Page 2 of 7
Study Number	A-579-01
Title	**Dilution of AAV CMT1A in Saline for Group 2**
Initial/Date:	

formulations and will be followed by the chemical acronym and a number corresponding to the concentration.

3.3 Labeling

Labels will be prepared containing all necessary information. Each label will be marked with the appropriate color code as indicated in Table 1.

Table 1. Study Color Codes

Group	Batch Number		Total vg (vg/animal)	Color Code
2 - Low	____-57901-	2	6.0×10^{13}	Blue

For each batch, label the appropriate number of sterile clear glass serum vial mixing/dosing containers for each group.

Page	Page 3 of 7
Study Number	A-579-01
Title	**Dilution of AAV CMT1A in Saline for Group 2**
Initial/Date:	

3.4 Materials and Supplies

See Table 2 for all required materials and supplies. Check the labels carefully to ensure that they are not expired and are the proper purity/grade. Document the supplier, lot number, and expiration date as appropriate on the form.

Table 2. Materials/Supplies

Chemical	Use	Supplier	Lot Number	Expiration Date	Provantis Dispense Container IDs
scAAV9.U6miR871 vector, AAV CMT1A (ARM-101 per receipt paperwork and vials) 4.6 x 10^{13} vector genomes (vg/mL)	Test Article (Stock)	Andelyn Biosciences (Supplier)	TT970-1		
0.9% Sodium Chloride Solution for Injection (Saline)	Vehicle				NA
Sterile Serologial Pipette with Pipet Aid	Measure Vehicle				NA
Sterile Polypropylene Tube (____-mL)	Pool Stock				NA
Sterile Clear Glass Serum Bottles with butyl stopper and crimp cap (____-mL)	Mixing/Dosing Container				NA
Wet Ice Cooler	Store and Transport Doses				NA

Page	Page 4 of 7	
Study Number	A-579-01	
Title	**Dilution of AAV CMT1A in Saline for Group 2**	
Initial/Date:		

3.5 Equipment

See Table 3 for all required major pieces of equipment. Check calibration of all equipment requiring calibration (e.g., balances) to ensure it is current. Document the identity of each piece of equipment on the form.

Table 3. Equipment

Equipment	Use	ID	Calibration Due
Biological Safety Cabinet (BSC)	Prepare Test Article Formulation		
Rainin SL-1000 Pipettor with Sterile Tips	Measure Test Article stock and vehicle Volumes		

3.6 Thawing of AAV Stock

Prior to dosing, remove 62 x 1 mL vials from frozen storage and thaw in a refrigerator at least overnight.

Document the room/unit ID/temperature and time placed in the refrigerator:

____.

Note the number of vials and Dispense Container ID:_______________________.

These vials will be used for the rest of the study.

Page	Page 5 of 7	amplifybio
Study Number	A-579-01	
Title	**Dilution of AAV CMT1A in Saline for Group 2**	
Initial/Date:		

On the day of dosing, remove the appropriate number of stock vials from refrigerated storage and keep on wet ice or chilled beads until ready for dilution. Note vials removed and at what time:

____.

3.7 Verification of Pipettes

Verify the pipettes operation and document in Table 4. Determine the density of water by measuring the temperature of the water used to calculate the % Recovery of the weighed pipette aliquot. For each pipette used, weigh one aliquot at each of the low, mid, and high range of the pipette on verified balance. Proceed with the formulation only if the pipette passes verification. The absolute % Recovery must be between 95 to 105 percent. Verification can be performed within 24 hours of use.

Table 4. Pipette Verification

**Thermometer ID:**	**Calibration Due:**	**Temperature of Water (ºC):**	
Density of Water @ 18.6 to 23.1°C		0.998 g/mL	
Density of Water @ 23.2 to 27.0°C		0.997 g/mL	
**Balance Verifcation (ID:________________) Calibration Due: _______________**			
**Weight Set (ID:________________) Calibration Due: _______________**			
**Nominal Weight (g)**	**Acceptance Range (g)**	**Actual Weight (g)**	
0.005	0.0049 to 0.0051		
100	98.0000 to 102.0000		
**Pipette (ID: ____)**			
**Pipette Setting (mL)**	**Amount Weighed (g)**	**% Recovery = (Weighed Amount ÷ (Density of Water * Setting)) * 100**	**Time Completed**
0.100			
0.500			
1.000			

Page	Page 6 of 7
Study Number	A-579-01
Title	**Dilution of AAV CMT1A in Saline for Group 2**
Initial/Date:	

3.8 Inventory of Test Article

The use and documentation of the bulk test article will be tracked in an electronic inventory.

3.9 Test Article Preparation

Transfer any needed materials into the biological safety cabinet for test article preparation in the Cell and Gene Therapy (CGT) Formulation Lab and prepare from low to high concentration and wipe with PeroxiGard solution.

Place the test article stock vials and saline in the test article BSC and gently invert the test article vials prior to use.

Pool the necessary vials to recover at least 22 mL by pipetting ~1 mL from each stock vial into a sterile 50 mL polypropylene tube.

Cap the tube and invert gently three times.

Using a pipettor with sterile tip, add the appropriate volume of pooled test article stock into a sterile clear glass septum vial according to Table 5.

Using a pipettor with sterile tips, add the appropriate volume of saline to the vial containing the measured test article stock.

Gently mix by pipetting the solution at least three times. Cap the vial.

The dose will expire 24 hours from the time of completion.

Label each vial according to Table 1 and release on wet ice immediately for dosing.

Table 5. Dose Preparation

Group	Target Dose Concentration (vg/mL)	Stock Concentration (vg/mL)	Volume of Stock (mL)	Volume of Saline (mL)	Total Target Volume (mL)	Completion Time/Recorded By Initial/Date
2	2.0×10^{13}	4.6×10^{13}	**17.400**	22.620	40.02	

Page	Page 7 of 7
Study Number	A-579-01
Title	**Dilution of AAV CMT1A in Saline for Group 2**
Initial/Date:	

3.10 Decontamination of Work Area and Equipment

Use PeroxiGard as the decontamination solvent.

Dispose of all waste in biohazard bag or biohazard sharps container.

Wipe down equipment with decon solvent before removing from BSC.

Wipe down interior of BSC with decon solvent.

Dispose of all waste as biohazard waste.

3.11 Distribution of Doses, Residual Thawed Stock

Place labels identifying the doses on the current form and complete the form.

The doses are to be released on wet ice (2° to 8ºC) and will labeled with an expiration date of 24 hours from completion time.

Any residual dose will be stored -85 to -60°C.

Any remaining unused test article stock will be returned to 2 to 8ºC storage pending final disposition.

4.0 REVIEW PROCESS

The formulation supervisor (or designee) will do a technical/QC review of the data. This review will consist of checking for clerical and Good Laboratory Practice (GLP) issues, checking balance calibration, cross-checking all formulation documentation (equipment IDs, lot numbers, etc.) and verification that the correct amount of test article was used. This review should be done prior to formulation use. If this review finds any non-clerical discrepancies, the formulation supervisor or task leader will be notified immediately.

Document Approvals

Number: METHOD-00088 Version: 2.0

Approval Task<br>Verdict: Approve	Sapna Varia,<br>(svaria@amplify-bio.com)<br>Approval<br>23-Dec-2023 20:35:42 GMT+0000

Page	Page 1 of 7	
Study Number	A-579-01	
Title	**Dilution of AAV CMT1A in Saline for Group 3**	
Initial/Date:		

1.0 SIGNATURES

Performed by:		Date:	
Steps Verified by:		Date:	
Technical Review:		Date:	
QC Review:		Date:	

2.0 PURPOSE

This documentation form describes the procedure to dilute scAAV9.U6miR871 vector, AAV CMT1A in Saline for Group 3 IT administration based on the protocol.

3.0 PROCEDURES

3.1 General Instructions

Initial and date the top of the page on the day that the work for that page was begun. Other entries made by the technician on a later date or entries made by another person will be initialed and dated near the data entry. The technicians' initials and dates on each page signify that they have followed the instructions as written, verified that the materials and equipment remained the same as those listed in the tables, and all equipment has been properly calibrated. The materials and equipment documented in the tables are used throughout the procedure by all technicians performing the procedure unless otherwise specified. Any additional information or observations documented during the use of the documentation form or any changes or corrections made to the form will be approved by the study director by writing "approved" and initialing and dating the entry or change. Procedure steps will be verified by a second person and documented in section 1.0.

3.2 Pre-Formulation Activities

Transfer any required materials and equipment to the Formulation Lab.

The formulation supervisor or a designee will assign a unique batch number for each formulation on the form. The batch number will increase in numerical order for the

formulations and will be followed by the chemical acronym and a number corresponding to the concentration.

3.3 Labeling

Labels will be prepared containing all necessary information. Each label will be marked with the appropriate color code as indicated in Table 1.

Table 1. Study Color Codes

Group	Batch Number		Total vg (vg/animal)	Color Code
3 - High	____-57901-	3	1.2×10^{14}	Green

For each batch, label the appropriate number of sterile clear glass serum vial mixing/dosing containers for each group.

Page	Page 3 of 7	
Study Number	A-579-01	
Title	**Dilution of AAV CMT1A in Saline for Group 3**	
Initial/Date:		

3.4 Materials and Supplies

See Table 2 for all required materials and supplies. Check the labels carefully to ensure that they are not expired and are the proper purity/grade. Document the supplier, lot number, and expiration date as appropriate on the form.

Table 2. Materials/Supplies

Chemical	Use	Supplier	Lot Number	Expiration Date	Provantis Dispense Container IDs
scAAV9.U6miR871 vector, AAV CMT1A (ARM-101 per receipt paperwork and vials) 4.6 x 10^{13} vector genomes (vg/mL)	Test Article (Stock)	Andelyn Biosciences	TT970-1		
0.9% Sodium Chloride Solution for Injection (Saline)	Vehicle				NA
Sterile Serologial Pipette with Pipet Aid	Measure Vehicle			NA	NA
Sterile Clear Glass Serum Bottles with butyl stopper and crimp cap (____-mL)	Mixing/Dosing Container			NA	NA
Sterile Polypropylene Tube (50-mL)	Pool Test Article				
Wet Ice Cooler	Store and Transport Doses	NA	NA	NA	NA

Page	Page 4 of 7	
Study Number	A-579-01	
Title	**Dilution of AAV CMT1A in Saline for Group 3**	
Initial/Date:		

3.5 Equipment

See Table 3 for all required major pieces of equipment. Check calibration of all equipment requiring calibration (e.g., balances) to ensure it is current. Document the identity of each piece of equipment on the form.

Table 3. Equipment

Equipment	Use	ID	Calibration Due
Biological Safety Cabinet (BSC)	Prepare Test Article Formulation		
Rainin SL-1000 Pipettor with Sterile Tips	Measure Test Article stock and vehicle Volumes		

3.6 AAV Stock

On the day of dosing, remove the appropriate number of stock vials from refrigerated storage and keep on wet ice or chilled beads until ready for dilution.

Note the number of vials and time and date removed:________________________

Page	Page 5 of 7
Study Number	A-579-01
Title	**Dilution of AAV CMT1A in Saline for Group 3**
Initial/Date:	

3.7 Verification of Pipettes

Verify the pipettes operation and document in Table 4. Determine the density of water by measuring the temperature of the water used to calculate the % Recovery of the weighed pipette aliquot. For each pipette used, weigh one aliquot at each of the low, mid, and high range of the pipette on verified balance. Proceed with the formulation only if the pipette passes verification. The absolute % Recovery must be between 95 to 105 percent. Verification can be performed within 24 hours of use.

Table 4. Pipette Verification

<table>
<tr><th>Thermometer ID:</th><th>Calibration Due:</th><th colspan="2">Temperature of Water (ºC):</th></tr>
<tr><td colspan="2">Density of Water @ 18.6 to 23.1°C</td><td colspan="2">0.998 g/mL</td></tr>
<tr><td colspan="2">Density of Water @ 23.2 to 27.0°C</td><td colspan="2">0.997 g/mL</td></tr>
<tr><td colspan="4">Balance Verifcation (ID:________________) Calibration Due: ______________</td></tr>
<tr><td colspan="4">Weight Set (ID:________________) Calibration Due: ______________</td></tr>
<tr><td>Nominal Weight (g)</td><td>Acceptance Range (g)</td><td colspan="2">Actual Weight (g)</td></tr>
<tr><td>0.005</td><td>0.0049 to 0.0051</td><td colspan="2"></td></tr>
<tr><td>100</td><td>98.0000 to 102.0000</td><td colspan="2"></td></tr>
<tr><td colspan="4">Pipette (ID: ____)</td></tr>
<tr><td>Pipette Setting (mL)</td><td>Amount Weighed (g)</td><td>% Recovery = (Weighed Amount ÷ (Density of Water * Setting)) * 100</td><td>Time Completed</td></tr>
<tr><td>0.100</td><td></td><td></td><td rowspan="3"></td></tr>
<tr><td>0.500</td><td></td><td></td></tr>
<tr><td>1.000</td><td></td><td></td></tr>
</table>

Page	Page 6 of 7
Study Number	A-579-01
Title	**Dilution of AAV CMT1A in Saline for Group 3**
Initial/Date:	

3.8 Inventory of Test Article

The use and documentation of the bulk test article will be tracked in an electronic inventory.

3.9 Test Article Preparation

Transfer any needed materials into the BSC for test article preparation in the Cell and Gene Therapy (CGT) Formulation Lab and prepare from low to high concentration and wipe with PeroxiGard solution.

Place the test article stock vials and saline in the test article BSC and gently invert the test article vials prior to use.

Pool the necessary vials to recover at least 36 mL by pipetting ~1 mL from each stock vial into a sterile 50 mL polypropylene tube.

Cap the tube and invert gently three times.

Using a serological pipet and pipettor with sterile tip, add the appropriate volume of pooled test article stock into a sterile clear glass septum vial according to Table 5.

Using a serological pipet and pipettor with sterile tips, add the appropriate volume of saline to the vial containing the measured test article stock.

Gently mix by pipetting the solution at least three times. Cap the vial.

The dose will expire 24 hours from the time of completion.

Label each vial according to Table 1 and release on wet ice immediately for dosing.

Table 5. Dose Preparation

Group	Target Dose Concentration (vg/mL)	Stock Concentration (vg/mL)	Volume of Stock (mL)	Volume of Saline (mL)	Total Target Volume (mL)	Completion Time/Recorded By Initial/Date
3	4.0×10^{13}	4.6×10^{13}	**34.800**	5.220	40.02	

Page	Page 7 of 7	amplifybio
Study Number	A-579-01	
Title	**Dilution of AAV CMT1A in Saline for Group 3**	
Initial/Date:		

3.10 Decontamination of Work Area and Equipment

Use PeroxiGard as the decontamination solvent.

Dispose of all waste in biohazard bag or biohazard sharps container.

Wipe down equipment with decon solvent before removing from BSC.

Wipe down interior of BSC with decon solvent.

Dispose of all waste as biohazard waste.

3.11 Distribution of Doses, Residual Thawed Stock and Saline

Place labels identifying the doses on the current form and complete the form.

The doses are to be released on wet ice (2° to 8ºC) and will be labeled with an expiration date of 24 hours from completion time.

Any residual dose will be stored -85 to -60°C.

Any remaining unused test article stock will be returned to 2 to 8ºC storage pending final disposition.

Any remaining unused saline will be discarded.

4.0 REVIEW PROCESS

The formulation supervisor (or designee) will do a technical/QC review of the data. This review will consist of checking for clerical and Good Laboratory Practice (GLP) issues, checking balance calibration, cross-checking all formulation documentation (equipment IDs, lot numbers, etc.) and verification that the correct amount of test article was used. This review should be done prior to formulation use. If this review finds any non-clerical discrepancies, the formulation supervisor or task leader will be notified immediately.

Document Approvals

Number: METHOD-00089 Version: 2.0

Approval Task<br>Verdict: Approve	Sapna Varia,<br>(svaria@amplify-bio.com)<br>Approval<br>23-Dec-2023 20:34:52 GMT+0000

Appendix C Intrathecal Catheter Surgery Method

Study Specific Method (SSM) for the Implantation Intrathecal Catheters and Access Ports in Cynomolgus Monkeys

AmplifyBio Study Number: A-579-01

AmplifyBio
1425 State Route 142 (Plain City-Georgesville Road)
West Jefferson, OH 43162

amplifybio

1 PURPOSE

The purpose of this Study Specific Method (SSM) is to describe the procedures to perform surgical implantation of intrathecal catheters with access ports in cynomolgus monkeys for purpose of dose administration.

2 ROLES/RESPONSIBILITIES

Study Surgeon:	Michael Stonerook
Study Surgeon:	Carolyn Fetzek
Study Surgeon:	Dakota Esterline
Study Surgeon (consultant):	David Moddrelle
Study Director:	Sapna Varia

3 SURGICAL PREPARATION AND PROCEDURES

3.1 Surgical Preparation

- The monkey will be sedated with an IM injection of ketamine (15 mg/kg) and brought to the surgical prep room.
- The animal will be intubated and maintained on isoflurane.
- A peripheral catheter will be placed for potential administration of IV fluids
- The fur will be shaved from the surgical area. This will involve the dorsum of the primate from the lower ribs to the sacrum and extend approximately halfway down the lateral sides.
- Skin preparation will involve scrubbing with chlorhexidine scrub and 70% alcohol in alternation for at least two rounds.
- After preparing for surgery, the primate will be transferred to the operating room, placed on a heated surgery pad in a ventral recumbent position.
- The animal will be draped in for surgery including application of an antimicrobial impregnated adhesive barrier (i.e. Ioban or equivalent) and sterile draping material.

3.2 Surgical Procedure

- An incision will be made over the dorsal processes of the lumbar spine (L4, L5, or L6), and a left hemi-laminectomy performed using a drill and a 2 mm burr until the dura is presented.
- The removed bone material generated during the hemi-laminectomy will be salvaged and used to fill the defect at the end of the procedure.
- A durotomy will be performed and a 3F polyurethane catheter introduced and extended cranially for approximately 10 cm until the tip resides in the

midthoracic region. CSF flow will be confirmed, and the catheter will be clamped to prevent leakage of CSF fluid.

- The catheter will be anchored to the surrounding musculature and the defect repaired with the salvaged bone material.
- A small incision and subcutaneous pocket will then be made on the contra-lateral side of the hemi-laminectomy and the distal portion of the catheter tunneled subcutaneously from the spine to the pocket, trimmed to an appropriate length. The catheter line will be unclamped and attached to a compatible access port. Port patency will be confirmed by utilizing a non-coring needle attached to a syringe to infuse a small amount of regular saline (0.09%) or heparinized saline (100 units/mL heparin in 0.9% saline).
- The port-flushing volume will be determined and will be annotated in the Surgical Record.
- The port will be anchored to the underlying muscle bed with non-absorbable suture and then skin incision edges apposed with an appropriate absorbable suture.
- The incision over the lumbar spine will be sutured with appropriately sized absorbable sutures.
- Tissue adhesive may be applied to incision sites.

Physiological variations exist in all animal species. Therefore, alternative procedures, locations and/or materials may be implemented at the discretion of the Surgeon, if deemed necessary. All procedures and/or modifications will be documented on the Surgical Record.

4 PHARMACEUTICAL AGENTS

Pharmaceutical Agents to be used:

Agent	Purpose	Target Dose	Route	Frequency
Ketamine	Anesthetic Induction	15 mg/kg	Intramuscular (IM)	One time prior to surgery
Isoflurane	Anesthetic Maintenance	1 to 3% (carrier gas: 100% O_2)	Inhalation	Maintained intraoperatively
Carprofen	Analgesia/anti-inflammatory	4 mg/kg	Intramuscular (IM)	One time prior to surgery
Buprenorphine	Analgesia	0.01-0.03mg/kg	Intramuscular (IM)	One time prior to surgery
2% Lidocaine	Local analgesia	0.5 mL maximum	Line Block (SC)	One time at incision sites
Enrofloxacin	Antibiotic	5 mg/kg	Intramuscular (IM)	One time prior to surgery

Recover each animal for at least 10 days and administer the following:

Agent	Purpose	Target Dose	Route	Frequency
Carprofen	Analgesic/anti-inflammatory	4 mg/kg	Intramuscular (IM)	SID x 3 days
Buprenorphine	Analgesia	0.01 mg/kg	Intramuscular (IM)	BID x 2 days
Enrofloxacin	Antibiotic	5 mg/kg	IM	SID x 2 days

Pharmaceutical agents and dosages may be extended at the discretion of a staff veterinarian depending on each individual animal's recovery. Actual dose given will be recorded on the appropriate study forms.

5 REVISIONS TO SSM

Revisions or changes to this SSM will be managed within Veeva QualityDocs and identified with a new version number. Any changes or corrections made to the procedure during execution will be approved by the Study Director before the change is executed and the approval will be documented on the SSM. Any minor corrections to the method which do not affect data analysis may be corrected directly on the method without a formal version change

Document Approvals

Number: METHOD-00080 Version: 1.0

Approval Task<br>Verdict: Approve	Sapna Varia,<br>(svaria@amplify-bio.com)<br>Approval<br>16-May-2023 20:57:10 GMT+0000

Appendix D In-Life Animal Data

Table D-1. Group Summary of Clinical Signs

AmplifyBio Study Number A-579-01

Group Summary of Clinical Signs

Observation Type: Routine<br>Sex: Male<br>From Day 1 (Start Date) to 85 (Start Date)	Group 1<br>Control<br>Total vg<br>0	Group 2<br>Low<br>Total vg<br>6x10^13	Group 3<br>High<br>Total vg<br>1.2x10^14
**Normal**			
Number of Animals Affected	2	4	4
% of Affected Animals	100	100	100
Number of Times Recorded	100	203	205
First to Last seen	1 - 84	1 - 84	1 - 84
**Discolored Skin, Face, Red**			
Number of Times Recorded	0	0	0
**Limb Weakness, Forelimb, Right**			
Number of Animals Affected	1	0	0
% of Affected Animals	50	0	0
Number of Times Recorded	5	0	0
First to Last seen	51 - 55	-	-
**Skin Abrasion, Face, Both**			
Number of Times Recorded	0	0	0
**Skin Abrasion, Face, Right**			
Number of Times Recorded	0	0	0
**Skin Abrasion, Forelimb, Left**			
Number of Animals Affected	0	1	0
% of Affected Animals	0	25	0
Number of Times Recorded	0	4	0
First to Last seen	-	23 - 26	-
**Skin Abrasion, Forelimb, Right**			
Number of Times Recorded	0	0	0
**Skin Abrasion, Fore Paw, Left**			
Number of Times Recorded	0	0	0
**Skin Alopecia, Body Dorsal**			
Number of Times Recorded	0	0	0

11/10/2023 10:11:37AM Page: 2

AmplifyBio Study Number A-579-01

Group Summary of Clinical Signs

Observation Type: Routine<br>Sex: Male<br>From Day 1 (Start Date) to 85 (Start Date)	Group 1<br>Control<br>Total vg<br>0	Group 2<br>Low<br>Total vg<br>6x10^13	Group 3<br>High<br>Total vg<br>1.2x10^14
**Skin Alopecia, Body Dorsal, Both**			
Number of Times Recorded	0	0	0
**Skin Alopecia, Head/Neck**			
Number of Times Recorded	0	0	0
**Skin Alopecia, Hind Limb, Both**			
Number of Times Recorded	0	0	0
**Low Food Consumption, 1/2 or Less of Total Food**			
Number of Animals Affected	0	2	0
% of Affected Animals	0	50	0
Number of Times Recorded	0	3	0
First to Last seen	-	73 - 74	-
**Bruising, Face, Right**			
Number of Times Recorded	0	0	0
**Shivering**			
Number of Times Recorded	0	0	0
**Discharge, Vagina, Red**			
Number of Times Recorded	0	0	0
**Diarrhea**			
Number of Animals Affected	2	1	2
% of Affected Animals	100	25	50
Number of Times Recorded	3	7	15
First to Last seen	12 - 12	11 - 83	2 - 26
**Soft Feces**			
Number of Animals Affected	2	2	3
% of Affected Animals	100	50	75
Number of Times Recorded	4	8	5
First to Last seen	13 - 34	2 - 84	27 - 32

AmplifyBio Study Number A-579-01

Group Summary of Clinical Signs

Observation Type: Detailed Clinical Observations<br>Sex: Male<br>From Day 1 (Start Date) to 85 (Start Date)	Group 1<br>Control<br>Total vg<br>0	Group 2<br>Low<br>Total vg<br>6x10^13	Group 3<br>High<br>Total vg<br>1.2x10^14
**Normal**			
Number of Animals Affected	2	4	4
% of Affected Animals	100	100	100
Number of Times Recorded	13	36	34
First to Last seen	1 - 71	1 - 85	1 - 85
**Discolored Skin, Head/Neck, Pale**			
Number of Animals Affected	0	1	0
% of Affected Animals	0	25	0
Number of Times Recorded	0	1	0
First to Last seen	-	22 - 22	-
**Skin Abrasion, Body Dorsal**			
Number of Times Recorded	0	0	0
**Skin Abrasion, Body Dorsal, Right**			
Number of Times Recorded	0	0	0
**Skin Abrasion, Tail, Both**			
Number of Times Recorded	0	0	0
**Skin Abrasion, Face, Both**			
Number of Times Recorded	0	0	0
**Skin Abrasion, Forelimb, Left**			
Number of Animals Affected	0	1	0
% of Affected Animals	0	25	0
Number of Times Recorded	0	1	0
First to Last seen	-	22 - 22	-
**Skin Abrasion, Forelimb, Right**			
Number of Times Recorded	0	0	0
**Skin Abrasion, Hind Limb, Left**			
Number of Times Recorded	0	0	0

AmplifyBio Study Number A-579-01

Group Summary of Clinical Signs

Observation Type: Detailed Clinical Observations<br>Sex: Male<br>From Day 1 (Start Date) to 85 (Start Date)	Group 1<br>Control<br>Total vg<br>0	Group 2<br>Low<br>Total vg<br>6x10^13	Group 3<br>High<br>Total vg<br>1.2x10^14
**Skin Abrasion, Fore Paw, Left**			
Number of Times Recorded	0	0	0
**Skin Abrasion, Fore Paw, Right**			
Number of Animals Affected	1	0	0
% of Affected Animals	50	0	0
Number of Times Recorded	5	0	0
First to Last seen	50 - 85	-	-
**Skin Alopecia, Body Dorsal**			
Number of Times Recorded	0	0	0
**Skin Alopecia, Body Dorsal, Left**			
Number of Times Recorded	0	0	0
**Skin Alopecia, Head/Neck**			
Number of Times Recorded	0	0	0
**Skin Alopecia, Head/Neck, Both**			
Number of Animals Affected	2	0	0
% of Affected Animals	100	0	0
Number of Times Recorded	2	0	0
First to Last seen	15 - 15	-	-
**Skin Alopecia, Forelimb, Both**			
Number of Times Recorded	0	0	0
**Skin Alopecia, Hind Limb, Both**			
Number of Times Recorded	0	0	0
**Bruising, Face, Right**			
Number of Times Recorded	0	0	0
**Scabbing, Body Dorsal**			
Number of Times Recorded	0	0	0

AmplifyBio Study Number A-579-01

Group Summary of Clinical Signs

Observation Type: Detailed Clinical Observations<br>Sex: Male<br>From Day 1 (Start Date) to 85 (Start Date)	Group 1<br>Control<br>Total vg<br>0	Group 2<br>Low<br>Total vg<br>6x10^13	Group 3<br>High<br>Total vg<br>1.2x10^14
**Discharge, Nose, Clear**			
Number of Animals Affected	0	1	0
% of Affected Animals	0	25	0
Number of Times Recorded	0	2	0
First to Last seen	-	22 - 29	-
**Discharge, Vagina, Red**			
Number of Times Recorded	0	0	0
**Diarrhea**			
Number of Animals Affected	0	1	2
% of Affected Animals	0	25	50
Number of Times Recorded	0	1	4
First to Last seen	-	85 - 85	1 - 22
**Open Mouth Breathing**			
Number of Animals Affected	0	1	0
% of Affected Animals	0	25	0
Number of Times Recorded	0	1	0
First to Last seen	-	22 - 22	-
**Soft Feces**			
Number of Animals Affected	0	0	2
% of Affected Animals	0	0	50
Number of Times Recorded	0	0	2
First to Last seen	-	-	29 - 29

AmplifyBio Study Number A-579-01

Group Summary of Clinical Signs

Observation Type: Routine<br>Sex: Female<br>From Day 1 (Start Date) to 85 (Start Date)	Group 1<br>Control<br>Total vg<br>0	Group 2<br>Low<br>Total vg<br>6x10^13	Group 3<br>High<br>Total vg<br>1.2x10^14
**Normal**			
Number of Animals Affected	2	4	4
% of Affected Animals	100	100	100
Number of Times Recorded	98	219	185
First to Last seen	1 - 77	1 - 84	1 - 83
**Discolored Skin, Face, Red**			
Number of Animals Affected	0	0	1
% of Affected Animals	0	0	25
Number of Times Recorded	0	0	3
First to Last seen	-	-	37 - 39
**Limb Weakness, Forelimb, Right**			
Number of Times Recorded	0	0	0
**Skin Abrasion, Face, Both**			
Number of Animals Affected	1	0	0
% of Affected Animals	50	0	0
Number of Times Recorded	5	0	0
First to Last seen	68 - 73	-	-
**Skin Abrasion, Face, Right**			
Number of Animals Affected	0	0	1
% of Affected Animals	0	0	25
Number of Times Recorded	0	0	2
First to Last seen	-	-	38 - 39
**Skin Abrasion, Forelimb, Left**			
Number of Times Recorded	0	0	0
**Skin Abrasion, Forelimb, Right**			
Number of Animals Affected	1	0	0
% of Affected Animals	50	0	0
Number of Times Recorded	3	0	0

AmplifyBio Study Number A-579-01

Group Summary of Clinical Signs

Observation Type: Routine<br>Sex: Female<br>From Day 1 (Start Date) to 85 (Start Date)	Group 1<br>Control<br>Total vg<br>0	Group 2<br>Low<br>Total vg<br>6x10^13	Group 3<br>High<br>Total vg<br>1.2x10^14
**Skin Abrasion, Forelimb, Right (Continued...)**			
First to Last seen	79 - 81	-	-
**Skin Abrasion, Fore Paw, Left**			
Number of Animals Affected	1	0	0
% of Affected Animals	50	0	0
Number of Times Recorded	4	0	0
First to Last seen	77 - 81	-	-
**Skin Alopecia, Body Dorsal**			
Number of Animals Affected	1	0	0
% of Affected Animals	50	0	0
Number of Times Recorded	3	0	0
First to Last seen	82 - 84	-	-
**Skin Alopecia, Body Dorsal, Both**			
Number of Animals Affected	0	0	1
% of Affected Animals	0	0	25
Number of Times Recorded	0	0	7
First to Last seen	-	-	1 - 7
**Skin Alopecia, Head/Neck**			
Number of Animals Affected	0	0	1
% of Affected Animals	0	0	25
Number of Times Recorded	0	0	7
First to Last seen	-	-	1 - 7
**Skin Alopecia, Hind Limb, Both**			
Number of Animals Affected	0	0	2
% of Affected Animals	0	0	50
Number of Times Recorded	0	0	13
First to Last seen	-	-	1 - 14

AmplifyBio Study Number A-579-01

Group Summary of Clinical Signs

Observation Type: Routine<br>Sex: Female<br>From Day 1 (Start Date) to 85 (Start Date)	Group 1<br>Control<br>Total vg<br>0	Group 2<br>Low<br>Total vg<br>6x10^13	Group 3<br>High<br>Total vg<br>1.2x10^14
**Low Food Consumption, 1/2 or Less of Total Food**			
Number of Animals Affected	0	1	1
% of Affected Animals	0	25	25
Number of Times Recorded	0	2	2
First to Last seen	-	74 - 77	45 - 73
**Bruising, Face, Right**			
Number of Animals Affected	0	0	1
% of Affected Animals	0	0	25
Number of Times Recorded	0	0	3
First to Last seen	-	-	40 - 42
**Shivering**			
Number of Animals Affected	1	0	0
% of Affected Animals	50	0	0
Number of Times Recorded	1	0	0
First to Last seen	12 - 12	-	-
**Discharge, Vagina, Red**			
Number of Animals Affected	1	1	2
% of Affected Animals	50	25	50
Number of Times Recorded	2	2	7
First to Last seen	61 - 62	60 - 61	2 - 70
**Diarrhea**			
Number of Animals Affected	0	0	2
% of Affected Animals	0	0	50
Number of Times Recorded	0	0	4
First to Last seen	-	-	53 - 84
**Soft Feces**			
Number of Animals Affected	0	0	2
% of Affected Animals	0	0	50

AmplifyBio Study Number A-579-01

Group Summary of Clinical Signs

Observation Type: Routine<br>Sex: Female<br>From Day 1 (Start Date) to 85 (Start Date)	Group 1<br>Control<br>Total vg<br>0	Group 2<br>Low<br>Total vg<br>6x10^13	Group 3<br>High<br>Total vg<br>1.2x10^14
**Soft Feces (Continued...)**			
Number of Times Recorded	0	0	4
First to Last seen	-	-	3 - 4

AmplifyBio Study Number A-579-01

Group Summary of Clinical Signs

Observation Type: Detailed Clinical Observations<br>Sex: Female<br>From Day 1 (Start Date) to 85 (Start Date)	Group 1<br>Control<br>Total vg<br>0	Group 2<br>Low<br>Total vg<br>6x10^13	Group 3<br>High<br>Total vg<br>1.2x10^14
**Normal**			
Number of Animals Affected	2	4	4
% of Affected Animals	100	100	100
Number of Times Recorded	14	36	26
First to Last seen	1 - 64	1 - 85	1 - 85
**Discolored Skin, Head/Neck, Pale**			
Number of Times Recorded	0	0	0
**Skin Abrasion, Body Dorsal**			
Number of Animals Affected	0	0	1
% of Affected Animals	0	0	25
Number of Times Recorded	0	0	7
First to Last seen	-	-	43 - 85
**Skin Abrasion, Body Dorsal, Right**			
Number of Animals Affected	0	1	0
% of Affected Animals	0	25	0
Number of Times Recorded	0	1	0
First to Last seen	-	8 - 8	-
**Skin Abrasion, Tail, Both**			
Number of Animals Affected	0	1	0
% of Affected Animals	0	25	0
Number of Times Recorded	0	1	0
First to Last seen	-	15 - 15	-
**Skin Abrasion, Face, Both**			
Number of Animals Affected	1	0	0
% of Affected Animals	50	0	0
Number of Times Recorded	1	0	0
First to Last seen	71 - 71	-	-

AmplifyBio Study Number A-579-01

Group Summary of Clinical Signs

Observation Type: Detailed Clinical Observations<br>Sex: Female<br>From Day 1 (Start Date) to 85 (Start Date)	Group 1<br>Control<br>Total vg<br>0	Group 2<br>Low<br>Total vg<br>6x10^13	Group 3<br>High<br>Total vg<br>1.2x10^14
**Skin Abrasion, Forelimb, Left**			
Number of Times Recorded	0	0	0
**Skin Abrasion, Forelimb, Right**			
Number of Animals Affected	1	0	0
% of Affected Animals	50	0	0
Number of Times Recorded	1	0	0
First to Last seen	78 - 78	-	-
**Skin Abrasion, Hind Limb, Left**			
Number of Animals Affected	1	0	0
% of Affected Animals	50	0	0
Number of Times Recorded	1	0	0
First to Last seen	78 - 78	-	-
**Skin Abrasion, Fore Paw, Left**			
Number of Animals Affected	1	0	0
% of Affected Animals	50	0	0
Number of Times Recorded	1	0	0
First to Last seen	78 - 78	-	-
**Skin Abrasion, Fore Paw, Right**			
Number of Times Recorded	0	0	0
**Skin Alopecia, Body Dorsal**			
Number of Animals Affected	0	0	1
% of Affected Animals	0	0	25
Number of Times Recorded	0	0	1
First to Last seen	-	-	1 - 1
**Skin Alopecia, Body Dorsal, Left**			
Number of Animals Affected	1	0	0
% of Affected Animals	50	0	0
Number of Times Recorded	2	0	0

AmplifyBio Study Number A-579-01

Group Summary of Clinical Signs

Observation Type: Detailed Clinical Observations<br>Sex: Female<br>From Day 1 (Start Date) to 85 (Start Date)	Group 1<br>Control<br>Total vg<br>0	Group 2<br>Low<br>Total vg<br>6x10^13	Group 3<br>High<br>Total vg<br>1.2x10^14
**Skin Alopecia, Body Dorsal, Left (Continued...)**			
First to Last seen	78 - 85	-	-
**Skin Alopecia, Head/Neck**			
Number of Animals Affected	0	0	1
% of Affected Animals	0	0	25
Number of Times Recorded	0	0	1
First to Last seen	-	-	1 - 1
**Skin Alopecia, Head/Neck, Both**			
Number of Times Recorded	0	0	0
**Skin Alopecia, Forelimb, Both**			
Number of Animals Affected	1	0	0
% of Affected Animals	50	0	0
Number of Times Recorded	2	0	0
First to Last seen	78 - 85	-	-
**Skin Alopecia, Hind Limb, Both**			
Number of Animals Affected	0	0	1
% of Affected Animals	0	0	25
Number of Times Recorded	0	0	1
First to Last seen	-	-	8 - 8
**Bruising, Face, Right**			
Number of Animals Affected	0	0	1
% of Affected Animals	0	0	25
Number of Times Recorded	0	0	1
First to Last seen	-	-	43 - 43
**Scabbing, Body Dorsal**			
Number of Animals Affected	0	1	1
% of Affected Animals	0	25	25
Number of Times Recorded	0	1	2

AmplifyBio Study Number A-579-01

Group Summary of Clinical Signs

Observation Type: Detailed Clinical Observations<br>Sex: Female<br>From Day 1 (Start Date) to 85 (Start Date)	Group 1<br>Control<br>Total vg<br>0	Group 2<br>Low<br>Total vg<br>6x10^13	Group 3<br>High<br>Total vg<br>1.2x10^14
**Scabbing, Body Dorsal (Continued...)**			
First to Last seen	-	1 - 1	1 - 8
**Discharge, Nose, Clear**			
Number of Times Recorded	0	0	0
**Discharge, Vagina, Red**			
Number of Animals Affected	2	1	2
% of Affected Animals	100	25	50
Number of Times Recorded	2	1	2
First to Last seen	15 - 22	22 - 22	29 - 64
**Diarrhea**			
Number of Animals Affected	1	0	1
% of Affected Animals	50	0	25
Number of Times Recorded	1	0	1
First to Last seen	36 - 36	-	1 - 1
**Open Mouth Breathing**			
Number of Times Recorded	0	0	0
**Soft Feces**			
Number of Times Recorded	0	0	0

AmplifyBio Study Number A-579-01

Group Summary of Clinical Signs

Key Page

Group Information

Short Name	Long Name	Type	Report Headings			
1	Group 1	Control	Group 1	Control	Total vg	0
2	Group 2	Dose	Group 2	Low	Total vg	6x10^13
3	Group 3	Dose	Group 3	High	Total vg	1.2x10^14

Table D-2. Group Mean Body Weight and Weight Gain

AmplifyBio Study Number A-579-01

Group Mean Body Weight & Weight Gain

Body Weight (kg)

Sex: Male		Day(s) Relative to Start Date						
		1	8	15	22	29	36	43
Group 1	Mean	2.62	2.55	2.72	2.64	2.75	2.83	2.72
Control	SD	0.35	0.38	0.36	0.37	0.43	0.45	0.44
Total vg	N	2	2	2	2	2	2	2
0		-	-	-	-	-	-	-
Group 2	Mean	2.65	2.57	2.72	2.68	2.77	2.88	2.74
Low	SD	0.34	0.33	0.32	0.33	0.38	0.40	0.40
Total vg	N	4	4	4	4	4	4	4
6x10^13		-	-	-	-	-	-	-
Group 3	Mean	2.64	2.51	2.65	2.55	2.70	2.77	2.67
High	SD	0.33	0.32	0.37	0.44	0.38	0.35	0.32
Total vg	N	4	4	4	4	4	4	4
1.2x10^14		-	-	-	-	-	-	-

AmplifyBio Study Number A-579-01

Group Mean Body Weight & Weight Gain

Body Weight (kg)

Sex: Male		Day(s) Relative to Start Date					
		50	57	64	71	78	85
Group 1	Mean	3.09	3.13	3.16	3.31	3.38	3.31
Control	SD	-	-	-	-	-	-
Total vg	N	1	1	1	1	1	1
0		-	-	-	-	-	-
Group 2	Mean	2.78	2.88	2.91	2.97	2.86	2.74
Low	SD	0.52	0.57	0.54	0.54	0.45	0.33
Total vg	N	2	2	2	2	2	2
6x10^13		-	-	-	-	-	-
Group 3	Mean	2.83	2.96	2.98	3.09	2.96	3.03
High	SD	0.54	0.51	0.54	0.57	0.62	0.58
Total vg	N	2	2	2	2	2	2
1.2x10^14		-	-	-	-	-	-

AmplifyBio Study Number A-579-01

Group Mean Body Weight & Weight Gain

Body Weight (kg)

Sex: Female		Day(s) Relative to Start Date						
		1	8	15	22	29	36	43
Group 1	Mean	3.92	3.86	4.02	3.87	3.92	4.07	3.92
Control	SD	1.81	1.72	1.68	1.63	1.72	1.75	1.65
Total vg	N	2	2	2	2	2	2	2
0		-	-	-	-	-	-	-
Group 2	Mean	2.91	2.81	2.95	2.90	2.97	3.04	2.91
Low	SD	0.29	0.31	0.21	0.17	0.24	0.29	0.27
Total vg	N	4	4	4	4	4	4	4
6x10^13		-	-	-	-	-	-	-
Group 3	Mean	2.79	2.69	2.81	2.78	2.85	2.95	2.80
High	SD	0.23	0.24	0.34	0.30	0.32	0.37	0.29
Total vg	N	4	4	4	4	4	4	4
1.2x10^14		-	-	-	-	-	-	-

AmplifyBio Study Number A-579-01

Group Mean Body Weight & Weight Gain

Body Weight (kg)

Sex: Female		Day(s) Relative to Start Date 50	57	64	71	78	85
Group 1	Mean	5.14	5.23	5.21	5.39	5.36	5.39
Control	SD	-	-	-	-	-	-
Total vg	N	1	1	1	1	1	1
0		-	-	-	-	-	-
Group 2	Mean	2.96	3.12	3.12	3.18	3.11	3.11
Low	SD	0.33	0.37	0.43	0.41	0.57	0.57
Total vg	N	2	2	2	2	2	2
6x10^13		-	-	-	-	-	-
Group 3	Mean	3.01	3.04	3.08	3.11	3.05	2.85
High	SD	0.17	0.15	0.08	0.03	0.10	0.04
Total vg	N	2	2	2	2	2	2
1.2x10^14		-	-	-	-	-	-

AmplifyBio Study Number A-579-01

Group Mean Body Weight & Weight Gain

Absolute BodyWeight Changes (kg)

Sex: Male		Day(s) Relative to Start Date						
		1 → 8	8 → 15	15 → 22	22 → 29	29 → 36	36 → 43	43 → 50
Group 1	Mean	-0.07	0.17	-0.08	0.11	0.08	-0.11	0.06
Control	SD	0.03	0.02	0.01	0.06	0.01	0.01	.
Total vg	N	2	2	2	2	2	2	1
0		.	.	.	.	.	.	.
Group 2	Mean	-0.08	0.15	-0.04	0.09	0.10	-0.14	0.05
Low	SD	0.05	0.03	0.03	0.06	0.04	0.05	0.02
Total vg	N	4	4	4	4	4	4	2
6x10^13		.	.	.	.	.	.	.
Group 3	Mean	-0.12	0.13	-0.09	0.15	0.07	-0.10	0.07
High	SD	0.06	0.05	0.09	0.10	0.09	0.03	0.07
Total vg	N	4	4	4	4	4	4	2
1.2x10^14		.	.	.	.	.	.	.

AmplifyBio Study Number A-579-01

Group Mean Body Weight & Weight Gain

Absolute BodyWeight Changes (kg)

Sex: Male		Day(s) Relative to Start Date				
		50 → 57	57 → 64	64 → 71	71 → 78	78 → 85
Group 1	Mean	0.04	0.03	0.15	0.07	-0.07
Control	SD	.	.	.	.	.
Total vg	N	1	1	1	1	1
0		.	.	.	.	.
Group 2	Mean	0.10	0.04	0.06	-0.11	-0.13
Low	SD	0.06	0.04	0.00	0.08	0.12
Total vg	N	2	2	2	2	2
6x10^13		.	.	.	.	.
Group 3	Mean	0.14	0.02	0.11	-0.13	0.07
High	SD	0.04	0.04	0.03	0.05	0.04
Total vg	N	2	2	2	2	2
1.2x10^14		.	.	.	.	.

AmplifyBio Study Number A-579-01

Group Mean Body Weight & Weight Gain

Absolute BodyWeight Changes (kg)

Sex: Female		Day(s) Relative to Start Date						
		1 → 8	8 → 15	15 → 22	22 → 29	29 → 36	36 → 43	43 → 50
Group 1	Mean	-0.07	0.16	-0.15	0.05	0.16	-0.16	0.06
Control	SD	0.09	0.04	0.04	0.08	0.04	0.11	.
Total vg	N	2	2	2	2	2	2	1
0		.	.	.	.	.	.	.
Group 2	Mean	-0.11	0.15	-0.06	0.08	0.07	-0.13	0.06
Low	SD	0.05	0.12	0.04	0.08	0.05	0.05	0.02
Total vg	N	4	4	4	4	4	4	2
6x10^13		.	.	.	.	.	.	.
Group 3	Mean	-0.10	0.12	-0.03	0.07	0.10	-0.15	0.04
High	SD	0.05	0.16	0.06	0.06	0.09	0.10	0.01
Total vg	N	4	4	4	4	4	4	2
1.2x10^14		.	.	.	.	.	.	.

AmplifyBio Study Number A-579-01

Group Mean Body Weight & Weight Gain

Absolute BodyWeight Changes (kg)

Sex: Female		Day(s) Relative to Start Date				
		50 → 57	57 → 64	64 → 71	71 → 78	78 → 85
Group 1	Mean	0.09	-0.02	0.18	-0.03	0.03
Control	SD	.	.	.	.	.
Total vg	N	1	1	1	1	1
0		.	.	.	.	.
Group 2	Mean	0.16	0.00	0.07	-0.07	-0.01
Low	SD	0.05	0.06	0.02	0.16	0.01
Total vg	N	2	2	2	2	2
6x10^13		.	.	.	.	.
Group 3	Mean	0.03	0.04	0.04	-0.06	-0.20
High	SD	0.02	0.07	0.11	0.07	0.06
Total vg	N	2	2	2	2	2
1.2x10^14		.	.	.	.	.

AmplifyBio Study Number A-579-01

Group Mean Body Weight & Weight Gain

Key Page

Measurement Descriptions

Headings Used	Description
Body Weight	Body Weight
Absolute BodyWeight Changes	Absolute BodyWeight Changes (kg)

Measurement/Statistics

Measurement	Descriptive	Comparative	Arithmetic /Adjusted	Transformation
Body Weight	Mean			
	Standard Deviation			
	Count (N)			
Absolute BodyWeight Changes	Mean			
	Standard Deviation			
	Count (N)			

Group Information

Short Name	Long Name	Type	Report Headings 1-4			
1	Group 1	Control	Group 1	Control	Total vg	0
2	Group 2	Dose	Group 2	Low	Total vg	6x10^13
3	Group 3	Dose	Group 3	High	Total vg	1.2x10^14

Table D-3. Individual Animal Clinical Observations

AmplifyBio Study Number A-579-01

Individual Animal Clinical Signs

Group 1 Control Total vg 0 Sex: Male	Observation Type: Routine	Day(s) Relative to Start Date						
		1	2	3	4	5	6	7
101	Normal	X	X	X	X	X	X	X
	Diarrhea	.	.	.	.	.	.	.
	Soft Feces	.	.	.	.	.	.	.
102	Normal	X	X	X	X	X	X	X
	Limb Weakness, Forelimb	.	.	.	.	.	.	.
	Diarrhea	.	.	.	.	.	.	.
	Soft Feces	.	.	.	.	.	.	.

X=Present

AmplifyBio Study Number A-579-01

Individual Animal Clinical Signs

Group 1<br>Control<br>Total vg<br>0<br>Sex: Male	Observation Type: Routine	Day(s) Relative to Start Date						
		9	10	10<br>A	11	11<br>A	12	12<br>A
101	Normal	X	X	.	X	.	.	.
	Diarrhea	.	.	.	.	.	X	.
	Soft Feces	.	.	.	.	.	.	.
102	Normal	X	X	.	X	.	.	.
	Limb Weakness, Forelimb	.	.	.	.	.	.	.
	Diarrhea	.	.	.	.	.	X	X
	Soft Feces	.	.	.	.	.	.	.

X=Present

AmplifyBio Study Number A-579-01

Individual Animal Clinical Signs

Group 1<br>Control<br>Total vg<br>0<br>Sex: Male	Observation Type: Routine	Day(s) Relative to Start Date<br>13	 13<br>A	 14	 16	 17	 18	 19
101	Normal	.	.	X	X	X	X	X
	Diarrhea	.	.	.	.	.	.	.
	Soft Feces	X	.	.	.	.	.	.
102	Normal	.	.	X	X	X	X	X
	Limb Weakness, Forelimb	.	.	.	.	.	.	.
	Diarrhea	.	.	.	.	.	.	.
	Soft Feces	X	.	.	.	.	.	.

X=Present

AmplifyBio Study Number A-579-01

Individual Animal Clinical Signs

Group 1<br>Control<br>Total vg<br>0<br>Sex: Male	Observation Type: Routine	Day(s) Relative to Start Date						
		20	21	21<br>A	23	24	25	26
101	Normal	X	X	.	X	X	X	X
	Diarrhea	.	.	.	.	.	.	.
	Soft Feces	.	.	.	.	.	.	.
102	Normal	X	X	.	X	X	X	X
	Limb Weakness, Forelimb	.	.	.	.	.	.	.
	Diarrhea	.	.	.	.	.	.	.
	Soft Feces	.	.	.	.	.	.	.

X=Present

AmplifyBio Study Number A-579-01

Individual Animal Clinical Signs

Group 1<br>Control<br>Total vg<br>0<br>Sex: Male	Observation Type: Routine	Day(s) Relative to Start Date						
		27	28	30	31	32	33	34
101	Normal	X	X	X	X	X	X	.
	Diarrhea	.	.	.	.	.	.	.
	Soft Feces	.	.	.	.	.	.	X
102	Normal	X	X	X	X	X	X	.
	Limb Weakness, Forelimb	.	.	.	.	.	.	.
	Diarrhea	.	.	.	.	.	.	.
	Soft Feces	.	.	.	.	.	.	X

X=Present

AmplifyBio Study Number A-579-01

Individual Animal Clinical Signs

Group 1 Control Total vg 0 Sex: Male	Observation Type: Routine	Day(s) Relative to Start Date						
		35	37	37 A	38	39	40	41
101	Normal	X	X	.	X	X	X	X
	Diarrhea	.	.	.	.	.	.	.
	Soft Feces	.	.	.	.	.	.	.
102	Normal	X	X	.	X	X	X	X
	Limb Weakness, Forelimb	.	.	.	.	.	.	.
	Diarrhea	.	.	.	.	.	.	.
	Soft Feces	.	.	.	.	.	.	.

X=Present

AmplifyBio Study Number A-579-01

Individual Animal Clinical Signs

Group 1<br>Control<br>Total vg<br>0<br>Sex: Male	Observation Type: Routine	Day(s) Relative to Start Date						
		42	44	45	45<br>A	46	47	48
101	Normal	X	.	.	.	.	.	.
	Diarrhea	.	.	.	.	.	.	.
	Soft Feces	.	.	.	.	.	.	.
102	Normal	X	X	X	.	X	X	X
	Limb Weakness, Forelimb	.	.	.	.	.	.	.
	Diarrhea	.	.	.	.	.	.	.
	Soft Feces	.	.	.	.	.	.	.

X=Present

AmplifyBio Study Number A-579-01

Individual Animal Clinical Signs

Group 1<br>Control<br>Total vg<br>0<br>Sex: Male	Observation Type: Routine	Day(s) Relative to Start Date						
		49	51	51<br>A	52	53	54	55
101	Normal	.	.	.	.	.	.	.
	Diarrhea	.	.	.	.	.	.	.
	Soft Feces	.	.	.	.	.	.	.
102	Normal	X	X	.	.	.	.	.
	Limb Weakness, Forelimb	.	.	RT	RT	RT	RT	RT
	Diarrhea	.	.	.	.	.	.	.
	Soft Feces	.	.	.	.	.	.	.

X=Present; RT=Right

AmplifyBio Study Number A-579-01

Individual Animal Clinical Signs

Group 1 Control Total vg 0 Sex: Male	Observation Type: Routine	Day(s) Relative to Start Date						
		56	58	59	60	60 A	61	61 A
101	Normal	.	.	.	.	.	.	.
	Diarrhea	.	.	.	.	.	.	.
	Soft Feces	.	.	.	.	.	.	.
102	Normal	X	X	X	X	.	X	.
	Limb Weakness, Forelimb	.	.	.	.	.	.	.
	Diarrhea	.	.	.	.	.	.	.
	Soft Feces	.	.	.	.	.	.	.

X=Present

AmplifyBio Study Number A-579-01

Individual Animal Clinical Signs

Group 1 Control Total vg 0 Sex: Male	Observation Type: Routine	Day(s) Relative to Start Date						
		62	63	65	66	67	68	69
101	Normal	.	.	.	.	.	.	.
	Diarrhea	.	.	.	.	.	.	.
	Soft Feces	.	.	.	.	.	.	.
102	Normal	X	X	X	X	X	X	X
	Limb Weakness, Forelimb	.	.	.	.	.	.	.
	Diarrhea	.	.	.	.	.	.	.
	Soft Feces	.	.	.	.	.	.	.

X=Present

AmplifyBio Study Number A-579-01

Individual Animal Clinical Signs

Group 1<br>Control<br>Total vg<br>0<br>Sex: Male	Observation Type: Routine	Day(s) Relative to Start Date<br>70	 72	 73	 73<br>A	 74	 74<br>A	 75
101	Normal	.	.	.	.	.	.	.
	Diarrhea	.	.	.	.	.	.	.
	Soft Feces	.	.	.	.	.	.	.
102	Normal	X	X	X	.	X	.	X
	Limb Weakness, Forelimb	.	.	.	.	.	.	.
	Diarrhea	.	.	.	.	.	.	.
	Soft Feces	.	.	.	.	.	.	.

X=Present

AmplifyBio Study Number A-579-01

Individual Animal Clinical Signs

Group 1<br>Control<br>Total vg<br>0<br>Sex: Male	Observation Type: Routine	Day(s) Relative to Start Date						
		76	77	77<br>A	78<br>A	79	80	80<br>A
101	Normal	.	.	.	.	.	.	.
	Diarrhea	.	.	.	.	.	.	.
	Soft Feces	.	.	.	.	.	.	.
102	Normal	X	X	.	.	X	X	.
	Limb Weakness, Forelimb	.	.	.	.	.	.	.
	Diarrhea	.	.	.	.	.	.	.
	Soft Feces	.	.	.	.	.	.	.

X=Present

AmplifyBio Study Number A-579-01

Individual Animal Clinical Signs

Group 1<br>Control<br>Total vg<br>0<br>Sex: Male	Observation Type: Routine	Day(s) Relative to Start Date<br>81	82	83	84			
101	Normal	.	.	.	.			
	Diarrhea	.	.	.	.			
	Soft Feces	.	.	.	.			
102	Normal	X	X	X	X			
	Limb Weakness, Forelimb	.	.	.	.			
	Diarrhea	.	.	.	.			
	Soft Feces	.	.	.	.			

X=Present

AmplifyBio Study Number A-579-01

Individual Animal Clinical Signs

Group 2<br>Low<br>Total vg<br>6x10^13<br>Sex: Male	Observation Type: Routine	Day(s) Relative to Start Date						
		1	2	3	4	5	6	7
201	Normal	X	X	X	X	X	X	X
202	Normal	X	X	X	X	X	X	X
203	Normal	X	.	X	X	X	X	X
	Skin Abrasion, Forelimb	.	.	.	.	.	.	.
	Low Food Consumption	.	.	.	.	.	.	.
	Diarrhea	.	.	.	.	.	.	.
	Soft Feces	.	X	.	.	.	.	.
204	Normal	X	X	X	X	X	X	X
	Low Food Consumption	.	.	.	.	.	.	.
	Soft Feces	.	.	.	.	.	.	.

X=Present

AmplifyBio Study Number A-579-01

Individual Animal Clinical Signs

Group 2<br>Low<br>Total vg<br>6x10^13<br>Sex: Male	Observation Type: Routine	Day(s) Relative to Start Date						
		9	10	10<br>A	11	11<br>A	12	12<br>A
201	Normal	X	X	.	X	.	X	.
202	Normal	X	X	.	X	.	X	.
203	Normal	X	X	.	X	.	X	.
	Skin Abrasion, Forelimb	.	.	.	.	.	.	.
	Low Food Consumption	.	.	.	.	.	.	.
	Diarrhea	.	.	.	.	X	.	.
	Soft Feces	.	.	.	.	.	.	.
204	Normal	X	X	.	X	.	X	.
	Low Food Consumption	.	.	.	.	.	.	.
	Soft Feces	.	.	.	.	.	.	.

X=Present

AmplifyBio Study Number A-579-01

Individual Animal Clinical Signs

Group 2 Low Total vg 6x10^13 Sex: Male	Observation Type: Routine	Day(s) Relative to Start Date						
		13	13 A	14	16	17	18	19
201	Normal	X	.	X	X	X	X	X
202	Normal	X	.	X	X	X	X	X
203	Normal	X	.	X	X	X	X	X
	Skin Abrasion, Forelimb	.	.	.	.	.	.	.
	Low Food Consumption	.	.	.	.	.	.	.
	Diarrhea	.	.	.	.	.	.	.
	Soft Feces	.	.	.	.	.	.	.
204	Normal	X	.	X	X	X	X	X
	Low Food Consumption	.	.	.	.	.	.	.
	Soft Feces	.	.	.	.	.	.	.

X=Present

AmplifyBio Study Number A-579-01

Individual Animal Clinical Signs

Group 2<br>Low<br>Total vg<br>6x10^13<br>Sex: Male	Observation Type: Routine	Day(s) Relative to Start Date						
		20	21	21<br>A	23	24	25	26
201	Normal	X	X	.	X	X	X	X
202	Normal	X	X	.	X	X	X	X
203 !	Normal	X	X	.	.	.	.	.
	Skin Abrasion, Forelimb	.	.	.	LT	LT	LT	LT
	Low Food Consumption	.	.	.	.	.	.	.
	Diarrhea	.	.	.	.	.	.	.
	Soft Feces	.	.	.	.	.	.	.
204	Normal	X	X	.	X	X	X	X
	Low Food Consumption	.	.	.	.	.	.	.
	Soft Feces	.	.	.	.	.	.	.

!=Result comment recorded against 1 or more clinical observations. X=Present; LT=Left

AmplifyBio Study Number A-579-01

Individual Animal Clinical Signs

Group 2<br>Low<br>Total vg<br>6x10^13<br>Sex: Male	Observation Type: Routine	Day(s) Relative to Start Date						
		27	28	30	31	32	33	34
201	Normal	X	X	X	X	X	X	X
202	Normal	X	X	X	X	X	X	X
203	Normal	X	X	X	X	X	X	X
	Skin Abrasion, Forelimb	.	.	.	.	.	.	.
	Low Food Consumption	.	.	.	.	.	.	.
	Diarrhea	.	.	.	.	.	.	.
	Soft Feces	.	.	.	.	.	.	.
204	Normal	X	X	X	X	X	X	X
	Low Food Consumption	.	.	.	.	.	.	.
	Soft Feces	.	.	.	.	.	.	.

X=Present

AmplifyBio Study Number A-579-01

Individual Animal Clinical Signs

Group 2 Low Total vg 6x10^13 Sex: Male	Observation Type: Routine	Day(s) Relative to Start Date						
		35	37	37 A	38	39	40	41
201	Normal	X	X	.	X	X	X	X
202	Normal	X	X	.	X	X	X	X
203	Normal	X	X	.	X	X	X	X
	Skin Abrasion, Forelimb	.	.	.	.	.	.	.
	Low Food Consumption	.	.	.	.	.	.	.
	Diarrhea	.	.	.	.	.	.	.
	Soft Feces	.	.	.	.	.	.	.
204	Normal	X	X	.	X	X	X	X
	Low Food Consumption	.	.	.	.	.	.	.
	Soft Feces	.	.	.	.	.	.	.

X=Present

AmplifyBio Study Number A-579-01

Individual Animal Clinical Signs

Group 2 Low Total vg 6x10^13 Sex: Male	Observation Type: Routine	Day(s) Relative to Start Date						
		42	44	45	45 A	46	47	48
201	Normal	X	.	.	.	.	.	.
202	Normal	X	.	.	.	.	.	.
203	Normal	X	X	X	.	X	.	X
	Skin Abrasion, Forelimb	.	.	.	.	.	.	.
	Low Food Consumption	.	.	.	.	.	.	.
	Diarrhea	.	.	.	.	.	.	.
	Soft Feces	.	.	.	.	.	X	.
204	Normal	X	X	X	.	X	.	X
	Low Food Consumption	.	.	.	.	.	.	.
	Soft Feces	.	.	.	.	.	X	.

X=Present

AmplifyBio Study Number A-579-01

Individual Animal Clinical Signs

Group 2 Low Total vg 6x10^13 Sex: Male	Observation Type: Routine	Day(s) Relative to Start Date						
		49	51	51 A	52	53	54	55
201	Normal	.	.	.	.	.	.	.
202	Normal	.	.	.	.	.	.	.
203	Normal	X	X	.	X	X	X	X
	Skin Abrasion, Forelimb	.	.	.	.	.	.	.
	Low Food Consumption	.	.	.	.	.	.	.
	Diarrhea	.	.	.	.	.	.	.
	Soft Feces	.	.	.	.	.	.	.
204	Normal	X	X	.	X	X	X	X
	Low Food Consumption	.	.	.	.	.	.	.
	Soft Feces	.	.	.	.	.	.	.

X=Present

AmplifyBio Study Number A-579-01

Individual Animal Clinical Signs

Group 2<br>Low<br>Total vg<br>6x10^13<br>Sex: Male	Observation Type: Routine	Day(s) Relative to Start Date<br>56	 58	 59	 60	 60<br>A	 61	 61<br>A
201	Normal	.	.	.	.	.	.	.
202	Normal	.	.	.	.	.	.	.
203	Normal	X	X	X	X	.	X	.
	Skin Abrasion, Forelimb	.	.	.	.	.	.	.
	Low Food Consumption	.	.	.	.	.	.	.
	Diarrhea	.	.	.	.	.	.	.
	Soft Feces	.	.	.	.	.	.	.
204	Normal	X	X	X	X	.	X	.
	Low Food Consumption	.	.	.	.	.	.	.
	Soft Feces	.	.	.	.	.	.	.

X=Present

AmplifyBio Study Number A-579-01

Individual Animal Clinical Signs

Group 2<br>Low<br>Total vg<br>6x10^13<br>Sex: Male	Observation Type: Routine	Day(s) Relative to Start Date						
		62	63	65	66	67	68	69
201	Normal	.	.	.	.	.	.	.
202	Normal	.	.	.	.	.	.	.
203	Normal	X	X	X	X	X	X	X
	Skin Abrasion, Forelimb	.	.	.	.	.	.	.
	Low Food Consumption	.	.	.	.	.	.	.
	Diarrhea	.	.	.	.	.	.	.
	Soft Feces	.	.	.	.	.	.	.
204	Normal	X	X	X	X	X	X	X
	Low Food Consumption	.	.	.	.	.	.	.
	Soft Feces	.	.	.	.	.	.	.

X=Present

AmplifyBio Study Number A-579-01

Individual Animal Clinical Signs

Group 2<br>Low<br>Total vg<br>6x10^13<br>Sex: Male	Observation Type: Routine	Day(s) Relative to Start Date						
		70	72	73	73<br>A	74	74<br>A	75
201	Normal	.	.	.	.	.	.	.
202	Normal	.	.	.	.	.	.	.
203	Normal	X	.	X	.	.	.	X
	Skin Abrasion, Forelimb	.	.	.	.	.	.	.
	Low Food Consumption	.	.	.	RFC	RFC	.	.
	Diarrhea	.	.	.	X	.	.	.
	Soft Feces	.	X	.	.	.	.	.
204	Normal	X	.	X	.	.	.	X
	Low Food Consumption	.	.	.	.	RFC	.	.
	Soft Feces	.	X	.	.	.	.	.

X=Present; RFC=1/2 or Less of Total Food

AmplifyBio Study Number A-579-01

Individual Animal Clinical Signs

Group 2<br>Low<br>Total vg<br>6x10^13<br>Sex: Male	Observation Type: Routine	Day(s) Relative to Start Date						
		76	77	77<br>A	78<br>A	79	80	80<br>A
201	Normal	.	.	.	.	.	.	.
202	Normal	.	.	.	.	.	.	.
203	Normal	X	X	.	.	.	.	.
	Skin Abrasion, Forelimb	.	.	.	.	.	.	.
	Low Food Consumption	.	.	.	.	.	.	.
	Diarrhea	.	.	.	X	X	.	.
	Soft Feces	.	.	.	.	.	X	.
204	Normal	X	X	.	.	X	.	X
	Low Food Consumption	.	.	.	.	.	.	.
	Soft Feces	.	.	.	.	.	X	.

X=Present

AmplifyBio Study Number A-579-01

Individual Animal Clinical Signs

Group 2<br>Low<br>Total vg<br>6x10^13<br>Sex: Male	Observation Type: Routine	Day(s) Relative to Start Date<br>81	82	83	84			
201	Normal	.	.	.	.			
202	Normal	.	.	.	.			
203	Normal	.	.	.	.			
	Skin Abrasion, Forelimb	.	.	.	.			
	Low Food Consumption	.	.	.	.			
	Diarrhea	X	X	X	.			
	Soft Feces	.	.	.	X			
204	Normal	X	X	X	X			
	Low Food Consumption	.	.	.	.			
	Soft Feces	.	.	.	.			

X=Present

12/16/2023 6:41:35PM Page: 27

AmplifyBio Study Number A-579-01

Individual Animal Clinical Signs

Group 3<br>High<br>Total vg<br>1.2x10^14<br>Sex: Male	Observation Type: Routine	Day(s) Relative to Start Date						
		1	2	3	4	5	6	7
301	Normal	X	X	X	X	X	.	X
	Diarrhea	.	.	.	.	.	X	.
302	Normal	X	.	X	X	X	.	X
	Diarrhea	.	X	.	.	.	X	.
	Soft Feces	.	.	.	.	.	.	.
303	Normal	X	X	X	X	X	X	X
	Soft Feces	.	.	.	.	.	.	.
304	Normal	X	X	X	X	X	X	X
	Soft Feces	.	.	.	.	.	.	.

X=Present

AmplifyBio Study Number A-579-01

Individual Animal Clinical Signs

Group 3<br>High<br>Total vg<br>1.2x10^14<br>Sex: Male	Observation Type: Routine	Day(s) Relative to Start Date						
		9	10	10<br>A	11	11<br>A	12	12<br>A
301	Normal	X	.	.	X	.	X	.
	Diarrhea	.	X	X	.	.	.	.
302	Normal	X	.	.	X	.	X	.
	Diarrhea	.	X	X	.	.	.	.
	Soft Feces	.	.	.	.	.	.	.
303	Normal	X	X	.	X	.	X	.
	Soft Feces	.	.	.	.	.	.	.
304	Normal	X	X	.	X	.	X	.
	Soft Feces	.	.	.	.	.	.	.

X=Present

AmplifyBio Study Number A-579-01

Individual Animal Clinical Signs

Group 3 High Total vg 1.2x10^14 Sex: Male	Observation Type: Routine	Day(s) Relative to Start Date						
		13	13 A	14	16	17	18	19
301	Normal	.	X	X	X	X	X	X
	Diarrhea	X	.	.	.	.	.	.
302	Normal	.	.	X	X	X	X	X
	Diarrhea	X	X	.	.	.	.	.
	Soft Feces	.	.	.	.	.	.	.
303	Normal	X	.	X	X	X	X	X
	Soft Feces	.	.	.	.	.	.	.
304	Normal	X	.	X	X	X	X	X
	Soft Feces	.	.	.	.	.	.	.

X=Present

AmplifyBio Study Number A-579-01

Individual Animal Clinical Signs

Group 3 High Total vg 1.2x10^14 Sex: Male	Observation Type: Routine	Day(s) Relative to Start Date						
		20	21	21 A	23	24	25	26
301	Normal	X	X	.	X	X	X	X
	Diarrhea	.	.	.	.	.	.	.
302	Normal	X	X	.	.	.	.	.
	Diarrhea	.	.	X	X	X	X	X
	Soft Feces	.	.	.	.	.	.	.
303	Normal	X	X	.	X	X	X	X
	Soft Feces	.	.	.	.	.	.	.
304	Normal	X	X	.	X	X	X	X
	Soft Feces	.	.	.	.	.	.	.

X=Present

AmplifyBio Study Number A-579-01

Individual Animal Clinical Signs

Group 3<br>High<br>Total vg<br>1.2x10^14<br>Sex: Male	Observation Type: Routine	Day(s) Relative to Start Date						
		27	28	30	31	32	33	34
301	Normal	X	X	X	X	X	X	X
	Diarrhea	.	.	.	.	.	.	.
302	Normal	.	.	.	X	X	X	X
	Diarrhea	.	.	.	.	.	.	.
	Soft Feces	X	X	X	.	.	.	.
303	Normal	X	X	X	X	.	X	X
	Soft Feces	.	.	.	.	X	.	.
304	Normal	X	X	X	X	.	X	X
	Soft Feces	.	.	.	.	X	.	.

X=Present

AmplifyBio Study Number A-579-01

Individual Animal Clinical Signs

Group 3 High Total vg 1.2x10^14 Sex: Male	Observation Type: Routine	Day(s) Relative to Start Date						
		35	37	37 A	38	39	40	41
301	Normal	X	X	.	X	X	X	X
	Diarrhea	.	.	.	.	.	.	.
302	Normal	X	X	.	X	X	X	X
	Diarrhea	.	.	.	.	.	.	.
	Soft Feces	.	.	.	.	.	.	.
303	Normal	X	X	.	X	X	X	X
	Soft Feces	.	.	.	.	.	.	.
304	Normal	X	X	.	X	X	X	X
	Soft Feces	.	.	.	.	.	.	.

X=Present

AmplifyBio Study Number A-579-01

Individual Animal Clinical Signs

Group 3 High Total vg 1.2x10^14 Sex: Male	Observation Type: Routine	Day(s) Relative to Start Date						
		42	44	45	45 A	46	47	48
301	Normal	X	.	.	.	.	.	.
	Diarrhea	.	.	.	.	.	.	.
302	Normal	X	.	.	.	.	.	.
	Diarrhea	.	.	.	.	.	.	.
	Soft Feces	.	.	.	.	.	.	.
303	Normal	X	X	X	.	X	X	X
	Soft Feces	.	.	.	.	.	.	.
304	Normal	X	X	X	.	X	X	X
	Soft Feces	.	.	.	.	.	.	.

X=Present

AmplifyBio Study Number A-579-01

Individual Animal Clinical Signs

Group 3<br>High<br>Total vg<br>1.2x10^14<br>Sex: Male	Observation Type: Routine	Day(s) Relative to Start Date						
		49	51	51<br>A	52	53	54	55
301	Normal	.	.	.	.	.	.	.
	Diarrhea	.	.	.	.	.	.	.
302	Normal	.	.	.	.	.	.	.
	Diarrhea	.	.	.	.	.	.	.
	Soft Feces	.	.	.	.	.	.	.
303	Normal	X	X	.	X	X	X	X
	Soft Feces	.	.	.	.	.	.	.
304	Normal	X	X	.	X	X	X	X
	Soft Feces	.	.	.	.	.	.	.

X=Present

AmplifyBio Study Number A-579-01

Individual Animal Clinical Signs

Group 3 High Total vg 1.2x10^14 Sex: Male	Observation Type: Routine	Day(s) Relative to Start Date						
		56	58	59	60	60 A	61	61 A
301	Normal	.	.	.	.	.	.	.
	Diarrhea	.	.	.	.	.	.	.
302	Normal	.	.	.	.	.	.	.
	Diarrhea	.	.	.	.	.	.	.
	Soft Feces	.	.	.	.	.	.	.
303	Normal	X	X	X	X	.	X	.
	Soft Feces	.	.	.	.	.	.	.
304	Normal	X	X	X	X	.	X	.
	Soft Feces	.	.	.	.	.	.	.

X=Present

AmplifyBio Study Number A-579-01

Individual Animal Clinical Signs

Group 3 High Total vg 1.2x10^14 Sex: Male	Observation Type: Routine	Day(s) Relative to Start Date						
		62	63	65	66	67	68	69
301	Normal	.	.	.	.	.	.	.
	Diarrhea	.	.	.	.	.	.	.
302	Normal	.	.	.	.	.	.	.
	Diarrhea	.	.	.	.	.	.	.
	Soft Feces	.	.	.	.	.	.	.
303	Normal	X	X	X	X	X	X	X
	Soft Feces	.	.	.	.	.	.	.
304	Normal	X	X	X	X	X	X	X
	Soft Feces	.	.	.	.	.	.	.

X=Present

12/16/2023 6:41:35PM Page: 37

AmplifyBio Study Number A-579-01

Individual Animal Clinical Signs

Group 3<br>High<br>Total vg<br>1.2x10^14<br>Sex: Male	Observation Type: Routine	Day(s) Relative to Start Date						
		70	72	73	73<br>A	74	74<br>A	75
301	Normal	.	.	.	.	.	.	.
	Diarrhea	.	.	.	.	.	.	.
302	Normal	.	.	.	.	.	.	.
	Diarrhea	.	.	.	.	.	.	.
	Soft Feces	.	.	.	.	.	.	.
303	Normal	X	X	X	.	X	.	X
	Soft Feces	.	.	.	.	.	.	.
304	Normal	X	X	X	.	X	.	X
	Soft Feces	.	.	.	.	.	.	.

X=Present

AmplifyBio Study Number A-579-01

Individual Animal Clinical Signs

Group 3 High Total vg 1.2x10^14 Sex: Male	Observation Type: Routine	Day(s) Relative to Start Date						
		76	77	77 A	78 A	79	80	80 A
301	Normal	.	.	.	.	.	.	.
	Diarrhea	.	.	.	.	.	.	.
302	Normal	.	.	.	.	.	.	.
	Diarrhea	.	.	.	.	.	.	.
	Soft Feces	.	.	.	.	.	.	.
303	Normal	X	X	.	.	X	X	.
	Soft Feces	.	.	.	.	.	.	.
304	Normal	X	X	.	.	X	X	.
	Soft Feces	.	.	.	.	.	.	.

X=Present

AmplifyBio Study Number A-579-01

Individual Animal Clinical Signs

Group 3 High Total vg 1.2x10^14 Sex: Male	Observation Type: Routine	Day(s) Relative to Start Date						
		81	82	83	84			
301	Normal	.	.	.	.			
	Diarrhea	.	.	.	.			
302	Normal	.	.	.	.			
	Diarrhea	.	.	.	.			
	Soft Feces	.	.	.	.			
303	Normal	X	X	X	X			
	Soft Feces	.	.	.	.			
304	Normal	X	X	X	X			
	Soft Feces	.	.	.	.			

X=Present

AmplifyBio Study Number A-579-01

Individual Animal Clinical Signs

Group 1<br>Control<br>Total vg<br>0<br>Sex: Female	Observation Type: Routine	Day(s) Relative to Start Date						
		1	2	3	4	5	6	7
151	Normal	X	X	X	X	X	X	X
	Shivering	.	.	.	.	.	.	.
152	Normal	X	X	X	X	X	X	X
	Skin Abrasion, Face	.	.	.	.	.	.	.
	Skin Abrasion, Forelimb	.	.	.	.	.	.	.
	Skin Abrasion, Fore Paw	.	.	.	.	.	.	.
	Skin Alopecia, Body Dorsal	.	.	.	.	.	.	.
	Discharge, Vagina	.	.	.	.	.	.	.

X=Present

AmplifyBio Study Number A-579-01

Individual Animal Clinical Signs

Group 1<br>Control<br>Total vg<br>0<br>Sex: Female	Observation Type: Routine	Day(s) Relative to Start Date<br>9	10	10<br>A	11	11<br>A	12	12<br>A
151	Normal	X	X	.	X	.	.	X
	Shivering	.	.	.	.	.	X	.
152	Normal	X	X	.	X	.	X	.
	Skin Abrasion, Face	.	.	.	.	.	.	.
	Skin Abrasion, Forelimb	.	.	.	.	.	.	.
	Skin Abrasion, Fore Paw	.	.	.	.	.	.	.
	Skin Alopecia, Body Dorsal	.	.	.	.	.	.	.
	Discharge, Vagina	.	.	.	.	.	.	.

X=Present

AmplifyBio Study Number A-579-01

Individual Animal Clinical Signs

Group 1<br>Control<br>Total vg<br>0<br>Sex: Female	Observation Type: Routine	Day(s) Relative to Start Date						
		13	13<br>A	14	16	17	18	19
151	Normal	X	.	X	X	X	X	X
	Shivering	.	.	.	.	.	.	.
152	Normal	X	.	X	X	X	X	X
	Skin Abrasion, Face	.	.	.	.	.	.	.
	Skin Abrasion, Forelimb	.	.	.	.	.	.	.
	Skin Abrasion, Fore Paw	.	.	.	.	.	.	.
	Skin Alopecia, Body Dorsal	.	.	.	.	.	.	.
	Discharge, Vagina	.	.	.	.	.	.	.

X=Present

12/16/2023 6:41:35PM Page: 43

AmplifyBio Study Number A-579-01

Individual Animal Clinical Signs

Group 1<br>Control<br>Total vg<br>0<br>Sex: Female	Observation Type: Routine	Day(s) Relative to Start Date						
		20	21	21<br>A	23	24	25	26
151	Normal	X	X	.	X	X	X	X
	Shivering	.	.	.	.	.	.	.
152	Normal	X	X	.	X	X	X	X
	Skin Abrasion, Face	.	.	.	.	.	.	.
	Skin Abrasion, Forelimb	.	.	.	.	.	.	.
	Skin Abrasion, Fore Paw	.	.	.	.	.	.	.
	Skin Alopecia, Body Dorsal	.	.	.	.	.	.	.
	Discharge, Vagina	.	.	.	.	.	.	.

X=Present

AmplifyBio Study Number A-579-01

Individual Animal Clinical Signs

Group 1<br>Control<br>Total vg<br>0<br>Sex: Female	Observation Type: Routine	Day(s) Relative to Start Date						
		27	28	30	31	32	33	34
151	Normal	X	X	X	X	X	X	X
	Shivering	.	.	.	.	.	.	.
152	Normal	X	X	X	X	X	X	X
	Skin Abrasion, Face	.	.	.	.	.	.	.
	Skin Abrasion, Forelimb	.	.	.	.	.	.	.
	Skin Abrasion, Fore Paw	.	.	.	.	.	.	.
	Skin Alopecia, Body Dorsal	.	.	.	.	.	.	.
	Discharge, Vagina	.	.	.	.	.	.	.

X=Present

AmplifyBio Study Number A-579-01

Individual Animal Clinical Signs

Group 1<br>Control<br>Total vg<br>0<br>Sex: Female	Observation Type: Routine	Day(s) Relative to Start Date						
		35	37	37<br>A	38	39	40	41
151	Normal	X	X	.	X	X	X	X
	Shivering	.	.	.	.	.	.	.
152	Normal	X	X	.	X	X	X	X
	Skin Abrasion, Face	.	.	.	.	.	.	.
	Skin Abrasion, Forelimb	.	.	.	.	.	.	.
	Skin Abrasion, Fore Paw	.	.	.	.	.	.	.
	Skin Alopecia, Body Dorsal	.	.	.	.	.	.	.
	Discharge, Vagina	.	.	.	.	.	.	.

X=Present

AmplifyBio Study Number A-579-01

Individual Animal Clinical Signs

Group 1<br>Control<br>Total vg<br>0<br>Sex: Female	Observation Type: Routine	Day(s) Relative to Start Date<br>42	44	45	45<br>A	46	47	48
151	Normal	X	.	.	.	.	.	.
	Shivering	.	.	.	.	.	.	.
152	Normal	X	X	X	.	X	X	X
	Skin Abrasion, Face	.	.	.	.	.	.	.
	Skin Abrasion, Forelimb	.	.	.	.	.	.	.
	Skin Abrasion, Fore Paw	.	.	.	.	.	.	.
	Skin Alopecia, Body Dorsal	.	.	.	.	.	.	.
	Discharge, Vagina	.	.	.	.	.	.	.

X=Present

AmplifyBio Study Number A-579-01

Individual Animal Clinical Signs

Group 1<br>Control<br>Total vg<br>0<br>Sex: Female	Observation Type: Routine	Day(s) Relative to Start Date						
		49	51	51<br>A	52	53	54	55
151	Normal	.	.	.	.	.	.	.
	Shivering	.	.	.	.	.	.	.
152	Normal	X	X	.	X	X	X	X
	Skin Abrasion, Face	.	.	.	.	.	.	.
	Skin Abrasion, Forelimb	.	.	.	.	.	.	.
	Skin Abrasion, Fore Paw	.	.	.	.	.	.	.
	Skin Alopecia, Body Dorsal	.	.	.	.	.	.	.
	Discharge, Vagina	.	.	.	.	.	.	.

X=Present

AmplifyBio Study Number A-579-01

Individual Animal Clinical Signs

Group 1 Control Total vg 0 Sex: Female	Observation Type: Routine	Day(s) Relative to Start Date						
		56	58	59	60	60 A	61	61 A
151	Normal	.	.	.	.	.	.	.
	Shivering	.	.	.	.	.	.	.
152	Normal	X	X	X	X	.	X	.
	Skin Abrasion, Face	.	.	.	.	.	.	.
	Skin Abrasion, Forelimb	.	.	.	.	.	.	.
	Skin Abrasion, Fore Paw	.	.	.	.	.	.	.
	Skin Alopecia, Body Dorsal	.	.	.	.	.	.	.
	Discharge, Vagina	.	.	.	.	.	.	Red

X=Present; Red=Red

AmplifyBio Study Number A-579-01

Individual Animal Clinical Signs

Group 1<br>Control<br>Total vg<br>0<br>Sex: Female	Observation Type: Routine	Day(s) Relative to Start Date 62	63	65	66	67	68	69
151	Normal	.	.	.	.	.	.	.
	Shivering	.	.	.	.	.	.	.
152 !	Normal	.	X	X	X	X	.	.
	Skin Abrasion, Face	.	.	.	.	.	BT	BT
	Skin Abrasion, Forelimb	.	.	.	.	.	.	.
	Skin Abrasion, Fore Paw	.	.	.	.	.	.	.
	Skin Alopecia, Body Dorsal	.	.	.	.	.	.	.
	Discharge, Vagina	Red	.	.	.	.	.	.

!=Result comment recorded against 1 or more clinical observations. X=Present; BT=Both; Red=Red

AmplifyBio Study Number A-579-01

Individual Animal Clinical Signs

Group 1<br>Control<br>Total vg<br>0<br>Sex: Female	Observation Type: Routine	Day(s) Relative to Start Date<br>70	 72	 73	 73<br>A	 74	 74<br>A	 75
151	Normal	.	.	.	.	.	.	.
	Shivering	.	.	.	.	.	.	.
152 !	Normal	.	.	.	.	X	.	X
	Skin Abrasion, Face	BT	BT	BT	.	.	.	.
	Skin Abrasion, Forelimb	.	.	.	.	.	.	.
	Skin Abrasion, Fore Paw	.	.	.	.	.	.	.
	Skin Alopecia, Body Dorsal	.	.	.	.	.	.	.
	Discharge, Vagina	.	.	.	.	.	.	.

!=Result comment recorded against 1 or more clinical observations. X=Present; BT=Both

AmplifyBio Study Number A-579-01

Individual Animal Clinical Signs

Group 1 Control Total vg 0 Sex: Female	Observation Type: Routine	Day(s) Relative to Start Date						
		76	77	77 A	78 A	79	80	80 A
151	Normal	.	.	.	.	.	.	.
	Shivering	.	.	.	.	.	.	.
152 !	Normal	X	X	.	.	.	.	.
	Skin Abrasion, Face	.	.	.	.	.	.	.
	Skin Abrasion, Forelimb	.	.	.	.	RT	RT	.
	Skin Abrasion, Fore Paw	.	.	LT	.	LT	LT	.
	Skin Alopecia, Body Dorsal	.	.	.	.	.	.	.
	Discharge, Vagina	.	.	.	.	.	.	.

!=Result comment recorded against 1 or more clinical observations. X=Present; RT=Right; LT=Left

AmplifyBio Study Number A-579-01

Individual Animal Clinical Signs

Group 1<br>Control<br>Total vg<br>0<br>Sex: Female	Observation Type: Routine	Day(s) Relative to Start Date						
		81	82	83	84			
151	Normal	.	.	.	.			
	Shivering	.	.	.	.			
152 !	Normal	.	.	.	.			
	Skin Abrasion, Face	.	.	.	.			
	Skin Abrasion, Forelimb	RT	.	.	.			
	Skin Abrasion, Fore Paw	LT	.	.	.			
	Skin Alopecia, Body Dorsal	.	X	X	X			
	Discharge, Vagina	.	.	.	.			

!=Result comment recorded against 1 or more clinical observations. RT=Right; LT=Left; X=Present

AmplifyBio Study Number A-579-01

Individual Animal Clinical Signs

Group 2<br>Low<br>Total vg<br>6x10^13<br>Sex: Female	Observation Type: Routine	Day(s) Relative to Start Date						
		1	2	3	4	5	6	7
251	Normal	X	X	X	X	X	X	X
252	Normal	X	X	X	X	X	X	X
253	Normal	X	X	X	X	X	X	X
	Discharge, Vagina	.	.	.	.	.	.	.
254	Normal	X	X	X	X	X	X	X
	Low Food Consumption	.	.	.	.	.	.	.

X=Present

AmplifyBio Study Number A-579-01

Individual Animal Clinical Signs

Group 2<br>Low<br>Total vg<br>6x10^13<br>Sex: Female	Observation Type: Routine	Day(s) Relative to Start Date						
		9	10	10<br>A	11	11<br>A	12	12<br>A
251	Normal	X	X	.	X	.	X	.
252	Normal	X	X	.	X	.	X	.
253	Normal	X	X	.	X	.	X	.
	Discharge, Vagina	.	.	.	.	.	.	.
254	Normal	X	X	.	X	.	X	.
	Low Food Consumption	.	.	.	.	.	.	.

X=Present

AmplifyBio Study Number A-579-01

Individual Animal Clinical Signs

Group 2<br>Low<br>Total vg<br>6x10^13<br>Sex: Female	Observation Type: Routine	Day(s) Relative to Start Date						
		13	13<br>A	14	16	17	18	19
251	Normal	X	.	X	X	X	X	X
252	Normal	X	.	X	X	X	X	X
253	Normal	X	.	X	X	X	X	X
	Discharge, Vagina	.	.	.	.	.	.	.
254	Normal	X	.	X	X	X	X	X
	Low Food Consumption	.	.	.	.	.	.	.

X=Present

AmplifyBio Study Number A-579-01

Individual Animal Clinical Signs

Group 2<br>Low<br>Total vg<br>6x10^13<br>Sex: Female	Observation Type: Routine	Day(s) Relative to Start Date						
		20	21	21<br>A	23	24	25	26
251	Normal	X	X	.	X	X	X	X
252	Normal	X	X	.	X	X	X	X
253	Normal	X	X	.	X	X	X	X
	Discharge, Vagina	.	.	.	.	.	.	.
254	Normal	X	X	.	X	X	X	X
	Low Food Consumption	.	.	.	.	.	.	.

X=Present

AmplifyBio Study Number A-579-01

Individual Animal Clinical Signs

Group 2<br>Low<br>Total vg<br>6x10^13<br>Sex: Female	Observation Type: Routine	Day(s) Relative to Start Date						
		27	28	30	31	32	33	34
251	Normal	X	X	X	X	X	X	X
252	Normal	X	X	X	X	X	X	X
253	Normal	X	X	X	X	X	X	X
	Discharge, Vagina	.	.	.	.	.	.	.
254	Normal	X	X	X	X	X	X	X
	Low Food Consumption	.	.	.	.	.	.	.

X=Present

AmplifyBio Study Number A-579-01

Individual Animal Clinical Signs

Group 2<br>Low<br>Total vg<br>6x10^13<br>Sex: Female	Observation Type: Routine	Day(s) Relative to Start Date						
		35	37	37<br>A	38	39	40	41
251	Normal	X	X	.	X	X	X	X
252	Normal	X	X	.	X	X	X	X
253	Normal	X	X	.	X	X	X	X
	Discharge, Vagina	.	.	.	.	.	.	.
254	Normal	X	X	.	X	X	X	X
	Low Food Consumption	.	.	.	.	.	.	.

X=Present

AmplifyBio Study Number A-579-01

Individual Animal Clinical Signs

Group 2<br>Low<br>Total vg<br>6x10^13<br>Sex: Female	Observation Type: Routine	Day(s) Relative to Start Date						
		42	44	45	45<br>A	46	47	48
251	Normal	X	.	.	.	.	.	.
252	Normal	X	.	.	.	.	.	.
253	Normal	X	X	X	.	X	X	X
	Discharge, Vagina	.	.	.	.	.	.	.
254	Normal	X	X	X	.	X	X	X
	Low Food Consumption	.	.	.	.	.	.	.

X=Present

AmplifyBio Study Number A-579-01

Individual Animal Clinical Signs

Group 2<br>Low<br>Total vg<br>6x10^13<br>Sex: Female	Observation Type: Routine	Day(s) Relative to Start Date						
		49	51	51<br>A	52	53	54	55
251	Normal	.	.	.	.	.	.	.
252	Normal	.	.	.	.	.	.	.
253	Normal	X	X	.	X	X	X	X
	Discharge, Vagina	.	.	.	.	.	.	.
254	Normal	X	X	.	X	X	X	X
	Low Food Consumption	.	.	.	.	.	.	.

X=Present

AmplifyBio Study Number A-579-01

Individual Animal Clinical Signs

Group 2<br>Low<br>Total vg<br>6x10^13<br>Sex: Female	Observation Type: Routine	Day(s) Relative to Start Date						
		56	58	59	60	60<br>A	61	61<br>A
251	Normal	.	.	.	.	.	.	.
252	Normal	.	.	.	.	.	.	.
253	Normal	X	X	X	X	.	.	.
	Discharge, Vagina	.	.	.	.	Red	Red	.
254	Normal	X	X	X	X	.	X	.
	Low Food Consumption	.	.	.	.	.	.	.

X=Present; Red=Red

AmplifyBio Study Number A-579-01

Individual Animal Clinical Signs

Group 2<br>Low<br>Total vg<br>6x10^13<br>Sex: Female	Observation Type: Routine	Day(s) Relative to Start Date						
		62	63	65	66	67	68	69
251	Normal	.	.	.	.	.	.	.
252	Normal	.	.	.	.	.	.	.
253	Normal	X	X	X	X	X	X	X
	Discharge, Vagina	.	.	.	.	.	.	.
254	Normal	X	X	X	X	X	X	X
	Low Food Consumption	.	.	.	.	.	.	.

X=Present

AmplifyBio Study Number A-579-01

Individual Animal Clinical Signs

Group 2<br>Low<br>Total vg<br>6x10^13<br>Sex: Female	Observation Type: Routine	Day(s) Relative to Start Date						
		70	72	73	73<br>A	74	74<br>A	75
251	Normal	.	.	.	.	.	.	.
252	Normal	.	.	.	.	.	.	.
253	Normal	X	X	X	.	X	.	X
	Discharge, Vagina	.	.	.	.	.	.	.
254	Normal	X	X	X	.	X	.	X
	Low Food Consumption	.	.	.	.	.	RFC	.

X=Present; RFC=1/2 or Less of Total Food

AmplifyBio Study Number A-579-01

Individual Animal Clinical Signs

Group 2<br>Low<br>Total vg<br>6x10^13<br>Sex: Female	Observation Type: Routine	Day(s) Relative to Start Date						
		76	77	77<br>A	78<br>A	79	80	80<br>A
251	Normal	.	.	.	.	.	.	.
252	Normal	.	.	.	.	.	.	.
253	Normal	X	X	.	.	X	X	.
	Discharge, Vagina	.	.	.	.	.	.	.
254	Normal	X	X	.	.	X	X	.
	Low Food Consumption	.	.	RFC	.	.	.	.

X=Present; RFC=1/2 or Less of Total Food

AmplifyBio Study Number A-579-01

Individual Animal Clinical Signs

Group 2<br>Low<br>Total vg<br>6x10^13<br>Sex: Female	Observation Type: Routine	Day(s) Relative to Start Date<br>81	82	83	84			
251	Normal	.	.	.	.			
252	Normal	.	.	.	.			
253	Normal	X	X	X	X			
	Discharge, Vagina	.	.	.	.			
254	Normal	X	X	X	X			
	Low Food Consumption	.	.	.	.			

X=Present

AmplifyBio Study Number A-579-01

Individual Animal Clinical Signs

Group 3 High Total vg 1.2x10^14 Sex: Female	Observation Type: Routine	Day(s) Relative to Start Date						
		1	2	3	4	5	6	7
351	Normal	X	X	X	X	X	X	X
	Discolored Skin, Face	.	.	.	.	.	.	.
	Skin Abrasion, Face	.	.	.	.	.	.	.
	Bruising, Face	.	.	.	.	.	.	.
352	Normal	.	.	.	.	.	.	.
	Skin Alopecia, Hind Limb	BT	BT	BT	BT	BT	BT	BT
353	Normal	X	.	.	.	X	X	X
	Low Food Consumption	.	.	.	.	.	.	.
	Discharge, Vagina	.	Red	Red	Red	.	.	.
	Diarrhea	.	.	.	.	.	.	.
	Soft Feces	.	.	X	X	.	.	.
354	Normal	.	.	.	.	.	.	.
	Skin Alopecia, Body Dorsal	BT	BT	BT	BT	BT	BT	BT
	Skin Alopecia, Head/Neck	X	X	X	X	X	X	X
	Skin Alopecia, Hind Limb	.	.	.	.	.	.	.
	Discharge, Vagina	.	.	.	.	.	.	.
	Diarrhea	.	.	.	.	.	.	.
	Soft Feces	.	.	X	X	.	.	.

X=Present; BT=Both; Red=Red

AmplifyBio Study Number A-579-01

Individual Animal Clinical Signs

Group 3<br>High<br>Total vg<br>1.2x10^14<br>Sex: Female	Observation Type: Routine	Day(s) Relative to Start Date<br>9	10	10<br>A	11	11<br>A	12	12<br>A
351	Normal	X	X	.	X	.	X	.
	Discolored Skin, Face	.	.	.	.	.	.	.
	Skin Abrasion, Face	.	.	.	.	.	.	.
	Bruising, Face	.	.	.	.	.	.	.
352	Normal	X	X	.	X	.	X	.
	Skin Alopecia, Hind Limb	.	.	.	.	.	.	.
353	Normal	X	X	.	.	.	X	.
	Low Food Consumption	.	.	.	.	.	.	.
	Discharge, Vagina	.	.	Red	Red	.	.	.
	Diarrhea	.	.	.	.	.	.	.
	Soft Feces	.	.	.	.	.	.	.
354	Normal	.	.	.	.	.	.	.
	Skin Alopecia, Body Dorsal	.	.	.	.	.	.	.
	Skin Alopecia, Head/Neck	.	.	.	.	.	.	.
	Skin Alopecia, Hind Limb	BT	BT	.	BT	.	BT	.
	Discharge, Vagina	.	.	.	.	.	.	.
	Diarrhea	.	.	.	.	.	.	.
	Soft Feces	.	.	.	.	.	.	.

X=Present; Red=Red; BT=Both

AmplifyBio Study Number A-579-01

Individual Animal Clinical Signs

Group 3<br>High<br>Total vg<br>1.2x10^14<br>Sex: Female	Observation Type: Routine	Day(s) Relative to Start Date						
		13	13<br>A	14	16	17	18	19
351	Normal	X	.	X	X	X	X	X
	Discolored Skin, Face	.	.	.	.	.	.	.
	Skin Abrasion, Face	.	.	.	.	.	.	.
	Bruising, Face	.	.	.	.	.	.	.
352	Normal	X	.	X	X	X	X	X
	Skin Alopecia, Hind Limb	.	.	.	.	.	.	.
353	Normal	X	.	X	X	X	X	X
	Low Food Consumption	.	.	.	.	.	.	.
	Discharge, Vagina	.	.	.	.	.	.	.
	Diarrhea	.	.	.	.	.	.	.
	Soft Feces	.	.	.	.	.	.	.
354	Normal	.	.	.	X	X	X	X
	Skin Alopecia, Body Dorsal	.	.	.	.	.	.	.
	Skin Alopecia, Head/Neck	.	.	.	.	.	.	.
	Skin Alopecia, Hind Limb	BT	.	BT	.	.	.	.
	Discharge, Vagina	.	.	.	.	.	.	.
	Diarrhea	.	.	.	.	.	.	.
	Soft Feces	.	.	.	.	.	.	.

X=Present; BT=Both

AmplifyBio Study Number A-579-01

Individual Animal Clinical Signs

Group 3 High Total vg 1.2x10^14 Sex: Female	Observation Type: Routine	Day(s) Relative to Start Date						
		20	21	21 A	23	24	25	26
351	Normal	X	X	.	X	X	X	X
	Discolored Skin, Face	.	.	.	.	.	.	.
	Skin Abrasion, Face	.	.	.	.	.	.	.
	Bruising, Face	.	.	.	.	.	.	.
352	Normal	X	X	.	X	X	X	X
	Skin Alopecia, Hind Limb	.	.	.	.	.	.	.
353	Normal	X	X	.	X	X	X	X
	Low Food Consumption	.	.	.	.	.	.	.
	Discharge, Vagina	.	.	.	.	.	.	.
	Diarrhea	.	.	.	.	.	.	.
	Soft Feces	.	.	.	.	.	.	.
354	Normal	X	X	.	X	X	X	X
	Skin Alopecia, Body Dorsal	.	.	.	.	.	.	.
	Skin Alopecia, Head/Neck	.	.	.	.	.	.	.
	Skin Alopecia, Hind Limb	.	.	.	.	.	.	.
	Discharge, Vagina	.	.	.	.	.	.	.
	Diarrhea	.	.	.	.	.	.	.
	Soft Feces	.	.	.	.	.	.	.

X=Present

AmplifyBio Study Number A-579-01

Individual Animal Clinical Signs

Group 3 High Total vg 1.2x10^14 Sex: Female	Observation Type: Routine	Day(s) Relative to Start Date						
		27	28	30	31	32	33	34
351	Normal	X	X	X	X	X	X	X
	Discolored Skin, Face	.	.	.	.	.	.	.
	Skin Abrasion, Face	.	.	.	.	.	.	.
	Bruising, Face	.	.	.	.	.	.	.
352	Normal	X	X	X	X	X	X	X
	Skin Alopecia, Hind Limb	.	.	.	.	.	.	.
353	Normal	X	X	X	X	X	X	X
	Low Food Consumption	.	.	.	.	.	.	.
	Discharge, Vagina	.	.	.	.	.	.	.
	Diarrhea	.	.	.	.	.	.	.
	Soft Feces	.	.	.	.	.	.	.
354	Normal	X	X	X	X	X	X	X
	Skin Alopecia, Body Dorsal	.	.	.	.	.	.	.
	Skin Alopecia, Head/Neck	.	.	.	.	.	.	.
	Skin Alopecia, Hind Limb	.	.	.	.	.	.	.
	Discharge, Vagina	.	.	.	.	.	.	.
	Diarrhea	.	.	.	.	.	.	.
	Soft Feces	.	.	.	.	.	.	.

X=Present

AmplifyBio Study Number A-579-01

Individual Animal Clinical Signs

Group 3 High Total vg 1.2x10^14 Sex: Female	Observation Type: Routine	Day(s) Relative to Start Date						
		35	37	37 A	38	39	40	41
351 !	Normal	X	X	.	.	.	.	.
	Discolored Skin, Face	.	.	Red	Red	Red	.	.
	Skin Abrasion, Face	.	.	.	RT	RT	.	.
	Bruising, Face	.	.	.	.	.	RT	RT
352	Normal	X	X	.	X	X	X	X
	Skin Alopecia, Hind Limb	.	.	.	.	.	.	.
353	Normal	X	X	.	X	X	X	X
	Low Food Consumption	.	.	.	.	.	.	.
	Discharge, Vagina	.	.	.	.	.	.	.
	Diarrhea	.	.	.	.	.	.	.
	Soft Feces	.	.	.	.	.	.	.
354	Normal	X	X	.	X	X	X	X
	Skin Alopecia, Body Dorsal	.	.	.	.	.	.	.
	Skin Alopecia, Head/Neck	.	.	.	.	.	.	.
	Skin Alopecia, Hind Limb	.	.	.	.	.	.	.
	Discharge, Vagina	.	.	.	.	.	.	.
	Diarrhea	.	.	.	.	.	.	.
	Soft Feces	.	.	.	.	.	.	.

!=Result comment recorded against 1 or more clinical observations. X=Present; Red=Red; RT=Right

AmplifyBio Study Number A-579-01

Individual Animal Clinical Signs

Group 3 High Total vg 1.2x10^14 Sex: Female	Observation Type: Routine	Day(s) Relative to Start Date						
		42	44	45	45 A	46	47	48
351	Normal	.	.	.	.	.	.	.
	Discolored Skin, Face	.	.	.	.	.	.	.
	Skin Abrasion, Face	.	.	.	.	.	.	.
	Bruising, Face	RT	.	.	.	.	.	.
352	Normal	X	.	.	.	.	.	.
	Skin Alopecia, Hind Limb	.	.	.	.	.	.	.
353	Normal	X	X	X	.	X	X	X
	Low Food Consumption	.	.	.	RFC	.	.	.
	Discharge, Vagina	.	.	.	.	.	.	.
	Diarrhea	.	.	.	.	.	.	.
	Soft Feces	.	.	.	.	.	.	.
354	Normal	X	X	X	.	X	X	X
	Skin Alopecia, Body Dorsal	.	.	.	.	.	.	.
	Skin Alopecia, Head/Neck	.	.	.	.	.	.	.
	Skin Alopecia, Hind Limb	.	.	.	.	.	.	.
	Discharge, Vagina	.	.	.	.	.	.	.
	Diarrhea	.	.	.	.	.	.	.
	Soft Feces	.	.	.	.	.	.	.

RT=Right; X=Present; RFC=1/2 or Less of Total Food

AmplifyBio Study Number A-579-01

Individual Animal Clinical Signs

Group 3 High Total vg 1.2x10^14 Sex: Female	Observation Type: Routine	Day(s) Relative to Start Date						
		49	51	51 A	52	53	54	55
351	Normal	.	.	.	.	.	.	.
	Discolored Skin, Face	.	.	.	.	.	.	.
	Skin Abrasion, Face	.	.	.	.	.	.	.
	Bruising, Face	.	.	.	.	.	.	.
352	Normal	.	.	.	.	.	.	.
	Skin Alopecia, Hind Limb	.	.	.	.	.	.	.
353	Normal	X	X	.	X	.	X	X
	Low Food Consumption	.	.	.	.	.	.	.
	Discharge, Vagina	.	.	.	.	.	.	.
	Diarrhea	.	.	.	.	X	.	.
	Soft Feces	.	.	.	.	.	.	.
354	Normal	X	X	.	X	.	X	X
	Skin Alopecia, Body Dorsal	.	.	.	.	.	.	.
	Skin Alopecia, Head/Neck	.	.	.	.	.	.	.
	Skin Alopecia, Hind Limb	.	.	.	.	.	.	.
	Discharge, Vagina	.	.	.	.	.	.	.
	Diarrhea	.	.	.	.	X	.	.
	Soft Feces	.	.	.	.	.	.	.

X=Present

AmplifyBio Study Number A-579-01

Individual Animal Clinical Signs

Group 3 High Total vg 1.2x10^14 Sex: Female	Observation Type: Routine	Day(s) Relative to Start Date 56	58	59	60	60 A	61	61 A
351	Normal	.	.	.	.	.	.	.
	Discolored Skin, Face	.	.	.	.	.	.	.
	Skin Abrasion, Face	.	.	.	.	.	.	.
	Bruising, Face	.	.	.	.	.	.	.
352	Normal	.	.	.	.	.	.	.
	Skin Alopecia, Hind Limb	.	.	.	.	.	.	.
353	Normal	X	X	X	X	.	X	.
	Low Food Consumption	.	.	.	.	.	.	.
	Discharge, Vagina	.	.	.	.	.	.	.
	Diarrhea	.	.	.	.	.	.	.
	Soft Feces	.	.	.	.	.	.	.
354	Normal	X	X	X	X	.	X	.
	Skin Alopecia, Body Dorsal	.	.	.	.	.	.	.
	Skin Alopecia, Head/Neck	.	.	.	.	.	.	.
	Skin Alopecia, Hind Limb	.	.	.	.	.	.	.
	Discharge, Vagina	.	.	.	.	.	.	.
	Diarrhea	.	.	.	.	.	.	.
	Soft Feces	.	.	.	.	.	.	.

X=Present

AmplifyBio Study Number A-579-01

Individual Animal Clinical Signs

Group 3<br>High<br>Total vg<br>1.2x10^14<br>Sex: Female	Observation Type: Routine	Day(s) Relative to Start Date						
		62	63	65	66	67	68	69
351	Normal	.	.	.	.	.	.	.
	Discolored Skin, Face	.	.	.	.	.	.	.
	Skin Abrasion, Face	.	.	.	.	.	.	.
	Bruising, Face	.	.	.	.	.	.	.
352	Normal	.	.	.	.	.	.	.
	Skin Alopecia, Hind Limb	.	.	.	.	.	.	.
353	Normal	X	X	X	X	X	X	X
	Low Food Consumption	.	.	.	.	.	.	.
	Discharge, Vagina	.	.	.	.	.	.	.
	Diarrhea	.	.	.	.	.	.	.
	Soft Feces	.	.	.	.	.	.	.
354	Normal	X	X	X	X	X	X	.
	Skin Alopecia, Body Dorsal	.	.	.	.	.	.	.
	Skin Alopecia, Head/Neck	.	.	.	.	.	.	.
	Skin Alopecia, Hind Limb	.	.	.	.	.	.	.
	Discharge, Vagina	.	.	.	.	.	.	Red
	Diarrhea	.	.	.	.	.	.	.
	Soft Feces	.	.	.	.	.	.	.

X=Present; Red=Red

AmplifyBio Study Number A-579-01

Individual Animal Clinical Signs

Group 3<br>High<br>Total vg<br>1.2x10^14<br>Sex: Female	Observation Type: Routine	Day(s) Relative to Start Date						
		70	72	73	73<br>A	74	74<br>A	75
351	Normal	.	.	.	.	.	.	.
	Discolored Skin, Face	.	.	.	.	.	.	.
	Skin Abrasion, Face	.	.	.	.	.	.	.
	Bruising, Face	.	.	.	.	.	.	.
352	Normal	.	.	.	.	.	.	.
	Skin Alopecia, Hind Limb	.	.	.	.	.	.	.
353 !	Normal	X	X	X	.	X	.	X
	Low Food Consumption	.	.	.	RFC	.	.	.
	Discharge, Vagina	.	.	.	.	.	.	.
	Diarrhea	.	.	.	.	.	.	.
	Soft Feces	.	.	.	.	.	.	.
354	Normal	.	X	X	.	X	.	X
	Skin Alopecia, Body Dorsal	.	.	.	.	.	.	.
	Skin Alopecia, Head/Neck	.	.	.	.	.	.	.
	Skin Alopecia, Hind Limb	.	.	.	.	.	.	.
	Discharge, Vagina	Red	.	.	.	.	.	.
	Diarrhea	.	.	.	.	.	.	.
	Soft Feces	.	.	.	.	.	.	.

!=Result comment recorded against 1 or more clinical observations. X=Present; RFC=1/2 or Less of Total Food; Red=Red

AmplifyBio Study Number A-579-01

Individual Animal Clinical Signs

Group 3 High Total vg 1.2x10^14 Sex: Female	Observation Type: Routine	Day(s) Relative to Start Date						
		76	77	77 A	78 A	79	80	80 A
351	Normal	.	.	.	.	.	.	.
	Discolored Skin, Face	.	.	.	.	.	.	.
	Skin Abrasion, Face	.	.	.	.	.	.	.
	Bruising, Face	.	.	.	.	.	.	.
352	Normal	.	.	.	.	.	.	.
	Skin Alopecia, Hind Limb	.	.	.	.	.	.	.
353	Normal	X	X	.	.	X	X	.
	Low Food Consumption	.	.	.	.	.	.	.
	Discharge, Vagina	.	.	.	.	.	.	.
	Diarrhea	.	.	.	.	.	.	.
	Soft Feces	.	.	.	.	.	.	.
354	Normal	X	X	.	.	X	X	.
	Skin Alopecia, Body Dorsal	.	.	.	.	.	.	.
	Skin Alopecia, Head/Neck	.	.	.	.	.	.	.
	Skin Alopecia, Hind Limb	.	.	.	.	.	.	.
	Discharge, Vagina	.	.	.	.	.	.	.
	Diarrhea	.	.	.	.	.	.	.
	Soft Feces	.	.	.	.	.	.	.

X=Present

AmplifyBio Study Number A-579-01

Individual Animal Clinical Signs

Group 3<br>High<br>Total vg<br>1.2x10^14<br>Sex: Female	Observation Type: Routine	Day(s) Relative to Start Date						
		81	82	83	84			
351	Normal	.	.	.	.			
	Discolored Skin, Face	.	.	.	.			
	Skin Abrasion, Face	.	.	.	.			
	Bruising, Face	.	.	.	.			
352	Normal	.	.	.	.			
	Skin Alopecia, Hind Limb	.	.	.	.			
353	Normal	X	X	X	.			
	Low Food Consumption	.	.	.	.			
	Discharge, Vagina	.	.	.	.			
	Diarrhea	.	.	.	X			
	Soft Feces	.	.	.	.			
354	Normal	X	X	X	.			
	Skin Alopecia, Body Dorsal	.	.	.	.			
	Skin Alopecia, Head/Neck	.	.	.	.			
	Skin Alopecia, Hind Limb	.	.	.	.			
	Discharge, Vagina	.	.	.	.			
	Diarrhea	.	.	.	X			
	Soft Feces	.	.	.	.			

X=Present

AmplifyBio Study Number A-579-01

Individual Animal Clinical Signs

Group 1<br>Control<br>Total vg<br>0<br>Sex: Male	Observation Type: Detailed Clinical Observations	Day(s) Relative to Start Date						
		1	8	15	22	29	36	43
101	Normal	X	X	.	X	X	X	X
	Skin Alopecia, Head/Neck	.	.	BT	.	.	.	.
102	Normal	X	X	.	X	X	X	X
	Skin Abrasion, Fore Paw	.	.	.	.	.	.	.
	Skin Alopecia, Head/Neck	.	.	BT	.	.	.	.

X=Present; BT=Both

AmplifyBio Study Number A-579-01

Individual Animal Clinical Signs

Group 1<br>Control<br>Total vg<br>0<br>Sex: Male	Observation Type: Detailed Clinical Observations	Day(s) Relative to Start Date						
		50	57	64	71	78	85	
101	Normal	.	.	.	.	.	.	
	Skin Alopecia, Head/Neck	.	.	.	.	.	.	
102 !	Normal	.	.	.	X	.	.	
	Skin Abrasion, Fore Paw	RT	RT	RT	.	RT	RT	
	Skin Alopecia, Head/Neck	.	.	.	.	.	.	

!=Result comment recorded against 1 or more clinical observations. X=Present; RT=Right

AmplifyBio Study Number A-579-01

Individual Animal Clinical Signs

Group 2<br>Low<br>Total vg<br>6x10^13<br>Sex: Male	Observation Type: Detailed Clinical Observations	Day(s) Relative to Start Date<br>1	8	15	22	29	36	43
201	Normal	X	X	X	.	.	X	X
	Discolored Skin, Head/Neck, Pale	.	.	.	X	.	.	.
	Discharge, Nose	.	.	.	CL	CL	.	.
	Open Mouth Breathing	.	.	.	X	.	.	.
202	Normal	X	X	X	X	X	X	X
203 !	Normal	X	X	X	.	X	X	X
	Skin Abrasion, Forelimb	.	.	.	LT	.	.	.
	Diarrhea	.	.	.	.	.	.	.
204	Normal	X	X	X	X	X	X	X

!=Result comment recorded against 1 or more clinical observations. X=Present; CL=Clear; LT=Left

AmplifyBio Study Number A-579-01

Individual Animal Clinical Signs

Group 2 Low Total vg 6x10^13 Sex: Male	Observation Type: Detailed Clinical Observations	Day(s) Relative to Start Date						
		50	57	64	71	78	85	
201	Normal	.	.	.	.	.	.	
	Discolored Skin, Head/Neck, Pale	.	.	.	.	.	.	
	Discharge, Nose	.	.	.	.	.	.	
	Open Mouth Breathing	.	.	.	.	.	.	
202	Normal	.	.	.	.	.	.	
203	Normal	X	X	X	X	X	.	
	Skin Abrasion, Forelimb	.	.	.	.	.	.	
	Diarrhea	.	.	.	.	.	X	
204	Normal	X	X	X	X	X	X	

X=Present

AmplifyBio Study Number A-579-01

Individual Animal Clinical Signs

Group 3<br>High<br>Total vg<br>1.2x10^14<br>Sex: Male	Observation Type: Detailed Clinical Observations	Day(s) Relative to Start Date<br>1	8	15	22	29	36	43
301	Normal	.	X	X	X	.	X	X
	Diarrhea	X	.	.	.	.	.	.
	Soft Feces	.	.	.	.	X	.	.
302	Normal	.	X	.	.	.	X	X
	Diarrhea	X	.	X	X	.	.	.
	Soft Feces	.	.	.	.	X	.	.
303	Normal	X	X	X	X	X	X	X
304	Normal	X	X	X	X	X	X	X

X=Present

AmplifyBio Study Number A-579-01

Individual Animal Clinical Signs

Group 3 High Total vg 1.2x10^14 Sex: Male	Observation Type: Detailed Clinical Observations	Day(s) Relative to Start Date						
		50	57	64	71	78	85	
301	Normal	.	.	.	.	.	.	
	Diarrhea	.	.	.	.	.	.	
	Soft Feces	.	.	.	.	.	.	
302	Normal	.	.	.	.	.	.	
	Diarrhea	.	.	.	.	.	.	
	Soft Feces	.	.	.	.	.	.	
303	Normal	X	X	X	X	X	X	
304	Normal	X	X	X	X	X	X	

X=Present

AmplifyBio Study Number A-579-01

Individual Animal Clinical Signs

Group 1<br>Control<br>Total vg<br>0<br>Sex: Female	Observation Type: Detailed Clinical Observations	Day(s) Relative to Start Date						
		1	8	15	22	29	36	43
151	Normal	X	X	.	X	X	X	X
	Discharge, Vagina	.	.	Red	.	.	.	.
152	Normal	X	X	X	.	X	.	X
	Skin Abrasion, Face	.	.	.	.	.	.	.
	Skin Abrasion, Forelimb	.	.	.	.	.	.	.
	Skin Abrasion, Hind Limb	.	.	.	.	.	.	.
	Skin Abrasion, Fore Paw	.	.	.	.	.	.	.
	Skin Alopecia, Body Dorsal	.	.	.	.	.	.	.
	Skin Alopecia, Forelimb	.	.	.	.	.	.	.
	Discharge, Vagina	.	.	.	Red	.	.	.
	Diarrhea	.	.	.	.	.	X	.

X=Present; Red=Red

AmplifyBio Study Number A-579-01

Individual Animal Clinical Signs

Group 1<br>Control<br>Total vg<br>0<br>Sex: Female	Observation Type: Detailed Clinical Observations	Day(s) Relative to Start Date						
		50	57	64	71	78	85	
151	Normal	.	.	.	.	.	.	
	Discharge, Vagina	.	.	.	.	.	.	
152	Normal	X	X	X	.	.	.	
	Skin Abrasion, Face	.	.	.	BT	.	.	
	Skin Abrasion, Forelimb	.	.	.	.	RT	.	
	Skin Abrasion, Hind Limb	.	.	.	.	LT	.	
	Skin Abrasion, Fore Paw	.	.	.	.	LT	.	
	Skin Alopecia, Body Dorsal	.	.	.	.	LT	LT	
	Skin Alopecia, Forelimb	.	.	.	.	BT	BT	
	Discharge, Vagina	.	.	.	.	.	.	
	Diarrhea	.	.	.	.	.	.	

X=Present; BT=Both; RT=Right; LT=Left

AmplifyBio Study Number A-579-01

Individual Animal Clinical Signs

Group 2<br>Low<br>Total vg<br>6x10^13<br>Sex: Female	Observation Type: Detailed Clinical Observations	Day(s) Relative to Start Date						
		1	8	15	22	29	36	43
251	Normal	X	X	X	X	X	X	X
252 !	Normal	X	.	.	X	X	X	X
	Skin Abrasion, Body Dorsal	.	RT	.	.	.	.	.
	Skin Abrasion, Tail	.	.	BT	.	.	.	.
253	Normal	X	X	X	X	X	X	X
254 !	Normal	.	X	X	.	X	X	X
	Scabbing, Body Dorsal	X	.	.	.	.	.	.
	Discharge, Vagina	.	.	.	Red	.	.	.

!=Result comment recorded against 1 or more clinical observations. X=Present; RT=Right; BT=Both; Red=Red

AmplifyBio Study Number A-579-01

Individual Animal Clinical Signs

Group 2<br>Low<br>Total vg<br>6x10^13<br>Sex: Female	Observation Type: Detailed Clinical Observations	Day(s) Relative to Start Date						
		50	57	64	71	78	85	
251	Normal	.	.	.	.	.	.	
252	Normal	.	.	.	.	.	.	
	Skin Abrasion, Body Dorsal	.	.	.	.	.	.	
	Skin Abrasion, Tail	.	.	.	.	.	.	
253	Normal	X	X	X	X	X	X	
254	Normal	X	X	X	X	X	X	
	Scabbing, Body Dorsal	.	.	.	.	.	.	
	Discharge, Vagina	.	.	.	.	.	.	

X=Present

AmplifyBio Study Number A-579-01

Individual Animal Clinical Signs

Group 3 High Total vg 1.2x10^14 Sex: Female	Observation Type: Detailed Clinical Observations	Day(s) Relative to Start Date						
		1	8	15	22	29	36	43
351 !	Normal	.	.	X	X	X	X	.
	Bruising, Face	.	.	.	.	.	.	RT
	Scabbing, Body Dorsal	X	X	.	.	.	.	.
352	Normal	X	X	X	X	.	X	X
	Discharge, Vagina	.	.	.	.	Red	.	.
353 !	Normal	.	X	X	X	X	X	.
	Skin Abrasion, Body Dorsal	.	.	.	.	.	.	X
	Discharge, Vagina	.	.	.	.	.	.	.
	Diarrhea	X	.	.	.	.	.	.
354	Normal	.	.	X	X	X	X	X
	Skin Alopecia, Body Dorsal	X	.	.	.	.	.	.
	Skin Alopecia, Head/Neck	X	.	.	.	.	.	.
	Skin Alopecia, Hind Limb	.	BT	.	.	.	.	.

!=Result comment recorded against 1 or more clinical observations. X=Present; RT=Right; Red=Red; BT=Both

AmplifyBio Study Number A-579-01

Individual Animal Clinical Signs

Group 3<br>High<br>Total vg<br>1.2x10^14<br>Sex: Female	Observation Type: Detailed Clinical Observations	Day(s) Relative to Start Date						
		50	57	64	71	78	85	
351	Normal	.	.	.	.	.	.	
	Bruising, Face	.	.	.	.	.	.	
	Scabbing, Body Dorsal	.	.	.	.	.	.	
352	Normal	.	.	.	.	.	.	
	Discharge, Vagina	.	.	.	.	.	.	
353 !	Normal	.	.	.	.	.	.	
	Skin Abrasion, Body Dorsal	X	X	X	X	X	X	
	Discharge, Vagina	.	.	Red	.	.	.	
	Diarrhea	.	.	.	.	.	.	
354	Normal	X	X	X	X	X	X	
	Skin Alopecia, Body Dorsal	.	.	.	.	.	.	
	Skin Alopecia, Head/Neck	.	.	.	.	.	.	
	Skin Alopecia, Hind Limb	.	.	.	.	.	.	

!=Result comment recorded against 1 or more clinical observations. X=Present; Red=Red

AmplifyBio Study Number A-579-01

Individual Animal Clinical Signs

Comment Information

Group	Sex	Animal	Day	Observation Type	Comment
Group 2	Male	203	23	Routine	second digit
Group 2	Male	203	24	Routine	second digit
Group 2	Male	203	25	Routine	second digit
Group 2	Male	203	26	Routine	second digit
Group 1	Female	152	68	Routine	upper lip
Group 1	Female	152	69	Routine	upper lip
Group 1	Female	152	70	Routine	upper lip
Group 1	Female	152	72	Routine	upper lip
Group 1	Female	152	73	Routine	upper lip
Group 1	Female	152	77 (A)	Routine	on top of hand
Group 1	Female	152	79	Routine	on top of hand (left forepaw)
Group 1	Female	152	80	Routine	on top of hand (left forepaw)
Group 1	Female	152	81	Routine	on top of hand (left forepaw)
Group 3	Female	351	38	Routine	abrasion over right eye
Group 3	Female	351	39	Routine	abrasion over right eye
Group 3	Female	353	73 (A)	Routine	Ate all EE
Group 1	Male	102	50	Detailed Clinical Observations	digit 4
Group 1	Male	102	57	Detailed Clinical Observations	digit 4
Group 1	Male	102	64	Detailed Clinical Observations	digit 4
Group 1	Male	102	78	Detailed Clinical Observations	Abrasion on digit 4
Group 1	Male	102	85	Detailed Clinical Observations	Abrasion on digit 4

AmplifyBio Study Number A-579-01

Individual Animal Clinical Signs

Comment Information (Continued)

Group	Sex	Animal	Day	Observation Type	Comment
Group 2	Male	203	22	Detailed Clinical Observations	digit 2
Group 2	Female	252	8	Detailed Clinical Observations	Skin abrasion on right hip
Group 2	Female	254	1	Detailed Clinical Observations	at incision site
Group 3	Female	351	1	Detailed Clinical Observations	at incision site
Group 3	Female	351	8	Detailed Clinical Observations	at incision site
Group 3	Female	353	43	Detailed Clinical Observations	on left ischial callosity
Group 3	Female	353	50	Detailed Clinical Observations	on left ischial callosity
Group 3	Female	353	57	Detailed Clinical Observations	on left ischial callosity
Group 3	Female	353	64	Detailed Clinical Observations	on left ischial callosity
Group 3	Female	353	71	Detailed Clinical Observations	on left ischial callosity
Group 3	Female	353	78	Detailed Clinical Observations	on left ischial callosity
Group 3	Female	353	85	Detailed Clinical Observations	on left ischial callosity

AmplifyBio Study Number A-579-01

Individual Animal Clinical Signs

Key Page

Group Information

Short Name	Long Name	Type	Report Headings			
1	Group 1	Control	Group 1	Control	Total vg	0
2	Group 2	Dose	Group 2	Low	Total vg	6x10^13
3	Group 3	Dose	Group 3	High	Total vg	1.2x10^14

Timeslot Definition

Abbreviation	Description
A	A - Unscheduled Observation

Table D-4. Individual Animal Body Weight and Weight Gain

AmplifyBio Study Number A-579-01

Individual Animal Body Weight & Weight Gain

Sex: Male Body Weight (kg)

Group 1 Control Total vg 0	Day(s) Relative to Start Date										
	1	8	15	22	29	36	43	50	57	64	71
101	2.37	2.28	2.46	2.38	2.44	2.51	2.41	-	-	-	-
102	2.87	2.82	2.97	2.90	3.05	3.14	3.03	3.09	3.13	3.16	3.31

AmplifyBio Study Number A-579-01

Individual Animal Body Weight & Weight Gain

Sex: Male Body Weight (kg)

Group 1 Control Total vg 0	Day(s) Relative to Start Date 78	85
101	-	-
102	3.38	3.31

AmplifyBio Study Number A-579-01

Individual Animal Body Weight & Weight Gain

Sex: Male Body Weight (kg)

Group 2 Low Total vg 6x10^13	Day(s) Relative to Start Date										
	1	8	15	22	29	36	43	50	57	64	71
201	2.50	2.38	2.54	2.46	2.58	2.62	2.44	-	-	-	-
202	2.97	2.85	3.02	2.98	3.11	3.23	3.06	-	-	-	-
203	2.88	2.86	2.96	2.95	3.07	3.20	3.11	3.14	3.28	3.29	3.35
204	2.25	2.20	2.35	2.33	2.33	2.45	2.35	2.41	2.47	2.53	2.59

AmplifyBio Study Number A-579-01

Individual Animal Body Weight & Weight Gain

Sex: Male Body Weight (kg)

Group 2 Low Total vg 6x10^13	Day(s) Relative to Start Date	
	78	85
201	-	-
202	-	-
203	3.18	2.97
204	2.54	2.50

AmplifyBio Study Number A-579-01

Individual Animal Body Weight & Weight Gain

Sex: Male Body Weight (kg)

Group 3 High Total vg 1.2x10^14	Day(s) Relative to Start Date										
	1	8	15	22	29	36	43	50	57	64	71
301	2.72	2.63	2.78	2.74	2.88	2.82	2.73	-	-	-	-
302	2.38	2.19	2.26	2.04	2.34	2.50	2.43	-	-	-	-
303	3.06	2.91	3.10	3.06	3.15	3.24	3.09	3.21	3.32	3.36	3.49
304	2.38	2.32	2.44	2.37	2.44	2.52	2.42	2.44	2.60	2.59	2.68

AmplifyBio Study Number A-579-01

Individual Animal Body Weight & Weight Gain

Sex: Male Body Weight (kg)

Group 3 High Total vg 1.2x10^14	Day(s) Relative to Start Date	
	78	85
301	-	-
302	-	-
303	3.40	3.44
304	2.52	2.62

AmplifyBio Study Number A-579-01

Individual Animal Body Weight & Weight Gain

Sex: Female Body Weight (kg)

Group 1 Control Total vg 0	Day(s) Relative to Start Date										
	1	8	15	22	29	36	43	50	57	64	71
151	2.64	2.64	2.83	2.71	2.70	2.83	2.75	-	-	-	-
152	5.20	5.07	5.20	5.02	5.13	5.31	5.08	5.14	5.23	5.21	5.39

AmplifyBio Study Number A-579-01

Individual Animal Body Weight & Weight Gain

Sex: Female Body Weight (kg)

Group 1 Control Total vg 0	Day(s) Relative to Start Date	
	78	85
151	-	-
152	5.36	5.39

AmplifyBio Study Number A-579-01

Individual Animal Body Weight & Weight Gain

Sex: Female Body Weight (kg)

Group 2 Low Total vg 6x10^13	Day(s) Relative to Start Date										
	1	8	15	22	29	36	43	50	57	64	71
251	3.09	2.96	3.10	3.02	3.11	3.21	3.12	-	-	-	-
252	2.64	2.56	2.74	2.71	2.74	2.78	2.69	-	-	-	-
253	3.22	3.16	3.15	3.06	3.24	3.35	3.15	3.19	3.38	3.42	3.47
254	2.70	2.54	2.81	2.79	2.79	2.80	2.66	2.73	2.85	2.81	2.89

AmplifyBio Study Number A-579-01

Individual Animal Body Weight & Weight Gain

Sex: Female Body Weight (kg)

Group 2 Low Total vg 6x10^13	Day(s) Relative to Start Date	
	78	85
251	-	-
252	-	-
253	3.51	3.51
254	2.71	2.70 [a]

[a] [RC:Transcribed from original data point inadvertently deleted with purge of path module data]

AmplifyBio Study Number A-579-01

Individual Animal Body Weight & Weight Gain

Sex: Female Body Weight (kg)

Group 3 High Total vg 1.2x10^14	Day(s) Relative to Start Date										
	1	8	15	22	29	36	43	50	57	64	71
351	2.47	2.38	2.37	2.37	2.40	2.47	2.39	-	-	-	-
352	2.88	2.79	2.77	2.81	2.95	2.93	2.87	-	-	-	-
353	3.00	2.95	3.17	3.08	3.16	3.36	3.08	3.13	3.14	3.13	3.09
354	2.81	2.65	2.94	2.87	2.89	3.03	2.86	2.89	2.93	3.02	3.13

AmplifyBio Study Number A-579-01

Individual Animal Body Weight & Weight Gain

Sex: Female Body Weight (kg)

Group 3 High Total vg 1.2x10^14	Day(s) Relative to Start Date	
	78	85
351	-	-
352	-	-
353	2.98	2.82
354	3.12	2.88

AmplifyBio Study Number A-579-01

Individual Animal Body Weight & Weight Gain

Sex: Male Absolute BodyWeight Changes (kg)

Group 1<br>Control<br>Total vg<br>0	Day(s) Relative<br>to Start Date										
	1 → 8	8 → 15	15 → 22	22 → 29	29 → 36	36 → 43	43 → 50	50 → 57	57 → 64	64 → 71	71 → 78
101	-0.09	0.18	-0.08	0.06	0.07	-0.10	.	.	.	.	.
102	-0.05	0.15	-0.07	0.15	0.09	-0.11	0.06	0.04	0.03	0.15	0.07

AmplifyBio Study Number A-579-01

Individual Animal Body Weight & Weight Gain

Sex: Male Absolute BodyWeight Changes (kg)

Group 1 Control Total vg 0	Day(s) Relative to Start Date 78 → 85
101	.
102	-0.07

AmplifyBio Study Number A-579-01

Individual Animal Body Weight & Weight Gain

Sex: Male Absolute BodyWeight Changes (kg)

Group 2 Low Total vg 6x10^13	Day(s) Relative to Start Date										
	1 → 8	8 → 15	15 → 22	22 → 29	29 → 36	36 → 43	43 → 50	50 → 57	57 → 64	64 → 71	71 → 78
201	-0.12	0.16	-0.08	0.12	0.04	-0.18	.	.	.	.	.
202	-0.12	0.17	-0.04	0.13	0.12	-0.17	.	.	.	.	.
203	-0.02	0.10	-0.01	0.12	0.13	-0.09	0.03	0.14	0.01	0.06	-0.17
204	-0.05	0.15	-0.02	0.00	0.12	-0.10	0.06	0.06	0.06	0.06	-0.05

AmplifyBio Study Number A-579-01

Individual Animal Body Weight & Weight Gain

Sex: Male Absolute BodyWeight Changes (kg)

Group 2 Low Total vg 6x10^13	Day(s) Relative to Start Date 78 → 85
201	.
202	.
203	-0.21
204	-0.04

AmplifyBio Study Number A-579-01

Individual Animal Body Weight & Weight Gain

Sex: Male Absolute BodyWeight Changes (kg)

Group 3 High Total vg 1.2x10^14	Day(s) Relative to Start Date										
	1 → 8	8 → 15	15 → 22	22 → 29	29 → 36	36 → 43	43 → 50	50 → 57	57 → 64	64 → 71	71 → 78
301	-0.09	0.15	-0.04	0.14	-0.06	-0.09	.	.	.	.	.
302	-0.19	0.07	-0.22	0.30	0.16	-0.07	.	.	.	.	.
303	-0.15	0.19	-0.04	0.09	0.09	-0.15	0.12	0.11	0.04	0.13	-0.09
304	-0.06	0.12	-0.07	0.07	0.08	-0.10	0.02	0.16	-0.01	0.09	-0.16

AmplifyBio Study Number A-579-01

Individual Animal Body Weight & Weight Gain

Sex: Male Absolute BodyWeight Changes (kg)

Group 3<br>High<br>Total vg<br>1.2x10^14	Day(s)<br>Relative<br>to Start Date<br>78 → 85
301	.
302	.
303	0.04
304	0.10

AmplifyBio Study Number A-579-01

Individual Animal Body Weight & Weight Gain

Sex: Female Absolute BodyWeight Changes (kg)

Group 1 Control Total vg 0	Day(s) Relative to Start Date										
	1 → 8	8 → 15	15 → 22	22 → 29	29 → 36	36 → 43	43 → 50	50 → 57	57 → 64	64 → 71	71 → 78
151	0.00	0.19	-0.12	-0.01	0.13	-0.08	.	.	.	.	.
152	-0.13	0.13	-0.18	0.11	0.18	-0.23	0.06	0.09	-0.02	0.18	-0.03

AmplifyBio Study Number A-579-01

Individual Animal Body Weight & Weight Gain

Sex: Female Absolute BodyWeight Changes (kg)

Group 1 Control Total vg 0	Day(s) Relative to Start Date 78 → 85
151	.
152	0.03

AmplifyBio Study Number A-579-01

Individual Animal Body Weight & Weight Gain

Sex: Female Absolute BodyWeight Changes (kg)

Group 2<br>Low<br>Total vg<br>6x10^13	Day(s) Relative to Start Date										
	1 → 8	8 → 15	15 → 22	22 → 29	29 → 36	36 → 43	43 → 50	50 → 57	57 → 64	64 → 71	71 → 78
251	-0.13	0.14	-0.08	0.09	0.10	-0.09	.	.	.	.	.
252	-0.08	0.18	-0.03	0.03	0.04	-0.09	.	.	.	.	.
253	-0.06	-0.01	-0.09	0.18	0.11	-0.20	0.04	0.19	0.04	0.05	0.04
254	-0.16	0.27	-0.02	0.00	0.01	-0.14	0.07	0.12	-0.04	0.08	-0.18

AmplifyBio Study Number A-579-01

Individual Animal Body Weight & Weight Gain

Sex: Female Absolute BodyWeight Changes (kg)

Group 2 Low Total vg 6x10^13	Day(s) Relative to Start Date 78 → 85
251	.
252	.
253	0.00
254	-0.01

AmplifyBio Study Number A-579-01

Individual Animal Body Weight & Weight Gain

Sex: Female Absolute BodyWeight Changes (kg)

Group 3 High Total vg 1.2x10^14	Day(s) Relative to Start Date										
	1 → 8	8 → 15	15 → 22	22 → 29	29 → 36	36 → 43	43 → 50	50 → 57	57 → 64	64 → 71	71 → 78
351	-0.09	-0.01	0.00	0.03	0.07	-0.08	.	.	.	.	.
352	-0.09	-0.02	0.04	0.14	-0.02	-0.06	.	.	.	.	.
353	-0.05	0.22	-0.09	0.08	0.20	-0.28	0.05	0.01	-0.01	-0.04	-0.11
354	-0.16	0.29	-0.07	0.02	0.14	-0.17	0.03	0.04	0.09	0.11	-0.01

AmplifyBio Study Number A-579-01

Individual Animal Body Weight & Weight Gain

Sex: Female Absolute BodyWeight Changes (kg)

Group 3<br>High<br>Total vg<br>1.2x10^14	Day(s)<br>Relative<br>to Start Date<br>78 → 85
351	.
352	.
353	-0.16
354	-0.24

AmplifyBio Study Number A-579-01

Individual Animal Body Weight & Weight Gain

Key Page

Measurement Descriptions

Headings Used	Description
Body Weight	Body Weight
Absolute BodyWeight Changes	Absolute BodyWeight Changes (kg)

Group Information

Short Name	Long Name	Type	Report Headings 1-4			
1	Group 1	Control	Group 1	Control	Total vg	0
2	Group 2	Dose	Group 2	Low	Total vg	6x10^13
3	Group 3	Dose	Group 3	High	Total vg	1.2x10^14

Comment Abbreviations

RC = Result Comment

Table D-5. Individual Animal Food Consumption

AmplifyBio Study Number A-579-01

Individual Animal Food Consumption

Sex: Male Qualitative Food Consumption

Group 1 Control Total vg 0	Day(s) Relative to Start Date										
	1	2	3	4	5	6	7	8	9	10	11
101	4	NA-F	4	4	4	4	4	NA-F	4	4	4
102	4	NA-F	4	4	4	4	4	NA-F	4	4	4

AmplifyBio Study Number A-579-01

Individual Animal Food Consumption

Sex: Male Qualitative Food Consumption

Group 1 Control Total vg 0	Day(s) Relative to Start Date										
	12	13	14	15	16	17	18	19	20	21	22
101	4	4	4	4	4	4	4	4	4	4	NA-F
102	4	4	4	4	4	4	4	4	4	4	NA-F

AmplifyBio Study Number A-579-01

Individual Animal Food Consumption

Sex: Male Qualitative Food Consumption

Group 1<br>Control<br>Total vg<br>0	Day(s) Relative to Start Date										
	23	24	25	26	27	28	29	30	31	32	33
101	4	4	4	4	4	4	4	4	4	4	4
102	4	4	4	4	4	4	4	4	4	4	4

AmplifyBio Study Number A-579-01

Individual Animal Food Consumption

Sex: Male Qualitative Food Consumption

Group 1 Control Total vg 0	Day(s) Relative to Start Date										
	34	35	36	37	38	39	40	41	42	43	44
101	4	4	4	4	4	4	4	4	4	NA-F	-
102	4	4	4	4	4	4	4	4	4	NA-F	4

AmplifyBio Study Number A-579-01

Individual Animal Food Consumption

Sex: Male Qualitative Food Consumption

Group 1 Control Total vg 0	Day(s) Relative to Start Date										
	45	46	47	48	49	50	51	52	53	54	55
101	-	-	-	-	-	-	-	-	-	-	-
102	4	4	4	4	4	NA-F	4	4	4	4	4

AmplifyBio Study Number A-579-01

Individual Animal Food Consumption

Sex: Male Qualitative Food Consumption

Group 1<br>Control<br>Total vg<br>0	Day(s) Relative to Start Date										
	56	57	58	59	60	61	62	63	64	65	66
101	-	-	-	-	-	-	-	-	-	-	-
102	4	4	4	4	4	4	4	4	4	4	4

AmplifyBio Study Number A-579-01

Individual Animal Food Consumption

Sex: Male Qualitative Food Consumption

Group 1 Control Total vg 0	Day(s) Relative to Start Date										
	67	68	69	70	71	72	73	74	75	76	77
101	-	-	-	-	-	-	-	-	-	-	-
102	4	4	4	4	4	4	4	4	4	4	4

AmplifyBio Study Number A-579-01

Individual Animal Food Consumption

Sex: Male Qualitative Food Consumption

Group 1<br>Control<br>Total vg<br>0	Day(s) Relative to Start Date							
	78	79	80	81	82	83	84	85
101	-	-	-	-	-	-	-	-
102	4	4	4	4	4	4	4	NA-F

AmplifyBio Study Number A-579-01

Individual Animal Food Consumption

Sex: Male Qualitative Food Consumption

Group 2 Low Total vg 6x10^13	Day(s) Relative to Start Date										
	1	2	3	4	5	6	7	8	9	10	11
201	4	NA-F	4	4	4	4	4	NA-F	3	4	4
202	4	NA-F	4	4	4	4	4	NA-F	3	4	4
203	4	NA-F	4	4	4	4	4	NA-F	4	4	4
204	4	NA-F	4	4	4	4	4	NA-F	4	4	4

AmplifyBio Study Number A-579-01

Individual Animal Food Consumption

Sex: Male Qualitative Food Consumption

Group 2 Low Total vg 6x10^13	Day(s) Relative to Start Date										
	12	13	14	15	16	17	18	19	20	21	22
201	4	4	4	4	4	4	4	4	4	4	NA-F
202	4	4	4	4	4	4	4	4	4	4	NA-F
203	4	4	4	4	4	4	4	4	4	4	NA-F
204	4	4	4	4	4	4	4	4	4	4	NA-F

AmplifyBio Study Number A-579-01

Individual Animal Food Consumption

Sex: Male Qualitative Food Consumption

Group 2 Low Total vg 6x10^13	Day(s) Relative to Start Date										
	23	24	25	26	27	28	29	30	31	32	33
201	4	4	4	4	4	4	4	4	4	4	4
202	4	4	4	4	4	4	4	4	4	4	4
203	4	4	4	4	4	4	4	4	4	4	4
204	4	4	4	4	4	4	4	4	4	4	4

AmplifyBio Study Number A-579-01

Individual Animal Food Consumption

Sex: Male Qualitative Food Consumption

Group 2 Low Total vg 6x10^13	Day(s) Relative to Start Date										
	34	35	36	37	38	39	40	41	42	43	44
201	4	4	4	4	4	4	4	4	4	NA-F	-
202	4	4	4	4	4	4	4	4	4	NA-F	-
203	4	4	4	4	4	4	4	4	4	NA-F	4
204	4	4	4	4	4	4	4	4	4	NA-F	4

AmplifyBio Study Number A-579-01

Individual Animal Food Consumption

Sex: Male Qualitative Food Consumption

Group 2 Low Total vg 6x10^13	Day(s) Relative to Start Date										
	45	46	47	48	49	50	51	52	53	54	55
201	-	-	-	-	-	-	-	-	-	-	-
202	-	-	-	-	-	-	-	-	-	-	-
203	4	4	4	4	4	NA-F	4	4	4	4	4
204	4	4	4	4	4	NA-F	4	4	4	4	4

AmplifyBio Study Number A-579-01

Individual Animal Food Consumption

Sex: Male Qualitative Food Consumption

Group 2 Low Total vg 6x10^13	Day(s) Relative to Start Date										
	56	57	58	59	60	61	62	63	64	65	66
201	-	-	-	-	-	-	-	-	-	-	-
202	-	-	-	-	-	-	-	-	-	-	-
203	4	4	4	4	4	4	4	4	4	4	4
204	4	4	4	4	4	4	4	4	4	4	4

AmplifyBio Study Number A-579-01

Individual Animal Food Consumption

Sex: Male Qualitative Food Consumption

Group 2 Low Total vg 6x10^13	Day(s) Relative to Start Date										
	67	68	69	70	71	72	73	74	75	76	77
201	-	-	-	-	-	-	-	-	-	-	-
202	-	-	-	-	-	-	-	-	-	-	-
203	4	4	4	4	4	4	4	3	3	3	4
204	4	4	4	4	4	4	4	3	3	3	4

AmplifyBio Study Number A-579-01

Individual Animal Food Consumption

Sex: Male Qualitative Food Consumption

Group 2 Low Total vg 6x10^13	Day(s) Relative to Start Date							
	78	79	80	81	82	83	84	85
201	-	-	-	-	-	-	-	-
202	-	-	-	-	-	-	-	-
203	4	4	4	3	4	4	4	NA-F
204	4	4	4	3	4	4	4	NA-F

AmplifyBio Study Number A-579-01

Individual Animal Food Consumption

Sex: Male Qualitative Food Consumption

Group 3 High Total vg 1.2x10^14	Day(s) Relative to Start Date										
	1	2	3	4	5	6	7	8	9	10	11
301	4	NA-F	4	4	4	4	4	NA-F	4	4	4
302	4	NA-F	4	4	4	4	4	NA-F	4	4	4
303	4	NA-F	4	4	4	4	4	NA-F	4	4	4
304	4	NA-F	4	4	4	4	4	NA-F	4	4	4

AmplifyBio Study Number A-579-01

Individual Animal Food Consumption

Sex: Male Qualitative Food Consumption

Group 3 High Total vg 1.2x10^14	Day(s) Relative to Start Date										
	12	13	14	15	16	17	18	19	20	21	22
301	4	4	4	4	4	4	4	4	4	4	NA-F
302	4	4	4	4	4	4	4	4	4	4	NA-F
303	4	4	4	4	4	4	4	4	4	4	NA-F
304	4	4	4	4	4	4	4	4	4	4	NA-F

AmplifyBio Study Number A-579-01

Individual Animal Food Consumption

Sex: Male Qualitative Food Consumption

Group 3 High Total vg 1.2x10^14	Day(s) Relative to Start Date										
	23	24	25	26	27	28	29	30	31	32	33
301	4	4	4	4	4	4	4	4	4	4	4
302	2	0	4	3	4	4	4	4	4	4	4
303	4	4	4	4	4	4	4	4	4	4	4
304	4	4	4	4	4	4	4	4	4	4	4

AmplifyBio Study Number A-579-01

Individual Animal Food Consumption

Sex: Male Qualitative Food Consumption

Group 3<br>High<br>Total vg<br>1.2x10^14	Day(s) Relative to Start Date										
	34	35	36	37	38	39	40	41	42	43	44
301	4	4	4	4	4	4	4	4	4	NA-F	-
302	4	4	4	4	4	4	4	4	4	NA-F	-
303	4	4	4	4	3	4	4	4	4	NA-F	4
304	4	4	4	4	3	4	4	4	4	NA-F	4

AmplifyBio Study Number A-579-01

Individual Animal Food Consumption

Sex: Male Qualitative Food Consumption

Group 3 High Total vg 1.2x10^14	Day(s) Relative to Start Date										
	45	46	47	48	49	50	51	52	53	54	55
301	-	-	-	-	-	-	-	-	-	-	-
302	-	-	-	-	-	-	-	-	-	-	-
303	4	4	4	4	4	NA-F	4	4	4	4	4
304	4	4	4	4	4	NA-F	4	4	4	4	4

AmplifyBio Study Number A-579-01

Individual Animal Food Consumption

Sex: Male Qualitative Food Consumption

Group 3 High Total vg 1.2x10^14	Day(s) Relative to Start Date										
	56	57	58	59	60	61	62	63	64	65	66
301	-	-	-	-	-	-	-	-	-	-	-
302	-	-	-	-	-	-	-	-	-	-	-
303	4	4	4	4	4	4	4	4	4	4	4
304	4	4	4	4	4	4	4	4	4	4	4

AmplifyBio Study Number A-579-01

Individual Animal Food Consumption

Sex: Male Qualitative Food Consumption

Group 3 High Total vg 1.2x10^14	Day(s) Relative to Start Date										
	67	68	69	70	71	72	73	74	75	76	77
301	-	-	-	-	-	-	-	-	-	-	-
302	-	-	-	-	-	-	-	-	-	-	-
303	4	4	4	4	4	4	4	4	4	4	4
304	4	4	4	4	4	4	4	4	4	4	4

AmplifyBio Study Number A-579-01

Individual Animal Food Consumption

Sex: Male Qualitative Food Consumption

Group 3<br>High<br>Total vg<br>1.2x10^14	Day(s) Relative to Start Date							
	78	79	80	81	82	83	84	85
301	-	-	-	-	-	-	-	-
302	-	-	-	-	-	-	-	-
303	4	4	4	4	4	4	4	NA-F
304	4	4	4	4	4	4	4	NA-F

AmplifyBio Study Number A-579-01

Individual Animal Food Consumption

Sex: Female Qualitative Food Consumption

Group 1<br>Control<br>Total vg<br>0	Day(s) Relative<br>to Start Date										
	1	2	3	4	5	6	7	8	9	10	11
151	4	NA-F	3	4	4	4	4	NA-F	4	4	4
152	4	NA-F	3	4	4	4	4	NA-F	4	4	4

AmplifyBio Study Number A-579-01

Individual Animal Food Consumption

Sex: Female Qualitative Food Consumption

Group 1 Control Total vg 0	Day(s) Relative to Start Date										
	12	13	14	15	16	17	18	19	20	21	22
151	4	4	4	4	4	4	4	4	4	4	NA-F
152	4	4	4	4	4	4	4	4	4	4	NA-F

AmplifyBio Study Number A-579-01

Individual Animal Food Consumption

Sex: Female Qualitative Food Consumption

Group 1 Control Total vg 0	Day(s) Relative to Start Date										
	23	24	25	26	27	28	29	30	31	32	33
151	4	4	4	4	4	4	4	4	4	4	4
152	4	4	4	4	4	4	4	4	4	4	4

AmplifyBio Study Number A-579-01

Individual Animal Food Consumption

Sex: Female Qualitative Food Consumption

Group 1 Control Total vg 0	Day(s) Relative to Start Date										
	34	35	36	37	38	39	40	41	42	43	44
151	4	4	4	4	4	4	4	4	4	NA-F	-
152	4	4	4	4	4	4	4	4	4	NA-F	4

AmplifyBio Study Number A-579-01

Individual Animal Food Consumption

Sex: Female Qualitative Food Consumption

Group 1 Control Total vg 0	Day(s) Relative to Start Date										
	45	46	47	48	49	50	51	52	53	54	55
151	-	-	-	-	-	-	-	-	-	-	-
152	4	4	4	4	4	NA-F	4	4	4	4	4

AmplifyBio Study Number A-579-01

Individual Animal Food Consumption

Sex: Female Qualitative Food Consumption

Group 1 Control Total vg 0	Day(s) Relative to Start Date										
	56	57	58	59	60	61	62	63	64	65	66
151	-	-	-	-	-	-	-	-	-	-	-
152	4	4	4	4	4	4	4	4	4	4	4

AmplifyBio Study Number A-579-01

Individual Animal Food Consumption

Sex: Female Qualitative Food Consumption

Group 1 Control Total vg 0	Day(s) Relative to Start Date										
	67	68	69	70	71	72	73	74	75	76	77
151	-	-	-	-	-	-	-	-	-	-	-
152	4	4	4	4	4	4	4	4	4	4	4

AmplifyBio Study Number A-579-01

Individual Animal Food Consumption

Sex: Female Qualitative Food Consumption

Group 1 Control Total vg 0	Day(s) Relative to Start Date							
	78	79	80	81	82	83	84	85
151	-	-	-	-	-	-	-	-
152	4	4	4	4	4	4	4	NA-F

AmplifyBio Study Number A-579-01

Individual Animal Food Consumption

Sex: Female Qualitative Food Consumption

Group 2 Low Total vg 6x10^13	Day(s) Relative to Start Date										
	1	2	3	4	5	6	7	8	9	10	11
251	4	NA-F	4	4	4	4	4	NA-F	4	4	4
252	4	NA-F	4	4	4	4	4	NA-F	4	4	4
253	4	NA-F	4	4	4	4	4	NA-F	3	4	4
254	4	NA-F	4	4	4	4	4	NA-F	3	4	4

AmplifyBio Study Number A-579-01

Individual Animal Food Consumption

Sex: Female Qualitative Food Consumption

Group 2<br>Low<br>Total vg<br>6x10^13	Day(s) Relative to Start Date										
	12	13	14	15	16	17	18	19	20	21	22
251	4	4	4	4	4	4	4	4	4	4	NA-F
252	4	4	4	4	4	4	4	4	4	4	NA-F
253	4	4	4	4	4	4	4	4	4	4	NA-F
254	4	4	4	4	4	4	4	4	4	4	NA-F

AmplifyBio Study Number A-579-01

Individual Animal Food Consumption

Sex: Female Qualitative Food Consumption

Group 2 Low Total vg 6x10^13	Day(s) Relative to Start Date										
	23	24	25	26	27	28	29	30	31	32	33
251	4	4	4	4	4	4	4	4	4	4	4
252	4	4	4	4	4	4	4	4	4	4	4
253	4	4	4	4	4	4	4	4	4	4	4
254	4	4	4	4	4	4	4	4	4	4	4

AmplifyBio Study Number A-579-01

Individual Animal Food Consumption

Sex: Female Qualitative Food Consumption

Group 2 Low Total vg 6x10^13	Day(s) Relative to Start Date										
	34	35	36	37	38	39	40	41	42	43	44
251	4	4	4	4	4	4	4	4	4	NA-F	-
252	4	4	4	4	4	4	4	4	4	NA-F	-
253	4	4	4	4	4	4	4	4	4	NA-F	4
254	4	4	4	4	4	4	4	4	4	NA-F	4

AmplifyBio Study Number A-579-01

Individual Animal Food Consumption

Sex: Female Qualitative Food Consumption

Group 2<br>Low<br>Total vg<br>6x10^13	Day(s) Relative to Start Date										
	45	46	47	48	49	50	51	52	53	54	55
251	-	-	-	-	-	-	-	-	-	-	-
252	-	-	-	-	-	-	-	-	-	-	-
253	4	4	4	4	4	NA-F	4	4	4	4	4
254	4	4	4	4	4	NA-F	4	4	4	4	4

AmplifyBio Study Number A-579-01

Individual Animal Food Consumption

Sex: Female Qualitative Food Consumption

Group 2 Low Total vg 6x10^13	Day(s) Relative to Start Date										
	56	57	58	59	60	61	62	63	64	65	66
251	-	-	-	-	-	-	-	-	-	-	-
252	-	-	-	-	-	-	-	-	-	-	-
253	4	4	4	4	4	4	4	4	4	4	4
254	4	4	4	4	4	4	4	4	4	4	4

AmplifyBio Study Number A-579-01

Individual Animal Food Consumption

Sex: Female Qualitative Food Consumption

Group 2 Low Total vg 6x10^13	Day(s) Relative to Start Date										
	67	68	69	70	71	72	73	74	75	76	77
251	-	-	-	-	-	-	-	-	-	-	-
252	-	-	-	-	-	-	-	-	-	-	-
253	4	4	4	4	4	4	4	4	4	4	4
254	4	4	4	4	4	4	4	4	4	4	4

AmplifyBio Study Number A-579-01

Individual Animal Food Consumption

Sex: Female Qualitative Food Consumption

Group 2 Low Total vg 6x10^13	Day(s) Relative to Start Date							
	78	79	80	81	82	83	84	85
251	-	-	-	-	-	-	-	-
252	-	-	-	-	-	-	-	-
253	4	4	4	3	4	4	4	NA-F
254	4	4	4	3	4	4	4	NA-F

AmplifyBio Study Number A-579-01

Individual Animal Food Consumption

Sex: Female Qualitative Food Consumption

Group 3 High Total vg 1.2x10^14	Day(s) Relative to Start Date										
	1	2	3	4	5	6	7	8	9	10	11
351	3	NA-F	4	3	4	4	4	NA-F	4	3	4
352	3	NA-F	4	3	4	4	4	NA-F	4	3	4
353	3	NA-F	4	4	4	4	4	NA-F	4	4	4
354	3	NA-F	4	4	4	4	4	NA-F	4	4	4

AmplifyBio Study Number A-579-01

Individual Animal Food Consumption

Sex: Female Qualitative Food Consumption

Group 3 High Total vg 1.2x10^14	Day(s) Relative to Start Date										
	12	13	14	15	16	17	18	19	20	21	22
351	4	4	3	4	4	4	4	4	4	4	NA-F
352	4	4	3	4	4	4	4	4	4	4	NA-F
353	4	4	4	4	4	4	4	4	4	4	NA-F
354	4	4	4	4	4	4	4	4	4	4	NA-F

AmplifyBio Study Number A-579-01

Individual Animal Food Consumption

Sex: Female Qualitative Food Consumption

Group 3 High Total vg 1.2x10^14	Day(s) Relative to Start Date										
	23	24	25	26	27	28	29	30	31	32	33
351	4	4	4	4	4	4	4	3	4	4	4
352	4	4	4	4	4	4	4	3	4	4	4
353	4	4	4	4	4	4	4	4	4	4	4
354	4	4	4	4	4	4	4	4	4	4	4

AmplifyBio Study Number A-579-01

Individual Animal Food Consumption

Sex: Female Qualitative Food Consumption

Group 3<br>High<br>Total vg<br>1.2x10^14	Day(s) Relative to Start Date										
	34	35	36	37	38	39	40	41	42	43	44
351	4	4	4	4	4	4	4	4	4	NA-F	-
352	4	4	4	4	4	4	4	4	4	NA-F	-
353	4	4	4	4	4	4	4	4	4	NA-F	4
354	4	4	4	4	4	4	4	4	4	NA-F	4

AmplifyBio Study Number A-579-01

Individual Animal Food Consumption

Sex: Female Qualitative Food Consumption

Group 3 High Total vg 1.2x10^14	Day(s) Relative to Start Date										
	45	46	47	48	49	50	51	52	53	54	55
351	-	-	-	-	-	-	-	-	-	-	-
352	-	-	-	-	-	-	-	-	-	-	-
353	4	4	4	4	4	NA-F	4	4	4	4	4
354	4	4	4	4	4	NA-F	4	4	4	4	4

AmplifyBio Study Number A-579-01

Individual Animal Food Consumption

Sex: Female Qualitative Food Consumption

Group 3 High Total vg 1.2x10^14	Day(s) Relative to Start Date										
	56	57	58	59	60	61	62	63	64	65	66
351	-	-	-	-	-	-	-	-	-	-	-
352	-	-	-	-	-	-	-	-	-	-	-
353	4	4	4	4	4	4	4	4	4	4	4
354	4	4	4	4	4	4	4	4	4	4	4

AmplifyBio Study Number A-579-01

Individual Animal Food Consumption

Sex: Female Qualitative Food Consumption

Group 3<br>High<br>Total vg<br>1.2x10^14	Day(s) Relative to Start Date										
	67	68	69	70	71	72	73	74	75	76	77
351	-	-	-	-	-	-	-	-	-	-	-
352	-	-	-	-	-	-	-	-	-	-	-
353	4	4	4	4	4	4	4	4	4	4	4
354	4	4	4	4	4	4	4	4	4	4	4

AmplifyBio Study Number A-579-01

Individual Animal Food Consumption

Sex: Female Qualitative Food Consumption

Group 3 High Total vg 1.2x10^14	Day(s) Relative to Start Date							
	78	79	80	81	82	83	84	85
351	-	-	-	-	-	-	-	-
352	-	-	-	-	-	-	-	-
353	4	4	4	4	4	4	3	NA-F
354	4	4	4	4	4	4	3	NA-F

AmplifyBio Study Number A-579-01

Individual Animal Food Consumption

Comments and Markers

Page	Measurement	Group	Sex	Subject	Day	Type	Marker
1	Qualitative Food Consumption	1	Male	101	2	Replacement	NA-F
1	Qualitative Food Consumption	1	Male	101	8	Replacement	NA-F
1	Qualitative Food Consumption	1	Male	102	2	Replacement	NA-F
1	Qualitative Food Consumption	1	Male	102	8	Replacement	NA-F
2	Qualitative Food Consumption	1	Male	101	22	Replacement	NA-F
2	Qualitative Food Consumption	1	Male	102	22	Replacement	NA-F
4	Qualitative Food Consumption	1	Male	101	43	Replacement	NA-F
4	Qualitative Food Consumption	1	Male	102	43	Replacement	NA-F
5	Qualitative Food Consumption	1	Male	102	50	Replacement	NA-F
8	Qualitative Food Consumption	1	Male	102	85	Replacement	NA-F
9	Qualitative Food Consumption	2	Male	201	2	Replacement	NA-F
9	Qualitative Food Consumption	2	Male	201	8	Replacement	NA-F
9	Qualitative Food Consumption	2	Male	202	2	Replacement	NA-F
9	Qualitative Food Consumption	2	Male	202	8	Replacement	NA-F
9	Qualitative Food Consumption	2	Male	203	2	Replacement	NA-F
9	Qualitative Food Consumption	2	Male	203	8	Replacement	NA-F
9	Qualitative Food Consumption	2	Male	204	2	Replacement	NA-F
9	Qualitative Food Consumption	2	Male	204	8	Replacement	NA-F
10	Qualitative Food Consumption	2	Male	201	22	Replacement	NA-F
10	Qualitative Food Consumption	2	Male	202	22	Replacement	NA-F
10	Qualitative Food Consumption	2	Male	203	22	Replacement	NA-F
10	Qualitative Food Consumption	2	Male	204	22	Replacement	NA-F
12	Qualitative Food Consumption	2	Male	201	43	Replacement	NA-F
12	Qualitative Food Consumption	2	Male	202	43	Replacement	NA-F
12	Qualitative Food Consumption	2	Male	203	43	Replacement	NA-F
12	Qualitative Food Consumption	2	Male	204	43	Replacement	NA-F
13	Qualitative Food Consumption	2	Male	203	50	Replacement	NA-F
13	Qualitative Food Consumption	2	Male	204	50	Replacement	NA-F
16	Qualitative Food Consumption	2	Male	203	85	Replacement	NA-F

AmplifyBio Study Number A-579-01

Individual Animal Food Consumption

Comments and Markers

Page	Measurement	Group	Sex	Subject	Day	Type	Marker
16	Qualitative Food Consumption	2	Male	204	85	Replacement	NA-F
17	Qualitative Food Consumption	3	Male	301	2	Replacement	NA-F
17	Qualitative Food Consumption	3	Male	301	8	Replacement	NA-F
17	Qualitative Food Consumption	3	Male	302	2	Replacement	NA-F
17	Qualitative Food Consumption	3	Male	302	8	Replacement	NA-F
17	Qualitative Food Consumption	3	Male	303	2	Replacement	NA-F
17	Qualitative Food Consumption	3	Male	303	8	Replacement	NA-F
17	Qualitative Food Consumption	3	Male	304	2	Replacement	NA-F
17	Qualitative Food Consumption	3	Male	304	8	Replacement	NA-F
18	Qualitative Food Consumption	3	Male	301	22	Replacement	NA-F
18	Qualitative Food Consumption	3	Male	302	22	Replacement	NA-F
18	Qualitative Food Consumption	3	Male	303	22	Replacement	NA-F
18	Qualitative Food Consumption	3	Male	304	22	Replacement	NA-F
20	Qualitative Food Consumption	3	Male	301	43	Replacement	NA-F
20	Qualitative Food Consumption	3	Male	302	43	Replacement	NA-F
20	Qualitative Food Consumption	3	Male	303	43	Replacement	NA-F
20	Qualitative Food Consumption	3	Male	304	43	Replacement	NA-F
21	Qualitative Food Consumption	3	Male	303	50	Replacement	NA-F
21	Qualitative Food Consumption	3	Male	304	50	Replacement	NA-F
24	Qualitative Food Consumption	3	Male	303	85	Replacement	NA-F
24	Qualitative Food Consumption	3	Male	304	85	Replacement	NA-F
25	Qualitative Food Consumption	1	Female	151	2	Replacement	NA-F
25	Qualitative Food Consumption	1	Female	151	8	Replacement	NA-F
25	Qualitative Food Consumption	1	Female	152	2	Replacement	NA-F
25	Qualitative Food Consumption	1	Female	152	8	Replacement	NA-F
26	Qualitative Food Consumption	1	Female	151	22	Replacement	NA-F
26	Qualitative Food Consumption	1	Female	152	22	Replacement	NA-F
28	Qualitative Food Consumption	1	Female	151	43	Replacement	NA-F
28	Qualitative Food Consumption	1	Female	152	43	Replacement	NA-F

AmplifyBio Study Number A-579-01

Individual Animal Food Consumption

Comments and Markers

Page	Measurement	Group	Sex	Subject	Day	Type	Marker
29	Qualitative Food Consumption	1	Female	152	50	Replacement	NA-F
32	Qualitative Food Consumption	1	Female	152	85	Replacement	NA-F
33	Qualitative Food Consumption	2	Female	251	2	Replacement	NA-F
33	Qualitative Food Consumption	2	Female	251	8	Replacement	NA-F
33	Qualitative Food Consumption	2	Female	252	2	Replacement	NA-F
33	Qualitative Food Consumption	2	Female	252	8	Replacement	NA-F
33	Qualitative Food Consumption	2	Female	253	2	Replacement	NA-F
33	Qualitative Food Consumption	2	Female	253	8	Replacement	NA-F
33	Qualitative Food Consumption	2	Female	254	2	Replacement	NA-F
33	Qualitative Food Consumption	2	Female	254	8	Replacement	NA-F
34	Qualitative Food Consumption	2	Female	251	22	Replacement	NA-F
34	Qualitative Food Consumption	2	Female	252	22	Replacement	NA-F
34	Qualitative Food Consumption	2	Female	253	22	Replacement	NA-F
34	Qualitative Food Consumption	2	Female	254	22	Replacement	NA-F
36	Qualitative Food Consumption	2	Female	251	43	Replacement	NA-F
36	Qualitative Food Consumption	2	Female	252	43	Replacement	NA-F
36	Qualitative Food Consumption	2	Female	253	43	Replacement	NA-F
36	Qualitative Food Consumption	2	Female	254	43	Replacement	NA-F
37	Qualitative Food Consumption	2	Female	253	50	Replacement	NA-F
37	Qualitative Food Consumption	2	Female	254	50	Replacement	NA-F
40	Qualitative Food Consumption	2	Female	253	85	Replacement	NA-F
40	Qualitative Food Consumption	2	Female	254	85	Replacement	NA-F
41	Qualitative Food Consumption	3	Female	351	2	Replacement	NA-F
41	Qualitative Food Consumption	3	Female	351	8	Replacement	NA-F
41	Qualitative Food Consumption	3	Female	352	2	Replacement	NA-F
41	Qualitative Food Consumption	3	Female	352	8	Replacement	NA-F
41	Qualitative Food Consumption	3	Female	353	2	Replacement	NA-F
41	Qualitative Food Consumption	3	Female	353	8	Replacement	NA-F
41	Qualitative Food Consumption	3	Female	354	2	Replacement	NA-F

AmplifyBio Study Number A-579-01

Individual Animal Food Consumption

Comments and Markers

Page	Measurement	Group	Sex	Subject	Day	Type	Marker
41	Qualitative Food Consumption	3	Female	354	8	Replacement	NA-F
42	Qualitative Food Consumption	3	Female	351	22	Replacement	NA-F
42	Qualitative Food Consumption	3	Female	352	22	Replacement	NA-F
42	Qualitative Food Consumption	3	Female	353	22	Replacement	NA-F
42	Qualitative Food Consumption	3	Female	354	22	Replacement	NA-F
44	Qualitative Food Consumption	3	Female	351	43	Replacement	NA-F
44	Qualitative Food Consumption	3	Female	352	43	Replacement	NA-F
44	Qualitative Food Consumption	3	Female	353	43	Replacement	NA-F
44	Qualitative Food Consumption	3	Female	354	43	Replacement	NA-F
45	Qualitative Food Consumption	3	Female	353	50	Replacement	NA-F
45	Qualitative Food Consumption	3	Female	354	50	Replacement	NA-F
48	Qualitative Food Consumption	3	Female	353	85	Replacement	NA-F
48	Qualitative Food Consumption	3	Female	354	85	Replacement	NA-F

AmplifyBio Study Number A-579-01

Individual Animal Food Consumption

Key Page

Replacement Values

Value	Description
NA-F	Not Applicable (Fasted)

Measurement Descriptions

Headings Used	Description
Qualitative Food Consumption	Qualitative Food Consumption

Group Information

Short Name	Long Name	Type	Report Headings 1-4			
1	Group 1	Control	Group 1	Control	Total vg	0
2	Group 2	Dose	Group 2	Low	Total vg	6x10^13
3	Group 3	Dose	Group 3	High	Total vg	1.2x10^14

Appendix E Ophthalmic Examination Contributing Scientist Report

A 12-Week Single Dose Biodistribution and Safety Study in Cynomolgus Monkeys

Ophthalmic Examinations Contributing Scientist Report

June 5, 2024

Study Number A-579-01

Testing Facility
AmplifyBio
1425 State Route 142 (Plain City-Georgesville Road)
West Jefferson, OH 43162

SIGNATURES

Prepared by:

DocuSigned by:

Bill Greentree

Signer Name: Bill Greentree
Signing Reason: I approve this document
Signing Time: 6/5/2024 | 2:41:19 PM EDT
5F037002276E48AE98E7F18B171A791F

9/8/2023

William Greentree, DVM, DACVO
Veterinary Ophthalmologist

Date

TABLE OF CONTENTS

Page

Signatures 2
1.0 Introduction 4
2.0 Materials and Methods 4
3.0 Deviations 4
4.0 Results 4
5.0 Conclusions 5

LIST OF ATTACHMENTS

Attachment 1. Group Summary of Ophthalmic Examinations 6
Attachment 2. Individual Animal Ophthalmic Examinations 10

1.0 INTRODUCTION

This contributing scientist report summarizes the ophthalmic findings in Study No. A-579-01. The purpose of this study was to evaluate the potential biodistribution and safety of scAAV9.U6miR871 (ARM-101) when administered once via intrathecal infusion to Cynomolgus monkeys for up to 12 weeks.

2.0 MATERIALS AND METHODS

Ophthalmic examinations were performed on all animals assigned to the study once during the pretest period, once on Week 6, and once for all surviving animals during Week 12 by a board-certified veterinary ophthalmologist.

Animals were sedated with ketamine for examinations.

The pupils of each assigned animal were dilated by instillation of drops of tropicamide ophthalmic solution prior to examination. The anterior segment of the eye, posterior segment of the eye, and adnexal structures were examined using a Kowa SL-17 portable slit lamp and a Hiene 180 indirect ophthalmoscope with Volk 30 Diopter condensing lens. Each eye examined was recorded as either "no abnormal findings (NADO)" or "cataract (Cat)" (see deviation QE-002014).

Nuclear cataracts are a common spontaneous background findings observed in Cynomolgus monkey and typical to see in monkeys of this age and were not considered findings.

3.0 DEVIATIONS

QE-002014: Protocol Section 11.8 "Ophthalmic Examinations" states "Each eye examined will be recorded as normal, abnormal, or not examined." Ophthalmic examinations were recorded as "no abnormal findings" or " Cataract" on Days -7, -6, -5, 39, 40, 41, 80, 81, and 82 instead of "normal, abnormal, or not examined" as required by the protocol. There was no study impact due to this documentation error as the animals were examined as intended. There was no study impact due to this documentation error as the animals were examined as intended.

4.0 RESULTS

Summary Data: Attachment 1
Individual Data: Attachment 2

Ocular examinations performed during this study revealed no clinical evidence of a test article-related ocular effect in any exposure group.

Cataracts were observed in one animal (Animal 151) during the pre-test examination (Day -5) and on the week of scheduled necropsy (Day 41). This observation was found in a control animal and is a common finding seen in Cynomolgus monkeys of this age, hence considered non-test article related.

5.0 CONCLUSIONS

There was no clinical evidence of test article-related ocular effects following a single administration of scAAV9.U6miR871 (ARM-101) via intrathecal infusion to Cynomolgus monkeys for up to 12 weeks.

Attachment 1. Group Summary of Ophthalmic Examinations

9/13/2023 5:58:20AM Page: 1

AmplifyBio Study Number A-579-01

Group Summary of Ophthalmic Exams

Observation Type: Ophthalmic Exam<br>Sex: Male<br>From Day -9999 (Start Date) to 9999 (Start Date)	Group 1<br>Vehicle	Group 2<br>Low	Group 3<br>High
**Left Eye, Cataract**			
Number of Times Recorded	0	0	0
**Left Eye, No Abnormal Findings**			
Number of Animals Affected	2	4	4
Number of Times Recorded	5	10	10
Number of Days Seen	132	264	270
First to Last seen	-5 - 81	-6 - 80	-7 - 82
**Right Eye, Cataract**			
Number of Times Recorded	0	0	0
**Right Eye, No Abnormal Findings**			
Number of Animals Affected	2	4	4
Number of Times Recorded	5	10	10
Number of Days Seen	132	264	270
First to Last seen	-5 - 81	-6 - 80	-7 - 82

9/13/2023 5:58:20AM Page: 2

AmplifyBio Study Number A-579-01

Group Summary of Ophthalmic Exams

Observation Type: Ophthalmic Exam<br>Sex: Female<br>From Day -9999 (Start Date) to 9999 (Start Date)	Group 1<br>Vehicle	Group 2<br>Low	Group 3<br>High
**Left Eye, Cataract**			
Number of Animals Affected	1	0	0
Number of Times Recorded	2	0	0
Number of Days Seen	46	0	0
First to Last seen	-5 - 41	-	-
**Left Eye, No Abnormal Findings**			
Number of Animals Affected	1	4	4
Number of Times Recorded	3	10	10
Number of Days Seen	86	264	270
First to Last seen	-5 - 81	-6 - 80	-7 - 82
**Right Eye, Cataract**			
Number of Animals Affected	1	0	0
Number of Times Recorded	2	0	0
Number of Days Seen	46	0	0
First to Last seen	-5 - 41	-	-
**Right Eye, No Abnormal Findings**			
Number of Animals Affected	1	4	4
Number of Times Recorded	3	10	10
Number of Days Seen	86	264	270
First to Last seen	-5 - 81	-6 - 80	-7 - 82

AmplifyBio Study Number A-579-01

Group Summary of Ophthalmic Exams

Key Page

Group Information

Short Name	Long Name	Type	Report Headings	
1	Group 1	Control	Group 1	Vehicle
2	Group 2	Dose	Group 2	Low
3	Group 3	Dose	Group 3	High

Attachment 2. Individual Animal Ophthalmic Examinations

AmplifyBio Study Number A-579-01

Individual Animal Ophthalmic Exams

Group 1<br>Vehicle<br>Sex: Male	Observation Type: Ophthalmic Exam	Day(s) Relative to Start Date						
		-7	-6	-5	39	40	41	80
101	Left Eye	.	.	NADO	.	.	NADO	.
	Right Eye	.	.	NADO	.	.	NADO	.
102	Left Eye	.	.	NADO	.	.	NADO	.
	Right Eye	.	.	NADO	.	.	NADO	.

NADO=No Abnormal Findings

AmplifyBio Study Number A-579-01

Individual Animal Ophthalmic Exams

Group 1<br>Vehicle<br>Sex: Male	Observation Type: Ophthalmic Exam	Day(s) Relative to Start Date						
		81	82					
101	Left Eye	.	.					
	Right Eye	.	.					
102	Left Eye	NADO	.					
	Right Eye	NADO	.					

NADO=No Abnormal Findings

AmplifyBio Study Number A-579-01

Individual Animal Ophthalmic Exams

Group 2<br>Low<br>Sex: Male	Observation Type: Ophthalmic Exam	Day(s) Relative to Start Date						
		-7	-6	-5	39	40	41	80
201	Left Eye	.	NADO	.	.	NADO	.	.
	Right Eye	.	NADO	.	.	NADO	.	.
202	Left Eye	.	NADO	.	.	NADO	.	.
	Right Eye	.	NADO	.	.	NADO	.	.
203	Left Eye	.	NADO	.	.	NADO	.	NADO
	Right Eye	.	NADO	.	.	NADO	.	NADO
204	Left Eye	.	NADO	.	.	NADO	.	NADO
	Right Eye	.	NADO	.	.	NADO	.	NADO

NADO=No Abnormal Findings

AmplifyBio Study Number A-579-01

Individual Animal Ophthalmic Exams

Group 2<br>Low<br>Sex: Male	Observation Type: Ophthalmic Exam	Day(s) Relative to Start Date						
		81	82					
201	Left Eye	.	.					
	Right Eye	.	.					
202	Left Eye	.	.					
	Right Eye	.	.					
203	Left Eye	.	.					
	Right Eye	.	.					
204	Left Eye	.	.					
	Right Eye	.	.					

AmplifyBio Study Number A-579-01

Individual Animal Ophthalmic Exams

Group 3<br>High<br>Sex: Male	Observation Type: Ophthalmic Exam	Day(s) Relative to Start Date						
		-7	-6	-5	39	40	41	80
301	Left Eye	NADO	.	.	NADO	.	.	.
	Right Eye	NADO	.	.	NADO	.	.	.
302	Left Eye	NADO	.	.	NADO	.	.	.
	Right Eye	NADO	.	.	NADO	.	.	.
303	Left Eye	NADO	.	.	NADO	.	.	.
	Right Eye	NADO	.	.	NADO	.	.	.
304	Left Eye	NADO	.	.	NADO	.	.	.
	Right Eye	NADO	.	.	NADO	.	.	.

NADO=No Abnormal Findings

AmplifyBio Study Number A-579-01

Individual Animal Ophthalmic Exams

Group 3<br>High<br>Sex: Male	Observation Type: Ophthalmic Exam	Day(s) Relative to Start Date						
		81	82					
301	Left Eye	.	.					
	Right Eye	.	.					
302	Left Eye	.	.					
	Right Eye	.	.					
303	Left Eye	.	NADO					
	Right Eye	.	NADO					
304	Left Eye	.	NADO					
	Right Eye	.	NADO					

NADO=No Abnormal Findings

AmplifyBio Study Number A-579-01

Individual Animal Ophthalmic Exams

Group 1<br>Vehicle<br>Sex: Female	Observation Type: Ophthalmic Exam	Day(s) Relative to Start Date						
		-7	-6	-5	39	40	41	80
151 !	Left Eye	.	.	Cat	.	.	Cat	.
	Right Eye	.	.	Cat	.	.	Cat	.
152	Left Eye	.	.	NADO	.	.	NADO	.
	Right Eye	.	.	NADO	.	.	NADO	.

!=Result comment recorded against 1 or more clinical observations. Cat=Cataract; NADO=No Abnormal Findings

AmplifyBio Study Number A-579-01

Individual Animal Ophthalmic Exams

Group 1<br>Vehicle<br>Sex: Female	Observation Type: Ophthalmic Exam	Day(s) Relative to Start Date						
		81	82					
151	Left Eye	.	.					
	Right Eye	.	.					
152	Left Eye	NADO	.					
	Right Eye	NADO	.					

NADO=No Abnormal Findings

9/13/2023 5:59:08AM Page: 9

AmplifyBio Study Number A-579-01

Individual Animal Ophthalmic Exams

Group 2<br>Low<br>Sex: Female	Observation Type: Ophthalmic Exam	Day(s) Relative to Start Date						
		-7	-6	-5	39	40	41	80
251	Left Eye	.	NADO	.	.	NADO	.	.
	Right Eye	.	NADO	.	.	NADO	.	.
252	Left Eye	.	NADO	.	.	NADO	.	.
	Right Eye	.	NADO	.	.	NADO	.	.
253	Left Eye	.	NADO	.	.	NADO	.	NADO
	Right Eye	.	NADO	.	.	NADO	.	NADO
254	Left Eye	.	NADO	.	.	NADO	.	NADO
	Right Eye	.	NADO	.	.	NADO	.	NADO

NADO=No Abnormal Findings

9/13/2023 5:59:08AM Page: 10

AmplifyBio Study Number A-579-01

Individual Animal Ophthalmic Exams

Group 2<br>Low<br>Sex: Female	Observation Type: Ophthalmic Exam	Day(s) Relative to Start Date						
		81	82					
251	Left Eye	.	.					
	Right Eye	.	.					
252	Left Eye	.	.					
	Right Eye	.	.					
253	Left Eye	.	.					
	Right Eye	.	.					
254	Left Eye	.	.					
	Right Eye	.	.					

AmplifyBio Study Number A-579-01

Individual Animal Ophthalmic Exams

Group 3<br>High<br>Sex: Female	Observation Type: Ophthalmic Exam	Day(s) Relative to Start Date						
		-7	-6	-5	39	40	41	80
351	Left Eye	NADO	.	.	NADO	.	.	.
	Right Eye	NADO	.	.	NADO	.	.	.
352	Left Eye	NADO	.	.	NADO	.	.	.
	Right Eye	NADO	.	.	NADO	.	.	.
353	Left Eye	NADO	.	.	NADO	.	.	.
	Right Eye	NADO	.	.	NADO	.	.	.
354	Left Eye	NADO	.	.	NADO	.	.	.
	Right Eye	NADO	.	.	NADO	.	.	.

NADO=No Abnormal Findings

AmplifyBio Study Number A-579-01

Individual Animal Ophthalmic Exams

Group 3<br>High<br>Sex: Female	Observation Type: Ophthalmic Exam	Day(s) Relative to Start Date						
		81	82					
351	Left Eye	.	.					
	Right Eye	.	.					
352	Left Eye	.	.					
	Right Eye	.	.					
353	Left Eye	.	NADO					
	Right Eye	.	NADO					
354	Left Eye	.	NADO					
	Right Eye	.	NADO					

NADO=No Abnormal Findings

AmplifyBio Study Number A-579-01

Individual Animal Ophthalmic Exams

Comment Information

Group	Sex	Animal	Day	Observation Type	Comment
Group 1	Female	151	-5	Ophthalmic Exam	grade 1
Group 1	Female	151	41	Ophthalmic Exam	grade 1

AmplifyBio Study Number A-579-01

Individual Animal Ophthalmic Exams

Key Page

Group Information

Short Name	Long Name	Type	Report Headings	
1	Group 1	Control	Group 1	Vehicle
2	Group 2	Dose	Group 2	Low
3	Group 3	Dose	Group 3	High

Appendix F Neurobehavioral Contributing Scientist Report

A 12-Week Single Dose Biodistribution and Safety Study in Cynomolgus Monkeys

Neurobehavioral Contributing Scientist Report

May 30, 2024

Study Number A-579-01

Testing Facility
AmplifyBio
1425 State Route 142 (Plain City-Georgesville Road)
West Jefferson, OH 43162

SIGNATURES

Prepared by:

DocuSigned by:

Michael Stonerook

Signer Name: Michael Stonerook
Signing Reason: I am the author of this document
Signing Time: 5/30/2024 | 1:27:15 PM EDT
63C2D48DB6B3417288EEA3C794CB2F31

5/30/2024

Michael Stonerook, PhD, DVM, DABT
Study Neurobehavioral Scientist

Date

TABLE OF CONTENTS

Page

Signatures 2

1.0 Introduction 4

2.0 Study Design and Methods 4

3.0 Non-Invasive Functional Neurological Observations 4

3.1 Statistical Analysis 5

4.0 Deviations 5

5.0 Results 5

6.0 Conclusions 7

LIST OF ATTACHMENTS

Attachment 1. Summary Tables 8

Attachment 2. Individual Tables 35

Attachment 3. Study Specific Method 101

1.0 INTRODUCTION

This contributing scientist report summarizes the neurobehavioral findings during the Non-Invasive Functional Neurological Observations (NIFNO) for Study No. A-579-01. The purpose of this study was to evaluate the potential biodistribution and safety of scAAV9.U6miR871 (ARM-101) when administered once via intrathecal infusion to Cynomolgus monkeys for up to 12 weeks.

2.0 STUDY DESIGN AND METHODS

The experimental design relating to neurobehavioral evaluations is outlined in Table 1.

Table 1. Experimental Design

Group	Target Dose (Total vg)	Target Dose Volume (mL)	Animal Identification Numbers			
			6-Week Cohort[a]		12-Week Cohort[b]	
			Male	Female	Male	Female
1 – Control	0	3	101	151	102	152
2 – Low	6×10^{13}	3	201-202	251-252	203-204	253-254
3 – High	1.2×10^{14}	3	301-302	351-352	303-304	353-354

vg=vector genome
a. Necropsy Day 43.
b. Necropsy Day 85.

All groups were dosed on Day 1 via a single intrathecal slow infusion over approximately 6 hours via a surgically implanted lumbar catheter (see QE-001542 for exceptions).

3.0 NON-INVASIVE FUNCTIONAL NEUROLOGICAL OBSERVATIONS

Neurobehavior was assessed using cage-side Non-Invasive Functional Neurological Observations (NIFNO) assessments. NIFNO assessments were performed on all animals once prior to Day 1 and on all animals assigned to study once per study week thereafter. Assessments were conducted by staff trained in performing neurobehavioral evaluations.

Assessments were performed as presented in Table 2. Details of these parameters are presented in Attachment 3.

Table 2. NIFNO Parameters

Mentation/Behavior	Facial Muscle Symmetry	Repetitive Motions or Movements
General Activity	Salivation	Stargazing
Posture	Lacrimation	Piloerection
Voluntary Movement/Gait	Eyelid Closure	Urine Stain
Vocalizations	Pupillary Diameter	Fecal Stain
Tremor Activity	Self-mutilation	Nasal Discharge
Convulsive Activity	Uncontrolled Retropulsion	

3.1 Statistical Analysis

Neurobehavioral observations were reported as individual events. No statistical analysis was performed.

4.0 DEVIATIONS

QE-001542: On Day 1, the dose administration was not exactly 6 hours for all three groups.

For Group 1, Animal 101 had dose administered starting at 0957. There is documentation that the pump was paused at 1414 due to the Huber needle detaching from the port, however, there is no documentation of when the pump was restarted following this pause. It is unclear how long the dose was administered with the pump stop time documented at 1632. For the other Group 1 animals, the dose administration time was as followed: Animal 102 (5hrs 58mins), Animal 151 (5hrs 56mins), and Animal 152 (5hrs 3mins).

For Group 2, the dose administration time for each animal is as followed: Animal 201 (5hrs 56mins), Animal 202 (5hrs 56mins), Animal 203 (5hrs 57mins), Animal 204 (5hrs 55mins), Animal 251 (5hrs 56mins), Animal 252 (5hrs 54mins), Animal 253 (5hrs 56mins), and Animal 254 (5hrs 55mins).

For Group 3, the dose administration time for each animal is as followed: Animal 301 (5hrs 55mins), Animal 302 (5hrs 55mins), Animal 303 (5hrs 51mins), Animal 304 (6hrs 2mins), Animal 351 (5hrs 56mins), Animal 352 (5hrs 56mins), Animal 353 (5hrs 57mins), and Animal 354 (5hrs 58mins).

The impact of +/- 10 minutes on a 6 hour treatment is not likely to have an impact. The failed attempt to give full treatment to Animal 152 will have no effect since this animal was a control and not receiving test article.

5.0 RESULTS

Summary Tables: Attachment 1
Individual Tables: Attachment 2
Study Specific Method: Attachment 3

The parameters that were evaluated had findings mainly related to general activity, repetitive movements, and stargazing, each of which was seen in males and females administered control (Formulation Buffer) or the test article scAAV9.U6miR871 (ARM-101). Other findings such as piloerection and self-mutilation were observed in one Group 2 female (Table 3).

Repetitive motions or movements are considered a stereotypic behavior observed in a subpopulation of male and female cynomolgus macaques in a research setting. There are a number of factors that may influence an unpredictable stereotypic behavior, such as number of

sedations and blood collections. Events of routine husbandry activities such as feeding and sedation may also be stressful and have an impact on the levels of stereotypic behaviors.

Stargazing is a complex behavior that involves an interaction of both cognitive and social behaviors and was observed only in one male each of both low and high dose during the study period. Similarly, changes in mentation/behavior and general activity have a number of factors that can influence the variability of these parameters that include the contents of the cage, cage location, cage size, environmental enrichment, and social interactions that routinely vary.

Results from the study indicates the changes in the score were ambiguous and merely a change of a score by a factor of 1 in animals both in the control group as well as in the those receiving scAAV9.U6miR871 (ARM-101).

Table 3. Summary of NIFNO Assessment Findings

Animal Number	Parameter – Observation	Score	Timepoint Following Post-Dose Administration (Pretest or Week Number)
102	General Activity – Minimal Activity	-1	Pretest, 9, and 11
	Repetitive Motions or Movements – Present	1	2, 4, and 5
152	General Activity – Minimal Activity	-1	1, 5, 7, 8, 9, 10, 11 and 12
204	Repetitive Motions or Movements – Present	1	Pretest, 1, 2, 4, 6, 7, 8, 9, 10, 11, and 12
	Stargazing – Present	1	Pretest, 2, 3, 4, 5, 6, 7, 8, 10, and 12
251	General Activity – Minimal Activity	-1	Pretest
	General Activity – Heightened	1	6
	Repetitive Motions or Movements – Present	1	6
253	Self-mutilation – Present	1	4
	Piloerection – Present	1	2
254	General Activity – Minimal Activity	-1	11
302	Mentation/Behavior – Anxious, enhanced arousal	1	Pretest
303	Vocalizations – Moderate, distressed	1	Pretest
	Repetitive Motions or Movements – Present	1	3
304	Repetitive Motions or Movements – Present	1	1
	Stargazing – Present	1	1, 2, 3, 4, 5, 6, 7, 8, 10, and 12
351	General Activity – Heightened	1	Pretest
	Repetitive Motions or Movements – Present	1	Pretest
353	General Activity – Minimal Activity	-1	2, 3, 5, 6, and 10
	Repetitive Motions or Movements – Present	1	7
354	Mentation/Behavior – Anxious, enhanced arousal	1	Pretest

6.0 CONCLUSIONS

A Non-Invasive Functional Neurological Observation method was used to assess cynomolgus monkeys once prior to Day 1 and weekly following a single intrathecal infusion of scAAV9.U6miR871 (ARM-101).

Dose administration of scAAV9.U6miR871 (ARM-101) administered as a single approximate 6-hour intrathecal infusion of 6×10^{13} or 1.2×10^{14} total vg/dose in male or female cynomolgus monkeys produced no neurological based observations that were test article-related.

Attachment 1. Summary Tables

AmplifyBio Study Number: A-579-01

Summary of Non-Invasive Functional Neurological Observations (6-Week and 12-Week Cohort Animals)

Pre-Test
Males

Parameter	Scores Observed	Count Percentage (%) Group 1 0 vg	Group 2 6 × 10^{13} vg	Group 3 1.2 × 10^{14} vg
Mentation/Behavior	0<br>1	2 (100.0%)<br>0 (0.0%)	4 (100.0%)<br>0 (0.0%)	3 (75.0%)<br>1 (25.0%)
General Activity	-1<br>0	1 (50.0%)<br>1 (50.0%)	4 (100.0%)<br>0 (0.0%)	4 (100.0%)<br>0 (0.0%)
Posture	0	2 (100.0%)	4 (100.0%)	4 (100.0%)
Voluntary Movement/Gait	0	2 (100.0%)	4 (100.0%)	4 (100.0%)
Vocalizations	0<br>1	2 (100.0%)<br>0 (0.0%)	4 (100.0%)<br>0 (0.0%)	3 (75.0%)<br>1 (25.0%)
Tremor Activity	0	2 (100.0%)	4 (100.0%)	4 (100.0%)
Convulsive Activity	0	2 (100.0%)	4 (100.0%)	4 (100.0%)
Facial Muscle Symmetry	0	2 (100.0%)	4 (100.0%)	4 (100.0%)
Salivation	0	2 (100.0%)	4 (100.0%)	4 (100.0%)
Lacrimation – Left Eye	0	2 (100.0%)	4 (100.0%)	4 (100.0%)
Lacrimation – Right Eye	0	2 (100.0%)	4 (100.0%)	4 (100.0%)
Eyelid Closure – Left Eye	0	2 (100.0%)	4 (100.0%)	4 (100.0%)
Eyelid Closure – Right Eye	0	2 (100.0%)	4 (100.0%)	4 (100.0%)
Pupillary Diameter – Left Eye	0	2 (100.0%)	4 (100.0%)	4 (100.0%)
Pupillary Diameter – Right Eye	0	2 (100.0%)	4 (100.0%)	4 (100.0%)
Self-Mutilation	0	2 (100.0%)	4 (100.0%)	4 (100.0%)
Uncontrolled Retropulsion	0	2 (100.0%)	4 (100.0%)	4 (100.0%)
Repetitive Motions or Movements	0<br>1	2 (100.0%)<br>0 (0.0%)	3 (75.0%)<br>1 (25.0%)	4 (100.0%)<br>0 (0.0%)
Stargazing	0<br>1	2 (100.0%)<br>0 (0.0%)	3 (75.0%)<br>1 (25.0%)	4 (100.0%)<br>0 (0.0%)
Piloerection	0	2 (100.0%)	4 (100.0%)	4 (100.0%)
Urine Stain	0	2 (100.0%)	4 (100.0%)	4 (100.0%)
Fecal Stain	0	2 (100.0%)	4 (100.0%)	4 (100.0%)
Nasal Discharge	0	2 (100.0%)	4 (100.0%)	4 (100.0%)

AmplifyBio Study Number: A-579-01

Summary of Non-Invasive Functional Neurological Observations (6-Week and 12-Week Cohort Animals)

Pre-Test
Females

Parameter	Scores Observed	Count Percentage (%) Group 1 0 vg	Group 2 6×10^{13} vg	Group 3 1.2×10^{14} vg
Mentation/Behavior	0	2 (100.0%)	4 (100.0%)	3 (75.0%)
	1	0 (0.0%)	0 (0.0%)	1 (25.0%)
General Activity	-1	0 (0.0%)	1 (25.0%)	0 (0.0%)
	0	2 (100.0%)	3 (75.0%)	3 (75.0%)
	1	0 (0.0%)	0 (0.0%)	1 (25.0%)
Posture	0	2 (100.0%)	4 (100.0%)	4 (100.0%)
Voluntary Movement/Gait	0	2 (100.0%)	4 (100.0%)	4 (100.0%)
Vocalizations	0	2 (100.0%)	4 (100.0%)	4 (100.0%)
Tremor Activity	0	2 (100.0%)	4 (100.0%)	4 (100.0%)
Convulsive Activity	0	2 (100.0%)	4 (100.0%)	4 (100.0%)
Facial Muscle Symmetry	0	2 (100.0%)	4 (100.0%)	4 (100.0%)
Salivation	0	2 (100.0%)	4 (100.0%)	4 (100.0%)
Lacrimation – Left Eye	0	2 (100.0%)	4 (100.0%)	4 (100.0%)
Lacrimation – Right Eye	0	2 (100.0%)	4 (100.0%)	4 (100.0%)
Eyelid Closure – Left Eye	0	2 (100.0%)	4 (100.0%)	4 (100.0%)
Eyelid Closure – Right Eye	0	2 (100.0%)	4 (100.0%)	4 (100.0%)
Pupillary Diameter – Left Eye	0	2 (100.0%)	4 (100.0%)	4 (100.0%)
Pupillary Diameter – Right Eye	0	2 (100.0%)	4 (100.0%)	4 (100.0%)
Self-Mutilation	0	2 (100.0%)	4 (100.0%)	4 (100.0%)
Uncontrolled Retropulsion	0	2 (100.0%)	4 (100.0%)	4 (100.0%)
Repetitive Motions or Movements	0	2 (100.0%)	4 (100.0%)	3 (75.0%)
	1	0 (0.0%)	0 (0.0%)	1 (25.0%)
Stargazing	0	2 (100.0%)	4 (100.0%)	4 (100.0%)
Piloerection	0	2 (100.0%)	4 (100.0%)	4 (100.0%)
Urine Stain	0	2 (100.0%)	4 (100.0%)	4 (100.0%)
Fecal Stain	0	2 (100.0%)	4 (100.0%)	4 (100.0%)
Nasal Discharge	0	2 (100.0%)	4 (100.0%)	4 (100.0%)

AmplifyBio Study Number: A-579-01

Summary of Non-Invasive Functional Neurological Observations (6-Week and 12-Week Cohort Animals)

Week 1
Males

Parameter	Scores Observed	Count Percentage (%) Group 1 0 vg	Group 2 6×10^{13} vg	Group 3 1.2×10^{14} vg
Mentation/Behavior	0	2 (100.0%)	4 (100.0%)	4 (100.0%)
General Activity	0	2 (100.0%)	4 (100.0%)	4 (100.0%)
Posture	0	2 (100.0%)	4 (100.0%)	4 (100.0%)
Voluntary Movement/Gait	0	2 (100.0%)	4 (100.0%)	4 (100.0%)
Vocalizations	0	2 (100.0%)	4 (100.0%)	4 (100.0%)
Tremor Activity	0	2 (100.0%)	4 (100.0%)	4 (100.0%)
Convulsive Activity	0	2 (100.0%)	4 (100.0%)	4 (100.0%)
Facial Muscle Symmetry	0	2 (100.0%)	4 (100.0%)	4 (100.0%)
Salivation	0	2 (100.0%)	4 (100.0%)	4 (100.0%)
Lacrimation – Left Eye	0	2 (100.0%)	4 (100.0%)	4 (100.0%)
Lacrimation – Right Eye	0	2 (100.0%)	4 (100.0%)	4 (100.0%)
Eyelid Closure – Left Eye	0	2 (100.0%)	4 (100.0%)	4 (100.0%)
Eyelid Closure – Right Eye	0	2 (100.0%)	4 (100.0%)	4 (100.0%)
Pupillary Diameter – Left Eye	0	2 (100.0%)	4 (100.0%)	4 (100.0%)
Pupillary Diameter – Right Eye	0	2 (100.0%)	4 (100.0%)	4 (100.0%)
Self-Mutilation	0	2 (100.0%)	4 (100.0%)	4 (100.0%)
Uncontrolled Retropulsion	0	2 (100.0%)	4 (100.0%)	4 (100.0%)
Repetitive Motions or Movements	0<br>1	2 (100.0%)<br>0 (0.0%)	3 (75.0%)<br>1 (25.0%)	3 (75.0%)<br>1 (25.0%)
Stargazing	0<br>1	2 (100.0%)<br>0 (0.0%)	4 (100.0%)<br>0 (0.0%)	3 (75.0%)<br>1 (25.0%)
Piloerection	0	2 (100.0%)	4 (100.0%)	4 (100.0%)
Urine Stain	0	2 (100.0%)	4 (100.0%)	4 (100.0%)
Fecal Stain	0	2 (100.0%)	4 (100.0%)	4 (100.0%)
Nasal Discharge	0	2 (100.0%)	4 (100.0%)	4 (100.0%)

AmplifyBio Study Number: A-579-01

Summary of Non-Invasive Functional Neurological Observations (6-Week and 12-Week Cohort Animals)

Week 1
Females

Parameter	Scores Observed	Count Percentage (%)		
		Group 1<br>0 vg	Group 2<br>6×10^{13} vg	Group 3<br>1.2×10^{14} vg
**Mentation/Behavior**	0	2 (100.0%)	4 (100.0%)	4 (100.0%)
**General Activity**	-1<br>0	1 (50.0%)<br>1 (50.0%)	0 (0.0%)<br>4 (100.0%)	0 (0.0%)<br>4 (100.0%)
**Posture**	0	2 (100.0%)	4 (100.0%)	4 (100.0%)
**Voluntary Movement/Gait**	0	2 (100.0%)	4 (100.0%)	4 (100.0%)
**Vocalizations**	0	2 (100.0%)	4 (100.0%)	4 (100.0%)
**Tremor Activity**	0	2 (100.0%)	4 (100.0%)	4 (100.0%)
**Convulsive Activity**	0	2 (100.0%)	4 (100.0%)	4 (100.0%)
**Facial Muscle Symmetry**	0	2 (100.0%)	4 (100.0%)	4 (100.0%)
**Salivation**	0	2 (100.0%)	4 (100.0%)	4 (100.0%)
**Lacrimation – Left Eye**	0	2 (100.0%)	4 (100.0%)	4 (100.0%)
**Lacrimation – Right Eye**	0	2 (100.0%)	4 (100.0%)	4 (100.0%)
**Eyelid Closure – Left Eye**	0	2 (100.0%)	4 (100.0%)	4 (100.0%)
**Eyelid Closure – Right Eye**	0	2 (100.0%)	4 (100.0%)	4 (100.0%)
**Pupillary Diameter – Left Eye**	0	2 (100.0%)	4 (100.0%)	4 (100.0%)
**Pupillary Diameter – Right Eye**	0	2 (100.0%)	4 (100.0%)	4 (100.0%)
**Self-Mutilation**	0	2 (100.0%)	4 (100.0%)	4 (100.0%)
**Uncontrolled Retropulsion**	0	2 (100.0%)	4 (100.0%)	4 (100.0%)
**Repetitive Motions or Movements**	0	2 (100.0%)	4 (100.0%)	4 (100.0%)
**Stargazing**	0	2 (100.0%)	4 (100.0%)	4 (100.0%)
**Piloerection**	0	2 (100.0%)	4 (100.0%)	4 (100.0%)
**Urine Stain**	0	2 (100.0%)	4 (100.0%)	4 (100.0%)
**Fecal Stain**	0	2 (100.0%)	4 (100.0%)	4 (100.0%)
**Nasal Discharge**	0	2 (100.0%)	4 (100.0%)	4 (100.0%)

AmplifyBio Study Number: A-579-01

Summary of Non-Invasive Functional Neurological Observations
(6-Week and 12-Week Cohort Animals)

Week 2
Males

Parameter	Scores Observed	Count Percentage (%)		
		Group 1<br>0 vg	Group 2<br>6×10^{13} vg	Group 3<br>1.2×10^{14} vg
Mentation/Behavior	0	2 (100.0%)	4 (100.0%)	4 (100.0%)
General Activity	0	2 (100.0%)	4 (100.0%)	4 (100.0%)
Posture	0	2 (100.0%)	4 (100.0%)	4 (100.0%)
Voluntary Movement/Gait	0	2 (100.0%)	4 (100.0%)	4 (100.0%)
Vocalizations	0	2 (100.0%)	4 (100.0%)	4 (100.0%)
Tremor Activity	0	2 (100.0%)	4 (100.0%)	4 (100.0%)
Convulsive Activity	0	2 (100.0%)	4 (100.0%)	4 (100.0%)
Facial Muscle Symmetry	0	2 (100.0%)	4 (100.0%)	4 (100.0%)
Salivation	0	2 (100.0%)	4 (100.0%)	4 (100.0%)
Lacrimation – Left Eye	0	2 (100.0%)	4 (100.0%)	4 (100.0%)
Lacrimation – Right Eye	0	2 (100.0%)	4 (100.0%)	4 (100.0%)
Eyelid Closure – Left Eye	0	2 (100.0%)	4 (100.0%)	4 (100.0%)
Eyelid Closure – Right Eye	0	2 (100.0%)	4 (100.0%)	4 (100.0%)
Pupillary Diameter – Left Eye	0	2 (100.0%)	4 (100.0%)	4 (100.0%)
Pupillary Diameter – Right Eye	0	2 (100.0%)	4 (100.0%)	4 (100.0%)
Self-Mutilation	0	2 (100.0%)	4 (100.0%)	4 (100.0%)
Uncontrolled Retropulsion	0	2 (100.0%)	4 (100.0%)	4 (100.0%)
Repetitive Motions or Movements	0<br>1	1 (50.0%)<br>1 (50.0%)	3 (75.0%)<br>1 (25.0%)	4 (100.0%)<br>0 (0.0%)
Stargazing	0<br>1	2 (100.0%)<br>0 (0.0%)	3 (75.0%)<br>1 (25.0%)	3 (75.0%)<br>1 (25.0%)
Piloerection	0	2 (100.0%)	4 (100.0%)	4 (100.0%)
Urine Stain	0	2 (100.0%)	4 (100.0%)	4 (100.0%)
Fecal Stain	0	2 (100.0%)	4 (100.0%)	4 (100.0%)
Nasal Discharge	0	2 (100.0%)	4 (100.0%)	4 (100.0%)

AmplifyBio Study Number: A-579-01

Summary of Non-Invasive Functional Neurological Observations (6-Week and 12-Week Cohort Animals)

Week 2
Females

Parameter	Scores Observed	Count Percentage (%)		
		Group 1<br>0 vg	Group 2<br>6×10^{13} vg	Group 3<br>1.2×10^{14} vg
**Mentation/Behavior**	0	2 (100.0%)	4 (100.0%)	4 (100.0%)
**General Activity**	-1<br>0	0 (0.0%)<br>2 (100.0%)	0 (0.0%)<br>4 (100.0%)	1 (25.0%)<br>3 (75.0%)
**Posture**	0	2 (100.0%)	4 (100.0%)	4 (100.0%)
**Voluntary Movement/Gait**	0	2 (100.0%)	4 (100.0%)	4 (100.0%)
**Vocalizations**	0	2 (100.0%)	4 (100.0%)	4 (100.0%)
**Tremor Activity**	0	2 (100.0%)	4 (100.0%)	4 (100.0%)
**Convulsive Activity**	0	2 (100.0%)	4 (100.0%)	4 (100.0%)
**Facial Muscle Symmetry**	0	2 (100.0%)	4 (100.0%)	4 (100.0%)
**Salivation**	0	2 (100.0%)	4 (100.0%)	4 (100.0%)
**Lacrimation – Left Eye**	0	2 (100.0%)	4 (100.0%)	4 (100.0%)
**Lacrimation – Right Eye**	0	2 (100.0%)	4 (100.0%)	4 (100.0%)
**Eyelid Closure – Left Eye**	0	2 (100.0%)	4 (100.0%)	4 (100.0%)
**Eyelid Closure – Right Eye**	0	2 (100.0%)	4 (100.0%)	4 (100.0%)
**Pupillary Diameter – Left Eye**	0	2 (100.0%)	4 (100.0%)	4 (100.0%)
**Pupillary Diameter – Right Eye**	0	2 (100.0%)	4 (100.0%)	4 (100.0%)
**Self-Mutilation**	0	2 (100.0%)	4 (100.0%)	4 (100.0%)
**Uncontrolled Retropulsion**	0	2 (100.0%)	4 (100.0%)	4 (100.0%)
**Repetitive Motions or Movements**	0	2 (100.0%)	4 (100.0%)	4 (100.0%)
**Stargazing**	0	2 (100.0%)	4 (100.0%)	4 (100.0%)
**Piloerection**	0<br>1	2 (100.0%)<br>0 (0.0%)	3 (75.0%)<br>1 (25.0%)	4 (100.0%)<br>0 (0.0%)
**Urine Stain**	0	2 (100.0%)	4 (100.0%)	4 (100.0%)
**Fecal Stain**	0	2 (100.0%)	4 (100.0%)	4 (100.0%)
**Nasal Discharge**	0	2 (100.0%)	4 (100.0%)	4 (100.0%)

AmplifyBio Study Number: A-579-01

Summary of Non-Invasive Functional Neurological Observations (6-Week and 12-Week Cohort Animals)

Week 3
Males

Parameter	Scores Observed	Count Percentage (%)		
		Group 1<br>0 vg	Group 2<br>6×10^{13} vg	Group 3<br>1.2×10^{14} vg
Mentation/Behavior	0	2 (100.0%)	4 (100.0%)	4 (100.0%)
General Activity	0	2 (100.0%)	4 (100.0%)	4 (100.0%)
Posture	0	2 (100.0%)	4 (100.0%)	4 (100.0%)
Voluntary Movement/Gait	0	2 (100.0%)	4 (100.0%)	4 (100.0%)
Vocalizations	0	2 (100.0%)	4 (100.0%)	4 (100.0%)
Tremor Activity	0	2 (100.0%)	4 (100.0%)	4 (100.0%)
Convulsive Activity	0	2 (100.0%)	4 (100.0%)	4 (100.0%)
Facial Muscle Symmetry	0	2 (100.0%)	4 (100.0%)	4 (100.0%)
Salivation	0	2 (100.0%)	4 (100.0%)	4 (100.0%)
Lacrimation – Left Eye	0	2 (100.0%)	4 (100.0%)	4 (100.0%)
Lacrimation – Right Eye	0	2 (100.0%)	4 (100.0%)	4 (100.0%)
Eyelid Closure – Left Eye	0	2 (100.0%)	4 (100.0%)	4 (100.0%)
Eyelid Closure – Right Eye	0	2 (100.0%)	4 (100.0%)	4 (100.0%)
Pupillary Diameter – Left Eye	0	2 (100.0%)	4 (100.0%)	4 (100.0%)
Pupillary Diameter – Right Eye	0	2 (100.0%)	4 (100.0%)	4 (100.0%)
Self-Mutilation	0	2 (100.0%)	4 (100.0%)	4 (100.0%)
Uncontrolled Retropulsion	0	2 (100.0%)	4 (100.0%)	4 (100.0%)
Repetitive Motions or Movements	0<br>1	2 (100.0%)<br>0 (0.0%)	4 (100.0%)<br>0 (0.0%)	3 (75.0%)<br>1 (25.0%)
Stargazing	0<br>1	2 (100.0%)<br>0 (0.0%)	3 (75.0%)<br>1 (25.0%)	3 (75.0%)<br>1 (25.0%)
Piloerection	0	2 (100.0%)	4 (100.0%)	4 (100.0%)
Urine Stain	0	2 (100.0%)	4 (100.0%)	4 (100.0%)
Fecal Stain	0	2 (100.0%)	4 (100.0%)	4 (100.0%)
Nasal Discharge	0	2 (100.0%)	4 (100.0%)	4 (100.0%)

AmplifyBio Study Number: A-579-01

Summary of Non-Invasive Functional Neurological Observations (6-Week and 12-Week Cohort Animals)

Week 3
Females

Parameter	Scores Observed	Count Percentage (%)		
		Group 1<br>0 vg	Group 2<br>6×10^{13} vg	Group 3<br>1.2×10^{14} vg
Mentation/Behavior	0	2 (100.0%)	4 (100.0%)	4 (100.0%)
General Activity	-1<br>0	0 (0.0%)<br>2 (100.0%)	0 (0.0%)<br>4 (100.0%)	1 (25.0%)<br>3 (75.0%)
Posture	0	2 (100.0%)	4 (100.0%)	4 (100.0%)
Voluntary Movement/Gait	0	2 (100.0%)	4 (100.0%)	4 (100.0%)
Vocalizations	0	2 (100.0%)	4 (100.0%)	4 (100.0%)
Tremor Activity	0	2 (100.0%)	4 (100.0%)	4 (100.0%)
Convulsive Activity	0	2 (100.0%)	4 (100.0%)	4 (100.0%)
Facial Muscle Symmetry	0	2 (100.0%)	4 (100.0%)	4 (100.0%)
Salivation	0	2 (100.0%)	4 (100.0%)	4 (100.0%)
Lacrimation – Left Eye	0	2 (100.0%)	4 (100.0%)	4 (100.0%)
Lacrimation – Right Eye	0	2 (100.0%)	4 (100.0%)	4 (100.0%)
Eyelid Closure – Left Eye	0	2 (100.0%)	4 (100.0%)	4 (100.0%)
Eyelid Closure – Right Eye	0	2 (100.0%)	4 (100.0%)	4 (100.0%)
Pupillary Diameter – Left Eye	0	2 (100.0%)	4 (100.0%)	4 (100.0%)
Pupillary Diameter – Right Eye	0	2 (100.0%)	4 (100.0%)	4 (100.0%)
Self-Mutilation	0	2 (100.0%)	4 (100.0%)	4 (100.0%)
Uncontrolled Retropulsion	0	2 (100.0%)	4 (100.0%)	4 (100.0%)
Repetitive Motions or Movements	0	2 (100.0%)	4 (100.0%)	4 (100.0%)
Stargazing	0	2 (100.0%)	4 (100.0%)	4 (100.0%)
Piloerection	0	2 (100.0%)	4 (100.0%)	4 (100.0%)
Urine Stain	0	2 (100.0%)	4 (100.0%)	4 (100.0%)
Fecal Stain	0	2 (100.0%)	4 (100.0%)	4 (100.0%)
Nasal Discharge	0	2 (100.0%)	4 (100.0%)	4 (100.0%)

AmplifyBio Study Number: A-579-01

Summary of Non-Invasive Functional Neurological Observations
(6-Week and 12-Week Cohort Animals)

Week 4
Males

Parameter	Scores Observed	Count Percentage (%) Group 1 0 vg	Group 2 6×10^{13} vg	Group 3 1.2×10^{14} vg
Mentation/Behavior	0	2 (100.0%)	4 (100.0%)	4 (100.0%)
General Activity	0	2 (100.0%)	4 (100.0%)	4 (100.0%)
Posture	0	2 (100.0%)	4 (100.0%)	4 (100.0%)
Voluntary Movement/Gait	0	2 (100.0%)	4 (100.0%)	4 (100.0%)
Vocalizations	0	2 (100.0%)	4 (100.0%)	4 (100.0%)
Tremor Activity	0	2 (100.0%)	4 (100.0%)	4 (100.0%)
Convulsive Activity	0	2 (100.0%)	4 (100.0%)	4 (100.0%)
Facial Muscle Symmetry	0	2 (100.0%)	4 (100.0%)	4 (100.0%)
Salivation	0	2 (100.0%)	4 (100.0%)	4 (100.0%)
Lacrimation – Left Eye	0	2 (100.0%)	4 (100.0%)	4 (100.0%)
Lacrimation – Right Eye	0	2 (100.0%)	4 (100.0%)	4 (100.0%)
Eyelid Closure – Left Eye	0	2 (100.0%)	4 (100.0%)	4 (100.0%)
Eyelid Closure – Right Eye	0	2 (100.0%)	4 (100.0%)	4 (100.0%)
Pupillary Diameter – Left Eye	0	2 (100.0%)	4 (100.0%)	4 (100.0%)
Pupillary Diameter – Right Eye	0	2 (100.0%)	4 (100.0%)	4 (100.0%)
Self-Mutilation	0	2 (100.0%)	4 (100.0%)	4 (100.0%)
Uncontrolled Retropulsion	0	2 (100.0%)	4 (100.0%)	4 (100.0%)
Repetitive Motions or Movements	0<br>1	1 (50.0%)<br>1 (50.0%)	3 (75.0%)<br>1 (25.0%)	4 (100.0%)<br>0 (0.0%)
Stargazing	0<br>1	2 (100.0%)<br>0 (0.0%)	3 (75.0%)<br>1 (25.0%)	3 (75.0%)<br>1 (25.0%)
Piloerection	0	2 (100.0%)	4 (100.0%)	4 (100.0%)
Urine Stain	0	2 (100.0%)	4 (100.0%)	4 (100.0%)
Fecal Stain	0	2 (100.0%)	4 (100.0%)	4 (100.0%)
Nasal Discharge	0	2 (100.0%)	4 (100.0%)	4 (100.0%)

AmplifyBio Study Number: A-579-01

Summary of Non-Invasive Functional Neurological Observations (6-Week and 12-Week Cohort Animals)

Week 4
Females

Parameter	Scores Observed	Count Percentage (%)		
		Group 1<br>0 vg	Group 2<br>6×10^{13} vg	Group 3<br>1.2×10^{14} vg
Mentation/Behavior	0	2 (100.0%)	4 (100.0%)	4 (100.0%)
General Activity	0	2 (100.0%)	4 (100.0%)	4 (100.0%)
Posture	0	2 (100.0%)	4 (100.0%)	4 (100.0%)
Voluntary Movement/Gait	0	2 (100.0%)	4 (100.0%)	4 (100.0%)
Vocalizations	0	2 (100.0%)	4 (100.0%)	4 (100.0%)
Tremor Activity	0	2 (100.0%)	4 (100.0%)	4 (100.0%)
Convulsive Activity	0	2 (100.0%)	4 (100.0%)	4 (100.0%)
Facial Muscle Symmetry	0	2 (100.0%)	4 (100.0%)	4 (100.0%)
Salivation	0	2 (100.0%)	4 (100.0%)	4 (100.0%)
Lacrimation – Left Eye	0	2 (100.0%)	4 (100.0%)	4 (100.0%)
Lacrimation – Right Eye	0	2 (100.0%)	4 (100.0%)	4 (100.0%)
Eyelid Closure – Left Eye	0	2 (100.0%)	4 (100.0%)	4 (100.0%)
Eyelid Closure – Right Eye	0	2 (100.0%)	4 (100.0%)	4 (100.0%)
Pupillary Diameter – Left Eye	0	2 (100.0%)	4 (100.0%)	4 (100.0%)
Pupillary Diameter – Right Eye	0	2 (100.0%)	4 (100.0%)	4 (100.0%)
Self-Mutilation	0<br>1	2 (100.0%)<br>0 (0.0%)	3 (75.0%)<br>1 (25.0%)	4 (100.0%)<br>0 (0.0%)
Uncontrolled Retropulsion	0	2 (100.0%)	4 (100.0%)	4 (100.0%)
Repetitive Motions or Movements	0	2 (100.0%)	4 (100.0%)	4 (100.0%)
Stargazing	0	2 (100.0%)	4 (100.0%)	4 (100.0%)
Piloerection	0	2 (100.0%)	4 (100.0%)	4 (100.0%)
Urine Stain	0	2 (100.0%)	4 (100.0%)	4 (100.0%)
Fecal Stain	0	2 (100.0%)	4 (100.0%)	4 (100.0%)
Nasal Discharge	0	2 (100.0%)	4 (100.0%)	4 (100.0%)

AmplifyBio Study Number: A-579-01

Summary of Non-Invasive Functional Neurological Observations (6-Week and 12-Week Cohort Animals)

Week 5
Males

Parameter	Scores Observed	Count Percentage (%) Group 1 0 vg	Group 2 6×10^{13} vg	Group 3 1.2×10^{14} vg
**Mentation/Behavior**	0	2 (100.0%)	4 (100.0%)	4 (100.0%)
**General Activity**	0	2 (100.0%)	4 (100.0%)	4 (100.0%)
**Posture**	0	2 (100.0%)	4 (100.0%)	4 (100.0%)
**Voluntary Movement/Gait**	0	2 (100.0%)	4 (100.0%)	4 (100.0%)
**Vocalizations**	0	2 (100.0%)	4 (100.0%)	4 (100.0%)
**Tremor Activity**	0	2 (100.0%)	4 (100.0%)	4 (100.0%)
**Convulsive Activity**	0	2 (100.0%)	4 (100.0%)	4 (100.0%)
**Facial Muscle Symmetry**	0	2 (100.0%)	4 (100.0%)	4 (100.0%)
**Salivation**	0	2 (100.0%)	4 (100.0%)	4 (100.0%)
**Lacrimation – Left Eye**	0	2 (100.0%)	4 (100.0%)	4 (100.0%)
**Lacrimation – Right Eye**	0	2 (100.0%)	4 (100.0%)	4 (100.0%)
**Eyelid Closure – Left Eye**	0	2 (100.0%)	4 (100.0%)	4 (100.0%)
**Eyelid Closure – Right Eye**	0	2 (100.0%)	4 (100.0%)	4 (100.0%)
**Pupillary Diameter – Left Eye**	0	2 (100.0%)	4 (100.0%)	4 (100.0%)
**Pupillary Diameter – Right Eye**	0	2 (100.0%)	4 (100.0%)	4 (100.0%)
**Self-Mutilation**	0	2 (100.0%)	4 (100.0%)	4 (100.0%)
**Uncontrolled Retropulsion**	0	2 (100.0%)	4 (100.0%)	4 (100.0%)
**Repetitive Motions or Movements**	0	1 (50.0%)	4 (100.0%)	4 (100.0%)
	1	1 (50.0%)	0 (0.0%)	0 (0.0%)
**Stargazing**	0	2 (100.0%)	3 (75.0%)	3 (75.0%)
	1	0 (0.0%)	1 (25.0%)	1 (25.0%)
**Piloerection**	0	2 (100.0%)	4 (100.0%)	4 (100.0%)
**Urine Stain**	0	2 (100.0%)	4 (100.0%)	4 (100.0%)
**Fecal Stain**	0	2 (100.0%)	4 (100.0%)	4 (100.0%)
**Nasal Discharge**	0	2 (100.0%)	4 (100.0%)	4 (100.0%)

Page 1 of 2

AmplifyBio Study Number: A-579-01

Summary of Non-Invasive Functional Neurological Observations (6-Week and 12-Week Cohort Animals)

Week 5
Females

Parameter	Scores Observed	Count Percentage (%) Group 1 0 vg	Group 2 6×10^{13} vg	Group 3 1.2×10^{14} vg
Mentation/Behavior	0	2 (100.0%)	4 (100.0%)	4 (100.0%)
General Activity	-1<br>0	1 (50.0%)<br>1 (50.0%)	0 (0.0%)<br>4 (100.0%)	1 (25.0%)<br>3 (75.0%)
Posture	0	2 (100.0%)	4 (100.0%)	4 (100.0%)
Voluntary Movement/Gait	0	2 (100.0%)	4 (100.0%)	4 (100.0%)
Vocalizations	0	2 (100.0%)	4 (100.0%)	4 (100.0%)
Tremor Activity	0	2 (100.0%)	4 (100.0%)	4 (100.0%)
Convulsive Activity	0	2 (100.0%)	4 (100.0%)	4 (100.0%)
Facial Muscle Symmetry	0	2 (100.0%)	4 (100.0%)	4 (100.0%)
Salivation	0	2 (100.0%)	4 (100.0%)	4 (100.0%)
Lacrimation – Left Eye	0	2 (100.0%)	4 (100.0%)	4 (100.0%)
Lacrimation – Right Eye	0	2 (100.0%)	4 (100.0%)	4 (100.0%)
Eyelid Closure – Left Eye	0	2 (100.0%)	4 (100.0%)	4 (100.0%)
Eyelid Closure – Right Eye	0	2 (100.0%)	4 (100.0%)	4 (100.0%)
Pupillary Diameter – Left Eye	0	2 (100.0%)	4 (100.0%)	4 (100.0%)
Pupillary Diameter – Right Eye	0	2 (100.0%)	4 (100.0%)	4 (100.0%)
Self-Mutilation	0	2 (100.0%)	4 (100.0%)	4 (100.0%)
Uncontrolled Retropulsion	0	2 (100.0%)	4 (100.0%)	4 (100.0%)
Repetitive Motions or Movements	0	2 (100.0%)	4 (100.0%)	4 (100.0%)
Stargazing	0	2 (100.0%)	4 (100.0%)	4 (100.0%)
Piloerection	0	2 (100.0%)	4 (100.0%)	4 (100.0%)
Urine Stain	0	2 (100.0%)	4 (100.0%)	4 (100.0%)
Fecal Stain	0	2 (100.0%)	4 (100.0%)	4 (100.0%)
Nasal Discharge	0	2 (100.0%)	4 (100.0%)	4 (100.0%)

AmplifyBio Study Number: A-579-01

Summary of Non-Invasive Functional Neurological Observations (6-Week and 12-Week Cohort Animals)

Week 6
Males

Parameter	Scores Observed	Count Percentage (%) Group 1 0 vg	Group 2 6×10^{13} vg	Group 3 1.2×10^{14} vg
Mentation/Behavior	0	2 (100.0%)	4 (100.0%)	4 (100.0%)
General Activity	0	2 (100.0%)	4 (100.0%)	4 (100.0%)
Posture	0	2 (100.0%)	4 (100.0%)	4 (100.0%)
Voluntary Movement/Gait	0	2 (100.0%)	4 (100.0%)	4 (100.0%)
Vocalizations	0	2 (100.0%)	4 (100.0%)	4 (100.0%)
Tremor Activity	0	2 (100.0%)	4 (100.0%)	4 (100.0%)
Convulsive Activity	0	2 (100.0%)	4 (100.0%)	4 (100.0%)
Facial Muscle Symmetry	0	2 (100.0%)	4 (100.0%)	4 (100.0%)
Salivation	0	2 (100.0%)	4 (100.0%)	4 (100.0%)
Lacrimation – Left Eye	0	2 (100.0%)	4 (100.0%)	4 (100.0%)
Lacrimation – Right Eye	0	2 (100.0%)	4 (100.0%)	4 (100.0%)
Eyelid Closure – Left Eye	0	2 (100.0%)	4 (100.0%)	4 (100.0%)
Eyelid Closure – Right Eye	0	2 (100.0%)	4 (100.0%)	4 (100.0%)
Pupillary Diameter – Left Eye	0	2 (100.0%)	4 (100.0%)	4 (100.0%)
Pupillary Diameter – Right Eye	0	2 (100.0%)	4 (100.0%)	4 (100.0%)
Self-Mutilation	0	2 (100.0%)	4 (100.0%)	4 (100.0%)
Uncontrolled Retropulsion	0	2 (100.0%)	4 (100.0%)	4 (100.0%)
Repetitive Motions or Movements	0<br>1	2 (100.0%)<br>0 (0.0%)	3 (75.0%)<br>1 (25.0%)	2 (100.0%)<br>0 (0.0%)
Stargazing	0<br>1	2 (100.0%)<br>0 (0.0%)	3 (75.0%)<br>1 (25.0%)	3 (75.0%)<br>1 (25.0%)
Piloerection	0	2 (100.0%)	4 (100.0%)	4 (100.0%)
Urine Stain	0	2 (100.0%)	4 (100.0%)	4 (100.0%)
Fecal Stain	0	2 (100.0%)	4 (100.0%)	4 (100.0%)
Nasal Discharge	0	2 (100.0%)	4 (100.0%)	4 (100.0%)

AmplifyBio Study Number: A-579-01

Summary of Non-Invasive Functional Neurological Observations (6-Week and 12-Week Cohort Animals)

Week 6
Females

Parameter	Scores Observed	Count Percentage (%) Group 1 0 vg	Group 2 6×10^{13} vg	Group 3 1.2×10^{14} vg
Mentation/Behavior	0	2 (100.0%)	4 (100.0%)	4 (100.0%)
General Activity	-1<br>0<br>1	0 (0.0%)<br>2 (100.0%)<br>0 (0.0%)	0 (0.0%)<br>3 (75.0%)<br>1 (25.0%)	1 (25.0%)<br>3 (75.0%)<br>0 (0.0%)
Posture	0	2 (100.0%)	4 (100.0%)	4 (100.0%)
Voluntary Movement/Gait	0	2 (100.0%)	4 (100.0%)	4 (100.0%)
Vocalizations	0	2 (100.0%)	4 (100.0%)	4 (100.0%)
Tremor Activity	0	2 (100.0%)	4 (100.0%)	4 (100.0%)
Convulsive Activity	0	2 (100.0%)	4 (100.0%)	4 (100.0%)
Facial Muscle Symmetry	0	2 (100.0%)	4 (100.0%)	4 (100.0%)
Salivation	0	2 (100.0%)	4 (100.0%)	4 (100.0%)
Lacrimation – Left Eye	0	2 (100.0%)	4 (100.0%)	4 (100.0%)
Lacrimation – Right Eye	0	2 (100.0%)	4 (100.0%)	4 (100.0%)
Eyelid Closure – Left Eye	0	2 (100.0%)	4 (100.0%)	4 (100.0%)
Eyelid Closure – Right Eye	0	2 (100.0%)	4 (100.0%)	4 (100.0%)
Pupillary Diameter – Left Eye	0	2 (100.0%)	4 (100.0%)	4 (100.0%)
Pupillary Diameter – Right Eye	0	2 (100.0%)	4 (100.0%)	4 (100.0%)
Self-Mutilation	0	2 (100.0%)	4 (100.0%)	4 (100.0%)
Uncontrolled Retropulsion	0	2 (100.0%)	4 (100.0%)	4 (100.0%)
Repetitive Motions or Movements	0<br>1	2 (100.0%)<br>0 (0.0%)	3 (75.0%)<br>1(25.0%)	4 (100.0%)<br>0 (0.0%)
Stargazing	0	2 (100.0%)	4 (100.0%)	4 (100.0%)
Piloerection	0	2 (100.0%)	4 (100.0%)	4 (100.0%)
Urine Stain	0	2 (100.0%)	4 (100.0%)	4 (100.0%)
Fecal Stain	0	2 (100.0%)	4 (100.0%)	4 (100.0%)
Nasal Discharge	0	2 (100.0%)	4 (100.0%)	4 (100.0%)

AmplifyBio Study Number: A-579-01

Summary of Non-Invasive Functional Neurological Observations (12-Week Cohort Animals)

Week 7
Males

Parameter	Scores Observed	Count Percentage (%)		
		Group 1 0 vg	Group 2 6×10^{13} vg	Group 3 1.2×10^{14} vg
Mentation/Behavior	0	1 (100.0%)	2 (100.0%)	2 (100.0%)
General Activity	0	1 (100.0%)	2 (100.0%)	2 (100.0%)
Posture	0	1 (100.0%)	2 (100.0%)	2 (100.0%)
Voluntary Movement/Gait	0	1 (100.0%)	2 (100.0%)	2 (100.0%)
Vocalizations	0	1 (100.0%)	2 (100.0%)	2 (100.0%)
Tremor Activity	0	1 (100.0%)	2 (100.0%)	2 (100.0%)
Convulsive Activity	0	1 (100.0%)	2 (100.0%)	2 (100.0%)
Facial Muscle Symmetry	0	1 (100.0%)	2 (100.0%)	2 (100.0%)
Salivation	0	1 (100.0%)	2 (100.0%)	2 (100.0%)
Lacrimation – Left Eye	0	1 (100.0%)	2 (100.0%)	2 (100.0%)
Lacrimation – Right Eye	0	1 (100.0%)	2 (100.0%)	2 (100.0%)
Eyelid Closure – Left Eye	0	1 (100.0%)	2 (100.0%)	2 (100.0%)
Eyelid Closure – Right Eye	0	1 (100.0%)	2 (100.0%)	2 (100.0%)
Pupillary Diameter – Left Eye	0	1 (100.0%)	2 (100.0%)	2 (100.0%)
Pupillary Diameter – Right Eye	0	1 (100.0%)	2 (100.0%)	2 (100.0%)
Self-Mutilation	0	1 (100.0%)	2 (100.0%)	2 (100.0%)
Uncontrolled Retropulsion	0	1 (100.0%)	2 (100.0%)	2 (100.0%)
Repetitive Motions or Movements	0	1 (100.0%)	1 (50.0%)	2 (100.0%)
	1	0 (0.0%)	1 (50.0%)	0 (0.0%)
Stargazing	0	1 (100.0%)	1 (50.0%)	1 (50.0%)
	1	0 (0.0%)	1 (50.0%)	1 (50.0%)
Piloerection	0	1 (100.0%)	2 (100.0%)	2 (100.0%)
Urine Stain	0	1 (100.0%)	2 (100.0%)	2 (100.0%)
Fecal Stain	0	1 (100.0%)	2 (100.0%)	2 (100.0%)
Nasal Discharge	0	1 (100.0%)	2 (100.0%)	2 (100.0%)

AmplifyBio Study Number: A-579-01

Summary of Non-Invasive Functional Neurological Observations (12-Week Cohort Animals)

Week 7
Females

Parameter	Scores Observed	Count Percentage (%) Group 1 0 vg	Group 2 6×10^{13} vg	Group 3 1.2×10^{14} vg
Mentation/Behavior	0	1 (100.0%)	2 (100.0%)	2 (100.0%)
General Activity	-1<br>0	1 (100.0%)<br>0 (0.0%)	0 (0.0%)<br>2 (100.0%)	0 (0.0%)<br>2 (100.0%)
Posture	0	1 (100.0%)	2 (100.0%)	2 (100.0%)
Voluntary Movement/Gait	0	1 (100.0%)	2 (100.0%)	2 (100.0%)
Vocalizations	0	1 (100.0%)	2 (100.0%)	2 (100.0%)
Tremor Activity	0	1 (100.0%)	2 (100.0%)	2 (100.0%)
Convulsive Activity	0	1 (100.0%)	2 (100.0%)	2 (100.0%)
Facial Muscle Symmetry	0	1 (100.0%)	2 (100.0%)	2 (100.0%)
Salivation	0	1 (100.0%)	2 (100.0%)	2 (100.0%)
Lacrimation – Left Eye	0	1 (100.0%)	2 (100.0%)	2 (100.0%)
Lacrimation – Right Eye	0	1 (100.0%)	2 (100.0%)	2 (100.0%)
Eyelid Closure – Left Eye	0	1 (100.0%)	2 (100.0%)	2 (100.0%)
Eyelid Closure – Right Eye	0	1 (100.0%)	2 (100.0%)	2 (100.0%)
Pupillary Diameter – Left Eye	0	1 (100.0%)	2 (100.0%)	2 (100.0%)
Pupillary Diameter – Right Eye	0	1 (100.0%)	2 (100.0%)	2 (100.0%)
Self-Mutilation	0	1 (100.0%)	2 (100.0%)	2 (100.0%)
Uncontrolled Retropulsion	0	1 (100.0%)	2 (100.0%)	2 (100.0%)
Repetitive Motions or Movements	0<br>1	1 (100.0%)<br>0 (0.0%)	2 (100.0%)<br>0 (0.0%)	1 (50.0%)<br>1 (50.0%)
Stargazing	0	1 (100.0%)	2 (100.0%)	2 (100.0%)
Piloerection	0	1 (100.0%)	2 (100.0%)	2 (100.0%)
Urine Stain	0	1 (100.0%)	2 (100.0%)	2 (100.0%)
Fecal Stain	0	1 (100.0%)	2 (100.0%)	2 (100.0%)
Nasal Discharge	0	1 (100.0%)	2 (100.0%)	2 (100.0%)

AmplifyBio Study Number: A-579-01

Summary of Non-Invasive Functional Neurological Observations (12-Week Cohort Animals)

Week 8
Males

Parameter	Scores Observed	Count Percentage (%)		
		Group 1<br>0 vg	Group 2<br>6×10^{13} vg	Group 3<br>1.2×10^{14} vg
**Mentation/Behavior**	0	1 (100.0%)	2 (100.0%)	2 (100.0%)
**General Activity**	0	1 (100.0%)	2 (100.0%)	2 (100.0%)
**Posture**	0	1 (100.0%)	2 (100.0%)	2 (100.0%)
**Voluntary Movement/Gait**	0	1 (100.0%)	2 (100.0%)	2 (100.0%)
**Vocalizations**	0	1 (100.0%)	2 (100.0%)	2 (100.0%)
**Tremor Activity**	0	1 (100.0%)	2 (100.0%)	2 (100.0%)
**Convulsive Activity**	0	1 (100.0%)	2 (100.0%)	2 (100.0%)
**Facial Muscle Symmetry**	0	1 (100.0%)	2 (100.0%)	2 (100.0%)
**Salivation**	0	1 (100.0%)	2 (100.0%)	2 (100.0%)
**Lacrimation – Left Eye**	0	1 (100.0%)	2 (100.0%)	2 (100.0%)
**Lacrimation – Right Eye**	0	1 (100.0%)	2 (100.0%)	2 (100.0%)
**Eyelid Closure – Left Eye**	0	1 (100.0%)	2 (100.0%)	2 (100.0%)
**Eyelid Closure – Right Eye**	0	1 (100.0%)	2 (100.0%)	2 (100.0%)
**Pupillary Diameter – Left Eye**	0	1 (100.0%)	2 (100.0%)	2 (100.0%)
**Pupillary Diameter – Right Eye**	0	1 (100.0%)	2 (100.0%)	2 (100.0%)
**Self-Mutilation**	0	1 (100.0%)	2 (100.0%)	2 (100.0%)
**Uncontrolled Retropulsion**	0	1 (100.0%)	2 (100.0%)	2 (100.0%)
**Repetitive Motions or Movements**	0<br>1	1 (100.0%)<br>0 (0.0%)	1 (50.0%)<br>1 (50.0%)	2 (100.0%)<br>0 (0.0%)
**Stargazing**	0<br>1	1 (100.0%)<br>0 (0.0%)	1 (50.0%)<br>1 (50.0%)	1 (50.0%)<br>1 (50.0%)
**Piloerection**	0	1 (100.0%)	2 (100.0%)	2 (100.0%)
**Urine Stain**	0	1 (100.0%)	2 (100.0%)	2 (100.0%)
**Fecal Stain**	0	1 (100.0%)	2 (100.0%)	2 (100.0%)
**Nasal Discharge**	0	1 (100.0%)	2 (100.0%)	2 (100.0%)

AmplifyBio Study Number: A-579-01

Summary of Non-Invasive Functional Neurological Observations (12-Week Cohort Animals)

Week 8
Females

Parameter	Scores Observed	Count Percentage (%)		
		Group 1 0 vg	Group 2 6×10^{13} vg	Group 3 1.2×10^{14} vg
**Mentation/Behavior**	0	1 (100.0%)	2 (100.0%)	2 (100.0%)
**General Activity**	-1	1 (100.0%)	0 (0.0%)	0 (0.0%)
	0	0 (0.0%)	2 (100.0%)	2 (100.0%)
**Posture**	0	1 (100.0%)	2 (100.0%)	2 (100.0%)
**Voluntary Movement/Gait**	0	1 (100.0%)	2 (100.0%)	2 (100.0%)
**Vocalizations**	0	1 (100.0%)	2 (100.0%)	2 (100.0%)
**Tremor Activity**	0	1 (100.0%)	2 (100.0%)	2 (100.0%)
**Convulsive Activity**	0	1 (100.0%)	2 (100.0%)	2 (100.0%)
**Facial Muscle Symmetry**	0	1 (100.0%)	2 (100.0%)	2 (100.0%)
**Salivation**	0	1 (100.0%)	2 (100.0%)	2 (100.0%)
**Lacrimation – Left Eye**	0	1 (100.0%)	2 (100.0%)	2 (100.0%)
**Lacrimation – Right Eye**	0	1 (100.0%)	2 (100.0%)	2 (100.0%)
**Eyelid Closure – Left Eye**	0	1 (100.0%)	2 (100.0%)	2 (100.0%)
**Eyelid Closure – Right Eye**	0	1 (100.0%)	2 (100.0%)	2 (100.0%)
**Pupillary Diameter – Left Eye**	0	1 (100.0%)	2 (100.0%)	2 (100.0%)
**Pupillary Diameter – Right Eye**	0	1 (100.0%)	2 (100.0%)	2 (100.0%)
**Self-Mutilation**	0	1 (100.0%)	2 (100.0%)	2 (100.0%)
**Uncontrolled Retropulsion**	0	1 (100.0%)	2 (100.0%)	2 (100.0%)
**Repetitive Motions or Movements**	0	1 (100.0%)	2 (100.0%)	2 (100.0%)
**Stargazing**	0	1 (100.0%)	2 (100.0%)	2 (100.0%)
**Piloerection**	0	1 (100.0%)	2 (100.0%)	2 (100.0%)
**Urine Stain**	0	1 (100.0%)	2 (100.0%)	2 (100.0%)
**Fecal Stain**	0	1 (100.0%)	2 (100.0%)	2 (100.0%)
**Nasal Discharge**	0	1 (100.0%)	2 (100.0%)	2 (100.0%)

AmplifyBio Study Number: A-579-01

Summary of Non-Invasive Functional Neurological Observations (12-Week Cohort Animals)

Week 9
Males

Parameter	Scores Observed	Count Percentage (%)		
		Group 1<br>0 vg	Group 2<br>6×10^{13} vg	Group 3<br>1.2×10^{14} vg
Mentation/Behavior	0	1 (100.0%)	2 (100.0%)	2 (100.0%)
General Activity	-1<br>0	1 (100.0%)<br>0 (0.0%)	0 (0.0%)<br>2 (100.0%)	0 (0.0%)<br>2 (100.0%)
Posture	0	1 (100.0%)	2 (100.0%)	2 (100.0%)
Voluntary Movement/Gait	0	1 (100.0%)	2 (100.0%)	2 (100.0%)
Vocalizations	0	1 (100.0%)	2 (100.0%)	2 (100.0%)
Tremor Activity	0	1 (100.0%)	2 (100.0%)	2 (100.0%)
Convulsive Activity	0	1 (100.0%)	2 (100.0%)	2 (100.0%)
Facial Muscle Symmetry	0	1 (100.0%)	2 (100.0%)	2 (100.0%)
Salivation	0	1 (100.0%)	2 (100.0%)	2 (100.0%)
Lacrimation – Left Eye	0	1 (100.0%)	2 (100.0%)	2 (100.0%)
Lacrimation – Right Eye	0	1 (100.0%)	2 (100.0%)	2 (100.0%)
Eyelid Closure – Left Eye	0	1 (100.0%)	2 (100.0%)	2 (100.0%)
Eyelid Closure – Right Eye	0	1 (100.0%)	2 (100.0%)	2 (100.0%)
Pupillary Diameter – Left Eye	0	1 (100.0%)	2 (100.0%)	2 (100.0%)
Pupillary Diameter – Right Eye	0	1 (100.0%)	2 (100.0%)	2 (100.0%)
Self-Mutilation	0	1 (100.0%)	2 (100.0%)	2 (100.0%)
Uncontrolled Retropulsion	0	1 (100.0%)	2 (100.0%)	2 (100.0%)
Repetitive Motions or Movements	0<br>1	1 (100.0%)<br>0 (0.0%)	1 (50.0%)<br>1 (50.0%)	2 (100.0%)<br>0 (0.0%)
Stargazing	0	1 (100.0%)	2 (100.0%)	2 (100.0%)
Piloerection	0	1 (100.0%)	2 (100.0%)	2 (100.0%)
Urine Stain	0	1 (100.0%)	2 (100.0%)	2 (100.0%)
Fecal Stain	0	1 (100.0%)	2 (100.0%)	2 (100.0%)
Nasal Discharge	0	1 (100.0%)	2 (100.0%)	2 (100.0%)

AmplifyBio Study Number: A-579-01

Summary of Non-Invasive Functional Neurological Observations (12-Week Cohort Animals)

Week 9
Females

Parameter	Scores Observed	Count Percentage (%)		
		Group 1<br>0 vg	Group 2<br>6×10^{13} vg	Group 3<br>1.2×10^{14} vg
Mentation/Behavior	0	1 (100.0%)	2 (100.0%)	2 (100.0%)
General Activity	-1<br>0	1 (100.0%)<br>0 (0.0%)	0 (0.0%)<br>2 (100.0%)	0 (0.0%)<br>2 (100.0%)
Posture	0	1 (100.0%)	2 (100.0%)	2 (100.0%)
Voluntary Movement/Gait	0	1 (100.0%)	2 (100.0%)	2 (100.0%)
Vocalizations	0	1 (100.0%)	2 (100.0%)	2 (100.0%)
Tremor Activity	0	1 (100.0%)	2 (100.0%)	2 (100.0%)
Convulsive Activity	0	1 (100.0%)	2 (100.0%)	2 (100.0%)
Facial Muscle Symmetry	0	1 (100.0%)	2 (100.0%)	2 (100.0%)
Salivation	0	1 (100.0%)	2 (100.0%)	2 (100.0%)
Lacrimation – Left Eye	0	1 (100.0%)	2 (100.0%)	2 (100.0%)
Lacrimation – Right Eye	0	1 (100.0%)	2 (100.0%)	2 (100.0%)
Eyelid Closure – Left Eye	0	1 (100.0%)	2 (100.0%)	2 (100.0%)
Eyelid Closure – Right Eye	0	1 (100.0%)	2 (100.0%)	2 (100.0%)
Pupillary Diameter – Left Eye	0	1 (100.0%)	2 (100.0%)	2 (100.0%)
Pupillary Diameter – Right Eye	0	1 (100.0%)	2 (100.0%)	2 (100.0%)
Self-Mutilation	0	1 (100.0%)	2 (100.0%)	2 (100.0%)
Uncontrolled Retropulsion	0	1 (100.0%)	2 (100.0%)	2 (100.0%)
Repetitive Motions or Movements	0	1 (100.0%)	2 (100.0%)	2 (100.0%)
Stargazing	0	1 (100.0%)	2 (100.0%)	2 (100.0%)
Piloerection	0	1 (100.0%)	2 (100.0%)	2 (100.0%)
Urine Stain	0	1 (100.0%)	2 (100.0%)	2 (100.0%)
Fecal Stain	0	1 (100.0%)	2 (100.0%)	2 (100.0%)
Nasal Discharge	0	1 (100.0%)	2 (100.0%)	2 (100.0%)

AmplifyBio Study Number: A-579-01

Summary of Non-Invasive Functional Neurological Observations (12-Week Cohort Animals)

Week 10
Males

Parameter	Scores Observed	Count Percentage (%) Group 1 0 vg	Group 2 6×10^{13} vg	Group 3 1.2×10^{14} vg
Mentation/Behavior	0	1 (100.0%)	2 (100.0%)	2 (100.0%)
General Activity	0	1 (100.0%)	2 (100.0%)	2 (100.0%)
Posture	0	1 (100.0%)	2 (100.0%)	2 (100.0%)
Voluntary Movement/Gait	0	1 (100.0%)	2 (100.0%)	2 (100.0%)
Vocalizations	0	1 (100.0%)	2 (100.0%)	2 (100.0%)
Tremor Activity	0	1 (100.0%)	2 (100.0%)	2 (100.0%)
Convulsive Activity	0	1 (100.0%)	2 (100.0%)	2 (100.0%)
Facial Muscle Symmetry	0	1 (100.0%)	2 (100.0%)	2 (100.0%)
Salivation	0	1 (100.0%)	2 (100.0%)	2 (100.0%)
Lacrimation – Left Eye	0	1 (100.0%)	2 (100.0%)	2 (100.0%)
Lacrimation – Right Eye	0	1 (100.0%)	2 (100.0%)	2 (100.0%)
Eyelid Closure – Left Eye	0	1 (100.0%)	2 (100.0%)	2 (100.0%)
Eyelid Closure – Right Eye	0	1 (100.0%)	2 (100.0%)	2 (100.0%)
Pupillary Diameter – Left Eye	0	1 (100.0%)	2 (100.0%)	2 (100.0%)
Pupillary Diameter – Right Eye	0	1 (100.0%)	2 (100.0%)	2 (100.0%)
Self-Mutilation	0	1 (100.0%)	2 (100.0%)	2 (100.0%)
Uncontrolled Retropulsion	0	1 (100.0%)	2 (100.0%)	2 (100.0%)
Repetitive Motions or Movements	0	1 (100.0%)	1 (50.0%)	2 (100.0%)
	1	0 (0.0%)	1 (50.0%)	0 (0.0%)
Stargazing	0	1 (100.0%)	1 (50.0%)	1 (50.0%)
	1	0 (0.0%)	1 (50.0%)	1 (50.0%)
Piloerection	0	1 (100.0%)	2 (100.0%)	2 (100.0%)
Urine Stain	0	1 (100.0%)	2 (100.0%)	2 (100.0%)
Fecal Stain	0	1 (100.0%)	2 (100.0%)	2 (100.0%)
Nasal Discharge	0	1 (100.0%)	2 (100.0%)	2 (100.0%)

AmplifyBio Study Number: A-579-01

Summary of Non-Invasive Functional Neurological Observations (12-Week Cohort Animals)

Week 10
Females

Parameter	Scores Observed	Count Percentage (%)		
		Group 1<br>0 vg	Group 2<br>6×10^{13} vg	Group 3<br>1.2×10^{14} vg
Mentation/Behavior	0	1 (100.0%)	2 (100.0%)	2 (100.0%)
General Activity	-1	1 (100.0%)	0 (0.0%)	1 (50.0%)
	0	0 (0.0%)	2 (100.0%)	1 (50.0%)
Posture	0	1 (100.0%)	2 (100.0%)	2 (100.0%)
Voluntary Movement/Gait	0	1 (100.0%)	2 (100.0%)	2 (100.0%)
Vocalizations	0	1 (100.0%)	2 (100.0%)	2 (100.0%)
Tremor Activity	0	1 (100.0%)	2 (100.0%)	2 (100.0%)
Convulsive Activity	0	1 (100.0%)	2 (100.0%)	2 (100.0%)
Facial Muscle Symmetry	0	1 (100.0%)	2 (100.0%)	2 (100.0%)
Salivation	0	1 (100.0%)	2 (100.0%)	2 (100.0%)
Lacrimation – Left Eye	0	1 (100.0%)	2 (100.0%)	2 (100.0%)
Lacrimation – Right Eye	0	1 (100.0%)	2 (100.0%)	2 (100.0%)
Eyelid Closure – Left Eye	0	1 (100.0%)	2 (100.0%)	2 (100.0%)
Eyelid Closure – Right Eye	0	1 (100.0%)	2 (100.0%)	2 (100.0%)
Pupillary Diameter – Left Eye	0	1 (100.0%)	2 (100.0%)	2 (100.0%)
Pupillary Diameter – Right Eye	0	1 (100.0%)	2 (100.0%)	2 (100.0%)
Self-Mutilation	0	1 (100.0%)	2 (100.0%)	2 (100.0%)
Uncontrolled Retropulsion	0	1 (100.0%)	2 (100.0%)	2 (100.0%)
Repetitive Motions or Movements	0	1 (100.0%)	2 (100.0%)	2 (100.0%)
Stargazing	0	1 (100.0%)	2 (100.0%)	2 (100.0%)
Piloerection	0	1 (100.0%)	2 (100.0%)	2 (100.0%)
Urine Stain	0	1 (100.0%)	2 (100.0%)	2 (100.0%)
Fecal Stain	0	1 (100.0%)	2 (100.0%)	2 (100.0%)
Nasal Discharge	0	1 (100.0%)	2 (100.0%)	2 (100.0%)

AmplifyBio Study Number: A-579-01

Summary of Non-Invasive Functional Neurological Observations
(12-Week Cohort Animals)

Week 11
Males

Parameter	Scores Observed	Count Percentage (%)		
		Group 1<br>0 vg	Group 2<br>6 × 10^{13} vg	Group 3<br>1.2 × 10^{14} vg
Mentation/Behavior	0	1 (100.0%)	2 (100.0%)	2 (100.0%)
General Activity	-1<br>0	1 (100.0%)<br>0 (0.0%)	0 (0.0%)<br>2 (100.0%)	0 (0.0%)<br>2 (100.0%)
Posture	0	1 (100.0%)	2 (100.0%)	2 (100.0%)
Voluntary Movement/Gait	0	1 (100.0%)	2 (100.0%)	2 (100.0%)
Vocalizations	0	1 (100.0%)	2 (100.0%)	2 (100.0%)
Tremor Activity	0	1 (100.0%)	2 (100.0%)	2 (100.0%)
Convulsive Activity	0	1 (100.0%)	2 (100.0%)	2 (100.0%)
Facial Muscle Symmetry	0	1 (100.0%)	2 (100.0%)	2 (100.0%)
Salivation	0	1 (100.0%)	2 (100.0%)	2 (100.0%)
Lacrimation – Left Eye	0	1 (100.0%)	2 (100.0%)	2 (100.0%)
Lacrimation – Right Eye	0	1 (100.0%)	2 (100.0%)	2 (100.0%)
Eyelid Closure – Left Eye	0	1 (100.0%)	2 (100.0%)	2 (100.0%)
Eyelid Closure – Right Eye	0	1 (100.0%)	2 (100.0%)	2 (100.0%)
Pupillary Diameter – Left Eye	0	1 (100.0%)	2 (100.0%)	2 (100.0%)
Pupillary Diameter – Right Eye	0	1 (100.0%)	2 (100.0%)	2 (100.0%)
Self-Mutilation	0	1 (100.0%)	2 (100.0%)	2 (100.0%)
Uncontrolled Retropulsion	0	1 (100.0%)	2 (100.0%)	2 (100.0%)
Repetitive Motions or Movements	0<br>1	1 (100.0%)<br>0 (0.0%)	1 (50.0%)<br>1 (50.0%)	2 (100.0%)<br>0 (0.0%)
Stargazing	0	1 (100.0%)	2 (100.0%)	2 (100.0%)
Piloerection	0	1 (100.0%)	2 (100.0%)	2 (100.0%)
Urine Stain	0	1 (100.0%)	2 (100.0%)	2 (100.0%)
Fecal Stain	0	1 (100.0%)	2 (100.0%)	2 (100.0%)
Nasal Discharge	0	1 (100.0%)	2 (100.0%)	2 (100.0%)

AmplifyBio Study Number: A-579-01

Summary of Non-Invasive Functional Neurological Observations (12-Week Cohort Animals)

Week 11
Females

Parameter	Scores Observed	Count Percentage (%)		
		Group 1<br>0 vg	Group 2<br>6 × 10^{13} vg	Group 3<br>1.2 × 10^{14} vg
Mentation/Behavior	0	1 (100.0%)	2 (100.0%)	2 (100.0%)
General Activity	-1<br>0	1 (100.0%)<br>0 (0.0%)	1 (50.0%)<br>1 (50.0%)	0 (0.0%)<br>2 (100.0%)
Posture	0	1 (100.0%)	2 (100.0%)	2 (100.0%)
Voluntary Movement/Gait	0	1 (100.0%)	2 (100.0%)	2 (100.0%)
Vocalizations	0	1 (100.0%)	2 (100.0%)	2 (100.0%)
Tremor Activity	0	1 (100.0%)	2 (100.0%)	2 (100.0%)
Convulsive Activity	0	1 (100.0%)	2 (100.0%)	2 (100.0%)
Facial Muscle Symmetry	0	1 (100.0%)	2 (100.0%)	2 (100.0%)
Salivation	0	1 (100.0%)	2 (100.0%)	2 (100.0%)
Lacrimation – Left Eye	0	1 (100.0%)	2 (100.0%)	2 (100.0%)
Lacrimation – Right Eye	0	1 (100.0%)	2 (100.0%)	2 (100.0%)
Eyelid Closure – Left Eye	0	1 (100.0%)	2 (100.0%)	2 (100.0%)
Eyelid Closure – Right Eye	0	1 (100.0%)	2 (100.0%)	2 (100.0%)
Pupillary Diameter – Left Eye	0	1 (100.0%)	2 (100.0%)	2 (100.0%)
Pupillary Diameter – Right Eye	0	1 (100.0%)	2 (100.0%)	2 (100.0%)
Self-Mutilation	0	1 (100.0%)	2 (100.0%)	2 (100.0%)
Uncontrolled Retropulsion	0	1 (100.0%)	2 (100.0%)	2 (100.0%)
Repetitive Motions or Movements	0	1 (100.0%)	2 (100.0%)	2 (100.0%)
Stargazing	0	1 (100.0%)	2 (100.0%)	2 (100.0%)
Piloerection	0	1 (100.0%)	2 (100.0%)	2 (100.0%)
Urine Stain	0	1 (100.0%)	2 (100.0%)	2 (100.0%)
Fecal Stain	0	1 (100.0%)	2 (100.0%)	2 (100.0%)
Nasal Discharge	0	1 (100.0%)	2 (100.0%)	2 (100.0%)

AmplifyBio Study Number: A-579-01

Summary of Non-Invasive Functional Neurological Observations (12-Week Cohort Animals)

Week 12
Males

Parameter	Scores Observed	Count Percentage (%) Group 1 0 vg	Group 2 6×10^{13} vg	Group 3 1.2×10^{14} vg
Mentation/Behavior	0	1 (100.0%)	2 (100.0%)	2 (100.0%)
General Activity	0	1 (100.0%)	2 (100.0%)	2 (100.0%)
Posture	0	1 (100.0%)	2 (100.0%)	2 (100.0%)
Voluntary Movement/Gait	0	1 (100.0%)	2 (100.0%)	2 (100.0%)
Vocalizations	0	1 (100.0%)	2 (100.0%)	2 (100.0%)
Tremor Activity	0	1 (100.0%)	2 (100.0%)	2 (100.0%)
Convulsive Activity	0	1 (100.0%)	2 (100.0%)	2 (100.0%)
Facial Muscle Symmetry	0	1 (100.0%)	2 (100.0%)	2 (100.0%)
Salivation	0	1 (100.0%)	2 (100.0%)	2 (100.0%)
Lacrimation – Left Eye	0	1 (100.0%)	2 (100.0%)	2 (100.0%)
Lacrimation – Right Eye	0	1 (100.0%)	2 (100.0%)	2 (100.0%)
Eyelid Closure – Left Eye	0	1 (100.0%)	2 (100.0%)	2 (100.0%)
Eyelid Closure – Right Eye	0	1 (100.0%)	2 (100.0%)	2 (100.0%)
Pupillary Diameter – Left Eye	0	1 (100.0%)	2 (100.0%)	2 (100.0%)
Pupillary Diameter – Right Eye	0	1 (100.0%)	2 (100.0%)	2 (100.0%)
Self-Mutilation	0	1 (100.0%)	2 (100.0%)	2 (100.0%)
Uncontrolled Retropulsion	0	1 (100.0%)	2 (100.0%)	2 (100.0%)
Repetitive Motions or Movements	0	1 (100.0%)	1 (50.0%)	2 (100.0%)
	1	0 (0.0%)	1 (50.0%)	0 (0.0%)
Stargazing	0	1 (100.0%)	1 (50.0%)	1 (50.0%)
	1	0 (0.0%)	1 (50.0%)	1 (50.0%)
Piloerection	0	1 (100.0%)	2 (100.0%)	2 (100.0%)
Urine Stain	0	1 (100.0%)	2 (100.0%)	2 (100.0%)
Fecal Stain	0	1 (100.0%)	2 (100.0%)	2 (100.0%)
Nasal Discharge	0	1 (100.0%)	2 (100.0%)	2 (100.0%)

AmplifyBio Study Number: A-579-01

Summary of Non-Invasive Functional Neurological Observations (12-Week Cohort Animals)

Week 12
Females

Parameter	Scores Observed	Count Percentage (%)		
		Group 1<br>0 vg	Group 2<br>6×10^{13} vg	Group 3<br>1.2×10^{14} vg
Mentation/Behavior	0	1 (100.0%)	2 (100.0%)	2 (100.0%)
General Activity	-1<br>0	1 (100.0%)<br>0 (0.0%)	0 (0.0%)<br>2 (100.0%)	0 (0.0%)<br>2 (100.0%)
Posture	0	1 (100.0%)	2 (100.0%)	2 (100.0%)
Voluntary Movement/Gait	0	1 (100.0%)	2 (100.0%)	2 (100.0%)
Vocalizations	0	1 (100.0%)	2 (100.0%)	2 (100.0%)
Tremor Activity	0	1 (100.0%)	2 (100.0%)	2 (100.0%)
Convulsive Activity	0	1 (100.0%)	2 (100.0%)	2 (100.0%)
Facial Muscle Symmetry	0	1 (100.0%)	2 (100.0%)	2 (100.0%)
Salivation	0	1 (100.0%)	2 (100.0%)	2 (100.0%)
Lacrimation – Left Eye	0	1 (100.0%)	2 (100.0%)	2 (100.0%)
Lacrimation – Right Eye	0	1 (100.0%)	2 (100.0%)	2 (100.0%)
Eyelid Closure – Left Eye	0	1 (100.0%)	2 (100.0%)	2 (100.0%)
Eyelid Closure – Right Eye	0	1 (100.0%)	2 (100.0%)	2 (100.0%)
Pupillary Diameter – Left Eye	0	1 (100.0%)	2 (100.0%)	2 (100.0%)
Pupillary Diameter – Right Eye	0	1 (100.0%)	2 (100.0%)	2 (100.0%)
Self-Mutilation	0	1 (100.0%)	2 (100.0%)	2 (100.0%)
Uncontrolled Retropulsion	0	1 (100.0%)	2 (100.0%)	2 (100.0%)
Repetitive Motions or Movements	0	1 (100.0%)	2 (100.0%)	2 (100.0%)
Stargazing	0	1 (100.0%)	2 (100.0%)	2 (100.0%)
Piloerection	0	1 (100.0%)	2 (100.0%)	2 (100.0%)
Urine Stain	0	1 (100.0%)	2 (100.0%)	2 (100.0%)
Fecal Stain	0	1 (100.0%)	2 (100.0%)	2 (100.0%)
Nasal Discharge	0	1 (100.0%)	2 (100.0%)	2 (100.0%)

Attachment 2. Individual Tables

AmplifyBio Study Number: A-579-01

Individual Non-Invasive Functional Neurological Observations – Scoring Data

Pre-Test

Group	Animal ID	Sex	Mentation/ Behavior	General Activity	Posture	Voluntary Movement/ Gait	Vocalizations
1 - Vehicle	101	Male	0	0	0	0	0
1 - Vehicle	102	Male	0	-1	0	0	0
2 - Low	201	Male	0	0	0	0	0
2 - Low	202	Male	0	0	0	0	0
2 - Low	203	Male	0	0	0	0	0
2 - Low	204	Male	0	0	0	0	0
3 - High	301	Male	0	0	0	0	0
3 - High	302	Male	1	0	0	0	0
3 - High	303	Male	0	0	0	0	1
3 - High	304	Male	0	0	0	0	0
1 - Vehicle	151	Female	0	0	0	0	0
1 - Vehicle	152	Female	0	0	0	0	0
2 - Low	251	Female	0	-1	0	0	0
2 - Low	252	Female	0	0	0	0	0
2 - Low	253	Female	0	0	0	0	0
2 - Low	254	Female	0	0	0	0	0
3 - High	351	Female	0	1	0	0	0
3 - High	352	Female	0	0	0	0	0
3 - High	353	Female	0	0	0	0	0
3 - High	354	Female	1	0	0	0	0

AmplifyBio Study Number: A-579-01

Individual Non-Invasive Functional Neurological Observations – Scoring Data (Continued)

Pre-Test

Group	Animal ID	Sex	Tremor Activity	Convulsive Activity	Facial Muscle Symmetry	Salivation
1 - Vehicle	101	Male	0	0	0	0
1 - Vehicle	102	Male	0	0	0	0
2 - Low	201	Male	0	0	0	0
2 - Low	202	Male	0	0	0	0
2 - Low	203	Male	0	0	0	0
2 - Low	204	Male	0	0	0	0
3 - High	301	Male	0	0	0	0
3 - High	302	Male	0	0	0	0
3 - High	303	Male	0	0	0	0
3 - High	304	Male	0	0	0	0
1 - Vehicle	151	Female	0	0	0	0
1 - Vehicle	152	Female	0	0	0	0
2 - Low	251	Female	0	0	0	0
2 - Low	252	Female	0	0	0	0
2 - Low	253	Female	0	0	0	0
2 - Low	254	Female	0	0	0	0
3 - High	351	Female	0	0	0	0
3 - High	352	Female	0	0	0	0
3 - High	353	Female	0	0	0	0
3 - High	354	Female	0	0	0	0

AmplifyBio Study Number: A-579-01

Individual Non-Invasive Functional Neurological Observations – Scoring Data (Continued)

Pre-Test

Group	Animal ID	Sex	Lacrimation		Eyelid Closure		Pupillary Diameter	
			Left Eye	Right Eye	Left Eye	Right Eye	Left Eye	Right Eye
1 - Vehicle	101	Male	0	0	0	0	0	0
1 - Vehicle	102	Male	0	0	0	0	0	0
2 - Low	201	Male	0	0	0	0	0	0
2 - Low	202	Male	0	0	0	0	0	0
2 - Low	203	Male	0	0	0	0	0	0
2 - Low	204	Male	0	0	0	0	0	0
3 - High	301	Male	0	0	0	0	0	0
3 - High	302	Male	0	0	0	0	0	0
3 - High	303	Male	0	0	0	0	0	0
3 - High	304	Male	0	0	0	0	0	0
1 - Vehicle	151	Female	0	0	0	0	0	0
1 - Vehicle	152	Female	0	0	0	0	0	0
2 - Low	251	Female	0	0	0	0	0	0
2 - Low	252	Female	0	0	0	0	0	0
2 - Low	253	Female	0	0	0	0	0	0
2 - Low	254	Female	0	0	0	0	0	0
3 - High	351	Female	0	0	0	0	0	0
3 - High	352	Female	0	0	0	0	0	0
3 - High	353	Female	0	0	0	0	0	0
3 - High	354	Female	0	0	0	0	0	0

.

AmplifyBio Study Number: A-579-01

Individual Non-Invasive Functional Neurological Observations – Stereotypical Behaviors (present or absent)

Pre-Test

Group	Animal ID	Sex	Self-Mutilation	Uncontrolled Retropulsion	Repetitive Motions or Movements	Stargazing
1 - Vehicle	101	Male	0	0	0	0
1 - Vehicle	102	Male	0	0	0	0
2 - Low	201	Male	0	0	0	0
2 - Low	202	Male	0	0	0	0
2 - Low	203	Male	0	0	0	0
2 - Low	204	Male	0	0	1	1
3 - High	301	Male	0	0	0	0
3 - High	302	Male	0	0	0	0
3 - High	303	Male	0	0	0	0
3 - High	304	Male	0	0	0	0
1 - Vehicle	151	Female	0	0	0	0
1 - Vehicle	152	Female	0	0	0	0
2 - Low	251	Female	0	0	0	0
2 - Low	252	Female	0	0	0	0
2 - Low	253	Female	0	0	0	0
2 - Low	254	Female	0	0	0	0
3 - High	351	Female	0	0	1	0
3 - High	352	Female	0	0	0	0
3 - High	353	Female	0	0	0	0
3 - High	354	Female	0	0	0	0

AmplifyBio Study Number: A-579-01

Individual Non-Invasive Functional Neurological Observations – Other General Observations (present or absent)

Pre-Test

Group	Animal ID	Sex	Piloerection	Urine Stain	Fecal Stain	Nasal Discharge
1 - Vehicle	101	Male	0	0	0	0
1 - Vehicle	102	Male	0	0	0	0
2 - Low	201	Male	0	0	0	0
2 - Low	202	Male	0	0	0	0
2 - Low	203	Male	0	0	0	0
2 - Low	204	Male	0	0	0	0
3 - High	301	Male	0	0	0	0
3 - High	302	Male	0	0	0	0
3 - High	303	Male	0	0	0	0
3 - High	304	Male	0	0	0	0
1 - Vehicle	151	Female	0	0	0	0
1 - Vehicle	152	Female	0	0	0	0
2 - Low	251	Female	0	0	0	0
2 - Low	252	Female	0	0	0	0
2 - Low	253	Female	0	0	0	0
2 - Low	254	Female	0	0	0	0
3 - High	351	Female	0	0	0	0
3 - High	352	Female	0	0	0	0
3 - High	353	Female	0	0	0	0
3 - High	354	Female	0	0	0	0

AmplifyBio Study Number: A-579-01

Individual Non-Invasive Functional Neurological Observations – Scoring Data

Week 1

Group	Animal ID	Sex	Mentation/ Behavior	General Activity	Posture	Voluntary Movement/ Gait	Vocalizations
1 - Vehicle	101	Male	0	0	0	0	0
1 - Vehicle	102	Male	0	0	0	0	0
2 - Low	201	Male	0	0	0	0	0
2 - Low	202	Male	0	0	0	0	0
2 - Low	203	Male	0	0	0	0	0
2 - Low	204	Male	0	0	0	0	0
3 - High	301	Male	0	0	0	0	0
3 - High	302	Male	0	0	0	0	0
3 - High	303	Male	0	0	0	0	0
3 - High	304	Male	0	0	0	0	0
1 - Vehicle	151	Female	0	0	0	0	0
1 - Vehicle	152	Female	0	-1	0	0	0
2 - Low	251	Female	0	0	0	0	0
2 - Low	252	Female	0	0	0	0	0
2 - Low	253	Female	0	0	0	0	0
2 - Low	254	Female	0	0	0	0	0
3 - High	351	Female	0	0	0	0	0
3 - High	352	Female	0	0	0	0	0
3 - High	353	Female	0	0	0	0	0
3 - High	354	Female	0	0	0	0	0

AmplifyBio Study Number: A-579-01

Individual Non-Invasive Functional Neurological Observations – Scoring Data (Continued)

Week 1

Group	Animal ID	Sex	Tremor Activity	Convulsive Activity	Facial Muscle Symmetry	Salivation
1 - Vehicle	101	Male	0	0	0	0
1 - Vehicle	102	Male	0	0	0	0
2 - Low	201	Male	0	0	0	0
2 - Low	202	Male	0	0	0	0
2 - Low	203	Male	0	0	0	0
2 - Low	204	Male	0	0	0	0
3 - High	301	Male	0	0	0	0
3 - High	302	Male	0	0	0	0
3 - High	303	Male	0	0	0	0
3 - High	304	Male	0	0	0	0
1 - Vehicle	151	Female	0	0	0	0
1 - Vehicle	152	Female	0	0	0	0
2 - Low	251	Female	0	0	0	0
2 - Low	252	Female	0	0	0	0
2 - Low	253	Female	0	0	0	0
2 - Low	254	Female	0	0	0	0
3 - High	351	Female	0	0	0	0
3 - High	352	Female	0	0	0	0
3 - High	353	Female	0	0	0	0
3 - High	354	Female	0	0	0	0

AmplifyBio Study Number: A-579-01

Individual Non-Invasive Functional Neurological Observations – Scoring Data (Continued)

Week 1

Group	Animal ID	Sex	Lacrimation		Eyelid Closure		Pupillary Diameter	
			Left Eye	Right Eye	Left Eye	Right Eye	Left Eye	Right Eye
1 - Vehicle	101	Male	0	0	0	0	0	0
1 - Vehicle	102	Male	0	0	0	0	0	0
2 - Low	201	Male	0	0	0	0	0	0
2 - Low	202	Male	0	0	0	0	0	0
2 - Low	203	Male	0	0	0	0	0	0
2 - Low	204	Male	0	0	0	0	0	0
3 - High	301	Male	0	0	0	0	0	0
3 - High	302	Male	0	0	0	0	0	0
3 - High	303	Male	0	0	0	0	0	0
3 - High	304	Male	0	0	0	0	0	0
1 - Vehicle	151	Female	0	0	0	0	0	0
1 - Vehicle	152	Female	0	0	0	0	0	0
2 - Low	251	Female	0	0	0	0	0	0
2 - Low	252	Female	0	0	0	0	0	0
2 - Low	253	Female	0	0	0	0	0	0
2 - Low	254	Female	0	0	0	0	0	0
3 - High	351	Female	0	0	0	0	0	0
3 - High	352	Female	0	0	0	0	0	0
3 - High	353	Female	0	0	0	0	0	0
3 - High	354	Female	0	0	0	0	0	0

AmplifyBio Study Number: A-579-01

Individual Non-Invasive Functional Neurological Observations – Stereotypical Behaviors (present or absent)

Week 1

Group[a]	Animal ID	Sex	Self-Mutilation	Uncontrolled Retropulsion	Repetitive Motions or Movements	Stargazing
1 - Vehicle	101	Male	0	0	0	0
1 - Vehicle	102	Male	0	0	0	0
2 - Low	201	Male	0	0	0	0
2 - Low	202	Male	0	0	0	0
2 - Low	203	Male	0	0	0	0
2 - Low	204	Male	0	0	1	0
3 - High	301	Male	0	0	0	0
3 - High	302	Male	0	0	0	0
3 - High	303	Male	0	0	0	0
3 - High	304	Male	0	0	1	1
1 - Vehicle	151	Female	0	0	0	0
1 - Vehicle	152	Female	0	0	0	0
2 - Low	251	Female	0	0	0	0
2 - Low	252	Female	0	0	0	0
2 - Low	253	Female	0	0	0	0
2 - Low	254	Female	0	0	0	0
3 - High	351	Female	0	0	0	0
3 - High	352	Female	0	0	0	0
3 - High	353	Female	0	0	0	0
3 - High	354	Female	0	0	0	0

AmplifyBio Study Number: A-579-01

Individual Non-Invasive Functional Neurological Observations – Other General Observations (present or absent)

Week 1

Group[a]	Animal ID	Sex	Piloerection	Urine Stain	Fecal Stain	Nasal Discharge
1 - Vehicle	101	Male	0	0	0	0
1 - Vehicle	102	Male	0	0	0	0
2 - Low	201	Male	0	0	0	0
2 - Low	202	Male	0	0	0	0
2 - Low	203	Male	0	0	0	0
2 - Low	204	Male	0	0	0	0
3 - High	301	Male	0	0	0	0
3 - High	302	Male	0	0	0	0
3 - High	303	Male	0	0	0	0
3 - High	304	Male	0	0	0	0
1 - Vehicle	151	Female	0	0	0	0
1 - Vehicle	152	Female	0	0	0	0
2 - Low	251	Female	0	0	0	0
2 - Low	252	Female	0	0	0	0
2 - Low	253	Female	0	0	0	0
2 - Low	254	Female	0	0	0	0
3 - High	351	Female	0	0	0	0
3 - High	352	Female	0	0	0	0
3 - High	353	Female	0	0	0	0
3 - High	354	Female	0	0	0	0

AmplifyBio Study Number: A-579-01

Individual Non-Invasive Functional Neurological Observations – Scoring Data

Week 2

Group	Animal ID	Sex	Mentation/ Behavior	General Activity	Posture	Voluntary Movement/ Gait	Vocalizations
1 - Vehicle	101	Male	0	0	0	0	0
1 - Vehicle	102	Male	0	0	0	0	0
2 - Low	201	Male	0	0	0	0	0
2 - Low	202	Male	0	0	0	0	0
2 - Low	203	Male	0	0	0	0	0
2 - Low	204	Male	0	0	0	0	0
3 - High	301	Male	0	0	0	0	0
3 - High	302	Male	0	0	0	0	0
3 - High	303	Male	0	0	0	0	0
3 - High	304	Male	0	0	0	0	0
1 - Vehicle	151	Female	0	0	0	0	0
1 - Vehicle	152	Female	0	0	0	0	0
2 - Low	251	Female	0	0	0	0	0
2 - Low	252	Female	0	0	0	0	0
2 - Low	253	Female	0	0	0	0	0
2 - Low	254	Female	0	0	0	0	0
3 - High	351	Female	0	0	0	0	0
3 - High	352	Female	0	0	0	0	0
3 - High	353	Female	0	-1	0	0	0
3 - High	354	Female	0	0	0	0	0

AmplifyBio Study Number: A-579-01

Individual Non-Invasive Functional Neurological Observations – Scoring Data (Continued)

Week 2

Group	Animal ID	Sex	Tremor Activity	Convulsive Activity	Facial Muscle Symmetry	Salivation
1 - Vehicle	101	Male	0	0	0	0
1 - Vehicle	102	Male	0	0	0	0
2 - Low	201	Male	0	0	0	0
2 - Low	202	Male	0	0	0	0
2 - Low	203	Male	0	0	0	0
2 - Low	204	Male	0	0	0	0
3 - High	301	Male	0	0	0	0
3 - High	302	Male	0	0	0	0
3 - High	303	Male	0	0	0	0
3 - High	304	Male	0	0	0	0
1 - Vehicle	151	Female	0	0	0	0
1 - Vehicle	152	Female	0	0	0	0
2 - Low	251	Female	0	0	0	0
2 - Low	252	Female	0	0	0	0
2 - Low	253	Female	0	0	0	0
2 - Low	254	Female	0	0	0	0
3 - High	351	Female	0	0	0	0
3 - High	352	Female	0	0	0	0
3 - High	353	Female	0	0	0	0
3 - High	354	Female	0	0	0	0

AmplifyBio Study Number: A-579-01

Individual Non-Invasive Functional Neurological Observations – Scoring Data (Continued)

Week 2

Group	Animal ID	Sex	Lacrimation		Eyelid Closure		Pupillary Diameter	
			Left Eye	Right Eye	Left Eye	Right Eye	Left Eye	Right Eye
1 - Vehicle	101	Male	0	0	0	0	0	0
1 - Vehicle	102	Male	0	0	0	0	0	0
2 - Low	201	Male	0	0	0	0	0	0
2 - Low	202	Male	0	0	0	0	0	0
2 - Low	203	Male	0	0	0	0	0	0
2 - Low	204	Male	0	0	0	0	0	0
3 - High	301	Male	0	0	0	0	0	0
3 - High	302	Male	0	0	0	0	0	0
3 - High	303	Male	0	0	0	0	0	0
3 - High	304	Male	0	0	0	0	0	0
1 - Vehicle	151	Female	0	0	0	0	0	0
1 - Vehicle	152	Female	0	0	0	0	0	0
2 - Low	251	Female	0	0	0	0	0	0
2 - Low	252	Female	0	0	0	0	0	0
2 - Low	253	Female	0	0	0	0	0	0
2 - Low	254	Female	0	0	0	0	0	0
3 - High	351	Female	0	0	0	0	0	0
3 - High	352	Female	0	0	0	0	0	0
3 - High	353	Female	0	0	0	0	0	0
3 - High	354	Female	0	0	0	0	0	0

AmplifyBio Study Number: A-579-01

Individual Non-Invasive Functional Neurological Observations – Stereotypical Behaviors (present or absent)

Week 2

Group	Animal ID	Sex	Self-Mutilation	Uncontrolled Retropulsion	Repetitive Motions or Movements	Stargazing
1 - Vehicle	101	Male	0	0	0	0
1 - Vehicle	102	Male	0	0	1	0
2 - Low	201	Male	0	0	0	0
2 - Low	202	Male	0	0	0	0
2 - Low	203	Male	0	0	0	0
2 - Low	204	Male	0	0	1	1
3 - High	301	Male	0	0	0	0
3 - High	302	Male	0	0	0	0
3 - High	303	Male	0	0	0	0
3 - High	304	Male	0	0	0	1
1 - Vehicle	151	Female	0	0	0	0
1 - Vehicle	152	Female	0	0	0	0
2 - Low	251	Female	0	0	0	0
2 - Low	252	Female	0	0	0	0
2 - Low	253	Female	0	0	0	0
2 - Low	254	Female	0	0	0	0
3 - High	351	Female	0	0	0	0
3 - High	352	Female	0	0	0	0
3 - High	353	Female	0	0	0	0
3 - High	354	Female	0	0	0	0

AmplifyBio Study Number: A-579-01

Individual Non-Invasive Functional Neurological Observations – Other General Observations (present or absent)

Week 2

Group	Animal ID	Sex	Piloerection	Urine Stain	Fecal Stain	Nasal Discharge
1 - Vehicle	101	Male	0	0	0	0
1 - Vehicle	102	Male	0	0	0	0
2 - Low	201	Male	0	0	0	0
2 - Low	202	Male	0	0	0	0
2 - Low	203	Male	0	0	0	0
2 - Low	204	Male	0	0	0	0
3 - High	301	Male	0	0	0	0
3 - High	302	Male	0	0	0	0
3 - High	303	Male	0	0	0	0
3 - High	304	Male	0	0	0	0
1 - Vehicle	151	Female	0	0	0	0
1 - Vehicle	152	Female	0	0	0	0
2 - Low	251	Female	0	0	0	0
2 - Low	252	Female	0	0	0	0
2 - Low	253	Female	1	0	0	0
2 - Low	254	Female	0	0	0	0
3 - High	351	Female	0	0	0	0
3 - High	352	Female	0	0	0	0
3 - High	353	Female	0	0	0	0
3 - High	354	Female	0	0	0	0

AmplifyBio Study Number: A-579-01

Individual Non-Invasive Functional Neurological Observations – Scoring Data

Week 3

Group	Animal ID	Sex	Mentation/ Behavior	General Activity	Posture	Voluntary Movement/ Gait	Vocalizations
1 - Vehicle	101	Male	0	0	0	0	0
1 - Vehicle	102	Male	0	0	0	0	0
2 - Low	201	Male	0	0	0	0	0
2 - Low	202	Male	0	0	0	0	0
2 - Low	203	Male	0	0	0	0	0
2 - Low	204	Male	0	0	0	0	0
3 - High	301	Male	0	0	0	0	0
3 - High	302	Male	0	0	0	0	0
3 - High	303	Male	0	0	0	0	0
3 - High	304	Male	0	0	0	0	0
1 - Vehicle	151	Female	0	0	0	0	0
1 - Vehicle	152	Female	0	0	0	0	0
2 - Low	251	Female	0	0	0	0	0
2 - Low	252	Female	0	0	0	0	0
2 - Low	253	Female	0	0	0	0	0
2 - Low	254	Female	0	0	0	0	0
3 - High	351	Female	0	0	0	0	0
3 - High	352	Female	0	0	0	0	0
3 - High	353	Female	0	-1	0	0	0
3 - High	354	Female	0	0	0	0	0

AmplifyBio Study Number: A-579-01

Individual Non-Invasive Functional Neurological Observations – Scoring Data (Continued)

Week 3

Group	Animal ID	Sex	Tremor Activity	Convulsive Activity	Facial Muscle Symmetry	Salivation
1 - Vehicle	101	Male	0	0	0	0
1 - Vehicle	102	Male	0	0	0	0
2 - Low	201	Male	0	0	0	0
2 - Low	202	Male	0	0	0	0
2 - Low	203	Male	0	0	0	0
2 - Low	204	Male	0	0	0	0
3 - High	301	Male	0	0	0	0
3 - High	302	Male	0	0	0	0
3 - High	303	Male	0	0	0	0
3 - High	304	Male	0	0	0	0
1 - Vehicle	151	Female	0	0	0	0
1 - Vehicle	152	Female	0	0	0	0
2 - Low	251	Female	0	0	0	0
2 - Low	252	Female	0	0	0	0
2 - Low	253	Female	0	0	0	0
2 - Low	254	Female	0	0	0	0
3 - High	351	Female	0	0	0	0
3 - High	352	Female	0	0	0	0
3 - High	353	Female	0	0	0	0
3 - High	354	Female	0	0	0	0

AmplifyBio Study Number: A-579-01

Individual Non-Invasive Functional Neurological Observations – Scoring Data (Continued)

Week 3

Group	Animal ID	Sex	Lacrimation		Eyelid Closure		Pupillary Diameter	
			Left Eye	Right Eye	Left Eye	Right Eye	Left Eye	Right Eye
1 - Vehicle	101	Male	0	0	0	0	0	0
1 - Vehicle	102	Male	0	0	0	0	0	0
2 - Low	201	Male	0	0	0	0	0	0
2 - Low	202	Male	0	0	0	0	0	0
2 - Low	203	Male	0	0	0	0	0	0
2 - Low	204	Male	0	0	0	0	0	0
3 - High	301	Male	0	0	0	0	0	0
3 - High	302	Male	0	0	0	0	0	0
3 - High	303	Male	0	0	0	0	0	0
3 - High	304	Male	0	0	0	0	0	0
1 - Vehicle	151	Female	0	0	0	0	0	0
1 - Vehicle	152	Female	0	0	0	0	0	0
2 - Low	251	Female	0	0	0	0	0	0
2 - Low	252	Female	0	0	0	0	0	0
2 - Low	253	Female	0	0	0	0	0	0
2 - Low	254	Female	0	0	0	0	0	0
3 - High	351	Female	0	0	0	0	0	0
3 - High	352	Female	0	0	0	0	0	0
3 - High	353	Female	0	0	0	0	0	0
3 - High	354	Female	0	0	0	0	0	0

AmplifyBio Study Number: A-579-01

Individual Non-Invasive Functional Neurological Observations – Stereotypical Behaviors (present or absent)

Week 3

Group	Animal ID	Sex	Self-Mutilation	Uncontrolled Retropulsion	Repetitive Motions or Movements	Stargazing
1 - Vehicle	101	Male	0	0	0	0
1 - Vehicle	102	Male	0	0	0	0
2 - Low	201	Male	0	0	0	0
2 - Low	202	Male	0	0	0	0
2 - Low	203	Male	0	0	0	0
2 - Low	204	Male	0	0	0	1
3 - High	301	Male	0	0	0	0
3 - High	302	Male	0	0	0	0
3 - High	303	Male	0	0	1	0
3 - High	304	Male	0	0	0	1
1 - Vehicle	151	Female	0	0	0	0
1 - Vehicle	152	Female	0	0	0	0
2 - Low	251	Female	0	0	0	0
2 - Low	252	Female	0	0	0	0
2 - Low	253	Female	0	0	0	0
2 - Low	254	Female	0	0	0	0
3 - High	351	Female	0	0	0	0
3 - High	352	Female	0	0	0	0
3 - High	353	Female	0	0	0	0
3 - High	354	Female	0	0	0	0

AmplifyBio Study Number: A-579-01

Individual Non-Invasive Functional Neurological Observations – Other General Observations (present or absent)

Week 3

Group	Animal ID	Sex	Piloerection	Urine Stain	Fecal Stain	Nasal Discharge
1 - Vehicle	101	Male	0	0	0	0
1 - Vehicle	102	Male	0	0	0	0
2 - Low	201	Male	0	0	0	0
2 - Low	202	Male	0	0	0	0
2 - Low	203	Male	0	0	0	0
2 - Low	204	Male	0	0	0	0
3 - High	301	Male	0	0	0	0
3 - High	302	Male	0	0	0	0
3 - High	303	Male	0	0	0	0
3 - High	304	Male	0	0	0	0
1 - Vehicle	151	Female	0	0	0	0
1 - Vehicle	152	Female	0	0	0	0
2 - Low	251	Female	0	0	0	0
2 - Low	252	Female	0	0	0	0
2 - Low	253	Female	0	0	0	0
2 - Low	254	Female	0	0	0	0
3 - High	351	Female	0	0	0	0
3 - High	352	Female	0	0	0	0
3 - High	353	Female	0	0	0	0
3 - High	354	Female	0	0	0	0

AmplifyBio Study Number: A-579-01

Individual Non-Invasive Functional Neurological Observations – Scoring Data

Week 4

Group	Animal ID	Sex	Mentation/ Behavior	General Activity	Posture	Voluntary Movement/ Gait	Vocalizations
1 - Vehicle	101	Male	0	0	0	0	0
1 - Vehicle	102	Male	0	0	0	0	0
2 - Low	201	Male	0	0	0	0	0
2 - Low	202	Male	0	0	0	0	0
2 - Low	203	Male	0	0	0	0	0
2 - Low	204	Male	0	0	0	0	0
3 - High	301	Male	0	0	0	0	0
3 - High	302	Male	0	0	0	0	0
3 - High	303	Male	0	0	0	0	0
3 - High	304	Male	0	0	0	0	0
1 - Vehicle	151	Female	0	0	0	0	0
1 - Vehicle	152	Female	0	0	0	0	0
2 - Low	251	Female	0	0	0	0	0
2 - Low	252	Female	0	0	0	0	0
2 - Low	253	Female	0	0	0	0	0
2 - Low	254	Female	0	0	0	0	0
3 - High	351	Female	0	0	0	0	0
3 - High	352	Female	0	0	0	0	0
3 - High	353	Female	0	0	0	0	0
3 - High	354	Female	0	0	0	0	0

AmplifyBio Study Number: A-579-01

Individual Non-Invasive Functional Neurological Observations – Scoring Data (Continued)

Week 4

Group	Animal ID	Sex	Tremor Activity	Convulsive Activity	Facial Muscle Symmetry	Salivation
1 - Vehicle	101	Male	0	0	0	0
1 - Vehicle	102	Male	0	0	0	0
2 - Low	201	Male	0	0	0	0
2 - Low	202	Male	0	0	0	0
2 - Low	203	Male	0	0	0	0
2 - Low	204	Male	0	0	0	0
3 - High	301	Male	0	0	0	0
3 - High	302	Male	0	0	0	0
3 - High	303	Male	0	0	0	0
3 - High	304	Male	0	0	0	0
1 - Vehicle	151	Female	0	0	0	0
1 - Vehicle	152	Female	0	0	0	0
2 - Low	251	Female	0	0	0	0
2 - Low	252	Female	0	0	0	0
2 - Low	253	Female	0	0	0	0
2 - Low	254	Female	0	0	0	0
3 - High	351	Female	0	0	0	0
3 - High	352	Female	0	0	0	0
3 - High	353	Female	0	0	0	0
3 - High	354	Female	0	0	0	0

AmplifyBio Study Number: A-579-01

Individual Non-Invasive Functional Neurological Observations – Scoring Data (Continued)

Week 4

Group	Animal ID	Sex	Lacrimation		Eyelid Closure		Pupillary Diameter	
			Left Eye	Right Eye	Left Eye	Right Eye	Left Eye	Right Eye
1 - Vehicle	101	Male	0	0	0	0	0	0
1 - Vehicle	102	Male	0	0	0	0	0	0
2 - Low	201	Male	0	0	0	0	0	0
2 - Low	202	Male	0	0	0	0	0	0
2 - Low	203	Male	0	0	0	0	0	0
2 - Low	204	Male	0	0	0	0	0	0
3 - High	301	Male	0	0	0	0	0	0
3 - High	302	Male	0	0	0	0	0	0
3 - High	303	Male	0	0	0	0	0	0
3 - High	304	Male	0	0	0	0	0	0
1 - Vehicle	151	Female	0	0	0	0	0	0
1 - Vehicle	152	Female	0	0	0	0	0	0
2 - Low	251	Female	0	0	0	0	0	0
2 - Low	252	Female	0	0	0	0	0	0
2 - Low	253	Female	0	0	0	0	0	0
2 - Low	254	Female	0	0	0	0	0	0
3 - High	351	Female	0	0	0	0	0	0
3 - High	352	Female	0	0	0	0	0	0
3 - High	353	Female	0	0	0	0	0	0
3 - High	354	Female	0	0	0	0	0	0

AmplifyBio Study Number: A-579-01

Individual Non-Invasive Functional Neurological Observations – Stereotypical Behaviors (present or absent)

Week 4

Group	Animal ID	Sex	Self-Mutilation	Uncontrolled Retropulsion	Repetitive Motions or Movements	Stargazing
1 - Vehicle	101	Male	0	0	0	0
1 - Vehicle	102	Male	0	0	1	0
2 - Low	201	Male	0	0	0	0
2 - Low	202	Male	0	0	0	0
2 - Low	203	Male	0	0	0	0
2 - Low	204	Male	0	0	1	1
3 - High	301	Male	0	0	0	0
3 - High	302	Male	0	0	0	0
3 - High	303	Male	0	0	0	0
3 - High	304	Male	0	0	0	1
1 - Vehicle	151	Female	0	0	0	0
1 - Vehicle	152	Female	0	0	0	0
2 - Low	251	Female	0	0	0	0
2 - Low	252	Female	0	0	0	0
2 - Low	253	Female	1	0	0	0
2 - Low	254	Female	0	0	0	0
3 - High	351	Female	0	0	0	0
3 - High	352	Female	0	0	0	0
3 - High	353	Female	0	0	0	0
3 - High	354	Female	0	0	0	0

AmplifyBio Study Number: A-579-01

Individual Non-Invasive Functional Neurological Observations – Other General Observations (present or absent)

Week 4

Group	Animal ID	Sex	Piloerection	Urine Stain	Fecal Stain	Nasal Discharge
1 - Vehicle	101	Male	0	0	0	0
1 - Vehicle	102	Male	0	0	0	0
2 - Low	201	Male	0	0	0	0
2 - Low	202	Male	0	0	0	0
2 - Low	203	Male	0	0	0	0
2 - Low	204	Male	0	0	0	0
3 - High	301	Male	0	0	0	0
3 - High	302	Male	0	0	0	0
3 - High	303	Male	0	0	0	0
3 - High	304	Male	0	0	0	0
1 - Vehicle	151	Female	0	0	0	0
1 - Vehicle	152	Female	0	0	0	0
2 - Low	251	Female	0	0	0	0
2 - Low	252	Female	0	0	0	0
2 - Low	253	Female	0	0	0	0
2 - Low	254	Female	0	0	0	0
3 - High	351	Female	0	0	0	0
3 - High	352	Female	0	0	0	0
3 - High	353	Female	0	0	0	0
3 - High	354	Female	0	0	0	0

AmplifyBio Study Number: A-579-01

Individual Non-Invasive Functional Neurological Observations – Scoring Data

Week 5

Group	Animal ID	Sex	Mentation/ Behavior	General Activity	Posture	Voluntary Movement/ Gait	Vocalizations
1 - Vehicle	101	Male	0	0	0	0	0
1 - Vehicle	102	Male	0	0	0	0	0
2 - Low	201	Male	0	0	0	0	0
2 - Low	202	Male	0	0	0	0	0
2 - Low	203	Male	0	0	0	0	0
2 - Low	204	Male	0	0	0	0	0
3 - High	301	Male	0	0	0	0	0
3 - High	302	Male	0	0	0	0	0
3 - High	303	Male	0	0	0	0	0
3 - High	304	Male	0	0	0	0	0
1 - Vehicle	151	Female	0	0	0	0	0
1 - Vehicle	152	Female	0	-1	0	0	0
2 - Low	251	Female	0	0	0	0	0
2 - Low	252	Female	0	0	0	0	0
2 - Low	253	Female	0	0	0	0	0
2 - Low	254	Female	0	0	0	0	0
3 - High	351	Female	0	0	0	0	0
3 - High	352	Female	0	0	0	0	0
3 - High	353	Female	0	-1	0	0	0
3 - High	354	Female	0	0	0	0	0

AmplifyBio Study Number: A-579-01

Individual Non-Invasive Functional Neurological Observations – Scoring Data (Continued)

Week 5

Group	Animal ID	Sex	Tremor Activity	Convulsive Activity	Facial Muscle Symmetry	Salivation
1 - Vehicle	101	Male	0	0	0	0
1 - Vehicle	102	Male	0	0	0	0
2 - Low	201	Male	0	0	0	0
2 - Low	202	Male	0	0	0	0
2 - Low	203	Male	0	0	0	0
2 - Low	204	Male	0	0	0	0
3 - High	301	Male	0	0	0	0
3 - High	302	Male	0	0	0	0
3 - High	303	Male	0	0	0	0
3 - High	304	Male	0	0	0	0
1 - Vehicle	151	Female	0	0	0	0
1 - Vehicle	152	Female	0	0	0	0
2 - Low	251	Female	0	0	0	0
2 - Low	252	Female	0	0	0	0
2 - Low	253	Female	0	0	0	0
2 - Low	254	Female	0	0	0	0
3 - High	351	Female	0	0	0	0
3 - High	352	Female	0	0	0	0
3 - High	353	Female	0	0	0	0
3 - High	354	Female	0	0	0	0

AmplifyBio Study Number: A-579-01

Individual Non-Invasive Functional Neurological Observations – Scoring Data (Continued)

Week 5

Group	Animal ID	Sex	Lacrimation		Eyelid Closure		Pupillary Diameter	
			Left Eye	Right Eye	Left Eye	Right Eye	Left Eye	Right Eye
1 - Vehicle	101	Male	0	0	0	0	0	0
1 - Vehicle	102	Male	0	0	0	0	0	0
2 - Low	201	Male	0	0	0	0	0	0
2 - Low	202	Male	0	0	0	0	0	0
2 - Low	203	Male	0	0	0	0	0	0
2 - Low	204	Male	0	0	0	0	0	0
3 - High	301	Male	0	0	0	0	0	0
3 - High	302	Male	0	0	0	0	0	0
3 - High	303	Male	0	0	0	0	0	0
3 - High	304	Male	0	0	0	0	0	0
1 - Vehicle	151	Female	0	0	0	0	0	0
1 - Vehicle	152	Female	0	0	0	0	0	0
2 - Low	251	Female	0	0	0	0	0	0
2 - Low	252	Female	0	0	0	0	0	0
2 - Low	253	Female	0	0	0	0	0	0
2 - Low	254	Female	0	0	0	0	0	0
3 - High	351	Female	0	0	0	0	0	0
3 - High	352	Female	0	0	0	0	0	0
3 - High	353	Female	0	0	0	0	0	0
3 - High	354	Female	0	0	0	0	0	0

AmplifyBio Study Number: A-579-01

Individual Non-Invasive Functional Neurological Observations – Stereotypical Behaviors (present or absent)

Week 5

Group	Animal ID	Sex	Self-Mutilation	Uncontrolled Retropulsion	Repetitive Motions or Movements	Stargazing
1 - Vehicle	101	Male	0	0	0	0
1 - Vehicle	102	Male	0	0	1	0
2 - Low	201	Male	0	0	0	0
2 - Low	202	Male	0	0	0	0
2 - Low	203	Male	0	0	0	0
2 - Low	204	Male	0	0	0	1
3 - High	301	Male	0	0	0	0
3 - High	302	Male	0	0	0	0
3 - High	303	Male	0	0	0	0
3 - High	304	Male	0	0	0	1
1 - Vehicle	151	Female	0	0	0	0
1 - Vehicle	152	Female	0	0	0	0
2 - Low	251	Female	0	0	0	0
2 - Low	252	Female	0	0	0	0
2 - Low	253	Female	0	0	0	0
2 - Low	254	Female	0	0	0	0
3 - High	351	Female	0	0	0	0
3 - High	352	Female	0	0	0	0
3 - High	353	Female	0	0	0	0
3 - High	354	Female	0	0	0	0

AmplifyBio Study Number: A-579-01

Individual Non-Invasive Functional Neurological Observations – Other General Observations (present or absent)

Week 5

Group	Animal ID	Sex	Piloerection	Urine Stain	Fecal Stain	Nasal Discharge
1 - Vehicle	101	Male	0	0	0	0
1 - Vehicle	102	Male	0	0	0	0
2 - Low	201	Male	0	0	0	0
2 - Low	202	Male	0	0	0	0
2 - Low	203	Male	0	0	0	0
2 - Low	204	Male	0	0	0	0
3 - High	301	Male	0	0	0	0
3 - High	302	Male	0	0	0	0
3 - High	303	Male	0	0	0	0
3 - High	304	Male	0	0	0	0
1 - Vehicle	151	Female	0	0	0	0
1 - Vehicle	152	Female	0	0	0	0
2 - Low	251	Female	0	0	0	0
2 - Low	252	Female	0	0	0	0
2 - Low	253	Female	0	0	0	0
2 - Low	254	Female	0	0	0	0
3 - High	351	Female	0	0	0	0
3 - High	352	Female	0	0	0	0
3 - High	353	Female	0	0	0	0
3 - High	354	Female	0	0	0	0

AmplifyBio Study Number: A-579-01

Individual Non-Invasive Functional Neurological Observations – Scoring Data

Week 6

Group	Animal ID	Sex	Mentation/ Behavior	General Activity	Posture	Voluntary Movement/ Gait	Vocalizations
1 - Vehicle	101	Male	0	0	0	0	0
1 - Vehicle	102	Male	0	0	0	0	0
2 - Low	201	Male	0	0	0	0	0
2 - Low	202	Male	0	0	0	0	0
2 - Low	203	Male	0	0	0	0	0
2 - Low	204	Male	0	0	0	0	0
3 - High	301	Male	0	0	0	0	0
3 - High	302	Male	0	0	0	0	0
3 - High	303	Male	0	0	0	0	0
3 - High	304	Male	0	0	0	0	0
1 - Vehicle	151	Female	0	0	0	0	0
1 - Vehicle	152	Female	0	0	0	0	0
2 - Low	251	Female	0	1	0	0	0
2 - Low	252	Female	0	0	0	0	0
2 - Low	253	Female	0	0	0	0	0
2 - Low	254	Female	0	0	0	0	0
3 - High	351	Female	0	0	0	0	0
3 - High	352	Female	0	0	0	0	0
3 - High	353	Female	0	-1	0	0	0
3 - High	354	Female	0	0	0	0	0

AmplifyBio Study Number: A-579-01

Individual Non-Invasive Functional Neurological Observations – Scoring Data (Continued)

Week 6

Group	Animal ID	Sex	Tremor Activity	Convulsive Activity	Facial Muscle Symmetry	Salivation
1 - Vehicle	101	Male	0	0	0	0
1 - Vehicle	102	Male	0	0	0	0
2 - Low	201	Male	0	0	0	0
2 - Low	202	Male	0	0	0	0
2 - Low	203	Male	0	0	0	0
2 - Low	204	Male	0	0	0	0
3 - High	301	Male	0	0	0	0
3 - High	302	Male	0	0	0	0
3 - High	303	Male	0	0	0	0
3 - High	304	Male	0	0	0	0
1 - Vehicle	151	Female	0	0	0	0
1 - Vehicle	152	Female	0	0	0	0
2 - Low	251	Female	0	0	0	0
2 - Low	252	Female	0	0	0	0
2 - Low	253	Female	0	0	0	0
2 - Low	254	Female	0	0	0	0
3 - High	351	Female	0	0	0	0
3 - High	352	Female	0	0	0	0
3 - High	353	Female	0	0	0	0
3 - High	354	Female	0	0	0	0

AmplifyBio Study Number: A-579-01

Individual Non-Invasive Functional Neurological Observations – Scoring Data (Continued)

Week 6

Group	Animal ID	Sex	Lacrimation		Eyelid Closure		Pupillary Diameter	
			Left Eye	Right Eye	Left Eye	Right Eye	Left Eye	Right Eye
1 - Vehicle	101	Male	0	0	0	0	0	0
1 - Vehicle	102	Male	0	0	0	0	0	0
2 - Low	201	Male	0	0	0	0	0	0
2 - Low	202	Male	0	0	0	0	0	0
2 - Low	203	Male	0	0	0	0	0	0
2 - Low	204	Male	0	0	0	0	0	0
3 - High	301	Male	0	0	0	0	0	0
3 - High	302	Male	0	0	0	0	0	0
3 - High	303	Male	0	0	0	0	0	0
3 - High	304	Male	0	0	0	0	0	0
1 - Vehicle	151	Female	0	0	0	0	0	0
1 - Vehicle	152	Female	0	0	0	0	0	0
2 - Low	251	Female	0	0	0	0	0	0
2 - Low	252	Female	0	0	0	0	0	0
2 - Low	253	Female	0	0	0	0	0	0
2 - Low	254	Female	0	0	0	0	0	0
3 - High	351	Female	0	0	0	0	0	0
3 - High	352	Female	0	0	0	0	0	0
3 - High	353	Female	0	0	0	0	0	0
3 - High	354	Female	0	0	0	0	0	0

AmplifyBio Study Number: A-579-01

Individual Non-Invasive Functional Neurological Observations – Stereotypical Behaviors (present or absent)

Week 6

Group	Animal ID	Sex	Self-Mutilation	Uncontrolled Retropulsion	Repetitive Motions or Movements	Stargazing
1 - Vehicle	101	Male	0	0	0	0
1 - Vehicle	102	Male	0	0	0	0
2 - Low	201	Male	0	0	0	0
2 - Low	202	Male	0	0	0	0
2 - Low	203	Male	0	0	0	0
2 - Low	204	Male	0	0	1	1
3 - High	301	Male	0	0	0	0
3 - High	302	Male	0	0	0	0
3 - High	303	Male	0	0	0	0
3 - High	304	Male	0	0	0	1
1 - Vehicle	151	Female	0	0	0	0
1 - Vehicle	152	Female	0	0	0	0
2 - Low	251	Female	0	0	1	0
2 - Low	252	Female	0	0	0	0
2 - Low	253	Female	0	0	0	0
2 - Low	254	Female	0	0	0	0
3 - High	351	Female	0	0	0	0
3 - High	352	Female	0	0	0	0
3 - High	353	Female	0	0	0	0
3 - High	354	Female	0	0	0	0

AmplifyBio Study Number: A-579-01

Individual Non-Invasive Functional Neurological Observations – Other General Observations (present or absent)

Week 6

Group	Animal ID	Sex	Piloerection	Urine Stain	Fecal Stain	Nasal Discharge
1 - Vehicle	101	Male	0	0	0	0
1 - Vehicle	102	Male	0	0	0	0
2 - Low	201	Male	0	0	0	0
2 - Low	202	Male	0	0	0	0
2 - Low	203	Male	0	0	0	0
2 - Low	204	Male	0	0	0	0
3 - High	301	Male	0	0	0	0
3 - High	302	Male	0	0	0	0
3 - High	303	Male	0	0	0	0
3 - High	304	Male	0	0	0	0
1 - Vehicle	151	Female	0	0	0	0
1 - Vehicle	152	Female	0	0	0	0
2 - Low	251	Female	0	0	0	0
2 - Low	252	Female	0	0	0	0
2 - Low	253	Female	0	0	0	0
2 - Low	254	Female	0	0	0	0
3 - High	351	Female	0	0	0	0
3 - High	352	Female	0	0	0	0
3 - High	353	Female	0	0	0	0
3 - High	354	Female	0	0	0	0

AmplifyBio Study Number: A-579-01

Individual Non-Invasive Functional Neurological Observations – Scoring Data

Week 7

Group	Animal ID	Sex	Mentation/ Behavior	General Activity	Posture	Voluntary Movement/ Gait	Vocalizations
1 - Vehicle	102	Male	0	0	0	0	0
2 - Low	203	Male	0	0	0	0	0
2 - Low	204	Male	0	0	0	0	0
3 - High	303	Male	0	0	0	0	0
3 - High	304	Male	0	0	0	0	0
1 - Vehicle	152	Female	0	-1	0	0	0
2 - Low	253	Female	0	0	0	0	0
2 - Low	254	Female	0	0	0	0	0
3 - High	353	Female	0	0	0	0	0
3 - High	354	Female	0	0	0	0	0

AmplifyBio Study Number: A-579-01

Individual Non-Invasive Functional Neurological Observations – Scoring Data (Continued)

Week 7

Group	Animal ID	Sex	Tremor Activity	Convulsive Activity	Facial Muscle Symmetry	Salivation
1 - Vehicle	102	Male	0	0	0	0
2 - Low	203	Male	0	0	0	0
2 - Low	204	Male	0	0	0	0
3 - High	303	Male	0	0	0	0
3 - High	304	Male	0	0	0	0
1 - Vehicle	152	Female	0	0	0	0
2 - Low	253	Female	0	0	0	0
2 - Low	254	Female	0	0	0	0
3 - High	353	Female	0	0	0	0
3 - High	354	Female	0	0	0	0

AmplifyBio Study Number: A-579-01

Individual Non-Invasive Functional Neurological Observations – Scoring Data (Continued)

Week 7

Group	Animal ID	Sex	Lacrimation		Eyelid Closure		Pupillary Diameter	
			Left Eye	Right Eye	Left Eye	Right Eye	Left Eye	Right Eye
1 - Vehicle	102	Male	0	0	0	0	0	0
2 - Low	203	Male	0	0	0	0	0	0
2 - Low	204	Male	0	0	0	0	0	0
3 - High	303	Male	0	0	0	0	0	0
3 - High	304	Male	0	0	0	0	0	0
1 - Vehicle	152	Female	0	0	0	0	0	0
2 - Low	253	Female	0	0	0	0	0	0
2 - Low	254	Female	0	0	0	0	0	0
3 - High	353	Female	0	0	0	0	0	0
3 - High	354	Female	0	0	0	0	0	0

AmplifyBio Study Number: A-579-01

Individual Non-Invasive Functional Neurological Observations – Stereotypical Behaviors (present or absent)

Week 7

Group	Animal ID	Sex	Self-Mutilation	Uncontrolled Retropulsion	Repetitive Motions or Movements	Stargazing
1 - Vehicle	102	Male	0	0	0	0
2 - Low	203	Male	0	0	0	0
2 - Low	204	Male	0	0	1	1
3 - High	303	Male	0	0	0	0
3 - High	304	Male	0	0	0	1
1 - Vehicle	152	Female	0	0	0	0
2 - Low	253	Female	0	0	0	0
2 - Low	254	Female	0	0	0	0
3 - High	353	Female	0	0	1	0
3 - High	354	Female	0	0	0	0

AmplifyBio Study Number: A-579-01

Individual Non-Invasive Functional Neurological Observations – Other General Observations (present or absent)

Week 7

Group	Animal ID	Sex	Piloerection	Urine Stain	Fecal Stain	Nasal Discharge
1 - Vehicle	102	Male	0	0	0	0
2 - Low	203	Male	0	0	0	0
2 - Low	204	Male	0	0	0	0
3 - High	303	Male	0	0	0	0
3 - High	304	Male	0	0	0	0
1 - Vehicle	152	Female	0	0	0	0
2 - Low	253	Female	0	0	0	0
2 - Low	254	Female	0	0	0	0
3 - High	353	Female	0	0	0	0
3 - High	354	Female	0	0	0	0

AmplifyBio Study Number: A-579-01

Individual Non-Invasive Functional Neurological Observations – Scoring Data

Week 8

Group	Animal ID	Sex	Mentation/ Behavior	General Activity	Posture	Voluntary Movement/ Gait	Vocalizations
1 - Vehicle	102	Male	0	0	0	0	0
2 - Low	203	Male	0	0	0	0	0
2 - Low	204	Male	0	0	0	0	0
3 - High	303	Male	0	0	0	0	0
3 - High	304	Male	0	0	0	0	0
1 - Vehicle	152	Female	0	-1	0	0	0
2 - Low	253	Female	0	0	0	0	0
2 - Low	254	Female	0	0	0	0	0
3 - High	353	Female	0	0	0	0	0
3 - High	354	Female	0	0	0	0	0

AmplifyBio Study Number: A-579-01

Individual Non-Invasive Functional Neurological Observations – Scoring Data (Continued)

Week 8

Group	Animal ID	Sex	Tremor Activity	Convulsive Activity	Facial Muscle Symmetry	Salivation
1 - Vehicle	102	Male	0	0	0	0
2 - Low	203	Male	0	0	0	0
2 - Low	204	Male	0	0	0	0
3 - High	303	Male	0	0	0	0
3 - High	304	Male	0	0	0	0
1 - Vehicle	152	Female	0	0	0	0
2 - Low	253	Female	0	0	0	0
2 - Low	254	Female	0	0	0	0
3 - High	353	Female	0	0	0	0
3 - High	354	Female	0	0	0	0

AmplifyBio Study Number: A-579-01

Individual Non-Invasive Functional Neurological Observations – Scoring Data (Continued)

Week 8

Group	Animal ID	Sex	Lacrimation		Eyelid Closure		Pupillary Diameter	
			Left Eye	Right Eye	Left Eye	Right Eye	Left Eye	Right Eye
1 - Vehicle	102	Male	0	0	0	0	0	0
2 - Low	203	Male	0	0	0	0	0	0
2 - Low	204	Male	0	0	0	0	0	0
3 - High	303	Male	0	0	0	0	0	0
3 - High	304	Male	0	0	0	0	0	0
1 - Vehicle	152	Female	0	0	0	0	0	0
2 - Low	253	Female	0	0	0	0	0	0
2 - Low	254	Female	0	0	0	0	0	0
3 - High	353	Female	0	0	0	0	0	0
3 - High	354	Female	0	0	0	0	0	0

AmplifyBio Study Number: A-579-01

Individual Non-Invasive Functional Neurological Observations – Stereotypical Behaviors (present or absent)

Week 8

Group	Animal ID	Sex	Self-Mutilation	Uncontrolled Retropulsion	Repetitive Motions or Movements	Stargazing
1 - Vehicle	102	Male	0	0	0	0
2 - Low	203	Male	0	0	0	0
2 - Low	204	Male	0	0	1	1
3 - High	303	Male	0	0	0	0
3 - High	304	Male	0	0	0	1
1 - Vehicle	152	Female	0	0	0	0
2 - Low	253	Female	0	0	0	0
2 - Low	254	Female	0	0	0	0
3 - High	353	Female	0	0	0	0
3 - High	354	Female	0	0	0	0

AmplifyBio Study Number: A-579-01

Individual Non-Invasive Functional Neurological Observations – Other General Observations (present or absent)

Week 8

Group	Animal ID	Sex	Piloerection	Urine Stain	Fecal Stain	Nasal Discharge
1 - Vehicle	102	Male	0	0	0	0
2 - Low	203	Male	0	0	0	0
2 - Low	204	Male	0	0	0	0
3 - High	303	Male	0	0	0	0
3 - High	304	Male	0	0	0	0
1 - Vehicle	152	Female	0	0	0	0
2 - Low	253	Female	0	0	0	0
2 - Low	254	Female	0	0	0	0
3 - High	353	Female	0	0	0	0
3 - High	354	Female	0	0	0	0

AmplifyBio Study Number: A-579-01

Individual Non-Invasive Functional Neurological Observations – Scoring Data

Week 9

Group	Animal ID	Sex	Mentation/ Behavior	General Activity	Posture	Voluntary Movement/ Gait	Vocalizations
1 - Vehicle	102	Male	0	-1	0	0	0
2 - Low	203	Male	0	0	0	0	0
2 - Low	204	Male	0	0	0	0	0
3 - High	303	Male	0	0	0	0	0
3 - High	304	Male	0	0	0	0	0
1 - Vehicle	152	Female	0	-1	0	0	0
2 - Low	253	Female	0	0	0	0	0
2 - Low	254	Female	0	0	0	0	0
3 - High	353	Female	0	0	0	0	0
3 - High	354	Female	0	0	0	0	0

AmplifyBio Study Number: A-579-01

Individual Non-Invasive Functional Neurological Observations – Scoring Data (Continued)

Week 9

Group	Animal ID	Sex	Tremor Activity	Convulsive Activity	Facial Muscle Symmetry	Salivation
1 - Vehicle	102	Male	0	0	0	0
2 - Low	203	Male	0	0	0	0
2 - Low	204	Male	0	0	0	0
3 - High	303	Male	0	0	0	0
3 - High	304	Male	0	0	0	0
1 - Vehicle	152	Female	0	0	0	0
2 - Low	253	Female	0	0	0	0
2 - Low	254	Female	0	0	0	0
3 - High	353	Female	0	0	0	0
3 - High	354	Female	0	0	0	0

AmplifyBio Study Number: A-579-01

Individual Non-Invasive Functional Neurological Observations – Scoring Data (Continued)

Week 9

Group	Animal ID	Sex	Lacrimation		Eyelid Closure		Pupillary Diameter	
			Left Eye	Right Eye	Left Eye	Right Eye	Left Eye	Right Eye
1 - Vehicle	102	Male	0	0	0	0	0	0
2 - Low	203	Male	0	0	0	0	0	0
2 - Low	204	Male	0	0	0	0	0	0
3 - High	303	Male	0	0	0	0	0	0
3 - High	304	Male	0	0	0	0	0	0
1 - Vehicle	152	Female	0	0	0	0	0	0
2 - Low	253	Female	0	0	0	0	0	0
2 - Low	254	Female	0	0	0	0	0	0
3 - High	353	Female	0	0	0	0	0	0
3 - High	354	Female	0	0	0	0	0	0

AmplifyBio Study Number: A-579-01

Individual Non-Invasive Functional Neurological Observations – Stereotypical Behaviors (present or absent)

Week 9

Group	Animal ID	Sex	Self-Mutilation	Uncontrolled Retropulsion	Repetitive Motions or Movements	Stargazing
1 - Vehicle	102	Male	0	0	0	0
2 - Low	203	Male	0	0	0	0
2 - Low	204	Male	0	0	1	0
3 - High	303	Male	0	0	0	0
3 - High	304	Male	0	0	0	0
1 - Vehicle	152	Female	0	0	0	0
2 - Low	253	Female	0	0	0	0
2 - Low	254	Female	0	0	0	0
3 - High	353	Female	0	0	0	0
3 - High	354	Female	0	0	0	0

AmplifyBio Study Number: A-579-01

Individual Non-Invasive Functional Neurological Observations – Other General Observations (present or absent)

Week 9

Group	Animal ID	Sex	Piloerection	Urine Stain	Fecal Stain	Nasal Discharge
1 - Vehicle	102	Male	0	0	0	0
2 - Low	203	Male	0	0	0	0
2 - Low	204	Male	0	0	0	0
3 - High	303	Male	0	0	0	0
3 - High	304	Male	0	0	0	0
1 - Vehicle	152	Female	0	0	0	0
2 - Low	253	Female	0	0	0	0
2 - Low	254	Female	0	0	0	0
3 - High	353	Female	0	0	0	0
3 - High	354	Female	0	0	0	0

AmplifyBio Study Number: A-579-01

Individual Non-Invasive Functional Neurological Observations – Scoring Data

Week 10

Group	Animal ID	Sex	Mentation/ Behavior	General Activity	Posture	Voluntary Movement/ Gait	Vocalizations
1 - Vehicle	102	Male	0	0	0	0	0
2 - Low	203	Male	0	0	0	0	0
2 - Low	204	Male	0	0	0	0	0
3 - High	303	Male	0	0	0	0	0
3 - High	304	Male	0	0	0	0	0
1 - Vehicle	152	Female	0	-1	0	0	0
2 - Low	253	Female	0	0	0	0	0
2 - Low	254	Female	0	0	0	0	0
3 - High	353	Female	0	-1	0	0	0
3 - High	354	Female	0	0	0	0	0

AmplifyBio Study Number: A-579-01

Individual Non-Invasive Functional Neurological Observations – Scoring Data (Continued)

Week 10

Group	Animal ID	Sex	Tremor Activity	Convulsive Activity	Facial Muscle Symmetry	Salivation
1 - Vehicle	102	Male	0	0	0	0
2 - Low	203	Male	0	0	0	0
2 - Low	204	Male	0	0	0	0
3 - High	303	Male	0	0	0	0
3 - High	304	Male	0	0	0	0
1 - Vehicle	152	Female	0	0	0	0
2 - Low	253	Female	0	0	0	0
2 - Low	254	Female	0	0	0	0
3 - High	353	Female	0	0	0	0
3 - High	354	Female	0	0	0	0

AmplifyBio Study Number: A-579-01

Individual Non-Invasive Functional Neurological Observations – Scoring Data (Continued)

Week 10

Group	Animal ID	Sex	Lacrimation		Eyelid Closure		Pupillary Diameter	
			Left Eye	Right Eye	Left Eye	Right Eye	Left Eye	Right Eye
1 - Vehicle	102	Male	0	0	0	0	0	0
2 - Low	203	Male	0	0	0	0	0	0
2 - Low	204	Male	0	0	0	0	0	0
3 - High	303	Male	0	0	0	0	0	0
3 - High	304	Male	0	0	0	0	0	0
1 - Vehicle	152	Female	0	0	0	0	0	0
2 - Low	253	Female	0	0	0	0	0	0
2 - Low	254	Female	0	0	0	0	0	0
3 - High	353	Female	0	0	0	0	0	0
3 - High	354	Female	0	0	0	0	0	0

AmplifyBio Study Number: A-579-01

Individual Non-Invasive Functional Neurological Observations – Stereotypical Behaviors (present or absent)

Week 10

Group	Animal ID	Sex	Self-Mutilation	Uncontrolled Retropulsion	Repetitive Motions or Movements	Stargazing
1 - Vehicle	102	Male	0	0	0	0
2 - Low	203	Male	0	0	0	0
2 - Low	204	Male	0	0	1	1
3 - High	303	Male	0	0	0	0
3 - High	304	Male	0	0	0	1
1 - Vehicle	152	Female	0	0	0	0
2 - Low	253	Female	0	0	0	0
2 - Low	254	Female	0	0	0	0
3 - High	353	Female	0	0	0	0
3 - High	354	Female	0	0	0	0

AmplifyBio Study Number: A-579-01

Individual Non-Invasive Functional Neurological Observations – Other General Observations (present or absent)

Week 10

Group	Animal ID	Sex	Piloerection	Urine Stain	Fecal Stain	Nasal Discharge
1 - Vehicle	102	Male	0	0	0	0
2 - Low	203	Male	0	0	0	0
2 - Low	204	Male	0	0	0	0
3 - High	303	Male	0	0	0	0
3 - High	304	Male	0	0	0	0
1 - Vehicle	152	Female	0	0	0	0
2 - Low	253	Female	0	0	0	0
2 - Low	254	Female	0	0	0	0
3 - High	353	Female	0	0	0	0
3 - High	354	Female	0	0	0	0

AmplifyBio Study Number: A-579-01

Individual Non-Invasive Functional Neurological Observations – Scoring Data

Week 11

Group	Animal ID	Sex	Mentation/ Behavior	General Activity	Posture	Voluntary Movement/ Gait	Vocalizations
1 - Vehicle	102	Male	0	-1	0	0	0
2 - Low	203	Male	0	0	0	0	0
2 - Low	204	Male	0	0	0	0	0
3 - High	303	Male	0	0	0	0	0
3 - High	304	Male	0	0	0	0	0
1 - Vehicle	152	Female	0	-1	0	0	0
2 - Low	253	Female	0	0	0	0	0
2 - Low	254	Female	0	-1	0	0	0
3 - High	353	Female	0	0	0	0	0
3 - High	354	Female	0	0	0	0	0

AmplifyBio Study Number: A-579-01

Individual Non-Invasive Functional Neurological Observations – Scoring Data (Continued)

Week 11

Group	Animal ID	Sex	Tremor Activity	Convulsive Activity	Facial Muscle Symmetry	Salivation
1 - Vehicle	102	Male	0	0	0	0
2 - Low	203	Male	0	0	0	0
2 - Low	204	Male	0	0	0	0
3 - High	303	Male	0	0	0	0
3 - High	304	Male	0	0	0	0
1 - Vehicle	152	Female	0	0	0	0
2 - Low	253	Female	0	0	0	0
2 - Low	254	Female	0	0	0	0
3 - High	353	Female	0	0	0	0
3 - High	354	Female	0	0	0	0

AmplifyBio Study Number: A-579-01

Individual Non-Invasive Functional Neurological Observations – Scoring Data (Continued)

Week 11

Group	Animal ID	Sex	Lacrimation		Eyelid Closure		Pupillary Diameter	
			Left Eye	Right Eye	Left Eye	Right Eye	Left Eye	Right Eye
1 - Vehicle	102	Male	0	0	0	0	0	0
2 - Low	203	Male	0	0	0	0	0	0
2 - Low	204	Male	0	0	0	0	0	0
3 - High	303	Male	0	0	0	0	0	0
3 - High	304	Male	0	0	0	0	0	0
1 - Vehicle	152	Female	0	0	0	0	0	0
2 - Low	253	Female	0	0	0	0	0	0
2 - Low	254	Female	0	0	0	0	0	0
3 - High	353	Female	0	0	0	0	0	0
3 - High	354	Female	0	0	0	0	0	0

AmplifyBio Study Number: A-579-01

Individual Non-Invasive Functional Neurological Observations – Stereotypical Behaviors (present or absent)

Week 11

Group	Animal ID	Sex	Self-Mutilation	Uncontrolled Retropulsion	Repetitive Motions or Movements	Stargazing
1 - Vehicle	102	Male	0	0	0	0
2 - Low	203	Male	0	0	0	0
2 - Low	204	Male	0	0	1	0
3 - High	303	Male	0	0	0	0
3 - High	304	Male	0	0	0	0
1 - Vehicle	152	Female	0	0	0	0
2 - Low	253	Female	0	0	0	0
2 - Low	254	Female	0	0	0	0
3 - High	353	Female	0	0	0	0
3 - High	354	Female	0	0	0	0

AmplifyBio Study Number: A-579-01

Individual Non-Invasive Functional Neurological Observations – Other General Observations (present or absent)

Week 11

Group	Animal ID	Sex	Piloerection	Urine Stain	Fecal Stain	Nasal Discharge
1 - Vehicle	102	Male	0	0	0	0
2 - Low	203	Male	0	0	0	0
2 - Low	204	Male	0	0	0	0
3 - High	303	Male	0	0	0	0
3 - High	304	Male	0	0	0	0
1 - Vehicle	152	Female	0	0	0	0
2 - Low	253	Female	0	0	0	0
2 - Low	254	Female	0	0	0	0
3 - High	353	Female	0	0	0	0
3 - High	354	Female	0	0	0	0

AmplifyBio Study Number: A-579-01

Individual Non-Invasive Functional Neurological Observations – Scoring Data

Week 12

Group	Animal ID	Sex	Mentation/ Behavior	General Activity	Posture	Voluntary Movement/ Gait	Vocalizations
1 - Vehicle	102	Male	0	0	0	0	0
2 - Low	203	Male	0	0	0	0	0
2 - Low	204	Male	0	0	0	0	0
3 - High	303	Male	0	0	0	0	0
3 - High	304	Male	0	0	0	0	0
1 - Vehicle	152	Female	0	-1	0	0	0
2 - Low	253	Female	0	0	0	0	0
2 - Low	254	Female	0	0	0	0	0
3 - High	353	Female	0	0	0	0	0
3 - High	354	Female	0	0	0	0	0

AmplifyBio Study Number: A-579-01

Individual Non-Invasive Functional Neurological Observations – Scoring Data (Continued)

Week 12

Group	Animal ID	Sex	Tremor Activity	Convulsive Activity	Facial Muscle Symmetry	Salivation
1 - Vehicle	102	Male	0	0	0	0
2 - Low	203	Male	0	0	0	0
2 - Low	204	Male	0	0	0	0
3 - High	303	Male	0	0	0	0
3 - High	304	Male	0	0	0	0
1 - Vehicle	152	Female	0	0	0	0
2 - Low	253	Female	0	0	0	0
2 - Low	254	Female	0	0	0	0
3 - High	353	Female	0	0	0	0
3 - High	354	Female	0	0	0	0

AmplifyBio Study Number: A-579-01

Individual Non-Invasive Functional Neurological Observations – Scoring Data (Continued)

Week 12

Group	Animal ID	Sex	Lacrimation		Eyelid Closure		Pupillary Diameter	
			Left Eye	Right Eye	Left Eye	Right Eye	Left Eye	Right Eye
1 - Vehicle	102	Male	0	0	0	0	0	0
2 - Low	203	Male	0	0	0	0	0	0
2 - Low	204	Male	0	0	0	0	0	0
3 - High	303	Male	0	0	0	0	0	0
3 - High	304	Male	0	0	0	0	0	0
1 - Vehicle	152	Female	0	0	0	0	0	0
2 - Low	253	Female	0	0	0	0	0	0
2 - Low	254	Female	0	0	0	0	0	0
3 - High	353	Female	0	0	0	0	0	0
3 - High	354	Female	0	0	0	0	0	0

AmplifyBio Study Number: A-579-01

Individual Non-Invasive Functional Neurological Observations – Stereotypical Behaviors (present or absent)

Week 12

Group	Animal ID	Sex	Self-Mutilation	Uncontrolled Retropulsion	Repetitive Motions or Movements	Stargazing
1 - Vehicle	102	Male	0	0	0	0
2 - Low	203	Male	0	0	0	0
2 - Low	204	Male	0	0	1	1
3 - High	303	Male	0	0	0	0
3 - High	304	Male	0	0	0	1
1 - Vehicle	152	Female	0	0	0	0
2 - Low	253	Female	0	0	0	0
2 - Low	254	Female	0	0	0	0
3 - High	353	Female	0	0	0	0
3 - High	354	Female	0	0	0	0

AmplifyBio Study Number: A-579-01

Individual Non-Invasive Functional Neurological Observations – Other General Observations (present or absent)

Week 12

Group	Animal ID	Sex	Piloerection	Urine Stain	Fecal Stain	Nasal Discharge
1 - Vehicle	102	Male	0	0	0	0
2 - Low	203	Male	0	0	0	0
2 - Low	204	Male	0	0	0	0
3 - High	303	Male	0	0	0	0
3 - High	304	Male	0	0	0	0
1 - Vehicle	152	Female	0	0	0	0
2 - Low	253	Female	0	0	0	0
2 - Low	254	Female	0	0	0	0
3 - High	353	Female	0	0	0	0
3 - High	354	Female	0	0	0	0

Attachment 3. Study Specific Method

Study Specific Method (SSM) for Non-Invasive Functional Neurological Observations in Cynomolgus Monkeys

AmplifyBio Study Number: A-579-01

AmplifyBio
1425 State Route 142 (Plain City-Georgesville Road)
West Jefferson, OH 43162

1 PURPOSE

The purpose of this Study Specific Method (SSM) is to describe the procedures and scoring criteria to perform Non-Invasive Functional Neurological Observations in Cynomolgus monkeys.

2 PROCEDURE

The scoring criteria outlined below will be used for the Non-Invasive Neurological Functional Observations as indicated in protocol section 11.4. The animals will be allotted an at least 30-minute period of undisturbed activity in the animal room (which includes the absence of study personnel) prior to data collection. After collections, individual and summary tables will be manually generated from the scoring paperwork, but these data will not have statistical analysis performed.

All observations will be performed by trained technicians, signed off on the Functional Observational Battery, while the animals are single housed in their home cage. If an animal cannot be scored on an observation, a "Not taken (NT)" will be recorded.

3 DEFINITIONS

Ataxia: Uncoordinated movements.

Clonic Convulsion: A convulsion in which the contractions are intermittent, the muscles are alternately contracting and relaxing.

Lacrimation: The secretion of tears.

Mentation: Mental activity.

Piloerection: The erection of hair.

Retropulsion: Backward walking.

Stereotypy: The constant repetition of certain movements or behaviors.

Tonic Convulsion: A convulsion in which muscle contraction is sustained.

4 SCORING CRITERIA

Mentation/Behavior

-2) stupor – extremely low arousal state; no activity
-1) depressed/dull – little activity; may be supine but awake
0) normal – active and alert
1) anxious – enhanced arousal state (agitation, alarmed, grimace)
2) aggressive – frenetic, agitated, hyperkinetic behavior; may include stereotypic behaviors, loud vocalizations

General Activity

-2) none – animal remains motionless
-1) minimal – animal may be in one location during entire observation period but shows response to observer presence or moves from floor to perch/perch to floor but with no exploratory activity
0) normal – intermittent activity, may include periods of sitting, climbing, exploration
1) heightened – animal remains in one position for approximately <30 s, repeat repositioning
2) severe – frenetic, wildly moving in cage; no periods of sitting or motionlessness

Posture

-3) recumbent
-2) falling or leaning when seated or moving
-1) wide based stance when moving
0) normal
1) excessive rigidity

Voluntary Movement/Gait

Note affected limb(s) if applicable.

-3) limb paralysis
-2) limb weakness
-1) ataxia
0) normal

Vocalizations

0) normal (minimal or no distressed vocalization)
1) moderate distressed vocalization
2) excessive, repeated distressed vocalization
3) loud, extreme, and repeated distressed vocalization

Tremor Activity

Note limb or body location of tremor if present.

0) none
1) slight (may include muscle twitching)
2) moderate
3) severe

Convulsive Activity

0) none
1) clonic seizures (jerking, contractions followed by relaxation)
2) tonic seizures (repetitive moderate/severe whole-body tremors, body is rigid and arched)
3) tonic/clonic seizures (loss of righting and alternating tonic and clonic seizures)

Facial Muscle Symmetry

A central line is envisioned down the anterior aspect of the head, dividing the face into two equal sides to compare one side of the face to the other.

-2) moderate to severe asymmetry (abnormal facial expressions or muscle positioning on one side only)
-1) mild asymmetry (abnormal facial expressions or muscle positioning on one side only)
0) normal (facial expressions equally displayed on both sides of the face)

Salivation

0) normal (none)
1) slight (area around mouth is wet)
2) moderate (area around mouth is excessively wet)
3) excessive (area around mouth is excessively wet along with excessive drool)

Lacrimation

Observe both eyes individually:

0) normal (none)
1) slight
2) moderate
3) severe

Eyelid Closure

Observe both eyes individually:

-2) eyelids nearly or completely closed
-1) slightly drooping or partially closed eyelids
0) normal (eyelids fully open)

Pupillary Diameter

Observe both eyes individually:

-1) pinpoint pupil (miosis)
0) normal pupil (¼ to ½ dilated)
1) pupil fully dilated (mydriasis)

Stereotypical Behaviors

Self-mutilation

0) absent
1) present

Uncontrolled retropulsion

0) absent
1) present

Repetitive motions or movements

0) absent
1) present

Stargazing

0) absent
1) present

Other General Observations

Piloerection

0) absent
1) present

Urine Stain

0) absent
1) present

Fecal Stain

0) absent
1) present

Nasal discharge

0) absent
1) present

5 REVISIONS TO SSM

Revisions or changes to this SSM will be managed within Veeva QualityDocs and identified with a new version number. Any changes or corrections made to the procedure during execution will be approved by the Study Director before the change is executed and the approval will be documented on the SSM. Any minor corrections to the method which do not affect data analysis may be corrected directly on the method without a formal version change.

Document Approvals

Number: METHOD-00083 Version: 1.0

Approval Task<br>Verdict: Approve	Sapna Varia,<br>(svaria@amplify-bio.com)<br>Approval<br>15-Jun-2023 23:45:06 GMT+0000

Appendix G Nerve Conduction Evaluation Contributing Scientist Report

A 12-Week Single Dose Biodistribution and Safety Study in Cynomolgus Monkeys

Nerve Conduction Evaluation Contributing Scientist Report

May 30, 2024

Study Number A-579-01

Testing Facility
AmplifyBio
1425 State Route 142 (Plain City-Georgesville Road)
West Jefferson, OH 43162

SIGNATURES

Prepared by:

DocuSigned by:

Signer Name: Bryce Visnick
Signing Reason: I am the author of this document
Signing Time: 5/30/2024 | 9:18:51 AM EDT
7EEA9E2004AC4CE0A0F65E226014AC05

5/30/2024

Bryce Visnick, BS
Contributing Scientist

Date

TABLE OF CONTENTS

Page

Signatures **2**

1.0 Introduction **4**

2.0 Study Design and Methods **4**

2.1 Data Capture and Analysis 4

3.0 Deviations **5**

4.0 Results **5**

5.0 Conclusions **6**

LIST OF ATTACHMENTS

Attachment 1. CMAP and Nerve Conduction Data Tables 7

Attachment 2. Study Specific Method 14

1.0 INTRODUCTION

This contributing scientist report summarizes the nerve conduction data collection and analysis for Study Number A-579-01 and any findings that may have been associated with it.

2.0 STUDY DESIGN AND METHODS

The study design relating to nerve conduction evaluations is outlined in Table 1.

Table 1. Experimental Design

Group	Target Dose (Total vg)	Target Dose Volume (mL)	Animal Identification Numbers			
			6-Week Cohort[a]		12-Week Cohort[b]	
			Male	Female	Male	Female
1 – Control	0	3	101	151	102	152
2 – Low	6×10^{13}	3	201-202	251-252	203-204	253-254
3 – High	1.2×10^{14}	3	301-302	351-352	303-304	353-354

vg=vector genome
a. Necropsy Day 43.
b. Necropsy Day 85.

All groups were dosed on Day 1 via a single intrathecal slow infusion over approximately 6 hours via a surgically implanted lumbar catheter (see QE-001542 for exceptions).

2.1 DATA CAPTURE AND ANALYSIS

Nicolet Viking EDX software was used to collect and analyze the nerve conduction data.

Tibial nerve Compound Muscle Action Potential (CMAP) waveforms were collected at the following time points:

- Pretest: all animals prior to dosing
- Week 6: all animals (see DEV-002401 for exceptions)
- Week 12: all surviving animals

Prior to each nerve conduction data collection, animals were sedated with ketamine. Once properly sedated, electrodes were briefly placed over the endplate zone of abductor hallucis muscle of each hindlimb. The tibial nerve conduction was evaluated with recorded Compound Muscle Action Potential (CMAP) traces. The CMAP recordings were used to measure latency, amplitude, and duration, and to calculate nerve conduction velocity. CMAPs were recorded bilaterally and reported as separate values. Details of the nerve conduction assessment procedure can be found in Attachment 2.

3.0 DEVIATIONS

QE-001542: On Day 1, the dose administration was not exactly 6 hours for all three groups.

For Group 1, Animal 101 had dose administered starting at 0957. There is documentation that the pump was paused at 1414 due to the Huber needle detaching from the port, however, there is no documentation of when the pump was restarted following this pause. It is unclear how long the dose was administered with the pump stop time documented at 1632. For the other Group 1 animals, the dose administration time was as followed: Animal 102 (5hrs 58mins), Animal 151 (5hrs 56mins), and Animal 152 (5hrs 3mins).

For Group 2, the dose administration time for each animal is as followed: Animal 201 (5hrs 56mins), Animal 202 (5hrs 56mins), Animal 203 (5hrs 57mins), Animal 204 (5hrs 55mins), Animal 251 (5hrs 56mins), Animal 252 (5hrs 54mins), Animal 253 (5hrs 56mins), and Animal 254 (5hrs 55mins).

For Group 3, the dose administration time for each animal is as followed: Animal 301 (5hrs 55mins), Animal 302 (5hrs 55mins), Animal 303 (5hrs 51mins), Animal 304 (6hrs 2mins), Animal 351 (5hrs 56mins), Animal 352 (5hrs 56mins), Animal 353 (5hrs 57mins), and Animal 354 (5hrs 58mins).

The impact of +/- 10 minutes on a 6 hour treatment is not likely to have an impact. The failed attempt to give full treatment to Animal 152 will have no effect since this animal was a control and not receiving test article.

DEV-002401: All animals were assessed for nerve conduction velocity on Week 6 instead of only the animals scheduled for Week 6 necropsy.

There is no study impact due to the inadvertently NCV testing for all animals instead of protocol-specified Week 6 animals only. It would be atypical for these kinds of evaluations to be performed only on one group at any timepoint and would normally be performed on all animals. This additional data does not impact the data integrity but gives a supportive information regarding animal welfare.

4.0 RESULTS

There were no abnormal nerve conduction findings observed in the tibial nerve prior to the initiation of dosing (Pretest Assessment).

Following the administration of ARM-101 in male and female Cynomolgus monkeys, there were no apparent test article related effects observed in the Nerve Conduction assessment of the tibial nerve when comparing dose groups or comparing pre-dose recordings to subsequent Week 6 and/or Week 12 recordings.

5.0 CONCLUSIONS

There were no adverse effects observed on the recorded nerve conduction parameters in male or female Cynomolgus monkeys when administered scAAV9.U6miR871 (ARM-101) via an intrathecal slow infusion.

Attachment 1. CMAP and Nerve Conduction Data Tables

Dose Group	Animal ID	Timepoint	Left Ankle Latency (ms)	Left Ankle Amplitude (mV)	Left Ankle Duration (ms)	Left Knee Latency (ms)	Left Knee Amplitude (mV)	Left Knee Duration (ms)	Left Ankle-Left Knee Distance (cm)	Left Ankle-Left Knee Velocity (m/s)
1	**101**	Prestudy	2.08	8.9	5.3	3.17	8.4	5.8	9.0	83
	**102**	Prestudy	2.46	6.0	5.1	3.31	6.4	5.2	7.5	88
	**151**	Prestudy	2.19	10.7	2.7	4.08	9.6	3.0	11.0	58
	**152**	Prestudy	2.15	13.6	5.2	3.35	15.0	5.5	10.0	83
**1 Average**			**2.22**	**9.8**	**4.6**	**3.48**	**9.9**	**4.9**	**9.4**	**78**
**1 StdDev**			**0.17**	**3.2**	**1.3**	**0.41**	**3.7**	**1.3**	**1.5**	**14**
2	**201**	Prestudy	2.46	3.5	6.3	4.10	2.3	6.3	9.5	58
	**202**	Prestudy	2.85	3.2	5.3	4.15	2.4	5.5	9.5	74
	**203**	Prestudy	2.40	6.3	6.1	4.00	5.0	2.0	8.5	53
	**204**	Prestudy	1.94	8.2	3.5	2.94	7.9	3.6	8.5	85
	**251**	Prestudy	2.00	10.6	2.4	3.60	7.8	2.4	10.0	62
	**252**	Prestudy	2.60	8.1	6.6	3.98	6.6	6.7	8.5	62
	**253**	Prestudy	2.04	7.9	5.4	3.23	10.6	6.0	9.5	80
	**254**	Prestudy	2.08	15.6	5.4	3.52	16.7	3.0	9.0	63
**2 Average**			**2.30**	**7.9**	**5.1**	**3.69**	**7.4**	**4.4**	**9.1**	**67**
**2 StdDev**			**0.33**	**4.0**	**1.5**	**0.44**	**4.7**	**1.9**	**0.6**	**11**
3	**301**	Prestudy	2.46	15.8	5.4	3.85	15.6	5.5	8.5	61
	**302**	Prestudy	2.02	6.1	4.7	3.27	8.5	5.4	8.0	64
	**303**	Prestudy	2.31	11.2	5.9	3.71	10.9	6.0	9.0	64
	**304**	Prestudy	2.25	8.0	2.5	3.81	8.6	5.3	9.0	58
	**351**	Prestudy	2.44	18.3	5.4	3.60	17.4	5.5	9.5	81
	**352**	Prestudy	1.75	11.7	5.4	3.65	11.9	5.7	10.5	55
	**353**	Prestudy	2.79	22.7	5.3	4.35	16.6	2.4	8.5	54
	**354**	Prestudy	2.92	5.9	6.4	4.81	5.3	6.3	9.0	47
**3 Average**			**2.37**	**12.5**	**5.1**	**3.88**	**11.9**	**5.3**	**9.0**	**61**
**3 StdDev**			**0.38**	**6.0**	**1.2**	**0.48**	**4.4**	**1.2**	**0.8**	**10**

Dose Group	Animal ID	Timepoint	Right Ankle Latency (ms)	Right Ankle Amplitude (mV)	Right Ankle Duration (ms)	Right Knee Latency (ms)	Right Knee Amplitude (mV)	Right Knee Duration (ms)	Right Ankle-Right Knee Distance (cm)	Right Ankle-Right Knee Velocity (m/s)
1	**101**	Prestudy	2.60	7.3	2.0	3.52	6.2	1.9	6.0	65
	**102**	Prestudy	2.15	9.6	5.2	3.69	8.7	5.6	10.0	65
	**151**	Prestudy	2.44	11.2	2.4	4.15	9.8	2.7	10.5	61
	**152**	Prestudy	1.77	9.9	5.9	3.21	15.8	4.5	9.5	66
**1 Average**			**2.24**	**9.5**	**3.9**	**3.64**	**10.1**	**3.7**	**9.0**	**64**
**1 StdDev**			**0.36**	**1.6**	**2.0**	**0.39**	**4.1**	**1.7**	**2.0**	**2**
2	**201**	Prestudy	1.75	9.0	5.8	3.46	9.0	5.8	10.0	59
	**202**	Prestudy	2.29	4.6	6.2	3.83	4.3	6.6	9.5	62
	**203**	Prestudy	2.00	14.5	2.5	3.25	12.5	2.8	8.5	68
	**204**	Prestudy	1.69	7.1	1.8	2.52	9.2	2.5	8.0	96
	**251**	Prestudy	2.00	12.9	5.4	3.40	11.3	5.2	10.0	72
	**252**	Prestudy	1.96	12.9	6.5	3.33	16.5	6.4	9.0	65
	**253**	Prestudy	2.33	7.8	5.8	3.75	6.2	5.6	10.0	71
	**254**	Prestudy	2.23	11.1	2.5	3.50	7.1	4.9	9.5	75
**2 Average**			**2.03**	**10.0**	**4.6**	**3.38**	**9.5**	**5.0**	**9.3**	**71**
**2 StdDev**			**0.24**	**3.4**	**1.9**	**0.40**	**3.9**	**1.5**	**0.8**	**11**
3	**301**	Prestudy	1.88	11.1	3.7	3.25	10.7	4.1	8.5	62
	**302**	Prestudy	1.60	6.4	3.6	3.08	7.9	3.5	8.5	57
	**303**	Prestudy	2.48	10.7	7.3	3.90	12.0	8.1	9.0	64
	**304**	Prestudy	2.48	9.7	3.9	3.69	9.2	4.3	8.0	66
	**351**	Prestudy	1.96	9.9	5.6	3.44	9.5	5.4	9.0	61
	**352**	Prestudy	2.54	17.9	4.9	3.88	18.5	5.1	8.0	60
	**353**	Prestudy	2.38	13.6	7.0	3.96	9.4	7.2	9.0	57
	**354**	Prestudy	2.85	7.0	6.7	4.60	9.7	6.4	9.0	51
**3 Average**			**2.27**	**10.8**	**5.3**	**3.73**	**10.9**	**5.5**	**8.6**	**60**
**3 StdDev**			**0.42**	**3.7**	**1.5**	**0.48**	**3.3**	**1.6**	**0.4**	**5**

Dose Group	Animal ID	Timepoint	Left Ankle Latency (ms)	Left Ankle Amplitude (mV)	Left Ankle Duration (ms)	Left Knee Latency (ms)	Left Knee Amplitude (mV)	Left Knee Duration (ms)	Left Ankle-Left Knee Distance (cm)	Left Ankle-Left Knee Velocity (m/s)
**1**	**101**	Week 6	2.73	10.4	2.0	3.73	11.2	2.1	8.0	80
	**102**	Week 6	2.54	7.7	1.6	4.27	6.0	1.7	10.5	61
	**151**	Week 6	2.73	4.3	5.4	3.94	7.2	5.5	9.5	79
	**152**	Week 6	2.08	5.9	1.6	3.38	7.1	1.8	8.5	66
**1 Average**			**2.52**	**7.1**	**2.7**	**3.83**	**7.9**	**2.8**	**9.1**	**71.5**
**1 StdDev**			**0.31**	**2.6**	**1.8**	**0.37**	**2.3**	**1.8**	**1.1**	**9.5**
**2**	**201**	Week 6	2.40	3.5	6.0	5.42	4.2	5.2	10.0	33
	**202**	Week 6	2.31	7.9	6.3	4.04	7.7	6.7	10.5	61
	**203**	Week 6	1.90	8.8	4.6	3.23	8.3	5.2	10.0	75
	**204**	Week 6	1.98	6.2	4.0	3.29	5.3	3.8	10.5	80
	**251**	Week 6	2.00	9.1	5.9	3.75	9.3	6.0	10.5	60
	**252**	Week 6	2.58	21.2	6.2	4.40	18.8	6.4	11.0	61
	**253**	Week 6	1.63	6.6	6.4	2.83	4.6	6.9	11.0	91
	**254**	Week 6	1.83	19.4	5.2	3.38	19.7	5.1	9.5	62
**2 Average**			**2.08**	**10.3**	**5.6**	**3.79**	**9.7**	**5.7**	**10.4**	**65.4**
**2 StdDev**			**0.32**	**6.4**	**0.9**	**0.82**	**6.1**	**1.0**	**0.5**	**17.3**
**3**	**301**	Week 6	2.00	12.3	4.7	3.77	12.4	5.0	9.5	54
	**302**	Week 6	1.75	10.8	5.2	3.54	10.0	5.2	10.0	56
	**303**	Week 6	2.06	13.0	6.6	4.00	11.0	6.7	13.0	67
	**304**	Week 6	2.63	10.0	5.2	4.08	10.1	5.1	9.0	62
	**351**	Week 6	2.38	19.1	8.4	4.27	16.2	8.8	11.0	58
	**352**	Week 6	2.54	18.4	6.2	4.67	17.6	6.8	11.5	54
	**353**	Week 6	2.04	13.5	4.5	3.40	13.3	5.0	10.0	74
	**354**	Week 6	2.00	13.0	6.5	3.71	10.6	7.0	10.0	59
**3 Average**			**2.18**	**13.8**	**5.9**	**3.93**	**12.7**	**6.2**	**10.5**	**60.5**
**3 StdDev**			**0.31**	**3.3**	**1.3**	**0.41**	**2.9**	**1.4**	**1.3**	**7.0**

Dose Group	Animal ID	Timepoint	Right Ankle Latency (ms)	Right Ankle Amplitude (mV)	Right Ankle Duration (ms)	Right Knee Latency (ms)	Right Knee Amplitude (mV)	Right Knee Duration (ms)	Right Ankle-Right Knee Distance (cm)	Right Ankle-Right Knee Velocity (m/s)
**1**	**101**	Week 6	2.56	9.3	2.2	3.81	6.6	2.2	9.0	72
	**102**	Week 6	2.25	5.7	1.7	3.56	5.1	1.9	9.0	69
	**151**	Week 6	2.27	9.2	2.3	3.42	6.4	2.9	9.5	83
	**152**	Week 6	1.65	3.8	4.0	2.90	5.4	4.2	8.5	68
**1 Average**			**2.18**	**7.0**	**2.6**	**3.42**	**5.9**	**2.8**	**9.0**	**73.0**
**1 StdDev**			**0.38**	**2.7**	**1.0**	**0.38**	**0.7**	**1.0**	**0.4**	**6.9**
**2**	**201**	Week 6	2.19	6.9	6.6	4.33	7.2	6.5	10.0	47
	**202**	Week 6	2.27	6.8	6.5	3.96	6.1	6.8	11.5	68
	**203**	Week 6	2.13	8.1	2.3	3.52	8.5	3.0	11.0	79
	**204**	Week 6	1.63	8.9	4.8	2.98	8.0	4.7	9.5	70
	**251**	Week 6	2.02	11.9	5.4	3.42	9.8	5.6	9.5	68
	**252**	Week 6	2.27	11.6	2.9	3.69	11.8	3.0	11.0	78
	**253**	Week 6	2.33	13.6	5.9	3.38	10.8	6.3	9.0	86
	**254**	Week 6	2.13	14.2	5.5	3.67	13.9	5.8	10.0	65
**2 Average**			**2.12**	**10.3**	**5.0**	**3.62**	**9.5**	**5.2**	**10.2**	**70.1**
**2 StdDev**			**0.22**	**3.0**	**1.6**	**0.40**	**2.6**	**1.5**	**0.9**	**11.7**
**3**	**301**	Week 6	2.00	12.1	5.0	3.35	11.2	4.8	10.5	78
	**302**	Week 6	2.40	11.0	4.1	4.46	9.5	4.3	12.0	58
	**303**	Week 6	2.75	9.3	6.4	4.60	10.0	6.5	11.5	62
	**304**	Week 6	2.15	13.0	6.1	3.56	11.1	6.8	10.5	74
	**351**	Week 6	1.94	12.5	8.5	3.69	11.8	8.4	10.0	57
	**352**	Week 6	2.00	24.7	5.7	3.69	17.7	6.3	11.0	65
	**353**	Week 6	2.23	14.7	5.0	3.21	14.6	5.5	9.0	92
	**354**	Week 6	3.48	20.1	4.7	5.02	18.1	4.4	9.0	58
**3 Average**			**2.37**	**14.7**	**5.7**	**3.95**	**13.0**	**5.9**	**10.4**	**68.0**
**3 StdDev**			**0.52**	**5.2**	**1.4**	**0.66**	**3.4**	**1.4**	**1.1**	**12.4**

Dose Group	Animal ID	Timepoint	Left Ankle Latency (ms)	Left Ankle Amplitude (mV)	Left Ankle Duration (ms)	Left Knee Latency (ms)	Left Knee Amplitude (mV)	Left Knee Duration (ms)	Left Ankle-Left Knee Distance (cm)	Left Ankle-Left Knee Velocity (m/s)
**1**	**102**	Week 12	2.50	21.9	3.8	4.25	20.8	3.9	11.0	63
	**152**	Week 12	2.38	22.8	5.6	3.88	21.7	6.3	10.5	70
**1 Average**			**2.44**	**22.4**	**4.7**	**4.07**	**21.3**	**5.1**	**10.8**	**66.5**
**1 StdDev**			**0.08**	**0.6**	**1.3**	**0.26**	**0.6**	**1.7**	**0.4**	**4.9**
**2**	**203**	Week 12	2.63	15.6	3.3	3.92	11.1	8.0	10.5	81
	**204**	Week 12	2.00	10.1	3.9	3.15	10.1	4.8	9.5	83
	**253**	Week 12	2.00	11.5	6.0	3.60	10.4	6.2	10.5	65
	**254**	Week 12	2.27	20.9	6.5	4.02	21.3	6.8	10.0	57
**2 Average**			**2.23**	**14.5**	**4.9**	**3.67**	**13.2**	**6.5**	**10.1**	**71.5**
**2 StdDev**			**0.30**	**4.8**	**1.6**	**0.39**	**5.4**	**1.3**	**0.5**	**12.6**
**3**	**303**	Week 12	2.23	8.9	2.8	3.92	8.8	2.8	11.0	65
	**304**	Week 12	2.00	9.0	4.2	3.23	7.9	4.9	10.5	85
	**353**	Week 12	2.44	14.3	4.5	3.75	13.1	4.8	10.0	76
	**354**	Week 12	3.25	13.2	5.4	5.44	10.3	5.8	11.0	50
**3 Average**			**2.48**	**11.4**	**4.2**	**4.09**	**10.0**	**4.6**	**10.6**	**69.0**
**3 StdDev**			**0.54**	**2.8**	**1.1**	**0.95**	**2.3**	**1.3**	**0.5**	**15.1**

Dose Group	Animal ID	Timepoint	Right Ankle Latency (ms)	Right Ankle Amplitude (mV)	Right Ankle Duration (ms)	Right Knee Latency (ms)	Right Knee Amplitude (mV)	Right Knee Duration (ms)	Right Ankle-Right Knee Distance (cm)	Right Ankle-Right Knee Velocity (m/s)
**1**	**102**	Week 12	1.73	9.5	6.3	3.44	10.8	6.4	10.5	61
	**152**	Week 12	2.06	24.3	4.9	3.63	21.6	5.0	10.5	67
**1 Average**			**1.90**	**16.9**	**5.6**	**3.54**	**16.2**	**5.7**	**10.5**	**64.0**
**1 StdDev**			**0.23**	**10.5**	**1.0**	**0.13**	**7.6**	**1.0**	**0.0**	**4.2**
**2**	**203**	Week 12	2.88	13.2	6.1	4.67	11.9	6.3	12.0	67
	**204**	Week 12	1.52	10.6	4.2	2.69	10.0	4.1	8.5	73
	**253**	Week 12	1.77	8.9	5.5	3.31	8.4	5.6	10.5	68
	**254**	Week 12	2.15	22.3	5.8	4.17	22.5	5.6	11.0	54
**2 Average**			**2.08**	**13.8**	**5.4**	**3.71**	**13.2**	**5.4**	**10.5**	**65.5**
**2 StdDev**			**0.59**	**6.0**	**0.8**	**0.88**	**6.4**	**0.9**	**1.5**	**8.1**
**3**	**303**	Week 12	2.46	10.5	6.6	4.04	10.2	7.0	10.5	66
	**304**	Week 12	2.00	9.0	4.7	3.92	7.4	5.5	11.5	60
	**353**	Week 12	1.75	9.3	6.0	3.50	9.4	6.3	10.5	60
	**354**	Week 12	2.81	15.0	5.4	4.10	14.5	5.8	8.0	62
**3 Average**			**2.26**	**11.0**	**5.7**	**3.89**	**10.4**	**6.2**	**10.1**	**62.0**
**3 StdDev**			**0.47**	**2.8**	**0.8**	**0.27**	**3.0**	**0.7**	**1.5**	**2.8**

Attachment 2. Study Specific Method

Study Specific Method (SSM) for the Nerve Conduction Velocity Testing in Cynomolgus Monkeys

AmplifyBio Study Number: A-579-01

AmplifyBio
1425 State Route 142 (Plain City-Georgesville Road)
West Jefferson, OH 43162

1 PURPOSE

The purpose of this Study Specific Method (SSM) is to describe the procedures to perform Nerve Conduction Velocity (NCV) testing in cynomolgus monkeys. The tibial nerve in each hind leg will be evaluated with signal traces recorded from the abductor hallucis muscle. The Viking Software by Natus Medical Incorporated will be used for evaluation (SOP-00463).

2 ROLES AND RESPONSIBILITIES

Roles and responsibilities are recorded on the individual reports generated during data collection.

3 PREPARATION AND PROCEDURES

3.1 Equipment

- Viking software
- Amplifier AT2+6
- Viking EDX control panel
- Surface electrodes for active and reference recording
- Needle electrode for ground
- SignaGel or similar

3.2 NCV Preparation

- The monkey will be sedated as directed by a staff veterinarian.
- If necessary, the fur on the hind leg will be clipped.
- The reference electrode will be placed slightly distal to the 1st metatarsophalangeal joint, on the medial surface of the joint.
- The active recording electrode will be placed over the medial foot, slightly anterior to the navicular tubercule at the most elevated point of the arch formed by the junction of plantar skin and dorsal foot skin.
- The ground electrode will be placed over the dorsum of the foot.

3.3 NCV Procedure

1. An assistant will apply the stimulation probe at the ankle with cathode side toward the electrodes.
2. The computer operator will turn on the probe at a low stimulation value (~10 mV).
3. The assistant will move the probe until the tibial nerve is located and a characteristic response is received by the software.

4. Once the tibial nerve is located, the computer operator will increase the stimulation until a maximum response is achieved.
5. The computer operator will average 5 waveforms and then stop the stimulation. A mark will be made on the animal skin at the cathode placement.
6. The stimulation will be turned on and the probe operator will follow the tibial nerve proximally up the leg to the knee.
7. Repeat steps 4 and 5 for the second stimulation point around the knee.
8. The distance between the two marks will be measured and entered into the software to report the NCV.
9. Reports from the Viking software will be generated and included in the study file.

4 REVISIONS TO SSM

Revisions or changes to this SSM will be managed within Veeva QualityDocs and identified with a new version number. Any changes or corrections made to the procedure during execution will be approved by the Study Director before the change is executed and the approval will be documented on the SSM. Any minor corrections to the method which do not affect data analysis may be corrected directly on the method without a formal version change

Document Approvals

Number: METHOD-00082 Version: 1.0

Approval Task<br>Verdict: Approve	Sapna Varia,<br>(svaria@amplify-bio.com)<br>Approval<br>08-Jun-2023 02:29:37 GMT+0000

Appendix H Electrocardiograph Evaluation Contributing Scientist Report

A 12-Week Single Dose Biodistribution and Safety Study in Cynomolgus Monkeys

Electrocardiograph Evaluation Contributing Scientist Report

May 30, 2024

Study Number A-579-01

Testing Facility
AmplifyBio
1425 State Route 142 (Plain City-Georgesville Road)
West Jefferson, OH 43162

SIGNATURES

Prepared by:

DocuSigned by:

Michael Stonerook

Signer Name: Michael Stonerook
Signing Reason: I am the author of this document
Signing Time: 5/30/2024 | 9:46:35 AM EDT
63C2D48DB6B3417288EEA3C794CB2F31

5/30/2024

Michael J. Stonerook, PhD, DVM, DABT<br>Study Physiologist	Date

TABLE OF CONTENTS

Page

Signatures **2**

1.0 Introduction **4**

2.0 Study Design and Methods **4**

2.1 Data Capture and Analysis 4

3.0 Deviations **5**

4.0 Results **5**

5.0 Conclusions **5**

1.0 INTRODUCTION

This contributing scientist report summarizes the electrocardiograph (ECG) data collection and analysis for Study Number A-579-01 and any findings that may have been associated with it.

2.0 STUDY DESIGN AND METHODS

The study design relating to ECG evaluations is outlined in Table 1.

Table 1. Experimental Design

Group	Target Dose (Total vg)	Target Dose Volume (mL)	Animal Identification Numbers			
			6-Week Cohort[a]		12-Week Cohort[b]	
			Male	Female	Male	Female
1 – Control	0	3	101	151	102	152
2 – Low	6×10^{13}	3	201-202	251-252	203-204	253-254
3 – High	1.2×10^{14}	3	301-302	351-352	303-304	353-354

vg=vector genome.
a. Necropsy Day 43.
b. Necropsy Day 85.

All groups were dosed on Day 1 via a single intrathecal slow infusion over approximately 6 hours via a surgically implanted lumbar catheter (see QE-001542 for exceptions).

2.1 DATA CAPTURE AND ANALYSIS

Emka Software was used to collect (emka IOX Ver. 2.10) and analyze (emka ecgAUTO Ver. 3.5) the ECG data.

ECG waveforms were collected at the following time points:

- Pretest: all animals prior to dosing
- Week 6: all animals
- Week 12: all surviving animals

Prior to each ECG data collection, animals were sedated with ketamine (10 to 16 mg/kg, intramuscular [IM]). Once properly sedated, ECG waveform tracings were collected for at least one minute using surface leads emulating a standard Lead II configuration. A board-certified veterinarian qualitatively assessed the ECG waveform strips for evidence of cardiac dysfunction by examining rate, rhythm, and morphology alterations.

3.0 DEVIATIONS

QE-001542: On Day 1, the dose administration was not exactly 6 hours for all three groups.

For Group 1, Animal 101 had dose administered starting at 0957. There is documentation that the pump was paused at 1414 due to the Huber needle detaching from the port, however, there is no documentation of when the pump was restarted following this pause. It is unclear how long the dose was administered with the pump stop time documented at 1632. For the other Group 1 animals, the dose administration time was as followed: Animal 102 (5hrs 58mins), Animal 151 (5hrs 56mins), and Animal 152 (5hrs 3mins).

For Group 2, the dose administration time for each animal is as followed: Animal 201 (5hrs 56mins), Animal 202 (5hrs 56mins), Animal 203 (5hrs 57mins), Animal 204 (5hrs 55mins), Animal 251 (5hrs 56mins), Animal 252 (5hrs 54mins), Animal 253 (5hrs 56mins), and Animal 254 (5hrs 55mins).

For Group 3, the dose administration time for each animal is as followed: Animal 301 (5hrs 55mins), Animal 302 (5hrs 55mins), Animal 303 (5hrs 51mins), Animal 304 (6hrs 2mins), Animal 351 (5hrs 56mins), Animal 352 (5hrs 56mins), Animal 353 (5hrs 57mins), and Animal 354 (5hrs 58mins).

The impact of +/- 10 minutes on a 6 hour treatment is not likely to have an impact. The failed attempt to give full treatment to Animal 152 will have no effect since this animal was a control and not receiving test article.

4.0 RESULTS

There were no abnormal ECG findings observed prior to the initiation of dosing that precluded the placement of any animal on study.

Following the administration of ARM-101 in male and female Cynomolgus monkeys, there were no apparent test article related effects observed in the qualitative assessment of ECG waveforms when comparing dose groups or comparing pre-dose recordings to subsequent Week 6 and/or Week 12 recordings. A single premature ventricular complex was observed in animal 301 during Week 6 but this abnormality is not considered dose related as it is considered a background arrythmia commonly observed in Cynomolgus monkeys.

5.0 CONCLUSIONS

There were no adverse effects observed on the recorded ECGs in male or female Cynomolgus monkeys when administered scAAV9.U6miR871 (ARM-101) via an intrathecal slow infusion.

Appendix I Clinical Pathology Contributing Scientist Report

A 12-Week Single Dose Biodistribution and Safety Study in Cynomolgus Monkeys

Clinical Pathology Contributing Scientist Report

June 3, 2024

Study Number A-579-01

Testing Facility
AmplifyBio
1425 State Route 142 (Plain City-Georgesville Road)
West Jefferson, OH 43162

SIGNATURES

Prepared by:

DocuSigned by:

Eric Snook

Signer Name: Eric Snook
Signing Reason: I am the author of this document
Signing Time: 6/3/2024 | 9:04:04 AM EDT
A96DB9479D624626BC1D94753A930CB6

6/3/2024

Eric Snook, DVM, PhD, DACVP<br>Study Clinical Pathologist	Date

TABLE OF CONTENTS

		Page
**Signatures**		**2**
**1.0**	**Introduction**	**4**
**2.0**	**Study Design and Methods**	**4**
	2.1 Hematology	5
	2.2 Coagulation	5
	2.3 Serum Chemistry	5
	2.4 Urinalysis	6
	2.5 Troponin I	6
**3.0**	**Data Capture and Analysis**	**6**
**4.0**	**Deviations**	**6**
**5.0**	**Results**	**7**
	5.1 Hematology	7
	5.2 Coagulation	7
	5.3 Serum Chemistry	8
	5.4 Urinalysis	8
	5.5 Troponin I	8
**6.0**	**Conclusions**	**8**

LIST OF ATTACHMENTS

Attachment 1.	Individual Animal Hematology Data and Test Methods/Definitions	9
Attachment 2.	Individual Animal Coagulation Data and Test Methods/Definitions	109
Attachment 3.	Individual Animal Serum Chemistry Data and Test Methods/Definitions	125
Attachment 4.	Individual Animal Urinalysis and Urine Sediment Data and Test Methods/Definitions	246
Attachment 5.	Troponin I Analysis Method Report and Results	360

1.0 INTRODUCTION

This contributing scientist report summarizes the clinical pathology findings during Study No. A-579-01. The purpose of this study was to evaluate the potential biodistribution and safety of scAAV9.U6miR871 (ARM-101) when administered once via intrathecal infusion to Cynomolgus monkeys for up to 12 weeks.

2.0 STUDY DESIGN AND METHODS

The experimental design relating to clinical pathology evaluations is outlined in Table 1.

Table 1. Experimental Design

Group	Target Dose (Total vg)[a]	Target Dose Volume (mL)	Animal Identification Numbers			
			6-Week Cohort[b]		12-Week Cohort[c]	
			Male	Female	Male	Female
1 – Control	0	3	101	151	102	152
2 – Low	6×10^{13}	3	201-202	251-252	203-204	253-254
3 – High	1.2×10^{14}	3	301-302	351-352	303-304	353-354

vg=vector genome.
a. See QE-001542 for dosing deviation.
b. Necropsy Day 43.
c. Necropsy Day 85.

Specimens for clinical pathology analysis were collected and analyzed as outlined in Table 2.

Table 2. Summary of Clinical Pathology Evaluations

Evaluation	Weeks/Days of Sample Collection	Analysis System
Hematology	Pretest, Day 2, Day 8, Day 22, Day 43, Day 50[a], Day 85[a]	Advia 2120i Hematology Analyzer and manual methods
Coagulation	Pretest, Day 2, Day 8, Day 22, Day 43, Day 50[a], Day 85[a]	Stago Compact Max Coagulation Analyzer
Serum Chemistry	Pretest, Day 2, Day 8, Day 22, Day 43, Day 50[a], Day 85[a]	Roche Cobas c501 Chemistry Analyzer
Urinalysis[b]	Pretest, Day 2, Day 8, Day 22, Day 43[c], Day 50[a], Day 85[a]	Roche Cobas u411 Urine Analyzer and manual methods
Troponin I	Pretest, Day 2, Day 8, Day 22, Day 43, Day 50[a], Day 85[a]	See Attachment 5

a. For 12-week Cohort only.
b. The total urine output collected overnight was provided to the clinical pathology laboratory.
c. See DEV-002185 for Week 6 urine collections occurring on Day 43 (Week 7).

2.1 HEMATOLOGY

The parameters listed in Table 3 were measured/evaluated for hematology:

Table 3. Hematology Parameters

Cell morphology[a,b]	Mean corpuscular volume
Erythrocyte count	Mean corpuscular hemoglobin
Hemoglobin	Mean corpuscular hemoglobin concentration
Hematocrit	Mean platelet volume[b]
Leukocyte count, total	Platelet count
Leukocyte differential (absolute and relative)	Reticulocyte count (absolute and relative)[b]

a. Slides were prepared from un-clotted samples.
b. See DEV-002204 for exceptions.

The methods used for hematology are outlined in Attachment 1.

2.2 COAGULATION

The parameters listed in Table 4 were measured for coagulation:

Table 4. Coagulation Parameters

Activated partial thromboplastin time	Prothrombin time
Fibrinogen	

The methods used for coagulation analysis are outlined in Attachment 2.

2.3 SERUM CHEMISTRY

The parameters listed in Table 5 were measured for serum chemistry:

Table 5. Serum Chemistry Parameters

Alanine aminotransferase	Creatine kinase
Albumin	Creatinine
Albumin/globulin ratio	Gamma glutamyltransferase
Alkaline phosphatase	Globulin
Aspartate aminotransferase	Glucose
Bilirubin, direct	Lactate dehydrogenase
Bilirubin, total	Phosphorus
Blood urea nitrogen	Potassium
Calcium	Sodium
Cholesterol	Total protein
Chloride	Triglycerides

The methods used for serum chemistry are outlined in Attachment 3.

2.4 URINALYSIS

The parameters listed in Table 6 were measured/evaluated for urinalysis:

Table 6. Urinalysis Parameters

Appearance (clarity and color)	pH[a]
Bilirubin[a]	Protein[a]
Glucose[a]	Specific gravity[b]
Ketones[a]	Total volume
Leukocytes[a]	Urobilinogen[a]
Nitrite[a]	Microscopic examination of sediment[c]
Occult blood[a]	

a. Qualitative results were obtained using reagent strips (urine dip parameters).
b. Results were obtained using a SPER Scientific Digital Refractometer.
c. Sediment was evaluated for white blood cells, red blood cells, casts, epithelial cells, mucus, sperm, bacteria, yeast, amorphous sediment, and crystals (without machine/by hand).

The methods used for urinalysis are outlined in Attachment 4.

2.5 TROPONIN I

Troponin I was analyzed at the test site (Antech Diagnostics GLP) and a method's report was provided. The method's report is outlined in Attachment 5.

3.0 DATA CAPTURE AND ANALYSIS

Provantis (Instem) was used as the electronic data capture and data analysis system for data collected at the testing facility. The individual animal data, excluding Troponin I, were reported using Provantis. Data captured at the test site for Troponin I are reported in Attachment 5.

4.0 DEVIATIONS

DEV-002185: Urine was collected on Day 43, occurring one day after the protocol required timepoint of Week 6. There was no study impact due to the urine collection on Day 43 one day after Week 6. The urine collection was performed as intended, hence there was no impact to the integrity of the data.

QE-001542: On Day 1, the dose administration was not exactly 6 hours for all three groups.

For Group 1, Animal 101 had dose administered starting at 0957. There is documentation that the pump was paused at 1414 due to the Huber needle detaching from the port, however, there is no documentation of when the pump was restarted following this pause. It is unclear how long the dose was administered with the pump stop time documented at 1632. For the other

Group 1 animals, the dose administration time was as followed: Animal 102 (5hrs 58mins), Animal 151 (5hrs 56mins), and Animal 152 (5hrs 3mins).

For Group 2, the dose administration time for each animal is as followed: Animal 201 (5hrs 56mins), Animal 202 (5hrs 56mins), Animal 203 (5hrs 57mins), Animal 204 (5hrs 55mins), Animal 251 (5hrs 56mins), Animal 252 (5hrs 54mins), Animal 253 (5hrs 56mins), and Animal 254 (5hrs 55mins).

For Group 3, the dose administration time for each animal is as followed: Animal 301 (5hrs 55mins), Animal 302 (5hrs 55mins), Animal 303 (5hrs 51mins), Animal 304 (6hrs 2mins), Animal 351 (5hrs 56mins), Animal 352 (5hrs 56mins), Animal 353 (5hrs 57mins), and Animal 354 (5hrs 58mins).

The impact of +/- 10 minutes on a 6 hour treatment is not likely to have an impact. The failed attempt to give full treatment to Animal 152 will have no effect since this animal was a control and not receiving test article.

DEV-002204: According to Protocol Section 11.10.1, Hematology Table 4, reticulocyte count (absolute and relative) and Mean platelet volume will be evaluated, and slides will be prepared from un-clotted samples for potential morphological assessment. For the pretest hematology collection, relative reticulocyte count (% reticulocytes) and Mean Platelet Volume were not evaluated. In addition, slides were only prepared for Animals 152, 253, 352, and 353.

There is no impact to the study, slide preparation for the animals listed in the deviation was not needed as there were no abnormal findings to confirm manually.

5.0 RESULTS

Statistical analyses for the results were not appropriate due to small sample sizes in the Group 1 (control) groups.

5.1 HEMATOLOGY

Individual Data: Attachment 1

There were no test article-related findings noted in the hematology data.

5.2 COAGULATION

Individual Data: Attachment 2

There were no test article-related findings noted in the coagulation data.

5.3 SERUM CHEMISTRY

Individual Data: Attachment 3

There are no test article-related findings in the chemistry data.

5.4 URINALYSIS

Individual Data: Attachment 4

There were no test article-related findings noted in the urine data.

5.5 TROPONIN I

Individual Data: Attachment 5

There were no test-article related findings in the troponin I data.

6.0 CONCLUSIONS

There are no test article-related findings in this study. Each group demonstrated at least mild fluctuations of values over the course of the study; however, there were no identifiable trends associated with the dose in any group.

Attachment 1. Individual Animal Hematology Data and Test Methods/Definitions

Hematology Test Methods/Definitions

Test Parameter	Test Method	Units
Absolute Reticulocytes	Calculation	^10^3 cells/µL
Hematocrit	Calculation	%
Hemoglobin	Cyanmethemoglobin	g/dL
Mean Corpuscular Hemoglobin	Calculation	pg
Mean Corpuscular Hemoglobin Concentration	Calculation	g/dL
Mean Corpuscular Volume	Flow Cytometry	fL
Mean Platelet Volume	Flow Cytometry	fL
Platelet Count	Flow Cytometry	^10^3 cells/µL
Relative Reticulocytes	Flow Cytometry	%
Red Blood Cells	Flow Cytometry	^10^6 cells/µL
Slide (Blood Smear) Review	Manual	N/A
White Blood Cells; Total Leukocyte Count	Flow Cytometry	^10^3 cells/µL
White Blood Cell Differential Parameters:		
Absolute Neutrophils	Calculation	^10^3 cells/µL
Absolute Lymphocytes	Calculation	^10^3 cells/µL
Absolute Monocytes	Calculation	^10^3 cells/µL
Absolute Eosinophils	Calculation	^10^3 cells/µL
Absolute Basophils	Calculation	^10^3 cells/µL
Absolute Large Unstained Cells	Calculation	^10^3 cells/µL
Relative Neutrophils	Flow Cytometry	%
Relative Lymphocytes	Flow Cytometry	%
Relative Monocytes	Flow Cytometry	%
Relative Eosinophils	Flow Cytometry	%
Relative Basophils	Flow Cytometry	%
Relative Large Unstained Cells	Flow Cytometry	%

N/A = Not applicable
WNL = Within normal limits

AmplifyBio Study Number A-579-01

Individual Animal Hematology Data

Sex: Male Day(s) Relative to Start Date

Group 1 Control Total vg 0	Hematology										
	Red Blood Cells (x10^6 cells/µL)	Red Blood Cells (x10^6 cells/µL)	Red Blood Cells (x10^6 cells/µL)	Red Blood Cells (x10^6 cells/µL)	Red Blood Cells (x10^6 cells/µL)	Red Blood Cells (x10^6 cells/µL)	Red Blood Cells (x10^6 cells/µL)	Hemoglobin (g/dL)	Hemoglobin (g/dL)	Hemoglobin (g/dL)	Hemoglobin (g/dL)
	-41	2	8	22	43	50	85	-41	2	8	22
101	5.37	4.96	4.96	5.49	5.62	-	-	13.1	12.2	12.2	13.4
102	5.83	5.67	5.55	6.13	5.95	5.92	6.05	13.9	13.2	13.0	14.3
Mean	5.60	5.32	5.26	5.81	5.79	5.92	6.05	13.5	12.7	12.6	13.9
SD	0.33	0.50	0.42	0.45	0.23	-	-	0.6	0.7	0.6	0.6
Max	5.83	5.67	5.55	6.13	5.95	5.92	6.05	13.9	13.2	13.0	14.3
Min	5.37	4.96	4.96	5.49	5.62	5.92	6.05	13.1	12.2	12.2	13.4

1/10/2024 11:19:59AM Page: 2

AmplifyBio Study Number A-579-01

Individual Animal Hematology Data

Sex: Male Day(s) Relative to Start Date

Group 1 Control Total vg 0	Hematology										
	Hemoglobin (g/dL)	Hemoglobin (g/dL)	Hemoglobin (g/dL)	Hematocrit (%)	Hematocrit (%)	Hematocrit (%)	Hematocrit (%)	Hematocrit (%)	Hematocrit (%)	Hematocrit (%)	MCV (fL)
	43	50	85	-41	2	8	22	43	50	85	-41
101	13.5	-	-	41.8	39.9	38.5	44.5	44.2	-	-	77.9
102	14.1	13.8	14.2	44.6	43.4	41.1	47.0	45.5	44.9	45.6	76.6
Mean	13.8	13.8	14.2	43.2	41.7	39.8	45.8	44.9	44.9	45.6	77.3
SD	0.4	-	-	2.0	2.5	1.8	1.8	0.9	-	-	0.9
Max	14.1	13.8	14.2	44.6	43.4	41.1	47.0	45.5	44.9	45.6	77.9
Min	13.5	13.8	14.2	41.8	39.9	38.5	44.5	44.2	44.9	45.6	76.6

1/10/2024 11:19:59AM Page: 3

AmplifyBio Study Number A-579-01

Individual Animal Hematology Data

Sex: Male Day(s) Relative to Start Date

Group 1 Control Total vg 0	Hematology										
	MCV (fL)	MCV (fL)	MCV (fL)	MCV (fL)	MCV (fL)	MCV (fL)	MCH (pg)	MCH (pg)	MCH (pg)	MCH (pg)	MCH (pg)
	2	8	22	43	50	85	-41	2	8	22	43
101	80.5	77.7	80.9	78.6	-	-	24.4	24.6	24.7	24.3	24.0
102	76.5	74.0	76.8	76.4	75.9	75.3	23.8	23.3	23.5	23.3	23.7
Mean	78.5	75.9	78.9	77.5	75.9	75.3	24.1	24.0	24.1	23.8	23.9
SD	2.8	2.6	2.9	1.6	-	-	0.4	0.9	0.8	0.7	0.2
Max	80.5	77.7	80.9	78.6	75.9	75.3	24.4	24.6	24.7	24.3	24.0
Min	76.5	74.0	76.8	76.4	75.9	75.3	23.8	23.3	23.5	23.3	23.7

AmplifyBio Study Number A-579-01

Individual Animal Hematology Data

Sex: Male Day(s) Relative to Start Date

Group 1 Control Total vg 0	Hematology										
	MCH (pg)	MCH (pg)	MCHC (g/dL)	MCHC (g/dL)	MCHC (g/dL)	MCHC (g/dL)	MCHC (g/dL)	MCHC (g/dL)	MCHC (g/dL)	RETIC (x10^3 cells/µL)	RETIC (x10^3 cells/µL)
	50	85	-41	2	8	22	43	50	85	-41	2
101	-	-	31.4	30.6	31.8	30.1	30.5	-	-	44.5	97.7
102	23.4	23.5	31.1	30.5	31.7	30.4	31.0	30.8	31.2	61.8	87.7
Mean	23.4	23.5	31.3	30.6	31.8	30.3	30.8	30.8	31.2	53.2	92.7
SD	-	-	0.2	0.1	0.1	0.2	0.4	-	-	12.2	7.1
Max	23.4	23.5	31.4	30.6	31.8	30.4	31.0	30.8	31.2	61.8	97.7
Min	23.4	23.5	31.1	30.5	31.7	30.1	30.5	30.8	31.2	44.5	87.7

1/10/2024 11:19:59AM Page: 5

AmplifyBio Study Number A-579-01

Individual Animal Hematology Data

Sex: Male Day(s) Relative to Start Date

Group 1 Control Total vg 0	Hematology										
	RETIC (x10^3 cells/µL)	RETIC (x10^3 cells/µL)	RETIC (x10^3 cells/µL)	RETIC (x10^3 cells/µL)	RETIC (x10^3 cells/µL)	% Retic (%)	% Retic (%)	% Retic (%)	% Retic (%)	% Retic (%)	% Retic (%)
	8	22	43	50	85	2	8	22	43	50	85
101	92.1	76.8	47.0	-	-	1.97	1.86	1.40	0.84	-	-
102	78.1	99.0	72.1	67.9	43.2	1.55	1.41	1.62	1.21	1.15	0.71
Mean	85.1	87.9	59.6	67.9	43.2	1.76	1.64	1.51	1.03	1.15	0.71
SD	9.9	15.7	17.7	-	-	0.30	0.32	0.16	0.26	-	-
Max	92.1	99.0	72.1	67.9	43.2	1.97	1.86	1.62	1.21	1.15	0.71
Min	78.1	76.8	47.0	67.9	43.2	1.55	1.41	1.40	0.84	1.15	0.71

1/10/2024 11:19:59AM Page: 6

AmplifyBio Study Number A-579-01

Individual Animal Hematology Data

Sex: Male Day(s) Relative to Start Date

Group 1 Control Total vg 0	Hematology										
	White Blood Cells (x10^3 cells/µL)	White Blood Cells (x10^3 cells/µL)	White Blood Cells (x10^3 cells/µL)	White Blood Cells (x10^3 cells/µL)	White Blood Cells (x10^3 cells/µL)	White Blood Cells (x10^3 cells/µL)	White Blood Cells (x10^3 cells/µL)	Neutrophils (x10^3 cells/µL)	Neutrophils (x10^3 cells/µL)	Neutrophils (x10^3 cells/µL)	Neutrophils (x10^3 cells/µL)
	-41	2	8	22	43	50	85	-41	2	8	22
101	8.03	15.31 [a]	4.65	10.38	6.15	-	-	5.37	7.97	1.99	2.74
102	15.02	13.21	10.03	11.50	8.63	11.66	9.88	11.63	5.86	5.42	4.67
Mean	11.53	14.26	7.34	10.94	7.39	11.66	9.88	8.50	6.92	3.71	3.71
SD	4.94	1.48	3.80	0.79	1.75	-	-	4.43	1.49	2.43	1.36
Max	15.02	15.31	10.03	11.50	8.63	11.66	9.88	11.63	7.97	5.42	4.67
Min	8.03	13.21	4.65	10.38	6.15	11.66	9.88	5.37	5.86	1.99	2.74

[a] [RC:Morphology WNL]

1/10/2024 11:19:59AM Page: 7

AmplifyBio Study Number A-579-01

Individual Animal Hematology Data

Sex: Male Day(s) Relative to Start Date

Group 1 Control Total vg 0	Hematology										
	Neutrophils (x10^3 cells/µL)	Neutrophils (x10^3 cells/µL)	Neutrophils (x10^3 cells/µL)	Lymphocytes (x10^3 cells/µL)	Lymphocytes (x10^3 cells/µL)	Lymphocytes (x10^3 cells/µL)	Lymphocytes (x10^3 cells/µL)	Lymphocytes (x10^3 cells/µL)	Lymphocytes (x10^3 cells/µL)	Lymphocytes (x10^3 cells/µL)	Monocytes (x10^3 cells/µL)
	43	50	85	-41	2	8	22	43	50	85	-41
101	0.81	-	-	2.27	5.89	2.32	6.92	4.88	-	-	0.34
102	2.65	5.93	3.42	2.79	6.52	4.09	6.09	5.42	5.14	5.81	0.53
Mean	1.73	5.93	3.42	2.53	6.21	3.21	6.51	5.15	5.14	5.81	0.44
SD	1.30	-	-	0.37	0.45	1.25	0.59	0.38	-	-	0.13
Max	2.65	5.93	3.42	2.79	6.52	4.09	6.92	5.42	5.14	5.81	0.53
Min	0.81	5.93	3.42	2.27	5.89	2.32	6.09	4.88	5.14	5.81	0.34

AmplifyBio Study Number A-579-01

Individual Animal Hematology Data

Sex: Male Day(s) Relative to Start Date

Group 1 Control Total vg 0	Hematology										
	Monocytes (x10^3 cells/µL)	Monocytes (x10^3 cells/µL)	Monocytes (x10^3 cells/µL)	Monocytes (x10^3 cells/µL)	Monocytes (x10^3 cells/µL)	Monocytes (x10^3 cells/µL)	Eosinophils (x10^3 cells/µL)	Eosinophils (x10^3 cells/µL)	Eosinophils (x10^3 cells/µL)	Eosinophils (x10^3 cells/µL)	Eosinophils (x10^3 cells/µL)
	2	8	22	43	50	85	-41	2	8	22	43
101	1.22	0.29	0.58	0.36	-	-	0.01	0.08	0.01	0.04	0.02
102	0.69	0.45	0.61	0.46	0.52	0.50	0.01	0.02	0.01	0.06	0.01
Mean	0.96	0.37	0.60	0.41	0.52	0.50	0.01	0.05	0.01	0.05	0.02
SD	0.37	0.11	0.02	0.07	-	-	0.00	0.04	0.00	0.01	0.01
Max	1.22	0.45	0.61	0.46	0.52	0.50	0.01	0.08	0.01	0.06	0.02
Min	0.69	0.29	0.58	0.36	0.52	0.50	0.01	0.02	0.01	0.04	0.01

AmplifyBio Study Number A-579-01

Individual Animal Hematology Data

Sex: Male Day(s) Relative to Start Date

Group 1 Control Total vg 0	Hematology										
	Eosinophils (x10^3 cells/µL)	Eosinophils (x10^3 cells/µL)	Basophils (x10^3 cells/µL)	Basophils (x10^3 cells/µL)	Basophils (x10^3 cells/µL)	Basophils (x10^3 cells/µL)	Basophils (x10^3 cells/µL)	Basophils (x10^3 cells/µL)	Basophils (x10^3 cells/µL)	LUC (x10^3 cells/µL)	LUC (x10^3 cells/µL)
	50	85	-41	2	8	22	43	50	85	-41	2
101	-	-	0.01	0.03	0.01	0.04	0.02	-	-	0.03	0.12
102	0.02	0.09	0.02	0.03	0.01	0.03	0.03	0.01	0.02	0.04	0.08
Mean	0.02	0.09	0.02	0.03	0.01	0.04	0.03	0.01	0.02	0.04	0.10
SD	-	-	0.01	0.00	0.00	0.01	0.01	-	-	0.01	0.03
Max	0.02	0.09	0.02	0.03	0.01	0.04	0.03	0.01	0.02	0.04	0.12
Min	0.02	0.09	0.01	0.03	0.01	0.03	0.02	0.01	0.02	0.03	0.08

AmplifyBio Study Number A-579-01

Individual Animal Hematology Data

Sex: Male Day(s) Relative to Start Date

Group 1 Control Total vg 0	Hematology										
	LUC (x10^3 cells/µL)	LUC (x10^3 cells/µL)	LUC (x10^3 cells/µL)	LUC (x10^3 cells/µL)	LUC (x10^3 cells/µL)	% Neutrophils (%)	% Neutrophils (%)	% Neutrophils (%)	% Neutrophils (%)	% Neutrophils (%)	% Neutrophils (%)
	8	22	43	50	85	-41	2	8	22	43	50
101	0.03	0.07	0.06	-	-	66.8	52.1	42.8	26.4	13.2 [a]	-
102	0.04	0.05	0.06	0.05	0.04	77.5	44.4	54.1	40.6	30.7	50.8
Mean	0.04	0.06	0.06	0.05	0.04	72.2	48.3	48.5	33.5	22.0	50.8
SD	0.01	0.01	0.00	-	-	7.6	5.4	8.0	10.0	12.4	-
Max	0.04	0.07	0.06	0.05	0.04	77.5	52.1	54.1	40.6	30.7	50.8
Min	0.03	0.05	0.06	0.05	0.04	66.8	44.4	42.8	26.4	13.2	50.8

[a] [RC:Number decreased confirmed.]

AmplifyBio Study Number A-579-01

Individual Animal Hematology Data

Sex: Male Day(s) Relative to Start Date

Group 1 Control Total vg 0	Hematology										
	% Neutrophils (%)	% Lymphocytes (%)	% Lymphocytes (%)	% Lymphocytes (%)	% Lymphocytes (%)	% Lymphocytes (%)	% Lymphocytes (%)	% Lymphocytes (%)	% Monocytes (%)	% Monocytes (%)	% Monocytes (%)
	85	-41	2	8	22	43	50	85	-41	2	8
101	-	28.2	38.5	49.9	66.7	79.4	-	-	4.30	8.00	6.20
102	34.6	18.6	49.4	40.8	53.0	62.8	44.1	58.8	3.50	5.30	4.50
Mean	34.6	23.4	44.0	45.4	59.9	71.1	44.1	58.8	3.90	6.65	5.35
SD	-	6.8	7.7	6.4	9.7	11.7	-	-	0.57	1.91	1.20
Max	34.6	28.2	49.4	49.9	66.7	79.4	44.1	58.8	4.30	8.00	6.20
Min	34.6	18.6	38.5	40.8	53.0	62.8	44.1	58.8	3.50	5.30	4.50

AmplifyBio Study Number A-579-01

Individual Animal Hematology Data

Sex: Male Day(s) Relative to Start Date

Group 1 Control Total vg 0	Hematology										
	% Monocytes (%)	% Monocytes (%)	% Monocytes (%)	% Monocytes (%)	% Eosinophils (%)	% Eosinophils (%)	% Eosinophils (%)	% Eosinophils (%)	% Eosinophils (%)	% Eosinophils (%)	% Eosinophils (%)
	22	43	50	85	-41	2	8	22	43	50	85
101	5.60	5.80	-	-	0.1	0.5	0.2	0.4	0.4	-	-
102	5.30	5.40	4.40	5.00	0.1	0.2	0.1	0.5	0.2	0.2	1.0
Mean	5.45	5.60	4.40	5.00	0.1	0.4	0.2	0.5	0.3	0.2	1.0
SD	0.21	0.28	-	-	0.0	0.2	0.1	0.1	0.1	-	-
Max	5.60	5.80	4.40	5.00	0.1	0.5	0.2	0.5	0.4	0.2	1.0
Min	5.30	5.40	4.40	5.00	0.1	0.2	0.1	0.4	0.2	0.2	1.0

AmplifyBio Study Number A-579-01

Individual Animal Hematology Data

Sex: Male Day(s) Relative to Start Date

Group 1 Control Total vg 0	Hematology										
	% BASO (%)	% BASO (%)	% BASO (%)	% BASO (%)	% BASO (%)	% BASO (%)	% BASO (%)	% LUC (%)	% LUC (%)	% LUC (%)	% LUC (%)
	-41	2	8	22	43	50	85	-41	2	8	22
101	0.2	0.2	0.2	0.4	0.3	-	-	0.4	0.8	0.6	0.7
102	0.1	0.2	0.1	0.3	0.3	0.1	0.2	0.3	0.6	0.4	0.4
Mean	0.2	0.2	0.2	0.4	0.3	0.1	0.2	0.4	0.7	0.5	0.6
SD	0.1	0.0	0.1	0.1	0.0	-	-	0.1	0.1	0.1	0.2
Max	0.2	0.2	0.2	0.4	0.3	0.1	0.2	0.4	0.8	0.6	0.7
Min	0.1	0.2	0.1	0.3	0.3	0.1	0.2	0.3	0.6	0.4	0.4

AmplifyBio Study Number A-579-01

Individual Animal Hematology Data

Sex: Male Day(s) Relative to Start Date

Group 1 Control Total vg 0	Hematology										
	% LUC (%)	% LUC (%)	% LUC (%)	Platelets (x10^3 cells/µL)	Platelets (x10^3 cells/µL)	Platelets (x10^3 cells/µL)	Platelets (x10^3 cells/µL)	Platelets (x10^3 cells/µL)	Platelets (x10^3 cells/µL)	Platelets (x10^3 cells/µL)	MPV (fL)
	43	50	85	-41	2	8	22	43	50	85	2
101	0.9	-	-	482	541 [a]	547	531 [b]	520	-	-	10.4
102	0.7	0.4	0.4	456	407	390	421	437	445	384	9.7
Mean	0.8	0.4	0.4	469	474	469	476	479	445	384	10.1
SD	0.1	-	-	18	95	111	78	59	-	-	0.5
Max	0.9	0.4	0.4	482	541	547	531	520	445	384	10.4
Min	0.7	0.4	0.4	456	407	390	421	437	445	384	9.7

[a] [RC:Morphology WNL]
[b] [RC:Number increased confirmed.]

AmplifyBio Study Number A-579-01

Individual Animal Hematology Data

Sex: Male Day(s) Relative to Start Date

Group 1 Control Total vg 0	Hematology				
	MPV (fL)	MPV (fL)	MPV (fL)	MPV (fL)	MPV (fL)
	8	22	43	50	85
101	9.9	10.3	10.3	-	-
102	9.3	9.3	9.6	9.1	9.3
Mean	9.6	9.8	10.0	9.1	9.3
SD	0.4	0.7	0.5	-	-
Max	9.9	10.3	10.3	9.1	9.3
Min	9.3	9.3	9.6	9.1	9.3

1/10/2024 11:19:59AM Page: 16

AmplifyBio Study Number A-579-01

Individual Animal Hematology Data

Sex: Male Day(s) Relative to Start Date

Group 2 Low Total vg 6x10^13	Hematology										
	Red Blood Cells (x10^6 cells/µL)	Red Blood Cells (x10^6 cells/µL)	Red Blood Cells (x10^6 cells/µL)	Red Blood Cells (x10^6 cells/µL)	Red Blood Cells (x10^6 cells/µL)	Red Blood Cells (x10^6 cells/µL)	Red Blood Cells (x10^6 cells/µL)	Hemoglobin (g/dL)	Hemoglobin (g/dL)	Hemoglobin (g/dL)	Hemoglobin (g/dL)
	-42	2	8	22	43	50	85	-42	2	8	22
201	5.27	5.02	5.15	5.56	5.66	-	-	12.4	11.8	12.1	13.0
202	5.44	5.21	4.95	5.46	5.12	-	-	13.2	12.3	12.2	13.2
203	6.50	6.21	5.95	6.45	6.50	6.21	7.06	13.6	12.8	12.3	13.3
204	5.38	5.09	4.88	5.46	5.76	5.43	5.59	13.2	12.3	11.9	13.1
Mean	5.65	5.38	5.23	5.73	5.76	5.82	6.33	13.1	12.3	12.1	13.2
SD	0.57	0.56	0.49	0.48	0.57	0.55	1.04	0.5	0.4	0.2	0.1
Max	6.50	6.21	5.95	6.45	6.50	6.21	7.06	13.6	12.8	12.3	13.3
Min	5.27	5.02	4.88	5.46	5.12	5.43	5.59	12.4	11.8	11.9	13.0

AmplifyBio Study Number A-579-01

Individual Animal Hematology Data

Sex: Male Day(s) Relative to Start Date

Group 2 Low Total vg 6x10^13	Hematology										
	Hemoglobin (g/dL)	Hemoglobin (g/dL)	Hemoglobin (g/dL)	Hematocrit (%)	Hematocrit (%)	Hematocrit (%)	Hematocrit (%)	Hematocrit (%)	Hematocrit (%)	Hematocrit (%)	MCV (fL)
	43	50	85	-42	2	8	22	43	50	85	-42
201	13.3	-	-	40.2	39.2	40.0	44.9	44.2	-	-	76.3
202	12.4	-	-	41.9	42.0	38.2	43.3	39.9	-	-	77.1
203	13.6	12.9	14.3	47.6	45.7	41.4	46.7	47.5	46.0	49.0	73.1
204	13.8	13.1	13.3	40.0	39.3	37.0	42.1	44.5	41.5	42.4	74.3
Mean	13.3	13.0	13.8	42.4	41.6	39.2	44.3	44.0	43.8	45.7	75.2
SD	0.6	0.1	0.7	3.6	3.1	1.9	2.0	3.1	3.2	4.7	1.8
Max	13.8	13.1	14.3	47.6	45.7	41.4	46.7	47.5	46.0	49.0	77.1
Min	12.4	12.9	13.3	40.0	39.2	37.0	42.1	39.9	41.5	42.4	73.1

AmplifyBio Study Number A-579-01

Individual Animal Hematology Data

Sex: Male Day(s) Relative to Start Date

Group 2 Low Total vg 6x10^13	Hematology										
	MCV (fL)	MCV (fL)	MCV (fL)	MCV (fL)	MCV (fL)	MCV (fL)	MCH (pg)	MCH (pg)	MCH (pg)	MCH (pg)	MCH (pg)
	2	8	22	43	50	85	-42	2	8	22	43
201	77.9	77.7	80.7	78.1	-	-	23.5	23.5	23.6	23.4	23.4
202	80.6	77.1	79.3	77.9	-	-	24.2	23.5	24.6	24.1	24.3
203	73.7	69.6	72.5	73.1	74.0	69.4	20.9	20.7	20.6	20.6	20.9
204	77.1	75.8	77.1	77.3	76.5	75.9	24.5	24.1	24.3	24.0	23.9
Mean	77.3	75.1	77.4	76.6	75.3	72.7	23.3	23.0	23.3	23.0	23.1
SD	2.8	3.7	3.6	2.4	1.8	4.6	1.6	1.5	1.8	1.6	1.5
Max	80.6	77.7	80.7	78.1	76.5	75.9	24.5	24.1	24.6	24.1	24.3
Min	73.7	69.6	72.5	73.1	74.0	69.4	20.9	20.7	20.6	20.6	20.9

AmplifyBio Study Number A-579-01

Individual Animal Hematology Data

Sex: Male Day(s) Relative to Start Date

Group 2 Low Total vg 6x10^13	Hematology										
	MCH (pg)	MCH (pg)	MCHC (g/dL)	MCHC (g/dL)	MCHC (g/dL)	MCHC (g/dL)	MCHC (g/dL)	MCHC (g/dL)	MCHC (g/dL)	RETIC (x10^3 cells/µL)	RETIC (x10^3 cells/µL)
	50	85	-42	2	8	22	43	50	85	-42	2
201	-	-	30.8	30.1	30.3	29.0	30.0	-	-	43.8	142.9
202	-	-	31.4	29.2	31.9	30.4	31.2	-	-	59.7	118.0
203	20.7	20.2	28.6	28.1	29.6	28.4	28.6	28.0	29.1	41.5	79.5
204	24.1	23.8	33.0	31.2	32.0	31.2	31.0	31.5	31.4	32.3	62.3
Mean	22.4	22.0	31.0	29.7	31.0	29.8	30.2	29.8	30.3	44.3	100.7
SD	2.4	2.5	1.8	1.3	1.2	1.3	1.2	2.5	1.6	11.4	36.5
Max	24.1	23.8	33.0	31.2	32.0	31.2	31.2	31.5	31.4	59.7	142.9
Min	20.7	20.2	28.6	28.1	29.6	28.4	28.6	28.0	29.1	32.3	62.3

AmplifyBio Study Number A-579-01

Individual Animal Hematology Data

Sex: Male Day(s) Relative to Start Date

Group 2 Low Total vg 6x10^13	Hematology										
	RETIC (x10^3 cells/µL)	RETIC (x10^3 cells/µL)	RETIC (x10^3 cells/µL)	RETIC (x10^3 cells/µL)	RETIC (x10^3 cells/µL)	% Retic (%)	% Retic (%)	% Retic (%)	% Retic (%)	% Retic (%)	% Retic (%)
	8	22	43	50	85	2	8	22	43	50	85
201	166.6	97.2	50.0	-	-	2.84	3.24	1.75	0.88	-	-
202	82.5	81.1	66.8	-	-	2.26	1.67	1.49	1.30	-	-
203	64.6	66.9	45.1	58.7	31.3	1.28	1.08	1.04	0.69	0.95	0.44
204	90.5	52.6	47.6	76.1	42.8	1.22	1.85 [a]	0.96	0.83	1.40	0.77
Mean	101.1	74.5	52.4	67.4	37.1	1.90	1.96	1.31	0.93	1.18	0.61
SD	45.0	19.1	9.8	12.3	8.1	0.79	0.91	0.37	0.26	0.32	0.23
Max	166.6	97.2	66.8	76.1	42.8	2.84	3.24	1.75	1.30	1.40	0.77
Min	64.6	52.6	45.1	58.7	31.3	1.22	1.08	0.96	0.69	0.95	0.44

[a] [RC:Morphology WNL]

AmplifyBio Study Number A-579-01

Individual Animal Hematology Data

Sex: Male Day(s) Relative to Start Date

Group 2 Low Total vg 6x10^13	Hematology										
	White Blood Cells (x10^3 cells/µL)	White Blood Cells (x10^3 cells/µL)	White Blood Cells (x10^3 cells/µL)	White Blood Cells (x10^3 cells/µL)	White Blood Cells (x10^3 cells/µL)	White Blood Cells (x10^3 cells/µL)	White Blood Cells (x10^3 cells/µL)	Neutrophils (x10^3 cells/µL)	Neutrophils (x10^3 cells/µL)	Neutrophils (x10^3 cells/µL)	Neutrophils (x10^3 cells/µL)
	-42	2	8	22	43	50	85	-42	2	8	22
201	12.23	9.58	6.58	8.50 [a]	6.65	-	-	6.97	2.95	2.17	1.23 [b]
202	7.00	11.23	5.51	8.89	5.89	-	-	3.46	4.80	2.04	2.50
203	6.43	9.12	4.05	6.13	5.80	7.93	4.29	3.08	2.76	0.94	1.30
204	11.74	9.89	8.44	16.07	10.47	11.76	6.84	9.41	7.24	6.66	11.68
Mean	9.35	9.96	6.15	9.90	7.20	9.85	5.57	5.73	4.44	2.95	4.18
SD	3.06	0.91	1.85	4.29	2.21	2.71	1.80	3.01	2.08	2.53	5.04
Max	12.23	11.23	8.44	16.07	10.47	11.76	6.84	9.41	7.24	6.66	11.68
Min	6.43	9.12	4.05	6.13	5.80	7.93	4.29	3.08	2.76	0.94	1.23

[a] [RC:NEUT Number decreased confirmed.]
[b] [RC:Number decreased confirmed.]

1/10/2024 11:19:59AM Page: 22

AmplifyBio Study Number A-579-01

Individual Animal Hematology Data

Sex: Male Day(s) Relative to Start Date

Group 2 Low Total vg 6x10^13	Hematology										
	Neutrophils (x10^3 cells/µL)	Neutrophils (x10^3 cells/µL)	Neutrophils (x10^3 cells/µL)	Lymphocytes (x10^3 cells/µL)	Lymphocytes (x10^3 cells/µL)	Lymphocytes (x10^3 cells/µL)	Lymphocytes (x10^3 cells/µL)	Lymphocytes (x10^3 cells/µL)	Lymphocytes (x10^3 cells/µL)	Lymphocytes (x10^3 cells/µL)	Monocytes (x10^3 cells/µL)
	43	50	85	-42	2	8	22	43	50	85	-42
201	2.43	-	-	4.29	5.71	3.50	6.31	3.39	-	-	0.87
202	1.87	-	-	2.95	5.70	3.13	5.66	3.62	-	-	0.48
203	0.89	1.87	1.05	3.03	5.68	2.74	4.32	4.50	5.44	2.49	0.23
204	4.86	7.19	3.13	1.88	2.33	1.54	3.80	5.03	4.10	3.44	0.40
Mean	2.51	4.53	2.09	3.04	4.86	2.73	5.02	4.14	4.77	2.97	0.50
SD	1.69	3.76	1.47	0.99	1.68	0.85	1.16	0.76	0.95	0.67	0.27
Max	4.86	7.19	3.13	4.29	5.71	3.50	6.31	5.03	5.44	3.44	0.87
Min	0.89	1.87	1.05	1.88	2.33	1.54	3.80	3.39	4.10	2.49	0.23

AmplifyBio Study Number A-579-01

Individual Animal Hematology Data

Sex: Male Day(s) Relative to Start Date

Group 2 Low Total vg 6x10^13	Hematology										
	Monocytes (x10^3 cells/µL)	Monocytes (x10^3 cells/µL)	Monocytes (x10^3 cells/µL)	Monocytes (x10^3 cells/µL)	Monocytes (x10^3 cells/µL)	Monocytes (x10^3 cells/µL)	Eosinophils (x10^3 cells/µL)	Eosinophils (x10^3 cells/µL)	Eosinophils (x10^3 cells/µL)	Eosinophils (x10^3 cells/µL)	Eosinophils (x10^3 cells/µL)
	2	8	22	43	50	85	-42	2	8	22	43
201	0.81	0.80	0.84	0.73	-	-	0.00	0.03	0.02	0.02	0.02
202	0.52	0.24	0.47	0.26	-	-	0.06	0.13	0.06	0.18	0.10
203	0.49	0.31	0.39	0.32	0.44	0.16	0.06	0.13	0.05	0.09	0.06
204	0.24	0.18	0.50	0.48	0.38	0.18	0.01	0.02	0.00	0.03	0.02
Mean	0.52	0.38	0.55	0.45	0.41	0.17	0.03	0.08	0.03	0.08	0.05
SD	0.23	0.28	0.20	0.21	0.04	0.01	0.03	0.06	0.03	0.07	0.04
Max	0.81	0.80	0.84	0.73	0.44	0.18	0.06	0.13	0.06	0.18	0.10
Min	0.24	0.18	0.39	0.26	0.38	0.16	0.00	0.02	0.00	0.02	0.02

AmplifyBio Study Number A-579-01

Individual Animal Hematology Data

Sex: Male Day(s) Relative to Start Date

Group 2 Low Total vg 6x10^13	Hematology										
	Eosinophils (x10^3 cells/µL)	Eosinophils (x10^3 cells/µL)	Basophils (x10^3 cells/µL)	Basophils (x10^3 cells/µL)	Basophils (x10^3 cells/µL)	Basophils (x10^3 cells/µL)	Basophils (x10^3 cells/µL)	Basophils (x10^3 cells/µL)	Basophils (x10^3 cells/µL)	LUC (x10^3 cells/µL)	LUC (x10^3 cells/µL)
	50	85	-42	2	8	22	43	50	85	-42	2
201	-	-	0.03	0.02	0.01	0.01	0.01	-	-	0.07	0.07
202	-	-	0.02	0.02	0.00	0.01	0.00	-	-	0.04	0.06
203	0.10	0.56	0.02	0.02	0.00	0.01	0.01	0.02	0.01	0.02	0.04
204	0.03	0.04	0.02	0.02	0.01	0.03	0.03	0.02	0.01	0.02	0.04
Mean	0.07	0.30	0.02	0.02	0.01	0.02	0.01	0.02	0.01	0.04	0.05
SD	0.05	0.37	0.00	0.00	0.01	0.01	0.01	0.00	0.00	0.02	0.02
Max	0.10	0.56	0.03	0.02	0.01	0.03	0.03	0.02	0.01	0.07	0.07
Min	0.03	0.04	0.02	0.02	0.00	0.01	0.00	0.02	0.01	0.02	0.04

AmplifyBio Study Number A-579-01

Individual Animal Hematology Data

Sex: Male Day(s) Relative to Start Date

Group 2 Low Total vg 6x10^13	Hematology										
	LUC (x10^3 cells/µL)	LUC (x10^3 cells/µL)	LUC (x10^3 cells/µL)	LUC (x10^3 cells/µL)	LUC (x10^3 cells/µL)	% Neutrophils (%)	% Neutrophils (%)	% Neutrophils (%)	% Neutrophils (%)	% Neutrophils (%)	% Neutrophils (%)
	8	22	43	50	85	-42	2	8	22	43	50
201	0.08	0.09	0.07	-	-	57.0	30.8	33.0	14.4 [a]	36.6	-
202	0.04	0.06	0.03	-	-	49.4	42.7	37.0	28.1	31.8	-
203	0.01	0.03	0.02	0.05	0.01	47.9	30.2	23.2	21.2	15.3	23.6
204	0.04	0.04	0.04	0.04	0.03	80.2	73.2	78.9	72.7	46.4	61.1
Mean	0.04	0.06	0.04	0.05	0.02	58.6	44.2	43.0	34.1	32.5	42.4
SD	0.03	0.03	0.02	0.01	0.01	14.9	20.2	24.6	26.3	13.0	26.5
Max	0.08	0.09	0.07	0.05	0.03	80.2	73.2	78.9	72.7	46.4	61.1
Min	0.01	0.03	0.02	0.04	0.01	47.9	30.2	23.2	14.4	15.3	23.6

[a] [RC:Number decreased confirmed.]

AmplifyBio Study Number A-579-01

Individual Animal Hematology Data

Sex: Male Day(s) Relative to Start Date

Group 2 Low Total vg 6x10^13	Hematology										
	% Neutrophils (%)	% Lymphocytes (%)	% Lymphocytes (%)	% Lymphocytes (%)	% Lymphocytes (%)	% Lymphocytes (%)	% Lymphocytes (%)	% Lymphocytes (%)	% Monocytes (%)	% Monocytes (%)	% Monocytes (%)
	85	-42	2	8	22	43	50	85	-42	2	8
201	-	35.1	59.6	53.2	74.2	51.0	-	-	7.10	8.40	12.10
202	-	42.1	50.7	56.8	63.7	61.6	-	-	6.80	4.70	4.30
203	24.5	47.1	62.3	67.6	70.4	77.6	68.6	58.1	3.50	5.30	7.60
204	45.8	16.0	23.5	18.3	23.6	48.1	34.9	50.2	3.40	2.40	2.10
Mean	35.2	35.1	49.0	49.0	58.0	59.6	51.8	54.2	5.20	5.20	6.53
SD	15.1	13.6	17.7	21.3	23.3	13.3	23.8	5.6	2.02	2.47	4.35
Max	45.8	47.1	62.3	67.6	74.2	77.6	68.6	58.1	7.10	8.40	12.10
Min	24.5	16.0	23.5	18.3	23.6	48.1	34.9	50.2	3.40	2.40	2.10

AmplifyBio Study Number A-579-01

Individual Animal Hematology Data

Sex: Male Day(s) Relative to Start Date

Group 2 Low Total vg 6x10^13	Hematology										
	% Monocytes (%)	% Monocytes (%)	% Monocytes (%)	% Monocytes (%)	% Eosinophils (%)	% Eosinophils (%)	% Eosinophils (%)	% Eosinophils (%)	% Eosinophils (%)	% Eosinophils (%)	% Eosinophils (%)
	22	43	50	85	-42	2	8	22	43	50	85
201	9.90	10.90	-	-	0.0	0.3	0.3	0.3	0.3	-	-
202	5.30	4.40	-	-	0.9	1.2	1.0	2.1	1.6	-	-
203	6.40	5.60	5.60	3.80	0.9	1.4	1.3	1.5	1.0	1.3	13.1
204	3.10	4.60	3.20	2.70	0.1	0.2	0.1	0.2	0.2	0.2	0.6
Mean	6.18	6.38	4.40	3.25	0.5	0.8	0.7	1.0	0.8	0.8	6.9
SD	2.84	3.06	1.70	0.78	0.5	0.6	0.6	0.9	0.7	0.8	8.8
Max	9.90	10.90	5.60	3.80	0.9	1.4	1.3	2.1	1.6	1.3	13.1
Min	3.10	4.40	3.20	2.70	0.0	0.2	0.1	0.2	0.2	0.2	0.6

AmplifyBio Study Number A-579-01

Individual Animal Hematology Data

Sex: Male Day(s) Relative to Start Date

Group 2 Low Total vg 6x10^13	Hematology										
	% BASO (%)	% BASO (%)	% BASO (%)	% BASO (%)	% BASO (%)	% BASO (%)	% BASO (%)	% LUC (%)	% LUC (%)	% LUC (%)	% LUC (%)
	-42	2	8	22	43	50	85	-42	2	8	22
201	0.2	0.2	0.1	0.2	0.2	-	-	0.6	0.7	1.2	1.0
202	0.2	0.2	0.1	0.2	0.1	-	-	0.6	0.6	0.8	0.7
203	0.3	0.2	0.1	0.1	0.2	0.3	0.3	0.3	0.5	0.2	0.4
204	0.2	0.2	0.1	0.2	0.3	0.2	0.2	0.2	0.4	0.5	0.2
Mean	0.2	0.2	0.1	0.2	0.2	0.3	0.3	0.4	0.6	0.7	0.6
SD	0.1	0.0	0.0	0.1	0.1	0.1	0.1	0.2	0.1	0.4	0.4
Max	0.3	0.2	0.1	0.2	0.3	0.3	0.3	0.6	0.7	1.2	1.0
Min	0.2	0.2	0.1	0.1	0.1	0.2	0.2	0.2	0.4	0.2	0.2

AmplifyBio Study Number A-579-01

Individual Animal Hematology Data

Sex: Male Day(s) Relative to Start Date

Group 2 Low Total vg 6x10^13	Hematology										
	% LUC (%)	% LUC (%)	% LUC (%)	Platelets (x10^3 cells/µL)	Platelets (x10^3 cells/µL)	Platelets (x10^3 cells/µL)	Platelets (x10^3 cells/µL)	Platelets (x10^3 cells/µL)	Platelets (x10^3 cells/µL)	Platelets (x10^3 cells/µL)	MPV (fL)
	43	50	85	-42	2	8	22	43	50	85	2
201	1.0	-	-	283	346 [a]	374 [a]	324 [a]	336 [a]	-	-	11.0
202	0.5	-	-	378	378	385	380	372	-	-	10.1
203	0.3	0.6	0.2	444	403	487	427	498	463	466	10.4
204	0.4	0.3	0.5	319	317	377	301 [a]	269 [a]	313 [a]	295	10.1
Mean	0.6	0.5	0.4	356	361	406	358	369	388	381	10.4
SD	0.3	0.2	0.2	71	37	54	57	96	106	121	0.4
Max	1.0	0.6	0.5	444	403	487	427	498	463	466	11.0
Min	0.3	0.3	0.2	283	317	374	301	269	313	295	10.1

[a] [RC:Clumping present]

AmplifyBio Study Number A-579-01

Individual Animal Hematology Data

Sex: Male Day(s) Relative to Start Date

Group 2 Low Total vg 6x10^13	Hematology				
	MPV (fL)	MPV (fL)	MPV (fL)	MPV (fL)	MPV (fL)
	8	22	43	50	85
201	10.6	11.4	10.7	-	-
202	9.0	9.7	8.9	-	-
203	9.5	10.0	9.8	9.3	9.4
204	9.4	10.9	11.1	10.8	9.6
Mean	9.6	10.5	10.1	10.1	9.5
SD	0.7	0.8	1.0	1.1	0.1
Max	10.6	11.4	11.1	10.8	9.6
Min	9.0	9.7	8.9	9.3	9.4

AmplifyBio Study Number A-579-01

Individual Animal Hematology Data

Sex: Male Day(s) Relative to Start Date

Group 3 High Total vg 1.2x10^14	Hematology										
	Red Blood Cells (x10^6 cells/µL)	Red Blood Cells (x10^6 cells/µL)	Red Blood Cells (x10^6 cells/µL)	Red Blood Cells (x10^6 cells/µL)	Red Blood Cells (x10^6 cells/µL)	Red Blood Cells (x10^6 cells/µL)	Red Blood Cells (x10^6 cells/µL)	Hemoglobin (g/dL)	Hemoglobin (g/dL)	Hemoglobin (g/dL)	Hemoglobin (g/dL)
	-43	2	8	22	43	50	85	-43	2	8	22
301	5.18	5.31	5.31	5.94	5.58	-	-	12.5	12.6	12.7	14.2
302	5.32	5.41	5.78	6.20	5.43	-	-	12.3	12.6	13.3	14.3
303	6.32	6.11	6.41	6.53	6.59	6.11	6.03	14.0	13.3	14.0	14.2
304	5.97	6.24	5.57	6.33	6.31	6.03	6.01	12.3	13.0	11.8	13.4
Mean	5.70	5.77	5.77	6.25	5.98	6.07	6.02	12.8	12.9	13.0	14.0
SD	0.54	0.48	0.47	0.25	0.56	0.06	0.01	0.8	0.3	0.9	0.4
Max	6.32	6.24	6.41	6.53	6.59	6.11	6.03	14.0	13.3	14.0	14.3
Min	5.18	5.31	5.31	5.94	5.43	6.03	6.01	12.3	12.6	11.8	13.4

AmplifyBio Study Number A-579-01

Individual Animal Hematology Data

Sex: Male Day(s) Relative to Start Date

Group 3 High Total vg 1.2x10^14	Hematology										
	Hemoglobin (g/dL)	Hemoglobin (g/dL)	Hemoglobin (g/dL)	Hematocrit (%)	Hematocrit (%)	Hematocrit (%)	Hematocrit (%)	Hematocrit (%)	Hematocrit (%)	Hematocrit (%)	MCV (fL)
	43	50	85	-43	2	8	22	43	50	85	-43
301	13.5	-	-	40.1	43.5	41.8	48.6	43.7	-	-	77.6
302	12.6	-	-	40.5	40.2	43.1	44.9	41.6	-	-	76.0
303	14.6	13.5	13.3	43.8	43.5	44.4	46.2	47.6	44.7	41.8	69.3
304	13.6	12.8	12.6	41.5	45.2	38.6	44.1	43.9	42.9	41.5	69.4
Mean	13.6	13.2	13.0	41.5	43.1	42.0	46.0	44.2	43.8	41.7	73.1
SD	0.8	0.5	0.5	1.7	2.1	2.5	2.0	2.5	1.3	0.2	4.4
Max	14.6	13.5	13.3	43.8	45.2	44.4	48.6	47.6	44.7	41.8	77.6
Min	12.6	12.8	12.6	40.1	40.2	38.6	44.1	41.6	42.9	41.5	69.3

AmplifyBio Study Number A-579-01

Individual Animal Hematology Data

Sex: Male Day(s) Relative to Start Date

Group 3 High Total vg 1.2x10^14	Hematology										
	MCV (fL)	MCV (fL)	MCV (fL)	MCV (fL)	MCV (fL)	MCV (fL)	MCH (pg)	MCH (pg)	MCH (pg)	MCH (pg)	MCH (pg)
	2	8	22	43	50	85	-43	2	8	22	43
301	82.0	78.6	81.8	78.3	-	-	24.2	23.7	23.8	23.9	24.2
302	74.2	74.6	72.4	76.6	-	-	23.1	23.2	23.0	23.0	23.3
303	71.2	69.2	70.8	72.2	73.1	69.3	22.1	21.9	21.9	21.7	22.2
304	72.4	69.2	69.7	69.6	71.2	69.1	20.6	20.8	21.2	21.2	21.5
Mean	75.0	72.9	73.7	74.2	72.2	69.2	22.5	22.4	22.5	22.5	22.8
SD	4.9	4.6	5.5	4.0	1.3	0.1	1.5	1.3	1.2	1.2	1.2
Max	82.0	78.6	81.8	78.3	73.1	69.3	24.2	23.7	23.8	23.9	24.2
Min	71.2	69.2	69.7	69.6	71.2	69.1	20.6	20.8	21.2	21.2	21.5

1/10/2024 11:19:59AM Page: 34

AmplifyBio Study Number A-579-01

Individual Animal Hematology Data

Sex: Male Day(s) Relative to Start Date

Group 3 High Total vg 1.2x10^14	Hematology										
	MCH (pg)	MCH (pg)	MCHC (g/dL)	MCHC (g/dL)	MCHC (g/dL)	MCHC (g/dL)	MCHC (g/dL)	MCHC (g/dL)	MCHC (g/dL)	RETIC (x10^3 cells/µL)	RETIC (x10^3 cells/µL)
	50	85	-43	2	8	22	43	50	85	-43	2
301	-	-	31.1	29.0	30.3	29.2	30.9	-	-	52.5	115.4
302	-	-	30.5	31.2	30.8	31.8	30.4	-	-	51.2	73.7
303	22.1	22.0	31.9	30.7	31.6	30.6	30.8	30.3	31.7	65.6	103.5
304	21.3	20.9	29.7	28.7	30.6	30.5	30.9	29.9	30.2	27.6	68.4
Mean	21.7	21.5	30.8	29.9	30.8	30.5	30.8	30.1	31.0	49.2	90.3
SD	0.6	0.8	0.9	1.2	0.6	1.1	0.2	0.3	1.1	15.8	22.8
Max	22.1	22.0	31.9	31.2	31.6	31.8	30.9	30.3	31.7	65.6	115.4
Min	21.3	20.9	29.7	28.7	30.3	29.2	30.4	29.9	30.2	27.6	68.4

AmplifyBio Study Number A-579-01

Individual Animal Hematology Data

Sex: Male Day(s) Relative to Start Date

Group 3 High Total vg 1.2x10^14	Hematology										
	RETIC (x10^3 cells/µL)	RETIC (x10^3 cells/µL)	RETIC (x10^3 cells/µL)	RETIC (x10^3 cells/µL)	RETIC (x10^3 cells/µL)	% Retic (%)	% Retic (%)	% Retic (%)	% Retic (%)	% Retic (%)	% Retic (%)
	8	22	43	50	85	2	8	22	43	50	85
301	72.1	97.3	34.6	-	-	2.17	1.36	1.64	0.62	-	-
302	45.0	52.2	40.1	-	-	1.36	0.78	0.84	0.74	-	-
303	71.3	87.7	78.2	131.9	44.8	1.69	1.11	1.34	1.19	2.16	0.74
304	65.8	55.9	28.3	69.5	30.8	1.10	1.18	0.88	0.45	1.15	0.51
Mean	63.6	73.3	45.3	100.7	37.8	1.58	1.11	1.18	0.75	1.66	0.63
SD	12.7	22.6	22.5	44.1	9.9	0.46	0.24	0.38	0.32	0.71	0.16
Max	72.1	97.3	78.2	131.9	44.8	2.17	1.36	1.64	1.19	2.16	0.74
Min	45.0	52.2	28.3	69.5	30.8	1.10	0.78	0.84	0.45	1.15	0.51

AmplifyBio Study Number A-579-01

Individual Animal Hematology Data

Sex: Male Day(s) Relative to Start Date

Group 3 High Total vg 1.2x10^14	Hematology										
	White Blood Cells (x10^3 cells/µL)	White Blood Cells (x10^3 cells/µL)	White Blood Cells (x10^3 cells/µL)	White Blood Cells (x10^3 cells/µL)	White Blood Cells (x10^3 cells/µL)	White Blood Cells (x10^3 cells/µL)	White Blood Cells (x10^3 cells/µL)	Neutrophils (x10^3 cells/µL)	Neutrophils (x10^3 cells/µL)	Neutrophils (x10^3 cells/µL)	Neutrophils (x10^3 cells/µL)
	-43	2	8	22	43	50	85	-43	2	8	22
301	7.93	9.69	8.16	9.05	5.98	-	-	4.38	3.30	2.09	2.37
302	9.76	13.58	9.26	14.68	4.22	-	-	7.21	9.45	6.01	11.39
303	10.77	14.04	11.51	17.33	10.20	13.91	11.59	4.88	5.39	3.58	4.86
304	19.12	18.90	16.81	14.58	16.34	13.87	10.43	14.83	9.98	11.35	5.45
Mean	11.90	14.05	11.44	13.91	9.19	13.89	11.01	7.83	7.03	5.76	6.02
SD	4.96	3.78	3.85	3.48	5.39	0.03	0.82	4.83	3.22	4.06	3.82
Max	19.12	18.90	16.81	17.33	16.34	13.91	11.59	14.83	9.98	11.35	11.39
Min	7.93	9.69	8.16	9.05	4.22	13.87	10.43	4.38	3.30	2.09	2.37

AmplifyBio Study Number A-579-01

Individual Animal Hematology Data

Sex: Male Day(s) Relative to Start Date

Group 3 High Total vg 1.2x10^14	Hematology										
	Neutrophils (x10^3 cells/µL)	Neutrophils (x10^3 cells/µL)	Neutrophils (x10^3 cells/µL)	Lymphocytes (x10^3 cells/µL)	Lymphocytes (x10^3 cells/µL)	Lymphocytes (x10^3 cells/µL)	Lymphocytes (x10^3 cells/µL)	Lymphocytes (x10^3 cells/µL)	Lymphocytes (x10^3 cells/µL)	Lymphocytes (x10^3 cells/µL)	Monocytes (x10^3 cells/µL)
	43	50	85	-43	2	8	22	43	50	85	-43
301	3.26	-	-	3.20	5.78	5.18	6.03	2.38	-	-	0.29
302	2.13	-	-	2.09	3.63	2.57	2.73	1.71	-	-	0.39
303	1.85	4.52	6.26	5.36	7.68	7.20	11.30	7.83	8.62	4.92	0.44
304	6.93	2.90	2.01	3.75	8.12	4.86	8.51	8.84	10.28	7.95	0.48
Mean	3.54	3.71	4.14	3.60	6.30	4.95	7.14	5.19	9.45	6.44	0.40
SD	2.34	1.15	3.01	1.36	2.05	1.90	3.65	3.67	1.17	2.14	0.08
Max	6.93	4.52	6.26	5.36	8.12	7.20	11.30	8.84	10.28	7.95	0.48
Min	1.85	2.90	2.01	2.09	3.63	2.57	2.73	1.71	8.62	4.92	0.29

AmplifyBio Study Number A-579-01

Individual Animal Hematology Data

Sex: Male Day(s) Relative to Start Date

Group 3 High Total vg 1.2x10^14	Hematology										
	Monocytes (x10^3 cells/µL)	Monocytes (x10^3 cells/µL)	Monocytes (x10^3 cells/µL)	Monocytes (x10^3 cells/µL)	Monocytes (x10^3 cells/µL)	Monocytes (x10^3 cells/µL)	Eosinophils (x10^3 cells/µL)	Eosinophils (x10^3 cells/µL)	Eosinophils (x10^3 cells/µL)	Eosinophils (x10^3 cells/µL)	Eosinophils (x10^3 cells/µL)
	2	8	22	43	50	85	-43	2	8	22	43
301	0.47	0.69	0.49	0.27	-	-	0.01	0.03	0.09	0.07	0.03
302	0.43	0.59	0.50	0.28	-	-	0.01	0.01	0.02	0.01	0.06
303	0.77	0.57	0.92	0.41	0.60	0.31	0.01	0.07	0.04	0.09	0.03
304	0.61	0.48	0.48	0.39	0.48	0.27	0.01	0.07	0.03	0.04	0.08
Mean	0.57	0.58	0.60	0.34	0.54	0.29	0.01	0.05	0.05	0.05	0.05
SD	0.15	0.09	0.22	0.07	0.08	0.03	0.00	0.03	0.03	0.04	0.02
Max	0.77	0.69	0.92	0.41	0.60	0.31	0.01	0.07	0.09	0.09	0.08
Min	0.43	0.48	0.48	0.27	0.48	0.27	0.01	0.01	0.02	0.01	0.03

AmplifyBio Study Number A-579-01

Individual Animal Hematology Data

Sex: Male Day(s) Relative to Start Date

Group 3 High Total vg 1.2x10^14	Hematology										
	Eosinophils (x10^3 cells/µL)	Eosinophils (x10^3 cells/µL)	Basophils (x10^3 cells/µL)	Basophils (x10^3 cells/µL)	Basophils (x10^3 cells/µL)	Basophils (x10^3 cells/µL)	Basophils (x10^3 cells/µL)	Basophils (x10^3 cells/µL)	Basophils (x10^3 cells/µL)	LUC (x10^3 cells/µL)	LUC (x10^3 cells/µL)
	50	85	-43	2	8	22	43	50	85	-43	2
301	-	-	0.02	0.03	0.03	0.04	0.00	-	-	0.04	0.06
302	-	-	0.01	0.01	0.01	0.02	0.01	-	-	0.05	0.05
303	0.07	0.06	0.02	0.04	0.03	0.06	0.03	0.04	0.02	0.05	0.09
304	0.10	0.11	0.02	0.03	0.03	0.02	0.03	0.03	0.02	0.03	0.09
Mean	0.09	0.09	0.02	0.03	0.03	0.04	0.02	0.04	0.02	0.04	0.07
SD	0.02	0.04	0.01	0.01	0.01	0.02	0.02	0.01	0.00	0.01	0.02
Max	0.10	0.11	0.02	0.04	0.03	0.06	0.03	0.04	0.02	0.05	0.09
Min	0.07	0.06	0.01	0.01	0.01	0.02	0.00	0.03	0.02	0.03	0.05

1/10/2024 11:19:59AM Page: 40

AmplifyBio Study Number A-579-01

Individual Animal Hematology Data

Sex: Male Day(s) Relative to Start Date

Group 3 High Total vg 1.2x10^14	Hematology										
	LUC (x10^3 cells/µL)	LUC (x10^3 cells/µL)	LUC (x10^3 cells/µL)	LUC (x10^3 cells/µL)	LUC (x10^3 cells/µL)	% Neutrophils (%)	% Neutrophils (%)	% Neutrophils (%)	% Neutrophils (%)	% Neutrophils (%)	% Neutrophils (%)
	8	22	43	50	85	-43	2	8	22	43	50
301	0.09	0.05	0.03	-	-	55.2	34.1	25.6	26.2	54.5	-
302	0.06	0.02	0.03	-	-	73.9	69.6	64.9	77.6	50.5	-
303	0.09	0.11	0.05	0.06	0.03	45.3	38.4	31.1	28.0	18.1	32.5
304	0.06	0.08	0.06	0.08	0.07	77.6	52.8	67.5	37.4	42.4	20.9
Mean	0.08	0.07	0.04	0.07	0.05	63.0	48.7	47.3	42.3	41.4	26.7
SD	0.02	0.04	0.02	0.01	0.03	15.3	16.1	22.0	24.0	16.3	8.2
Max	0.09	0.11	0.06	0.08	0.07	77.6	69.6	67.5	77.6	54.5	32.5
Min	0.06	0.02	0.03	0.06	0.03	45.3	34.1	25.6	26.2	18.1	20.9

AmplifyBio Study Number A-579-01

Individual Animal Hematology Data

Sex: Male Day(s) Relative to Start Date

Group 3 High Total vg 1.2x10^14	Hematology										
	% Neutrophils (%)	% Lymphocytes (%)	% Lymphocytes (%)	% Lymphocytes (%)	% Lymphocytes (%)	% Lymphocytes (%)	% Lymphocytes (%)	% Lymphocytes (%)	% Monocytes (%)	% Monocytes (%)	% Monocytes (%)
	85	-43	2	8	22	43	50	85	-43	2	8
301	-	40.3	59.7	63.5	66.7	39.8	-	-	3.60	4.90	8.50
302	-	21.4	26.7	27.7	18.6	40.5	-	-	4.00	3.20	6.40
303	54.0	49.8	54.7	62.5	65.2	76.8	62.0	42.4	4.10	5.50	5.00
304	19.3	19.6	43.0	28.9	58.3	54.1	74.1	76.2	2.50	3.20	2.90
Mean	36.7	32.8	46.0	45.7	52.2	52.8	68.1	59.3	3.55	4.20	5.70
SD	24.5	14.7	14.7	20.0	22.7	17.3	8.6	23.9	0.73	1.18	2.36
Max	54.0	49.8	59.7	63.5	66.7	76.8	74.1	76.2	4.10	5.50	8.50
Min	19.3	19.6	26.7	27.7	18.6	39.8	62.0	42.4	2.50	3.20	2.90

AmplifyBio Study Number A-579-01

Individual Animal Hematology Data

Sex: Male Day(s) Relative to Start Date

Group 3 High Total vg 1.2x10^14	Hematology										
	% Monocytes (%)	% Monocytes (%)	% Monocytes (%)	% Monocytes (%)	% Eosinophils (%)	% Eosinophils (%)	% Eosinophils (%)	% Eosinophils (%)	% Eosinophils (%)	% Eosinophils (%)	% Eosinophils (%)
	22	43	50	85	-43	2	8	22	43	50	85
301	5.40	4.60	-	-	0.2	0.3	1.1	0.8	0.5	-	-
302	3.40	6.70	-	-	0.1	0.1	0.2	0.1	1.5	-	-
303	5.30	4.10	4.30	2.70	0.1	0.5	0.4	0.5	0.2	0.5	0.5
304	3.30	2.40	3.50	2.60	0.1	0.4	0.2	0.2	0.5	0.7	1.0
Mean	4.35	4.45	3.90	2.65	0.1	0.3	0.5	0.4	0.7	0.6	0.8
SD	1.16	1.77	0.57	0.07	0.1	0.2	0.4	0.3	0.6	0.1	0.4
Max	5.40	6.70	4.30	2.70	0.2	0.5	1.1	0.8	1.5	0.7	1.0
Min	3.30	2.40	3.50	2.60	0.1	0.1	0.2	0.1	0.2	0.5	0.5

AmplifyBio Study Number A-579-01

Individual Animal Hematology Data

Sex: Male Day(s) Relative to Start Date

Group 3 High Total vg 1.2x10^14	Hematology										
	% BASO (%)	% BASO (%)	% BASO (%)	% BASO (%)	% BASO (%)	% BASO (%)	% BASO (%)	% LUC (%)	% LUC (%)	% LUC (%)	% LUC (%)
	-43	2	8	22	43	50	85	-43	2	8	22
301	0.2	0.3	0.3	0.4	0.1	-	-	0.5	0.7	1.0	0.5
302	0.1	0.1	0.1	0.2	0.1	-	-	0.5	0.4	0.6	0.2
303	0.2	0.3	0.3	0.3	0.3	0.3	0.1	0.5	0.7	0.7	0.6
304	0.1	0.2	0.2	0.2	0.2	0.2	0.2	0.1	0.5	0.3	0.5
Mean	0.2	0.2	0.2	0.3	0.2	0.3	0.2	0.4	0.6	0.7	0.5
SD	0.1	0.1	0.1	0.1	0.1	0.1	0.1	0.2	0.2	0.3	0.2
Max	0.2	0.3	0.3	0.4	0.3	0.3	0.2	0.5	0.7	1.0	0.6
Min	0.1	0.1	0.1	0.2	0.1	0.2	0.1	0.1	0.4	0.3	0.2

AmplifyBio Study Number A-579-01

Individual Animal Hematology Data

Sex: Male Day(s) Relative to Start Date

Group 3 High Total vg 1.2x10^14	Hematology										
	% LUC (%)	% LUC (%)	% LUC (%)	Platelets (x10^3 cells/µL)	Platelets (x10^3 cells/µL)	Platelets (x10^3 cells/µL)	Platelets (x10^3 cells/µL)	Platelets (x10^3 cells/µL)	Platelets (x10^3 cells/µL)	Platelets (x10^3 cells/µL)	MPV (fL)
	43	50	85	-43	2	8	22	43	50	85	2
301	0.5	-	-	400	387	554	354	359	-	-	10.8
302	0.6	-	-	298	327	299	383	509	-	-	10.5
303	0.5	0.4	0.3	496	514	573	558	559 [a]	542 [b]	545 [b]	9.3
304	0.4	0.6	0.6	262	290	321	279	277	284	255	11.1
Mean	0.5	0.5	0.5	364	380	437	394	426	413	400	10.4
SD	0.1	0.1	0.2	106	98	147	118	131	182	205	0.8
Max	0.6	0.6	0.6	496	514	573	558	559	542	545	11.1
Min	0.4	0.4	0.3	262	290	299	279	277	284	255	9.3

[a] [RC:Clumping present]
[b] [RC:Morphology WNL]

AmplifyBio Study Number A-579-01

Individual Animal Hematology Data

Sex: Male Day(s) Relative to Start Date

Group 3 High Total vg 1.2x10^14	Hematology				
	MPV (fL)	MPV (fL)	MPV (fL)	MPV (fL)	MPV (fL)
	8	22	43	50	85
301	9.9	12.1	9.9	-	-
302	10.7	11.4	9.4	-	-
303	9.1	9.3	9.5	9.4	8.8
304	10.2	10.9	11.1	10.8	10.6
Mean	10.0	10.9	10.0	10.1	9.7
SD	0.7	1.2	0.8	1.0	1.3
Max	10.7	12.1	11.1	10.8	10.6
Min	9.1	9.3	9.4	9.4	8.8

AmplifyBio Study Number A-579-01

Individual Animal Hematology Data

Sex: Female Day(s) Relative to Start Date

Group 1 Control Total vg 0	Hematology										
	Red Blood Cells (x10^6 cells/µL)	Red Blood Cells (x10^6 cells/µL)	Red Blood Cells (x10^6 cells/µL)	Red Blood Cells (x10^6 cells/µL)	Red Blood Cells (x10^6 cells/µL)	Red Blood Cells (x10^6 cells/µL)	Red Blood Cells (x10^6 cells/µL)	Hemoglobin (g/dL)	Hemoglobin (g/dL)	Hemoglobin (g/dL)	Hemoglobin (g/dL)
	-41	2	8	22	43	50	85	-41	2	8	22
151	4.77	4.67	4.65	5.09	5.05	-	-	12.7	12.2	12.3	13.4
152	5.32	5.36	5.33	6.03	5.89	5.77	5.61	12.7	12.6	12.5	14.3
Mean	5.05	5.02	4.99	5.56	5.47	5.77	5.61	12.7	12.4	12.4	13.9
SD	0.39	0.49	0.48	0.66	0.59	-	-	0.0	0.3	0.1	0.6
Max	5.32	5.36	5.33	6.03	5.89	5.77	5.61	12.7	12.6	12.5	14.3
Min	4.77	4.67	4.65	5.09	5.05	5.77	5.61	12.7	12.2	12.3	13.4

AmplifyBio Study Number A-579-01

Individual Animal Hematology Data

Sex: Female Day(s) Relative to Start Date

Group 1 Control Total vg 0	Hematology										
	Hemoglobin (g/dL)	Hemoglobin (g/dL)	Hemoglobin (g/dL)	Hematocrit (%)	Hematocrit (%)	Hematocrit (%)	Hematocrit (%)	Hematocrit (%)	Hematocrit (%)	Hematocrit (%)	MCV (fL)
	43	50	85	-41	2	8	22	43	50	85	-41
151	13.3	-	-	39.8	40.2	38.8	42.8	41.5	-	-	83.4
152	13.6	13.3	13.3	41.1	42.1	41.7	46.9	47.8	45.6	43.3	77.3
Mean	13.5	13.3	13.3	40.5	41.2	40.3	44.9	44.7	45.6	43.3	80.4
SD	0.2	-	-	0.9	1.3	2.1	2.9	4.5	-	-	4.3
Max	13.6	13.3	13.3	41.1	42.1	41.7	46.9	47.8	45.6	43.3	83.4
Min	13.3	13.3	13.3	39.8	40.2	38.8	42.8	41.5	45.6	43.3	77.3

AmplifyBio Study Number A-579-01

Individual Animal Hematology Data

Sex: Female Day(s) Relative to Start Date

Group 1 Control Total vg 0	Hematology										
	MCV (fL)	MCV (fL)	MCV (fL)	MCV (fL)	MCV (fL)	MCV (fL)	MCH (pg)	MCH (pg)	MCH (pg)	MCH (pg)	MCH (pg)
	2	8	22	43	50	85	-41	2	8	22	43
151	86.0	83.3	84.0	82.3	-	-	26.6	26.2	26.4	26.2	26.4
152	78.5	78.3	77.7	81.1	79.0	77.2	23.8	23.5	23.5	23.7	23.2
Mean	82.3	80.8	80.9	81.7	79.0	77.2	25.2	24.9	25.0	25.0	24.8
SD	5.3	3.5	4.5	0.8	-	-	2.0	1.9	2.1	1.8	2.3
Max	86.0	83.3	84.0	82.3	79.0	77.2	26.6	26.2	26.4	26.2	26.4
Min	78.5	78.3	77.7	81.1	79.0	77.2	23.8	23.5	23.5	23.7	23.2

AmplifyBio Study Number A-579-01

Individual Animal Hematology Data

Sex: Female Day(s) Relative to Start Date

Group 1 Control Total vg 0	Hematology										
	MCH (pg)	MCH (pg)	MCHC (g/dL)	MCHC (g/dL)	MCHC (g/dL)	MCHC (g/dL)	MCHC (g/dL)	MCHC (g/dL)	MCHC (g/dL)	RETIC (x10^3 cells/µL)	RETIC (x10^3 cells/µL)
	50	85	-41	2	8	22	43	50	85	-41	2
151	-	-	31.8	30.4	31.7	31.2	32.1	-	-	66.2	126.2
152	23.0	23.7	30.8	29.9	30.1	30.5	28.6	29.1	30.7	91.3	111.6
Mean	23.0	23.7	31.3	30.2	30.9	30.9	30.4	29.1	30.7	78.8	118.9
SD	-	-	0.7	0.4	1.1	0.5	2.5	-	-	17.7	10.3
Max	23.0	23.7	31.8	30.4	31.7	31.2	32.1	29.1	30.7	91.3	126.2
Min	23.0	23.7	30.8	29.9	30.1	30.5	28.6	29.1	30.7	66.2	111.6

AmplifyBio Study Number A-579-01

Individual Animal Hematology Data

Sex: Female Day(s) Relative to Start Date

Group 1 Control Total vg 0	Hematology										
	RETIC (x10^3 cells/µL)	RETIC (x10^3 cells/µL)	RETIC (x10^3 cells/µL)	RETIC (x10^3 cells/µL)	RETIC (x10^3 cells/µL)	% Retic (%)	% Retic (%)	% Retic (%)	% Retic (%)	% Retic (%)	% Retic (%)
	8	22	43	50	85	2	8	22	43	50	85
151	99.4	104.4	63.0	-	-	2.70	2.14	2.05	1.25	-	-
152	201.9	107.3	112.5	118.9	71.7	2.08	3.79	1.78	1.91	2.06	1.28
Mean	150.7	105.9	87.8	118.9	71.7	2.39	2.97	1.92	1.58	2.06	1.28
SD	72.5	2.1	35.0	-	-	0.44	1.17	0.19	0.47	-	-
Max	201.9	107.3	112.5	118.9	71.7	2.70	3.79	2.05	1.91	2.06	1.28
Min	99.4	104.4	63.0	118.9	71.7	2.08	2.14	1.78	1.25	2.06	1.28

AmplifyBio Study Number A-579-01

Individual Animal Hematology Data

Sex: Female Day(s) Relative to Start Date

Group 1 Control Total vg 0	Hematology										
	White Blood Cells (x10^3 cells/µL)	White Blood Cells (x10^3 cells/µL)	White Blood Cells (x10^3 cells/µL)	White Blood Cells (x10^3 cells/µL)	White Blood Cells (x10^3 cells/µL)	White Blood Cells (x10^3 cells/µL)	White Blood Cells (x10^3 cells/µL)	Neutrophils (x10^3 cells/µL)	Neutrophils (x10^3 cells/µL)	Neutrophils (x10^3 cells/µL)	Neutrophils (x10^3 cells/µL)
	-41	2	8	22	43	50	85	-41	2	8	22
151	14.15	10.73	8.71	10.01	6.90	-	-	11.53	5.29	4.20	2.54
152	17.27 [a]	15.14	7.91	10.63	21.38	9.65	6.99	14.24	10.25	4.36	3.63
Mean	15.71	12.94	8.31	10.32	14.14	9.65	6.99	12.89	7.77	4.28	3.09
SD	2.21	3.12	0.57	0.44	10.24	-	-	1.92	3.51	0.11	0.77
Max	17.27	15.14	8.71	10.63	21.38	9.65	6.99	14.24	10.25	4.36	3.63
Min	14.15	10.73	7.91	10.01	6.90	9.65	6.99	11.53	5.29	4.20	2.54

[a] [RC:Morphology WNL]

AmplifyBio Study Number A-579-01

Individual Animal Hematology Data

Sex: Female Day(s) Relative to Start Date

Group 1 Control Total vg 0	Hematology										
	Neutrophils (x10^3 cells/µL)	Neutrophils (x10^3 cells/µL)	Neutrophils (x10^3 cells/µL)	Lymphocytes (x10^3 cells/µL)	Lymphocytes (x10^3 cells/µL)	Lymphocytes (x10^3 cells/µL)	Lymphocytes (x10^3 cells/µL)	Lymphocytes (x10^3 cells/µL)	Lymphocytes (x10^3 cells/µL)	Lymphocytes (x10^3 cells/µL)	Monocytes (x10^3 cells/µL)
	43	50	85	-41	2	8	22	43	50	85	-41
151	2.97	-	-	2.14	5.05	4.10	6.91	3.66	-	-	0.42
152	15.64	3.16	1.90	2.28 [a]	3.94	2.91	5.68	4.60	5.37	4.35	0.63
Mean	9.31	3.16	1.90	2.21	4.50	3.51	6.30	4.13	5.37	4.35	0.53
SD	8.96	-	-	0.10	0.78	0.84	0.87	0.66	-	-	0.15
Max	15.64	3.16	1.90	2.28	5.05	4.10	6.91	4.60	5.37	4.35	0.63
Min	2.97	3.16	1.90	2.14	3.94	2.91	5.68	3.66	5.37	4.35	0.42

[a] [RC:%LYMPH WNL]

AmplifyBio Study Number A-579-01

Individual Animal Hematology Data

Sex: Female Day(s) Relative to Start Date

Group 1 Control Total vg 0	Hematology										
	Monocytes (x10^3 cells/µL)	Monocytes (x10^3 cells/µL)	Monocytes (x10^3 cells/µL)	Monocytes (x10^3 cells/µL)	Monocytes (x10^3 cells/µL)	Monocytes (x10^3 cells/µL)	Eosinophils (x10^3 cells/µL)	Eosinophils (x10^3 cells/µL)	Eosinophils (x10^3 cells/µL)	Eosinophils (x10^3 cells/µL)	Eosinophils (x10^3 cells/µL)
	2	8	22	43	50	85	-41	2	8	22	43
151	0.29	0.31	0.36	0.21	-	-	0.00	0.05	0.05	0.10	0.03
152	0.46	0.32	0.68	0.71	0.56	0.30	0.07	0.43	0.29	0.57	0.34
Mean	0.38	0.32	0.52	0.46	0.56	0.30	0.04	0.24	0.17	0.34	0.19
SD	0.12	0.01	0.23	0.35	-	-	0.05	0.27	0.17	0.33	0.22
Max	0.46	0.32	0.68	0.71	0.56	0.30	0.07	0.43	0.29	0.57	0.34
Min	0.29	0.31	0.36	0.21	0.56	0.30	0.00	0.05	0.05	0.10	0.03

AmplifyBio Study Number A-579-01

Individual Animal Hematology Data

Sex: Female Day(s) Relative to Start Date

Group 1 Control Total vg 0	Hematology										
	Eosinophils (x10^3 cells/µL)	Eosinophils (x10^3 cells/µL)	Basophils (x10^3 cells/µL)	Basophils (x10^3 cells/µL)	Basophils (x10^3 cells/µL)	Basophils (x10^3 cells/µL)	Basophils (x10^3 cells/µL)	Basophils (x10^3 cells/µL)	Basophils (x10^3 cells/µL)	LUC (x10^3 cells/µL)	LUC (x10^3 cells/µL)
	50	85	-41	2	8	22	43	50	85	-41	2
151	-	-	0.02	0.02	0.01	0.04	0.01	-	-	0.03	0.04
152	0.46	0.40	0.03	0.03	0.01	0.03	0.06	0.04	0.01	0.03	0.04
Mean	0.46	0.40	0.03	0.03	0.01	0.04	0.04	0.04	0.01	0.03	0.04
SD	-	-	0.01	0.01	0.00	0.01	0.04	-	-	0.00	0.00
Max	0.46	0.40	0.03	0.03	0.01	0.04	0.06	0.04	0.01	0.03	0.04
Min	0.46	0.40	0.02	0.02	0.01	0.03	0.01	0.04	0.01	0.03	0.04

1/10/2024 11:19:59AM Page: 55

AmplifyBio Study Number A-579-01

Individual Animal Hematology Data

Sex: Female Day(s) Relative to Start Date

Group 1 Control Total vg 0	Hematology										
	LUC (x10^3 cells/µL)	LUC (x10^3 cells/µL)	LUC (x10^3 cells/µL)	LUC (x10^3 cells/µL)	LUC (x10^3 cells/µL)	% Neutrophils (%)	% Neutrophils (%)	% Neutrophils (%)	% Neutrophils (%)	% Neutrophils (%)	% Neutrophils (%)
	8	22	43	50	85	-41	2	8	22	43	50
151	0.03	0.06	0.02	-	-	81.5	49.3	48.2	25.4	43.0	-
152	0.02	0.04	0.04	0.06	0.03	82.5	67.7	55.1	34.1	73.1	32.8
Mean	0.03	0.05	0.03	0.06	0.03	82.0	58.5	51.7	29.8	58.1	32.8
SD	0.01	0.01	0.01	-	-	0.7	13.0	4.9	6.2	21.3	-
Max	0.03	0.06	0.04	0.06	0.03	82.5	67.7	55.1	34.1	73.1	32.8
Min	0.02	0.04	0.02	0.06	0.03	81.5	49.3	48.2	25.4	43.0	32.8

AmplifyBio Study Number A-579-01

Individual Animal Hematology Data

Sex: Female Day(s) Relative to Start Date

Group 1 Control Total vg 0	Hematology										
	% Neutrophils (%)	% Lymphocytes (%)	% Lymphocytes (%)	% Lymphocytes (%)	% Lymphocytes (%)	% Lymphocytes (%)	% Lymphocytes (%)	% Lymphocytes (%)	% Monocytes (%)	% Monocytes (%)	% Monocytes (%)
	85	-41	2	8	22	43	50	85	-41	2	8
151	-	15.2	47.0	47.1	69.0	53.0	-	-	3.00	2.70	3.60
152	27.1	13.2	26.0	36.8	53.5	21.5	55.6	62.3	3.60	3.00	4.00
Mean	27.1	14.2	36.5	42.0	61.3	37.3	55.6	62.3	3.30	2.85	3.80
SD	-	1.4	14.8	7.3	11.0	22.3	-	-	0.42	0.21	0.28
Max	27.1	15.2	47.0	47.1	69.0	53.0	55.6	62.3	3.60	3.00	4.00
Min	27.1	13.2	26.0	36.8	53.5	21.5	55.6	62.3	3.00	2.70	3.60

AmplifyBio Study Number A-579-01

Individual Animal Hematology Data

Sex: Female Day(s) Relative to Start Date

Group 1 Control Total vg 0	Hematology										
	% Monocytes (%)	% Monocytes (%)	% Monocytes (%)	% Monocytes (%)	% Eosinophils (%)	% Eosinophils (%)	% Eosinophils (%)	% Eosinophils (%)	% Eosinophils (%)	% Eosinophils (%)	% Eosinophils (%)
	22	43	50	85	-41	2	8	22	43	50	85
151	3.60	3.10	-	-	0.0	0.4	0.6	1.0	0.4	-	-
152	6.40	3.30	5.80	4.30	0.4	2.8	3.7	5.4	1.6	4.8	5.8
Mean	5.00	3.20	5.80	4.30	0.2	1.6	2.2	3.2	1.0	4.8	5.8
SD	1.98	0.14	-	-	0.3	1.7	2.2	3.1	0.8	-	-
Max	6.40	3.30	5.80	4.30	0.4	2.8	3.7	5.4	1.6	4.8	5.8
Min	3.60	3.10	5.80	4.30	0.0	0.4	0.6	1.0	0.4	4.8	5.8

AmplifyBio Study Number A-579-01

Individual Animal Hematology Data

Sex: Female Day(s) Relative to Start Date

Group 1 Control Total vg 0	Hematology										
	% BASO (%)	% BASO (%)	% BASO (%)	% BASO (%)	% BASO (%)	% BASO (%)	% BASO (%)	% LUC (%)	% LUC (%)	% LUC (%)	% LUC (%)
	-41	2	8	22	43	50	85	-41	2	8	22
151	0.1	0.2	0.1	0.4	0.2	-	-	0.2	0.3	0.4	0.6
152	0.2	0.2	0.1	0.3	0.3	0.4	0.2	0.1	0.2	0.2	0.4
Mean	0.2	0.2	0.1	0.4	0.3	0.4	0.2	0.2	0.3	0.3	0.5
SD	0.1	0.0	0.0	0.1	0.1	-	-	0.1	0.1	0.1	0.1
Max	0.2	0.2	0.1	0.4	0.3	0.4	0.2	0.2	0.3	0.4	0.6
Min	0.1	0.2	0.1	0.3	0.2	0.4	0.2	0.1	0.2	0.2	0.4

AmplifyBio Study Number A-579-01

Individual Animal Hematology Data

Sex: Female Day(s) Relative to Start Date

Group 1 Control Total vg 0	Hematology										
	% LUC (%)	% LUC (%)	% LUC (%)	Platelets (x10^3 cells/µL)	Platelets (x10^3 cells/µL)	Platelets (x10^3 cells/µL)	Platelets (x10^3 cells/µL)	Platelets (x10^3 cells/µL)	Platelets (x10^3 cells/µL)	Platelets (x10^3 cells/µL)	MPV (fL)
	43	50	85	-41	2	8	22	43	50	85	2
151	0.3	-	-	285	407	356	390	312	-	-	10.0
152	0.2	0.6	0.4	432	500	454	396 [a]	461	516	448	9.3
Mean	0.3	0.6	0.4	359	454	405	393	387	516	448	9.7
SD	0.1	-	-	104	66	69	4	105	-	-	0.5
Max	0.3	0.6	0.4	432	500	454	396	461	516	448	10.0
Min	0.2	0.6	0.4	285	407	356	390	312	516	448	9.3

[a] [RC:Enlarged Platelets.]

AmplifyBio Study Number A-579-01

Individual Animal Hematology Data

Sex: Female Day(s) Relative to Start Date

Group 1 Control Total vg 0	Hematology				
	MPV (fL)	MPV (fL)	MPV (fL)	MPV (fL)	MPV (fL)
	8	22	43	50	85
151	9.8	9.7	9.7	-	-
152	9.2	10.4	9.9	9.4	9.0
Mean	9.5	10.1	9.8	9.4	9.0
SD	0.4	0.5	0.1	-	-
Max	9.8	10.4	9.9	9.4	9.0
Min	9.2	9.7	9.7	9.4	9.0

AmplifyBio Study Number A-579-01

Individual Animal Hematology Data

Sex: Female Day(s) Relative to Start Date

Group 2 Low Total vg 6x10^13	Hematology										
	Red Blood Cells (x10^6 cells/µL)	Red Blood Cells (x10^6 cells/µL)	Red Blood Cells (x10^6 cells/µL)	Red Blood Cells (x10^6 cells/µL)	Red Blood Cells (x10^6 cells/µL)	Red Blood Cells (x10^6 cells/µL)	Red Blood Cells (x10^6 cells/µL)	Hemoglobin (g/dL)	Hemoglobin (g/dL)	Hemoglobin (g/dL)	Hemoglobin (g/dL)
	-42	2	8	22	43	50	85	-42	2	8	22
251	5.87	5.54	5.68	6.34	6.08	-	-	13.3	12.3	13.1	14.4
252	5.82	5.66	5.55	6.11	5.79	-	-	13.4	12.8	12.7	13.9
253	5.55	5.44	5.37	5.91	5.69	5.56	5.39	13.6	13.2	13.6	14.4
254	5.53	5.38	5.49	5.77	5.75	5.61	5.98	12.9	12.5	12.9	13.4
Mean	5.69	5.51	5.52	6.03	5.83	5.59	5.69	13.3	12.7	13.1	14.0
SD	0.18	0.12	0.13	0.25	0.17	0.04	0.42	0.3	0.4	0.4	0.5
Max	5.87	5.66	5.68	6.34	6.08	5.61	5.98	13.6	13.2	13.6	14.4
Min	5.53	5.38	5.37	5.77	5.69	5.56	5.39	12.9	12.3	12.7	13.4

1/10/2024 11:19:59AM Page: 62

AmplifyBio Study Number A-579-01

Individual Animal Hematology Data

Sex: Female Day(s) Relative to Start Date

Group 2 Low Total vg 6x10^13	Hematology										
	Hemoglobin (g/dL)	Hemoglobin (g/dL)	Hemoglobin (g/dL)	Hematocrit (%)	Hematocrit (%)	Hematocrit (%)	Hematocrit (%)	Hematocrit (%)	Hematocrit (%)	Hematocrit (%)	MCV (fL)
	43	50	85	-42	2	8	22	43	50	85	-42
251	13.8	-	-	43.8	42.2	42.4	47.8	45.4	-	-	74.7
252	13.2	-	-	43.8	44.1	41.3	49.1	43.2	-	-	75.3
253	13.7	13.5	13.1	44.5	42.7	41.5	47.0	44.6	44.6	42.2	80.1
254	13.4	13.0	13.7	42.4	43.6	42.1	47.5	45.4	44.9	45.5	76.8
Mean	13.5	13.3	13.4	43.6	43.2	41.8	47.9	44.7	44.8	43.9	76.7
SD	0.3	0.4	0.4	0.9	0.9	0.5	0.9	1.0	0.2	2.3	2.4
Max	13.8	13.5	13.7	44.5	44.1	42.4	49.1	45.4	44.9	45.5	80.1
Min	13.2	13.0	13.1	42.4	42.2	41.3	47.0	43.2	44.6	42.2	74.7

AmplifyBio Study Number A-579-01

Individual Animal Hematology Data

Sex: Female Day(s) Relative to Start Date

Group 2 Low Total vg 6x10^13	Hematology										
	MCV (fL)	MCV (fL)	MCV (fL)	MCV (fL)	MCV (fL)	MCV (fL)	MCH (pg)	MCH (pg)	MCH (pg)	MCH (pg)	MCH (pg)
	2	8	22	43	50	85	-42	2	8	22	43
251	76.2	74.6	75.4	74.7	-	-	22.6	22.2	23.0	22.8	22.7
252	77.9	74.4	80.4	74.7	-	-	23.0	22.7	22.8	22.7	22.8
253	78.6	77.4	79.5	78.4	80.3	78.3	24.5	24.2	25.4	24.3	24.0
254	81.1	76.8	82.4	79.1	79.9	76.1	23.3	23.2	23.5	23.3	23.3
Mean	78.5	75.8	79.4	76.7	80.1	77.2	23.4	23.1	23.7	23.3	23.2
SD	2.0	1.5	2.9	2.4	0.3	1.6	0.8	0.9	1.2	0.7	0.6
Max	81.1	77.4	82.4	79.1	80.3	78.3	24.5	24.2	25.4	24.3	24.0
Min	76.2	74.4	75.4	74.7	79.9	76.1	22.6	22.2	22.8	22.7	22.7

AmplifyBio Study Number A-579-01

Individual Animal Hematology Data

Sex: Female Day(s) Relative to Start Date

Group 2 Low Total vg 6x10^13	Hematology										
	MCH (pg)	MCH (pg)	MCHC (g/dL)	MCHC (g/dL)	MCHC (g/dL)	MCHC (g/dL)	MCHC (g/dL)	MCHC (g/dL)	MCHC (g/dL)	RETIC (x10^3 cells/µL)	RETIC (x10^3 cells/µL)
	50	85	-42	2	8	22	43	50	85	-42	2
251	-	-	30.3	29.1	30.8	30.2	30.4	-	-	61.4	115.5
252	-	-	30.6	29.1	30.6	28.2	30.5	-	-	75.5	114.7
253	24.2	24.3	30.6	30.8	32.8	30.6	30.7	30.2	31.1	99.8	104.8
254	23.1	23.0	30.4	28.6	30.6	28.2	29.5	28.9	30.2	63.1	94.7
Mean	23.7	23.7	30.5	29.4	31.2	29.3	30.3	29.6	30.7	75.0	107.4
SD	0.8	0.9	0.2	1.0	1.1	1.3	0.5	0.9	0.6	17.7	9.8
Max	24.2	24.3	30.6	30.8	32.8	30.6	30.7	30.2	31.1	99.8	115.5
Min	23.1	23.0	30.3	28.6	30.6	28.2	29.5	28.9	30.2	61.4	94.7

AmplifyBio Study Number A-579-01

Individual Animal Hematology Data

Sex: Female Day(s) Relative to Start Date

Group 2 Low Total vg 6x10^13	Hematology										
	RETIC (x10^3 cells/µL)	RETIC (x10^3 cells/µL)	RETIC (x10^3 cells/µL)	RETIC (x10^3 cells/µL)	RETIC (x10^3 cells/µL)	% Retic (%)	% Retic (%)	% Retic (%)	% Retic (%)	% Retic (%)	% Retic (%)
	8	22	43	50	85	2	8	22	43	50	85
251	99.2	65.2	40.6	-	-	2.08	1.75	1.03	0.67	-	-
252	117.1	132.2	48.3	-	-	2.03	2.11	2.16	0.83	-	-
253	98.8	73.5	73.6	106.2	46.8	1.93	1.84	1.24	1.29	1.91	0.87
254	58.3	120.5	32.2	114.9	39.8	1.76	1.06	2.09	0.56	2.05	0.67
Mean	93.4	97.9	48.7	110.6	43.3	1.95	1.69	1.63	0.84	1.98	0.77
SD	24.9	33.4	17.9	6.2	4.9	0.14	0.45	0.58	0.32	0.10	0.14
Max	117.1	132.2	73.6	114.9	46.8	2.08	2.11	2.16	1.29	2.05	0.87
Min	58.3	65.2	32.2	106.2	39.8	1.76	1.06	1.03	0.56	1.91	0.67

1/10/2024 11:19:59AM Page: 66

AmplifyBio Study Number A-579-01

Individual Animal Hematology Data

Sex: Female Day(s) Relative to Start Date

Group 2 Low Total vg 6x10^13	Hematology										
	White Blood Cells (x10^3 cells/µL)	White Blood Cells (x10^3 cells/µL)	White Blood Cells (x10^3 cells/µL)	White Blood Cells (x10^3 cells/µL)	White Blood Cells (x10^3 cells/µL)	White Blood Cells (x10^3 cells/µL)	White Blood Cells (x10^3 cells/µL)	Neutrophils (x10^3 cells/µL)	Neutrophils (x10^3 cells/µL)	Neutrophils (x10^3 cells/µL)	Neutrophils (x10^3 cells/µL)
	-42	2	8	22	43	50	85	-42	2	8	22
251	14.46	11.14	5.96	11.30	6.59	-	-	10.16	5.43	1.61	3.01
252	11.92	16.28	7.86	16.09	8.21	-	-	7.50	7.79	3.13	3.11
253	11.01 [a]	9.16	8.24	11.56	10.43	13.84	6.68	8.94	5.22	5.03	6.38
254	16.53	12.82	7.02	11.00	11.50	12.85	9.69	12.64	6.40	3.11	3.75
Mean	13.48	12.35	7.27	12.49	9.18	13.35	8.19	9.81	6.21	3.22	4.06
SD	2.50	3.02	1.01	2.41	2.21	0.70	2.13	2.18	1.17	1.40	1.58
Max	16.53	16.28	8.24	16.09	11.50	13.84	9.69	12.64	7.79	5.03	6.38
Min	11.01	9.16	5.96	11.00	6.59	12.85	6.68	7.50	5.22	1.61	3.01

[a] [RC:Morphology WNL]

AmplifyBio Study Number A-579-01

Individual Animal Hematology Data

Sex: Female Day(s) Relative to Start Date

Group 2 Low Total vg 6x10^13	Hematology										
	Neutrophils (x10^3 cells/µL)	Neutrophils (x10^3 cells/µL)	Neutrophils (x10^3 cells/µL)	Lymphocytes (x10^3 cells/µL)	Lymphocytes (x10^3 cells/µL)	Lymphocytes (x10^3 cells/µL)	Lymphocytes (x10^3 cells/µL)	Lymphocytes (x10^3 cells/µL)	Lymphocytes (x10^3 cells/µL)	Lymphocytes (x10^3 cells/µL)	Monocytes (x10^3 cells/µL)
	43	50	85	-42	2	8	22	43	50	85	-42
251	1.85	-	-	3.82	5.12	3.99	7.69	4.39	-	-	0.39
252	2.47	-	-	3.95	7.72	4.19	11.96	5.27	-	-	0.30
253	6.04	8.10	2.95	1.62 [a]	3.29	2.72	4.46	3.74	5.14	3.33	0.41
254	4.33	4.70	4.12	3.34	5.73	3.39	6.50	6.48	7.48	5.13	0.49
Mean	3.67	6.40	3.54	3.18	5.47	3.57	7.65	4.97	6.31	4.23	0.40
SD	1.90	2.40	0.83	1.07	1.83	0.66	3.17	1.19	1.65	1.27	0.08
Max	6.04	8.10	4.12	3.95	7.72	4.19	11.96	6.48	7.48	5.13	0.49
Min	1.85	4.70	2.95	1.62	3.29	2.72	4.46	3.74	5.14	3.33	0.30

[a] [RC:%LYMPH WNL]

AmplifyBio Study Number A-579-01

Individual Animal Hematology Data

Sex: Female Day(s) Relative to Start Date

Group 2 Low Total vg 6x10^13	Hematology										
	Monocytes (x10^3 cells/µL)	Monocytes (x10^3 cells/µL)	Monocytes (x10^3 cells/µL)	Monocytes (x10^3 cells/µL)	Monocytes (x10^3 cells/µL)	Monocytes (x10^3 cells/µL)	Eosinophils (x10^3 cells/µL)	Eosinophils (x10^3 cells/µL)	Eosinophils (x10^3 cells/µL)	Eosinophils (x10^3 cells/µL)	Eosinophils (x10^3 cells/µL)
	2	8	22	43	50	85	-42	2	8	22	43
251	0.45	0.29	0.40	0.21	-	-	0.05	0.06	0.04	0.13	0.10
252	0.45	0.37	0.56	0.26	-	-	0.11	0.19	0.10	0.29	0.14
253	0.56	0.40	0.61	0.57	0.48	0.34	0.01	0.03	0.06	0.05	0.04
254	0.57	0.44	0.60	0.05	0.52	0.34	0.01	0.03	0.04	0.05	0.52
Mean	0.51	0.38	0.54	0.27	0.50	0.34	0.05	0.08	0.06	0.13	0.20
SD	0.07	0.06	0.10	0.22	0.03	0.00	0.05	0.08	0.03	0.11	0.22
Max	0.57	0.44	0.61	0.57	0.52	0.34	0.11	0.19	0.10	0.29	0.52
Min	0.45	0.29	0.40	0.05	0.48	0.34	0.01	0.03	0.04	0.05	0.04

AmplifyBio Study Number A-579-01

Individual Animal Hematology Data

Sex: Female Day(s) Relative to Start Date

Group 2 Low Total vg 6x10^13	Hematology										
	Eosinophils (x10^3 cells/µL)	Eosinophils (x10^3 cells/µL)	Basophils (x10^3 cells/µL)	Basophils (x10^3 cells/µL)	Basophils (x10^3 cells/µL)	Basophils (x10^3 cells/µL)	Basophils (x10^3 cells/µL)	Basophils (x10^3 cells/µL)	Basophils (x10^3 cells/µL)	LUC (x10^3 cells/µL)	LUC (x10^3 cells/µL)
	50	85	-42	2	8	22	43	50	85	-42	2
251	-	-	0.02	0.03	0.01	0.02	0.01	-	-	0.03	0.05
252	-	-	0.02	0.05	0.02	0.07	0.02	-	-	0.04	0.08
253	0.07	0.04	0.00	0.02	0.01	0.01	0.01	0.02	0.01	0.02	0.04
254	0.06	0.06	0.02	0.03	0.01	0.05	0.03	0.03	0.00	0.03	0.07
Mean	0.07	0.05	0.02	0.03	0.01	0.04	0.02	0.03	0.01	0.03	0.06
SD	0.01	0.01	0.01	0.01	0.01	0.03	0.01	0.01	0.01	0.01	0.02
Max	0.07	0.06	0.02	0.05	0.02	0.07	0.03	0.03	0.01	0.04	0.08
Min	0.06	0.04	0.00	0.02	0.01	0.01	0.01	0.02	0.00	0.02	0.04

AmplifyBio Study Number A-579-01

Individual Animal Hematology Data

Sex: Female Day(s) Relative to Start Date

Group 2 Low Total vg 6x10^13	Hematology										
	LUC (x10^3 cells/µL)	LUC (x10^3 cells/µL)	LUC (x10^3 cells/µL)	LUC (x10^3 cells/µL)	LUC (x10^3 cells/µL)	% Neutrophils (%)	% Neutrophils (%)	% Neutrophils (%)	% Neutrophils (%)	% Neutrophils (%)	% Neutrophils (%)
	8	22	43	50	85	-42	2	8	22	43	50
251	0.02	0.05	0.01	-	-	70.3	48.7	27.0	26.7	28.1	-
252	0.05	0.10	0.05	-	-	62.9	47.9	39.8	19.3	30.1	-
253	0.02	0.04	0.02	0.02	0.02	81.2	56.9	61.1	55.2	57.9	58.6
254	0.03	0.04	0.10	0.06	0.03	76.5	49.9	44.3	34.1	37.7	36.6
Mean	0.03	0.06	0.05	0.04	0.03	72.7	50.9	43.1	33.8	38.5	47.6
SD	0.01	0.03	0.04	0.03	0.01	7.9	4.1	14.1	15.5	13.6	15.6
Max	0.05	0.10	0.10	0.06	0.03	81.2	56.9	61.1	55.2	57.9	58.6
Min	0.02	0.04	0.01	0.02	0.02	62.9	47.9	27.0	19.3	28.1	36.6

AmplifyBio Study Number A-579-01

Individual Animal Hematology Data

Sex: Female Day(s) Relative to Start Date

Group 2 Low Total vg 6x10^13	Hematology										
	% Neutrophils (%)	% Lymphocytes (%)	% Lymphocytes (%)	% Lymphocytes (%)	% Lymphocytes (%)	% Lymphocytes (%)	% Lymphocytes (%)	% Lymphocytes (%)	% Monocytes (%)	% Monocytes (%)	% Monocytes (%)
	85	-42	2	8	22	43	50	85	-42	2	8
251	-	26.4	45.9	67.0	68.1	66.7	-	-	2.70	4.10	4.80
252	-	33.2	47.4	53.3	74.3	64.2	-	-	2.50	2.70	4.70
253	44.2	14.7	35.9	33.0	38.6	35.9	37.2	49.9	3.80	6.10	4.90
254	42.5	20.2	44.7	48.3	59.1	56.3	58.2	53.0	3.00	4.40	6.30
Mean	43.4	23.6	43.5	50.4	60.0	55.8	47.7	51.5	3.00	4.33	5.18
SD	1.2	8.0	5.2	14.0	15.6	14.0	14.8	2.2	0.57	1.40	0.75
Max	44.2	33.2	47.4	67.0	74.3	66.7	58.2	53.0	3.80	6.10	6.30
Min	42.5	14.7	35.9	33.0	38.6	35.9	37.2	49.9	2.50	2.70	4.70

AmplifyBio Study Number A-579-01

Individual Animal Hematology Data

Sex: Female Day(s) Relative to Start Date

Group 2 Low Total vg 6x10^13	Hematology										
	% Monocytes (%)	% Monocytes (%)	% Monocytes (%)	% Monocytes (%)	% Eosinophils (%)	% Eosinophils (%)	% Eosinophils (%)	% Eosinophils (%)	% Eosinophils (%)	% Eosinophils (%)	% Eosinophils (%)
	22	43	50	85	-42	2	8	22	43	50	85
251	3.50	3.20	-	-	0.3	0.6	0.7	1.1	1.6	-	-
252	3.50	3.20	-	-	0.9	1.2	1.3	1.8	1.7	-	-
253	5.30	5.50	3.50	5.00	0.1	0.4	0.7	0.4	0.3	0.5	0.6
254	5.40	0.40	4.00	3.50	0.0	0.3	0.5	0.5	4.5	0.4	0.7
Mean	4.43	3.08	3.75	4.25	0.3	0.6	0.8	1.0	2.0	0.5	0.7
SD	1.07	2.09	0.35	1.06	0.4	0.4	0.3	0.6	1.8	0.1	0.1
Max	5.40	5.50	4.00	5.00	0.9	1.2	1.3	1.8	4.5	0.5	0.7
Min	3.50	0.40	3.50	3.50	0.0	0.3	0.5	0.4	0.3	0.4	0.6

AmplifyBio Study Number A-579-01

Individual Animal Hematology Data

Sex: Female Day(s) Relative to Start Date

Group 2 Low Total vg 6x10^13	Hematology										
	% BASO (%)	% BASO (%)	% BASO (%)	% BASO (%)	% BASO (%)	% BASO (%)	% BASO (%)	% LUC (%)	% LUC (%)	% LUC (%)	% LUC (%)
	-42	2	8	22	43	50	85	-42	2	8	22
251	0.2	0.3	0.1	0.2	0.2	-	-	0.2	0.4	0.4	0.4
252	0.1	0.3	0.2	0.4	0.3	-	-	0.4	0.5	0.6	0.6
253	0.0	0.2	0.1	0.1	0.1	0.2	0.1	0.2	0.5	0.2	0.3
254	0.1	0.2	0.1	0.4	0.2	0.2	0.1	0.2	0.5	0.4	0.4
Mean	0.1	0.3	0.1	0.3	0.2	0.2	0.1	0.3	0.5	0.4	0.4
SD	0.1	0.1	0.1	0.2	0.1	0.0	0.0	0.1	0.1	0.2	0.1
Max	0.2	0.3	0.2	0.4	0.3	0.2	0.1	0.4	0.5	0.6	0.6
Min	0.0	0.2	0.1	0.1	0.1	0.2	0.1	0.2	0.4	0.2	0.3

AmplifyBio Study Number A-579-01

Individual Animal Hematology Data

Sex: Female Day(s) Relative to Start Date

Group 2 Low Total vg 6x10^13	Hematology										
	% LUC (%)	% LUC (%)	% LUC (%)	Platelets (x10^3 cells/µL)	Platelets (x10^3 cells/µL)	Platelets (x10^3 cells/µL)	Platelets (x10^3 cells/µL)	Platelets (x10^3 cells/µL)	Platelets (x10^3 cells/µL)	Platelets (x10^3 cells/µL)	MPV (fL)
	43	50	85	-42	2	8	22	43	50	85	2
251	0.2	-	-	371	392	436	392	302	-	-	9.4
252	0.6	-	-	413	521	530	484	369	-	-	9.8
253	0.2	0.1	0.2	440	469 [a]	508	434 [b]	393 [a]	431	419	9.7
254	0.8	0.5	0.3	415	426 [a]	426	415	321	472 [a]	410	10.6
Mean	0.5	0.3	0.3	410	452	475	431	346	452	415	9.9
SD	0.3	0.3	0.1	29	56	52	39	42	29	6	0.5
Max	0.8	0.5	0.3	440	521	530	484	393	472	419	10.6
Min	0.2	0.1	0.2	371	392	426	392	302	431	410	9.4

[a] [RC:Clumping present]
[b] [RC:Clumping present.]

AmplifyBio Study Number A-579-01

Individual Animal Hematology Data

Sex: Female Day(s) Relative to Start Date

Group 2 Low Total vg 6x10^13	Hematology				
	MPV (fL)	MPV (fL)	MPV (fL)	MPV (fL)	MPV (fL)
	8	22	43	50	85
251	9.3	9.5	9.1	-	-
252	9.2	10.1	9.3	-	-
253	9.6	10.0	9.9	9.6	9.3
254	10.2	11.0	10.4	10.6	10.2
Mean	9.6	10.2	9.7	10.1	9.8
SD	0.5	0.6	0.6	0.7	0.6
Max	10.2	11.0	10.4	10.6	10.2
Min	9.2	9.5	9.1	9.6	9.3

AmplifyBio Study Number A-579-01

Individual Animal Hematology Data

Sex: Female Day(s) Relative to Start Date

Group 3 High Total vg 1.2x10^14	Hematology										
	Red Blood Cells (x10^6 cells/µL)	Red Blood Cells (x10^6 cells/µL)	Red Blood Cells (x10^6 cells/µL)	Red Blood Cells (x10^6 cells/µL)	Red Blood Cells (x10^6 cells/µL)	Red Blood Cells (x10^6 cells/µL)	Red Blood Cells (x10^6 cells/µL)	Hemoglobin (g/dL)	Hemoglobin (g/dL)	Hemoglobin (g/dL)	Hemoglobin (g/dL)
	-43	2	8	22	43	50	85	-43	2	8	22
351	5.19	4.86	5.12	5.74	5.25	-	-	12.0	11.1	11.7	12.6
352	5.65	5.60	5.35	5.94	5.88	-	-	12.7	12.4	12.1	13.1
353	5.63	5.45	5.11	5.64	5.52	5.46	5.37	13.9	13.1	12.4	13.6
354	6.01	5.67	5.50	5.81	6.03	5.74	5.88	14.8	13.7	13.5	14.3
Mean	5.62	5.40	5.27	5.78	5.67	5.60	5.63	13.4	12.6	12.4	13.4
SD	0.34	0.37	0.19	0.13	0.35	0.20	0.36	1.2	1.1	0.8	0.7
Max	6.01	5.67	5.50	5.94	6.03	5.74	5.88	14.8	13.7	13.5	14.3
Min	5.19	4.86	5.11	5.64	5.25	5.46	5.37	12.0	11.1	11.7	12.6

AmplifyBio Study Number A-579-01

Individual Animal Hematology Data

Sex: Female Day(s) Relative to Start Date

Group 3 High Total vg 1.2x10^14	Hematology										
	Hemoglobin (g/dL)	Hemoglobin (g/dL)	Hemoglobin (g/dL)	Hematocrit (%)	Hematocrit (%)	Hematocrit (%)	Hematocrit (%)	Hematocrit (%)	Hematocrit (%)	Hematocrit (%)	MCV (fL)
	43	50	85	-43	2	8	22	43	50	85	-43
351	11.9	-	-	39.8	36.5	37.9	42.5	38.9	-	-	76.7
352	13.0	-	-	40.1	40.9	38.1	41.8	41.2	-	-	71.0
353	13.3	13.2	12.4	44.1	43.9	39.8	44.7	43.1	43.0	40.6	78.3
354	14.6	14.2	14.3	46.5	45.1	42.1	45.8	47.1	45.0	45.0	77.3
Mean	13.2	13.7	13.4	42.6	41.6	39.5	43.7	42.6	44.0	42.8	75.8
SD	1.1	0.7	1.3	3.2	3.8	1.9	1.9	3.5	1.4	3.1	3.3
Max	14.6	14.2	14.3	46.5	45.1	42.1	45.8	47.1	45.0	45.0	78.3
Min	11.9	13.2	12.4	39.8	36.5	37.9	41.8	38.9	43.0	40.6	71.0

AmplifyBio Study Number A-579-01

Individual Animal Hematology Data

Sex: Female Day(s) Relative to Start Date

Group 3 High Total vg 1.2x10^14	Hematology										
	MCV (fL)	MCV (fL)	MCV (fL)	MCV (fL)	MCV (fL)	MCV (fL)	MCH (pg)	MCH (pg)	MCH (pg)	MCH (pg)	MCH (pg)
	2	8	22	43	50	85	-43	2	8	22	43
351	75.1	74.0	74.1	74.1	-	-	23.2	22.9	22.8	22.0	22.6
352	73.0	71.1	70.5	70.2	-	-	22.4	22.2	22.5	22.1	22.2
353	80.6	77.9	79.3	78.0	78.8	75.7	24.6	24.0	24.2	24.1	24.1
354	79.5	76.5	78.8	78.1	78.5	76.6	24.7	24.1	24.5	24.5	24.2
Mean	77.1	74.9	75.7	75.1	78.7	76.2	23.7	23.3	23.5	23.2	23.3
SD	3.6	3.0	4.2	3.8	0.2	0.6	1.1	0.9	1.0	1.3	1.0
Max	80.6	77.9	79.3	78.1	78.8	76.6	24.7	24.1	24.5	24.5	24.2
Min	73.0	71.1	70.5	70.2	78.5	75.7	22.4	22.2	22.5	22.0	22.2

AmplifyBio Study Number A-579-01

Individual Animal Hematology Data

Sex: Female Day(s) Relative to Start Date

Group 3 High Total vg 1.2x10^14	Hematology										
	MCH (pg)	MCH (pg)	MCHC (g/dL)	MCHC (g/dL)	MCHC (g/dL)	MCHC (g/dL)	MCHC (g/dL)	MCHC (g/dL)	MCHC (g/dL)	RETIC (x10^3 cells/µL)	RETIC (x10^3 cells/µL)
	50	85	-43	2	8	22	43	50	85	-43	2
351	-	-	30.2	30.4	30.8	29.7	30.5	-	-	63.5	62.6
352	-	-	31.6	30.5	31.6	31.4	31.6	-	-	53.7	76.5
353	24.1	23.0	31.4	29.8	31.1	30.4	30.8	30.6	30.4	45.6	53.1
354	24.7	24.4	31.9	30.3	32.0	31.1	31.0	31.4	31.9	47.0	115.5
Mean	24.4	23.7	31.3	30.3	31.4	30.7	31.0	31.0	31.2	52.5	76.9
SD	0.4	1.0	0.7	0.3	0.5	0.8	0.5	0.6	1.1	8.2	27.5
Max	24.7	24.4	31.9	30.5	32.0	31.4	31.6	31.4	31.9	63.5	115.5
Min	24.1	23.0	30.2	29.8	30.8	29.7	30.5	30.6	30.4	45.6	53.1

AmplifyBio Study Number A-579-01

Individual Animal Hematology Data

Sex: Female Day(s) Relative to Start Date

Group 3 High Total vg 1.2x10^14	Hematology										
	RETIC (x10^3 cells/µL)	RETIC (x10^3 cells/µL)	RETIC (x10^3 cells/µL)	RETIC (x10^3 cells/µL)	RETIC (x10^3 cells/µL)	% Retic (%)	% Retic (%)	% Retic (%)	% Retic (%)	% Retic (%)	% Retic (%)
	8	22	43	50	85	2	8	22	43	50	85
351	51.5	46.6	28.5	-	-	1.29	1.00	0.81	0.54	-	-
352	56.9	61.8	41.7	-	-	1.37	1.06	1.04	0.71	-	-
353	85.4	119.3	50.1	61.5	19.8	0.97	1.67	2.12	0.91	1.13	0.37
354	89.0	130.6	71.5	134.6	67.6	2.04	1.62	2.25	1.19	2.35	1.15
Mean	70.7	89.6	48.0	98.1	43.7	1.42	1.34	1.56	0.84	1.74	0.76
SD	19.2	41.6	18.0	51.7	33.8	0.45	0.36	0.74	0.28	0.86	0.55
Max	89.0	130.6	71.5	134.6	67.6	2.04	1.67	2.25	1.19	2.35	1.15
Min	51.5	46.6	28.5	61.5	19.8	0.97	1.00	0.81	0.54	1.13	0.37

AmplifyBio Study Number A-579-01

Individual Animal Hematology Data

Sex: Female Day(s) Relative to Start Date

Group 3 High Total vg 1.2x10^14	Hematology										
	White Blood Cells (x10^3 cells/µL)	White Blood Cells (x10^3 cells/µL)	White Blood Cells (x10^3 cells/µL)	White Blood Cells (x10^3 cells/µL)	White Blood Cells (x10^3 cells/µL)	White Blood Cells (x10^3 cells/µL)	White Blood Cells (x10^3 cells/µL)	Neutrophils (x10^3 cells/µL)	Neutrophils (x10^3 cells/µL)	Neutrophils (x10^3 cells/µL)	Neutrophils (x10^3 cells/µL)
	-43	2	8	22	43	50	85	-43	2	8	22
351	8.39	8.69	8.63	10.72	5.57	-	-	5.74	3.83	3.85	2.71
352	10.69 [a]	8.54	4.23	8.32	6.13	-	-	9.00	4.89	2.04	3.55
353	11.97 [a]	6.32	11.59	16.49	8.88	15.52	7.59	9.84	2.25	7.50	12.04
354	7.63	8.21	7.53	8.94	6.36	7.66	7.61	4.73	3.16	4.58	2.97
Mean	9.67	7.94	8.00	11.12	6.74	11.59	7.60	7.33	3.53	4.49	5.32
SD	2.01	1.10	3.04	3.72	1.47	5.56	0.01	2.48	1.11	2.27	4.50
Max	11.97	8.69	11.59	16.49	8.88	15.52	7.61	9.84	4.89	7.50	12.04
Min	7.63	6.32	4.23	8.32	5.57	7.66	7.59	4.73	2.25	2.04	2.71

[a] [RC:Morphology WNL]

AmplifyBio Study Number A-579-01

Individual Animal Hematology Data

Sex: Female Day(s) Relative to Start Date

Group 3 High Total vg 1.2x10^14	Hematology										
	Neutrophils (x10^3 cells/µL)	Neutrophils (x10^3 cells/µL)	Neutrophils (x10^3 cells/µL)	Lymphocytes (x10^3 cells/µL)	Lymphocytes (x10^3 cells/µL)	Lymphocytes (x10^3 cells/µL)	Lymphocytes (x10^3 cells/µL)	Lymphocytes (x10^3 cells/µL)	Lymphocytes (x10^3 cells/µL)	Lymphocytes (x10^3 cells/µL)	Monocytes (x10^3 cells/µL)
	43	50	85	-43	2	8	22	43	50	85	-43
351	1.70	-	-	2.38	4.38	4.02	7.23	3.56	-	-	0.21
352	3.02	-	-	1.29 [a]	3.28	1.72	4.39	2.75	-	-	0.34
353	6.06	11.84	4.48	1.73 [a]	3.60	3.54	3.43	2.18	3.02	2.53	0.36
354	1.41	1.60	2.85	2.47	4.57	2.55	5.13	4.46	5.48	4.25	0.38
Mean	3.05	6.72	3.67	1.97	3.96	2.96	5.05	3.24	4.25	3.39	0.32
SD	2.13	7.24	1.15	0.56	0.62	1.03	1.61	0.99	1.74	1.22	0.08
Max	6.06	11.84	4.48	2.47	4.57	4.02	7.23	4.46	5.48	4.25	0.38
Min	1.41	1.60	2.85	1.29	3.28	1.72	3.43	2.18	3.02	2.53	0.21

[a] [RC:%LYMPH WNL]

AmplifyBio Study Number A-579-01

Individual Animal Hematology Data

Sex: Female Day(s) Relative to Start Date

Group 3 High Total vg 1.2x10^14	Hematology										
	Monocytes (x10^3 cells/µL)	Monocytes (x10^3 cells/µL)	Monocytes (x10^3 cells/µL)	Monocytes (x10^3 cells/µL)	Monocytes (x10^3 cells/µL)	Monocytes (x10^3 cells/µL)	Eosinophils (x10^3 cells/µL)	Eosinophils (x10^3 cells/µL)	Eosinophils (x10^3 cells/µL)	Eosinophils (x10^3 cells/µL)	Eosinophils (x10^3 cells/µL)
	2	8	22	43	50	85	-43	2	8	22	43
351	0.36	0.58	0.63	0.20	-	-	0.03	0.05	0.05	0.05	0.05
352	0.26	0.37	0.26	0.24	-	-	0.02	0.07	0.02	0.08	0.08
353	0.37	0.46	0.84	0.58	0.58	0.41	0.00	0.02	0.02	0.12	0.04
354	0.38	0.31	0.59	0.36	0.44	0.35	0.02	0.05	0.04	0.15	0.08
Mean	0.34	0.43	0.58	0.35	0.51	0.38	0.02	0.05	0.03	0.10	0.06
SD	0.06	0.12	0.24	0.17	0.10	0.04	0.01	0.02	0.02	0.04	0.02
Max	0.38	0.58	0.84	0.58	0.58	0.41	0.03	0.07	0.05	0.15	0.08
Min	0.26	0.31	0.26	0.20	0.44	0.35	0.00	0.02	0.02	0.05	0.04

AmplifyBio Study Number A-579-01

Individual Animal Hematology Data

Sex: Female Day(s) Relative to Start Date

Group 3 High Total vg 1.2x10^14	Hematology										
	Eosinophils (x10^3 cells/µL)	Eosinophils (x10^3 cells/µL)	Basophils (x10^3 cells/µL)	Basophils (x10^3 cells/µL)	Basophils (x10^3 cells/µL)	Basophils (x10^3 cells/µL)	Basophils (x10^3 cells/µL)	Basophils (x10^3 cells/µL)	Basophils (x10^3 cells/µL)	LUC (x10^3 cells/µL)	LUC (x10^3 cells/µL)
	50	85	-43	2	8	22	43	50	85	-43	2
351	-	-	0.01	0.03	0.04	0.02	0.01	-	-	0.02	0.05
352	-	-	0.01	0.01	0.02	0.01	0.01	-	-	0.02	0.03
353	0.04	0.12	0.02	0.02	0.03	0.03	0.01	0.03	0.01	0.01	0.06
354	0.10	0.09	0.01	0.01	0.01	0.03	0.01	0.01	0.02	0.02	0.04
Mean	0.07	0.11	0.01	0.02	0.03	0.02	0.01	0.02	0.02	0.02	0.05
SD	0.04	0.02	0.01	0.01	0.01	0.01	0.00	0.01	0.01	0.00	0.01
Max	0.10	0.12	0.02	0.03	0.04	0.03	0.01	0.03	0.02	0.02	0.06
Min	0.04	0.09	0.01	0.01	0.01	0.01	0.01	0.01	0.01	0.01	0.03

AmplifyBio Study Number A-579-01

Individual Animal Hematology Data

Sex: Female Day(s) Relative to Start Date

Group 3 High Total vg 1.2x10^14	Hematology										
	LUC (x10^3 cells/µL)	LUC (x10^3 cells/µL)	LUC (x10^3 cells/µL)	LUC (x10^3 cells/µL)	LUC (x10^3 cells/µL)	% Neutrophils (%)	% Neutrophils (%)	% Neutrophils (%)	% Neutrophils (%)	% Neutrophils (%)	% Neutrophils (%)
	8	22	43	50	85	-43	2	8	22	43	50
351	0.10	0.09	0.05	-	-	68.4	44.1	44.6	25.3	30.5	-
352	0.08	0.03	0.03	-	-	84.2	57.3	48.1	42.7	49.3	-
353	0.03	0.03	0.02	0.02	0.04	82.2	35.7	64.8	73.0	68.2	76.3
354	0.03	0.06	0.03	0.04	0.04	62.0	38.5	60.9	33.3	22.1	20.8
Mean	0.06	0.05	0.03	0.03	0.04	74.2	43.9	54.6	43.6	42.5	48.6
SD	0.04	0.03	0.01	0.01	0.00	10.7	9.6	9.8	20.9	20.5	39.2
Max	0.10	0.09	0.05	0.04	0.04	84.2	57.3	64.8	73.0	68.2	76.3
Min	0.03	0.03	0.02	0.02	0.04	62.0	35.7	44.6	25.3	22.1	20.8

AmplifyBio Study Number A-579-01

Individual Animal Hematology Data

Sex: Female Day(s) Relative to Start Date

Group 3 High Total vg 1.2x10^14	Hematology										
	% Neutrophils (%)	% Lymphocytes (%)	% Lymphocytes (%)	% Lymphocytes (%)	% Lymphocytes (%)	% Lymphocytes (%)	% Lymphocytes (%)	% Lymphocytes (%)	% Monocytes (%)	% Monocytes (%)	% Monocytes (%)
	85	-43	2	8	22	43	50	85	-43	2	8
351	-	28.4	50.4	46.5	67.4	63.9	-	-	2.50	4.10	6.70
352	-	12.1	38.4	40.5	52.8	44.9	-	-	3.20	3.10	8.70
353	59.1	14.5	56.9	30.5	20.8	24.5	19.4	33.4	3.00	5.90	4.00
354	37.5	32.3	55.6	33.8	57.5	70.1	71.5	55.9	4.90	4.70	4.10
Mean	48.3	21.8	50.3	37.8	49.6	50.9	45.5	44.7	3.40	4.45	5.88
SD	15.3	10.0	8.4	7.1	20.2	20.6	36.8	15.9	1.04	1.17	2.26
Max	59.1	32.3	56.9	46.5	67.4	70.1	71.5	55.9	4.90	5.90	8.70
Min	37.5	12.1	38.4	30.5	20.8	24.5	19.4	33.4	2.50	3.10	4.00

AmplifyBio Study Number A-579-01

Individual Animal Hematology Data

Sex: Female Day(s) Relative to Start Date

Group 3 High Total vg 1.2x10^14	Hematology										
	% Monocytes (%)	% Monocytes (%)	% Monocytes (%)	% Monocytes (%)	% Eosinophils (%)	% Eosinophils (%)	% Eosinophils (%)	% Eosinophils (%)	% Eosinophils (%)	% Eosinophils (%)	% Eosinophils (%)
	22	43	50	85	-43	2	8	22	43	50	85
351	5.90	3.60	-	-	0.3	0.6	0.6	0.5	0.9	-	-
352	3.10	4.00	-	-	0.2	0.8	0.5	1.0	1.2	-	-
353	5.10	6.60	3.70	5.40	0.0	0.3	0.2	0.7	0.4	0.2	1.6
354	6.60	5.70	5.70	4.70	0.3	0.6	0.6	1.7	1.3	1.2	1.2
Mean	5.18	4.98	4.70	5.05	0.2	0.6	0.5	1.0	1.0	0.7	1.4
SD	1.51	1.42	1.41	0.49	0.1	0.2	0.2	0.5	0.4	0.7	0.3
Max	6.60	6.60	5.70	5.40	0.3	0.8	0.6	1.7	1.3	1.2	1.6
Min	3.10	3.60	3.70	4.70	0.0	0.3	0.2	0.5	0.4	0.2	1.2

1/10/2024 11:19:59AM Page: 88

AmplifyBio Study Number A-579-01

Individual Animal Hematology Data

Sex: Female Day(s) Relative to Start Date

Group 3 High Total vg 1.2x10^14	Hematology										
	% BASO (%)	% BASO (%)	% BASO (%)	% BASO (%)	% BASO (%)	% BASO (%)	% BASO (%)	% LUC (%)	% LUC (%)	% LUC (%)	% LUC (%)
	-43	2	8	22	43	50	85	-43	2	8	22
351	0.1	0.3	0.4	0.2	0.3	-	-	0.3	0.5	1.2	0.8
352	0.1	0.1	0.4	0.2	0.2	-	-	0.2	0.3	1.8	0.3
353	0.2	0.3	0.3	0.2	0.1	0.2	0.1	0.1	0.9	0.2	0.2
354	0.2	0.2	0.2	0.3	0.2	0.2	0.2	0.3	0.4	0.4	0.7
Mean	0.2	0.2	0.3	0.2	0.2	0.2	0.2	0.2	0.5	0.9	0.5
SD	0.1	0.1	0.1	0.1	0.1	0.0	0.1	0.1	0.3	0.7	0.3
Max	0.2	0.3	0.4	0.3	0.3	0.2	0.2	0.3	0.9	1.8	0.8
Min	0.1	0.1	0.2	0.2	0.1	0.2	0.1	0.1	0.3	0.2	0.2

AmplifyBio Study Number A-579-01

Individual Animal Hematology Data

Sex: Female Day(s) Relative to Start Date

Group 3 High Total vg 1.2x10^14	Hematology										
	% LUC (%)	% LUC (%)	% LUC (%)	Platelets (x10^3 cells/µL)	Platelets (x10^3 cells/µL)	Platelets (x10^3 cells/µL)	Platelets (x10^3 cells/µL)	Platelets (x10^3 cells/µL)	Platelets (x10^3 cells/µL)	Platelets (x10^3 cells/µL)	MPV (fL)
	43	50	85	-43	2	8	22	43	50	85	2
351	0.8	-	-	351	338	329	307	311	-	-	10.1
352	0.4	-	-	429	411	321	351	353	-	-	9.6
353	0.2	0.1	0.5	233	193	384	210	355	333	296	11.6
354	0.5	0.5	0.6	308	273	292	276	308	287	206	11.1
Mean	0.5	0.3	0.6	330	304	332	286	332	310	251	10.6
SD	0.3	0.3	0.1	82	93	38	59	26	33	64	0.9
Max	0.8	0.5	0.6	429	411	384	351	355	333	296	11.6
Min	0.2	0.1	0.5	233	193	292	210	308	287	206	9.6

AmplifyBio Study Number A-579-01

Individual Animal Hematology Data

Sex: Female Day(s) Relative to Start Date

Group 3 High Total vg 1.2x10^14	Hematology				
	MPV (fL)	MPV (fL)	MPV (fL)	MPV (fL)	MPV (fL)
	8	22	43	50	85
351	10.0	11.2	10.5	-	-
352	9.1	10.0	9.6	-	-
353	9.5	11.4	10.0	10.7	10.0
354	10.7	11.3	10.9	10.8	11.5
Mean	9.8	11.0	10.3	10.8	10.8
SD	0.7	0.7	0.6	0.1	1.1
Max	10.7	11.4	10.9	10.8	11.5
Min	9.1	10.0	9.6	10.7	10.0

AmplifyBio Study Number A-579-01

Individual Animal Hematology Data

Comments and Markers

Page	Day	Group	Sex	Subject	Measurement	Type	Marker
6	2	1	Male	101	WBC	Result	
				Comment: Morphology WNL			
10	43	1	Male	101	% NEUT	Result	
				Comment: Number decreased confirmed.			
14	2	1	Male	101	PLT	Result	
				Comment: Morphology WNL			
14	22	1	Male	101	PLT	Result	
				Comment: Number increased confirmed.			
20	8	2	Male	204	% Retics	Result	
				Comment: Morphology WNL			
21	22	2	Male	201	WBC	Result	
				Comment: NEUT Number decreased confirmed.			
21	22	2	Male	201	NEUT	Result	
				Comment: Number decreased confirmed.			
25	22	2	Male	201	% NEUT	Result	
				Comment: Number decreased confirmed.			
29	2	2	Male	201	PLT	Result	
				Comment: Clumping present			
29	8	2	Male	201	PLT	Result	
				Comment: Clumping present			
29	22	2	Male	201	PLT	Result	
				Comment: Clumping present			
29	22	2	Male	204	PLT	Result	
				Comment: Clumping present			
29	43	2	Male	201	PLT	Result	
				Comment: Clumping present			
29	43	2	Male	204	PLT	Result	
				Comment: Clumping present			

AmplifyBio Study Number A-579-01

Individual Animal Hematology Data

Comments and Markers

Page	Day	Group	Sex	Subject	Measurement	Type	Marker
29	50	2	Male	204	PLT	Result	
				Comment: Clumping present			
44	43	3	Male	303	PLT	Result	
				Comment: Clumping present			
44	50	3	Male	303	PLT	Result	
				Comment: Morphology WNL			
44	85	3	Male	303	PLT	Result	
				Comment: Morphology WNL			
51	-41	1	Female	152	WBC	Result	
				Comment: Morphology WNL			
52	-41	1	Female	152	LYMPH	Result	
				Comment: %LYMPH WNL			
59	22	1	Female	152	PLT	Result	
				Comment: Enlarged Platelets.			
66	-42	2	Female	253	WBC	Result	
				Comment: Morphology WNL			
67	-42	2	Female	253	LYMPH	Result	
				Comment: %LYMPH WNL			
74	2	2	Female	253	PLT	Result	
				Comment: Clumping present			
74	2	2	Female	254	PLT	Result	
				Comment: Clumping present			
74	22	2	Female	253	PLT	Result	
				Comment: Clumping present.			
74	43	2	Female	253	PLT	Result	
				Comment: Clumping present			
74	50	2	Female	254	PLT	Result	
				Comment: Clumping Present			

AmplifyBio Study Number A-579-01

Individual Animal Hematology Data

Comments and Markers

Page	Day	Group	Sex	Subject	Measurement	Type	Marker
81	-43	3	Female	352	WBC	Result	
				Comment: Morphology WNL			
81	-43	3	Female	353	WBC	Result	
				Comment: Morphology WNL			
82	-43	3	Female	352	LYMPH	Result	
				Comment: %LYMPH WNL			
82	-43	3	Female	353	LYMPH	Result	
				Comment: %LYMPH WNL			

AmplifyBio Study Number A-579-01

Individual Animal Hematology Data

Key Page

Measurement Descriptions

Headings Used	Description
Hematology - Red Blood Cells	Red Blood Cells
Hematology - Hemoglobin	Hemoglobin
Hematology - Hematocrit	Hematocrit
Hematology - MCV	Mean Corpuscular Volume
Hematology - MCH	Mean Corpuscular Hemoglobin
Hematology - MCHC	Mean Corpuscular HGB Concentration
Hematology - RETIC	Reticulocytes
Hematology - % Retic	% Reticulocytes
Hematology - White Blood Cells	White Blood Cells
Hematology - Neutrophils	Neutrophils
Hematology - Lymphocytes	Lymphocytes
Hematology - Monocytes	Monocytes
Hematology - Eosinophils	Eosinophils
Hematology - Basophils	Basophils
Hematology - LUC	Large Unstained Cells
Hematology - % Neutrophils	% Neutrophils
Hematology - % Lymphocytes	% Lymphocytes
Hematology - % Monocytes	% Monocytes
Hematology - % Eosinophils	% Eosinophils
Hematology - % BASO	% Basophils
Hematology - % LUC	% Large Unstained Cells
Hematology - Platelets	Platelets
Hematology - MPV	Mean Platelet Volume

1/10/2024 11:19:59AM Page: 95

AmplifyBio Study Number A-579-01

Individual Animal Hematology Data

Key Page

Unit Descriptions

Headings Used	Description
%	%
fL	fL
g/dL	g/dL
pg	pg
x10^3 cells/µL	x10^3 cells/µL
x10^6 cells/µL	x10^6 cells/µL

Measurement/Statistics

Measurement	Descriptive
Hematology - Red Blood Cells	Mean
	Standard Deviation
	Maximum
	Minimum
Hematology - Hemoglobin	Mean
	Standard Deviation
	Maximum
	Minimum
Hematology - Hematocrit	Mean
	Standard Deviation
	Maximum
	Minimum
Hematology - MCV	Mean
	Standard Deviation
	Maximum
	Minimum

AmplifyBio Study Number A-579-01

Individual Animal Hematology Data

Key Page

Measurement/Statistics (Continued)

Measurement	Descriptive
Hematology - MCH	Mean
	Standard Deviation
	Maximum
	Minimum
Hematology - MCHC	Mean
	Standard Deviation
	Maximum
	Minimum
Hematology - RETIC	Mean
	Standard Deviation
	Maximum
	Minimum
Hematology - % Retic	Mean
	Standard Deviation
	Maximum
	Minimum
Hematology - White Blood Cells	Mean
	Standard Deviation
	Maximum
	Minimum
Hematology - Neutrophils	Mean
	Standard Deviation
	Maximum
	Minimum

AmplifyBio Study Number A-579-01

Individual Animal Hematology Data

Key Page

Measurement/Statistics (Continued)

Measurement	Descriptive
Hematology - Lymphocytes	Mean
	Standard Deviation
	Maximum
	Minimum
Hematology - Monocytes	Mean
	Standard Deviation
	Maximum
	Minimum
Hematology - Eosinophils	Mean
	Standard Deviation
	Maximum
	Minimum
Hematology - Basophils	Mean
	Standard Deviation
	Maximum
	Minimum
Hematology - LUC	Mean
	Standard Deviation
	Maximum
	Minimum
Hematology - % Neutrophils	Mean
	Standard Deviation
	Maximum
	Minimum

AmplifyBio Study Number A-579-01

Individual Animal Hematology Data

Key Page

Measurement/Statistics (Continued)

Measurement	Descriptive
Hematology - % Lymphocytes	Mean
	Standard Deviation
	Maximum
	Minimum
Hematology - % Monocytes	Mean
	Standard Deviation
	Maximum
	Minimum
Hematology - % Eosinophils	Mean
	Standard Deviation
	Maximum
	Minimum
Hematology - % BASO	Mean
	Standard Deviation
	Maximum
	Minimum
Hematology - % LUC	Mean
	Standard Deviation
	Maximum
	Minimum
Hematology - Platelets	Mean
	Standard Deviation
	Maximum
	Minimum

AmplifyBio Study Number A-579-01

Individual Animal Hematology Data

Key Page

Measurement/Statistics (Continued)

Measurement	Descriptive
Hematology - MPV	Mean
	Standard Deviation
	Maximum
	Minimum

Group Information

Short Name	Long Name	Type	Report Headings 1-4			
1	Group 1	Control	Group 1	Control	Total vg	0
2	Group 2	Dose	Group 2	Low	Total vg	6x10^13
3	Group 3	Dose	Group 3	High	Total vg	1.2x10^14

Comment Abbreviations

RC = Result Comment

Attachment 2. Individual Animal Coagulation Data and Test Methods/Definitions

Coagulation Test Methods/Definitions

Test Parameter	Test Method	Units
Activated Partial Thromboplastin Time	Physical Clot Detection	seconds
Fibrinogen	Physical Clot Detection	mg/dL
Prothrombin Time	Physical Clot Detection	seconds

10/30/2023 10:42:45AM Page: 1

AmplifyBio Study Number A-579-01

Individual Animal Coagulation Data

Sex: Male Day(s) Relative to Start Date

Group 1 Control Total vg 0	APTT (sec)	APTT (sec)	APTT (sec)	APTT (sec)	APTT (sec)	APTT (sec)	APTT (sec)	PT (sec)	PT (sec)	PT (sec)	PT (sec)
	-41	2	8	22	43	50	85	-41	2	8	22
101	19.0	19.3	20.3	20.9	19.9	-	-	10.2	10.5	10.3	11.1
102	18.6	18.8	19.8	20.3	20.0	19.1	19.7	10.7	11.7	11.4	12.0
Mean	18.8	19.1	20.1	20.6	20.0	19.1	19.7	10.5	11.1	10.9	11.6
SD	0.3	0.4	0.4	0.4	0.1	-	-	0.4	0.8	0.8	0.6
Max	19.0	19.3	20.3	20.9	20.0	19.1	19.7	10.7	11.7	11.4	12.0
Min	18.6	18.8	19.8	20.3	19.9	19.1	19.7	10.2	10.5	10.3	11.1

AmplifyBio Study Number A-579-01

Individual Animal Coagulation Data

Sex: Male Day(s) Relative to Start Date

Group 1 Control Total vg 0	PT (sec)	PT (sec)	PT (sec)	FIB (mg/dL)	FIB (mg/dL)	FIB (mg/dL)	FIB (mg/dL)	FIB (mg/dL)	FIB (mg/dL)	FIB (mg/dL)
	43	50	85	-41	2	8	22	43	50	85
101	10.0	-	-	258	299	256	250	262	-	-
102	11.4	10.5	11.3	235	232	189	202	222	200	192
Mean	10.7	10.5	11.3	247	266	223	226	242	200	192
SD	1.0	-	-	16	47	47	34	28	-	-
Max	11.4	10.5	11.3	258	299	256	250	262	200	192
Min	10.0	10.5	11.3	235	232	189	202	222	200	192

10/30/2023 10:42:45AM Page: 3

AmplifyBio Study Number A-579-01

Individual Animal Coagulation Data

Sex: Male Day(s) Relative to Start Date

Group 2 Low Total vg 6x10^13	APTT (sec)	APTT (sec)	APTT (sec)	APTT (sec)	APTT (sec)	APTT (sec)	APTT (sec)	PT (sec)	PT (sec)	PT (sec)	PT (sec)
	-42	2	8	22	43	50	85	-42	2	8	22
201	17.0	17.5	18.4	18.3	19.2	-	-	11.6	11.1	10.5	10.5
202	16.1	17.0	17.4	18.1	17.6	-	-	11.1	11.0	10.9	10.8
203	18.2	18.0	18.2	18.7	19.7	18.2	18.9	11.4	11.3	11.4	11.7
204	16.1	17.0	18.6	18.3	17.8	18.1	18.4	10.6	10.7	11.0	10.8
Mean	16.9	17.4	18.2	18.4	18.6	18.2	18.7	11.2	11.0	11.0	11.0
SD	1.0	0.5	0.5	0.3	1.0	0.1	0.4	0.4	0.3	0.4	0.5
Max	18.2	18.0	18.6	18.7	19.7	18.2	18.9	11.6	11.3	11.4	11.7
Min	16.1	17.0	17.4	18.1	17.6	18.1	18.4	10.6	10.7	10.5	10.5

AmplifyBio Study Number A-579-01

Individual Animal Coagulation Data

Sex: Male Day(s) Relative to Start Date

Group 2 Low Total vg 6x10^13	PT (sec)	PT (sec)	PT (sec)	FIB (mg/dL)	FIB (mg/dL)	FIB (mg/dL)	FIB (mg/dL)	FIB (mg/dL)	FIB (mg/dL)	FIB (mg/dL)
	43	50	85	-42	2	8	22	43	50	85
201	11.4	-	-	298	350	259	237	244	-	-
202	11.5	-	-	220	312	240	241	213	-	-
203	11.9	11.3	12.5	253	357	273	285	264	258	258
204	10.2	10.7	10.6	308	373	239	217	224	273	211
Mean	11.3	11.0	11.6	270	348	253	245	236	266	235
SD	0.7	0.4	1.3	41	26	16	29	23	11	33
Max	11.9	11.3	12.5	308	373	273	285	264	273	258
Min	10.2	10.7	10.6	220	312	239	217	213	258	211

AmplifyBio Study Number A-579-01

Individual Animal Coagulation Data

Sex: Male Day(s) Relative to Start Date

Group 3 High Total vg 1.2x10^14	APTT (sec)	APTT (sec)	APTT (sec)	APTT (sec)	APTT (sec)	APTT (sec)	APTT (sec)	PT (sec)	PT (sec)	PT (sec)	PT (sec)
	-43	2	8	22	43	50	85	-43	2	8	22
301	17.1	18.8	19.0	19.4	19.2	-	-	11.5	11.3	10.6	11.6
302	18.1	18.5	19.0	18.9	19.3	-	-	11.0	10.6	10.9	10.4
303	17.5	18.8	19.7	19.5	20.3	20.2	18.7	10.4	11.4	11.6	10.4
304	19.3	20.6	20.9	20.4	19.8	20.3	19.7	11.5	11.3	11.5	11.1
Mean	18.0	19.2	19.7	19.6	19.7	20.3	19.2	11.1	11.2	11.2	10.9
SD	1.0	1.0	0.9	0.6	0.5	0.1	0.7	0.5	0.4	0.5	0.6
Max	19.3	20.6	20.9	20.4	20.3	20.3	19.7	11.5	11.4	11.6	11.6
Min	17.1	18.5	19.0	18.9	19.2	20.2	18.7	10.4	10.6	10.6	10.4

AmplifyBio Study Number A-579-01

Individual Animal Coagulation Data

Sex: Male Day(s) Relative to Start Date

Group 3 High Total vg 1.2x10^14	PT (sec)	PT (sec)	PT (sec)	FIB (mg/dL)	FIB (mg/dL)	FIB (mg/dL)	FIB (mg/dL)	FIB (mg/dL)	FIB (mg/dL)	FIB (mg/dL)
	43	50	85	-43	2	8	22	43	50	85
301	11.6	-	-	255	395	251	217	154	-	-
302	10.8	-	-	267	291	320	323	169	-	-
303	10.7	10.3	11.0	306	288	255	279	239	232	231
304	11.0	10.9	11.2	310	402	257	247	237	251	217
Mean	11.0	10.6	11.1	285	344	271	267	200	242	224
SD	0.4	0.4	0.1	28	63	33	45	45	13	10
Max	11.6	10.9	11.2	310	402	320	323	239	251	231
Min	10.7	10.3	11.0	255	288	251	217	154	232	217

10/30/2023 10:42:45AM Page: 7

AmplifyBio Study Number A-579-01

Individual Animal Coagulation Data

Sex: Female Day(s) Relative to Start Date

Group 1 Control Total vg 0	APTT (sec)	APTT (sec)	APTT (sec)	APTT (sec)	APTT (sec)	APTT (sec)	APTT (sec)	PT (sec)	PT (sec)	PT (sec)	PT (sec)
	-41	2	8	22	43	50	85	-41	2	8	22
151	16.3	18.0	17.5	18.0	18.2	-	-	10.8	11.8	11.2	10.9
152	17.2	17.9	19.8	20.8 [a]	18.2	19.6 [b]	19.7	10.1	10.9	10.8	10.4 [a]
Mean	16.8	18.0	18.7	19.4	18.2	19.6	19.7	10.5	11.4	11.0	10.7
SD	0.6	0.1	1.6	2.0	0.0	-	-	0.5	0.6	0.3	0.4
Max	17.2	18.0	19.8	20.8	18.2	19.6	19.7	10.8	11.8	11.2	10.9
Min	16.3	17.9	17.5	18.0	18.2	19.6	19.7	10.1	10.9	10.8	10.4

[a] [SC:Hemolyzed Sample]
[b] [SC:Hemolysis +1]

AmplifyBio Study Number A-579-01

Individual Animal Coagulation Data

Sex: Female Day(s) Relative to Start Date

Group 1 Control Total vg 0	PT (sec)	PT (sec)	PT (sec)	FIB (mg/dL)	FIB (mg/dL)	FIB (mg/dL)	FIB (mg/dL)	FIB (mg/dL)	FIB (mg/dL)	FIB (mg/dL)
	43	50	85	-41	2	8	22	43	50	85
151	11.7	-	-	345	322	270	247	258	-	-
152	10.6	11.2 [a]	10.6	343	334	267	244 [b]	244	232 [a]	220
Mean	11.2	11.2	10.6	344	328	269	246	251	232	220
SD	0.8	-	-	1	8	2	2	10	-	-
Max	11.7	11.2	10.6	345	334	270	247	258	232	220
Min	10.6	11.2	10.6	343	322	267	244	244	232	220

[a] [SC:Hemolysis +1]
[b] [SC:Hemolyzed Sample]

10/30/2023 10:42:45AM Page: 9

AmplifyBio Study Number A-579-01

Individual Animal Coagulation Data

Sex: Female Day(s) Relative to Start Date

Group 2 Low Total vg 6x10^13	APTT (sec)	APTT (sec)	APTT (sec)	APTT (sec)	APTT (sec)	APTT (sec)	APTT (sec)	PT (sec)	PT (sec)	PT (sec)	PT (sec)
	-42	2	8	22	43	50	85	-42	2	8	22
251	18.8	18.9	19.6	20.0	21.4	-	-	10.4	10.8	10.4	10.4
252	17.4	17.7	18.4	20.0	19.7	-	-	11.4	11.3	10.8	11.0
253	16.8	16.8	17.6	20.1	19.3	18.9	18.1	10.2	10.2	10.0	10.2
254	16.6	18.6	18.2	19.4	18.5	18.0	17.9	10.6	11.0	10.5	11.1
Mean	17.4	18.0	18.5	19.9	19.7	18.5	18.0	10.7	10.8	10.4	10.7
SD	1.0	0.9	0.8	0.3	1.2	0.6	0.1	0.5	0.5	0.3	0.4
Max	18.8	18.9	19.6	20.1	21.4	18.9	18.1	11.4	11.3	10.8	11.1
Min	16.6	16.8	17.6	19.4	18.5	18.0	17.9	10.2	10.2	10.0	10.2

AmplifyBio Study Number A-579-01

Individual Animal Coagulation Data

Sex: Female Day(s) Relative to Start Date

Group 2 Low Total vg 6x10^13	PT (sec)	PT (sec)	PT (sec)	FIB (mg/dL)	FIB (mg/dL)	FIB (mg/dL)	FIB (mg/dL)	FIB (mg/dL)	FIB (mg/dL)	FIB (mg/dL)
	43	50	85	-42	2	8	22	43	50	85
251	10.2	-	-	226	263	193	216	165	-	-
252	11.8	-	-	231	208	178	188	138	-	-
253	10.2	10.5	10.7	307	239	207	232	200	214	173
254	10.7	11.0	11.5	217	263	239	194	183	165	170
Mean	10.7	10.8	11.1	245	243	204	208	172	190	172
SD	0.8	0.4	0.6	42	26	26	20	27	35	2
Max	11.8	11.0	11.5	307	263	239	232	200	214	173
Min	10.2	10.5	10.7	217	208	178	188	138	165	170

AmplifyBio Study Number A-579-01

Individual Animal Coagulation Data

Sex: Female Day(s) Relative to Start Date

Group 3 High Total vg 1.2x10^14	APTT (sec)	APTT (sec)	APTT (sec)	APTT (sec)	APTT (sec)	APTT (sec)	APTT (sec)	PT (sec)	PT (sec)	PT (sec)	PT (sec)
	-43	2	8	22	43	50	85	-43	2	8	22
351	18.5	18.0	18.4	20.5	18.6	-	-	10.5	11.1	10.2	10.6
352	21.3	20.1	21.9	21.6	22.5	-	-	10.8	11.0	10.1	10.7
353	19.7	21.6	20.2	22.2	20.9	21.4	19.8	11.0	10.9	10.5	10.5
354	17.2	18.4	18.9	18.6	18.4	18.6	17.9	10.9	11.2	11.1	10.2
Mean	19.2	19.5	19.9	20.7	20.1	20.0	18.9	10.8	11.1	10.5	10.5
SD	1.7	1.7	1.6	1.6	2.0	2.0	1.3	0.2	0.1	0.4	0.2
Max	21.3	21.6	21.9	22.2	22.5	21.4	19.8	11.0	11.2	11.1	10.7
Min	17.2	18.0	18.4	18.6	18.4	18.6	17.9	10.5	10.9	10.1	10.2

AmplifyBio Study Number A-579-01

Individual Animal Coagulation Data

Sex: Female Day(s) Relative to Start Date

Group 3 High Total vg 1.2x10^14	PT (sec)	PT (sec)	PT (sec)	FIB (mg/dL)	FIB (mg/dL)	FIB (mg/dL)	FIB (mg/dL)	FIB (mg/dL)	FIB (mg/dL)	FIB (mg/dL)
	43	50	85	-43	2	8	22	43	50	85
351	9.7	-	-	258	235	296	211	216	-	-
352	10.5	-	-	242	224	424	218	186	-	-
353	10.7	10.4	10.7	268	779	279	265	475	307	428
354	10.9	11.0	11.0	253	258	206	222	205	191	179
Mean	10.5	10.7	10.9	255	374	301	229	271	249	304
SD	0.5	0.4	0.2	11	270	91	24	137	82	176
Max	10.9	11.0	11.0	268	779	424	265	475	307	428
Min	9.7	10.4	10.7	242	224	206	211	186	191	179

AmplifyBio Study Number A-579-01

Individual Animal Coagulation Data

Comments and Markers

Page	Day	Group	Sex	Subject	Measurement	Type	Marker
7	22	1	Female	152	APTT	Sample	
			Comment: Hemolyzed Sample				
7	22	1	Female	152	PT	Sample	
			Comment: Hemolyzed Sample				
7	50	1	Female	152	APTT	Sample	
			Comment: Hemolysis +1				
8	22	1	Female	152	FIB	Sample	
			Comment: Hemolyzed Sample				
8	50	1	Female	152	PT	Sample	
			Comment: Hemolysis +1				
8	50	1	Female	152	FIB	Sample	
			Comment: Hemolysis +1				

AmplifyBio Study Number A-579-01

Individual Animal Coagulation Data

Key Page

Measurement Descriptions

Headings Used	Description
APTT	Activated Partial Thromboplastin Time
PT	Prothrombin Time
FIB	Fibrinogen

Unit Descriptions

Headings Used	Description
mg/dL	mg/dL
sec	Seconds

Measurement/Statistics

Measurement	Descriptive
APTT	Mean
	Standard Deviation
	Maximum
	Minimum
PT	Mean
	Standard Deviation
	Maximum
	Minimum
FIB	Mean
	Standard Deviation
	Maximum
	Minimum

AmplifyBio Study Number A-579-01

Individual Animal Coagulation Data

Key Page

Group Information

Short Name	Long Name	Type	Report Headings 1-4			
1	Group 1	Control	Group 1	Control	Total vg	0
2	Group 2	Dose	Group 2	Low	Total vg	6x10^13
3	Group 3	Dose	Group 3	High	Total vg	1.2x10^14

Comment Abbreviations

SC = Sample Comment

Attachment 3. Individual Animal Serum Chemistry Data and Test Methods/Definitions

Serum Chemistry Test Methods/Definitions

Test Parameter	Test Method	Units
Albumin	Roche: BCG Gen. 2	g/dL
Albumin to Globulin Ratio	Calculation	None
Alanine Aminotransferase	Roche: IFCC without pyridoxal phosphate	U/L
Alkaline Phosphatase	Roche: IFCC Gen. 2, Tietz NW	U/L
Aspartate Aminotransferase	Roche: IFCC without pyridoxal phosphate STAT	U/L
Blood Urea Nitrogen	Roche: Urease/GLDH STAT	mg/dL
Calcium	Roche: 5-Nitro-5’-methyl-BAPTA STAT	mg/dL
Chloride	Roche: I.S.E. indirect potentiometry	mmol/L
Cholesterol	Roche: CHOD-PAP Gen. 2 stand. Abell-Kendall	mg/dL
Creatine Kinase	Roche: IFCC liquid	U/L
Creatinine	Roche: Jaffe rate-blanked and compensated Gen. 2	mg/dL
Direct Bilirubin	Roche: Jendrassik-Grof	mg/dL
Gamma-glutamyl Transferase	Roche: Liquid stand. Szasz	U/L
Globulin	Calculation	g/dL
Glucose	Roche: HK STAT	mg/dL
Lactate Dehydrogenase	Roche: IFCC liquid ver. 2 (ACN# 80)	U/L
Phosphorus	Roche: Molybdate UV ver. 2 STAT	mg/dL
Potassium	Roche: I.S.E. indirect potentiometry	mmol/L
Sodium	Roche: I.S.E. indirect potentiometry	mmol/L
Total Protein	Roche: Biuret Gen. 2 STAT	g/dL
Total Bilirubin	Gen. 3 (ACN 297)	mg/dL
Triglycerides	Roche: GPO-PAP	mg/dL
Lipemic Index*	Roche: Serum Index Gen.2	None
Icteric Index*		
Hemolytic Index*		

* The index parameters are run on all samples; these parameters are included in the study file but are not used for reporting purposes.

3/8/2024 1:41:07PM Page: 1

AmplifyBio Study Number A-579-01

Individual Animal Serum Chemistry Data

Sex: Male Day(s) Relative to Start Date

Group 1 Control Total vg 0	Serum Chemistry										
	ALT (U/L)	ALT (U/L)	ALT (U/L)	ALT (U/L)	ALT (U/L)	ALT (U/L)	ALT (U/L)	AST (U/L)	AST (U/L)	AST (U/L)	AST (U/L)
	-41	2	8	22	43	50	85	-41	2	8	22
101	50	91	56	43	53	-	-	85 [a]	152	57	49
102	38	42	33	29	31	25	47	71	72	49	50
Mean	44	67	45	36	42	25	47	78	112	53	50
SD	8	35	16	10	16	-	-	10	57	6	1
Max	50	91	56	43	53	25	47	85	152	57	50
Min	38	42	33	29	31	25	47	71	72	49	49

[a] [RC:Serum index interference hemolysis]

3/8/2024 1:41:07PM Page: 2

AmplifyBio Study Number A-579-01

Individual Animal Serum Chemistry Data

Sex: Male Day(s) Relative to Start Date

Group 1 Control Total vg 0	Serum Chemistry										
	AST (U/L)	AST (U/L)	AST (U/L)	Alkaline Phosphatase (U/L)	Alkaline Phosphatase (U/L)	Alkaline Phosphatase (U/L)	Alkaline Phosphatase (U/L)	Alkaline Phosphatase (U/L)	Alkaline Phosphatase (U/L)	Alkaline Phosphatase (U/L)	Total Bilirubin (mg/dL)
	43	50	85	-41	2	8	22	43	50	85	-41
101	61	-	-	763	549	534	439	513	-	-	0.22
102	40	44	69	725	727	708	786	796	784	752	0.10 E[a]
Mean	51	44	69	744	638	621	613	655	784	752	0.22
SD	15	-	-	27	126	123	245	200	-	-	-
Max	61	44	69	763	727	708	786	796	784	752	0.22
Min	40	44	69	725	549	534	439	513	784	752	0.22

E = Exclude
[a] [RC:< Linearity, FC:Result below linearity]

3/8/2024 1:41:07PM Page: 3

AmplifyBio Study Number A-579-01

Individual Animal Serum Chemistry Data

Sex: Male Day(s) Relative to Start Date

Group 1 Control Total vg 0	Serum Chemistry										
	Total Bilirubin (mg/dL)	Total Bilirubin (mg/dL)	Total Bilirubin (mg/dL)	Total Bilirubin (mg/dL)	Total Bilirubin (mg/dL)	Total Bilirubin (mg/dL)	Direct Bilirubin (mg/dL)	Direct Bilirubin (mg/dL)	Direct Bilirubin (mg/dL)	Direct Bilirubin (mg/dL)	Direct Bilirubin (mg/dL)
	2	8	22	43	50	85	-41	2	8	22	43
101	0.09 E[a]	0.18	0.10 E[a]	0.09 E[a]	-	-	0.05 E[a]	0.03 E[a]	0.04 E[a]	0.03 E[a]	0.04 E[a]
102	0.10 E[a]	0.18	0.16	0.15 E[a]	0.14 E[a]	0.10 E[a]	0.03 E[a]	0.03 E[a]	0.04 E[a]	0.03 E[a]	0.05 E[a]
Mean	-	0.18	0.16	-	-	-	-	-	-	-	-
SD	-	0.00	-	-	-	-	-	-	-	-	-
Max	-	0.18	0.16	-	-	-	-	-	-	-	-
Min	-	0.18	0.16	-	-	-	-	-	-	-	-

E = Exclude
[a] [RC:< Linearity, FC:Result below linearity]

3/8/2024 1:41:07PM Page: 4

AmplifyBio Study Number A-579-01

Individual Animal Serum Chemistry Data

Sex: Male Day(s) Relative to Start Date

Group 1 Control Total vg 0	Serum Chemistry										
	Direct Bilirubin (mg/dL)	Direct Bilirubin (mg/dL)	LDH (U/L)	LDH (U/L)	LDH (U/L)	LDH (U/L)	LDH (U/L)	LDH (U/L)	LDH (U/L)	Creatine Kinase (U/L)	Creatine Kinase (U/L)
	50	85	-41	2	8	22	43	50	85	-41	2
101	-	-	722	743 [c]	392	328	427	-	-	618	1471
102	0.04 E[a]	0.02 E[a]	1125 E[b]	556	407	466 [c]	416	389	486	445	700
Mean	-	-	722	650	400	397	422	389	486	532	1086
SD	-	-	-	132	11	98	8	-	-	122	545
Max	-	-	722	743	407	466	427	389	486	618	1471
Min	-	-	722	556	392	328	416	389	486	445	700

E = Exclude
[a] [RC:< Linearity, FC:Result below linearity]
[b] [FC:Result above linearity]
[c] [RC:Serum index interference hemolysis]

3/8/2024 1:41:07PM Page: 5

AmplifyBio Study Number A-579-01

Individual Animal Serum Chemistry Data

Sex: Male Day(s) Relative to Start Date

Group 1 Control Total vg 0	Serum Chemistry										
	Creatine Kinase (U/L)	Creatine Kinase (U/L)	Creatine Kinase (U/L)	Creatine Kinase (U/L)	Creatine Kinase (U/L)	Cholesterol (mg/dL)	Cholesterol (mg/dL)	Cholesterol (mg/dL)	Cholesterol (mg/dL)	Cholesterol (mg/dL)	Cholesterol (mg/dL)
	8	22	43	50	85	-41	2	8	22	43	50
101	716	365	1065	-	-	153	153	169	173	178	-
102	890	787	185	804	1072	154	156	148	155	162	146
Mean	803	576	625	804	1072	154	155	159	164	170	146
SD	123	298	622	-	-	1	2	15	13	11	-
Max	890	787	1065	804	1072	154	156	169	173	178	146
Min	716	365	185	804	1072	153	153	148	155	162	146

3/8/2024 1:41:07PM Page: 6

AmplifyBio Study Number A-579-01

Individual Animal Serum Chemistry Data

Sex: Male Day(s) Relative to Start Date

Group 1 Control Total vg 0	Serum Chemistry									
	Cholesterol (mg/dL)	TRIG (mg/dL)	TRIG (mg/dL)	TRIG (mg/dL)	TRIG (mg/dL)	TRIG (mg/dL)	TRIG (mg/dL)	TRIG (mg/dL)	Blood Urea Nitrogen (mg/dL)	Blood Urea Nitrogen (mg/dL)	Blood Urea Nitrogen (mg/dL)
	85	-41	2	8	22	43	50	85	-41	2	8
101	-	59	75	63	47	48	-	-	23	8	25
102	140	48	74	56	50	50	44	41	16	11	21
Mean	140	54	75	60	49	49	44	41	20	10	23
SD	-	8	1	5	2	1	-	-	5	2	3
Max	140	59	75	63	50	50	44	41	23	11	25
Min	140	48	74	56	47	48	44	41	16	8	21

3/8/2024 1:41:07PM Page: 7

AmplifyBio Study Number A-579-01

Individual Animal Serum Chemistry Data

Sex: Male Day(s) Relative to Start Date

Group 1 Control Total vg 0	Serum Chemistry										
	Blood Urea Nitrogen (mg/dL)	Blood Urea Nitrogen (mg/dL)	Blood Urea Nitrogen (mg/dL)	Blood Urea Nitrogen (mg/dL)	Creatinine (mg/dL)	Creatinine (mg/dL)	Creatinine (mg/dL)	Creatinine (mg/dL)	Creatinine (mg/dL)	Creatinine (mg/dL)	Creatinine (mg/dL)
	22	43	50	85	-41	2	8	22	43	50	85
101	23	17	-	-	0.7	0.7	0.6	0.6	0.5	-	-
102	21	18	19	22	0.7	0.7	0.7	0.7	0.7	0.7	0.6
Mean	22	18	19	22	0.7	0.7	0.7	0.7	0.6	0.7	0.6
SD	1	1	-	-	0.0	0.0	0.1	0.1	0.1	-	-
Max	23	18	19	22	0.7	0.7	0.7	0.7	0.7	0.7	0.6
Min	21	17	19	22	0.7	0.7	0.6	0.6	0.5	0.7	0.6

3/8/2024 1:41:07PM Page: 8

AmplifyBio Study Number A-579-01

Individual Animal Serum Chemistry Data

Sex: Male Day(s) Relative to Start Date

Group 1 Control Total vg 0	Serum Chemistry										
	Total Protein (g/dL)	Total Protein (g/dL)	Total Protein (g/dL)	Total Protein (g/dL)	Total Protein (g/dL)	Total Protein (g/dL)	Total Protein (g/dL)	ALB (g/dL)	ALB (g/dL)	ALB (g/dL)	ALB (g/dL)
	-41	2	8	22	43	50	85	-41	2	8	22
101	6.6	6.9	6.8	7.2	7.1	-	-	3.8	4.0	4.0	4.2
102	6.9	6.8	6.5	6.7	6.8	6.6	6.3	3.7	4.1	4.0	4.2
Mean	6.8	6.9	6.7	7.0	7.0	6.6	6.3	3.8	4.1	4.0	4.2
SD	0.2	0.1	0.2	0.4	0.2	-	-	0.1	0.1	0.0	0.0
Max	6.9	6.9	6.8	7.2	7.1	6.6	6.3	3.8	4.1	4.0	4.2
Min	6.6	6.8	6.5	6.7	6.8	6.6	6.3	3.7	4.0	4.0	4.2

3/8/2024 1:41:07PM Page: 9

AmplifyBio Study Number A-579-01

Individual Animal Serum Chemistry Data

Sex: Male Day(s) Relative to Start Date

Group 1 Control Total vg 0	Serum Chemistry										
	ALB (g/dL)	ALB (g/dL)	ALB (g/dL)	Globulin (g/dL)	Globulin (g/dL)	Globulin (g/dL)	Globulin (g/dL)	Globulin (g/dL)	Globulin (g/dL)	Globulin (g/dL)	A/G Ratio
	43	50	85	-41	2	8	22	43	50	85	-41
101	4.2	-	-	2.8	2.9	2.8	3.0	2.9	-	-	1.4
102	4.2	4.2	3.8	3.2	2.7	2.5	2.5	2.6	2.4	2.5	1.2
Mean	4.2	4.2	3.8	3.0	2.8	2.7	2.8	2.8	2.4	2.5	1.3
SD	0.0	-	-	0.3	0.1	0.2	0.4	0.2	-	-	0.1
Max	4.2	4.2	3.8	3.2	2.9	2.8	3.0	2.9	2.4	2.5	1.4
Min	4.2	4.2	3.8	2.8	2.7	2.5	2.5	2.6	2.4	2.5	1.2

3/8/2024 1:41:07PM Page: 10

AmplifyBio Study Number A-579-01

Individual Animal Serum Chemistry Data

Sex: Male Day(s) Relative to Start Date

Group 1 Control Total vg 0	Serum Chemistry										
	A/G Ratio	A/G Ratio	A/G Ratio	A/G Ratio	A/G Ratio	A/G Ratio	Calcium (mg/dL)	Calcium (mg/dL)	Calcium (mg/dL)	Calcium (mg/dL)	Calcium (mg/dL)
	2	8	22	43	50	85	-41	2	8	22	43
101	1.4	1.4	1.4	1.4	-	-	9.4	10.2	9.5	10.4	10.3
102	1.5	1.6	1.7	1.6	1.8	1.5	10.2	10.7	10.6	11.0	10.6
Mean	1.5	1.5	1.6	1.5	1.8	1.5	9.8	10.5	10.1	10.7	10.5
SD	0.1	0.1	0.2	0.1	-	-	0.6	0.4	0.8	0.4	0.2
Max	1.5	1.6	1.7	1.6	1.8	1.5	10.2	10.7	10.6	11.0	10.6
Min	1.4	1.4	1.4	1.4	1.8	1.5	9.4	10.2	9.5	10.4	10.3

3/8/2024 1:41:07PM Page: 11

AmplifyBio Study Number A-579-01

Individual Animal Serum Chemistry Data

Sex: Male Day(s) Relative to Start Date

Group 1 Control Total vg 0	Serum Chemistry										
	Calcium (mg/dL)	Calcium (mg/dL)	Phosphorus (mg/dL)	Phosphorus (mg/dL)	Phosphorus (mg/dL)	Phosphorus (mg/dL)	Phosphorus (mg/dL)	Phosphorus (mg/dL)	Phosphorus (mg/dL)	Sodium (mmol/L)	Sodium (mmol/L)
	50	85	-41	2	8	22	43	50	85	-41	2
101	-	-	6.2	5.1	7.3	7.9	6.4	-	-	148	146
102	10.6	10.2	5.9	6.7	7.3	7.9	7.3	6.5	6.9	149	150
Mean	10.6	10.2	6.1	5.9	7.3	7.9	6.9	6.5	6.9	149	148
SD	-	-	0.2	1.1	0.0	0.0	0.6	-	-	1	3
Max	10.6	10.2	6.2	6.7	7.3	7.9	7.3	6.5	6.9	149	150
Min	10.6	10.2	5.9	5.1	7.3	7.9	6.4	6.5	6.9	148	146

3/8/2024 1:41:07PM Page: 12

AmplifyBio Study Number A-579-01

Individual Animal Serum Chemistry Data

Sex: Male Day(s) Relative to Start Date

Group 1 Control Total vg 0	Serum Chemistry										
	Sodium (mmol/L)	Sodium (mmol/L)	Sodium (mmol/L)	Sodium (mmol/L)	Sodium (mmol/L)	Chloride (mmol/L)	Chloride (mmol/L)	Chloride (mmol/L)	Chloride (mmol/L)	Chloride (mmol/L)	Chloride (mmol/L)
	8	22	43	50	85	-41	2	8	22	43	50
101	146	145	144	-	-	108	105	108	102	108	-
102	145	148	145	144	145	109	107	105	103	105	106
Mean	146	147	145	144	145	109	106	107	103	107	106
SD	1	2	1	-	-	1	1	2	1	2	-
Max	146	148	145	144	145	109	107	108	103	108	106
Min	145	145	144	144	145	108	105	105	102	105	106

3/8/2024 1:41:07PM Page: 13

AmplifyBio Study Number A-579-01

Individual Animal Serum Chemistry Data

Sex: Male Day(s) Relative to Start Date

Group 1 Control Total vg 0	Serum Chemistry										
	Chloride (mmol/L)	Potassium (mmol/L)	Potassium (mmol/L)	Potassium (mmol/L)	Potassium (mmol/L)	Potassium (mmol/L)	Potassium (mmol/L)	Potassium (mmol/L)	Glucose (mg/dL)	Glucose (mg/dL)	Glucose (mg/dL)
	85	-41	2	8	22	43	50	85	-41	2	8
101	-	4.9	4.5	4.0	4.6	4.1	-	-	44	77	43
102	107	4.5	4.7	4.3	4.9	3.9	4.5	4.2	52	91	66
Mean	107	4.7	4.6	4.2	4.8	4.0	4.5	4.2	48	84	55
SD	-	0.3	0.1	0.2	0.2	0.1	-	-	6	10	16
Max	107	4.9	4.7	4.3	4.9	4.1	4.5	4.2	52	91	66
Min	107	4.5	4.5	4.0	4.6	3.9	4.5	4.2	44	77	43

3/8/2024 1:41:07PM Page: 14

AmplifyBio Study Number A-579-01

Individual Animal Serum Chemistry Data

Sex: Male Day(s) Relative to Start Date

Group 1 Control Total vg 0	Serum Chemistry										
	Glucose (mg/dL)	Glucose (mg/dL)	Glucose (mg/dL)	Glucose (mg/dL)	GGT (U/L)	GGT (U/L)	GGT (U/L)	GGT (U/L)	GGT (U/L)	GGT (U/L)	GGT (U/L)
	22	43	50	85	-41	2	8	22	43	50	85
101	65	54	-	-	85	80	76	84	95	-	-
102	68	68	73	83	109	128	126	142	140	135	122
Mean	67	61	73	83	97	104	101	113	118	135	122
SD	2	10	-	-	17	34	35	41	32	-	-
Max	68	68	73	83	109	128	126	142	140	135	122
Min	65	54	73	83	85	80	76	84	95	135	122

3/8/2024 1:41:07PM Page: 15

AmplifyBio Study Number A-579-01

Individual Animal Serum Chemistry Data

Sex: Male Day(s) Relative to Start Date

Group 2 Low Total vg 6x10^13	Serum Chemistry										
	ALT (U/L)	ALT (U/L)	ALT (U/L)	ALT (U/L)	ALT (U/L)	ALT (U/L)	ALT (U/L)	AST (U/L)	AST (U/L)	AST (U/L)	AST (U/L)
	-42	2	8	22	43	50	85	-42	2	8	22
201	78	63	68	54	66	-	-	63	60	46	47
202	46	49	44	44	40	-	-	55	63 [a]	54	54
203	31	75	60	34	36	31	19	78	181	70	65
204	59	68	58	50	44	43	45	101	178	52	57
Mean	54	64	58	46	47	37	32	74	121	56	56
SD	20	11	10	9	13	8	18	20	68	10	7
Max	78	75	68	54	66	43	45	101	181	70	65
Min	31	49	44	34	36	31	19	55	60	46	47

[a] [RC:Serum index interference hemolysis]

3/8/2024 1:41:07PM Page: 16

AmplifyBio Study Number A-579-01

Individual Animal Serum Chemistry Data

Sex: Male Day(s) Relative to Start Date

Group 2 Low Total vg 6x10^13	Serum Chemistry										
	AST (U/L)	AST (U/L)	AST (U/L)	Alkaline Phosphatase (U/L)	Alkaline Phosphatase (U/L)	Alkaline Phosphatase (U/L)	Alkaline Phosphatase (U/L)	Alkaline Phosphatase (U/L)	Alkaline Phosphatase (U/L)	Alkaline Phosphatase (U/L)	Total Bilirubin (mg/dL)
	43	50	85	-42	2	8	22	43	50	85	-42
201	39	-	-	668	446	449	481	436	-	-	0.26
202	39	-	-	679	490	450	501	507	-	-	0.19
203	73	60	57	858	566	546	640	638	709	550	0.15 E[a]
204	49	45	51	470	448	390	518	551	573	598	0.44
Mean	50	53	54	669	488	459	535	533	641	574	0.30
SD	16	11	4	159	56	65	72	85	96	34	0.13
Max	73	60	57	858	566	546	640	638	709	598	0.44
Min	39	45	51	470	446	390	481	436	573	550	0.19

E = Exclude
[a] [RC:< Linearity, FC:Result below linearity]

3/8/2024 1:41:07PM Page: 17

AmplifyBio Study Number A-579-01

Individual Animal Serum Chemistry Data

Sex: Male Day(s) Relative to Start Date

Group 2 Low Total vg 6x10^13	Serum Chemistry										
	Total Bilirubin (mg/dL)	Total Bilirubin (mg/dL)	Total Bilirubin (mg/dL)	Total Bilirubin (mg/dL)	Total Bilirubin (mg/dL)	Total Bilirubin (mg/dL)	Direct Bilirubin (mg/dL)	Direct Bilirubin (mg/dL)	Direct Bilirubin (mg/dL)	Direct Bilirubin (mg/dL)	Direct Bilirubin (mg/dL)
	2	8	22	43	50	85	-42	2	8	22	43
201	0.16	0.26	0.06 E[b]	0.12 E[a]	-	-	0.06 E[a]	0.05 E[a]	0.06 E[a]	0.03 E[b]	0.03 E[a]
202	0.15	0.22	0.17	0.13 E[a]	-	-	0.04 E[a]	0.03 E[a]	0.06 E[a]	0.04 E[b]	0.04 E[a]
203	0.14 E[a]	0.23	0.14 E[b]	0.18	0.19	0.10 E[a]	0.04 E[a]	0.05 E[a]	0.08 E[a]	0.03 E[b]	0.04 E[a]
204	0.39	0.36	0.27	0.21	0.29	0.27	0.10 E[a]	0.11 E[a]	0.09 E[c]	0.07 E[b]	0.06 E[a]
Mean	0.23	0.27	0.22	0.20	0.24	0.27	-	-	-	-	-
SD	0.14	0.06	0.07	0.02	0.07	-	-	-	-	-	-
Max	0.39	0.36	0.27	0.21	0.29	0.27	-	-	-	-	-
Min	0.15	0.22	0.17	0.18	0.19	0.27	-	-	-	-	-

E = Exclude
[a] [RC:< Linearity, FC:Result below linearity]
[b] [RC:< Linearity]
[c] [RC:< Linearity, FC:Results below linearity]

AmplifyBio Study Number A-579-01

Individual Animal Serum Chemistry Data

Sex: Male Day(s) Relative to Start Date

Group 2 Low Total vg 6x10^13	Serum Chemistry									
	Direct Bilirubin (mg/dL)	Direct Bilirubin (mg/dL)	LDH (U/L)	LDH (U/L)	LDH (U/L)	LDH (U/L)	LDH (U/L)	LDH (U/L)	LDH (U/L)	Creatine Kinase (U/L)	Creatine Kinase (U/L)
	50	85	-42	2	8	22	43	50	85	-42	2
201	-	-	456	663	580	678 [c]	297	-	-	299	2229 E[e]
202	-	-	881	683 [c]	524 [c]	548 [c]	334	-	-	308	1203
203	0.05 E[a]	0.04 E[a]	1142 E[b]	1211 E[d]	715	630 [c]	1110 E[b]	634 [c]	466 [c]	1076	1478
204	0.08 E[a]	0.07 E[a]	969 E[b]	1166 E[d]	414	441 [c]	544 [c]	336	291	1212	3400 E[e]
Mean	-	-	669	673	558	574	392	485	379	724	1341
SD	-	-	301	14	125	104	133	211	124	488	194
Max	-	-	881	683	715	678	544	634	466	1212	1478
Min	-	-	456	663	414	441	297	336	291	299	1203

E = Exclude
[a] [RC:< Linearity, FC:Result below linearity]
[b] [FC:Result above linearity]
[c] [RC:Serum index interference hemolysis]
[d] [RC:> Linearity, FC:Result above linearity, reran to confirm]
[e] [FC:Result above linearity, within 20% of RSN1]

3/8/2024 1:41:07PM Page: 19

AmplifyBio Study Number A-579-01

Individual Animal Serum Chemistry Data

Sex: Male Day(s) Relative to Start Date

Group 2 Low Total vg 6x10^13	Serum Chemistry										
	Creatine Kinase (U/L)	Creatine Kinase (U/L)	Creatine Kinase (U/L)	Creatine Kinase (U/L)	Creatine Kinase (U/L)	Cholesterol (mg/dL)	Cholesterol (mg/dL)	Cholesterol (mg/dL)	Cholesterol (mg/dL)	Cholesterol (mg/dL)	Cholesterol (mg/dL)
	8	22	43	50	85	-42	2	8	22	43	50
201	198	180	350	-	-	160	168	240	232	202	-
202	1013	1448	443	-	-	142	128	158	162	132	-
203	1575	1640	718	634	1179	141	137	164	145	137	108
204	556	605	375	454	554	126	113	150	134	136	131
Mean	836	968	472	544	867	142	137	178	168	152	120
SD	595	692	169	127	442	14	23	42	44	34	16
Max	1575	1640	718	634	1179	160	168	240	232	202	131
Min	198	180	350	454	554	126	113	150	134	132	108

3/8/2024 1:41:07PM Page: 20

AmplifyBio Study Number A-579-01

Individual Animal Serum Chemistry Data

Sex: Male Day(s) Relative to Start Date

Group 2 Low Total vg 6x10^13	Serum Chemistry										
	Cholesterol (mg/dL)	TRIG (mg/dL)	TRIG (mg/dL)	TRIG (mg/dL)	TRIG (mg/dL)	TRIG (mg/dL)	TRIG (mg/dL)	TRIG (mg/dL)	Blood Urea Nitrogen (mg/dL)	Blood Urea Nitrogen (mg/dL)	Blood Urea Nitrogen (mg/dL)
	85	-42	2	8	22	43	50	85	-42	2	8
201	-	101	28	35	26	36	-	-	11	9	12
202	-	67	60	74	72	52	-	-	15	19	18
203	112	74	80	54	63	58	84	63	22	19	20
204	119	45	32	48	80	73	53	42	20	16	24
Mean	116	72	50	53	60	55	69	53	17	16	19
SD	5	23	25	16	24	15	22	15	5	5	5
Max	119	101	80	74	80	73	84	63	22	19	24
Min	112	45	28	35	26	36	53	42	11	9	12

3/8/2024 1:41:07PM Page: 21

AmplifyBio Study Number A-579-01

Individual Animal Serum Chemistry Data

Sex: Male Day(s) Relative to Start Date

Group 2 Low Total vg 6x10^13	Serum Chemistry										
	Blood Urea Nitrogen (mg/dL)	Blood Urea Nitrogen (mg/dL)	Blood Urea Nitrogen (mg/dL)	Blood Urea Nitrogen (mg/dL)	Creatinine (mg/dL)	Creatinine (mg/dL)	Creatinine (mg/dL)	Creatinine (mg/dL)	Creatinine (mg/dL)	Creatinine (mg/dL)	Creatinine (mg/dL)
	22	43	50	85	-42	2	8	22	43	50	85
201	15	14	-	-	0.6	0.6	0.6	0.6	0.6	-	-
202	21	17	-	-	0.9	1.0	0.9	1.1	0.8	-	-
203	22	23	22	19	0.7	0.8	0.7	0.8	0.8	0.7	0.8
204	27	24	26	22	0.7	0.7	0.7	0.6	0.7	0.6	0.6
Mean	21	20	24	21	0.7	0.8	0.7	0.8	0.7	0.7	0.7
SD	5	5	3	2	0.1	0.2	0.1	0.2	0.1	0.1	0.1
Max	27	24	26	22	0.9	1.0	0.9	1.1	0.8	0.7	0.8
Min	15	14	22	19	0.6	0.6	0.6	0.6	0.6	0.6	0.6

3/8/2024 1:41:07PM Page: 22

AmplifyBio Study Number A-579-01

Individual Animal Serum Chemistry Data

Sex: Male Day(s) Relative to Start Date

Group 2 Low Total vg 6x10^13	Serum Chemistry										
	Total Protein (g/dL)	Total Protein (g/dL)	Total Protein (g/dL)	Total Protein (g/dL)	Total Protein (g/dL)	Total Protein (g/dL)	Total Protein (g/dL)	ALB (g/dL)	ALB (g/dL)	ALB (g/dL)	ALB (g/dL)
	-42	2	8	22	43	50	85	-42	2	8	22
201	7.0	7.1	7.2	7.1	7.2	-	-	4.2	4.5	4.5	4.5
202	6.8	7.1	6.8	7.1	6.2	-	-	4.3	4.7	4.5	4.8
203	6.8	7.1	6.9	7.0	6.9	6.8	5.7	4.2	4.3	4.3	4.5
204	6.6	6.9	6.9	7.1	7.3	7.4	6.2	3.9	4.2	4.2	4.5
Mean	6.8	7.1	7.0	7.1	6.9	7.1	6.0	4.2	4.4	4.4	4.6
SD	0.2	0.1	0.2	0.1	0.5	0.4	0.4	0.2	0.2	0.1	0.1
Max	7.0	7.1	7.2	7.1	7.3	7.4	6.2	4.3	4.7	4.5	4.8
Min	6.6	6.9	6.8	7.0	6.2	6.8	5.7	3.9	4.2	4.2	4.5

3/8/2024 1:41:07PM Page: 23

AmplifyBio Study Number A-579-01

Individual Animal Serum Chemistry Data

Sex: Male Day(s) Relative to Start Date

Group 2 Low Total vg 6x10^13	Serum Chemistry										
	ALB (g/dL)	ALB (g/dL)	ALB (g/dL)	Globulin (g/dL)	Globulin (g/dL)	Globulin (g/dL)	Globulin (g/dL)	Globulin (g/dL)	Globulin (g/dL)	Globulin (g/dL)	A/G Ratio
	43	50	85	-42	2	8	22	43	50	85	-42
201	4.6	-	-	2.8	2.6	2.7	2.6	2.6	-	-	1.5
202	4.3	-	-	2.5	2.4	2.3	2.3	1.9	-	-	1.7
203	4.3	4.3	3.4	2.6	2.8	2.6	2.5	2.6	2.5	2.3	1.6
204	4.5	4.4	3.8	2.7	2.7	2.7	2.6	2.8	3.0	2.4	1.4
Mean	4.4	4.4	3.6	2.7	2.6	2.6	2.5	2.5	2.8	2.4	1.6
SD	0.1	0.1	0.3	0.1	0.2	0.2	0.1	0.4	0.4	0.1	0.1
Max	4.6	4.4	3.8	2.8	2.8	2.7	2.6	2.8	3.0	2.4	1.7
Min	4.3	4.3	3.4	2.5	2.4	2.3	2.3	1.9	2.5	2.3	1.4

3/8/2024 1:41:07PM Page: 24

AmplifyBio Study Number A-579-01

Individual Animal Serum Chemistry Data

Sex: Male Day(s) Relative to Start Date

Group 2 Low Total vg 6x10^13	Serum Chemistry										
	A/G Ratio	A/G Ratio	A/G Ratio	A/G Ratio	A/G Ratio	A/G Ratio	Calcium (mg/dL)	Calcium (mg/dL)	Calcium (mg/dL)	Calcium (mg/dL)	Calcium (mg/dL)
	2	8	22	43	50	85	-42	2	8	22	43
201	1.7	1.7	1.7	1.8	-	-	9.8	10.0	10.1	10.4	10.1
202	2.0	2.0	2.1	2.3	-	-	10.1	10.5	10.3	10.9	10.0
203	1.5	1.7	1.8	1.7	1.7	1.5	10.7	10.8	10.2	10.6	10.5
204	1.6	1.6	1.7	1.6	1.5	1.6	9.3	9.9	9.4	9.9	10.0
Mean	1.7	1.8	1.8	1.9	1.6	1.6	10.0	10.3	10.0	10.5	10.2
SD	0.2	0.2	0.2	0.3	0.1	0.1	0.6	0.4	0.4	0.4	0.2
Max	2.0	2.0	2.1	2.3	1.7	1.6	10.7	10.8	10.3	10.9	10.5
Min	1.5	1.6	1.7	1.6	1.5	1.5	9.3	9.9	9.4	9.9	10.0

AmplifyBio Study Number A-579-01

Individual Animal Serum Chemistry Data

Sex: Male Day(s) Relative to Start Date

Group 2 Low Total vg 6x10^13	Serum Chemistry										
	Calcium (mg/dL)	Calcium (mg/dL)	Phosphorus (mg/dL)	Phosphorus (mg/dL)	Phosphorus (mg/dL)	Phosphorus (mg/dL)	Phosphorus (mg/dL)	Phosphorus (mg/dL)	Phosphorus (mg/dL)	Sodium (mmol/L)	Sodium (mmol/L)
	50	85	-42	2	8	22	43	50	85	-42	2
201	-	-	6.5	6.7	7.5	8.6	7.9	-	-	151	147
202	-	-	4.4	5.7	5.5	6.0	6.1	-	-	149	150
203	10.4	9.8	5.3	6.6	7.5	6.9	7.6	7.5	6.7	150	153
204	10.0	9.8	4.8	4.7	6.9	5.9	6.5	7.0	7.5	149	150
Mean	10.2	9.8	5.3	5.9	6.9	6.9	7.0	7.3	7.1	150	150
SD	0.3	0.0	0.9	0.9	0.9	1.3	0.9	0.4	0.6	1	2
Max	10.4	9.8	6.5	6.7	7.5	8.6	7.9	7.5	7.5	151	153
Min	10.0	9.8	4.4	4.7	5.5	5.9	6.1	7.0	6.7	149	147

3/8/2024 1:41:07PM Page: 26

AmplifyBio Study Number A-579-01

Individual Animal Serum Chemistry Data

Sex: Male Day(s) Relative to Start Date

Group 2 Low Total vg 6x10^13	Serum Chemistry										
	Sodium (mmol/L)	Sodium (mmol/L)	Sodium (mmol/L)	Sodium (mmol/L)	Sodium (mmol/L)	Chloride (mmol/L)	Chloride (mmol/L)	Chloride (mmol/L)	Chloride (mmol/L)	Chloride (mmol/L)	Chloride (mmol/L)
	8	22	43	50	85	-42	2	8	22	43	50
201	147	151	147	-	-	109	107	109	105	107	-
202	146	148	146	-	-	113	107	108	106	109	-
203	146	149	147	148	142	111	106	108	106	103	106
204	148	148	148	148	148	109	106	108	109	104	106
Mean	147	149	147	148	145	111	107	108	107	106	106
SD	1	1	1	0	4	2	1	1	2	3	0
Max	148	151	148	148	148	113	107	109	109	109	106
Min	146	148	146	148	142	109	106	108	105	103	106

3/8/2024 1:41:07PM Page: 27

AmplifyBio Study Number A-579-01

Individual Animal Serum Chemistry Data

Sex: Male Day(s) Relative to Start Date

Group 2 Low Total vg 6x10^13	Serum Chemistry										
	Chloride (mmol/L)	Potassium (mmol/L)	Potassium (mmol/L)	Potassium (mmol/L)	Potassium (mmol/L)	Potassium (mmol/L)	Potassium (mmol/L)	Potassium (mmol/L)	Glucose (mg/dL)	Glucose (mg/dL)	Glucose (mg/dL)
	85	-42	2	8	22	43	50	85	-42	2	8
201	-	4.9	3.7	4.2	4.3	4.4	-	-	67	86	68
202	-	4.7	4.7	4.3	4.3	4.0	-	-	54	114	58
203	108	5.2	5.0	4.4	4.3	4.8	5.2	4.6	57	81	55
204	108	4.2	4.4	3.9	4.1	3.9	4.0	3.5	54	85	63
Mean	108	4.8	4.5	4.2	4.3	4.3	4.6	4.1	58	92	61
SD	0	0.4	0.6	0.2	0.1	0.4	0.8	0.8	6	15	6
Max	108	5.2	5.0	4.4	4.3	4.8	5.2	4.6	67	114	68
Min	108	4.2	3.7	3.9	4.1	3.9	4.0	3.5	54	81	55

3/8/2024 1:41:07PM Page: 28

AmplifyBio Study Number A-579-01

Individual Animal Serum Chemistry Data

Sex: Male Day(s) Relative to Start Date

Group 2 Low Total vg 6x10^13	Serum Chemistry										
	Glucose (mg/dL)	Glucose (mg/dL)	Glucose (mg/dL)	Glucose (mg/dL)	GGT (U/L)	GGT (U/L)	GGT (U/L)	GGT (U/L)	GGT (U/L)	GGT (U/L)	GGT (U/L)
	22	43	50	85	-42	2	8	22	43	50	85
201	85	66	-	-	112	102	100	110	104	-	-
202	97	65	-	-	58	55	53	66	66	-	-
203	72	83	80	58	80	80	84	100	99	97	53
204	48	82	51	67	59	66	67	77	85	88	97
Mean	76	74	66	63	77	76	76	88	89	93	75
SD	21	10	21	6	25	20	20	20	17	6	31
Max	97	83	80	67	112	102	100	110	104	97	97
Min	48	65	51	58	58	55	53	66	66	88	53

3/8/2024 1:41:07PM Page: 29

AmplifyBio Study Number A-579-01

Individual Animal Serum Chemistry Data

Sex: Male Day(s) Relative to Start Date

Group 3 High Total vg 1.2x10^14	Serum Chemistry										
	ALT (U/L)	ALT (U/L)	ALT (U/L)	ALT (U/L)	ALT (U/L)	ALT (U/L)	ALT (U/L)	AST (U/L)	AST (U/L)	AST (U/L)	AST (U/L)
	-43	2	8	22	43	50	85	-43	2	8	22
301	107	81	60	49	65	-	-	208	160	56	55
302	84	116	59	53	131	-	-	139	174	55	89
303	76	40	65	37	59	49	32	147	59	67	62
304	82	89	68	41	38	54	46	178 [a]	201	57	41
Mean	87	82	63	45	73	52	39	168	149	59	62
SD	14	31	4	7	40	4	10	32	62	6	20
Max	107	116	68	53	131	54	46	208	201	67	89
Min	76	40	59	37	38	49	32	139	59	55	41

[a] [RC:Serum index interference hemolysis]

3/8/2024 1:41:07PM Page: 30

AmplifyBio Study Number A-579-01

Individual Animal Serum Chemistry Data

Sex: Male Day(s) Relative to Start Date

Group 3 High Total vg 1.2x10^14	Serum Chemistry										
	AST (U/L)	AST (U/L)	AST (U/L)	Alkaline Phosphatase (U/L)	Alkaline Phosphatase (U/L)	Alkaline Phosphatase (U/L)	Alkaline Phosphatase (U/L)	Alkaline Phosphatase (U/L)	Alkaline Phosphatase (U/L)	Alkaline Phosphatase (U/L)	Total Bilirubin (mg/dL)
	43	50	85	-43	2	8	22	43	50	85	-43
301	55	-	-	895	633	523	818	702	-	-	0.38
302	76	-	-	599	414	371	338	324	-	-	0.31
303	57	51	40	925	614	654	671	795	758	805	0.34
304	40	46	43	752	579	466	529	538	553	589	0.31
Mean	57	49	42	793	560	504	589	590	656	697	0.34
SD	15	4	2	150	100	118	205	207	145	153	0.03
Max	76	51	43	925	633	654	818	795	758	805	0.38
Min	40	46	40	599	414	371	338	324	553	589	0.31

3/8/2024 1:41:07PM Page: 31

AmplifyBio Study Number A-579-01

Individual Animal Serum Chemistry Data

Sex: Male Day(s) Relative to Start Date

Group 3 High Total vg 1.2x10^14	Serum Chemistry										
	Total Bilirubin (mg/dL)	Total Bilirubin (mg/dL)	Total Bilirubin (mg/dL)	Total Bilirubin (mg/dL)	Total Bilirubin (mg/dL)	Total Bilirubin (mg/dL)	Direct Bilirubin (mg/dL)	Direct Bilirubin (mg/dL)	Direct Bilirubin (mg/dL)	Direct Bilirubin (mg/dL)	Direct Bilirubin (mg/dL)
	2	8	22	43	50	85	-43	2	8	22	43
301	0.22	0.11 E[a]	0.14 E[a]	0.16	-	-	0.08 E[a]	0.06 E[a]	0.04 E[a]	0.05 E[a]	0.04 E[a]
302	0.18	0.17	0.16	0.09 E[a]	-	-	0.07 E[a]	0.05 E[a]	0.06 E[a]	0.03 E[a]	0.04 E[a]
303	0.18	0.29	0.14 E[a]	0.33	0.20	0.24	0.08 E[a]	0.04 E[a]	0.08 E[a]	0.03 E[a]	0.08 E[a]
304	0.22	0.13 E[a]	0.07 E[a]	0.12 E[a]	0.11 E[a]	0.07 E[a]	0.06 E[a]	0.06 E[a]	0.04 E[a]	0.02 E[a]	0.03 E[a]
Mean	0.20	0.23	0.16	0.25	0.20	0.24	-	-	-	-	-
SD	0.02	0.08	-	0.12	-	-	-	-	-	-	-
Max	0.22	0.29	0.16	0.33	0.20	0.24	-	-	-	-	-
Min	0.18	0.17	0.16	0.16	0.20	0.24	-	-	-	-	-

E = Exclude
[a] [RC:< Linearity, FC:Result below linearity]

3/8/2024 1:41:07PM Page: 32

AmplifyBio Study Number A-579-01

Individual Animal Serum Chemistry Data

Sex: Male Day(s) Relative to Start Date

Group 3 High Total vg 1.2x10^14	Serum Chemistry										
	Direct Bilirubin (mg/dL)	Direct Bilirubin (mg/dL)	LDH (U/L)	LDH (U/L)	LDH (U/L)	LDH (U/L)	LDH (U/L)	LDH (U/L)	LDH (U/L)	Creatine Kinase (U/L)	Creatine Kinase (U/L)
	50	85	-43	2	8	22	43	50	85	-43	2
301	-	-	1600 E[b]	929	778	483 [d]	440	-	-	10753 E[b]	2801 E[b]
302	-	-	909	849	421	628 [d]	498	-	-	1901	1472
303	0.05 E[a]	0.05 E[a]	1302 E[b]	449	765 [d]	756 [d]	423 [d]	452 [d]	560	3238 E[b]	402
304	0.03 E[a]	0.02 E[a]	1124 E[b]	1037 E[c]	471 [d]	379	328 [d]	328	342	7367 E[b]	11417 E[b]
Mean	-	-	909	742	609	562	422	390	451	1901	937
SD	-	-	-	257	189	165	71	88	154	-	757
Max	-	-	909	929	778	756	498	452	560	1901	1472
Min	-	-	909	449	421	379	328	328	342	1901	402

E = Exclude
[a] [RC:< Linearity, FC:Result below linearity]
[b] [FC:Result above linearity]
[c] [RC:> Linearity, FC:Result above linearity]
[d] [RC:Serum index interference hemolysis]

3/8/2024 1:41:07PM Page: 33

AmplifyBio Study Number A-579-01

Individual Animal Serum Chemistry Data

Sex: Male Day(s) Relative to Start Date

Group 3 High Total vg 1.2x10^14	Serum Chemistry										
	Creatine Kinase (U/L)	Creatine Kinase (U/L)	Creatine Kinase (U/L)	Creatine Kinase (U/L)	Creatine Kinase (U/L)	Cholesterol (mg/dL)	Cholesterol (mg/dL)	Cholesterol (mg/dL)	Cholesterol (mg/dL)	Cholesterol (mg/dL)	Cholesterol (mg/dL)
	8	22	43	50	85	-43	2	8	22	43	50
301	267	314	739	-	-	140	158	186	199	175	-
302	451	4359 E[a]	1135	-	-	120	126	168	162	139	-
303	674	1170	452	203	475	135	137	161	159	140	133
304	279	188	201	255	616	132	117	146	165	145	129
Mean	418	557	632	229	546	132	135	165	171	150	131
SD	190	534	401	37	100	9	18	17	19	17	3
Max	674	1170	1135	255	616	140	158	186	199	175	133
Min	267	188	201	203	475	120	117	146	159	139	129

E = Exclude
[a] [FC:Result above linearity]

3/8/2024 1:41:07PM Page: 34

AmplifyBio Study Number A-579-01

Individual Animal Serum Chemistry Data

Sex: Male Day(s) Relative to Start Date

Group 3 High Total vg 1.2x10^14	Serum Chemistry										
	Cholesterol (mg/dL)	TRIG (mg/dL)	TRIG (mg/dL)	TRIG (mg/dL)	TRIG (mg/dL)	TRIG (mg/dL)	TRIG (mg/dL)	TRIG (mg/dL)	Blood Urea Nitrogen (mg/dL)	Blood Urea Nitrogen (mg/dL)	Blood Urea Nitrogen (mg/dL)
	85	-43	2	8	22	43	50	85	-43	2	8
301	-	43	27	35	49	49	-	-	20	12	14
302	-	74	38	62	54	51	-	-	17	17	23
303	102	44	32	48	41	55	47	40	30	14	31
304	119	61	32	68	62	72	68	40	19	13	19
Mean	111	56	32	53	52	57	58	40	22	14	22
SD	12	15	5	15	9	10	15	0	6	2	7
Max	119	74	38	68	62	72	68	40	30	17	31
Min	102	43	27	35	41	49	47	40	17	12	14

3/8/2024 1:41:07PM Page: 35

AmplifyBio Study Number A-579-01

Individual Animal Serum Chemistry Data

Sex: Male Day(s) Relative to Start Date

Group 3 High Total vg 1.2x10^14	Serum Chemistry										
	Blood Urea Nitrogen (mg/dL)	Blood Urea Nitrogen (mg/dL)	Blood Urea Nitrogen (mg/dL)	Blood Urea Nitrogen (mg/dL)	Creatinine (mg/dL)	Creatinine (mg/dL)	Creatinine (mg/dL)	Creatinine (mg/dL)	Creatinine (mg/dL)	Creatinine (mg/dL)	Creatinine (mg/dL)
	22	43	50	85	-43	2	8	22	43	50	85
301	18	17	-	-	0.8	0.9	0.6	0.8	0.6	-	-
302	29	14	-	-	0.8	0.8	1.1	0.9	0.5	-	-
303	21	29	25	16	0.7	0.7	0.7	0.7	0.8	0.6	0.6
304	15	19	19	9	0.7	0.8	0.7	0.6	0.7	0.7	0.5
Mean	21	20	22	13	0.8	0.8	0.8	0.8	0.7	0.7	0.6
SD	6	7	4	5	0.1	0.1	0.2	0.1	0.1	0.1	0.1
Max	29	29	25	16	0.8	0.9	1.1	0.9	0.8	0.7	0.6
Min	15	14	19	9	0.7	0.7	0.6	0.6	0.5	0.6	0.5

3/8/2024 1:41:07PM Page: 36

AmplifyBio Study Number A-579-01

Individual Animal Serum Chemistry Data

Sex: Male Day(s) Relative to Start Date

Group 3 High Total vg 1.2x10^14	Serum Chemistry										
	Total Protein (g/dL)	Total Protein (g/dL)	Total Protein (g/dL)	Total Protein (g/dL)	Total Protein (g/dL)	Total Protein (g/dL)	Total Protein (g/dL)	ALB (g/dL)	ALB (g/dL)	ALB (g/dL)	ALB (g/dL)
	-43	2	8	22	43	50	85	-43	2	8	22
301	7.1	7.7	7.5	7.8	7.0	-	-	4.1	4.0	4.0	4.5
302	6.9	7.4	7.9	7.9	6.9	-	-	4.1	4.5	4.5	4.7
303	6.7	7.1	7.2	7.0	7.0	6.9	5.7	4.2	4.4	4.4	4.5
304	6.6	6.9	6.4	6.7	6.6	6.6	5.5	4.3	4.2	4.0	4.3
Mean	6.8	7.3	7.3	7.4	6.9	6.8	5.6	4.2	4.3	4.2	4.5
SD	0.2	0.3	0.6	0.6	0.2	0.2	0.1	0.1	0.2	0.3	0.2
Max	7.1	7.7	7.9	7.9	7.0	6.9	5.7	4.3	4.5	4.5	4.7
Min	6.6	6.9	6.4	6.7	6.6	6.6	5.5	4.1	4.0	4.0	4.3

3/8/2024 1:41:07PM Page: 37

AmplifyBio Study Number A-579-01

Individual Animal Serum Chemistry Data

Sex: Male Day(s) Relative to Start Date

Group 3 High Total vg 1.2x10^14	Serum Chemistry										
	ALB (g/dL)	ALB (g/dL)	ALB (g/dL)	Globulin (g/dL)	Globulin (g/dL)	Globulin (g/dL)	Globulin (g/dL)	Globulin (g/dL)	Globulin (g/dL)	Globulin (g/dL)	A/G Ratio
	43	50	85	-43	2	8	22	43	50	85	-43
301	4.2	-	-	3.0	3.7	3.5	3.3	2.8	-	-	1.4
302	4.2	-	-	2.8	2.9	3.4	3.2	2.7	-	-	1.5
303	4.6	4.4	3.8	2.5	2.7	2.8	2.5	2.4	2.5	1.9	1.7
304	4.2	4.3	3.6	2.3	2.7	2.4	2.4	2.4	2.3	1.9	1.9
Mean	4.3	4.4	3.7	2.7	3.0	3.0	2.9	2.6	2.4	1.9	1.6
SD	0.2	0.1	0.1	0.3	0.5	0.5	0.5	0.2	0.1	0.0	0.2
Max	4.6	4.4	3.8	3.0	3.7	3.5	3.3	2.8	2.5	1.9	1.9
Min	4.2	4.3	3.6	2.3	2.7	2.4	2.4	2.4	2.3	1.9	1.4

3/8/2024 1:41:07PM Page: 38

AmplifyBio Study Number A-579-01

Individual Animal Serum Chemistry Data

Sex: Male Day(s) Relative to Start Date

Group 3 High Total vg 1.2x10^14	Serum Chemistry										
	A/G Ratio	A/G Ratio	A/G Ratio	A/G Ratio	A/G Ratio	A/G Ratio	Calcium (mg/dL)	Calcium (mg/dL)	Calcium (mg/dL)	Calcium (mg/dL)	Calcium (mg/dL)
	2	8	22	43	50	85	-43	2	8	22	43
301	1.1	1.1	1.4	1.5	-	-	9.5	10.4	10.6	10.8	9.9
302	1.6	1.3	1.5	1.6	-	-	10.1	10.6	10.7	11.0	10.7
303	1.6	1.6	1.8	1.9	1.8	2.0	10.8	10.9	11.0	11.4	10.9
304	1.6	1.7	1.8	1.8	1.9	1.9	10.0	10.7	10.1	10.8	10.5
Mean	1.5	1.4	1.6	1.7	1.9	2.0	10.1	10.7	10.6	11.0	10.5
SD	0.3	0.3	0.2	0.2	0.1	0.1	0.5	0.2	0.4	0.3	0.4
Max	1.6	1.7	1.8	1.9	1.9	2.0	10.8	10.9	11.0	11.4	10.9
Min	1.1	1.1	1.4	1.5	1.8	1.9	9.5	10.4	10.1	10.8	9.9

3/8/2024 1:41:07PM Page: 39

AmplifyBio Study Number A-579-01

Individual Animal Serum Chemistry Data

Sex: Male Day(s) Relative to Start Date

Group 3 High Total vg 1.2x10^14	Serum Chemistry										
	Calcium (mg/dL)	Calcium (mg/dL)	Phosphorus (mg/dL)	Phosphorus (mg/dL)	Phosphorus (mg/dL)	Phosphorus (mg/dL)	Phosphorus (mg/dL)	Phosphorus (mg/dL)	Phosphorus (mg/dL)	Sodium (mmol/L)	Sodium (mmol/L)
	50	85	-43	2	8	22	43	50	85	-43	2
301	-	-	6.2	7.4	8.1	9.5	8.1	-	-	148	152
302	-	-	4.2	5.9	6.3	7.3	7.5	-	-	151	144
303	10.8	10.4	6.0	7.2	7.9	8.5	8.2	8.0	7.0	145	148
304	10.6	9.6	5.4	6.3	6.8	7.2	6.8	7.6	7.4	153	151
Mean	10.7	10.0	5.5	6.7	7.3	8.1	7.7	7.8	7.2	149	149
SD	0.1	0.6	0.9	0.7	0.9	1.1	0.6	0.3	0.3	4	4
Max	10.8	10.4	6.2	7.4	8.1	9.5	8.2	8.0	7.4	153	152
Min	10.6	9.6	4.2	5.9	6.3	7.2	6.8	7.6	7.0	145	144

3/8/2024 1:41:07PM Page: 40

AmplifyBio Study Number A-579-01

Individual Animal Serum Chemistry Data

Sex: Male Day(s) Relative to Start Date

Group 3 High Total vg 1.2x10^14	Serum Chemistry										
	Sodium (mmol/L)	Sodium (mmol/L)	Sodium (mmol/L)	Sodium (mmol/L)	Sodium (mmol/L)	Chloride (mmol/L)	Chloride (mmol/L)	Chloride (mmol/L)	Chloride (mmol/L)	Chloride (mmol/L)	Chloride (mmol/L)
	8	22	43	50	85	-43	2	8	22	43	50
301	146	150	146	-	-	107	110	109	108	107	-
302	144	132	146	-	-	106	104	98	88	105	-
303	142	148	145	145	142	105	106	100	101	102	102
304	146	148	147	148	146	111	106	109	109	108	108
Mean	145	145	146	147	144	107	107	104	102	106	105
SD	2	8	1	2	3	3	3	6	10	3	4
Max	146	150	147	148	146	111	110	109	109	108	108
Min	142	132	145	145	142	105	104	98	88	102	102

3/8/2024 1:41:07PM Page: 41

AmplifyBio Study Number A-579-01

Individual Animal Serum Chemistry Data

Sex: Male Day(s) Relative to Start Date

Group 3 High Total vg 1.2x10^14	Serum Chemistry										
	Chloride (mmol/L)	Potassium (mmol/L)	Potassium (mmol/L)	Potassium (mmol/L)	Potassium (mmol/L)	Potassium (mmol/L)	Potassium (mmol/L)	Potassium (mmol/L)	Glucose (mg/dL)	Glucose (mg/dL)	Glucose (mg/dL)
	85	-43	2	8	22	43	50	85	-43	2	8
301	-	3.4	4.5	4.3	5.0	3.9	-	-	66	69	56
302	-	4.9	4.5	3.6	3.4	4.2	-	-	48	109	63
303	103	4.5	4.9	4.2	4.8	4.6	4.2	4.1	53	64	44
304	109	3.9	4.2	4.2	4.7	4.5	4.4	3.7	54	88	59
Mean	106	4.2	4.5	4.1	4.5	4.3	4.3	3.9	55	83	56
SD	4	0.7	0.3	0.3	0.7	0.3	0.1	0.3	8	20	8
Max	109	4.9	4.9	4.3	5.0	4.6	4.4	4.1	66	109	63
Min	103	3.4	4.2	3.6	3.4	3.9	4.2	3.7	48	64	44

3/8/2024 1:41:07PM Page: 42

AmplifyBio Study Number A-579-01

Individual Animal Serum Chemistry Data

Sex: Male Day(s) Relative to Start Date

Group 3 High Total vg 1.2x10^14	Serum Chemistry										
	Glucose (mg/dL)	Glucose (mg/dL)	Glucose (mg/dL)	Glucose (mg/dL)	GGT (U/L)	GGT (U/L)	GGT (U/L)	GGT (U/L)	GGT (U/L)	GGT (U/L)	GGT (U/L)
	22	43	50	85	-43	2	8	22	43	50	85
301	41	49	-	-	106	97	93	139	132	-	-
302	96	48	-	-	138	146	132	107	162	-	-
303	62	62	54	68	103	100	99	111	126	129	117
304	78	71	88	65	82	81	69	86	94	97	95
Mean	69	58	71	67	107	106	98	111	129	113	106
SD	23	11	24	2	23	28	26	22	28	23	16
Max	96	71	88	68	138	146	132	139	162	129	117
Min	41	48	54	65	82	81	69	86	94	97	95

3/8/2024 1:41:07PM Page: 43

AmplifyBio Study Number A-579-01

Individual Animal Serum Chemistry Data

Sex: Female Day(s) Relative to Start Date

Group 1 Control Total vg 0	Serum Chemistry										
	ALT (U/L)	ALT (U/L)	ALT (U/L)	ALT (U/L)	ALT (U/L)	ALT (U/L)	ALT (U/L)	AST (U/L)	AST (U/L)	AST (U/L)	AST (U/L)
	-41	2	8	22	43	50	85	-41	2	8	22
151	190	146	110	206	169	-	-	99	83	48	63
152	85 [a]	106	51	27	26	27	39	185 [a]	466	41	28
Mean	138	126	81	117	98	27	39	142	275	45	46
SD	74	28	42	127	101	-	-	61	271	5	25
Max	190	146	110	206	169	27	39	185	466	48	63
Min	85	106	51	27	26	27	39	99	83	41	28

[a] [RC:Serum index interference hemolysis]

3/8/2024 1:41:07PM Page: 44

AmplifyBio Study Number A-579-01

Individual Animal Serum Chemistry Data

Sex: Female Day(s) Relative to Start Date

Group 1 Control Total vg 0	Serum Chemistry										
	AST (U/L)	AST (U/L)	AST (U/L)	Alkaline Phosphatase (U/L)	Alkaline Phosphatase (U/L)	Alkaline Phosphatase (U/L)	Alkaline Phosphatase (U/L)	Alkaline Phosphatase (U/L)	Alkaline Phosphatase (U/L)	Alkaline Phosphatase (U/L)	Total Bilirubin (mg/dL)
	43	50	85	-41	2	8	22	43	50	85	-41
151	72	-	-	259	200	172	161	161	-	-	0.33
152	29	37 [a]	34	233	234	252	226	240	244	191	0.35
Mean	51	37	34	246	217	212	194	201	244	191	0.34
SD	30	-	-	18	24	57	46	56	-	-	0.01
Max	72	37	34	259	234	252	226	240	244	191	0.35
Min	29	37	34	233	200	172	161	161	244	191	0.33

[a] [RC:Serum index interference hemolysis]

3/8/2024 1:41:07PM Page: 45

AmplifyBio Study Number A-579-01

Individual Animal Serum Chemistry Data

Sex: Female Day(s) Relative to Start Date

Group 1 Control Total vg 0	Serum Chemistry										
	Total Bilirubin (mg/dL)	Total Bilirubin (mg/dL)	Total Bilirubin (mg/dL)	Total Bilirubin (mg/dL)	Total Bilirubin (mg/dL)	Total Bilirubin (mg/dL)	Direct Bilirubin (mg/dL)	Direct Bilirubin (mg/dL)	Direct Bilirubin (mg/dL)	Direct Bilirubin (mg/dL)	Direct Bilirubin (mg/dL)
	2	8	22	43	50	85	-41	2	8	22	43
151	0.14 E[a]	0.18	0.14 E[a]	0.18	-	-	0.07 E[a]	0.05 E[a]	0.04 E[a]	0.04 E[a]	0.05 E[a]
152	0.31	0.21	0.18	0.21	0.26	0.14 E[a]	0.07 E[a]	0.08 E[a]	0.05 E[a]	0.05 E[a]	0.07 E[a]
Mean	0.31	0.20	0.18	0.20	0.26	-	-	-	-	-	-
SD	-	0.02	-	0.02	-	-	-	-	-	-	-
Max	0.31	0.21	0.18	0.21	0.26	-	-	-	-	-	-
Min	0.31	0.18	0.18	0.18	0.26	-	-	-	-	-	-

E = Exclude
[a] [RC:< Linearity, FC:Result below linearity]

AmplifyBio Study Number A-579-01

Individual Animal Serum Chemistry Data

Sex: Female Day(s) Relative to Start Date

Group 1 Control Total vg 0	Serum Chemistry										
	Direct Bilirubin (mg/dL)	Direct Bilirubin (mg/dL)	LDH (U/L)	LDH (U/L)	LDH (U/L)	LDH (U/L)	LDH (U/L)	LDH (U/L)	LDH (U/L)	Creatine Kinase (U/L)	Creatine Kinase (U/L)
	50	85	-41	2	8	22	43	50	85	-41	2
151	-	-	1009 E[b]	484	631	608 [e]	608	-	-	4118 E[b]	541
152	0.05 E[a]	0.04 E[a]	2905 E[c]	5577 E[d]	580 [e]	524	491	538 [e]	474	8572 E	73517 E[x]
Mean	-	-	-	484	606	566	550	538	474	-	541
SD	-	-	-	-	36	59	83	-	-	-	-
Max	-	-	-	484	631	608	608	538	474	-	541
Min	-	-	-	484	580	524	491	538	474	-	541

E = Exclude; [x] = See Comments and Markers page
[a] [RC:< Linearity, FC:Result below linearity]
[b] [FC:Result above linearity]
[c] [RC:> Linearity]
[d] [RC:> Linearity, FC:Above linearity]
[e] [RC:Serum index interference hemolysis]

3/8/2024 1:41:07PM Page: 47

AmplifyBio Study Number A-579-01

Individual Animal Serum Chemistry Data

Sex: Female Day(s) Relative to Start Date

Group 1 Control Total vg 0	Serum Chemistry										
	Creatine Kinase (U/L)	Creatine Kinase (U/L)	Creatine Kinase (U/L)	Creatine Kinase (U/L)	Creatine Kinase (U/L)	Cholesterol (mg/dL)	Cholesterol (mg/dL)	Cholesterol (mg/dL)	Cholesterol (mg/dL)	Cholesterol (mg/dL)	Cholesterol (mg/dL)
	8	22	43	50	85	-41	2	8	22	43	50
151	296	629	2176 E[a]	-	-	97	84	115	114	106	-
152	1186	140	342	981	827	107	113	146	175	157	159
Mean	741	385	342	981	827	102	99	131	145	132	159
SD	629	346	-	-	-	7	21	22	43	36	-
Max	1186	629	342	981	827	107	113	146	175	157	159
Min	296	140	342	981	827	97	84	115	114	106	159

E = Exclude
[a] [FC:Result above linearity]

AmplifyBio Study Number A-579-01

Individual Animal Serum Chemistry Data

Sex: Female Day(s) Relative to Start Date

Group 1 Control Total vg 0	Serum Chemistry									
	Cholesterol (mg/dL)	TRIG (mg/dL)	TRIG (mg/dL)	TRIG (mg/dL)	TRIG (mg/dL)	TRIG (mg/dL)	TRIG (mg/dL)	TRIG (mg/dL)	Blood Urea Nitrogen (mg/dL)	Blood Urea Nitrogen (mg/dL)	Blood Urea Nitrogen (mg/dL)
	85	-41	2	8	22	43	50	85	-41	2	8
151	-	71	61	61	56	62	-	-	13	8	16
152	165	117	104	57	50	47	65	60	15	11	16
Mean	165	94	83	59	53	55	65	60	14	10	16
SD	-	33	30	3	4	11	-	-	1	2	0
Max	165	117	104	61	56	62	65	60	15	11	16
Min	165	71	61	57	50	47	65	60	13	8	16

3/8/2024 1:41:07PM Page: 49

AmplifyBio Study Number A-579-01

Individual Animal Serum Chemistry Data

Sex: Female Day(s) Relative to Start Date

Group 1 Control Total vg 0	Serum Chemistry										
	Blood Urea Nitrogen (mg/dL)	Blood Urea Nitrogen (mg/dL)	Blood Urea Nitrogen (mg/dL)	Blood Urea Nitrogen (mg/dL)	Creatinine (mg/dL)	Creatinine (mg/dL)	Creatinine (mg/dL)	Creatinine (mg/dL)	Creatinine (mg/dL)	Creatinine (mg/dL)	Creatinine (mg/dL)
	22	43	50	85	-41	2	8	22	43	50	85
151	19	16	-	-	0.7	0.8	0.6	0.7	0.6	-	-
152	17	16	15	13	0.7	0.7	0.6	0.8	0.7	0.7	0.6
Mean	18	16	15	13	0.7	0.8	0.6	0.8	0.7	0.7	0.6
SD	1	0	-	-	0.0	0.1	0.0	0.1	0.1	-	-
Max	19	16	15	13	0.7	0.8	0.6	0.8	0.7	0.7	0.6
Min	17	16	15	13	0.7	0.7	0.6	0.7	0.6	0.7	0.6

3/8/2024 1:41:07PM Page: 50

AmplifyBio Study Number A-579-01

Individual Animal Serum Chemistry Data

Sex: Female Day(s) Relative to Start Date

Group 1 Control Total vg 0	Serum Chemistry										
	Total Protein (g/dL)	Total Protein (g/dL)	Total Protein (g/dL)	Total Protein (g/dL)	Total Protein (g/dL)	Total Protein (g/dL)	Total Protein (g/dL)	ALB (g/dL)	ALB (g/dL)	ALB (g/dL)	ALB (g/dL)
	-41	2	8	22	43	50	85	-41	2	8	22
151	7.0	7.0	7.4	6.9	6.9	-	-	4.1	4.2	4.1	4.1
152	6.8	7.3	7.8	8.0	7.9	7.9	7.3	3.5	4.0	4.2	4.4
Mean	6.9	7.2	7.6	7.5	7.4	7.9	7.3	3.8	4.1	4.2	4.3
SD	0.1	0.2	0.3	0.8	0.7	-	-	0.4	0.1	0.1	0.2
Max	7.0	7.3	7.8	8.0	7.9	7.9	7.3	4.1	4.2	4.2	4.4
Min	6.8	7.0	7.4	6.9	6.9	7.9	7.3	3.5	4.0	4.1	4.1

3/8/2024 1:41:07PM Page: 51

AmplifyBio Study Number A-579-01

Individual Animal Serum Chemistry Data

Sex: Female Day(s) Relative to Start Date

Group 1 Control Total vg 0	Serum Chemistry										
	ALB (g/dL)	ALB (g/dL)	ALB (g/dL)	Globulin (g/dL)	Globulin (g/dL)	Globulin (g/dL)	Globulin (g/dL)	Globulin (g/dL)	Globulin (g/dL)	Globulin (g/dL)	A/G Ratio
	43	50	85	-41	2	8	22	43	50	85	-41
151	4.1	-	-	2.9	2.8	3.3	2.8	2.8	-	-	1.4
152	4.4	4.3	3.9	3.3	3.3	3.6	3.6	3.5	3.6	3.4	1.1
Mean	4.3	4.3	3.9	3.1	3.1	3.5	3.2	3.2	3.6	3.4	1.3
SD	0.2	-	-	0.3	0.4	0.2	0.6	0.5	-	-	0.2
Max	4.4	4.3	3.9	3.3	3.3	3.6	3.6	3.5	3.6	3.4	1.4
Min	4.1	4.3	3.9	2.9	2.8	3.3	2.8	2.8	3.6	3.4	1.1

AmplifyBio Study Number A-579-01

Individual Animal Serum Chemistry Data

Sex: Female Day(s) Relative to Start Date

Group 1 Control Total vg 0	Serum Chemistry										
	A/G Ratio	A/G Ratio	A/G Ratio	A/G Ratio	A/G Ratio	A/G Ratio	Calcium (mg/dL)	Calcium (mg/dL)	Calcium (mg/dL)	Calcium (mg/dL)	Calcium (mg/dL)
	2	8	22	43	50	85	-41	2	8	22	43
151	1.5	1.2	1.5	1.5	-	-	10.1	10.6	10.3	10.3	9.9
152	1.2	1.2	1.2	1.3	1.2	1.1	8.4	10.1	9.4	10.9	10.7
Mean	1.4	1.2	1.4	1.4	1.2	1.1	9.3	10.4	9.9	10.6	10.3
SD	0.2	0.0	0.2	0.1	-	-	1.2	0.4	0.6	0.4	0.6
Max	1.5	1.2	1.5	1.5	1.2	1.1	10.1	10.6	10.3	10.9	10.7
Min	1.2	1.2	1.2	1.3	1.2	1.1	8.4	10.1	9.4	10.3	9.9

AmplifyBio Study Number A-579-01

Individual Animal Serum Chemistry Data

Sex: Female Day(s) Relative to Start Date

Group 1 Control Total vg 0	Serum Chemistry										
	Calcium (mg/dL)	Calcium (mg/dL)	Phosphorus (mg/dL)	Phosphorus (mg/dL)	Phosphorus (mg/dL)	Phosphorus (mg/dL)	Phosphorus (mg/dL)	Phosphorus (mg/dL)	Phosphorus (mg/dL)	Sodium (mmol/L)	Sodium (mmol/L)
	50	85	-41	2	8	22	43	50	85	-41	2
151	-	-	4.4	6.3	5.9	5.7	5.5	-	-	148	155
152	10.2	9.9	3.9	1.9	5.4	5.9	3.7	5.9	5.1	148	145
Mean	10.2	9.9	4.2	4.1	5.7	5.8	4.6	5.9	5.1	148	150
SD	-	-	0.4	3.1	0.4	0.1	1.3	-	-	0	7
Max	10.2	9.9	4.4	6.3	5.9	5.9	5.5	5.9	5.1	148	155
Min	10.2	9.9	3.9	1.9	5.4	5.7	3.7	5.9	5.1	148	145

3/8/2024 1:41:07PM Page: 54

AmplifyBio Study Number A-579-01

Individual Animal Serum Chemistry Data

Sex: Female Day(s) Relative to Start Date

Group 1 Control Total vg 0	Serum Chemistry										
	Sodium (mmol/L)	Sodium (mmol/L)	Sodium (mmol/L)	Sodium (mmol/L)	Sodium (mmol/L)	Chloride (mmol/L)	Chloride (mmol/L)	Chloride (mmol/L)	Chloride (mmol/L)	Chloride (mmol/L)	Chloride (mmol/L)
	8	22	43	50	85	-41	2	8	22	43	50
151	144	147	146	-	-	111	111	107	105	108	-
152	144	145	145	144	145	109	109	107	104	108	110
Mean	144	146	146	144	145	110	110	107	105	108	110
SD	0	1	1	-	-	1	1	0	1	0	-
Max	144	147	146	144	145	111	111	107	105	108	110
Min	144	145	145	144	145	109	109	107	104	108	110

3/8/2024 1:41:07PM Page: 55

AmplifyBio Study Number A-579-01

Individual Animal Serum Chemistry Data

Sex: Female Day(s) Relative to Start Date

Group 1 Control Total vg 0	Serum Chemistry										
	Chloride (mmol/L)	Potassium (mmol/L)	Potassium (mmol/L)	Potassium (mmol/L)	Potassium (mmol/L)	Potassium (mmol/L)	Potassium (mmol/L)	Potassium (mmol/L)	Glucose (mg/dL)	Glucose (mg/dL)	Glucose (mg/dL)
	85	-41	2	8	22	43	50	85	-41	2	8
151	-	4.2	4.3	3.5	4.1	3.6	-	-	60	96	72
152	109	3.5	4.7	3.7	4.9	5.1	5.7	3.8	35	91	49
Mean	109	3.9	4.5	3.6	4.5	4.4	5.7	3.8	48	94	61
SD	-	0.5	0.3	0.1	0.6	1.1	-	-	18	4	16
Max	109	4.2	4.7	3.7	4.9	5.1	5.7	3.8	60	96	72
Min	109	3.5	4.3	3.5	4.1	3.6	5.7	3.8	35	91	49

3/8/2024 1:41:07PM Page: 56

AmplifyBio Study Number A-579-01

Individual Animal Serum Chemistry Data

Sex: Female Day(s) Relative to Start Date

Group 1 Control Total vg 0	Serum Chemistry										
	Glucose (mg/dL)	Glucose (mg/dL)	Glucose (mg/dL)	Glucose (mg/dL)	GGT (U/L)	GGT (U/L)	GGT (U/L)	GGT (U/L)	GGT (U/L)	GGT (U/L)	GGT (U/L)
	22	43	50	85	-41	2	8	22	43	50	85
151	73	58	-	-	68	64	67	64	71	-	-
152	53	50	57	54	33	36	33	44	41	37	43
Mean	63	54	57	54	51	50	50	54	56	37	43
SD	14	6	-	-	25	20	24	14	21	-	-
Max	73	58	57	54	68	64	67	64	71	37	43
Min	53	50	57	54	33	36	33	44	41	37	43

3/8/2024 1:41:07PM Page: 57

AmplifyBio Study Number A-579-01

Individual Animal Serum Chemistry Data

Sex: Female Day(s) Relative to Start Date

Group 2 Low Total vg 6x10^13	Serum Chemistry										
	ALT (U/L)	ALT (U/L)	ALT (U/L)	ALT (U/L)	ALT (U/L)	ALT (U/L)	ALT (U/L)	AST (U/L)	AST (U/L)	AST (U/L)	AST (U/L)
	-42	2	8	22	43	50	85	-42	2	8	22
251	94	116	76	54	46	-	-	87	118 [a]	60	47
252	56	113	66	60	66	-	-	94	247	66	63
253	97	131	71	83	70	64	50	119	63	54	40
254	89	115	51	66	57	59	59	56	198	34	50
Mean	84	119	66	66	60	62	55	89	157	54	50
SD	19	8	11	13	11	4	6	26	82	14	10
Max	97	131	76	83	70	64	59	119	247	66	63
Min	56	113	51	54	46	59	50	56	63	34	40

[a] [RC:Serum index interference hemolysis]

3/8/2024 1:41:07PM Page: 58

AmplifyBio Study Number A-579-01

Individual Animal Serum Chemistry Data

Sex: Female Day(s) Relative to Start Date

Group 2 Low Total vg 6x10^13	Serum Chemistry										
	AST (U/L)	AST (U/L)	AST (U/L)	Alkaline Phosphatase (U/L)	Alkaline Phosphatase (U/L)	Alkaline Phosphatase (U/L)	Alkaline Phosphatase (U/L)	Alkaline Phosphatase (U/L)	Alkaline Phosphatase (U/L)	Alkaline Phosphatase (U/L)	Total Bilirubin (mg/dL)
	43	50	85	-42	2	8	22	43	50	85	-42
251	35	-	-	551	384	327	361	288	-	-	0.27
252	55	-	-	399	297	231	263	201	-	-	0.28
253	44	39	36	273	205	182	182	195	187	161	0.37
254	35	35	48	543	370	301	357	321	345	335	0.35
Mean	42	37	42	442	314	260	291	251	266	248	0.32
SD	10	3	8	132	82	66	85	63	112	123	0.05
Max	55	39	48	551	384	327	361	321	345	335	0.37
Min	35	35	36	273	205	182	182	195	187	161	0.27

3/8/2024 1:41:07PM Page: 59

AmplifyBio Study Number A-579-01

Individual Animal Serum Chemistry Data

Sex: Female Day(s) Relative to Start Date

Group 2 Low Total vg 6x10^13	Serum Chemistry										
	Total Bilirubin (mg/dL)	Total Bilirubin (mg/dL)	Total Bilirubin (mg/dL)	Total Bilirubin (mg/dL)	Total Bilirubin (mg/dL)	Total Bilirubin (mg/dL)	Direct Bilirubin (mg/dL)	Direct Bilirubin (mg/dL)	Direct Bilirubin (mg/dL)	Direct Bilirubin (mg/dL)	Direct Bilirubin (mg/dL)
	2	8	22	43	50	85	-42	2	8	22	43
251	0.13 E[a]	0.21	0.10 E[b]	0.12 E[a]	-	-	0.06 E[a]	0.04 E[a]	0.06 E[c]	0.04 E[b]	0.03 E[a]
252	0.23	0.20	0.15 E[b]	0.15 E[a]	-	-	0.08 E[a]	0.07 E[a]	0.06 E[a]	0.04 E[b]	0.04 E[a]
253	0.19	0.24	0.23	0.26	0.18	0.17	0.08 E[a]	0.05 E[a]	0.07 E[a]	0.06 E[b]	0.07 E[a]
254	0.30	0.14 E[a]	0.14 E[b]	0.18	0.14 E[a]	0.22	0.07 E[a]	0.07 E[a]	0.04 E[a]	0.04 E[b]	0.05 E[a]
Mean	0.24	0.22	0.23	0.22	0.18	0.20	-	-	-	-	-
SD	0.06	0.02	-	0.06	-	0.04	-	-	-	-	-
Max	0.30	0.24	0.23	0.26	0.18	0.22	-	-	-	-	-
Min	0.19	0.20	0.23	0.18	0.18	0.17	-	-	-	-	-

E = Exclude
[a] [RC:< Linearity, FC:Result below linearity]
[b] [RC:< Linearity]
[c] [RC:< Linearity, FC:Results below linearity]

3/8/2024 1:41:07PM Page: 60

AmplifyBio Study Number A-579-01

Individual Animal Serum Chemistry Data

Sex: Female Day(s) Relative to Start Date

Group 2 Low Total vg 6x10^13	Serum Chemistry										
	Direct Bilirubin (mg/dL)	Direct Bilirubin (mg/dL)	LDH (U/L)	LDH (U/L)	LDH (U/L)	LDH (U/L)	LDH (U/L)	LDH (U/L)	LDH (U/L)	Creatine Kinase (U/L)	Creatine Kinase (U/L)
	50	85	-42	2	8	22	43	50	85	-42	2
251	-	-	829	959 [c]	809	556	333	-	-	1008	921
252	-	-	1214 E[b]	1446 E[d]	988	525 [c]	386	-	-	885	8739 E[b]
253	0.05 E[a]	0.05 E[a]	1599 E[b]	694 [c]	1130 E[b]	445	548	529	386 [c]	2578 E[b]	541
254	0.04 E[a]	0.05 E[a]	933	884	538	557	374	431	509 [c]	386	2734 E[e]
Mean	-	-	881	846	778	521	410	480	448	760	731
SD	-	-	74	137	227	53	95	69	87	329	269
Max	-	-	933	959	988	557	548	529	509	1008	921
Min	-	-	829	694	538	445	333	431	386	386	541

E = Exclude
[a] [RC:< Linearity, FC:Result below linearity]
[b] [FC:Result above linearity]
[c] [RC:Serum index interference hemolysis]
[d] [RC:> Linearity, FC:Result above linearity, reran to confirm]
[e] [FC:Result above linearity, within 20% of RSN1]

3/8/2024 1:41:07PM Page: 61

AmplifyBio Study Number A-579-01

Individual Animal Serum Chemistry Data

Sex: Female Day(s) Relative to Start Date

Group 2 Low Total vg 6x10^13	Serum Chemistry										
	Creatine Kinase (U/L)	Creatine Kinase (U/L)	Creatine Kinase (U/L)	Creatine Kinase (U/L)	Creatine Kinase (U/L)	Cholesterol (mg/dL)	Cholesterol (mg/dL)	Cholesterol (mg/dL)	Cholesterol (mg/dL)	Cholesterol (mg/dL)	Cholesterol (mg/dL)
	8	22	43	50	85	-42	2	8	22	43	50
251	1155	375	686	-	-	133	121	160	165	139	-
252	403	3127 E[a]	1682	-	-	124	116	151	162	135	-
253	219	431	1183	177	492	112	130	147	165	149	141
254	214	3064 E[a]	239	268	1373	152	137	172	184	160	144
Mean	498	403	948	223	933	130	126	158	169	146	143
SD	447	40	623	64	623	17	9	11	10	11	2
Max	1155	431	1682	268	1373	152	137	172	184	160	144
Min	214	375	239	177	492	112	116	147	162	135	141

E = Exclude
[a] [FC:Result above linearity]

3/8/2024 1:41:07PM Page: 62

AmplifyBio Study Number A-579-01

Individual Animal Serum Chemistry Data

Sex: Female Day(s) Relative to Start Date

Group 2 Low Total vg 6x10^13	Serum Chemistry										
	Cholesterol (mg/dL)	TRIG (mg/dL)	TRIG (mg/dL)	TRIG (mg/dL)	TRIG (mg/dL)	TRIG (mg/dL)	TRIG (mg/dL)	TRIG (mg/dL)	Blood Urea Nitrogen (mg/dL)	Blood Urea Nitrogen (mg/dL)	Blood Urea Nitrogen (mg/dL)
	85	-42	2	8	22	43	50	85	-42	2	8
251	-	38	54	34	34	41	-	-	18	13	19
252	-	44	35	27	37	38	-	-	17	11	15
253	132	60	82	42	51	41	73	45	13	9	14
254	159	68	49	62	50	62	79	48	15	12	19
Mean	146	53	55	41	43	46	76	47	16	11	17
SD	19	14	20	15	9	11	4	2	2	2	3
Max	159	68	82	62	51	62	79	48	18	13	19
Min	132	38	35	27	34	38	73	45	13	9	14

3/8/2024 1:41:07PM Page: 63

AmplifyBio Study Number A-579-01

Individual Animal Serum Chemistry Data

Sex: Female Day(s) Relative to Start Date

Group 2 Low Total vg 6x10^13	Serum Chemistry										
	Blood Urea Nitrogen (mg/dL)	Blood Urea Nitrogen (mg/dL)	Blood Urea Nitrogen (mg/dL)	Blood Urea Nitrogen (mg/dL)	Creatinine (mg/dL)	Creatinine (mg/dL)	Creatinine (mg/dL)	Creatinine (mg/dL)	Creatinine (mg/dL)	Creatinine (mg/dL)	Creatinine (mg/dL)
	22	43	50	85	-42	2	8	22	43	50	85
251	21	18	-	-	0.7	0.8	0.6	0.7	0.5	-	-
252	18	17	-	-	0.7	0.8	0.6	0.8	0.7	-	-
253	18	13	15	15	0.9	0.8	0.7	0.8	0.8	0.8	0.7
254	17	14	18	15	0.8	0.8	0.7	0.7	0.8	0.7	0.7
Mean	19	16	17	15	0.8	0.8	0.7	0.8	0.7	0.8	0.7
SD	2	2	2	0	0.1	0.0	0.1	0.1	0.1	0.1	0.0
Max	21	18	18	15	0.9	0.8	0.7	0.8	0.8	0.8	0.7
Min	17	13	15	15	0.7	0.8	0.6	0.7	0.5	0.7	0.7

AmplifyBio Study Number A-579-01

Individual Animal Serum Chemistry Data

Sex: Female Day(s) Relative to Start Date

Group 2 Low Total vg 6x10^13	Serum Chemistry										
	Total Protein (g/dL)	Total Protein (g/dL)	Total Protein (g/dL)	Total Protein (g/dL)	Total Protein (g/dL)	Total Protein (g/dL)	Total Protein (g/dL)	ALB (g/dL)	ALB (g/dL)	ALB (g/dL)	ALB (g/dL)
	-42	2	8	22	43	50	85	-42	2	8	22
251	6.4	6.8	6.8	7.2	6.4	-	-	3.6	3.9	3.9	4.3
252	7.3	7.8	7.5	8.3	7.2	-	-	4.4	4.7	4.5	5.0
253	7.1	7.3	7.3	7.6	7.5	7.4	6.3	3.7	4.0	4.0	4.2
254	6.7	6.9	7.0	7.1	7.1	7.0	6.7	3.8	3.8	3.7	3.9
Mean	6.9	7.2	7.2	7.6	7.1	7.2	6.5	3.9	4.1	4.0	4.4
SD	0.4	0.5	0.3	0.5	0.5	0.3	0.3	0.4	0.4	0.3	0.5
Max	7.3	7.8	7.5	8.3	7.5	7.4	6.7	4.4	4.7	4.5	5.0
Min	6.4	6.8	6.8	7.1	6.4	7.0	6.3	3.6	3.8	3.7	3.9

3/8/2024 1:41:07PM Page: 65

AmplifyBio Study Number A-579-01

Individual Animal Serum Chemistry Data

Sex: Female Day(s) Relative to Start Date

Group 2 Low Total vg 6x10^13	Serum Chemistry										
	ALB (g/dL)	ALB (g/dL)	ALB (g/dL)	Globulin (g/dL)	Globulin (g/dL)	Globulin (g/dL)	Globulin (g/dL)	Globulin (g/dL)	Globulin (g/dL)	Globulin (g/dL)	A/G Ratio
	43	50	85	-42	2	8	22	43	50	85	-42
251	3.8	-	-	2.8	2.9	2.9	2.9	2.6	-	-	1.3
252	4.3	-	-	2.9	3.1	3.0	3.3	2.9	-	-	1.5
253	4.2	4.1	3.5	3.4	3.3	3.3	3.4	3.3	3.3	2.8	1.1
254	4.0	4.0	3.8	2.9	3.1	3.3	3.2	3.1	3.0	2.9	1.3
Mean	4.1	4.1	3.7	3.0	3.1	3.1	3.2	3.0	3.2	2.9	1.3
SD	0.2	0.1	0.2	0.3	0.2	0.2	0.2	0.3	0.2	0.1	0.2
Max	4.3	4.1	3.8	3.4	3.3	3.3	3.4	3.3	3.3	2.9	1.5
Min	3.8	4.0	3.5	2.8	2.9	2.9	2.9	2.6	3.0	2.8	1.1

3/8/2024 1:41:07PM Page: 66

AmplifyBio Study Number A-579-01

Individual Animal Serum Chemistry Data

Sex: Female Day(s) Relative to Start Date

Group 2 Low Total vg 6x10^13	Serum Chemistry										
	A/G Ratio	A/G Ratio	A/G Ratio	A/G Ratio	A/G Ratio	A/G Ratio	Calcium (mg/dL)	Calcium (mg/dL)	Calcium (mg/dL)	Calcium (mg/dL)	Calcium (mg/dL)
	2	8	22	43	50	85	-42	2	8	22	43
251	1.3	1.3	1.5	1.5	-	-	9.4	10.2	9.8	10.3	9.9
252	1.5	1.5	1.5	1.5	-	-	10.0	11.7	10.6	11.8	10.4
253	1.2	1.2	1.2	1.3	1.2	1.3	9.7	10.5	10.2	10.9	10.5
254	1.2	1.1	1.2	1.3	1.3	1.3	10.1	11.0	9.9	11.0	10.4
Mean	1.3	1.3	1.4	1.4	1.3	1.3	9.8	10.9	10.1	11.0	10.3
SD	0.1	0.2	0.2	0.1	0.1	0.0	0.3	0.7	0.4	0.6	0.3
Max	1.5	1.5	1.5	1.5	1.3	1.3	10.1	11.7	10.6	11.8	10.5
Min	1.2	1.1	1.2	1.3	1.2	1.3	9.4	10.2	9.8	10.3	9.9

3/8/2024 1:41:07PM Page: 67

AmplifyBio Study Number A-579-01

Individual Animal Serum Chemistry Data

Sex: Female Day(s) Relative to Start Date

Group 2 Low Total vg 6x10^13	Serum Chemistry										
	Calcium (mg/dL)	Calcium (mg/dL)	Phosphorus (mg/dL)	Phosphorus (mg/dL)	Phosphorus (mg/dL)	Phosphorus (mg/dL)	Phosphorus (mg/dL)	Phosphorus (mg/dL)	Phosphorus (mg/dL)	Sodium (mmol/L)	Sodium (mmol/L)
	50	85	-42	2	8	22	43	50	85	-42	2
251	-	-	4.5	4.9	6.0	6.0	5.5	-	-	148	149
252	-	-	4.2	6.2	5.1	7.0	5.2	-	-	148	157
253	10.1	9.9	3.4	4.4	5.2	4.2	5.1	5.7	5.0	148	149
254	10.6	10.2	5.0	5.1	5.1	7.9	6.8	6.7	6.5	148	154
Mean	10.4	10.1	4.3	5.2	5.4	6.3	5.7	6.2	5.8	148	152
SD	0.4	0.2	0.7	0.8	0.4	1.6	0.8	0.7	1.1	0	4
Max	10.6	10.2	5.0	6.2	6.0	7.9	6.8	6.7	6.5	148	157
Min	10.1	9.9	3.4	4.4	5.1	4.2	5.1	5.7	5.0	148	149

3/8/2024 1:41:07PM Page: 68

AmplifyBio Study Number A-579-01

Individual Animal Serum Chemistry Data

Sex: Female Day(s) Relative to Start Date

Group 2 Low Total vg 6x10^13	Serum Chemistry										
	Sodium (mmol/L)	Sodium (mmol/L)	Sodium (mmol/L)	Sodium (mmol/L)	Sodium (mmol/L)	Chloride (mmol/L)	Chloride (mmol/L)	Chloride (mmol/L)	Chloride (mmol/L)	Chloride (mmol/L)	Chloride (mmol/L)
	8	22	43	50	85	-42	2	8	22	43	50
251	145	147	146	-	-	111	107	108	107	108	-
252	144	155	146	-	-	109	110	107	107	109	-
253	147	151	145	147	145	110	111	109	108	105	108
254	143	153	147	150	146	111	111	108	109	106	109
Mean	145	152	146	149	146	110	110	108	108	107	109
SD	2	3	1	2	1	1	2	1	1	2	1
Max	147	155	147	150	146	111	111	109	109	109	109
Min	143	147	145	147	145	109	107	107	107	105	108

3/8/2024 1:41:07PM Page: 69

AmplifyBio Study Number A-579-01

Individual Animal Serum Chemistry Data

Sex: Female Day(s) Relative to Start Date

Group 2 Low Total vg 6x10^13	Serum Chemistry										
	Chloride (mmol/L)	Potassium (mmol/L)	Potassium (mmol/L)	Potassium (mmol/L)	Potassium (mmol/L)	Potassium (mmol/L)	Potassium (mmol/L)	Potassium (mmol/L)	Glucose (mg/dL)	Glucose (mg/dL)	Glucose (mg/dL)
	85	-42	2	8	22	43	50	85	-42	2	8
251	-	3.8	4.5	3.8	4.0	3.8	-	-	37	57	63
252	-	4.8	5.6	4.4	5.2	4.3	-	-	37	79	45
253	108	4.0	4.2	3.5	4.2	4.3	4.0	3.5	54	86	47
254	109	4.1	4.8	3.5	4.4	4.5	4.6	4.4	57	91	74
Mean	109	4.2	4.8	3.8	4.5	4.2	4.3	4.0	46	78	57
SD	1	0.4	0.6	0.4	0.5	0.3	0.4	0.6	11	15	14
Max	109	4.8	5.6	4.4	5.2	4.5	4.6	4.4	57	91	74
Min	108	3.8	4.2	3.5	4.0	3.8	4.0	3.5	37	57	45

3/8/2024 1:41:07PM Page: 70

AmplifyBio Study Number A-579-01

Individual Animal Serum Chemistry Data

Sex: Female Day(s) Relative to Start Date

Group 2 Low Total vg 6x10^13	Serum Chemistry										
	Glucose (mg/dL)	Glucose (mg/dL)	Glucose (mg/dL)	Glucose (mg/dL)	GGT (U/L)	GGT (U/L)	GGT (U/L)	GGT (U/L)	GGT (U/L)	GGT (U/L)	GGT (U/L)
	22	43	50	85	-42	2	8	22	43	50	85
251	73	55	-	-	43	39	41	45	44	-	-
252	70	51	-	-	46	43	40	45	41	-	-
253	74	80	98	66	37	46	45	45	45	45	38
254	93	107	88	73	77	62	55	69	68	68	66
Mean	78	73	93	70	51	48	45	51	50	57	52
SD	10	26	7	5	18	10	7	12	12	16	20
Max	93	107	98	73	77	62	55	69	68	68	66
Min	70	51	88	66	37	39	40	45	41	45	38

3/8/2024 1:41:07PM Page: 71

AmplifyBio Study Number A-579-01

Individual Animal Serum Chemistry Data

Sex: Female Day(s) Relative to Start Date

Group 3 High Total vg 1.2x10^14	Serum Chemistry										
	ALT (U/L)	ALT (U/L)	ALT (U/L)	ALT (U/L)	ALT (U/L)	ALT (U/L)	ALT (U/L)	AST (U/L)	AST (U/L)	AST (U/L)	AST (U/L)
	-43	2	8	22	43	50	85	-43	2	8	22
351	24	34	39	28	22	-	-	66 [a]	68	46	50
352	73	69	51	54	59	-	-	100 [a]	125	44	52
353	147	39	33	30	21	24	17	252 [a]	69	39	38
354	121	107	104	104	86	255	64	93	73	52	57
Mean	91	62	57	54	47	140	41	128	84	45	49
SD	54	34	32	35	31	163	33	84	28	5	8
Max	147	107	104	104	86	255	64	252	125	52	57
Min	24	34	33	28	21	24	17	66	68	39	38

[a] [RC:Serum index interference hemolysis]

3/8/2024 1:41:07PM Page: 72

AmplifyBio Study Number A-579-01

Individual Animal Serum Chemistry Data

Sex: Female Day(s) Relative to Start Date

Group 3 High Total vg 1.2x10^14	Serum Chemistry										
	AST (U/L)	AST (U/L)	AST (U/L)	Alkaline Phosphatase (U/L)	Alkaline Phosphatase (U/L)	Alkaline Phosphatase (U/L)	Alkaline Phosphatase (U/L)	Alkaline Phosphatase (U/L)	Alkaline Phosphatase (U/L)	Alkaline Phosphatase (U/L)	Total Bilirubin (mg/dL)
	43	50	85	-43	2	8	22	43	50	85	-43
351	61	-	-	522	324	278	291	220	-	-	0.22
352	46	-	-	376	265	295	237	220	-	-	0.13 E[a]
353	33	30	32	377	276	221	260	283	251	262	0.56
354	59	86	40	497	365	315	316	294	303	328	0.21
Mean	50	58	36	443	308	277	276	254	277	295	0.33
SD	13	40	6	77	46	40	35	40	37	47	0.20
Max	61	86	40	522	365	315	316	294	303	328	0.56
Min	33	30	32	376	265	221	237	220	251	262	0.21

E = Exclude
[a] [RC:< Linearity, FC:Result below linearity]

3/8/2024 1:41:07PM Page: 73

AmplifyBio Study Number A-579-01

Individual Animal Serum Chemistry Data

Sex: Female Day(s) Relative to Start Date

Group 3 High Total vg 1.2x10^14	Serum Chemistry										
	Total Bilirubin (mg/dL)	Total Bilirubin (mg/dL)	Total Bilirubin (mg/dL)	Total Bilirubin (mg/dL)	Total Bilirubin (mg/dL)	Total Bilirubin (mg/dL)	Direct Bilirubin (mg/dL)	Direct Bilirubin (mg/dL)	Direct Bilirubin (mg/dL)	Direct Bilirubin (mg/dL)	Direct Bilirubin (mg/dL)
	2	8	22	43	50	85	-43	2	8	22	43
351	0.15	0.13 E[a]	0.08 E[a]	0.13 E[a]	-	-	0.05 E[a]	0.04 E[a]	0.04 E[a]	0.03 E[a]	0.04 E[a]
352	0.11 E[a]	0.14 E[a]	0.06 E[a]	0.12 E[a]	-	-	0.02 E[a]	0.03 E[a]	0.03 E[a]	0.02 E[a]	0.03 E[a]
353	0.14 E[a]	0.26	0.18	0.16	0.22	0.09 E[a]	0.10 E[a]	0.04 E[a]	0.07 E[a]	0.04 E[a]	0.04 E[a]
354	0.12 E[a]	0.27	0.07 E[a]	0.17	0.11 E[a]	0.21	0.05 E[a]	0.03 E[a]	0.06 E[a]	0.03 E[a]	0.04 E[a]
Mean	0.15	0.27	0.18	0.17	0.22	0.21	-	-	-	-	-
SD	-	0.01	-	0.01	-	-	-	-	-	-	-
Max	0.15	0.27	0.18	0.17	0.22	0.21	-	-	-	-	-
Min	0.15	0.26	0.18	0.16	0.22	0.21	-	-	-	-	-

E = Exclude
[a] [RC:< Linearity, FC:Result below linearity]

3/8/2024 1:41:07PM Page: 74

AmplifyBio Study Number A-579-01

Individual Animal Serum Chemistry Data

Sex: Female Day(s) Relative to Start Date

Group 3 High Total vg 1.2x10^14	Serum Chemistry										
	Direct Bilirubin (mg/dL)	Direct Bilirubin (mg/dL)	LDH (U/L)	LDH (U/L)	LDH (U/L)	LDH (U/L)	LDH (U/L)	LDH (U/L)	LDH (U/L)	Creatine Kinase (U/L)	Creatine Kinase (U/L)
	50	85	-43	2	8	22	43	50	85	-43	2
351	-	-	825	845	786	681	975	-	-	631	655
352	-	-	872	881	546 [c]	610	553	-	-	759	1416
353	0.06 E[a]	0.02 E[a]	1335 E[b]	665 [c]	538	554	394	416	402	6390 E[b]	1245
354	0.04 E[a]	0.04 E[a]	1112 E[b]	683	488 [c]	926	975 [c]	749	563	570	1880
Mean	-	-	849	769	590	693	724	583	483	653	1299
SD	-	-	33	110	133	164	297	235	114	96	506
Max	-	-	872	881	786	926	975	749	563	759	1880
Min	-	-	825	665	488	554	394	416	402	570	655

E = Exclude
[a] [RC:< Linearity, FC:Result below linearity]
[b] [FC:Result above linearity]
[c] [RC:Serum index interference hemolysis]

3/8/2024 1:41:07PM Page: 75

AmplifyBio Study Number A-579-01

Individual Animal Serum Chemistry Data

Sex: Female Day(s) Relative to Start Date

Group 3 High Total vg 1.2x10^14	Serum Chemistry										
	Creatine Kinase (U/L)	Creatine Kinase (U/L)	Creatine Kinase (U/L)	Creatine Kinase (U/L)	Creatine Kinase (U/L)	Cholesterol (mg/dL)	Cholesterol (mg/dL)	Cholesterol (mg/dL)	Cholesterol (mg/dL)	Cholesterol (mg/dL)	Cholesterol (mg/dL)
	8	22	43	50	85	-43	2	8	22	43	50
351	346	606	4313 E[a]	-	-	157	137	186	196	180	-
352	466	678	528	-	-	131	137	188	186	158	-
353	271	222	583	337	883	174	112	177	189	207	194
354	337	272	339	310	599	140	141	161	159	166	162
Mean	355	445	483	324	741	151	132	178	183	178	178
SD	81	231	128	19	201	19	13	12	16	22	23
Max	466	678	583	337	883	174	141	188	196	207	194
Min	271	222	339	310	599	131	112	161	159	158	162

E = Exclude
[a] [FC:Result above linearity]

3/8/2024 1:41:07PM Page: 76

AmplifyBio Study Number A-579-01

Individual Animal Serum Chemistry Data

Sex: Female Day(s) Relative to Start Date

Group 3 High Total vg 1.2x10^14	Serum Chemistry										
	Cholesterol (mg/dL)	TRIG (mg/dL)	TRIG (mg/dL)	TRIG (mg/dL)	TRIG (mg/dL)	TRIG (mg/dL)	TRIG (mg/dL)	TRIG (mg/dL)	Blood Urea Nitrogen (mg/dL)	Blood Urea Nitrogen (mg/dL)	Blood Urea Nitrogen (mg/dL)
	85	-43	2	8	22	43	50	85	-43	2	8
351	-	83	64	68	47	83	-	-	25	12	16
352	-	52	138	91	54	63	-	-	21	10	15
353	139	40	95	50	72	41	37	39	22	12	17
354	146	41	47	53	50	54	33	37	16	9	17
Mean	143	54	86	66	56	60	35	38	21	11	16
SD	5	20	40	19	11	18	3	1	4	2	1
Max	146	83	138	91	72	83	37	39	25	12	17
Min	139	40	47	50	47	41	33	37	16	9	15

3/8/2024 1:41:07PM Page: 77

AmplifyBio Study Number A-579-01

Individual Animal Serum Chemistry Data

Sex: Female Day(s) Relative to Start Date

Group 3 High Total vg 1.2x10^14	Serum Chemistry										
	Blood Urea Nitrogen (mg/dL)	Blood Urea Nitrogen (mg/dL)	Blood Urea Nitrogen (mg/dL)	Blood Urea Nitrogen (mg/dL)	Creatinine (mg/dL)	Creatinine (mg/dL)	Creatinine (mg/dL)	Creatinine (mg/dL)	Creatinine (mg/dL)	Creatinine (mg/dL)	Creatinine (mg/dL)
	22	43	50	85	-43	2	8	22	43	50	85
351	18	19	-	-	0.9	0.8	0.8	0.8	0.6	-	-
352	16	18	-	-	0.7	0.7	0.6	0.7	0.5	-	-
353	21	16	22	10	0.9	0.9	0.7	0.7	0.7	0.8	0.7
354	16	15	18	12	0.6	0.6	0.7	0.6	0.6	0.6	0.6
Mean	18	17	20	11	0.8	0.8	0.7	0.7	0.6	0.7	0.7
SD	2	2	3	1	0.2	0.1	0.1	0.1	0.1	0.1	0.1
Max	21	19	22	12	0.9	0.9	0.8	0.8	0.7	0.8	0.7
Min	16	15	18	10	0.6	0.6	0.6	0.6	0.5	0.6	0.6

AmplifyBio Study Number A-579-01

Individual Animal Serum Chemistry Data

Sex: Female Day(s) Relative to Start Date

Group 3 High Total vg 1.2x10^14	Serum Chemistry Total Protein (g/dL)	Total Protein (g/dL)	Total Protein (g/dL)	Total Protein (g/dL)	Total Protein (g/dL)	Total Protein (g/dL)	Total Protein (g/dL)	ALB (g/dL)	ALB (g/dL)	ALB (g/dL)	ALB (g/dL)
	-43	2	8	22	43	50	85	-43	2	8	22
351	6.8	6.4	6.9	6.9	6.6	-	-	3.8	3.7	3.8	4.1
352	7.1	7.3	7.4	7.5	7.0	-	-	3.7	4.2	4.0	4.4
353	6.3	6.2	6.3	6.5	6.7	7.0	6.5	4.1	3.0	3.6	4.0
354	7.4	7.4	7.4	7.4	7.6	7.5	6.9	4.4	4.5	4.5	4.6
Mean	6.9	6.8	7.0	7.1	7.0	7.3	6.7	4.0	3.9	4.0	4.3
SD	0.5	0.6	0.5	0.5	0.5	0.4	0.3	0.3	0.7	0.4	0.3
Max	7.4	7.4	7.4	7.5	7.6	7.5	6.9	4.4	4.5	4.5	4.6
Min	6.3	6.2	6.3	6.5	6.6	7.0	6.5	3.7	3.0	3.6	4.0

3/8/2024 1:41:07PM Page: 79

AmplifyBio Study Number A-579-01

Individual Animal Serum Chemistry Data

Sex: Female Day(s) Relative to Start Date

Group 3 High Total vg 1.2x10^14	Serum Chemistry										
	ALB (g/dL)	ALB (g/dL)	ALB (g/dL)	Globulin (g/dL)	Globulin (g/dL)	Globulin (g/dL)	Globulin (g/dL)	Globulin (g/dL)	Globulin (g/dL)	Globulin (g/dL)	A/G Ratio
	43	50	85	-43	2	8	22	43	50	85	-43
351	3.7	-	-	3.0	2.7	3.1	2.8	2.9	-	-	1.3
352	4.1	-	-	3.4	3.1	3.4	3.1	2.9	-	-	1.1
353	3.5	3.9	2.7	2.2	3.2	2.7	2.5	3.2	3.1	3.8	1.9
354	4.6	4.6	4.1	3.0	2.9	2.9	2.8	3.0	2.9	2.8	1.5
Mean	4.0	4.3	3.4	2.9	3.0	3.0	2.8	3.0	3.0	3.3	1.5
SD	0.5	0.5	1.0	0.5	0.2	0.3	0.2	0.1	0.1	0.7	0.3
Max	4.6	4.6	4.1	3.4	3.2	3.4	3.1	3.2	3.1	3.8	1.9
Min	3.5	3.9	2.7	2.2	2.7	2.7	2.5	2.9	2.9	2.8	1.1

3/8/2024 1:41:07PM Page: 80

AmplifyBio Study Number A-579-01

Individual Animal Serum Chemistry Data

Sex: Female Day(s) Relative to Start Date

Group 3 High Total vg 1.2x10^14	Serum Chemistry										
	A/G Ratio	A/G Ratio	A/G Ratio	A/G Ratio	A/G Ratio	A/G Ratio	Calcium (mg/dL)	Calcium (mg/dL)	Calcium (mg/dL)	Calcium (mg/dL)	Calcium (mg/dL)
	2	8	22	43	50	85	-43	2	8	22	43
351	1.4	1.2	1.5	1.3	-	-	9.6	9.6	9.7	10.2	9.5
352	1.4	1.2	1.4	1.4	-	-	9.2	10.1	9.7	10.5	9.5
353	0.9	1.3	1.6	1.1	1.3	0.7	9.7	10.3	9.7	10.2	9.8
354	1.6	1.6	1.6	1.5	1.6	1.5	10.3	10.7	10.3	10.7	10.5
Mean	1.3	1.3	1.5	1.3	1.5	1.1	9.7	10.2	9.9	10.4	9.8
SD	0.3	0.2	0.1	0.2	0.2	0.6	0.5	0.5	0.3	0.2	0.5
Max	1.6	1.6	1.6	1.5	1.6	1.5	10.3	10.7	10.3	10.7	10.5
Min	0.9	1.2	1.4	1.1	1.3	0.7	9.2	9.6	9.7	10.2	9.5

AmplifyBio Study Number A-579-01

Individual Animal Serum Chemistry Data

Sex: Female Day(s) Relative to Start Date

Group 3 High Total vg 1.2x10^14	Serum Chemistry										
	Calcium (mg/dL)	Calcium (mg/dL)	Phosphorus (mg/dL)	Phosphorus (mg/dL)	Phosphorus (mg/dL)	Phosphorus (mg/dL)	Phosphorus (mg/dL)	Phosphorus (mg/dL)	Phosphorus (mg/dL)	Sodium (mmol/L)	Sodium (mmol/L)
	50	85	-43	2	8	22	43	50	85	-43	2
351	-	-	4.3	4.7	4.6	5.2	5.8	-	-	147	144
352	-	-	4.4	5.8	5.7	6.4	6.9	-	-	146	147
353	9.7	9.0	5.4	4.3	5.7	5.4	4.0	5.1	4.8	147	144
354	10.2	9.8	6.0	7.2	6.8	6.6	6.9	6.8	5.5	150	151
Mean	10.0	9.4	5.0	5.5	5.7	5.9	5.9	6.0	5.2	148	147
SD	0.4	0.6	0.8	1.3	0.9	0.7	1.4	1.2	0.5	2	3
Max	10.2	9.8	6.0	7.2	6.8	6.6	6.9	6.8	5.5	150	151
Min	9.7	9.0	4.3	4.3	4.6	5.2	4.0	5.1	4.8	146	144

3/8/2024 1:41:07PM Page: 82

AmplifyBio Study Number A-579-01

Individual Animal Serum Chemistry Data

Sex: Female Day(s) Relative to Start Date

Group 3 High Total vg 1.2x10^14	Serum Chemistry										
	Sodium (mmol/L)	Sodium (mmol/L)	Sodium (mmol/L)	Sodium (mmol/L)	Sodium (mmol/L)	Chloride (mmol/L)	Chloride (mmol/L)	Chloride (mmol/L)	Chloride (mmol/L)	Chloride (mmol/L)	Chloride (mmol/L)
	8	22	43	50	85	-43	2	8	22	43	50
351	143	144	142	-	-	110	113	108	105	109	-
352	145	148	142	-	-	113	109	108	108	108	-
353	146	149	147	145	142	109	101	107	108	109	105
354	145	149	146	145	142	109	110	106	107	109	108
Mean	145	148	144	145	142	110	108	107	107	109	107
SD	1	2	3	0	0	2	5	1	1	1	2
Max	146	149	147	145	142	113	113	108	108	109	108
Min	143	144	142	145	142	109	101	106	105	108	105

AmplifyBio Study Number A-579-01

Individual Animal Serum Chemistry Data

Sex: Female Day(s) Relative to Start Date

Group 3 High Total vg 1.2x10^14	Serum Chemistry										
	Chloride (mmol/L)	Potassium (mmol/L)	Potassium (mmol/L)	Potassium (mmol/L)	Potassium (mmol/L)	Potassium (mmol/L)	Potassium (mmol/L)	Potassium (mmol/L)	Glucose (mg/dL)	Glucose (mg/dL)	Glucose (mg/dL)
	85	-43	2	8	22	43	50	85	-43	2	8
351	-	3.8	4.8	4.1	4.3	4.4	-	-	54	81	52
352	-	4.2	4.0	3.9	3.9	3.8	-	-	29	73	48
353	105	4.4	4.5	3.6	4.4	3.9	3.9	3.5	56	87	49
354	106	4.6	5.0	3.8	4.4	4.3	4.0	3.4	44	72	57
Mean	106	4.3	4.6	3.9	4.3	4.1	4.0	3.5	46	78	52
SD	1	0.3	0.4	0.2	0.2	0.3	0.1	0.1	12	7	4
Max	106	4.6	5.0	4.1	4.4	4.4	4.0	3.5	56	87	57
Min	105	3.8	4.0	3.6	3.9	3.8	3.9	3.4	29	72	48

3/8/2024 1:41:07PM Page: 84

AmplifyBio Study Number A-579-01

Individual Animal Serum Chemistry Data

Sex: Female Day(s) Relative to Start Date

Group 3 High Total vg 1.2x10^14	Serum Chemistry										
	Glucose (mg/dL)	Glucose (mg/dL)	Glucose (mg/dL)	Glucose (mg/dL)	GGT (U/L)	GGT (U/L)	GGT (U/L)	GGT (U/L)	GGT (U/L)	GGT (U/L)	GGT (U/L)
	22	43	50	85	-43	2	8	22	43	50	85
351	79	55	-	-	47	44	45	37	46	-	-
352	76	49	-	-	51	70	55	66	61	-	-
353	41	73	61	70	44	27	36	41	33	39	32
354	62	69	62	59	74	70	68	74	75	75	70
Mean	65	62	62	65	54	53	51	55	54	57	51
SD	17	11	1	8	14	21	14	18	18	25	27
Max	79	73	62	70	74	70	68	74	75	75	70
Min	41	49	61	59	44	27	36	37	33	39	32

3/8/2024 1:41:07PM Page: 85

AmplifyBio Study Number A-579-01

Individual Animal Serum Chemistry Data

Comments and Markers

Page	Day	Group	Sex	Subject	Measurement	Type	Marker
1	-41	1	Male	101	AST	Result	
				Comment: Serum index interference hemolysis			
2	-41	1	Male	102	TBILI	Quality Flag	E (Exclude)
				Comment: Result below linearity			
2	-41	1	Male	102	TBILI	Result	
				Comment: < Linearity			
3	-41	1	Male	101	DBILI	Quality Flag	E (Exclude)
				Comment: Result below linearity			
3	-41	1	Male	101	DBILI	Result	
				Comment: < Linearity			
3	-41	1	Male	102	DBILI	Quality Flag	E (Exclude)
				Comment: Result below linearity			
3	-41	1	Male	102	DBILI	Result	
				Comment: < Linearity			
3	2	1	Male	101	TBILI	Quality Flag	E (Exclude)
				Comment: Result below linearity			
3	2	1	Male	101	TBILI	Result	
				Comment: < Linearity			
3	2	1	Male	101	DBILI	Quality Flag	E (Exclude)
				Comment: Result below linearity			
3	2	1	Male	101	DBILI	Result	
				Comment: < Linearity			
3	2	1	Male	102	TBILI	Quality Flag	E (Exclude)
				Comment: Result below linearity			
3	2	1	Male	102	TBILI	Result	
				Comment: < Linearity			

3/8/2024 1:41:07PM Page: 86

AmplifyBio Study Number A-579-01

Individual Animal Serum Chemistry Data

Comments and Markers

Page	Day	Group	Sex	Subject	Measurement	Type	Marker
3	2	1	Male	102	DBILI	Quality Flag	E (Exclude)
				Comment: Result below linearity			
3	2	1	Male	102	DBILI	Result	
				Comment: < Linearity			
3	8	1	Male	101	DBILI	Quality Flag	E (Exclude)
				Comment: Result below linearity			
3	8	1	Male	101	DBILI	Result	
				Comment: < Linearity			
3	8	1	Male	102	DBILI	Quality Flag	E (Exclude)
				Comment: Result below linearity			
3	8	1	Male	102	DBILI	Result	
				Comment: < Linearity			
3	22	1	Male	101	TBILI	Quality Flag	E (Exclude)
				Comment: Result below linearity			
3	22	1	Male	101	TBILI	Result	
				Comment: < Linearity			
3	22	1	Male	101	DBILI	Quality Flag	E (Exclude)
				Comment: Result below linearity			
3	22	1	Male	101	DBILI	Result	
				Comment: < Linearity			
3	22	1	Male	102	DBILI	Quality Flag	E (Exclude)
				Comment: Result below linearity			
3	22	1	Male	102	DBILI	Result	
				Comment: < Linearity			
3	43	1	Male	101	TBILI	Quality Flag	E (Exclude)
				Comment: Result below linearity			
3	43	1	Male	101	TBILI	Result	
				Comment: < Linearity			

3/8/2024 1:41:07PM Page: 87

AmplifyBio Study Number A-579-01

Individual Animal Serum Chemistry Data

Comments and Markers

Page	Day	Group	Sex	Subject	Measurement	Type	Marker
3	43	1	Male	101	DBILI	Quality Flag	E (Exclude)
				Comment: Result below linearity			
3	43	1	Male	101	DBILI	Result	
				Comment: < Linearity			
3	43	1	Male	102	TBILI	Quality Flag	E (Exclude)
				Comment: Result below linearity			
3	43	1	Male	102	TBILI	Result	
				Comment: < Linearity			
3	43	1	Male	102	DBILI	Quality Flag	E (Exclude)
				Comment: Result below linearity			
3	43	1	Male	102	DBILI	Result	
				Comment: < Linearity			
3	50	1	Male	102	TBILI	Quality Flag	E (Exclude)
				Comment: Result below linearity			
3	50	1	Male	102	TBILI	Result	
				Comment: < Linearity			
3	85	1	Male	102	TBILI	Quality Flag	E (Exclude)
				Comment: Result below linearity			
3	85	1	Male	102	TBILI	Result	
				Comment: < Linearity			
4	-41	1	Male	102	LDH	Quality Flag	E (Exclude)
				Comment: Result above linearity			
4	2	1	Male	101	LDH	Result	
				Comment: Serum index interference hemolysis			
4	22	1	Male	102	LDH	Result	
				Comment: Serum index interference hemolysis			

3/8/2024 1:41:07PM Page: 88

AmplifyBio Study Number A-579-01

Individual Animal Serum Chemistry Data

Comments and Markers

Page	Day	Group	Sex	Subject	Measurement	Type	Marker
4	50	1	Male	102	DBILI	Quality Flag	E (Exclude)
				Comment: Result below linearity			
4	50	1	Male	102	DBILI	Result	
				Comment: < Linearity			
4	85	1	Male	102	DBILI	Quality Flag	E (Exclude)
				Comment: Result below linearity			
4	85	1	Male	102	DBILI	Result	
				Comment: < Linearity			
15	2	2	Male	202	AST	Result	
				Comment: Serum index interference hemolysis			
16	-42	2	Male	203	TBILI	Quality Flag	E (Exclude)
				Comment: Result below linearity			
16	-42	2	Male	203	TBILI	Result	
				Comment: < Linearity			
17	-42	2	Male	201	DBILI	Quality Flag	E (Exclude)
				Comment: Result below linearity			
17	-42	2	Male	201	DBILI	Result	
				Comment: < Linearity			
17	-42	2	Male	202	DBILI	Quality Flag	E (Exclude)
				Comment: Result below linearity			
17	-42	2	Male	202	DBILI	Result	
				Comment: < Linearity			
17	-42	2	Male	203	DBILI	Quality Flag	E (Exclude)
				Comment: Result below linearity			
17	-42	2	Male	203	DBILI	Result	
				Comment: < Linearity			

AmplifyBio Study Number A-579-01

Individual Animal Serum Chemistry Data

Comments and Markers

Page	Day	Group	Sex	Subject	Measurement	Type	Marker
17	-42	2	Male	204	DBILI	Quality Flag	E (Exclude)
				Comment: Result below linearity			
17	-42	2	Male	204	DBILI	Result	
				Comment: < Linearity			
17	2	2	Male	201	DBILI	Quality Flag	E (Exclude)
				Comment: Result below linearity			
17	2	2	Male	201	DBILI	Result	
				Comment: < Linearity			
17	2	2	Male	202	DBILI	Quality Flag	E (Exclude)
				Comment: Result below linearity			
17	2	2	Male	202	DBILI	Result	
				Comment: < Linearity			
17	2	2	Male	203	TBILI	Quality Flag	E (Exclude)
				Comment: Result below linearity			
17	2	2	Male	203	TBILI	Result	
				Comment: < Linearity			
17	2	2	Male	203	DBILI	Quality Flag	E (Exclude)
				Comment: Result below linearity			
17	2	2	Male	203	DBILI	Result	
				Comment: < Linearity			
17	2	2	Male	204	DBILI	Quality Flag	E (Exclude)
				Comment: Result below linearity			
17	2	2	Male	204	DBILI	Result	
				Comment: < Linearity			
17	8	2	Male	201	DBILI	Quality Flag	E (Exclude)
				Comment: Result below linearity			
17	8	2	Male	201	DBILI	Result	
				Comment: < Linearity			

AmplifyBio Study Number A-579-01

Individual Animal Serum Chemistry Data

Comments and Markers

Page	Day	Group	Sex	Subject	Measurement	Type	Marker
17	8	2	Male	202	DBILI	Quality Flag	E (Exclude)
				Comment: Result below linearity			
17	8	2	Male	202	DBILI	Result	
				Comment: < Linearity			
17	8	2	Male	203	DBILI	Quality Flag	E (Exclude)
				Comment: Result below linearity			
17	8	2	Male	203	DBILI	Result	
				Comment: < Linearity			
17	8	2	Male	204	DBILI	Quality Flag	E (Exclude)
				Comment: Results below linearity			
17	8	2	Male	204	DBILI	Result	
				Comment: < Linearity			
17	22	2	Male	201	TBILI	Quality Flag	E (Exclude)
17	22	2	Male	201	TBILI	Result	
				Comment: < Linearity			
17	22	2	Male	201	DBILI	Quality Flag	E (Exclude)
17	22	2	Male	201	DBILI	Result	
				Comment: < Linearity			
17	22	2	Male	202	DBILI	Quality Flag	E (Exclude)
17	22	2	Male	202	DBILI	Result	
				Comment: < Linearity			
17	22	2	Male	203	TBILI	Quality Flag	E (Exclude)
17	22	2	Male	203	TBILI	Result	
				Comment: < Linearity			
17	22	2	Male	203	DBILI	Quality Flag	E (Exclude)
17	22	2	Male	203	DBILI	Result	
				Comment: < Linearity			

3/8/2024 1:41:07PM Page: 91

AmplifyBio Study Number A-579-01

Individual Animal Serum Chemistry Data

Comments and Markers

Page	Day	Group	Sex	Subject	Measurement	Type	Marker
17	22	2	Male	204	DBILI	Quality Flag	E (Exclude)
17	22	2	Male	204	DBILI	Result	
				Comment: < Linearity			
17	43	2	Male	201	TBILI	Quality Flag	E (Exclude)
				Comment: Result below linearity			
17	43	2	Male	201	TBILI	Result	
				Comment: < Linearity			
17	43	2	Male	201	DBILI	Quality Flag	E (Exclude)
				Comment: Result below linearity			
17	43	2	Male	201	DBILI	Result	
				Comment: < Linearity			
17	43	2	Male	202	TBILI	Quality Flag	E (Exclude)
				Comment: Result below linearity			
17	43	2	Male	202	TBILI	Result	
				Comment: < Linearity			
17	43	2	Male	202	DBILI	Quality Flag	E (Exclude)
				Comment: Result below linearity			
17	43	2	Male	202	DBILI	Result	
				Comment: < Linearity			
17	43	2	Male	203	DBILI	Quality Flag	E (Exclude)
				Comment: Result below linearity			
17	43	2	Male	203	DBILI	Result	
				Comment: < Linearity			
17	43	2	Male	204	DBILI	Quality Flag	E (Exclude)
				Comment: Result below linearity			
17	43	2	Male	204	DBILI	Result	
				Comment: < Linearity			

3/8/2024 1:41:07PM Page: 92

AmplifyBio Study Number A-579-01

Individual Animal Serum Chemistry Data

Comments and Markers

Page	Day	Group	Sex	Subject	Measurement	Type	Marker
17	85	2	Male	203	TBILI	Quality Flag	E (Exclude)
				Comment: Result below linearity			
17	85	2	Male	203	TBILI	Result	
				Comment: < Linearity			
18	-42	2	Male	203	LDH	Quality Flag	E (Exclude)
				Comment: Result above linearity			
18	-42	2	Male	204	LDH	Quality Flag	E (Exclude)
				Comment: Result above linearity			
18	2	2	Male	201	CK	Quality Flag	E (Exclude)
				Comment: Result above linearity, within 20% of RSN1			
18	2	2	Male	202	LDH	Result	
				Comment: Serum index interference hemolysis			
18	2	2	Male	203	LDH	Quality Flag	E (Exclude)
				Comment: Result above linearity, reran to confirm			
18	2	2	Male	203	LDH	Result	
				Comment: > Linearity			
18	2	2	Male	204	LDH	Quality Flag	E (Exclude)
				Comment: Result above linearity, reran to confirm			
18	2	2	Male	204	LDH	Result	
				Comment: > Linearity			
18	2	2	Male	204	CK	Quality Flag	E (Exclude)
				Comment: Result above linearity, within 20% of RSN1			
18	8	2	Male	202	LDH	Result	
				Comment: Serum index interference hemolysis			
18	22	2	Male	201	LDH	Result	
				Comment: Serum index interference hemolysis			
18	22	2	Male	202	LDH	Result	
				Comment: Serum index interference hemolysis			

AmplifyBio Study Number A-579-01

Individual Animal Serum Chemistry Data

Comments and Markers

Page	Day	Group	Sex	Subject	Measurement	Type	Marker
18	22	2	Male	203	LDH	Result	
				Comment: Serum index interference hemolysis			
18	22	2	Male	204	LDH	Result	
				Comment: Serum index interference hemolysis			
18	43	2	Male	203	LDH	Quality Flag	E (Exclude)
				Comment: Result above linearity			
18	43	2	Male	204	LDH	Result	
				Comment: Serum index interference hemolysis			
18	50	2	Male	203	DBILI	Quality Flag	E (Exclude)
				Comment: Result below linearity			
18	50	2	Male	203	DBILI	Result	
				Comment: < Linearity			
18	50	2	Male	203	LDH	Result	
				Comment: Serum index interference hemolysis			
18	50	2	Male	204	DBILI	Quality Flag	E (Exclude)
				Comment: Result below linearity			
18	50	2	Male	204	DBILI	Result	
				Comment: < Linearity			
18	85	2	Male	203	DBILI	Quality Flag	E (Exclude)
				Comment: Result below linearity			
18	85	2	Male	203	DBILI	Result	
				Comment: < Linearity			
18	85	2	Male	203	LDH	Result	
				Comment: Serum index interference hemolysis			
18	85	2	Male	204	DBILI	Quality Flag	E (Exclude)
				Comment: Result below linearity			
18	85	2	Male	204	DBILI	Result	
				Comment: < Linearity			

AmplifyBio Study Number A-579-01

Individual Animal Serum Chemistry Data

Comments and Markers

Page	Day	Group	Sex	Subject	Measurement	Type	Marker
29	-43	3	Male	304	AST	Result	
				Comment: Serum index interference hemolysis			
31	-43	3	Male	301	DBILI	Quality Flag	E (Exclude)
				Comment: Result below linearity			
31	-43	3	Male	301	DBILI	Result	
				Comment: < Linearity			
31	-43	3	Male	302	DBILI	Quality Flag	E (Exclude)
				Comment: Result below linearity			
31	-43	3	Male	302	DBILI	Result	
				Comment: < Linearity			
31	-43	3	Male	303	DBILI	Quality Flag	E (Exclude)
				Comment: Result below linearity			
31	-43	3	Male	303	DBILI	Result	
				Comment: < Linearity			
31	-43	3	Male	304	DBILI	Quality Flag	E (Exclude)
				Comment: Result below linearity			
31	-43	3	Male	304	DBILI	Result	
				Comment: < Linearity			
31	2	3	Male	301	DBILI	Quality Flag	E (Exclude)
				Comment: Result below linearity			
31	2	3	Male	301	DBILI	Result	
				Comment: < Linearity			
31	2	3	Male	302	DBILI	Quality Flag	E (Exclude)
				Comment: Result below linearity			
31	2	3	Male	302	DBILI	Result	
				Comment: < Linearity			

3/8/2024 1:41:07PM Page: 95

AmplifyBio Study Number A-579-01

Individual Animal Serum Chemistry Data

Comments and Markers

Page	Day	Group	Sex	Subject	Measurement	Type	Marker
31	2	3	Male	303	DBILI	Quality Flag	E (Exclude)
				Comment: Result below linearity			
31	2	3	Male	303	DBILI	Result	
				Comment: < Linearity			
31	2	3	Male	304	DBILI	Quality Flag	E (Exclude)
				Comment: Result below linearity			
31	2	3	Male	304	DBILI	Result	
				Comment: < Linearity			
31	8	3	Male	301	TBILI	Quality Flag	E (Exclude)
				Comment: Result below linearity			
31	8	3	Male	301	TBILI	Result	
				Comment: < Linearity			
31	8	3	Male	301	DBILI	Quality Flag	E (Exclude)
				Comment: Result below linearity			
31	8	3	Male	301	DBILI	Result	
				Comment: < Linearity			
31	8	3	Male	302	DBILI	Quality Flag	E (Exclude)
				Comment: Result below linearity			
31	8	3	Male	302	DBILI	Result	
				Comment: < Linearity			
31	8	3	Male	303	DBILI	Quality Flag	E (Exclude)
				Comment: Result below linearity			
31	8	3	Male	303	DBILI	Result	
				Comment: < Linearity			
31	8	3	Male	304	TBILI	Quality Flag	E (Exclude)
				Comment: Result below linearity			
31	8	3	Male	304	TBILI	Result	
				Comment: < Linearity			

3/8/2024 1:41:07PM Page: 96

AmplifyBio Study Number A-579-01

Individual Animal Serum Chemistry Data

Comments and Markers

Page	Day	Group	Sex	Subject	Measurement	Type	Marker
31	8	3	Male	304	DBILI	Quality Flag	E (Exclude)
				Comment: Result below linearity			
31	8	3	Male	304	DBILI	Result	
				Comment: < Linearity			
31	22	3	Male	301	TBILI	Quality Flag	E (Exclude)
				Comment: Result below linearity			
31	22	3	Male	301	TBILI	Result	
				Comment: < Linearity			
31	22	3	Male	301	DBILI	Quality Flag	E (Exclude)
				Comment: Result below linearity			
31	22	3	Male	301	DBILI	Result	
				Comment: < Linearity			
31	22	3	Male	302	DBILI	Quality Flag	E (Exclude)
				Comment: Result below linearity			
31	22	3	Male	302	DBILI	Result	
				Comment: < Linearity			
31	22	3	Male	303	TBILI	Quality Flag	E (Exclude)
				Comment: Result below linearity			
31	22	3	Male	303	TBILI	Result	
				Comment: < Linearity			
31	22	3	Male	303	DBILI	Quality Flag	E (Exclude)
				Comment: Result below linearity			
31	22	3	Male	303	DBILI	Result	
				Comment: < Linearity			
31	22	3	Male	304	TBILI	Quality Flag	E (Exclude)
				Comment: Result below linearity			
31	22	3	Male	304	TBILI	Result	
				Comment: < Linearity			

AmplifyBio Study Number A-579-01

Individual Animal Serum Chemistry Data

Comments and Markers

Page	Day	Group	Sex	Subject	Measurement	Type	Marker
31	22	3	Male	304	DBILI	Quality Flag	E (Exclude)
				Comment: Result below linearity			
31	22	3	Male	304	DBILI	Result	
				Comment: < Linearity			
31	43	3	Male	301	DBILI	Quality Flag	E (Exclude)
				Comment: Result below linearity			
31	43	3	Male	301	DBILI	Result	
				Comment: < Linearity			
31	43	3	Male	302	TBILI	Quality Flag	E (Exclude)
				Comment: Result below linearity			
31	43	3	Male	302	TBILI	Result	
				Comment: < Linearity			
31	43	3	Male	302	DBILI	Quality Flag	E (Exclude)
				Comment: Result below linearity			
31	43	3	Male	302	DBILI	Result	
				Comment: < Linearity			
31	43	3	Male	303	DBILI	Quality Flag	E (Exclude)
				Comment: Result below linearity			
31	43	3	Male	303	DBILI	Result	
				Comment: < Linearity			
31	43	3	Male	304	TBILI	Quality Flag	E (Exclude)
				Comment: Result below linearity			
31	43	3	Male	304	TBILI	Result	
				Comment: < Linearity			
31	43	3	Male	304	DBILI	Quality Flag	E (Exclude)
				Comment: Result below linearity			
31	43	3	Male	304	DBILI	Result	
				Comment: < Linearity			

AmplifyBio Study Number A-579-01

Individual Animal Serum Chemistry Data

Comments and Markers

Page	Day	Group	Sex	Subject	Measurement	Type	Marker
31	50	3	Male	304	TBILI	Quality Flag	E (Exclude)
				Comment: Result below linearity			
31	50	3	Male	304	TBILI	Result	
				Comment: < Linearity			
31	85	3	Male	304	TBILI	Quality Flag	E (Exclude)
				Comment: Result below linearity			
31	85	3	Male	304	TBILI	Result	
				Comment: < Linearity			
32	-43	3	Male	301	LDH	Quality Flag	E (Exclude)
				Comment: Result above linearity			
32	-43	3	Male	301	CK	Quality Flag	E (Exclude)
				Comment: Result above linearity			
32	-43	3	Male	303	LDH	Quality Flag	E (Exclude)
				Comment: Result above linearity			
32	-43	3	Male	303	CK	Quality Flag	E (Exclude)
				Comment: Result above linearity			
32	-43	3	Male	304	LDH	Quality Flag	E (Exclude)
				Comment: Result above linearity			
32	-43	3	Male	304	CK	Quality Flag	E (Exclude)
				Comment: Result above linearity			
32	2	3	Male	301	CK	Quality Flag	E (Exclude)
				Comment: Result above linearity			
32	2	3	Male	304	LDH	Quality Flag	E (Exclude)
				Comment: Result above linearity			
32	2	3	Male	304	LDH	Result	
				Comment: > Linearity			
32	2	3	Male	304	CK	Quality Flag	E (Exclude)
				Comment: Result above linearity			

3/8/2024 1:41:07PM Page: 99

AmplifyBio Study Number A-579-01

Individual Animal Serum Chemistry Data

Comments and Markers

Page	Day	Group	Sex	Subject	Measurement	Type	Marker
32	8	3	Male	303	LDH	Result	
				Comment: Serum index interference hemolysis			
32	8	3	Male	304	LDH	Result	
				Comment: Serum index interference hemolysis			
32	22	3	Male	301	LDH	Result	
				Comment: Serum index interference hemolysis			
32	22	3	Male	302	LDH	Result	
				Comment: Serum index interference hemolysis			
32	22	3	Male	303	LDH	Result	
				Comment: Serum index interference hemolysis			
32	43	3	Male	303	LDH	Result	
				Comment: Serum index interference hemolysis			
32	43	3	Male	304	LDH	Result	
				Comment: Serum index interference hemolysis			
32	50	3	Male	303	DBILI	Quality Flag	E (Exclude)
				Comment: Result below linearity			
32	50	3	Male	303	DBILI	Result	
				Comment: < Linearity			
32	50	3	Male	303	LDH	Result	
				Comment: Serum index interference hemolysis			
32	50	3	Male	304	DBILI	Quality Flag	E (Exclude)
				Comment: Result below linearity			
32	50	3	Male	304	DBILI	Result	
				Comment: < Linearity			
32	85	3	Male	303	DBILI	Quality Flag	E (Exclude)
				Comment: Result below linearity			
32	85	3	Male	303	DBILI	Result	
				Comment: < Linearity			

AmplifyBio Study Number A-579-01

Individual Animal Serum Chemistry Data

Comments and Markers

Page	Day	Group	Sex	Subject	Measurement	Type	Marker
32	85	3	Male	304	DBILI	Quality Flag	E (Exclude)
				Comment: Result below linearity			
32	85	3	Male	304	DBILI	Result	
				Comment: < Linearity			
33	22	3	Male	302	CK	Quality Flag	E (Exclude)
				Comment: Result above linearity			
43	-41	1	Female	152	ALT	Result	
				Comment: Serum index interference hemolysis			
43	-41	1	Female	152	AST	Result	
				Comment: Serum index interference hemolysis			
44	50	1	Female	152	AST	Result	
				Comment: Serum index interference hemolysis			
45	-41	1	Female	151	DBILI	Quality Flag	E (Exclude)
				Comment: Result below linearity			
45	-41	1	Female	151	DBILI	Result	
				Comment: < Linearity			
45	-41	1	Female	152	DBILI	Quality Flag	E (Exclude)
				Comment: Result below linearity			
45	-41	1	Female	152	DBILI	Result	
				Comment: < Linearity			
45	2	1	Female	151	TBILI	Quality Flag	E (Exclude)
				Comment: Result below linearity			
45	2	1	Female	151	TBILI	Result	
				Comment: < Linearity			
45	2	1	Female	151	DBILI	Quality Flag	E (Exclude)
				Comment: Result below linearity			
45	2	1	Female	151	DBILI	Result	
				Comment: < Linearity			

AmplifyBio Study Number A-579-01

Individual Animal Serum Chemistry Data

Comments and Markers

Page	Day	Group	Sex	Subject	Measurement	Type	Marker
45	2	1	Female	152	DBILI	Quality Flag	E (Exclude)
				Comment: Result below linearity			
45	2	1	Female	152	DBILI	Result	
				Comment: < Linearity			
45	8	1	Female	151	DBILI	Quality Flag	E (Exclude)
				Comment: Result below linearity			
45	8	1	Female	151	DBILI	Result	
				Comment: < Linearity			
45	8	1	Female	152	DBILI	Quality Flag	E (Exclude)
				Comment: Result below linearity			
45	8	1	Female	152	DBILI	Result	
				Comment: < Linearity			
45	22	1	Female	151	TBILI	Quality Flag	E (Exclude)
				Comment: Result below linearity			
45	22	1	Female	151	TBILI	Result	
				Comment: < Linearity			
45	22	1	Female	151	DBILI	Quality Flag	E (Exclude)
				Comment: Result below linearity			
45	22	1	Female	151	DBILI	Result	
				Comment: < Linearity			
45	22	1	Female	152	DBILI	Quality Flag	E (Exclude)
				Comment: Result below linearity			
45	22	1	Female	152	DBILI	Result	
				Comment: < Linearity			
45	43	1	Female	151	DBILI	Quality Flag	E (Exclude)
				Comment: Result below linearity			
45	43	1	Female	151	DBILI	Result	
				Comment: < Linearity			

AmplifyBio Study Number A-579-01

Individual Animal Serum Chemistry Data

Comments and Markers

Page	Day	Group	Sex	Subject	Measurement	Type	Marker
45	43	1	Female	152	DBILI	Quality Flag	E (Exclude)
			Comment: Result below linearity				
45	43	1	Female	152	DBILI	Result	
			Comment: < Linearity				
45	85	1	Female	152	TBILI	Quality Flag	E (Exclude)
			Comment: Result below linearity				
45	85	1	Female	152	TBILI	Result	
			Comment: < Linearity				
46	-41	1	Female	151	LDH	Quality Flag	E (Exclude)
			Comment: Result above linearity				
46	-41	1	Female	151	CK	Quality Flag	E (Exclude)
			Comment: Result above linearity				
46	-41	1	Female	152	LDH	Quality Flag	E (Exclude)
46	-41	1	Female	152	LDH	Result	
			Comment: > Linearity				
46	-41	1	Female	152	CK	Quality Flag	E (Exclude)
46	2	1	Female	152	LDH	Quality Flag	E (Exclude)
			Comment: Above linearity				
46	2	1	Female	152	LDH	Result	
			Comment: > Linearity				
46	2	1	Female	152	CK	Quality Flag	E (Exclude)
			Comment: Result above linearity				
46	2	1	Female	152	CK	Result	
			Comment: > Linearity				
46	8	1	Female	152	LDH	Result	
			Comment: Serum index interference hemolysis				
46	22	1	Female	151	LDH	Result	
			Comment: Serum index interference hemolysis				

3/8/2024 1:41:07PM Page: 103

AmplifyBio Study Number A-579-01

Individual Animal Serum Chemistry Data

Comments and Markers

Page	Day	Group	Sex	Subject	Measurement	Type	Marker
46	50	1	Female	152	DBILI	Quality Flag	E (Exclude)
			Comment: Result below linearity				
46	50	1	Female	152	DBILI	Result	
			Comment: < Linearity				
46	50	1	Female	152	LDH	Result	
			Comment: Serum index interference hemolysis				
46	85	1	Female	152	DBILI	Quality Flag	E (Exclude)
			Comment: Result below linearity				
46	85	1	Female	152	DBILI	Result	
			Comment: < Linearity				
47	43	1	Female	151	CK	Quality Flag	E (Exclude)
			Comment: Result above linearity				
57	2	2	Female	251	AST	Result	
			Comment: Serum index interference hemolysis				
59	-42	2	Female	251	DBILI	Quality Flag	E (Exclude)
			Comment: Result below linearity				
59	-42	2	Female	251	DBILI	Result	
			Comment: < Linearity				
59	-42	2	Female	252	DBILI	Quality Flag	E (Exclude)
			Comment: Result below linearity				
59	-42	2	Female	252	DBILI	Result	
			Comment: < Linearity				
59	-42	2	Female	253	DBILI	Quality Flag	E (Exclude)
			Comment: Result below linearity				
59	-42	2	Female	253	DBILI	Result	
			Comment: < Linearity				

AmplifyBio Study Number A-579-01

Individual Animal Serum Chemistry Data

Comments and Markers

Page	Day	Group	Sex	Subject	Measurement	Type	Marker
59	-42	2	Female	254	DBILI	Quality Flag	E (Exclude)
				Comment: Result below linearity			
59	-42	2	Female	254	DBILI	Result	
				Comment: < Linearity			
59	2	2	Female	251	TBILI	Quality Flag	E (Exclude)
				Comment: Result below linearity			
59	2	2	Female	251	TBILI	Result	
				Comment: < Linearity			
59	2	2	Female	251	DBILI	Quality Flag	E (Exclude)
				Comment: Result below linearity			
59	2	2	Female	251	DBILI	Result	
				Comment: < Linearity			
59	2	2	Female	252	DBILI	Quality Flag	E (Exclude)
				Comment: Result below linearity			
59	2	2	Female	252	DBILI	Result	
				Comment: < Linearity			
59	2	2	Female	253	DBILI	Quality Flag	E (Exclude)
				Comment: Result below linearity			
59	2	2	Female	253	DBILI	Result	
				Comment: < Linearity			
59	2	2	Female	254	DBILI	Quality Flag	E (Exclude)
				Comment: Result below linearity			
59	2	2	Female	254	DBILI	Result	
				Comment: < Linearity			
59	8	2	Female	251	DBILI	Quality Flag	E (Exclude)
				Comment: Results below linearity			
59	8	2	Female	251	DBILI	Result	
				Comment: < Linearity			

3/8/2024 1:41:07PM Page: 105

AmplifyBio Study Number A-579-01

Individual Animal Serum Chemistry Data

Comments and Markers

Page	Day	Group	Sex	Subject	Measurement	Type	Marker
59	8	2	Female	252	DBILI	Quality Flag	E (Exclude)
			Comment: Result below linearity				
59	8	2	Female	252	DBILI	Result	
			Comment: < Linearity				
59	8	2	Female	253	DBILI	Quality Flag	E (Exclude)
			Comment: Result below linearity				
59	8	2	Female	253	DBILI	Result	
			Comment: < Linearity				
59	8	2	Female	254	TBILI	Quality Flag	E (Exclude)
			Comment: Result below linearity				
59	8	2	Female	254	TBILI	Result	
			Comment: < Linearity				
59	8	2	Female	254	DBILI	Quality Flag	E (Exclude)
			Comment: Result below linearity				
59	8	2	Female	254	DBILI	Result	
			Comment: < Linearity				
59	22	2	Female	251	TBILI	Quality Flag	E (Exclude)
59	22	2	Female	251	TBILI	Result	
			Comment: < Linearity				
59	22	2	Female	251	DBILI	Quality Flag	E (Exclude)
59	22	2	Female	251	DBILI	Result	
			Comment: < Linearity				
59	22	2	Female	252	TBILI	Quality Flag	E (Exclude)
59	22	2	Female	252	TBILI	Result	
			Comment: < Linearity				
59	22	2	Female	252	DBILI	Quality Flag	E (Exclude)
59	22	2	Female	252	DBILI	Result	
			Comment: < Linearity				

AmplifyBio Study Number A-579-01

Individual Animal Serum Chemistry Data

Comments and Markers

Page	Day	Group	Sex	Subject	Measurement	Type	Marker
59	22	2	Female	253	DBILI	Quality Flag	E (Exclude)
59	22	2	Female	253	DBILI	Result	
				Comment: < Linearity			
59	22	2	Female	254	TBILI	Quality Flag	E (Exclude)
59	22	2	Female	254	TBILI	Result	
				Comment: < Linearity			
59	22	2	Female	254	DBILI	Quality Flag	E (Exclude)
59	22	2	Female	254	DBILI	Result	
				Comment: < Linearity			
59	43	2	Female	251	TBILI	Quality Flag	E (Exclude)
				Comment: Result below linearity			
59	43	2	Female	251	TBILI	Result	
				Comment: < Linearity			
59	43	2	Female	251	DBILI	Quality Flag	E (Exclude)
				Comment: Result below linearity			
59	43	2	Female	251	DBILI	Result	
				Comment: < Linearity			
59	43	2	Female	252	TBILI	Quality Flag	E (Exclude)
				Comment: Result below linearity			
59	43	2	Female	252	TBILI	Result	
				Comment: < Linearity			
59	43	2	Female	252	DBILI	Quality Flag	E (Exclude)
				Comment: Result below linearity			
59	43	2	Female	252	DBILI	Result	
				Comment: < Linearity			
59	43	2	Female	253	DBILI	Quality Flag	E (Exclude)
				Comment: Result below linearity			
59	43	2	Female	253	DBILI	Result	
				Comment: < Linearity			

3/8/2024 1:41:07PM Page: 107

AmplifyBio Study Number A-579-01

Individual Animal Serum Chemistry Data

Comments and Markers

Page	Day	Group	Sex	Subject	Measurement	Type	Marker
59	43	2	Female	254	DBILI	Quality Flag	E (Exclude)
			Comment: Result below linearity				
59	43	2	Female	254	DBILI	Result	
			Comment: < Linearity				
59	50	2	Female	254	TBILI	Quality Flag	E (Exclude)
			Comment: Result below linearity				
59	50	2	Female	254	TBILI	Result	
			Comment: < Linearity				
60	-42	2	Female	252	LDH	Quality Flag	E (Exclude)
			Comment: Result above linearity				
60	-42	2	Female	253	LDH	Quality Flag	E (Exclude)
			Comment: Result above linearity				
60	-42	2	Female	253	CK	Quality Flag	E (Exclude)
			Comment: Result above linearity				
60	2	2	Female	251	LDH	Result	
			Comment: Serum index interference hemolysis				
60	2	2	Female	252	LDH	Quality Flag	E (Exclude)
			Comment: Result above linearity, reran to confirm				
60	2	2	Female	252	LDH	Result	
			Comment: > Linearity				
60	2	2	Female	252	CK	Quality Flag	E (Exclude)
			Comment: Result above linearity				
60	2	2	Female	253	LDH	Result	
			Comment: Serum index interference hemolysis				
60	2	2	Female	254	CK	Quality Flag	E (Exclude)
			Comment: Result above linearity, within 20% of RSN1				
60	8	2	Female	253	LDH	Quality Flag	E (Exclude)
			Comment: Result above linearity				

3/8/2024 1:41:07PM Page: 108

AmplifyBio Study Number A-579-01

Individual Animal Serum Chemistry Data

Comments and Markers

Page	Day	Group	Sex	Subject	Measurement	Type	Marker
60	22	2	Female	252	LDH	Result	
				Comment: Serum index interference hemolysis			
60	50	2	Female	253	DBILI	Quality Flag	E (Exclude)
				Comment: Result below linearity			
60	50	2	Female	253	DBILI	Result	
				Comment: < Linearity			
60	50	2	Female	254	DBILI	Quality Flag	E (Exclude)
				Comment: Result below linearity			
60	50	2	Female	254	DBILI	Result	
				Comment: < Linearity			
60	85	2	Female	253	DBILI	Quality Flag	E (Exclude)
				Comment: Result below linearity			
60	85	2	Female	253	DBILI	Result	
				Comment: < Linearity			
60	85	2	Female	253	LDH	Result	
				Comment: Serum index interference hemolysis			
60	85	2	Female	254	DBILI	Quality Flag	E (Exclude)
				Comment: Result below linearity			
60	85	2	Female	254	DBILI	Result	
				Comment: < Linearity			
60	85	2	Female	254	LDH	Result	
				Comment: Serum index interference hemolysis			
61	22	2	Female	252	CK	Quality Flag	E (Exclude)
				Comment: Result above linearity			
61	22	2	Female	254	CK	Quality Flag	E (Exclude)
				Comment: Result above linearity			
71	-43	3	Female	351	AST	Result	
				Comment: Serum index interference hemolysis			

3/8/2024 1:41:07PM Page: 109

AmplifyBio Study Number A-579-01

Individual Animal Serum Chemistry Data

Comments and Markers

Page	Day	Group	Sex	Subject	Measurement	Type	Marker
71	-43	3	Female	352	AST	Result	
				Comment: Serum index interference hemolysis			
71	-43	3	Female	353	AST	Result	
				Comment: Serum index interference hemolysis			
72	-43	3	Female	352	TBILI	Quality Flag	E (Exclude)
				Comment: Result below linearity			
72	-43	3	Female	352	TBILI	Result	
				Comment: < Linearity			
73	-43	3	Female	351	DBILI	Quality Flag	E (Exclude)
				Comment: Result below linearity			
73	-43	3	Female	351	DBILI	Result	
				Comment: < Linearity			
73	-43	3	Female	352	DBILI	Quality Flag	E (Exclude)
				Comment: Result below linearity			
73	-43	3	Female	352	DBILI	Result	
				Comment: < Linearity			
73	-43	3	Female	353	DBILI	Quality Flag	E (Exclude)
				Comment: Result below linearity			
73	-43	3	Female	353	DBILI	Result	
				Comment: < Linearity			
73	-43	3	Female	354	DBILI	Quality Flag	E (Exclude)
				Comment: Result below linearity			
73	-43	3	Female	354	DBILI	Result	
				Comment: < Linearity			
73	2	3	Female	351	DBILI	Quality Flag	E (Exclude)
				Comment: Result below linearity			
73	2	3	Female	351	DBILI	Result	
				Comment: < Linearity			

AmplifyBio Study Number A-579-01

Individual Animal Serum Chemistry Data

Comments and Markers

Page	Day	Group	Sex	Subject	Measurement	Type	Marker
73	2	3	Female	352	TBILI	Quality Flag	E (Exclude)
			Comment: Result below linearity				
73	2	3	Female	352	TBILI	Result	
			Comment: < Linearity				
73	2	3	Female	352	DBILI	Quality Flag	E (Exclude)
			Comment: Result below linearity				
73	2	3	Female	352	DBILI	Result	
			Comment: < Linearity				
73	2	3	Female	353	TBILI	Quality Flag	E (Exclude)
			Comment: Result below linearity				
73	2	3	Female	353	TBILI	Result	
			Comment: < Linearity				
73	2	3	Female	353	DBILI	Quality Flag	E (Exclude)
			Comment: Result below linearity				
73	2	3	Female	353	DBILI	Result	
			Comment: < Linearity				
73	2	3	Female	354	TBILI	Quality Flag	E (Exclude)
			Comment: Result below linearity				
73	2	3	Female	354	TBILI	Result	
			Comment: < Linearity				
73	2	3	Female	354	DBILI	Quality Flag	E (Exclude)
			Comment: Result below linearity				
73	2	3	Female	354	DBILI	Result	
			Comment: < Linearity				
73	8	3	Female	351	TBILI	Quality Flag	E (Exclude)
			Comment: Result below linearity				
73	8	3	Female	351	TBILI	Result	
			Comment: < Linearity				

3/8/2024 1:41:07PM Page: 111

AmplifyBio Study Number A-579-01

Individual Animal Serum Chemistry Data

Comments and Markers

Page	Day	Group	Sex	Subject	Measurement	Type	Marker
73	8	3	Female	351	DBILI	Quality Flag	E (Exclude)
				Comment: Result below linearity			
73	8	3	Female	351	DBILI	Result	
				Comment: < Linearity			
73	8	3	Female	352	TBILI	Quality Flag	E (Exclude)
				Comment: Result below linearity			
73	8	3	Female	352	TBILI	Result	
				Comment: < Linearity			
73	8	3	Female	352	DBILI	Quality Flag	E (Exclude)
				Comment: Result below linearity			
73	8	3	Female	352	DBILI	Result	
				Comment: < Linearity			
73	8	3	Female	353	DBILI	Quality Flag	E (Exclude)
				Comment: Result below linearity			
73	8	3	Female	353	DBILI	Result	
				Comment: < Linearity			
73	8	3	Female	354	DBILI	Quality Flag	E (Exclude)
				Comment: Result below linearity			
73	8	3	Female	354	DBILI	Result	
				Comment: < Linearity			
73	22	3	Female	351	TBILI	Quality Flag	E (Exclude)
				Comment: Result below linearity			
73	22	3	Female	351	TBILI	Result	
				Comment: < Linearity			
73	22	3	Female	351	DBILI	Quality Flag	E (Exclude)
				Comment: Result below linearity			
73	22	3	Female	351	DBILI	Result	
				Comment: < Linearity			

AmplifyBio Study Number A-579-01

Individual Animal Serum Chemistry Data

Comments and Markers

Page	Day	Group	Sex	Subject	Measurement	Type	Marker
73	22	3	Female	352	TBILI	Quality Flag	E (Exclude)
			Comment: Result below linearity				
73	22	3	Female	352	TBILI	Result	
			Comment: < Linearity				
73	22	3	Female	352	DBILI	Quality Flag	E (Exclude)
			Comment: Result below linearity				
73	22	3	Female	352	DBILI	Result	
			Comment: < Linearity				
73	22	3	Female	353	DBILI	Quality Flag	E (Exclude)
			Comment: Result below linearity				
73	22	3	Female	353	DBILI	Result	
			Comment: < Linearity				
73	22	3	Female	354	TBILI	Quality Flag	E (Exclude)
			Comment: Result below linearity				
73	22	3	Female	354	TBILI	Result	
			Comment: < Linearity				
73	22	3	Female	354	DBILI	Quality Flag	E (Exclude)
			Comment: Result below linearity				
73	22	3	Female	354	DBILI	Result	
			Comment: < Linearity				
73	43	3	Female	351	TBILI	Quality Flag	E (Exclude)
			Comment: Result below linearity				
73	43	3	Female	351	TBILI	Result	
			Comment: < Linearity				
73	43	3	Female	351	DBILI	Quality Flag	E (Exclude)
			Comment: Result below linearity				
73	43	3	Female	351	DBILI	Result	
			Comment: < Linearity				

AmplifyBio Study Number A-579-01

Individual Animal Serum Chemistry Data

Comments and Markers

Page	Day	Group	Sex	Subject	Measurement	Type	Marker
73	43	3	Female	352	TBILI	Quality Flag	E (Exclude)
			Comment: Result below linearity				
73	43	3	Female	352	TBILI	Result	
			Comment: < Linearity				
73	43	3	Female	352	DBILI	Quality Flag	E (Exclude)
			Comment: Result below linearity				
73	43	3	Female	352	DBILI	Result	
			Comment: < Linearity				
73	43	3	Female	353	DBILI	Quality Flag	E (Exclude)
			Comment: Result below linearity				
73	43	3	Female	353	DBILI	Result	
			Comment: < Linearity				
73	43	3	Female	354	DBILI	Quality Flag	E (Exclude)
			Comment: Result below linearity				
73	43	3	Female	354	DBILI	Result	
			Comment: < Linearity				
73	50	3	Female	354	TBILI	Quality Flag	E (Exclude)
			Comment: Result below linearity				
73	50	3	Female	354	TBILI	Result	
			Comment: < Linearity				
73	85	3	Female	353	TBILI	Quality Flag	E (Exclude)
			Comment: Result below linearity				
73	85	3	Female	353	TBILI	Result	
			Comment: < Linearity				
74	-43	3	Female	353	LDH	Quality Flag	E (Exclude)
			Comment: Result above linearity				
74	-43	3	Female	353	CK	Quality Flag	E (Exclude)
			Comment: Result above linearity				

3/8/2024 1:41:07PM Page: 114

AmplifyBio Study Number A-579-01

Individual Animal Serum Chemistry Data

Comments and Markers

Page	Day	Group	Sex	Subject	Measurement	Type	Marker
74	-43	3	Female	354	LDH	Quality Flag	E (Exclude)
				Comment: Result above linearity			
74	2	3	Female	353	LDH	Result	
				Comment: Serum index interference hemolysis			
74	8	3	Female	352	LDH	Result	
				Comment: Serum index interference hemolysis			
74	8	3	Female	354	LDH	Result	
				Comment: Serum index interference hemolysis			
74	43	3	Female	354	LDH	Result	
				Comment: Serum index interference hemolysis			
74	50	3	Female	353	DBILI	Quality Flag	E (Exclude)
				Comment: Result below linearity			
74	50	3	Female	353	DBILI	Result	
				Comment: < Linearity			
74	50	3	Female	354	DBILI	Quality Flag	E (Exclude)
				Comment: Result below linearity			
74	50	3	Female	354	DBILI	Result	
				Comment: < Linearity			
74	85	3	Female	353	DBILI	Quality Flag	E (Exclude)
				Comment: Result below linearity			
74	85	3	Female	353	DBILI	Result	
				Comment: < Linearity			
74	85	3	Female	354	DBILI	Quality Flag	E (Exclude)
				Comment: Result below linearity			
74	85	3	Female	354	DBILI	Result	
				Comment: < Linearity			
75	43	3	Female	351	CK	Quality Flag	E (Exclude)
				Comment: Result above linearity			

AmplifyBio Study Number A-579-01

Individual Animal Serum Chemistry Data

Key Page

Quality Flags

Symbol	IES Status	Description
E	Excluded	Data Excluded

Measurement Descriptions

Headings Used	Description
Serum Chemistry - ALT	Alanine Aminotransferase
Serum Chemistry - AST	Aspartate Aminotransferase
Serum Chemistry - Alkaline Phosphatase	Alkaline Phosphatase
Serum Chemistry - Total Bilirubin	Total Bilirubin
Serum Chemistry - Direct Bilirubin	Direct Bilirubin
Serum Chemistry - LDH	Lactate Dehydrogenase
Serum Chemistry - Creatine Kinase	Creatine Kinase
Serum Chemistry - Cholesterol	Cholesterol
Serum Chemistry - TRIG	Triglycerides
Serum Chemistry - Blood Urea Nitrogen	Blood Urea Nitrogen
Serum Chemistry - Creatinine	Creatinine
Serum Chemistry - Total Protein	Total Protein
Serum Chemistry - ALB	Albumin
Serum Chemistry - Globulin	Globulin
Serum Chemistry - A/G Ratio	A/G Ratio
Serum Chemistry - Calcium	Calcium
Serum Chemistry - Phosphorus	Phosphorus
Serum Chemistry - Sodium	Sodium
Serum Chemistry - Chloride	Chloride
Serum Chemistry - Potassium	Potassium

3/8/2024 1:41:07PM Page: 116

AmplifyBio Study Number A-579-01

Individual Animal Serum Chemistry Data

Key Page

Measurement Descriptions (Continued)

Headings Used	Description
Serum Chemistry - Glucose	Glucose
Serum Chemistry - GGT	Gamma Glutamyl Transferase

Unit Descriptions

Headings Used	Description
g/dL	g/dL
mg/dL	mg/dL
mmol/L	mmol/L
U/L	U/L

Measurement/Statistics

Measurement	Descriptive
Serum Chemistry - ALT	Mean
	Standard Deviation
	Maximum
	Minimum
Serum Chemistry - AST	Mean
	Standard Deviation
	Maximum
	Minimum

3/8/2024 1:41:07PM Page: 117

AmplifyBio Study Number A-579-01

Individual Animal Serum Chemistry Data

Key Page

Measurement/Statistics (Continued)

Measurement	Descriptive
Serum Chemistry - Alkaline Phosphatase	Mean
	Standard Deviation
	Maximum
	Minimum
Serum Chemistry - Total Bilirubin	Mean
	Standard Deviation
	Maximum
	Minimum
Serum Chemistry - LDH	Mean
	Standard Deviation
	Maximum
	Minimum
Serum Chemistry - Creatine Kinase	Mean
	Standard Deviation
	Maximum
	Minimum
Serum Chemistry - Cholesterol	Mean
	Standard Deviation
	Maximum
	Minimum
Serum Chemistry - TRIG	Mean
	Standard Deviation
	Maximum
	Minimum

AmplifyBio Study Number A-579-01

Individual Animal Serum Chemistry Data

Key Page

Measurement/Statistics (Continued)

Measurement	Descriptive
Serum Chemistry - Blood Urea Nitrogen	Mean
	Standard Deviation
	Maximum
	Minimum
Serum Chemistry - Creatinine	Mean
	Standard Deviation
	Maximum
	Minimum
Serum Chemistry - Total Protein	Mean
	Standard Deviation
	Maximum
	Minimum
Serum Chemistry - ALB	Mean
	Standard Deviation
	Maximum
	Minimum
Serum Chemistry - Globulin	Mean
	Standard Deviation
	Maximum
	Minimum
Serum Chemistry - A/G Ratio	Mean
	Standard Deviation
	Maximum
	Minimum

AmplifyBio Study Number A-579-01

Individual Animal Serum Chemistry Data

Key Page

Measurement/Statistics (Continued)

Measurement	Descriptive
Serum Chemistry - Calcium	Mean
	Standard Deviation
	Maximum
	Minimum
Serum Chemistry - Phosphorus	Mean
	Standard Deviation
	Maximum
	Minimum
Serum Chemistry - Sodium	Mean
	Standard Deviation
	Maximum
	Minimum
Serum Chemistry - Chloride	Mean
	Standard Deviation
	Maximum
	Minimum
Serum Chemistry - Potassium	Mean
	Standard Deviation
	Maximum
	Minimum
Serum Chemistry - Glucose	Mean
	Standard Deviation
	Maximum
	Minimum

AmplifyBio Study Number A-579-01

Individual Animal Serum Chemistry Data

Key Page

Measurement/Statistics (Continued)

Measurement	Descriptive
Serum Chemistry - GGT	Mean
	Standard Deviation
	Maximum
	Minimum

Group Information

Short Name	Long Name	Type	Report Headings 1-4			
1	Group 1	Control	Group 1	Control	Total vg	0
2	Group 2	Dose	Group 2	Low	Total vg	6x10^13
3	Group 3	Dose	Group 3	High	Total vg	1.2x10^14

Comment Abbreviations

RC = Result Comment, FC = Flag Comment

Attachment 4. Individual Animal Urinalysis and Urine Sediment Data and Test Methods/Definitions

Urinalysis Test Methods/Definitions

Test Parameter	Test Method
Appearance (Clarity)	Manual
Appearance (Color)	Manual
Bilirubin	2,6-dichlorobenzene-diazonium-tetrafluoroborate
Blood	Pseudoperoxidase
Glucose	Glucose oxidase/peroxidase
Ketones	Sodium nitroprusside and glycine
Leukocytes	Indoxylcarbonic acid ester hydrolysis to indoxyl
Nitrites	Aromatic amine
pH	Methyl red and bromothymol blue
Protein	3’,3”,5’,5”-tetrachlorophenol-3,4,5,6-tetrabromosulfophthalein
Specific Gravity	Manual
Urobilinogen	4-methoxybenzene-diazonium-tetrafluoroborate in acid
Urine Sediment	Manual
Volume	Manual

Urinalysis Parameters – Result Definition

Test Parameter	Arbitrary Result	Equivalent Quantitative Result
Glucose	Negative	Normal
	1+	50 mg/dL
	2+	100 mg/dL
	3+	250 mg/dL
	4+	1000 mg/dL
Protein	Negative	Negative
	1+	15 mg/dL
	2+	30 mg/dL
	3+	100 mg/dL
	4+	500 mg/dL
pH	5.0, 6.0, 6.5, 7.0, 8.0, or 9.0	5.0, 6.0, 6.5, 7.0, 8.0, or 9.0
Ketone	Negative	Negative
	1+	5 mg/dL
	2+	15 mg/dL
	3+	50 mg/dL
	4+	150 mg/dL
Urobilinogen	Negative	Normal
	1+	1 mg/dL
	2+	4 mg/dL
	3+	8 mg/dL
	4+	12 mg/dL
Bilirubin	Negative	Negative
	1+	1 mg/dL
	2+	3 mg/dL
	3+	6 mg/dL
Nitrite	Negative	Not Applicable
	Positive	Not Applicable
Leukocytes	Negative	Negative
	1+	25/µL
	2+	100/µL
	3+	500/µL
Blood	Negative	Negative
	1+	10 Erythrocytes/µL
	2+	25 Erythrocytes/µL
	3+	50 Erythrocytes/µL
	4+	150 Erythrocytes/µL
	5+	250 Erythrocytes/µL

Urine Sediment Grading Criteria

	Species	Grading Criteria				
Low Power Field (LPF)		0	1	2	3	4
Epithelial Cells	All	0-1	2-5	6-10	11-20	20+
Casts	All	0	1-5	6-10	11-20	20+
Crystals	All	0	1-10	11-25	26-50	50+
High Power Field (HPF)						
Red Blood Cells	All	0-5	6-10	11-25	26-50	50+
White Blood Cells	All	0-5	5-10	11-15	16-25	25+
Sperm	All	0	1-5	6-10	11-20	20+
Yeast	All	0	1-10	11-25	26-50	50+
Mucus	All	0	10-25%	26-50%	51-75%	>75%
Bacteria	All	0	10-25%	26-50%	51-75%	>75%
Amorphous Sediment	All	0	10-25%	26-50%	51-75%	>75%

10/30/2023 10:42:49AM Page: 1

AmplifyBio Study Number A-579-01

Individual Animal Urinalysis Data

Sex: Male Day(s) Relative to Start Date

Group 1 Control Total vg 0	Urinalysis										
	Urine Color	Urine Color	Urine Color	Urine Color	Urine Color	Urine Color	Urine Color	Urine Clarity	Urine Clarity	Urine Clarity	Urine Clarity
	-41	2	8	22	43	50	85	-41	2	8	22
101	yellow	yellow	yellow	amber	yellow	-	-	clear	sl.cloudy	v. cloudy	v. cloudy
102	yellow	yellow	yellow	yellow	yellow	yellow	yellow	sl.cloudy	v. cloudy	sl.cloudy	v. cloudy
Mean	-	-	-	-	-	-	-	-	-	-	-
SD	-	-	-	-	-	-	-	-	-	-	-
Max	-	-	-	-	-	-	-	-	-	-	-
Min	-	-	-	-	-	-	-	-	-	-	-

AmplifyBio Study Number A-579-01

Individual Animal Urinalysis Data

Sex: Male Day(s) Relative to Start Date

Group 1 Control Total vg 0	Urinalysis										
	Urine Clarity	Urine Clarity	Urine Clarity	Urine Bilirubin	Urine Bilirubin	Urine Bilirubin	Urine Bilirubin	Urine Bilirubin	Urine Bilirubin	Urine Bilirubin	Qual. Urine Glucose
	43	50	85	-41	2	8	22	43	50	85	-41
101	clear	-	-	NEG	NEG	NEG	NEG	NEG	-	-	NEG
102	sl.cloudy	clear	clear	NEG	NEG	NEG	NEG	NEG	NEG	NEG	NEG
Mean	-	-	-	-	-	-	-	-	-	-	-
SD	-	-	-	-	-	-	-	-	-	-	-
Max	-	-	-	-	-	-	-	-	-	-	-
Min	-	-	-	-	-	-	-	-	-	-	-

AmplifyBio Study Number A-579-01

Individual Animal Urinalysis Data

Sex: Male Day(s) Relative to Start Date

Group 1 Control Total vg 0	Urinalysis										
	Qual. Urine Glucose	Qual. Urine Glucose	Qual. Urine Glucose	Qual. Urine Glucose	Qual. Urine Glucose	Qual. Urine Glucose	Urine Ketone	Urine Ketone	Urine Ketone	Urine Ketone	Urine Ketone
	2	8	22	43	50	85	-41	2	8	22	43
101	NEG	NEG	NEG	NEG	-	-	2+	NEG	2+	NEG	NEG
102	NEG	NEG	NEG	NEG	NEG	NEG	NEG	NEG	1+	NEG	NEG
Mean	-	-	-	-	-	-	-	-	-	-	-
SD	-	-	-	-	-	-	-	-	-	-	-
Max	-	-	-	-	-	-	-	-	-	-	-
Min	-	-	-	-	-	-	-	-	-	-	-

AmplifyBio Study Number A-579-01

Individual Animal Urinalysis Data

Sex: Male Day(s) Relative to Start Date

Group 1 Control Total vg 0	Urinalysis										
	Urine Ketone	Urine Ketone	Urine Leukocytes	Urine Leukocytes	Urine Leukocytes	Urine Leukocytes	Urine Leukocytes	Urine Leukocytes	Urine Leukocytes	Urine Nitrites	Urine Nitrites
	50	85	-41	2	8	22	43	50	85	-41	2
101	-	-	NEG	NEG	NEG	1+	NEG	-	-	POS	NEG
102	NEG	NEG	NEG	NEG	1+	1+	1+	NEG	NEG	POS	POS
Mean	-	-	-	-	-	-	-	-	-	-	-
SD	-	-	-	-	-	-	-	-	-	-	-
Max	-	-	-	-	-	-	-	-	-	-	-
Min	-	-	-	-	-	-	-	-	-	-	-

10/30/2023 10:42:49AM Page: 5

AmplifyBio Study Number A-579-01

Individual Animal Urinalysis Data

Sex: Male Day(s) Relative to Start Date

Group 1 Control Total vg 0	Urinalysis										
	Urine Nitrites	Urine Nitrites	Urine Nitrites	Urine Nitrites	Urine Nitrites	Urine Blood	Urine Blood	Urine Blood	Urine Blood	Urine Blood	Urine Blood
	8	22	43	50	85	-41	2	8	22	43	50
101	NEG	POS	NEG	-	-	3+	3+	3+	5+	1+	-
102	POS	POS	POS	NEG	NEG	2+	1+	2+	5+	2+	3+
Mean	-	-	-	-	-	-	-	-	-	-	-
SD	-	-	-	-	-	-	-	-	-	-	-
Max	-	-	-	-	-	-	-	-	-	-	-
Min	-	-	-	-	-	-	-	-	-	-	-

AmplifyBio Study Number A-579-01

Individual Animal Urinalysis Data

Sex: Male Day(s) Relative to Start Date

Group 1 Control Total vg 0	Urinalysis									
	Urine Blood	pH	pH	pH	pH	pH	pH	pH	U. Protein Qualitative	U. Protein Qualitative	U. Protein Qualitative
	85	-41	2	8	22	43	50	85	-41	2	8
101	-	7.0	8.0	8.0	9.0	8.0	-	-	3+	2+	3+
102	NEG	8.0	8.0	8.0	8.0	9.0	9.0	8.0	2+	1+	2+
Mean	-	7.5	8.0	8.0	8.5	8.5	9.0	8.0	-	-	-
SD	-	0.7	0.0	0.0	0.7	0.7	-	-	-	-	-
Max	-	8.0	8.0	8.0	9.0	9.0	9.0	8.0	-	-	-
Min	-	7.0	8.0	8.0	8.0	8.0	9.0	8.0	-	-	-

AmplifyBio Study Number A-579-01

Individual Animal Urinalysis Data

Sex: Male Day(s) Relative to Start Date

Group 1 Control Total vg 0	Urinalysis										
	U. Protein Qualitative	U. Protein Qualitative	U. Protein Qualitative	U. Protein Qualitative	Urobilinogen (Qualit.)	Urobilinogen (Qualit.)	Urobilinogen (Qualit.)	Urobilinogen (Qualit.)	Urobilinogen (Qualit.)	Urobilinogen (Qualit.)	Urobilinogen (Qualit.)
	22	43	50	85	-41	2	8	22	43	50	85
101	2+	2+	-	-	NEG	NEG	NEG	NEG	NEG	-	-
102	2+	2+	1+	1+	NEG	NEG	NEG	NEG	NEG	NEG	NEG
Mean	-	-	-	-	-	-	-	-	-	-	-
SD	-	-	-	-	-	-	-	-	-	-	-
Max	-	-	-	-	-	-	-	-	-	-	-
Min	-	-	-	-	-	-	-	-	-	-	-

AmplifyBio Study Number A-579-01

Individual Animal Urinalysis Data

Sex: Male Day(s) Relative to Start Date

Group 1 Control Total vg 0	Urinalysis										
	Urine Volume (mL)	Urine Volume (mL)	Urine Volume (mL)	Urine Volume (mL)	Urine Volume (mL)	Urine Volume (mL)	Urine Volume (mL)	Specific Gravity	Specific Gravity	Specific Gravity	Specific Gravity
	-41	2	8	22	43	50	85	-41	2	8	22
101	45.0	76.0	47.0	13.0	42.0	-	-	1.015	1.010	1.020	1.033
102	44.0	40.0	27.0	31.0	23.0	48.0	53.0	1.019	1.016	1.035	1.032
Mean	44.5	58.0	37.0	22.0	32.5	48.0	53.0	1.017	1.013	1.028	1.033
SD	0.7	25.5	14.1	12.7	13.4	-	-	0.003	0.004	0.011	0.001
Max	45.0	76.0	47.0	31.0	42.0	48.0	53.0	1.019	1.016	1.035	1.033
Min	44.0	40.0	27.0	13.0	23.0	48.0	53.0	1.015	1.010	1.020	1.032

AmplifyBio Study Number A-579-01

Individual Animal Urinalysis Data

Sex: Male Day(s) Relative to Start Date

Group 1 Control Total vg 0	Urinalysis		
	Specific Gravity	Specific Gravity	Specific Gravity
	43	50	85
101	1.026	.	.
102	1.029	1.031	1.025
Mean	1.028	1.031	1.025
SD	0.002	.	.
Max	1.029	1.031	1.025
Min	1.026	1.031	1.025

AmplifyBio Study Number A-579-01

Individual Animal Urinalysis Data

Sex: Male Day(s) Relative to Start Date

Group 2 Low Total vg 6x10^13	Urinalysis										
	Urine Color	Urine Color	Urine Color	Urine Color	Urine Color	Urine Color	Urine Color	Urine Color	Urine Clarity	Urine Clarity	Urine Clarity
	-42	-41	2	8	22	43	50	85	-42	-41	2
201	-	yellow	yellow	yellow	yellow	yellow	-	-	-	sl.cloudy	sl.cloudy
202	yellow	-	yellow	yellow	yellow	yellow	-	-	clear	-	sl.cloudy
203	yellow	-	yellow	amber	amber	yellow	yellow	yellow	sl.cloudy	-	v. cloudy
204	yellow	-	yellow	yellow	yellow	yellow	yellow	yellow	sl.cloudy	-	sl.cloudy
Mean	-	-	-	-	-	-	-	-	-	-	-
SD	-	-	-	-	-	-	-	-	-	-	-
Max	-	-	-	-	-	-	-	-	-	-	-
Min	-	-	-	-	-	-	-	-	-	-	-

10/30/2023 10:42:49AM Page: 11

AmplifyBio Study Number A-579-01

Individual Animal Urinalysis Data

Sex: Male Day(s) Relative to Start Date

Group 2 Low Total vg 6x10^13	Urinalysis										
	Urine Clarity	Urine Clarity	Urine Clarity	Urine Clarity	Urine Clarity	Urine Bilirubin	Urine Bilirubin	Urine Bilirubin	Urine Bilirubin	Urine Bilirubin	Urine Bilirubin
	8	22	43	50	85	-42	-41	2	8	22	43
201	v. cloudy	clear	clear	-	-	-	NEG	NEG	NEG	NEG	NEG
202	sl.cloudy	v. cloudy	clear	-	-	NEG	-	NEG	NEG	NEG	NEG
203	v. cloudy	v. cloudy	sl.cloudy	sl.cloudy	v. cloudy	NEG	-	NEG	NEG	NEG	NEG
204	clear	v. cloudy	clear	sl.cloudy	v. cloudy	NEG	-	NEG	NEG	NEG	NEG
Mean	-	-	-	-	-	-	-	-	-	-	-
SD	-	-	-	-	-	-	-	-	-	-	-
Max	-	-	-	-	-	-	-	-	-	-	-
Min	-	-	-	-	-	-	-	-	-	-	-

10/30/2023 10:42:49AM Page: 12

AmplifyBio Study Number A-579-01

Individual Animal Urinalysis Data

Sex: Male Day(s) Relative to Start Date

Group 2 Low Total vg 6x10^13	Urinalysis										
	Urine Bilirubin	Urine Bilirubin	Qual. Urine Glucose	Qual. Urine Glucose	Qual. Urine Glucose	Qual. Urine Glucose	Qual. Urine Glucose	Qual. Urine Glucose	Qual. Urine Glucose	Qual. Urine Glucose	Urine Ketone
	50	85	-42	-41	2	8	22	43	50	85	-42
201	-	-	-	1+	NEG	NEG	NEG	NEG	-	-	-
202	-	-	NEG	-	NEG	NEG	NEG	NEG	-	-	2+
203	NEG	NEG	NEG	-	NEG	NEG	NEG	NEG	NEG	NEG	NEG
204	NEG	NEG	NEG	-	NEG	NEG	NEG	NEG	NEG	NEG	2+
Mean	-	-	-	-	-	-	-	-	-	-	-
SD	-	-	-	-	-	-	-	-	-	-	-
Max	-	-	-	-	-	-	-	-	-	-	-
Min	-	-	-	-	-	-	-	-	-	-	-

AmplifyBio Study Number A-579-01

Individual Animal Urinalysis Data

Sex: Male Day(s) Relative to Start Date

Group 2 Low Total vg 6x10^13	Urinalysis										
	Urine Ketone	Urine Ketone	Urine Ketone	Urine Ketone	Urine Ketone	Urine Ketone	Urine Ketone	Urine Leukocytes	Urine Leukocytes	Urine Leukocytes	Urine Leukocytes
	-41	2	8	22	43	50	85	-42	-41	2	8
201	2+	NEG	NEG	NEG	NEG	-	-	-	NEG	NEG	NEG
202	-	NEG	1+	NEG	NEG	-	-	NEG	-	NEG	NEG
203	-	NEG	1+	NEG	NEG	NEG	NEG	1+	-	NEG	1+
204	-	NEG	2+	NEG	NEG	1+	NEG	NEG	-	NEG	NEG
Mean	-	-	-	-	-	-	-	-	-	-	-
SD	-	-	-	-	-	-	-	-	-	-	-
Max	-	-	-	-	-	-	-	-	-	-	-
Min	-	-	-	-	-	-	-	-	-	-	-

AmplifyBio Study Number A-579-01

Individual Animal Urinalysis Data

Sex: Male Day(s) Relative to Start Date

Group 2 Low Total vg 6x10^13	Urinalysis										
	Urine Leukocytes	Urine Leukocytes	Urine Leukocytes	Urine Leukocytes	Urine Nitrites	Urine Nitrites	Urine Nitrites	Urine Nitrites	Urine Nitrites	Urine Nitrites	Urine Nitrites
	22	43	50	85	-42	-41	2	8	22	43	50
201	NEG	NEG	-	-	-	NEG	NEG	NEG	POS	NEG	-
202	1+	NEG	-	-	POS	-	NEG	NEG	NEG	POS	-
203	1+	NEG	NEG	NEG	NEG	-	POS	POS	NEG	NEG	POS
204	1+	NEG	NEG	NEG	NEG	-	NEG	NEG	NEG	NEG	NEG
Mean	-	-	-	-	-	-	-	-	-	-	-
SD	-	-	-	-	-	-	-	-	-	-	-
Max	-	-	-	-	-	-	-	-	-	-	-
Min	-	-	-	-	-	-	-	-	-	-	-

10/30/2023 10:42:49AM Page: 15

AmplifyBio Study Number A-579-01

Individual Animal Urinalysis Data

Sex: Male Day(s) Relative to Start Date

Group 2 Low Total vg 6x10^13	Urinalysis										
	Urine Nitrites	Urine Blood	Urine Blood	Urine Blood	Urine Blood	Urine Blood	Urine Blood	Urine Blood	Urine Blood	pH	pH
	85	-42	-41	2	8	22	43	50	85	-42	-41
201	-	-	2+	2+	2+	NEG	1+	-	-	-	8.0
202	-	2+	-	2+	2+	NEG	NEG	-	-	9.0	-
203	NEG	NEG	-	3+	2+	1+	NEG	1+	3+	9.0	-
204	NEG	NEG	-	1+	1+	3+	NEG	NEG	NEG	8.0	-
Mean	-	-	-	-	-	-	-	-	-	8.7	8.0
SD	-	-	-	-	-	-	-	-	-	0.6	-
Max	-	-	-	-	-	-	-	-	-	9.0	8.0
Min	-	-	-	-	-	-	-	-	-	8.0	8.0

AmplifyBio Study Number A-579-01

Individual Animal Urinalysis Data

Sex: Male Day(s) Relative to Start Date

Group 2 Low Total vg 6x10^13	Urinalysis										
	pH	pH	pH	pH	pH	pH	U. Protein Qualitative	U. Protein Qualitative	U. Protein Qualitative	U. Protein Qualitative	U. Protein Qualitative
	2	8	22	43	50	85	-42	-41	2	8	22
201	8.0	8.0	8.0	8.0	-	-	-	1+	1+	2+	1+
202	8.0	8.0	8.0	9.0	-	-	1+	-	1+	1+	1+
203	8.0	8.0	8.0	8.0	8.0	8.0	1+	-	1+	2+	1+
204	8.0	8.0	8.0	8.0	8.0	8.0	2+	-	1+	2+	1+
Mean	8.0	8.0	8.0	8.3	8.0	8.0	-	-	-	-	-
SD	0.0	0.0	0.0	0.5	0.0	0.0	-	-	-	-	-
Max	8.0	8.0	8.0	9.0	8.0	8.0	-	-	-	-	-
Min	8.0	8.0	8.0	8.0	8.0	8.0	-	-	-	-	-

AmplifyBio Study Number A-579-01

Individual Animal Urinalysis Data

Sex: Male Day(s) Relative to Start Date

Group 2 Low Total vg 6x10^13	Urinalysis										
	U. Protein Qualitative	U. Protein Qualitative	U. Protein Qualitative	Urobilinogen (Qualit.)	Urobilinogen (Qualit.)	Urobilinogen (Qualit.)	Urobilinogen (Qualit.)	Urobilinogen (Qualit.)	Urobilinogen (Qualit.)	Urobilinogen (Qualit.)	Urobilinogen (Qualit.)
	43	50	85	-42	-41	2	8	22	43	50	85
201	1+	-	-	-	NEG	NEG	NEG	NEG	NEG	-	-
202	1+	-	-	NEG	-	NEG	NEG	NEG	NEG	-	-
203	1+	1+	1+	NEG	-	NEG	NEG	NEG	NEG	NEG	NEG
204	1+	1+	NEG	NEG	-	NEG	NEG	NEG	NEG	NEG	NEG
Mean	-	-	-	-	-	-	-	-	-	-	-
SD	-	-	-	-	-	-	-	-	-	-	-
Max	-	-	-	-	-	-	-	-	-	-	-
Min	-	-	-	-	-	-	-	-	-	-	-

10/30/2023 10:42:49AM Page: 18

AmplifyBio Study Number A-579-01

Individual Animal Urinalysis Data

Sex: Male Day(s) Relative to Start Date

Group 2 Low Total vg 6x10^13	Urinalysis										
	Urine Volume (mL)	Urine Volume (mL)	Urine Volume (mL)	Urine Volume (mL)	Urine Volume (mL)	Urine Volume (mL)	Urine Volume (mL)	Urine Volume (mL)	Specific Gravity	Specific Gravity	Specific Gravity
	-42	-41	2	8	22	43	50	85	-42	-41	2
201	-	48.0	76.0	48.0	81.0	72.0	-	-	.	1.022	1.015
202	56.0	-	74.0	42.0	65.0	65.0	-	-	1.017	.	1.016
203	15.0	-	40.0	100.0	31.0	38.0	33.0	48.0	1.033	.	1.019
204	42.0	-	73.0	24.0	14.0	67.0	36.0	36.0	1.021	.	1.015
Mean	37.7	48.0	65.8	53.5	47.8	60.5	34.5	42.0	1.024	1.022	1.016
SD	20.8	-	17.2	32.6	30.7	15.3	2.1	8.5	0.008	.	0.002
Max	56.0	48.0	76.0	100.0	81.0	72.0	36.0	48.0	1.033	1.022	1.019
Min	15.0	48.0	40.0	24.0	14.0	38.0	33.0	36.0	1.017	1.022	1.015

10/30/2023 10:42:49AM Page: 19

AmplifyBio Study Number A-579-01

Individual Animal Urinalysis Data

Sex: Male Day(s) Relative to Start Date

Group 2 Low Total vg 6x10^13	Urinalysis				
	Specific Gravity	Specific Gravity	Specific Gravity	Specific Gravity	Specific Gravity
	8	22	43	50	85
201	1.027	1.024	1.014	.	.
202	1.022	1.022	1.022	.	.
203	1.041	1.036	1.029	1.020	1.021
204	1.014	1.028	1.022	1.033	1.023
Mean	1.026	1.028	1.022	1.027	1.022
SD	0.011	0.006	0.006	0.009	0.001
Max	1.041	1.036	1.029	1.033	1.023
Min	1.014	1.022	1.014	1.020	1.021

AmplifyBio Study Number A-579-01

Individual Animal Urinalysis Data

Sex: Male Day(s) Relative to Start Date

Group 3 High Total vg 1.2x10^14	Urinalysis										
	Urine Color	Urine Color	Urine Color	Urine Color	Urine Color	Urine Color	Urine Color	Urine Clarity	Urine Clarity	Urine Clarity	Urine Clarity
	-43	2	8	22	43	50	85	-43	2	8	22
301	yellow	yellow	yellow	yellow	yellow	-	-	clear	v. cloudy	v. cloudy	v. cloudy
302	yellow	yellow	brown	amber	yellow	-	-	clear	v. cloudy	v. cloudy	v. cloudy
303	yellow	yellow	yellow	yellow	yellow	yellow	yellow	v. cloudy	sl.cloudy	sl.cloudy	v. cloudy
304	yellow	yellow	yellow	amber	yellow	yellow	amber	clear	v. cloudy	sl.cloudy	v. cloudy
Mean	-	-	-	-	-	-	-	-	-	-	-
SD	-	-	-	-	-	-	-	-	-	-	-
Max	-	-	-	-	-	-	-	-	-	-	-
Min	-	-	-	-	-	-	-	-	-	-	-

AmplifyBio Study Number A-579-01

Individual Animal Urinalysis Data

Sex: Male Day(s) Relative to Start Date

Group 3 High Total vg 1.2x10^14	Urinalysis										
	Urine Clarity	Urine Clarity	Urine Clarity	Urine Bilirubin	Urine Bilirubin	Urine Bilirubin	Urine Bilirubin	Urine Bilirubin	Urine Bilirubin	Urine Bilirubin	Qual. Urine Glucose
	43	50	85	-43	2	8	22	43	50	85	-43
301	sl.cloudy	-	-	NEG	NEG	NEG	NEG	NEG	-	-	NEG
302	sl.cloudy	-	-	NEG	NEG	NEG	NEG	NEG	-	-	NEG
303	clear	v. cloudy	sl.cloudy	NEG	NEG	NEG	NEG	NEG	NEG	NEG	NEG
304	clear	v. cloudy	sl.cloudy	NEG	NEG	NEG	NEG	NEG	NEG	NEG	1+
Mean	-	-	-	-	-	-	-	-	-	-	-
SD	-	-	-	-	-	-	-	-	-	-	-
Max	-	-	-	-	-	-	-	-	-	-	-
Min	-	-	-	-	-	-	-	-	-	-	-

10/30/2023 10:42:49AM Page: 22

AmplifyBio Study Number A-579-01

Individual Animal Urinalysis Data

Sex: Male Day(s) Relative to Start Date

Group 3 High Total vg 1.2x10^14	Urinalysis										
	Qual. Urine Glucose	Qual. Urine Glucose	Qual. Urine Glucose	Qual. Urine Glucose	Qual. Urine Glucose	Qual. Urine Glucose	Urine Ketone	Urine Ketone	Urine Ketone	Urine Ketone	Urine Ketone
	2	8	22	43	50	85	-43	2	8	22	43
301	NEG	NEG	NEG	NEG	-	-	4+	1+	NEG	1+	2+
302	NEG	NEG	NEG	NEG	-	-	3+	NEG	1+	NEG	NEG
303	NEG	NEG	NEG	NEG	NEG	NEG	4+	NEG	3+	1+	4+
304	NEG	NEG	NEG	NEG	NEG	NEG	3+	NEG	1+	NEG	1+
Mean	-	-	-	-	-	-	-	-	-	-	-
SD	-	-	-	-	-	-	-	-	-	-	-
Max	-	-	-	-	-	-	-	-	-	-	-
Min	-	-	-	-	-	-	-	-	-	-	-

10/30/2023 10:42:49AM Page: 23

AmplifyBio Study Number A-579-01

Individual Animal Urinalysis Data

Sex: Male Day(s) Relative to Start Date

Group 3 High Total vg 1.2x10^14	Urinalysis										
	Urine Ketone	Urine Ketone	Urine Leukocytes	Urine Leukocytes	Urine Leukocytes	Urine Leukocytes	Urine Leukocytes	Urine Leukocytes	Urine Leukocytes	Urine Nitrites	Urine Nitrites
	50	85	-43	2	8	22	43	50	85	-43	2
301	-	-	2+	NEG	NEG	1+	NEG	-	-	NEG	NEG
302	-	-	NEG	1+	3+	2+	NEG	-	-	NEG	POS
303	NEG	1+	1+	NEG	NEG	1+	NEG	1+	NEG	NEG	POS
304	NEG	NEG	NEG	NEG	NEG	1+	NEG	NEG	1+	NEG	NEG
Mean	-	-	-	-	-	-	-	-	-	-	-
SD	-	-	-	-	-	-	-	-	-	-	-
Max	-	-	-	-	-	-	-	-	-	-	-
Min	-	-	-	-	-	-	-	-	-	-	-

AmplifyBio Study Number A-579-01

Individual Animal Urinalysis Data

Sex: Male Day(s) Relative to Start Date

Group 3 High Total vg 1.2x10^14	Urinalysis										
	Urine Nitrites	Urine Nitrites	Urine Nitrites	Urine Nitrites	Urine Nitrites	Urine Blood	Urine Blood	Urine Blood	Urine Blood	Urine Blood	Urine Blood
	8	22	43	50	85	-43	2	8	22	43	50
301	NEG	NEG	NEG	-	-	1+	3+	3+	1+	4+	-
302	NEG	NEG	POS	-	-	NEG	4+	5+	4+	1+	-
303	NEG	NEG	POS	POS	POS	3+	2+	2+	1+	2+	NEG
304	POS	NEG	NEG	NEG	POS	2+	3+	1+	1+	2+	2+
Mean	-	-	-	-	-	-	-	-	-	-	-
SD	-	-	-	-	-	-	-	-	-	-	-
Max	-	-	-	-	-	-	-	-	-	-	-
Min	-	-	-	-	-	-	-	-	-	-	-

AmplifyBio Study Number A-579-01

Individual Animal Urinalysis Data

Sex: Male Day(s) Relative to Start Date

Group 3 High Total vg 1.2x10^14	Urinalysis									
	Urine Blood	pH	pH	pH	pH	pH	pH	pH	U. Protein Qualitative	U. Protein Qualitative	U. Protein Qualitative
	85	-43	2	8	22	43	50	85	-43	2	8
301	-	7.0	8.0	8.0	8.0	8.0	-	-	2+	2+	2+
302	-	6.0	8.0	8.0	8.0	9.0	-	-	1+	2+	3+
303	NEG	8.0	8.0	8.0	8.0	9.0	8.0	8.0	2+	2+	2+
304	1+	8.0	7.0	9.0	8.0	9.0	8.0	8.0	2+	2+	1+
Mean	-	7.3	7.8	8.3	8.0	8.8	8.0	8.0	-	-	-
SD	-	1.0	0.5	0.5	0.0	0.5	0.0	0.0	-	-	-
Max	-	8.0	8.0	9.0	8.0	9.0	8.0	8.0	-	-	-
Min	-	6.0	7.0	8.0	8.0	8.0	8.0	8.0	-	-	-

AmplifyBio Study Number A-579-01

Individual Animal Urinalysis Data

Sex: Male Day(s) Relative to Start Date

Group 3 High Total vg 1.2x10^14	Urinalysis										
	U. Protein Qualitative	U. Protein Qualitative	U. Protein Qualitative	U. Protein Qualitative	Urobilinogen (Qualit.)	Urobilinogen (Qualit.)	Urobilinogen (Qualit.)	Urobilinogen (Qualit.)	Urobilinogen (Qualit.)	Urobilinogen (Qualit.)	Urobilinogen (Qualit.)
	22	43	50	85	-43	2	8	22	43	50	85
301	1+	2+	-	-	NEG	NEG	NEG	NEG	NEG	-	-
302	2+	1+	-	-	NEG	NEG	1+	NEG	NEG	-	-
303	1+	1+	2+	NEG	NEG	NEG	NEG	NEG	NEG	NEG	NEG
304	1+	2+	2+	1+	NEG	NEG	NEG	NEG	NEG	NEG	NEG
Mean	-	-	-	-	-	-	-	-	-	-	-
SD	-	-	-	-	-	-	-	-	-	-	-
Max	-	-	-	-	-	-	-	-	-	-	-
Min	-	-	-	-	-	-	-	-	-	-	-

10/30/2023 10:42:49AM Page: 27

AmplifyBio Study Number A-579-01

Individual Animal Urinalysis Data

Sex: Male Day(s) Relative to Start Date

Group 3 High Total vg 1.2x10^14	Urinalysis										
	Urine Volume (mL)	Urine Volume (mL)	Urine Volume (mL)	Urine Volume (mL)	Urine Volume (mL)	Urine Volume (mL)	Urine Volume (mL)	Specific Gravity	Specific Gravity	Specific Gravity	Specific Gravity
	-43	2	8	22	43	50	85	-43	2	8	22
301	21.0	55.0	53.0	68.0	45.0	-	-	1.027	1.017	1.020	1.020
302	34.0	39.0	12.0	55.0	55.0	-	-	1.020	1.015	1.016	1.019
303	30.0	57.0	50.0	56.0	18.0	14.0	66.0	1.030	1.019	1.017	1.029
304	22.0	29.0	28.0	25.0	28.0	28.0	32.0	1.028	1.015	1.025	1.025
Mean	26.8	45.0	35.8	51.0	36.5	21.0	49.0	1.026	1.017	1.020	1.023
SD	6.3	13.4	19.4	18.3	16.6	9.9	24.0	0.004	0.002	0.004	0.005
Max	34.0	57.0	53.0	68.0	55.0	28.0	66.0	1.030	1.019	1.025	1.029
Min	21.0	29.0	12.0	25.0	18.0	14.0	32.0	1.020	1.015	1.016	1.019

AmplifyBio Study Number A-579-01

Individual Animal Urinalysis Data

Sex: Male Day(s) Relative to Start Date

Group 3 High Total vg 1.2x10^14	Urinalysis		
	Specific Gravity	Specific Gravity	Specific Gravity
	43	50	85
301	1.022	.	.
302	1.018	.	.
303	1.017	1.031	1.016
304	1.028	1.022	1.014
Mean	1.021	1.027	1.015
SD	0.005	0.006	0.001
Max	1.028	1.031	1.016
Min	1.017	1.022	1.014

AmplifyBio Study Number A-579-01

Individual Animal Urinalysis Data

Sex: Female Day(s) Relative to Start Date

Group 1 Control Total vg 0	Urinalysis										
	Urine Color	Urine Color	Urine Color	Urine Color	Urine Color	Urine Color	Urine Color	Urine Color	Urine Clarity	Urine Clarity	Urine Clarity
	-41	-40	2	8	22	43	50	85	-41	-40	2
151	-	yellow	yellow	yellow	yellow	yellow	-	-	-	v. cloudy	clear
152	yellow	-	yellow	yellow	red	yellow	yellow	yellow	sl.cloudy	-	sl.cloudy
Mean	-	-	-	-	-	-	-	-	-	-	-
SD	-	-	-	-	-	-	-	-	-	-	-
Max	-	-	-	-	-	-	-	-	-	-	-
Min	-	-	-	-	-	-	-	-	-	-	-

AmplifyBio Study Number A-579-01

Individual Animal Urinalysis Data

Sex: Female Day(s) Relative to Start Date

Group 1 Control Total vg 0	Urinalysis										
	Urine Clarity	Urine Clarity	Urine Clarity	Urine Clarity	Urine Clarity	Urine Bilirubin	Urine Bilirubin	Urine Bilirubin	Urine Bilirubin	Urine Bilirubin	Urine Bilirubin
	8	22	43	50	85	-41	-40	2	8	22	43
151	sl.cloudy	sl.cloudy	sl.cloudy	-	-	-	NEG	NEG	NEG	NEG	NEG
152	sl.cloudy	bloody	clear	clear	clear	NEG	-	NEG	NEG	NEG	NEG
Mean	-	-	-	-	-	-	-	-	-	-	-
SD	-	-	-	-	-	-	-	-	-	-	-
Max	-	-	-	-	-	-	-	-	-	-	-
Min	-	-	-	-	-	-	-	-	-	-	-

10/30/2023 10:42:49AM Page: 31

AmplifyBio Study Number A-579-01

Individual Animal Urinalysis Data

Sex: Female Day(s) Relative to Start Date

Group 1 Control Total vg 0	Urinalysis										
	Urine Bilirubin	Urine Bilirubin	Qual. Urine Glucose	Qual. Urine Glucose	Qual. Urine Glucose	Qual. Urine Glucose	Qual. Urine Glucose	Qual. Urine Glucose	Qual. Urine Glucose	Qual. Urine Glucose	Urine Ketone
	50	85	-41	-40	2	8	22	43	50	85	-41
151	-	-	-	NEG	NEG	NEG	NEG	NEG	-	-	-
152	NEG	NEG	NEG	-	2+	NEG	NEG	NEG	NEG	NEG	4+
Mean	-	-	-	-	-	-	-	-	-	-	-
SD	-	-	-	-	-	-	-	-	-	-	-
Max	-	-	-	-	-	-	-	-	-	-	-
Min	-	-	-	-	-	-	-	-	-	-	-

AmplifyBio Study Number A-579-01

Individual Animal Urinalysis Data

Sex: Female Day(s) Relative to Start Date

Group 1 Control Total vg 0	Urinalysis										
	Urine Ketone	Urine Ketone	Urine Ketone	Urine Ketone	Urine Ketone	Urine Ketone	Urine Ketone	Urine Leukocytes	Urine Leukocytes	Urine Leukocytes	Urine Leukocytes
	-40	2	8	22	43	50	85	-41	-40	2	8
151	NEG	NEG	NEG	NEG	NEG	-	-	-	3+	3+	1+
152	-	NEG	4+	2+	NEG	1+	NEG	3+	-	1+	1+
Mean	-	-	-	-	-	-	-	-	-	-	-
SD	-	-	-	-	-	-	-	-	-	-	-
Max	-	-	-	-	-	-	-	-	-	-	-
Min	-	-	-	-	-	-	-	-	-	-	-

AmplifyBio Study Number A-579-01

Individual Animal Urinalysis Data

Sex: Female Day(s) Relative to Start Date

Group 1 Control Total vg 0	Urinalysis										
	Urine Leukocytes	Urine Leukocytes	Urine Leukocytes	Urine Leukocytes	Urine Nitrites	Urine Nitrites	Urine Nitrites	Urine Nitrites	Urine Nitrites	Urine Nitrites	Urine Nitrites
	22	43	50	85	-41	-40	2	8	22	43	50
151	1+	2+	-	-	-	NEG	NEG	POS	POS	NEG	-
152	2+	NEG	1+	NEG	POS	-	POS	POS	POS	NEG	NEG
Mean	-	-	-	-	-	-	-	-	-	-	-
SD	-	-	-	-	-	-	-	-	-	-	-
Max	-	-	-	-	-	-	-	-	-	-	-
Min	-	-	-	-	-	-	-	-	-	-	-

10/30/2023 10:42:49AM Page: 34

AmplifyBio Study Number A-579-01

Individual Animal Urinalysis Data

Sex: Female Day(s) Relative to Start Date

Group 1 Control Total vg 0	Urinalysis										
	Urine Nitrites	Urine Blood	Urine Blood	Urine Blood	Urine Blood	Urine Blood	Urine Blood	Urine Blood	Urine Blood	pH	pH
	85	-41	-40	2	8	22	43	50	85	-41	-40
151	-	-	2+	3+	1+	2+	5+	-	-	-	8.0
152	NEG	4+	-	4+	1+	5+	4+	NEG	1+	9.0	-
Mean	-	-	-	-	-	-	-	-	-	9.0	8.0
SD	-	-	-	-	-	-	-	-	-	-	-
Max	-	-	-	-	-	-	-	-	-	9.0	8.0
Min	-	-	-	-	-	-	-	-	-	9.0	8.0

AmplifyBio Study Number A-579-01

Individual Animal Urinalysis Data

Sex: Female Day(s) Relative to Start Date

Group 1 Control Total vg 0	Urinalysis										
	pH	pH	pH	pH	pH	pH	U. Protein Qualitative	U. Protein Qualitative	U. Protein Qualitative	U. Protein Qualitative	U. Protein Qualitative
	2	8	22	43	50	85	-41	-40	2	8	22
151	8.0	8.0	8.0	8.0	-	-	-	2+	2+	1+	2+
152	8.0	8.0	9.0	9.0	8.0	8.0	2+	-	2+	2+	3+
Mean	8.0	8.0	8.5	8.5	8.0	8.0	-	-	-	-	-
SD	0.0	0.0	0.7	0.7	-	-	-	-	-	-	-
Max	8.0	8.0	9.0	9.0	8.0	8.0	-	-	-	-	-
Min	8.0	8.0	8.0	8.0	8.0	8.0	-	-	-	-	-

10/30/2023 10:42:49AM Page: 36

AmplifyBio Study Number A-579-01

Individual Animal Urinalysis Data

Sex: Female Day(s) Relative to Start Date

Group 1 Control Total vg 0	Urinalysis										
	U. Protein Qualitative	U. Protein Qualitative	U. Protein Qualitative	Urobilinogen (Qualit.)	Urobilinogen (Qualit.)	Urobilinogen (Qualit.)	Urobilinogen (Qualit.)	Urobilinogen (Qualit.)	Urobilinogen (Qualit.)	Urobilinogen (Qualit.)	Urobilinogen (Qualit.)
	43	50	85	-41	-40	2	8	22	43	50	85
151	2+	-	-	-	NEG	NEG	NEG	NEG	NEG	-	-
152	1+	1+	2+	NEG	-	NEG	NEG	NEG	NEG	NEG	NEG
Mean	-	-	-	-	-	-	-	-	-	-	-
SD	-	-	-	-	-	-	-	-	-	-	-
Max	-	-	-	-	-	-	-	-	-	-	-
Min	-	-	-	-	-	-	-	-	-	-	-

AmplifyBio Study Number A-579-01

Individual Animal Urinalysis Data

Sex: Female Day(s) Relative to Start Date

Group 1 Control Total vg 0	Urinalysis										
	Urine Volume (mL)	Urine Volume (mL)	Urine Volume (mL)	Urine Volume (mL)	Urine Volume (mL)	Urine Volume (mL)	Urine Volume (mL)	Urine Volume (mL)	Specific Gravity	Specific Gravity	Specific Gravity
	-41	-40	2	8	22	43	50	85	-41	-40	2
151	-	42.0	23.0	73.0	34.0	70.0	-	-	.	1.023	1.007
152	6.0	-	106.0	57.0	63.0	84.0	64.0	47.0	1.200	.	1.014
Mean	6.0	42.0	64.5	65.0	48.5	77.0	64.0	47.0	1.200	1.023	1.011
SD	-	-	58.7	11.3	20.5	9.9	-	-	.	.	0.005
Max	6.0	42.0	106.0	73.0	63.0	84.0	64.0	47.0	1.200	1.023	1.014
Min	6.0	42.0	23.0	57.0	34.0	70.0	64.0	47.0	1.200	1.023	1.007

AmplifyBio Study Number A-579-01

Individual Animal Urinalysis Data

Sex: Female Day(s) Relative to Start Date

Group 1 Control Total vg 0	Urinalysis				
	Specific Gravity	Specific Gravity	Specific Gravity	Specific Gravity	Specific Gravity
	8	22	43	50	85
151	1.014	1.014	1.012	.	.
152	1.029	1.022	1.023	1.029	1.033
Mean	1.022	1.018	1.018	1.029	1.033
SD	0.011	0.006	0.008	.	.
Max	1.029	1.022	1.023	1.029	1.033
Min	1.014	1.014	1.012	1.029	1.033

AmplifyBio Study Number A-579-01

Individual Animal Urinalysis Data

Sex: Female Day(s) Relative to Start Date

Group 2 Low Total vg 6x10^13	Urinalysis										
	Urine Color	Urine Color	Urine Color	Urine Color	Urine Color	Urine Color	Urine Color	Urine Clarity	Urine Clarity	Urine Clarity	Urine Clarity
	-42	2	8	22	43	50	85	-42	2	8	22
251	yellow	yellow	yellow	yellow	yellow	-	-	clear	sl.cloudy	sl.cloudy	clear
252	yellow	yellow	yellow	yellow	yellow	-	-	sl.cloudy	v. cloudy	sl.cloudy	sl.cloudy
253	yellow	yellow	yellow	yellow	yellow	yellow	yellow	clear	clear	sl.cloudy	sl.cloudy
254	yellow	yellow	yellow	yellow	yellow	yellow	yellow	clear	clear	sl.cloudy	sl.cloudy
Mean	-	-	-	-	-	-	-	-	-	-	-
SD	-	-	-	-	-	-	-	-	-	-	-
Max	-	-	-	-	-	-	-	-	-	-	-
Min	-	-	-	-	-	-	-	-	-	-	-

AmplifyBio Study Number A-579-01

Individual Animal Urinalysis Data

Sex: Female Day(s) Relative to Start Date

Group 2 Low Total vg 6x10^13	Urinalysis										
	Urine Clarity	Urine Clarity	Urine Clarity	Urine Bilirubin	Urine Bilirubin	Urine Bilirubin	Urine Bilirubin	Urine Bilirubin	Urine Bilirubin	Urine Bilirubin	Qual. Urine Glucose
	43	50	85	-42	2	8	22	43	50	85	-42
251	clear	-	-	NEG	NEG	NEG	NEG	NEG	-	-	NEG
252	sl.cloudy	-	-	NEG	NEG	NEG	NEG	NEG	-	-	NEG
253	sl.cloudy	clear	v. cloudy	NEG	NEG	NEG	NEG	NEG	NEG	NEG	NEG
254	clear	sl.cloudy	clear	NEG	NEG	NEG	NEG	NEG	NEG	NEG	NEG
Mean	-	-	-	-	-	-	-	-	-	-	-
SD	-	-	-	-	-	-	-	-	-	-	-
Max	-	-	-	-	-	-	-	-	-	-	-
Min	-	-	-	-	-	-	-	-	-	-	-

AmplifyBio Study Number A-579-01

Individual Animal Urinalysis Data

Sex: Female Day(s) Relative to Start Date

Group 2 Low Total vg 6x10^13	Urinalysis										
	Qual. Urine Glucose	Qual. Urine Glucose	Qual. Urine Glucose	Qual. Urine Glucose	Qual. Urine Glucose	Qual. Urine Glucose	Urine Ketone	Urine Ketone	Urine Ketone	Urine Ketone	Urine Ketone
	2	8	22	43	50	85	-42	2	8	22	43
251	NEG	NEG	NEG	NEG	-	-	NEG	NEG	1+	NEG	NEG
252	NEG	NEG	NEG	NEG	-	-	3+	NEG	2+	NEG	NEG
253	NEG	NEG	NEG	NEG	NEG	NEG	NEG	NEG	NEG	NEG	NEG
254	NEG	NEG	NEG	NEG	1+	NEG	1+	NEG	NEG	NEG	NEG
Mean	-	-	-	-	-	-	-	-	-	-	-
SD	-	-	-	-	-	-	-	-	-	-	-
Max	-	-	-	-	-	-	-	-	-	-	-
Min	-	-	-	-	-	-	-	-	-	-	-

AmplifyBio Study Number A-579-01

Individual Animal Urinalysis Data

Sex: Female Day(s) Relative to Start Date

Group 2 Low Total vg 6x10^13	Urinalysis										
	Urine Ketone	Urine Ketone	Urine Leukocytes	Urine Leukocytes	Urine Leukocytes	Urine Leukocytes	Urine Leukocytes	Urine Leukocytes	Urine Leukocytes	Urine Nitrites	Urine Nitrites
	50	85	-42	2	8	22	43	50	85	-42	2
251	-	-	NEG	NEG	NEG	NEG	NEG	-	-	NEG	POS
252	-	-	2+	NEG	1+	1+	1+	-	-	POS	POS
253	NEG	NEG	2+	NEG	NEG	3+	2+	3+	NEG	NEG	POS
254	NEG	NEG	2+	1+	2+	2+	1+	1+	1+	POS	NEG
Mean	-	-	-	-	-	-	-	-	-	-	-
SD	-	-	-	-	-	-	-	-	-	-	-
Max	-	-	-	-	-	-	-	-	-	-	-
Min	-	-	-	-	-	-	-	-	-	-	-

AmplifyBio Study Number A-579-01

Individual Animal Urinalysis Data

Sex: Female Day(s) Relative to Start Date

Group 2 Low Total vg 6x10^13	Urinalysis										
	Urine Nitrites	Urine Nitrites	Urine Nitrites	Urine Nitrites	Urine Nitrites	Urine Blood	Urine Blood	Urine Blood	Urine Blood	Urine Blood	Urine Blood
	8	22	43	50	85	-42	2	8	22	43	50
251	NEG	NEG	NEG	-	-	NEG	1+	1+	NEG	NEG	-
252	NEG	NEG	NEG	-	-	4+	5+	NEG	NEG	NEG	-
253	NEG	NEG	NEG	POS	NEG	NEG	NEG	3+	1+	3+	1+
254	NEG	NEG	NEG	NEG	NEG	NEG	1+	1+	5+	NEG	5+
Mean	-	-	-	-	-	-	-	-	-	-	-
SD	-	-	-	-	-	-	-	-	-	-	-
Max	-	-	-	-	-	-	-	-	-	-	-
Min	-	-	-	-	-	-	-	-	-	-	-

AmplifyBio Study Number A-579-01

Individual Animal Urinalysis Data

Sex: Female Day(s) Relative to Start Date

Group 2 Low Total vg 6x10^13	Urinalysis									
	Urine Blood	pH	pH	pH	pH	pH	pH	pH	U. Protein Qualitative	U. Protein Qualitative	U. Protein Qualitative
	85	-42	2	8	22	43	50	85	-42	2	8
251	-	9.0	8.0	7.0	8.0	8.0	-	-	1+	NEG	1+
252	-	8.0	9.0	8.0	8.0	9.0	-	-	1+	2+	2+
253	4+	8.0	9.0	8.0	8.0	9.0	8.0	9.0	1+	NEG	1+
254	NEG	8.0	8.0	7.0	9.0	8.0	8.0	8.0	1+	1+	2+
Mean	-	8.3	8.5	7.5	8.3	8.5	8.0	8.5	-	-	-
SD	-	0.5	0.6	0.6	0.5	0.6	0.0	0.7	-	-	-
Max	-	9.0	9.0	8.0	9.0	9.0	8.0	9.0	-	-	-
Min	-	8.0	8.0	7.0	8.0	8.0	8.0	8.0	-	-	-

AmplifyBio Study Number A-579-01

Individual Animal Urinalysis Data

Sex: Female Day(s) Relative to Start Date

Group 2 Low Total vg 6x10^13	Urinalysis										
	U. Protein Qualitative	U. Protein Qualitative	U. Protein Qualitative	U. Protein Qualitative	Urobilinogen (Qualit.)	Urobilinogen (Qualit.)	Urobilinogen (Qualit.)	Urobilinogen (Qualit.)	Urobilinogen (Qualit.)	Urobilinogen (Qualit.)	Urobilinogen (Qualit.)
	22	43	50	85	-42	2	8	22	43	50	85
251	1+	1+	-	-	NEG	NEG	NEG	NEG	NEG	-	-
252	1+	NEG	-	-	NEG	NEG	NEG	NEG	NEG	-	-
253	1+	1+	1+	2+	NEG	NEG	NEG	NEG	NEG	NEG	NEG
254	2+	NEG	1+	1+	NEG	NEG	NEG	NEG	NEG	NEG	NEG
Mean	-	-	-	-	-	-	-	-	-	-	-
SD	-	-	-	-	-	-	-	-	-	-	-
Max	-	-	-	-	-	-	-	-	-	-	-
Min	-	-	-	-	-	-	-	-	-	-	-

AmplifyBio Study Number A-579-01

Individual Animal Urinalysis Data

Sex: Female Day(s) Relative to Start Date

Group 2 Low Total vg 6x10^13	Urinalysis										
	Urine Volume (mL)	Urine Volume (mL)	Urine Volume (mL)	Urine Volume (mL)	Urine Volume (mL)	Urine Volume (mL)	Urine Volume (mL)	Specific Gravity	Specific Gravity	Specific Gravity	Specific Gravity
	-42	2	8	22	43	50	85	-42	2	8	22
251	65.0	75.0	54.0	132.0	118.0	-	-	1.017	1.012	1.015	1.014
252	41.0	76.0	74.0	71.0	62.0	-	-	1.018	1.015	1.028	1.023
253	40.0	78.0	43.0	56.0	41.0	62.0	18.0	1.015	1.011	1.018	1.020
254	98.0	153.0	98.0	59.0	103.0	54.0	26.0	1.008	1.007	1.012	1.013
Mean	61.0	95.5	67.3	79.5	81.0	58.0	22.0	1.015	1.011	1.018	1.018
SD	27.2	38.4	24.2	35.6	35.7	5.7	5.7	0.005	0.003	0.007	0.005
Max	98.0	153.0	98.0	132.0	118.0	62.0	26.0	1.018	1.015	1.028	1.023
Min	40.0	75.0	43.0	56.0	41.0	54.0	18.0	1.008	1.007	1.012	1.013

AmplifyBio Study Number A-579-01

Individual Animal Urinalysis Data

Sex: Female Day(s) Relative to Start Date

Group 2 Low Total vg 6x10^13	Urinalysis		
	Specific Gravity	Specific Gravity	Specific Gravity
	43	50	85
251	1.015	.	.
252	1.014	.	.
253	1.014	1.018	1.017
254	1.008	1.012	1.009
Mean	1.013	1.015	1.013
SD	0.003	0.004	0.006
Max	1.015	1.018	1.017
Min	1.008	1.012	1.009

AmplifyBio Study Number A-579-01

Individual Animal Urinalysis Data

Sex: Female Day(s) Relative to Start Date

Group 3 High Total vg 1.2x10^14	Urinalysis										
	Urine Color	Urine Color	Urine Color	Urine Color	Urine Color	Urine Color	Urine Color	Urine Clarity	Urine Clarity	Urine Clarity	Urine Clarity
	-43	2	8	22	43	50	85	-43	2	8	22
351	yellow	yellow	yellow	amber	yellow	-	-	v. cloudy	v. cloudy	sl.cloudy	v. cloudy
352	yellow	yellow	yellow	yellow	yellow	-	-	sl.cloudy	sl.cloudy	sl.cloudy	sl.cloudy
353	yellow	red	yellow	yellow	yellow	yellow	yellow	v. cloudy	v. cloudy	sl.cloudy	sl.cloudy
354	yellow	yellow	yellow	yellow	yellow	yellow	yellow	sl.cloudy	v. cloudy	sl.cloudy	v. cloudy
Mean	-	-	-	-	-	-	-	-	-	-	-
SD	-	-	-	-	-	-	-	-	-	-	-
Max	-	-	-	-	-	-	-	-	-	-	-
Min	-	-	-	-	-	-	-	-	-	-	-

AmplifyBio Study Number A-579-01

Individual Animal Urinalysis Data

Sex: Female Day(s) Relative to Start Date

Group 3 High Total vg 1.2x10^14	Urinalysis										
	Urine Clarity	Urine Clarity	Urine Clarity	Urine Bilirubin	Urine Bilirubin	Urine Bilirubin	Urine Bilirubin	Urine Bilirubin	Urine Bilirubin	Urine Bilirubin	Qual. Urine Glucose
	43	50	85	-43	2	8	22	43	50	85	-43
351	sl.cloudy	-	-	NEG	NEG	NEG	NEG	NEG	-	-	NEG
352	clear	-	-	NEG	NEG	NEG	NEG	NEG	-	-	NEG
353	clear	v. cloudy	sl.cloudy	NEG	NEG	NEG	NEG	NEG	NEG	NEG	1+
354	clear	v. cloudy	sl.cloudy	NEG	NEG	NEG	NEG	NEG	NEG	NEG	NEG
Mean	-	-	-	-	-	-	-	-	-	-	-
SD	-	-	-	-	-	-	-	-	-	-	-
Max	-	-	-	-	-	-	-	-	-	-	-
Min	-	-	-	-	-	-	-	-	-	-	-

10/30/2023 10:42:49AM Page: 50

AmplifyBio Study Number A-579-01

Individual Animal Urinalysis Data

Sex: Female Day(s) Relative to Start Date

Group 3 High Total vg 1.2x10^14	Urinalysis										
	Qual. Urine Glucose	Qual. Urine Glucose	Qual. Urine Glucose	Qual. Urine Glucose	Qual. Urine Glucose	Qual. Urine Glucose	Urine Ketone	Urine Ketone	Urine Ketone	Urine Ketone	Urine Ketone
	2	8	22	43	50	85	-43	2	8	22	43
351	NEG	NEG	NEG	NEG	-	-	4+	NEG	NEG	NEG	1+
352	NEG	NEG	NEG	NEG	-	-	NEG	NEG	3+	NEG	3+
353	NEG	NEG	NEG	NEG	NEG	NEG	NEG	1+	NEG	NEG	1+
354	NEG	NEG	NEG	NEG	NEG	NEG	1+	NEG	1+	NEG	NEG
Mean	-	-	-	-	-	-	-	-	-	-	-
SD	-	-	-	-	-	-	-	-	-	-	-
Max	-	-	-	-	-	-	-	-	-	-	-
Min	-	-	-	-	-	-	-	-	-	-	-

AmplifyBio Study Number A-579-01

Individual Animal Urinalysis Data

Sex: Female Day(s) Relative to Start Date

Group 3 High Total vg 1.2x10^14	Urinalysis										
	Urine Ketone	Urine Ketone	Urine Leukocytes	Urine Leukocytes	Urine Leukocytes	Urine Leukocytes	Urine Leukocytes	Urine Leukocytes	Urine Leukocytes	Urine Nitrites	Urine Nitrites
	50	85	-43	2	8	22	43	50	85	-43	2
351	-	-	NEG	1+	1+	NEG	NEG	-	-	NEG	NEG
352	-	-	NEG	1+	3+	1+	1+	-	-	NEG	POS
353	1+	NEG	3+	2+	NEG	2+	1+	1+	2+	NEG	POS
354	NEG	1+	NEG	1+	1+	1+	1+	2+	2+	NEG	NEG
Mean	-	-	-	-	-	-	-	-	-	-	-
SD	-	-	-	-	-	-	-	-	-	-	-
Max	-	-	-	-	-	-	-	-	-	-	-
Min	-	-	-	-	-	-	-	-	-	-	-

10/30/2023 10:42:49AM Page: 52

AmplifyBio Study Number A-579-01

Individual Animal Urinalysis Data

Sex: Female Day(s) Relative to Start Date

Group 3 High Total vg 1.2x10^14	Urinalysis										
	Urine Nitrites	Urine Nitrites	Urine Nitrites	Urine Nitrites	Urine Nitrites	Urine Blood	Urine Blood	Urine Blood	Urine Blood	Urine Blood	Urine Blood
	8	22	43	50	85	-43	2	8	22	43	50
351	NEG	NEG	NEG	-	-	5+	2+	3+	NEG	NEG	-
352	NEG	NEG	POS	-	-	1+	3+	2+	NEG	1+	-
353	NEG	NEG	NEG	POS	NEG	4+	5+	3+	NEG	5+	2+
354	NEG	NEG	NEG	NEG	NEG	1+	2+	2+	NEG	5+	3+
Mean	-	-	-	-	-	-	-	-	-	-	-
SD	-	-	-	-	-	-	-	-	-	-	-
Max	-	-	-	-	-	-	-	-	-	-	-
Min	-	-	-	-	-	-	-	-	-	-	-

AmplifyBio Study Number A-579-01

Individual Animal Urinalysis Data

Sex: Female Day(s) Relative to Start Date

Group 3 High Total vg 1.2x10^14	Urinalysis									
	Urine Blood	pH	pH	pH	pH	pH	pH	pH	U. Protein Qualitative	U. Protein Qualitative	U. Protein Qualitative
	85	-43	2	8	22	43	50	85	-43	2	8
351	-	8.0	8.0	8.0	8.0	9.0	-	-	1+	1+	2+
352	-	9.0	9.0	8.0	8.0	9.0	-	-	1+	1+	2+
353	5+	7.0	7.0	8.0	8.0	8.0	8.0	9.0	2+	3+	2+
354	2+	8.0	8.0	8.0	8.0	8.0	7.0	8.0	1+	1+	1+
Mean	-	8.0	8.0	8.0	8.0	8.5	7.5	8.5	-	-	-
SD	-	0.8	0.8	0.0	0.0	0.6	0.7	0.7	-	-	-
Max	-	9.0	9.0	8.0	8.0	9.0	8.0	9.0	-	-	-
Min	-	7.0	7.0	8.0	8.0	8.0	7.0	8.0	-	-	-

AmplifyBio Study Number A-579-01

Individual Animal Urinalysis Data

Sex: Female Day(s) Relative to Start Date

Group 3 High Total vg 1.2x10^14	Urinalysis										
	U. Protein Qualitative	U. Protein Qualitative	U. Protein Qualitative	U. Protein Qualitative	Urobilinogen (Qualit.)	Urobilinogen (Qualit.)	Urobilinogen (Qualit.)	Urobilinogen (Qualit.)	Urobilinogen (Qualit.)	Urobilinogen (Qualit.)	Urobilinogen (Qualit.)
	22	43	50	85	-43	2	8	22	43	50	85
351	1+	1+	-	-	NEG	NEG	NEG	NEG	NEG	-	-
352	1+	2+	-	-	NEG	NEG	NEG	NEG	NEG	-	-
353	NEG	2+	2+	2+	NEG	NEG	NEG	NEG	NEG	NEG	NEG
354	1+	1+	2+	1+	NEG	NEG	NEG	NEG	NEG	NEG	NEG
Mean	-	-	-	-	-	-	-	-	-	-	-
SD	-	-	-	-	-	-	-	-	-	-	-
Max	-	-	-	-	-	-	-	-	-	-	-
Min	-	-	-	-	-	-	-	-	-	-	-

AmplifyBio Study Number A-579-01

Individual Animal Urinalysis Data

Sex: Female Day(s) Relative to Start Date

Group 3 High Total vg 1.2x10^14	Urinalysis										
	Urine Volume (mL)	Urine Volume (mL)	Urine Volume (mL)	Urine Volume (mL)	Urine Volume (mL)	Urine Volume (mL)	Urine Volume (mL)	Specific Gravity	Specific Gravity	Specific Gravity	Specific Gravity
	-43	2	8	22	43	50	85	-43	2	8	22
351	25.0	38.0	26.0	17.0	36.0	-	-	1.026	1.017	1.021	1.032
352	69.0	47.0	7.0	30.0	46.0	-	-	1.019	1.012	1.018	1.015
353	25.0	95.0	10.0	87.0	70.0	71.0	82.0	1.010	1.008	1.011	1.013
354	79.0	76.0	47.0	39.0	62.0	49.0	43.0	1.014	1.011	1.008	1.024
Mean	49.5	64.0	22.5	43.3	53.5	60.0	62.5	1.017	1.012	1.015	1.021
SD	28.6	26.3	18.3	30.5	15.4	15.6	27.6	0.007	0.004	0.006	0.009
Max	79.0	95.0	47.0	87.0	70.0	71.0	82.0	1.026	1.017	1.021	1.032
Min	25.0	38.0	7.0	17.0	36.0	49.0	43.0	1.010	1.008	1.008	1.013

10/30/2023 10:42:49AM Page: 56

AmplifyBio Study Number A-579-01

Individual Animal Urinalysis Data

Sex: Female Day(s) Relative to Start Date

Group 3 High Total vg 1.2x10^14	Urinalysis		
	Specific Gravity	Specific Gravity	Specific Gravity
	43	50	85
351	1.029	.	.
352	1.022	.	.
353	1.018	1.014	1.009
354	1.016	1.009	1.014
Mean	1.021	1.012	1.012
SD	0.006	0.004	0.004
Max	1.029	1.014	1.014
Min	1.016	1.009	1.009

AmplifyBio Study Number A-579-01

Individual Animal Urinalysis Data

Key Page

Measurement Descriptions

Headings Used	Description
Urinalysis - Urine Color	Urine Color
Urinalysis - Urine Clarity	Urine Clarity
Urinalysis - Urine Bilirubin	Urine Bilirubin
Urinalysis - Qual. Urine Glucose	Urine Glucose (Qualitative)
Urinalysis - Urine Ketone	Urine Ketone
Urinalysis - Urine Leukocytes	Urine Leukocytes
Urinalysis - Urine Nitrites	Urine Nitrites
Urinalysis - Urine Blood	Urine Blood
Urinalysis - pH	Urine pH
Urinalysis - U. Protein Qualitative	Urine Protein (Qualitative)
Urinalysis - Urobilinogen (Qualit.)	Urobilinogen (Qualitative)
Urinalysis - Urine Volume	Urine Volume
Urinalysis - Specific Gravity	Specific Gravity

Unit Descriptions

Headings Used	Description
mL	mL

Measurement/Statistics

Measurement	Descriptive
Urinalysis - pH	Mean
	Standard Deviation
	Maximum
	Minimum

AmplifyBio Study Number A-579-01

Individual Animal Urinalysis Data

Key Page

Measurement/Statistics (Continued)

Measurement	Descriptive
Urinalysis - Urine Volume	Mean
	Standard Deviation
	Maximum
	Minimum
Urinalysis - Specific Gravity	Mean
	Standard Deviation
	Maximum
	Minimum

Group Information

Short Name	Long Name	Type	Report Headings 1-4			
1	Group 1	Control	Group 1	Control	Total vg	0
2	Group 2	Dose	Group 2	Low	Total vg	6x10^13
3	Group 3	Dose	Group 3	High	Total vg	1.2x10^14

10/30/2023 10:42:47AM Page: 1

AmplifyBio Study Number A-579-01

Individual Animal Urine Microscopic Data

Sex: Male Day(s) Relative to Start Date

Group 1 Control Total vg 0	Urine Microscopic										
	Urine WBC	Urine WBC	Urine WBC	Urine WBC	Urine WBC	Urine WBC	Urine WBC	Urine RBC (#/HPF)	Urine RBC (#/HPF)	Urine RBC (#/HPF)	Urine RBC (#/HPF)
	-41	2	8	22	43	50	85	-41	2	8	22
101	0	0	0	0	0	-	-	0	0	0	0
102	0	0	0	0	0	0	0	0	0	0	0
Mean	0	0	0	0	0	0	0	0	0	0	0
SD	0	0	0	0	0	-	-	0	0	0	0
Max	0	0	0	0	0	0	0	0	0	0	0
Min	0	0	0	0	0	0	0	0	0	0	0

AmplifyBio Study Number A-579-01

Individual Animal Urine Microscopic Data

Sex: Male Day(s) Relative to Start Date

Group 1 Control Total vg 0	Urine Microscopic										
	Urine RBC (#/HPF)	Urine RBC (#/HPF)	Urine RBC (#/HPF)	Casts (#/LPF)	Casts (#/LPF)	Casts (#/LPF)	Casts (#/LPF)	Casts (#/LPF)	Casts (#/LPF)	Casts (#/LPF)	Epithelial Cells (#/LPF)
	43	50	85	-41	2	8	22	43	50	85	-41
101	1	-	-	2	4	2	2	0	-	-	1
102	0	1	2	2	3	3	2	1	0	0	2
Mean	1	1	2	2	4	3	2	1	0	0	2
SD	1	-	-	0	1	1	0	1	-	-	1
Max	1	1	2	2	4	3	2	1	0	0	2
Min	0	1	2	2	3	2	2	0	0	0	1

AmplifyBio Study Number A-579-01

Individual Animal Urine Microscopic Data

Sex: Male Day(s) Relative to Start Date

Group 1 Control Total vg 0	Urine Microscopic										
	Epithelial Cells (#/LPF)	Epithelial Cells (#/LPF)	Epithelial Cells (#/LPF)	Epithelial Cells (#/LPF)	Epithelial Cells (#/LPF)	Epithelial Cells (#/LPF)	Mucus	Mucus	Mucus	Mucus	Mucus
	2	8	22	43	50	85	-41	2	8	22	43
101	3	2	3	0	-	-	0	3	1	4	0
102	2	2	4	0	1	1	1	2	3	3	1
Mean	3	2	4	0	1	1	1	3	2	4	1
SD	1	0	1	0	-	-	1	1	1	1	1
Max	3	2	4	0	1	1	1	3	3	4	1
Min	2	2	3	0	1	1	0	2	1	3	0

AmplifyBio Study Number A-579-01

Individual Animal Urine Microscopic Data

Sex: Male Day(s) Relative to Start Date

Group 1 Control Total vg 0	Urine Microscopic Mucus	Mucus	Sperm (#/HPF)	Sperm (#/HPF)	Sperm (#/HPF)	Sperm (#/HPF)	Sperm (#/HPF)	Sperm (#/HPF)	Sperm (#/HPF)	Bacteria	Bacteria
	50	85	-41	2	8	22	43	50	85	-41	2
101	-	-	0	4	2	0	0	-	-	1	4
102	0	0	0	3	2	0	0	0	0	1	3
Mean	0	0	0	4	2	0	0	0	0	1	4
SD	-	-	0	1	0	0	0	-	-	0	1
Max	0	0	0	4	2	0	0	0	0	1	4
Min	0	0	0	3	2	0	0	0	0	1	3

AmplifyBio Study Number A-579-01

Individual Animal Urine Microscopic Data

Sex: Male Day(s) Relative to Start Date

Group 1 Control Total vg 0	Urine Microscopic										
	Bacteria	Bacteria	Bacteria	Bacteria	Bacteria	Yeast	Yeast	Yeast	Yeast	Yeast	Yeast
	8	22	43	50	85	-41	2	8	22	43	50
101	2	4	2	-	-	0	0	0	0	0	-
102	2	3	3	2	0	0	0	0	0	0	0
Mean	2	4	3	2	0	0	0	0	0	0	0
SD	0	1	1	-	-	0	0	0	0	0	-
Max	2	4	3	2	0	0	0	0	0	0	0
Min	2	3	2	2	0	0	0	0	0	0	0

AmplifyBio Study Number A-579-01

Individual Animal Urine Microscopic Data

Sex: Male Day(s) Relative to Start Date

Group 1 Control Total vg 0	Urine Microscopic										
	Yeast	Amorphous	Amorphous	Amorphous	Amorphous	Amorphous	Amorphous	Amorphous	Crystals	Crystals	Crystals
	85	-41	2	8	22	43	50	85	-41	2	8
101	-	1	0	0	0	0	-	-	0	2 [a]	1 [a]
102	0	1	0	0	0	2	3	1	0	3 [a]	4 [b]
Mean	0	1	0	0	0	1	3	1	0	3	3
SD	-	0	0	0	0	1	-	-	0	1	2
Max	0	1	0	0	0	2	3	1	0	3	4
Min	0	1	0	0	0	0	3	1	0	2	1

[a] [RC:Calcium Oxalate]
[b] [RC:Calcium Oxalate and Triple Phosphate]

10/30/2023 10:42:47AM Page: 7

AmplifyBio Study Number A-579-01

Individual Animal Urine Microscopic Data

Sex: Male Day(s) Relative to Start Date

Group 1 Control Total vg 0	Urine Microscopic			
	Crystals	Crystals	Crystals	Crystals
	22	43	50	85
101	0	0	-	-
102	0	0	0	0
Mean	0	0	0	0
SD	0	0	-	-
Max	0	0	0	0
Min	0	0	0	0

AmplifyBio Study Number A-579-01

Individual Animal Urine Microscopic Data

Sex: Male Day(s) Relative to Start Date

Group 2 Low Total vg 6x10^13	Urine Microscopic										
	Urine WBC	Urine WBC	Urine WBC	Urine WBC	Urine WBC	Urine WBC	Urine WBC	Urine WBC	Urine RBC (#/HPF)	Urine RBC (#/HPF)	Urine RBC (#/HPF)
	-42	-41	2	8	22	43	50	85	-42	-41	2
201	-	0	0	0	0	0	-	-	-	0	0
202	0	-	0	0	0	0	-	-	0	-	0
203	0	-	0	0	0	0	0	1	0	-	0
204	0	-	0	0	0	0	0	1	0	-	0
Mean	0	0	0	0	0	0	0	1	0	0	0
SD	0	-	0	0	0	0	0	0	0	-	0
Max	0	0	0	0	0	0	0	1	0	0	0
Min	0	0	0	0	0	0	0	1	0	0	0

AmplifyBio Study Number A-579-01

Individual Animal Urine Microscopic Data

Sex: Male Day(s) Relative to Start Date

Group 2 Low Total vg 6x10^13	Urine Microscopic										
	Urine RBC (#/HPF)	Urine RBC (#/HPF)	Urine RBC (#/HPF)	Urine RBC (#/HPF)	Urine RBC (#/HPF)	Casts (#/LPF)	Casts (#/LPF)	Casts (#/LPF)	Casts (#/LPF)	Casts (#/LPF)	Casts (#/LPF)
	8	22	43	50	85	-42	-41	2	8	22	43
201	0	0	1	-	-	-	2	4	2	1	1
202	0	0	0	-	-	0	-	3	3	1	0
203	0	0	0	1	0	0	-	2	3	2	2
204	0	0	1	0	1	0	-	3	2	2	0
Mean	0	0	1	1	1	0	2	3	3	2	1
SD	0	0	1	1	1	0	-	1	1	1	1
Max	0	0	1	1	1	0	2	4	3	2	2
Min	0	0	0	0	0	0	2	2	2	1	0

10/30/2023 10:42:47AM Page: 10

AmplifyBio Study Number A-579-01

Individual Animal Urine Microscopic Data

Sex: Male Day(s) Relative to Start Date

Group 2 Low Total vg 6x10^13	Urine Microscopic										
	Casts (#/LPF)	Casts (#/LPF)	Epithelial Cells (#/LPF)	Epithelial Cells (#/LPF)	Epithelial Cells (#/LPF)	Epithelial Cells (#/LPF)	Epithelial Cells (#/LPF)	Epithelial Cells (#/LPF)	Epithelial Cells (#/LPF)	Epithelial Cells (#/LPF)	Mucus
	50	85	-42	-41	2	8	22	43	50	85	-42
201	-	-	-	0	3	2	1	2	-	-	-
202	-	-	1	-	3	2	0	0	-	-	0
203	0	2	0	-	3	2	1	1	0	3	0
204	1	2	1	-	2	1	1	0	1	2	0
Mean	1	2	1	0	3	2	1	1	1	3	0
SD	1	0	1	-	1	1	1	1	1	1	0
Max	1	2	1	0	3	2	1	2	1	3	0
Min	0	2	0	0	2	1	0	0	0	2	0

AmplifyBio Study Number A-579-01

Individual Animal Urine Microscopic Data

Sex: Male Day(s) Relative to Start Date

Group 2 Low Total vg 6x10^13	Urine Microscopic										
	Mucus	Mucus	Mucus	Mucus	Mucus	Mucus	Mucus	Sperm (#/HPF)	Sperm (#/HPF)	Sperm (#/HPF)	Sperm (#/HPF)
	-41	2	8	22	43	50	85	-42	-41	2	8
201	1	3	4	4	0	-	-	-	0	3	4
202	-	3	4	3	0	-	-	0	-	3	4
203	-	2	3	3	0	0	0	0	-	3	4
204	-	3	2	3	0	0	0	0	-	4	2
Mean	1	3	3	3	0	0	0	0	0	3	4
SD	-	1	1	1	0	0	0	0	-	1	1
Max	1	3	4	4	0	0	0	0	0	4	4
Min	1	2	2	3	0	0	0	0	0	3	2

AmplifyBio Study Number A-579-01

Individual Animal Urine Microscopic Data

Sex: Male Day(s) Relative to Start Date

Group 2 Low Total vg 6x10^13	Urine Microscopic										
	Sperm (#/HPF)	Sperm (#/HPF)	Sperm (#/HPF)	Sperm (#/HPF)	Bacteria	Bacteria	Bacteria	Bacteria	Bacteria	Bacteria	Bacteria
	22	43	50	85	-42	-41	2	8	22	43	50
201	2	0	-	-	-	3	4	4	4	3	-
202	2	0	-	-	2	-	4	4	4	2	-
203	4	0	0	0	0	-	4	4	4	2	0
204	4	0	0	0	1	-	4	2	4	2	2
Mean	3	0	0	0	1	3	4	4	4	2	1
SD	1	0	0	0	1	-	0	1	0	1	1
Max	4	0	0	0	2	3	4	4	4	3	2
Min	2	0	0	0	0	3	4	2	4	2	0

AmplifyBio Study Number A-579-01

Individual Animal Urine Microscopic Data

Sex: Male Day(s) Relative to Start Date

Group 2 Low Total vg 6x10^13	Urine Microscopic										
	Bacteria	Yeast	Yeast	Yeast	Yeast	Yeast	Yeast	Yeast	Yeast	Amorphous	Amorphous
	85	-42	-41	2	8	22	43	50	85	-42	-41
201	-	-	0	0	0	0	0	-	-	-	0
202	-	0	-	0	0	0	0	-	-	2	-
203	3	0	-	0	0	0	0	0	0	4	-
204	3	0	-	0	0	0	0	0	0	3	-
Mean	3	0	0	0	0	0	0	0	0	3	0
SD	0	0	-	0	0	0	0	0	0	1	-
Max	3	0	0	0	0	0	0	0	0	4	0
Min	3	0	0	0	0	0	0	0	0	2	0

10/30/2023 10:42:47AM Page: 14

AmplifyBio Study Number A-579-01

Individual Animal Urine Microscopic Data

Sex: Male Day(s) Relative to Start Date

Group 2 Low Total vg 6x10^13	Urine Microscopic										
	Amorphous	Amorphous	Amorphous	Amorphous	Amorphous	Amorphous	Crystals	Crystals	Crystals	Crystals	Crystals
	2	8	22	43	50	85	-42	-41	2	8	22
201	0	0	0	1	-	-	-	0	2 [b]	1 [b]	1 [b]
202	0	0	0	1	-	-	4 [a]	-	3 [b]	3 [b]	0
203	0	0	0	2	4	2	0	-	2 [b]	2 [b]	0
204	0	0	0	2	3	3	0	-	2 [b]	1 [b]	2 [b]
Mean	0	0	0	2	4	3	1	0	2	2	1
SD	0	0	0	1	1	1	2	-	1	1	1
Max	0	0	0	2	4	3	4	0	3	3	2
Min	0	0	0	1	3	2	0	0	2	1	0

[a] [RC:Calcium phosphate]
[b] [RC:Calcium Oxalate]

AmplifyBio Study Number A-579-01

Individual Animal Urine Microscopic Data

Sex: Male Day(s) Relative to Start Date

Group 2 Low Total vg 6x10^13	Urine Microscopic		
	Crystals	Crystals	Crystals
	43	50	85
201	0	-	-
202	0	-	-
203	0	0	0
204	0	0	0
Mean	0	0	0
SD	0	0	0
Max	0	0	0
Min	0	0	0

AmplifyBio Study Number A-579-01

Individual Animal Urine Microscopic Data

Sex: Male Day(s) Relative to Start Date

Group 3 High Total vg 1.2x10^14	Urine Microscopic										
	Urine WBC	Urine WBC	Urine WBC	Urine WBC	Urine WBC	Urine WBC	Urine WBC	Urine RBC (#/HPF)	Urine RBC (#/HPF)	Urine RBC (#/HPF)	Urine RBC (#/HPF)
	-43	2	8	22	43	50	85	-43	2	8	22
301	0	0	0	0	0	-	-	0	0	0	0
302	0	0	0	0	0	-	-	0	0	0	0
303	0	0	0	0	0	0	0 [a]	0	0	0	0
304	0	0	0	0	0	0	0 [a]	0	0	0	0
Mean	0	0	0	0	0	0	0	0	0	0	0
SD	0	0	0	0	0	0	0	0	0	0	0
Max	0	0	0	0	0	0	0	0	0	0	0
Min	0	0	0	0	0	0	0	0	0	0	0

[a] [SC:Suspect parasitic pinworm egg, veterinary contacted]

AmplifyBio Study Number A-579-01

Individual Animal Urine Microscopic Data

Sex: Male Day(s) Relative to Start Date

Group 3 High Total vg 1.2x10^14	Urine Microscopic										
	Urine RBC (#/HPF)	Urine RBC (#/HPF)	Urine RBC (#/HPF)	Casts (#/LPF)	Casts (#/LPF)	Casts (#/LPF)	Casts (#/LPF)	Casts (#/LPF)	Casts (#/LPF)	Casts (#/LPF)	Epithelial Cells (#/LPF)
	43	50	85	-43	2	8	22	43	50	85	-43
301	1	-	-	0	2	3	0	0	-	-	0
302	0	-	-	0	2	4	2	0	-	-	0
303	1	0	0 [a]	0	3	3	0	1	1	0 [a]	2
304	0	0	0 [a]	0	3	4	1	0	2	2 [a]	1
Mean	1	0	0	0	3	4	1	0	2	1	1
SD	1	0	0	0	1	1	1	1	1	1	1
Max	1	0	0	0	3	4	2	1	2	2	2
Min	0	0	0	0	2	3	0	0	1	0	0

[a] [SC:Suspect parasitic pinworm egg, veterinary contacted]

AmplifyBio Study Number A-579-01

Individual Animal Urine Microscopic Data

Sex: Male Day(s) Relative to Start Date

Group 3 High Total vg 1.2x10^14	Urine Microscopic										
	Epithelial Cells (#/LPF)	Epithelial Cells (#/LPF)	Epithelial Cells (#/LPF)	Epithelial Cells (#/LPF)	Epithelial Cells (#/LPF)	Epithelial Cells (#/LPF)	Mucus	Mucus	Mucus	Mucus	Mucus
	2	8	22	43	50	85	-43	2	8	22	43
301	2	3	3	2	-	-	0	3	3	4	0
302	1	3	3	2	-	-	0	3	4	4	0
303	2	2	0	2	0	1 [a]	0	3	4	4	0
304	2	3	1	0	1	2 [a]	0	3	3	4	0
Mean	2	3	2	2	1	2	0	3	4	4	0
SD	1	1	2	1	1	1	0	0	1	0	0
Max	2	3	3	2	1	2	0	3	4	4	0
Min	1	2	0	0	0	1	0	3	3	4	0

[a] [SC:Suspect parasitic pinworm egg, veterinary contacted]

AmplifyBio Study Number A-579-01

Individual Animal Urine Microscopic Data

Sex: Male Day(s) Relative to Start Date

Group 3 High Total vg 1.2x10^14	Urine Microscopic										
	Mucus	Mucus	Sperm (#/HPF)	Sperm (#/HPF)	Sperm (#/HPF)	Sperm (#/HPF)	Sperm (#/HPF)	Sperm (#/HPF)	Sperm (#/HPF)	Bacteria	Bacteria
	50	85	-43	2	8	22	43	50	85	-43	2
301	-	-	0	4	3	3	0	-	-	0	4
302	-	-	0	4	4	3	0	-	-	0	3
303	1	0 [a]	0	4	4	3	0	2	0 [a]	3	4
304	1	0 [a]	0	4	4	3	0	2	0 [a]	2	4
Mean	1	0	0	4	4	3	0	2	0	1	4
SD	0	0	0	0	1	0	0	0	0	2	1
Max	1	0	0	4	4	3	0	2	0	3	4
Min	1	0	0	4	3	3	0	2	0	0	3

[a] [SC:Suspect parasitic pinworm egg, veterinary contacted]

AmplifyBio Study Number A-579-01

Individual Animal Urine Microscopic Data

Sex: Male Day(s) Relative to Start Date

Group 3 High Total vg 1.2x10^14	Urine Microscopic										
	Bacteria	Bacteria	Bacteria	Bacteria	Bacteria	Yeast	Yeast	Yeast	Yeast	Yeast	Yeast
	8	22	43	50	85	-43	2	8	22	43	50
301	4	4	4	-	-	0	0	0	0	0	-
302	4	4	4	-	-	0	0	0	0	0	-
303	4	4	4	3	4 [a]	0	0	0	0	0	0
304	4	4	3	3	4 [a]	0	0	0	0	0	0
Mean	4	4	4	3	4	0	0	0	0	0	0
SD	0	0	1	0	0	0	0	0	0	0	0
Max	4	4	4	3	4	0	0	0	0	0	0
Min	4	4	3	3	4	0	0	0	0	0	0

[a] [RC:Suspect parasitic pinworm egg, veterinary contacted, SC:Suspect parasitic pinworm egg, veterinary contacted]

AmplifyBio Study Number A-579-01

Individual Animal Urine Microscopic Data

Sex: Male Day(s) Relative to Start Date

Group 3 High Total vg 1.2x10^14	Urine Microscopic										
	Yeast	Amorphous	Amorphous	Amorphous	Amorphous	Amorphous	Amorphous	Amorphous	Crystals	Crystals	Crystals
	85	-43	2	8	22	43	50	85	-43	2	8
301	-	1	0	0	0	3	-	-	0	1 [c]	2 [c]
302	-	0	0	0	0	3	-	-	0	3 [c]	1 [c]
303	0 [a]	3	0	0	0	1	4	1 [a]	0	2 [c]	3 [c]
304	0 [a]	2	0	0	0	4	4	2 [a]	3 [b]	2 [c]	2 [c]
Mean	0	2	0	0	0	3	4	2	1	2	2
SD	0	1	0	0	0	1	0	1	2	1	1
Max	0	3	0	0	0	4	4	2	3	3	3
Min	0	0	0	0	0	1	4	1	0	1	1

[a] [SC:Suspect parasitic pinworm egg, veterinary contacted]
[b] [RC:Triple Phosphate]
[c] [RC:Calcium Oxalate]

AmplifyBio Study Number A-579-01

Individual Animal Urine Microscopic Data

Sex: Male Day(s) Relative to Start Date

Group 3 High Total vg 1.2x10^14	Urine Microscopic			
	Crystals	Crystals	Crystals	Crystals
	22	43	50	85
301	0	0	-	-
302	1 [a]	0	-	-
303	0	0	4 [b]	1 [c]
304	0	0	0	1 [d]
Mean	0	0	2	1
SD	1	0	3	0
Max	1	0	4	1
Min	0	0	0	1

[a] [RC:Calcium Oxalate]
[b] [RC:Triple Phosphate]
[c] [SC:Suspect parasitic pinworm egg, veterinary contacted]
[d] [RC:Sulfadiazine, SC:Suspect parasitic pinworm egg, veterinary contacted]

AmplifyBio Study Number A-579-01

Individual Animal Urine Microscopic Data

Sex: Female Day(s) Relative to Start Date

Group 1 Control Total vg 0	Urine Microscopic										
	Urine WBC	Urine WBC	Urine WBC	Urine WBC	Urine WBC	Urine WBC	Urine WBC	Urine WBC	Urine RBC (#/HPF)	Urine RBC (#/HPF)	Urine RBC (#/HPF)
	-41	-40	2	8	22	43	50	85	-41	-40	2
151	-	0	0	0	0	1	-	-	-	0	0
152	0	-	0	0	1	0	1	1	0	-	0
Mean	0	0	0	0	1	1	1	1	0	0	0
SD	-	-	0	0	1	1	-	-	-	-	0
Max	0	0	0	0	1	1	1	1	0	0	0
Min	0	0	0	0	0	0	1	1	0	0	0

AmplifyBio Study Number A-579-01

Individual Animal Urine Microscopic Data

Sex: Female Day(s) Relative to Start Date

Group 1 Control Total vg 0	Urine Microscopic										
	Urine RBC (#/HPF)	Urine RBC (#/HPF)	Urine RBC (#/HPF)	Urine RBC (#/HPF)	Urine RBC (#/HPF)	Casts (#/LPF)	Casts (#/LPF)	Casts (#/LPF)	Casts (#/LPF)	Casts (#/LPF)	Casts (#/LPF)
	8	22	43	50	85	-41	-40	2	8	22	43
151	0	0	3	-	-	-	3	4	2	1	1
152	0	3	2	0	0	0	-	4	2	2	0
Mean	0	2	3	0	0	0	3	4	2	2	1
SD	0	2	1	-	-	-	-	0	0	1	1
Max	0	3	3	0	0	0	3	4	2	2	1
Min	0	0	2	0	0	0	3	4	2	1	0

AmplifyBio Study Number A-579-01

Individual Animal Urine Microscopic Data

Sex: Female Day(s) Relative to Start Date

Group 1 Control Total vg 0	Urine Microscopic										
	Casts (#/LPF)	Casts (#/LPF)	Epithelial Cells (#/LPF)	Epithelial Cells (#/LPF)	Epithelial Cells (#/LPF)	Epithelial Cells (#/LPF)	Epithelial Cells (#/LPF)	Epithelial Cells (#/LPF)	Epithelial Cells (#/LPF)	Epithelial Cells (#/LPF)	Mucus
	50	85	-41	-40	2	8	22	43	50	85	-41
151	-	-	-	0	2	2	3	2	-	-	-
152	0	0	4	-	3	1	2	3	3	1	0
Mean	0	0	4	0	3	2	3	3	3	1	0
SD	-	-	-	-	1	1	1	1	-	-	-
Max	0	0	4	0	3	2	3	3	3	1	0
Min	0	0	4	0	2	1	2	2	3	1	0

10/30/2023 10:42:47AM Page: 26

AmplifyBio Study Number A-579-01

Individual Animal Urine Microscopic Data

Sex: Female Day(s) Relative to Start Date

Group 1 Control Total vg 0	Urine Microscopic										
	Mucus	Mucus	Mucus	Mucus	Mucus	Mucus	Mucus	Sperm (#/HPF)	Sperm (#/HPF)	Sperm (#/HPF)	Sperm (#/HPF)
	-40	2	8	22	43	50	85	-41	-40	2	8
151	2	2	3	4	1	-	-	-	0	0	0
152	-	1	3	4	0	0	0	0	-	0	0
Mean	2	2	3	4	1	0	0	0	0	0	0
SD	-	1	0	0	1	-	-	-	-	0	0
Max	2	2	3	4	1	0	0	0	0	0	0
Min	2	1	3	4	0	0	0	0	0	0	0

10/30/2023 10:42:47AM Page: 27

AmplifyBio Study Number A-579-01

Individual Animal Urine Microscopic Data

Sex: Female Day(s) Relative to Start Date

Group 1 Control Total vg 0	Urine Microscopic										
	Sperm (#/HPF)	Sperm (#/HPF)	Sperm (#/HPF)	Sperm (#/HPF)	Bacteria	Bacteria	Bacteria	Bacteria	Bacteria	Bacteria	Bacteria
	22	43	50	85	-41	-40	2	8	22	43	50
151	0	0	-	-	-	3	4	3	4	3	-
152	0	0	0	0	0	-	4	3	2	2	2
Mean	0	0	0	0	0	3	4	3	3	3	2
SD	0	0	-	-	-	-	0	0	1	1	-
Max	0	0	0	0	0	3	4	3	4	3	2
Min	0	0	0	0	0	3	4	3	2	2	2

AmplifyBio Study Number A-579-01

Individual Animal Urine Microscopic Data

Sex: Female Day(s) Relative to Start Date

Group 1 Control Total vg 0	Urine Microscopic										
	Bacteria	Yeast	Yeast	Yeast	Yeast	Yeast	Yeast	Yeast	Yeast	Amorphous	Amorphous
	85	-41	-40	2	8	22	43	50	85	-41	-40
151	-	-	0	4	1	2	0	-	-	-	0
152	0	0	-	3	0	2	0	0	0	2	-
Mean	0	0	0	4	1	2	0	0	0	2	0
SD	-	-	-	1	1	0	0	-	-	-	-
Max	0	0	0	4	1	2	0	0	0	2	0
Min	0	0	0	3	0	2	0	0	0	2	0

AmplifyBio Study Number A-579-01

Individual Animal Urine Microscopic Data

Sex: Female Day(s) Relative to Start Date

Group 1 Control Total vg 0	Urine Microscopic										
	Amorphous	Amorphous	Amorphous	Amorphous	Amorphous	Amorphous	Crystals	Crystals	Crystals	Crystals	Crystals
	2	8	22	43	50	85	-41	-40	2	8	22
151	0	0	0	2	-	-	-	2 [a]	3 [a]	3 [b]	0
152	0	0	0	0	1	0	0	-	2 [a]	3 [b]	1 [a]
Mean	0	0	0	1	1	0	0	2	3	3	1
SD	0	0	0	1	-	-	-	-	1	0	1
Max	0	0	0	2	1	0	0	2	3	3	1
Min	0	0	0	0	1	0	0	2	2	3	0

[a] [RC:Calcium Oxalate]
[b] [RC:Calcium Oxalate and Triple Phosphate]

AmplifyBio Study Number A-579-01

Individual Animal Urine Microscopic Data

Sex: Female Day(s) Relative to Start Date

Group 1 Control Total vg 0	Urine Microscopic		
	Crystals	Crystals	Crystals
	43	50	85
151	0	-	-
152	0	0	0
Mean	0	0	0
SD	0	-	-
Max	0	0	0
Min	0	0	0

AmplifyBio Study Number A-579-01

Individual Animal Urine Microscopic Data

Sex: Female Day(s) Relative to Start Date

Group 2 Low Total vg 6x10^13	Urine Microscopic										
	Urine WBC	Urine WBC	Urine WBC	Urine WBC	Urine WBC	Urine WBC	Urine WBC	Urine RBC (#/HPF)	Urine RBC (#/HPF)	Urine RBC (#/HPF)	Urine RBC (#/HPF)
	-42	2	8	22	43	50	85	-42	2	8	22
251	0	0	0	0	0	-	-	0	0	0	0
252	1	0	0	0	0	-	-	1	0	0	0
253	0	0	0	0	0	0	2	0	0	0	0
254	0	0	0	0	0	0	2	0	0	0	0
Mean	0	0	0	0	0	0	2	0	0	0	0
SD	1	0	0	0	0	0	0	1	0	0	0
Max	1	0	0	0	0	0	2	1	0	0	0
Min	0	0	0	0	0	0	2	0	0	0	0

10/30/2023 10:42:47AM Page: 32

AmplifyBio Study Number A-579-01

Individual Animal Urine Microscopic Data

Sex: Female Day(s) Relative to Start Date

Group 2 Low Total vg 6x10^13	Urine Microscopic										
	Urine RBC (#/HPF)	Urine RBC (#/HPF)	Urine RBC (#/HPF)	Casts (#/LPF)	Casts (#/LPF)	Casts (#/LPF)	Casts (#/LPF)	Casts (#/LPF)	Casts (#/LPF)	Casts (#/LPF)	Epithelial Cells (#/LPF)
	43	50	85	-42	2	8	22	43	50	85	-42
251	2	-	-	0	3	2	3	0	-	-	1
252	0	-	-	0	2	3	2	1	-	-	0
253	0	1	1	1	4	3	2	2	0	1	2
254	0	0	0	0	3	2	2	0	1	1	0
Mean	1	1	1	0	3	3	2	1	1	1	1
SD	1	1	1	1	1	1	1	1	1	0	1
Max	2	1	1	1	4	3	3	2	1	1	2
Min	0	0	0	0	2	2	2	0	0	1	0

10/30/2023 10:42:47AM Page: 33

AmplifyBio Study Number A-579-01

Individual Animal Urine Microscopic Data

Sex: Female Day(s) Relative to Start Date

Group 2 Low Total vg 6x10^13	Urine Microscopic										
	Epithelial Cells (#/LPF)	Epithelial Cells (#/LPF)	Epithelial Cells (#/LPF)	Epithelial Cells (#/LPF)	Epithelial Cells (#/LPF)	Epithelial Cells (#/LPF)	Mucus	Mucus	Mucus	Mucus	Mucus
	2	8	22	43	50	85	-42	2	8	22	43
251	3	2	1	0	-	-	0	2	3	2	0
252	2	1	2	1	-	-	0	3	3	4	0
253	3	2	2	1	2	3	0	3	3	4	1
254	2	0	2	3	2	3	0	3	2	4	0
Mean	3	1	2	1	2	3	0	3	3	4	0
SD	1	1	1	1	0	0	0	1	1	1	1
Max	3	2	2	3	2	3	0	3	3	4	1
Min	2	0	1	0	2	3	0	2	2	2	0

AmplifyBio Study Number A-579-01

Individual Animal Urine Microscopic Data

Sex: Female Day(s) Relative to Start Date

Group 2 Low Total vg 6x10^13	Urine Microscopic										
	Mucus	Mucus	Sperm (#/HPF)	Sperm (#/HPF)	Sperm (#/HPF)	Sperm (#/HPF)	Sperm (#/HPF)	Sperm (#/HPF)	Sperm (#/HPF)	Bacteria	Bacteria
	50	85	-42	2	8	22	43	50	85	-42	2
251	-	-	0	0	0	0	0	-	-	1	4
252	-	-	0	0	0	0	0	-	-	3	4
253	0	0	0	0	0	0	0	0	0	1	4
254	1	0	0	0	0	0	0	0	0	0	4
Mean	1	0	0	0	0	0	0	0	0	1	4
SD	1	0	0	0	0	0	0	0	0	1	0
Max	1	0	0	0	0	0	0	0	0	3	4
Min	0	0	0	0	0	0	0	0	0	0	4

AmplifyBio Study Number A-579-01

Individual Animal Urine Microscopic Data

Sex: Female Day(s) Relative to Start Date

Group 2 Low Total vg 6x10^13	Urine Microscopic										
	Bacteria	Bacteria	Bacteria	Bacteria	Bacteria	Yeast	Yeast	Yeast	Yeast	Yeast	Yeast
	8	22	43	50	85	-42	2	8	22	43	50
251	4	3	1	-	-	0	3	1	2	0	-
252	3	3	3	-	-	0	2	3	4	0	-
253	4	3	3	3	3	0	3	3	3	0	0
254	3	3	3	3	1	0	3	2	4	0	0
Mean	4	3	3	3	2	0	3	2	3	0	0
SD	1	0	1	0	1	0	1	1	1	0	0
Max	4	3	3	3	3	0	3	3	4	0	0
Min	3	3	1	3	1	0	2	1	2	0	0

10/30/2023 10:42:47AM Page: 36

AmplifyBio Study Number A-579-01

Individual Animal Urine Microscopic Data

Sex: Female Day(s) Relative to Start Date

Group 2 Low Total vg 6x10^13	Urine Microscopic										
	Yeast	Amorphous	Amorphous	Amorphous	Amorphous	Amorphous	Amorphous	Amorphous	Crystals	Crystals	Crystals
	85	-42	2	8	22	43	50	85	-42	2	8
251	-	3	0	0	0	0	-	-	0	2 [b]	2 [b]
252	-	0	0	0	0	2	-	-	4 [a]	3 [b]	3 [b]
253	1	2	0	0	0	2	3	2	0	2 [b]	2 [b]
254	0	0	0	0	0	0	4	0	0	1 [b]	1 [b]
Mean	1	1	0	0	0	1	4	1	1	2	2
SD	1	2	0	0	0	1	1	1	2	1	1
Max	1	3	0	0	0	2	4	2	4	3	3
Min	0	0	0	0	0	0	3	0	0	1	1

[a] [RC:Triple Phosphate]
[b] [RC:Calcium Oxalate]

AmplifyBio Study Number A-579-01

Individual Animal Urine Microscopic Data

Sex: Female Day(s) Relative to Start Date

Group 2 Low Total vg 6x10^13	Urine Microscopic			
	Crystals	Crystals	Crystals	Crystals
	22	43	50	85
251	1 [a]	0	-	-
252	0	0	-	-
253	0	0	0	0
254	0	0	0	0
Mean	0	0	0	0
SD	1	0	0	0
Max	1	0	0	0
Min	0	0	0	0

[a] [RC:Calcium Oxalate]

AmplifyBio Study Number A-579-01

Individual Animal Urine Microscopic Data

Sex: Female Day(s) Relative to Start Date

Group 3 High Total vg 1.2x10^14	Urine Microscopic										
	Urine WBC	Urine WBC	Urine WBC	Urine WBC	Urine WBC	Urine WBC	Urine WBC	Urine RBC (#/HPF)	Urine RBC (#/HPF)	Urine RBC (#/HPF)	Urine RBC (#/HPF)
	-43	2	8	22	43	50	85	-43	2	8	22
351	0	0	0	0	0	-	-	0	0	0	0
352	0	0	0	0	0	-	-	0	0	0	0
353	0	0	0	0	0	0	2	0	0	0	0
354	0	0	0	0	0	0	1	0	0	0	0
Mean	0	0	0	0	0	0	2	0	0	0	0
SD	0	0	0	0	0	0	1	0	0	0	0
Max	0	0	0	0	0	0	2	0	0	0	0
Min	0	0	0	0	0	0	1	0	0	0	0

AmplifyBio Study Number A-579-01

Individual Animal Urine Microscopic Data

Sex: Female Day(s) Relative to Start Date

Group 3 High Total vg 1.2x10^14	Urine Microscopic										
	Urine RBC (#/HPF)	Urine RBC (#/HPF)	Urine RBC (#/HPF)	Casts (#/LPF)	Casts (#/LPF)	Casts (#/LPF)	Casts (#/LPF)	Casts (#/LPF)	Casts (#/LPF)	Casts (#/LPF)	Epithelial Cells (#/LPF)
	43	50	85	-43	2	8	22	43	50	85	-43
351	0	-	-	0	3	4	1	0	-	-	1
352	1	-	-	1	3	3	0	1	-	-	2
353	1	0	0	3	3	2	1	0	0	2	0
354	1	1	0	0	3	3	2	0	3	0	1
Mean	1	1	0	1	3	3	1	0	2	1	1
SD	1	1	0	1	0	1	1	1	2	1	1
Max	1	1	0	3	3	4	2	1	3	2	2
Min	0	0	0	0	3	2	0	0	0	0	0

AmplifyBio Study Number A-579-01

Individual Animal Urine Microscopic Data

Sex: Female Day(s) Relative to Start Date

Group 3 High Total vg 1.2x10^14	Urine Microscopic										
	Epithelial Cells (#/LPF)	Epithelial Cells (#/LPF)	Epithelial Cells (#/LPF)	Epithelial Cells (#/LPF)	Epithelial Cells (#/LPF)	Epithelial Cells (#/LPF)	Mucus	Mucus	Mucus	Mucus	Mucus
	2	8	22	43	50	85	-43	2	8	22	43
351	3	3	2	0	-	-	0	4	4	4	0
352	3	2	1	0	-	-	1	4	3	3	0
353	3	1	0	1	1	3	2	4	2	3	0
354	3	2	1	0	0	3	0	4	3	4	1
Mean	3	2	1	0	1	3	1	4	3	4	0
SD	0	1	1	1	1	0	1	0	1	1	1
Max	3	3	2	1	1	3	2	4	4	4	1
Min	3	1	0	0	0	3	0	4	2	3	0

10/30/2023 10:42:47AM Page: 41

AmplifyBio Study Number A-579-01

Individual Animal Urine Microscopic Data

Sex: Female Day(s) Relative to Start Date

Group 3 High Total vg 1.2x10^14	Urine Microscopic										
	Mucus	Mucus	Sperm (#/HPF)	Sperm (#/HPF)	Sperm (#/HPF)	Sperm (#/HPF)	Sperm (#/HPF)	Sperm (#/HPF)	Sperm (#/HPF)	Bacteria	Bacteria
	50	85	-43	2	8	22	43	50	85	-43	2
351	-	-	0	0	0	0	0	-	-	0	4
352	-	-	0	0	0	0	0	-	-	2	4
353	1	0	0	0	0	0	0	0	0	2	4
354	1	0	0	0	0	0	0	0	0	3	4
Mean	1	0	0	0	0	0	0	0	0	2	4
SD	0	0	0	0	0	0	0	0	0	1	0
Max	1	0	0	0	0	0	0	0	0	3	4
Min	1	0	0	0	0	0	0	0	0	0	4

10/30/2023 10:42:47AM Page: 42

AmplifyBio Study Number A-579-01

Individual Animal Urine Microscopic Data

Sex: Female Day(s) Relative to Start Date

Group 3 High Total vg 1.2x10^14	Urine Microscopic										
	Bacteria	Bacteria	Bacteria	Bacteria	Bacteria	Yeast	Yeast	Yeast	Yeast	Yeast	Yeast
	8	22	43	50	85	-43	2	8	22	43	50
351	4	4	4	-	-	0	4	2	3	0	-
352	3	4	3	-	-	0	4	2	3	0	-
353	3	3	3	3	3	1	3	3	4	0	0
354	3	4	1	2	2	0	3	3	3	0	0
Mean	3	4	3	3	3	0	4	3	3	0	0
SD	1	1	1	1	1	1	1	1	1	0	0
Max	4	4	4	3	3	1	4	3	4	0	0
Min	3	3	1	2	2	0	3	2	3	0	0

AmplifyBio Study Number A-579-01

Individual Animal Urine Microscopic Data

Sex: Female Day(s) Relative to Start Date

Group 3 High Total vg 1.2x10^14	Urine Microscopic										
	Yeast	Amorphous	Amorphous	Amorphous	Amorphous	Amorphous	Amorphous	Amorphous	Crystals	Crystals	Crystals
	85	-43	2	8	22	43	50	85	-43	2	8
351	-	4	0	0	0	4	-	-	2 [a]	0	2 [b]
352	-	1	0	0	0	2	-	-	0	1 [b]	2 [b]
353	0	0	0	0	0	0	2	2	1 [a]	0	2 [b]
354	0	2	0	0	0	0	2	1	0	0	3 [b]
Mean	0	2	0	0	0	2	2	2	1	0	2
SD	0	2	0	0	0	2	0	1	1	1	1
Max	0	4	0	0	0	4	2	2	2	1	3
Min	0	0	0	0	0	0	2	1	0	0	2

[a] [RC:Triple Phosphate]
[b] [RC:Calcium Oxalate]

10/30/2023 10:42:47AM Page: 44

AmplifyBio Study Number A-579-01

Individual Animal Urine Microscopic Data

Sex: Female Day(s) Relative to Start Date

Group 3 High Total vg 1.2x10^14	Urine Microscopic			
	Crystals	Crystals	Crystals	Crystals
	22	43	50	85
351	0	0	-	-
352	0	2 [a]	-	-
353	0	0	0	0
354	0	0	0	4 [b]
Mean	0	1	0	2
SD	0	1	0	3
Max	0	2	0	4
Min	0	0	0	0

[a] [RC:Triple Phosphate]
[b] [RC:Triple Phosphate and calcium oxalate]

AmplifyBio Study Number A-579-01

Individual Animal Urine Microscopic Data

Comments and Markers

Page	Day	Group	Sex	Subject	Measurement	Type	Marker
6	2	1	Male	101	Crystals	Result	
				Comment: Calcium Oxalate			
6	2	1	Male	102	Crystals	Result	
				Comment: Calcium Oxalate			
6	8	1	Male	101	Crystals	Result	
				Comment: Calcium Oxalate			
6	8	1	Male	102	Crystals	Result	
				Comment: Calcium Oxalate and Triple Phosphate			
14	-42	2	Male	202	Crystals	Result	
				Comment: Calcium phosphate			
14	2	2	Male	201	Crystals	Result	
				Comment: Calcium Oxalate			
14	2	2	Male	202	Crystals	Result	
				Comment: Calcium Oxalate			
14	2	2	Male	203	Crystals	Result	
				Comment: Calcium Oxalate			
14	2	2	Male	204	Crystals	Result	
				Comment: Calcium Oxalate			
14	8	2	Male	201	Crystals	Result	
				Comment: Calcium Oxalate			
14	8	2	Male	202	Crystals	Result	
				Comment: Calcium Oxalate			
14	8	2	Male	203	Crystals	Result	
				Comment: Calcium Oxalate			
14	8	2	Male	204	Crystals	Result	
				Comment: Calcium Oxalate			
14	22	2	Male	201	Crystals	Result	
				Comment: Calcium Oxalate			

10/30/2023 10:42:47AM Page: 46

AmplifyBio Study Number A-579-01

Individual Animal Urine Microscopic Data

Comments and Markers

Page	Day	Group	Sex	Subject	Measurement	Type	Marker
14	22	2	Male	204	Crystals	Result	
				Comment: Calcium Oxalate			
16	85	3	Male	303	Urine WBC	Sample	
				Comment: Suspect parasitic pinworm egg, veterinary contacted			
16	85	3	Male	304	Urine WBC	Sample	
				Comment: Suspect parasitic pinworm egg, veterinary contacted			
17	85	3	Male	303	Urine RBC	Sample	
				Comment: Suspect parasitic pinworm egg, veterinary contacted			
17	85	3	Male	303	Casts	Sample	
				Comment: Suspect parasitic pinworm egg, veterinary contacted			
17	85	3	Male	304	Urine RBC	Sample	
				Comment: Suspect parasitic pinworm egg, veterinary contacted			
17	85	3	Male	304	Casts	Sample	
				Comment: Suspect parasitic pinworm egg, veterinary contacted			
18	85	3	Male	303	Epithelial Cells	Sample	
				Comment: Suspect parasitic pinworm egg, veterinary contacted			
18	85	3	Male	304	Epithelial Cells	Sample	
				Comment: Suspect parasitic pinworm egg, veterinary contacted			
19	85	3	Male	303	Mucus	Sample	
				Comment: Suspect parasitic pinworm egg, veterinary contacted			
19	85	3	Male	303	Sperm	Sample	
				Comment: Suspect parasitic pinworm egg, veterinary contacted			
19	85	3	Male	304	Mucus	Sample	
				Comment: Suspect parasitic pinworm egg, veterinary contacted			
19	85	3	Male	304	Sperm	Sample	
				Comment: Suspect parasitic pinworm egg, veterinary contacted			

AmplifyBio Study Number A-579-01

Individual Animal Urine Microscopic Data

Comments and Markers

Page	Day	Group	Sex	Subject	Measurement	Type	Marker
20	85	3	Male	303	Bacteria	Result	
				Comment: Suspect parasitic pinworm egg, veterinary contacted			
20	85	3	Male	303	Bacteria	Sample	
				Comment: Suspect parasitic pinworm egg, veterinary contacted			
20	85	3	Male	304	Bacteria	Result	
				Comment: Suspect parasitic pinworm egg, veterinary contacted			
20	85	3	Male	304	Bacteria	Sample	
				Comment: Suspect parasitic pinworm egg, veterinary contacted			
21	-43	3	Male	304	Crystals	Result	
				Comment: Triple Phosphate			
21	2	3	Male	301	Crystals	Result	
				Comment: Calcium Oxalate			
21	2	3	Male	302	Crystals	Result	
				Comment: Calcium Oxalate			
21	2	3	Male	303	Crystals	Result	
				Comment: Calcium Oxalate			
21	2	3	Male	304	Crystals	Result	
				Comment: Calcium Oxalate			
21	8	3	Male	301	Crystals	Result	
				Comment: Calcium Oxalate			
21	8	3	Male	302	Crystals	Result	
				Comment: Calcium Oxalate			
21	8	3	Male	303	Crystals	Result	
				Comment: Calcium Oxalate			
21	8	3	Male	304	Crystals	Result	
				Comment: Calcium Oxalate			
21	85	3	Male	303	Yeast	Sample	
				Comment: Suspect parasitic pinworm egg, veterinary contacted			

AmplifyBio Study Number A-579-01

Individual Animal Urine Microscopic Data

Comments and Markers

Page	Day	Group	Sex	Subject	Measurement	Type	Marker
21	85	3	Male	303	Amorphous	Sample	
				Comment: Suspect parasitic pinworm egg, veterinary contacted			
21	85	3	Male	304	Yeast	Sample	
				Comment: Suspect parasitic pinworm egg, veterinary contacted			
21	85	3	Male	304	Amorphous	Sample	
				Comment: Suspect parasitic pinworm egg, veterinary contacted			
22	22	3	Male	302	Crystals	Result	
				Comment: Calcium Oxalate			
22	50	3	Male	303	Crystals	Result	
				Comment: Triple Phosphate			
22	85	3	Male	303	Crystals	Sample	
				Comment: Suspect parasitic pinworm egg, veterinary contacted			
22	85	3	Male	304	Crystals	Result	
				Comment: Sulfadiazine			
22	85	3	Male	304	Crystals	Sample	
				Comment: Suspect parasitic pinworm egg, veterinary contacted			
29	-40	1	Female	151	Crystals	Result	
				Comment: Calcium Oxalate			
29	2	1	Female	151	Crystals	Result	
				Comment: Calcium Oxalate			
29	2	1	Female	152	Crystals	Result	
				Comment: Calcium Oxalate			
29	8	1	Female	151	Crystals	Result	
				Comment: Calcium Oxalate and Triple Phosphate			
29	8	1	Female	152	Crystals	Result	
				Comment: Calcium Oxalate and Triple Phosphate			
29	22	1	Female	152	Crystals	Result	
				Comment: Calcium Oxalate			

AmplifyBio Study Number A-579-01

Individual Animal Urine Microscopic Data

Comments and Markers

Page	Day	Group	Sex	Subject	Measurement	Type	Marker
36	-42	2	Female	252	Crystals	Result	
			Comment: Triple Phosphate				
36	2	2	Female	251	Crystals	Result	
			Comment: Calcium Oxalate				
36	2	2	Female	252	Crystals	Result	
			Comment: Calcium Oxalate				
36	2	2	Female	253	Crystals	Result	
			Comment: Calcium Oxalate				
36	2	2	Female	254	Crystals	Result	
			Comment: Calcium Oxalate				
36	8	2	Female	251	Crystals	Result	
			Comment: Calcium Oxalate				
36	8	2	Female	252	Crystals	Result	
			Comment: Calcium Oxalate				
36	8	2	Female	253	Crystals	Result	
			Comment: Calcium Oxalate				
36	8	2	Female	254	Crystals	Result	
			Comment: Calcium Oxalate				
37	22	2	Female	251	Crystals	Result	
			Comment: Calcium Oxalate				
43	-43	3	Female	351	Crystals	Result	
			Comment: Triple Phosphate				
43	-43	3	Female	353	Crystals	Result	
			Comment: Triple Phosphate				
43	2	3	Female	352	Crystals	Result	
			Comment: Calcium Oxalate				
43	8	3	Female	351	Crystals	Result	
			Comment: Calcium Oxalate				

10/30/2023 10:42:47AM Page: 50

AmplifyBio Study Number A-579-01

Individual Animal Urine Microscopic Data

Comments and Markers

Page	Day	Group	Sex	Subject	Measurement	Type	Marker
43	8	3	Female	352	Crystals	Result	
			Comment: Calcium Oxalate				
43	8	3	Female	353	Crystals	Result	
			Comment: Calcium Oxalate				
43	8	3	Female	354	Crystals	Result	
			Comment: Calcium Oxalate				
44	43	3	Female	352	Crystals	Result	
			Comment: Triple Phosphate				
44	85	3	Female	354	Crystals	Result	
			Comment: Triple Phosphate and calcium oxalate				

AmplifyBio Study Number A-579-01

Individual Animal Urine Microscopic Data

Key Page

Measurement Descriptions

Headings Used	Description
Urine Microscopic - Urine WBC	Urine WBC
Urine Microscopic - Urine RBC	Urine RBC
Urine Microscopic - Casts	Casts
Urine Microscopic - Epithelial Cells	Epithelial Cells
Urine Microscopic - Mucus	Mucus
Urine Microscopic - Sperm	Sperm
Urine Microscopic - Bacteria	Bacteria
Urine Microscopic - Yeast	Yeast
Urine Microscopic - Amorphous	Amorphous
Urine Microscopic - Crystals	Crystals

Unit Descriptions

Headings Used	Description
#/HPF	#/HPF
#/LPF	#/LPF

Measurement/Statistics

Measurement	Descriptive
Urine Microscopic - Urine WBC	Mean
	Standard Deviation
	Maximum
	Minimum

AmplifyBio Study Number A-579-01

Individual Animal Urine Microscopic Data

Key Page

Measurement/Statistics (Continued)

Measurement	Descriptive
Urine Microscopic - Urine RBC	Mean
	Standard Deviation
	Maximum
	Minimum
Urine Microscopic - Casts	Mean
	Standard Deviation
	Maximum
	Minimum
Urine Microscopic - Epithelial Cells	Mean
	Standard Deviation
	Maximum
	Minimum
Urine Microscopic - Mucus	Mean
	Standard Deviation
	Maximum
	Minimum
Urine Microscopic - Sperm	Mean
	Standard Deviation
	Maximum
	Minimum
Urine Microscopic - Bacteria	Mean
	Standard Deviation
	Maximum
	Minimum

AmplifyBio Study Number A-579-01

Individual Animal Urine Microscopic Data

Key Page

Measurement/Statistics (Continued)

Measurement	Descriptive
Urine Microscopic - Yeast	Mean
	Standard Deviation
	Maximum
	Minimum
Urine Microscopic - Amorphous	Mean
	Standard Deviation
	Maximum
	Minimum
Urine Microscopic - Crystals	Mean
	Standard Deviation
	Maximum
	Minimum

Group Information

Short Name	Long Name	Type	Report Headings 1-4			
1	Group 1	Control	Group 1	Control	Total vg	0
2	Group 2	Dose	Group 2	Low	Total vg	6x10^13
3	Group 3	Dose	Group 3	High	Total vg	1.2x10^14

Comment Abbreviations

RC = Result Comment, SC = Sample Comment

Attachment 5. Troponin I Analysis Method Report and Results

ANTECH GLP®

Antech Diagnostics
600 Airport Blvd., Suite 500
Morrisville, NC 27560

For

AmplifyBio
1425 State Route 142 (Plain City-Georgesville Road)
West Jefferson, OH 43162

Study A-579-01

A 12-Week Single Dose Biodistribution and Safety Study in Cynomolgus Monkeys

Katelin Leonesio, BS

Date 12/19/23

Study Design and Methods

Serum was collected from each animal at time periods designated as Pretest, Week 1, Week 2, Week 4, Day 43, Week 8 (12-Week Cohort only) and Day 85 (12-Week Cohort only). Frozen serum samples were transported to Antech GLP by overnight courier. All samples were evaluated for Troponin I analysis. Results were entered into the ClinAxys v2.7 computer system.

The cardiac Troponin I (TRI) assay was a solid-phase chemiluminescent immunoassay. The bound complex, and thus also the photon output as measured by the luminometer (Siemens Healthcare), was inversely proportional to the concentration of cTnI in the sample. The lowest level of quantitation for cTnI was 0.2 ng/mL.

Samples were stored at -70° ± 15° C after analysis for a minimum of 30 days and then discarded per protocol. The raw data was archived at Antech GLP.

A 12-Week Single Dose Biodistribution and Safety Study in Cynomolgus Monkeys

Individual Animal Data
(Attachment)

The attached reports are FINAL reports. The data for these reports are electronically authorized but no electronic signature is applied.

Antech GLP
600 Airport Blvd, Suite 500
Morrisville, NC 27560

Study: A-579-01 Species: MONKEY

Time point: Printed: 12/19/2023

Animal Number	Group /Sex	Occn. Code	TRI ng/mL
101	1M	PRETEST	<0.20
		WEEK 1	<0.20
		WEEK 2	<0.20
		WEEK 4	<0.20
		DAY 43	<0.20
102	1M	PRETEST	<0.20
		WEEK 1	<0.20
		WEEK 2	<0.20
		WEEK 4	<0.20
		DAY 43	<0.20
		WEEK 8	<0.20
		DAY 85	<0.20

David Brown
Laboratory Director

Antech GLP
600 Airport Blvd, Suite 500
Morrisville, NC 27560

Study: A-579-01 Species: MONKEY

Time point: Printed: 12/19/2023

Animal Number	Group /Sex	Occn. Code	COM	TRI ng/mL
201	2M	PRETEST		<0.20
		WEEK 1		<0.20
		WEEK 2		<0.20
		WEEK 4	HEM	<0.20
		DAY 43		<0.20
202	2M	PRETEST		<0.20
		WEEK 1	HEM	<0.20
		WEEK 2		<0.20
		WEEK 4	HEM	<0.20
		DAY 43		<0.20
203	2M	PRETEST		<0.20
		WEEK 1		<0.20
		WEEK 2		<0.20
		WEEK 4	HEM	<0.20
		DAY 43		<0.20
		WEEK 8		<0.20
		DAY 85		<0.20
204	2M	PRETEST		<0.20
		WEEK 1		<0.20
		WEEK 2		<0.20
		WEEK 4		<0.20
		DAY 43		<0.20
		WEEK 8		<0.20
		DAY 85		<0.20

David Brown
Laboratory Director

Antech GLP
600 Airport Blvd, Suite 500
Morrisville, NC 27560

Study: A-579-01 | Species: MONKEY

Time point: | Printed: 12/19/2023

Animal Number	Group /Sex	Occn. Code	COM	TRI ng/mL
301	3M	PRETEST		<0.20
		WEEK 1		<0.20
		WEEK 2		<0.20
		WEEK 4	HEM	<0.20
		DAY 43		<0.20
302	3M	PRETEST		<0.20
		WEEK 1		<0.20
		WEEK 2		<0.20
		WEEK 4	HEM	<0.20
		DAY 43		<0.20
303	3M	PRETEST		<0.20
		WEEK 1		<0.20
		WEEK 2		<0.20
		WEEK 4	HEM	<0.20
		DAY 43		<0.20
		WEEK 8		<0.20
		DAY 85		<0.20
304	3M	PRETEST		<0.20
		WEEK 1		<0.20
		WEEK 2		<0.20
		WEEK 4		<0.20
		DAY 43		<0.20
		WEEK 8		QNS
		DAY 85		<0.20

David Brown
Laboratory Director

Page 3

Antech GLP
600 Airport Blvd, Suite 500
Morrisville, NC 27560

Study: A-579-01 Species: MONKEY

Time point: Printed: 12/19/2023

Animal Number	Group /Sex	Occn. Code	COM	TRI ng/mL
151	1F	PRETEST	HEM	<0.20
		WEEK 1		<0.20
		WEEK 2		<0.20
		WEEK 4		<0.20
		DAY 43		<0.20
152	1F	PRETEST		<0.20
		WEEK 1		0.74
		WEEK 2		<0.20
		WEEK 4	HEM	<0.20
		DAY 43	HEM	<0.20
		WEEK 8		<0.20
		DAY 85		<0.20

David Brown
Laboratory Director

Antech GLP
600 Airport Blvd, Suite 500
Morrisville, NC 27560

Study: A-579-01 Species: MONKEY

Time point: Printed: 12/19/2023

Animal Number	Group /Sex	Occn. Code	COM	TRI ng/mL
251	2F	PRETEST	HEM	<0.20
		WEEK 1		<0.20
		WEEK 2		<0.20
		WEEK 4		<0.20
		DAY 43		<0.20
252	2F	PRETEST		<0.20
		WEEK 1		<0.20
		WEEK 2		<0.20
		WEEK 4		<0.20
		DAY 43		<0.20
253	2F	PRETEST		<0.20
		WEEK 1		<0.20
		WEEK 2		<0.20
		WEEK 4		<0.20
		DAY 43		<0.20
		WEEK 8		<0.20
		DAY 85		<0.20
254	2F	PRETEST		<0.20
		WEEK 1		0.31
		WEEK 2		<0.20
		WEEK 4		<0.20
		DAY 43		<0.20
		WEEK 8		<0.20
		DAY 85		<0.20

David Brown
Laboratory Director

Page 5

Antech GLP
600 Airport Blvd, Suite 500
Morrisville, NC 27560

Study: A-579-01 Species: MONKEY

Time point: Printed: 12/19/2023

Animal Number	Group /Sex	Occn. Code	COM	TRI ng/mL
351	3F	PRETEST		<0.20
		WEEK 1		<0.20
		WEEK 2		<0.20
		WEEK 4		<0.20
		DAY 43		<0.20
352	3F	PRETEST		<0.20
		WEEK 1		<0.20
		WEEK 2		<0.20
		WEEK 4		<0.20
		DAY 43		<0.20
353	3F	PRETEST		<0.20
		WEEK 1		<0.20
		WEEK 2		<0.20
		WEEK 4		<0.20
		DAY 43		<0.20
		WEEK 8		<0.20
		DAY 85		<0.20
354	3F	PRETEST		<0.20
		WEEK 1		<0.20
		WEEK 2		<0.20
		WEEK 4		<0.20
		DAY 43		<0.20
		WEEK 8		<0.20
		DAY 85		<0.20

David Brown
Laboratory Director

Page 6

Antech GLP
600 Airport Blvd, Suite 500
Morrisville, NC 27560

Study: A-579-01

Time point:

Species: MONKEY

Printed: 12/19/2023

Codes and Descriptions for Result Comments

Code	Description
QNS	Quantity Not Sufficient

David Brown
Laboratory Director

Antech GLP
600 Airport Blvd, Suite 500
Morrisville, NC 27560

Study: A-579-01 Species: MONKEY

Time point: Printed: 12/19/2023

Codes and Descriptions for 'C' type tests

Code	Description
HEM	Hemolyzed

David Brown
Laboratory Director

Antech GLP
600 Airport Blvd, Suite 500
Morrisville, NC 27560

Study: A-579-01 Species: MONKEY

Time point: Printed: 12/19/2023

Test Codes and Descriptions

Code	Description
TRI	Troponin I
COM	Comment

David Brown
Laboratory Director

ANTECH GLP
600 AIRPORT BLVD. · SUITE 500 · MORRISVILLE, NC 27560
Phone: 919-277-0822 Fax: 919-277-0825

List of common abbreviations found in raw data or reports:

EE - Entry Error (To be used when data was incorrectly entered into a computer or instrument.)
ET - Electronic Transfer
RE - Recording Error (To be used for manually entered information in error.)
SP - Spelling Error
NA - Not Applicable
RPT or REP - Repeat
DIL - Dilution
Hem - Hemolyzed
Lip - Lipemic
Ict - Icteric
Neut - Neutrophil
Lymph - Lymphocyte
Mono - Monocyte
Eos - Eosinophil
Baso - Basophil
SL - Slight
MO - Moderate
MA - Many
ME - Manual Entry
MK - Marked
TNTC - Too Numerous To Count
NS - None Seen
Poly - Polychromasia
Aniso – Anisocytosis
Poik - Poikilocytosis
Tar - Target Cell
QNS - Quantity Not Sufficient
PLT - Platelets
RET - Retics
NFS - No Further Samples
SS - Short Sample
EOD – End of Day
QNSR- Quantity Not Sufficient to Repeat

ANTECH GLP
600 AIRPORT BLVD. · SUITE 500 · MORRISVILLE, NC 27560
Phone: 919-277-0822 · Fax 919-277-0825

Good Laboratory Practices Statement

A 12-Week Single Dose Biodistribution and Safety Study in Cynomolgus Monkeys

Study Number: A-579-01

As Principal Investigator, I confirm that the clinical pathology portion of this study performed at Antech GLP was in compliance with the Nonclinical Laboratory Studies Good Laboratory Practice Regulations issued by the U.S. Food and Drug Administration (FDA), Title 21 of the Code of Federal Regulations (CFR) Part 58.

Katelin Leonesio 12/19/23

Katelin Leonesio, Study Manager Date

ANTECH GLP
600 AIRPORT BLVD. · SUITE 500 · MORRISVILLE, NC 27560
Phone: 919-277-0822 · Fax: 919-277-0825

Quality Assurance Audit Report

CONFIDENTIAL

To:	AmplifyBio
Protocol referenced:	A 12-Week Single Dose Biodistribution and Safety Study in Cynomolgus Monkeys (A-579-01)
Study Director:	Sapna Varia, PhD (and Study Director's Management)
From Quality Assurance Auditor:	Miracle Hezekiah and Carolyn Nguyen
Timeperiod(s), material audited, Auditor's Initials & date(s) of audit(s):	Pretest: 6/16/23, Study Data, MH 6/29/23<br>Week 1: 6/21/23, Study Data, MH 6/30/23<br>Week 1: 6/22/23, Study Data, MH 6/30/23<br>Week 1: 6/23/23, Study Data, MH 6/30/23<br>Week 2: 6/27/23, Study Data, MH 7/12/23<br>Week 2: 6/28/23, Study Data, MH 7/12/23<br>Week 2: 6/29/23, Study Data, MH 7/12/23<br>Week 4: 7/11/23, Study Data, MH 7/19/23<br>Week 4: 7/12/23, Study Data, MH 7/19/23<br>Week 4: 7/13/23, Study Data, MH 7/19/23<br>Day 43: 8/01/23, Study Data, MH 8/11/23<br>Day 43: 8/02/23, Study Data, MH 8/11/23<br>Day 43: 8/03/23, Study Data, MH 8/11/23<br>Week 8: 8/08/23, Study Data, MH 8/16/23<br>Week 8: 8/09/23, Study Data, MH 8/16/23<br>Week 8: 8/10/23, Study Data, MH 8/16/23<br>Day 85: 9/12/23, Study Data, CN 9/21/23<br>Day 85: 9/13/23, Study Data, CN 9/21/23<br>Day 85: 9/14/23, Study Data, CN 9/21/23<br>Method Report: Draft, Method, MH 11/08/23<br>Method Report: Draft v2, Method, MH 11/16/23<br>Method Report: Final, Method, MH 12/19/23

A 12-Week Single Dose Biodistribution and Safety Study in Cynomolgus Monkeys
(A-579-01)

ANTECH GLP

Quality Assurance Audit Report

CONFIDENTIAL

Date the Audit Report was issued: December 19, 2023

Miracle Hezekiah
QA Auditor's Signature

19Dec23
Date

Acceptance of Responsibility:

Mathew Leonessa
Principal Investigator

12/19/23
Date

Initials: MH = Miracle Hezekiah CN = Carolyn Nguyen

A 12-Week Single Dose Biodistribution and Safety Study in Cynomolgus Monkeys
(A-579-01)

ANTECH GLP

Quality Assurance Audit Report

CONFIDENTIAL

PRETEST: 6/16/23 TIMEPERIOD

1) Audit Finding: There were no audit findings. A response is not required.

WEEK 1: 6/21/23, WEEK 1: 6/22/23 AND WEEK 1: 6/23/23 TIMEPERIODS

2) Audit Finding: There were no audit findings. A response is not required.

WEEK 2: 6/27/23, WEEK 2: 6/28/23 AND WEEK 2: 6/29/23 TIMEPERIODS

3) Audit Finding: There were no audit findings. A response is not required.

WEEK 4: 7/11/23, WEEK 4: 7/12/23 AND WEEK 4: 7/13/23 TIMEPERIODS

4) Audit Finding: There were no audit findings. A response is not required.

DAY 43: 8/01/23, DAY 43: 8/02/23 AND DAY 43: 8/03/23 TIMEPERIODS

5) Audit Finding: There were no audit findings. A response is not required.

WEEK 8: 8/08/23, WEEK 8: 8/09/23 AND WEEK 8: 8/10/23 TIMEPERIODS

6) Audit Finding: There were no audit findings. A response is not required.

DAY 85: 9/12/23, DAY 85: 9/13/23 AND DAY 85: 9/14/23 TIMEPERIODS

7) Audit Finding: There were no audit findings. A response is not required.

METHOD REPORT: DRAFT

8) Audit Finding: Clarify abbreviation i.e. cTnI (Troponin I).

Response: Corrected.

A 12-Week Single Dose Biodistribution and Safety Study in Cynomolgus Monkeys
(A-579-01)

ANTECH GLP

Quality Assurance Audit Report

CONFIDENTIAL

METHOD REPORT: DRAFT V2

9) Audit Finding: Audit Finding: There were no audit findings. A response is not required.

METHOD REPORT: FINAL

10) Audit Finding: Audit Finding: There were no audit findings. A response is not required.

Reviewed by / Date: MH 19Dec23

Title: QA Auditor

Appendix J IFN-γ ELISpot Analysis Contributing Scientist Report

A 12-Week Single Dose Biodistribution and Safety Study in Cynomolgus Monkeys

IFN-γ ELISpot Analysis Contributing Scientist Report

May 29, 2024

Study Number A-579-01

Testing Facility
AmplifyBio
1425 State Route 142 (Plain City-Georgesville Road)
West Jefferson, OH 43162

SIGNATURES

Prepared by:

DocuSigned by:

Victoria Best

Signer Name: Victoria Best
Signing Reason: I have reviewed this document
Signing Time: 5/29/2024 | 11:31:23 AM EDT
8CEA356BED8F4722918CBF5894389D23

5/29/2024

Victoria Best, PhD
Director, Translational Sciences and
Analytical Strategy

Date

TABLE OF CONTENTS

Page

Signatures **2**

1.0 Introduction **4**

2.0 Study Design and Method **4**

3.0 ELISpot Assay Approach **5**

3.1 Stimulation Conditions for ELISpot 5

3.2 Positive Reference Samples 5

3.3 ELISpot Setup 6

4.0 Data Capture and Analysis **6**

5.0 Deviations **7**

6.0 Results **9**

6.1 Assay Performance 9

6.2 Assay Results 10

7.0 Conclusions **12**

8.0 References **12**

LIST OF ATTACHMENTS

Attachment 1. PBMC Isolation, Cryopreservation, and Storage Methods 13

Attachment 2. IFN-γ Peptide ELISpot Methods 44

1.0 INTRODUCTION

This contributing scientist report summarizes the analytical findings during study No. A-579-01. The purpose of this study was to evaluate the potential biodistribution and safety of scAAV9.U6miR871 (ARM-101) when administered once via intrathecal infusion to Cynomolgus monkeys for up to 12 weeks.

2.0 STUDY DESIGN AND METHOD

The study design relating to IFN-γ ELISpot analysis is outlined below.

Table 1. Study Design

Group	Target Dose (Total vg)[a]	Target Dose Volume (mL)	Animal Identification			
			6-Week Cohort[b]		12-Week Cohort[c]	
			Male	Female	Male	Female
1 – Control	0	3	101	151	102	152
2 – Low	6×10^{13}	3	201-202	251-252	203-204	253-254
3 – High	1.2×10^{14}	3	301-302	351-352	303-304	353-354

vg = vector genome.
a. See QE-001542 for dosing deviation.
b. Necropsy Day 43.
c. Necropsy Day 85.

Blood (~ 6 mL) was collected in Na-Heparin tubes from all animals assigned to the study once during the pretest period (all animals), on Day 43 (all animals), and on Day 85 (for animals assigned to the 12-week cohort only) (see DEV-002199 for exceptions). Blood was processed for peripheral blood mononuclear cells (PBMCs) within six hours of collection using 50 ml SepMate™ tubes (see DEV-002201 for exceptions). Freshly isolated PBMCs were resuspended in cold Cryostor CS10 into at least two sterile conical tubes at a final concentration of 1×10^7 cells/mL (5×10^6 viable cells/vial) (see QE-001798 for exceptions). Residual samples were either combined with a previous vial (≤ 0.250 mL) or aliquoted into a separate vial (> 0.250 mL). Vials of isolated PBMCs were cryopreserved at < -145°C in a liquid nitrogen (LN2) vapor-phase storage tank. PBMC isolation, cryopreservation, and storage were executed according to a study-specific method (Attachment 1). Cryopreserved PBMCs were then tested for the presence of AAV9-specific T cell responses by a qualified IFN-γ ELISpot assay as described below in Section 3.0. The qualified IFN-γ ELISpot assay was executed according to a study-specific method (Attachment 2). ELISpot plates following analysis and retained PBMC aliquots were discarded following study director and Sponsor approval.

3.0 ELISPOT ASSAY APPROACH

3.1 STIMULATION CONDITIONS FOR ELISPOT

For interpretation of AAV9-specific T cell responses, three additional stimulation controls were included. First, the Unstimulated negative control condition (cells in media) but without peptide) was used to establish the background nonspecific reactivity of the cells. Second, a Mitogen positive control condition (cells in media + Phytohemagglutinin) was used to establish assay suitability and IFN-γ responsive potential of the cells. Third, a Peptide positive control (cells in media + cynomolgus CMV peptide pool) condition was used to establish the ability of the cells to process and present peptide antigen to T cells, in animals that are CMV responsive.

Table 2. Stimulation Conditions for ELISpot

Condition	Reagent	Vendor/ Product
Unstimulated (negative)	RPMI+ 0.2% DMSO	Corning, catalog No. 10-040-CV
Mitogen stimulated (positive)- PHA	Phytohemagglutinin-L (PHA-L)	Sigma Aldrich/ L2769
Peptide stimulation (positive)-CMV	Single pool of overlapping peptides derived of monkey cytomegalovirus (CMV) UL55 and UL83. Development grade (Average 85% purity). Contains 2% DMSO	JPT Peptide Technologies/ PMCyCMV-UL55 PM-CyCMV-UL83
Test Antigen-AAV	Single pool of 182 peptides derived from AAV9 Capsid, 15mer overlapping by 22 amino acids, >95% purity. Contains 0.2% DMSO.	ProImmune Inc, custom order

3.2 POSITIVE REFERENCE SAMPLES

The assay performance on the study samples was assessed using two reference PBMC samples of known reactivity (low to high responders) to AAV9 capsid tested by the qualified IFN-γ ELISpot method.

1. Reference Sample 1 (RS1 or HHU20220621; CTL Technologies Catalog No. CTL-UP1)
2. Reference Sample 2 (RS2, HHU20211104, or LP650; CTL Technologies Catalog No. CTL-CP1)

3.3 ELISpot Setup

To enumerate T-cell responses to AAV9, a qualified IFN-γ ELISpot assay was utilized. The assay was developed and qualified in-house for linearity, limit of detection (LOD), precision, specificity, accuracy, and assay implementation. The method is based on a commercial ELISpot assay kit suitable for human and non-human primates (NHP) IFN-γ detection. It is directly translatable for use in NHPs, or humans.

Briefly, cryopreserved NHP PBMCs were thawed, washed, and rested for 3 hours (±15 minutes) before testing in the IFN-γ ELISpot assay. The viable recovery of test samples, including the human PBMC reference samples, were evaluated for quality control purposes prior to use in the assay. For each sample, a total of 2×10^5 viable PBMCs/well were plated in 200 µL media according to the various stimulation conditions listed in Table 2 and incubated for 23 to 25 hours at 37°C + 5% CO_2 (see DEV-002362 for exceptions). Human PBMC reference samples with known responsivity to AAV9 were included in each assay for quality control of assay performance (see Section 3.2). Lastly, plates were evaluated as described below.

Response-Determining Criteria: Plates were evaluated using an empirical method to define positive AAV9 T cell responses based on a previously published report comparing false positive and false negative ELISpot measurements across various statistical- and empirical-based approaches (Kalyuzhny, 2012). The empirical method utilized for this study uses a conservative approach that minimizes false positives, but as a consequence would not identify low-level responses 100% of the time. This type of approach is preferred for the application of this assay in immune monitoring of AAV gene therapy (where the clinical significance of barely detectable responses remains to be determined).

Thus, AAV9 responses were considered positive if both of the following empirical criteria were met (Kalyuzhny, 2012; Moodie et al., 2010):

1. SFC (Spot-Forming Cells) in test condition ≥ 2X SFC Unstimulated condition
2. SFC (Spot-Forming Cells) in test condition ≥ 50 SFC/ 1×10^6 PBMCs.

4.0 DATA CAPTURE AND ANALYSIS

All ELISpot plates were scanned and analyzed by ImmunoSpot S6 Universal M2 Analyzer (ImmunoSpot 7 Software) according to facility SOPs. Instrument settings adjusting the visual sensitivity of spot identification and the discrimination of T-cell derived IFN-γ responses based on spot size, were adjusted manually for each plate using positive peptide and negative control (DMSO) wells. If necessary, the parameters for the sensitivity of counting spots were adjusted to exclude artifact or background spots using a QC module within the Immunospot software.

5.0 DEVIATIONS

QE-001542: On Day 1, the dose administration was not exactly 6 hours for all three groups.

For Group 1, Animal 101 had dose administered starting at 0957. There is documentation that the pump was paused at 1414 due to the Huber needle detaching from the port, however, there is no documentation of when the pump was restarted following this pause. It is unclear how long the dose was administered with the pump stop time documented at 1632. For the other Group 1 animals, the dose administration time was as followed: Animal 102 (5hrs 58mins), Animal 151 (5hrs 56mins), and Animal 152 (5hrs 3mins).

For Group 2, the dose administration time for each animal is as followed: Animal 201 (5hrs 56mins), Animal 202 (5hrs 56mins), Animal 203 (5hrs 57mins), Animal 204 (5hrs 55mins), Animal 251 (5hrs 56mins), Animal 252 (5hrs 54mins), Animal 253 (5hrs 56mins), and Animal 254 (5hrs 55mins).

For Group 3, the dose administration time for each animal is as followed: Animal 301 (5hrs 55mins), Animal 302 (5hrs 55mins), Animal 303 (5hrs 51mins), Animal 304 (6hrs 2mins), Animal 351 (5hrs 56mins), Animal 352 (5hrs 56mins), Animal 353 (5hrs 57mins), and Animal 354 (5hrs 58mins).

The impact of +/- 10 minutes on a 6 hour treatment is not likely to have an impact. The failed attempt to give full treatment to Animal 152 will have no effect since this animal was a control and not receiving test article.

DEV-002199: Protocol section 11.10.5 ELISpot Evaluation: Blood (~6 mL) will be collected in Na-Heparin tubes from all animals assigned to the study once during the pretest period (all animals) and during Week 6 (all animals) and Week 12 (for animals assigned to the 12-week cohort only).

On 16Jun2023, blood collected and processed to PBMC did not enough cell count and only one aliquot of PBMC was collected for Animals 151, 201, 204, 251, 353 and 354 thus, not meeting the protocol requirement of at least 2 aliquots of PBMCs per sample. To account for this, a secondary blood collection was scheduled for 21Jun2023 with IACUC approval. This allowed for a second pre-study PBMC aliquot to be collected for these animals but was done in two separate blood collections, therefore not meeting the protocol requirement of blood being collected once for all animals at the pre-study timepoint.

There is minimal impact on the study based on events described in this deviation. In order to collect 2 aliquots of PBMCs per sample during the pre-study time point a second blood collection was scheduled before Day 1. Although the second blood draw was not laid out in the protocol the decision was made in order to meet the other protocol requirement of the second aliquot. Based on cell count and viability data, samples were processed appropriately, and samples can now be used in downstream processing.

DEV-002201: Protocol section 11.10.5 states "Blood will be processed for peripheral blood mononuclear cells (PBMC) isolation within approximately 6 hours of collection and cryopreserved according to facility SOPs and study specific methods." Data shows that 10 samples (Sample IDs 152, 204, 253, 254, 301, 302, 303, 304, 353, and 354) processed on 16JUN2023 were processed > 8 hours.

There is minimal impact to the study for these samples being processed outside of the 8-hour window. Based on cell count and viability data, processing time did not negatively affect samples.

QE-001798: Protocol section 11.10.5 Cytokine Analysis states "The PBMC will be aliquoted into at least two aliquots until the sample is exhausted depending upon the cell counts (target 10 × 10^6 cells/mL) until the sample is exhausted depending upon the cell counts (target 10 × 10^6 cells/mL) and cryopreserved according to the study specific method."

For Day 43 PBMC processing there was only one PBMC aliquot frozen for several samples. On 01Aug2023, for animal 151 there was only one aliquot of PBMC sample frozen. Then on 02Aug2023, animals 201, 251 and 252 had only one aliquot of PBMC sample frozen. Then on 03Aug2023, animal 353 had only one aliquot of PBMC sample frozen.

The impact to study is minimal due to less than two aliquots of PBMC frozen as described in this deviation. The second PBMC aliquot was to be used as a bank aliquot for the Sponsor in case of need to repeat the analysis. The target concentration was more important than obtaining multiple vials. Total available cell count is subject to the animal's blood composition at the time of collection. Hence there was no impact to the data integrity.

DEV-002362: Based on the study-specific method, "A-579-01 Study Specific Method for IFN-y Peptide ELISpot" (Method-00081) cells should be plated at a target final concentration of 2 × 10^6 cells/mL (2 × 10^5 per well). During ELISpot Batch A-579.08Nov2023.002 (ran on 08Nov2023) the cells were inadvertently plated at the following concentrations for the indicated samples:

1. Sample A-579-01.204.PBMC.16Jun2023.01 was plated at 160,000 cells/well.
2. Sample A-579-01.204.PBMC.13Sep2023.01 was plated at 137,000 cells/well.

This was an intentional choice to not plate at the target volume as it was realized during processing that there was a math error in the original cell counts. In an attempt to update and properly document cell counts, samples were resuspended and plated at the concentrations stated above. This was verified with subject matter expert before being plated and were deemed the correct action for the given samples.

There is no impact due to the math error listed in the deviation that led to the use of samples that did not meet the target concentration. The downstream calculations are now based on these concentrations and not the target concentration. This does not impact the ability to interpret the data and therefore there is no impact on the study.

6.0 RESULTS

6.1 ASSAY PERFORMANCE

The assay performance on the study samples was assessed using reference PBMC samples of known reactivity to AAV9. Reference Sample 1 (RS1) demonstrated a low-positive average response of 114.5 SFC/ 1 × 10^6 PBMCs across 11 assay plates tested. Reference Sample 2 (RS2) was a non-responder to AAV9 capsid, demonstrating an average negative response of 26.8 SFC/ 1 × 10^6 PBMCs across 11 assay plates tested.

The total detection rate of the low-positive RS1 sample was 8/11 measurements, or 72.7 %, consistent with other published reports showing a detection rate of 59% (Kalyuzhny, 2012; Moodie et al., 2010). The false positive rate for RS2 was demonstrated to be 1/11 measurements, or 9.1% higher than the 3% false positive rate published by others using identical scoring criteria. Based on these data and the study results described in the next section, the sensitivity performance of this assay was deemed appropriate for this study (Figure 1).

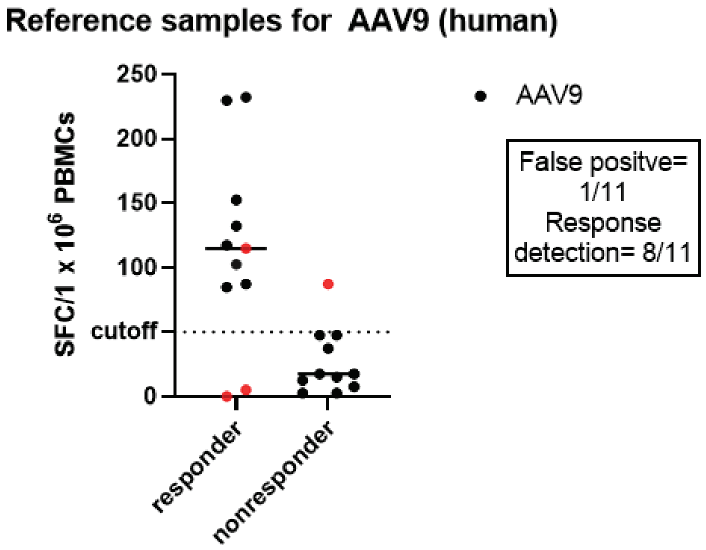

Figure 1. IFN-γ ELISpot Assay Performance
Summary XY graph showing individual responses by AAV9 Responder and AAV9 non-responder reference human PBMCs. Cumulative Individual responses to AAV9 peptide pool (182 peptides, 15-mers, overlapping by 11 amino acids) are shown for each. The number of SFCs per 1 × 10^6 PBMCs plated is shown on the y-axis, with each reference sample depicted on the x-axis. The cut-off value of 50 SFC/ 1 × 10^6 PBMCs for identifying positive responses is depicted by a dotted line. Detection rate (8/11, or 72.7%) and False positive rate (1/11, or 9.1%) based on an empirical response-determining formula (Response positive if > 2X Unstimulated AND ≥ 50 SFC/ 1 × 10^6 PBMCs) are shown. Red circles indicate responses that were not detected (responder), or falsely detected (non-responder).

6.2 ASSAY RESULTS

All of the study animals in treatment Group 1 (Control), Group 2 (6×10^{13} vg), and Group 3 (1.2×10^{14} vg) showed high levels of responsivity to positive control mitogen conditions ranging from 292.5 to 3735 SFC/ 1×10^6 PBMCs while showing minimal background responsivity to unstimulated conditions ranging from 0 to 7.5 SFC/ 1×10^6 PBMCs (Figure 2 and Figure 3). A single study animal, Animal 152 (Group 1), also showed a consistent response to the CMV peptide pool.

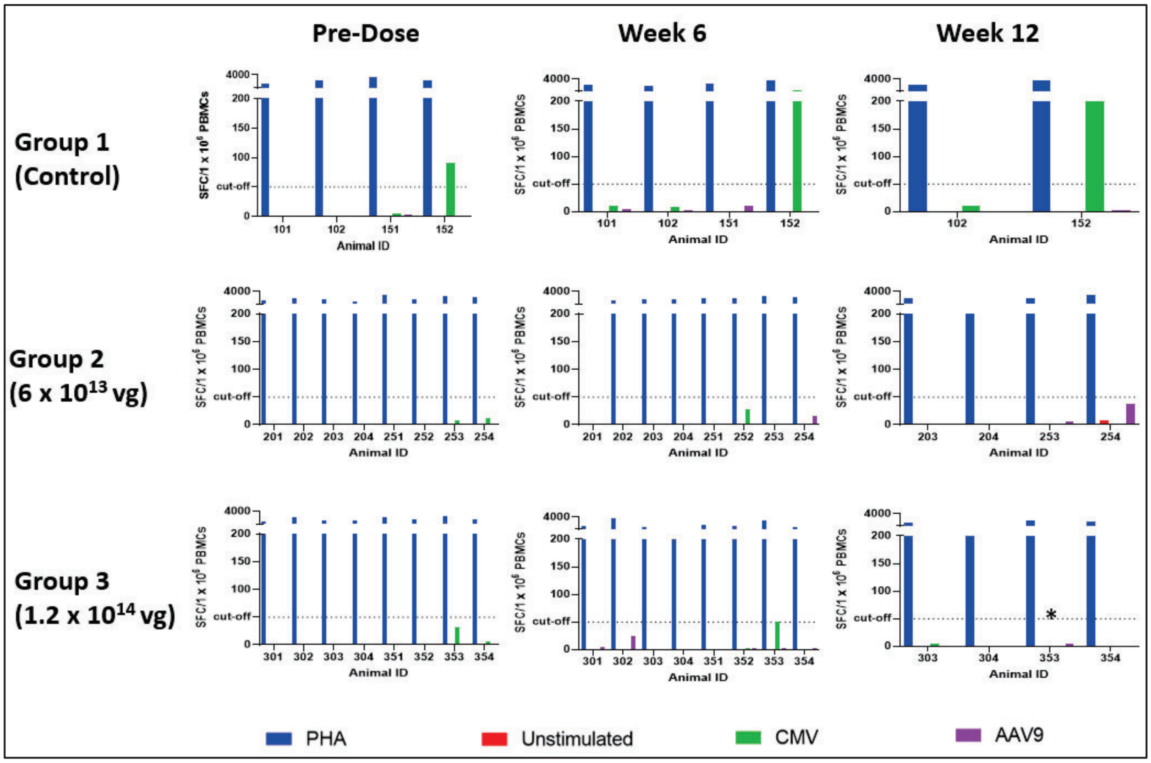

Figure 2. Enumeration of AAV-9 specific T cell responses by IFN-γ ELISpot

Bar graphs showing individual responses by animals belonging to Group 1 (Control), Group 2 (6×10^{13} vg) and Group 3 (1.2×10^{14} vg) study cohorts. Individual responses to PHA, Unstimulated, CMV and AAV9 antigen stimulation conditions are shown at the Pre-Dose (Left Column), Week 6 (Center Column) and Week 12 (Right Column) timepoints. The number of SFCs per 1×10^6 PBMCs plated is shown on the y-axis, for the individual animals (x-axis). The cut-off value of 50 SFC/ 1×10^6 PBMCs for identifying positive responses is depicted by a dotted line. * = CMV antigen stimulation was not performed for Animal 353 at the Week 12 timepoint due to limited sample availability.

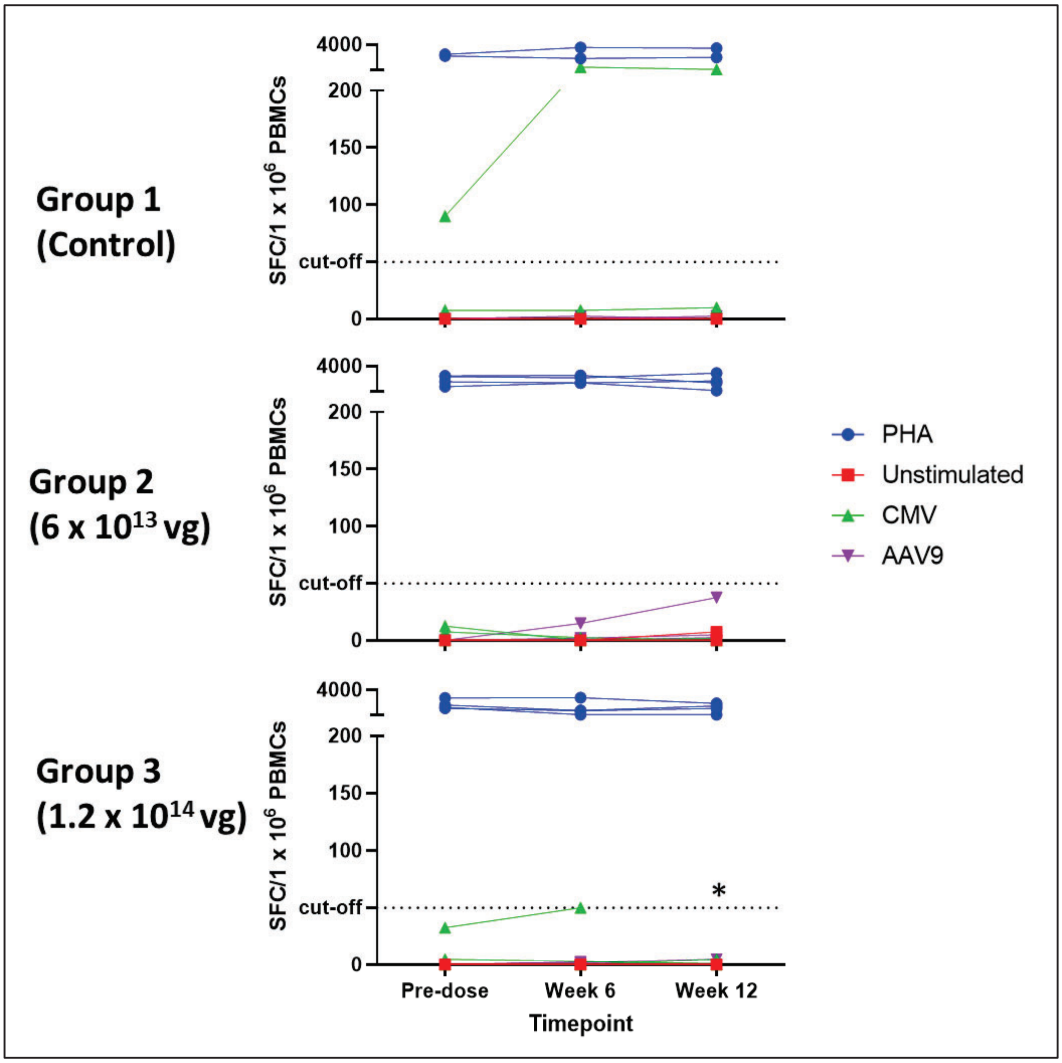

Figure 3. Time course of AAV-9 specific T cell responses by IFN-γ ELISpot

Line graph showing individual responses by animals belonging to Group 1 (Control, Upper), Group 2 (6×10^{13} vg, Center) and Group 3 (1.2×10^{14} vg, Lower) study cohorts. Individual responses to PHA, Unstimulated, CMV and AAV9 antigen stimulation conditions are shown at the Pre-Dose, Week 6, and Week 12 timepoints. The number of SFCs per 1×10^6 PBMCs plated is shown on the y-axis, for each study timepoint (x-axis). The cut-off value of 50 SFC/ 1×10^6 PBMCs for identifying positive responses is depicted by a dotted line. * = CMV antigen stimulation was not performed for Animal 353 at the Week 12 timepoint due to limited sample availability.

7.0 CONCLUSIONS

There were no identifiable trends associated with the dose in any group. While responses to positive control mitogen conditions were consistent across all study animals and test plates, none of the study animals showed detectable responses to the test antigen AAV9 at the Pre-dose, 6-week, or 12-week time points.

8.0 REFERENCES

Kalyuzhny, A. E. (2012). *Handbook of ELISPOT: Methods and Protocols* (2nd ed.). Humana Press. https://doi.org/10.1007/978-1-61779-325-7

Moodie, Z., Price, L., Gouttefangeas, C., et al. (2010). Response definition criteria for ELISPOT assays revisited. *Cancer Immunol Immunother* 59, 1489–1501. https://doi.org/10.1007/s00262-010-0875-4

Attachment 1. PBMC Isolation, Cryopreservation, and Storage Methods

Page	Page 1 of 7	amplifybio
Study Number	A-579-01	
Title	**A-579-01 Study Specific Method For PBMC Isolation, Cryopreservation and Storage**	
Batch ID:		
Performed By Initial and Date:		

1.0 PURPOSE

This study-specific method (SSM) describes the procedure for PBMC isolation, cryopreservation and storage per A-579-01 study requirements. The scientist's initials and dates on each page ("Performed by Initial and Date") signify that they have followed the instructions as written, verified that the materials and equipment remained the same as those listed in the tables, and all equipment has been properly calibrated. Any deviation to this method will be approved by the study director.

2.0 MATERIALS

Record the manufacturer, catalog number, lot number, and expiration date as applicable for each of the following materials.

Material	Manufacturer	Catalog Number	Lot Number	Storage Condition	Expiration Date
CryoStor CS10	Biolife Solutions	210502	23013	2 to 8ºC	JAN2024
Ficoll-Paque PREMIUM	Cytiva	17544202	10335095	Ambient	DEC2024
HBSS	Corning	MT21022CM	33422008	2 to 8ºC	DEC2025
Sepmate-50	StemCell Technologies	85450	464691	Ambient	18AUG2027
Conical Tubes (50mL)				Ambient	
Conical tubes (15mL)				Ambient	
Cryovials				Ambient	

Quality Control Review: ____________________ Technical Review: ______________________
Initials/Date Initials/Date

Page	Page 2 of 7	
Study Number	A-579-01	
Title	**A-579-01 Study Specific Method For PBMC Isolation, Cryopreservation and Storage**	
Batch ID:		
Performed By Initial and Date:		

3.0 EQUIPMENT

Record the equipment ID as applicable below.

Equipment	Equipment ID
Biosafety Cabinet (BSC), ISO Class 2	
Refrigerator, 2 to 8 °C	
Freezer, -60 to -85 °C	
Centrifuge	
Mr. Frosty Freezing Container	
Mr. Frosty Freezing Container	
Mr. Frosty Freezing Container	
Mr. Frosty Freezing Container	
Micropipette	
Micropipette	
Micropipette	
Micropipette	
Sample Rocker	

Quality Control Review: ____________________ Technical Review: ______________________
Initials/Date Initials/Date

Page	Page 3 of 7	
Study Number	A-579-01	
Title	**A-579-01 Study Specific Method For PBMC Isolation, Cryopreservation and Storage**	
Batch ID:		
Performed By Initial and Date:		

4.0 PREPARATION

4.1 Prepare the BSC by wiping down the inside surface of the BSC using 70% ethanol or equivalent, fill out the BSC use log.

4.2 Gather an appropriate stock of pipetting strippets (range 5mL to 50mL), conical tubes (appropriately labeled), transfer pipettes, spray all with 70% ethanol and set aside within the BSC.

4.3 Prepare the appropriate number of Sepmate 50 tubes by adding 15mL of Ficoll below the frit barrier. Label the tubes and set aside in the BSC.

4.4 Aliquot an appropriate amount of HBSS wash buffer into sterile 50mL conicals for use in the processing, label the tubes and set aside within the BSC.

4.5 Place an appropriate number of Mr. Frosty freezing devices inside the 2-8 °C refrigerator to chill.

4.6 Verify blood samples were placed on rocker at room temperature upon receipt.

5.0 PROCEDURE

Table 1

**Animal ID**				
**Sample ID**				
**Time Point**				
**Collection Time [a]**				
**Blood Condition[b]**				
**Usable Blood Volume (mL)**				
**Processing Start Time[c]**				
**Processing End Time[d]**				
**Total Processing Time[e]**				

a. Collection time is a transcription from the Sample Collection Form.
b. NORM = Normal; HEM = Hemolyzed; CLT = Clotted; BIL=Bilirubin
c. Recorded at the time at which the blood sample tube is first opened.
d. Recorded at the time PBMC samples are transferred to the -60 to -85° C freezer.
e. Calculated = processing end time – processing start time.

Quality Control Review: ____________________ Initials/Date   Technical Review: ____________________ Initials/Date

Page	Page 4 of 7	amplifybio
Study Number	A-579-01	
Title	**A-579-01 Study Specific Method For PBMC Isolation, Cryopreservation and Storage**	
Batch ID:		
Performed By Initial and Date:		

5.1 **BLOOD DILUTION**: For each approximate 6mL blood sample, dilute the blood with sufficient volume of HBSS to achieve a total volume of 20mL in a clean 50mL conical tube appropriately labeled with animal ID. Record usable blood volume in **Table 1** (eg. 5.2 mL).

5.2 Carefully and slowly add 20mL of diluted blood with a serological pipette to the prepared Sepmate tube with Ficoll from step 4.3 (above the frit barrier). Keep the tube vertical and let diluted blood stream down the side of the tube.

5.3 Centrifuge the sample at 1200 x g for 10 minutes at room temperature, with brake set to 3. Note: The centrifuge will take additional time to come to a complete stop.

5.4 Remove the sample from the centrifuge, and check for a buffy coat layer. If no buffy coat layer is visible, contact your supervisor and record the steps taken in the comment section.

5.5 Using a transfer pipette, transfer the buffy coat layer into a clean, labeled 50mL conical tube prefilled with 20 mL of HBSS.
Caution: Make sure to not transfer too much of the platelet rich plasma layer, or the residual layer of Ficoll from above the frit barrier.

5.6 **WASH 1:** Bring the total volume of the tube to approximately 40mL with HBSS, centrifuge at 400 x g for 10 minutes at room temperature, with brake set to 9.

5.7 Check for cell pellet after centrifugation. If no pellet is visible, contact your supervisor and record the steps taken in comment section.

5.8 Carefully aspirate the sample supernatant.

5.9 **WASH 2:** Resuspend the cell pellet in approximately 40mL total volume of HBSS, centrifuge at 400 x g for 10 minutes at room temperature, with brake set to 9.

5.10 Check for cell pellet after centrifugation. If no pellet is visible, contact your supervisor and record the steps taken in the comment section.

5.11 Carefully aspirate the sample supernatant.

Quality Control Review: ____________________ Technical Review: ______________________
Initials/Date Initials/Date

Page	Page 5 of 7	amplifybio
Study Number	A-579-01	
Title	**A-579-01 Study Specific Method For PBMC Isolation, Cryopreservation and Storage**	
Batch ID:		
Performed By Initial and Date:		

5.12 Add 2mL HBSS to the sample tube. Gently resuspend the cell pellet using a 1000 µL pipette. Obtain a viable cell count using ImmunoSpot according to SOP-00458 and FORMTEMP-00663. Perform necessary calculations using cell count data in FORMTEMP-00663 and record in **Table 2**.
A second operator will verify calculations.

Table 2

**Sample ID**				
**Counting Resuspension Volume (mL HBSS)**	2	2	2	2
**Viable cells/mL**[a,b]				
**%Viability**[a]				
**Total Viable Cell yield**[a,b]				
**Viable cells/mL of processed blood (total viable cells / useable blood volume)**[b]				
**Cryostor CS10 resuspension volume (mL)** [a,b,c]				
**Residual Cell Volume (mL)** [b]				
**Number of Aliquots (cryovials)**				
**Cryopreservation, concentration of frozen cells (cells/mL)**[c]	$1x10^7$	$1x10^7$	$1x10^7$	$1x10^7$

a. All values provided are based on average of duplicate sample readings taken on the instrument (FORMTEMP-00663).
b. All data rounded to two decimal places
c. Target concentration of frozen cells is $1x10^7$ cells/mL.

Calculations verified by (Initial/date): ________________________

5.13 Prefill cryopreservation labels using the electronic template according to the following format and label all required cryovials per **Table 2**:

Sample ID<br>Timepoint NaHep Cyno<br>[Total viable cell count]<br>(Ex: 5 x 10^6 viable cells)

Affix copies of all labels in Section 5.21.

Quality Control Review: ____________________ Technical Review: ______________________
Initials/Date Initials/Date

Page	Page 6 of 7	amplifybio
Study Number	A-579-01	
Title	**A-579-01 Study Specific Method For PBMC Isolation, Cryopreservation and Storage**	
Batch ID:		
Performed By Initial and Date:		

5.14 Bring the total volume of the cell suspension to 10mL by adding 8mL of HBSS. Transfer to a labeled 15mL conical for the final cell spin.

5.15 Centrifuge sample at 400 x g for 5 minutes at room temperature, with brake set to 9.

5.16 Check for cell pellet. If no pellet is visible, contact your supervisor and record in the comments.

5.17 Remove an appropriate number of Mr. Frosty freezing devices and Cryostor CS10 Cryomedia from the refrigerator. Verify Mr. frosty freezing devices were placed into the refrigerator for at least 10 minutes (Step 4.5).

5.18 Aspirate HBSS supernatant. Resuspend the pellet in the volume of Cryostor CS10 Cryomedia calculated in **Table 2**. Aliquot 0.5 mL per cryovial (5 x 10^6 viable cells/ cryovial) or 10 x 10^6 viable cells/mL.

For residual sample: ≤ 0.250 mL, combine to the previous cryovial,

> 0.250 mL, aliquot to its own cryovial.

Record Residual cell volume in **Table 2**.

5.19 Place cryovials into the Mr. Frosty freezing device and place in 2-8°C refrigerator for 10 minutes. Record start and stop time in **Table 3**.

Table 3

**Mr. Frosty Start Time**		**Mr. Frosty Stop Time**	

5.20 Transfer Mr. Frosty freezing device into the -60 to -85°C freezer. Record the time of transfer to -60 to -85°C freezer as the "process end time" and record total processing time in **Table 1**.

NOTE: Samples will be transferred to the LN2 24 hours +/- 4 hours and be recorded according to SOP-00381.

Quality Control Review: ____________________ Technical Review: ______________________
Initials/Date Initials/Date

Page	Page 7 of 7	
Study Number	A-579-01	
Title	**A-579-01 Study Specific Method For PBMC Isolation, Cryopreservation and Storage**	
Batch ID:		
Performed By Initial and Date:		

5.21 **Affix copies of sample labels here:**

Comments:

Quality Control Review: ____________________ Technical Review: ______________________
Initials/Date Initials/Date

Document Approvals

Number: METHOD-00086 Version: 3.0

Approval Task<br>Verdict: Approve	Sapna Varia,<br>(svaria@amplify-bio.com)<br>Approval<br>12-Sep-2023 13:09:24 GMT+0000

Page	Page 1 of 7
Study Number	A-579-01
Title	**A-579-01 Study Specific Method For PBMC Isolation, Cryopreservation and Storage**
Batch ID:	
Performed By Initial and Date:	

1.0 PURPOSE

This study-specific method (SSM) describes the procedure for PBMC isolation, cryopreservation and storage per A-579-01 study requirements. The scientist's initials and dates on each page ("Performed by Initial and Date") signify that they have followed the instructions as written, verified that the materials and equipment remained the same as those listed in the tables, and all equipment has been properly calibrated. Any deviation to this method will be approved by the study director.

2.0 MATERIALS

Record the manufacturer, catalog number, lot number, and expiration date as applicable for each of the following materials.

Material	Manufacturer	Catalog Number	Lot Number	Storage Condition	Expiration Date
CryoStor CS10	Biolife Solutions	210502	23013	2 to 8ºC	JAN2024
Ficoll-Paque PREMIUM	Cytiva	17544202	10335095	Ambient	DEC2024
HBSS	Corning	MT21022CM	33422008	2 to 8ºC	DEC2025
Sepmate-50	StemCell Technologies	85450	464691	Ambient	18AUG2027
Conical Tubes (50mL)				Ambient	
Conical tubes (15mL)				Ambient	
Cryovials				Ambient	

Quality Control Review: ____________________ Technical Review: ______________________
Initials/Date Initials/Date

Page	Page 2 of 7	
Study Number	A-579-01	
Title	**A-579-01 Study Specific Method For PBMC Isolation, Cryopreservation and Storage**	
Batch ID:		
Performed By Initial and Date:		

3.0 EQUIPMENT

Record the equipment ID as applicable below.

Equipment	Equipment ID
Biosafety Cabinet (BSC), ISO Class 2	
Refrigerator, 2 to 8 °C	
Freezer, -60 to -85 °C	
Centrifuge	
Mr. Frosty Freezing Container	
Mr. Frosty Freezing Container	
Mr. Frosty Freezing Container	
Mr. Frosty Freezing Container	
Micropipette	
Micropipette	
Micropipette	
Micropipette	
Sample Rocker	

Quality Control Review: ____________________ Technical Review: ____________________
Initials/Date Initials/Date

Page	Page 3 of 7	
Study Number	A-579-01	
Title	**A-579-01 Study Specific Method For PBMC Isolation, Cryopreservation and Storage**	
Batch ID:		
Performed By Initial and Date:		

4.0 PREPARATION

4.1 Prepare the BSC by wiping down the inside surface of the BSC using 70% ethanol or equivalent, fill out the BSC use log.

4.2 Gather an appropriate stock of pipetting strippets (range 5mL to 50mL), conical tubes (appropriately labeled), transfer pipettes, spray all with 70% ethanol and set aside within the BSC.

4.3 Prepare the appropriate number of Sepmate 50 tubes by adding 15mL of Ficoll below the frit barrier. Label the tubes and set aside in the BSC.

4.4 Aliquot an appropriate amount of HBSS wash buffer into sterile 50mL conicals for use in the processing, label the tubes and set aside within the BSC.

4.5 Place an appropriate number of Mr. Frosty freezing devices inside the 2-8 °C refrigerator to chill.

4.6 Verify blood samples were placed on rocker at room temperature upon receipt.

5.0 PROCEDURE

Table 1

**Animal ID**				
**Sample ID**				
**Time Point**				
**Collection Time** [a]				
**Blood Condition**[b]				
**Usable Blood Volume (mL)**				
**Processing Start Time**[c]				
**Processing End Time**[d]				
**Total Processing Time**[e]				

a. Collection time is a transcription from the Sample Collection Form.
b. NORM = Normal; HEM = Hemolyzed; CLT = Clotted; BIL=Bilirubin
c. Recorded at the time at which the blood sample tube is first opened.
d. Recorded at the time PBMC samples are transferred to the -60 to -85° C freezer.
e. Calculated from the processing start time and the processing end time.

Quality Control Review: ____________________ Initials/Date   Technical Review: ______________________ Initials/Date

Page	Page 4 of 7	
Study Number	A-579-01	
Title	**A-579-01 Study Specific Method For PBMC Isolation, Cryopreservation and Storage**	
Batch ID:		
Performed By Initial and Date:		

5.1 **BLOOD DILUTION**: For each approximate 6mL blood sample, dilute the blood with sufficient volume of HBSS to achieve a total volume of 20mL in a clean 50mL conical tube appropriately labeled with animal ID. Record usable blood volume in **Table 1** (eg. 5.2 mL).

5.2 Carefully and slowly add 20mL of diluted blood with a serological pipette to the prepared Sepmate tube with Ficoll from step 4.3 (above the frit barrier). Keep the tube vertical and let diluted blood stream down the side of the tube.

5.3 Centrifuge the sample at 1200 x g for 10 minutes at room temperature, with brake set to 3. Note: The centrifuge will take additional time to come to a complete stop.

5.4 Remove the sample from the centrifuge, and check for a buffy coat layer. If no buffy coat layer is visible, contact your supervisor and record the steps taken in the comment section.

5.5 Using a transfer pipette, transfer the buffy coat layer into a clean, labeled 50mL conical tube prefilled with 20 mL of HBSS.
Caution: Make sure to not transfer too much of the platelet rich plasma layer, or the residual layer of Ficoll from above the frit barrier.

5.6 **WASH 1:** Bring the total volume of the tube to approximately 40mL with HBSS, centrifuge at 400 x g for 10 minutes at room temperature, with brake set to 9.

5.7 Check for cell pellet after centrifugation. If no pellet is visible, contact your supervisor and record the steps taken in comment section.

5.8 Carefully aspirate the sample supernatant.

5.9 **WASH 2:** Resuspend the cell pellet in approximately 40mL total volume of HBSS, centrifuge at 400 x g for 10 minutes at room temperature, with brake set to 9.

5.10 Check for cell pellet after centrifugation. If no pellet is visible, contact your supervisor and record the steps taken in the comment section.

5.11 Carefully aspirate the sample supernatant.

Quality Control Review: ____________________ Initials/Date   Technical Review: ______________________ Initials/Date

Page	Page 5 of 7	
Study Number	A-579-01	
Title	**A-579-01 Study Specific Method For PBMC Isolation, Cryopreservation and Storage**	
Batch ID:		
Performed By Initial and Date:		

5.12 Add 2mL HBSS to the sample tube. Gently resuspend the cell pellet using a 1000 µL pipette. Obtain a viable cell count using ImmunoSpot according to SOP-00458 and FORMTEMP-00663. Perform necessary calculations using cell count data in FORMTEMP-00663 and record in **Table 2**.
A second operator will verify calculations.

Table 2

**Sample ID**				
**Counting Resuspension Volume (mL HBSS)**	2	2	2	2
**Viable cells/mL[a]**				
**%Viability[a]**				
**Total Viable Cell yield (viable cells/mL x volume)**				
**Viable cells/mL of processed blood (total viable cells / useable blood volume)**				
**Cryostor CS10 resuspension volume (mL)**				
**Residual Cell Volume (mL)**				
**Number of Aliquots (cryovials)**				
**Cryopreservation, concentration of frozen cells (cells/mL)[b]**				

a. All values provided are based on average of duplicate sample readings taken on the instrument (FORMTEMP-00663).
b. Target concentration of frozen cells is $1x10^7$ cells/mL.

Calculations verified by (Initial/date): ________________________

5.13 Prefill cryopreservation labels using the electronic template according to the following format and label all required cryovials per **Table 2**:

Sample ID<br>Timepoint NaHep Cyno<br>[Total viable cell count]<br>(Ex: 5 x 10^6 viable cells)

Affix copies of all labels in Section 5.21.

Quality Control Review: ____________________ Technical Review: ______________________
Initials/Date Initials/Date

Page	Page 6 of 7	amplifybio
Study Number	A-579-01	
Title	**A-579-01 Study Specific Method For PBMC Isolation, Cryopreservation and Storage**	
Batch ID:		
Performed By Initial and Date:		

5.14 Bring the total volume of the cell suspension to 10mL by adding 8mL of HBSS. Transfer to a labeled 15mL conical for the final cell spin.

5.15 Centrifuge sample at 400 x g for 5 minutes at room temperature, with brake set to 9.

5.16 Check for cell pellet. If no pellet is visible, contact your supervisor and record in the comments.

5.17 Remove an appropriate number of Mr. Frosty freezing devices and Cryostor CS10 Cryomedia from the refrigerator.

5.18 Resuspend in the volume of Cryostor CS10 Cryomedia calculated in **Table 2**. Aliquot 0.5 mL per cryovial (5×10^6 viable cells/ cryovial) or 10×10^6 viable cells/mL.

For residual sample: ≤ 0.250 mL, combine to the previous cryovial,

> 0.250 mL, aliquot to its own cryovial.

Record Residual cell volume in **Table 2**.

5.19 Place cryovials into the Mr. Frosty freezing device and place in 2-8°C refrigerator for a minimum of 10 minutes. Record start and stop time in **Table 3**.

Table 3

**Mr. Frosty Start Time**		**Mr. Frosty Stop Time**	

5.20 Transfer Mr. Frosty freezing device into the -60 to -85°C freezer. Record the time of transfer to -60 to -85°C freezer as the "process end time" and record total processing time in **Table 1**.

Quality Control Review: ____________________ Technical Review: ______________________
Initials/Date Initials/Date

Page	Page 7 of 7
Study Number	A-579-01
Title	**A-579-01 Study Specific Method For PBMC Isolation, Cryopreservation and Storage**
Batch ID:	
Performed By Initial and Date:	

5.21 **Affix copies of sample labels here:**

Comments:

Quality Control Review: ____________________ Initials/Date

Technical Review: ____________________ Initials/Date

Document Approvals

Number: METHOD-00086 Version: 2.0

Approval Task<br>Verdict: Approve	Sapna Varia,<br>(svaria@amplify-bio.com)<br>Approval<br>31-Jul-2023 17:11:18 GMT+0000

Page	Page 1 of 13	
Study Number	A-579-01	
Title	**A-579-01 Study Specific Method For PBMC Isolation, Cryopreservation and Storage**	
Batch ID:		

1.0 PURPOSE

This form describes the procedure for PBMC isolation, cryopreservation and storage per A-579-01 study requirements.

2.0 PROCEDURES

2.1 General Instructions

Initial and date as appropriate next to each step. Each step requires the signature of the operator performing the work. In the cases where the operator is unable to authenticate immediately (when working in a hood or biosafety cabinet) a recorder will sign to verify that operations were completed as specified. The recorder's signature also verifies that they have performed any tasks assigned to them in the same step. All steps must be performed and recorded in order before moving onto subsequent steps unless otherwise stated by the section. Other entries made by the technician on a later date or entries made by another person will be initialed and dated near the data entry. The technicians' initials and dates on each page signify that they have followed the instructions as written, verified that the materials and equipment remained the same as those listed in the tables, and all equipment has been properly calibrated. The materials and equipment documented in the tables are used throughout the procedure by all technicians performing the procedure unless otherwise specified. Any corrections made to the form will be approved by the study director by writing "approved" and initialing and dating the entry or change.

2.2 Batch ID

The Batch ID used throughout this study will include the Study number.collection date.run number.

Sample IDs will be recorded for each batch throughout the document and will contain Animal ID.aliquot details(such as date/time).

2.3 Preparation Activities

Prepare working stations ensuring that all required materials for the steps are available prior to start and that none are expired or damaged. Ensure all equipment is available for operation and is cleaned and prepared.

Page	Page 2 of 13	amplifybio
Study Number	A-579-01	
Title	**A-579-01 Study Specific Method For PBMC Isolation, Cryopreservation and Storage**	
Batch ID:		

2.4 Sample receipt

Ensure any prepared labels are accurate to the samples being used as well as those recorded within this form.

Sample ID	Animal ID	Collection Time*	Time Received*

*Collection Time will be transcribed from received sample collection information sheet.

**More rows may be added as needed.

Recorded by: ____________________ Initials/Date

Verified by: ____________________ Initials/Date

QC Review: ____________________ Initials/Date

Technical Review: ____________________ Initials/Date

Page	Page 3 of 13	
Study Number	A-579-01	
Title	**A-579-01 Study Specific Method For PBMC Isolation, Cryopreservation and Storage**	
Batch ID:		

2.5 Materials/Reagents

See **Table 1** for all required materials. Check the labels carefully to ensure that they are not expired and are the proper purity/grade. Document the lot number, catalog number and expiration date (if applicable) as appropriate on the form. Ensure any consumables are labeled appropriately prior to use.

Any extra materials used will be recorded in blank rows.

*An equivalent material may be used if the prerecorded material is unavailable.

Table 1. Materials

Material/Reagent	Manufacturer	Catalog Number	Lot Number	Expiration Date
CryoStor CS10*	Biolife Solutions	210502		
Mr. Frosty Freezing Container*	Thermo	5100-0001		
Ficoll-Paque PREMIUM	GE Healthcare	17-5442-02		
HBSS	Corning			
DMSO	Sigma			
Sepmate -15	Stemcell	85415		
Sepmate - 50	Stemcell	85460		

Recorded by: ______________________ Initials/Date

Verified by: ______________________ Initials/Date

QC Review: ______________________ Initials/Date

Technical Review: ______________________ Initials/Date

Page	Page 4 of 13	
Study Number	A-579-01	
Title	**A-579-01 Study Specific Method For PBMC Isolation, Cryopreservation and Storage**	
Batch ID:		

2.6 Equipment

See Table 2 for all required major pieces of equipment. Check calibration of all equipment requiring calibration (e.g., balances, centrifuges, pipettes) to ensure it is current. Document the identity of each piece of equipment on the form.

Any extra equipment used will be recorded in blank rows.

Table 2. Equipment

Equipment	Manufacturer	Serial Number/Equipment ID
Biosafety Cabinet (BSC), ISO Class 2		
Refrigerator, 2 to 8°C		
Freezer, -60 to -85 °C		
Centrifuge		
Micropipette, 100-1000 µL	Rainin	
Micropipette, 20-200 µL	Rainin	
Micropipette, 2-20 µL	Rainin	
Micropipette, 0.2-2 µL	Rainin	

Recorded by: ____________________ Initials/Date

Verified by: ____________________ Initials/Date

QC Review: ____________________ Initials/Date

Technical Review: ____________________ Initials/Date

Page	Page 5 of 13	amplifybio
Study Number	A-579-01	
Title	**A-579-01 Study Specific Method For PBMC Isolation, Cryopreservation and Storage**	
Batch ID:		

Step	PBMC Separation and Collection	Operator Initial/Date	Recorder Initial/Date
**1.0**	The following steps will be performed by an operator inside the BSC unless noted otherwise. The recorder will be signing for the identified recorder tasks as well as ensuring the operator has performed all of their required tasks.<br><br>**Operator:**<br><br>Name of Operator inside BSC: ____<br><br>**Recorder:**<br>Sign each of the following steps performed by the operator inside the BSC after the step is completed. Ensure all operations are completed and any requirements for you, the recorder, are complete.		
**1.1**	**Operator:**<br>Within the BSC, verbally communicate the condition of each sample to the recorder.<br><br>**Recorder:**<br>Record the condition of each sample in the following table. (see table below)<br>*more rows may be added and changes in sample condition may be recorded as needed.		

Animal ID	Tube Type	Usable Blood Volume (mL)	Sample Condition at Receipt (NORMAL/SAT/HEM/CLT)

*more rows may be added and changes in sample condition may be recorded as needed.

QC Review: ____________________ Initials/Date

Technical Review: ____________________ Initials/Date

Page	Page 6 of 13	
Study Number	A-579-01	
Title	**A-579-01 Study Specific Method For PBMC Isolation, Cryopreservation and Storage**	
Batch ID:		

Step	PBMC Separation and Collection	Operator Initial/Date	Recorder Initial/Date
1.2	**Operator:**<br>Within the BSC, transfer the blood to a new sterile conical tube. Communicate the volume to the recorder.<br><br>Dilute the blood 1:2 to 1:4 with HBSS. Communicate the volume of HBSS added to the recorder.<br><br>Invert the Ficoll several times.<br><br>*For Usable Blood Volume less than 3mL, pipette 4.5mL of Ficoll through the central hole of the 15mL SepMate insert. Communicate the volume added to the recorder.<br>**For Usable Blood Volume equal to or greater than 3mL, pipette 15 mL of Ficoll through the central hole of the 50mL SepMate insert. Commnicate the volume added to the recorder.<br>***Note: 1-2mL of Ficoll will be above the insert. Any small bubbles present will not affect the samples.<br><br>**Recorder:**<br>Record the volumes used in the following table:<br><br>Ensure the operator has performed all tasks.		

Animal ID	Usable Blood Volume (mL)	Volume HBSS Added (mL)	Volume Ficoll Added (mL)

*more rows may be added as needed.

QC Review: ____________________
Initials/Date

Technical Review: ____________________
Initials/Date

Page	Page 7 of 13	
Study Number	A-579-01	
Title	**A-579-01 Study Specific Method For PBMC Isolation, Cryopreservation and Storage**	
Batch ID:		

Step	PBMC Separation and Collection	Operator Initial/Date	Recorder Initial/Date
1.3	**Operator:**<br>Within the BSC, keeping the SepMate tube vertical, gently layer the diluted sample **ON TOP** of Ficoll with a transfer pipet for each tube. Complete for each sample. Ensure that tubes are labeled appropriately.<br><br>**Recorder:**<br>After the diluted sample(s) have been layered on top of the Ficoll, centrifuge at 1200 x g for 20 minutes at RT with the brake on 7 (out of 9).<br>*The spin will take an additional 30 seconds to 1 minute to come to a complete stop.<br><br>Record the Start and Stop time of centrifugation<br><br>Start Time: ______ Stop Time: ______<br><br>After centrifugation, return the samples to the operator within the BSC.<br><br>Ensure the operator has completed all tasks.		
1.4	**Operator:**<br>Within the BSC, for each SepMate tube, pour off the top layer containing the PBMCs into the new, sterile conical tubes. Do not hold the SepMate tube inverted for more than 2 seconds.<br><br>**Recorder:**<br>Verify operator has completed the step.		

QC Review: ____________________ Technical Review: ____________________
Initials/Date Initials/Date

Page	Page 8 of 13
Study Number	A-579-01
Title	**A-579-01 Study Specific Method For PBMC Isolation, Cryopreservation and Storage**
Batch ID:	

Step	PBMC Separation and Collection	Operator Initial/Date	Recorder Initial/Date
1.5	**Operator:**<br>Within the BSC, Fill the conical tube with HBSS (up to 10mL for a 15mL conical tube, up to 40mL for a 50mL conical tube). Communicate the volume added to the Recorder.<br><br>**Recorder:**<br>Record the total volume in the following table:<br><br>Once operator steps are complete, centrifuge at 400 x g for 10 minutes at RT **with the brake back to 9.**		

Animal ID	Volume filled to with HBSS (mL)

*more rows may be added as needed.

QC Review: ____________________ Initials/Date

Technical Review: ____________________ Initials/Date

Page	Page 9 of 13	amplifybio
Study Number	A-579-01	
Title	**A-579-01 Study Specific Method For PBMC Isolation, Cryopreservation and Storage**	
Batch ID:		

Step	PBMC Separation and Collection	Operator Initial/Date	Recorder Initial/Date
**2.0**	**Operator:**<br>Within the BSC, aspirate the supernatant. Resuspend cell pellet in 10 mL or 40mL of HBSS. Commnicate the volume to the recorder.<br><br>**Recorder:**<br>Record the volume added in the following table:<br><br>Ensure the operator has completed all tasks.<br><br>Centrifuge at 400 x g for 10 minutes at RT.		

Animal ID	Volume of HBSS resuspended in (mL)

QC Review: ____________________ Initials/Date
Technical Review: ____________________ Initials/Date

Page	Page 10 of 13	amplifybio
Study Number	A-579-01	
Title	**A-579-01 Study Specific Method For PBMC Isolation, Cryopreservation and Storage**	
Batch ID:		

Step	Counting	Operator Initial/Date	Recorder Initial/Date
2.1	**Operator:**<br>Within the BSC, Aspirate supernatant.<br><br>Resuspend cell pellet in HBSS at a target of half the original blood volume.<br><br>**Recorder:**<br>Record the final suspension volume in the following table:<br><br>Ensure the operator has completed all tasks.<br><br>*more rows may be added as needed.		
2.2	**Operator:**<br>Run the samples on the Vi-CELL BLU or S6 Universal M2 Analyzer. See FORMTEMP-00588 Vi-CELL BLU Cell Count Form or FORMTEMP-00663 S6 Universal M2 Analyzer: Cell Counting Form for cell counts and concentrations.		

Animal ID	Final Suspension Volume (mL)

QC Review: ____________________ Initials/Date

Technical Review: ____________________ Initials/Date

Page	Page 11 of 13	amplifybio
Study Number	A-579-01	
Title	**A-579-01 Study Specific Method For PBMC Isolation, Cryopreservation and Storage**	
Batch ID:		

Step	Cryopreserving Mononuclear Cells	Operator Initial/Date	Recorder Initial/Date
**3.1**	**Operator:**<br>Label the cryogenic vials and keep them in the BSC. Transfer the Mr. Frosty to the 2-8˚C refrigerator.<br><br>Centrifuge the tube from step 2.1 at 400 x g for 10 minutes at RT.<br><br>Remove the CryoStor CS10 freezing media from the 2-8ºC refrigerator.<br><br>**Recorder:**<br>Ensure the operator has completed all steps.		
**3.2**	The following steps will be performed by an operator inside the BSC until noted otherwise. The Recorder will be signing for the identified Recorder tasks as well as ensuring the operator has performed all of their required tasks.<br><br>**Operator:**<br><br>Name of Operator inside BSC: ________<br><br>**Recorder:**<br>Sign each of the following steps performed by the operator inside the BSC after the step is completed. Ensure all operations are completed and any requirements for you, the recorder, are complete.		

QC Review: ________________ Initials/Date   Technical Review: ________________ Initials/Date

Page	Page 12 of 13
Study Number	A-579-01
Title	**A-579-01 Study Specific Method For PBMC Isolation, Cryopreservation and Storage**
Batch ID:	

Step	Cryopreserving Mononuclear Cells	Operator Initial/Date	Recorder Initial/Date
**3.3**	**Operator:**<br>Carefully aspirate the supernatant, leaving a small amount of medium to ensure the cell pellet is not disturbed.<br><br>Based upon the cell counts, add 1mL cold (2-8°C) CryoStor CS10 media per cryovial being preserved. Mix gently with pipette after media addition. Cells are to be frozen back at a target goal of $5x10^6$ cells per vial. See SOP-00457, Section 13.3.4 for calculations.<br><br>Transfer 0.5 to 1mL of suspended sample to each cryogenic vial based on the cell concentration. Transfer the cryogenic vials to the cold Mr. Frosty.<br><br>**Recorder:**<br>Ensure the operator has completed all steps. Verify cryovials are transferred to Mr.Frosty and record the number of vials cryopreserved, concentration and total volume frozen in the table below.		

Animal ID	Number of Vials Cryopreserved	Final Concentration ($x10^6$ cells/mL)	Total Volume Frozen (mL)

QC Review: ____________________ Initials/Date

Technical Review: ____________________ Initials/Date

Page	Page 13 of 13	
Study Number	A-579-01	
Title	**A-579-01 Study Specific Method For PBMC Isolation, Cryopreservation and Storage**	
Batch ID:		

Step	Cryopreserving Mononuclear Cells	Operator Initial/Date	Recorder Initial/Date
3.4	**Operator:**<br>Outside of the BSC, Incubate the cryovials in within a Mr. Frosty in a refrigerator set to maintain a temperature of 2-8°C for approximately 10 minutes after CryoStor CS10 is added and samples are transferred.<br><br>**Recorder:**<br>Record the start and stop time of incubation.<br><br>Start Time: ____ Stop Time: ____		
3.5	**Operator:**<br>Outside of the BSC, Store the Mr. Frosty in the -60°C to -85°C freezer.<br><br>**Recorder:**<br>Verify samples were stored and record the time and location of storage.<br><br>Storage Time: ____ Storage location: ____		

Comments:

____

____

____

____

____

QC Review: ____________________ Technical Review: ____________________
Initials/Date Initials/Date

Document Approvals
Number: METHOD-00086 Version: 1.0

Approval Task<br>Verdict: Approve	Sapna Varia,<br>(svaria@amplify-bio.com)<br>Approval<br>14-Jun-2023 19:16:11 GMT+0000

Attachment 2. IFN-γ Peptide ELISpot Methods

Page:	Page 1 of 19	
Study Number:	A-579-01	
Title:	**A-579-01 Study Specific Method for IFN-γ Peptide ELISpot**	
Batch ID:		
Performed By Initial and Date:		

1. Purpose

This study-specific method (SSM) describes the procedure for IFN-γ Peptide ELISpot per A-579-01 study requirements. The scientist's initials and dates on each page ("Performed by Initial and Date") signify that they have followed the instructions as written, verified that the materials and equipment remained the same as those listed in the tables, and all equipment has been properly calibrated. Any deviation to this method will be approved by the study director.

2. Media Preparation

2.1. Equipment:

Record the equipment utilized in the following table.

Equipment (Description)	Equipment ID
Biosafety Cabinet	
Water bath (37°C ± 2°C)	
Refrigerator 2-8°C	
Freezer (-30 to -15°C)	
Micropipette	
Micropipette	
Micropipette	
Micropipette	

Technical Review: ____________________________ QC Review: ____________________________

Page:	Page 2 of 19
Study Number:	A-579-01
Title:	**A-579-01 Study Specific Method for IFN-γ Peptide ELISpot**
Batch ID:	
Performed By Initial and Date:	

2.2. Materials:

Record the materials utilized in the following table.

Reagents/Materials	Manufacturer	Cat. No.	Lot No.	Expiration Date
RPMI	Corning	10-040-CV		
FBS	Cytiva	SH30071.03		
0.2 µM Vacuum Filter				

2.3. Preparing the Complete RPMI/10% FBS (R10) Media

Table 1. Volume of Complete R10 Media Needed

Calculate the Volume of Complete R10 Media Needed.

(A) **Number of Samples**	(B) **Volume Needed Per Sample (mL)**	(C) **Total Volume of Complete R10 Media Needed (mL)** (C)=(A) x (B) x (1.1)	(D) **Volume of FBS (mL)** (D) = (C) x (0.1)	(E) **Volume of RPMI Media Needed (mL)** (E) = (C) – (D)
	25			

Calculations Verified by: ______________________________
Initials/Date

1. Retrieve the necessary volume of frozen FBS and place in 37°C water bath to thaw and agitate periodically.
   * Do not hold FBS at 37°C for longer than necessary to thaw.
2. Add the FBS to the RPMI Media (see **Table 1** for volumes) and filter using a 0.2 µm vacuum filter.
3. Label bottle with RPMI/10% FBS, expiration date (24 hours), initials, and storage requirements.
4. Store in refrigerator (2-8°C) for future use or place in water bath for immediate use.

Technical Review: ________________________ QC Review: ____________________________

Page:	Page 3 of 19	amplifybio
Study Number:	A-579-01	
Title:	**A-579-01 Study Specific Method for IFN-γ Peptide ELISpot**	
Batch ID:		
Performed By Initial and Date:		

3. Preparing the ELISpot Plate(s)

3.1. Equipment:

Record the equipment utilized in the following table.

Equipment (Description)	Equipment ID
Biosafety Cabinet	
Centrifuge	
Micropipette	
Micropipette	
Micropipette	
Micropipette	
Multi-channel Pipette	
Liquid Nitrogen Storage (-195 to -135°C)	
Freezer (-85 to -60°C)	
Refrigerator 2-8°C	
Incubator (37°C ± 2°C, 5% ± 2% CO_2)	
Water bath (37°C ± 2°C)	

Technical Review: ________________________ QC Review: ____________________________

Page:	Page 4 of 19	
Study Number:	A-579-01	
Title:	**A-579-01 Study Specific Method for IFN-γ Peptide ELISpot**	
Batch ID:		
Performed By Initial and Date:		

3.2. Materials:

Record the materials utilized in the following table.

Reagents/Materials	Manufacturer	Cat. No.	Lot No.	Expiration Date
Single-color Enzymatic Hu IFN- γ Plate	Cellular Technology Limited	hIFNgp-2M/10		
Control Reagent: AAV9 Complete Pool Working Stock	AmplifyBio	AAV9-CompletePool-WS-4µg/mL		
Control Reagent: CyCMV UL55/UL83 Pool Working Stock	AmplifyBio	UL55-UL83-Pool-WS-2µg/mL		
Control Reagent: PHA-L Working Stock	AmplifyBio	PHA-L-WS-2ug/mL		
Control Reagent: 0.2% DMSO Working Stock	AmplifyBio	0.2%DMSO-WS		
Control Reagent: CEFx Ultra SuperStim Pool	AmplifyBio	CEFX-WS-2µg/mL		
1X PBS				

Technical Review: ________________________ QC Review: ____________________________

Page:	Page 5 of 19
Study Number:	A-579-01
Title:	**A-579-01 Study Specific Method for IFN-γ Peptide ELISpot**
Batch ID:	
Performed By Initial and Date:	

3.3. Preparing the Cells

1. Prepare 10 mL aliquots of R10 media in 15 mL conical tubes, make one aliquot for each cryovial being thawed, and place into a 37°C water bath, along with remaining complete media.
2. Remove the cryovial(s) from the LN2 and place on dry ice immediately. Keep on dry ice until ready to thaw.
3. Transfer the cryovial(s) to a 37°C water bath and rapidly swirl the cryovial(s) at the surface of the water.
   *Do not allow water to touch the caps of the cryovial.
4. When a small bit of ice remains in the vial, remove the cryovial(s) from the water and clean and dry off the outside of the vial(s) with ethanol, and transfer to a BSC II.
5. Slowly (dropwise) dispense up to 1 mL of R10 media (using aliquoted media from the 15mL conical tube) into the cryovial and gently pipette 1 to 2 times slowly to mix.
6. Transfer the entire cell suspension slowly (dropwise) from the cryovial to the corresponding 15mL conical tube with the remaining aliquoted media.
7. Add an additional 1mL of R10 media (from the corresponding aliquot) to the cryovial to collect any residual cells remaining and pipette the entire suspension back to the 15mL conical tube.
8. Centrifuge the cell suspension for 5 minutes, 400 x g, room temperature.
9. Carefully aspirate the supernatant. Gently resuspend the cell pellet in 10 mL of fresh R10 media.
10. Centrifuge the cell suspension for 5 minutes, 400 x g, room temperature.
11. Carefully aspirate the supernatant. Gently resuspend the cell pellet in 0.5 mL of fresh R10 media.

Technical Review: ____________________ QC Review: ____________________

Page:	Page 6 of 19
Study Number:	A-579-01
Title:	**A-579-01 Study Specific Method for IFN-γ Peptide ELISpot**
Batch ID:	
Performed By Initial and Date:	

12. For NHP samples, place the 15 mL conical tubes containing the cells into an incubator set at 37°C/5% CO_2 with caps vented for 3 hours ± 15 minutes.

Time Placed in Incubator: **Tubes for Plate 1** ____________________
Tubes for Plate 2 ____________________
Tubes for Plate 3 ____________________

3.4. Loading the Control Reagent(s)

1. Remove control reagents (2X) working stock solutions from the freezer and allow to thaw.
   Note: If making fresh control reagents follow FORMTEMP-00654 for specific instructions.
2. Label the side of the IFN-γ plate with "BatchID.initials.PlateNumber" (plate number will be 01, 02, or 03).

Plate ID: **Plate 1** ________________________________
Plate 2 ________________________________
Plate 3 ________________________________

3. Using a multichannel pipette, wash the plate with 150 µL/well of sterile PBS. Be sure to not touch the well membrane.
4. Flick the PBS out of the plate onto a sterile gauze pad or wypall and dispose of.
5. Add 100 µL of the prepared control reagent (2X) solution to the designated wells. Refer to the ELISpot IFN-γ Plate Map Form (FORMTEMP-00653) for control reagent locations.
6. Add 200 µL of complete R10 media to the designated "Blank" wells.
7. Once all control reagents and blanks have been added, place the plate into the incubator while cells are being counted.

Time Placed in Incubator: **Plate 1** ____________________
Plate 2 ____________________
Plate 3 ____________________

Technical Review: ______________________ QC Review: ________________________

Page:	Page 7 of 19	
Study Number:	A-579-01	
Title:	**A-579-01 Study Specific Method for IFN-γ Peptide ELISpot**	
Batch ID:		
Performed By Initial and Date:		

3.5. Plating the Cells

1. Remove the NHP sample tubes from the incubator.

Time out of Incubator:

Tubes for Plate 1 ______________________

Tubes for Plate 2 ______________________

Tubes for Plate 3 ______________________

2. Count the cells according to FORMTEMP-00663 S6 Universal M2 Analyzer: Cell Counting and record counts in **Table 2**.
3. Add the remaining volume of media needed to reach the desired concentration found in **Table 2**.
4. Remove the plate from the incubator.

Time Out of Incubator:

Plate 1 ______________________

Plate 2 ______________________

Plate 3 ______________________

5. Add 100 µL, dropwise around the well, of the desired concentration of cells (refer to **Table 2**) to each designated well within the plate. Refer to the ELISpot IFN-γ Plate Map Form (FORMTEMP-00653) for sample locations.
6. Place the finished plate onto the bottom shelf of an incubator set at 37°C/5% CO_2 for 24 ± 1 hours.

*Be very gentle when handling plates and be sure not to tilt when transferring to the incubator. Carefully open and close incubator doors. Do not stack plates.

Time Placed in Incubator:

Plate 1 ______________________

Plate 2 ______________________

Plate 3 ______________________

Technical Review: ________________________ QC Review: ____________________________

Page:	Page 8 of 19	
Study Number:	A-579-01	
Title:	**A-579-01 Study Specific Method for IFN-γ Peptide ELISpot**	
Batch ID:		
Performed By Initial and Date:		

Table 2. Concentration

**Sample ID**						
**Total Cells at Freezing (x 10^6)**						
**Counting Resuspension Volume (mL)**	0.5	0.5	0.5	0.5	0.5	0.5
**% Viability[a]**						
**Viable cells/mL[a,b]**						
**Total Viable Cell yield[a,b]**						
**% Recovery[a]**						
**Desired Final Concentration (x 10^6 cells/mL)[c]**	2	2	2	2	2	2
**Total Final Resuspension Volume[a] (mL)**						
**Volume of R10 Media Added to Reach Final Resuspension Volume (mL)**						

a. All values provided are based on average of duplicate sample readings taken on the instrument (FORMTEMP-00663).
b. All data rounded to two decimal places.
c. Target final concentration is $2x10^6$ per mL ($2x10^5$ per well), well concentration can range from $5x10^4$ to $5x10^5$ per qualification. Study director approval is required for changes in well concentration.

Calculations Verified by: ____________________________
Initials/Date

Technical Review: ____________________________ QC Review: ________________________________

Page:	Page 9 of 19
Study Number:	A-579-01
Title:	**A-579-01 Study Specific Method for IFN-γ Peptide ELISpot**
Batch ID:	
Performed By Initial and Date:	

Table 2. Concentration Continued

**Sample ID**						
**Total Cells at Freezing (x 10^6)**						
**Counting Resuspension Volume (mL)**	0.5	0.5	0.5	0.5	0.5	0.5
**% Viability[a]**						
**Viable cells/mL[a,b]**						
**Total Viable Cell yield[a,b]**						
**% Recovery[a]**						
**Desired Final Concentration (x 10^6 cells/mL)[c]**	2	2	2	2	2	2
**Total Final Resuspension Volume[a] (mL)**						
**Volume of R10 Media Added to Reach Final Resuspension Volume (mL)**						

a. All values provided are based on average of duplicate sample readings taken on the instrument (FORMTEMP-00663).
b. All data rounded to two decimal places.
c. Target final concentration is $2x10^6$ per mL ($2x10^5$ per well), well concentration can range from $5x10^4$ to $5x10^5$ per qualification. Study director approval is required for changes in well concentration.

Calculations Verified by: ____________________
Initials/Date

Technical Review: ____________________ QC Review: ____________________

Page:	Page 10 of 19	amplifybio
Study Number:	A-579-01	
Title:	**A-579-01 Study Specific Method for IFN-γ Peptide ELISpot**	
Batch ID:		
Performed By Initial and Date:		

4. Developing the ELISpot Plate(s)

4.1. Equipment:

Record the equipment utilized in the following table.

Equipment (Description)	Equipment ID
Biosafety Cabinet	
Micropipette	
Micropipette	
Micropipette	
Micropipette	
Multi-channel Pipette	
Refrigerator 2-8°C	
Microplate Washer	
Water Dispenser	

4.2. Materials:

Record the materials utilized in the following table.

Reagents/Materials	Manufacturer	Cat. No.	Lot No.	Expiration Date
Single-color Enzymatic Hu IFN- γ Kit (This includes Anti-human IFN-γ (Biotin) Detection Antibody, Diluent B, Diluent C, Diluent Blue, Strep-AP, S1, S2, and S3)	Cellular Technology Limited	hIFNgp-2M/10		
0.1 µm low protein binding filter				
1X PBS				

Technical Review: ______________________ QC Review: ________________________

Page:	Page 11 of 19
Study Number:	A-579-01
Title:	**A-579-01 Study Specific Method for IFN-γ Peptide ELISpot**
Batch ID:	
Performed By Initial and Date:	

4.3. Microplate Washer Buffers:

*Verify the plate washer has been primed according to SOP-00459 by checking the Agilent Plate Washer Use Log (FORMTEMP-00686).

Reagents/Materials	Cat. No.	Lot No.	Batch ID	Expiration Date
1X PBS				
0.05% Tween-PBS				
Distilled Water				

Technical Review: ________________________ QC Review: ____________________________

Page:	Page 12 of 19
Study Number:	A-579-01
Title:	**A-579-01 Study Specific Method for IFN-γ Peptide ELISpot**
Batch ID:	
Performed By Initial and Date:	

4.4. Detection Solution

1. In a 15 mL or 50 mL conical tube, add the Anti-human IFN- γ (Biotin) Detection Antibody to Diluent B.

(A) 1x Volume of Anti-human IFN-γ (Biotin) Detection Antibody	(B) 1x Volume of Diluent B	(C) Number of Plate(s)	(D) Total Volume of Anti-human IFN-γ (Biotin) Detection Antibody (A * C = D)	(E) Total Volume of Diluent B (B * C = E)
40 µL	10 mL			

2. Transfer the solution to a syringe with a 0.1 µm low protein binding filter attached and filter into a new 15 mL or 50 mL conical tube.
3. Remove the plate from the incubator and flick all liquid out of the plate into an absorbent pad in either the BSC, or a biohazard bin. Dispose of the pad into biohazard.
4. Using a multi-channel pipette, add 200 µL of PBS to each well to start the wash process.

Time of Wash: **Plate 1** ______________________

Plate 2 ______________________

Plate 3 ______________________

5. Using the protocol "1. ELISpot_Day_2_1_PBS_2_Tween" on the plate washer, wash the plate once with PBS, 200 µL/well, and then twice with 0.05% Tween-PBS, 200 µL/well.
6. Flick the residual buffer out of the plate into a biohazard bin.
7. Add the detection solution to a reagent reservoir.
8. Using a multi-channel pipette, add 80 µL of detection solution to each well.
9. With the plate lying flat, incubate for two hours ± 15 minutes at room temperature.

Incubation Start Time: **Plate 1** ______________________

Plate 2 ______________________

Plate 3 ______________________

Technical Review: ________________________ QC Review: ____________________________

Page:	Page 13 of 19	
Study Number:	A-579-01	
Title:	**A-579-01 Study Specific Method for IFN-γ Peptide ELISpot**	
Batch ID:		
Performed By Initial and Date:		

4.5. Tertiary Solution

1. Dilute Strep AP at a 1:1000 factor in Diluent C. Add Strep-AP to Diluent C in a light sensitive 15 or 50 mL conical tube.

(A) 1x Volume of Volume of Strep AP	(B) 1x Volume of Diluent C	(C) Number of Plate(s)	(D) Total Volume of Volume of Strep AP (A * C = D)	(E) Total Volume of Diluent C (B * C = E)
10 µL	10 mL			

2. When the incubation has finished, using the protocol "2. ELISpot_Day_2 _3_Tween" on the plate washer, wash the plate three times with 0.05% Tween-PBS, 200 µL/well.

Time of Wash: **Plate 1** ____________________

Plate 2 ____________________

Plate 3 ____________________

3. Flick the residual buffer out of the plate into a biohazard bin.
4. Add the tertiary solution to a reagent reservoir.
5. Using a multi-channel pipette, add 80 µL of tertiary solution to each well.
6. With the plate lying flat, incubate at room temperature for 30 minutes ± 5 minutes protected from light.

Incubation Start Time: **Plate 1** ____________________

Plate 2 ____________________

Plate 3 ____________________

Technical Review: ________________________ QC Review: ____________________________

Page:	Page 14 of 19
Study Number:	A-579-01
Title:	**A-579-01 Study Specific Method for IFN-γ Peptide ELISpot**
Batch ID:	
Performed By Initial and Date:	

4.6. Blue Developer Solution

*Prepare the Blue Developer Solution within 10 minutes of use and keep protected from direct light.

1. In a 15 or 50 mL light sensitive conical tube, add S1 to Diluent Blue. Mix well by inverting the tube.

(A) 1x Volume of Volume of S1	(B) 1x Volume of Diluent Blue	(C) Number of Plate(s)	(D) Total Volume of Volume of S1 (A * C = D)	(E) Total Volume of Diluent Blue (B * C = E)
160 µL	10 mL			

2. Add S2 to the same tube. Mix well by inverting the tube.

(A) 1x Volume of Volume of S2	(B) Number of Plate(s)	(C) Total Volume of Volume of S2 (A * B = C)
160 µL		

3. Add S3 to the same tube. Mix well by inverting the tube.

(A) 1x Volume of Volume of S3	(B) Number of Plate(s)	(C) Total Volume of Volume of S3 (A * B = C)
92 µL		

4. When the incubation has finished, using the protocol "3. ELISpot_Day_2 _2_Tween_2_dH2O" on the plate washer, wash the plate twice with 0.05% Tween-PBS, 200 µL/well, and then twice with distilled water, 200 µL/well.

Time of Wash: **Plate 1** ____________________

Plate 2 ____________________

Plate 3 ____________________

5. Flick the residual buffer out of the plate into a biohazard bin.

6. Add the blue developer solution to a reagent reservoir.

7. Using a multi-channel pipette, add 80 µL of blue developer solution to each well.

Technical Review: ________________________ QC Review: ____________________________

Page:	Page 15 of 19	
Study Number:	A-579-01	
Title:	**A-579-01 Study Specific Method for IFN-γ Peptide ELISpot**	
Batch ID:		
Performed By Initial and Date:		

8. With the plate lying flat, incubate at room temperature for 15 ± 1 minute protected from light.

Incubation Start Time: **Plate 1** ______________________

Plate 2 ______________________

Plate 3 ______________________

4.7. Stopping the Reaction

1. To stop the reaction, gently rinse the membrane with tap water. Remove the water by flicking the plate into the sink and repeat three times.

Time of Wash: **Plate 1** ______________________

Plate 2 ______________________

Plate 3 ______________________

2. Remove the protective underdrain and rinse the back of the plate with tap water. Make sure all pools of water have been flicked out.
3. Dry the plate, by leaving it face down on a paper towel in a drawer protected from light, leaving it in a running BSC with lights off, and/or placing it face down on a running BSCs front grate. Any combinations of drying methods may be used to dry the plate.

Drying Start Time: **Plate 1** ___________ **Drying Method** ______________________

Plate 2 ___________ ______________________

Plate 3 ___________ ______________________

*__Note__: Plate can be scanned once dry for up to 96 hours after stopping the reaction.

Technical Review: ________________________ QC Review: ____________________________

Page:	Page 16 of 19	
Study Number:	A-579-01	
Title:	**A-579-01 Study Specific Method for IFN-γ Peptide ELISpot**	
Batch ID:		
Performed By Initial and Date:		

5. Analyzing the ELISpot Plate(s)

5.1. Equipment:

Equipment (Description)	Equipment ID
S6 Universal M2 Analyzer	SPOT-01

* Rows may be added as needed

5.2. Plate ID's:

Plate ID's: **Plate 1** ______________________

Plate 2 ______________________

Plate 3 ______________________

Technical Review: ______________________ QC Review: ______________________

Page:	Page 17 of 19	amplifybio
Study Number:	A-579-01	
Title:	**A-579-01 Study Specific Method for IFN-γ Peptide ELISpot**	
Batch ID:		
Performed By Initial and Date:		

5.3. Scanning Plate(s)

*Verify the ImmunoQualify Reference plate has been run and passed according to SOP-00456 by checking the ImmunoSpot S6 Analyzer Usage Log (FORMTEMP-00687).

1. Select the Scan button from the Switchboard display. This will call up the ImmunoCapture™ scanning software.
2. Click "Eject" and place the plate in the plate holder. Click "Load".
3. In the "Image Acquisition Controls" box under "Configuration" select "ELISpot Clear Plates".
4. A preview of the wells will be shown, choose a positive peptide well (e.g., CEFx) and click "Find Focus".
5. Click "Start Scan" and when prompted name the plate as "BatchID.initials.PlateNumber" (plate number will be 01, 02, or 03). Set the saving location when prompted to: Data (D:)\Plates\(A-579-01). Click "Ok".
6. A plate map will open, select the wells to be scanned. Click "Ok".

Scan Start Time: **Plate 1** ________________

Plate 2 ________________

Plate 3 ________________

7. Once the plate is finished scanning click "Eject" and remove the plate, click "Load" to retract the plate holder. Repeat steps 2-5 if scanning additional plates or click "Exit" to leave the ImmunoCapture™ scanning software.
8. Store the plate with the lid on in the cabinet next to the analyzer.

Technical Review: ________________________ QC Review: ____________________________

Page:	Page 18 of 19	amplifybio
Study Number:	A-579-01	
Title:	**A-579-01 Study Specific Method for IFN-γ Peptide ELISpot**	
Batch ID:		
Performed By Initial and Date:		

5.4. Counting Plate(s)

1. Click on the "Count" button in the ImmunoSpot single-color switchboard.

2. Click on the "Smart Count" command.

3. Select "Load plate(s)".

4. Checkmark the box next to the plate that was scanned in the section "Scanning Plate(s)" and click "Ok".

5. Click "Adjust Parameters".

6. Change Spot Separation to 3.

7. Click "Gating".

8. Click "Collect Pos Wells", when prompted select known positive peptide wells (e.g., CMV, CEFx, etc.). Click "Finish" and allow the analyzer to detect spots. Click "Ok".

***Note**: Positive peptide wells should be from the species being tested, if available. Otherwise, CEFx from the reference sample can be used.

9. Click "Collect Neg Wells", when prompted select negative wells (e.g., DMSO control, negative peptide wells, etc.). Click "Finish" and allow the analyzer to detect spots. Click "Ok".

10. Click "Auto-adjust" and allow the analyzer to adjust gates. Click "Ok".

11. Click "Back" then click "Start AutoCount" and allow the analyzer to count spots. Click "Ok".

12. The analyzer will ask to set up a count for another plate. Click "Yes" to set up another count if counting more than one plate. Click "No" to leave the spot counting software.

Technical Review: ______________________ QC Review: ________________________

Page:	Page 19 of 19	amplifybio
Study Number:	A-579-01	
Title:	**A-579-01 Study Specific Method for IFN-γ Peptide ELISpot**	
Batch ID:		
Performed By Initial and Date:		

5.5. Saving Data to the Network

1. Open File Explorer on the PC.
2. Navigate to the Data (D:)\Plates\(A-579-01) and find the plate saved in the section "Scanning Plate(s)".
3. Drag that folder to the network \\ab-file1\AnalySci\Immunospot\A-579-01\ELISpot.

Comments:

Technical Review: ____________ QC Review: ____________

Document Approvals

Number: METHOD-00081 Version: 3.0

Approval Task<br>Verdict: Approve	Sapna Varia,<br>(svaria@amplify-bio.com)<br>Approval<br>11-Dec-2023 15:40:27 GMT+0000

Task: Retirement Approval Task<br>Verdict: Approve - Retire Document	Sapna Varia, Director of Analytical Operations<br>(svaria@amplify-bio.com)<br>Approval<br>30-Dec-2023 23:12:50 GMT+0000

Page:	Page 1 of 19
Study Number:	A-579-01
Title:	**A-579-01 Study Specific Method for IFN-γ Peptide ELISpot**
Batch ID:	
Performed By Initial and Date:	

1. Purpose

This study-specific method (SSM) describes the procedure for IFN-γ Peptide ELISpot per A-579-01 study requirements. The scientist's initials and dates on each page ("Performed by Initial and Date") signify that they have followed the instructions as written, verified that the materials and equipment remained the same as those listed in the tables, and all equipment has been properly calibrated. Any deviation to this method will be approved by the study director.

2. Media Preparation

2.1. Equipment:

Record the equipment utilized in the following table.

Equipment (Description)	Equipment ID
Biosafety Cabinet	
Water bath (37°C ± 2°C)	
Refrigerator 2-8°C	
Freezer (-30 to -15°C)	
Micropipette	
Micropipette	
Micropipette	
Micropipette	

Technical Review: ____________________ QC Review: ____________________

Page:	Page 2 of 19	amplifybio
Study Number:	A-579-01	
Title:	**A-579-01 Study Specific Method for IFN-γ Peptide ELISpot**	
Batch ID:		
Performed By Initial and Date:		

2.2. Materials:

Record the materials utilized in the following table.

Reagents/Materials	Manufacturer	Cat. No.	Lot No.	Expiration Date
RPMI	Corning	10-040-CV		
FBS	Cytiva	SH30071.03		
0.2 µM Vacuum Filter				

2.3. Preparing the Complete RPMI/10% FBS (R10) Media

Table 1. Volume of Complete R10 Media Needed

Calculate the Volume of Complete R10 Media Needed.

(A) **Number of Samples**	(B) **Volume Needed Per Sample (mL)**	(C) **Total Volume of Complete R10 Media Needed (mL)** (C)=(A) x (B) x (1.1)	(D) **Volume of FBS (mL)** (D) = (C) x (0.1)	(E) **Volume of RPMI Media Needed (mL)** (E) = (C) – (D)
	25			

Calculations Verified by: ______________________________
Initials/Date

1. Retrieve the necessary volume of frozen FBS and place in 37°C water bath to thaw and agitate periodically.
   * Do not hold FBS at 37°C for longer than necessary to thaw.
2. Add the FBS to the RPMI Media (see **Table 1** for volumes) and filter using a 0.2 µm vacuum filter.
3. Label bottle with RPMI/10% DMSO, expiration date (24 hours), initials, and storage requirements.
4. Store in refrigerator (2-8°C) for future use or place in water bath for immediate use.

Technical Review: ________________________ QC Review: ____________________________

Page:	Page 3 of 19	
Study Number:	A-579-01	
Title:	**A-579-01 Study Specific Method for IFN-γ Peptide ELISpot**	
Batch ID:		
Performed By Initial and Date:		

3. Preparing the ELISpot Plate(s)

3.1. Equipment:

Record the equipment utilized in the following table.

Equipment (Description)	Equipment ID
Biosafety Cabinet	
Centrifuge	
Micropipette	
Micropipette	
Micropipette	
Micropipette	
Multi-channel Pipette	
Liquid Nitrogen Storage (-195 to -135°C)	
Freezer (-85 to -60°C)	
Refrigerator 2-8°C	
Incubator (37°C ± 2°C, 5% ± 2% CO_2)	
Water bath (37°C ± 2°C)	

Technical Review: ______________________ QC Review: ________________________

Page:	Page 4 of 19
Study Number:	A-579-01
Title:	**A-579-01 Study Specific Method for IFN-γ Peptide ELISpot**
Batch ID:	
Performed By Initial and Date:	

3.2. Materials:

Record the materials utilized in the following table.

Reagents/Materials	Manufacturer	Cat. No.	Lot No.	Expiration Date
Single-color Enzymatic Hu IFN- γ Plate	Cellular Technology Limited	hIFNgp-2M/10		
Control Reagent: AAV9 Complete Pool Working Stock	AmplifyBio	AAV9-CompletePool-WS-4µg/mL		
Control Reagent: CyCMV UL55/UL83 Pool Working Stock	AmplifyBio	UL55-UL83-Pool-WS-2µg/mL		
Control Reagent: PHA-L Working Stock	AmplifyBio	PHA-L-WS-2ug/mL		
Control Reagent: 0.2% DMSO Working Stock	AmplifyBio	0.2%DMSO-WS		
Control Reagent: CEFx Ultra SuperStim Pool	AmplifyBio	CEFX-WS-2µg/mL		
1X PBS				

Technical Review: ______________________ QC Review: ______________________

Page:	Page 5 of 19
Study Number:	A-579-01
Title:	**A-579-01 Study Specific Method for IFN-γ Peptide ELISpot**
Batch ID:	
Performed By Initial and Date:	

3.3. Preparing the Cells

1. Prepare 10 mL aliquots of R10 media in 15 mL conical tubes, make one aliquot for each cryovial being thawed, and place into a 37°C water bath, along with remaining complete media.
2. Remove the cryovial(s) from the LN2 and place on dry ice immediately. Keep on dry ice until ready to thaw.
3. Transfer the cryovial(s) to a 37°C water bath and rapidly swirl the cryovial(s) at the surface of the water.
   *Do not allow water to touch the caps of the cryovial.
4. When a small bit of ice remains in the vial, remove the cryovial(s) from the water and clean and dry off the outside of the vial(s) with ethanol, and transfer to a BSC II.
5. Slowly (dropwise) dispense up to 1 mL of R10 media (using aliquoted media from the 15mL conical tube) into the cryovial and gently pipette 1 to 2 times slowly to mix.
6. Transfer the entire cell suspension slowly (dropwise) from the cryovial to the corresponding 15mL conical tube with the remaining aliquoted media.
7. Add an additional 1mL of R10 media (from the corresponding aliquot) to the cryovial to collect any residual cells remaining and pipette the entire suspension back to the 15mL conical tube.
8. Centrifuge the cell suspension for 5 minutes, 400 x g, room temperature.
9. Carefully aspirate the supernatant. Gently resuspend the cell pellet in 10 mL of fresh R10 media.
10. Centrifuge the cell suspension for 5 minutes, 400 x g, room temperature.
11. Carefully aspirate the supernatant. Gently resuspend the cell pellet in 0.5 mL of fresh R10 media.

Technical Review: ______________________ QC Review: ________________________

Page:	Page 6 of 19	
Study Number:	A-579-01	
Title:	**A-579-01 Study Specific Method for IFN-γ Peptide ELISpot**	
Batch ID:		
Performed By Initial and Date:		

12. For NHP samples, place the 15 mL conical tubes containing the cells into an incubator set at 37°C/5% CO_2 with caps vented for 3 hours ± 15 minutes.

Time Placed in Incubator: **Tubes for Plate 1** ____________________

Tubes for Plate 2 ____________________

Tubes for Plate 3 ____________________

3.4. Loading the Control Reagent(s)

1. Remove control reagents (2X) working stock solutions from the freezer and allow to thaw.
   Note: If making fresh control reagents follow FORMTEMP-00654 for specific instructions.
2. Label the side of the IFN-γ plate with "BatchID.initials.PlateNumber" (plate number will be 01, 02, or 03).

Plate ID: **Plate 1** ________________________________

Plate 2 ________________________________

Plate 3 ________________________________

3. Using a multichannel pipette, wash the plate with 150 µL/well of sterile PBS. Be sure to not touch the well membrane.
4. Flick the PBS out of the plate onto a sterile gauze pad or wypall and dispose of.
5. Add 100 µL of the prepared control reagent (2X) solution to the designated wells. Refer to the ELISpot IFN-γ Plate Map Form (FORMTEMP-00653) for control reagent locations.
6. Add 200 µL of complete R10 media to the designated "Blank" wells.
7. Once all control reagents and blanks have been added, place the plate into the incubator while cells are being counted.

Time Placed in Incubator: **Plate 1** ____________________

Plate 2 ____________________

Plate 3 ____________________

Technical Review: ______________________ QC Review: ________________________

Page:	Page 7 of 19	
Study Number:	A-579-01	
Title:	**A-579-01 Study Specific Method for IFN-γ Peptide ELISpot**	
Batch ID:		
Performed By Initial and Date:		

3.5. Plating the Cells

1. Remove the NHP sample tubes from the incubator.

Time out of Incubator:

Tubes for Plate 1 ______________________

Tubes for Plate 2 ______________________

Tubes for Plate 3 ______________________

2. Count the cells according to FORMTEMP-00663 S6 Universal M2 Analyzer: Cell Counting and record counts in **Table 2**.
3. Add the remaining volume of media needed to reach the desired concentration found in **Table 2**.
4. Remove the plate from the incubator.

Time Out of Incubator:

Plate 1 ______________________

Plate 2 ______________________

Plate 3 ______________________

5. Add 100 µL, dropwise around the well, of the desired concentration of cells (refer to **Table 2**) to each designated well within the plate. Refer to the ELISpot IFN-γ Plate Map Form (FORMTEMP-00653) for sample locations.
6. Place the finished plate onto the bottom shelf of an incubator set at 37°C/5% CO_2 for 24 ± 1 hours.

*Be very gentle when handling plates and be sure not to tilt when transferring to the incubator. Carefully open and close incubator doors. Do not stack plates.

Time Placed in Incubator:

Plate 1 ______________________

Plate 2 ______________________

Plate 3 ______________________

Technical Review: ________________________ QC Review: ____________________________

Page:	Page 8 of 19	amplifybio
Study Number:	A-579-01	
Title:	**A-579-01 Study Specific Method for IFN-γ Peptide ELISpot**	
Batch ID:		
Performed By Initial and Date:		

Table 2. Concentration

**Sample ID**						
**Total Cells at Freezing (x 10^6)**						
**Counting Resuspension Volume (mL)**	0.5	0.5	0.5	0.5	0.5	0.5
**% Viability[a]**						
**Viable cells/mL[a,b]**						
**Total Viable Cell yield[a,b]**						
**% Recovery[a]**						
**Desired Final Concentration (x 10^6 cells/mL)[c]**	2	2	2	2	2	2
**Total Final Resuspension Volume[a] (mL)**						
**Volume of R10 Media Added to Reach Final Resuspension Volume (mL)**						

a. All values provided are based on average of duplicate sample readings taken on the instrument (FORMTEMP-00663).
b. All data rounded to two decimal places.
c. Target final concentration is $2x10^6$ per mL ($2x10^5$ per well), well concentration can range from $5x10^4$ to $5x10^5$ per qualification. Study director approval is required for changes in well concentration.

Calculations Verified by: ____________________________
Initials/Date

Technical Review: ___________________________ QC Review: ________________________________

Page:	Page 9 of 19
Study Number:	A-579-01
Title:	**A-579-01 Study Specific Method for IFN-γ Peptide ELISpot**
Batch ID:	
Performed By Initial and Date:	

Table 2. Concentration Continued

**Sample ID**						
**Total Cells at Freezing (x 10^6)**						
**Counting Resuspension Volume (mL)**	0.5	0.5	0.5	0.5	0.5	0.5
**% Viability[a]**						
**Viable cells/mL[a,b]**						
**Total Viable Cell yield[a,b]**						
**% Recovery[a]**						
**Desired Final Concentration (x 10^6 cells/mL)[c]**	2	2	2	2	2	2
**Total Final Resuspension Volume[a] (mL)**						
**Volume of R10 Media Added to Reach Final Resuspension Volume (mL)**						

a. All values provided are based on average of duplicate sample readings taken on the instrument (FORMTEMP-00663).
b. All data rounded to two decimal places.
c. Target final concentration is $2x10^6$ per mL ($2x10^5$ per well), well concentration can range from $5x10^4$ to $5x10^5$ per qualification. Study director approval is required for changes in well concentration.

Calculations Verified by: ____________________
Initials/Date

Technical Review: ____________________ QC Review: ____________________

Page:	Page 10 of 19	
Study Number:	A-579-01	
Title:	**A-579-01 Study Specific Method for IFN-γ Peptide ELISpot**	
Batch ID:		
Performed By Initial and Date:		

4. Developing the ELISpot Plate(s)

4.1. Equipment:

Record the equipment utilized in the following table.

Equipment (Description)	Equipment ID
Biosafety Cabinet	
Micropipette	
Micropipette	
Micropipette	
Micropipette	
Multi-channel Pipette	
Refrigerator 2-8°C	
Microplate Washer	
Water Dispenser	

4.2. Materials:

Record the materials utilized in the following table.

Reagents/Materials	Manufacturer	Cat. No.	Lot No.	Expiration Date
Single-color Enzymatic Hu IFN- γ Kit (This includes Anti-human IFN-γ (Biotin) Detection Antibody, Diluent B, Diluent C, Diluent Blue, Strep-AP, S1, S2, and S3)	Cellular Technology Limited	hIFNgp-2M/10		
0.1 µm low protein binding filter				
1X PBS				

Technical Review: ______________________ QC Review: ______________________

Page:	Page 11 of 19	
Study Number:	A-579-01	
Title:	**A-579-01 Study Specific Method for IFN-γ Peptide ELISpot**	
Batch ID:		
Performed By Initial and Date:		

4.3. Microplate Washer Buffers:

*Verify the plate washer has been primed according to SOP-00459 by checking the Agilent Plate Washer Use Log (FORMTEMP-00686).

Reagents/Materials	Cat. No.	Lot No.	Batch ID	Expiration Date
1X PBS				
0.05% Tween-PBS				
Distilled Water				

Technical Review: ________________________ QC Review: ____________________________

Page:	Page 12 of 19	
Study Number:	A-579-01	
Title:	**A-579-01 Study Specific Method for IFN-γ Peptide ELISpot**	
Batch ID:		
Performed By Initial and Date:		

4.4. Detection Solution

1. In a 15 mL or 50 mL conical tube, add the Anti-human IFN- γ (Biotin) Detection Antibody to Diluent B.

(A) 1x Volume of Anti-human IFN-γ (Biotin) Detection Antibody	(B) 1x Volume of Diluent B	(C) Number of Plate(s)	(D) Total Volume of Anti-human IFN-γ (Biotin) Detection Antibody (A * C = D)	(E) Total Volume of Diluent B (B * C = E)
40 µL	10 mL			

2. Transfer the solution to a syringe with a 0.1 µm low protein binding filter attached and filter into a new 15 mL or 50 mL conical tube.

3. Remove the plate from the incubator and flick all liquid out of the plate into an absorbent pad in either the BSC, or a biohazard bin. Dispose of the pad into biohazard.

4. Using a multi-channel pipette, add 200 µL of PBS to each well to start the wash process.

Time of Wash: **Plate 1** ____________________

Plate 2 ____________________

Plate 3 ____________________

5. Using the protocol "1. ELISpot_Day_2_1_PBS_2_Tween" on the plate washer, wash the plate once with PBS, 200 µL/well, and then twice with 0.05% Tween-PBS, 200 µL/well.

6. Flick the residual buffer out of the plate into a biohazard bin.

7. Add the detection solution to a reagent reservoir.

8. Using a multi-channel pipette, add 80 µL of detection solution to each well.

9. With the plate lying flat, incubate for two hours ± 15 minutes at room temperature.

Incubation Start Time: **Plate 1** ____________________

Plate 2 ____________________

Plate 3 ____________________

Technical Review: ______________________ QC Review: ___________________________

Page:	Page 13 of 19	amplifybio
Study Number:	A-579-01	
Title:	**A-579-01 Study Specific Method for IFN-γ Peptide ELISpot**	
Batch ID:		
Performed By Initial and Date:		

4.5. Tertiary Solution

1. Dilute Strep AP at a 1:1000 factor in Diluent C. Add Strep-AP to Diluent C in a light sensitive 15 or 50 mL conical tube.

(A) 1x Volume of Volume of Strep AP	(B) 1x Volume of Diluent C	(C) Number of Plate(s)	(D) Total Volume of Volume of Strep AP (A * C = D)	(E) Total Volume of Diluent C (B * C = E)
10 µL	10 mL			

2. When the incubation has finished, using the protocol "2. ELISpot_Day_2 _3_Tween" on the plate washer, wash the plate three times with 0.05% Tween-PBS, 200 µL/well.

Time of Wash: **Plate 1** ____________________

Plate 2 ____________________

Plate 3 ____________________

3. Flick the residual buffer out of the plate into a biohazard bin.
4. Add the tertiary solution to a reagent reservoir.
5. Using a multi-channel pipette, add 80 µL of tertiary solution to each well.
6. With the plate lying flat, incubate at room temperature for 30 minutes ± 5 minutes protected from light.

Incubation Start Time: **Plate 1** ____________________

Plate 2 ____________________

Plate 3 ____________________

Technical Review: ________________________ QC Review: ____________________________

Page:	Page 14 of 19	amplifybio
Study Number:	A-579-01	
Title:	**A-579-01 Study Specific Method for IFN-γ Peptide ELISpot**	
Batch ID:		
Performed By Initial and Date:		

4.6. Blue Developer Solution

*Prepare the Blue Developer Solution within 10 minutes of use and keep protected from direct light.

1. In a 15 or 50 mL light sensitive conical tube, add S1 to Diluent Blue. Mix well by inverting the tube.

(A) 1x Volume of Volume of S1	(B) 1x Volume of Diluent Blue	(C) Number of Plate(s)	(D) Total Volume of Volume of S1 (A * C = D)	(E) Total Volume of Diluent Blue (B * C = E)
160 µL	10 mL			

2. Add S2 to the same tube. Mix well by inverting the tube.

(A) 1x Volume of Volume of S2	(B) Number of Plate(s)	(C) Total Volume of Volume of S2 (A * B = C)
160 µL		

3. Add S3 to the same tube. Mix well by inverting the tube.

(A) 1x Volume of Volume of S3	(B) Number of Plate(s)	(C) Total Volume of Volume of S3 (A * B = C)
92 µL		

4. When the incubation has finished, using the protocol "3. ELISpot_Day_2 _2_Tween_2_dH2O" on the plate washer, wash the plate twice with 0.05% Tween-PBS, 200 µL/well, and then twice with distilled water, 200 µL/well.

Time of Wash: **Plate 1** ____________________

Plate 2 ____________________

Plate 3 ____________________

5. Flick the residual buffer out of the plate into a biohazard bin.

6. Add the blue developer solution to a reagent reservoir.

7. Using a multi-channel pipette, add 80 µL of blue developer solution to each well.

Technical Review: ______________________ QC Review: __________________________

Page:	Page 15 of 19
Study Number:	A-579-01
Title:	**A-579-01 Study Specific Method for IFN-γ Peptide ELISpot**
Batch ID:	
Performed By Initial and Date:	

8. With the plate lying flat, incubate at room temperature for 15 ± 1 minute protected from light.

Incubation Start Time: **Plate 1** ____________________

Plate 2 ____________________

Plate 3 ____________________

4.7. Stopping the Reaction

1. To stop the reaction, gently rinse the membrane with tap water. Remove the water by flicking the plate into the sink and repeat three times.

Time of Wash: **Plate 1** ____________________

Plate 2 ____________________

Plate 3 ____________________

2. Remove the protective underdrain and rinse the back of the plate with tap water. Make sure all pools of water have been flicked out.
3. Dry the plate, by leaving it face down on a paper towel in a drawer protected from light, leaving it in a running BSC with lights off, and/or placing it face down on a running BSCs front grate. Any combinations of drying methods may be used to dry the plate.

Drying Start Time: **Plate 1** __________ **Drying Method** ____________________

Plate 2 __________ ____________________

Plate 3 __________ ____________________

***Note**: Plate can be scanned once dry for up to 96 hours after stopping the reaction.

Technical Review: ____________________ QC Review: ____________________

Page:	Page 16 of 19	
Study Number:	A-579-01	
Title:	**A-579-01 Study Specific Method for IFN-γ Peptide ELISpot**	
Batch ID:		
Performed By Initial and Date:		

5. Analyzing the ELISpot Plate(s)

5.1. Equipment:

Equipment (Description)	Equipment ID
S6 Universal M2 Analyzer	SPOT-01

* Rows may be added as needed

5.2. Plate ID's:

Plate ID's: **Plate 1** ______________________

Plate 2 ______________________

Plate 3 ______________________

Technical Review: ______________________ QC Review: ______________________

Page:	Page 17 of 19	
Study Number:	A-579-01	
Title:	**A-579-01 Study Specific Method for IFN-γ Peptide ELISpot**	
Batch ID:		
Performed By Initial and Date:		

5.3. Scanning Plate(s)

*Verify the ImmunoQualify Reference plate has been run and passed according to SOP-00456 by checking the ImmunoSpot S6 Analyzer Usage Log (FORMTEMP-00687).

1. Select the Scan button from the Switchboard display. This will call up the ImmunoCapture™ scanning software.
2. Click "Eject" and place the plate in the plate holder. Click "Load".
3. In the "Image Acquisition Controls" box under "Configuration" select "ELISpot Clear Plates".
4. A preview of the wells will be shown, choose a positive peptide well (e.g., CEFx) and click "Find Focus".
5. Click "Start Scan" and when prompted name the plate as "BatchID.initials.PlateNumber" (plate number will be 01, 02, or 03). Set the saving location when prompted to: Data (D:)\Plates\(A-579-01). Click "Ok".
6. A plate map will open, select the wells to be scanned. Click "Ok".

Scan Start Time: **Plate 1** ________________

Plate 2 ________________

Plate 3 ________________

7. Once the plate is finished scanning click "Eject" and remove the plate, click "Load" to retract the plate holder. Repeat steps 2-5 if scanning additional plates or click "Exit" to leave the ImmunoCapture™ scanning software.
8. Store the plate with the lid on in the cabinet next to the analyzer.

Technical Review: ________________________ QC Review: ____________________________

Page:	Page 18 of 19	
Study Number:	A-579-01	
Title:	**A-579-01 Study Specific Method for IFN-γ Peptide ELISpot**	
Batch ID:		
Performed By Initial and Date:		

5.4. Counting Plate(s)

1. Click on the “Count” button in the ImmunoSpot single-color switchboard.
2. Click on the “Smart Count” command.
3. Select “Load plate(s)”.
4. Checkmark the box next to the plate that was scanned in the section “Scanning Plate(s)” and click “Ok”.
5. Click “Adjust Parameters”.
6. Change Spot Separation to 3.
7. Click “Gating”.
8. Click “Collect Pos Wells”, when prompted select known positive peptide wells (e.g., CMV, CEFx, etc.). Click “Finish” and allow the analyzer to detect spots. Click “Ok”.

   ***Note**: Positive peptide wells should be from the species being tested, if available. Otherwise, CEFx from the reference sample can be used.
9. Click “Collect Neg Wells”, when prompted select negative wells (e.g., DMSO control, negative peptide wells, etc.). Click “Finish” and allow the analyzer to detect spots. Click “Ok”.
10. Click “Auto-adjust” and allow the analyzer to adjust gates. Click “Ok”.
11. Click “Back” then click “Start AutoCount” and allow the analyzer to count spots. Click “Ok”.
12. The analyzer will ask to set up a count for another plate. Click “Yes” to set up another count if counting more than one plate. Click “No” to leave the spot counting software.

Technical Review: ________________________ QC Review: ____________________________

Page:	Page 19 of 19	
Study Number:	A-579-01	
Title:	**A-579-01 Study Specific Method for IFN-γ Peptide ELISpot**	
Batch ID:		
Performed By Initial and Date:		

5.5. Saving Data to the Network

1. Open File Explorer on the PC.
2. Navigate to the Data (D:)\Plates\(A-579-01) and find the plate saved in the section "Scanning Plate(s)".
3. Drag that folder to the network \\ab-file1\AnalySci\Immunospot\A-579-01\ELISpot.

Comments:

Technical Review: ______________ QC Review: ______________

Document Approvals
Number: METHOD-00081 Version: 2.0

Approval Task<br>Verdict: Approve	Sapna Varia,<br>(svaria@amplify-bio.com)<br>Approval<br>03-Nov-2023 14:27:14 GMT+0000

Page:	Page 1 of 19	
Study Number:	A-579-01	
Title:	**A-579-01 Study Specific Method for IFN-γ Peptide ELISpot**	
Batch ID:		
Performed By Initial and Date:		

1. Purpose

This study-specific method (SSM) describes the procedure for IFN-γ Peptide ELISpot per A-579-01 study requirements. The scientist's initials and dates on each page ("Performed by Initial and Date") signify that they have followed the instructions as written, verified that the materials and equipment remained the same as those listed in the tables, and all equipment has been properly calibrated. Any deviation to this method will be approved by the study director.

2. Media Preparation

2.1. Equipment:

Record the equipment utilized in the following table.

Equipment (Description)	Equipment ID
Biosafety Cabinet	
Water bath (37°C ± 2°C)	
Refrigerator 2-8°C	
Freezer (-30 to -15°C)	
Micropipette	
Micropipette	
Micropipette	
Micropipette	

Technical Review: ____________________________ QC Review: ____________________________

Page:	Page 2 of 19	amplifybio
Study Number:	A-579-01	
Title:	**A-579-01 Study Specific Method for IFN-γ Peptide ELISpot**	
Batch ID:		
Performed By Initial and Date:		

2.2. Materials:

Record the materials utilized in the following table.

Reagents/Materials	Manufacturer	Cat. No.	Lot No.	Expiration Date
RPMI	Corning	10-040-CV		
FBS	Cytiva	SH30071.03		
0.2 µM Vacuum Filter				

2.3. Preparing the Complete RPMI/10% FBS (R10) Media

Table 1. Volume of Complete R10 Media Needed

Calculate the Volume of Complete R10 Media Needed.

(A) **Number of Samples**	(B) **Volume Needed Per Sample (mL)**	(C) **Total Volume of Complete R10 Media Needed (mL)** (C)=(A) x (B) x (1.1)	(D) **Volume of FBS (mL)** (D) = (C) x (0.1)	(E) **Volume of RPMI Media Needed (mL)** (E) = (C) – (D)
	25			

Calculations Verified by: ______________________________
Initials/Date

1. Retrieve the necessary volume of frozen FBS and place in 37°C water bath to thaw and agitate periodically.
   * Do not hold FBS at 37°C for longer than necessary to thaw.
2. Add the FBS to the RPMI Media (see **Table 1** for volumes) and filter using a 0.2 µm vacuum filter.
3. Label bottle with RPMI/10% DMSO, expiration date (24 hours), initials, and storage requirements.
4. Store in refrigerator (2-8°C) for future use or place in water bath for immediate use.

Technical Review: ________________________ QC Review: ____________________________

Page:	Page 3 of 19
Study Number:	A-579-01
Title:	**A-579-01 Study Specific Method for IFN-γ Peptide ELISpot**
Batch ID:	
Performed By Initial and Date:	

3. Preparing the ELISpot Plate(s)

3.1. Equipment:

Record the equipment utilized in the following table.

Equipment (Description)	Equipment ID
Biosafety Cabinet	
Centrifuge	
Micropipette	
Micropipette	
Micropipette	
Micropipette	
Multi-channel Pipette	
Liquid Nitrogen Storage (-195 to -135°C)	
Freezer (-85 to -60°C)	
Refrigerator 2-8°C	
Incubator (37°C ± 2°C, 5% ± 2% CO_2)	
Water bath (37°C ± 2°C)	

Technical Review: ____________________ QC Review: ____________________

Page:	Page 4 of 19	amplifybio
Study Number:	A-579-01	
Title:	**A-579-01 Study Specific Method for IFN-γ Peptide ELISpot**	
Batch ID:		
Performed By Initial and Date:		

3.2. Materials:

Record the materials utilized in the following table.

Reagents/Materials	Manufacturer	Cat. No.	Lot No.	Expiration Date
Single-color Enzymatic Hu IFN- γ Plate	Cellular Technology Limited	hIFNgp-2M/10		
Control Reagent: AAV9 Complete Pool Working Stock	AmplifyBio	AAV9-CompletePool-WS-4µg/mL		
Control Reagent: CyCMV UL55/UL83 Pool Working Stock	AmplifyBio	UL55-UL83-Pool-WS-2µg/mL		
Control Reagent: PHA-L Working Stock	AmplifyBio	PHA-L-WS-2ug/mL		
Control Reagent: 0.2% DMSO Working Stock	AmplifyBio	0.2%DMSO-WS		
Control Reagent: CEFx Ultra SuperStim Pool	AmplifyBio	CEFX-WS-2µg/mL		
1X PBS				

Technical Review: ____________________ QC Review: ____________________

Page:	Page 5 of 19	
Study Number:	A-579-01	
Title:	**A-579-01 Study Specific Method for IFN-γ Peptide ELISpot**	
Batch ID:		
Performed By Initial and Date:		

3.3. Preparing the Cells

1. Prepare 10 mL aliquots of R10 media in 15 mL conical tubes, make one aliquot for each cryovial being thawed, and place into a 37°C water bath, along with remaining complete media.
2. Remove the cryovial(s) from the LN2 and place on dry ice immediately. Keep on dry ice until ready to thaw.
3. Transfer the cryovial(s) to a 37°C water bath and rapidly swirl the cryovial(s) at the surface of the water.
   *Do not allow water to touch the caps of the cryovial.
4. When a small bit of ice remains in the vial, remove the cryovial(s) from the water and clean and dry off the outside of the vial(s) with ethanol, and transfer to a BSC II.
5. Slowly (dropwise) dispense up to 1 mL of R10 media (using aliquoted media from the 15mL conical tube) into the cryovial and gently pipette 1 to 2 times slowly to mix.
6. Transfer the entire cell suspension slowly (dropwise) from the cryovial to the corresponding 15mL conical tube with the remaining aliquoted media.
7. Add an additional 1mL of R10 media (from the corresponding aliquot) to the cryovial to collect any residual cells remaining and pipette the entire suspension back to the 15mL conical tube.
8. Centrifuge the cell suspension for 5 minutes, 400 x g, room temperature.
9. Carefully aspirate the supernatant. Gently resuspend the cell pellet in 10 mL of fresh R10 media.
10. Centrifuge the cell suspension for 5 minutes, 400 x g, room temperature.
11. Carefully aspirate the supernatant. Gently resuspend the cell pellet in 0.5 mL of fresh R10 media.

Technical Review: ______________________ QC Review: ______________________

Page:	Page 6 of 19	
Study Number:	A-579-01	
Title:	**A-579-01 Study Specific Method for IFN-γ Peptide ELISpot**	
Batch ID:		
Performed By Initial and Date:		

12. For NHP samples, place the 15 mL conical tubes containing the cells into an incubator set at 37°C/5% CO_2 with caps vented for 3 hours ± 15 minutes.

Time Placed in Incubator: **Tubes for Plate 1** ____________________

Tubes for Plate 2 ____________________

Tubes for Plate 3 ____________________

3.4. Loading the Control Reagent(s)

1. Remove control reagents (2X) working stock solutions from the freezer and allow to thaw.
Note: If making fresh control reagents follow FORMTEMP-00654 for specific instructions.
2. Label the side of the IFN-γ plate with "BatchID.initials.PlateNumber" (plate number will be 01, 02, or 03).

Plate ID: **Plate 1** ________________________________

Plate 2 ________________________________

Plate 3 ________________________________

3. Using a multichannel pipette, wash the plate with 150 µL/well of sterile PBS. Be sure to not touch the well membrane.
4. Flick the PBS out of the plate onto a sterile gauze pad or wypall and dispose of.
5. Add 100 µL of the prepared control reagent (2X) solution to the designated wells. Refer to the ELISpot IFN-γ Plate Map Form (FORMTEMP-00653) for control reagent locations.
6. Add 200 µL of complete R10 media to the designated "Blank" wells.
7. Once all control reagents and blanks have been added, place the plate into the incubator while cells are being counted.

Time Placed in Incubator: **Plate 1** ____________________

Plate 2 ____________________

Plate 3 ____________________

Technical Review: ______________________ QC Review: ________________________

Page:	Page 7 of 19	
Study Number:	A-579-01	
Title:	**A-579-01 Study Specific Method for IFN-γ Peptide ELISpot**	
Batch ID:		
Performed By Initial and Date:		

3.5. Plating the Cells

1. Remove the NHP sample tubes from the incubator.

Time out of Incubator:

Tubes for Plate 1 ____________________

Tubes for Plate 2 ____________________

Tubes for Plate 3 ____________________

2. Count the cells according to FORMTEMP-00663 S6 Universal M2 Analyzer: Cell Counting and record counts in **Table 2**.
3. Add the remaining volume of media needed to reach the desired concentration found in **Table 2**.
4. Remove the plate from the incubator.

Time Out of Incubator:

Plate 1 ____________________

Plate 2 ____________________

Plate 3 ____________________

5. Add 100 µL, dropwise around the well, of the desired concentration of cells (refer to **Table 2**) to each designated well within the plate. Refer to the ELISpot IFN-γ Plate Map Form (FORMTEMP-00653) for sample locations.
6. Place the finished plate onto the bottom shelf of an incubator set at 37°C/5% CO_2 for 24 ± 1 hours.

*Be very gentle when handling plates and be sure not to tilt when transferring to the incubator. Carefully open and close incubator doors. Do not stack plates.

Time Placed in Incubator:

Plate 1 ____________________

Plate 2 ____________________

Plate 3 ____________________

Technical Review: ____________________ QC Review: ____________________

Page:	Page 8 of 19	amplifybio
Study Number:	A-579-01	
Title:	**A-579-01 Study Specific Method for IFN-γ Peptide ELISpot**	
Batch ID:		
Performed By Initial and Date:		

Table 2. Concentration

**Sample ID**						
**Total Cells at Freezing (x 10^6)**						
**Counting Resuspension Volume (mL)**	0.5	0.5	0.5	0.5	0.5	0.5
**% Viability[a]**						
**Viable cells/mL[a,b]**						
**Total Viable Cell yield[a,b]**						
**% Recovery[a]**						
**Desired Final Concentration (x 10^6 cells/mL)[c]**	2	2	2	2	2	2
**Total Final Resuspension Volume[a] (mL)**						
**Volume of R10 Media Added to Reach Final Resuspension Volume (mL)**						

a. All values provided are based on average of duplicate sample readings taken on the instrument (FORMTEMP-00663).
b. All data rounded to two decimal places.
c. Target final concentration is $2x10^6$ per mL ($2x10^5$ per well), well concentration can range from $5x10^4$ to $5x10^5$ per qualification. Study director approval is required for changes in well concentration.

Calculations Verified by: ____________________________
Initials/Date

Technical Review: ___________________________ QC Review: ________________________________

Page:	Page 9 of 19	amplifybio
Study Number:	A-579-01	
Title:	**A-579-01 Study Specific Method for IFN-γ Peptide ELISpot**	
Batch ID:		
Performed By Initial and Date:		

Table 2. Concentration Continued

**Sample ID**						
**Total Cells at Freezing (x 10^6)**						
**Counting Resuspension Volume (mL)**	0.5	0.5	0.5	0.5	0.5	0.5
**% Viability[a]**						
**Viable cells/mL[a,b]**						
**Total Viable Cell yield[a,b]**						
**% Recovery[a]**						
**Desired Final Concentration (x 10^6 cells/mL)[c]**	2	2	2	2	2	2
**Total Final Resuspension Volume[a] (mL)**						
**Volume of R10 Media Added to Reach Final Resuspension Volume (mL)**						

d. All values provided are based on average of duplicate sample readings taken on the instrument (FORMTEMP-00663).
e. All data rounded to two decimal places.
f. Target final concentration is $2x10^6$ per mL ($2x10^5$ per well), well concentration can range from $5x10^4$ to $5x10^5$ per qualification. Study director approval is required for changes in well concentration.

Calculations Verified by: ______________________
Initials/Date

Technical Review: ______________________ QC Review: ______________________

Page:	Page 10 of 19	amplifybio
Study Number:	A-579-01	
Title:	**A-579-01 Study Specific Method for IFN-γ Peptide ELISpot**	
Batch ID:		
Performed By Initial and Date:		

4. Developing the ELISpot Plate(s)

4.1. Equipment:

Record the equipment utilized in the following table.

Equipment (Description)	Equipment ID
Biosafety Cabinet	
Micropipette	
Micropipette	
Micropipette	
Micropipette	
Multi-channel Pipette	
Refrigerator 2-8°C	
Microplate Washer	
Water Dispenser	

4.2. Materials:

Record the materials utilized in the following table.

Reagents/Materials	Manufacturer	Cat. No.	Lot No.	Expiration Date
Single-color Enzymatic Hu IFN- γ Kit (This includes Anti-human IFN-γ (Biotin) Detection Antibody, Diluent B, Diluent C, Diluent Blue, Strep-AP, S1, S2, and S3)	Cellular Technology Limited	hIFNgp-2M/10		
0.1 µm low protein binding filter				
1X PBS				

Technical Review: ______________________ QC Review: ______________________

Page:	Page 11 of 19
Study Number:	A-579-01
Title:	**A-579-01 Study Specific Method for IFN-γ Peptide ELISpot**
Batch ID:	
Performed By Initial and Date:	

4.3. Microplate Washer Buffers:

*Verify the plate washer has been primed according to SOP-00459 by checking the Agilent Plate Washer Use Log (FORMTEMP-00686).

Reagents/Materials	Cat. No.	Lot No.	Batch ID	Expiration Date
1X PBS				
0.05% Tween-PBS				
Distilled Water				

Technical Review: ______________________ QC Review: ___________________________

Page:	Page 12 of 19	
Study Number:	A-579-01	
Title:	**A-579-01 Study Specific Method for IFN-γ Peptide ELISpot**	
Batch ID:		
Performed By Initial and Date:		

4.4. Detection Solution

1. In a 15 mL or 50 mL conical tube, add the Anti-human IFN- γ (Biotin) Detection Antibody to Diluent B.

(A) 1x Volume of Anti-human IFN-γ (Biotin) Detection Antibody	(B) 1x Volume of Diluent B	(C) Number of Plate(s)	(D) Total Volume of Anti-human IFN-γ (Biotin) Detection Antibody (A * C = D)	(E) Total Volume of Diluent B (B * C = E)
40 µL	10 mL			

2. Transfer the solution to a syringe with a 0.1 µm low protein binding filter attached and filter into a new 15 mL or 50 mL conical tube.
3. Remove the plate from the incubator and flick all liquid out of the plate into an absorbent pad in either the BSC, or a biohazard bin. Dispose of the pad into biohazard.
4. Using a multi-channel pipette, add 200 µL of PBS to each well to start the wash process.

Time of Wash: **Plate 1** ____________________

Plate 2 ____________________

Plate 3 ____________________

5. Using the protocol "1. ELISpot_Day_2_1_PBS_2_Tween" on the plate washer, wash the plate once with PBS, 200 µL/well, and then twice with 0.05% Tween-PBS, 200 µL/well.
6. Flick the residual buffer out of the plate into a biohazard bin.
7. Add the detection solution to a reagent reservoir.
8. Using a multi-channel pipette, add 80 µL of detection solution to each well.
9. With the plate lying flat, incubate for two hours ± 15 minutes at room temperature.

Incubation Start Time: **Plate 1** ____________________

Plate 2 ____________________

Plate 3 ____________________

Technical Review: ______________________ QC Review: __________________________

Page:	Page 13 of 19	
Study Number:	A-579-01	
Title:	**A-579-01 Study Specific Method for IFN-γ Peptide ELISpot**	
Batch ID:		
Performed By Initial and Date:		

4.5. Tertiary Solution

1. Dilute Strep AP at a 1:1000 factor in Diluent C. Add Strep-AP to Diluent C in a light sensitive 15 or 50 mL conical tube.

(A) 1x Volume of Volume of Strep AP	(B) 1x Volume of Diluent C	(C) Number of Plate(s)	(D) Total Volume of Volume of Strep AP (A * C = D)	(E) Total Volume of Diluent C (B * C = E)
10 µL	10 mL			

2. When the incubation has finished, using the protocol "2. ELISpot_Day_2 _3_Tween" on the plate washer, wash the plate three times with 0.05% Tween-PBS, 200 µL/well.

Time of Wash: **Plate 1** ____________________

Plate 2 ____________________

Plate 3 ____________________

3. Flick the residual buffer out of the plate into a biohazard bin.

4. Add the tertiary solution to a reagent reservoir.

5. Using a multi-channel pipette, add 80 µL of tertiary solution to each well.

6. With the plate lying flat, incubate at room temperature for 30 minutes ± 5 minutes protected from light.

Incubation Start Time: **Plate 1** ____________________

Plate 2 ____________________

Plate 3 ____________________

Technical Review: ________________________ QC Review: ____________________________

Page:	Page 14 of 19	amplifybio
Study Number:	A-579-01	
Title:	**A-579-01 Study Specific Method for IFN-γ Peptide ELISpot**	
Batch ID:		
Performed By Initial and Date:		

4.6. Blue Developer Solution

*Prepare the Blue Developer Solution within 10 minutes of use and keep protected from direct light.

1. In a 15 or 50 mL light sensitive conical tube, add S1 to Diluent Blue. Mix well by inverting the tube.

(A) 1x Volume of Volume of S1	(B) 1x Volume of Diluent Blue	(C) Number of Plate(s)	(D) Total Volume of Volume of S1 (A * C = D)	(E) Total Volume of Diluent Blue (B * C = E)
160 µL	10 mL			

2. Add S2 to the same tube. Mix well by inverting the tube.

(A) 1x Volume of Volume of S2	(B) Number of Plate(s)	(C) Total Volume of Volume of S2 (A * B = C)
160 µL		

3. Add S3 to the same tube. Mix well by inverting the tube.

(A) 1x Volume of Volume of S3	(B) Number of Plate(s)	(C) Total Volume of Volume of S3 (A * B = C)
92 µL		

4. When the incubation has finished, using the protocol "3. ELISpot_Day_2 _2_Tween_2_dH2O" on the plate washer, wash the plate twice with 0.05% Tween-PBS, 200 µL/well, and then twice with distilled water, 200 µL/well.

Time of Wash: **Plate 1** ____________________

Plate 2 ____________________

Plate 3 ____________________

5. Flick the residual buffer out of the plate into a biohazard bin.

6. Add the blue developer solution to a reagent reservoir.

7. Using a multi-channel pipette, add 80 µL of blue developer solution to each well.

Technical Review: ______________________ QC Review: __________________________

Page:	Page 15 of 19	
Study Number:	A-579-01	
Title:	**A-579-01 Study Specific Method for IFN-γ Peptide ELISpot**	
Batch ID:		
Performed By Initial and Date:		

8. With the plate lying flat, incubate at room temperature for 15 ± 1 minute protected from light.

Incubation Start Time: **Plate 1** ____________________

Plate 2 ____________________

Plate 3 ____________________

4.7. Stopping the Reaction

1. To stop the reaction, gently rinse the membrane with tap water. Remove the water by flicking the plate into the sink and repeat three times.

Time of Wash: **Plate 1** ____________________

Plate 2 ____________________

Plate 3 ____________________

2. Remove the protective underdrain and rinse the back of the plate with tap water. Make sure all pools of water have been flicked out.
3. Dry the plate, by leaving it face down on a paper towel in a drawer protected from light, leaving it in a running BSC with lights off, and/or placing it face down on a running BSCs front grate. Any combinations of drying methods may be used to dry the plate.

Drying Start Time: **Plate 1** __________ **Drying Method** ____________________

Plate 2 __________ ____________________

Plate 3 __________ ____________________

***Note**: Plate can be scanned once dry for up to 96 hours after stopping the reaction.

Technical Review: ____________________ QC Review: ____________________

Page:	Page 16 of 19	
Study Number:	A-579-01	
Title:	**A-579-01 Study Specific Method for IFN-γ Peptide ELISpot**	
Batch ID:		
Performed By Initial and Date:		

5. Analyzing the ELISpot Plate(s)

5.1. Equipment:

Equipment (Description)	Equipment ID
S6 Universal M2 Analyzer	SPOT-01

* Rows may be added as needed

5.2. Plate ID's:

Plate ID's: **Plate 1** ______________________

Plate 2 ______________________

Plate 3 ______________________

Technical Review: ______________________ QC Review: ______________________

Page:	Page 17 of 19	
Study Number:	A-579-01	
Title:	**A-579-01 Study Specific Method for IFN-γ Peptide ELISpot**	
Batch ID:		
Performed By Initial and Date:		

5.3. Scanning Plate(s)

*Verify the ImmunoQualify Reference plate has been run and passed according to SOP-00456 by checking the ImmunoSpot S6 Analyzer Usage Log (FORMTEMP-00687).

1. Select the Scan button from the Switchboard display. This will call up the ImmunoCapture™ scanning software.
2. Click "Eject" and place the plate in the plate holder. Click "Load".
3. In the "Image Acquisition Controls" box under "Configuration" select "ELISpot Clear Plates".
4. A preview of the wells will be shown, choose a positive peptide well (e.g., CEFx) and click "Find Focus".
5. Click "Start Scan" and when prompted name the plate as "BatchID.initials.PlateNumber" (plate number will be 01, 02, or 03). Set the saving location when prompted to: Data (D:)\Plates\(A-579-01). Click "Ok".
6. A plate map will open, select the wells to be scanned. Click "Ok".

Scan Start Time: **Plate 1** ________________

Plate 2 ________________

Plate 3 ________________

7. Once the plate is finished scanning click "Eject" and remove the plate, click "Load" to retract the plate holder. Repeat steps 2-5 if scanning additional plates or click "Exit" to leave the ImmunoCapture™ scanning software.
8. Store the plate with the lid on in the cabinet next to the analyzer.

Technical Review: ________________________ QC Review: ____________________________

Page:	Page 18 of 19	
Study Number:	A-579-01	
Title:	**A-579-01 Study Specific Method for IFN-γ Peptide ELISpot**	
Batch ID:		
Performed By Initial and Date:		

5.4. Counting Plate(s)

1. Click on the "Count" button in the ImmunoSpot single-color switchboard.
2. Click on the "Smart Count" command.
3. Select "Load plate(s)".
4. Checkmark the box next to the plate that was scanned in the section "Scanning Plate(s)" and click "Ok".
5. Click "Adjust Parameters".
6. Change Spot Separation to 3.
7. Click "Gating".
8. Click "Collect Pos Wells", when prompted select known positive peptide wells (e.g., CMV, CEFx, etc.). Click "Finish" and allow the analyzer to detect spots. Click "Ok".

   ***Note**: Positive peptide wells should be from the species being tested, if available. Otherwise, CEFx from the reference sample can be used.
9. Click "Collect Neg Wells", when prompted select negative wells (e.g., DMSO control, negative peptide wells, etc.). Click "Finish" and allow the analyzer to detect spots. Click "Ok".
10. Click "Auto-adjust" and allow the analyzer to adjust gates. Click "Ok".
11. Click "Back" then click "Start AutoCount" and allow the analyzer to count spots. Click "Ok".
12. The analyzer will ask to set up a count for another plate. Click "Yes" to set up another count if counting more than one plate. Click "No" to leave the spot counting software.

Technical Review: ________________________ QC Review: ____________________________

Page:	Page 19 of 19	
Study Number:	A-579-01	
Title:	**A-579-01 Study Specific Method for IFN-γ Peptide ELISpot**	
Batch ID:		
Performed By Initial and Date:		

5.5. Saving Data to the Network

1. Open File Explorer on the PC.
2. Navigate to the Data (D:)\Plates\(A-579-01) and find the plate saved in the section "Scanning Plate(s)".
3. Drag that folder to the network \\ab-file1\AnalySci\Immunospot\A-579-01\ELISpot.

Comments:

Technical Review: ______________________ QC Review: ______________________

Document Approvals

Number: METHOD-00081 Version: 1.0

Approval Task<br>Verdict: Approve	Sapna Varia,<br>(svaria@amplify-bio.com)<br>Approval<br>30-Oct-2023 14:40:40 GMT+0000

Appendix K Anatomic Pathology Contributing Scientist Report

A 12-Week Single Dose Biodistribution and Safety Study in Cynomolgus Monkeys

Anatomic Pathology Contributing Scientist Report

May 30, 2024

Study Number A-579-01

<u>Testing Facility</u>
AmplifyBio
1425 State Route 142 (Plain City-Georgesville Road)
West Jefferson, OH 43162

SIGNATURES

Prepared by:

DocuSigned by:

Joseph Novak

Signer Name: Joseph Novak
Signing Reason: I am the author of this document
Signing Time: 5/30/2024 | 9:13:10 AM EDT
7ED2BD0DC3124D49BC2086A4E65EE1ED

5/30/2024

Joseph Novak, DVM, DACVP
Study Pathologist

Date

TABLE OF CONTENTS

Page

Signatures **2**
1.0 Introduction **4**
2.0 Study Design and Methods **4**
3.0 Data Capture and Analysis **6**
4.0 Deviations **6**
5.0 Results **7**
5.1 Mortality 7
5.2 Terminal Body Weights and Organ Weights 7
5.3 Gross Pathology 7
5.4 Histopathology 7
6.0 Conclusions **10**

LIST OF ATTACHMENTS

Attachment 1. Incidence Summary of Gross Observations 11
Attachment 2. Incidence Summary of Microscopic Observations 24
Attachment 3. Individual Animal Organ Weights and Weight Ratios 42
Attachment 4. Individual Animal Gross and Microscopic Observations 72

1.0 INTRODUCTION

This contributing scientist report summarizes the anatomic pathology findings during Study No. A-579-01. The purpose of this study was to evaluate the potential biodistribution and safety of scAAV9.U6miR871 (ARM-101) when administered once via intrathecal infusion to Cynomolgus monkeys for up to 12 weeks.

2.0 STUDY DESIGN AND METHODS

The study design for anatomic pathology evaluations is outlined in Table 1.

Table 1. Experimental Design

Group	Target Dose (Total vg)[a]	Target Dose Volume (mL)	Animal Identification			
			6-Week Cohort[b]		12-Week Cohort[c]	
			Male	Female	Male	Female
1 – Control	0	3	101	151	102	152
2 – Low	6×10^{13}	3	201-202	251-252	203-204	253-254
3 – High	1.2×10^{14}	3	301-302	351-352	303-304	353-354

vg = vector genome.
a. See QE-001542 for dosing deviation.
b. Necropsy Day 43.
c. Necropsy Day 85.

All scheduled necropsies were conducted with a board-certified veterinary pathologist available for consultation. Each necropsy included a gross examination with collection of tissues as listed in Table 2 (see QE-001785 for deviation regarding stomach collection). Additional tissue collection was conducted as specified in the protocol.

Table 2. Tissues Collected

Tissue Collected	OW	HE	Tissue Collected	OW	HE
Animal identification[a]			Nerve, Median (proximal)		
Adrenal glands (paired)	X	X	Nerve, sciatic (distal)		X
Aorta		X	Nerve, sciatic (proximal)		X
Bone marrow (sternum)		X	Nerve, Optic		
Bone marrow smear (rib)[b]			Nerve, Ulnar (distal)		
Brain	X	X	Nerve, Ulnar (proximal)		
Cecum		X	Ovaries (paired)	X	X
Cervix		X	Pancreas		X
Colon		X	Parathyroid gland[e]		X
DRG, cervical		X	Pituitary gland	X	X
DRG, thoracic		X	Prostate		X
DRG, lumbar		X	Rectum		X
Duodenum		X	Salivary glands (mandibular)		X
Epididymides (paired)[c]	X	X	Seminal Vesicle		X
Esophagus		X	Skeletal muscle (thigh)		X
Eyes (paired)[d]		X	Skin		X
Femur		X	Spinal cord (cervical)		X
Gallbladder		X	Spinal cord (thoracic)		X
Gross lesions		X	Spinal cord (lumbar)		X
Heart	X	X	Spinal cord (sacral)		
Ileum		X	Spinal roots (lumbar)		
Jejunum		X	Spleen	X	X
Kidneys (paired)	X	X	Stomach (cardiac, fundus, pyloris)		X
Liver	X	X	Testes (paired)[c]	X	X
Lungs	X	X	Thymus	X	X
Lymph node, mandibular		X	Thyroid glands (paired)[f]	X	X
Lymph node, mesenteric		X	Trachea		X
Mammary gland		X	Urinary bladder		X
Nerve, Median (distal)			Uterus[g]	X	X
Nerve, Femoral (distal)			Vagina		X
Nerve, Femoral (proximal)			Skeletal Muscle (Tibialis anterior, L)		
Skeletal Muscle (Biceps brachii, L)			Skeletal Muscle (Tibialis anterior, R)		
Skeletal Muscle (Biceps brachii, R)					

OW = Organ Weights; HE = Histopathologic Evaluation; DRG = Dorsal Root Ganglion.

a. Collected but not processed.
b. Bone marrow cytology smears were prepared for all animals at scheduled necropsy.
c. Preserved in Modified Davidson's fixative, then transferred to 10% NBF.
d. Preserved in Davidson's fixative, then transferred to 10% NBF.
e. Examined only if in routine section.
f. Weighed with parathyroids embedded.
g. Weighed with cervix.

The collected tissues were fixed in 10% neutral-buffered formalin (except as noted in Table 2).

All protocol-required tissues were routinely processed to slides, stained with hematoxylin and eosin, and evaluated microscopically by a board-certified veterinary pathologist.

3.0 DATA CAPTURE AND ANALYSIS

Provantis (Instem) was used as the electronic data capture and data analysis system.

4.0 DEVIATIONS

QE-001542: On Day 1, the dose administration was not exactly 6 hours for all three groups.

For Group 1, Animal 101 had dose administered starting at 0957. There is documentation that the pump was paused at 1414 due to the Huber needle detaching from the port, however, there is no documentation of when the pump was restarted following this pause. It is unclear how long the dose was administered with the pump stop time documented at 1632. For the other Group 1 animals, the dose administration time was as followed: Animal 102 (5hrs 58mins), Animal 151 (5hrs 56mins), and Animal 152 (5hrs 3mins).

For Group 2, the dose administration time for each animal is as followed: Animal 201 (5hrs 56mins), Animal 202 (5hrs 56mins), Animal 203 (5hrs 57mins), Animal 204 (5hrs 55mins), Animal 251 (5hrs 56mins), Animal 252 (5hrs 54mins), Animal 253 (5hrs 56mins), and Animal 254 (5hrs 55mins).

For Group 3, the dose administration time for each animal is as followed: Animal 301 (5hrs 55mins), Animal 302 (5hrs 55mins), Animal 303 (5hrs 51mins), Animal 304 (6hrs 2mins), Animal 351 (5hrs 56mins), Animal 352 (5hrs 56mins), Animal 353 (5hrs 57mins), and Animal 354 (5hrs 58mins).

The impact of +/- 10 minutes on a 6 hour treatment is not likely to have an impact. The failed attempt to give full treatment to Animal 152 will have no effect since this animal was a control and not receiving test article.

QE-001785: The stomach was collected from all animals at necropsy on Day 43 for histopathologic evaluation, although not required by protocol. There was no study impact due to the collection of stomach from all animals as it was part of the standard list for tissue collection.

5.0 RESULTS

5.1 Mortality

All animals survived to scheduled termination.

5.2 Terminal Body Weights and Organ Weights

Individual Data: Attachment 3

There were not enough animals assigned to each cohort to statistically compare treatment groups (Groups 2 and 3) to controls (Group 1).

5.3 Gross Pathology

Summary Data: Attachment 1
Individual Data: Attachment 4

In the 6-week cohort, no test article-associated gross lesions were identified.

In the 12-week cohort, female Animal 353 had enlarged inguinal and mesenteric lymph nodes which corresponded to the microscopic finding of increased cellularity, lymphocyte.

Remaining gross lesions are common background lesions in nonhuman primates unrelated to treatment.

5.4 Histopathology

Summary Data: Attachment 2
Individual Data: Attachment 4

Histopathologic grades were assigned according to the following scale: minimal (1), mild (2), moderate (3), and marked (4), based on increasing extent and/or complexity of change.

Test article-related microscopic findings were limited to dorsal root ganglion and spinal cord and were present in both 6-week and 12-week cohorts. The dorsal root ganglion lesions included degeneration and necrosis of neurons and autophagy of neurons. Spinal cord lesions consisted of Wallerian degeneration of axons. Intracellular vacuolization of dorsal root ganglion neurons was noted but was a fixation artifact. See Table 3 and Table 4 for a summary of the test article-related microscopic findings.

In the 6-week cohort, Group 2 male Animal 202 had a mild cellular infiltrate of the liver consisting of mononuclear cells admixed with eosinophils. The infiltrate was primarily portal but extended into the hepatic parenchyma.

In the 12-week cohort, Group 3 male Animal 304 had mild, focal, mononuclear, perivascular infiltrate of the cerebrum. Additionally, Group 3 female Animal 353 had increased cellularity, lymphocyte, in inguinal and mesenteric lymph nodes.

Remaining lesions are common background lesions in nonhuman primates unrelated to treatment.

Table 3. Summary of Test Article-Related Microscopic Findings in the 6-Week Cohort (Day 43) Male and Female Cynomolgus Macaques

**Sex**	**Males**			**Females**		
**Group**	**1**	**2**	**3**	**1**	**2**	**3**
**Dose (Total vg)**	**0**	**6×10^{13}**	**1.2×10^{14}**	**0**	**6×10^{13}**	**1.2×10^{14}**
**No. of animals examined**	**1**	**2**	**2**	**1**	**2**	**2**
**Spinal cord (no. examined)**	**1**	**2**	**2**	**1**	**2**	**2**
Lumbar, axon degeneration, Wallerian	0	0	0	0	0	1 (1)
**Dorsal root ganglion (no. examined)**	**1**	**2**	**2**	**1**	**2**	**2**
Cervical (neck), necrosis, neuron	0	0	1 (1)	0	0	0
Cervical (neck), degeneration, neuron	0	0	1 (2)	0	0	0
Cervical (neck), autophagy, neuron	0	0	0	0	0	1 (1)
Thoracic, degeneration, neuron	0	1 (1)	1 (1)	0	0	0
Thoracic, autophagy, neuron	0	0	1 (1)	0	0	1 (1)
Thoracic, increased cellularity, mononuclear cell	0	1 (2)	0	0	0	0
Lumbar, degeneration, neuron	0	1 (1)	0	0	0	0
Lumbar, autophagy, neuron	0	0	0	0	0	1 (1)
Lumbar, increased cellularity, mononuclear cell	0	1 (2)	0	0	0	0

Average severity (in parentheses) was calculated by adding all the grades of affected animals in the group and diving by the number affected.

Table 4. Summary of Test Article-Related Microscopic Findings in the 12-Week Cohort (Day 85) Male and Female Cynomolgus Macaques

**Sex**	**Males**			**Females**		
**Group**	**1**	**2**	**3**	**1**	**2**	**3**
**Dose (Total vg)**	**0**	**$6×10^{13}$**	**$1.2×10^{14}$**	**0**	**$6×10^{13}$**	**$1.2×10^{14}$**
**No. of animals examined**	**1**	**2**	**2**	**1**	**2**	**2**
**Spinal cord (no. examined)**	**1**	**2**	**2**	**1**	**2**	**2**
Cervical (neck), axon degeneration, Wallerian	0	0	0	0	1 (1)	0
Thoracic, axon degeneration, Wallerian	0	0	1 (1)	0	1 (1)	0
Lumbar, axon degeneration, Wallerian	0	1 (1)	0	0	1 (1)	0
**Dorsal root ganglion (no. examined)**	**1**	**2**	**2**	**1**	**2**	**2**
Cervical (neck), necrosis, neuron	0	1 (1)	0	0	1 (1)	0
Cervical (neck), degeneration, neuron	0	1 (1)	1 (1)	0	1 (1)	0
Cervical (neck), increased cellularity, mononuclear cell	0	0	0	0	1 (2)	0
Thoracic, necrosis, neuron	0	0	1 (1)	0	0	0
Thoracic, degeneration, neuron	0	0	1 (1)	0	0	1 (1)
Thoracic, autophagy, neuron	0	0	0	1 (1)	0	0
Lumbar, necrosis, neuron	0	0	1 (1)	0	0	0
Lumbar, degeneration, neuron	0	1 (1)	0	0	1 (1)	0
Lumbar, autophagy, neuron	0	0	0	0	0	1 (1)
Lumbar, increased cellularity, mononuclear cell	0	0	0	0	1 (2)	1 (2)

The average severity (in parathesis) was calculated by adding all the grades of affected animals in the group and dividing by the number affected.

6.0 CONCLUSIONS

Cynomolgus macaques given scAAV9.U6miR871 (ARM-101) via intrathecal slow infusion and necropsied at 6 or 12-weeks had minimal to mild lesions within the spinal cord and dorsal root ganglion. Lesions were present in the 6-week and 12-week cohorts to the same or similar degree. Within the spinal cord, there was minimal Wallerian degeneration of axons. In dorsal root ganglions, there was minimal degeneration and necrosis of neurons. Occasionally there was increased mononuclear cells within the ganglions. None of the animals receiving test article displayed any clinical signs. Autophagy noted in several dorsal root ganglions represents programed cell death of neurons and is a common background finding in nonhuman primates but may also follow lethal injury to sensory cells. Intracytoplasmic vacuolization of neurons as seen in this study is consistent with fixation artifact.

Enlarged inguinal and mesenteric lymph nodes from Animal 353 corresponded to microscopic finding of increased cellularity, lymphocyte and is indicative of nonspecific immune stimulation which may or may not be associated with test article. The cause of focal, perivascular, mononuclear infiltrate in Animal 304 is unknown but may be induced by mild non-specific trauma.

Attachment 1. Incidence Summary of Gross Observations

AmplifyBio Study Number A-579-01

Incidence Summary of Gross Observations - 6 Week Cohort

Removal Reason(s): ALL<br>Summary: Incidence	Male			Female		
	Group 1 Control Total vg 0	Group 2 Low Total vg 6x10^13	Group 3 High Total vg 1.2x10^14	Group 1 Control Total vg 0	Group 2 Low Total vg 6x10^13	Group 3 High Total vg 1.2x10^14
Number of Animals:	1	2	2	1	2	2
Number of Completed Animals:	1	2	2	1	2	2
**ADRENAL GLANDS**						
Submitted	1	2	2	1	2	2
No Visible Lesions	1	2	2	1	2	2
**AORTA**						
Submitted	1	2	2	1	2	2
No Visible Lesions	1	2	2	1	2	2
**BONE MARROW**						
Submitted	1	2	2	1	2	2
No Visible Lesions	1	2	2	1	2	2
**BRAIN**						
Submitted	1	2	2	1	2	2
No Visible Lesions	1	2	2	1	2	2
**CECUM**						
Submitted	1	2	2	1	2	2
No Visible Lesions	1	2	2	1	2	2
**CERVIX**						
Submitted	.	.	.	1	2	2
No Visible Lesions	.	.	.	1	2	2
**COLON**						
Submitted	1	2	2	1	2	2
No Visible Lesions	1	2	2	1	2	2
**DUODENUM**						
Submitted	1	2	2	1	2	2
No Visible Lesions	1	2	2	1	2	2
**EPIDIDYMIS**						
Submitted	1	2	2	.	.	.
No Visible Lesions	1	2	2	.	.	.
**ESOPHAGUS**						
Submitted	1	2	2	1	2	2
No Visible Lesions	1	2	2	1	2	2
**EYES**						
Submitted	1	2	2	1	2	2

AmplifyBio Study Number A-579-01

Incidence Summary of Gross Observations - 6 Week Cohort

Removal Reason(s): ALL<br>Summary: Incidence	Male			Female		
	Group 1 Control Total vg 0	Group 2 Low Total vg 6x10^13	Group 3 High Total vg 1.2x10^14	Group 1 Control Total vg 0	Group 2 Low Total vg 6x10^13	Group 3 High Total vg 1.2x10^14
Number of Animals:	1	2	2	1	2	2
Number of Completed Animals:	1	2	2	1	2	2
**EYES (Continued...)**						
No Visible Lesions	1	2	2	1	2	2
**FEMUR**						
Submitted	1	2	2	1	2	2
No Visible Lesions	1	2	2	1	2	2
**GALLBLADDER**						
Submitted	1	2	2	1	2	2
No Visible Lesions	1	2	2	1	2	2
**HEART**						
Submitted	1	2	2	1	2	2
No Visible Lesions	1	2	2	1	2	2
**ILEUM**						
Submitted	1	2	2	1	2	2
No Visible Lesions	1	2	2	1	2	2
**JEJUNUM**						
Submitted	1	2	2	1	2	2
No Visible Lesions	1	2	2	1	2	2
**KIDNEYS**						
Submitted	1	2	2	1	2	2
No Visible Lesions	1	2	2	1	1	2
RIGHT; CYST(S); CLEAR	0	0	0	0	1	0
**LIVER**						
Submitted	1	2	2	1	2	2
No Visible Lesions	1	2	2	1	2	2
**LUNGS**						
Submitted	1	2	2	1	2	2
No Visible Lesions	1	2	1	1	2	2
LEFT; CAUDAL LOBE; FOCUS; WHITE, HARD	0	0	1	0	0	0
LEFT; CRANIAL LOBE; FOCUS; WHITE, HARD	0	0	1	0	0	0
**LYMPH NODES, MANDIBULAR**						
Submitted	1	2	2	1	2	2
No Visible Lesions	1	2	2	1	2	2

10/30/2023 10:44:38AM Page: 3

AmplifyBio Study Number A-579-01

Incidence Summary of Gross Observations - 6 Week Cohort

Removal Reason(s): ALL<br>Summary: Incidence	Male			Female		
	Group 1 Control Total vg 0	Group 2 Low Total vg 6x10^13	Group 3 High Total vg 1.2x10^14	Group 1 Control Total vg 0	Group 2 Low Total vg 6x10^13	Group 3 High Total vg 1.2x10^14
Number of Animals:	1	2	2	1	2	2
Number of Completed Animals:	1	2	2	1	2	2
**LYMPH NODES, MESENTERIC**						
Submitted	1	2	2	1	2	2
No Visible Lesions	1	2	2	1	2	2
**MAMMARY GLAND**						
Submitted	1	2	2	1	2	2
No Visible Lesions	1	2	2	1	2	2
**OPTIC NERVE**						
Submitted	1	2	2	1	2	2
No Visible Lesions	1	2	2	1	2	2
**OVARIES**						
Submitted	.	.	.	1	2	2
No Visible Lesions	.	.	.	1	2	1
RIGHT; CYST(S); CLEAR	.	.	.	0	0	1
**PANCREAS**						
Submitted	1	2	2	1	2	2
No Visible Lesions	1	2	2	1	2	2
**PARATHYROID GLAND**						
Submitted	1	2	2	1	2	2
No Visible Lesions	1	2	2	1	2	2
**PITUITARY GLAND**						
Submitted	1	2	2	1	2	2
No Visible Lesions	1	2	2	1	2	2
**PROSTATE**						
Submitted	1	2	2	.	.	.
No Visible Lesions	1	2	2	.	.	.
**RECTUM**						
Submitted	1	2	2	1	2	2
No Visible Lesions	1	2	2	1	2	2
**SALIVARY GLAND**						
Submitted	1	2	2	1	2	2
No Visible Lesions	1	2	2	1	2	2

10/30/2023 10:44:38AM Page: 4

AmplifyBio Study Number A-579-01

Incidence Summary of Gross Observations - 6 Week Cohort

Removal Reason(s): ALL<br>Summary: Incidence	Male			Female		
	Group 1 Control Total vg 0	Group 2 Low Total vg 6x10^13	Group 3 High Total vg 1.2x10^14	Group 1 Control Total vg 0	Group 2 Low Total vg 6x10^13	Group 3 High Total vg 1.2x10^14
Number of Animals:	1	2	2	1	2	2
Number of Completed Animals:	1	2	2	1	2	2
**SCIATIC NERVE**						
Submitted	1	2	2	1	2	2
No Visible Lesions	1	2	2	1	2	2
**SEMINAL VESICLES**						
Submitted	1	2	2	.	.	.
No Visible Lesions	1	2	2	.	.	.
**SKELETAL MUSCLE**						
Submitted	1	2	2	1	2	2
No Visible Lesions	1	2	2	1	2	2
**SKIN**						
Submitted	1	2	2	1	2	2
No Visible Lesions	1	2	2	1	2	2
**SPINAL CORD**						
Submitted	1	2	2	1	2	2
No Visible Lesions	1	2	2	1	2	2
**SPLEEN**						
Submitted	1	2	2	1	2	2
No Visible Lesions	1	2	2	1	2	2
**STOMACH**						
Submitted	1	2	2	1	2	2
No Visible Lesions	1	2	2	1	2	2
**TESTES**						
Submitted	1	2	2	.	.	.
No Visible Lesions	1	2	2	.	.	.
**THYMUS**						
Submitted	1	2	2	1	2	2
No Visible Lesions	1	2	2	1	2	2
**THYROID GLANDS**						
Submitted	1	2	2	1	2	2
No Visible Lesions	1	2	2	1	2	2
**TRACHEA**						
Submitted	1	2	2	1	2	2

AmplifyBio Study Number A-579-01

Incidence Summary of Gross Observations - 6 Week Cohort

Removal Reason(s): ALL<br>Summary: Incidence	Male			Female		
	Group 1 Control Total vg 0	Group 2 Low Total vg 6x10^13	Group 3 High Total vg 1.2x10^14	Group 1 Control Total vg 0	Group 2 Low Total vg 6x10^13	Group 3 High Total vg 1.2x10^14
Number of Animals:	1	2	2	1	2	2
Number of Completed Animals:	1	2	2	1	2	2
**TRACHEA (Continued...)**						
No Visible Lesions	1	2	2	1	2	2
**URINARY BLADDER**						
Submitted	1	2	2	1	2	2
No Visible Lesions	1	2	2	1	2	2
**UTERUS**						
Submitted	.	.	.	1	2	2
No Visible Lesions	.	.	.	1	2	2
**VAGINA**						
Submitted	.	.	.	1	2	2
No Visible Lesions	.	.	.	1	2	2
**FEMORAL NERVE**						
Submitted	1	2	2	1	2	2
No Visible Lesions	1	2	2	1	2	2
**NERVE, MEDIAN**						
Submitted	1	2	2	1	2	2
No Visible Lesions	1	2	2	1	2	2
**ULNAR NERVE**						
Submitted	1	2	2	1	2	2
No Visible Lesions	1	2	2	1	2	2
**SPINAL ROOTS**						
Submitted	1	2	2	1	2	2
No Visible Lesions	1	2	2	1	2	2
**GANGLION, DORSAL ROOT**						
Submitted	1	2	2	1	2	2
No Visible Lesions	1	2	2	1	2	2

10/30/2023 10:44:38AM Page: 6

AmplifyBio Study Number A-579-01

Incidence Summary of Gross Observations - 6 Week Cohort

Key Page

Report Request Items

Animals Excluded:	152, 102, 253, 254, 203, 204, 353, 354, 303, 304
Groups:	All
Observation Type:	Gross
Tissues:	All
Removal Reasons:	Core Necropsy

Measurement/Statistics

Measurement	Descriptive	Comparative	Arithmetic /Adjusted	Transformation
Pathology Observation	Count Positives			

Group Information

Short Name	Long Name	Type	Report Headings			
1	Group 1	Control	Group 1	Control	Total vg	0
2	Group 2	Dose	Group 2	Low	Total vg	6x10^13
3	Group 3	Dose	Group 3	High	Total vg	1.2x10^14

Removal Reason Grouping

Grouping Name	Abbreviation	Removal Reasons
Core Necropsy	CN	Core Necropsy

10/30/2023 10:44:38AM Page: 1

AmplifyBio Study Number A-579-01

Incidence Summary of Gross Observations - 12 Week Cohort

Removal Reason(s): ALL	Male			Female		
Summary: Incidence	Group 1 Control Total vg 0	Group 2 Low Total vg 6x10^13	Group 3 High Total vg 1.2x10^14	Group 1 Control Total vg 0	Group 2 Low Total vg 6x10^13	Group 3 High Total vg 1.2x10^14
Number of Animals:	1	2	2	1	2	2
Number of Completed Animals:	1	2	2	1	2	2
**ADRENAL GLANDS**						
Submitted	1	2	2	1	2	2
No Visible Lesions	1	2	2	1	2	2
**AORTA**						
Submitted	1	2	2	1	2	2
No Visible Lesions	1	2	2	1	2	2
**BONE MARROW**						
Submitted	1	2	2	1	2	2
No Visible Lesions	1	2	2	1	2	2
**BRAIN**						
Submitted	1	2	2	1	2	2
No Visible Lesions	1	2	2	0	2	1
BILATERAL; DILATATION	0	0	0	0	0	1
UNILATERAL; LEFT; DILATATION	0	0	0	1	0	0
**CECUM**						
Submitted	1	2	2	1	2	2
No Visible Lesions	1	2	2	1	2	2
**CERVIX**						
Submitted	.	.	.	1	2	2
No Visible Lesions	.	.	.	1	2	2
**COLON**						
Submitted	1	2	2	1	2	2
No Visible Lesions	1	2	2	1	2	2
**DUODENUM**						
Submitted	1	2	2	1	2	2
No Visible Lesions	1	2	2	1	2	2
**EPIDIDYMIS**						
Submitted	1	2	2	.	.	.
No Visible Lesions	1	2	2	.	.	.
**ESOPHAGUS**						
Submitted	1	2	2	1	2	2
No Visible Lesions	1	2	2	1	2	2

10/30/2023 10:44:38AM Page: 2

AmplifyBio Study Number A-579-01

Incidence Summary of Gross Observations - 12 Week Cohort

Removal Reason(s): ALL	Male			Female		
Summary: Incidence	Group 1 Control Total vg 0	Group 2 Low Total vg 6x10^13	Group 3 High Total vg 1.2x10^14	Group 1 Control Total vg 0	Group 2 Low Total vg 6x10^13	Group 3 High Total vg 1.2x10^14
Number of Animals:	1	2	2	1	2	2
Number of Completed Animals:	1	2	2	1	2	2
**EYES**						
Submitted	1	2	2	1	2	2
No Visible Lesions	1	2	2	1	2	2
**FEMUR**						
Submitted	1	2	2	1	2	2
No Visible Lesions	1	2	2	1	2	2
**GALLBLADDER**						
Submitted	1	2	2	1	2	2
No Visible Lesions	1	2	2	1	2	2
**HEART**						
Submitted	1	2	2	1	2	2
No Visible Lesions	1	2	2	1	2	2
**ILEUM**						
Submitted	1	2	2	1	2	2
No Visible Lesions	1	2	2	1	2	2
**JEJUNUM**						
Submitted	1	2	2	1	2	2
No Visible Lesions	1	2	2	1	2	2
**KIDNEYS**						
Submitted	1	2	2	1	2	2
No Visible Lesions	1	2	2	1	2	2
**LIVER**						
Submitted	1	2	2	1	2	2
No Visible Lesions	1	2	2	1	2	2
**LUNGS**						
Submitted	1	2	2	1	2	2
No Visible Lesions	1	2	2	1	1	2
DIFFUSE; FOCI; WHITE	0	0	0	0	1	0
**LYMPH NODES, INGUINAL**						
Submitted	.	.	.	0	0	1
LEFT; ENLARGED; ONE	.	.	.	.	.	1

10/30/2023 10:44:38AM Page: 3

AmplifyBio Study Number A-579-01

Incidence Summary of Gross Observations - 12 Week Cohort

Removal Reason(s): ALL	Male			Female		
Summary: Incidence	Group 1 Control Total vg 0	Group 2 Low Total vg 6x10^13	Group 3 High Total vg 1.2x10^14	Group 1 Control Total vg 0	Group 2 Low Total vg 6x10^13	Group 3 High Total vg 1.2x10^14
Number of Animals:	1	2	2	1	2	2
Number of Completed Animals:	1	2	2	1	2	2
**LYMPH NODES, MANDIBULAR**						
Submitted	1	2	2	1	2	2
No Visible Lesions	1	2	2	1	2	2
**LYMPH NODES, MESENTERIC**						
Submitted	1	2	2	1	2	2
No Visible Lesions	1	2	2	1	2	1
DIFFUSE; ENLARGED; MULTIPLE	0	0	0	0	0	1
**MAMMARY GLAND**						
Submitted	1	2	2	1	2	2
No Visible Lesions	1	2	2	1	2	2
**OPTIC NERVE**						
Submitted	1	2	2	1	2	2
No Visible Lesions	1	2	2	1	2	2
**OVARIES**						
Submitted	.	.	.	1	2	2
No Visible Lesions	.	.	.	1	2	2
**PANCREAS**						
Submitted	1	2	2	1	2	2
No Visible Lesions	1	2	2	1	2	2
**PARATHYROID GLAND**						
Submitted	1	2	2	1	2	2
No Visible Lesions	1	2	2	1	2	2
**PITUITARY GLAND**						
Submitted	1	2	2	1	2	2
No Visible Lesions	1	2	2	1	2	2
**PROSTATE**						
Submitted	1	2	2	.	.	.
No Visible Lesions	1	2	2	.	.	.
**RECTUM**						
Submitted	1	2	2	1	2	2
No Visible Lesions	1	2	2	1	2	2

10/30/2023 10:44:38AM Page: 4

AmplifyBio Study Number A-579-01

Incidence Summary of Gross Observations - 12 Week Cohort

Removal Reason(s): ALL<br>Summary: Incidence	Male			Female		
	Group 1 Control Total vg 0	Group 2 Low Total vg 6x10^13	Group 3 High Total vg 1.2x10^14	Group 1 Control Total vg 0	Group 2 Low Total vg 6x10^13	Group 3 High Total vg 1.2x10^14
Number of Animals:	1	2	2	1	2	2
Number of Completed Animals:	1	2	2	1	2	2
**SALIVARY GLAND**						
Submitted	1	2	2	1	2	2
No Visible Lesions	1	2	2	1	2	2
**SCIATIC NERVE**						
Submitted	1	2	2	1	2	2
No Visible Lesions	1	2	2	1	2	2
**SEMINAL VESICLES**						
Submitted	1	2	2	.	.	.
No Visible Lesions	1	2	2	.	.	.
**SKELETAL MUSCLE**						
Submitted	1	2	2	1	2	2
No Visible Lesions	1	2	2	1	2	2
**SKIN**						
Submitted	1	2	2	1	2	2
No Visible Lesions	0	2	2	1	2	1
LEFT; DORSAL; CRUST(S)	0	0	0	0	0	1
RIGHT; FORELIMB; CRUST(S)	1	0	0	0	0	0
**SPINAL CORD**						
Submitted	1	2	2	1	2	2
No Visible Lesions	1	2	2	1	2	2
**SPLEEN**						
Submitted	1	2	2	1	2	2
No Visible Lesions	1	2	2	1	2	2
**STOMACH**						
Submitted	1	2	2	1	2	2
No Visible Lesions	1	2	2	1	2	2
**TESTES**						
Submitted	1	2	2	.	.	.
No Visible Lesions	1	2	2	.	.	.
**THYMUS**						
Submitted	1	2	2	1	2	2
No Visible Lesions	1	2	2	1	2	2

AmplifyBio Study Number A-579-01

Incidence Summary of Gross Observations - 12 Week Cohort

Removal Reason(s): ALL	Male			Female		
Summary: Incidence	Group 1 Control Total vg 0	Group 2 Low Total vg 6x10^13	Group 3 High Total vg 1.2x10^14	Group 1 Control Total vg 0	Group 2 Low Total vg 6x10^13	Group 3 High Total vg 1.2x10^14
Number of Animals:	1	2	2	1	2	2
Number of Completed Animals:	1	2	2	1	2	2
**THYROID GLANDS**						
Submitted	1	2	2	1	2	2
No Visible Lesions	1	2	2	1	2	2
**TRACHEA**						
Submitted	1	2	2	1	2	2
No Visible Lesions	1	2	2	1	2	2
**URINARY BLADDER**						
Submitted	1	2	2	1	2	2
No Visible Lesions	1	2	2	1	2	2
**UTERUS**						
Submitted	.	.	.	1	2	2
No Visible Lesions	.	.	.	1	2	2
**VAGINA**						
Submitted	.	.	.	1	2	2
No Visible Lesions	.	.	.	1	2	2
**FEMORAL NERVE**						
Submitted	1	2	2	1	2	2
No Visible Lesions	1	2	2	1	2	2
**NERVE, MEDIAN**						
Submitted	1	2	2	1	2	2
No Visible Lesions	1	2	2	1	2	2
**ULNAR NERVE**						
Submitted	1	2	2	1	2	2
No Visible Lesions	1	2	2	1	2	2
**SPINAL ROOTS**						
Submitted	1	2	2	1	2	2
No Visible Lesions	1	2	2	1	2	2
**GANGLION, DORSAL ROOT**						
Submitted	1	2	2	1	2	2
No Visible Lesions	1	2	2	1	2	2

10/30/2023 10:44:38AM Page: 6

AmplifyBio Study Number A-579-01

Incidence Summary of Gross Observations - 12 Week Cohort

Key Page

Report Request Items

Animals Excluded:	151, 101, 251, 252, 201, 202, 351, 352, 301, 302
Groups:	All
Observation Type:	Gross
Tissues:	All
Removal Reasons:	Recovery Necropsy 1

Measurement/Statistics

Measurement	Descriptive	Comparative	Arithmetic /Adjusted	Transformation
Pathology Observation	Count Positives			

Group Information

Short Name	Long Name	Type	Report Headings			
1	Group 1	Control	Group 1	Control	Total vg	0
2	Group 2	Dose	Group 2	Low	Total vg	6x10^13
3	Group 3	Dose	Group 3	High	Total vg	1.2x10^14

Removal Reason Grouping

Grouping Name	Abbreviation	Removal Reasons
Recovery Necropsy 1	R1	Recovery Necropsy 1

Attachment 2. Incidence Summary of Microscopic Observations

10/30/2023 10:46:15AM Page: 1

AmplifyBio Study Number A-579-01

Incidence Summary of Microscopic Observations - 6 Week Cohort

Removal Reason(s): ALL<br>Summary: Average Grade (Mean)	Male			Female		
	Group 1 Control Total vg 0	Group 2 Low Total vg 6x10^13	Group 3 High Total vg 1.2x10^14	Group 1 Control Total vg 0	Group 2 Low Total vg 6x10^13	Group 3 High Total vg 1.2x10^14
Number of Animals:	1	2	2	1	2	2
Number of Completed Animals:	1	2	2	1	2	2
**ADRENAL GLANDS**						
Examined	1	2	2	1	2	2
No Visible Lesions	0	1	2	1	1	2
MEDULLA; INFILTRATE, CELLULAR; MONONUCLEAR CELL	0	1	0	0	0	0
.... MILD	0	1	0	0	0	0
.... Average Grade	0	2	0	0	0	0
CORTEX; MINERALIZATION	1	0	0	0	1	0
.... MILD	1	0	0	0	1	0
.... Average Grade	2	0	0	0	2	0
**AORTA**						
Examined	1	2	2	1	2	2
No Visible Lesions	1	2	2	1	2	2
**BONE MARROW**						
Examined	1	2	2	1	2	2
No Visible Lesions	1	2	2	1	2	2
**BRAIN**						
Examined	1	2	2	1	2	2
No Visible Lesions	1	2	2	1	2	2
**CECUM**						
Examined	1	2	2	1	2	2
No Visible Lesions	1	2	2	1	2	2
**CERVIX**						
Examined	.	.	.	1	2	2
No Visible Lesions	.	.	.	1	2	2
**COLON**						
Examined	1	2	2	1	2	2
No Visible Lesions	1	2	2	1	2	2
**DUODENUM**						
Examined	1	2	2	1	2	2
No Visible Lesions	1	2	2	1	2	2

10/30/2023 10:46:15AM Page: 2

AmplifyBio Study Number A-579-01

Incidence Summary of Microscopic Observations - 6 Week Cohort

Removal Reason(s): ALL<br>Summary: Average Grade (Mean)	Male			Female		
	Group 1 Control Total vg 0	Group 2 Low Total vg 6x10^13	Group 3 High Total vg 1.2x10^14	Group 1 Control Total vg 0	Group 2 Low Total vg 6x10^13	Group 3 High Total vg 1.2x10^14
Number of Animals:	1	2	2	1	2	2
Number of Completed Animals:	1	2	2	1	2	2
**EPIDIDYMIS**						
Examined	1	2	2	.	.	.
No Visible Lesions	1	2	2	.	.	.
**ESOPHAGUS**						
Examined	1	2	2	1	2	2
No Visible Lesions	1	2	2	1	2	2
**EYES**						
Examined	1	2	2	1	2	2
No Visible Lesions	1	2	2	1	2	2
**(EMUR**						
Examined	1	2	2	1	2	2
No Visible Lesions	1	2	2	1	2	2
**GALLBLADDER**						
Examined	1	2	2	1	2	2
No Visible Lesions	1	2	2	1	2	2
**HEART**						
Examined	1	2	2	1	2	2
No Visible Lesions	0	2	2	1	2	2
INFILTRATE, CELLULAR; MONONUCLEAR CELL	1	0	0	0	0	0
.... MINIMAL	1	0	0	0	0	0
.... Average Grade	1	0	0	0	0	0
**ILEUM**						
Examined	1	2	2	1	2	2
No Visible Lesions	1	2	2	1	2	2
**oEoUNUM**						
Examined	1	2	2	1	2	2
No Visible Lesions	1	2	2	1	2	2
**n IDNEYS**						
Examined	1	2	2	1	2	2
No Visible Lesions	1	2	2	1	2	2
**LIVER**						
Examined	1	2	2	1	2	2

10/30/2023 10:46:15AM Page: 3

AmplifyBio Study Number A-579-01

Incidence Summary of Microscopic Observations - 6 Week Cohort

Removal Reason(s): ALL<br>Summary: Average Grade (Mean)	Male			Female		
	Group 1 Control Total vg 0	Group 2 Low Total vg 6x10^13	Group 3 High Total vg 1.2x10^14	Group 1 Control Total vg 0	Group 2 Low Total vg 6x10^13	Group 3 High Total vg 1.2x10^14
Number of Animals:	1	2	2	1	2	2
Number of Completed Animals:	1	2	2	1	2	2
**LIVER tCi uedı.) FJIIK**						
No Visible Lesions	0	1	2	1	2	2
PORTAL; INFILTRATE, CELLULAR; EOSINOPHILIC	0	1	0	0	0	0
.... MILD	0	1	0	0	0	0
.... Average Grade	0	2	0	0	0	0
INFLAMMATION; EOSINOPHILIC	1	0	0	0	0	0
.... MILD	1	0	0	0	0	0
.... Average Grade	2	0	0	0	0	0
**LUNGS**						
Examined	1	2	2	1	2	2
No Visible Lesions	1	2	1	1	2	2
PERIVASCULAR; INFILTRATE, CELLULAR; HISTIOCYTIC	0	0	1	0	0	0
.... MILD	0	0	1	0	0	0
.... Average Grade	0	0	2	0	0	0
**LYMPH NODES, MANDIBULAR**						
Examined	1	2	2	1	2	2
No Visible Lesions	1	2	2	1	2	2
**LYMPH NODES, MESENTERIC**						
Examined	1	2	2	1	2	2
No Visible Lesions	1	2	2	1	2	2
**MAMMARY GLAND**						
Examined	1	2	2	1	2	2
No Visible Lesions	1	2	2	1	2	2
**OVARIES**						
Examined	.	.	.	1	2	2
No Visible Lesions	.	.	.	1	2	2
**PANCREAS**						
Examined	1	2	2	1	2	2
No Visible Lesions	1	2	2	1	2	2
**PARATHYROID GLAND**						
Examined	1	2	2	1	2	1

10/30/2023 10:46:15AM Page: 4

AmplifyBio Study Number A-579-01

Incidence Summary of Microscopic Observations - 6 Week Cohort

Removal Reason(s): ALL<br>Summary: Average Grade (Mean)	Male			Female		
	Group 1 Control Total vg 0	Group 2 Low Total vg 6x10^13	Group 3 High Total vg 1.2x10^14	Group 1 Control Total vg 0	Group 2 Low Total vg 6x10^13	Group 3 High Total vg 1.2x10^14
Number of Animals:	1	2	2	1	2	2
Number of Completed Animals:	1	2	2	1	2	2
**PARATHYROID GLAND tCi uedı.)FJIIK**						
No Visible Lesions	1	2	2	1	2	1
Not Examined: No Section	.	.	.	0	0	1
**PITUITARY GLAND**						
Examined	1	2	2	1	2	2
No Visible Lesions	1	2	2	1	2	2
**PROSTATE**						
Examined	1	2	2	.	.	.
No Visible Lesions	1	2	2	.	.	.
**RECTUM**						
Examined	1	2	2	1	2	2
No Visible Lesions	1	2	2	1	2	2
**SALIVARY GLAND**						
Examined	1	2	2	1	2	2
No Visible Lesions	1	2	2	0	1	2
SUBMANDIBULAR; INFILTRATE, CELLULAR; LYMPHOPLASMACYTIC	0	0	0	1	1	0
.... MILD	0	0	0	1	1	0
.... Average Grade	0	0	0	2	2	0
**SCIATIC NERVE**						
Examined	1	2	2	1	2	2
No Visible Lesions	1	2	2	1	2	2
**SEMINAL VESICLES**						
Examined	1	2	2	.	.	.
No Visible Lesions	1	2	2	.	.	.
**Sn ELETAL MUSCLE**						
Examined	1	2	2	1	2	2
No Visible Lesions	1	2	2	1	2	2
**Sn IN**						
Examined	1	2	2	1	2	2
No Visible Lesions	1	2	2	1	2	2

AmplifyBio Study Number A-579-01

Incidence Summary of Microscopic Observations - 6 Week Cohort

Removal Reason(s): ALL	Male			Female		
Summary: Average Grade (Mean)	Group 1 Control Total vg 0	Group 2 Low Total vg 6x10^13	Group 3 High Total vg 1.2x10^14	Group 1 Control Total vg 0	Group 2 Low Total vg 6x10^13	Group 3 High Total vg 1.2x10^14
Number of Animals:	1	2	2	1	2	2
Number of Completed Animals:	1	2	2	1	2	2
**SPINAL CORD**						
Examined	1	2	2	1	2	2
No Visible Lesions	1	2	2	1	2	1
LUMBAR; AXON; DEGENERATION; WALLERIAN	0	0	0	0	0	1
.... MINIMAL	0	0	0	0	0	1
.... Average Grade	0	0	0	0	0	1
**SPLEEN**						
Examined	1	2	2	1	2	2
No Visible Lesions	1	2	2	1	2	2
**STOMACH**						
Examined	1	2	2	1	2	2
No Visible Lesions	1	2	2	1	2	2
**TESTES**						
Examined	1	2	2	.	.	.
No Visible Lesions	1	2	2	.	.	.
**THYMUS**						
Examined	1	2	2	1	2	2
No Visible Lesions	1	1	2	1	1	0
CORTEX; LYMPHOID ATROPHY	0	1	0	0	1	2
.... MINIMAL	0	1	0	0	1	2
.... Average Grade	0	1	0	0	1	1
**THYROID GLANDS**						
Examined	1	2	2	1	2	2
No Visible Lesions	1	2	2	1	2	2
**TRACHEA**						
Examined	1	2	2	1	2	2
No Visible Lesions	1	2	2	1	2	2
**URINARY BLADDER**						
Examined	1	2	2	1	2	2
No Visible Lesions	1	2	2	1	2	2
**UTERUS**						
Examined	.	.	.	1	2	2

AmplifyBio Study Number A-579-01

Incidence Summary of Microscopic Observations - 6 Week Cohort

Removal Reason(s): ALL	Male			Female		
Summary: Average Grade (Mean)	Group 1 Control Total vg 0	Group 2 Low Total vg 6x10^13	Group 3 High Total vg 1.2x10^14	Group 1 Control Total vg 0	Group 2 Low Total vg 6x10^13	Group 3 High Total vg 1.2x10^14
Number of Animals:	1	2	2	1	2	2
Number of Completed Animals:	1	2	2	1	2	2
**UTERUS tCi uedı.)FJJJK**						
No Visible Lesions	.	.	.	1	2	2
**VAGINA**						
Examined	.	.	.	1	2	2
No Visible Lesions	.	.	.	1	2	2
**SPINAL ROOTS**						
Examined	1	2	2	1	2	2
No Visible Lesions	1	2	2	1	2	2
**GANGLION, DORSAL ROOT**						
Examined	1	2	2	1	2	2
No Visible Lesions	1	1	0	1	2	0
CERVICAL (NECK); NEURON; NECROSIS	0	0	1	0	0	0
.... MINIMAL	0	0	1	0	0	0
.... Average Grade	0	0	1	0	0	0
LUMBAR; INCREASED CELLULARITY; MONONUCLEAR CELL	0	1	0	0	0	0
.... MILD	0	1	0	0	0	0
.... Average Grade	0	2	0	0	0	0
THORACIC; INCREASED CELLULARITY; MONONUCLEAR CELL	0	1	0	0	0	0
.... MILD	0	1	0	0	0	0
.... Average Grade	0	2	0	0	0	0
CERVICAL (NECK); NEURON; DEGENERATION	0	0	1	0	0	0
.... MILD	0	0	1	0	0	0
.... Average Grade	0	0	2	0	0	0
LUMBAR; NEURON; DEGENERATION	0	1	0	0	0	0
.... MINIMAL	0	1	0	0	0	0
.... Average Grade	0	1	0	0	0	0
THORACIC; NEURON; DEGENERATION	0	1	1	0	0	0
.... MINIMAL	0	1	1	0	0	0
.... Average Grade	0	1	1	0	0	0
CERVICAL (NECK); AUTOPHAGY, NEURON	0	0	0	0	0	1

10/30/2023 10:46:15AM Page: 7

AmplifyBio Study Number A-579-01

Incidence Summary of Microscopic Observations - 6 Week Cohort

Removal Reason(s): ALL<br>Summary: Average Grade (Mean)	Male			Female		
	Group 1 Control Total vg 0	Group 2 Low Total vg 6x10^13	Group 3 High Total vg 1.2x10^14	Group 1 Control Total vg 0	Group 2 Low Total vg 6x10^13	Group 3 High Total vg 1.2x10^14
Number of Animals:	1	2	2	1	2	2
Number of Completed Animals:	1	2	2	1	2	2
**GANGLION, DORSAL ROOT tCi uedı.) FJIIK**						
.... MINIMAL	0	0	0	0	0	1
.... Average Grade	0	0	0	0	0	1
LUMBAR; AUTOPHAGY, NEURON	0	0	0	0	0	1
.... MINIMAL	0	0	0	0	0	1
.... Average Grade	0	0	0	0	0	1
THORACIC; AUTOPHAGY, NEURON	0	0	1	0	0	1
.... MINIMAL	0	0	1	0	0	1
.... Average Grade	0	0	1	0	0	1
LUMBAR; NEURON; VACUOLIZATION CYTOPLASM	0	0	0	0	0	2
.... MILD	0	0	0	0	0	2
.... Average Grade	0	0	0	0	0	2

10/30/2023 10:46:15AM Page: 8

AmplifyBio Study Number A-579-01

Incidence Summary of Microscopic Observations - 6 Week Cohort

Key Page

R)pi re R)q.)se Ie)ms

Animals Excluded:	152, 102, 253, 254, 203, 204, 353, 354, 303, 304
Groups:	All
Observation Type:	Histo - Neoplastic and Non-Neoplastic
Tissues:	All
Removal Reasons:	Core Necropsy

M)as. r)m)ue/Seacdsedcs

Measurement	Descriptive	Comparative	Arithmetic /Adjusted	Transformation
Pathology Observation	Mean			
Pathology Observation	Count Positives			

Gri . p Iufi rmaed u

Short Name	Long Name	Type	Report Headings			
1	Group 1	Control	Group 1	Control	Total vg	0
2	Group 2	Dose	Group 2	Low	Total vg	6x10^13
3	Group 3	Dose	Group 3	High	Total vg	1.2x10^14

R)mi val R)asi u Gri . pdıg

Grouping Name	Abbreviation	Removal Reasons
Core Necropsy	CN	Core Necropsy

10/30/6063 102 5210AM 4aPe21

AmplifyBio Study Number A-579-01

Incidence Summary of Microscopic Observations - 16 Week Cohort

Femoval FeasonRs(2A)) Summary2AveraPe Lrade RMean(	Male			gemale		
	Lroup 1 Control Gotal vP 0	Lroup 6) o^ Gotal vP w. 10x1 3	Lroup 3 TiPh Gotal vP 1H6. 10x 1:	Lroup 1 Control Gotal vP 0	Lroup 6) o^ Gotal vP w. 10x1 3	Lroup 3 TiPh Gotal vP 1H6. 10x 1:
Number of Animals2	1	6	6	1	6	6
Number of Completed Animals2	1	6	6	1	6	6
**ADRENAL GLANDS**						
E. amined	1	6	6	1	6	6
No Visible) esions	1	6	6	1	6	6
**AORTA**						
E. amined	1	6	6	1	6	6
No Visible) esions	1	6	6	1	6	6
**BONE MARROW**						
E. amined	1	6	6	1	6	6
No Visible) esions	1	6	6	1	6	6
**BRAIN**						
E. amined	1	6	6	1	6	6
No Visible) esions	1	6	1	1	6	6
CEF EBF DMU4 EF IVASCD) AF UINgI) GF AGE; CE)) D) AF UMONONDC) EAF CE)) ; gOCA)	0	0	1	0	0	0
HHHHMI) ,	0	0	1	0	0	0
HHHHAveraPe Lrade	0	0	6	0	0	0
**CECUM**						
E. amined	1	6	6	1	6	6
No Visible) esions	1	6	6	1	6	6
**CERVIX**						
E. amined	H	H	H	1	6	6
No Visible) esions	H	H	H	1	6	6
**COLON**						
E. amined	1	6	6	1	6	6
No Visible) esions	1	6	6	1	6	6
**DUODENUM**						
E. amined	1	6	6	1	6	6
No Visible) esions	1	6	6	1	6	6
**EPIDIDYMIS**						
E. amined	1	6	6	H	H	H
No Visible) esions	1	6	6	H	H	H

10/30/6063 102 5210AM 4aPe26

AmplifyBio Study Number A-579-01

Incidence Summary of Microscopic Observations - 16 Week Cohort

Femoval Feason Rs(2A)) Summary2AveraPe Lrade RMean(	Male Lroup 1 Control Gotal vP 0	Lroup 6) o^ Gotal vP w. 10x1 3	Lroup 3 TiPh Gotal vP 1H6. 10x 1:	gemale Lroup 1 Control Gotal vP 0	Lroup 6) o^ Gotal vP w. 10x1 3	Lroup 3 TiPh Gotal vP 1H6. 10x 1:
Number of Animals2	1	6	6	1	6	6
Number of Completed Animals2	1	6	6	1	6	6
**ESOPHAGUS**						
E. amined	1	6	6	1	6	6
No Visible) esions	1	6	6	1	6	6
**EYES**						
E. amined	1	6	6	1	6	6
No Visible) esions	1	6	6	1	6	6
**(EMUR**						
E. amined	1	6	6	1	6	6
No Visible) esions	1	6	6	1	6	6
**GALLBLADDER**						
E. amined	1	6	6	1	6	6
No Visible) esions	1	6	6	1	6	6
**HEART**						
E. amined	1	6	6	1	6	6
No Visible) esions	1	6	6	1	6	1
INgI) GFAGE; CE)) D) AFUMONONDC) EAF CE))	0	0	0	0	0	1
HHHHMINIMA)	0	0	0	0	0	1
HHHHAveraPe Lrade	0	0	0	0	0	1
**ILEUM**						
E. amined	1	6	6	1	6	6
No Visible) esions	1	6	6	1	6	6
**oEoUNUM**						
E. amined	1	6	6	1	6	6
No Visible) esions	1	6	6	1	6	6
**n IDNEYS**						
E. amined	1	6	6	1	6	6
No Visible) esions	1	6	6	1	6	6
**LIVER**						
E. amined	1	6	6	1	6	6
No Visible) esions	1	6	1	1	1	6
INg) AMMAGIONUMONONDC) EAF CE)) ; gOCA)	0	0	1	0	1	0
HHHHMINIMA)	0	0	1	0	1	0

10/30/6063 102 5210AM 4aPe23

AmplifyBio Study Number A-579-01

Incidence Summary of Microscopic Observations - 16 Week Cohort

F emoval F easonRs(2A))<br>Summary2AveraPe L rade RMean(	Male			gemale		
	L roup 1 Control Gotal vP 0	L roup 6) o^ Gotal vP w. 10x1 3	L roup 3 T iPh Gotal vP 1H6. 10x 1:	L roup 1 Control Gotal vP 0	L roup 6) o^ Gotal vP w. 10x1 3	L roup 3 T iPh Gotal vP 1H6. 10x 1:
Number of Animals2	1	6	6	1	6	6
Number of Completed Animals2	1	6	6	1	6	6
**LIVER tCi uedı.) FJIIK**						
HHHHAveraPe L rade	0	0	1	0	1	0
**LUNGS**						
E. amined	1	6	6	1	6	6
No Visible) esions	1	6	6	1	1	6
A) VEO) DSUINgI) GF AGE; CE)) D) AF U T ISGIOCXGIC	0	0	0	0	1	0
HHHHMI) ,	0	0	0	0	1	0
HHHHAveraPe L rade	0	0	0	0	6	0
**LYMPH NODES, INGUINAL**						
E. amined	H	H	H	0	0	1
INCF EASE, CE)) D) AF IGXU) XM4T OCXGIC	H	H	H	H	H	1
HHHHMI) ,	H	H	H	H	H	1
HHHHAveraPe L rade	H	H	H	H	H	6
**LYMPH NODES, MANDIBULAR**						
E. amined	1	6	6	1	6	6
No Visible) esions	1	6	6	1	6	6
**LYMPH NODES, MESENTERIC**						
E. amined	1	6	6	1	6	6
No Visible) esions	1	6	6	1	6	1
INCF EASE, CE)) D) AF IGXU) XM4T OCXGIC	0	0	0	0	0	1
HHHHMI) ,	0	0	0	0	0	1
HHHHAveraPe L rade	0	0	0	0	0	6
**MAMMARY GLAND**						
E. amined	1	6	6	1	6	6
No Visible) esions	1	6	6	1	6	6
**OVARIES**						
E. amined	H	H	H	1	6	6
No Visible) esions	H	H	H	1	6	1
CXSG	H	H	H	0	0	1
**PANCREAS**						
E. amined	1	6	6	1	6	6

10/30/6063 102 5210AM 4aPe2:

AmplifyBio Study Number A-579-01

Incidence Summary of Microscopic Observations - 16 Week Cohort

Femoval FeasonRs(2A)) Summary2 AveraPe Lrade RMean(	Male			gemale		
	Lroup 1 Control Gotal vP 0	Lroup 6) o^ Gotal vP w. 10x1 3	Lroup 3 TiPh Gotal vP 1H6. 10x 1:	Lroup 1 Control Gotal vP 0	Lroup 6) o^ Gotal vP w. 10x1 3	Lroup 3 TiPh Gotal vP 1H6. 10x 1:
Number of Animals2	1	6	6	1	6	6
Number of Completed Animals2	1	6	6	1	6	6
**PANCREAS tCi uedı.) FJIIK**						
No Visible) esions	1	6	6	1	6	6
**PARATHYROID GLAND**						
E. amined	1	6	6	1	6	6
No Visible) esions	1	1	1	0	6	6
4EFIVASCD) AFUINgI) GFAGE; CE)) D) AFU MONONDC) EAF CE))	0	1	1	0	0	0
HHHHMI) ,	0	1	1	0	0	0
HHHHAveraPe Lrade	0	6	6	0	0	0
ECGO4IC GISSDE; GTXMDS	0	0	0	1	0	0
**PITUITARY GLAND**						
E. amined	1	6	6	1	6	6
No Visible) esions	1	6	1	0	6	6
4AFS , ISGA) ISUCXSG	0	0	1	0	0	0
4AFS , ISGA) ISUCXSGUMD) GI4) E	0	0	0	1	0	0
**PROSTATE**						
E. amined	1	6	6	H	H	H
No Visible) esions	1	6	6	H	H	H
**RECTUM**						
E. amined	1	6	6	1	6	6
No Visible) esions	1	6	6	1	6	6
**SALIVARY GLAND**						
E. amined	1	6	6	1	6	6
No Visible) esions	0	6	6	0	6	6
SDBMAN, IBD) AFUINgI) GFAGE; CE)) D) AFU) XM4TO4) ASMACXGIC	1	0	0	1	0	0
HHHHMI) ,	1	0	0	1	0	0
HHHHAveraPe Lrade	6	0	0	6	0	0
**SCIATIC NERVE**						
E. amined	1	6	6	1	6	6
No Visible) esions	1	6	6	1	6	1
) EgGU, ISGA) UINgI) GFAGE; CE)) D) AFU MONONDC) EAF CE))	0	0	0	0	0	1

10/30/6063 102 5210AM 4aPe25

AmplifyBio Study Number A-579-01

Incidence Summary of Microscopic Observations - 16 Week Cohort

Femoval FeasonRs(2A)) Summary2AveraPe Lrade RMean(	Male			gemale		
	Lroup 1 Control Gotal vP 0	Lroup 6) o^ Gotal vP w. 10x1 3	Lroup 3 TiPh Gotal vP 1I6. 10x 1:	Lroup 1 Control Gotal vP 0	Lroup 6) o^ Gotal vP w. 10x1 3	Lroup 3 TiPh Gotal vP 1I6. 10x 1:
Number of Animals2	1	6	6	1	6	6
Number of Completed Animals2	1	6	6	1	6	6
**SCIATIC NERVE tCi uedı.) FJIIK**						
HHHMI) ,	0	0	0	0	0	1
HHHAveraPe Lrade	0	0	0	0	0	6
**SEMINAL VESICLES**						
E. amined	1	6	6	H	H	H
No Visible) esions	1	6	6	H	H	H
**Sn ELETAL MUSCLE**						
E. amined	1	6	6	1	6	6
No Visible) esions	1	6	6	1	6	6
**Sn IN**						
E. amined	1	6	6	1	6	6
No Visible) esions	0	6	6	1	6	1
E4I, EFMISUEFOSIONUgOCA)	1	0	0	0	0	0
HHHMI) ,	1	0	0	0	0	0
HHHAveraPe Lrade	6	0	0	0	0	0
, EFMISUINg) AMMAGIONUNEDGFO4TI) IC	1	0	0	0	0	0
HHHMI) ,	1	0	0	0	0	0
HHHAveraPe Lrade	6	0	0	0	0	0
, EFMISU4EFIVASCD) AFUINg) AMMAGIONU 4) ASMACXGIC	1	0	0	0	0	0
HHHMI) ,	1	0	0	0	0	0
HHHAveraPe Lrade	6	0	0	0	0	0
D) CEF	0	0	0	0	0	1
, EFMISUgOFEILN BO, X	1	0	0	0	0	0
**SPINAL CORD**						
E. amined	1	6	6	1	6	6
No Visible) esions	1	1	1	1	1	6
CEFVICA) RNECZ(UAYONU, ELENEFAGIONU WA)) EFIAN	0	0	0	0	1	0
HHHMINIMA)	0	0	0	0	1	0
HHHAveraPe Lrade	0	0	0	0	1	0
) DMBAFUAYONU, ELENEFAGIONUWA)) EFIAN	0	1	0	0	1	0

10/30/6063 102 5210AM 4aPe2w

AmplifyBio Study Number A-579-01

Incidence Summary of Microscopic Observations - 16 Week Cohort

Femoval FeasonRs(2A)) Summary2AveraPe Lrade RMean(	Male			gemale		
	Lroup 1 Control Gotal vP 0	Lroup 6) o^ Gotal vP w. 10x1 3	Lroup 3 TiPh Gotal vP 1H6. 10x 1:	Lroup 1 Control Gotal vP 0	Lroup 6) o^ Gotal vP w. 10x1 3	Lroup 3 TiPh Gotal vP 1H6. 10x 1:
Number of Animals2	1	6	6	1	6	6
Number of Completed Animals2	1	6	6	1	6	6
**SPINAL CORD tCi uedı.) FJJIK**						
HHHHMINIMA)	0	1	0	0	1	0
HHHHAveraPe Lrade	0	1	0	0	1	0
GTOFACICUAYONU, ELENEFAGIONUWA)) EFIAN	0	0	1	0	1	0
HHHHMINIMA)	0	0	1	0	1	0
HHHHAveraPe Lrade	0	0	1	0	1	0
**SPLEEN**						
E. amined	1	6	6	1	6	6
No Visible) esions	1	1	6	1	6	6
) XM4T gO)) IC) EUAMX) OI, , E4OSIGION	0	1	0	0	0	0
HHHHMINIMA)	0	1	0	0	0	0
HHHHAveraPe Lrade	0	1	0	0	0	0
**STOMACH**						
E. amined	1	6	6	1	6	6
No Visible) esions	1	6	6	1	6	6
**TESTES**						
E. amined	1	6	6	H	H	H
No Visible) esions	1	6	6	H	H	H
**THYMUS**						
E. amined	1	6	6	1	6	6
No Visible) esions	1	6	1	1	1	6
COFGEYU) XM4TOI, AGFO4TX	0	0	1	0	1	0
HHHHMINIMA)	0	0	1	0	1	0
HHHHAveraPe Lrade	0	0	1	0	1	0
**THYROID GLANDS**						
E. amined	1	6	6	1	6	6
No Visible) esions	1	1	6	0	6	1
INCFEASE, CE)) D) AFIGXUMONONDC) EAF CE))	0	1	0	0	0	0
HHHHMI) ,	0	1	0	0	0	0
HHHHAveraPe Lrade	0	6	0	0	0	0
ECGO4IC GISSDE; GTXMDS	0	0	0	1	0	1

10/30/6063 102 5210AM 4aPe27

AmplifyBio Study Number A-579-01

Incidence Summary of Microscopic Observations - 16 Week Cohort

Femoval FeasonRs(2A)) Summary2AveraPe Lrade RMean(	Male			gemale		
	Lroup 1 Control Gotal vP 0	Lroup 6) o^ Gotal vP w. 10x1 3	Lroup 3 TiPh Gotal vP 1H6. 10x 1:	Lroup 1 Control Gotal vP 0	Lroup 6) o^ Gotal vP w. 10x1 3	Lroup 3 TiPh Gotal vP 1H6. 10x 1:
Number of Animals2	1	6	6	1	6	6
Number of Completed Animals2	1	6	6	1	6	6
**TRACHEA**						
E. amined	1	6	6	1	6	6
No Visible) esions	1	6	6	1	6	6
**URINARY BLADDER**						
E. amined	1	6	6	1	6	6
No Visible) esions	1	6	6	1	1	6
MDCOSAUINg) AMMAGIONUMONONDC) EAF CE))	0	0	0	0	1	0
HHHHMI) ,	0	0	0	0	1	0
HHHHAveraPe Lrade	0	0	0	0	6	0
**UTERUS**						
E. amined	H	H	H	1	6	6
No Visible) esions	H	H	H	1	6	6
**VAGINA**						
E. amined	H	H	H	1	6	6
No Visible) esions	H	H	H	1	6	6
**SPINAL ROOTS**						
E. amined	1	6	6	1	6	6
No Visible) esions	1	6	6	1	6	6
**GANGLION, DORSAL ROOT**						
E. amined	1	6	6	1	6	6
No Visible) esions	1	1	0	0	1	0
CEFVICA) RNECZ(UNEDFONUNECFOSIS	0	1	0	0	1	0
HHHHMINIMA)	0	1	0	0	1	0
HHHHAveraPe Lrade	0	1	0	0	1	0
) DMBAFUNEDFONUNECFOSIS	0	0	1	0	0	0
HHHHMINIMA)	0	0	1	0	0	0
HHHHAveraPe Lrade	0	0	1	0	0	0
GTOFACICUNEDFONUNECFOSIS	0	0	1	0	0	0
HHHHMINIMA)	0	0	1	0	0	0
HHHHAveraPe Lrade	0	0	1	0	0	0
CEFVICA) RNECZ(UINCFEASE, CE)) D) AFIGXU MONONDC) EAF CE))	0	0	0	0	1	0

10/30/6063 102 5210AM 4aPe2K

AmplifyBio Study Number A-579-01

Incidence Summary of Microscopic Observations - 16 Week Cohort

Femoval FeasonRs(2A))<br>Summary2AveraPe Lrade RMean(	Male			gemale		
	Lroup 1 Control Gotal vP 0	Lroup 6) o^ Gotal vP w. 10x1 3	Lroup 3 TiPh Gotal vP 1H6. 10x 1:	Lroup 1 Control Gotal vP 0	Lroup 6) o^ Gotal vP w. 10x1 3	Lroup 3 TiPh Gotal vP 1H6. 10x 1:
Number of Animals2	1	6	6	1	6	6
Number of Completed Animals2	1	6	6	1	6	6
**GANGLION, DORSAL ROOT tCi uedı.) FJJIK**						
HHHHMI) ,	0	0	0	0	1	0
HHHHAveraPe Lrade	0	0	0	0	6	0
) DMBAFUINCFEASE, CE)) D) AFIGXU MONONDC) EAF CE))	0	0	0	0	1	1
HHHHMI) ,	0	0	0	0	1	1
HHHHAveraPe Lrade	0	0	0	0	6	6
CEFVICA) RNECZ(UNEDFONU, ELENEFAGION	0	1	1	0	1	0
HHHHMINIMA)	0	1	1	0	1	0
HHHHAveraPe Lrade	0	1	1	0	1	0
) DMBAFUNEDFONU, ELENEFAGION	0	1	0	0	1	0
HHHHMINIMA)	0	1	0	0	1	0
HHHHAveraPe Lrade	0	1	0	0	1	0
GTOFACICUNEDFONU, ELENEFAGION	0	0	1	0	0	1
HHHHMINIMA)	0	0	1	0	0	1
HHHHAveraPe Lrade	0	0	1	0	0	1
) DMBAFUADGO4TALX; NEDFON	0	0	0	0	0	1
HHHHMINIMA)	0	0	0	0	0	1
HHHHAveraPe Lrade	0	0	0	0	0	1
GTOFACICUADGO4TALX; NEDFON	0	0	0	1	0	0
HHHHMINIMA)	0	0	0	1	0	0
HHHHAveraPe Lrade	0	0	0	1	0	0
GTOFACICUNEDFONUVACDO) I8AGION CXGO4) ASM	0	0	1	0	0	0
HHHHMINIMA)	0	0	1	0	0	0
HHHHAveraPe Lrade	0	0	1	0	0	0

AmplifyBio Study Number A-579-01

Incidence Summary of Microscopic Observations - 16 Week Cohort

Zey 4aPe

R)pi re R)q.)se Ie)ms

Animals E. cluded2	151; 101; 651; 656; 601; 606; 351; 356; 301; 306
Lroups2	All
Observation Gype2	Tisto - Neoplastic and Non-Neoplastic
Gissues2	All
Femoval Feasons2	Fecovery Necropsy 1

M)as. r)m)ue/Seacdsedcs

Measurement	, escriptive	Comparative	Arithmetic /Adjusted	Gransformation
4atholoPy Observation	Mean			
4atholoPy Observation	Count 4ositives			

Gri . p Iufi rmaed u

Short Name	) onP Name	Gype	Feport TeadinPs			
1	Lroup 1	Control	Lroup 1	Control	Gotal vP	0
6	Lroup 6	, ose	Lroup 6	) o^	Gotal vP	w. 10x13
3	Lroup 3	, ose	Lroup 3	TiPh	Gotal vP	1I6. 10x1:

R)mi val R)asi u Gri . pdıg

LroupinP Name	Abbreviation	Femoval Feasons
Fecovery Necropsy 1	F1	Fecovery Necropsy 1

Attachment 3. Individual Animal Organ Weights and Weight Ratios

AmplifyBio Study Number A-579-01

Individual Animal Organ Weight & Weight Ratios

Sex: Male Day(s): -9999 → 9999 Relative to Start Date

Group 1 Control Total vg 0			Organ Weight									
	Day of Death	Terminal BW (kg)	Adrenal Glands Wt. (g)	Adrenal Glands:BR (%)	Adrenal Glands:TBW. (%)	Brain Weight (g)	Brain:TBW. (%)	Epididymis Weight (g)	Epididymis: BR (%)	Epididymis: TBW. (%)	Heart Weight (g)
101	43	2.41	0.3957	0.506	0.02	78.20	3.24	0.2068	0.264	0.01	12.4903
102	85	3.31	0.8953	1.355	0.03	66.05	2.00	1.7159	2.598	0.05	14.0013

AmplifyBio Study Number A-579-01

Individual Animal Organ Weight & Weight Ratios

Sex: Male Day(s): -9999 → 9999 Relative to Start Date

Group 1 Control Total vg 0	Organ Weight										
	Heart: BR (%)	Heart:TBW. (%)	Kidneys Weight (g)	Kidneys: BR (%)	Kidneys:TBW. (%)	Liver Weight (g)	Liver: BR (%)	Liver:TBW. (%)	Lungs Weight (g)	Lungs: BR (%)	Lungs:TBW. (%)
101	15.972	0.52	12.96	16.573	0.54	58.23	74.463	2.42	25.69	32.852	1.07
102	21.198	0.42	17.34	26.253	0.52	77.59	117.472	2.34	31.03	46.980	0.94

AmplifyBio Study Number A-579-01

Individual Animal Organ Weight & Weight Ratios

Sex: Male Day(s): -9999 → 9999 Relative to Start Date

Group 1 Control Total vg 0	Organ Weight										
	Pituitary Gland Weight (g)	Pituitary Gland: BR (%)	Pituitary Gland:TBW. (%)	Spleen Weight (g)	Spleen: BR (%)	Spleen:TBW. (%)	Testes Weight (g)	Testes: BR (%)	Testes:TBW. (%)	Thymus Weight (g)	Thymus: BR (%)
101	0.0277	0.035	0.00	1.8105	2.315	0.08	0.96	1.228	0.04	2.962	3.788
102	0.0618	0.094	0.00	3.9948	6.048	0.12	2.62 [a]	3.967	0.08	3.799 [b]	5.752

[a] [RC:actual weight: 2.6193]
[b] [RC:actual weight: 3.7985]

AmplifyBio Study Number A-579-01

Individual Animal Organ Weight & Weight Ratios

Sex: Male Day(s): -9999 → 9999 Relative to Start Date

Group 1 Control Total vg 0	Organ Weight			
	Thymus:TBW. (%)	Thyroid/ Parathyroid (g)	Thyroid/Para :BR (%)	Thyroid/Para :TBW. (%)
101	0.12	0.3340	0.427	0.01
102	0.11	0.3645	0.552	0.01

AmplifyBio Study Number A-579-01

Individual Animal Organ Weight & Weight Ratios

Sex: Male Day(s): -9999 → 9999 Relative to Start Date

Group 2 Low Total vg 6x10^13			Organ Weight								
	Day of Death	Terminal BW (kg)	Adrenal Glands Wt. (g)	Adrenal Glands:BR (%)	Adrenal Glands:TBW. (%)	Brain Weight (g)	Brain:TBW. (%)	Epididymis Weight (g)	Epididymis: BR (%)	Epididymis: TBW. (%)	Heart Weight (g)
201	43	2.44	0.4191	0.668	0.02	62.70	2.57	0.3469	0.553	0.01	15.8394
202	43	3.06	0.5209	0.647	0.02	80.49	2.63	0.7151	0.888	0.02	14.5600
203	85	2.97	0.4026	0.647	0.01	62.24	2.10	0.8398	1.349	0.03	11.0195
204	85	2.50	0.3882	0.564	0.02	68.87	2.75	0.4356	0.632	0.02	10.2243

10/30/2023 10:46:10AM Page: 6

AmplifyBio Study Number A-579-01

Individual Animal Organ Weight & Weight Ratios

Sex: Male Day(s): -9999 → 9999 Relative to Start Date

Group 2 Low Total vg 6x10^13	Organ Weight										
	Heart: BR (%)	Heart:TBW. (%)	Kidneys Weight (g)	Kidneys: BR (%)	Kidneys:TBW. (%)	Liver Weight (g)	Liver: BR (%)	Liver:TBW. (%)	Lungs Weight (g)	Lungs: BR (%)	Lungs:TBW. (%)
201	25.262	0.65	17.35	27.671	0.71	66.34	105.805	2.72	23.15	36.922	0.95
202	18.089	0.48	11.75	14.598	0.38	66.47	82.582	2.17	27.30	33.917	0.89
203	17.705	0.37	12.06	19.377	0.41	71.93	115.569	2.42	36.03	57.889	1.21
204	14.846	0.41	12.58	18.266	0.50	59.19	85.945	2.37	24.40	35.429	0.98

AmplifyBio Study Number A-579-01

Individual Animal Organ Weight & Weight Ratios

Sex: Male Day(s): -9999 → 9999 Relative to Start Date

Group 2 Low Total vg 6x10^13	Organ Weight										
	Pituitary Gland Weight (g)	Pituitary Gland: BR (%)	Pituitary Gland:TBW. (%)	Spleen Weight (g)	Spleen: BR (%)	Spleen:TBW. (%)	Testes Weight (g)	Testes: BR (%)	Testes:TBW. (%)	Thymus Weight (g)	Thymus: BR (%)
201	0.0321	0.051	0.00	1.7901	2.855	0.07	0.99	1.579	0.04	3.768	6.010
202	0.0396	0.049	0.00	3.0854	3.833	0.10	1.74	2.162	0.06	2.664	3.310
203	0.0452	0.073	0.00	3.3199	5.334	0.11	2.29 [a]	3.679	0.08	1.793 [c]	2.881
204	0.0458	0.067	0.00	2.8773	4.178	0.12	1.04 [b]	1.510	0.04	2.474	3.592

[a] [RC:Actual weight 2.2883g]
[b] [RC:Actual weight 1.0383g]
[c] [RC:Actual weight 1.7930g]

AmplifyBio Study Number A-579-01

Individual Animal Organ Weight & Weight Ratios

Sex: Male Day(s): -9999 → 9999 Relative to Start Date

Group 2 Low Total vg 6x10^13	Organ Weight			
	Thymus:TBW. (%)	Thyroid/ Parathyroid (g)	Thyroid/Para :BR (%)	Thyroid/Para :TBW. (%)
201	0.15	0.3959	0.631	0.02
202	0.09	0.5437	0.675	0.02
203	0.06	0.3235	0.520	0.01
204	0.10	0.4536 [a]	0.659	0.02

[a] [RC:Actual weight 2.4724g]

AmplifyBio Study Number A-579-01

Individual Animal Organ Weight & Weight Ratios

Sex: Male Day(s): -9999 → 9999 Relative to Start Date

Group 3 High Total vg 1.2x10^14			Organ Weight									
	Day of Death	Terminal BW (kg)	Adrenal Glands Wt. (g)	Adrenal Glands:BR (%)	Adrenal Glands:TBW. (%)	Brain Weight (g)	Brain:TBW. (%)	Epididymis Weight (g)	Epididymis: BR (%)	Epididymis: TBW. (%)	Heart Weight (g)
301	43	2.73	0.4957	0.736	0.02	67.31	2.47	0.7159	1.064	0.03	12.4127
302	43	2.43	0.6618	0.972	0.03	68.07	2.80	0.5773	0.848	0.02	11.9665
303	85	3.44	0.6166	0.812	0.02	75.98	2.21	1.1665	1.535	0.03	11.8923
304	85	2.62	0.4716	0.629	0.02	74.98	2.86	0.9744	1.300	0.04	12.7277

AmplifyBio Study Number A-579-01

Individual Animal Organ Weight & Weight Ratios

Sex: Male Day(s): -9999 → 9999 Relative to Start Date

Group 3 High Total vg 1.2x10^14	Organ Weight										
	Heart: BR (%)	Heart:TBW. (%)	Kidneys Weight (g)	Kidneys: BR (%)	Kidneys:TBW. (%)	Liver Weight (g)	Liver: BR (%)	Liver:TBW. (%)	Lungs Weight (g)	Lungs: BR (%)	Lungs:TBW. (%)
301	18.441	0.45	13.00	19.314	0.48	64.00	95.082	2.34	35.90	53.335	1.32
302	17.580	0.49	15.08	22.154	0.62	67.07	98.531	2.76	22.48	33.025	0.93
303	15.652	0.35	15.16	19.953	0.44	61.74	81.258	1.79	20.89	27.494	0.61
304	16.975	0.49	12.90	17.205	0.49	54.56	72.766	2.08	40.91	54.561	1.56

AmplifyBio Study Number A-579-01

Individual Animal Organ Weight & Weight Ratios

Sex: Male Day(s): -9999 → 9999 Relative to Start Date

Group 3 High Total vg 1.2x10^14	Organ Weight										
	Pituitary Gland Weight (g)	Pituitary Gland: BR (%)	Pituitary Gland:TBW. (%)	Spleen Weight (g)	Spleen: BR (%)	Spleen:TBW. (%)	Testes Weight (g)	Testes: BR (%)	Testes:TBW. (%)	Thymus Weight (g)	Thymus: BR (%)
301	0.0524	0.078	0.00	3.8894	5.778	0.14	1.49	2.214	0.05	4.034	5.993
302	0.0410	0.060	0.00	2.9518	4.336	0.12	1.05	1.543	0.04	2.931	4.306
303	0.0487	0.064	0.00	4.3526	5.729	0.13	1.66 [a]	2.185	0.05	3.683 [c]	4.847
304	0.0318	0.042	0.00	4.5875	6.118	0.18	0.33 [b]	0.440	0.01	0.416 [d]	0.555

[a] [RC:Actual weight 1.6626g]
[b] [RC:Actual weight 1.3275g]
[c] [RC:Actual weight 3.6831g]
[d] [RC:Actual weight 3.4163g]

AmplifyBio Study Number A-579-01

Individual Animal Organ Weight & Weight Ratios

Sex: Male Day(s): -9999 → 9999 Relative to Start Date

Group 3 High Total vg 1.2x10^14	Organ Weight			
	Thymus:TBW. (%)	Thyroid/ Parathyroid (g)	Thyroid/Para :BR (%)	Thyroid/Para :TBW. (%)
301	0.15	0.6235	0.926	0.02
302	0.12	0.5846	0.859	0.02
303	0.11	0.1487	0.196	0.00
304	0.02	0.4207	0.561	0.02

AmplifyBio Study Number A-579-01

Individual Animal Organ Weight & Weight Ratios

Sex: Female Day(s): -9999 → 9999 Relative to Start Date

Group 1 Control Total vg 0			Organ Weight								
	Day of Death	Terminal BW (kg)	Adrenal Glands Wt. (g)	Adrenal Glands:BR (%)	Adrenal Glands:TBW. (%)	Brain Weight (g)	Brain:TBW. (%)	Heart Weight (g)	Heart: BR (%)	Heart:TBW. (%)	Kidneys Weight (g)
151	43	2.75	0.6976	1.320	0.03	52.86	1.92	9.3089	17.610	0.34	16.32
152	85	5.39	0.8432	1.190	0.02	70.87	1.31	18.5018	26.107	0.34	12.31

10/30/2023 10:46:10AM Page: 14

AmplifyBio Study Number A-579-01

Individual Animal Organ Weight & Weight Ratios

Sex: Female Day(s): -9999 → 9999 Relative to Start Date

Group 1 Control Total vg 0	Organ Weight										
	Kidneys: BR (%)	Kidneys:TBW. (%)	Liver Weight (g)	Liver: BR (%)	Liver:TBW. (%)	Lungs Weight (g)	Lungs: BR (%)	Lungs:TBW. (%)	Ovaries Weight (g)	Ovaries: BR (%)	Ovaries:TBW. (%)
151	30.874	0.59	75.78	143.360	2.76	36.46	68.975	1.33	0.3428	0.649	0.01
152	17.370	0.23	97.64	137.773	1.81	28.00	39.509	0.52	0.3969	0.560	0.01

10/30/2023 10:46:10AM Page: 15

AmplifyBio Study Number A-579-01

Individual Animal Organ Weight & Weight Ratios

Sex: Female Day(s): -9999 → 9999 Relative to Start Date

Group 1 Control Total vg 0	Organ Weight										
	Pituitary Gland Weight (g)	Pituitary Gland: BR (%)	Pituitary Gland:TBW. (%)	Spleen Weight (g)	Spleen: BR (%)	Spleen:TBW. (%)	Thymus Weight (g)	Thymus: BR (%)	Thymus:TBW. (%)	Thyroid/ Parathyroid (g)	Thyroid/Para :BR (%)
151	0.0691	0.131	0.00	3.1961	6.046	0.12	4.513	8.538	0.16	0.3371	0.638
152	0.0443	0.063	0.00	5.3775	7.588	0.10	5.052 [a]	7.129	0.09	0.3968	0.560

[a] [RC:Actual weight 5.5021g]

AmplifyBio Study Number A-579-01

Individual Animal Organ Weight & Weight Ratios

Sex: Female Day(s): -9999 → 9999 Relative to Start Date

Group 1 Control Total vg 0	Organ Weight			
	Thyroid/Para :TBW. (%)	Uterus/ Cervix (g)	Uterus/ Cervix:BR (%)	Uterus/ Cervix:TBW. (%)
151	0.01	5.1556	9.753	0.19
152	0.01	15.6418	22.071	0.29

AmplifyBio Study Number A-579-01

Individual Animal Organ Weight & Weight Ratios

Sex: Female Day(s): -9999 → 9999 Relative to Start Date

Group 2 Low Total vg 6x10^13			Organ Weight									
	Day of Death	Terminal BW (kg)	Adrenal Glands Wt. (g)	Adrenal Glands:BR (%)	Adrenal Glands:TBW. (%)	Brain Weight (g)	Brain:TBW. (%)	Heart Weight (g)	Heart: BR (%)	Heart:TBW. (%)	Kidneys Weight (g)
251	43	3.12	0.7163	1.153	0.02	62.13	1.99	14.4180	23.206	0.46	14.95
252	43	2.69	0.4762	0.862	0.02	55.23	2.05	11.8516	21.459	0.44	14.61
253	85	3.51	0.7133	1.207	0.02	59.09	1.68	10.2367	17.324	0.29	15.50
254	85	2.70 [a]	0.4362	0.788	0.02	55.33	2.05	10.0959 [b]	18.247	0.37	11.35

[a] [RC:Transcribed from original data point inadvertently deleted with purge of path module data]
[b] [RC:original incorrect entry 0.0959g]

AmplifyBio Study Number A-579-01

Individual Animal Organ Weight & Weight Ratios

Sex: Female Day(s): -9999 → 9999 Relative to Start Date

Group 2 Low Total vg 6x10^13	Organ Weight										
	Kidneys: BR (%)	Kidneys:TBW. (%)	Liver Weight (g)	Liver: BR (%)	Liver:TBW. (%)	Lungs Weight (g)	Lungs: BR (%)	Lungs:TBW. (%)	Ovaries Weight (g)	Ovaries: BR (%)	Ovaries:TBW. (%)
251	24.062	0.48	76.11	122.501	2.44	32.66	52.567	1.05	0.2055	0.331	0.01
252	26.453	0.54	78.51	142.151	2.92	28.58	51.747	1.06	0.3053	0.553	0.01
253	26.231	0.44	83.22	140.836	2.37	31.30	52.970	0.89	0.2727	0.461	0.01
254	20.513	0.42	62.20	112.416	2.30	41.87	75.673	1.55	0.4062	0.734	0.02

AmplifyBio Study Number A-579-01

Individual Animal Organ Weight & Weight Ratios

Sex: Female Day(s): -9999 → 9999 Relative to Start Date

Group 2 Low Total vg 6x10^13	Organ Weight										
	Pituitary Gland Weight (g)	Pituitary Gland: BR (%)	Pituitary Gland:TBW. (%)	Spleen Weight (g)	Spleen: BR (%)	Spleen:TBW. (%)	Thymus Weight (g)	Thymus: BR (%)	Thymus:TBW. (%)	Thyroid/ Parathyroid (g)	Thyroid/Para :BR (%)
251	0.0335	0.054	0.00	3.2814	5.282	0.11	3.032	4.880	0.10	0.3225	0.519
252	0.0320	0.058	0.00	3.7092	6.716	0.14	2.220	4.020	0.08	0.6206	1.124
253	0.0682	0.115	0.00	2.9891	5.059	0.09	1.249 [a]	2.114	0.04	0.3084	0.522
254	0.0604	0.109	0.00	1.9618	3.546	0.07	1.542 [b]	2.787	0.06	0.3136	0.567

[a] [RC:Actual weight 1.2494g]
[b] [RC:original incorrect entry 0.541g]

AmplifyBio Study Number A-579-01

Individual Animal Organ Weight & Weight Ratios

Sex: Female Day(s): -9999 → 9999 Relative to Start Date

Group 2 Low Total vg 6x10^13	Organ Weight			
	Thyroid/Para :TBW. (%)	Uterus/ Cervix (g)	Uterus/ Cervix:BR (%)	Uterus/ Cervix:TBW. (%)
251	0.01	2.5236	4.062	0.08
252	0.02	9.1057	16.487	0.34
253	0.01	5.1515	8.718	0.15
254	0.01	8.6399	15.615	0.32

AmplifyBio Study Number A-579-01

Individual Animal Organ Weight & Weight Ratios

Sex: Female Day(s): -9999 → 9999 Relative to Start Date

Group 3 High Total vg 1.2x10^14			Organ Weight									
	Day of Death	Terminal BW (kg)	Adrenal Glands Wt. (g)	Adrenal Glands:BR (%)	Adrenal Glands:TBW. (%)	Brain Weight (g)	Brain:TBW. (%)	Heart Weight (g)	Heart: BR (%)	Heart:TBW. (%)	Kidneys Weight (g)
351	43	2.39	0.4195	0.671	0.02	62.51	2.62	10.9024	17.441	0.46	11.91
352	43	2.87	0.5156	0.765	0.02	67.42	2.35	10.7123	15.889	0.37	16.81
353	85	2.82	0.7916	1.288	0.03	61.47	2.18	11.1221	18.094	0.39	14.43
354	85	2.88	0.7849	1.066	0.03	73.66	2.56	13.9208	18.899	0.48	17.50

AmplifyBio Study Number A-579-01

Individual Animal Organ Weight & Weight Ratios

Sex: Female Day(s): -9999 → 9999 Relative to Start Date

Group 3 High Total vg 1.2x10^14	Organ Weight										
	Kidneys: BR (%)	Kidneys:TBW. (%)	Liver Weight (g)	Liver: BR (%)	Liver:TBW. (%)	Lungs Weight (g)	Lungs: BR (%)	Lungs:TBW. (%)	Ovaries Weight (g)	Ovaries: BR (%)	Ovaries:TBW. (%)
351	19.053	0.50	67.09	107.327	2.81	23.29	37.258	0.97	0.3599	0.576	0.02
352	24.933	0.59	73.58	109.137	2.56	29.27	43.414	1.02	0.3643	0.540	0.01
353	23.475	0.51	70.35	114.446	2.49	30.16	49.065	1.07	0.2969	0.483	0.01
354	23.758	0.61	106.30	144.312	3.69	29.63	40.225	1.03	0.5532	0.751	0.02

AmplifyBio Study Number A-579-01

Individual Animal Organ Weight & Weight Ratios

Sex: Female Day(s): -9999 → 9999 Relative to Start Date

Group 3 High Total vg 1.2x10^14	Organ Weight										
	Pituitary Gland Weight (g)	Pituitary Gland: BR (%)	Pituitary Gland:TBW. (%)	Spleen Weight (g)	Spleen: BR (%)	Spleen:TBW. (%)	Thymus Weight (g)	Thymus: BR (%)	Thymus:TBW. (%)	Thyroid/ Parathyroid (g)	Thyroid/Para :BR (%)
351	0.0550	0.088	0.00	2.0516	3.282	0.09	2.355	3.767	0.10	0.1719	0.275
352	0.0596	0.088	0.00	2.4764	3.673	0.09	1.768	2.622	0.06	0.2615	0.388
353	0.0595	0.097	0.00	2.2819	3.712	0.08	1.380 [a]	2.245	0.05	0.5013	0.816
354	0.0501	0.068	0.00	5.6639	7.689	0.20	2.252	3.057	0.08	0.4862	0.660

[a] [RC:Actual weight 1.3798g]

AmplifyBio Study Number A-579-01

Individual Animal Organ Weight & Weight Ratios

Sex: Female Day(s): -9999 → 9999 Relative to Start Date

Group 3 High Total vg 1.2x10^14	Organ Weight			
	Thyroid/Para :TBW. (%)	Uterus/ Cervix (g)	Uterus/ Cervix:BR (%)	Uterus/ Cervix:TBW. (%)
351	0.01	3.5890	5.741	0.15
352	0.01	6.2268	9.236	0.22
353	0.02	9.3591	15.225	0.33
354	0.02	11.4224	15.507	0.40

AmplifyBio Study Number A-579-01

Individual Animal Organ Weight & Weight Ratios

Comments and Markers

Page	Day	Group	Sex	Subject	Measurement	Type	Marker
3	-9999 - 9999	1	Male	102	Testes	Result	
			Comment:	actual weight: 2.6193			
3	-9999 - 9999	1	Male	102	Thymus	Result	
			Comment:	actual weight: 3.7985			
7	-9999 - 9999	2	Male	203	Testes	Result	
			Comment:	Actual weight 2.2883g			
7	-9999 - 9999	2	Male	203	Thymus	Result	
			Comment:	Actual weight 1.7930g			
7	-9999 - 9999	2	Male	204	Testes	Result	
			Comment:	Actual weight 1.0383g			
8	-9999 - 9999	2	Male	204	Thyroid/ Parathyroid	Result	
			Comment:	Actual weight 2.4724g			
11	-9999 - 9999	3	Male	303	Testes	Result	
			Comment:	Actual weight 1.6626g			
11	-9999 - 9999	3	Male	303	Thymus	Result	
			Comment:	Actual weight 3.6831g			
11	-9999 - 9999	3	Male	304	Testes	Result	
			Comment:	Actual weight 1.3275g			
11	-9999 - 9999	3	Male	304	Thymus	Result	
			Comment:	Actual weight 3.4163g			
15	-9999 - 9999	1	Female	152	Thymus	Result	
			Comment:	Actual weight 5.5021g			
17	-9999 - 9999	2	Female	254	Terminal BW	Result	
			Comment:	Transcribed from original data point inadvertently deleted with purge of path module data			
17	-9999 - 9999	2	Female	254	Heart	Result	
			Comment:	original incorrect entry 0.0959g			
19	-9999 - 9999	2	Female	253	Thymus	Result	
			Comment:	Actual weight 1.2494g			

AmplifyBio Study Number A-579-01

Individual Animal Organ Weight & Weight Ratios

Comments and Markers

Page	Day	Group	Sex	Subject	Measurement	Type	Marker
19	-9999 - 9999	2	Female	254	Thymus	Result	
			Comment:	original incorrect entry 0.541g			
23	-9999 - 9999	3	Female	353	Thymus	Result	
			Comment:	Actual weight 1.3798g			

AmplifyBio Study Number A-579-01

Individual Animal Organ Weight & Weight Ratios

Key Page

Measurement Descriptions

Headings Used	Description
Day of Death	Day of Death
Terminal BW	Terminal Body Weight
Organ Weight - Adrenal Glands Wt.	Adrenal Glands
Organ Weight - Adrenal Glands:BR	Adrenal Glands:BR
Organ Weight - Adrenal Glands:TBW.	Adrenal Glands:TBW(kg)
Organ Weight - Brain Weight	Brain
Organ Weight - Brain:TBW.	Brain:TBW(kg)
Organ Weight - Epididymis Weight	Epididymis
Organ Weight - Epididymis: BR	Epididymis:BR
Organ Weight - Epididymis: TBW.	Epididymis:TBW(kg)
Organ Weight - Heart Weight	Heart
Organ Weight - Heart: BR	Heart:BR
Organ Weight - Heart:TBW.	Heart:TBW(kg)
Organ Weight - Kidneys Weight	Kidneys
Organ Weight - Kidneys: BR	Kidneys:BR
Organ Weight - Kidneys:TBW.	Kidneys:TBW(kg)
Organ Weight - Liver Weight	Liver
Organ Weight - Liver: BR	Liver:BR
Organ Weight - Liver:TBW.	Liver:TBW(kg)
Organ Weight - Lungs Weight	Lungs
Organ Weight - Lungs: BR	Lungs:BR
Organ Weight - Lungs:TBW.	Lungs:TBW(kg)
Organ Weight - Ovaries Weight	Ovaries
Organ Weight - Ovaries: BR	Ovaries:BR
Organ Weight - Ovaries:TBW.	Ovaries:TBW(kg)
Organ Weight - Pituitary Gland Weight	Pituitary Gland
Organ Weight - Pituitary Gland: BR	Pituitary Gland:BR

AmplifyBio Study Number A-579-01

Individual Animal Organ Weight & Weight Ratios

Key Page

Measurement Descriptions (Continued)

Headings Used	Description
Organ Weight - Pituitary Gland:TBW.	Pituitary Gland:TBW(kg)
Organ Weight - Spleen Weight	Spleen
Organ Weight - Spleen: BR	Spleen:BR
Organ Weight - Spleen:TBW.	Spleen:TBW(kg)
Organ Weight - Testes Weight	Testes
Organ Weight - Testes: BR	Testes:BR
Organ Weight - Testes:TBW.	Testes:TBW(kg)
Organ Weight - Thymus Weight	Thymus
Organ Weight - Thymus: BR	Thymus:BR
Organ Weight - Thymus:TBW.	Thymus:TBW(kg)
Organ Weight - Thyroid/ Parathyroid	Thyroid/Parathyroid
Organ Weight - Thyroid/Para :BR	Thyroid/Parathyroid:BR
Organ Weight - Thyroid/Para :TBW.	Thyroid/Parathyroid:TBW(kg)
Organ Weight - Uterus/ Cervix	Uterus/Cervix
Organ Weight - Uterus/ Cervix:BR	Uterus/Cervix:BR
Organ Weight - Uterus/ Cervix:TBW.	Uterus/Cervix:TBW(kg)

Unit Descriptions

Headings Used	Description
%	%
g	g
kg	kg

AmplifyBio Study Number A-579-01

Individual Animal Organ Weight & Weight Ratios

Key Page

Group Information

Short Name	Long Name	Type	Report Headings 1-4			
1	Group 1	Control	Group 1	Control	Total vg	0
2	Group 2	Dose	Group 2	Low	Total vg	6x10^13
3	Group 3	Dose	Group 3	High	Total vg	1.2x10^14

Comment Abbreviations

RC = Result Comment

Attachment 4. Individual Animal Gross and Microscopic Observations

10/30/2023 10:47:43AM Page: 1

AmplifyBio Study Number A-579-01

Individual Animal Gross & Microscopic Observations

Animal:	101	Group:	1	Sex:	Male
Species:	Nonhuman Primate	Strain:	Cynomolgus Monkey		
Study Type:	Subchronic (>14 d but < 6 mo) - Other				
		Dose:	Group 1 Control Total vg 0		
Death Date:	8/1/2023	Removal Reason:	Core Necropsy		
Necropsy Date:	8/1/2023	Study Day (Week) of Death:	43 (7)		
				Gross Status:	Complete
				Histo Status:	Complete

Terminal Bodyweight: 2.41kg

Organ Weights:

Adrenal Glands:	0.3957g	Brain:	78.20g	Epididymis:	0.2068g
Heart:	12.4903g	Kidneys:	12.96g	Liver:	58.23g
Lungs:	25.69g	Pituitary Gland:	0.0277g	Spleen:	1.8105g
Testes:	0.96g	Thymus:	2.962g	Thyroid/Parathyroid:	0.3340g

Gross Pathology Observations [Correlation]:

Gross Pathology - The following Tissues had No Visible Lesions:

ADRENAL GLANDS; AORTA; BONE MARROW; BRAIN; CECUM; COLON; DUODENUM; EPIDIDYMIS; ESOPHAGUS; EYES; FEMUR; GALLBLADDER; HEART; ILEUM; JEJUNUM; KIDNEYS; LIVER; LUNGS; LYMPH NODES, MANDIBULAR; LYMPH NODES, MESENTERIC; MAMMARY GLAND; OPTIC NERVE; PANCREAS; PARATHYROID GLAND; PITUITARY GLAND; PROSTATE; RECTUM; SALIVARY GLAND; SCIATIC NERVE; SEMINAL VESICLES; SKELETAL MUSCLE; SKIN; SPINAL CORD; SPLEEN; STOMACH; TESTES; THYMUS; THYROID GLANDS; TRACHEA; URINARY BLADDER; FEMORAL NERVE; NERVE, MEDIAN; ULNAR NERVE; SPINAL ROOTS; GANGLION, DORSAL ROOT

Histopathology Observations [Correlation]:

ADRENAL GLANDS : CORTEX; MINERALIZATION; MILD
HEART : INFILTRATE, CELLULAR; MONONUCLEAR CELL, MINIMAL
LIVER : INFLAMMATION; EOSINOPHILIC, MILD

Histopathology - The following Tissues were Within Normal Limits:

AORTA; BONE MARROW; BRAIN; CECUM; COLON; DUODENUM; EPIDIDYMIS; ESOPHAGUS; EYES; FEMUR; GALLBLADDER; ILEUM; JEJUNUM; KIDNEYS; LUNGS; LYMPH NODES, MANDIBULAR; LYMPH NODES, MESENTERIC; MAMMARY GLAND; PANCREAS; PARATHYROID GLAND; PITUITARY GLAND; PROSTATE; RECTUM; SALIVARY GLAND; SCIATIC NERVE; SEMINAL VESICLES; SKELETAL MUSCLE; SKIN; SPINAL CORD; SPLEEN; STOMACH; TESTES; THYMUS; THYROID GLANDS; TRACHEA; URINARY BLADDER; SPINAL ROOTS; GANGLION, DORSAL ROOT

10/30/2023 10:47:43AM Page: 2

AmplifyBio Study Number A-579-01

Individual Animal Gross & Microscopic Observations

Animal:	102	Group:	1	Sex:	Male
Species:	Nonhuman Primate	Strain:	Cynomolgus Monkey		
Study Type:	Subchronic (>14 d but < 6 mo) - Other				
		Dose:	Group 1 Control Total vg 0		
Death Date:	9/12/2023	Removal Reason:	Recovery Necropsy 1		
Necropsy Date:	9/12/2023	Study Day (Week) of Death:	85 (13)		
				Gross Status:	Complete
				Histo Status:	Complete

Terminal Bodyweight: 3.31kg

Organ Weights:

Adrenal Glands:	0.8953g	Brain:	66.05g	Epididymis:	1.7159g
Heart:	14.0013g	Kidneys:	17.34g	Liver:	77.59g
Lungs:	31.03g	Pituitary Gland:	0.0618g	Spleen:	3.9948g
Testes:	2.62g	Thymus:	3.799g	Thyroid/Parathyroid:	0.3645g

Gross Pathology Observations [Correlation]:

SKIN : RIGHT; FORELIMB; CRUST(S); TGL1 : (Comment) 7x4mm, on 4th digit, collected histo and 3 frozen vials (TGL)

Gross Pathology - The following Tissues had No Visible Lesions:

ADRENAL GLANDS; AORTA; BONE MARROW; BRAIN; CECUM; COLON; DUODENUM; EPIDIDYMIS; ESOPHAGUS; EYES; FEMUR; GALLBLADDER; HEART; ILEUM; JEJUNUM; KIDNEYS; LIVER; LUNGS; LYMPH NODES, MANDIBULAR; LYMPH NODES, MESENTERIC; MAMMARY GLAND; OPTIC NERVE; PANCREAS; PARATHYROID GLAND; PITUITARY GLAND; PROSTATE; RECTUM; SALIVARY GLAND; SCIATIC NERVE; SEMINAL VESICLES; SKELETAL MUSCLE; SPINAL CORD; SPLEEN; STOMACH; TESTES; THYMUS; THYROID GLANDS; TRACHEA; URINARY BLADDER; FEMORAL NERVE; NERVE, MEDIAN; ULNAR NERVE; SPINAL ROOTS; GANGLION, DORSAL ROOT

Histopathology Observations [Correlation]:

SALIVARY GLAND : SUBMANDIBULAR; INFILTRATE, CELLULAR; LYMPHOPLASMACYTIC, MILD

SKIN : EPIDERMIS; EROSION; FOCAL, MILD [SKIN : RIGHT; FORELIMB; CRUST(S); TGL1 : (Comment) 7x4mm, on 4th digit, collected histo and 3 frozen vials (G)]

SKIN : DERMIS; INFLAMMATION; NEUTROPHILIC, MILD

SKIN : DERMIS; PERIVASCULAR; INFLAMMATION; PLASMACYTIC, MILD

SKIN : DERMIS; FOREIGN BODY : (Comment) hair shaft

Histopathology - The following Tissues were Within Normal Limits:

ADRENAL GLANDS; AORTA; BONE MARROW; BRAIN; CECUM; COLON; DUODENUM; EPIDIDYMIS; ESOPHAGUS; EYES; FEMUR; GALLBLADDER; HEART; ILEUM; JEJUNUM; KIDNEYS; LIVER; LUNGS; LYMPH NODES, MANDIBULAR; LYMPH NODES, MESENTERIC; MAMMARY GLAND; PANCREAS; PARATHYROID GLAND; PITUITARY GLAND; PROSTATE; RECTUM; SCIATIC NERVE; SEMINAL VESICLES; SKELETAL MUSCLE; SPINAL CORD; SPLEEN; STOMACH; TESTES; THYMUS; THYROID GLANDS; TRACHEA; URINARY BLADDER; SPINAL ROOTS; GANGLION, DORSAL ROOT

AmplifyBio Study Number A-579-01

Individual Animal Gross & Microscopic Observations

Animal:	151	Group:	1	Sex:	Female
Species:	Nonhuman Primate	Strain:	Cynomolgus Monkey		
Study Type:	Subchronic (>14 d but < 6 mo) - Other				
		Dose:	Group 1 Control Total vg 0		
Death Date:	8/1/2023	Removal Reason:	Core Necropsy		
Necropsy Date:	8/1/2023	Study Day (Week) of Death:	43 (7)		
				Gross Status:	Complete
				Histo Status:	Complete

Terminal Bodyweight: 2.75kg

Organ Weights:

Adrenal Glands:	0.6976g	Brain:	52.86g	Heart:	9.3089g
Kidneys:	16.32g	Liver:	75.78g	Lungs:	36.46g
Ovaries:	0.3428g	Pituitary Gland:	0.0691g	Spleen:	3.1961g
Thymus:	4.513g	Thyroid/Parathyroid:	0.3371g	Uterus/Cervix:	5.1556g

Gross Pathology Observations [Correlation]:

Gross Pathology - The following Tissues had No Visible Lesions:

ADRENAL GLANDS; AORTA; BONE MARROW; BRAIN; CECUM; CERVIX; COLON; DUODENUM; ESOPHAGUS; EYES; FEMUR; GALLBLADDER; HEART; ILEUM; JEJUNUM; KIDNEYS; LIVER; LUNGS; LYMPH NODES, MANDIBULAR; LYMPH NODES, MESENTERIC; MAMMARY GLAND; OPTIC NERVE; OVARIES; PANCREAS; PARATHYROID GLAND; PITUITARY GLAND; RECTUM; SALIVARY GLAND; SCIATIC NERVE; SKELETAL MUSCLE; SKIN; SPINAL CORD; SPLEEN; STOMACH; THYMUS; THYROID GLANDS; TRACHEA; URINARY BLADDER; UTERUS; VAGINA; FEMORAL NERVE; NERVE, MEDIAN; ULNAR NERVE; SPINAL ROOTS; GANGLION, DORSAL ROOT

Histopathology Observations [Correlation]:

SALIVARY GLAND : SUBMANDIBULAR; INFILTRATE, CELLULAR; LYMPHOPLASMACYTIC, MILD

Histopathology - The following Tissues were Within Normal Limits:

ADRENAL GLANDS; AORTA; BONE MARROW; BRAIN; CECUM; CERVIX; COLON; DUODENUM; ESOPHAGUS; EYES; FEMUR; GALLBLADDER; HEART; ILEUM; JEJUNUM; KIDNEYS; LIVER; LUNGS; LYMPH NODES, MANDIBULAR; LYMPH NODES, MESENTERIC; MAMMARY GLAND; OVARIES; PANCREAS; PARATHYROID GLAND; PITUITARY GLAND; RECTUM; SCIATIC NERVE; SKELETAL MUSCLE; SKIN; SPINAL CORD; SPLEEN; STOMACH; THYMUS; THYROID GLANDS; TRACHEA; URINARY BLADDER; UTERUS; VAGINA; SPINAL ROOTS; GANGLION, DORSAL ROOT

10/30/2023 10:47:43AM Page: 4

AmplifyBio Study Number A-579-01

Individual Animal Gross & Microscopic Observations

Animal:	152	Group:	1	Sex:	Female
Species:	Nonhuman Primate	Strain:	Cynomolgus Monkey		
Study Type:	Subchronic (>14 d but < 6 mo) - Other				
		Dose:	Group 1 Control Total vg 0		
Death Date:	9/12/2023	Removal Reason:	Recovery Necropsy 1		
Necropsy Date:	9/12/2023	Study Day (Week) of Death:	85 (13)		
				Gross Status:	Complete
				Histo Status:	Complete

Terminal Bodyweight: 5.39kg

Organ Weights:

Adrenal Glands:	0.8432g	Brain:	70.87g	Heart:	18.5018g
Kidneys:	12.31g	Liver:	97.64g	Lungs:	28.00g
Ovaries:	0.3969g	Pituitary Gland:	0.0443g	Spleen:	5.3775g
Thymus:	5.052g	Thyroid/Parathyroid:	0.3968g	Uterus/Cervix:	15.6418g

Gross Pathology Observations [Correlation]:

BRAIN : UNILATERAL; LEFT; DILATATION; TGL1 : (Comment) 19x10x1mm (TGL)

Gross Pathology - The following Tissues had No Visible Lesions:

ADRENAL GLANDS; AORTA; BONE MARROW; CECUM; CERVIX; COLON; DUODENUM; ESOPHAGUS; EYES; FEMUR; GALLBLADDER; HEART; ILEUM; JEJUNUM; KIDNEYS; LIVER; LUNGS; LYMPH NODES, MANDIBULAR; LYMPH NODES, MESENTERIC; MAMMARY GLAND; OPTIC NERVE; OVARIES; PANCREAS; PARATHYROID GLAND; PITUITARY GLAND; RECTUM; SALIVARY GLAND; SCIATIC NERVE; SKELETAL MUSCLE; SKIN; SPINAL CORD; SPLEEN; STOMACH; THYMUS; THYROID GLANDS; TRACHEA; URINARY BLADDER; UTERUS; VAGINA; FEMORAL NERVE; NERVE, MEDIAN; ULNAR NERVE; SPINAL ROOTS; GANGLION, DORSAL ROOT

Histopathology Observations [Correlation]:

PARATHYROID GLAND : ECTOPIC TISSUE, THYMUS

PITUITARY GLAND : PARS DISTALIS; CYST; MULTIPLE

SALIVARY GLAND : SUBMANDIBULAR; INFILTRATE, CELLULAR; LYMPHOPLASMACYTIC, MILD

THYROID GLANDS : ECTOPIC TISSUE, THYMUS

NO CORRELATE : NO CORRELATE [BRAIN : UNILATERAL; LEFT; DILATATION; TGL1 : (Comment) 19x10x1mm (G)]

GANGLION, DORSAL ROOT : THORACIC; AUTOPHAGY, NEURON; MINIMAL

Histopathology - The following Tissues were Within Normal Limits:

ADRENAL GLANDS; AORTA; BONE MARROW; BRAIN; CECUM; CERVIX; COLON; DUODENUM; ESOPHAGUS; EYES; FEMUR; GALLBLADDER; HEART; ILEUM; JEJUNUM; KIDNEYS; LIVER; LUNGS; LYMPH NODES, MANDIBULAR; LYMPH NODES, MESENTERIC; MAMMARY GLAND; OVARIES; PANCREAS; RECTUM; SCIATIC NERVE; SKELETAL MUSCLE; SKIN; SPINAL CORD; SPLEEN; STOMACH; THYMUS; TRACHEA; URINARY BLADDER; UTERUS; VAGINA; SPINAL ROOTS

10/30/2023 10:47:43AM Page: 5

AmplifyBio Study Number A-579-01

Individual Animal Gross & Microscopic Observations

Animal:	201	Group:	2	Sex:	Male
Species:	Nonhuman Primate	Strain:	Cynomolgus Monkey		
Study Type:	Subchronic (>14 d but < 6 mo) - Other				
		Dose:	Group 2 Low Total vg 6x10^13		
Death Date:	8/2/2023	Removal Reason:	Core Necropsy		
Necropsy Date:	8/2/2023	Study Day (Week) of Death:	43 (7)		
				Gross Status:	Complete
				Histo Status:	Complete

Terminal Bodyweight: 2.44kg

Organ Weights:

Adrenal Glands:	0.4191g	Brain:	62.70g	Epididymis:	0.3469g
Heart:	15.8394g	Kidneys:	17.35g	Liver:	66.34g
Lungs:	23.15g	Pituitary Gland:	0.0321g	Spleen:	1.7901g
Testes:	0.99g	Thymus:	3.768g	Thyroid/Parathyroid:	0.3959g

Gross Pathology Observations [Correlation]:

Gross Pathology - The following Tissues had No Visible Lesions:

ADRENAL GLANDS; AORTA; BONE MARROW; BRAIN; CECUM; COLON; DUODENUM; EPIDIDYMIS; ESOPHAGUS; EYES; FEMUR; GALLBLADDER; HEART; ILEUM; JEJUNUM; KIDNEYS; LIVER; LUNGS; LYMPH NODES, MANDIBULAR; LYMPH NODES, MESENTERIC; MAMMARY GLAND; OPTIC NERVE; PANCREAS; PARATHYROID GLAND; PITUITARY GLAND; PROSTATE; RECTUM; SALIVARY GLAND; SCIATIC NERVE; SEMINAL VESICLES; SKELETAL MUSCLE; SKIN; SPINAL CORD; SPLEEN; STOMACH; TESTES; THYMUS; THYROID GLANDS; TRACHEA; URINARY BLADDER; FEMORAL NERVE; NERVE, MEDIAN; ULNAR NERVE; SPINAL ROOTS; GANGLION, DORSAL ROOT

Histopathology Observations [Correlation]:

ADRENAL GLANDS : MEDULLA; INFILTRATE, CELLULAR; MONONUCLEAR CELL, MILD
THYMUS : CORTEX; LYMPHOID ATROPHY; MINIMAL

Histopathology - The following Tissues were Within Normal Limits:

AORTA; BONE MARROW; BRAIN; CECUM; COLON; DUODENUM; EPIDIDYMIS; ESOPHAGUS; EYES; FEMUR; GALLBLADDER; HEART; ILEUM; JEJUNUM; KIDNEYS; LIVER; LUNGS; LYMPH NODES, MANDIBULAR; LYMPH NODES, MESENTERIC; MAMMARY GLAND; PANCREAS; PARATHYROID GLAND; PITUITARY GLAND; PROSTATE; RECTUM; SALIVARY GLAND; SCIATIC NERVE; SEMINAL VESICLES; SKELETAL MUSCLE; SKIN; SPINAL CORD; SPLEEN; STOMACH; TESTES; THYROID GLANDS; TRACHEA; URINARY BLADDER; SPINAL ROOTS; GANGLION, DORSAL ROOT

AmplifyBio Study Number A-579-01

Individual Animal Gross & Microscopic Observations

Animal:	202	Group:	2	Sex:	Male
Species:	Nonhuman Primate	Strain:	Cynomolgus Monkey		
Study Type:	Subchronic (>14 d but < 6 mo) - Other				
		Dose:	Group 2 Low Total vg 6x10^13		
Death Date:	8/2/2023	Removal Reason:	Core Necropsy		
Necropsy Date:	8/2/2023	Study Day (Week) of Death:	43 (7)		
				Gross Status:	Complete
				Histo Status:	Complete

Terminal Bodyweight: 3.06kg

Organ Weights:

Adrenal Glands:	0.5209g	Brain:	80.49g	Epididymis:	0.7151g
Heart:	14.5600g	Kidneys:	11.75g	Liver:	66.47g
Lungs:	27.30g	Pituitary Gland:	0.0396g	Spleen:	3.0854g
Testes:	1.74g	Thymus:	2.664g	Thyroid/Parathyroid:	0.5437g

Gross Pathology Observations [Correlation]:

Gross Pathology - The following Tissues had No Visible Lesions:

ADRENAL GLANDS; AORTA; BONE MARROW; BRAIN; CECUM; COLON; DUODENUM; EPIDIDYMIS; ESOPHAGUS; EYES; FEMUR; GALLBLADDER; HEART; ILEUM; JEJUNUM; KIDNEYS; LIVER; LUNGS; LYMPH NODES, MANDIBULAR; LYMPH NODES, MESENTERIC; MAMMARY GLAND; OPTIC NERVE; PANCREAS; PARATHYROID GLAND; PITUITARY GLAND; PROSTATE; RECTUM; SALIVARY GLAND; SCIATIC NERVE; SEMINAL VESICLES; SKELETAL MUSCLE; SKIN; SPINAL CORD; SPLEEN; STOMACH; TESTES; THYMUS; THYROID GLANDS; TRACHEA; URINARY BLADDER; FEMORAL NERVE; NERVE, MEDIAN; ULNAR NERVE; SPINAL ROOTS; GANGLION, DORSAL ROOT

Histopathology Observations [Correlation]:

LIVER : PORTAL; INFILTRATE, CELLULAR; EOSINOPHILIC, MILD

GANGLION, DORSAL ROOT : LUMBAR; INCREASED CELLULARITY; MONONUCLEAR CELL, MILD

GANGLION, DORSAL ROOT : THORACIC; INCREASED CELLULARITY; MONONUCLEAR CELL, MILD

GANGLION, DORSAL ROOT : LUMBAR; NEURON; DEGENERATION; MINIMAL

GANGLION, DORSAL ROOT : THORACIC; NEURON; DEGENERATION; MINIMAL

Histopathology - The following Tissues were Within Normal Limits:

ADRENAL GLANDS; AORTA; BONE MARROW; BRAIN; CECUM; COLON; DUODENUM; EPIDIDYMIS; ESOPHAGUS; EYES; FEMUR; GALLBLADDER; HEART; ILEUM; JEJUNUM; KIDNEYS; LUNGS; LYMPH NODES, MANDIBULAR; LYMPH NODES, MESENTERIC; MAMMARY GLAND; PANCREAS; PARATHYROID GLAND; PITUITARY GLAND; PROSTATE; RECTUM; SALIVARY GLAND; SCIATIC NERVE; SEMINAL VESICLES; SKELETAL MUSCLE; SKIN; SPINAL CORD; SPLEEN; STOMACH; TESTES; THYMUS; THYROID GLANDS; TRACHEA; URINARY BLADDER; SPINAL ROOTS

AmplifyBio Study Number A-579-01

Individual Animal Gross & Microscopic Observations

Animal:	203	Group:	2	Sex:	Male
Species:	Nonhuman Primate	Strain:	Cynomolgus Monkey		
Study Type:	Subchronic (>14 d but < 6 mo) - Other				
		Dose:	Group 2 Low Total vg 6x10^13		
Death Date:	9/13/2023	Removal Reason:	Recovery Necropsy 1		
Necropsy Date:	9/13/2023	Study Day (Week) of Death:	85 (13)		
				Gross Status:	Complete
				Histo Status:	Complete

Terminal Bodyweight: 2.97kg

Organ Weights:

Adrenal Glands:	0.4026g	Brain:	62.24g	Epididymis:	0.8398g
Heart:	11.0195g	Kidneys:	12.06g	Liver:	71.93g
Lungs:	36.03g	Pituitary Gland:	0.0452g	Spleen:	3.3199g
Testes:	2.29g	Thymus:	1.793g	Thyroid/Parathyroid:	0.3235g

Gross Pathology Observations [Correlation]:

MESENTERY : (Comment) Lining the mesentary fat of the GI, greater than 25 parasites seen visually, collected for Histology, not collected for frozen TGL processing per study director. One parasite found attached to the right liver lobe. Parasites are white, curled, up to 4x4x2mm.

Gross Pathology - The following Tissues had No Visible Lesions:

ADRENAL GLANDS; AORTA; BONE MARROW; BRAIN; CECUM; COLON; DUODENUM; EPIDIDYMIS; ESOPHAGUS; EYES; FEMUR; GALLBLADDER; HEART; ILEUM; JEJUNUM; KIDNEYS; LIVER; LUNGS; LYMPH NODES, MANDIBULAR; LYMPH NODES, MESENTERIC; MAMMARY GLAND; OPTIC NERVE; PANCREAS; PARATHYROID GLAND; PITUITARY GLAND; PROSTATE; RECTUM; SALIVARY GLAND; SCIATIC NERVE; SEMINAL VESICLES; SKELETAL MUSCLE; SKIN; SPINAL CORD; SPLEEN; STOMACH; TESTES; THYMUS; THYROID GLANDS; TRACHEA; URINARY BLADDER; FEMORAL NERVE; NERVE, MEDIAN; ULNAR NERVE; SPINAL ROOTS; GANGLION, DORSAL ROOT

Histopathology Observations [Correlation]:

Histopathology - The following Tissues were Within Normal Limits:

ADRENAL GLANDS; AORTA; BONE MARROW; BRAIN; CECUM; COLON; DUODENUM; EPIDIDYMIS; ESOPHAGUS; EYES; FEMUR; GALLBLADDER; HEART; ILEUM; JEJUNUM; KIDNEYS; LIVER; LUNGS; LYMPH NODES, MANDIBULAR; LYMPH NODES, MESENTERIC; MAMMARY GLAND; PANCREAS; PARATHYROID GLAND; PITUITARY GLAND; PROSTATE; RECTUM; SALIVARY GLAND; SCIATIC NERVE; SEMINAL VESICLES; SKELETAL MUSCLE; SKIN; SPINAL CORD; SPLEEN; STOMACH; TESTES; THYMUS; THYROID GLANDS; TRACHEA; URINARY BLADDER; SPINAL ROOTS; GANGLION, DORSAL ROOT

AmplifyBio Study Number A-579-01

Individual Animal Gross & Microscopic Observations

Animal:	204	Group:	2	Sex:	Male
Species:	Nonhuman Primate	Strain:	Cynomolgus Monkey		
Study Type:	Subchronic (>14 d but < 6 mo) - Other				
		Dose:	Group 2 Low Total vg 6x10^13		
Death Date:	9/13/2023	Removal Reason:	Recovery Necropsy 1		
Necropsy Date:	9/13/2023	Study Day (Week) of Death:	85 (13)		
				Gross Status:	Complete
				Histo Status:	Complete

Terminal Bodyweight: 2.50kg

Organ Weights:

Adrenal Glands:	0.3882g	Brain:	68.87g	Epididymis:	0.4356g
Heart:	10.2243g	Kidneys:	12.58g	Liver:	59.19g
Lungs:	24.40g	Pituitary Gland:	0.0458g	Spleen:	2.8773g
Testes:	1.04g	Thymus:	2.474g	Thyroid/Parathyroid:	0.4536g

Gross Pathology Observations [Correlation]:

Gross Pathology - The following Tissues had No Visible Lesions:

ADRENAL GLANDS; AORTA; BONE MARROW; BRAIN; CECUM; COLON; DUODENUM; EPIDIDYMIS; ESOPHAGUS; EYES; FEMUR; GALLBLADDER; HEART; ILEUM; JEJUNUM; KIDNEYS; LIVER; LUNGS; LYMPH NODES, MANDIBULAR; LYMPH NODES, MESENTERIC; MAMMARY GLAND; OPTIC NERVE; PANCREAS; PARATHYROID GLAND; PITUITARY GLAND; PROSTATE; RECTUM; SALIVARY GLAND; SCIATIC NERVE; SEMINAL VESICLES; SKELETAL MUSCLE; SKIN; SPINAL CORD; SPLEEN; STOMACH; TESTES; THYMUS; THYROID GLANDS; TRACHEA; URINARY BLADDER; FEMORAL NERVE; NERVE, MEDIAN; ULNAR NERVE; SPINAL ROOTS; GANGLION, DORSAL ROOT

Histopathology Observations [Correlation]:

PARATHYROID GLAND : PERIVASCULAR; INFILTRATE, CELLULAR; MONONUCLEAR CELL, MILD
SPINAL CORD : LUMBAR; AXON; DEGENERATION; WALLERIAN, MINIMAL
SPLEEN : LYMPH FOLLICLE; AMYLOID DEPOSITION; MINIMAL
THYROID GLANDS : INCREASED CELLULARITY; MONONUCLEAR CELL, MILD
GANGLION, DORSAL ROOT : CERVICAL (NECK); NEURON; NECROSIS; MINIMAL
GANGLION, DORSAL ROOT : CERVICAL (NECK); NEURON; DEGENERATION; MINIMAL
GANGLION, DORSAL ROOT : LUMBAR; NEURON; DEGENERATION; MINIMAL

Histopathology - The following Tissues were Within Normal Limits:

ADRENAL GLANDS; AORTA; BONE MARROW; BRAIN; CECUM; COLON; DUODENUM; EPIDIDYMIS; ESOPHAGUS; EYES; FEMUR; GALLBLADDER; HEART; ILEUM; JEJUNUM; KIDNEYS; LIVER; LUNGS; LYMPH NODES, MANDIBULAR; LYMPH NODES, MESENTERIC; MAMMARY GLAND; PANCREAS; PITUITARY GLAND; PROSTATE; RECTUM; SALIVARY GLAND; SCIATIC NERVE; SEMINAL VESICLES; SKELETAL MUSCLE; SKIN; STOMACH; TESTES; THYMUS; TRACHEA; URINARY BLADDER; SPINAL ROOTS

AmplifyBio Study Number A-579-01

Individual Animal Gross & Microscopic Observations

Animal: 251		Group: 2		Sex:	Female
Species: Nonhuman Primate		Strain: Cynomolgus Monkey			
Study Type: Subchronic (>14 d but < 6 mo) - Other					
		Dose: Group 2 Low Total vg 6x10^13			
Death Date:	8/2/2023	Removal Reason:	Core Necropsy		
Necropsy Date:	8/2/2023	Study Day (Week) of Death:	43 (7)		
				Gross Status:	Complete
				Histo Status:	Complete

Terminal Bodyweight: 3.12kg

Organ Weights:

Adrenal Glands:	0.7163g	Brain:	62.13g	Heart:	14.4180g
Kidneys:	14.95g	Liver:	76.11g	Lungs:	32.66g
Ovaries:	0.2055g	Pituitary Gland:	0.0335g	Spleen:	3.2814g
Thymus:	3.032g	Thyroid/Parathyroid:	0.3225g	Uterus/Cervix:	2.5236g

Gross Pathology Observations [Correlation]:

KIDNEYS : RIGHT; CYST(S); CLEAR, TGL1 : (Comment) 2x2x1mm, Histology section prioritized, no frozen sample taken. (TGL)

Gross Pathology - The following Tissues had No Visible Lesions:

ADRENAL GLANDS; AORTA; BONE MARROW; BRAIN; CECUM; CERVIX; COLON; DUODENUM; ESOPHAGUS; EYES; FEMUR; GALLBLADDER; HEART; ILEUM; JEJUNUM; LIVER; LUNGS; LYMPH NODES, MANDIBULAR; LYMPH NODES, MESENTERIC; MAMMARY GLAND; OPTIC NERVE; OVARIES; PANCREAS; PARATHYROID GLAND; PITUITARY GLAND; RECTUM; SALIVARY GLAND; SCIATIC NERVE; SKELETAL MUSCLE; SKIN; SPINAL CORD; SPLEEN; STOMACH; THYMUS; THYROID GLANDS; TRACHEA; URINARY BLADDER; UTERUS; VAGINA; FEMORAL NERVE; NERVE, MEDIAN; ULNAR NERVE; SPINAL ROOTS; GANGLION, DORSAL ROOT

Histopathology Observations [Correlation]:

ADRENAL GLANDS : CORTEX; MINERALIZATION; MILD

SALIVARY GLAND : SUBMANDIBULAR; INFILTRATE, CELLULAR; LYMPHOPLASMACYTIC, MILD

THYMUS : CORTEX; LYMPHOID ATROPHY; MINIMAL

NO CORRELATE : NO CORRELATE [KIDNEYS : RIGHT; CYST(S); CLEAR, TGL1 : (Comment) 2x2x1mm, Histology section prioritized, no frozen sample taken. (G)]

Histopathology - The following Tissues were Within Normal Limits:

AORTA; BONE MARROW; BRAIN; CECUM; CERVIX; COLON; DUODENUM; ESOPHAGUS; EYES; FEMUR; GALLBLADDER; HEART; ILEUM; JEJUNUM; KIDNEYS; LIVER; LUNGS; LYMPH NODES, MANDIBULAR; LYMPH NODES, MESENTERIC; MAMMARY GLAND; OVARIES; PANCREAS; PARATHYROID GLAND; PITUITARY GLAND; RECTUM; SCIATIC NERVE; SKELETAL MUSCLE; SKIN; SPINAL CORD; SPLEEN; STOMACH; THYROID GLANDS; TRACHEA; URINARY BLADDER; UTERUS; VAGINA; SPINAL ROOTS; GANGLION, DORSAL ROOT

AmplifyBio Study Number A-579-01

Individual Animal Gross & Microscopic Observations

Animal:	252	Group:	2	Sex:	Female
Species:	Nonhuman Primate	Strain:	Cynomolgus Monkey		
Study Type:	Subchronic (>14 d but < 6 mo) - Other				
		Dose:	Group 2 Low Total vg 6x10^13		
Death Date:	8/2/2023	Removal Reason:	Core Necropsy		
Necropsy Date:	8/2/2023	Study Day (Week) of Death:	43 (7)		
				Gross Status:	Complete
				Histo Status:	Complete

Terminal Bodyweight: 2.69kg

Organ Weights:

Adrenal Glands:	0.4762g	Brain:	55.23g	Heart:	11.8516g
Kidneys:	14.61g	Liver:	78.51g	Lungs:	28.58g
Ovaries:	0.3053g	Pituitary Gland:	0.0320g	Spleen:	3.7092g
Thymus:	2.220g	Thyroid/Parathyroid:	0.6206g	Uterus/Cervix:	9.1057g

Gross Pathology Observations [Correlation]:

Gross Pathology - The following Tissues had No Visible Lesions:

ADRENAL GLANDS; AORTA; BONE MARROW; BRAIN; CECUM; CERVIX; COLON; DUODENUM; ESOPHAGUS; EYES; FEMUR; GALLBLADDER; HEART; ILEUM; JEJUNUM; KIDNEYS; LIVER; LUNGS; LYMPH NODES, MANDIBULAR; LYMPH NODES, MESENTERIC; MAMMARY GLAND; OPTIC NERVE; OVARIES; PANCREAS; PARATHYROID GLAND; PITUITARY GLAND; RECTUM; SALIVARY GLAND; SCIATIC NERVE; SKELETAL MUSCLE; SKIN; SPINAL CORD; SPLEEN; STOMACH; THYMUS; THYROID GLANDS; TRACHEA; URINARY BLADDER; UTERUS; VAGINA; FEMORAL NERVE; NERVE, MEDIAN; ULNAR NERVE; SPINAL ROOTS; GANGLION, DORSAL ROOT

Histopathology Observations [Correlation]:

Histopathology - The following Tissues were Within Normal Limits:

ADRENAL GLANDS; AORTA; BONE MARROW; BRAIN; CECUM; CERVIX; COLON; DUODENUM; ESOPHAGUS; EYES; FEMUR; GALLBLADDER; HEART; ILEUM; JEJUNUM; KIDNEYS; LIVER; LUNGS; LYMPH NODES, MANDIBULAR; LYMPH NODES, MESENTERIC; MAMMARY GLAND; OVARIES; PANCREAS; PARATHYROID GLAND; PITUITARY GLAND; RECTUM; SALIVARY GLAND; SCIATIC NERVE; SKELETAL MUSCLE; SKIN; SPINAL CORD; SPLEEN; STOMACH; THYMUS; THYROID GLANDS; TRACHEA; URINARY BLADDER; UTERUS; VAGINA; SPINAL ROOTS; GANGLION, DORSAL ROOT

AmplifyBio Study Number A-579-01

Individual Animal Gross & Microscopic Observations

Animal:	253	Group:	2	Sex:	Female
Species:	Nonhuman Primate	Strain:	Cynomolgus Monkey		
Study Type:	Subchronic (>14 d but < 6 mo) - Other				
		Dose:	Group 2 Low Total vg 6x10^13		
Death Date:	9/13/2023	Removal Reason:	Recovery Necropsy 1		
Necropsy Date:	9/13/2023	Study Day (Week) of Death:	85 (13)		
				Gross Status:	Complete
				Histo Status:	Complete

Terminal Bodyweight: 3.51kg

Organ Weights:

Adrenal Glands:	0.7133g	Brain:	59.09g	Heart:	10.2367g
Kidneys:	15.50g	Liver:	83.22g	Lungs:	31.30g
Ovaries:	0.2727g	Pituitary Gland:	0.0682g	Spleen:	2.9891g
Thymus:	1.249g	Thyroid/Parathyroid:	0.3084g	Uterus/Cervix:	5.1515g

Gross Pathology Observations [Correlation]:

EYES : (Comment) Left eye was damaged (popped) at necropsy.

Gross Pathology - The following Tissues had No Visible Lesions:

ADRENAL GLANDS; AORTA; BONE MARROW; BRAIN; CECUM; CERVIX; COLON; DUODENUM; ESOPHAGUS; EYES; FEMUR; GALLBLADDER; HEART; ILEUM; JEJUNUM; KIDNEYS; LIVER; LUNGS; LYMPH NODES, MANDIBULAR; LYMPH NODES, MESENTERIC; MAMMARY GLAND; OPTIC NERVE; OVARIES; PANCREAS; PARATHYROID GLAND; PITUITARY GLAND; RECTUM; SALIVARY GLAND; SCIATIC NERVE; SKELETAL MUSCLE; SKIN; SPINAL CORD; SPLEEN; STOMACH; THYMUS; THYROID GLANDS; TRACHEA; URINARY BLADDER; UTERUS; VAGINA; FEMORAL NERVE; NERVE, MEDIAN; ULNAR NERVE; SPINAL ROOTS; GANGLION, DORSAL ROOT

Histopathology Observations [Correlation]:

Histopathology - The following Tissues were Within Normal Limits:

ADRENAL GLANDS; AORTA; BONE MARROW; BRAIN; CECUM; CERVIX; COLON; DUODENUM; ESOPHAGUS; EYES; FEMUR; GALLBLADDER; HEART; ILEUM; JEJUNUM; KIDNEYS; LIVER; LUNGS; LYMPH NODES, MANDIBULAR; LYMPH NODES, MESENTERIC; MAMMARY GLAND; OVARIES; PANCREAS; PARATHYROID GLAND; PITUITARY GLAND; RECTUM; SALIVARY GLAND; SCIATIC NERVE; SKELETAL MUSCLE; SKIN; SPINAL CORD; SPLEEN; STOMACH; THYMUS; THYROID GLANDS; TRACHEA; URINARY BLADDER; UTERUS; VAGINA; SPINAL ROOTS; GANGLION, DORSAL ROOT

10/30/2023 10:47:43AM Page: 12

AmplifyBio Study Number A-579-01

Individual Animal Gross & Microscopic Observations

Animal:	254	Group:	2	Sex:	Female
Species:	Nonhuman Primate	Strain:	Cynomolgus Monkey		
Study Type:	Subchronic (>14 d but < 6 mo) - Other				
		Dose:	Group 2 Low Total vg 6x10^13		
Death Date:	9/13/2023	Removal Reason:	Recovery Necropsy 1		
Necropsy Date:	9/13/2023	Study Day (Week) of Death:	85 (13)		
				Gross Status:	Complete
				Histo Status:	Complete

Terminal Bodyweight: 2.70kg

Organ Weights:

Adrenal Glands:	0.4362g	Brain:	55.33g	Heart:	10.0959g
Kidneys:	11.35g	Liver:	62.20g	Lungs:	41.87g
Ovaries:	0.4062g	Pituitary Gland:	0.0604g	Spleen:	1.9618g
Thymus:	1.542g	Thyroid/Parathyroid:	0.3136g	Uterus/Cervix:	8.6399g

Gross Pathology Observations [Correlation]:

LUNGS : DIFFUSE; FOCI; WHITE, TGL1 : (Comment) No externally visible foci on left cranial and right middle lobes, all other lobes affected. up to 4x2mm (TGL)

Gross Pathology - The following Tissues had No Visible Lesions:

ADRENAL GLANDS; AORTA; BONE MARROW; BRAIN; CECUM; CERVIX; COLON; DUODENUM; ESOPHAGUS; EYES; FEMUR; GALLBLADDER; HEART; ILEUM; JEJUNUM; KIDNEYS; LIVER; LYMPH NODES, MANDIBULAR; LYMPH NODES, MESENTERIC; MAMMARY GLAND; OPTIC NERVE; OVARIES; PANCREAS; PARATHYROID GLAND; PITUITARY GLAND; RECTUM; SALIVARY GLAND; SCIATIC NERVE; SKELETAL MUSCLE; SKIN; SPINAL CORD; SPLEEN; STOMACH; THYMUS; THYROID GLANDS; TRACHEA; URINARY BLADDER; UTERUS; VAGINA; FEMORAL NERVE; NERVE, MEDIAN; ULNAR NERVE; SPINAL ROOTS; GANGLION, DORSAL ROOT

Histopathology Observations [Correlation]:

LIVER : INFLAMMATION; MONONUCLEAR CELL, FOCAL, MINIMAL
LUNGS : ALVEOLUS; INFILTRATE, CELLULAR; HISTIOCYTIC, MILD [LUNGS : DIFFUSE; FOCI; WHITE, TGL1 : (Comment) No externally visible foci on left cranial and right middle lobes, all other lobes affected. up to 4x2mm (G)]
SPINAL CORD : CERVICAL (NECK); AXON; DEGENERATION; WALLERIAN, MINIMAL
SPINAL CORD : LUMBAR; AXON; DEGENERATION; WALLERIAN, MINIMAL
SPINAL CORD : THORACIC; AXON; DEGENERATION; WALLERIAN, MINIMAL
THYMUS : CORTEX; LYMPHOID ATROPHY; MINIMAL
URINARY BLADDER : MUCOSA; INFLAMMATION; MONONUCLEAR CELL, MILD
GANGLION, DORSAL ROOT : CERVICAL (NECK); NEURON; NECROSIS; MINIMAL
GANGLION, DORSAL ROOT : CERVICAL (NECK); INCREASED CELLULARITY; MONONUCLEAR CELL, MILD
GANGLION, DORSAL ROOT : LUMBAR; INCREASED CELLULARITY; MONONUCLEAR CELL, MILD
GANGLION, DORSAL ROOT : CERVICAL (NECK); NEURON; DEGENERATION; MINIMAL
GANGLION, DORSAL ROOT : LUMBAR; NEURON; DEGENERATION; MINIMAL

Histopathology - The following Tissues were Within Normal Limits:

AmplifyBio Study Number A-579-01

Individual Animal Gross & Microscopic Observations

Animal: 254 (Continued) Group: 2 Sex: Female

ADRENAL GLANDS; AORTA; BONE MARROW; BRAIN; CECUM; CERVIX; COLON; DUODENUM; ESOPHAGUS; EYES; FEMUR; GALLBLADDER; HEART; ILEUM; JEJUNUM; KIDNEYS; LYMPH NODES, MANDIBULAR; LYMPH NODES, MESENTERIC; MAMMARY GLAND; OVARIES; PANCREAS; PARATHYROID GLAND; PITUITARY GLAND; RECTUM; SALIVARY GLAND; SCIATIC NERVE; SKELETAL MUSCLE; SKIN; SPLEEN; STOMACH; THYROID GLANDS; TRACHEA; UTERUS; VAGINA; SPINAL ROOTS

10/30/2023 10:47:43AM Page: 14

AmplifyBio Study Number A-579-01

Individual Animal Gross & Microscopic Observations

Animal:	301	Group:	3	Sex:	Male
Species:	Nonhuman Primate	Strain:	Cynomolgus Monkey		
Study Type:	Subchronic (>14 d but < 6 mo) - Other				
		Dose:	Group 3 High Total vg 1.2x10^14		
Death Date:	8/3/2023	Removal Reason:	Core Necropsy		
Necropsy Date:	8/3/2023	Study Day (Week) of Death:	43 (7)		
				Gross Status:	Complete
				Histo Status:	Complete

Terminal Bodyweight: 2.73kg

Organ Weights:

Adrenal Glands:	0.4957g	Brain:	67.31g	Epididymis:	0.7159g
Heart:	12.4127g	Kidneys:	13.00g	Liver:	64.00g
Lungs:	35.90g	Pituitary Gland:	0.0524g	Spleen:	3.8894g
Testes:	1.49g	Thymus:	4.034g	Thyroid/Parathyroid:	0.6235g

Gross Pathology Observations [Correlation]:

Gross Pathology - The following Tissues had No Visible Lesions:

ADRENAL GLANDS; AORTA; BONE MARROW; BRAIN; CECUM; COLON; DUODENUM; EPIDIDYMIS; ESOPHAGUS; EYES; FEMUR; GALLBLADDER; HEART; ILEUM; JEJUNUM; KIDNEYS; LIVER; LUNGS; LYMPH NODES, MANDIBULAR; LYMPH NODES, MESENTERIC; MAMMARY GLAND; OPTIC NERVE; PANCREAS; PARATHYROID GLAND; PITUITARY GLAND; PROSTATE; RECTUM; SALIVARY GLAND; SCIATIC NERVE; SEMINAL VESICLES; SKELETAL MUSCLE; SKIN; SPINAL CORD; SPLEEN; STOMACH; TESTES; THYMUS; THYROID GLANDS; TRACHEA; URINARY BLADDER; FEMORAL NERVE; NERVE, MEDIAN; ULNAR NERVE; SPINAL ROOTS; GANGLION, DORSAL ROOT

Histopathology Observations [Correlation]:

GANGLION, DORSAL ROOT : CERVICAL (NECK); NEURON; NECROSIS; MINIMAL

Histopathology - The following Tissues were Within Normal Limits:

ADRENAL GLANDS; AORTA; BONE MARROW; BRAIN; CECUM; COLON; DUODENUM; EPIDIDYMIS; ESOPHAGUS; EYES; FEMUR; GALLBLADDER; HEART; ILEUM; JEJUNUM; KIDNEYS; LIVER; LUNGS; LYMPH NODES, MANDIBULAR; LYMPH NODES, MESENTERIC; MAMMARY GLAND; PANCREAS; PARATHYROID GLAND; PITUITARY GLAND; PROSTATE; RECTUM; SALIVARY GLAND; SCIATIC NERVE; SEMINAL VESICLES; SKELETAL MUSCLE; SKIN; SPINAL CORD; SPLEEN; STOMACH; TESTES; THYMUS; THYROID GLANDS; TRACHEA; URINARY BLADDER; SPINAL ROOTS

AmplifyBio Study Number A-579-01

Individual Animal Gross & Microscopic Observations

Animal:	302	Group:	3	Sex:	Male
Species:	Nonhuman Primate	Strain:	Cynomolgus Monkey		
Study Type:	Subchronic (>14 d but < 6 mo) - Other				
		Dose:	Group 3 High Total vg 1.2x10^14		
Death Date:	8/3/2023	Removal Reason:	Core Necropsy		
Necropsy Date:	8/3/2023	Study Day (Week) of Death:	43 (7)		
				Gross Status:	Complete
				Histo Status:	Complete

Terminal Bodyweight: 2.43kg

Organ Weights:

Adrenal Glands:	0.6618g	Brain:	68.07g	Epididymis:	0.5773g
Heart:	11.9665g	Kidneys:	15.08g	Liver:	67.07g
Lungs:	22.48g	Pituitary Gland:	0.0410g	Spleen:	2.9518g
Testes:	1.05g	Thymus:	2.931g	Thyroid/Parathyroid:	0.5846g

Gross Pathology Observations [Correlation]:

LUNGS : LEFT; CAUDAL LOBE; FOCUS; WHITE, HARD, TGL2 : (Comment) up to 15x10mm (TGL)
LUNGS : LEFT; CRANIAL LOBE; FOCUS; WHITE, HARD, TGL1 : (Comment) 5x5mm (TGL)

Gross Pathology - The following Tissues had No Visible Lesions:

ADRENAL GLANDS; AORTA; BONE MARROW; BRAIN; CECUM; COLON; DUODENUM; EPIDIDYMIS; ESOPHAGUS; EYES; FEMUR; GALLBLADDER; HEART; ILEUM; JEJUNUM; KIDNEYS; LIVER; LYMPH NODES, MANDIBULAR; LYMPH NODES, MESENTERIC; MAMMARY GLAND; OPTIC NERVE; PANCREAS; PARATHYROID GLAND; PITUITARY GLAND; PROSTATE; RECTUM; SALIVARY GLAND; SCIATIC NERVE; SEMINAL VESICLES; SKELETAL MUSCLE; SKIN; SPINAL CORD; SPLEEN; STOMACH; TESTES; THYMUS; THYROID GLANDS; TRACHEA; URINARY BLADDER; FEMORAL NERVE; NERVE, MEDIAN; ULNAR NERVE; SPINAL ROOTS; GANGLION, DORSAL ROOT

Histopathology Observations [Correlation]:

LUNGS : PERIVASCULAR; INFILTRATE, CELLULAR; HISTIOCYTIC, MILD [LUNGS : LEFT; CRANIAL LOBE; FOCUS; WHITE, HARD, TGL1 : (Comment) 5x5mm (G) | LUNGS : LEFT; CAUDAL LOBE; FOCUS; WHITE, HARD, TGL2 : (Comment) up to 15x10mm (G)]
GANGLION, DORSAL ROOT : CERVICAL (NECK); NEURON; DEGENERATION; MILD
GANGLION, DORSAL ROOT : THORACIC; NEURON; DEGENERATION; MINIMAL
GANGLION, DORSAL ROOT : THORACIC; AUTOPHAGY, NEURON; MINIMAL

Histopathology - The following Tissues were Within Normal Limits:

ADRENAL GLANDS; AORTA; BONE MARROW; BRAIN; CECUM; COLON; DUODENUM; EPIDIDYMIS; ESOPHAGUS; EYES; FEMUR; GALLBLADDER; HEART; ILEUM; JEJUNUM; KIDNEYS; LIVER; LYMPH NODES, MANDIBULAR; LYMPH NODES, MESENTERIC; MAMMARY GLAND; PANCREAS; PARATHYROID GLAND; PITUITARY GLAND; PROSTATE; RECTUM; SALIVARY GLAND; SCIATIC NERVE; SEMINAL VESICLES; SKELETAL MUSCLE; SKIN; SPINAL CORD; SPLEEN; STOMACH; TESTES; THYMUS; THYROID GLANDS; TRACHEA; URINARY BLADDER; SPINAL ROOTS

AmplifyBio Study Number A-579-01

Individual Animal Gross & Microscopic Observations

Animal:	303	Group:	3	Sex:	Male
Species:	Nonhuman Primate	Strain:	Cynomolgus Monkey		
Study Type:	Subchronic (>14 d but < 6 mo) - Other				
		Dose:	Group 3 High Total vg 1.2x10^14		
Death Date:	9/14/2023	Removal Reason:	Recovery Necropsy 1		
Necropsy Date:	9/14/2023	Study Day (Week) of Death:	85 (13)		
				Gross Status:	Complete
				Histo Status:	Complete

Terminal Bodyweight: 3.44kg

Organ Weights:

Adrenal Glands:	0.6166g	Brain:	75.98g	Epididymis:	1.1665g
Heart:	11.8923g	Kidneys:	15.16g	Liver:	61.74g
Lungs:	20.89g	Pituitary Gland:	0.0487g	Spleen:	4.3526g
Testes:	1.66g	Thymus:	3.683g	Thyroid/Parathyroid:	0.1487g

Gross Pathology Observations [Correlation]:

Gross Pathology - The following Tissues had No Visible Lesions:

ADRENAL GLANDS; AORTA; BONE MARROW; BRAIN; CECUM; COLON; DUODENUM; EPIDIDYMIS; ESOPHAGUS; EYES; FEMUR; GALLBLADDER; HEART; ILEUM; JEJUNUM; KIDNEYS; LIVER; LUNGS; LYMPH NODES, MANDIBULAR; LYMPH NODES, MESENTERIC; MAMMARY GLAND; OPTIC NERVE; PANCREAS; PARATHYROID GLAND; PITUITARY GLAND; PROSTATE; RECTUM; SALIVARY GLAND; SCIATIC NERVE; SEMINAL VESICLES; SKELETAL MUSCLE; SKIN; SPINAL CORD; SPLEEN; STOMACH; TESTES; THYMUS; THYROID GLANDS; TRACHEA; URINARY BLADDER; FEMORAL NERVE; NERVE, MEDIAN; ULNAR NERVE; SPINAL ROOTS; GANGLION, DORSAL ROOT

Histopathology Observations [Correlation]:

PARATHYROID GLAND : PERIVASCULAR; INFILTRATE, CELLULAR; MONONUCLEAR CELL, MILD
SPINAL CORD : THORACIC; AXON; DEGENERATION; WALLERIAN, MINIMAL
THYMUS : CORTEX; LYMPHOID ATROPHY; MINIMAL
GANGLION, DORSAL ROOT : LUMBAR; NEURON; NECROSIS; MINIMAL
GANGLION, DORSAL ROOT : THORACIC; NEURON; NECROSIS; MINIMAL
GANGLION, DORSAL ROOT : CERVICAL (NECK); NEURON; DEGENERATION; MINIMAL
GANGLION, DORSAL ROOT : THORACIC; NEURON; DEGENERATION; MINIMAL

Histopathology - The following Tissues were Within Normal Limits:

ADRENAL GLANDS; AORTA; BONE MARROW; BRAIN; CECUM; COLON; DUODENUM; EPIDIDYMIS; ESOPHAGUS; EYES; FEMUR; GALLBLADDER; HEART; ILEUM; JEJUNUM; KIDNEYS; LIVER; LUNGS; LYMPH NODES, MANDIBULAR; LYMPH NODES, MESENTERIC; MAMMARY GLAND; PANCREAS; PITUITARY GLAND; PROSTATE; RECTUM; SALIVARY GLAND; SCIATIC NERVE; SEMINAL VESICLES; SKELETAL MUSCLE; SKIN; SPLEEN; STOMACH; TESTES; THYROID GLANDS; TRACHEA; URINARY BLADDER; SPINAL ROOTS

10/30/2023 10:47:43AM Page: 17

AmplifyBio Study Number A-579-01

Individual Animal Gross & Microscopic Observations

Animal:	304	Group:	3	Sex:	Male
Species:	Nonhuman Primate	Strain:	Cynomolgus Monkey		
Study Type:	Subchronic (>14 d but < 6 mo) - Other				
		Dose:	Group 3 High Total vg 1.2x10^14		
Death Date:	9/14/2023	Removal Reason:	Recovery Necropsy 1		
Necropsy Date:	9/14/2023	Study Day (Week) of Death:	85 (13)		
				Gross Status:	Complete
				Histo Status:	Complete

Terminal Bodyweight: 2.62kg

Organ Weights:

Adrenal Glands:	0.4716g	Brain:	74.98g	Epididymis:	0.9744g
Heart:	12.7277g	Kidneys:	12.90g	Liver:	54.56g
Lungs:	40.91g	Pituitary Gland:	0.0318g	Spleen:	4.5875g
Testes:	0.33g	Thymus:	0.416g	Thyroid/Parathyroid:	0.4207g

Gross Pathology Observations [Correlation]:

Gross Pathology - The following Tissues had No Visible Lesions:

ADRENAL GLANDS; AORTA; BONE MARROW; BRAIN; CECUM; COLON; DUODENUM; EPIDIDYMIS; ESOPHAGUS; EYES; FEMUR; GALLBLADDER; HEART; ILEUM; JEJUNUM; KIDNEYS; LIVER; LUNGS; LYMPH NODES, MANDIBULAR; LYMPH NODES, MESENTERIC; MAMMARY GLAND; OPTIC NERVE; PANCREAS; PARATHYROID GLAND; PITUITARY GLAND; PROSTATE; RECTUM; SALIVARY GLAND; SCIATIC NERVE; SEMINAL VESICLES; SKELETAL MUSCLE; SKIN; SPINAL CORD; SPLEEN; STOMACH; TESTES; THYMUS; THYROID GLANDS; TRACHEA; URINARY BLADDER; FEMORAL NERVE; NERVE, MEDIAN; ULNAR NERVE; SPINAL ROOTS; GANGLION, DORSAL ROOT

Histopathology Observations [Correlation]:

BRAIN : CEREBRUM; PERIVASCULAR; INFILTRATE, CELLULAR; MONONUCLEAR CELL, FOCAL, MILD : (Comment) Section 2

LIVER : INFLAMMATION; MONONUCLEAR CELL, FOCAL, MINIMAL

PITUITARY GLAND : PARS DISTALIS; CYST

GANGLION, DORSAL ROOT : THORACIC; NEURON; VACUOLIZATION CYTOPLASM; MINIMAL

Histopathology - The following Tissues were Within Normal Limits:

ADRENAL GLANDS; AORTA; BONE MARROW; CECUM; COLON; DUODENUM; EPIDIDYMIS; ESOPHAGUS; EYES; FEMUR; GALLBLADDER; HEART; ILEUM; JEJUNUM; KIDNEYS; LUNGS; LYMPH NODES, MANDIBULAR; LYMPH NODES, MESENTERIC; MAMMARY GLAND; PANCREAS; PARATHYROID GLAND; PROSTATE; RECTUM; SALIVARY GLAND; SCIATIC NERVE; SEMINAL VESICLES; SKELETAL MUSCLE; SKIN; SPINAL CORD; SPLEEN; STOMACH; TESTES; THYMUS; THYROID GLANDS; TRACHEA; URINARY BLADDER; SPINAL ROOTS

AmplifyBio Study Number A-579-01

Individual Animal Gross & Microscopic Observations

Animal:	351	Group:	3	Sex:	Female
Species:	Nonhuman Primate	Strain:	Cynomolgus Monkey		
Study Type:	Subchronic (>14 d but < 6 mo) - Other				
		Dose:	Group 3 High Total vg 1.2x10^14		
Death Date:	8/3/2023	Removal Reason:	Core Necropsy		
Necropsy Date:	8/3/2023	Study Day (Week) of Death:	43 (7)		
				Gross Status:	Complete
				Histo Status:	Complete

Terminal Bodyweight: 2.39kg

Organ Weights:

Adrenal Glands:	0.4195g	Brain:	62.51g	Heart:	10.9024g
Kidneys:	11.91g	Liver:	67.09g	Lungs:	23.29g
Ovaries:	0.3599g	Pituitary Gland:	0.0550g	Spleen:	2.0516g
Thymus:	2.355g	Thyroid/Parathyroid:	0.1719g	Uterus/Cervix:	3.5890g

Gross Pathology Observations [Correlation]:

Gross Pathology - The following Tissues had No Visible Lesions:

ADRENAL GLANDS; AORTA; BONE MARROW; BRAIN; CECUM; CERVIX; COLON; DUODENUM; ESOPHAGUS; EYES; FEMUR; GALLBLADDER; HEART; ILEUM; JEJUNUM; KIDNEYS; LIVER; LUNGS; LYMPH NODES, MANDIBULAR; LYMPH NODES, MESENTERIC; MAMMARY GLAND; OPTIC NERVE; OVARIES; PANCREAS; PARATHYROID GLAND; PITUITARY GLAND; RECTUM; SALIVARY GLAND; SCIATIC NERVE; SKELETAL MUSCLE; SKIN; SPINAL CORD; SPLEEN; STOMACH; THYMUS; THYROID GLANDS; TRACHEA; URINARY BLADDER; UTERUS; VAGINA; FEMORAL NERVE; NERVE, MEDIAN; ULNAR NERVE; SPINAL ROOTS; GANGLION, DORSAL ROOT

Histopathology Observations [Correlation]:

THYMUS : CORTEX; LYMPHOID ATROPHY; MINIMAL

GANGLION, DORSAL ROOT : CERVICAL (NECK); AUTOPHAGY, NEURON; MINIMAL

GANGLION, DORSAL ROOT : LUMBAR; AUTOPHAGY, NEURON; MINIMAL

GANGLION, DORSAL ROOT : THORACIC; AUTOPHAGY, NEURON; MINIMAL

GANGLION, DORSAL ROOT : LUMBAR; NEURON; VACUOLIZATION CYTOPLASM; MILD

Histopathology - The following Tissues were Within Normal Limits:

ADRENAL GLANDS; AORTA; BONE MARROW; BRAIN; CECUM; CERVIX; COLON; DUODENUM; ESOPHAGUS; EYES; FEMUR; GALLBLADDER; HEART; ILEUM; JEJUNUM; KIDNEYS; LIVER; LUNGS; LYMPH NODES, MANDIBULAR; LYMPH NODES, MESENTERIC; MAMMARY GLAND; OVARIES; PANCREAS; PITUITARY GLAND; RECTUM; SALIVARY GLAND; SCIATIC NERVE; SKELETAL MUSCLE; SKIN; SPINAL CORD; SPLEEN; STOMACH; THYROID GLANDS; TRACHEA; URINARY BLADDER; UTERUS; VAGINA; SPINAL ROOTS

Histopathology - The following Tissues were Not Examined:

PARATHYROID GLAND - No Section

AmplifyBio Study Number A-579-01

Individual Animal Gross & Microscopic Observations

Animal:	352	Group:	3	Sex:	Female
Species:	Nonhuman Primate	Strain:	Cynomolgus Monkey		
Study Type:	Subchronic (>14 d but < 6 mo) - Other				
		Dose:	Group 3 High Total vg 1.2x10^14		
Death Date:	8/3/2023	Removal Reason:	Core Necropsy		
Necropsy Date:	8/3/2023	Study Day (Week) of Death:	43 (7)		
				Gross Status:	Complete
				Histo Status:	Complete

Terminal Bodyweight: 2.87kg

Organ Weights:

Adrenal Glands:	0.5156g	Brain:	67.42g	Heart:	10.7123g
Kidneys:	16.81g	Liver:	73.58g	Lungs:	29.27g
Ovaries:	0.3643g	Pituitary Gland:	0.0596g	Spleen:	2.4764g
Thymus:	1.768g	Thyroid/Parathyroid:	0.2615g	Uterus/Cervix:	6.2268g

Gross Pathology Observations [Correlation]:

OVARIES : (Comment) Left ovary collected for frozen sampling, Right ovary collect as gross lesion frozen sampling.

OVARIES : RIGHT; CYST(S); CLEAR, TGL1 : (Comment) 9x6x3mm (TGL)

Gross Pathology - The following Tissues had No Visible Lesions:

ADRENAL GLANDS; AORTA; BONE MARROW; BRAIN; CECUM; CERVIX; COLON; DUODENUM; ESOPHAGUS; EYES; FEMUR; GALLBLADDER; HEART; ILEUM; JEJUNUM; KIDNEYS; LIVER; LUNGS; LYMPH NODES, MANDIBULAR; LYMPH NODES, MESENTERIC; MAMMARY GLAND; OPTIC NERVE; PANCREAS; PARATHYROID GLAND; PITUITARY GLAND; RECTUM; SALIVARY GLAND; SCIATIC NERVE; SKELETAL MUSCLE; SKIN; SPINAL CORD; SPLEEN; STOMACH; THYMUS; THYROID GLANDS; TRACHEA; URINARY BLADDER; UTERUS; VAGINA; FEMORAL NERVE; NERVE, MEDIAN; ULNAR NERVE; SPINAL ROOTS; GANGLION, DORSAL ROOT

Histopathology Observations [Correlation]:

SPINAL CORD : LUMBAR; AXON; DEGENERATION; WALLERIAN, MINIMAL

THYMUS : CORTEX; LYMPHOID ATROPHY; MINIMAL

NO CORRELATE : NO CORRELATE [OVARIES : RIGHT; CYST(S); CLEAR, TGL1 : (Comment) 9x6x3mm (G)]

GANGLION, DORSAL ROOT : LUMBAR; NEURON; VACUOLIZATION CYTOPLASM; MILD

Histopathology - The following Tissues were Within Normal Limits:

ADRENAL GLANDS; AORTA; BONE MARROW; BRAIN; CECUM; CERVIX; COLON; DUODENUM; ESOPHAGUS; EYES; FEMUR; GALLBLADDER; HEART; ILEUM; JEJUNUM; KIDNEYS; LIVER; LUNGS; LYMPH NODES, MANDIBULAR; LYMPH NODES, MESENTERIC; MAMMARY GLAND; OVARIES; PANCREAS; PARATHYROID GLAND; PITUITARY GLAND; RECTUM; SALIVARY GLAND; SCIATIC NERVE; SKELETAL MUSCLE; SKIN; SPLEEN; STOMACH; THYROID GLANDS; TRACHEA; URINARY BLADDER; UTERUS; VAGINA; SPINAL ROOTS

AmplifyBio Study Number A-579-01

Individual Animal Gross & Microscopic Observations

Animal:	353	Group:	3	Sex:	Female
Species:	Nonhuman Primate	Strain:	Cynomolgus Monkey		
Study Type:	Subchronic (>14 d but < 6 mo) - Other				
		Dose:	Group 3 High Total vg 1.2x10^14		
Death Date:	9/14/2023	Removal Reason:	Recovery Necropsy 1		
Necropsy Date:	9/14/2023	Study Day (Week) of Death:	85 (13)		
				Gross Status:	Complete
				Histo Status:	Complete

Terminal Bodyweight: 2.82kg

Organ Weights:

Adrenal Glands:	0.7916g	Brain:	61.47g	Heart:	11.1221g
Kidneys:	14.43g	Liver:	70.35g	Lungs:	30.16g
Ovaries:	0.2969g	Pituitary Gland:	0.0595g	Spleen:	2.2819g
Thymus:	1.380g	Thyroid/Parathyroid:	0.5013g	Uterus/Cervix:	9.3591g

Gross Pathology Observations [Correlation]:

LYMPH NODES, INGUINAL : LEFT; ENLARGED; ONE, TGL2 : (Comment) 15x10x5mm (TGL)
LYMPH NODES, MESENTERIC : DIFFUSE; ENLARGED; MULTIPLE, TGL3 : (Comment) up to 10x4x3mm, Distal along mesenteric border of the GI (TGL)
SKIN : LEFT; DORSAL; CRUST(S); TGL1 : (Comment) 25x20mm, calloused pad next to tail (TGL)

Gross Pathology - The following Tissues had No Visible Lesions:

ADRENAL GLANDS; AORTA; BONE MARROW; BRAIN; CECUM; CERVIX; COLON; DUODENUM; ESOPHAGUS; EYES; FEMUR; GALLBLADDER; HEART; ILEUM; JEJUNUM; KIDNEYS; LIVER; LUNGS; LYMPH NODES, MANDIBULAR; MAMMARY GLAND; OPTIC NERVE; OVARIES; PANCREAS; PARATHYROID GLAND; PITUITARY GLAND; RECTUM; SALIVARY GLAND; SCIATIC NERVE; SKELETAL MUSCLE; SPINAL CORD; SPLEEN; STOMACH; THYMUS; THYROID GLANDS; TRACHEA; URINARY BLADDER; UTERUS; VAGINA; FEMORAL NERVE; NERVE, MEDIAN; ULNAR NERVE; SPINAL ROOTS; GANGLION, DORSAL ROOT

Histopathology Observations [Correlation]:

LYMPH NODES, INGUINAL : INCREASED CELLULARITY; LYMPHOCYTIC, MILD [LYMPH NODES, INGUINAL : LEFT; ENLARGED; ONE, TGL2 : (Comment) 15x10x5mm (G)]
LYMPH NODES, MESENTERIC : INCREASED CELLULARITY; LYMPHOCYTIC, MILD [LYMPH NODES, MESENTERIC : DIFFUSE; ENLARGED; MULTIPLE, TGL3 : (Comment) up to 10x4x3mm, Distal along mesenteric border of the GI (G)]
SKIN : ULCER [SKIN : LEFT; DORSAL; CRUST(S); TGL1 : (Comment) 25x20mm, calloused pad next to tail (G)]
GANGLION, DORSAL ROOT : LUMBAR; INCREASED CELLULARITY; MONONUCLEAR CELL, MILD
GANGLION, DORSAL ROOT : LUMBAR; AUTOPHAGY, NEURON; MINIMAL

Histopathology - The following Tissues were Within Normal Limits:

ADRENAL GLANDS; AORTA; BONE MARROW; BRAIN; CECUM; CERVIX; COLON; DUODENUM; ESOPHAGUS; EYES; FEMUR; GALLBLADDER; HEART; ILEUM; JEJUNUM; KIDNEYS; LIVER; LUNGS; LYMPH NODES, MANDIBULAR; MAMMARY GLAND; OVARIES; PANCREAS; PARATHYROID GLAND; PITUITARY GLAND; RECTUM; SALIVARY GLAND; SCIATIC NERVE; SKELETAL MUSCLE; SPINAL CORD; SPLEEN; STOMACH; THYMUS; THYROID GLANDS; TRACHEA; URINARY BLADDER; UTERUS; VAGINA; SPINAL ROOTS

10/30/2023 10:47:43AM Page: 21

AmplifyBio Study Number A-579-01

Individual Animal Gross & Microscopic Observations

Animal:	354	Group:	3	Sex:	Female
Species:	Nonhuman Primate	Strain:	Cynomolgus Monkey		
Study Type:	Subchronic (>14 d but < 6 mo) - Other				
		Dose:	Group 3 High Total vg 1.2x10^14		
Death Date:	9/14/2023	Removal Reason:	Recovery Necropsy 1		
Necropsy Date:	9/14/2023	Study Day (Week) of Death:	85 (13)		
				Gross Status:	Complete
				Histo Status:	Complete

Terminal Bodyweight: 2.88kg

Organ Weights:

Adrenal Glands:	0.7849g	Brain:	73.66g	Heart:	13.9208g
Kidneys:	17.50g	Liver:	106.30g	Lungs:	29.63g
Ovaries:	0.5532g	Pituitary Gland:	0.0501g	Spleen:	5.6639g
Thymus:	2.252g	Thyroid/Parathyroid:	0.4862g	Uterus/Cervix:	11.4224g

Gross Pathology Observations [Correlation]:

BRAIN : BILATERAL; DILATATION; TGL1 : (Comment) lateral ventricles 30x9x4mm (TGL)

Gross Pathology - The following Tissues had No Visible Lesions:

ADRENAL GLANDS; AORTA; BONE MARROW; CECUM; CERVIX; COLON; DUODENUM; ESOPHAGUS; EYES; FEMUR; GALLBLADDER; HEART; ILEUM; JEJUNUM; KIDNEYS; LIVER; LUNGS; LYMPH NODES, MANDIBULAR; LYMPH NODES, MESENTERIC; MAMMARY GLAND; OPTIC NERVE; OVARIES; PANCREAS; PARATHYROID GLAND; PITUITARY GLAND; RECTUM; SALIVARY GLAND; SCIATIC NERVE; SKELETAL MUSCLE; SKIN; SPINAL CORD; SPLEEN; STOMACH; THYMUS; THYROID GLANDS; TRACHEA; URINARY BLADDER; UTERUS; VAGINA; FEMORAL NERVE; NERVE, MEDIAN; ULNAR NERVE; SPINAL ROOTS; GANGLION, DORSAL ROOT

Histopathology Observations [Correlation]:

HEART : INFILTRATE, CELLULAR; MONONUCLEAR CELL, MINIMAL

OVARIES : CYST

SCIATIC NERVE : LEFT; DISTAL; INFILTRATE, CELLULAR; MONONUCLEAR CELL, MILD

THYROID GLANDS : ECTOPIC TISSUE, THYMUS

NO CORRELATE : NO CORRELATE [BRAIN : BILATERAL; DILATATION; TGL1 : (Comment) lateral ventricles 30x9x4mm (G)]

GANGLION, DORSAL ROOT : THORACIC; NEURON; DEGENERATION; MINIMAL

Histopathology - The following Tissues were Within Normal Limits:

ADRENAL GLANDS; AORTA; BONE MARROW; BRAIN; CECUM; CERVIX; COLON; DUODENUM; ESOPHAGUS; EYES; FEMUR; GALLBLADDER; ILEUM; JEJUNUM; KIDNEYS; LIVER; LUNGS; LYMPH NODES, MANDIBULAR; LYMPH NODES, MESENTERIC; MAMMARY GLAND; PANCREAS; PARATHYROID GLAND; PITUITARY GLAND; RECTUM; SALIVARY GLAND; SKELETAL MUSCLE; SKIN; SPINAL CORD; SPLEEN; STOMACH; THYMUS; TRACHEA; URINARY BLADDER; UTERUS; VAGINA; SPINAL ROOTS

AmplifyBio Study Number A-579-01

Individual Animal Gross & Microscopic Observations

Key Page

Codes

(TGL) = Trackable Gross Lesion, (MPF) = Major Pathological Finding, (?) = Questionable, (E) = Excluded,
(C) = Clinical Observation, (M) = Mass, (G) = Gross Pathology, (H) = Histopathology

Report Request Items

Animals Included:	All
Groups:	All
Observation Type:	Gross and Histo
Tissues:	All
Removal Reasons:	All

Unit Descriptions

Headings Used	Description
g	g
kg	kg

Group Information

Short Name	Long Name	Type
1	Group 1	Control
2	Group 2	Dose
3	Group 3	Dose

Appendix L dPCR Contributing Scientist Report

A 12-Week Single Dose Biodistribution and Safety Study in Cynomolgus Monkeys

dPCR Contributing Scientist Report

October 16, 2024

Study Number A-579-01

Testing Facility
AmplifyBio
1425 State Route 142 (Plain City-Georgesville Road)
West Jefferson, OH 43162

SIGNATURES

Prepared by:

Signed by:

Sapna Varia

Signer Name: Sapna Varia
Signing Reason: I approve this document
Signing Time: 10/16/2024 | 9:35:36 AM EDT
5D64C6F0F9AF4A7B9EBF31D648E66020

10/16/2024

Sapna Varia, PhD
Director, Analytical Sciences Operations

Date

TABLE OF CONTENTS

Page

Signatures 2

1.0 Introduction 4

2.0 Study Design and Methods 4

2.1 Materials and Methods 4

2.1.1 Frozen Tissue Samples 4
2.1.2 DNA Extraction 5
2.1.3 DNA Quantification and Dilution 5
2.1.4 dPCR Assay 6

3.0 Data Capture and dPCR Analysis 7

3.1 dPCR Assay Performance Quality Control 8

4.0 Deviations 9

5.0 Results 11

6.0 Conclusion 15

LIST OF ATTACHMENTS

Attachment 1. Study Specific Methods 16

Attachment 2. Final Data Table 33

Attachment 3. Precision Principal Investigator Report 61

Attachment 4. Memos 295

1.0 INTRODUCTION

This contributing scientist report summarizes the methods and analytical findings of the digital PCR (dPCR) analysis during Study No. A-579-01. The purpose of this study was to evaluate the potential biodistribution and safety of scAAV9.U6miR871 (ARM-101) when administered once via intrathecal infusion to Cynomolgus monkeys for up to 12 weeks.

2.0 STUDY DESIGN AND METHODS

The study design relating to dPCR analysis is outlined below.

Table 1. Study Design

Group	Target Dose (Total vg)	Target Dose Volume (mL)	Animal Identification			
			6-Week Cohort[a]/Day 43		12-Week Cohort[b]/Day 85	
			Male	Female	Male	Female
1 – Control*	0	3	101	151	102	152
2 – Low	6×10^{13}	3	201-202	251-252	203-204	253-254
3 – High	1.2×10^{14}	3	301-302	351-352	303-304	353-354

vg = vector genome. "*" = Control group was represented as Vehicle for the dPCR data analysis.
a. Necropsy Day 43.
b. Necropsy Day 85.

Flash-frozen tissues were collected from Day 43 and Day 85 necropsies and processed to extract total DNA according to METHOD-00108. Total DNA samples were then tested for AAV2-ITR and B2M vector genomes (copies/µg genomic DNA) according to METHOD-00112. Memos referenced are included in Attachment 4; additional clarifying memos are located in the study file.

2.1 Materials and Methods

2.1.1 Frozen Tissue Samples

Flash-frozen tissues collected from Day 43 and Day 85 necropsies were transferred to Analytics for analysis (see MEMO-00002 and MEMO-00008 in Attachment 4 for the list of tissue abbreviations):

Table 2. Flash-frozen Tissue Collection List

Tissue Type	Tissue Type
Brain	Ovary
Muscle, Skeletal, Bicep Brachii Right	Testis
Muscle, Skeletal, Bicep Brachii Left	Gross Lesion
Muscle, Skeletal, Tibialis Anterior Right	Heart
Muscle, Skeletal, Tibialis Anterior Left	Kidney
Ganglion, Dorsal Root, Cervical	Liver
Ganglion, Dorsal Root, Thoracic	Lung
Ganglion, Dorsal Root, Lumbar	Nerve, Femoral, Distal
Nerve Median, Distal	Nerve, Femoral, Proximal
Nerve Median, Proximal	Spinal Cord, Cervical
Nerve, Sciatic, Distal	Spinal Cord, Lumbar
Nerve, Sciatic, Proximal	Spinal Cord, Thoracic
Nerve, Optic	Spinal Cord, Sacral
Nerve, Ulnar, Distal	Spinal Roots, Lumbar
Nerve, Ulnar, Proximal	Spleen

2.1.2 DNA Extraction

Frozen tissue samples were weighed and lysis buffer ATL (DNeasy 96 Blood and Tissue Kit) volumes were determined based on tissue weight (see DEV-002235 and DEV-002415 for exceptions). Frozen tissue samples were added to lysis Buffer ATL (DNeasy 96 Blood and Tissue Kit) and homogenized using the QIAGEN TissueLyser II system. Following homogenization, Proteinase K was added to the lysed samples and samples were incubated overnight (20 hours ± 2 hours) at 56°C (± 2°C) and 800 rcf (± 100 rcf). RNase A was added to the tissue lysate samples to remove unwanted RNA. Buffer AL was then added to RNase A-treated homogenates.

Five hundred microliters (500 µL) of homogenate was transferred to the designated wells of a QIAcube sample block (S-block) and loaded to the QIAcube HT automated nucleic acid extraction system. A custom protocol using the DNeasy 96 Blood and Tissue Kit was utilized with the QIAcube HT Prep Manager. Total DNA was eluted in a final volume of 100 µL Buffer AE for downstream dPCR quantitation (DEV-002224). Retention tissue homogenate (if applicable) and DNA was stored at -85°C to -60°C (see DEV-002236 for exceptions).

2.1.3 DNA Quantification and Dilution

DNA concentrations were measured on a QIAxpert spectrophotometer system (QIAGEN) and stock samples were diluted to the appropriate target concentration (25 ng/µL) for dPCR testing (see DEV-002298 and DEV-002425 for exceptions). However, some samples could not be diluted to the target concentration due to low DNA recovery during extraction (< 25 ng/µL); these samples were tested at their original concentration without any further dilution. Following dPCR analysis, samples with observed copies/µL concentrations

exceeding the upper limit of quantification (ULOQ) of the assay for the target gene AAV2-ITR were further diluted as needed to obtain a result within the linear range. Actual DNA input values (µg) used for dPCR analysis are included in Attachment 2.

2.1.4 dPCR Assay

2.1.4.1 Primers and Probe Amplicon Targets

The AAV2-FAM (target AAV2-ITR amplicon) custom primers and probe mix was obtained from BioRad (Assay ID dEXD15274642, sequences unavailable). A proprietary *M. fascicularis* B2M-VIC housekeeping gene target primer-probe mix was also used (Thermo Fisher Scientific; Assay ID Mf07269219_s1, sequences unavailable).

2.1.4.2 Positive DNA Controls

Positive control DNA (gBlock-AAV2-ITR) containing the AAV2-ITR amplicon was obtained from IDT (Table 3). gBlock-AAV2-ITR was prepared at a concentration of 3.13×10^2 copies/µL and used as the positive plate control for AAV2-ITR dPCR plates. Monkey Cynomolgus Kidney Genomic DNA (Zyagen) was prepared at a concentration of 25 ng/µL and used as the positive plate control for the B2M dPCR plates (see DEV-002312 for exceptions).

Table 3. gBlock-AAV2-ITR sequence

Material	Sequence
gBlock -AAV2-ITR	CTT ACT ACG GTT CCA TGA CGG GCG TCG GGC GAC CTT TGG TCG CCC GGC CTC AGT GAG CGA GCG AGC GCG CAG AGA GGG AGT GGC CAA CTC CAT CAC TAG GGG TTC CTG CGG CCG CAC GCG TGG AGC TAG TTA TTA ATA GCG GCC GCA TGA GGG CCT ATT TCC CAT GAT TCC TTC ATA TTT GCA TAT ACG ATA CAA GGC TGT TAG AGA GAT AAT TGG AAT TAA TTT GAC TGT AAA CAC AAA GAT ATT AGT ACA AAA TAC GTG ACG TAG AAA GTA ATA ATT TCT TGG GTA GTT TGC AGT TTT AAA ATT ATG TTT TAA AAT GGA CTA TCA TAT GCT TAC CGT AAC TTG AAA GTA TTT CGA TTT CTT GGC TTT ATA TAT CTT GTG GAA AGG ACC ATT AGT TCT CGA GTG AGC GAG GGG TTG CTG TTG ATT GAA GAC TGT AAA GCC ACA GAT GGT CCC CTG CCT ACT AGA TTT TTT CCG GTG ATG CGG TTT TGG CAG TAC ATC AAT GGA CGT GGA TAG CGG TTT GAC TCA CGG GGA TTT CCA AGT CTC CAC CCC ATT GAC GTC AAT GGG AGT TTG TTT TGG CAC CAA AAT CAA CGG GAC TTT CCA AAA TGT CGT AAC AAC TCC GCC CCA TTG ACG CAA ATG GGC GGT AGA TGT GTA CGG TGG GAG GTC TAT ATA AGC AGA GCT GGT TTA GTG AAC CGT CAG ATC GAC GGT ATC GAT AAG CTT GAT TCG AGC CAC CAT GGT GAG CAA GGG CGA GGA GCT GTT CAC CGG GGT GGT GCC CAT CCT GGT CGA GCT GGA CGG CGA CGT AAA CGG CCA CAA GTT CAG CGT GTC CGG CGA GGG CGA GGG CGA TGC CAC CTA CGG CAA GCT GAC CCT GAA GTT CAT CTG CAC CAC CGG CAA GCT GCC CGT GCC CTG GCC CAC CCT CGT GAC CAC CCT GAC CTA CGG CGT GCA GTG CTT CAG CCG CTA CCC CGA CCA CAT GAA GCA GCA CGA CTT CTT CAA GTC CGC CAT GCC CGA AGG CTA CGT CCA GGA GCG CAC CAT CTT CTT CAA GGA CGA CGG CAA CTA CAA GAC CCG CGC CGA GGT GAA GTT CGA GGG CGA CAC CCT GGT GAA CCG CAT CGA GCT GAA GGG CAT CGA CTT CAA GGA GGA CGG CAA CAT CCT GGG GCA CAA GCT GGA GTA CAA CTA CAA CAG CCA CAA CGT CTA TAT CAT GGC CGA CAA

2.1.4.3 No Template Control

No Template Controls (NTC) were included in each dPCR plate in triplicate wells as a negative control to assess potential contamination of the reaction components. Ultra-purified H_2O was used as the NTC.

2.1.4.4 dPCR assay conditions

Before sample testing, a study-specific dPCR method for quantifying the AAV2-ITR gene target was qualified at AmplifyBio (A-579-02). dPCR was performed on study DNA samples using the QIAcuity Eight digital PCR system (QIAGEN) with QIAcuity 96-well Nanoplates (8.5k), which allows a single dPCR reaction to be analyzed across 8500 partitions for absolute quantitation of the target gene. dPCR reactions were prepared by adding a 3 µL in triplicate plate wells for each of the unknown study DNA samples (DNA input ~25 ng/µL or alternative concentration as applicable recorded in Attachment 2), positive plate control, or Ultra-purified H_2O (No-template Control; NTC) to 11 µL of the appropriate master mix (QIAcuity Probe PCR Kit). Master mixes were prepared separately for quantifying the AAV2-ITR gene and B2M housekeeping gene in singleplex reactions (total reaction volume of 14 µL; 12 µL loaded to the 8.5K nanoplate) using thermocycling conditions listed in Table 4.

Completed dPCR plates were analyzed in the QIAcuity Software Suite (v2.1) using a common threshold of 30 relative fluorescence units (RFU) for the AAV2-ITR target and a common threshold of 60 RFU for the B2M target. The threshold was adjusted as needed during data analysis to ensure appropriate separation between the positive and negative partitions. The QIAcuity Software calculated copies/µL based on positive partitions (RFU above the set threshold), and data was exported from the software for further analysis.

Table 4. dPCR Cycling Conditions

Step	Temperature (°C)	Duration (min:sec)	Number of Cycles
1	95	02:00	1X
2	95	00:15	40X
	60	00:30	

3.0 DATA CAPTURE AND DPCR ANALYSIS

All appropriate analytical data analyzed by QIAcuity are located in Attachment 2.

For the dPCR data analysis, a validated script-based dPCR Data Analysis Pipeline System (DPCRDAP) was utilized on raw data for generation of copies per µg of genomic DNA input for AAV2-ITR and B2M genomes in each tissue sample from all three study groups (Group 1 [Control/Vehicle]; Group 2 [Low Dose] and Group 3 [High Dose]). During sample analysis using the DPCRDAP, nerve samples were renamed with five-letter codes for reporting purposes (see MEMO-00008 in Attachment 4 for details). The dPCR Data Analysis Pipeline calculates reportable values from the raw data exported from the QIAcuity

system. The reportable value outputs provided by Module 1 were used to generate a final data table which was used as an input for the statistical calculations (see Attachment 2).

Statistical analysis was performed by the test site (Precision Consulting, LLC) and results are included in Attachment 3.

3.1 dPCR Assay Performance Quality Control

The summary of the plate acceptance criteria is outlined in Table 5. In certain instances, data point(s) were considered for censoring following manager review.

Table 5. Plate Acceptance Criteria

Parameter	Expected Result	Action
Sample Replicates	CV% ≤ 20%	Flag for review if not met, acceptance of result at discretion of management or Subject Matter Expert (SME). One of the three replicates was censored if it was an outlier.
Positive Control Result	Positive (> 4 positive partitions)	Flag for review if not met, acceptance of result at discretion of management or SME. One of the three replicates may be censored if it was an outlier.
NTC Result	Negative (≤ 4 positive partitions)	Flag for review if not met, acceptance of result at discretion of management or SME. One of the three replicates was censored if it was an outlier.
% of positive partitions	< 95%	Flag for review/ retest at higher dilution per recommendation of management or SME.
% of valid partitions	> 80%	If < 80% flag for review by management or SME.
Unexpected results	Situation-specific	Notify management or SME (and Study Director)

The assay limits established during qualification (A-579-02) were considered as well during data review (Table 6).

Table 6. Assay Limits for the AAV2-ITR Gene Target

Limits	Concentration (copies/uL)
Limit of Detection (LOD)	6.93 copies/µL
Lower Limit of Quantitation (LLOQ)	14.9 copies/µL
Upper Limit of Quantitation (ULOQ)	16800 copies/µL

Samples were considered for repeats if any the following parameters were true upon review:

- The percentage CV was still above the accepted criteria after censoring.
- A sample result was above the ULOQ and not in the assay's linear range. The sample was diluted before being retested.
- A plate with % Valid Partitions ≤ 80%. The entire plate was repeated.
- Any sample tested below the target input DNA concentration with a result < LLOQ was re-extracted and retested if an additional tissue sample was available.

- Samples < LLOQ were otherwise not considered for censoring or repeat testing.
- A sample had an expected value of zero for a target (e.g., samples treated with the 'vehicle control') but a positive result was observed.

Results selected for inclusion in the final data table which did not meet the above listed requirements are indicated in MEMO-00017 (Attachment 4).

4.0 DEVIATIONS

DEV-002224: Batch A-579-01.006 tissue samples from Animal 252 were being extracted for DNA on 10Oct2023 according to METHOD-00108 when the QIAcube disconnected from the computer and the run was stopped. Since the run was disconnected right before the final step where DNA is eluted from the filter plate, the elution was performed manually in concurrence with the Study Director. Due to the disconnection, manual elution was performed, and 33 samples had “NA” results on the QIAxpert, leading to re-extraction of the leftover homogenate (Batch A-579-01.201). There were 6 samples that still didn't have sufficient DNA to obtain a useable measurement on the QIAxpert, and those samples were manually eluted a second time from the Batch A-579-01.201 filter plate. Although measurements were obtained for all samples from Animal 252, 11 nerve and one spinal cord samples had low concentrations with > 20%CV. Samples were used for dPCR analysis and results were obtained. The final impact is minor.

DEV-002235: For Batch A-579-01.003 during execution of METHOD-00108, Sample A-579-01.201.BRAN.02Aug2023.03 had 540 µL Buffer ATL, 50 µL Proteinase K and 10 µL RNase A added during the tissue homogenization procedure. Based on tissue weight and the indicated buffer volumes in the SSM, the incorrect volumes of Proteinase K and RNase A were added to the tissue sample (10µL less for Proteinase K and 2µL less for the RNase A). There is minimal impact on the study based on the events described in the deviation. An acceptable amount of quality DNA was extracted for downstream PCR reactions. The difference in the volume of the Proteinase K and RNase A that should have been added was small enough that the difference in extraction efficiency was negligible.

DEV-002236: The nucleic acid extraction in METHOD-00108 indicated to store DNA samples at -85°C to -60°C after QIAxpert readings were complete. The DNA samples from Batch A-579-01.002 for Animal 151 were inadvertently left at room temperature over the weekend instead of being stored at -85°C to -60°C. There was no study impact due to the samples inadvertently left at room temperature instead of being stored at -85°C to -60°C as per the method. There was no impact to the concentration and purity of the DNA samples analyzed after they were returned to the correct storage conditions. Hence there was no impact to the integrity of the data acquired from these samples.

DEV-002298: The nucleic acid extraction in METHOD-00108 indicated that for dilution calculations the target concentration for DNA was 25 ng/µL, if possible, based on the starting average QIAxpert concentration obtained. Due to a transcription error, two samples

were inadvertently diluted when they should have been left undiluted (concentrations were < 25 ng/µl). Sample A-579-01.252.NVSP.02Aug2023.04 had an average concentration of 0.75 ng/µl (entered as 51 ng/µl) and sample A-579-01.252.NVUD.02Aug2023.11 had an average concentration of 4.7 ng/µl (entered as 34.9 ng/µl). There was no impact to the study due to the error in dilution of DNA for the Animal 252 nerve samples (Nerve, ulnar, distal [right, distal]; nerve, sciatic, proximal [left, distal]) as described in the deviation. The reportable value of copies/µg of DNA input could be back calculated from the actual DNA concentration once diluted. Thus, there was no impact to the data integrity.

DEV-002312: The dPCR SSM METHOD-00112 indicated that positive controls were to be labeled "Pos Con" and that the gBlock (AAV2-ITR) would be the positive control for the AAV2-ITR target and AB-gDNA (NHP DNA) would be the positive control for the B2M target. For Batch A-579-01.004 dPCR, there was a comment on the dPCR plate loading tab that the incorrect positive control was added for plates A-579-01.004.CLC.P05.02 and A-579-01.004.CLC.P06.03. Due to this error the positive control was not positive for these plates (result was less than four positive partitions). There is minimal impact to the study due to the wrong positive control being added to the dPCR plates. The positive control was added as a control to determine if dPCR worked and these plates showed positive copies/ µL read out for all the samples on the plates proving that the dPCR worked. Hence, missing the positive control on the plates did not impact the integrity of the data.

DEV-002848 (Addendum to DEV-002415): METHOD-00108 A-579-01 Study Specific Method for DNA Extraction specified to determine appropriate volumes for TissueLyser homogenization and QIAcube extraction based on tissue starting weight (volumes recorded in Table 3). Incorrect buffer volumes were inadvertently used for a sample based weight during homogenization and QIAcube extraction. Sample A-579-01.102.LVER.12Sep2023.02 was recorded for DNA extraction on 23Oct2023 instead of sample A-579-01.102.LVER.12Sep2023.01 that was used. This is an addendum to DEV-002415. There is no study impact due to the incorrect buffer volumes inadvertently used for the sample from Animal 102 during homogenization and QIAcube extraction. The differences in buffer amounts did not have any impact on the DNA extraction. Samples were processed and results showed an ample amount of quality DNA was extracted from this sample to continue processing downstream for dPCR. Hence, there was no impact to the integrity of the data.

DEV-002415: METHOD-00108 A-579-01 Study Specific Method for DNA Extraction specified to determine appropriate volumes for TissueLyser homogenization and QIAcube extraction based on tissue starting weight (volumes recorded in Table 3). Incorrect buffer volumes were inadvertently used for two samples based on their weight during homogenization and QIAcube extraction. Sample A-579-01.102.SCSR.12Sep2023.01 was recorded for DNA extraction on 23Oct2023 instead of sample A-579-01.102.SCSR.12Sep2023.03 that was used. Sample A-579-01.102.SCSR.12Sep2023.01 weighed 0.02722g whereas, sample A-579-01.102.SCSR.12Sep2023.03 weighed 0.06271g. Similarly, Sample A-579-01.354.DRGL.14Sep2023.01 was recorded for DNA extraction

instead of A-579-01.354.DRGL.14Sep2023.02. Sample A-579-01.354.DRGL.14Sep2023.01 weighed 0.03626g instead of sample A-579-01.354.DRGL.14Sep2023.02 that weighed 0.02907g. There is no study impact due to the incorrect buffer volumes inadvertently used for the two samples from Animal 102 and 354 during homogenization and QIAcube extraction. The differences in buffer amounts did not have any impact on the DNA extraction. Samples were processed and results showed an ample amount of quality DNA was extracted from this sample to continue processing downstream for dPCR. Hence, there was no impact to the integrity of the data.

DEV-002425: The nucleic acid extraction in METHOD-00108 indicated that for dilution calculations the target concentration for DNA was 25 ng/µL, if possible, based on the starting average QIAxpert concentration obtained. The QIAxpert readings for the average DNA concentration calculation were not copied correctly for some Animal 252 tissue samples (brain, skeletal muscle [tibialis anterior, right], skeletal muscle [tibialis anterior, left], dorsal root ganglion [cervical, thoracic, and lumbar sections], nerve median distal, nerve median proximal). This led to the incorrect concentration of DNA to be calculated and copied to the dilution file. There is no study impact based on the events described in the deviation. The difference in concentration after the dilution is negligible and did not affect downstream sample processing and data analysis. These concentrations were used in downstream calculations but were able to reconstruct and recalculate based on the original concentration and hence there was no impact to the data.

5.0 RESULTS

Final Data Table: Attachment 2
Precision Principal Investigator Report: Attachment 3

Biodistribution pattern of AAV2-ITR encoded vector copies per µg DNA:

Vector genome copy number (copies per µg of DNA) were detected in all of the low dose (Group 2; 6×10^{13} vg) and high dose (Group 3; 1.2×10^{14} vg) animal tissues harvested at both the Day 43 (Figure 1) and Day 85 (Figure 2) time points evaluated. The data represented in this report are not normalized to the housekeeping gene B2M. The estimated mean log vector genome copies in low dose group (Group 2; 6×10^{13} vg) was 4.95, whereas in High dose group (Group 3; 1.2×10^{14} vg) was 5.06 compared to the control group (0.18 mean log; Group 1) indicating no dose response withing dosed groups, but significant dose response between control and dosed groups. There were no significant sex-related differences observed in the mean log copies of the vector genome in males or females at Day 43 or Day 85 (see Attachment 2 for details).

Table 7. Estimated Mean and 95% Confidence Interval (CI) Across Groups, Timepoint and Sex

Comparator	Category	Estimated Mean Log	Standard Error	Lower 95% CL	Upper 95% CI
Group	High Dose	5.07	0.16	4.73	5.40
Group	Low Dose	4.95	0.16	4.61	5.29
Group	Vehicle	0.18	0.23	-0.30	0.66
Timepoint	Day 43	4.33	0.65	2.96	5.70
Timepoint	Day 85	3.75	0.65	2.38	5.12
Gender	Female	4.00	0.66	2.62	5.38
Gender	Male	4.08	0.66	2.70	5.47

Group 1 (Control; 0 vg per animal)

Vector genomes were either ≤ 0 copies/µg DNA, Indeterminate (IND) or below quantification Limit (BQL) in any of the control (Group 1) animal tissues at both Day 43 and Day 85 time points.

Group 2 (Low Dose; 6 × 10^{13} vg per animal)

Highest vector genome copies were generally observed in the liver tissue, spleen, and central nervous system (CNS) tissues, such as dorsal root ganglia, spinal cord, and brain, compared to other organs at Day 43 (Figure 1). All the nerve tissues (Femoral, Median, Sciatic, Ulnar) showed high mean log vector copies per µg DNA at Day 43 (Figure 1) for low dose animal tissues which persisted until Day 85 (Figure 2).

Group 3 (High Dose; 1.2 × 10^{14} vg per animal)

High dose (Group 3) showed a similar level of vector copy number than low dose Group 2 in all tissues tested indicating minimal dose response except for DRG cervical and spinal cord cervical. Highest mean vector copies per µg DNA (6-8 mean $\log_{10}$) was generally observed in the liver tissues, spleen, and CNS organs, such as dorsal root ganglia and spinal cord, as compared to other organs at Day 43 (Figure 1). The same tissues had a slightly lower mean vector copies of 4-6 logs at Day 85 (Figure 2) compared to Day 43 except DRG lumbar and liver. All the nerve tissues (Femoral, Median, Sciatic, Ulnar) showed high accumulation of the vector (4-6 mean $\log_{10}$ vector copies per µg DNA) at Day 43 for high dose animal tissues and persisted until Day 85.

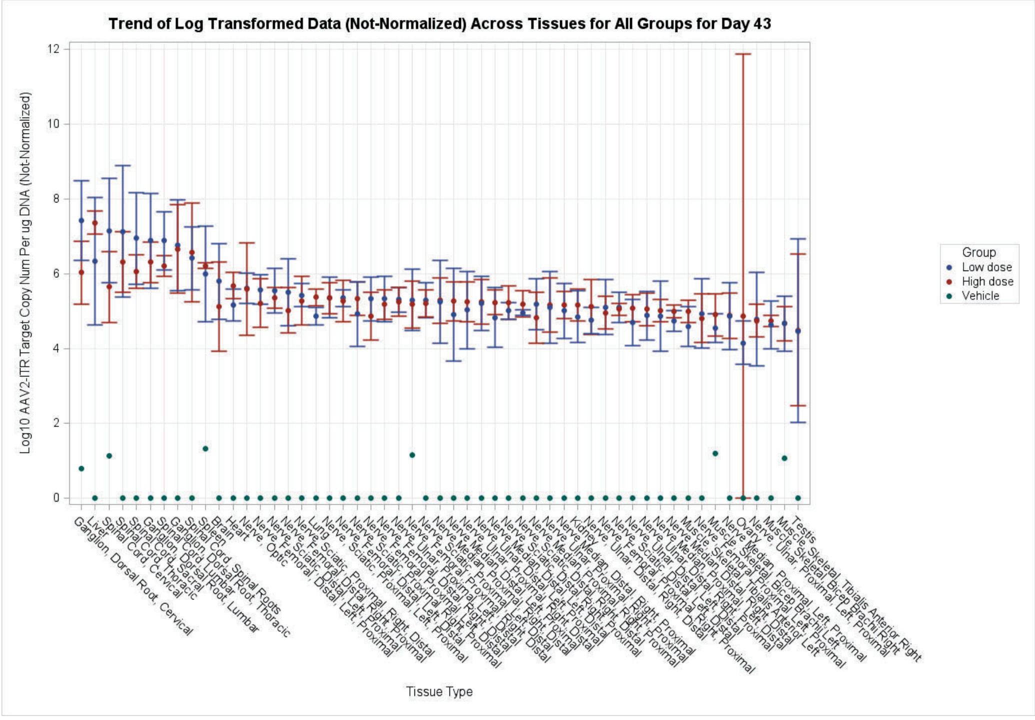

Figure 1. Trend of Log Transformed AAV2-ITR Mean Target Copy number per μg DNA (Not Normalized to the housekeeping Gene B2M) Across Tissues for All Groups for Day 43. Error bars for the low dose and high dose groups are calculated based on the 95% confidence intervals for the mean of each of these groups. The error bars have been omitted for the vehicle group.

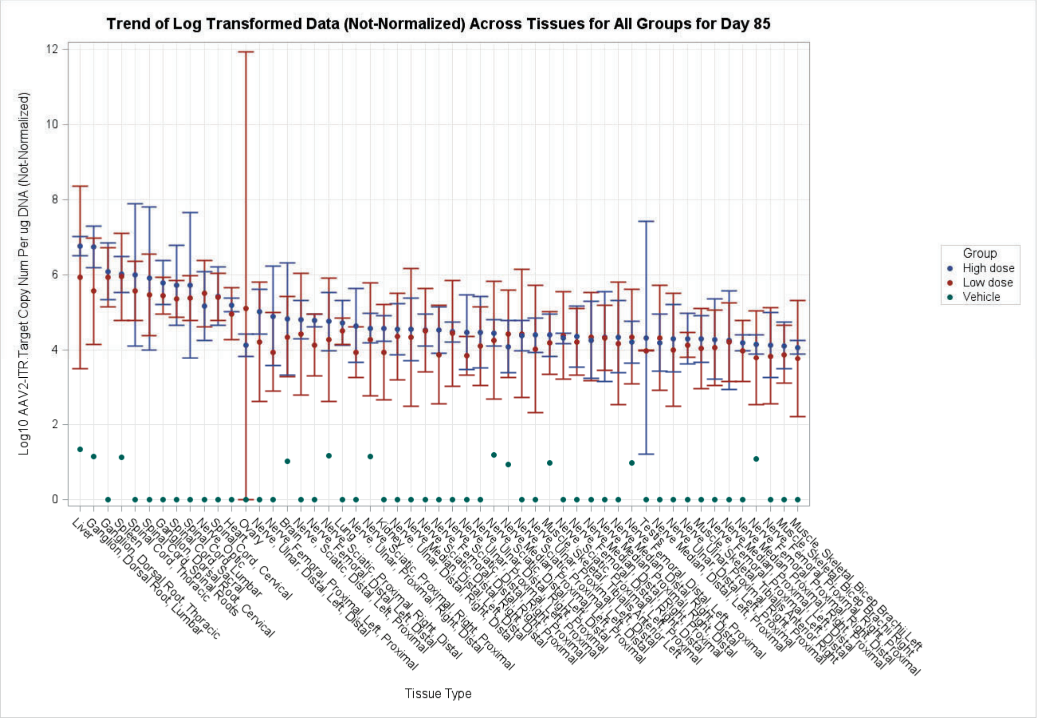

Figure 2. Trend of Log Transformed AAV2-ITR Mean Target Copy number per µg DNA (Not Normalized to housekeeping Gene B2M) Across Tissues for All Groups for Day 85. Error bars for the low dose and high dose groups are calculated based on the 95% confidence intervals for the mean of each of these groups. The error bars have been omitted for the vehicle group.

6.0 CONCLUSION

Overall, the biodistribution pattern of the vector AAV2-ITR of ARM-101 test article upon intrathecal slow infusion showed greater prevalence of vector on Day 43 compared to vehicle control (mean log copies higher than the Day 85 mean) in the CNS (spinal cord, dorsal root ganglia, and brain), liver and heart. All the nerve tissues (Femoral, Median, Sciatic, Ulnar) and spleen showed high vector copies per µg DNA at Day 43 and persisted until Day 85. There was no dose response observed in dosed groups (Low 6×10^{13} vg or High 1.2×10^{14} vg per animal Dose) but showed very high vector copies when compared to the Vehicle Control group. There were no sex-related differences observed in the mean copies of the vector genome in males and females.

Attachment 1. Study Specific Methods

A-579-01 Study Specific Method For DNA Extraction and Isolation From Frozen Tissue Samples

Batch ID:

Purpose

This study-specific method (SSM) describes the procedure for DNA extraction and isolation per A-579-01 study requirements. The scientist's electronic signature (e-sig) under each section signifies they have followed the instructions as written, verified that the materials and equipment remained the same as those listed in the tables in the Recording tab, and all equipment has been properly calibrated. Any deviation from this method will be approved by the study director.

Instructions

Enter the batch ID at the top of every tab in this workbook. In the Recording tab, enter the samples that will be processed as part of this batch ID. Using eInfotree in the menu bar, convert the workbook to an eInfotree wrapped copy. **Save to the network location: \\ab-file1\AnalySci\General Data\A-579-01**

Sub-folders may be used to organize electronic forms.

Initiate the procedure by following the instructions in the TLII Instructions tab. Proceed as the experiment progresses from tab to tab until the batch of samples is complete. Fill in material and equipment details in the Recording tab and verify each day of use as applicable. Save and sign using eInfotree electronic signatures after each session whenever data is added or changed.

Additional comments may be made throughout the workbook as needed, and signed using eInfotree.

Yellow highlighted cells indicate locations where user input (enter text) is required.

Additionally Tables 1, 2, 3, and 4 must be filled out.

DNA Sample Processing - Recording

Batch ID:

The performing operator will apply e-signature using eInfotree (do for each day if multiple days)
Verifier/record (if applicable) will apply e-signature using eInfotree (do for each day if multiple days)

Record and sign for materials and equipment on each day items are used. If needed, additional day(s) can be signed on for individual components (highlight cells for items used and apply e-sig to highlighted cells).

Table 1: Materials

Name	Manufacture	Catalog No.	Lot No.	Expiration Date	Record Day 1	Record Day 2
Stainless Steel Beads, 5 mm	QIAGEN	69989				
Collection Microtubes, racked	QIAGEN	19560				
Collection Microtube Caps	QIAGEN	19566			Apply eSig	
DNeasy 96 Blood and Tissue Kit	QIAGEN					Apply eSig
Buffer ATL	QIAGEN					
Proteinase K	QIAGEN					
RNase A	QIAGEN	19101				
Buffer AL	QIAGEN					
Buffer AW1	QIAGEN					
Buffer AW2	QIAGEN					Apply eSig
Absolute Ethanol (200 proof), Molecular Biology Grade						
Buffer AE	QIAGEN					
QIAxpert Slides	QIAGEN	990700				

Table 2: Equipment ("NA" if not used)

Description	Manufacturer	ID/Serial Number	Record Day 1	Record Day 2
Freezer -60 to -85°C				
Biosafety Cabinet (BSC) Class II				
Pipette				
Pipette				
Pipette				
Pipette				Apply eSig
Pipette				
Pipette			Apply eSig	
Multichannel Pipette				
Multichannel Pipette				
Shaking incubator	VWR			
Shaking incubator	VWR			
TissueLyser II	QIAGEN			
TissueLyser II	QIAGEN			
Centrifuge				
QIAcube HT	QIAGEN			Apply eSig
QIAxpert	QIAGEN			

Table 3: Samples to be processed and required information

*Position refers to the collection microtube rack position (if applicable) and QIAcube plate position.

Position*	Sample ID	Tissue Weight (g)	Volume Buffer ATL (µL)	Volume Proteinase K (µL)	Volume RNase A (µL)	Volume Buffer AL (µL)
A1						
B1						
C1						
D1						
E1						
F1						
G1						
H1						
A2						
B2						
C2						
D2						
E2						
F2						
G2						
H2						
A3						
B3						
C3						
D3						
E3						
F3						
G3						
H3						
A4						
B4						
C4						
D4						
E4						

F4						
G4						
H4						
A5						
B5						
C5						
D5						
E5						
F5						
G5						
H5						
A6						
B6						
C6						
D6						
E6						
F6						
G6						
H6						
A7						
B7						
C7						
D7						
E7						
F7						
G7						
H7						
A8						
B8						
C8						
D8						
E8						
F8						
G8						
H8						
A9						
B9						
C9						
D9						
E9						
F9						
G9						
H9						
A10						
B10						
C10						
D10						
E10						
F10						
G10						
H10						
A11						
B11						
C11						
D11						
E11						
F11						
G11						
H11						
A12						
B12						
C12						
D12						
E12						
F12						
G12						
H12						

Quality Control will apply e-signature using eInfotree
Technical Review will apply e-signature using eInfotree

TissueLyser Homogenization for DNA Samples

Batch ID: ______

The performing operator will apply e-signature using eInfotree (for each section/day as needed).
Verifier/record (if applicable) will apply e-signature using eInfotree (for each section/day as needed).

Workspace Preparation

1 Prepare the workspace (BSC) by wiping down the interior surfaces and required equipment with a Nuclease cleaner then 70% ethanol.
2 Turn on the plate shaker and set the temperature to 56°C.
3 Place the bottle of Buffer ATL on the plate shaker to ensure precipitated material dissolves. Leave bottle until complete dissolution.
4 Remove samples from the freezer and keep them on dry ice.

Tissue Weight Determination

1. Record Weights

1.1 Record weights for each sample in Table 3 - weights may be taken prior to homogenization or transcribed from other forms (e.g. Tissue Weighing eInfotree forms using empty and full tube weights)
Weights transcribed from (list filename or N/A): ______

1.2 Determine appropriate volume of reagents to use per the following key:
Enter volumes in Table 3 in the Recording tab.

Sample size (g)	≤0.030	0.031 - 0.040	0.041 - 0.050	0.051 - 0.060	0.061 - 0.070	0.071 - 0.080	0.081 - 0.090	0.091 - 0.100	0.101 - 0.110
Buffer ATL Volume (µL)	270	360	450	540	630	720	810	900	990
Proteinase K Volume (µL)	30	40	50	60	70	80	90	100	110
Rnase A (µL)	6	8	10	12	14	16	18	20	22

Sample size (g)	0.111 0.120	0.121 – 0.130	0.131 – 0.140	0.141 – 0.150	0.151 – 0.160	0.161 – 0.170	0.171 – 0.180
Buffer ATL Volume (µL)	1080	1170	1260	1350	1440	1530	1620
Proteinase K Volume (µL)	120	130	140	150	160	170	180
Rnase A (µL)	24	26	28	30	32	34	36

Tissue Homogenization Using the TissueLyser II

2. Prepare Sample Tubes

2.1 Dispense one 5mm stainless steel bead into each Collection Sample tube or 2 mL tube (samples over 0.110 g) being used per Table 3.
2.2 Add Buffer ATL to the appropriate wells of each tube per Table 3.
2.3 Transfer each tissue from its tube to the indicated location/tube (Table 3) ensuring that the tissue is not thawed prior to submerging in Buffer ATL.
2.5 Securely seal each strip of collection microtubes using collection microtube caps (or cap 2 mL tubes appropriately).

3. Homogenize Using TissueLyser

3.1 Load sample tubes into the TissueLyser adapter set then onto the TissueLyser II.
3.2 Run samples at 30Hz for 5 minutes.
3.3 Remove the samples post run and check that all sample tube caps are still tight.
3.4 Load samples onto the TissueLyser (rotate racks so innermost tubes are on the outside) and repeat step 3.2
3.5 Remove the samples post run and check that all sample tube caps are still tight. Centrifuge at 1000 x g for 30 seconds to remove droplets from lid.

4. Proteinase K Treatment and Incubation

4.1 Add the appropriate amount of proteinase K to each tube containing homogenized per Table 3 in Recording tab.
4.2 Securely seal each strip of tubes using caps and parafilm. Invert the tubes 2-3 times to mix.
4.3 Incubate samples at 56°C ± 2°C and 800 rcf ± 100 rcf for 20 hours ± 2 hours.
Start Temperature: ______ Stop Temperature: ______
Start Time: ______ Stop Time: ______
4.4 Remove sample from incubator and vortex briefly 3-5 seconds. If lysates appear viscous or gelatinous, vortex vigorously for approximately 1-2 minutes (add comment for impacted samples and action taken).
4.5 Place samples on wet ice for 2 minutes ± 1 minute.

Start Time: ______ Stop Time: ______
Maintain samples at room temperature after cooling.

5. RNase Treatment

5.1 Centrifuge samples at 1000 x g for 30 seconds to remove droplets from lids and precipitate any unhomogenized tissue.
5.2 Add the appropriate volume of RNase A (Table 3 from Recording tab) to samples, re-cap, and invert the collection microtube racks ~2-3 times to mix.
5.3 Incubate samples at room temperature for 5 minutes ± 1 minute.

Start Time: ______ Stop Time: ______

5.4 Centrifuge at 1000 x g for 30 seconds to remove droplets from lids and precipitate any unhomogenized tissue.
5.5 Proceed to DNA extraction or store samples at -85°C to -60°C if not using immediately.
Proceeding: ("Yes" or "NA") ______ Storage Location ("NA" if not storing) ______

Quality Control will apply e-signature using eInfotree
Technical Review will apply e-signature using eInfotree

Comments:

Nucleic Acid Extraction Utilizing the QIAcube HT

Batch ID: ______

The performing operator will apply e-signature using eInfotree (for each section/day as needed).
Verifier/record (if applicable) will apply e-signature using eInfotree (for each section/day as needed).

Workspace Preparation

1 Prepare all work areas (bench and/or cabinets) by wiping down the surfaces and required equipment with a Nuclease cleaner then 70% ethanol.
2 Prepare the QIAcube instrument by wiping down the worktable with a Nuclease cleaner then 70% ethanol.

Experiment Setup

1. Setup Experiment on the QIAcube HT

1.1 Power on the QIAcube HT and computer. Login with your user credentials
1.2 Create a new Custom DNeasy Blood and Tissue CR2012 V2 experiment and save as "BatchID.initials.unitID.runs" and record the experiment name.
Name: ______
1.3 Verify the file was saved to the network location as: \\ab-file1\AnalySci\Qiacube\Experiments
Optional subfolder (e.g. \Study no.\DNA): ______
1.4 Follow the on-screen setup prompts.
1.5 Navigate to the Setup tab and enter the kit lot number and kit expiration date.
1.6 Select the following options:
Sample Type: **Tissue**
Pre-treatment: **Any**
Select Protocol: **Custom DNeasy 96 Blood and Tissue- 2x ETOH wash**
Vacuum Performance Check: **Selected**
1.7 Verify the following Advanced Settings:
Vacuum Intensity: **35kPa**
Vacuum Time: **300s**
Total Elution Volume: **100µL**
Number of Elution Steps: **1**
Top Elute: **Leave Unselected**
1.8 Navigate to the Labware Selection tab and select sample input.
Import sample IDs from a sample input file and record file name: ______
Input labware selection: **QIAGEN S-Block 96 Well**
Output labware selection: **Use new output labware**
1.9 Verify Sample IDs with Table 3 from Recording tab
1.10 Navigate to the Assignment tab. Click and drag to highlight the number of columns being used, starting from column 1, for the sample input (this should match with the S-block layout, i.e. Table 3). Repeat for the output section with the same number of columns highlighted. Click Assign.

2. Prepare and Stage Samples and Materials on the QIAcube HT

2.1 Navigate to the Worktable tab and follow the on-screen prompts.
2.2 Ensure all reagents are prepared according to the instructions listed on the bottle. If reagents have not been prepared, prepare them according to the instructions on the bottle and record in the DNeasy Blood and Tissue 96 Reagent Prep Form.
2.3 Transfer reagents to QIAGEN reagent troughs according to the on-screen prompts on the Worktable screen. Ensure correct location placement on the instrument.
2.4 Add Buffer AL to RNase-treated homogenate. Determine appropriate volume of reagents to use per the following key:
Enter volumes in Table 3 in the Recording tab.
Depending on volumes required, total volume may exceed the homogenate original tube - as needed, sample homgenate will be transferred to a tube of sufficient capacity to accomodate the buffer AL addition.

**Sample size (g)**	**≤0.030**	**0.031 - 0.040**	**0.041 - 0.050**	**0.051 - 0.060**	**0.061 - 0.070**	**0.071 - 0.080**	**0.081 - 0.090**	**0.091 - 0.100**	**0.101 - 0.110**
**Buffer AL Volume (µL)**	300	400	500	600	700	800	900	1000	1100
**Sample size (g)**	**0.111 0.120**	**0.121 – 0.130**	**0.131 – 0.140**	**0.141 – 0.150**	**0.151 – 0.160**	**0.161- 0.170**	**0.171 – 0.180**		
**Buffer AL Volume (µL)**	1200	1300	1400	1500	1600	1700	1800		

2.5 Transfer 500 µL of each homogenate to the appropriate wells of a new S-block (Table 3). Save any remaining homogenate (if applicable) at -85°C to -60°C.
Storage Location for retention homogenate: ______
2.6 Load the S-block containing samples into the QIAcube ensuring proper positioning and orientation per the worktable.
2.7 Ensure experimental layout is correct and verify correct addition of all plasticware on the instrument deck.

QIAcube HT Extraction

3. Nucleic Acid Extraction: DNA

3.1 Click the Run button to begin the extraction. Monitor the instrument to completion.
3.2 Check the filter plate during the vacuum performance check to ensure samples passed through. Repeat the vacuum step as needed.
Note: Use a pipette tip to GENTLY scrape the top of the filter if needed to aid in the complete pass through of the sample.
Make a note of which samples required scraping in the comments area on this sheet.
3.3 Upon completion of the run, create an advanced report. Save as "BatchID.initials.unitID.runs" and record the report name.
Name: ______
3.4 Verify the file was saved at the network location as: \\ab-file1\AnalySci\Qiacube\Reports
Optional subfolder (e.g. \Study no.\DNA): ______
3.5 Cover the elution plate with the lid and remove samples from the QIAcube HT.
3.6 Maintain elution plate on wet ice or cold block in a BSC or PCR Workstation. Prepare QIAxpert slides as needed (See QIAxpert Instructions tab).
3.7 Make one 25 µL aliquot of stock on a second elution plate. Cap sample rows with elution microtube caps for the stock DNA and aliquots. Label the columns and the plates.
Note: Maintain samples on wet ice until completion of the QIAxpert tab in case samples need to be repeated.

Quality Control will apply e-signature using eInfotree
Technical Review will apply e-signature using eInfotree

Comments:

Nucleic Acid Analysis Utilizing the QIAxpert

Batch ID: ____________

The performing operator will apply e-signature using eInfotree (for each section/day as needed).
Verifier/record (if applicable) will apply e-signature using eInfotree (for each section/day as needed).

Workspace Preparation

1 Prepare all work areas (bench and/or cabinets) by wiping down the surfaces and required equipment with a Nuclease cleaner then 70% ethanol.

UV Spectrophotometer Measuring DNA Concentration

1. Experiment Setup

1.1 Maintain samples on wet ice or cold block.
1.2 Power on the QIAxpert and wait until after automatic system function check before use.
1.3 Label slides sequentially with a number (ex: 01, 02, etc.).
1.4 Transfer 2µL of Buffer AE to position H2 on the slides as the blank.
1.5 Transfer 2µL of samples to corresponding position(s) per Slide Layout (Table 4). Samples will be analyzed in triplicate, which can be done within a single slide or across multiple slides.

2. Sample Measurement

2.1 Select measure to start a new experiment.
2.2 Select the operator performing the analysis.
2.3 Select the appropriate wells being analyzed as blanks or containing sample. Push the next arrow when this is complete.
2.4 When prompted, insert the slide into the chamber.
2.5 Title the experiment “Batch ID.initials.UnitID.runs”. Select the next arrow.
2.6 Enter sample IDs. This can be done from a .txt File corresponding to the appropriate experiment, or manually.
Location of files used (N/A if sample IDs were entered manually): ____________
2.7 Select the “next” arrow when completed.
2.8 Verify Sample IDs input are the same as recorded for the slide (Slide Layout, Table 4).
2.9 Select the analyze app A260 DNA.
2.10 Discard the slide when prompted and analyze the data.
2.11 Repeat Steps 2.1-2.10 for each slide.
2.12 Proceed to downstream calculations per SOP-00408. CV% will be calculated and samples >20% CV will be repeated in triplicate if censoring is not possible. One of three replicates may be censored (whether to pass CV% or in the case of an aberrant result such as a negative reading or a "N/A" response.) If a second triplicate reading does not result in a passing sample result, defer to Study Director for guidance or acceptance of results. Note, when completing FORMTEMP-00673 for dilution calculations, the target concentration for DNA is 25 ng/µL, if possible based on the starting average QIAxpert concentration obtained.
2.13 After confirming no repeats are required, store samples at -85°C to -60°C
Storage Location of DNA and aliquot(s): ____________

Quality Control will apply e-signature using eInfotree
Technical Review will apply e-signature using eInfotree

Table 4: Slide Layout (additional sections may be added as needed)

	Slide Number:			Slide Number:	
	1	2		1	2
A			A		
B			B		
C			C		
D			D		
E			E		
F			F		
G			G		
H		BLANK	H		BLANK
	Slide Number:			Slide Number:	
	1	2		1	2
A			A		
B			B		
C			C		
D			D		
E			E		
F			F		
G			G		
H		BLANK	H		BLANK
	Slide Number: ___________			Slide Number: ___________	
	1	2		1	2
A			A		
B			B		
C			C		
D			D		
E			E		
F			F		
G			G		
H		BLANK	H		BLANK
	Slide Number: ___________			Slide Number: ___________	
	1	2		1	2
A			A		
B			B		
C			C		
D			D		
E			E		
F			F		
G			G		
H		BLANK	H		BLANK
	Slide Number: ___________			Slide Number: ___________	
	1	2		1	2
A			A		
B			B		
C			C		
D			D		
E			E		
F			F		
G			G		
H		BLANK	H		BLANK
	Slide Number: ___________			Slide Number: ___________	
	1	2		1	2
A			A		
B			B		
C			C		
D			D		
E			E		
F			F		
G			G		
H		BLANK	H		BLANK
	Slide Number: ___________			Slide Number: ___________	
	1	2		1	2
A			A		
B			B		
C			C		
D			D		
E			E		
F			F		
G			G		
H		BLANK	H		BLANK

A-579-01 Study Specific Method For dPCR

Batch ID:

Purpose
This study-specific method (SSM) describes the procedure for analyzing DNA on the QIACuity per A-579-01 study requirements and SOP-00465. The scientist's electronic signature (e-sig) under each section signifies they have followed the instructions as written, verified that the materials and equipment remained the same as those listed in the tables in the Recording tab, and all equipment has been properly calibrated. Any deviation from this method will be approved by the study director.

Instructions
Enter the batch ID at the top of every tab in this workbook. In the Recording tab, enter the samples that will be processed as part of this batch ID. Using eInfotree in the menu bar, convert the workbook to an eInfotree wrapped copy. **Save to the network location: \\ab-file1\AnalySci\General Data\A-579-01**
Sub-folders may be used to organize electronic forms.

Initiate the procedure by following the instructions in dPCR Plate Loading tab. Proceed as the experiment progresses from tab to tab until the batch of samples is complete. Fill in material and equipment details in the Recording tab and verify each day of use as applicable. Save and sign using eInfotree electronic signatures after each session whenever data is added or changed.

Additional comments may be made throughout the workbook as needed, and signed using eInfotree.

Yellow highlighted cells indicate locations where user input (enter text) is required.

Additionally Tables 1, 2, 3, and 4 must be filled out.

dPCR - Recording

Batch ID:

The performing operator will apply e-signature using eInfotree (do for each day if multiple days)

Verifier/record (if applicable) will apply e-signature using eInfotree (do for each day if multiple days)

Record and sign for materials and equipment on each day items are used. If needed, additional day(s) can be signed on for individual components (highlight cells for items used and apply e-sig to highlighted cells). Lines may be added or removed as need for materials or equipment tables.

Table 1: Materials

Name	Manufacture	Catalog No.	Lot No.	Expiration Date	Record Day 1
QIAcuity Probe PCR kit	QIAGEN	250102			Apply eSig
4X Probe PCR Master Mix	QIAGEN	1120965			
RNase-Free Water	QIAGEN				
Ultra-purified H2O (upH2O)	Invitrogen				
20X AAV2-ITR Primer-probe	BIO-RAD				
20X B2M Primer-probe					
QIAcuity Nanoplate 8.5k 96-well	QIAGEN	250021			
QIAcuity Nanoplate Seals	QIAGEN	MH-93953-1			
96-well PCR plate	Applied Biosystems	4346907			
96-well PCR plate seal	Applied Biosystems	4311971			
gBlock*	AB	NA			
AB-gDNA*	AB	NA			

*On the dPCR plates, this is labeled "Pos Con" and will be the positive control for AAV2-ITR (gBlock) or B2M (AB-gDNA)

Table 2: Equipment ("NA" if not used)

Description	Manufacturer	ID/Serial Number	Record Day 1
QIAcuity Eight	QIAGEN		Apply eSig
Refrigerator, 2 to 8 °C			
Freezer, -15 to -30 °C			
Freezer, -60 to -85 °C			
PCR workstation			
PCR workstation			
PCR workstation			
Centrifuge			
Pipette			
Pipette			
Pipette			
Pipette			
Pipette			
Pipette			
Pipette			
Pipette			
Pipette			

Table 3: Samples to be analyzed

*Position refers to the dilution plate position. **P = Pass, R = Retest, DR = Dilute and retest (>ULOQ), <LLOQ (no retesting needed)

Position*	Sample ID	Plate ID(s)	dPCRDAP Flags AAV2-ITR	Final Result**	dPCRDAP Flags B2M	Final Result**
A1						
B1						
C1						
D1						
E1						
F1						
G1						
H1						
A2						
B2						
C2						
D2						
E2						
F2						
G2						
H2						
A3						
B3						
C3						
D3						
E3						
F3						
G3						
H3						
A4						
B4						
C4						
D4						
E4						
F4						
G4						
H4						
A5						
B5						
C5						
D5						
E5						

F5						
G5						
H5						
A6						
B6						
C6						
D6						
E6						
F6						
G6						
H6						
A7						
B7						
C7						
D7						
E7						
F7						
G7						
H7						
A8						
B8						
C8						
D8						
E8						
F8						
G8						
H8						
A9						
B9						
C9						
D9						
E9						
F9						
G9						
H9						
A10						
B10						
C10						
D10						
E10						
F10						

G10						
H10						
A11						
B11						
C11						
D11						
E11						
F11						
G11						
H11						
A12						
B12						
C12						
D12						
E12						
F12						
G12						
H12						

Quality Control will apply e-signature using eInfotree
Technical Review will apply e-signature using eInfotree

dPCR Plate Loading

Batch ID:

The performing operator will apply e-signature using eInfotree (for each section/day as needed).

Verifier/record (if applicable) will apply e-signature using eInfotree (for each section/day as needed).

Workspace Preparation

1 Prepare the PCR workstations by turning on the UV light. Wipe down the interior surfaces and required equipment with a Nuclease cleaner then 70% ethanol.

2 Remove diluted (25 ng/µL target concentration) samples from the freezer according to the dPCR Plate Map(s). Maintain cold (2-8 °C or ice/cold block).

3 Fill out Table 4 with the number of reactions needed and calculate the amount of each reagent needed : (volume per reaction * number of reaction * safety factor).

4 Remove reagents from freezer. Maintain cold (2-8 °C or ice/cold block).

Table 4. AAV2-ITR and B2M Master Mix Reagent Amounts

Reagent	Volume per Reaction (µL)	Number of Reactions	Safety Factor	Total Volume (µL)
Rnase-free water	6.8		1.1	
4X Probe PCR master mix	3.5		1.1	
[Target Gene Primer/probe mix]	0.7		1.1	

Calculations will be verified by a secondary person and they will apply e-signature using eInfoTree.

1. Prepare Master Mixes

1.1 Inside a PCR Workstation dedicated to master mix preparation, mix reaction solutions by briefly (~5sec) vortexing, then centrifuge briefly (~5sec) to collect all liquids at the bottom of the tubes.

1.2 Prepare master mixes in appropriately sized tubes according to Table 4 for each target (AAV2-ITR and B2M). Add the components in the order in which they are listed from top to bottom.

1.3 Mix master mix preparations by vortexing briefly (~5sec), then centrifuge briefly (~5sec) if possible to collect all liquids at the bottom of the tubes.

1.4 Dispense 11.0 µl of the appropriate master mix into the wells specified in the associated dPCR Plate Map(s).

1.5 Cover the plate(s) with a plate seal. Plates in a batch which are not being loaded immediately can be stored in a 2-8 °C refrigerator until ready for subsequent steps.

2. Load Samples

2.1 In a PCR Workstation dedicated to handling DNA samples, add 3.0 µl of DNA sample, controls, and Ultra-purified H2O (upH2O) (NTC) to the wells specified in the associated dPCR Plate Map.

2.2 Seal the plate with a plate seal.

2.3 Briefly vortex (~30 sec) the plate to mix, then centrifuge at 4 °C, 1000 x g for 1 minute.
Note: centrifugation may be repeated if reaction mixture is not fully collected at the bottom of wells, or if there are any visible bubbles in the wells.

3. Load Nanoplate

3.1 In a PCR Workstation dedicated to handling DNA samples, prepare an 8.5K 96-well Nanoplate in a Nanoplate tray and bring the centrifuged master mix plate containing DNA samples.

3.2 Transfer 12 µl of the reaction mix from the reaction assembly plate to the equivalent position of the 8.5K 96-well Nanoplate.

3.3 Apply the nanoplate seal by removing the white protective foil. Align the seal over the Nanoplate so there is minimal overlap on the sides of the plate. Use the QIAcuity plate roller to affix the blue seal to the Nanoplate. Remove the thin transparent cover film.

Repeat Steps 2 and 3 for all plates in the batch.

Quality Control will apply e-signature using eInfotree

Technical Review will apply e-signature using eInfotree

Comments:

QIAcuity Operation - Running dPCR Plates on the QIAcuity Instrument

Batch ID:

The performing operator will apply e-signature using eInfotree (for each section/day as needed).
Verifier/record (if applicable) will apply e-signature using eInfotree (for each section/day as needed).

Experiment Setup in QIAcuity Software Suite

This may be performed in advance. If using a template, always verify the existing parameters.

1 In the QIAcuity Software Suite, create an experiment for each plate in the batch according to SOP-00465 using the following parameters:

Plate Name: "BatchID.Initials.UnitID.Runs"

Plate Type: 8.5K 96-well Nanoplate

Thermocycling Conditions:

Step	Temperature (°C)	Duration (min:sec)	Number of Cycles
1	95	02:00	1X
2	95	00:15	40X
	60	00:30	

Imaging Channels to select: Yellow and Green

Reaction Mixes:

Mix Name	Target Name	Dye	Channel
AAV2-ITR	AAV2-ITR	FAM	Green
B2M	B2M	VIC	Yellow

2 Define Sample Names and assign Reaction Mix(es) and Sample Names to the Plate Layout according to the dPCR Plate Map. Alternatively, an input file (.csv) may be imported from the network to fill in the experiment Plate Layout.

3 Record experiment names generated for this batch (step 1); "NA" if not used or add lines as needed:

Plate Name

Plate Name

Plate Name

Plate Name

Running dPCR Plates

1. Loading the Nanoplate to QIAcuity

1.1 For each prepared Nanoplate, log into the QIAcuity Software Suite and open the corresponding experiment file. In the General Data tab, scan or type in the barcode from the Nanoplate. Save and close.

1.2 At the QIAcuity instrument, log into the touch screen and open the tray with an available plate slot.

1.3 Remove the Nanoplate from the tray and insert into the desired QIAcuity plate slot with barcode facing in. Close the tray.

1.4 Verify the correct experiment populates on the screen, and start the plate run.

Quality Control will apply e-signature using eInfotree

Technical Review will apply e-signature using eInfotree

Comments:

Post-Run Data Analysis - QIAcuity Software Suite

Batch ID:

The performing operator will apply e-signature using eInfotree (for each section/day as needed).
Verifier/record (if applicable) will apply e-signature using eInfotree (for each section/day as needed).

Experiment Analysis in QIAcuity Software Suite

1 Verify the completed Nanoplate was removed from the instrument after the completion of the run.
2 In the QIAcuity Software Suite, open the completed experiment and navigate to the Analyze subtab.
3 Under Absolute Quantification, select all wells to analyze and the target(s) to analyze. Click Show results.
4 In the 1D Scatterplot view, set the threshold to a common value of 30 for the AAV2-ITR target or a common value of 60 for the B2M target and click Recalculate.
If needed, adjust the scale of the plot Y-axis to ensure adequate visualization of the positive and negative populations. If needed, the threshold may be adjusted to ensure accurate separation of the positive and negative populations.
4 Carefully review the signal map, reference channel, 1D Scatterplot, and source images (if applicable). Notify the SME or Study Director if aberrant observations are noted.
5 Navigate to List subtab, select Export to CSV > Current Results. Save the file to the network at: \\ab-file1\AnalySci\Qiacuity\A-579-01
6 Add the List and 1D Scatterplot tabs (at a minimum) to a report. Click Create Report and include Run details, Plate general data, Plate Layout and Reaction Mixes List. Click Generate Report.
7 Under the Reports subtab, select the new report and click Add Signature. When prompted, download the report to: \\ab-file1\AnalySci\Qiacuity\A-579-01
Enter password and reason for signature (e.g. Analyzed plate)
8 Navigate to Plates environment. On the plate that was just analyzed, click the ellipses and select Export. When prompted, save to:
\\ab-file1\AnalySci\Qiacuity\A-579-01

Repeat all steps for each plate in this batch.

Quality Control will apply e-signature using eInfotree
Technical Review will apply e-signature using eInfotree

Comments:

dPCR Data Analysis using the Data Analysis Pipeline (DAP)

Batch ID: ______

The performing operator will apply e-signature using eInfotree (for each section/day as needed).
Verifier/record (if applicable) will apply e-signature using eInfotree (for each section/day as needed).

dPCRDAP Analysis for Reportable Values

For each .csv file exported to the QIAcuity network folder, open and "Save As" to the following folder:
\\ab-file1\CMPT_CIFS\dpcr\studies\A-579-01\input\

1 During the save, change the filetype to **CSV UTF-8** and rename as:
"StudyID.BatchNum.Initials.UnitID.RunNum.AnalysisRun.Gene.dpcrdata"

AnalysisRun = 01 for the first plate, then increment number as needed.

**Plate Name**		**Renamed CSV UTF-8 File:**	
**Plate Name**		**Renamed CSV UTF-8 File:**	
**Plate Name**		**Renamed CSV UTF-8 File:**	
**Plate Name**		**Renamed CSV UTF-8 File:**	

2 Verify a Study Design File was made for the plate(s) being analyzed (SOP-00666).

3 Log into PuTTY and enter **ab-cmpt01** as the host name. In Saved Sessions select this option, or if no option available then type in the host name. Click Load, then Open. Log in with AmplifyBio credentials as prompted.
Enter the following command in the Command Prompt Window:

4 dpcr_analysis.sh StudyID.BatchNum.Initials.UnitID.RunNum.AnalysisRun AAV2-ITR B2M s 0.075 3
(fill in the StudyID.BatchNum.Initials.UnitID.RunNum.AnalysisRun based on the plate being analyzed)

5 Verify and review the Output file located in: \\ab-file1\CMPT_CIFS\dpcr\studies\A-579-01\output\

6 Note any flags in the Recording tab list of samples.

7 Perform censoring as needed per SOP-00666. Add detail regarding censoring below in Comments.

Confirmation of Sample/Plate Acceptance Criteria

The expected criteria for the assay are below for reference (based on SOP-00465):

	AAV2-ITR	B2M	%CV	% Positive Partitions	%Valid Partitions
**gBlock Positive Control**	Positive Result >4 partitions	NA	≤20%	<95%	>80%
**B2M gDNA Positive Control**	NA	Positive Result >4 partitions	≤20%	<95%	>80%
**NTC**	Negative result ≤ 4 partitions	Negative result ≤ 4 partitions	NA	NA	>80%
**Unknown Study Sample**	Positive result >LOD may observed in dose groups, negative results <LOD expected in control groups	Positive result >4 partitions	≤20%	<95%	>80%

Assay Limits for the AAV2-ITR gene target (A-579-02):

Limit of Detection (LOD)	6.93 copies/µL
Lower Limit of Quantitation (LLOQ)	14.9 copies/µL
Upper Limit of Quantitation (ULOQ)	16800 copies/µL

One of three replicates may be censored per SOP-00465 if applicable.
All results will be reviewed by the SME or Analytics Director and flags will be evaluated for final disposition and recorded in the Recording tab.
If CV% fails, censoring or retesting is not required if the result (average concentration) is <LLOQ.
An individual sample will be repeated no more than 2 times unless otherwise determined by SME or Analytics Director and dependent upon available sample.

Quality Control will apply e-signature using eInfotree
Technical Review will apply e-signature using eInfotree

Comments:

Attachment 2. Final Data Table

Source.Name	Sample Name	DNA Input (ug)	AAV2-ITR.Target Copy Num Per ug DNA	B2M.Target Copy Num Per ug DNA	AAV2-ITR/B2M ratio	AAV2-ITR.Comment	AAV2-ITR.CV Flag
A-579-01.001.MC.P01.01.01.dpcranalysis.xlsx	A-579-01.101.BRAN.01Aug2023.02	0.0591	0	372090	0.00000	BQL	
A-579-01.001.MC.P01.01.01.dpcranalysis.xlsx	A-579-01.101.DRGC.01Aug2023.02	0.075	0	282887	0.00000	BQL	
A-579-01.001.MC.P01.01.01.dpcranalysis.xlsx	A-579-01.101.DRGL.01Aug2023.01	0.075	0	366333	0.00000	BQL	
A-579-01.001.MC.P01.01.01.dpcranalysis.xlsx	A-579-01.101.DRGT.01Aug2023.02	0.0738	0	413506	0.00000	BQL	
A-579-01.002.MC.P01.01.01.dpcranalysis.xlsx	A-579-01.101.HTHT.01Aug2023.02	0.048	0	393594	0.00000	BQL	
A-579-01.002.MC.P01.01.01.dpcranalysis.xlsx	A-579-01.101.KDNY.01Aug2023.03	0.075	0	378100	0.00000	BQL	
A-579-01.002.MC.P01.01.01.dpcranalysis.xlsx	A-579-01.101.LUNG.01Aug2023.02	0.075	0	354380	0.00000	BQL	
A-579-01.002.MC.P01.01.01.dpcranalysis.xlsx	A-579-01.101.LVER.01Aug2023.02	0.075	0	444920	0.00000	BQL	
A-579-01.001.MC.P01.01.01.dpcranalysis.xlsx	A-579-01.101.MUAL.01Aug2023.03	0.0312	0	390265	0.00000	BQL	
A-579-01.001.MC.P01.01.01.dpcranalysis.xlsx	A-579-01.101.MUAR.01Aug2023.01	0.0402	134	467572	0.00029	BQL	
A-579-01.001.MC.P01.01.01.dpcranalysis.xlsx	A-579-01.101.MUBL.01Aug2023.03	0.0519	0	459500	0.00000	BQL	
A-579-01.001.MC.P01.01.01.dpcranalysis.xlsx	A-579-01.101.MUBR.01Aug2023.02	0.0372	0	372857	0.00000	BQL	
A-579-01.002.MC.P01.01.01.dpcranalysis.xlsx	A-579-01.101.NFDLD.01Aug2023.06	0.0552	0	288006	0.00000	BQL	
A-579-01.002.MC.P01.01.01.dpcranalysis.xlsx	A-579-01.101.NFDLP.01Aug2023.03	0.0453	0	346590	0.00000	BQL	
A-579-01.002.MC.P01.01.01.dpcranalysis.xlsx	A-579-01.101.NFDRD.01Aug2023.10	0.075	0	248086	0.00000	BQL	
A-579-01.002.MC.P01.01.01.dpcranalysis.xlsx	A-579-01.101.NFDRP.01Aug2023.09	0.0531	0	286591	0.00000	BQL	
A-579-01.002.MC.P01.01.01.dpcranalysis.xlsx	A-579-01.101.NFPLD.01Aug2023.05	0.0693	0	340229	0.00000	BQL	
A-579-01.002.MC.P01.01.01.dpcranalysis.xlsx	A-579-01.101.NFPLP.01Aug2023.01	0.075	0	277079	0.00000	BQL	
A-579-01.002.MC.P01.01.01.dpcranalysis.xlsx	A-579-01.101.NFPRD.01Aug2023.10	0.027	197	415722	0.00047	BQL	
A-579-01.002.MC.P01.01.01.dpcranalysis.xlsx	A-579-01.101.NFPRP.01Aug2023.09	0.075	0	345134	0.00000	BQL	
A-579-01.001.MC.P01.01.01.dpcranalysis.xlsx	A-579-01.101.NMDLD.01Aug2023.06	0.0672	0	331260	0.00000	BQL	
A-579-01.001.MC.P01.01.01.dpcranalysis.xlsx	A-579-01.101.NMDLP.01Aug2023.01	0.0147	0	348794	0.00000	BQL	
A-579-01.001.MC.P01.01.01.dpcranalysis.xlsx	A-579-01.101.NMDRD.01Aug2023.11	0.075	0	341544	0.00000	BQL	
A-579-01.001.MC.P01.01.01.dpcranalysis.xlsx	A-579-01.101.NMDRP.01Aug2023.09	0.0567	0	408988	0.00000	BQL	
A-579-01.001.MC.P01.01.01.dpcranalysis.xlsx	A-579-01.101.NMPLD.01Aug2023.05	0.075	0	321583	0.00000	BQL	
A-579-01.001.MC.P01.01.01.dpcranalysis.xlsx	A-579-01.101.NMPLP.01Aug2023.01	0.0369	0	393391	0.00000	BQL	
A-579-01.001.MC.P01.01.01.dpcranalysis.xlsx	A-579-01.101.NMPRD.01Aug2023.12	0.0474	0	386073	0.00000	BQL	
A-579-01.001.MC.P01.01.01.dpcranalysis.xlsx	A-579-01.101.NMPRP.01Aug2023.08	0.0672	0	347750	0.00000	BQL	
A-579-01.001.MC.P01.01.01.dpcranalysis.xlsx	A-579-01.101.NSDLD.01Aug2023.06	0.0651	0	344237	0.00000	BQL	
A-579-01.001.MC.P01.01.01.dpcranalysis.xlsx	A-579-01.101.NSDLP.01Aug2023.01	0.0582	0	429093	0.00000	BQL	
A-579-01.001.MC.P01.01.01.dpcranalysis.xlsx	A-579-01.101.NSDRD.01Aug2023.10	0.075	0	239431	0.00000	BQL	
A-579-01.001.MC.P01.01.01.dpcranalysis.xlsx	A-579-01.101.NSDRP.01Aug2023.08	0.0657	0	417734	0.00000	BQL	
A-579-01.001.MC.P01.01.01.dpcranalysis.xlsx	A-579-01.101.NSPLD.01Aug2023.04	0.075	0	301348	0.00000	BQL	
A-579-01.001.MC.P01.01.01.dpcranalysis.xlsx	A-579-01.101.NSPLP.01Aug2023.02	0.075	0	353858	0.00000	BQL	
A-579-01.001.MC.P01.01.01.dpcranalysis.xlsx	A-579-01.101.NSPRD.01Aug2023.10	0.0666	0	401277	0.00000	BQL	
A-579-01.001.MC.P01.01.01.dpcranalysis.xlsx	A-579-01.101.NSPRP.01Aug2023.07	0.075	0	387943	0.00000	BQL	
A-579-01.001.MC.P01.01.01.dpcranalysis.xlsx	A-579-01.101.NUDLD.01Aug2023.06	0.075	0	364218	0.00000	BQL	
A-579-01.001.MC.P01.01.01.dpcranalysis.xlsx	A-579-01.101.NUDLP.01Aug2023.03	0.075	0	394044	0.00000	BQL	
A-579-01.001.MC.P01.01.01.dpcranalysis.xlsx	A-579-01.101.NUDRD.01Aug2023.12	0.075	0	331072	0.00000	BQL	

IND: indeterminate, CV% >20 (only applicable to results above the lower limit of quantitation[LLOQ])
BQL: below quantitation limit (below LLOQ)
>ULOQ: above upper limit of quantitation
LLOQ = 14.9 copies/µL
ULOQ = 16800 copies/µL

Source.Name	Sample Name	DNA Input (ug)	AAV2-ITR.Target Copy Num Per ug DNA	B2M.Target Copy Num Per ug DNA	AAV2-ITR/B2M ratio	AAV2-ITR.Comment	AAV2-ITR.CV Flag
A-579-01.001.MC.P01.01.01.dpcranalysis.xlsx	A-579-01.101.NUDRP.01Aug2023.08	0.075	0	313108	0.00000	BQL	
A-579-01.002.MC.P01.01.01.dpcranalysis.xlsx	A-579-01.101.NUPLD.01Aug2023.05	0.0387	0	397282	0.00000	BQL	
A-579-01.001.MC.P01.01.01.dpcranalysis.xlsx	A-579-01.101.NUPLP.01Aug2023.03	0.075	0	296949	0.00000	BQL	
A-579-01.002.MC.P01.01.01.dpcranalysis.xlsx	A-579-01.101.NUPRD.01Aug2023.10	0.075	0	453681	0.00000	BQL	
A-579-01.002.MC.P01.01.01.dpcranalysis.xlsx	A-579-01.101.NUPRP.01Aug2023.08	0.0138	0	371676	0.00000	BQL	
A-579-01.001.MC.P01.01.01.dpcranalysis.xlsx	A-579-01.101.NVOT.01Aug2023.07	0.0363	0	347744	0.00000	BQL	
A-579-01.002.MC.P01.01.01.dpcranalysis.xlsx	A-579-01.101.SCCV.01Aug2023.03	0.0306	0	390627	0.00000	BQL	
A-579-01.002.MC.P01.01.01.dpcranalysis.xlsx	A-579-01.101.SCLU.01Aug2023.02	0.075	0	347620	0.00000	BQL	
A-579-01.002.MC.P01.01.01.dpcranalysis.xlsx	A-579-01.101.SCSA.01Aug2023.02	0.075	0	273249	0.00000	BQL	
A-579-01.002.MC.P01.01.01.dpcranalysis.xlsx	A-579-01.101.SCSR.01Aug2023.03	0.075	0	350622	0.00000	BQL	
A-579-01.002.MC.P01.01.01.dpcranalysis.xlsx	A-579-01.101.SCTH.01Aug2023.01	0.0108	0	443809	0.00000	BQL	
A-579-01.002.MC.P01.01.03.dpcranalysis.xlsx	A-579-01.101.SPLN.01Aug2023.03	0.075	448	448233	0.00100	BQL	
A-579-01.002.MC.P01.01.01.dpcranalysis.xlsx	A-579-01.101.TEST.01Aug2023.02	0.075	0	511784	0.00000	BQL	
A-579-01.021.MC.P01.01.02.dpcranalysis.xlsx	A-579-01.102.BRAN.12Sep2023.02	0.0423	0	365125	0.00000	BQL	
A-579-01.021.MC.P01.01.02.dpcranalysis.xlsx	A-579-01.102.DRGC.12Sep2023.02	0.075	0	400792	0.00000	BQL	
A-579-01.021.MC.P01.01.01.dpcranalysis.xlsx	A-579-01.102.DRGL.12Sep2023.01	0.075	0	392075	0.00000	BQL	
A-579-01.021.MC.P01.01.01.dpcranalysis.xlsx	A-579-01.102.DRGT.12Sep2023.02	0.075	0	334451	0.00000	BQL	
A-579-01.022.MC.P01.01.02.dpcranalysis.xlsx	A-579-01.102.HTHT.12Sep2023.02	0.0471	0	356851	0.00000	BQL	
A-579-01.022.MC.P01.01.01.dpcranalysis.xlsx	A-579-01.102.KDNY.12Sep2023.01	0.075	0	281101	0.00000	BQL	
A-579-01.022.MC.P01.01.02.dpcranalysis.xlsx	A-579-01.102.LUNG.12Sep2023.03	0.075	0	175	0.00000	BQL	
A-579-01.022.MC.P01.01.02.dpcranalysis.xlsx	A-579-01.102.LVER.12Sep2023.02*	0.075	0	226473	0.00000	BQL	
A-579-01.021.MC.P01.01.02.dpcranalysis.xlsx	A-579-01.102.MUAL.12Sep2023.03	0.075	0	355460	0.00000	BQL	
A-579-01.021.MC.P01.01.01.dpcranalysis.xlsx	A-579-01.102.MUAR.12Sep2023.01	0.075	0	417169	0.00000	BQL	
A-579-01.021.MC.P01.01.01.dpcranalysis.xlsx	A-579-01.102.MUBL.12Sep2023.01	0.075	0	312629	0.00000	BQL	
A-579-01.021.MC.P01.01.01.dpcranalysis.xlsx	A-579-01.102.MUBR.12Sep2023.01	0.0603	0	363714	0.00000	BQL	
A-579-01.022.MC.P01.01.02.dpcranalysis.xlsx	A-579-01.102.NFDLD.12Sep2023.04	0.075	0	382536	0.00000	BQL	
A-579-01.022.MC.P01.01.02.dpcranalysis.xlsx	A-579-01.102.NFDLP.12Sep2023.03	0.0579	92	448157	0.00021	BQL	
A-579-01.022.MC.P01.01.01.dpcranalysis.xlsx	A-579-01.102.NFDRD.12Sep2023.12	0.0495	0	354026	0.00000	BQL	
A-579-01.022.MC.P01.01.01.dpcranalysis.xlsx	A-579-01.102.NFDRP.12Sep2023.09	0.075	0	347212	0.00000	BQL	
A-579-01.022.MC.P01.01.01.dpcranalysis.xlsx	A-579-01.102.NFPLD.12Sep2023.04	0.075	0	308902	0.00000	BQL	
A-579-01.022.MC.P01.01.01.dpcranalysis.xlsx	A-579-01.102.NFPLP.12Sep2023.01	0.075	0	279645	0.00000	BQL	
A-579-01.022.MC.P01.01.01.dpcranalysis.xlsx	A-579-01.102.NFPRD.12Sep2023.12	0.027	146	370170	0.00039	IND	x
A-579-01.022.MC.P01.01.01.dpcranalysis.xlsx	A-579-01.102.NFPRP.12Sep2023.08	0.075	0	317974	0.00000	BQL	
A-579-01.021.MC.P01.01.01.dpcranalysis.xlsx	A-579-01.102.NMDLD.12Sep2023.05	0.0549	0	343523	0.00000	BQL	
A-579-01.021.MC.P01.01.01.dpcranalysis.xlsx	A-579-01.102.NMDLP.12Sep2023.02	0.0465	0	347361	0.00000	BQL	
A-579-01.021.MC.P01.01.01.dpcranalysis.xlsx	A-579-01.102.NMDRD.12Sep2023.11	0.075	0	382934	0.00000	BQL	
A-579-01.021.MC.P01.01.01.dpcranalysis.xlsx	A-579-01.102.NMDRP.12Sep2023.08	0.075	0	291405	0.00000	BQL	
A-579-01.021.MC.P01.01.01.dpcranalysis.xlsx	A-579-01.102.NMPLD.12Sep2023.05	0.075	0	403175	0.00000	BQL	
A-579-01.021.MC.P01.01.02.dpcranalysis.xlsx	A-579-01.102.NMPLP.12Sep2023.02	0.075	74	351951	0.00021	BQL	

IND: indeterminate, CV% >20 (only applicable to results above the lower limit of quantitation[LLOQ])
BQL: below quantitation limit (below LLOQ)
>ULOQ: above upper limit of quantitation
LLOQ = 14.9 copies/µL
ULOQ = 16800 copies/µL
*See DEV-002848

Source.Name	Sample Name	DNA Input (ug)	AAV2-ITR.Target Copy Num Per ug DNA	B2M.Target Copy Num Per ug DNA	AAV2-ITR/B2M ratio	AAV2-ITR.Comment	AAV2-ITR.CV Flag
A-579-01.021.MC.P01.01.01.dpcranalysis.xlsx	A-579-01.102.NMPRD.12Sep2023.11	0.0543	0	369337	0.00000	BQL	
A-579-01.021.MC.P01.01.01.dpcranalysis.xlsx	A-579-01.102.NMPRP.12Sep2023.08	0.075	0	361100	0.00000	BQL	
A-579-01.021.MC.P01.01.01.dpcranalysis.xlsx	A-579-01.102.NSDLD.12Sep2023.06	0.075	0	401470	0.00000	BQL	
A-579-01.021.MC.P01.01.01.dpcranalysis.xlsx	A-579-01.102.NSDLP.12Sep2023.01	0.0375	0	410094	0.00000	BQL	
A-579-01.021.MC.P01.01.01.dpcranalysis.xlsx	A-579-01.102.NSDRD.12Sep2023.10	0.075	0	361586	0.00000	BQL	
A-579-01.021.MC.P01.01.01.dpcranalysis.xlsx	A-579-01.102.NSDRP.12Sep2023.07	0.075	0	362799	0.00000	BQL	
A-579-01.021.MC.P01.01.01.dpcranalysis.xlsx	A-579-01.102.NSPLD.12Sep2023.06	0.075	0	340928	0.00000	BQL	
A-579-01.021.MC.P01.01.01.dpcranalysis.xlsx	A-579-01.102.NSPLP.12Sep2023.01	0.075	0	212153	0.00000	BQL	
A-579-01.021.MC.P01.01.02.dpcranalysis.xlsx	A-579-01.102.NSPRD.12Sep2023.12	0.0387	0	345351	0.00000	BQL	
A-579-01.021.MC.P01.01.02.dpcranalysis.xlsx	A-579-01.102.NSPRP.12Sep2023.07	0.075	212	331660	0.00064	BQL	
A-579-01.021.MC.P01.01.01.dpcranalysis.xlsx	A-579-01.102.NUDLD.12Sep2023.05	0.0726	0	290574	0.00000	BQL	
A-579-01.021.MC.P01.01.01.dpcranalysis.xlsx	A-579-01.102.NUDLP.12Sep2023.03	0.069	0	331083	0.00000	BQL	
A-579-01.021.MC.P01.01.01.dpcranalysis.xlsx	A-579-01.102.NUDRD.12Sep2023.10	0.0645	0	420485	0.00000	BQL	
A-579-01.021.MC.P01.01.01.dpcranalysis.xlsx	A-579-01.102.NUDRP.12Sep2023.09	0.075	0	359047	0.00000	BQL	
A-579-01.022.MC.P01.01.02.dpcranalysis.xlsx	A-579-01.102.NUPLD.12Sep2023.06	0.075	0	375928	0.00000	BQL	
A-579-01.021.MC.P01.01.02.dpcranalysis.xlsx	A-579-01.102.NUPLP.12Sep2023.02	0.075	0	219455	0.00000	BQL	
A-579-01.022.MC.P01.01.01.dpcranalysis.xlsx	A-579-01.102.NUPRD.12Sep2023.10	0.0669	0	403391	0.00000	BQL	
A-579-01.022.MC.P01.01.01.dpcranalysis.xlsx	A-579-01.102.NUPRP.12Sep2023.09	0.0534	0	431754	0.00000	BQL	
A-579-01.021.MC.P01.01.01.dpcranalysis.xlsx	A-579-01.102.NVOT.12Sep2023.08	0.075	0	374777	0.00000	BQL	
A-579-01.022.MC.P01.01.01.dpcranalysis.xlsx	A-579-01.102.SCCV.12Sep2023.03	0.0534	0	338552	0.00000	BQL	
A-579-01.022.MC.P01.01.01.dpcranalysis.xlsx	A-579-01.102.SCLU.12Sep2023.01	0.075	0	385286	0.00000	BQL	
A-579-01.022.MC.P01.01.01.dpcranalysis.xlsx	A-579-01.102.SCSA.12Sep2023.03	0.0492	0	338817	0.00000	BQL	
A-579-01.022.MC.P01.01.01.dpcranalysis.xlsx	A-579-01.102.SCSR.12Sep2023.01*	0.075	0	336703	0.00000	BQL	
A-579-01.022.MC.P01.01.01.dpcranalysis.xlsx	A-579-01.102.SCTH.12Sep2023.03	0.075	0	367771	0.00000	BQL	
A-579-01.022.MC.P01.01.01.dpcranalysis.xlsx	A-579-01.102.SPLN.12Sep2023.01	0.075	0	331501	0.00000	BQL	
A-579-01.022.MC.P01.01.01.dpcranalysis.xlsx	A-579-01.102.TEST.12Sep2023.03	0.075	0	357429	0.00000	BQL	
A-579-01.022.MC.P01.01.02.dpcranalysis.xlsx	A-579-01.102.TGL1.12Sep2023.03	0.075	0	313264	0.00000	BQL	
A-579-01.003.MC.P01.01.01.dpcranalysis.xlsx	A-579-01.151.BRAN.01Aug2023.03	0.0261	0	580902	0.00000	BQL	
A-579-01.003.MC.P01.01.01.dpcranalysis.xlsx	A-579-01.151.DRGC.01Aug2023.02	0.075	37	429959	0.00009	IND	x
A-579-01.003.MC.P01.01.01.dpcranalysis.xlsx	A-579-01.151.DRGL.01Aug2023.03	0.075	0	305508	0.00000	BQL	
A-579-01.003.MC.P01.01.01.dpcranalysis.xlsx	A-579-01.151.DRGT.01Aug2023.03	0.075	0	341357	0.00000	BQL	
A-579-01.004.MC.P01.01.01.dpcranalysis.xlsx	A-579-01.151.HTHT.01Aug2023.02	0.075	0	333387	0.00000	BQL	
A-579-01.004.MC.P01.01.01.dpcranalysis.xlsx	A-579-01.151.KDNY.01Aug2023.03	0.075	0	463089	0.00000	BQL	
A-579-01.004.MC.P01.01.01.dpcranalysis.xlsx	A-579-01.151.LUNG.01Aug2023.02	0.075	0	212962	0.00000	BQL	
A-579-01.004.MC.P01.01.01.dpcranalysis.xlsx	A-579-01.151.LVER.01Aug2023.01	0.075	0	286384	0.00000	BQL	
A-579-01.003.MC.P01.01.01.dpcranalysis.xlsx	A-579-01.151.MUAL.01Aug2023.03	0.0417	0	365824	0.00000	BQL	
A-579-01.003.MC.P01.01.01.dpcranalysis.xlsx	A-579-01.151.MUAR.01Aug2023.02	0.0555	0	503899	0.00000	BQL	
A-579-01.003.MC.P01.01.01.dpcranalysis.xlsx	A-579-01.151.MUBL.01Aug2023.03	0.0222	251	377905	0.00066	BQL	
A-579-01.003.MC.P01.01.01.dpcranalysis.xlsx	A-579-01.151.MUBR.01Aug2023.03	0.033	0	326384	0.00000	BQL	

IND: indeterminate, CV% >20 (only applicable to results above the lower limit of quantitation[LLOQ])
BQL: below quantitation limit (below LLOQ)
>ULOQ: above upper limit of quantitation
LLOQ = 14.9 copies/µL
ULOQ = 16800 copies/µL
*See DEV-002848

Source.Name	Sample Name	DNA Input (ug)	AAV2-ITR.Target Copy Num Per ug DNA	B2M.Target Copy Num Per ug DNA	AAV2-ITR/B2M ratio	AAV2-ITR.Comment	AAV2-ITR.CV Flag
A-579-01.004.MC.P01.01.01.dpcranalysis.xlsx	A-579-01.151.NFDLD.01Aug2023.06	0.0468	0	325321	0.00000	BQL	
A-579-01.004.MC.P01.01.01.dpcranalysis.xlsx	A-579-01.151.NFDLP.01Aug2023.03	0.03	0	442431	0.00000	BQL	
A-579-01.004.MC.P01.01.01.dpcranalysis.xlsx	A-579-01.151.NFDRD.01Aug2023.12	0.0414	0	495647	0.00000	BQL	
A-579-01.004.MC.P01.01.01.dpcranalysis.xlsx	A-579-01.151.NFDRP.01Aug2023.08	0.0438	0	425849	0.00000	BQL	
A-579-01.004.MC.P01.01.01.dpcranalysis.xlsx	A-579-01.151.NFPLD.01Aug2023.06	0.0429	0	341787	0.00000	BQL	
A-579-01.004.MC.P01.01.01.dpcranalysis.xlsx	A-579-01.151.NFPLP.01Aug2023.03	0.0336	0	445347	0.00000	BQL	
A-579-01.004.MC.P01.01.01.dpcranalysis.xlsx	A-579-01.151.NFPRD.01Aug2023.11	0.0387	0	335096	0.00000	BQL	
A-579-01.004.MC.P01.01.01.dpcranalysis.xlsx	A-579-01.151.NFPRP.01Aug2023.07	0.0516	0	307240	0.00000	BQL	
A-579-01.003.MC.P01.01.01.dpcranalysis.xlsx	A-579-01.151.NMDLD.01Aug2023.05	0.057	0	400359	0.00000	BQL	
A-579-01.003.MC.P01.01.01.dpcranalysis.xlsx	A-579-01.151.NMDLP.01Aug2023.03	0.0564	0	423285	0.00000	BQL	
A-579-01.003.MC.P01.01.01.dpcranalysis.xlsx	A-579-01.151.NMDRD.01Aug2023.11	0.075	0	459791	0.00000	BQL	
A-579-01.003.MC.P01.01.01.dpcranalysis.xlsx	A-579-01.151.NMDRP.01Aug2023.07	0.0261	0	463734	0.00000	BQL	
A-579-01.003.MC.P01.01.01.dpcranalysis.xlsx	A-579-01.151.NMPLD.01Aug2023.04	0.048	0	428449	0.00000	BQL	
A-579-01.003.MC.P01.01.01.dpcranalysis.xlsx	A-579-01.151.NMPLP.01Aug2023.03	0.0216	0	510373	0.00000	BQL	
A-579-01.003.MC.P01.01.01.dpcranalysis.xlsx	A-579-01.151.NMPRD.01Aug2023.12	0.0426	0	369970	0.00000	BQL	
A-579-01.003.MC.P01.01.01.dpcranalysis.xlsx	A-579-01.151.NMPRP.01Aug2023.08	0.075	0	344556	0.00000	BQL	
A-579-01.003.MC.P01.01.01.dpcranalysis.xlsx	A-579-01.151.NSDLD.01Aug2023.04	0.0276	0	410447	0.00000	BQL	
A-579-01.003.MC.P01.01.01.dpcranalysis.xlsx	A-579-01.151.NSDLP.01Aug2023.03	0.0318	0	450759	0.00000	BQL	
A-579-01.003.MC.P01.01.01.dpcranalysis.xlsx	A-579-01.151.NSDRD.01Aug2023.12	0.075	0	436305	0.00000	BQL	
A-579-01.003.MC.P01.01.01.dpcranalysis.xlsx	A-579-01.151.NSDRP.01Aug2023.07	0.0309	0	446414	0.00000	BQL	
A-579-01.003.MC.P01.01.01.dpcranalysis.xlsx	A-579-01.151.NSPLD.01Aug2023.05	0.0717	0	379751	0.00000	BQL	
A-579-01.003.MC.P01.01.01.dpcranalysis.xlsx	A-579-01.151.NSPLP.01Aug2023.01	0.0315	0	358963	0.00000	BQL	
A-579-01.003.MC.P01.01.01.dpcranalysis.xlsx	A-579-01.151.NSPRD.01Aug2023.11	0.0315	0	342104	0.00000	BQL	
A-579-01.003.MC.P01.01.01.dpcranalysis.xlsx	A-579-01.151.NSPRP.01Aug2023.07	0.075	0	290596	0.00000	BQL	
A-579-01.003.MC.P01.01.01.dpcranalysis.xlsx	A-579-01.151.NUDLD.01Aug2023.06	0.0594	0	354879	0.00000	BQL	
A-579-01.003.MC.P01.01.01.dpcranalysis.xlsx	A-579-01.151.NUDLP.01Aug2023.03	0.0498	0	355932	0.00000	BQL	
A-579-01.003.MC.P01.01.01.dpcranalysis.xlsx	A-579-01.151.NUDRD.01Aug2023.11	0.0465	0	349443	0.00000	BQL	
A-579-01.003.MC.P01.01.01.dpcranalysis.xlsx	A-579-01.151.NUDRP.01Aug2023.08	0.0225	0	479329	0.00000	BQL	
A-579-01.004.MC.P01.01.02.dpcranalysis.xlsx	A-579-01.151.NUPLD.01Aug2023.05	0.0741	0	315123	0.00000	BQL	
A-579-01.003.MC.P01.01.01.dpcranalysis.xlsx	A-579-01.151.NUPLP.01Aug2023.01	0.0315	0	398785	0.00000	BQL	
A-579-01.004.MC.P01.01.01.dpcranalysis.xlsx	A-579-01.151.NUPRD.01Aug2023.10	0.0249	0	359558	0.00000	BQL	
A-579-01.004.MC.P01.01.01.dpcranalysis.xlsx	A-579-01.151.NUPRP.01Aug2023.09	0.009	0	811922	0.00000	BQL	
A-579-01.003.MC.P01.01.01.dpcranalysis.xlsx	A-579-01.151.NVOT.01Aug2023.09	0.0573	0	412914	0.00000	BQL	
A-579-01.004.MC.P01.01.01.dpcranalysis.xlsx	A-579-01.151.OVAS.01Aug2023.03	0.075	0	454403	0.00000	BQL	
A-579-01.004.MC.P01.01.01.dpcranalysis.xlsx	A-579-01.151.SCCV.01Aug2023.03	0.0285	184	369600	0.00050	IND	x
A-579-01.004.MC.P01.01.01.dpcranalysis.xlsx	A-579-01.151.SCLU.01Aug2023.01	0.0027	0	329544	0.00000	BQL	
A-579-01.004.MC.P01.01.01.dpcranalysis.xlsx	A-579-01.151.SCSA.01Aug2023.02	0.075	0	370823	0.00000	BQL	
A-579-01.004.MC.P01.01.01.dpcranalysis.xlsx	A-579-01.151.SCSR.01Aug2023.01	0.0342	0	376690	0.00000	BQL	
A-579-01.004.MC.P01.01.01.dpcranalysis.xlsx	A-579-01.151.SCTH.01Aug2023.02	0.0384	0	374883	0.00000	BQL	

IND: indeterminate, CV% >20 (only applicable to results above the lower limit of quantitation[LLOQ])
BQL: below quantitation limit (below LLOQ)
>ULOQ: above upper limit of quantitation
LLOQ = 14.9 copies/µL
ULOQ = 16800 copies/µL

Source.Name	Sample Name	DNA Input (ug)	AAV2-ITR.Target Copy Num Per ug DNA	B2M.Target Copy Num Per ug DNA	AAV2-ITR/B2M ratio	AAV2-ITR.Comment	AAV2-ITR.CV Flag
A-579-01.004.MC.P01.01.01.dpcranalysis.xlsx	A-579-01.151.SPLN.01Aug2023.01	0.075	0	347181	0.00000	BQL	
A-579-01.023.MC.P01.01.02.dpcranalysis.xlsx	A-579-01.152.BRAN.12Sep2023.02	0.0459	114	339111	0.00034	BQL	
A-579-01.023.MC.P01.01.02.dpcranalysis.xlsx	A-579-01.152.DRGC.12Sep2023.02	0.0504	0	359722	0.00000	BQL	
A-579-01.023.MC.P01.01.02.dpcranalysis.xlsx	A-579-01.152.DRGL.12Sep2023.01	0.075	208	448821	0.00046	IND	x
A-579-01.023.MC.P01.01.02.dpcranalysis.xlsx	A-579-01.152.DRGT.12Sep2023.03	0.075	0	370477	0.00000	BQL	
A-579-01.024.MC.P01.01.02.dpcranalysis.xlsx	A-579-01.152.HTHT.12Sep2023.02	0.0633	0	341828	0.00000	BQL	
A-579-01.024.MC.P01.01.02.dpcranalysis.xlsx	A-579-01.152.KDNY.12Sep2023.01	0.075	0	351073	0.00000	BQL	
A-579-01.024.MC.P01.01.02.dpcranalysis.xlsx	A-579-01.152.LUNG.12Sep2023.03	0.075	0	413803	0.00000	BQL	
A-579-01.024.MC.P01.01.02.dpcranalysis.xlsx	A-579-01.152.LVER.12Sep2023.01	0.075	468	434859	0.00108	BQL	
A-579-01.023.MC.P01.01.02.dpcranalysis.xlsx	A-579-01.152.MUAL.12Sep2023.01	0.0576	94	368922	0.00026	BQL	
A-579-01.023.MC.P01.01.01.dpcranalysis.xlsx	A-579-01.152.MUAR.12Sep2023.01	0.0054	0	42130	0.00000	BQL	
A-579-01.023.MC.P01.01.02.dpcranalysis.xlsx	A-579-01.152.MUBL.12Sep2023.01	0.0072	0	83475	0.00000	BQL	
A-579-01.023.MC.P01.01.01.dpcranalysis.xlsx	A-579-01.152.MUBR.12Sep2023.02	0.0264	0	380864	0.00000	BQL	
A-579-01.024.MC.P01.01.01.dpcranalysis.xlsx	A-579-01.152.NFDLD.12Sep2023.04	0.0699	0	378367	0.00000	BQL	
A-579-01.024.MC.P01.01.02.dpcranalysis.xlsx	A-579-01.152.NFDLP.12Sep2023.02	0.0579	0	387575	0.00000	BQL	
A-579-01.024.MC.P01.01.01.dpcranalysis.xlsx	A-579-01.152.NFDRD.12Sep2023.12	0.0636	0	373818	0.00000	BQL	
A-579-01.024.MC.P01.01.02.dpcranalysis.xlsx	A-579-01.152.NFDRP.12Sep2023.08	0.0663	0	328293	0.00000	BQL	
A-579-01.024.MC.P01.01.02.dpcranalysis.xlsx	A-579-01.152.NFPLD.12Sep2023.04	0.0399	0	369035	0.00000	BQL	
A-579-01.024.MC.P01.01.02.dpcranalysis.xlsx	A-579-01.152.NFPLP.12Sep2023.02	0.0558	0	339425	0.00000	BQL	
A-579-01.024.MC.P01.01.02.dpcranalysis.xlsx	A-579-01.152.NFPRD.12Sep2023.10	0.0144	0	346257	0.00000	BQL	
A-579-01.024.MC.P01.01.02.dpcranalysis.xlsx	A-579-01.152.NFPRP.12Sep2023.09	0.039	0	327421	0.00000	BQL	
A-579-01.023.MC.P01.01.02.dpcranalysis.xlsx	A-579-01.152.NMDLD.12Sep2023.05	0.0297	0	376704	0.00000	BQL	
A-579-01.023.MC.P01.01.01.dpcranalysis.xlsx	A-579-01.152.NMDLP.12Sep2023.03	0.0336	0	361333	0.00000	BQL	
A-579-01.023.MC.P01.01.02.dpcranalysis.xlsx	A-579-01.152.NMDRD.12Sep2023.10	0.0147	0	392810	0.00000	BQL	
A-579-01.023.MC.P01.01.01.dpcranalysis.xlsx	A-579-01.152.NMDRP.12Sep2023.09	0.018	0	421711	0.00000	BQL	
A-579-01.023.MC.P01.01.02.dpcranalysis.xlsx	A-579-01.152.NMPLD.12Sep2023.05	0.0387	0	350651	0.00000	BQL	
A-579-01.023.MC.P01.01.01.dpcranalysis.xlsx	A-579-01.152.NMPLP.12Sep2023.01	0.0282	0	357894	0.00000	BQL	
A-579-01.023.MC.P01.01.01.dpcranalysis.xlsx	A-579-01.152.NMPRD.12Sep2023.11	0.0108	0	431623	0.00000	BQL	
A-579-01.023.MC.P01.01.01.dpcranalysis.xlsx	A-579-01.152.NMPRP.12Sep2023.07	0.0363	0	387102	0.00000	BQL	
A-579-01.023.MC.P01.01.02.dpcranalysis.xlsx	A-579-01.152.NSDLD.12Sep2023.06	0.0234	236	473577	0.00050	BQL	
A-579-01.023.MC.P01.01.02.dpcranalysis.xlsx	A-579-01.152.NSDLP.12Sep2023.02	0.0438	0	440441	0.00000	BQL	
A-579-01.023.MC.P01.01.02.dpcranalysis.xlsx	A-579-01.152.NSDRD.12Sep2023.10	0.0474	0	378310	0.00000	BQL	
A-579-01.023.MC.P01.01.02.dpcranalysis.xlsx	A-579-01.152.NSDRP.12Sep2023.09	0.0231	0	440848	0.00000	BQL	
A-579-01.023.MC.P01.01.02.dpcranalysis.xlsx	A-579-01.152.NSPLD.12Sep2023.04	0.0405	0	384119	0.00000	BQL	
A-579-01.023.MC.P01.01.02.dpcranalysis.xlsx	A-579-01.152.NSPLP.12Sep2023.02	0.057	0	392504	0.00000	BQL	
A-579-01.023.MC.P01.01.02.dpcranalysis.xlsx	A-579-01.152.NSPRD.12Sep2023.12	0.0234	223	294987	0.00076	BQL	
A-579-01.023.MC.P01.01.02.dpcranalysis.xlsx	A-579-01.152.NSPRP.12Sep2023.07	0.0585	0	424092	0.00000	BQL	
A-579-01.023.MC.P01.01.01.dpcranalysis.xlsx	A-579-01.152.NUDLD.12Sep2023.05	0.0549	0	358500	0.00000	BQL	
A-579-01.023.MC.P01.01.01.dpcranalysis.xlsx	A-579-01.152.NUDLP.12Sep2023.02	0.0351	0	340334	0.00000	BQL	

IND: indeterminate, CV% >20 (only applicable to results above the lower limit of quantitation[LLOQ])
BQL: below quantitation limit (below LLOQ)
>ULOQ: above upper limit of quantitation
LLOQ = 14.9 copies/μL
ULOQ = 16800 copies/μL

Source.Name	Sample Name	DNA Input (ug)	AAV2-ITR.Target Copy Num Per ug DNA	B2M.Target Copy Num Per ug DNA	AAV2-ITR/B2M ratio	AAV2-ITR.Comment	AAV2-ITR.CV Flag
A-579-01.023.MC.P01.01.01.dpcranalysis.xlsx	A-579-01.152.NUDRD.12Sep2023.10	0.0408	0	343194	0.00000	BQL	
A-579-01.023.MC.P01.01.01.dpcranalysis.xlsx	A-579-01.152.NUDRP.12Sep2023.09	0.0315	0	322237	0.00000	BQL	
A-579-01.024.MC.P01.01.02.dpcranalysis.xlsx	A-579-01.152.NUPLD.12Sep2023.05	0.0408	0	389238	0.00000	BQL	
A-579-01.023.MC.P01.01.02.dpcranalysis.xlsx	A-579-01.152.NUPLP.12Sep2023.03	0.0141	0	421688	0.00000	BQL	
A-579-01.024.MC.P01.01.02.dpcranalysis.xlsx	A-579-01.152.NUPRD.12Sep2023.10	0.0171	0	428474	0.00000	BQL	
A-579-01.024.MC.P01.01.02.dpcranalysis.xlsx	A-579-01.152.NUPRP.12Sep2023.09	0.0123	0	283813	0.00000	BQL	
A-579-01.023.MC.P01.01.01.dpcranalysis.xlsx	A-579-01.152.NVOT.12Sep2023.07	0.057	0	315770	0.00000	BQL	
A-579-01.024.MC.P01.01.02.dpcranalysis.xlsx	A-579-01.152.OVAS.12Sep2023.02	0.075	0	308747	0.00000	BQL	
A-579-01.024.MC.P01.01.01.dpcranalysis.xlsx	A-579-01.152.SCCV.12Sep2023.03	0.0297	0	281666	0.00000	BQL	
A-579-01.024.MC.P01.01.01.dpcranalysis.xlsx	A-579-01.152.SCLU.12Sep2023.02	0.075	0	347567	0.00000	BQL	
A-579-01.024.MC.P01.01.01.dpcranalysis.xlsx	A-579-01.152.SCSA.12Sep2023.03	0.075	0	357772	0.00000	BQL	
A-579-01.024.MC.P01.01.01.dpcranalysis.xlsx	A-579-01.152.SCSR.12Sep2023.03	0.075	0	390936	0.00000	BQL	
A-579-01.024.MC.P01.01.01.dpcranalysis.xlsx	A-579-01.152.SCTH.12Sep2023.03	0.0399	0	310000	0.00000	BQL	
A-579-01.024.MC.P01.01.02.dpcranalysis.xlsx	A-579-01.152.SPLN.12Sep2023.01	0.075	183	314001	0.00058	IND	x
A-579-01.005.MC.P01.01.01.dpcranalysis.xlsx	A-579-01.201.BRAN.02Aug2023.03	0.0267	5012787	390864	12.82489		
A-579-01.071.MC.P01.03.01.dpcranalysis.xlsx	A-579-01.201.DRGC.02Aug2023.01	0.0038	56158421	312175	179.89378		
A-579-01.046.MC.P01.02.01.dpcranalysis.xlsx	A-579-01.201.DRGL.02Aug2023.02	0.019	4596495	254333	18.07272		
A-579-01.071.MC.P01.03.01.dpcranalysis.xlsx	A-579-01.201.DRGT.02Aug2023.01	0.0038	30886456	356791	86.56731		
A-579-01.006.MC.P01.01.02.dpcranalysis.xlsx	A-579-01.201.HTHT.02Aug2023.01	0.075	67125	385625	0.17407		
A-579-01.006.MC.P01.01.01.dpcranalysis.xlsx	A-579-01.201.KDNY.02Aug2023.02	0.075	28604	321446	0.08898		
A-579-01.006.MC.P01.01.01.dpcranalysis.xlsx	A-579-01.201.LUNG.02Aug2023.03	0.075	115173	320482	0.35938		
A-579-01.046.MC.P01.02.02.dpcranalysis.xlsx	A-579-01.201.LVER.02Aug2023.02	0.019	55348	392184	0.14113		
A-579-01.005.MC.P01.01.01.dpcranalysis.xlsx	A-579-01.201.MUAL.02Aug2023.02	0.075	22276	396586	0.05617		
A-579-01.005.MC.P01.01.01.dpcranalysis.xlsx	A-579-01.201.MUAR.02Aug2023.01	0.075	19009	417244	0.04556		
A-579-01.005.MC.P01.01.01.dpcranalysis.xlsx	A-579-01.201.MUBL.02Aug2023.01	0.0519	36012	402592	0.08945		
A-579-01.005.MC.P01.01.01.dpcranalysis.xlsx	A-579-01.201.MUBR.02Aug2023.01	0.075	14600	325671	0.04483		
A-579-01.006.MC.P01.01.01.dpcranalysis.xlsx	A-579-01.201.NFDLD.02Aug2023.05	0.0636	226223	465419	0.48606		
A-579-01.006.MC.P01.01.01.dpcranalysis.xlsx	A-579-01.201.NFDLP.02Aug2023.01	0.0621	263144	384260	0.68481		
A-579-01.006.MC.P01.01.01.dpcranalysis.xlsx	A-579-01.201.NFDRD.02Aug2023.12	0.075	207542	188714	1.09977		
A-579-01.006.MC.P01.01.01.dpcranalysis.xlsx	A-579-01.201.NFDRP.02Aug2023.07	0.0492	222919	338068	0.65939		
A-579-01.006.MC.P01.01.01.dpcranalysis.xlsx	A-579-01.201.NFPLD.02Aug2023.05	0.0603	183703	383402	0.47914		
A-579-01.006.MC.P01.01.01.dpcranalysis.xlsx	A-579-01.201.NFPLP.02Aug2023.02	0.0348	49563	304245	0.16291		
A-579-01.006.MC.P01.01.01.dpcranalysis.xlsx	A-579-01.201.NFPRD.02Aug2023.11	0.051	285884	384122	0.74425		
A-579-01.006.MC.P01.01.01.dpcranalysis.xlsx	A-579-01.201.NFPRP.02Aug2023.08	0.0582	70994	363816	0.19514		
A-579-01.005.MC.P01.01.01.dpcranalysis.xlsx	A-579-01.201.NMDLD.02Aug2023.06	0.0327	164175	386919	0.42431		
A-579-01.005.MC.P01.01.01.dpcranalysis.xlsx	A-579-01.201.NMDLP.02Aug2023.03	0.0351	171802	379343	0.45289		
A-579-01.005.MC.P01.01.01.dpcranalysis.xlsx	A-579-01.201.NMDRD.02Aug2023.10	0.0603	71161	289682	0.24565		
A-579-01.005.MC.P01.01.01.dpcranalysis.xlsx	A-579-01.201.NMDRP.02Aug2023.08	0.0471	230331	416210	0.55340		
A-579-01.005.MC.P01.01.01.dpcranalysis.xlsx	A-579-01.201.NMPLD.02Aug2023.06	0.075	168510	352371	0.47822		

IND: indeterminate, CV% >20 (only applicable to results above the lower limit of quantitation[LLOQ])
BQL: below quantitation limit (below LLOQ)
>ULOQ: above upper limit of quantitation
LLOQ = 14.9 copies/μL
ULOQ = 16800 copies/μL

Source.Name	Sample Name	DNA Input (ug)	AAV2-ITR.Target Copy Num Per ug DNA	B2M.Target Copy Num Per ug DNA	AAV2-ITR/B2M ratio	AAV2-ITR.Comment	AAV2-ITR.CV Flag
A-579-01.005.MC.P01.01.01.dpcranalysis.xlsx	A-579-01.201.NMPLP.02Aug2023.03	0.075	42044	349210	0.12040		
A-579-01.005.MC.P01.01.01.dpcranalysis.xlsx	A-579-01.201.NMPRD.02Aug2023.10	0.0147	1544413	355937	4.33901		
A-579-01.005.MC.P01.01.01.dpcranalysis.xlsx	A-579-01.201.NMPRP.02Aug2023.09	0.075	90564	359116	0.25219		
A-579-01.005.MC.P01.01.01.dpcranalysis.xlsx	A-579-01.201.NSDLD.02Aug2023.05	0.075	151300	379512	0.39867		
A-579-01.005.MC.P01.01.01.dpcranalysis.xlsx	A-579-01.201.NSDLP.02Aug2023.02	0.0693	198162	363481	0.54518		
A-579-01.005.MC.P01.01.01.dpcranalysis.xlsx	A-579-01.201.NSDRD.02Aug2023.12	0.075	90123	369581	0.24385		
A-579-01.005.MC.P01.01.01.dpcranalysis.xlsx	A-579-01.201.NSDRP.02Aug2023.07	0.075	73217	415707	0.17613		
A-579-01.005.MC.P01.01.01.dpcranalysis.xlsx	A-579-01.201.NSPLD.02Aug2023.05	0.075	106257	382324	0.27792		
A-579-01.005.MC.P01.01.01.dpcranalysis.xlsx	A-579-01.201.NSPLP.02Aug2023.01	0.075	150995	332105	0.45466		
A-579-01.005.MC.P01.01.01.dpcranalysis.xlsx	A-579-01.201.NSPRD.02Aug2023.10	0.075	227596	380707	0.59783		
A-579-01.005.MC.P01.01.01.dpcranalysis.xlsx	A-579-01.201.NSPRP.02Aug2023.07	0.075	216515	356303	0.60767		
A-579-01.005.MC.P01.01.01.dpcranalysis.xlsx	A-579-01.201.NUDLD.02Aug2023.04	0.075	71052	399205	0.17798		
A-579-01.005.MC.P01.01.01.dpcranalysis.xlsx	A-579-01.201.NUDLP.02Aug2023.03	0.0615	144477	366345	0.39437		
A-579-01.005.MC.P01.01.01.dpcranalysis.xlsx	A-579-01.201.NUDRD.02Aug2023.10	0.0717	72063	388401	0.18554		
A-579-01.005.MC.P01.01.01.dpcranalysis.xlsx	A-579-01.201.NUDRP.02Aug2023.09	0.0465	163082	355088	0.45927		
A-579-01.006.MC.P01.01.01.dpcranalysis.xlsx	A-579-01.201.NUPLD.02Aug2023.04	0.0243	263311	371970	0.70788		
A-579-01.005.MC.P01.01.01.dpcranalysis.xlsx	A-579-01.201.NUPLP.02Aug2023.03	0.0465	144616	373394	0.38730		
A-579-01.006.MC.P01.01.01.dpcranalysis.xlsx	A-579-01.201.NUPRD.02Aug2023.10	0.0519	202483	364962	0.55481		
A-579-01.006.MC.P01.01.02.dpcranalysis.xlsx	A-579-01.201.NUPRP.02Aug2023.09	0.0636	73863	334492	0.22082		
A-579-01.005.MC.P01.01.02.dpcranalysis.xlsx	A-579-01.201.NVOT.02Aug2023.08	0.0372	823234	322884	2.54962		
A-579-01.071.MC.P01.03.01.dpcranalysis.xlsx	A-579-01.201.SCCV.02Aug2023.02	0.0038	26474123	378896	69.87165		
A-579-01.046.MC.P01.02.01.dpcranalysis.xlsx	A-579-01.201.SCLU.02Aug2023.03	0.012	6768767	420117	16.11164		
A-579-01.046.MC.P01.02.02.dpcranalysis.xlsx	A-579-01.201.SCSA.02Aug2023.02	0.019	8957384	324726	27.58441		
A-579-01.006.MC.P01.01.01.dpcranalysis.xlsx	A-579-01.201.SCSR.02Aug2023.01	0.0573	2322192	341978	6.79047		
A-579-01.046.MC.P01.02.01.dpcranalysis.xlsx	A-579-01.201.SCTH.02Aug2023.02	0.013	10639390	344508	30.88288		
A-579-01.046.MC.P01.02.01.dpcranalysis.xlsx	A-579-01.201.SPLN.02Aug2023.02	0.019	3320014	299281	11.09331		
A-579-01.006.MC.P01.01.02.dpcranalysis.xlsx	A-579-01.201.TEST.02Aug2023.02	0.075	19348	304864	0.06346		
A-579-01.007.MC.P01.01.01.dpcranalysis.xlsx	A-579-01.202.BRAN.02Aug2023.02	0.0297	246815	400438	0.61636		
A-579-01.007.MC.P01.01.01.dpcranalysis.xlsx	A-579-01.202.DRGC.02Aug2023.03	0.075	2705043	369762	7.31564		
A-579-01.007.MC.P01.01.01.dpcranalysis.xlsx	A-579-01.202.DRGL.02Aug2023.03	0.075	747836	413398	1.80900		
A-579-01.007.MC.P01.01.01.dpcranalysis.xlsx	A-579-01.202.DRGT.02Aug2023.01	0.075	1715442	380377	4.50985		
A-579-01.008.MC.P01.01.01.dpcranalysis.xlsx	A-579-01.202.HTHT.02Aug2023.02	0.075	130094	318229	0.40881		
A-579-01.008.MC.P01.01.01.dpcranalysis.xlsx	A-579-01.202.KDNY.02Aug2023.03	0.075	147622	345178	0.42767		
A-579-01.008.MC.P01.01.01.dpcranalysis.xlsx	A-579-01.202.LUNG.02Aug2023.01	0.075	54538	510571	0.10682		
A-579-01.047.MC.P01.02.01.dpcranalysis.xlsx	A-579-01.202.LVER.02Aug2023.02	0.019	7674775	187575	40.91567		
A-579-01.007.MC.P01.01.01.dpcranalysis.xlsx	A-579-01.202.MUAL.02Aug2023.01	0.0585	78041	322072	0.24231		
A-579-01.007.MC.P01.01.01.dpcranalysis.xlsx	A-579-01.202.MUAR.02Aug2023.02	0.075	25542	337220	0.07574		
A-579-01.007.MC.P01.01.01.dpcranalysis.xlsx	A-579-01.202.MUBL.02Aug2023.03	0.075	22475	397158	0.05659		
A-579-01.007.MC.P01.01.01.dpcranalysis.xlsx	A-579-01.202.MUBR.02Aug2023.03	0.0348	95452	318057	0.30011		

IND: indeterminate, CV% >20 (only applicable to results above the lower limit of quantitation[LLOQ])
BQL: below quantitation limit (below LLOQ)
>ULOQ: above upper limit of quantitation
LLOQ = 14.9 copies/μL
ULOQ = 16800 copies/μL

Source.Name	Sample Name	DNA Input (ug)	AAV2-ITR.Target Copy Num Per ug DNA	B2M.Target Copy Num Per ug DNA	AAV2-ITR/B2M ratio	AAV2-ITR.Comment	AAV2-ITR.CV Flag
A-579-01.008.MC.P01.01.01.dpcranalysis.xlsx	A-579-01.202.NFDLD.02Aug2023.05	0.0246	340913	378436	0.90085		
A-579-01.008.MC.P01.01.01.dpcranalysis.xlsx	A-579-01.202.NFDLP.02Aug2023.01	0.0276	619162	379691	1.63070		
A-579-01.008.MC.P01.01.02.dpcranalysis.xlsx	A-579-01.202.NFDRD.02Aug2023.12	0.075	278955	350495	0.79589		
A-579-01.008.MC.P01.01.01.dpcranalysis.xlsx	A-579-01.202.NFDRP.02Aug2023.07	0.0081	1641054	473580	3.46521		
A-579-01.008.MC.P01.01.01.dpcranalysis.xlsx	A-579-01.202.NFPLD.02Aug2023.04	0.0531	418831	337318	1.24165		
A-579-01.008.MC.P01.01.01.dpcranalysis.xlsx	A-579-01.202.NFPLP.02Aug2023.03	0.0228	588246	312503	1.88237		
A-579-01.008.MC.P01.01.01.dpcranalysis.xlsx	A-579-01.202.NFPRD.02Aug2023.10	0.0366	540199	360710	1.49760		
A-579-01.008.MC.P01.01.01.dpcranalysis.xlsx	A-579-01.202.NFPRP.02Aug2023.07	0.0441	410339	342063	1.19960		
A-579-01.007.MC.P01.01.02.dpcranalysis.xlsx	A-579-01.202.NMDLD.02Aug2023.06	0.0435	83871	332782	0.25203		
A-579-01.007.MC.P01.01.01.dpcranalysis.xlsx	A-579-01.202.NMDLP.02Aug2023.03	0.0165	744602	345446	2.15548		
A-579-01.007.MC.P01.01.01.dpcranalysis.xlsx	A-579-01.202.NMDRD.02Aug2023.12	0.0636	87258	392455	0.22234		
A-579-01.007.MC.P01.01.01.dpcranalysis.xlsx	A-579-01.202.NMDRP.02Aug2023.09	0.0684	206821	348683	0.59315		
A-579-01.007.MC.P01.01.01.dpcranalysis.xlsx	A-579-01.202.NMPLD.02Aug2023.05	0.075	256188	398440	0.64298		
A-579-01.007.MC.P01.01.01.dpcranalysis.xlsx	A-579-01.202.NMPLP.02Aug2023.03	0.0456	526105	378256	1.39087		
A-579-01.007.MC.P01.01.01.dpcranalysis.xlsx	A-579-01.202.NMPRD.02Aug2023.11	0.0642	244142	368390	0.66273		
A-579-01.007.MC.P01.01.01.dpcranalysis.xlsx	A-579-01.202.NMPRP.02Aug2023.09	0.075	313986	335863	0.93486		
A-579-01.007.MC.P01.01.01.dpcranalysis.xlsx	A-579-01.202.NSDLD.02Aug2023.04	0.075	158940	382343	0.41570		
A-579-01.007.MC.P01.01.01.dpcranalysis.xlsx	A-579-01.202.NSDLP.02Aug2023.01	0.0219	719775	508858	1.41449		
A-579-01.007.MC.P01.01.02.dpcranalysis.xlsx	A-579-01.202.NSDRD.02Aug2023.11	0.075	85073	390292	0.21797		
A-579-01.007.MC.P01.01.01.dpcranalysis.xlsx	A-579-01.202.NSDRP.02Aug2023.09	0.075	94385	471819	0.20004		
A-579-01.007.MC.P01.01.01.dpcranalysis.xlsx	A-579-01.202.NSPLD.02Aug2023.04	0.0351	507124	434106	1.16820		
A-579-01.007.MC.P01.01.01.dpcranalysis.xlsx	A-579-01.202.NSPLP.02Aug2023.02	0.0405	606713	422224	1.43695		
A-579-01.007.MC.P01.01.01.dpcranalysis.xlsx	A-579-01.202.NSPRD.02Aug2023.10	0.075	165057	393369	0.41960		
A-579-01.007.MC.P01.01.01.dpcranalysis.xlsx	A-579-01.202.NSPRP.02Aug2023.09	0.075	110326	467743	0.23587		
A-579-01.007.MC.P01.01.01.dpcranalysis.xlsx	A-579-01.202.NUDLD.02Aug2023.05	0.0327	130781	410196	0.31883		
A-579-01.007.MC.P01.01.01.dpcranalysis.xlsx	A-579-01.202.NUDLP.02Aug2023.01	0.0138	757894	384459	1.97133		
A-579-01.007.MC.P01.01.01.dpcranalysis.xlsx	A-579-01.202.NUDRD.02Aug2023.12	0.0642	70080	330737	0.21189		
A-579-01.007.MC.P01.01.01.dpcranalysis.xlsx	A-579-01.202.NUDRP.02Aug2023.09	0.06	170668	363774	0.46916		
A-579-01.008.MC.P01.01.01.dpcranalysis.xlsx	A-579-01.202.NUPLD.02Aug2023.04	0.075	319337	351388	0.90879		
A-579-01.007.MC.P01.01.01.dpcranalysis.xlsx	A-579-01.202.NUPLP.02Aug2023.03	0.051	416495	319676	1.30287		
A-579-01.008.MC.P01.01.02.dpcranalysis.xlsx	A-579-01.202.NUPRD.02Aug2023.12	0.075	433552	301420	1.43837		
A-579-01.008.MC.P01.01.01.dpcranalysis.xlsx	A-579-01.202.NUPRP.02Aug2023.07	0.0201	777940	394345	1.97274		
A-579-01.007.MC.P01.01.01.dpcranalysis.xlsx	A-579-01.202.NVOT.02Aug2023.07	0.0489	526398	333175	1.57994		
A-579-01.008.MC.P01.01.01.dpcranalysis.xlsx	A-579-01.202.SCCV.02Aug2023.02	0.0162	751449	239757	3.13421		
A-579-01.008.MC.P01.01.01.dpcranalysis.xlsx	A-579-01.202.SCLU.02Aug2023.02	0.0204	1677003	412405	4.06640		
A-579-01.008.MC.P01.01.01.dpcranalysis.xlsx	A-579-01.202.SCSA.02Aug2023.01	0.075	792475	366900	2.15992		
A-579-01.008.MC.P01.01.01.dpcranalysis.xlsx	A-579-01.202.SCSR.02Aug2023.01	0.0441	512952	431397	1.18905		
A-579-01.008.MC.P01.01.01.dpcranalysis.xlsx	A-579-01.202.SCTH.02Aug2023.03	0.0438	446977	432828	1.03269		
A-579-01.047.MC.P01.02.02.dpcranalysis.xlsx	A-579-01.202.SPLN.02Aug2023.01	0.019	6983863	343074	20.35674		

IND: indeterminate, CV% >20 (only applicable to results above the lower limit of quantitation[LLOQ])
BQL: below quantitation limit (below LLOQ)
>ULOQ: above upper limit of quantitation
LLOQ = 14.9 copies/µL
ULOQ = 16800 copies/µL

Source.Name	Sample Name	DNA Input (ug)	AAV2-ITR.Target Copy Num Per ug DNA	B2M.Target Copy Num Per ug DNA	AAV2-ITR/B2M ratio	AAV2-ITR.Comment	AAV2-ITR.CV Flag
A-579-01.008.MC.P01.01.01.dpcranalysis.xlsx	A-579-01.202.TEST.02Aug2023.02	0.075	47252	409491	0.11539		
A-579-01.025.MC.P01.01.01.dpcranalysis.xlsx	A-579-01.203.BRAN.13Sep2023.02	0.0618	44707	334550	0.13363		
A-579-01.025.MC.P01.01.01.dpcranalysis.xlsx	A-579-01.203.DRGC.13Sep2023.03	0.075	318105	337232	0.94328		
A-579-01.025.MC.P01.01.01.dpcranalysis.xlsx	A-579-01.203.DRGL.13Sep2023.03	0.0549	723780	274849	2.63337		
A-579-01.025.MC.P01.01.01.dpcranalysis.xlsx	A-579-01.203.DRGT.13Sep2023.02	0.075	982819	355034	2.76824		
A-579-01.026.MC.P01.01.01.dpcranalysis.xlsx	A-579-01.203.HTHT.13Sep2023.03	0.0513	66289	253728	0.26126		
A-579-01.065.MC.P01.02.01.dpcranalysis.xlsx	A-579-01.203.KDNY.13Sep2023.02	0.075	14382	417909	0.03441		
A-579-01.026.MC.P01.01.01.dpcranalysis.xlsx	A-579-01.203.LUNG.13Sep2023.02	0.075	45702	296912	0.15393		
A-579-01.054.MC.P01.02.01.dpcranalysis.xlsx	A-579-01.203.LVER.13Sep2023.02	0.00375	2920836	185323	15.76081		
A-579-01.025.MC.P01.01.01.dpcranalysis.xlsx	A-579-01.203.MUAL.13Sep2023.01	0.075	22388	337232	0.06639		
A-579-01.025.MC.P01.01.01.dpcranalysis.xlsx	A-579-01.203.MUAR.13Sep2023.01	0.075	12067	372823	0.03237		
A-579-01.025.MC.P01.01.01.dpcranalysis.xlsx	A-579-01.203.MUBL.13Sep2023.03	0.075	3053	283335	0.01077		
A-579-01.025.MC.P01.01.01.dpcranalysis.xlsx	A-579-01.203.MUBR.13Sep2023.02	0.075	3336	285973	0.01166		
A-579-01.026.MC.P01.01.01.dpcranalysis.xlsx	A-579-01.203.NFDLD.13Sep2023.04	0.075	19216	312517	0.06149		
A-579-01.026.MC.P01.01.01.dpcranalysis.xlsx	A-579-01.203.NFDLP.13Sep2023.01	0.075	18169	297441	0.06108		
A-579-01.026.MC.P01.01.01.dpcranalysis.xlsx	A-579-01.203.NFDRD.13Sep2023.12	0.075	51557	322311	0.15996		
A-579-01.026.MC.P01.01.01.dpcranalysis.xlsx	A-579-01.203.NFDRP.13Sep2023.08	0.075	16677	273890	0.06089		
A-579-01.026.MC.P01.01.02.dpcranalysis.xlsx	A-579-01.203.NFPLD.13Sep2023.06	0.0411	35315	264535	0.13350		
A-579-01.026.MC.P01.01.02.dpcranalysis.xlsx	A-579-01.203.NFPLP.13Sep2023.03	0.075	10843	258851	0.04189		
A-579-01.026.MC.P01.01.01.dpcranalysis.xlsx	A-579-01.203.NFPRD.13Sep2023.12	0.0486	9843	318063	0.03095		
A-579-01.026.MC.P01.01.01.dpcranalysis.xlsx	A-579-01.203.NFPRP.13Sep2023.09	0.0384	20273	338832	0.05983		
A-579-01.025.MC.P01.01.01.dpcranalysis.xlsx	A-579-01.203.NMDLD.13Sep2023.04	0.0603	19067	279296	0.06827		
A-579-01.025.MC.P01.01.01.dpcranalysis.xlsx	A-579-01.203.NMDLP.13Sep2023.03	0.0468	31869	330256	0.09650	IND	x
A-579-01.025.MC.P01.01.01.dpcranalysis.xlsx	A-579-01.203.NMDRD.13Sep2023.11	0.0693	21421	285886	0.07493		
A-579-01.025.MC.P01.01.01.dpcranalysis.xlsx	A-579-01.203.NMDRP.13Sep2023.07	0.075	10688	338315	0.03159		
A-579-01.025.MC.P01.01.01.dpcranalysis.xlsx	A-579-01.203.NMPLD.13Sep2023.05	0.0522	44503	386395	0.11518		
A-579-01.025.MC.P01.01.02.dpcranalysis.xlsx	A-579-01.203.NMPLP.13Sep2023.03	0.0525	9285	297227	0.03124		
A-579-01.025.MC.P01.01.01.dpcranalysis.xlsx	A-579-01.203.NMPRD.13Sep2023.10	0.0684	13021	298039	0.04369		
A-579-01.025.MC.P01.01.01.dpcranalysis.xlsx	A-579-01.203.NMPRP.13Sep2023.09	0.0381	7002	379445	0.01845		
A-579-01.025.MC.P01.01.02.dpcranalysis.xlsx	A-579-01.203.NSDLD.13Sep2023.06	0.0642	100769	320375	0.31454		
A-579-01.025.MC.P01.01.01.dpcranalysis.xlsx	A-579-01.203.NSDLP.13Sep2023.03	0.0471	218754	268244	0.81550		
A-579-01.025.MC.P01.01.01.dpcranalysis.xlsx	A-579-01.203.NSDRD.13Sep2023.12	0.075	133050	384484	0.34605		
A-579-01.025.MC.P01.01.01.dpcranalysis.xlsx	A-579-01.203.NSDRP.13Sep2023.08	0.075	92419	272614	0.33901		
A-579-01.025.MC.P01.01.01.dpcranalysis.xlsx	A-579-01.203.NSPLD.13Sep2023.04	0.0288	396683	375407	1.05667		
A-579-01.025.MC.P01.01.02.dpcranalysis.xlsx	A-579-01.203.NSPLP.13Sep2023.02	0.0627	143963	288329	0.49930		
A-579-01.025.MC.P01.01.02.dpcranalysis.xlsx	A-579-01.203.NSPRD.13Sep2023.10	0.075	62627	335300	0.18678		
A-579-01.025.MC.P01.01.01.dpcranalysis.xlsx	A-579-01.203.NSPRP.13Sep2023.08	0.0663	92538	370222	0.24995		
A-579-01.025.MC.P01.01.02.dpcranalysis.xlsx	A-579-01.203.NUDLD.13Sep2023.04	0.0645	66180	272023	0.24329		
A-579-01.065.MC.P01.02.02.dpcranalysis.xlsx	A-579-01.203.NUDLP.13Sep2023.02	0.0699	19880	302893	0.06564		

IND: indeterminate, CV% >20 (only applicable to results above the lower limit of quantitation[LLOQ])
BQL: below quantitation limit (below LLOQ)
>ULOQ: above upper limit of quantitation
LLOQ = 14.9 copies/μL
ULOQ = 16800 copies/μL

Source.Name	Sample Name	DNA Input (ug)	AAV2-ITR.Target Copy Num Per ug DNA	B2M.Target Copy Num Per ug DNA	AAV2-ITR/B2M ratio	AAV2-ITR.Comment	AAV2-ITR.CV Flag
A-579-01.025.MC.P01.01.02.dpcranalysis.xlsx	A-579-01.203.NUDRD.13Sep2023.11	0.0558	23651	225518	0.10487		
A-579-01.025.MC.P01.01.02.dpcranalysis.xlsx	A-579-01.203.NUDRP.13Sep2023.09	0.06	7767	222390	0.03492		
A-579-01.026.MC.P01.01.01.dpcranalysis.xlsx	A-579-01.203.NUPLD.13Sep2023.06	0.0447	37972	355157	0.10692		
A-579-01.065.MC.P01.02.01.dpcranalysis.xlsx	A-579-01.203.NUPLP.13Sep2023.02	0.0639	10430	396922	0.02628		
A-579-01.026.MC.P01.01.01.dpcranalysis.xlsx	A-579-01.203.NUPRD.13Sep2023.10	0.0474	14667	227869	0.06436		
A-579-01.026.MC.P01.01.02.dpcranalysis.xlsx	A-579-01.203.NUPRP.13Sep2023.07	0.075	7720	283855	0.02720		
A-579-01.025.MC.P01.01.02.dpcranalysis.xlsx	A-579-01.203.NVOT.13Sep2023.09	0.0741	173489	307821	0.56360		
A-579-01.026.MC.P01.01.01.dpcranalysis.xlsx	A-579-01.203.SCCV.13Sep2023.03	0.075	233140	372300	0.62622		
A-579-01.026.MC.P01.01.01.dpcranalysis.xlsx	A-579-01.203.SCLU.13Sep2023.01	0.0654	285195	357942	0.79676		
A-579-01.026.MC.P01.01.01.dpcranalysis.xlsx	A-579-01.203.SCSA.13Sep2023.02	0.075	154249	352190	0.43797		
A-579-01.026.MC.P01.01.01.dpcranalysis.xlsx	A-579-01.203.SCSR.13Sep2023.03	0.06	547454	365626	1.49731		
A-579-01.026.MC.P01.01.01.dpcranalysis.xlsx	A-579-01.203.SCTH.13Sep2023.01	0.0723	765798	368086	2.08049		
A-579-01.026.MC.P01.01.01.dpcranalysis.xlsx	A-579-01.203.SPLN.13Sep2023.02	0.075	751252	216502	3.46995		
A-579-01.026.MC.P01.01.01.dpcranalysis.xlsx	A-579-01.203.TEST.13Sep2023.02	0.075	9809	278581	0.03521		
A-579-01.027.MC.P01.01.02.dpcranalysis.xlsx	A-579-01.204.BRAN.13Sep2023.02	0.0516	27491	357502	0.07690		
A-579-01.027.MC.P01.01.01.dpcranalysis.xlsx	A-579-01.204.DRGC.13Sep2023.03	0.075	190829	398627	0.47872		
A-579-01.027.MC.P01.01.01.dpcranalysis.xlsx	A-579-01.204.DRGL.13Sep2023.02	0.075	20452	400954	0.05101		
A-579-01.055.MC.P01.02.01.dpcranalysis.xlsx	A-579-01.204.DRGT.13Sep2023.03	0.00375	1960871	365406	5.36628		
A-579-01.028.MC.P01.01.01.dpcranalysis.xlsx	A-579-01.204.HTHT.13Sep2023.02	0.0495	61195	374578	0.16337		
A-579-01.028.MC.P01.01.01.dpcranalysis.xlsx	A-579-01.204.KDNY.13Sep2023.01	0.075	12523	269796	0.04642		
A-579-01.028.MC.P01.01.01.dpcranalysis.xlsx	A-579-01.204.LUNG.13Sep2023.01	0.075	17372	387931	0.04478		
A-579-01.055.MC.P01.02.01.dpcranalysis.xlsx	A-579-01.204.LVER.13Sep2023.02	0.00375	4951644	358164	13.82509		
A-579-01.027.MC.P01.01.02.dpcranalysis.xlsx	A-579-01.204.MUAL.13Sep2023.02	0.075	8142	366147	0.02224		
A-579-01.027.MC.P01.01.01.dpcranalysis.xlsx	A-579-01.204.MUAR.13Sep2023.03	0.075	12255	412403	0.02972		
A-579-01.027.MC.P01.01.02.dpcranalysis.xlsx	A-579-01.204.MUBL.13Sep2023.02	0.075	7957	391795	0.02031		
A-579-01.066.MC.P01.02.01.dpcranalysis.xlsx	A-579-01.204.MUBR.13Sep2023.02	0.075	12279	404028	0.03039		
A-579-01.028.MC.P01.01.01.dpcranalysis.xlsx	A-579-01.204.NFDLD.13Sep2023.06	0.0399	5044	381251	0.01323	BQL	
A-579-01.028.MC.P01.01.01.dpcranalysis.xlsx	A-579-01.204.NFDLP.13Sep2023.01	0.0369	41966	411678	0.10194		
A-579-01.028.MC.P01.01.01.dpcranalysis.xlsx	A-579-01.204.NFDRD.13Sep2023.11	0.036	9359	385726	0.02426		
A-579-01.066.MC.P01.02.01.dpcranalysis.xlsx	A-579-01.204.NFDRP.13Sep2023.07	0.0498	8995	398194	0.02259		
A-579-01.043.MC.P01.02.01.dpcranalysis.xlsx	A-579-01.204.NFPLD.13Sep2023.05	0.0456	4665	381469	0.01223		
A-579-01.028.MC.P01.01.02.dpcranalysis.xlsx	A-579-01.204.NFPLP.13Sep2023.01	0.0741	2331	321386	0.00725	BQL	
A-579-01.028.MC.P01.01.01.dpcranalysis.xlsx	A-579-01.204.NFPRD.13Sep2023.12	0.0381	12465	367062	0.03396		
A-579-01.028.MC.P01.01.02.dpcranalysis.xlsx	A-579-01.204.NFPRP.13Sep2023.08	0.075	17271	365736	0.04722		
A-579-01.027.MC.P01.01.01.dpcranalysis.xlsx	A-579-01.204.NMDLD.13Sep2023.06	0.0426	166346	449424	0.37013		
A-579-01.027.MC.P01.01.01.dpcranalysis.xlsx	A-579-01.204.NMDLP.13Sep2023.03	0.0306	54566	431133	0.12657		
A-579-01.027.MC.P01.01.01.dpcranalysis.xlsx	A-579-01.204.NMDRD.13Sep2023.12	0.0459	76466	425307	0.17979		
A-579-01.027.MC.P01.01.01.dpcranalysis.xlsx	A-579-01.204.NMDRP.13Sep2023.09	0.0192	46020	436163	0.10551		
A-579-01.027.MC.P01.01.02.dpcranalysis.xlsx	A-579-01.204.NMPLD.13Sep2023.06	0.0303	34935	422356	0.08272		

IND: indeterminate, CV% >20 (only applicable to results above the lower limit of quantitation[LLOQ])
BQL: below quantitation limit (below LLOQ)
>ULOQ: above upper limit of quantitation
LLOQ = 14.9 copies/μL
ULOQ = 16800 copies/μL

Source.Name	Sample Name	DNA Input (ug)	AAV2-ITR.Target Copy Num Per ug DNA	B2M.Target Copy Num Per ug DNA	AAV2-ITR/B2M ratio	AAV2-ITR.Comment	AAV2-ITR.CV Flag
A-579-01.027.MC.P01.01.02.dpcranalysis.xlsx	A-579-01.204.NMPLP.13Sep2023.03	0.036	18601	388694	0.04785		
A-579-01.027.MC.P01.01.01.dpcranalysis.xlsx	A-579-01.204.NMPRD.13Sep2023.11	0.0276	33135	460918	0.07189		
A-579-01.027.MC.P01.01.01.dpcranalysis.xlsx	A-579-01.204.NMPRP.13Sep2023.07	0.075	25368	360777	0.07031		
A-579-01.027.MC.P01.01.01.dpcranalysis.xlsx	A-579-01.204.NSDLD.13Sep2023.04	0.075	24559	324868	0.07560		
A-579-01.027.MC.P01.01.01.dpcranalysis.xlsx	A-579-01.204.NSDLP.13Sep2023.03	0.075	65259	400537	0.16293		
A-579-01.027.MC.P01.01.01.dpcranalysis.xlsx	A-579-01.204.NSDRD.13Sep2023.11	0.0387	12520	349928	0.03578		
A-579-01.027.MC.P01.01.01.dpcranalysis.xlsx	A-579-01.204.NSDRP.13Sep2023.09	0.0354	14770	402375	0.03671		
A-579-01.027.MC.P01.01.01.dpcranalysis.xlsx	A-579-01.204.NSPLD.13Sep2023.05	0.075	17086	358655	0.04764		
A-579-01.027.MC.P01.01.01.dpcranalysis.xlsx	A-579-01.204.NSPLP.13Sep2023.02	0.075	8316	364796	0.02280		
A-579-01.043.MC.P01.02.01.dpcranalysis.xlsx	A-579-01.204.NSPRD.13Sep2023.12	0.0558	7877	405155	0.01944		
A-579-01.027.MC.P01.01.01.dpcranalysis.xlsx	A-579-01.204.NSPRP.13Sep2023.08	0.075	4468	341955	0.01307		
A-579-01.027.MC.P01.01.01.dpcranalysis.xlsx	A-579-01.204.NUDLD.13Sep2023.06	0.075	60965	360441	0.16914		
A-579-01.027.MC.P01.01.01.dpcranalysis.xlsx	A-579-01.204.NUDLP.13Sep2023.03	0.0696	22667	368425	0.06152		
A-579-01.027.MC.P01.01.01.dpcranalysis.xlsx	A-579-01.204.NUDRD.13Sep2023.11	0.075	56516	375648	0.15045		
A-579-01.066.MC.P01.02.02.dpcranalysis.xlsx	A-579-01.204.NUDRP.13Sep2023.09	0.075	40572	287681	0.14103		
A-579-01.028.MC.P01.01.01.dpcranalysis.xlsx	A-579-01.204.NUPLD.13Sep2023.04	0.0402	33834	438168	0.07722		
A-579-01.027.MC.P01.01.01.dpcranalysis.xlsx	A-579-01.204.NUPLP.13Sep2023.03	0.075	2997	380153	0.00788		
A-579-01.028.MC.P01.01.01.dpcranalysis.xlsx	A-579-01.204.NUPRD.13Sep2023.12	0.0606	15921	333274	0.04777		
A-579-01.028.MC.P01.01.01.dpcranalysis.xlsx	A-579-01.204.NUPRP.13Sep2023.09	0.0561	12094	404536	0.02990		
A-579-01.027.MC.P01.01.01.dpcranalysis.xlsx	A-579-01.204.NVOT.13Sep2023.08	0.036	690511	346046	1.99543		
A-579-01.028.MC.P01.01.01.dpcranalysis.xlsx	A-579-01.204.SCCV.13Sep2023.03	0.075	143391	393954	0.36398		
A-579-01.028.MC.P01.01.01.dpcranalysis.xlsx	A-579-01.204.SCLU.13Sep2023.02	0.075	433913	370197	1.17211		
A-579-01.028.MC.P01.01.01.dpcranalysis.xlsx	A-579-01.204.SCSA.13Sep2023.03	0.075	121140	352738	0.34343		
A-579-01.028.MC.P01.01.01.dpcranalysis.xlsx	A-579-01.204.SCSR.13Sep2023.03	0.075	28572	369625	0.07730		
A-579-01.028.MC.P01.01.01.dpcranalysis.xlsx	A-579-01.204.SCTH.13Sep2023.01	0.0495	198828	280198	0.70960		
A-579-01.028.MC.P01.01.01.dpcranalysis.xlsx	A-579-01.204.SPLN.13Sep2023.01	0.075	1208206	332708	3.63143		
A-579-01.043.MC.P01.02.01.dpcranalysis.xlsx	A-579-01.204.TEST.13Sep2023.02	0.075	9762	456711	0.02137		
A-579-01.009.MC.P01.01.01.dpcranalysis.xlsx	A-579-01.251.BRAN.02Aug2023.03	0.0456	211596	341363	0.61986		
A-579-01.072.MC.P01.03.01.dpcranalysis.xlsx	A-579-01.251.DRGC.02Aug2023.03	0.0038	45242596	290463	155.76019		
A-579-01.072.MC.P01.03.01.dpcranalysis.xlsx	A-579-01.251.DRGL.02Aug2023.01	0.0038	42146877	286521	147.09871		
A-579-01.048.MC.P01.02.01.dpcranalysis.xlsx	A-579-01.251.DRGT.02Aug2023.03	0.019	963789	38959	24.73837		
A-579-01.010.MC.P01.01.01.dpcranalysis.xlsx	A-579-01.251.HTHT.02Aug2023.03	0.0711	263657	351667	0.74973		
A-579-01.010.MC.P01.01.01.dpcranalysis.xlsx	A-579-01.251.KDNY.02Aug2023.02	0.075	201413	337773	0.59630		
A-579-01.010.MC.P01.01.01.dpcranalysis.xlsx	A-579-01.251.LUNG.02Aug2023.02	0.075	78829	338246	0.23305		
A-579-01.048.MC.P01.02.01.dpcranalysis.xlsx	A-579-01.251.LVER.02Aug2023.02	0.019	7579109	314484	24.10012		
A-579-01.009.MC.P01.01.01.dpcranalysis.xlsx	A-579-01.251.MUAL.02Aug2023.03	0.075	76596	368685	0.20775		
A-579-01.009.MC.P01.01.01.dpcranalysis.xlsx	A-579-01.251.MUAR.02Aug2023.01	0.075	48813	367870	0.13269		
A-579-01.009.MC.P01.01.01.dpcranalysis.xlsx	A-579-01.251.MUBL.02Aug2023.03	0.075	79532	346540	0.22950		
A-579-01.009.MC.P01.01.01.dpcranalysis.xlsx	A-579-01.251.MUBR.02Aug2023.01	0.0645	90078	400559	0.22488		

IND: indeterminate, CV% >20 (only applicable to results above the lower limit of quantitation[LLOQ])
BQL: below quantitation limit (below LLOQ)
>ULOQ: above upper limit of quantitation
LLOQ = 14.9 copies/μL
ULOQ = 16800 copies/μL

Source.Name	Sample Name	DNA Input (ug)	AAV2-ITR.Target Copy Num Per ug DNA	B2M.Target Copy Num Per ug DNA	AAV2-ITR/B2M ratio	AAV2-ITR.Comment	AAV2-ITR.CV Flag
A-579-01.010.MC.P01.01.01.dpcranalysis.xlsx	A-579-01.251.NFDLD.02Aug2023.05	0.0297	152382	396824	0.38400		
A-579-01.010.MC.P01.01.01.dpcranalysis.xlsx	A-579-01.251.NFDLP.02Aug2023.01	0.0564	208709	309531	0.67428		
A-579-01.010.MC.P01.01.01.dpcranalysis.xlsx	A-579-01.251.NFDRD.02Aug2023.10	0.0597	551613	366306	1.50588		
A-579-01.010.MC.P01.01.01.dpcranalysis.xlsx	A-579-01.251.NFDRP.02Aug2023.07	0.0129	421628	441778	0.95439		
A-579-01.010.MC.P01.01.01.dpcranalysis.xlsx	A-579-01.251.NFPLD.02Aug2023.06	0.0486	418809	353793	1.18377		
A-579-01.010.MC.P01.01.01.dpcranalysis.xlsx	A-579-01.251.NFPLP.02Aug2023.01	0.0564	80004	323696	0.24716		
A-579-01.010.MC.P01.01.01.dpcranalysis.xlsx	A-579-01.251.NFPRD.02Aug2023.12	0.036	302724	381980	0.79251		
A-579-01.010.MC.P01.01.01.dpcranalysis.xlsx	A-579-01.251.NFPRP.02Aug2023.09	0.0516	240514	340766	0.70580		
A-579-01.009.MC.P01.01.01.dpcranalysis.xlsx	A-579-01.251.NMDLD.02Aug2023.05	0.0369	131223	288410	0.45499		
A-579-01.009.MC.P01.01.01.dpcranalysis.xlsx	A-579-01.251.NMDLP.02Aug2023.01	0.0489	22256	355554	0.06259		
A-579-01.009.MC.P01.01.01.dpcranalysis.xlsx	A-579-01.251.NMDRD.02Aug2023.10	0.0603	44074	330227	0.13347		
A-579-01.009.MC.P01.01.01.dpcranalysis.xlsx	A-579-01.251.NMDRP.02Aug2023.09	0.0612	108584	322671	0.33652		
A-579-01.009.MC.P01.01.01.dpcranalysis.xlsx	A-579-01.251.NMPLD.02Aug2023.06	0.0618	61414	328947	0.18670		
A-579-01.009.MC.P01.01.01.dpcranalysis.xlsx	A-579-01.251.NMPLP.02Aug2023.03	0.075	31696	387364	0.08182		
A-579-01.009.MC.P01.01.01.dpcranalysis.xlsx	A-579-01.251.NMPRD.02Aug2023.12	0.0516	44795	350081	0.12795		
A-579-01.009.MC.P01.01.01.dpcranalysis.xlsx	A-579-01.251.NMPRP.02Aug2023.08	0.075	405204	325615	1.24442		
A-579-01.009.MC.P01.01.01.dpcranalysis.xlsx	A-579-01.251.NSDLD.02Aug2023.05	0.0345	214910	341248	0.62978		
A-579-01.009.MC.P01.01.02.dpcranalysis.xlsx	A-579-01.251.NSDLP.02Aug2023.01	0.0114	806965	269193	2.99772		
A-579-01.009.MC.P01.01.02.dpcranalysis.xlsx	A-579-01.251.NSDRD.02Aug2023.10	0.075	108929	400316	0.27211		
A-579-01.009.MC.P01.01.01.dpcranalysis.xlsx	A-579-01.251.NSDRP.02Aug2023.08	0.0399	157883	370105	0.42659		
A-579-01.009.MC.P01.01.01.dpcranalysis.xlsx	A-579-01.251.NSPLD.02Aug2023.06	0.075	191047	358033	0.53360		
A-579-01.009.MC.P01.01.01.dpcranalysis.xlsx	A-579-01.251.NSPLP.02Aug2023.02	0.075	317576	374764	0.84740		
A-579-01.009.MC.P01.01.01.dpcranalysis.xlsx	A-579-01.251.NSPRD.02Aug2023.12	0.0216	467380	458046	1.02038		
A-579-01.009.MC.P01.01.01.dpcranalysis.xlsx	A-579-01.251.NSPRP.02Aug2023.09	0.075	159432	396300	0.40230		
A-579-01.009.MC.P01.01.01.dpcranalysis.xlsx	A-579-01.251.NUDLD.02Aug2023.06	0.0744	47739	394935	0.12088		
A-579-01.009.MC.P01.01.01.dpcranalysis.xlsx	A-579-01.251.NUDLP.02Aug2023.03	0.0336	73972	435792	0.16974		
A-579-01.009.MC.P01.01.01.dpcranalysis.xlsx	A-579-01.251.NUDRD.02Aug2023.11	0.075	27073	349421	0.07748		
A-579-01.009.MC.P01.01.01.dpcranalysis.xlsx	A-579-01.251.NUDRP.02Aug2023.08	0.0393	24198	356341	0.06791		
A-579-01.010.MC.P01.01.01.dpcranalysis.xlsx	A-579-01.251.NUPLD.02Aug2023.04	0.0156	110026	397235	0.27698		
A-579-01.009.MC.P01.01.01.dpcranalysis.xlsx	A-579-01.251.NUPLP.02Aug2023.01	0.0279	39871	326951	0.12195		
A-579-01.010.MC.P01.01.01.dpcranalysis.xlsx	A-579-01.251.NUPRD.02Aug2023.11	0.0186	118724	352735	0.33658		
A-579-01.010.MC.P01.01.01.dpcranalysis.xlsx	A-579-01.251.NUPRP.02Aug2023.09	0.033	161057	366842	0.43903		
A-579-01.009.MC.P01.01.01.dpcranalysis.xlsx	A-579-01.251.NVOT.02Aug2023.09	0.075	207947	342060	0.60792		
A-579-01.010.MC.P01.01.01.dpcranalysis.xlsx	A-579-01.251.OVAS.02Aug2023.03	0.075	16148	366956	0.04401		
A-579-01.072.MC.P01.03.02.dpcranalysis.xlsx	A-579-01.251.SCCV.02Aug2023.01	0.0038	70841842	400474	176.89512	>ULOQ	
A-579-01.048.MC.P01.02.01.dpcranalysis.xlsx	A-579-01.251.SCLU.02Aug2023.03	0.0015	14502662	313133	46.31465		
A-579-01.048.MC.P01.02.01.dpcranalysis.xlsx	A-579-01.251.SCSA.02Aug2023.02	0.019	17134182	330818	51.79345	>ULOQ	
A-579-01.041.MC.P01.02.01.dpcranalysis.xlsx	A-579-01.251.SCSR.02Aug2023.02	0.075	4821609	371342	12.98427	>ULOQ	
A-579-01.048.MC.P01.02.01.dpcranalysis.xlsx	A-579-01.251.SCTH.02Aug2023.03	0.002	170507633	130923	1302.34718	>ULOQ	

IND: indeterminate, CV% >20 (only applicable to results above the lower limit of quantitation[LLOQ])
BQL: below quantitation limit (below LLOQ)
>ULOQ: above upper limit of quantitation
LLOQ = 14.9 copies/µL
ULOQ = 16800 copies/µL

Source.Name	Sample Name	DNA Input (ug)	AAV2-ITR.Target Copy Num Per ug DNA	B2M.Target Copy Num Per ug DNA	AAV2-ITR/B2M ratio	AAV2-ITR.Comment	AAV2-ITR.CV Flag
A-579-01.010.MC.P01.01.01.dpcranalysis.xlsx	A-579-01.251.SPLN.02Aug2023.02	0.075	176444	151231	1.16671		
A-579-01.042.MC.P01.02.02.dpcranalysis.xlsx	A-579-01.252.BRAN.02Aug2023.01	0.075	610437	415613	1.46876		
A-579-01.073.MC.P01.03.01.dpcranalysis.xlsx	A-579-01.252.DRGC.02Aug2023.02	0.00372	72594265	338358	214.54842	>ULOQ	
A-579-01.011.MC.P01.01.03.dpcranalysis.xlsx	A-579-01.252.DRGL.02Aug2023.02	0.0018	24928167	412378	60.44983		
A-579-01.073.MC.P01.03.01.dpcranalysis.xlsx	A-579-01.252.DRGT.02Aug2023.01	0.0038	21973000	340986	64.43960		
A-579-01.012.MC.P01.01.01.dpcranalysis.xlsx	A-579-01.252.HTHT.02Aug2023.01	0.0408	222593	332431	0.66959		
A-579-01.012.MC.P01.01.01.dpcranalysis.xlsx	A-579-01.252.KDNY.02Aug2023.02	0.075	33245	302089	0.11005		
A-579-01.012.MC.P01.01.01.dpcranalysis.xlsx	A-579-01.252.LUNG.02Aug2023.02	0.075	59074	509706	0.11590		
A-579-01.049.MC.P01.02.01.dpcranalysis.xlsx	A-579-01.252.LVER.02Aug2023.01	0.008	7503183	316167	23.73173		
A-579-01.011.MC.P01.01.01.dpcranalysis.xlsx	A-579-01.252.MUAL.02Aug2023.02	0.0738	18794	415846	0.04519		
A-579-01.011.MC.P01.01.03.dpcranalysis.xlsx	A-579-01.252.MUAR.02Aug2023.03	0.0753	211450	469679	0.45020		
A-579-01.011.MC.P01.01.01.dpcranalysis.xlsx	A-579-01.252.MUBL.02Aug2023.01	0.0399	26087	433146	0.06023		
A-579-01.011.MC.P01.01.01.dpcranalysis.xlsx	A-579-01.252.MUBR.02Aug2023.02	0.0399	28835	373298	0.07724		
A-579-01.012.MC.P01.01.01.dpcranalysis.xlsx	A-579-01.252.NFDLD.02Aug2023.05	0.0018	224389	142372	1.57607		
A-579-01.012.MC.P01.01.02.dpcranalysis.xlsx	A-579-01.252.NFDLP.02Aug2023.03	0.0111	621360	342559	1.81388		
A-579-01.042.MC.P01.02.02.dpcranalysis.xlsx	A-579-01.252.NFDRD.02Aug2023.10	0.075	68357	389396	0.17555		
A-579-01.042.MC.P01.02.01.dpcranalysis.xlsx	A-579-01.252.NFDRP.02Aug2023.09	0.0603	72299	300199	0.24084		
A-579-01.042.MC.P01.02.01.dpcranalysis.xlsx	A-579-01.252.NFPLD.02Aug2023.06	0.0603	70627	352020	0.20063		
A-579-01.042.MC.P01.02.01.dpcranalysis.xlsx	A-579-01.252.NFPLP.02Aug2023.02	0.075	26015	356795	0.07291		
A-579-01.042.MC.P01.02.01.dpcranalysis.xlsx	A-579-01.252.NFPRD.02Aug2023.10	0.0519	36326	379061	0.09583		
A-579-01.012.MC.P01.01.01.dpcranalysis.xlsx	A-579-01.252.NFPRP.02Aug2023.07	0.0564	41512	341014	0.12173		
A-579-01.042.MC.P01.02.01.dpcranalysis.xlsx	A-579-01.252.NMDLD.02Aug2023.05	0.075	12956	293396	0.04416		
A-579-01.042.MC.P01.02.01.dpcranalysis.xlsx	A-579-01.252.NMDLP.02Aug2023.02	0.075	16358	396785	0.04123		
A-579-01.011.MC.P01.01.01.dpcranalysis.xlsx	A-579-01.252.NMDRD.02Aug2023.12	0.0627	35718	418556	0.08534		
A-579-01.011.MC.P01.01.03.dpcranalysis.xlsx	A-579-01.252.NMDRP.02Aug2023.07	0.0705	22718	374843	0.06061		
A-579-01.011.MC.P01.01.01.dpcranalysis.xlsx	A-579-01.252.NMPLD.02Aug2023.05	0.0744	11911	386857	0.03079		
A-579-01.011.MC.P01.01.03.dpcranalysis.xlsx	A-579-01.252.NMPLP.02Aug2023.01	0.0624	45377	67083	0.67642		
A-579-01.011.MC.P01.01.01.dpcranalysis.xlsx	A-579-01.252.NMPRD.02Aug2023.10	0.0303	65919	433908	0.15192		
A-579-01.011.MC.P01.01.01.dpcranalysis.xlsx	A-579-01.252.NMPRP.02Aug2023.07	0.0201	52842	327967	0.16112		
A-579-01.011.MC.P01.01.01.dpcranalysis.xlsx	A-579-01.252.NSDLD.02Aug2023.05	0.0231	55646	329960	0.16865		
A-579-01.042.MC.P01.02.01.dpcranalysis.xlsx	A-579-01.252.NSDLP.02Aug2023.02	0.075	151200	378703	0.39926		
A-579-01.042.MC.P01.02.01.dpcranalysis.xlsx	A-579-01.252.NSDRD.02Aug2023.10	0.075	87117	361244	0.24116		
A-579-01.011.MC.P01.01.01.dpcranalysis.xlsx	A-579-01.252.NSDRP.02Aug2023.07	0.0015	111907	115942	0.96520	BQL	
A-579-01.045.MC.P01.02.01.dpcranalysis.xlsx	A-579-01.252.NSPLD.02Aug2023.05	0.075	145700	364261	0.39999		
A-579-01.011.MC.P01.01.01.dpcranalysis.xlsx	A-579-01.252.NSPLP.02Aug2023.02	0.075	106630	465677	0.22898		
A-579-01.011.MC.P01.01.01.dpcranalysis.xlsx	A-579-01.252.NSPRD.02Aug2023.11	0.0156	309944	290440	1.06715		
A-579-01.011.MC.P01.01.01.dpcranalysis.xlsx	A-579-01.252.NSPRP.02Aug2023.09	0.0021	14007	94689	0.14792	BQL	
A-579-01.042.MC.P01.02.01.dpcranalysis.xlsx	A-579-01.252.NUDLD.02Aug2023.04	0.075	15863	439208	0.03612		
A-579-01.011.MC.P01.01.03.dpcranalysis.xlsx	A-579-01.252.NUDLP.02Aug2023.01	0.0066	90109	246580	0.36544		

IND: indeterminate, CV% >20 (only applicable to results above the lower limit of quantitation[LLOQ])
BQL: below quantitation limit (below LLOQ)
>ULOQ: above upper limit of quantitation
LLOQ = 14.9 copies/µL
ULOQ = 16800 copies/µL

Source.Name	Sample Name	DNA Input (ug)	AAV2-ITR.Target Copy Num Per ug DNA	B2M.Target Copy Num Per ug DNA	AAV2-ITR/B2M ratio	AAV2-ITR.Comment	AAV2-ITR.CV Flag
A-579-01.011.MC.P01.01.01.dpcranalysis.xlsx	A-579-01.252.NUDRD.02Aug2023.11	0.0102	80404	343366	0.23416		
A-579-01.042.MC.P01.02.01.dpcranalysis.xlsx	A-579-01.252.NUDRP.02Aug2023.07	0.075	55048	339310	0.16224	IND	x
A-579-01.012.MC.P01.01.01.dpcranalysis.xlsx	A-579-01.252.NUPLD.02Aug2023.05	0.0462	194465	434929	0.44712		
A-579-01.011.MC.P01.01.01.dpcranalysis.xlsx	A-579-01.252.NUPLP.02Aug2023.01	0.0009	6056	145071	0.04175	BQL	
A-579-01.012.MC.P01.01.01.dpcranalysis.xlsx	A-579-01.252.NUPRD.02Aug2023.11	0.0636	13291	424182	0.03133		
A-579-01.012.MC.P01.01.01.dpcranalysis.xlsx	A-579-01.252.NUPRP.02Aug2023.07	0.0411	28831	403672	0.07142		
A-579-01.011.MC.P01.01.01.dpcranalysis.xlsx	A-579-01.252.NVOT.02Aug2023.09	0.075	331259	261159	1.26842		
A-579-01.042.MC.P01.02.01.dpcranalysis.xlsx	A-579-01.252.OVAS.02Aug2023.01	0.075	13034	356011	0.03661		
A-579-01.049.MC.P01.02.01.dpcranalysis.xlsx	A-579-01.252.SCCV.02Aug2023.01	0.002	30020667	335207	89.55868		
A-579-01.049.MC.P01.02.01.dpcranalysis.xlsx	A-579-01.252.SCLU.02Aug2023.01	0.019	21495932	375421	57.25819	>ULOQ	
A-579-01.073.MC.P01.03.01.dpcranalysis.xlsx	A-579-01.252.SCSA.02Aug2023.02	0.007	51760200	355133	145.74864		
A-579-01.061.MC.P01.02.01.dpcranalysis.xlsx	A-579-01.252.SCSR.02Aug2023.02	0.0198	8381428	370764	22.60581		
A-579-01.073.MC.P01.03.01.dpcranalysis.xlsx	A-579-01.252.SCTH.02Aug2023.01	0.0038	41148947	323916	127.03594		
A-579-01.012.MC.P01.01.01.dpcranalysis.xlsx	A-579-01.252.SPLN.02Aug2023.02	0.075	246935	307322	0.80351		
A-579-01.043.MC.P01.02.02.dpcranalysis.xlsx	A-579-01.253.BRAN.13Sep2023.02	0.0216	2407	414361	0.00581	IND	x
A-579-01.029.MC.P01.01.01.dpcranalysis.xlsx	A-579-01.253.DRGC.13Sep2023.02	0.075	139851	294896	0.47424		
A-579-01.029.MC.P01.01.02.dpcranalysis.xlsx	A-579-01.253.DRGL.13Sep2023.03	0.075	513641	320208	1.60409		
A-579-01.029.MC.P01.01.01.dpcranalysis.xlsx	A-579-01.253.DRGT.13Sep2023.02	0.075	169014	329429	0.51305		
A-579-01.030.MC.P01.01.02.dpcranalysis.xlsx	A-579-01.253.HTHT.13Sep2023.02	0.0444	45090	277619	0.16242		
A-579-01.067.MC.P01.02.01.dpcranalysis.xlsx	A-579-01.253.KDNY.13Sep2023.02	0.075	644	334457	0.00193	BQL	
A-579-01.030.MC.P01.01.01.dpcranalysis.xlsx	A-579-01.253.LUNG.13Sep2023.03	0.075	25660	315205	0.08141		
A-579-01.067.MC.P01.02.01.dpcranalysis.xlsx	A-579-01.253.LVER.13Sep2023.01	0.075	4537	342564	0.01324		
A-579-01.029.MC.P01.01.01.dpcranalysis.xlsx	A-579-01.253.MUAL.13Sep2023.02	0.0726	4507	284551	0.01584		
A-579-01.067.MC.P01.02.01.dpcranalysis.xlsx	A-579-01.253.MUAR.13Sep2023.02	0.075	1448	337388	0.00429	BQL	
A-579-01.043.MC.P01.02.02.dpcranalysis.xlsx	A-579-01.253.MUBL.13Sep2023.02	0.0405	465	364553	0.00128	BQL	
A-579-01.043.MC.P01.02.01.dpcranalysis.xlsx	A-579-01.253.MUBR.13Sep2023.02	0.0438	2747	351161	0.00782	BQL	
A-579-01.043.MC.P01.02.02.dpcranalysis.xlsx	A-579-01.253.NFDLD.13Sep2023.05	0.027	5585	368381	0.01516	IND	x
A-579-01.043.MC.P01.02.02.dpcranalysis.xlsx	A-579-01.253.NFDLP.13Sep2023.01	0.009	2036	329544	0.00618	IND	x
A-579-01.030.MC.P01.01.01.dpcranalysis.xlsx	A-579-01.253.NFDRD.13Sep2023.10	0.0729	3354	349437	0.00960		
A-579-01.043.MC.P01.02.02.dpcranalysis.xlsx	A-579-01.253.NFDRP.13Sep2023.07	0.0372	505	325970	0.00155	BQL	
A-579-01.030.MC.P01.01.02.dpcranalysis.xlsx	A-579-01.253.NFPLD.13Sep2023.05	0.075	2320	278992	0.00832	BQL	
A-579-01.030.MC.P01.01.01.dpcranalysis.xlsx	A-579-01.253.NFPLP.13Sep2023.02	0.0657	3413	222466	0.01534		
A-579-01.043.MC.P01.02.02.dpcranalysis.xlsx	A-579-01.253.NFPRD.13Sep2023.10	0.0447	424	344175	0.00123	BQL	
A-579-01.043.MC.P01.02.02.dpcranalysis.xlsx	A-579-01.253.NFPRP.13Sep2023.08	0.0189	429	270407	0.00159	IND	x
A-579-01.043.MC.P01.02.01.dpcranalysis.xlsx	A-579-01.253.NMDLD.13Sep2023.06	0.0645	521	339755	0.00153	BQL	
A-579-01.043.MC.P01.02.02.dpcranalysis.xlsx	A-579-01.253.NMDLP.13Sep2023.03	0.0708	1069	460784	0.00232	BQL	
A-579-01.043.MC.P01.02.02.dpcranalysis.xlsx	A-579-01.253.NMDRD.13Sep2023.11	0.075	472	268940	0.00176	BQL	
A-579-01.029.MC.P01.01.02.dpcranalysis.xlsx	A-579-01.253.NMDRP.13Sep2023.09	0.075	3210	304929	0.01053		
A-579-01.043.MC.P01.02.02.dpcranalysis.xlsx	A-579-01.253.NMPLD.13Sep2023.06	0.075	1686	324231	0.00520	BQL	

IND: indeterminate, CV% >20 (only applicable to results above the lower limit of quantitation[LLOQ])
BQL: below quantitation limit (below LLOQ)
>ULOQ: above upper limit of quantitation
LLOQ = 14.9 copies/µL
ULOQ = 16800 copies/µL

Source.Name	Sample Name	DNA Input (ug)	AAV2-ITR.Target Copy Num Per ug DNA	B2M.Target Copy Num Per ug DNA	AAV2-ITR/B2M ratio	AAV2-ITR.Comment	AAV2-ITR.CV Flag
A-579-01.029.MC.P01.01.01.dpcranalysis.xlsx	A-579-01.253.NMPLP.13Sep2023.01	0.0228	9845	278383	0.03537		
A-579-01.043.MC.P01.02.01.dpcranalysis.xlsx	A-579-01.253.NMPRD.13Sep2023.10	0.0558	4996	419665	0.01191		
A-579-01.043.MC.P01.02.02.dpcranalysis.xlsx	A-579-01.253.NMPRP.13Sep2023.08	0.0474	1982	367692	0.00539	BQL	
A-579-01.029.MC.P01.01.01.dpcranalysis.xlsx	A-579-01.253.NSDLD.13Sep2023.06	0.075	665	205308	0.00324	BQL	
A-579-01.043.MC.P01.02.02.dpcranalysis.xlsx	A-579-01.253.NSDLP.13Sep2023.02	0.0705	925	372499	0.00248	BQL	
A-579-01.029.MC.P01.01.02.dpcranalysis.xlsx	A-579-01.253.NSDRD.13Sep2023.11	0.0663	5651	273359	0.02067		
A-579-01.029.MC.P01.01.01.dpcranalysis.xlsx	A-579-01.253.NSDRP.13Sep2023.09	0.075	2144	263872	0.00813	BQL	
A-579-01.043.MC.P01.02.02.dpcranalysis.xlsx	A-579-01.253.NSPLD.13Sep2023.06	0.0693	1148	348717	0.00329	BQL	
A-579-01.043.MC.P01.02.01.dpcranalysis.xlsx	A-579-01.253.NSPLP.13Sep2023.01	0.075	4226	268246	0.01575		
A-579-01.043.MC.P01.02.01.dpcranalysis.xlsx	A-579-01.253.NSPRD.13Sep2023.10	0.075	1062	270567	0.00392	BQL	
A-579-01.029.MC.P01.01.01.dpcranalysis.xlsx	A-579-01.253.NSPRP.13Sep2023.08	0.075	2004	342415	0.00585	BQL	
A-579-01.043.MC.P01.02.01.dpcranalysis.xlsx	A-579-01.253.NUDLD.13Sep2023.05	0.0384	527	328672	0.00160	BQL	
A-579-01.043.MC.P01.02.02.dpcranalysis.xlsx	A-579-01.253.NUDLP.13Sep2023.03	0.0465	413	330310	0.00125	IND	x
A-579-01.043.MC.P01.02.02.dpcranalysis.xlsx	A-579-01.253.NUDRD.13Sep2023.10	0.0369	2134	432255	0.00494	BQL	
A-579-01.029.MC.P01.01.01.dpcranalysis.xlsx	A-579-01.253.NUDRP.13Sep2023.09	0.075	1853	300639	0.00616	BQL	
A-579-01.043.MC.P01.02.02.dpcranalysis.xlsx	A-579-01.253.NUPLD.13Sep2023.04	0.0636	265	412659	0.00064	BQL	
A-579-01.043.MC.P01.02.02.dpcranalysis.xlsx	A-579-01.253.NUPLP.13Sep2023.02	0.033	4858	373736	0.01300	BQL	
A-579-01.043.MC.P01.02.02.dpcranalysis.xlsx	A-579-01.253.NUPRD.13Sep2023.12	0.0534	2024	342594	0.00591	BQL	
A-579-01.030.MC.P01.01.02.dpcranalysis.xlsx	A-579-01.253.NUPRP.13Sep2023.08	0.0405	23857	370706	0.06436		
A-579-01.029.MC.P01.01.01.dpcranalysis.xlsx	A-579-01.253.NVOT.13Sep2023.07	0.075	73845	347057	0.21278		
A-579-01.067.MC.P01.02.01.dpcranalysis.xlsx	A-579-01.253.OVAS.13Sep2023.01	0.075	441579	376077	1.17417		
A-579-01.030.MC.P01.01.01.dpcranalysis.xlsx	A-579-01.253.SCCV.13Sep2023.01	0.0669	135850	333461	0.40739		
A-579-01.030.MC.P01.01.01.dpcranalysis.xlsx	A-579-01.253.SCLU.13Sep2023.01	0.0447	67839	364689	0.18602		
A-579-01.030.MC.P01.01.01.dpcranalysis.xlsx	A-579-01.253.SCSA.13Sep2023.03	0.0489	256018	306931	0.83412		
A-579-01.030.MC.P01.01.01.dpcranalysis.xlsx	A-579-01.253.SCSR.13Sep2023.03	0.0144	652653	345463	1.88921		
A-579-01.030.MC.P01.01.01.dpcranalysis.xlsx	A-579-01.253.SCTH.13Sep2023.03	0.0117	104645	264564	0.39554		
A-579-01.030.MC.P01.01.01.dpcranalysis.xlsx	A-579-01.253.SPLN.13Sep2023.02	0.075	112865	289122	0.39037		
A-579-01.031.MC.P01.01.01.dpcranalysis.xlsx	A-579-01.254.BRAN.13Sep2023.03	0.0321	85657	359043	0.23857		
A-579-01.031.MC.P01.01.02.dpcranalysis.xlsx	A-579-01.254.DRGC.13Sep2023.02	0.0399	715456	311421	2.29739		
A-579-01.056.MC.P01.02.01.dpcranalysis.xlsx	A-579-01.254.DRGL.13Sep2023.01	0.00375	2520373	335116	7.52089		
A-579-01.056.MC.P01.02.01.dpcranalysis.xlsx	A-579-01.254.DRGT.13Sep2023.02	0.00375	1845387	329952	5.59289		
A-579-01.032.MC.P01.01.01.dpcranalysis.xlsx	A-579-01.254.HTHT.13Sep2023.02	0.075	407885	387128	1.05362		
A-579-01.032.MC.P01.01.02.dpcranalysis.xlsx	A-579-01.254.KDNY.13Sep2023.01	0.075	48813	236469	0.20643		
A-579-01.032.MC.P01.01.01.dpcranalysis.xlsx	A-579-01.254.LUNG.13Sep2023.03	0.075	52030	334575	0.15551		
A-579-01.056.MC.P01.02.01.dpcranalysis.xlsx	A-579-01.254.LVER.13Sep2023.02	0.00375	8000782	289072	27.67747		
A-579-01.068.MC.P01.02.01.dpcranalysis.xlsx	A-579-01.254.MUAL.13Sep2023.03	0.027	71988	411358	0.17500		
A-579-01.068.MC.P01.02.01.dpcranalysis.xlsx	A-579-01.254.MUAR.13Sep2023.02	0.0312	65917	299983	0.21973		
A-579-01.031.MC.P01.01.02.dpcranalysis.xlsx	A-579-01.254.MUBL.13Sep2023.01	0.0219	103913	399479	0.26012		
A-579-01.031.MC.P01.01.01.dpcranalysis.xlsx	A-579-01.254.MUBR.13Sep2023.03	0.0441	29674	340106	0.08725		

IND: indeterminate, CV% >20 (only applicable to results above the lower limit of quantitation[LLOQ])
BQL: below quantitation limit (below LLOQ)
>ULOQ: above upper limit of quantitation
LLOQ = 14.9 copies/μL
ULOQ = 16800 copies/μL

Source.Name	Sample Name	DNA Input (ug)	AAV2-ITR.Target Copy Num Per ug DNA	B2M.Target Copy Num Per ug DNA	AAV2-ITR/B2M ratio	AAV2-ITR.Comment	AAV2-ITR.CV Flag
A-579-01.032.MC.P01.01.02.dpcranalysis.xlsx	A-579-01.254.NFDLD.13Sep2023.06	0.0561	64098	457246	0.14018		
A-579-01.032.MC.P01.01.01.dpcranalysis.xlsx	A-579-01.254.NFDLP.13Sep2023.03	0.0576	158821	380666	0.41722		
A-579-01.032.MC.P01.01.01.dpcranalysis.xlsx	A-579-01.254.NFDRD.13Sep2023.10	0.075	41527	401470	0.10344		
A-579-01.032.MC.P01.01.01.dpcranalysis.xlsx	A-579-01.254.NFDRP.13Sep2023.09	0.063	41815	315904	0.13237		
A-579-01.032.MC.P01.01.02.dpcranalysis.xlsx	A-579-01.254.NFPLD.13Sep2023.06	0.075	44557	352772	0.12631		
A-579-01.032.MC.P01.01.01.dpcranalysis.xlsx	A-579-01.254.NFPLP.13Sep2023.03	0.0708	70435	310696	0.22670		
A-579-01.032.MC.P01.01.01.dpcranalysis.xlsx	A-579-01.254.NFPRD.13Sep2023.10	0.0702	25660	412269	0.06224		
A-579-01.032.MC.P01.01.01.dpcranalysis.xlsx	A-579-01.254.NFPRP.13Sep2023.09	0.075	15306	332858	0.04598		
A-579-01.031.MC.P01.01.02.dpcranalysis.xlsx	A-579-01.254.NMDLD.13Sep2023.06	0.0447	132984	417839	0.31827		
A-579-01.031.MC.P01.01.01.dpcranalysis.xlsx	A-579-01.254.NMDLP.13Sep2023.03	0.0399	100795	356866	0.28245		
A-579-01.031.MC.P01.01.01.dpcranalysis.xlsx	A-579-01.254.NMDRD.13Sep2023.10	0.0573	66384	285725	0.23233		
A-579-01.031.MC.P01.01.01.dpcranalysis.xlsx	A-579-01.254.NMDRP.13Sep2023.07	0.051	164724	348152	0.47314		
A-579-01.031.MC.P01.01.02.dpcranalysis.xlsx	A-579-01.254.NMPLD.13Sep2023.05	0.0267	26136	280891	0.09305		
A-579-01.031.MC.P01.01.01.dpcranalysis.xlsx	A-579-01.254.NMPLP.13Sep2023.01	0.039	330568	342031	0.96648		
A-579-01.031.MC.P01.01.01.dpcranalysis.xlsx	A-579-01.254.NMPRD.13Sep2023.10	0.0456	93026	404680	0.22988		
A-579-01.031.MC.P01.01.02.dpcranalysis.xlsx	A-579-01.254.NMPRP.13Sep2023.08	0.075	22101	373520	0.05917		
A-579-01.031.MC.P01.01.02.dpcranalysis.xlsx	A-579-01.254.NSDLD.13Sep2023.06	0.0627	63592	307029	0.20712		
A-579-01.031.MC.P01.01.02.dpcranalysis.xlsx	A-579-01.254.NSDLP.13Sep2023.01	0.075	38313	354135	0.10819		
A-579-01.031.MC.P01.01.01.dpcranalysis.xlsx	A-579-01.254.NSDRD.13Sep2023.11	0.0669	131978	404570	0.32622		
A-579-01.031.MC.P01.01.02.dpcranalysis.xlsx	A-579-01.254.NSDRP.13Sep2023.08	0.0168	213458	373125	0.57208		
A-579-01.031.MC.P01.01.01.dpcranalysis.xlsx	A-579-01.254.NSPLD.13Sep2023.06	0.066	70283	299063	0.23501		
A-579-01.031.MC.P01.01.01.dpcranalysis.xlsx	A-579-01.254.NSPLP.13Sep2023.03	0.075	69141	296999	0.23280		
A-579-01.031.MC.P01.01.01.dpcranalysis.xlsx	A-579-01.254.NSPRD.13Sep2023.11	0.015	242231	322778	0.75046		
A-579-01.056.MC.P01.02.01.dpcranalysis.xlsx	A-579-01.254.NSPRP.13Sep2023.07	0.00375	158580	345296	0.45926		
A-579-01.031.MC.P01.01.02.dpcranalysis.xlsx	A-579-01.254.NUDLD.13Sep2023.04	0.075	35495	276117	0.12855		
A-579-01.031.MC.P01.01.02.dpcranalysis.xlsx	A-579-01.254.NUDLP.13Sep2023.03	0.075	57605	303669	0.18970		
A-579-01.031.MC.P01.01.01.dpcranalysis.xlsx	A-579-01.254.NUDRD.13Sep2023.11	0.075	96395	335726	0.28712		
A-579-01.031.MC.P01.01.01.dpcranalysis.xlsx	A-579-01.254.NUDRP.13Sep2023.07	0.075	45895	352576	0.13017		
A-579-01.032.MC.P01.01.01.dpcranalysis.xlsx	A-579-01.254.NUPLD.13Sep2023.04	0.0213	37734	425609	0.08866		
A-579-01.031.MC.P01.01.01.dpcranalysis.xlsx	A-579-01.254.NUPLP.13Sep2023.01	0.075	16206	291436	0.05561		
A-579-01.032.MC.P01.01.01.dpcranalysis.xlsx	A-579-01.254.NUPRD.13Sep2023.11	0.075	12184	298331	0.04084		
A-579-01.032.MC.P01.01.01.dpcranalysis.xlsx	A-579-01.254.NUPRP.13Sep2023.09	0.0747	15622	360926	0.04328		
A-579-01.031.MC.P01.01.02.dpcranalysis.xlsx	A-579-01.254.NVOT.13Sep2023.08	0.0471	1217658	356643	3.41422		
A-579-01.032.MC.P01.01.02.dpcranalysis.xlsx	A-579-01.254.OVAS.13Sep2023.02	0.075	37324	294112	0.12690		
A-579-01.032.MC.P01.01.01.dpcranalysis.xlsx	A-579-01.254.SCCV.13Sep2023.01	0.0339	950761	357668	2.65822		
A-579-01.032.MC.P01.01.01.dpcranalysis.xlsx	A-579-01.254.SCLU.13Sep2023.03	0.075	402715	359408	1.12049		
A-579-01.032.MC.P01.01.01.dpcranalysis.xlsx	A-579-01.254.SCSA.13Sep2023.01	0.075	609261	399121	1.52651		
A-579-01.032.MC.P01.01.01.dpcranalysis.xlsx	A-579-01.254.SCSR.13Sep2023.01	0.075	765081	375461	2.03771		
A-579-01.032.MC.P01.01.01.dpcranalysis.xlsx	A-579-01.254.SCTH.13Sep2023.02	0.0654	1213141	322871	3.75736		

IND: indeterminate, CV% >20 (only applicable to results above the lower limit of quantitation[LLOQ])
BQL: below quantitation limit (below LLOQ)
>ULOQ: above upper limit of quantitation
LLOQ = 14.9 copies/µL
ULOQ = 16800 copies/µL

Source.Name	Sample Name	DNA Input (ug)	AAV2-ITR.Target Copy Num Per ug DNA	B2M.Target Copy Num Per ug DNA	AAV2-ITR/B2M ratio	AAV2-ITR.Comment	AAV2-ITR.CV Flag
A-579-01.056.MC.P01.02.01.dpcranalysis.xlsx	A-579-01.254.SPLN.13Sep2023.01	0.00375	6622187	369252	17.93408		
A-579-01.032.MC.P01.01.01.dpcranalysis.xlsx	A-579-01.254.TGL1.13Sep2023.01	0.075	55129	351120	0.15701		
A-579-01.013.MC.P01.01.01.dpcranalysis.xlsx	A-579-01.301.BRAN.03Aug2023.02	0.075	888278	483087	1.83875		
A-579-01.013.MC.P01.01.01.dpcranalysis.xlsx	A-579-01.301.DRGC.03Aug2023.02	0.0387	5797791	439300	13.19780		
A-579-01.013.MC.P01.01.01.dpcranalysis.xlsx	A-579-01.301.DRGL.03Aug2023.03	0.075	1628492	477549	3.41010		
A-579-01.013.MC.P01.01.01.dpcranalysis.xlsx	A-579-01.301.DRGT.03Aug2023.01	0.0453	2075297	491432	4.22296		
A-579-01.014.MC.P01.01.01.dpcranalysis.xlsx	A-579-01.301.HTHT.03Aug2023.02	0.0615	532873	395308	1.34799		
A-579-01.014.MC.P01.01.01.dpcranalysis.xlsx	A-579-01.301.KDNY.03Aug2023.02	0.075	154852	315828	0.49031		
A-579-01.014.MC.P01.01.02.dpcranalysis.xlsx	A-579-01.301.LUNG.03Aug2023.02	0.075	168607	359557	0.46893		
A-579-01.074.MC.P01.03.01.dpcranalysis.xlsx	A-579-01.301.LVER.03Aug2023.02	0.0038	28392123	279718	101.50283		
A-579-01.013.MC.P01.01.01.dpcranalysis.xlsx	A-579-01.301.MUAL.03Aug2023.03	0.0297	92343	537327	0.17186		
A-579-01.013.MC.P01.01.03.dpcranalysis.xlsx	A-579-01.301.MUAR.03Aug2023.02	0.0711	108505	530622	0.20449		
A-579-01.013.MC.P01.01.03.dpcranalysis.xlsx	A-579-01.301.MUBL.03Aug2023.01	0.0144	255451	642688	0.39747		
A-579-01.013.MC.P01.01.03.dpcranalysis.xlsx	A-579-01.301.MUBR.03Aug2023.01	0.075	74228	401352	0.18494		
A-579-01.014.MC.P01.01.02.dpcranalysis.xlsx	A-579-01.301.NFDLD.03Aug2023.04	0.0687	334329	354218	0.94385		
A-579-01.014.MC.P01.01.01.dpcranalysis.xlsx	A-579-01.301.NFDLP.03Aug2023.02	0.0393	390658	412899	0.94613		
A-579-01.014.MC.P01.01.01.dpcranalysis.xlsx	A-579-01.301.NFDRD.03Aug2023.11	0.075	230067	398508	0.57732		
A-579-01.014.MC.P01.01.01.dpcranalysis.xlsx	A-579-01.301.NFDRP.03Aug2023.07	0.0585	277431	365723	0.75858		
A-579-01.014.MC.P01.01.02.dpcranalysis.xlsx	A-579-01.301.NFPLD.03Aug2023.04	0.075	132720	262621	0.50537		
A-579-01.014.MC.P01.01.01.dpcranalysis.xlsx	A-579-01.301.NFPLP.03Aug2023.03	0.075	120518	415091	0.29034		
A-579-01.014.MC.P01.01.01.dpcranalysis.xlsx	A-579-01.301.NFPRD.03Aug2023.11	0.0435	218915	347500	0.62997		
A-579-01.014.MC.P01.01.01.dpcranalysis.xlsx	A-579-01.301.NFPRP.03Aug2023.09	0.039	145564	382583	0.38048		
A-579-01.013.MC.P01.01.01.dpcranalysis.xlsx	A-579-01.301.NMDLD.03Aug2023.05	0.021	351689	556178	0.63233		
A-579-01.013.MC.P01.01.01.dpcranalysis.xlsx	A-579-01.301.NMDLP.03Aug2023.03	0.018	391507	584215	0.67014		
A-579-01.013.MC.P01.01.01.dpcranalysis.xlsx	A-579-01.301.NMDRD.03Aug2023.10	0.0597	127446	460796	0.27658		
A-579-01.013.MC.P01.01.01.dpcranalysis.xlsx	A-579-01.301.NMDRP.03Aug2023.09	0.075	126616	324875	0.38974		
A-579-01.062.MC.P01.02.01.dpcranalysis.xlsx	A-579-01.301.NMPLD.03Aug2023.05	0.075	157354	300372	0.52386		
A-579-01.013.MC.P01.01.01.dpcranalysis.xlsx	A-579-01.301.NMPLP.03Aug2023.03	0.0594	86781	402300	0.21571		
A-579-01.013.MC.P01.01.01.dpcranalysis.xlsx	A-579-01.301.NMPRD.03Aug2023.12	0.0417	416027	361448	1.15100		
A-579-01.013.MC.P01.01.01.dpcranalysis.xlsx	A-579-01.301.NMPRP.03Aug2023.07	0.0639	94830	388765	0.24393		
A-579-01.013.MC.P01.01.03.dpcranalysis.xlsx	A-579-01.301.NSDLD.03Aug2023.04	0.075	150323	328141	0.45810		
A-579-01.013.MC.P01.01.01.dpcranalysis.xlsx	A-579-01.301.NSDLP.03Aug2023.01	0.051	323107	337858	0.95634		
A-579-01.013.MC.P01.01.01.dpcranalysis.xlsx	A-579-01.301.NSDRD.03Aug2023.10	0.0666	278416	361407	0.77037		
A-579-01.013.MC.P01.01.03.dpcranalysis.xlsx	A-579-01.301.NSDRP.03Aug2023.07	0.075	210803	327591	0.64349		
A-579-01.013.MC.P01.01.01.dpcranalysis.xlsx	A-579-01.301.NSPLD.03Aug2023.04	0.075	206516	371056	0.55656		
A-579-01.013.MC.P01.01.01.dpcranalysis.xlsx	A-579-01.301.NSPLP.03Aug2023.03	0.066	373439	393421	0.94921		
A-579-01.013.MC.P01.01.01.dpcranalysis.xlsx	A-579-01.301.NSPRD.03Aug2023.11	0.0276	653807	323336	2.02207		
A-579-01.013.MC.P01.01.01.dpcranalysis.xlsx	A-579-01.301.NSPRP.03Aug2023.09	0.0657	559041	385913	1.44862		
A-579-01.013.MC.P01.01.01.dpcranalysis.xlsx	A-579-01.301.NUDLD.03Aug2023.04	0.0462	204374	366939	0.55697		

IND: indeterminate, CV% >20 (only applicable to results above the lower limit of quantitation[LLOQ])
BQL: below quantitation limit (below LLOQ)
>ULOQ: above upper limit of quantitation
LLOQ = 14.9 copies/µL
ULOQ = 16800 copies/µL

Source.Name	Sample Name	DNA Input (ug)	AAV2-ITR.Target Copy Num Per ug DNA	B2M.Target Copy Num Per ug DNA	AAV2-ITR/B2M ratio	AAV2-ITR.Comment	AAV2-ITR.CV Flag
A-579-01.013.MC.P01.01.01.dpcranalysis.xlsx	A-579-01.301.NUDLP.03Aug2023.03	0.0441	556423	372190	1.49500		
A-579-01.013.MC.P01.01.01.dpcranalysis.xlsx	A-579-01.301.NUDRD.03Aug2023.10	0.0348	285310	353963	0.80605		
A-579-01.013.MC.P01.01.03.dpcranalysis.xlsx	A-579-01.301.NUDRP.03Aug2023.09	0.0648	255003	380117	0.67085		
A-579-01.014.MC.P01.01.02.dpcranalysis.xlsx	A-579-01.301.NUPLD.03Aug2023.04	0.075	313712	346640	0.90501		
A-579-01.013.MC.P01.01.03.dpcranalysis.xlsx	A-579-01.301.NUPLP.03Aug2023.03	0.0672	108365	282719	0.38329		
A-579-01.014.MC.P01.01.01.dpcranalysis.xlsx	A-579-01.301.NUPRD.03Aug2023.11	0.0702	553718	409045	1.35369		
A-579-01.014.MC.P01.01.02.dpcranalysis.xlsx	A-579-01.301.NUPRP.03Aug2023.08	0.0078	468013	229376	2.04038		
A-579-01.013.MC.P01.01.01.dpcranalysis.xlsx	A-579-01.301.NVOT.03Aug2023.09	0.0447	1383254	406846	3.39995		
A-579-01.014.MC.P01.01.01.dpcranalysis.xlsx	A-579-01.301.SCCV.03Aug2023.02	0.0543	3306020	339180	9.74710		
A-579-01.014.MC.P01.01.02.dpcranalysis.xlsx	A-579-01.301.SCLU.03Aug2023.02	0.0558	2292161	354177	6.47179		
A-579-01.014.MC.P01.01.01.dpcranalysis.xlsx	A-579-01.301.SCSA.03Aug2023.03	0.075	1427303	296271	4.81756		
A-579-01.014.MC.P01.01.01.dpcranalysis.xlsx	A-579-01.301.SCSR.03Aug2023.01	0.0504	3988819	317222	12.57421		
A-579-01.062.MC.P01.02.01.dpcranalysis.xlsx	A-579-01.301.SCTH.03Aug2023.01	0.0237	4897401	298883	16.38566		
A-579-01.014.MC.P01.01.01.dpcranalysis.xlsx	A-579-01.301.SPLN.03Aug2023.03	0.075	1576556	570671	2.76263		
A-579-01.014.MC.P01.01.01.dpcranalysis.xlsx	A-579-01.301.TEST.03Aug2023.03	0.075	21925	851250	0.02576		
A-579-01.015.MC.P01.01.01.dpcranalysis.xlsx	A-579-01.302.BRAN.03Aug2023.01	0.0336	259264	429556	0.60356		
A-579-01.015.MC.P01.01.01.dpcranalysis.xlsx	A-579-01.302.DRGC.03Aug2023.03	0.0384	1134741	393094	2.88669		
A-579-01.015.MC.P01.01.02.dpcranalysis.xlsx	A-579-01.302.DRGL.03Aug2023.02	0.075	830284	426384	1.94727		
A-579-01.015.MC.P01.01.02.dpcranalysis.xlsx	A-579-01.302.DRGT.03Aug2023.02	0.075	1468824	428615	3.42691		
A-579-01.016.MC.P01.01.01.dpcranalysis.xlsx	A-579-01.302.HTHT.03Aug2023.03	0.0456	681118	300703	2.26509		
A-579-01.016.MC.P01.01.01.dpcranalysis.xlsx	A-579-01.302.KDNY.03Aug2023.01	0.075	60169	280442	0.21455		
A-579-01.063.MC.P01.02.01.dpcranalysis.xlsx	A-579-01.302.LUNG.03Aug2023.01	0.075	362513	219035	1.65505		
A-579-01.075.MC.P01.03.01.dpcranalysis.xlsx	A-579-01.302.LVER.03Aug2023.02	0.0038	36251404	340421	106.48990		
A-579-01.015.MC.P01.01.01.dpcranalysis.xlsx	A-579-01.302.MUAL.03Aug2023.01	0.0726	92684	440781	0.21027		
A-579-01.015.MC.P01.01.01.dpcranalysis.xlsx	A-579-01.302.MUAR.03Aug2023.02	0.075	28597	403418	0.07089		
A-579-01.015.MC.P01.01.02.dpcranalysis.xlsx	A-579-01.302.MUBL.03Aug2023.01	0.0735	78105	452771	0.17250		
A-579-01.015.MC.P01.01.01.dpcranalysis.xlsx	A-579-01.302.MUBR.03Aug2023.03	0.0603	53106	439216	0.12091		
A-579-01.016.MC.P01.01.01.dpcranalysis.xlsx	A-579-01.302.NFDLD.03Aug2023.06	0.0588	132143	384278	0.34387		
A-579-01.016.MC.P01.01.01.dpcranalysis.xlsx	A-579-01.302.NFDLP.03Aug2023.01	0.045	231601	380053	0.60939		
A-579-01.016.MC.P01.01.01.dpcranalysis.xlsx	A-579-01.302.NFDRD.03Aug2023.10	0.0345	196487	347295	0.56576		
A-579-01.016.MC.P01.01.01.dpcranalysis.xlsx	A-579-01.302.NFDRP.03Aug2023.07	0.0099	112782	282121	0.39977		
A-579-01.016.MC.P01.01.01.dpcranalysis.xlsx	A-579-01.302.NFPLD.03Aug2023.05	0.0483	148821	381807	0.38978		
A-579-01.016.MC.P01.01.01.dpcranalysis.xlsx	A-579-01.302.NFPLP.03Aug2023.03	0.0267	166200	314292	0.52881		
A-579-01.016.MC.P01.01.01.dpcranalysis.xlsx	A-579-01.302.NFPRD.03Aug2023.12	0.0186	275183	382140	0.72011		
A-579-01.016.MC.P01.01.01.dpcranalysis.xlsx	A-579-01.302.NFPRP.03Aug2023.07	0.0516	163424	404074	0.40444		
A-579-01.015.MC.P01.01.01.dpcranalysis.xlsx	A-579-01.302.NMDLD.03Aug2023.05	0.0198	152939	414909	0.36861		
A-579-01.015.MC.P01.01.01.dpcranalysis.xlsx	A-579-01.302.NMDLP.03Aug2023.03	0.0288	130035	272577	0.47706		
A-579-01.015.MC.P01.01.01.dpcranalysis.xlsx	A-579-01.302.NMDRD.03Aug2023.12	0.0363	117837	500979	0.23521		
A-579-01.015.MC.P01.01.01.dpcranalysis.xlsx	A-579-01.302.NMDRP.03Aug2023.08	0.0615	106719	485485	0.21982		

IND: indeterminate, CV% >20 (only applicable to results above the lower limit of quantitation[LLOQ])
BQL: below quantitation limit (below LLOQ)
>ULOQ: above upper limit of quantitation
LLOQ = 14.9 copies/µL
ULOQ = 16800 copies/µL

Source.Name	Sample Name	DNA Input (ug)	AAV2-ITR.Target Copy Num Per ug DNA	B2M.Target Copy Num Per ug DNA	AAV2-ITR/B2M ratio	AAV2-ITR.Comment	AAV2-ITR.CV Flag
A-579-01.015.MC.P01.01.01.dpcranalysis.xlsx	A-579-01.302.NMPLD.03Aug2023.06	0.075	69894	321148	0.21764		
A-579-01.015.MC.P01.01.02.dpcranalysis.xlsx	A-579-01.302.NMPLP.03Aug2023.02	0.069	22562	480677	0.04694		
A-579-01.015.MC.P01.01.01.dpcranalysis.xlsx	A-579-01.302.NMPRD.03Aug2023.12	0.0474	98788	454459	0.21737		
A-579-01.015.MC.P01.01.01.dpcranalysis.xlsx	A-579-01.302.NMPRP.03Aug2023.09	0.0678	22414	502479	0.04461		
A-579-01.015.MC.P01.01.01.dpcranalysis.xlsx	A-579-01.302.NSDLD.03Aug2023.04	0.0699	126901	395866	0.32057		
A-579-01.015.MC.P01.01.01.dpcranalysis.xlsx	A-579-01.302.NSDLP.03Aug2023.02	0.0423	203557	369715	0.55058		
A-579-01.015.MC.P01.01.02.dpcranalysis.xlsx	A-579-01.302.NSDRD.03Aug2023.10	0.075	85661	379540	0.22570		
A-579-01.015.MC.P01.01.01.dpcranalysis.xlsx	A-579-01.302.NSDRP.03Aug2023.07	0.075	78332	428985	0.18260		
A-579-01.015.MC.P01.01.02.dpcranalysis.xlsx	A-579-01.302.NSPLD.03Aug2023.06	0.075	84084	460077	0.18276		
A-579-01.015.MC.P01.01.01.dpcranalysis.xlsx	A-579-01.302.NSPLP.03Aug2023.02	0.075	99450	396119	0.25106		
A-579-01.015.MC.P01.01.01.dpcranalysis.xlsx	A-579-01.302.NSPRD.03Aug2023.10	0.0651	98530	452724	0.21764		
A-579-01.015.MC.P01.01.02.dpcranalysis.xlsx	A-579-01.302.NSPRP.03Aug2023.09	0.075	133532	318528	0.41922		
A-579-01.015.MC.P01.01.01.dpcranalysis.xlsx	A-579-01.302.NUDLD.03Aug2023.05	0.0309	104419	360375	0.28975		
A-579-01.015.MC.P01.01.01.dpcranalysis.xlsx	A-579-01.302.NUDLP.03Aug2023.01	0.0375	99468	331483	0.30007		
A-579-01.015.MC.P01.01.01.dpcranalysis.xlsx	A-579-01.302.NUDRD.03Aug2023.12	0.0291	61533	371184	0.16577		
A-579-01.015.MC.P01.01.02.dpcranalysis.xlsx	A-579-01.302.NUDRP.03Aug2023.08	0.0519	54368	318453	0.17073		
A-579-01.016.MC.P01.01.02.dpcranalysis.xlsx	A-579-01.302.NUPLD.03Aug2023.06	0.0234	92406	371718	0.24859		
A-579-01.015.MC.P01.01.01.dpcranalysis.xlsx	A-579-01.302.NUPLP.03Aug2023.02	0.0354	26061	381111	0.06838		
A-579-01.016.MC.P01.01.01.dpcranalysis.xlsx	A-579-01.302.NUPRD.03Aug2023.11	0.0408	144815	380665	0.38043		
A-579-01.016.MC.P01.01.01.dpcranalysis.xlsx	A-579-01.302.NUPRP.03Aug2023.07	0.0435	121258	372443	0.32558		
A-579-01.015.MC.P01.01.01.dpcranalysis.xlsx	A-579-01.302.NVOT.03Aug2023.09	0.0132	939273	428414	2.19244		
A-579-01.016.MC.P01.01.01.dpcranalysis.xlsx	A-579-01.302.SCCV.03Aug2023.03	0.0579	334307	177454	1.88391		
A-579-01.016.MC.P01.01.01.dpcranalysis.xlsx	A-579-01.302.SCLU.03Aug2023.01	0.0201	945406	467317	2.02305		
A-579-01.016.MC.P01.01.01.dpcranalysis.xlsx	A-579-01.302.SCSA.03Aug2023.02	0.0702	558371	346357	1.61213		
A-579-01.016.MC.P01.01.01.dpcranalysis.xlsx	A-579-01.302.SCSR.03Aug2023.03	0.015	717204	415800	1.72488		
A-579-01.016.MC.P01.01.01.dpcranalysis.xlsx	A-579-01.302.SCTH.03Aug2023.03	0.0597	712413	372012	1.91503		
A-579-01.016.MC.P01.01.01.dpcranalysis.xlsx	A-579-01.302.SPLN.03Aug2023.03	0.075	1539471	450570	3.41672		
A-579-01.016.MC.P01.01.01.dpcranalysis.xlsx	A-579-01.302.TEST.03Aug2023.01	0.075	45771	304478	0.15032		
A-579-01.033.MC.P01.01.01.dpcranalysis.xlsx	A-579-01.303.BRAN.14Sep2023.02	0.075	11665	338252	0.03449		
A-579-01.057.MC.P01.02.02.dpcranalysis.xlsx	A-579-01.303.DRGC.14Sep2023.03	0.00375	2136027	239363	8.92381		
A-579-01.057.MC.P01.02.01.dpcranalysis.xlsx	A-579-01.303.DRGL.14Sep2023.02	0.00375	3298649	372139	8.86403		
A-579-01.057.MC.P01.02.01.dpcranalysis.xlsx	A-579-01.303.DRGT.14Sep2023.01	0.00375	2259787	282912	7.98760		
A-579-01.034.MC.P01.01.01.dpcranalysis.xlsx	A-579-01.303.HTHT.14Sep2023.02	0.075	119541	423074	0.28255		
A-579-01.034.MC.P01.01.01.dpcranalysis.xlsx	A-579-01.303.KDNY.14Sep2023.03	0.075	23004	254323	0.09045		
A-579-01.034.MC.P01.01.01.dpcranalysis.xlsx	A-579-01.303.LUNG.14Sep2023.03	0.075	111676	252187	0.44283		
A-579-01.057.MC.P01.02.01.dpcranalysis.xlsx	A-579-01.303.LVER.14Sep2023.03	0.00375	3802773	94167	40.38324		
A-579-01.069.MC.P01.02.01.dpcranalysis.xlsx	A-579-01.303.MUAL.14Sep2023.03	0.0576	24827	447036	0.05554		
A-579-01.033.MC.P01.01.01.dpcranalysis.xlsx	A-579-01.303.MUAR.14Sep2023.01	0.0558	41573	447958	0.09281		
A-579-01.033.MC.P01.01.01.dpcranalysis.xlsx	A-579-01.303.MUBL.14Sep2023.02	0.075	13118	411376	0.03189		

IND: indeterminate, CV% >20 (only applicable to results above the lower limit of quantitation[LLOQ])
BQL: below quantitation limit (below LLOQ)
>ULOQ: above upper limit of quantitation
LLOQ = 14.9 copies/μL
ULOQ = 16800 copies/μL

Source.Name	Sample Name	DNA Input (ug)	AAV2-ITR.Target Copy Num Per ug DNA	B2M.Target Copy Num Per ug DNA	AAV2-ITR/B2M ratio	AAV2-ITR.Comment	AAV2-ITR.CV Flag
A-579-01.069.MC.P01.02.01.dpcranalysis.xlsx	A-579-01.303.MUBR.14Sep2023.03	0.075	25940	395976	0.06551		
A-579-01.034.MC.P01.01.01.dpcranalysis.xlsx	A-579-01.303.NFDLD.14Sep2023.04	0.0639	53437	339820	0.15725		
A-579-01.034.MC.P01.01.02.dpcranalysis.xlsx	A-579-01.303.NFDLP.14Sep2023.01	0.075	54973	377393	0.14567		
A-579-01.034.MC.P01.01.01.dpcranalysis.xlsx	A-579-01.303.NFDRD.14Sep2023.10	0.075	22437	272626	0.08230		
A-579-01.034.MC.P01.01.01.dpcranalysis.xlsx	A-579-01.303.NFDRP.14Sep2023.08	0.0468	30596	339311	0.09017		
A-579-01.034.MC.P01.01.01.dpcranalysis.xlsx	A-579-01.303.NFPLD.14Sep2023.04	0.0729	63925	248044	0.25772		
A-579-01.034.MC.P01.01.02.dpcranalysis.xlsx	A-579-01.303.NFPLP.14Sep2023.02	0.075	308439	395500	0.77987		
A-579-01.034.MC.P01.01.01.dpcranalysis.xlsx	A-579-01.303.NFPRD.14Sep2023.11	0.0198	13420	332865	0.04032		
A-579-01.034.MC.P01.01.02.dpcranalysis.xlsx	A-579-01.303.NFPRP.14Sep2023.09	0.075	5061	329280	0.01537		
A-579-01.033.MC.P01.01.01.dpcranalysis.xlsx	A-579-01.303.NMDLD.14Sep2023.05	0.0648	160373	384028	0.41761		
A-579-01.033.MC.P01.01.02.dpcranalysis.xlsx	A-579-01.303.NMDLP.14Sep2023.03	0.0552	62670	391848	0.15994		
A-579-01.033.MC.P01.01.01.dpcranalysis.xlsx	A-579-01.303.NMDRD.14Sep2023.12	0.0567	107877	352971	0.30562		
A-579-01.033.MC.P01.01.01.dpcranalysis.xlsx	A-579-01.303.NMDRP.14Sep2023.09	0.0348	137613	424291	0.32434		
A-579-01.033.MC.P01.01.01.dpcranalysis.xlsx	A-579-01.303.NMPLD.14Sep2023.06	0.0471	92085	397766	0.23151		
A-579-01.033.MC.P01.01.02.dpcranalysis.xlsx	A-579-01.303.NMPLP.14Sep2023.01	0.0432	22167	325630	0.06807		
A-579-01.033.MC.P01.01.01.dpcranalysis.xlsx	A-579-01.303.NMPRD.14Sep2023.12	0.0117	275932	642205	0.42966		
A-579-01.033.MC.P01.01.01.dpcranalysis.xlsx	A-579-01.303.NMPRP.14Sep2023.08	0.0504	20110	356176	0.05646		
A-579-01.033.MC.P01.01.02.dpcranalysis.xlsx	A-579-01.303.NSDLD.14Sep2023.05	0.0249	19482	417076	0.04671		
A-579-01.069.MC.P01.02.01.dpcranalysis.xlsx	A-579-01.303.NSDLP.14Sep2023.03	0.0606	47244	382151	0.12363		
A-579-01.033.MC.P01.01.01.dpcranalysis.xlsx	A-579-01.303.NSDRD.14Sep2023.11	0.0513	80379	440804	0.18235		
A-579-01.033.MC.P01.01.01.dpcranalysis.xlsx	A-579-01.303.NSDRP.14Sep2023.07	0.0615	32932	388676	0.08473		
A-579-01.033.MC.P01.01.01.dpcranalysis.xlsx	A-579-01.303.NSPLD.14Sep2023.04	0.0648	15821	386815	0.04090		
A-579-01.033.MC.P01.01.01.dpcranalysis.xlsx	A-579-01.303.NSPLP.14Sep2023.02	0.0435	18801	413359	0.04548		
A-579-01.033.MC.P01.01.01.dpcranalysis.xlsx	A-579-01.303.NSPRD.14Sep2023.10	0.009	169898	711667	0.23873		
A-579-01.069.MC.P01.02.01.dpcranalysis.xlsx	A-579-01.303.NSPRP.14Sep2023.09	0.075	43748	399523	0.10950		
A-579-01.033.MC.P01.01.02.dpcranalysis.xlsx	A-579-01.303.NUDLD.14Sep2023.05	0.0645	61047	331236	0.18430		
A-579-01.033.MC.P01.01.01.dpcranalysis.xlsx	A-579-01.303.NUDLP.14Sep2023.03	0.0684	42635	308587	0.13816		
A-579-01.033.MC.P01.01.01.dpcranalysis.xlsx	A-579-01.303.NUDRD.14Sep2023.10	0.075	43954	207598	0.21173		
A-579-01.033.MC.P01.01.01.dpcranalysis.xlsx	A-579-01.303.NUDRP.14Sep2023.09	0.0594	56864	373420	0.15228		
A-579-01.045.MC.P01.02.01.dpcranalysis.xlsx	A-579-01.303.NUPLD.14Sep2023.06	0.075	44271	396343	0.11170		
A-579-01.033.MC.P01.01.01.dpcranalysis.xlsx	A-579-01.303.NUPLP.14Sep2023.01	0.075	17691	402254	0.04398		
A-579-01.034.MC.P01.01.01.dpcranalysis.xlsx	A-579-01.303.NUPRD.14Sep2023.10	0.0483	70792	484870	0.14600		
A-579-01.034.MC.P01.01.01.dpcranalysis.xlsx	A-579-01.303.NUPRP.14Sep2023.07	0.0681	21680	383071	0.05659		
A-579-01.033.MC.P01.01.01.dpcranalysis.xlsx	A-579-01.303.NVOT.14Sep2023.09	0.0552	253691	388052	0.65375		
A-579-01.034.MC.P01.01.01.dpcranalysis.xlsx	A-579-01.303.SCCV.14Sep2023.03	0.075	84448	382256	0.22092		
A-579-01.045.MC.P01.02.01.dpcranalysis.xlsx	A-579-01.303.SCLU.14Sep2023.02	0.0582	12709	387975	0.03276		
A-579-01.034.MC.P01.01.01.dpcranalysis.xlsx	A-579-01.303.SCSA.14Sep2023.03	0.075	109013	395876	0.27537		
A-579-01.034.MC.P01.01.01.dpcranalysis.xlsx	A-579-01.303.SCSR.14Sep2023.03	0.075	23732	374074	0.06344		
A-579-01.034.MC.P01.01.01.dpcranalysis.xlsx	A-579-01.303.SCTH.14Sep2023.02	0.0285	127981	123396	1.03715		

IND: indeterminate, CV% >20 (only applicable to results above the lower limit of quantitation[LLOQ])
BQL: below quantitation limit (below LLOQ)
>ULOQ: above upper limit of quantitation
LLOQ = 14.9 copies/µL
ULOQ = 16800 copies/µL

Source.Name	Sample Name	DNA Input (ug)	AAV2-ITR.Target Copy Num Per ug DNA	B2M.Target Copy Num Per ug DNA	AAV2-ITR/B2M ratio	AAV2-ITR.Comment	AAV2-ITR.CV Flag
A-579-01.044.MC.P01.02.01.dpcranalysis.xlsx	A-579-01.303.SPLN.14Sep2023.03	0.075	2175538	202073	10.76610		
A-579-01.034.MC.P01.01.01.dpcranalysis.xlsx	A-579-01.303.TEST.14Sep2023.03	0.075	37097	411364	0.09018		
A-579-01.035.MC.P01.01.01.dpcranalysis.xlsx	A-579-01.304.BRAN.14Sep2023.03	0.0468	70728	339709	0.20820		
A-579-01.035.MC.P01.01.02.dpcranalysis.xlsx	A-579-01.304.DRGC.14Sep2023.01	0.0636	479819	322881	1.48606		
A-579-01.058.MC.P01.02.01.dpcranalysis.xlsx	A-579-01.304.DRGL.14Sep2023.02	0.00375	16906400	367285	46.03070		
A-579-01.035.MC.P01.01.01.dpcranalysis.xlsx	A-579-01.304.DRGT.14Sep2023.03	0.0507	342765	350414	0.97817		
A-579-01.036.MC.P01.01.01.dpcranalysis.xlsx	A-579-01.304.HTHT.14Sep2023.01	0.075	221978	376905	0.58895		
A-579-01.036.MC.P01.01.01.dpcranalysis.xlsx	A-579-01.304.KDNY.14Sep2023.01	0.075	26724	188826	0.14153		
A-579-01.036.MC.P01.01.01.dpcranalysis.xlsx	A-579-01.304.LUNG.14Sep2023.01	0.075	20767	302829	0.06858		
A-579-01.058.MC.P01.02.01.dpcranalysis.xlsx	A-579-01.304.LVER.14Sep2023.02	0.00375	8254649	316213	26.10468		
A-579-01.035.MC.P01.01.01.dpcranalysis.xlsx	A-579-01.304.MUAL.14Sep2023.03	0.0351	54883	365649	0.15010		
A-579-01.035.MC.P01.01.02.dpcranalysis.xlsx	A-579-01.304.MUAR.14Sep2023.01	0.075	32433	346603	0.09357		
A-579-01.035.MC.P01.01.02.dpcranalysis.xlsx	A-579-01.304.MUBL.14Sep2023.03	0.075	8846	267297	0.03309		
A-579-01.035.MC.P01.01.01.dpcranalysis.xlsx	A-579-01.304.MUBR.14Sep2023.03	0.0678	28695	359884	0.07973		
A-579-01.036.MC.P01.01.01.dpcranalysis.xlsx	A-579-01.304.NFDLD.14Sep2023.05	0.057	48222	401014	0.12025		
A-579-01.036.MC.P01.01.01.dpcranalysis.xlsx	A-579-01.304.NFDLP.14Sep2023.01	0.0591	11074	424714	0.02607		
A-579-01.036.MC.P01.01.01.dpcranalysis.xlsx	A-579-01.304.NFDRD.14Sep2023.10	0.075	7629	332192	0.02297		
A-579-01.036.MC.P01.01.01.dpcranalysis.xlsx	A-579-01.304.NFDRP.14Sep2023.08	0.0696	54786	370457	0.14789		
A-579-01.036.MC.P01.01.01.dpcranalysis.xlsx	A-579-01.304.NFPLD.14Sep2023.04	0.045	5289	285942	0.01850		
A-579-01.036.MC.P01.01.01.dpcranalysis.xlsx	A-579-01.304.NFPLP.14Sep2023.02	0.075	21498	367422	0.05851		
A-579-01.036.MC.P01.01.01.dpcranalysis.xlsx	A-579-01.304.NFPRD.14Sep2023.12	0.0594	24142	397625	0.06071		
A-579-01.036.MC.P01.01.02.dpcranalysis.xlsx	A-579-01.304.NFPRP.14Sep2023.08	0.0621	5785	337916	0.01712		
A-579-01.035.MC.P01.01.02.dpcranalysis.xlsx	A-579-01.304.NMDLD.14Sep2023.04	0.0429	50566	384625	0.13147		
A-579-01.035.MC.P01.01.01.dpcranalysis.xlsx	A-579-01.304.NMDLP.14Sep2023.03	0.0261	23233	337216	0.06890		
A-579-01.035.MC.P01.01.01.dpcranalysis.xlsx	A-579-01.304.NMDRD.14Sep2023.11	0.0657	21291	392398	0.05426		
A-579-01.035.MC.P01.01.01.dpcranalysis.xlsx	A-579-01.304.NMDRP.14Sep2023.09	0.0465	6160	390615	0.01577		
A-579-01.035.MC.P01.01.02.dpcranalysis.xlsx	A-579-01.304.NMPLD.14Sep2023.06	0.0636	20400	315892	0.06458		
A-579-01.035.MC.P01.01.02.dpcranalysis.xlsx	A-579-01.304.NMPLP.14Sep2023.03	0.0339	7365	378248	0.01947		
A-579-01.035.MC.P01.01.02.dpcranalysis.xlsx	A-579-01.304.NMPRD.14Sep2023.12	0.0408	13638	284392	0.04795		
A-579-01.035.MC.P01.01.01.dpcranalysis.xlsx	A-579-01.304.NMPRP.14Sep2023.07	0.075	10054	302649	0.03322		
A-579-01.035.MC.P01.01.01.dpcranalysis.xlsx	A-579-01.304.NSDLD.14Sep2023.05	0.0705	56655	293477	0.19305		
A-579-01.035.MC.P01.01.01.dpcranalysis.xlsx	A-579-01.304.NSDLP.14Sep2023.03	0.0429	86676	347552	0.24939		
A-579-01.035.MC.P01.01.01.dpcranalysis.xlsx	A-579-01.304.NSDRD.14Sep2023.11	0.075	27944	335073	0.08340		
A-579-01.035.MC.P01.01.02.dpcranalysis.xlsx	A-579-01.304.NSDRP.14Sep2023.09	0.0405	45388	363654	0.12481		
A-579-01.035.MC.P01.01.02.dpcranalysis.xlsx	A-579-01.304.NSPLD.14Sep2023.05	0.06	13600	295272	0.04606		
A-579-01.035.MC.P01.01.01.dpcranalysis.xlsx	A-579-01.304.NSPLP.14Sep2023.03	0.075	17960	361100	0.04974		
A-579-01.035.MC.P01.01.01.dpcranalysis.xlsx	A-579-01.304.NSPRD.14Sep2023.11	0.0297	87771	377733	0.23236		
A-579-01.035.MC.P01.01.02.dpcranalysis.xlsx	A-579-01.304.NSPRP.14Sep2023.07	0.0267	40005	311251	0.12853		
A-579-01.035.MC.P01.01.01.dpcranalysis.xlsx	A-579-01.304.NUDLD.14Sep2023.05	0.075	53125	298654	0.17788		

IND: indeterminate, CV% >20 (only applicable to results above the lower limit of quantitation[LLOQ])
BQL: below quantitation limit (below LLOQ)
>ULOQ: above upper limit of quantitation
LLOQ = 14.9 copies/µL
ULOQ = 16800 copies/µL

Source.Name	Sample Name	DNA Input (ug)	AAV2-ITR.Target Copy Num Per ug DNA	B2M.Target Copy Num Per ug DNA	AAV2-ITR/B2M ratio	AAV2-ITR.Comment	AAV2-ITR.CV Flag
A-579-01.035.MC.P01.01.01.dpcranalysis.xlsx	A-579-01.304.NUDLP.14Sep2023.03	0.048	13994	303421	0.04612		
A-579-01.035.MC.P01.01.01.dpcranalysis.xlsx	A-579-01.304.NUDRD.14Sep2023.12	0.075	30097	297951	0.10101		
A-579-01.035.MC.P01.01.01.dpcranalysis.xlsx	A-579-01.304.NUDRP.14Sep2023.09	0.069	9180	280859	0.03269		
A-579-01.036.MC.P01.01.01.dpcranalysis.xlsx	A-579-01.304.NUPLD.14Sep2023.04	0.0189	16173	390173	0.04145		
A-579-01.035.MC.P01.01.01.dpcranalysis.xlsx	A-579-01.304.NUPLP.14Sep2023.01	0.0621	4895	314155	0.01558		
A-579-01.036.MC.P01.01.02.dpcranalysis.xlsx	A-579-01.304.NUPRD.14Sep2023.11	0.075	71829	360705	0.19914		
A-579-01.036.MC.P01.01.01.dpcranalysis.xlsx	A-579-01.304.NUPRP.14Sep2023.07	0.051	9267	364037	0.02545		
A-579-01.035.MC.P01.01.01.dpcranalysis.xlsx	A-579-01.304.NVOT.14Sep2023.08	0.075	48204	367242	0.13126		
A-579-01.036.MC.P01.01.01.dpcranalysis.xlsx	A-579-01.304.SCCV.14Sep2023.03	0.0654	168014	383637	0.43795		
A-579-01.036.MC.P01.01.01.dpcranalysis.xlsx	A-579-01.304.SCLU.14Sep2023.01	0.0021	7476222	498044	15.01115		
A-579-01.036.MC.P01.01.01.dpcranalysis.xlsx	A-579-01.304.SCSA.14Sep2023.01	0.0303	2768057	374873	7.38398		
A-579-01.058.MC.P01.02.01.dpcranalysis.xlsx	A-579-01.304.SCSR.14Sep2023.02	0.00375	5465476	370471	14.75277		
A-579-01.058.MC.P01.02.01.dpcranalysis.xlsx	A-579-01.304.SCTH.14Sep2023.02	0.00375	17963307	372587	48.21242		
A-579-01.036.MC.P01.01.02.dpcranalysis.xlsx	A-579-01.304.SPLN.14Sep2023.02	0.075	1497011	226632	6.60547		
A-579-01.036.MC.P01.01.02.dpcranalysis.xlsx	A-579-01.304.TEST.14Sep2023.02	0.075	12020	313264	0.03837		
A-579-01.017.MC.P01.01.01.dpcranalysis.xlsx	A-579-01.351.BRAN.03Aug2023.03	0.0279	15514	357092	0.04344		
A-579-01.017.MC.P01.01.01.dpcranalysis.xlsx	A-579-01.351.DRGC.03Aug2023.03	0.075	637311	420921	1.51409		
A-579-01.052.MC.P01.02.01.dpcranalysis.xlsx	A-579-01.351.DRGL.03Aug2023.01	0.019	2594716	432207	6.00341		
A-579-01.076.MC.P01.03.01.dpcranalysis.xlsx	A-579-01.351.DRGT.03Aug2023.01	0.0038	59114018	332426	177.82593		
A-579-01.018.MC.P01.01.02.dpcranalysis.xlsx	A-579-01.351.HTHT.03Aug2023.02	0.075	236367	338016	0.69928		
A-579-01.018.MC.P01.01.02.dpcranalysis.xlsx	A-579-01.351.KDNY.03Aug2023.03	0.075	221051	441793	0.50035		
A-579-01.018.MC.P01.01.02.dpcranalysis.xlsx	A-579-01.351.LUNG.03Aug2023.01	0.075	220211	406448	0.54179		
A-579-01.076.MC.P01.03.01.dpcranalysis.xlsx	A-579-01.351.LVER.03Aug2023.01	0.0038	12525088	391263	32.01193		
A-579-01.017.MC.P01.01.01.dpcranalysis.xlsx	A-579-01.351.MUAL.03Aug2023.02	0.018	180548	506567	0.35642		
A-579-01.017.MC.P01.01.02.dpcranalysis.xlsx	A-579-01.351.MUAR.03Aug2023.02	0.0483	60246	397942	0.15139		
A-579-01.017.MC.P01.01.01.dpcranalysis.xlsx	A-579-01.351.MUBL.03Aug2023.03	0.0513	53007	400433	0.13237		
A-579-01.017.MC.P01.01.01.dpcranalysis.xlsx	A-579-01.351.MUBR.03Aug2023.02	0.0585	57683	421946	0.13671		
A-579-01.018.MC.P01.01.02.dpcranalysis.xlsx	A-579-01.351.NFDLD.03Aug2023.05	0.072	72168	361881	0.19943		
A-579-01.018.MC.P01.01.01.dpcranalysis.xlsx	A-579-01.351.NFDLP.03Aug2023.01	0.0237	43930	403224	0.10895		
A-579-01.018.MC.P01.01.01.dpcranalysis.xlsx	A-579-01.351.NFDRD.03Aug2023.10	0.0714	66902	388105	0.17238		
A-579-01.018.MC.P01.01.01.dpcranalysis.xlsx	A-579-01.351.NFDRP.03Aug2023.07	0.048	32968	397017	0.08304		
A-579-01.018.MC.P01.01.01.dpcranalysis.xlsx	A-579-01.351.NFPLD.03Aug2023.06	0.066	19711	386442	0.05101		
A-579-01.018.MC.P01.01.01.dpcranalysis.xlsx	A-579-01.351.NFPLP.03Aug2023.01	0.0687	22117	368062	0.06009		
A-579-01.018.MC.P01.01.01.dpcranalysis.xlsx	A-579-01.351.NFPRD.03Aug2023.12	0.0186	39762	454222	0.08754		
A-579-01.018.MC.P01.01.02.dpcranalysis.xlsx	A-579-01.351.NFPRP.03Aug2023.08	0.0513	42437	300290	0.14132		
A-579-01.017.MC.P01.01.01.dpcranalysis.xlsx	A-579-01.351.NMDLD.03Aug2023.06	0.0603	138824	360494	0.38509		
A-579-01.017.MC.P01.01.01.dpcranalysis.xlsx	A-579-01.351.NMDLP.03Aug2023.01	0.0474	78300	417903	0.18736		
A-579-01.017.MC.P01.01.01.dpcranalysis.xlsx	A-579-01.351.NMDRD.03Aug2023.10	0.0507	86007	337362	0.25494		
A-579-01.017.MC.P01.01.01.dpcranalysis.xlsx	A-579-01.351.NMDRP.03Aug2023.08	0.0396	108088	396125	0.27286		

IND: indeterminate, CV% >20 (only applicable to results above the lower limit of quantitation[LLOQ])
BQL: below quantitation limit (below LLOQ)
>ULOQ: above upper limit of quantitation
LLOQ = 14.9 copies/µL
ULOQ = 16800 copies/µL

Source.Name	Sample Name	DNA Input (ug)	AAV2-ITR.Target Copy Num Per ug DNA	B2M.Target Copy Num Per ug DNA	AAV2-ITR/B2M ratio	AAV2-ITR.Comment	AAV2-ITR.CV Flag
A-579-01.017.MC.P01.01.01.dpcranalysis.xlsx	A-579-01.351.NMPLD.03Aug2023.06	0.0672	76979	378431	0.20342		
A-579-01.017.MC.P01.01.02.dpcranalysis.xlsx	A-579-01.351.NMPLP.03Aug2023.03	0.0351	103923	403986	0.25724		
A-579-01.017.MC.P01.01.01.dpcranalysis.xlsx	A-579-01.351.NMPRD.03Aug2023.11	0.0573	84081	481677	0.17456		
A-579-01.017.MC.P01.01.02.dpcranalysis.xlsx	A-579-01.351.NMPRP.03Aug2023.08	0.075	45192	364709	0.12391		
A-579-01.017.MC.P01.01.01.dpcranalysis.xlsx	A-579-01.351.NSDLD.03Aug2023.04	0.075	87422	374453	0.23347		
A-579-01.017.MC.P01.01.01.dpcranalysis.xlsx	A-579-01.351.NSDLP.03Aug2023.02	0.048	308544	386021	0.79929		
A-579-01.017.MC.P01.01.01.dpcranalysis.xlsx	A-579-01.351.NSDRD.03Aug2023.11	0.0345	203994	488810	0.41733		
A-579-01.017.MC.P01.01.01.dpcranalysis.xlsx	A-579-01.351.NSDRP.03Aug2023.09	0.0288	364211	395014	0.92202		
A-579-01.017.MC.P01.01.01.dpcranalysis.xlsx	A-579-01.351.NSPLD.03Aug2023.06	0.075	147728	399429	0.36985		
A-579-01.017.MC.P01.01.02.dpcranalysis.xlsx	A-579-01.351.NSPLP.03Aug2023.01	0.075	322196	428437	0.75203		
A-579-01.017.MC.P01.01.01.dpcranalysis.xlsx	A-579-01.351.NSPRD.03Aug2023.10	0.0327	261776	420970	0.62184		
A-579-01.017.MC.P01.01.01.dpcranalysis.xlsx	A-579-01.351.NSPRP.03Aug2023.08	0.075	163333	439768	0.37141		
A-579-01.017.MC.P01.01.01.dpcranalysis.xlsx	A-579-01.351.NUDLD.03Aug2023.04	0.0501	64076	338049	0.18955		
A-579-01.017.MC.P01.01.02.dpcranalysis.xlsx	A-579-01.351.NUDLP.03Aug2023.01	0.0399	84035	455386	0.18454		
A-579-01.017.MC.P01.01.01.dpcranalysis.xlsx	A-579-01.351.NUDRD.03Aug2023.11	0.0456	46963	397066	0.11828		
A-579-01.017.MC.P01.01.02.dpcranalysis.xlsx	A-579-01.351.NUDRP.03Aug2023.08	0.0237	104793	399266	0.26246		
A-579-01.018.MC.P01.01.01.dpcranalysis.xlsx	A-579-01.351.NUPLD.03Aug2023.04	0.0426	149246	429498	0.34749		
A-579-01.017.MC.P01.01.01.dpcranalysis.xlsx	A-579-01.351.NUPLP.03Aug2023.01	0.075	81150	328552	0.24699		
A-579-01.018.MC.P01.01.01.dpcranalysis.xlsx	A-579-01.351.NUPRD.03Aug2023.10	0.0447	129383	323597	0.39983		
A-579-01.018.MC.P01.01.01.dpcranalysis.xlsx	A-579-01.351.NUPRP.03Aug2023.07	0.0441	39016	344360	0.11330		
A-579-01.017.MC.P01.01.01.dpcranalysis.xlsx	A-579-01.351.NVOT.03Aug2023.09	0.0063	28237	251704	0.11218	BQL	
A-579-01.064.MC.P01.02.01.dpcranalysis.xlsx	A-579-01.351.OVAS.03Aug2023.03	0.075	268893	266625	1.00851		
A-579-01.018.MC.P01.01.01.dpcranalysis.xlsx	A-579-01.351.SCCV.03Aug2023.03	0.057	140426	398640	0.35226		
A-579-01.018.MC.P01.01.01.dpcranalysis.xlsx	A-579-01.351.SCLU.03Aug2023.02	0.06	1932941	417317	4.63183		
A-579-01.018.MC.P01.01.01.dpcranalysis.xlsx	A-579-01.351.SCSA.03Aug2023.03	0.0408	2502500	370828	6.74840		
A-579-01.076.MC.P01.03.01.dpcranalysis.xlsx	A-579-01.351.SCSR.03Aug2023.02	0.0038	54128912	381033	142.05821		
A-579-01.052.MC.P01.02.01.dpcranalysis.xlsx	A-579-01.351.SCTH.03Aug2023.02	0.019	6717667	246081	27.29863		
A-579-01.052.MC.P01.02.01.dpcranalysis.xlsx	A-579-01.351.SPLN.03Aug2023.03	0.019	1652074	273589	6.03851		
A-579-01.019.MC.P01.01.01.dpcranalysis.xlsx	A-579-01.352.BRAN.03Aug2023.01	0.0489	91100	470436	0.19365		
A-579-01.019.MC.P01.01.01.dpcranalysis.xlsx	A-579-01.352.DRGC.03Aug2023.02	0.075	345893	424237	0.81533		
A-579-01.053.MC.P01.02.03.dpcranalysis.xlsx	A-579-01.352.DRGL.03Aug2023.03	0.019	5276674	283758	18.59569		
A-579-01.019.MC.P01.01.02.dpcranalysis.xlsx	A-579-01.352.DRGT.03Aug2023.02	0.0615	2581691	357911	7.21323		
A-579-01.020.MC.P01.01.01.dpcranalysis.xlsx	A-579-01.352.HTHT.03Aug2023.02	0.075	683542	390034	1.75252		
A-579-01.020.MC.P01.01.01.dpcranalysis.xlsx	A-579-01.352.KDNY.03Aug2023.02	0.075	228396	470307	0.48563		
A-579-01.020.MC.P01.01.02.dpcranalysis.xlsx	A-579-01.352.LUNG.03Aug2023.03	0.075	252168	493211	0.51128		
A-579-01.077.MC.P01.03.01.dpcranalysis.xlsx	A-579-01.352.LVER.03Aug2023.03	0.0038	24203544	361654	66.92451		
A-579-01.019.MC.P01.01.01.dpcranalysis.xlsx	A-579-01.352.MUAL.03Aug2023.02	0.0429	63941	375455	0.17030		
A-579-01.019.MC.P01.01.01.dpcranalysis.xlsx	A-579-01.352.MUAR.03Aug2023.02	0.0489	27674	377170	0.07337		
A-579-01.019.MC.P01.01.01.dpcranalysis.xlsx	A-579-01.352.MUBL.03Aug2023.01	0.048	40833	439639	0.09288		

IND: indeterminate, CV% >20 (only applicable to results above the lower limit of quantitation[LLOQ])
BQL: below quantitation limit (below LLOQ)
>ULOQ: above upper limit of quantitation
LLOQ = 14.9 copies/µL
ULOQ = 16800 copies/µL

Source.Name	Sample Name	DNA Input (ug)	AAV2-ITR.Target Copy Num Per ug DNA	B2M.Target Copy Num Per ug DNA	AAV2-ITR/B2M ratio	AAV2-ITR.Comment	AAV2-ITR.CV Flag
A-579-01.019.MC.P01.01.01.dpcranalysis.xlsx	A-579-01.352.MUBR.03Aug2023.03	0.033	43874	431059	0.10178		
A-579-01.020.MC.P01.01.01.dpcranalysis.xlsx	A-579-01.352.NFDLD.03Aug2023.06	0.0429	388660	402650	0.96526		
A-579-01.020.MC.P01.01.01.dpcranalysis.xlsx	A-579-01.352.NFDLP.03Aug2023.01	0.075	194084	412235	0.47081		
A-579-01.020.MC.P01.01.02.dpcranalysis.xlsx	A-579-01.352.NFDRD.03Aug2023.11	0.075	187171	389237	0.48087		
A-579-01.020.MC.P01.01.01.dpcranalysis.xlsx	A-579-01.352.NFDRP.03Aug2023.07	0.0447	133392	374127	0.35654		
A-579-01.020.MC.P01.01.01.dpcranalysis.xlsx	A-579-01.352.NFPLD.03Aug2023.06	0.075	77336	348413	0.22197		
A-579-01.020.MC.P01.01.01.dpcranalysis.xlsx	A-579-01.352.NFPLP.03Aug2023.02	0.075	43064	351120	0.12265		
A-579-01.020.MC.P01.01.01.dpcranalysis.xlsx	A-579-01.352.NFPRD.03Aug2023.12	0.0285	232580	330924	0.70282		
A-579-01.020.MC.P01.01.01.dpcranalysis.xlsx	A-579-01.352.NFPRP.03Aug2023.08	0.075	75868	367721	0.20632		
A-579-01.019.MC.P01.01.01.dpcranalysis.xlsx	A-579-01.352.NMDLD.03Aug2023.06	0.0459	130107	449240	0.28962		
A-579-01.019.MC.P01.01.01.dpcranalysis.xlsx	A-579-01.352.NMDLP.03Aug2023.03	0.0249	300166	378487	0.79307		
A-579-01.019.MC.P01.01.01.dpcranalysis.xlsx	A-579-01.352.NMDRD.03Aug2023.12	0.0627	78760	431514	0.18252		
A-579-01.019.MC.P01.01.02.dpcranalysis.xlsx	A-579-01.352.NMDRP.03Aug2023.09	0.0546	323705	483821	0.66906		
A-579-01.019.MC.P01.01.02.dpcranalysis.xlsx	A-579-01.352.NMPLD.03Aug2023.04	0.0492	147498	371171	0.39739		
A-579-01.019.MC.P01.01.01.dpcranalysis.xlsx	A-579-01.352.NMPLP.03Aug2023.01	0.0282	178492	450697	0.39603		
A-579-01.019.MC.P01.01.02.dpcranalysis.xlsx	A-579-01.352.NMPRD.03Aug2023.11	0.0216	426773	427713	0.99780		
A-579-01.019.MC.P01.01.02.dpcranalysis.xlsx	A-579-01.352.NMPRP.03Aug2023.09	0.0357	220137	306510	0.71821		
A-579-01.019.MC.P01.01.02.dpcranalysis.xlsx	A-579-01.352.NSDLD.03Aug2023.05	0.075	109415	347965	0.31444		
A-579-01.019.MC.P01.01.01.dpcranalysis.xlsx	A-579-01.352.NSDLP.03Aug2023.02	0.075	138986	382760	0.36311		
A-579-01.019.MC.P01.01.01.dpcranalysis.xlsx	A-579-01.352.NSDRD.03Aug2023.10	0.075	132720	331900	0.39988		
A-579-01.019.MC.P01.01.01.dpcranalysis.xlsx	A-579-01.352.NSDRP.03Aug2023.07	0.075	153365	389536	0.39371		
A-579-01.019.MC.P01.01.02.dpcranalysis.xlsx	A-579-01.352.NSPLD.03Aug2023.05	0.075	280271	376180	0.74504		
A-579-01.019.MC.P01.01.02.dpcranalysis.xlsx	A-579-01.352.NSPLP.03Aug2023.02	0.075	223347	330605	0.67557		
A-579-01.019.MC.P01.01.02.dpcranalysis.xlsx	A-579-01.352.NSPRD.03Aug2023.11	0.0462	85545	350500	0.24407		
A-579-01.019.MC.P01.01.01.dpcranalysis.xlsx	A-579-01.352.NSPRP.03Aug2023.08	0.075	200088	402929	0.49658		
A-579-01.019.MC.P01.01.01.dpcranalysis.xlsx	A-579-01.352.NUDLD.03Aug2023.06	0.0702	171716	449622	0.38191		
A-579-01.019.MC.P01.01.01.dpcranalysis.xlsx	A-579-01.352.NUDLP.03Aug2023.03	0.0525	225520	419964	0.53700		
A-579-01.019.MC.P01.01.01.dpcranalysis.xlsx	A-579-01.352.NUDRD.03Aug2023.12	0.0543	389138	424039	0.91769		
A-579-01.019.MC.P01.01.02.dpcranalysis.xlsx	A-579-01.352.NUDRP.03Aug2023.09	0.0582	119890	311741	0.38458		
A-579-01.020.MC.P01.01.01.dpcranalysis.xlsx	A-579-01.352.NUPLD.03Aug2023.04	0.0507	259713	470422	0.55209		
A-579-01.019.MC.P01.01.01.dpcranalysis.xlsx	A-579-01.352.NUPLP.03Aug2023.03	0.0723	45221	468894	0.09644		
A-579-01.020.MC.P01.01.01.dpcranalysis.xlsx	A-579-01.352.NUPRD.03Aug2023.12	0.0657	104798	395253	0.26514		
A-579-01.020.MC.P01.01.01.dpcranalysis.xlsx	A-579-01.352.NUPRP.03Aug2023.09	0.0585	218663	444634	0.49178		
A-579-01.019.MC.P01.01.01.dpcranalysis.xlsx	A-579-01.352.NVOT.03Aug2023.07	0.069	680028	401557	1.69348		
A-579-01.020.MC.P01.01.02.dpcranalysis.xlsx	A-579-01.352.OVAS.03Aug2023.02	0.075	21308	511327	0.04167		
A-579-01.020.MC.P01.01.02.dpcranalysis.xlsx	A-579-01.352.SCCV.03Aug2023.03	0.0744	269124	332726	0.80885		
A-579-01.020.MC.P01.01.01.dpcranalysis.xlsx	A-579-01.352.SCLU.03Aug2023.01	0.0651	1731412	260681	6.64188		
A-579-01.020.MC.P01.01.01.dpcranalysis.xlsx	A-579-01.352.SCSA.03Aug2023.03	0.075	902235	505232	1.78578		
A-579-01.020.MC.P01.01.01.dpcranalysis.xlsx	A-579-01.352.SCSR.03Aug2023.01	0.075	1270248	386792	3.28406		

IND: indeterminate, CV% >20 (only applicable to results above the lower limit of quantitation[LLOQ])
BQL: below quantitation limit (below LLOQ)
>ULOQ: above upper limit of quantitation
LLOQ = 14.9 copies/µL
ULOQ = 16800 copies/µL

Source.Name	Sample Name	DNA Input (ug)	AAV2-ITR.Target Copy Num Per ug DNA	B2M.Target Copy Num Per ug DNA	AAV2-ITR/B2M ratio	AAV2-ITR.Comment	AAV2-ITR.CV Flag
A-579-01.020.MC.P01.01.01.dpcranalysis.xlsx	A-579-01.352.SCTH.03Aug2023.03	0.0405	816644	342579	2.38381		
A-579-01.020.MC.P01.01.02.dpcranalysis.xlsx	A-579-01.352.SPLN.03Aug2023.03	0.075	1928416	546056	3.53154		
A-579-01.020.MC.P01.01.01.dpcranalysis.xlsx	A-579-01.352.TGL1.03Aug2023.01	0.075	42243	540556	0.07815		
A-579-01.037.MC.P01.01.01.dpcranalysis.xlsx	A-579-01.353.BRAN.14Sep2023.02	0.0549	16998	328095	0.05181		
A-579-01.037.MC.P01.01.01.dpcranalysis.xlsx	A-579-01.353.DRGC.14Sep2023.01	0.075	294989	274643	1.07408		
A-579-01.059.MC.P01.02.01.dpcranalysis.xlsx	A-579-01.353.DRGL.14Sep2023.03	0.00375	6356373	192204	33.07090		
A-579-01.037.MC.P01.01.01.dpcranalysis.xlsx	A-579-01.353.DRGT.14Sep2023.03	0.075	785014	279396	2.80968		
A-579-01.038.MC.P01.01.01.dpcranalysis.xlsx	A-579-01.353.HTHT.14Sep2023.02	0.0645	145434	349052	0.41665		
A-579-01.038.MC.P01.01.02.dpcranalysis.xlsx	A-579-01.353.KDNY.14Sep2023.01	0.075	62916	251804	0.24986		
A-579-01.038.MC.P01.01.01.dpcranalysis.xlsx	A-579-01.353.LUNG.14Sep2023.02	0.075	112044	367802	0.30463		
A-579-01.059.MC.P01.02.01.dpcranalysis.xlsx	A-579-01.353.LVER.14Sep2023.01	0.00375	4903982	141182	34.73513		
A-579-01.037.MC.P01.01.02.dpcranalysis.xlsx	A-579-01.353.MUAL.14Sep2023.03	0.075	34375	273625	0.12563		
A-579-01.037.MC.P01.01.01.dpcranalysis.xlsx	A-579-01.353.MUAR.14Sep2023.01	0.075	20160	275520	0.07317		
A-579-01.037.MC.P01.01.01.dpcranalysis.xlsx	A-579-01.353.MUBL.14Sep2023.03	0.075	16042	280772	0.05714		
A-579-01.037.MC.P01.01.02.dpcranalysis.xlsx	A-579-01.353.MUBR.14Sep2023.03	0.0522	9250	225824	0.04096		
A-579-01.038.MC.P01.01.01.dpcranalysis.xlsx	A-579-01.353.NFDLD.14Sep2023.04	0.075	82077	338035	0.24281		
A-579-01.038.MC.P01.01.01.dpcranalysis.xlsx	A-579-01.353.NFDLP.14Sep2023.02	0.075	11317	407599	0.02776		
A-579-01.038.MC.P01.01.01.dpcranalysis.xlsx	A-579-01.353.NFDRD.14Sep2023.11	0.0612	119076	368651	0.32301		
A-579-01.038.MC.P01.01.01.dpcranalysis.xlsx	A-579-01.353.NFDRP.14Sep2023.07	0.075	81748	332279	0.24602		
A-579-01.038.MC.P01.01.02.dpcranalysis.xlsx	A-579-01.353.NFPLD.14Sep2023.05	0.0591	85007	272325	0.31215		
A-579-01.038.MC.P01.01.01.dpcranalysis.xlsx	A-579-01.353.NFPLP.14Sep2023.03	0.075	550959	311323	1.76974		
A-579-01.038.MC.P01.01.02.dpcranalysis.xlsx	A-579-01.353.NFPRD.14Sep2023.12	0.0558	13469	404909	0.03327		
A-579-01.038.MC.P01.01.02.dpcranalysis.xlsx	A-579-01.353.NFPRP.14Sep2023.09	0.0543	79449	331050	0.23999		
A-579-01.037.MC.P01.01.01.dpcranalysis.xlsx	A-579-01.353.NMDLD.14Sep2023.04	0.0726	20510	339484	0.06042		
A-579-01.037.MC.P01.01.01.dpcranalysis.xlsx	A-579-01.353.NMDLP.14Sep2023.01	0.075	8816	341046	0.02585		
A-579-01.037.MC.P01.01.01.dpcranalysis.xlsx	A-579-01.353.NMDRD.14Sep2023.12	0.0504	30407	386204	0.07873		
A-579-01.037.MC.P01.01.01.dpcranalysis.xlsx	A-579-01.353.NMDRP.14Sep2023.08	0.0441	23546	221429	0.10634		
A-579-01.037.MC.P01.01.01.dpcranalysis.xlsx	A-579-01.353.NMPLD.14Sep2023.04	0.0336	48028	312500	0.15369		
A-579-01.037.MC.P01.01.01.dpcranalysis.xlsx	A-579-01.353.NMPLP.14Sep2023.02	0.0582	36916	354867	0.10403		
A-579-01.037.MC.P01.01.01.dpcranalysis.xlsx	A-579-01.353.NMPRD.14Sep2023.10	0.075	13039	404619	0.03223		
A-579-01.037.MC.P01.01.02.dpcranalysis.xlsx	A-579-01.353.NMPRP.14Sep2023.09	0.06	18785	296485	0.06336		
A-579-01.037.MC.P01.01.01.dpcranalysis.xlsx	A-579-01.353.NSDLD.14Sep2023.04	0.075	30445	345557	0.08811		
A-579-01.037.MC.P01.01.02.dpcranalysis.xlsx	A-579-01.353.NSDLP.14Sep2023.03	0.075	27524	291415	0.09445		
A-579-01.037.MC.P01.01.02.dpcranalysis.xlsx	A-579-01.353.NSDRD.14Sep2023.12	0.075	34739	233427	0.14882		
A-579-01.037.MC.P01.01.01.dpcranalysis.xlsx	A-579-01.353.NSDRP.14Sep2023.08	0.075	33189	297323	0.11163		
A-579-01.037.MC.P01.01.02.dpcranalysis.xlsx	A-579-01.353.NSPLD.14Sep2023.05	0.0444	43718	329095	0.13284		
A-579-01.037.MC.P01.01.01.dpcranalysis.xlsx	A-579-01.353.NSPLP.14Sep2023.02	0.0609	19212	332130	0.05785		
A-579-01.037.MC.P01.01.01.dpcranalysis.xlsx	A-579-01.353.NSPRD.14Sep2023.10	0.075	12136	374043	0.03245		
A-579-01.037.MC.P01.01.01.dpcranalysis.xlsx	A-579-01.353.NSPRP.14Sep2023.07	0.0537	70608	336765	0.20967		

IND: indeterminate, CV% >20 (only applicable to results above the lower limit of quantitation[LLOQ])
BQL: below quantitation limit (below LLOQ)
>ULOQ: above upper limit of quantitation
LLOQ = 14.9 copies/µL
ULOQ = 16800 copies/µL

Source.Name	Sample Name	DNA Input (ug)	AAV2-ITR.Target Copy Num Per ug DNA	B2M.Target Copy Num Per ug DNA	AAV2-ITR/B2M ratio	AAV2-ITR.Comment	AAV2-ITR.CV Flag
A-579-01.037.MC.P01.01.01.dpcranalysis.xlsx	A-579-01.353.NUDLD.14Sep2023.05	0.0648	101910	308965	0.32984		
A-579-01.037.MC.P01.01.01.dpcranalysis.xlsx	A-579-01.353.NUDLP.14Sep2023.03	0.075	73858	449587	0.16428		
A-579-01.037.MC.P01.01.01.dpcranalysis.xlsx	A-579-01.353.NUDRD.14Sep2023.11	0.0381	114413	331407	0.34523		
A-579-01.037.MC.P01.01.01.dpcranalysis.xlsx	A-579-01.353.NUDRP.14Sep2023.08	0.0525	148293	321609	0.46110		
A-579-01.038.MC.P01.01.01.dpcranalysis.xlsx	A-579-01.353.NUPLD.14Sep2023.04	0.0297	12245	373365	0.03280		
A-579-01.037.MC.P01.01.02.dpcranalysis.xlsx	A-579-01.353.NUPLP.14Sep2023.03	0.054	110743	293883	0.37683		
A-579-01.038.MC.P01.01.02.dpcranalysis.xlsx	A-579-01.353.NUPRD.14Sep2023.10	0.0414	141116	343727	0.41055		
A-579-01.038.MC.P01.01.01.dpcranalysis.xlsx	A-579-01.353.NUPRP.14Sep2023.07	0.0414	77338	302477	0.25568		
A-579-01.037.MC.P01.01.01.dpcranalysis.xlsx	A-579-01.353.NVOT.14Sep2023.08	0.075	53138	335938	0.15818		
A-579-01.038.MC.P01.01.01.dpcranalysis.xlsx	A-579-01.353.OVAS.14Sep2023.02	0.075	14047	242511	0.05792		
A-579-01.038.MC.P01.01.01.dpcranalysis.xlsx	A-579-01.353.SCCV.14Sep2023.02	0.0135	319960	428780	0.74621		
A-579-01.038.MC.P01.01.01.dpcranalysis.xlsx	A-579-01.353.SCLU.14Sep2023.02	0.0198	321882	290677	1.10735		
A-579-01.038.MC.P01.01.01.dpcranalysis.xlsx	A-579-01.353.SCSA.14Sep2023.03	0.0471	194088	335950	0.57773		
A-579-01.038.MC.P01.01.01.dpcranalysis.xlsx	A-579-01.353.SCSR.14Sep2023.01	0.075	365307	331246	1.10283		
A-579-01.038.MC.P01.01.01.dpcranalysis.xlsx	A-579-01.353.SCTH.14Sep2023.02	0.042	77422	228478	0.33886		
A-579-01.038.MC.P01.01.01.dpcranalysis.xlsx	A-579-01.353.SPLN.14Sep2023.03	0.075	819566	251328	3.26094		
A-579-01.038.MC.P01.01.02.dpcranalysis.xlsx	A-579-01.353.TGL1.14Sep2023.02	0.075	27795	267764	0.10380		
A-579-01.038.MC.P01.01.01.dpcranalysis.xlsx	A-579-01.353.TGL2.14Sep2023.02	0.075	835850	272129	3.07152		
A-579-01.038.MC.P01.01.01.dpcranalysis.xlsx	A-579-01.353.TGL3.14Sep2023.03	0.075	302400	247340	1.22261		
A-579-01.039.MC.P01.01.01.dpcranalysis.xlsx	A-579-01.354.BRAN.14Sep2023.03	0.075	1403201	404591	3.46820		
A-579-01.039.MC.P01.01.01.dpcranalysis.xlsx	A-579-01.354.DRGC.14Sep2023.01	0.075	522972	273516	1.91203		
A-579-01.060.MC.P01.02.01.dpcranalysis.xlsx	A-579-01.354.DRGL.14Sep2023.02	0.00375	2921831	318889	9.16254		
A-579-01.060.MC.P01.02.01.dpcranalysis.xlsx	A-579-01.354.DRGT.14Sep2023.01	0.00375	3933689	284542	13.82462		
A-579-01.040.MC.P01.01.01.dpcranalysis.xlsx	A-579-01.354.HTHT.14Sep2023.01	0.0573	164270	263451	0.62353		
A-579-01.040.MC.P01.01.01.dpcranalysis.xlsx	A-579-01.354.KDNY.14Sep2023.03	0.075	52229	242088	0.21575		
A-579-01.040.MC.P01.01.01.dpcranalysis.xlsx	A-579-01.354.LUNG.14Sep2023.03	0.075	32138	185470	0.17328		
A-579-01.060.MC.P01.02.01.dpcranalysis.xlsx	A-579-01.354.LVER.14Sep2023.02	0.00375	7694773	292967	26.26497		
A-579-01.039.MC.P01.01.01.dpcranalysis.xlsx	A-579-01.354.MUAL.14Sep2023.02	0.0387	8317	246176	0.03378		
A-579-01.039.MC.P01.01.01.dpcranalysis.xlsx	A-579-01.354.MUAR.14Sep2023.03	0.0696	5530	365086	0.01515		
A-579-01.039.MC.P01.01.01.dpcranalysis.xlsx	A-579-01.354.MUBL.14Sep2023.01	0.0531	10387	323331	0.03212		
A-579-01.039.MC.P01.01.01.dpcranalysis.xlsx	A-579-01.354.MUBR.14Sep2023.03	0.0618	4274	281472	0.01518		
A-579-01.040.MC.P01.01.01.dpcranalysis.xlsx	A-579-01.354.NFDLD.14Sep2023.05	0.075	71400	298604	0.23911		
A-579-01.040.MC.P01.01.01.dpcranalysis.xlsx	A-579-01.354.NFDLP.14Sep2023.02	0.075	9727	354461	0.02744		
A-579-01.040.MC.P01.01.01.dpcranalysis.xlsx	A-579-01.354.NFDRD.14Sep2023.11	0.075	12975	241895	0.05364		
A-579-01.040.MC.P01.01.01.dpcranalysis.xlsx	A-579-01.354.NFDRP.14Sep2023.07	0.0588	10476	304516	0.03440		
A-579-01.040.MC.P01.01.01.dpcranalysis.xlsx	A-579-01.354.NFPLD.14Sep2023.04	0.075	4945	270424	0.01829		
A-579-01.040.MC.P01.01.02.dpcranalysis.xlsx	A-579-01.354.NFPLP.14Sep2023.03	0.0579	11673	307178	0.03800		
A-579-01.040.MC.P01.01.01.dpcranalysis.xlsx	A-579-01.354.NFPRD.14Sep2023.10	0.0573	9791	321031	0.03050		
A-579-01.040.MC.P01.01.01.dpcranalysis.xlsx	A-579-01.354.NFPRP.14Sep2023.07	0.075	13666	279807	0.04884		

IND: indeterminate, CV% >20 (only applicable to results above the lower limit of quantitation[LLOQ])
BQL: below quantitation limit (below LLOQ)
>ULOQ: above upper limit of quantitation
LLOQ = 14.9 copies/μL
ULOQ = 16800 copies/μL

Source.Name	Sample Name	DNA Input (ug)	AAV2-ITR.Target Copy Num Per ug DNA	B2M.Target Copy Num Per ug DNA	AAV2-ITR/B2M ratio	AAV2-ITR.Comment	AAV2-ITR.CV Flag
A-579-01.043.MC.P01.02.01.dpcranalysis.xlsx	A-579-01.354.NMDLD.14Sep2023.05	0.0663	9386	417536	0.02248		
A-579-01.039.MC.P01.01.01.dpcranalysis.xlsx	A-579-01.354.NMDLP.14Sep2023.01	0.075	4970	381715	0.01302		
A-579-01.039.MC.P01.01.01.dpcranalysis.xlsx	A-579-01.354.NMDRD.14Sep2023.10	0.0573	3653	321063	0.01138		
A-579-01.070.MC.P01.02.01.dpcranalysis.xlsx	A-579-01.354.NMDRP.14Sep2023.08	0.0543	5910	445576	0.01326		
A-579-01.039.MC.P01.01.02.dpcranalysis.xlsx	A-579-01.354.NMPLD.14Sep2023.04	0.075	1237	330988	0.00374	BQL	
A-579-01.039.MC.P01.01.01.dpcranalysis.xlsx	A-579-01.354.NMPLP.14Sep2023.02	0.075	3899	304715	0.01280		
A-579-01.039.MC.P01.01.01.dpcranalysis.xlsx	A-579-01.354.NMPRD.14Sep2023.10	0.0444	5268	390644	0.01349		
A-579-01.039.MC.P01.01.01.dpcranalysis.xlsx	A-579-01.354.NMPRP.14Sep2023.07	0.0273	16082	329624	0.04879		
A-579-01.039.MC.P01.01.01.dpcranalysis.xlsx	A-579-01.354.NSDLD.14Sep2023.05	0.075	19647	342440	0.05737		
A-579-01.039.MC.P01.01.01.dpcranalysis.xlsx	A-579-01.354.NSDLP.14Sep2023.01	0.0366	150392	414186	0.36310		
A-579-01.039.MC.P01.01.01.dpcranalysis.xlsx	A-579-01.354.NSDRD.14Sep2023.11	0.0648	18726	395730	0.04732		
A-579-01.039.MC.P01.01.02.dpcranalysis.xlsx	A-579-01.354.NSDRP.14Sep2023.08	0.075	17779	308420	0.05765		
A-579-01.039.MC.P01.01.02.dpcranalysis.xlsx	A-579-01.354.NSPLD.14Sep2023.04	0.075	37763	367649	0.10271		
A-579-01.070.MC.P01.02.02.dpcranalysis.xlsx	A-579-01.354.NSPLP.14Sep2023.03	0.075	27860	393857	0.07074		
A-579-01.039.MC.P01.01.01.dpcranalysis.xlsx	A-579-01.354.NSPRD.14Sep2023.11	0.0228	63831	434205	0.14701		
A-579-01.039.MC.P01.01.02.dpcranalysis.xlsx	A-579-01.354.NSPRP.14Sep2023.09	0.075	17728	369588	0.04797		
A-579-01.039.MC.P01.01.01.dpcranalysis.xlsx	A-579-01.354.NUDLD.14Sep2023.05	0.0645	355014	2275	0.00641		
A-579-01.039.MC.P01.01.01.dpcranalysis.xlsx	A-579-01.354.NUDLP.14Sep2023.03	0.075	3869	365630	0.01058		
A-579-01.039.MC.P01.01.02.dpcranalysis.xlsx	A-579-01.354.NUDRD.14Sep2023.12	0.075	10466	470083	0.02227		
A-579-01.039.MC.P01.01.01.dpcranalysis.xlsx	A-579-01.354.NUDRP.14Sep2023.09	0.075	9293	324016	0.02868		
A-579-01.040.MC.P01.01.01.dpcranalysis.xlsx	A-579-01.354.NUPLD.14Sep2023.05	0.075	44651	331364	0.13475		
A-579-01.039.MC.P01.01.01.dpcranalysis.xlsx	A-579-01.354.NUPLP.14Sep2023.02	0.0171	82349	359634	0.22898		
A-579-01.040.MC.P01.01.02.dpcranalysis.xlsx	A-579-01.354.NUPRD.14Sep2023.10	0.075	5580	259952	0.02147		
A-579-01.040.MC.P01.01.02.dpcranalysis.xlsx	A-579-01.354.NUPRP.14Sep2023.09	0.075	9871	219007	0.04507		
A-579-01.039.MC.P01.01.01.dpcranalysis.xlsx	A-579-01.354.NVOT.14Sep2023.08	0.075	755353	376476	2.00638		
A-579-01.045.MC.P01.02.01.dpcranalysis.xlsx	A-579-01.354.OVAS.14Sep2023.02	0.075	12605	368209	0.03423		
A-579-01.040.MC.P01.01.01.dpcranalysis.xlsx	A-579-01.354.SCCV.14Sep2023.01	0.075	1195973	354175	3.37679		
A-579-01.040.MC.P01.01.01.dpcranalysis.xlsx	A-579-01.354.SCLU.14Sep2023.02	0.0501	2469496	260570	9.47730		
A-579-01.040.MC.P01.01.01.dpcranalysis.xlsx	A-579-01.354.SCSA.14Sep2023.01	0.075	1381738	379692	3.63910		
A-579-01.060.MC.P01.02.01.dpcranalysis.xlsx	A-579-01.354.SCSR.14Sep2023.03	0.003	9303311	302027	30.80295		
A-579-01.060.MC.P01.02.01.dpcranalysis.xlsx	A-579-01.354.SCTH.14Sep2023.01	0.00375	6254702	301604	20.73816		
A-579-01.040.MC.P01.01.01.dpcranalysis.xlsx	A-579-01.354.SPLN.14Sep2023.01	0.075	449164	260680	1.72305		

IND: indeterminate, CV% >20 (only applicable to results above the lower limit of quantitation[LLOQ])
BQL: below quantitation limit (below LLOQ)
>ULOQ: above upper limit of quantitation
LLOQ = 14.9 copies/µL
ULOQ = 16800 copies/µL

Attachment 3. Precision Principal Investigator Report

A 12-week Single Dose Biodistribution and Safety Study in Cynomolgus Monkeys

Amplify Bio Study Number

A-579-01

Delegated Phase(s):

Biodistribution Statistical Analysis

Principal Investigator (PI):

Zachary Cole, Director of Academic Consulting, Precision Consulting, LLC

[signature]	April 2nd, 2024
**PI Signature**	**Date**

Study Objectives and Design Overview

The primary objective of this analysis was to quantify the vector genome copies of the target gene with and without normalization to house-keeping gene at the two timepoints (Day 43 or Day 85) by digital PCR (dPCR). The analysis included assessing differences in biodistribution/accumulation of the target gene between tissues, groups (dose levels), and timepoints (Day 43 and Day 85). The study design involves administering varying doses of a vector to different groups and quantifying the target gene copies in collected tissue samples using digital PCR (dPCR). This study included three dose groups of Vehicle, Low Dose, and High Dose with both male and female animals. Data points included for the analyses were from day 43 and day 85 post-administration. The sample included a total of 20 animals (10 per timepoint) and 52 tissues collected per animal, resulting in 1,040 samples for analysis.

Table 1

Number of Samples Per Dose Group, Gender, and Timepoint

Dose Groups	Targe Dose	Number of Animals			
		Day 43 Cohort		Day 85 Cohort	
		Male	Female	Male	Female
Group 1	Vehicle	1	1	1	1
Group 2	Low Dose of the vector	2	2	2	2
Group 3	High Dose of the vector	2	2	2	2

Statistical Analysis Overview

Statistical comparisons were conducted to generate estimates of, and evaluate differences in,

- biodistribution of a target gene between tissues and/or the accumulation within a tissue
- evaluate differences in biodistribution/accumulation of a target gene between groups.

- evaluate differences in biodistribution/accumulation of a target gene between timepoints across all groups.

Non-detectable values were imputed using the minimum value observed divided by two. Linear mixed effects models with a random intercept for each animal were specified to account for repeated measures within each of the 20 animals These models were estimated using Restricted Maximum Likelihood (REML) and Satterthwaite method was applied to determine the denominator degrees of freedom. Then, the estimates the average amount of the test article for sex, dose, each tissue, and each timepoint, along with 95% confidence intervals were calculated.

The variables being assessed were normalized and not normalized data on AAV2-ITR target copies/ug of DNA. Mixed effect models were performed to account for repeated observation by the 20 animals. In these models, the identifier for animal was included as the random factor, and group, tissue, timepoint, and gender were included as fixed factors. Estimates of the fixed effects were assessed to examine the main effects of these variables on AAV2-ITR target copies/ug of DNA. Furthermore, ANOVA was used to compare the three group types for each tissue and timepoint separately. For each of these ANOVAs turkey post hoc tests were performed to compare the groups and test for statistical significance between them. Additionally, pairwise comparisons were made for each of the tissues for each time point and group separately. Moreover, the two timepoints were compared for each tissue and group separately. For all these comparisons, tests of differences, p-values, and 95% confidence intervals were calculated.

The analyses were performed based on both normalized and not-normalized data for AAV2-ITR target copies/ug of DNA. The data were normalized by dividing AAV2-ITR target copies/ug of DNA for each sample by B2M target copies/ug of DNA.

Prior to running the statistical analyses, the distribution of the data was examined using histograms to identify an appropriate type of data transformation. For this purpose, original data were compared with log10-transfomred data and square-root transformed. Through the visual inspections of the data, it was found that using log10-transformed data resulted in overall less skewed data for both normalized and not-normalized AAV2-ITR target copies/ug of DNA Thus, this type of data transformation was applied for all statistical analyses. Figures 1 and 2 show how data transformation substantially improves skewness in the data for Timepoint = Day 85 and Group = High Dose.

Figure 1

Histograms of Log10 AAV2-ITR.Target Copy Num Per ug DNA (Not-Normalized) for Different Tissues for the Original Data for Timepoint = Day 85 and Group = High Dose

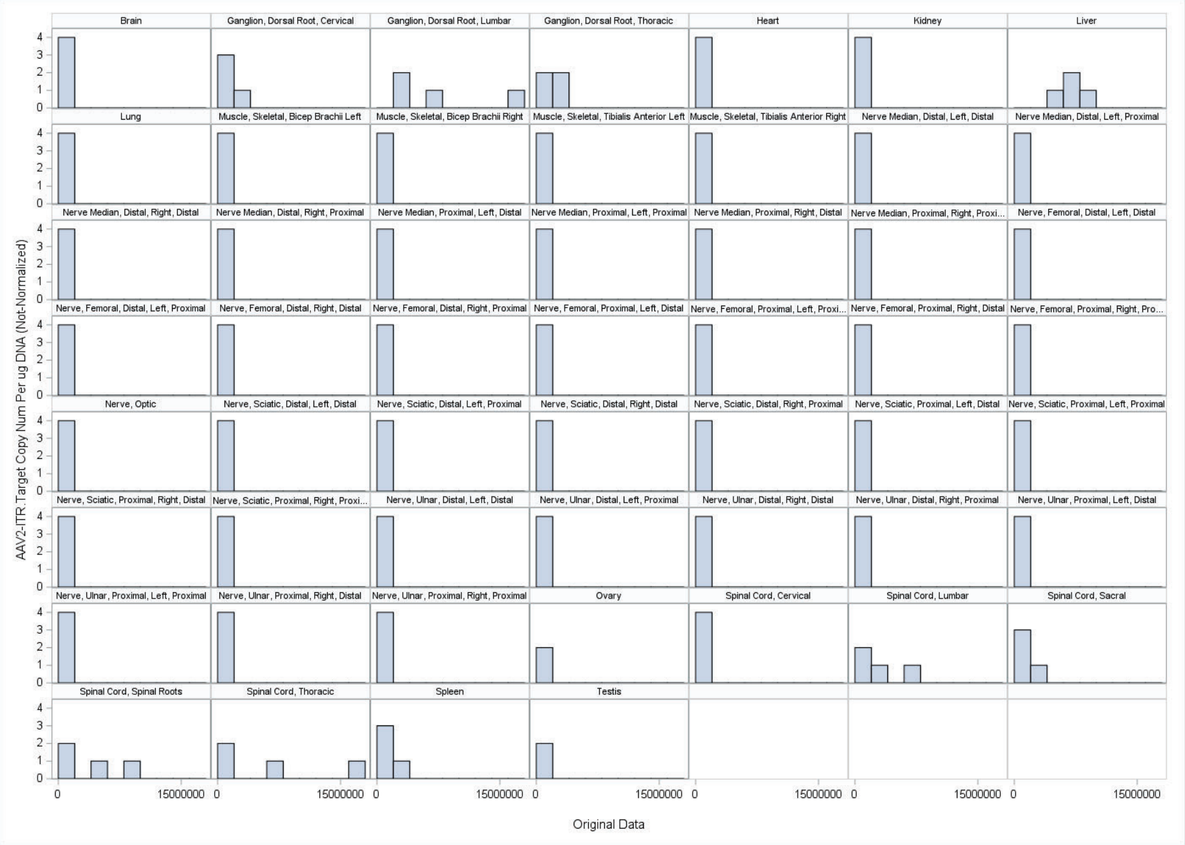

Figure 2

Histograms of Log10 AAV2-ITR.Target Copy Num Per ug DNA (Not-Normalized) for Different Tissues for the Log10-Transformed Data for Timepoint = Day 85 and Group = High Dose

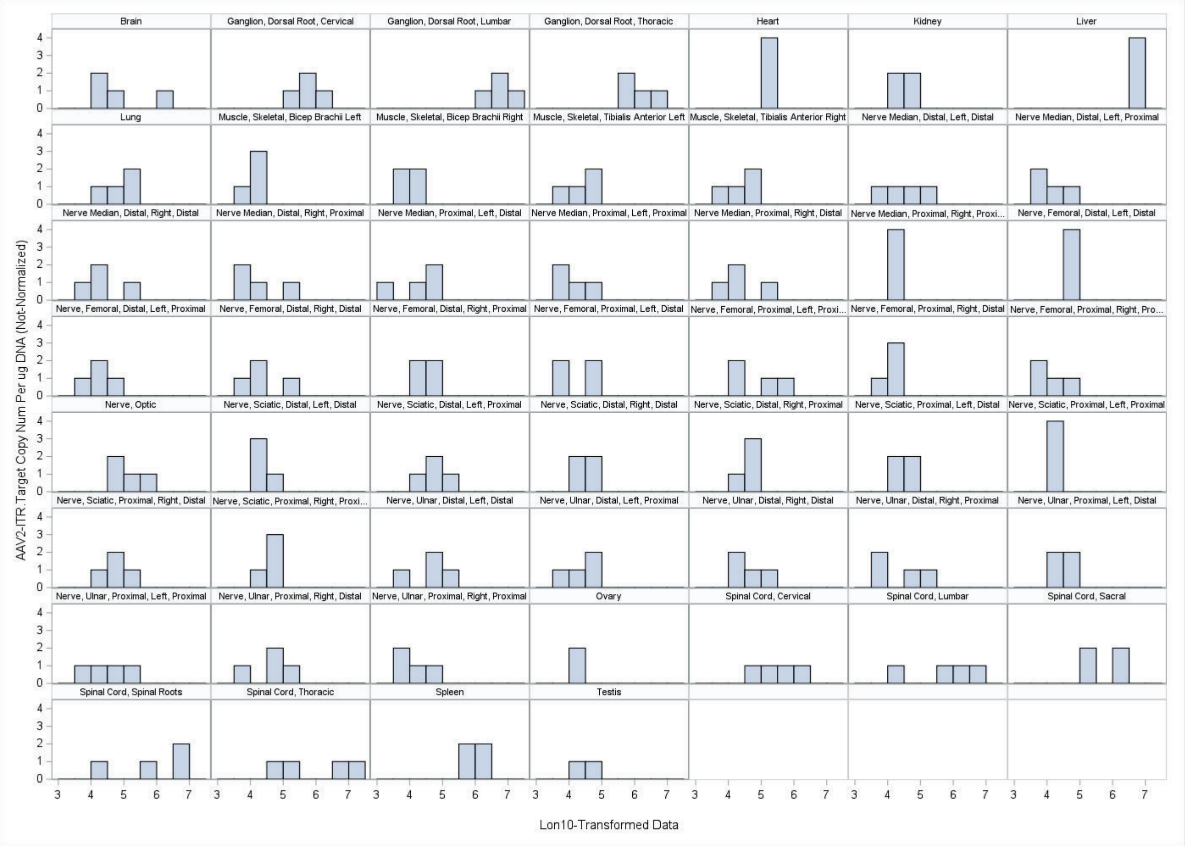

Results

Mixed-fixed effects models were performed for both normalized and not-normalized data log10 AAV2-ITR target copies/ug of DNA. In these models, the identifier for animal was used as the random factor to account for repeated measurements for animals in the sample. In these models, group, timepoint, gender, and tissues were included as fixed factors. Table 2 shows the estimates of fixed effects for these factors. From this table it can be seen that for the normalized data, the main effects of group, timepoint, and tissue were statistically significant, meaning that after accounting for repeated measurements from the different animals in the sample, there were statistically significant differences in data log10 AAV2-ITR target copies/ug of DNA across group, timepoint, and tissue. However, no statistically significant main effect was identified for gender. On the other hand, for not-normalized data, statistically significant differences were identified in group and tissue, but not for gender or timepoint. These results indicated that, for not normalized data, no statistically significant differences were identified between male and female animals or between Day 43 and Day 85.

Table 2

Estimates of Fixed-Effects for the Fixed-Factors Included in the Mixed-Effect Models for both Normalized and Not-Normalized Data

Type of Data	Variable	Df1	Df2	F	p-value
Normalized	Group	2	17	8.61	.003
	Timepoint	1	18	6.60	.019
	Gender	1	18	0.03	.857
	Tissue	52	968	17.04	< .001
Not normalized	Group	2	17	178.89	< .001
	Timepoint	1	18	0.40	.535
	Gender	1	18	0.01	.923
	Tissue	52	968	16.19	< .001

Tables 3 and 4 present the estimated means for log10 AAV2-ITR target copies/ug of DNA for normalized and not-normalized data, respectively, based on the results of the mixed-effect models.

Table 3

Estimated Means Based on the Results of Linear Mixed-Effect Models for Different Categories of Group, Gender, Timepoint, and Tissue for Normalized Data

	Category	Estimated Mean	Standard Error	Lower 95% CL	Upper 95% CI
Group	High Dose	0.25	0.04	0.17	0.33
Group	Low Dose	0.27	0.04	0.19	0.35
Group	Vehicle	0.00	0.06	-0.12	0.12
Timepoint	Day 43	0.28	0.04	0.20	0.37
Timepoint	Day 85	0.13	0.04	0.04	0.22
Gender	Female	0.21	0.05	0.11	0.32
Gender	Male	0.20	0.05	0.10	0.30
Tissue	Brain	0.18	0.07	0.05	0.32
Tissue	Ganglion, Dorsal Root, Cervical	0.67	0.07	0.53	0.81
Tissue	Ganglion, Dorsal Root, Lumbar	0.80	0.07	0.67	0.94
Tissue	Ganglion, Dorsal Root, Thoracic	0.79	0.07	0.65	0.93
Tissue	Heart	0.17	0.07	0.04	0.31
Tissue	Kidney	0.07	0.07	-0.07	0.21
Tissue	Lung	0.09	0.07	-0.05	0.23
Tissue	Liver	1.09	0.07	0.95	1.23
Tissue	Muscle, Skeletal, Tibialis Anterior Left	0.04	0.07	-0.10	0.18
Tissue	Muscle, Skeletal, Tibialis Anterior Right	0.03	0.07	-0.10	0.17
Tissue	Muscle, Skeletal, Bicep Brachii Left	0.03	0.07	-0.10	0.17
Tissue	Muscle, Skeletal, Bicep Brachii Right	0.03	0.07	-0.11	0.17
Tissue	Nerve, Femoral, Distal, Left, Distal	0.11	0.07	-0.03	0.25
Tissue	Nerve, Femoral, Distal, Left, Proximal	0.12	0.07	-0.02	0.25
Tissue	Nerve, Femoral, Distal, Right, Distal	0.10	0.07	-0.04	0.24
Tissue	Nerve, Femoral, Distal, Right, Proximal	0.11	0.07	-0.03	0.24
Tissue	Nerve, Femoral, Proximal, Left, Distal	0.09	0.07	-0.05	0.22
Tissue	Nerve, Femoral, Proximal, Left, Proximal	0.09	0.07	-0.04	0.23
Tissue	Nerve, Femoral, Proximal, Right, Distal	0.09	0.07	-0.05	0.23
Tissue	Nerve, Femoral, Proximal, Right, Proximal	0.07	0.07	-0.07	0.20
Tissue	Nerve Median, Distal, Left, Distal	0.08	0.07	-0.06	0.22
Tissue	Nerve Median, Distal, Left, Proximal	0.09	0.07	-0.05	0.22

	Category	Estimated Mean	Standard Error	Lower 95% CL	Upper 95% CI
Tissue	Nerve Median, Distal, Right, Distal	0.05	0.07	-0.09	0.19
Tissue	Nerve Median, Distal, Right, Proximal	0.08	0.07	-0.06	0.21
Tissue	Nerve Median, Proximal, Left, Distal	0.06	0.07	-0.07	0.20
Tissue	Nerve Median, Proximal, Left, Proximal	0.07	0.07	-0.06	0.21
Tissue	Nerve Median, Proximal, Right, Distal	0.11	0.07	-0.03	0.25
Tissue	Nerve Median, Proximal, Right, Proximal	0.07	0.07	-0.07	0.20
Tissue	Nerve, Sciatic, Distal, Left, Distal	0.07	0.07	-0.06	0.21
Tissue	Nerve, Sciatic, Distal, Left, Proximal	0.14	0.07	0.01	0.28
Tissue	Nerve, Sciatic, Distal, Right, Distal	0.07	0.07	-0.06	0.21
Tissue	Nerve, Sciatic, Distal, Right, Proximal	0.09	0.07	-0.05	0.23
Tissue	Nerve, Sciatic, Proximal, Left, Distal	0.10	0.07	-0.04	0.24
Tissue	Nerve, Sciatic, Proximal, Left, Proximal	0.11	0.07	-0.03	0.24
Tissue	Nerve, Sciatic, Proximal, Right, Distal	0.12	0.07	-0.02	0.26
Tissue	Nerve, Sciatic, Proximal, Right, Proximal	0.09	0.07	-0.05	0.23
Tissue	Nerve, Ulnar, Distal, Left, Distal	0.06	0.07	-0.07	0.20
Tissue	Nerve, Ulnar, Distal, Left, Proximal	0.09	0.07	-0.04	0.23
Tissue	Nerve, Ulnar, Distal, Right, Distal	0.07	0.07	-0.07	0.21
Tissue	Nerve, Ulnar, Distal, Right, Proximal	0.07	0.07	-0.07	0.20
Tissue	Nerve, Ulnar, Proximal, Left, Distal	0.09	0.07	-0.05	0.22
Tissue	Nerve, Ulnar, Proximal, Left, Proximal	0.06	0.07	-0.08	0.20
Tissue	Nerve, Ulnar, Proximal, Right, Distal	0.09	0.07	-0.05	0.23
Tissue	Nerve, Ulnar, Proximal, Right, Proximal	0.09	0.07	-0.05	0.23
Tissue	Nerve, Optic	0.27	0.07	0.13	0.41
Tissue	Ovary	0.07	0.09	-0.11	0.26
Tissue	Spinal Cord, Cervical	0.54	0.07	0.40	0.67
Tissue	Spinal Cord, Lumbar	0.60	0.07	0.46	0.73
Tissue	Spinal Cord, Sacral	0.56	0.07	0.42	0.70
Tissue	Spinal Cord, Spinal Roots	0.63	0.07	0.49	0.76
Tissue	Spinal Cord, Thoracic	0.79	0.07	0.65	0.92
Tissue	Spleen	0.57	0.07	0.44	0.71
Tissue	Testis	0.03	0.09	-0.16	0.21

Table 3

Estimated Means Based on the Results of Linear Mixed-Effect Models for Different Categories of Group, Gender, Timepoint, and Tissue for Not-Normalized Data

	Category	Estimated Mean	Standard Error	Lower 95% CL	Upper 95% CI
Group	High Dose	5.07	0.16	4.73	5.40
Group	Low Dose	4.95	0.16	4.61	5.29
Group	Vehicle	0.18	0.23	-0.30	0.66
Timepoint	Day 43	4.33	0.65	2.96	5.70
Timepoint	Day 85	3.75	0.65	2.38	5.12
Gender	Female	4.00	0.66	2.62	5.38
Gender	Male	4.08	0.66	2.70	5.47
Tissue	Brain	4.12	0.47	3.15	5.10
Tissue	Ganglion, Dorsal Root, Cervical	5.02	0.47	4.05	6.00
Tissue	Ganglion, Dorsal Root, Lumbar	5.22	0.47	4.25	6.20
Tissue	Ganglion, Dorsal Root, Thoracic	5.09	0.47	4.12	6.07
Tissue	Heart	4.21	0.47	3.23	5.18
Tissue	Kidney	3.71	0.47	2.73	4.68
Tissue	Lung	3.90	0.47	2.92	4.87
Tissue	Liver	5.42	0.47	4.44	6.39
Tissue	Muscle, Skeletal, Tibialis Anterior Left	3.74	0.47	2.76	4.71
Tissue	Muscle, Skeletal, Tibialis Anterior Right	3.64	0.47	2.67	4.62
Tissue	Muscle, Skeletal, Bicep Brachii Left	3.58	0.47	2.61	4.56
Tissue	Muscle, Skeletal, Bicep Brachii Right	3.48	0.47	2.50	4.45
Tissue	Nerve, Femoral, Distal, Left, Distal	3.91	0.47	2.94	4.89
Tissue	Nerve, Femoral, Distal, Left, Proximal	3.97	0.47	2.99	4.95
Tissue	Nerve, Femoral, Distal, Right, Distal	3.82	0.47	2.84	4.79
Tissue	Nerve, Femoral, Distal, Right, Proximal	3.79	0.47	2.82	4.77
Tissue	Nerve, Femoral, Proximal, Left, Distal	3.71	0.47	2.74	4.69
Tissue	Nerve, Femoral, Proximal, Left, Proximal	3.72	0.47	2.75	4.70
Tissue	Nerve, Femoral, Proximal, Right, Distal	3.91	0.47	2.93	4.89
Tissue	Nerve, Femoral, Proximal, Right, Proximal	3.61	0.47	2.64	4.59
Tissue	Nerve Median, Distal, Left, Distal	3.79	0.47	2.82	4.77
Tissue	Nerve Median, Distal, Left, Proximal	3.74	0.47	2.77	4.72
Tissue	Nerve Median, Distal, Right, Distal	3.66	0.47	2.68	4.63
Tissue	Nerve Median, Distal, Right, Proximal	3.76	0.47	2.79	4.74
Tissue	Nerve Median, Proximal, Left, Distal	3.67	0.47	2.70	4.65

	Category	Estimated Mean	Standard Error	Lower 95% CL	Upper 95% CI
Tissue	Nerve Median, Proximal, Left, Proximal	3.75	0.47	2.78	4.73
Tissue	Nerve Median, Proximal, Right, Distal	3.85	0.47	2.87	4.82
Tissue	Nerve Median, Proximal, Right, Proximal	3.64	0.47	2.66	4.61
Tissue	Nerve, Sciatic, Distal, Left, Distal	3.90	0.47	2.92	4.87
Tissue	Nerve, Sciatic, Distal, Left, Proximal	4.03	0.47	3.06	5.01
Tissue	Nerve, Sciatic, Distal, Right, Distal	3.85	0.47	2.87	4.82
Tissue	Nerve, Sciatic, Distal, Right, Proximal	3.84	0.47	2.86	4.81
Tissue	Nerve, Sciatic, Proximal, Left, Distal	3.87	0.47	2.89	4.84
Tissue	Nerve, Sciatic, Proximal, Left, Proximal	3.89	0.47	2.91	4.86
Tissue	Nerve, Sciatic, Proximal, Right, Distal	4.07	0.47	3.10	5.05
Tissue	Nerve, Sciatic, Proximal, Right, Proximal	3.95	0.47	2.97	4.92
Tissue	Nerve, Ulnar, Distal, Left, Distal	3.81	0.47	2.83	4.78
Tissue	Nerve, Ulnar, Distal, Left, Proximal	3.76	0.47	2.78	4.73
Tissue	Nerve, Ulnar, Distal, Right, Distal	3.76	0.47	2.78	4.73
Tissue	Nerve, Ulnar, Distal, Right, Proximal	3.70	0.47	2.73	4.68
Tissue	Nerve, Ulnar, Proximal, Left, Distal	3.80	0.47	2.83	4.78
Tissue	Nerve, Ulnar, Proximal, Left, Proximal	3.57	0.47	2.60	4.55
Tissue	Nerve, Ulnar, Proximal, Right, Distal	3.78	0.47	2.80	4.75
Tissue	Nerve, Ulnar, Proximal, Right, Proximal	3.74	0.47	2.77	4.72
Tissue	Nerve, Optic	4.38	0.47	3.40	5.35
Tissue	Ovary	3.70	0.49	2.69	4.70
Tissue	Spinal Cord, Cervical	4.84	0.47	3.87	5.82
Tissue	Spinal Cord, Lumbar	4.84	0.47	3.87	5.82
Tissue	Spinal Cord, Sacral	4.82	0.47	3.85	5.80
Tissue	Spinal Cord, Spinal Roots	4.88	0.47	3.90	5.85
Tissue	Spinal Cord, Thoracic	5.01	0.47	4.03	5.98
Tissue	Spleen	5.09	0.47	4.11	6.06
Tissue	Testis	3.42	0.49	2.41	4.42

The comparisons of the 53 tissues for each group and time point, resulted in a total of 8268 comparisons. The same number of comparisons were done for not-normalized data. For the normalized data, a total of 1016 significant differences between the tissues were identified ($p < .05$), while this number for not-normalized data was 1458. Detailed results of these analyses for normalized and not normalized data can be found in the files "Comparing Tissues for Normalized Data.pdf" and "Comparing Tissues for Not-Normalized Data.pdf", respectively. Also, to compare Day 43 with Day 85 for each tissue and group, a total of 159 comparisons needed to be made. For normalized data, 25 of these comparisons were statistically significant ($p < .05$), while for not-normalized data 40 comparisons were found to be statistically significant ($p < .05$). Detailed results of these analyses for normalized and not normalized data are provided the files "Comparing Timepoints for Normalized Data.pdf" and "Comparing Timepoints for Not-Normalized Data.pdf", respectively. Additionally, to compare the three groups of vehicle, low dose, and high dose for each tissue and timepoint, a total of 318 comparisons were done. For normalized data 27, comparisons were statistically significant ($p < .05$), while for not-normalized data 214 comparisons were significant ($p < .05$). The results of these analyses for normalized and not normalized data can be found in the files "Comparing Groups for Normalized Data.pdf" and "Comparing Groups for Not-Normalized Data.pdf", respectively.

Figure 3 shows the line charts comparing the mean of log10 AAV2-ITR target copies/ug of DNA for not-normalized data between Day 43 and Day 85 for each group.

Figure 3

Comparing Mean Log10 AAV2-ITR.Target Copy Num Per ug DNA (Not-Normalized) by Group for Different Tissues

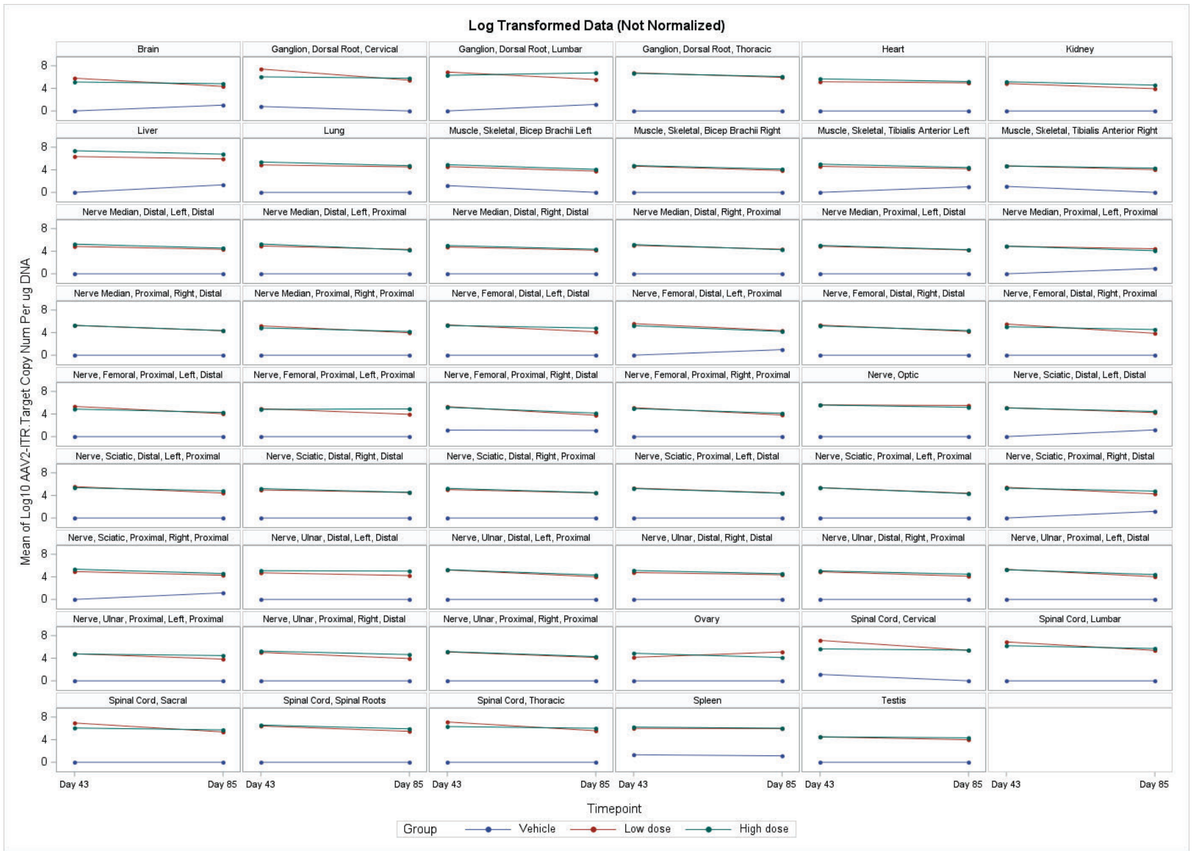

Figures 4 shows the line charts comparing the mean of log10 AAV2-ITR target copies/ug of DNA for normalized data between Day 43 and Day 85 for each group.

Figure 4

Comparing Mean Log10 AAV2-ITR.Target Copy Num Per ug DNA (Normalized) by Group for Different Tissues

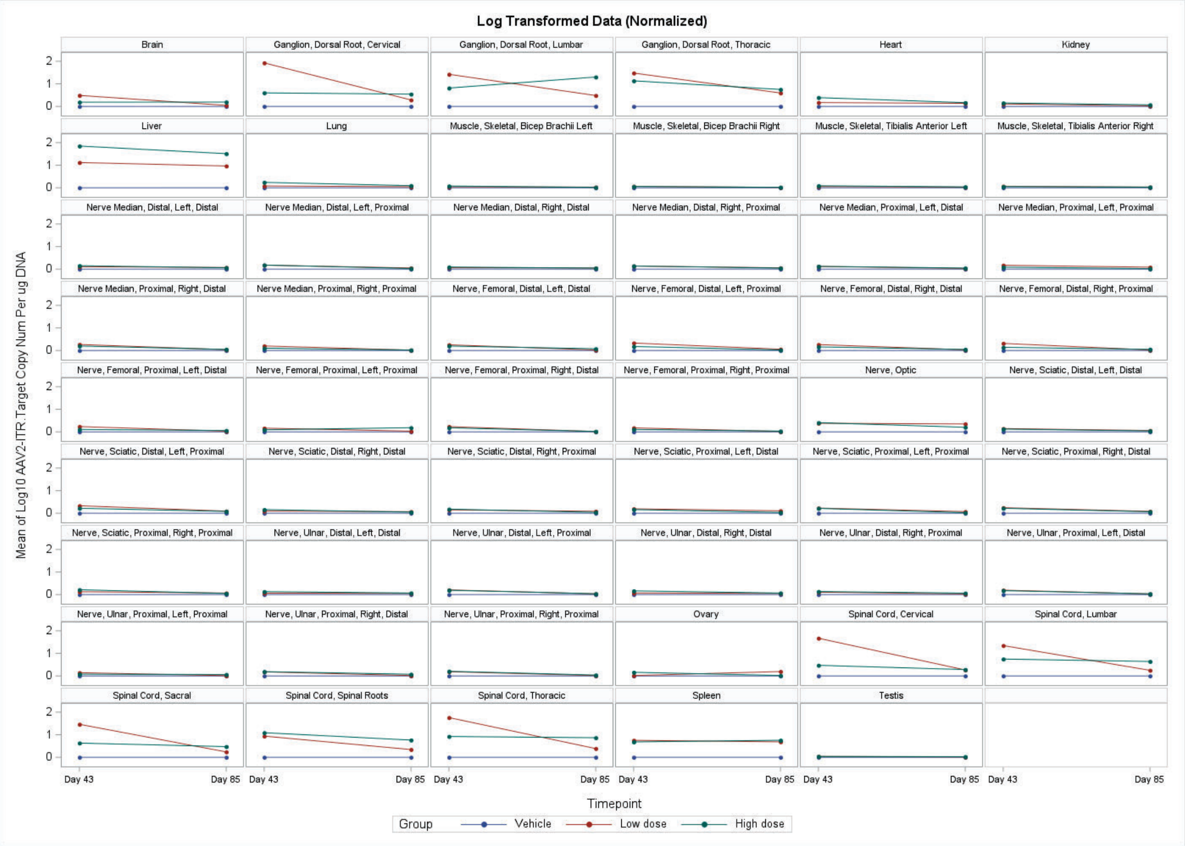

Figures 5 shows the boxplots of log10 AAV2-ITR target copies/ug of DNA for not-normalized data by group across the different tissues for Day 43.

Figure 5

Boxplots of Log10 AAV2-ITR.Target Copy Num Per ug DNA (Not-Normalized) by Group for Different Tissues for Day 43

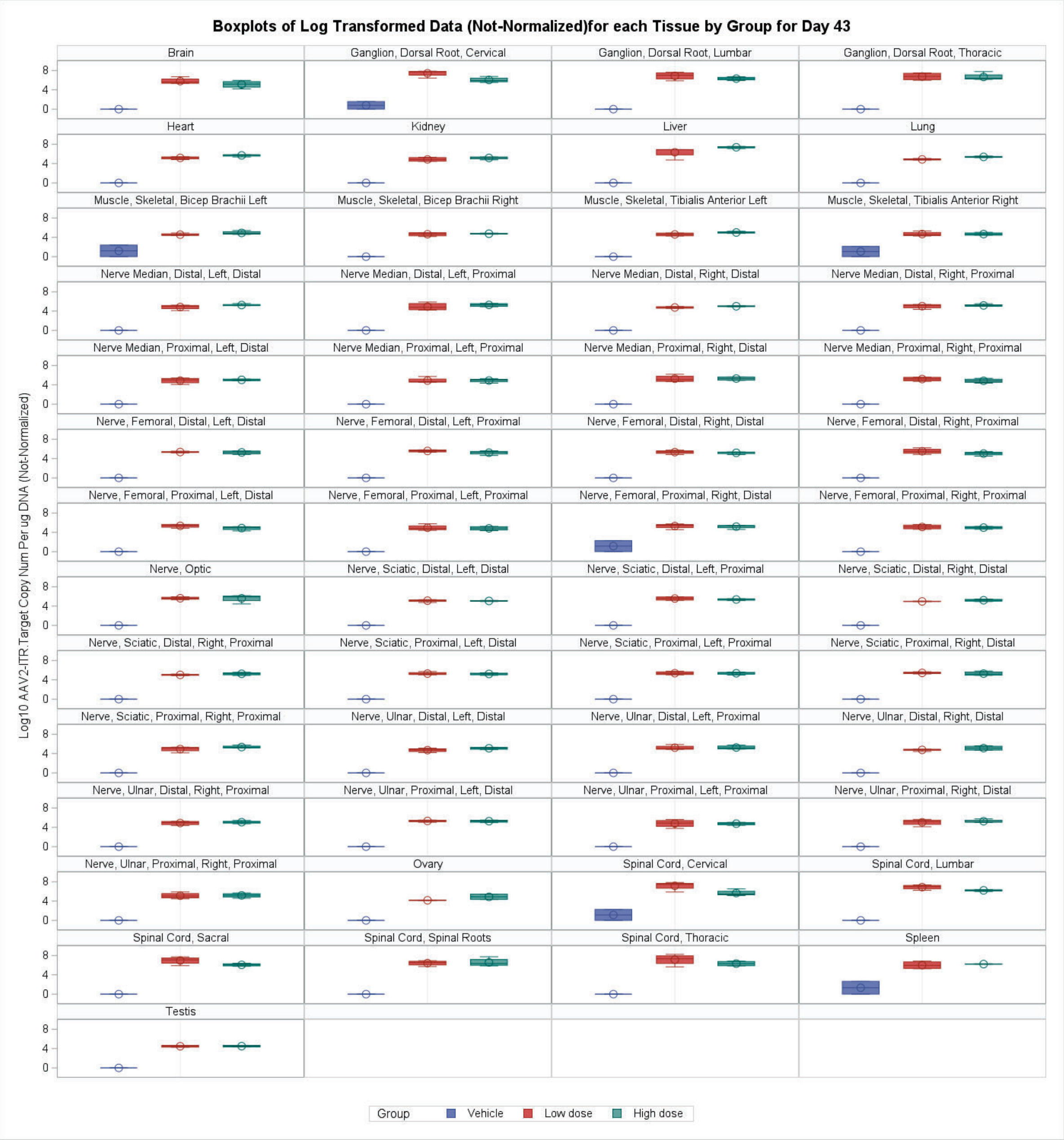

Figures 6 shows the boxplots of log10 AAV2-ITR target copies/ug of DNA for not-normalized data by group across the different tissues for Day 85.

Figure 6

Boxplots of Log10 AAV2-ITR.Target Copy Num Per ug DNA (Not-Normalized) by Group for Different Tissues for Day 85

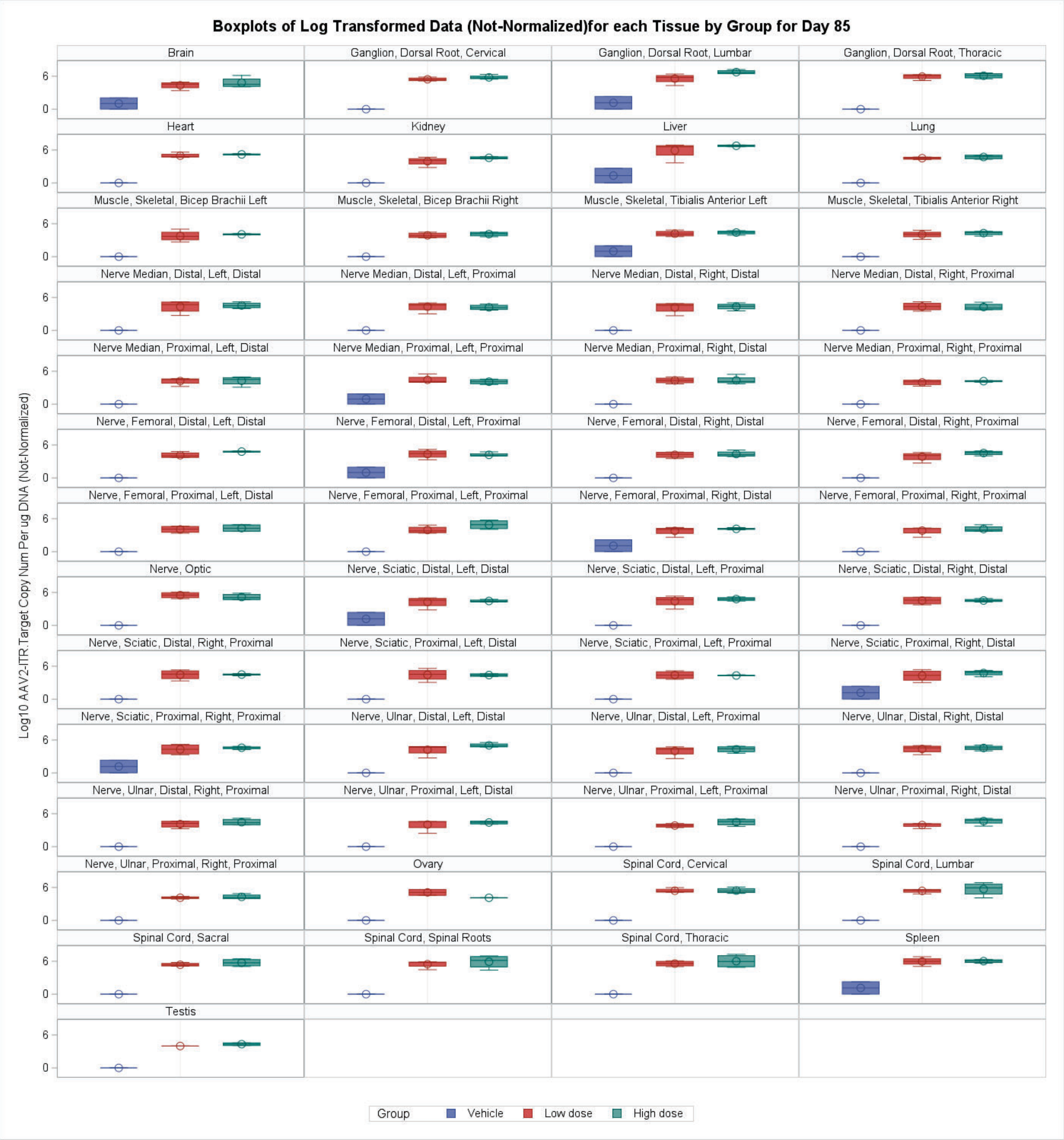

Figures 7 shows the boxplots of log10 AAV2-ITR target copies/ug of DNA for normalized data by group across the different tissues for day 43.

Figure 7

Boxplots of Log10 AAV2-ITR.Target Copy Num Per ug DNA (Normalized) by Group for Different Tissues for Day 43

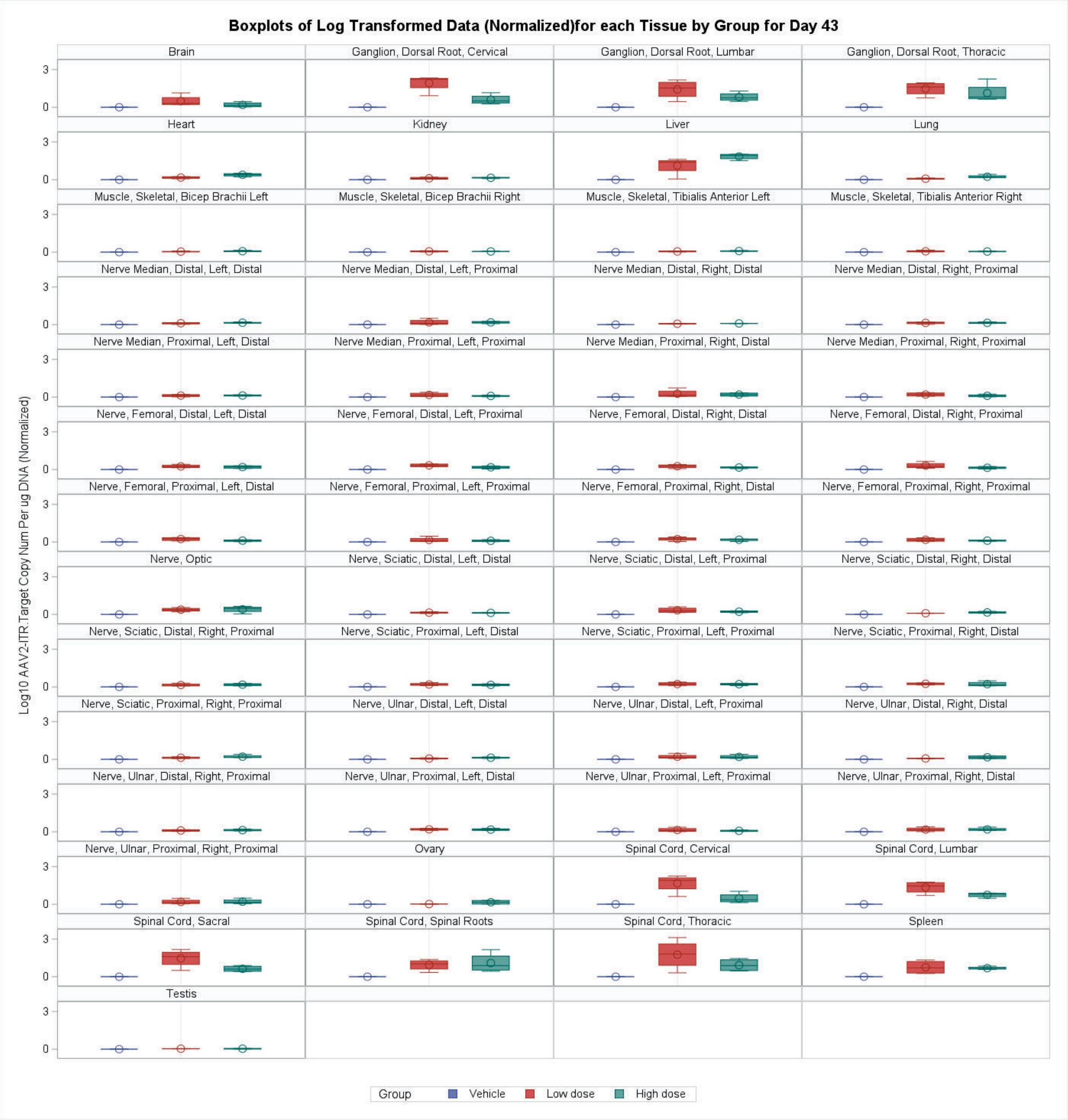

Figures 8 shows the boxplots of log10 AAV2-ITR target copies/ug of DNA for normalized data by group across the different tissues for day 85.

Figure 8

Boxplots of Log10 AAV2-ITR.Target Copy Num Per ug DNA (Normalized) by Group for Different Tissues for Day 85

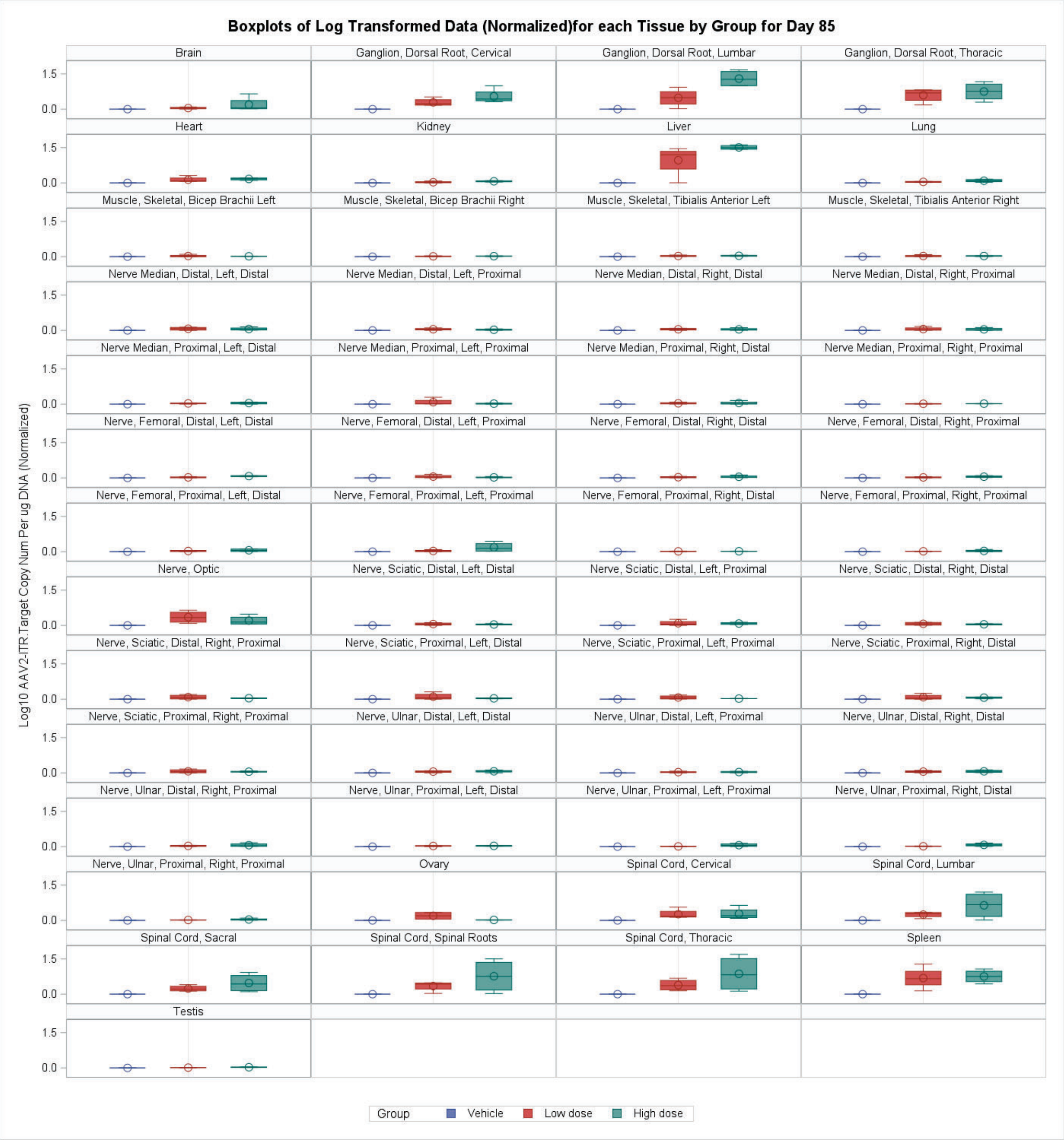

Figures 9-12 show the changes in mean log10 AAV2-ITR target copies/ug of DNA for normalized and not-normalized data by group across the different tissues for each timepoint separately. In these figures, error bars represent 95% confidence intervals for mean log10 AAV2-ITR target copies/ug of DNA. These bars were omitted for the vehicle group. This decision was made due to the atypically wide confidence intervals that resulted from plotting the vehicle group's data for some of the tissues. This was due to higher levels of variability for these tissues for the vehicle group compared to the other two groups for the different tissues. A consequence of including these bars would be a visually cluttered and potentially misleading representation of the data. Detailed information on the variability and confidence intervals for each group across the tissues and timepoints are provided in the documents “Statistics by Group for Normalized Data.pdf “ and “Statistics by Group for Not-Normalized Data.pdf “.

Figures 9 shows the changes in mean log10 AAV2-ITR target copies/ug of DNA for not-normalized data by group across the different tissues for day 43.

Figure 9

Assessment of Trends in Mean Log10 AAV2-ITR.Target Copy Num Per ug DNA (Not-Normalized) by Group Across Tissues for Day 43

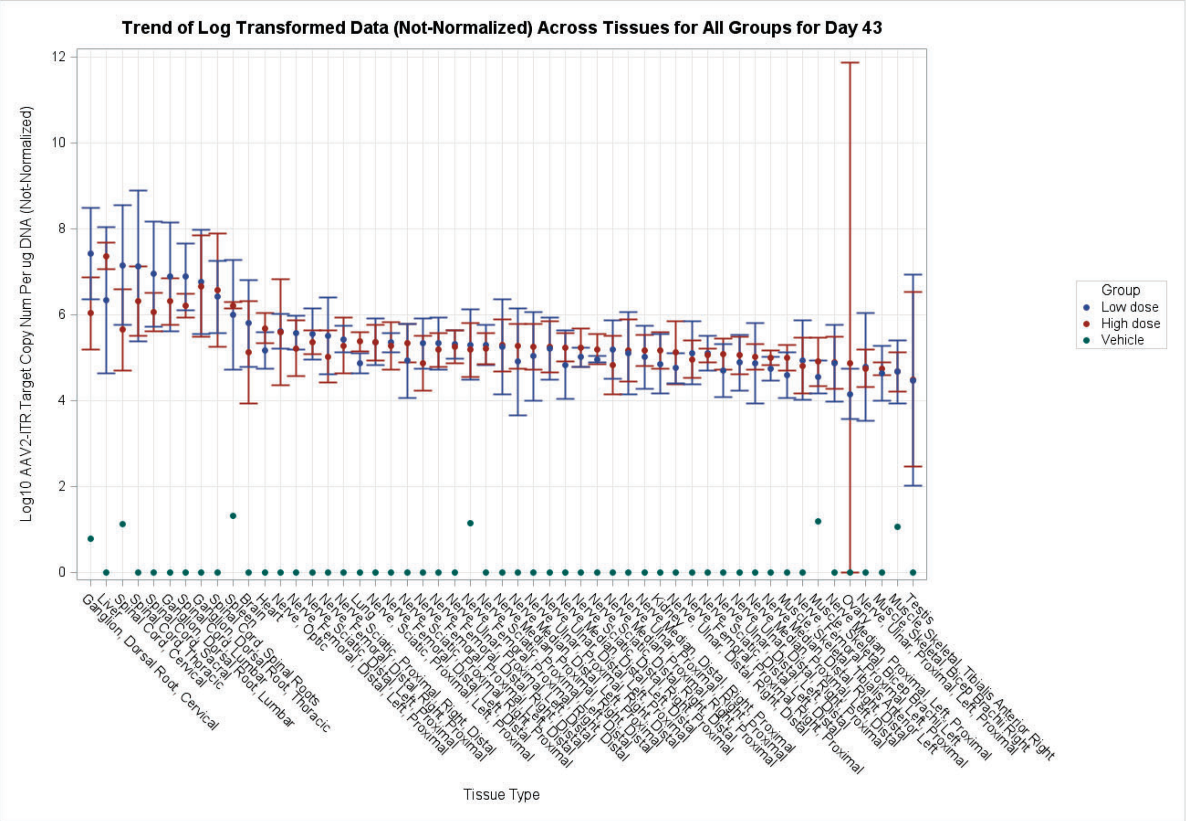

Note: Error bars for the low dose and high dose groups are calculated based on the 95% confidence intervals for the mean of each of these groups. The error bars have been omitted for the vehicle group.

Figures 10 shows the changes in mean log10 AAV2-ITR target copies/ug of DNA for not-normalized data by group across the different tissues for day 85.

Figure 10

Assessment of Trends in Mean Log10 AAV2-ITR.Target Copy Num Per ug DNA (Not-Normalized) by Group Across Tissues for Day 85

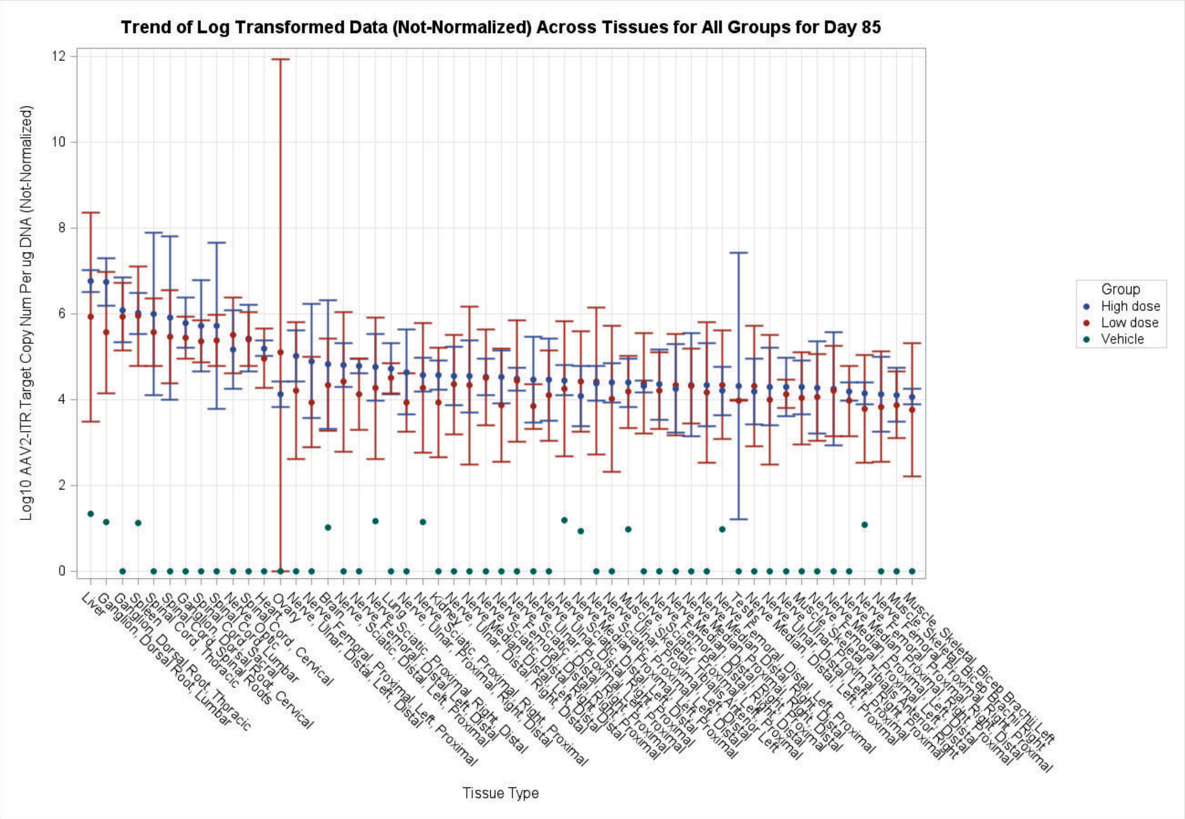

Note: Error bars for the low dose and high dose groups are calculated based on the 95% confidence intervals for the mean of each of these groups. The error bars have been omitted for the vehicle group.

Figures 11 shows the changes in mean log10 AAV2-ITR target copies/ug of DNA for normalized data by group across the different tissues for day 43.

Figure 11

Assessment of Trends in Mean Log10 AAV2-ITR.Target Copy Num Per ug DNA (Normalized) by Group Across Tissues for Day 43

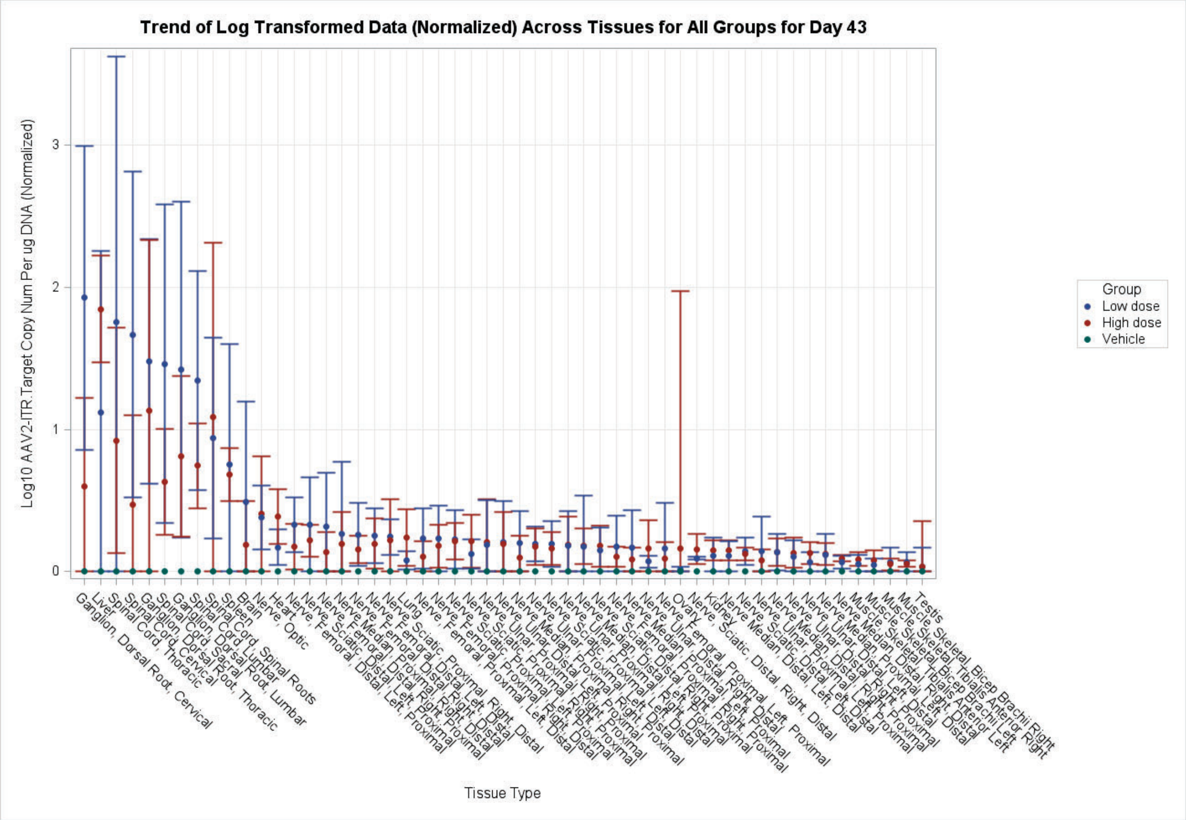

Note: Error bars for the low dose and high dose groups are calculated based on the 95% confidence intervals for the mean of each of these groups. The error bars have been omitted for the vehicle group.

Figures 12 shows the changes in mean log10 AAV2-ITR target copies/ug of DNA for normalized data by group across the different tissues for day 85.

Figure 12

Assessment of Trends in Mean Log10 AAV2-ITR.Target Copy Num Per ug DNA (Normalized) by Group Across Tissues for Day 85

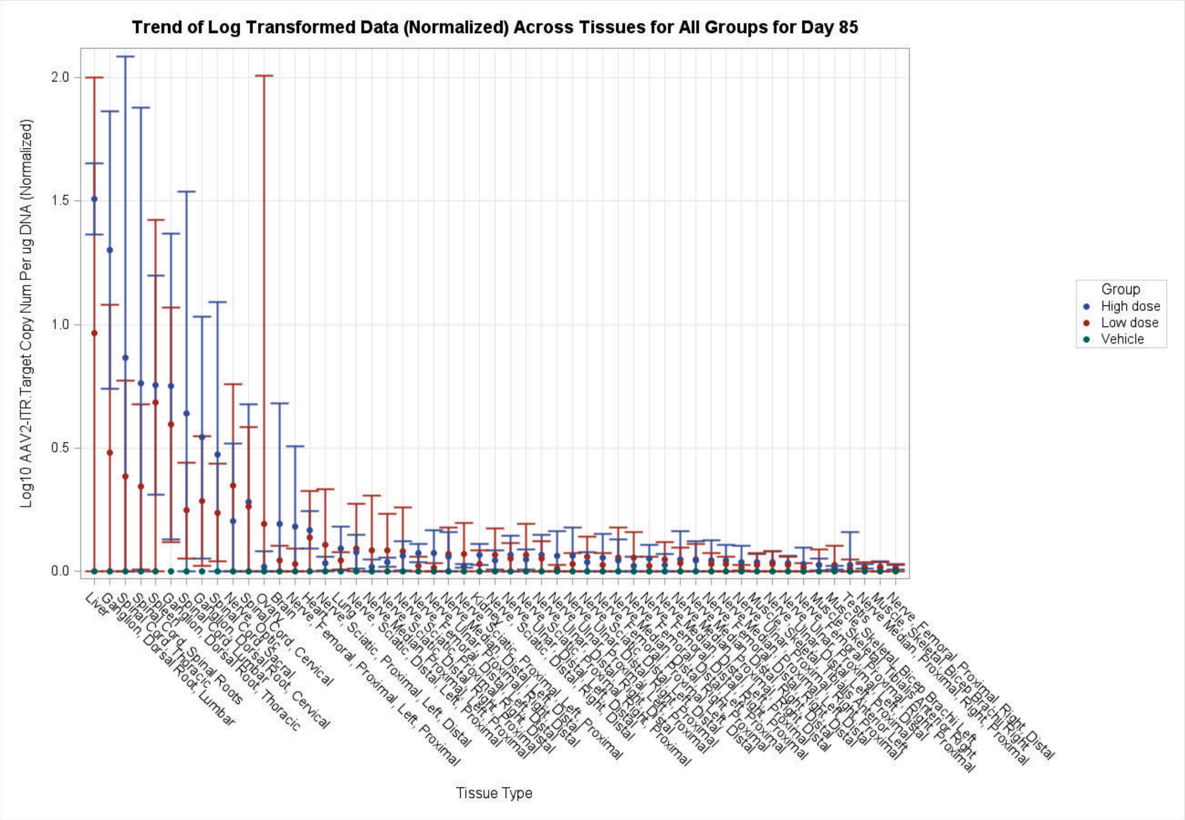

Note: Error bars for the low dose and high dose groups are calculated based on the 95% confidence intervals for the mean of each of these groups. The error bars have been omitted for the vehicle group.

These figures show that the confidence intervals for some tissues across the groups and time points are substantially wider than the typical patterns observed in the data. The primary reason for observing these wide confidence intervals the small sample size and higher levels of variations in the data, as determined by standard deviation, for the data used to create those CIs.

The lower the sample size is, and the higher the standard deviation of the data is, the wider the width of the confidence interval will be. For the specific case of the Ovary tissue, wider CIs can be seen for the High Dose category for both normalized and not-normalized data for Day 43 and Day 85. The reason for observing these wide confidence intervals is that the sample size for the High Dose category for this tissue for each time point is low ($n = 2$) and the standard deviation for this category is highest among the groups with only two samples for both Day 43 and Day 85.

Summary of the Results of Mixed-Effects Modeling for each Category of Group, Timepoint, Gender, and Tissue

Data	Effect	Category	Estimate	Standard_Error	DF	t_Value	Lower_CI	Upper_CI
Not Normalized	Group	High Dose	5.06532	0.16046	17.00000	31.56816	4.72679	5.40385
Not Normalized	Group	Low Dose	4.94791	0.16046	17.00000	30.83640	4.60937	5.28644
Not Normalized	Group	Vehicle	0.18119	0.22692	17.00000	0.79847	-0.29757	0.65995
Not Normalized	Timepoint	Day 43	4.33045	0.65149	18.00000	6.64703	2.96173	5.69917
Not Normalized	Timepoint	Day 85	3.75261	0.65149	18.00000	5.76007	2.38389	5.12133
Not Normalized	Gender	Female	4.00120	0.65843	18.00000	6.07690	2.61790	5.38451
Not Normalized	Gender	Male	4.08185	0.65843	18.00000	6.19938	2.69854	5.46516
Not Normalized	Tissue	Brain	4.12379	0.47025	22.00822	8.76940	3.14858	5.09900
Not Normalized	Tissue	Ganglion, Dorsal Root, Cervical	5.02111	0.47025	22.00822	10.67759	4.04590	5.99633
Not Normalized	Tissue	Ganglion, Dorsal Root, Lumbar	5.22216	0.47025	22.00822	11.10511	4.24694	6.19737
Not Normalized	Tissue	Ganglion, Dorsal Root, Thoracic	5.09377	0.47025	22.00822	10.83209	4.11855	6.06898
Not Normalized	Tissue	Heart	4.20764	0.47025	22.00822	8.94770	3.23242	5.18285
Not Normalized	Tissue	Kidney	3.70915	0.47025	22.00822	7.88764	2.73393	4.68436
Not Normalized	Tissue	Lung	3.89720	0.47025	22.00822	8.28755	2.92199	4.87242
Not Normalized	Tissue	Liver	5.41710	0.47025	22.00822	11.51967	4.44189	6.39232
Not Normalized	Tissue	Muscle, Skeletal, Tibialis Anterior Left	3.73673	0.47025	22.00822	7.94629	2.76151	4.71194
Not Normalized	Tissue	Muscle, Skeletal, Tibialis Anterior Right	3.64355	0.47025	22.00822	7.74815	2.66834	4.61876
Not Normalized	Tissue	Muscle, Skeletal, Bicep Brachii Left	3.58095	0.47025	22.00822	7.61502	2.60573	4.55616
Not Normalized	Tissue	Muscle, Skeletal, Bicep Brachii Right	3.47753	0.47025	22.00822	7.39509	2.50231	4.45274
Not Normalized	Tissue	Nerve, Femoral, Distal, Left, Distal	3.91169	0.47025	22.00822	8.31837	2.93648	4.88691
Not Normalized	Tissue	Nerve, Femoral, Distal, Left, Proximal	3.96999	0.47025	22.00822	8.44233	2.99477	4.94520
Not Normalized	Tissue	Nerve, Femoral, Distal, Right, Distal	3.81710	0.47025	22.00822	8.11721	2.84189	4.79231
Not Normalized	Tissue	Nerve, Femoral, Distal, Right, Proximal	3.79223	0.47025	22.00822	8.06432	2.81702	4.76744
Not Normalized	Tissue	Nerve, Femoral, Proximal, Left, Distal	3.71101	0.47025	22.00822	7.89161	2.73580	4.68623
Not Normalized	Tissue	Nerve, Femoral, Proximal, Left, Proximal	3.72387	0.47025	22.00822	7.91895	2.74866	4.69908
Not Normalized	Tissue	Nerve, Femoral, Proximal, Right, Distal	3.90985	0.47025	22.00822	8.31444	2.93464	4.88506
Not Normalized	Tissue	Nerve, Femoral, Proximal, Right, Proximal	3.61064	0.47025	22.00822	7.67817	2.63543	4.58586
Not Normalized	Tissue	Nerve Median, Distal, Left, Distal	3.79464	0.47025	22.00822	8.06945	2.81943	4.76985
Not Normalized	Tissue	Nerve Median, Distal, Left, Proximal	3.74120	0.47025	22.00822	7.95581	2.76599	4.71642
Not Normalized	Tissue	Nerve Median, Distal, Right, Distal	3.65581	0.47025	22.00822	7.77422	2.68060	4.63102
Not Normalized	Tissue	Nerve Median, Distal, Right, Proximal	3.76160	0.47025	22.00822	7.99918	2.78638	4.73681
Not Normalized	Tissue	Nerve Median, Proximal, Left, Distal	3.67401	0.47025	22.00822	7.81291	2.69879	4.64922
Not Normalized	Tissue	Nerve Median, Proximal, Left, Proximal	3.75304	0.47025	22.00822	7.98097	2.77782	4.72825
Not Normalized	Tissue	Nerve Median, Proximal, Right, Distal	3.84651	0.47025	22.00822	8.17976	2.87130	4.82173
Not Normalized	Tissue	Nerve Median, Proximal, Right, Proximal	3.63937	0.47025	22.00822	7.73925	2.66415	4.61458
Not Normalized	Tissue	Nerve, Sciatic, Distal, Left, Distal	3.89675	0.47025	22.00822	8.28659	2.92154	4.87196
Not Normalized	Tissue	Nerve, Sciatic, Distal, Left, Proximal	4.03125	0.47025	22.00822	8.57260	3.05604	5.00646
Not Normalized	Tissue	Nerve, Sciatic, Distal, Right, Distal	3.84655	0.47025	22.00822	8.17984	2.87134	4.82177
Not Normalized	Tissue	Nerve, Sciatic, Distal, Right, Proximal	3.83966	0.47025	22.00822	8.16518	2.86444	4.81487
Not Normalized	Tissue	Nerve, Sciatic, Proximal, Left, Distal	3.86609	0.47025	22.00822	8.22139	2.89088	4.84131
Not Normalized	Tissue	Nerve, Sciatic, Proximal, Left, Proximal	3.88599	0.47025	22.00822	8.26370	2.91077	4.86120
Not Normalized	Tissue	Nerve, Sciatic, Proximal, Right, Distal	4.07058	0.47025	22.00822	8.65624	3.09536	5.04579
Not Normalized	Tissue	Nerve, Sciatic, Proximal, Right, Proximal	3.94508	0.47025	22.00822	8.38935	2.96986	4.92029
Not Normalized	Tissue	Nerve, Ulnar, Distal, Left, Distal	3.80833	0.47025	22.00822	8.09856	2.83312	4.78354
Not Normalized	Tissue	Nerve, Ulnar, Distal, Left, Proximal	3.75734	0.47025	22.00822	7.99013	2.78213	4.73255
Not Normalized	Tissue	Nerve, Ulnar, Distal, Right, Distal	3.75934	0.47025	22.00822	7.99439	2.78413	4.73456
Not Normalized	Tissue	Nerve, Ulnar, Distal, Right, Proximal	3.70478	0.47025	22.00822	7.87837	2.72957	4.68000
Not Normalized	Tissue	Nerve, Ulnar, Proximal, Left, Distal	3.80047	0.47025	22.00822	8.08184	2.82525	4.77568
Not Normalized	Tissue	Nerve, Ulnar, Proximal, Left, Proximal	3.57336	0.47025	22.00822	7.59889	2.59815	4.54858
Not Normalized	Tissue	Nerve, Ulnar, Proximal, Right, Distal	3.77705	0.47025	22.00822	8.03205	2.80184	4.75227
Not Normalized	Tissue	Nerve, Ulnar, Proximal, Right, Proximal	3.74194	0.47025	22.00822	7.95737	2.76672	4.71715
Not Normalized	Tissue	Nerve, Optic	4.37976	0.47025	22.00822	9.31373	3.40455	5.35498
Not Normalized	Tissue	Ovary	3.69762	0.48727	25.36305	7.58843	2.69480	4.70045
Not Normalized	Tissue	Spinal Cord, Cervical	4.84425	0.47025	22.00822	10.30148	3.86904	5.81946
Not Normalized	Tissue	Spinal Cord, Lumbar	4.84082	0.47025	22.00822	10.29420	3.86561	5.81604
Not Normalized	Tissue	Spinal Cord, Sacral	4.82134	0.47025	22.00822	10.25277	3.84613	5.79656
Not Normalized	Tissue	Spinal Cord, Spinal Roots	4.87566	0.47025	22.00822	10.36828	3.90045	5.85088
Not Normalized	Tissue	Spinal Cord, Thoracic	5.00690	0.47025	22.00822	10.64737	4.03169	5.98212
Not Normalized	Tissue	Spleen	5.08593	0.47025	22.00822	10.81543	4.11072	6.06115
Not Normalized	Tissue	Testis	3.41635	0.48727	25.36305	7.01120	2.41353	4.41918
Normalized	Group	High Dose	0.25010	0.03957	17.00000	6.32088	0.16662	0.33358
Normalized	Group	Low Dose	0.26933	0.03957	17.00000	6.80673	0.18585	0.35281
Normalized	Group	Vehicle	0.00002	0.05596	17.00000	0.00031	-0.11804	0.11808
Normalized	Timepoint	Day 43	0.28360	0.04185	18.00000	6.77709	0.19569	0.37152
Normalized	Timepoint	Day 85	0.13195	0.04185	18.00000	3.15304	0.04403	0.21986
Normalized	Gender	Female	0.21459	0.04884	18.00000	4.39409	0.11199	0.31719
Normalized	Gender	Male	0.20096	0.04884	18.00000	4.11506	0.09836	0.30356
Normalized	Tissue	Brain	0.18279	0.06982	290.12883	2.61810	0.04538	0.32021
Normalized	Tissue	Ganglion, Dorsal Root, Cervical	0.67128	0.06982	290.12883	9.61457	0.53387	0.80870

Lower_CI and Upper_CI represent the lower and upper bounds of the 95% confidence interval for the estimated mean, respectively.

Summary of the Results of Mixed-Effects Modeling for each Category of Group, Timepoint, Gender, and Tissue

Data	Effect	Category	Estimate	Standard_Error	DF	t_Value	Lower_CI	Upper_CI
Normalized	Tissue	Ganglion, Dorsal Root, Lumbar	0.80367	0.06982	290.12883	11.51076	0.66626	0.94109
Normalized	Tissue	Ganglion, Dorsal Root, Thoracic	0.79108	0.06982	290.12883	11.33039	0.65366	0.92850
Normalized	Tissue	Heart	0.17270	0.06982	290.12883	2.47355	0.03528	0.31012
Normalized	Tissue	Kidney	0.07197	0.06982	290.12883	1.03083	-0.06544	0.20939
Normalized	Tissue	Lung	0.09127	0.06982	290.12883	1.30719	-0.04615	0.22868
Normalized	Tissue	Liver	1.08815	0.06982	290.12883	15.58526	0.95073	1.22557
Normalized	Tissue	Muscle, Skeletal, Tibialis Anterior Left	0.04180	0.06982	290.12883	0.59873	-0.09561	0.17922
Normalized	Tissue	Muscle, Skeletal, Tibialis Anterior Right	0.03493	0.06982	290.12883	0.50023	-0.10249	0.17234
Normalized	Tissue	Muscle, Skeletal, Bicep Brachii Left	0.03318	0.06982	290.12883	0.47518	-0.10424	0.17059
Normalized	Tissue	Muscle, Skeletal, Bicep Brachii Right	0.03085	0.06982	290.12883	0.44189	-0.10656	0.16827
Normalized	Tissue	Nerve, Femoral, Distal, Left, Distal	0.10939	0.06982	290.12883	1.56682	-0.02802	0.24681
Normalized	Tissue	Nerve, Femoral, Distal, Left, Proximal	0.11720	0.06982	290.12883	1.67866	-0.02021	0.25462
Normalized	Tissue	Nerve, Femoral, Distal, Right, Distal	0.09940	0.06982	290.12883	1.42374	-0.03801	0.23682
Normalized	Tissue	Nerve, Femoral, Distal, Right, Proximal	0.10552	0.06982	290.12883	1.51137	-0.03189	0.24294
Normalized	Tissue	Nerve, Femoral, Proximal, Left, Distal	0.08584	0.06982	290.12883	1.22946	-0.05158	0.22326
Normalized	Tissue	Nerve, Femoral, Proximal, Left, Proximal	0.09362	0.06982	290.12883	1.34093	-0.04379	0.23104
Normalized	Tissue	Nerve, Femoral, Proximal, Right, Distal	0.08863	0.06982	290.12883	1.26941	-0.04879	0.22605
Normalized	Tissue	Nerve, Femoral, Proximal, Right, Proximal	0.06608	0.06982	290.12883	0.94644	-0.07134	0.20350
Normalized	Tissue	Nerve Median, Distal, Left, Distal	0.07802	0.06982	290.12883	1.11740	-0.05940	0.21543
Normalized	Tissue	Nerve Median, Distal, Left, Proximal	0.08682	0.06982	290.12883	1.24351	-0.05060	0.22424
Normalized	Tissue	Nerve Median, Distal, Right, Distal	0.05065	0.06982	290.12883	0.72548	-0.08676	0.18807
Normalized	Tissue	Nerve Median, Distal, Right, Proximal	0.07549	0.06982	290.12883	1.08123	-0.06193	0.21291
Normalized	Tissue	Nerve Median, Proximal, Left, Distal	0.06356	0.06982	290.12883	0.91031	-0.07386	0.20097
Normalized	Tissue	Nerve Median, Proximal, Left, Proximal	0.07318	0.06982	290.12883	1.04810	-0.06424	0.21059
Normalized	Tissue	Nerve Median, Proximal, Right, Distal	0.10947	0.06982	290.12883	1.56789	-0.02795	0.24689
Normalized	Tissue	Nerve Median, Proximal, Right, Proximal	0.06750	0.06982	290.12883	0.96680	-0.06992	0.20492
Normalized	Tissue	Nerve, Sciatic, Distal, Left, Distal	0.07310	0.06982	290.12883	1.04705	-0.06431	0.21052
Normalized	Tissue	Nerve, Sciatic, Distal, Left, Proximal	0.14412	0.06982	290.12883	2.06417	0.00670	0.28154
Normalized	Tissue	Nerve, Sciatic, Distal, Right, Distal	0.07382	0.06982	290.12883	1.05734	-0.06359	0.21124
Normalized	Tissue	Nerve, Sciatic, Distal, Right, Proximal	0.09065	0.06982	290.12883	1.29837	-0.04677	0.22807
Normalized	Tissue	Nerve, Sciatic, Proximal, Left, Distal	0.09890	0.06982	290.12883	1.41656	-0.03851	0.23632
Normalized	Tissue	Nerve, Sciatic, Proximal, Left, Proximal	0.10692	0.06982	290.12883	1.53139	-0.03050	0.24434
Normalized	Tissue	Nerve, Sciatic, Proximal, Right, Distal	0.12161	0.06982	290.12883	1.74181	-0.01580	0.25903
Normalized	Tissue	Nerve, Sciatic, Proximal, Right, Proximal	0.09141	0.06982	290.12883	1.30930	-0.04600	0.22883
Normalized	Tissue	Nerve, Ulnar, Distal, Left, Distal	0.06309	0.06982	290.12883	0.90357	-0.07433	0.20050
Normalized	Tissue	Nerve, Ulnar, Distal, Left, Proximal	0.09339	0.06982	290.12883	1.33765	-0.04402	0.23081
Normalized	Tissue	Nerve, Ulnar, Distal, Right, Distal	0.07077	0.06982	290.12883	1.01357	-0.06665	0.20818
Normalized	Tissue	Nerve, Ulnar, Distal, Right, Proximal	0.06697	0.06982	290.12883	0.95924	-0.07044	0.20439
Normalized	Tissue	Nerve, Ulnar, Proximal, Left, Distal	0.08614	0.06982	290.12883	1.23375	-0.05128	0.22356
Normalized	Tissue	Nerve, Ulnar, Proximal, Left, Proximal	0.05876	0.06982	290.12883	0.84166	-0.07865	0.19618
Normalized	Tissue	Nerve, Ulnar, Proximal, Right, Distal	0.09211	0.06982	290.12883	1.31920	-0.04531	0.22952
Normalized	Tissue	Nerve, Ulnar, Proximal, Right, Proximal	0.08981	0.06982	290.12883	1.28638	-0.04760	0.22723
Normalized	Tissue	Nerve, Optic	0.26805	0.06982	290.12883	3.83914	0.13063	0.40546
Normalized	Tissue	Ovary	0.07234	0.09360	599.30018	0.77288	-0.11148	0.25616
Normalized	Tissue	Spinal Cord, Cervical	0.53696	0.06982	290.12883	7.69076	0.39955	0.67438
Normalized	Tissue	Spinal Cord, Lumbar	0.59602	0.06982	290.12883	8.53659	0.45860	0.73344
Normalized	Tissue	Spinal Cord, Sacral	0.56032	0.06982	290.12883	8.02523	0.42290	0.69773
Normalized	Tissue	Spinal Cord, Spinal Roots	0.62642	0.06982	290.12883	8.97201	0.48900	0.76384
Normalized	Tissue	Spinal Cord, Thoracic	0.78644	0.06982	290.12883	11.26387	0.64902	0.92385
Normalized	Tissue	Spleen	0.57409	0.06982	290.12883	8.22245	0.43667	0.71150
Normalized	Tissue	Testis	0.02838	0.09360	599.30018	0.30325	-0.15544	0.21221

Lower_CI and Upper_CI represent the lower and upper bounds of the 95% confidence interval for the estimated mean, respectively.

n, mean, standard deviation lower and upper 95% confidence intervals for Day 85 and Day 43 Across Tissue and Group for Log10 AAV2-ITR Target Copies/ug of DNA for Normalized Data

Tissue	Timepoint	Group	n	Mean	Std	CI_lower	CI_upper
Brain	Day 43	Vehicle	2	0	0		
Brain	Day 43	Low Dose	4	0.487789215	0.443758514	0	1.193908036
Brain	Day 43	High Dose	4	0.188390092	0.192936026	0	0.495394364
Brain	Day 85	Vehicle	2	7.31318E-05	0.000103424		
Brain	Day 85	Low Dose	4	0.045520956	0.038098588	0	0.106144311
Brain	Day 85	High Dose	4	0.192233287	0.306758719	0	0.680354863
Ganglion, Dorsal Root, Cervical	Day 43	Vehicle	2	1.87127E-05	2.64637E-05		
Ganglion, Dorsal Root, Cervical	Day 43	Low Dose	4	1.926524938	0.673465548	0.854890966	2.998158911
Ganglion, Dorsal Root, Cervical	Day 43	High Dose	4	0.600284343	0.392095095	0	1.224195137
Ganglion, Dorsal Root, Cervical	Day 85	Vehicle	2	0	0		
Ganglion, Dorsal Root, Cervical	Day 85	Low Dose	4	0.2862897	0.164501629	0.0245309	0.548048501
Ganglion, Dorsal Root, Cervical	Day 85	High Dose	4	0.543302956	0.30818893	0.052905595	1.033700317
Ganglion, Dorsal Root, Lumbar	Day 43	Vehicle	2	0	0		
Ganglion, Dorsal Root, Lumbar	Day 43	Low Dose	4	1.422008987	0.744382433	0.237530426	2.606487549
Ganglion, Dorsal Root, Lumbar	Day 43	High Dose	4	0.812834619	0.354539893	0.248682533	1.376986705
Ganglion, Dorsal Root, Lumbar	Day 85	Vehicle	2	0.00010063	0.000142312		
Ganglion, Dorsal Root, Lumbar	Day 85	Low Dose	4	0.48201424	0.375781319	0	1.079966175
Ganglion, Dorsal Root, Lumbar	Day 85	High Dose	4	1.301455389	0.352189344	0.741043551	1.861867228
Ganglion, Dorsal Root, Thoracic	Day 43	Vehicle	2	0	0		
Ganglion, Dorsal Root, Thoracic	Day 43	Low Dose	4	1.477475812	0.540757892	0.617009333	2.33794229
Ganglion, Dorsal Root, Thoracic	Day 43	High Dose	4	1.132740428	0.755033259	0	2.334166831
Ganglion, Dorsal Root, Thoracic	Day 85	Vehicle	2	0	0		
Ganglion, Dorsal Root, Thoracic	Day 85	Low Dose	4	0.594738481	0.298074484	0.120435461	1.069041502
Ganglion, Dorsal Root, Thoracic	Day 85	High Dose	4	0.750444881	0.38866105	0.13199842	1.368891343
Heart	Day 43	Vehicle	2	0	0		
Heart	Day 43	Low Dose	4	0.171031773	0.078734025	0.045748368	0.296315177
Heart	Day 43	High Dose	4	0.388646453	0.120697753	0.196589394	0.580703512
Heart	Day 85	Vehicle	2	0	0		
Heart	Day 85	Low Dose	4	0.136100824	0.118781757	0	0.325109106
Heart	Day 85	High Dose	4	0.167727483	0.047504081	0.092137889	0.243317077
Kidney	Day 43	Vehicle	2	0	0		
Kidney	Day 43	Low Dose	4	0.110026565	0.081992269	0	0.240494562
Kidney	Day 43	High Dose	4	0.151448674	0.044724417	0.080282145	0.222615202
Kidney	Day 85	Vehicle	2	0	0		
Kidney	Day 85	Low Dose	4	0.029183682	0.035779329	0	0.086116578
Kidney	Day 85	High Dose	4	0.06919934	0.026740978	0.026648477	0.111750203
Liver	Day 43	Vehicle	2	0	0		
Liver	Day 43	Low Dose	4	1.11816025	0.715195557	0	2.256195978
Liver	Day 43	High Dose	4	1.848200233	0.237219532	1.470731021	2.225669446
Liver	Day 85	Vehicle	2	0.000233511	0.000330234		
Liver	Day 85	Low Dose	4	0.964636747	0.651279537	0	2.000967825
Liver	Day 85	High Dose	4	1.509642311	0.090783537	1.365185446	1.654099177
Lung	Day 43	Vehicle	2	0	0		
Lung	Day 43	Low Dose	4	0.079005403	0.042033552	0.012120643	0.145890164
Lung	Day 43	High Dose	4	0.239610895	0.123276547	0.0434504	0.435771391
Lung	Day 85	Vehicle	2	0	0		
Lung	Day 85	Low Dose	4	0.044491879	0.021647497	0.010045881	0.078937876
Lung	Day 85	High Dose	4	0.09322732	0.056473956	0.003364654	0.183089986
Muscle, Skeletal, Bicep Brachii Left	Day 43	Vehicle	2	0.000143992	0.000203636		
Muscle, Skeletal, Bicep Brachii Left	Day 43	Low Dose	4	0.044060319	0.031022319	0	0.093423751
Muscle, Skeletal, Bicep Brachii Left	Day 43	High Dose	4	0.076755052	0.047395324	0.001338514	0.152171589
Muscle, Skeletal, Bicep Brachii Left	Day 85	Vehicle	2	0	0		
Muscle, Skeletal, Bicep Brachii Left	Day 85	Low Dose	4	0.028588007	0.047999203	0	0.10496545
Muscle, Skeletal, Bicep Brachii Left	Day 85	High Dose	4	0.016408759	0.005152727	0.00820962	0.024607898
Muscle, Skeletal, Bicep Brachii Right	Day 43	Vehicle	2	0	0		
Muscle, Skeletal, Bicep Brachii Right	Day 43	Low Dose	4	0.063358264	0.045098894	0	0.135120668
Muscle, Skeletal, Bicep Brachii Right	Day 43	High Dose	4	0.055253241	0.013488147	0.03379059	0.076715893
Muscle, Skeletal, Bicep Brachii Right	Day 85	Vehicle	2	0	0		
Muscle, Skeletal, Bicep Brachii Right	Day 85	Low Dose	4	0.014437859	0.015186369	0	0.038602761
Muscle, Skeletal, Bicep Brachii Right	Day 85	High Dose	4	0.021213602	0.011778318	0.00247167	0.039955534
Muscle, Skeletal, Tibialis Anterior Left	Day 43	Vehicle	2	0	0		
Muscle, Skeletal, Tibialis Anterior Left	Day 43	Low Dose	4	0.054784514	0.038841934	0	0.116590698

n, mean, standard deviation lower and upper 95% confidence intervals for Day 85 and Day 43 Across Tissue and Group for Log10 AAV2-ITR Target Copies/ug of DNA for Normalized Data

Tissue	Timepoint	Group	n	Mean	Std	CI_lower	CI_upper
Muscle, Skeletal, Tibialis Anterior Left	Day 43	High Dose	4	0.088112175	0.030280808	0.039928653	0.136295698
Muscle, Skeletal, Tibialis Anterior Left	Day 85	Vehicle	2	5.54296E-05	7.83893E-05		
Muscle, Skeletal, Tibialis Anterior Left	Day 85	Low Dose	4	0.028582426	0.029180638	0	0.075015333
Muscle, Skeletal, Tibialis Anterior Left	Day 85	High Dose	4	0.037508212	0.022074769	0.002382328	0.072634096
Muscle, Skeletal, Tibialis Anterior Right	Day 43	Vehicle	2	6.21787E-05	8.7934E-05		
Muscle, Skeletal, Tibialis Anterior Right	Day 43	Low Dose	4	0.066649299	0.064803581	0	0.169766257
Muscle, Skeletal, Tibialis Anterior Right	Day 43	High Dose	4	0.050630069	0.024859734	0.011072685	0.090187454
Muscle, Skeletal, Tibialis Anterior Right	Day 85	Vehicle	2	0	0		
Muscle, Skeletal, Tibialis Anterior Right	Day 85	Low Dose	4	0.028669178	0.038775446	0	0.090369565
Muscle, Skeletal, Tibialis Anterior Right	Day 85	High Dose	4	0.0286473	0.015224149	0.004422282	0.052872318
Nerve Median, Distal, Left, Distal	Day 43	Vehicle	2	0	0		
Nerve Median, Distal, Left, Distal	Day 43	Low Dose	4	0.108211641	0.066231269	0.002822913	0.213600369
Nerve Median, Distal, Left, Distal	Day 43	High Dose	4	0.15025685	0.043851494	0.080479338	0.220034362
Nerve Median, Distal, Left, Distal	Day 85	Vehicle	2	0	0		
Nerve Median, Distal, Left, Distal	Day 85	Low Dose	4	0.071527571	0.066989802	0	0.178123295
Nerve Median, Distal, Left, Distal	Day 85	High Dose	4	0.060082561	0.063638332	0	0.161345348
Nerve Median, Distal, Left, Proximal	Day 43	Vehicle	2	0	0		
Nerve Median, Distal, Left, Proximal	Day 43	Low Dose	4	0.176302944	0.225135549	0	0.534543841
Nerve Median, Distal, Left, Proximal	Day 43	High Dose	4	0.180082748	0.078463081	0.055230477	0.304935019
Nerve Median, Distal, Left, Proximal	Day 85	Vehicle	2	0	0		
Nerve Median, Distal, Left, Proximal	Day 85	Low Dose	4	0.050202496	0.044240613	0	0.120599183
Nerve Median, Distal, Left, Proximal	Day 85	High Dose	4	0.027517698	0.026548707	0	0.069762614
Nerve Median, Distal, Right, Distal	Day 43	Vehicle	2	0	0		
Nerve Median, Distal, Right, Distal	Day 43	Low Dose	4	0.068140329	0.028021442	0.023551962	0.112728696
Nerve Median, Distal, Right, Distal	Day 43	High Dose	4	0.092305053	0.014249958	0.06963019	0.114979916
Nerve Median, Distal, Right, Distal	Day 85	Vehicle	2	0	0		
Nerve Median, Distal, Right, Distal	Day 85	Low Dose	4	0.048668832	0.040407397	0	0.112966018
Nerve Median, Distal, Right, Distal	Day 85	High Dose	4	0.044148268	0.049165223	0	0.122381109
Nerve Median, Distal, Right, Proximal	Day 43	Vehicle	2	0	0		
Nerve Median, Distal, Right, Proximal	Day 43	Low Dose	4	0.136266964	0.0811268	0.007176122	0.265357806
Nerve Median, Distal, Right, Proximal	Day 43	High Dose	4	0.139120437	0.060364684	0.043066754	0.235174121
Nerve Median, Distal, Right, Proximal	Day 85	Vehicle	2	0	0		
Nerve Median, Distal, Right, Proximal	Day 85	Low Dose	4	0.057465583	0.075713693	0	0.177942964
Nerve Median, Distal, Right, Proximal	Day 85	High Dose	4	0.04460095	0.054563822	0	0.131424167
Nerve Median, Proximal, Left, Distal	Day 43	Vehicle	2	0	0		
Nerve Median, Proximal, Left, Distal	Day 43	Low Dose	4	0.118220231	0.091476778	0	0.263780198
Nerve Median, Proximal, Left, Distal	Day 43	High Dose	4	0.123549249	0.049357904	0.045009811	0.202088688
Nerve Median, Proximal, Left, Distal	Day 85	Vehicle	2	0	0		
Nerve Median, Proximal, Left, Distal	Day 85	Low Dose	4	0.030687167	0.019696215	0	0.06202824
Nerve Median, Proximal, Left, Distal	Day 85	High Dose	4	0.04533082	0.038967743	0	0.107337195
Nerve Median, Proximal, Left, Proximal	Day 43	Vehicle	2	0	0		
Nerve Median, Proximal, Left, Proximal	Day 43	Low Dose	4	0.171616999	0.162734257	0	0.430563516
Nerve Median, Proximal, Left, Proximal	Day 43	High Dose	4	0.087267013	0.051672671	0.005044262	0.169489764
Nerve Median, Proximal, Left, Proximal	Day 85	Vehicle	2	4.58328E-05	6.48174E-05		
Nerve Median, Proximal, Left, Proximal	Day 85	Low Dose	4	0.085611134	0.138750986	0	0.306394916
Nerve Median, Proximal, Left, Proximal	Day 85	High Dose	4	0.021369643	0.017694857	0	0.049526109
Nerve Median, Proximal, Right, Distal	Day 43	Vehicle	2	0	0		
Nerve Median, Proximal, Right, Distal	Day 43	Low Dose	4	0.265498808	0.317547788	0	0.770788201
Nerve Median, Proximal, Right, Distal	Day 43	High Dose	4	0.19712309	0.138721893	0	0.417860578
Nerve Median, Proximal, Right, Distal	Day 85	Vehicle	2	0	0		
Nerve Median, Proximal, Right, Distal	Day 85	Low Dose	4	0.035930539	0.037378163	0	0.095407538
Nerve Median, Proximal, Right, Distal	Day 85	High Dose	4	0.048792097	0.071209094	0	0.162101656
Nerve Median, Proximal, Right, Proximal	Day 43	Vehicle	2	0	0		
Nerve Median, Proximal, Right, Proximal	Day 43	Low Dose	4	0.200075529	0.14032175	0	0.423358745
Nerve Median, Proximal, Right, Proximal	Day 43	High Dose	4	0.099889027	0.095338421	0	0.251593731
Nerve Median, Proximal, Right, Proximal	Day 85	Vehicle	2	0	0		
Nerve Median, Proximal, Right, Proximal	Day 85	Low Dose	4	0.01618857	0.013095419	0	0.037026304
Nerve Median, Proximal, Right, Proximal	Day 85	High Dose	4	0.021353627	0.005364156	0.012818058	0.029889195
Nerve, Femoral, Distal, Left, Distal	Day 43	Vehicle	2	0	0		
Nerve, Femoral, Distal, Left, Distal	Day 43	Low Dose	4	0.250769907	0.122027817	0.05659642	0.444943395
Nerve, Femoral, Distal, Left, Distal	Day 43	High Dose	4	0.197353401	0.110061026	0.022221748	0.372485053
Nerve, Femoral, Distal, Left, Distal	Day 85	Vehicle	2	0	0		

n, mean, standard deviation lower and upper 95% confidence intervals for Day 85 and Day 43 Across Tissue and Group for Log10 AAV2-ITR Target Copies/ug of DNA for Normalized Data

Tissue	Timepoint	Group	n	Mean	Std	CI_lower	CI_upper
Nerve, Femoral, Distal, Left, Distal	Day 85	Low Dose	4	0.023783413	0.02401655	0	0.061999103
Nerve, Femoral, Distal, Left, Distal	Day 85	High Dose	4	0.075064235	0.022346712	0.039505629	0.11062284
Nerve, Femoral, Distal, Left, Proximal	Day 43	Vehicle	2	0	0		
Nerve, Femoral, Distal, Left, Proximal	Day 43	Low Dose	4	0.329938671	0.121547171	0.136529998	0.523347343
Nerve, Femoral, Distal, Left, Proximal	Day 43	High Dose	4	0.177075339	0.101652018	0.015324295	0.338826383
Nerve, Femoral, Distal, Left, Proximal	Day 85	Vehicle	2	4.46328E-05	6.31203E-05		
Nerve, Femoral, Distal, Left, Proximal	Day 85	Low Dose	4	0.055504764	0.065973275	0	0.160482968
Nerve, Femoral, Distal, Left, Proximal	Day 85	High Dose	4	0.023471886	0.02372609	0	0.06122539
Nerve, Femoral, Distal, Right, Distal	Day 43	Vehicle	2	0	0		
Nerve, Femoral, Distal, Right, Distal	Day 43	Low Dose	4	0.261412937	0.140485927	0.037868477	0.484957397
Nerve, Femoral, Distal, Right, Distal	Day 43	High Dose	4	0.15805761	0.06057441	0.061670206	0.254445014
Nerve, Femoral, Distal, Right, Distal	Day 85	Vehicle	2	0	0		
Nerve, Femoral, Distal, Right, Distal	Day 85	Low Dose	4	0.030437875	0.028284863	0	0.075445404
Nerve, Femoral, Distal, Right, Distal	Day 85	High Dose	4	0.047115415	0.050628237	0	0.127676237
Nerve, Femoral, Distal, Right, Proximal	Day 43	Vehicle	2	0	0		
Nerve, Femoral, Distal, Right, Proximal	Day 43	Low Dose	4	0.313628843	0.238529546	0	0.69318258
Nerve, Femoral, Distal, Right, Proximal	Day 43	High Dose	4	0.139573885	0.086124452	0.002530663	0.276617106
Nerve, Femoral, Distal, Right, Proximal	Day 85	Vehicle	2	0	0		
Nerve, Femoral, Distal, Right, Proximal	Day 85	Low Dose	4	0.022507508	0.023393172	0	0.059731265
Nerve, Femoral, Distal, Right, Proximal	Day 85	High Dose	4	0.051902228	0.034444628	0	0.106711318
Nerve, Femoral, Proximal, Left, Distal	Day 43	Vehicle	2	0	0		
Nerve, Femoral, Proximal, Left, Distal	Day 43	Low Dose	4	0.234798448	0.132472398	0.024005301	0.445591595
Nerve, Femoral, Proximal, Left, Distal	Day 43	High Dose	4	0.107313331	0.068244586	0	0.215905696
Nerve, Femoral, Proximal, Left, Distal	Day 85	Vehicle	2	0	0		
Nerve, Femoral, Proximal, Left, Distal	Day 85	Low Dose	4	0.028738196	0.02809094	0	0.07343715
Nerve, Femoral, Proximal, Left, Distal	Day 85	High Dose	4	0.058348978	0.058719276	0	0.15178445
Nerve, Femoral, Proximal, Left, Proximal	Day 43	Vehicle	2	0	0		
Nerve, Femoral, Proximal, Left, Proximal	Day 43	Low Dose	4	0.162944861	0.199663515	0	0.480654069
Nerve, Femoral, Proximal, Left, Proximal	Day 43	High Dose	4	0.092660847	0.070861213	0	0.205416849
Nerve, Femoral, Proximal, Left, Proximal	Day 85	Vehicle	2	0	0		
Nerve, Femoral, Proximal, Left, Proximal	Day 85	Low Dose	4	0.029077367	0.040264648	0	0.093147407
Nerve, Femoral, Proximal, Left, Proximal	Day 85	High Dose	4	0.183429736	0.203905558	0	0.507888981
Nerve, Femoral, Proximal, Right, Distal	Day 43	Vehicle	2	0.00010276	0.000145325		
Nerve, Femoral, Proximal, Right, Distal	Day 43	Low Dose	4	0.233084574	0.147092068	0	0.467140878
Nerve, Femoral, Proximal, Right, Distal	Day 43	High Dose	4	0.178837388	0.09546902	0.026924872	0.330749904
Nerve, Femoral, Proximal, Right, Distal	Day 85	Vehicle	2	8.56071E-05	0.000121067		
Nerve, Femoral, Proximal, Right, Distal	Day 85	Low Dose	4	0.013624519	0.01050369	0	0.030338234
Nerve, Femoral, Proximal, Right, Distal	Day 85	High Dose	4	0.017505924	0.005666774	0.008488822	0.026523027
Nerve, Femoral, Proximal, Right, Proximal	Day 43	Vehicle	2	0	0		
Nerve, Femoral, Proximal, Right, Proximal	Day 43	Low Dose	4	0.175394773	0.137136426	0	0.393609429
Nerve, Femoral, Proximal, Right, Proximal	Day 43	High Dose	4	0.106600368	0.044130423	0.036379017	0.176821719
Nerve, Femoral, Proximal, Right, Proximal	Day 85	Vehicle	2	0	0		
Nerve, Femoral, Proximal, Right, Proximal	Day 85	Low Dose	4	0.016372493	0.010769782	0	0.033509621
Nerve, Femoral, Proximal, Right, Proximal	Day 85	High Dose	4	0.032030967	0.041433745	0	0.097961302
Nerve, Optic	Day 43	Vehicle	2	0	0		
Nerve, Optic	Day 43	Low Dose	4	0.380945243	0.142279939	0.154546109	0.607344376
Nerve, Optic	Day 43	High Dose	4	0.406015195	0.255651831	0	0.812814308
Nerve, Optic	Day 85	Vehicle	2	0	0		
Nerve, Optic	Day 85	Low Dose	4	0.349804997	0.256969451	0	0.758700737
Nerve, Optic	Day 85	High Dose	4	0.203462962	0.197992381	0	0.518513023
Nerve, Sciatic, Distal, Left, Distal	Day 43	Vehicle	2	0	0		
Nerve, Sciatic, Distal, Left, Distal	Day 43	Low Dose	4	0.144124386	0.059209182	0.049909364	0.238339407
Nerve, Sciatic, Distal, Left, Distal	Day 43	High Dose	4	0.123604281	0.03000695	0.075856528	0.171352035
Nerve, Sciatic, Distal, Left, Distal	Day 85	Vehicle	2	0.000108197	0.000153014		
Nerve, Sciatic, Distal, Left, Distal	Day 85	Low Dose	4	0.058394204	0.052134863	0	0.141352405
Nerve, Sciatic, Distal, Left, Distal	Day 85	High Dose	4	0.039345908	0.025877322	0	0.080522503
Nerve, Sciatic, Distal, Left, Proximal	Day 43	Vehicle	2	0	0		
Nerve, Sciatic, Distal, Left, Proximal	Day 43	Low Dose	4	0.329878312	0.208529146	0	0.661694717
Nerve, Sciatic, Distal, Left, Proximal	Day 43	High Dose	4	0.217893319	0.069508001	0.107290578	0.328496059
Nerve, Sciatic, Distal, Left, Proximal	Day 85	Vehicle	2	0	0		
Nerve, Sciatic, Distal, Left, Proximal	Day 85	Low Dose	4	0.092560243	0.114161372	0	0.274216462
Nerve, Sciatic, Distal, Left, Proximal	Day 85	High Dose	4	0.080261057	0.043893512	0.010416684	0.150105429

n, mean, standard deviation lower and upper 95% confidence intervals for Day 85 and Day 43 Across Tissue and Group for Log10 AAV2-ITR Target Copies/ug of DNA for Normalized Data

Tissue	Timepoint	Group	n	Mean	Std	CI_lower	CI_upper
Nerve, Sciatic, Distal, Right, Distal	Day 43	Vehicle	2	0	0		
Nerve, Sciatic, Distal, Right, Distal	Day 43	Low Dose	4	0.094689459	0.007733064	0.082384429	0.106994489
Nerve, Sciatic, Distal, Right, Distal	Day 43	High Dose	4	0.158502022	0.066184929	0.053187031	0.263817013
Nerve, Sciatic, Distal, Right, Distal	Day 85	Vehicle	2	0	0		
Nerve, Sciatic, Distal, Right, Distal	Day 85	Low Dose	4	0.068957235	0.065784275	0	0.173634697
Nerve, Sciatic, Distal, Right, Distal	Day 85	High Dose	4	0.04696616	0.023890435	0.008951147	0.084981174
Nerve, Sciatic, Distal, Right, Proximal	Day 43	Vehicle	2	0	0		
Nerve, Sciatic, Distal, Right, Proximal	Day 43	Low Dose	4	0.14933915	0.103154603	0	0.313481142
Nerve, Sciatic, Distal, Right, Proximal	Day 43	High Dose	4	0.179134069	0.090938677	0.034430341	0.323837798
Nerve, Sciatic, Distal, Right, Proximal	Day 85	Vehicle	2	0	0		
Nerve, Sciatic, Distal, Right, Proximal	Day 85	Low Dose	4	0.085607482	0.092411262	0	0.232654422
Nerve, Sciatic, Distal, Right, Proximal	Day 85	High Dose	4	0.039174964	0.011869517	0.020287914	0.058062014
Nerve, Sciatic, Proximal, Left, Distal	Day 43	Vehicle	2	0	0		
Nerve, Sciatic, Proximal, Left, Distal	Day 43	Low Dose	4	0.193610317	0.100346088	0.033937299	0.353283335
Nerve, Sciatic, Proximal, Left, Distal	Day 43	High Dose	4	0.16088546	0.072697928	0.045206834	0.276564086
Nerve, Sciatic, Proximal, Left, Distal	Day 85	Vehicle	2	0	0		
Nerve, Sciatic, Proximal, Left, Distal	Day 85	Low Dose	4	0.106618768	0.143080699	0	0.334292089
Nerve, Sciatic, Proximal, Left, Distal	Day 85	High Dose	4	0.033399719	0.017896832	0.004921865	0.061877572
Nerve, Sciatic, Proximal, Left, Proximal	Day 43	Vehicle	2	0	0		
Nerve, Sciatic, Proximal, Left, Proximal	Day 43	Low Dose	4	0.226428229	0.129273785	0.02072479	0.432131668
Nerve, Sciatic, Proximal, Left, Proximal	Day 43	High Dose	4	0.213709928	0.082369258	0.082642057	0.344777799
Nerve, Sciatic, Proximal, Left, Proximal	Day 85	Vehicle	2	0	0		
Nerve, Sciatic, Proximal, Left, Proximal	Day 85	Low Dose	4	0.070839986	0.080139972	0	0.198360564
Nerve, Sciatic, Proximal, Left, Proximal	Day 85	High Dose	4	0.023625663	0.004559272	0.016370844	0.030880482
Nerve, Sciatic, Proximal, Right, Distal	Day 43	Vehicle	2	0	0		
Nerve, Sciatic, Proximal, Right, Distal	Day 43	Low Dose	4	0.244125041	0.079455211	0.117694069	0.370556012
Nerve, Sciatic, Proximal, Right, Distal	Day 43	High Dose	4	0.217668551	0.184016039	0	0.510479133
Nerve, Sciatic, Proximal, Right, Distal	Day 85	Vehicle	2	0.000163993	0.000231921		
Nerve, Sciatic, Proximal, Right, Distal	Day 85	Low Dose	4	0.081896293	0.11239568	0	0.260742902
Nerve, Sciatic, Proximal, Right, Distal	Day 85	High Dose	4	0.064287396	0.036910825	0.005554037	0.123020754
Nerve, Sciatic, Proximal, Right, Proximal	Day 43	Vehicle	2	0	0		
Nerve, Sciatic, Proximal, Right, Proximal	Day 43	Low Dose	4	0.126230911	0.064267886	0.023966363	0.228495458
Nerve, Sciatic, Proximal, Right, Proximal	Day 43	High Dose	4	0.21330938	0.118110273	0.02536958	0.401249181
Nerve, Sciatic, Proximal, Right, Proximal	Day 85	Vehicle	2	0.000138488	0.000195851		
Nerve, Sciatic, Proximal, Right, Proximal	Day 85	Low Dose	4	0.067299306	0.077993591	0	0.191404514
Nerve, Sciatic, Proximal, Right, Proximal	Day 85	High Dose	4	0.050163634	0.025666009	0.009323287	0.091003981
Nerve, Ulnar, Distal, Left, Distal	Day 43	Vehicle	2	0	0		
Nerve, Ulnar, Distal, Left, Distal	Day 43	Low Dose	4	0.064073396	0.043884737	0	0.133903806
Nerve, Ulnar, Distal, Left, Distal	Day 43	High Dose	4	0.129661698	0.049502247	0.050892577	0.208430819
Nerve, Ulnar, Distal, Left, Distal	Day 85	Vehicle	2	0	0		
Nerve, Ulnar, Distal, Left, Distal	Day 85	Low Dose	4	0.053913828	0.039504336	0	0.116774043
Nerve, Ulnar, Distal, Left, Distal	Day 85	High Dose	4	0.067784683	0.049690353	0	0.146853124
Nerve, Ulnar, Distal, Left, Proximal	Day 43	Vehicle	2	0	0		
Nerve, Ulnar, Distal, Left, Proximal	Day 43	Low Dose	4	0.205172706	0.18173103	0	0.494347328
Nerve, Ulnar, Distal, Left, Proximal	Day 43	High Dose	4	0.192814637	0.143989935	0	0.421934754
Nerve, Ulnar, Distal, Left, Proximal	Day 85	Vehicle	2	0	0		
Nerve, Ulnar, Distal, Left, Proximal	Day 85	Low Dose	4	0.032379237	0.031261738	0	0.082123638
Nerve, Ulnar, Distal, Left, Proximal	Day 85	High Dose	4	0.036603538	0.029254421	0	0.083153851
Nerve, Ulnar, Distal, Right, Distal	Day 43	Vehicle	2	0	0		
Nerve, Ulnar, Distal, Right, Distal	Day 43	Low Dose	4	0.070290144	0.026243368	0.02853109	0.112049198
Nerve, Ulnar, Distal, Right, Distal	Day 43	High Dose	4	0.163667879	0.123179545	0	0.359674023
Nerve, Ulnar, Distal, Right, Distal	Day 85	Vehicle	2	0	0		
Nerve, Ulnar, Distal, Right, Distal	Day 85	Low Dose	4	0.05398485	0.044514148	0	0.124816793
Nerve, Ulnar, Distal, Right, Distal	Day 85	High Dose	4	0.065889477	0.05169628	0	0.148149795
Nerve, Ulnar, Distal, Right, Proximal	Day 43	Vehicle	2	0	0		
Nerve, Ulnar, Distal, Right, Proximal	Day 43	Low Dose	4	0.106258062	0.070159486	0	0.217897461
Nerve, Ulnar, Distal, Right, Proximal	Day 43	High Dose	4	0.13348293	0.066666461	0.027401713	0.239564146
Nerve, Ulnar, Distal, Right, Proximal	Day 85	Vehicle	2	0	0		
Nerve, Ulnar, Distal, Right, Proximal	Day 85	Low Dose	4	0.032004656	0.027321994	0	0.075480045
Nerve, Ulnar, Distal, Right, Proximal	Day 85	High Dose	4	0.063121634	0.07145457	0	0.176821799
Nerve, Ulnar, Proximal, Left, Distal	Day 43	Vehicle	2	0	0		
Nerve, Ulnar, Proximal, Left, Distal	Day 43	Low Dose	4	0.194975902	0.077105642	0.072283618	0.317668185

n, mean, standard deviation lower and upper 95% confidence intervals for Day 85 and Day 43 Across Tissue and Group for Log10 AAV2-ITR Target Copies/ug of DNA for Normalized Data

Tissue	Timepoint	Group	n	Mean	Std	CI_lower	CI_upper
Nerve, Ulnar, Proximal, Left, Distal	Day 43	High Dose	4	0.174189548	0.080615818	0.045911791	0.302467304
Nerve, Ulnar, Proximal, Left, Distal	Day 85	Vehicle	2	0	0		
Nerve, Ulnar, Proximal, Left, Distal	Day 85	Low Dose	4	0.028397442	0.019365781	0	0.059212721
Nerve, Ulnar, Proximal, Left, Distal	Day 85	High Dose	4	0.033134943	0.020368247	0.000724516	0.06554537
Nerve, Ulnar, Proximal, Left, Proximal	Day 43	Vehicle	2	0	0		
Nerve, Ulnar, Proximal, Left, Proximal	Day 43	Low Dose	4	0.143043776	0.155368618	0	0.390269918
Nerve, Ulnar, Proximal, Left, Proximal	Day 43	High Dose	4	0.076372659	0.052089033	0	0.159257934
Nerve, Ulnar, Proximal, Left, Proximal	Day 85	Vehicle	2	0	0		
Nerve, Ulnar, Proximal, Left, Proximal	Day 85	Low Dose	4	0.010946327	0.009000731	0	0.025268499
Nerve, Ulnar, Proximal, Left, Proximal	Day 85	High Dose	4	0.06345765	0.062162631	0	0.162372269
Nerve, Ulnar, Proximal, Right, Distal	Day 43	Vehicle	2	0	0		
Nerve, Ulnar, Proximal, Right, Distal	Day 43	Low Dose	4	0.179542364	0.156735119	0	0.428942915
Nerve, Ulnar, Proximal, Right, Distal	Day 43	High Dose	4	0.189993683	0.122719554	0	0.385267878
Nerve, Ulnar, Proximal, Right, Distal	Day 85	Vehicle	2	0	0		
Nerve, Ulnar, Proximal, Right, Distal	Day 85	Low Dose	4	0.016824676	0.010345339	0.000362933	0.033286419
Nerve, Ulnar, Proximal, Right, Distal	Day 85	High Dose	4	0.074166443	0.058086719	0	0.166595375
Nerve, Ulnar, Proximal, Right, Proximal	Day 43	Vehicle	2	0	0		
Nerve, Ulnar, Proximal, Right, Proximal	Day 43	Low Dose	4	0.186960312	0.19786673	0	0.501810434
Nerve, Ulnar, Proximal, Right, Proximal	Day 43	High Dose	4	0.206412411	0.191593132	0	0.511279839
Nerve, Ulnar, Proximal, Right, Proximal	Day 85	Vehicle	2	0	0		
Nerve, Ulnar, Proximal, Right, Proximal	Day 85	Low Dose	4	0.017483859	0.007048741	0.006267738	0.028699979
Nerve, Ulnar, Proximal, Right, Proximal	Day 85	High Dose	4	0.038212754	0.040799336	0	0.103133602
Ovary	Day 43	Vehicle	1	0			
Ovary	Day 43	Low Dose	2	0.017159452	0.002182362	0	0.036767194
Ovary	Day 43	High Dose	2	0.160302094	0.201625994	0	1.97183978
Ovary	Day 85	Vehicle	1	0			
Ovary	Day 85	Low Dose	2	0.194590202	0.201812896	0	2.007807133
Ovary	Day 85	High Dose	2	0.019535829	0.0069547	0	0.082021325
Spinal Cord, Cervical	Day 43	Vehicle	2	0.0001082	0.000153018		
Spinal Cord, Cervical	Day 43	Low Dose	4	1.668489707	0.721473006	0.520465156	2.816514257
Spinal Cord, Cervical	Day 43	High Dose	4	0.469933733	0.398005897	0	1.103249932
Spinal Cord, Cervical	Day 85	Vehicle	2	0	0		
Spinal Cord, Cervical	Day 85	Low Dose	4	0.264417935	0.20199229	0	0.585832744
Spinal Cord, Cervical	Day 85	High Dose	4	0.281920814	0.247770967	0	0.676179713
Spinal Cord, Lumbar	Day 43	Vehicle	2	0	0		
Spinal Cord, Lumbar	Day 43	Low Dose	4	1.344585853	0.48581903	0.571539365	2.117632342
Spinal Cord, Lumbar	Day 43	High Dose	4	0.74693007	0.187615019	0.448392707	1.045467433
Spinal Cord, Lumbar	Day 85	Vehicle	2	0	0		
Spinal Cord, Lumbar	Day 85	Low Dose	4	0.247975401	0.121571305	0.054528326	0.441422477
Spinal Cord, Lumbar	Day 85	High Dose	4	0.640601979	0.564228419	0	1.538415303
Spinal Cord, Sacral	Day 43	Vehicle	2	0	0		
Spinal Cord, Sacral	Day 43	Low Dose	4	1.461239961	0.704846405	0.339672042	2.58280788
Spinal Cord, Sacral	Day 43	High Dose	4	0.628973589	0.234490816	0.255846373	1.002100805
Spinal Cord, Sacral	Day 85	Vehicle	2	0	0		
Spinal Cord, Sacral	Day 85	Low Dose	4	0.237978284	0.124105358	0.040498965	0.435457603
Spinal Cord, Sacral	Day 85	High Dose	4	0.473388107	0.387678669	0	1.090271381
Spinal Cord, Spinal Roots	Day 43	Vehicle	2	0	0		
Spinal Cord, Spinal Roots	Day 43	Low Dose	4	0.937619601	0.444150804	0.230876558	1.644362643
Spinal Cord, Spinal Roots	Day 43	High Dose	4	1.08885751	0.769329482	0	2.313032394
Spinal Cord, Spinal Roots	Day 85	Vehicle	2	0	0		
Spinal Cord, Spinal Roots	Day 85	Low Dose	4	0.343283789	0.210415054	0.008466483	0.678101095
Spinal Cord, Spinal Roots	Day 85	High Dose	4	0.762335205	0.700325514	0	1.876709377
Spinal Cord, Thoracic	Day 43	Vehicle	2	0	0		
Spinal Cord, Thoracic	Day 43	Low Dose	4	1.75850518	1.173410772	0	3.625663568
Spinal Cord, Thoracic	Day 43	High Dose	4	0.921501225	0.498398217	0.128438444	1.714564006
Spinal Cord, Thoracic	Day 85	Vehicle	2	0	0		
Spinal Cord, Thoracic	Day 85	Low Dose	4	0.385905335	0.242948467	0	0.772490561
Spinal Cord, Thoracic	Day 85	High Dose	4	0.866264273	0.766199339	0	2.0854584
Spleen	Day 43	Vehicle	2	0.000216745	0.000306524		
Spleen	Day 43	Low Dose	4	0.750999941	0.536010907	0	1.603912906
Spleen	Day 43	High Dose	4	0.681079561	0.11654673	0.495627706	0.866531416
Spleen	Day 85	Vehicle	2	0.000126212	0.000178491		

n, mean, standard deviation lower and upper 95% confidence intervals for Day 85 and Day 43 Across Tissue and Group for Log10 AAV2-ITR Target Copies/ug of DNA for Normalized Data

Tissue	Timepoint	Group	n	Mean	Std	CI_lower	CI_upper
Spleen	Day 85	Low Dose	4	0.684098179	0.464021523	0	1.422459969
Spleen	Day 85	High Dose	4	0.754079832	0.279071104	0.31001543	1.198144234
Testis	Day 43	Vehicle	1	0			
Testis	Day 43	Low Dose	2	0.037075047	0.014640088	0	0.168611021
Testis	Day 43	High Dose	2	0.035932257	0.035197335	0	0.352167763
Testis	Day 85	Vehicle	1	0			
Testis	Day 85	Low Dose	2	0.012106665	0.004131747	0	0.049228939
Testis	Day 85	High Dose	2	0.026925538	0.014952185	0	0.161265592

n, mean, standard deviation lower and upper 95% confidence intervals for Day 85 and Day 43 Across Tissue and Group for Log10 AAV2-ITR Target Copies/ug of DNA for Not Normalized Data

Tissue	Timepoint	Group	n	Mean	Std	CI_lower	CI_upper
Brain	Day 43	Vehicle	2	0	0		
Brain	Day 43	Low Dose	4	5.800901145	0.632889067	4.793833408	6.807968882
Brain	Day 43	High Dose	4	5.128140453	0.744263142	3.94385171	6.312429195
Brain	Day 85	Vehicle	2	1.030782781	1.457746989		
Brain	Day 85	Low Dose	4	4.351001291	0.677133909	3.273530138	5.428472445
Brain	Day 85	High Dose	4	4.823517213	0.944596827	3.320452872	6.326581554
Ganglion, Dorsal Root, Cervical	Day 43	Vehicle	2	0.790177331	1.117479498		
Ganglion, Dorsal Root, Cervical	Day 43	Low Dose	4	7.424509757	0.666860797	6.363385417	8.485634097
Ganglion, Dorsal Root, Cervical	Day 43	High Dose	4	6.040363869	0.525965072	5.203436068	6.877291669
Ganglion, Dorsal Root, Cervical	Day 85	Vehicle	2	0	0		
Ganglion, Dorsal Root, Cervical	Day 85	Low Dose	4	5.445867598	0.30966749	4.953117518	5.938617677
Ganglion, Dorsal Root, Cervical	Day 85	High Dose	4	5.799743016	0.369818915	5.211278597	6.388207436
Ganglion, Dorsal Root, Lumbar	Day 43	Vehicle	2	0	0		
Ganglion, Dorsal Root, Lumbar	Day 43	Low Dose	4	6.889422459	0.791844119	5.629421763	8.149423154
Ganglion, Dorsal Root, Lumbar	Day 43	High Dose	4	6.316865872	0.338150148	5.778793528	6.854938216
Ganglion, Dorsal Root, Lumbar	Day 85	Vehicle	2	1.160114698	1.64064994		
Ganglion, Dorsal Root, Lumbar	Day 85	Low Dose	4	5.570624789	0.890816023	4.153137707	6.98811187
Ganglion, Dorsal Root, Lumbar	Day 85	High Dose	4	6.753813028	0.349201969	6.19815477	7.309471286
Ganglion, Dorsal Root, Thoracic	Day 43	Vehicle	2	0	0		
Ganglion, Dorsal Root, Thoracic	Day 43	Low Dose	4	6.762504084	0.763678584	5.54732104	7.977687128
Ganglion, Dorsal Root, Thoracic	Day 43	High Dose	4	6.666911337	0.743390537	5.484011104	7.849811571
Ganglion, Dorsal Root, Thoracic	Day 85	Vehicle	2	0	0		
Ganglion, Dorsal Root, Thoracic	Day 85	Low Dose	4	5.944734135	0.496745113	5.154301811	6.735166459
Ganglion, Dorsal Root, Thoracic	Day 85	High Dose	4	6.094685984	0.472781384	5.342385299	6.846986669
Heart	Day 43	Vehicle	2	0	0		
Heart	Day 43	Low Dose	4	5.177427157	0.267794176	4.751306864	5.60354745
Heart	Day 43	High Dose	4	5.692050387	0.21825911	5.344751438	6.039349335
Heart	Day 85	Vehicle	2	0	0		
Heart	Day 85	Low Dose	4	4.968198724	0.434256535	4.277199672	5.659197776
Heart	Day 85	High Dose	4	5.200515394	0.11260957	5.021328439	5.379702349
Kidney	Day 43	Vehicle	2	0	0		
Kidney	Day 43	Low Dose	4	4.862856006	0.43590597	4.169232333	5.556479678
Kidney	Day 43	High Dose	4	5.168121068	0.270197129	4.738177139	5.598064996
Kidney	Day 85	Vehicle	2	0	0		
Kidney	Day 85	Low Dose	4	3.93840361	0.798066601	2.668501557	5.208305663
Kidney	Day 85	High Dose	4	4.576357393	0.214372118	4.235243516	4.91747127
Liver	Day 43	Vehicle	2	0	0		
Liver	Day 43	Low Dose	4	6.345759616	1.068441351	4.645631	8.045888231
Liver	Day 43	High Dose	4	7.373545626	0.197493804	7.059288912	7.687802341
Liver	Day 85	Vehicle	2	1.335530854	1.888725847		
Liver	Day 85	Low Dose	4	5.930054356	1.525986797	3.501868833	8.358239878
Liver	Day 85	High Dose	4	6.768386019	0.160609076	6.512821138	7.023950899
Lung	Day 43	Vehicle	2	0	0		
Lung	Day 43	Low Dose	4	4.866539143	0.146936058	4.632731086	5.100347199
Lung	Day 43	High Dose	4	5.382683501	0.138355159	5.16252957	5.602837433
Lung	Day 85	Vehicle	2	0	0		
Lung	Day 85	Low Dose	4	4.506344413	0.222172166	4.152818918	4.859869907
Lung	Day 85	High Dose	4	4.730443347	0.375533599	4.132885589	5.328001104
Muscle, Skeletal, Bicep Brachii Left	Day 43	Vehicle	2	1.200424353	1.6976564		
Muscle, Skeletal, Bicep Brachii Left	Day 43	Low Dose	4	4.556287426	0.244899031	4.166598418	4.945976433
Muscle, Skeletal, Bicep Brachii Left	Day 43	High Dose	4	4.908840279	0.351883573	4.34891499	5.468765567
Muscle, Skeletal, Bicep Brachii Left	Day 85	Vehicle	2	0	0		
Muscle, Skeletal, Bicep Brachii Left	Day 85	Low Dose	4	3.767772598	0.977279221	2.212703274	5.322841921
Muscle, Skeletal, Bicep Brachii Left	Day 85	High Dose	4	4.071624613	0.113466904	3.891073448	4.252175779
Muscle, Skeletal, Bicep Brachii Right	Day 43	Vehicle	2	0	0		
Muscle, Skeletal, Bicep Brachii Right	Day 43	Low Dose	4	4.639681236	0.397107598	4.007794432	5.27156804
Muscle, Skeletal, Bicep Brachii Right	Day 43	High Dose	4	4.749748325	0.094684216	4.599084609	4.900412041
Muscle, Skeletal, Bicep Brachii Right	Day 85	Vehicle	2	0	0		
Muscle, Skeletal, Bicep Brachii Right	Day 85	Low Dose	4	3.880961998	0.488685866	3.103353734	4.658570262
Muscle, Skeletal, Bicep Brachii Right	Day 85	High Dose	4	4.117237972	0.393007963	3.491874603	4.742601341
Muscle, Skeletal, Tibialis Anterior Left	Day 43	Vehicle	2	0	0		
Muscle, Skeletal, Tibialis Anterior Left	Day 43	Low Dose	4	4.599605741	0.334695237	4.067030931	5.132180551
Muscle, Skeletal, Tibialis Anterior Left	Day 43	High Dose	4	4.9987005	0.187828252	4.699823837	5.297577163
Muscle, Skeletal, Tibialis Anterior Left	Day 85	Vehicle	2	0.98927285	1.399043082		
Muscle, Skeletal, Tibialis Anterior Left	Day 85	Low Dose	4	4.193025987	0.527904626	3.353011924	5.033040051

n, mean, standard deviation lower and upper 95% confidence intervals for Day 85 and Day 43 Across Tissue and Group for Log10 AAV2-ITR Target Copies/ug of DNA for Not Normalized Data

Tissue	Timepoint	Group	n	Mean	Std	CI_lower	CI_upper
Muscle, Skeletal, Tibialis Anterior Left	Day 85	High Dose	4	4.397663516	0.348417558	3.843253431	4.9520736
Muscle, Skeletal, Tibialis Anterior Right	Day 43	Vehicle	2	1.065022071	1.506168657		
Muscle, Skeletal, Tibialis Anterior Right	Day 43	Low Dose	4	4.675003184	0.46599902	3.933494754	5.416511613
Muscle, Skeletal, Tibialis Anterior Right	Day 43	High Dose	4	4.678453485	0.284577957	4.22562645	5.131280519
Muscle, Skeletal, Tibialis Anterior Right	Day 85	Vehicle	2	0	0		
Muscle, Skeletal, Tibialis Anterior Right	Day 85	Low Dose	4	4.037507323	0.679082306	2.956935835	5.118078812
Muscle, Skeletal, Tibialis Anterior Right	Day 85	High Dose	4	4.294279019	0.390113587	3.673521247	4.915036791
Nerve Median, Distal, Left, Distal	Day 43	Vehicle	2	0	0		
Nerve Median, Distal, Left, Distal	Day 43	Low Dose	4	4.842361008	0.501455716	4.044433063	5.640288953
Nerve Median, Distal, Left, Distal	Day 43	High Dose	4	5.246863575	0.201606108	4.926063267	5.567663882
Nerve Median, Distal, Left, Distal	Day 85	Vehicle	2	0	0		
Nerve Median, Distal, Left, Distal	Day 85	Low Dose	4	4.335593199	1.15863785	2.491941827	6.17924457
Nerve Median, Distal, Left, Distal	Day 85	High Dose	4	4.548382329	0.530089658	3.704891392	5.391873266
Nerve Median, Distal, Left, Proximal	Day 43	Vehicle	2	0	0		
Nerve Median, Distal, Left, Proximal	Day 43	Low Dose	4	4.917041883	0.781443568	3.673590785	6.160492981
Nerve Median, Distal, Left, Proximal	Day 43	High Dose	4	5.269482922	0.323021867	4.755483048	5.783482795
Nerve Median, Distal, Left, Proximal	Day 85	Vehicle	2	0	0		
Nerve Median, Distal, Left, Proximal	Day 85	Low Dose	4	4.318266232	0.88326284	2.912797952	5.723734513
Nerve Median, Distal, Left, Proximal	Day 85	High Dose	4	4.201229083	0.483924947	3.431196504	4.971261662
Nerve Median, Distal, Right, Distal	Day 43	Vehicle	2	0	0		
Nerve Median, Distal, Right, Distal	Day 43	Low Dose	4	4.747537981	0.179704616	4.461587836	5.033488126
Nerve Median, Distal, Right, Distal	Day 43	High Dose	4	5.001865724	0.101977781	4.839596318	5.164135131
Nerve Median, Distal, Right, Distal	Day 85	Vehicle	2	0	0		
Nerve Median, Distal, Right, Distal	Day 85	Low Dose	4	4.177919157	1.031838419	2.536033975	5.81980434
Nerve Median, Distal, Right, Distal	Day 85	High Dose	4	4.351719941	0.606721314	3.386290939	5.317148943
Nerve Median, Distal, Right, Proximal	Day 43	Vehicle	2	0	0		
Nerve Median, Distal, Right, Proximal	Day 43	Low Dose	4	5.017526025	0.463750669	4.279595223	5.755456827
Nerve Median, Distal, Right, Proximal	Day 43	High Dose	4	5.168666797	0.230147378	4.802450961	5.534882634
Nerve Median, Distal, Right, Proximal	Day 85	Vehicle	2	0	0		
Nerve Median, Distal, Right, Proximal	Day 85	Low Dose	4	4.353813642	0.744660537	3.168892555	5.53873473
Nerve Median, Distal, Right, Proximal	Day 85	High Dose	4	4.267982347	0.643948253	3.243316978	5.292647715
Nerve Median, Proximal, Left, Distal	Day 43	Vehicle	2	0	0		
Nerve Median, Proximal, Left, Distal	Day 43	Low Dose	4	4.874864992	0.592806418	3.931577694	5.818152289
Nerve Median, Proximal, Left, Distal	Day 43	High Dose	4	5.024123817	0.184417957	4.730673694	5.31757394
Nerve Median, Proximal, Left, Distal	Day 85	Vehicle	2	0	0		
Nerve Median, Proximal, Left, Distal	Day 85	Low Dose	4	4.209016527	0.661374091	3.156622761	5.261410294
Nerve Median, Proximal, Left, Distal	Day 85	High Dose	4	4.262028986	0.82430486	2.950376007	5.573681964
Nerve Median, Proximal, Left, Proximal	Day 43	Vehicle	2	0	0		
Nerve Median, Proximal, Left, Proximal	Day 43	Low Dose	4	4.875660741	0.567579911	3.972514446	5.778807036
Nerve Median, Proximal, Left, Proximal	Day 43	High Dose	4	4.89004252	0.381710288	4.282656272	5.497428767
Nerve Median, Proximal, Left, Proximal	Day 85	Vehicle	2	0.938368649	1.327053669		
Nerve Median, Proximal, Left, Proximal	Day 85	Low Dose	4	4.437479032	0.734016382	3.269495171	5.605462893
Nerve Median, Proximal, Left, Proximal	Day 85	High Dose	4	4.092814578	0.444101154	3.386150539	4.799478617
Nerve Median, Proximal, Right, Distal	Day 43	Vehicle	2	0	0		
Nerve Median, Proximal, Right, Distal	Day 43	Low Dose	4	5.261664487	0.693769165	4.157722929	6.365606045
Nerve Median, Proximal, Right, Distal	Day 43	High Dose	4	5.29218327	0.385000297	4.679561884	5.904804656
Nerve Median, Proximal, Right, Distal	Day 85	Vehicle	2	0	0		
Nerve Median, Proximal, Right, Distal	Day 85	Low Dose	4	4.325578051	0.544313572	3.459453692	5.191702409
Nerve Median, Proximal, Right, Distal	Day 85	High Dose	4	4.353147497	0.749657555	3.16027504	5.546019955
Nerve Median, Proximal, Right, Proximal	Day 43	Vehicle	2	0	0		
Nerve Median, Proximal, Right, Proximal	Day 43	Low Dose	4	5.196134838	0.424617322	4.520473925	5.871795751
Nerve Median, Proximal, Right, Proximal	Day 43	High Dose	4	4.831316645	0.426192289	4.153149607	5.509483683
Nerve Median, Proximal, Right, Proximal	Day 85	Vehicle	2	0	0		
Nerve Median, Proximal, Right, Proximal	Day 85	Low Dose	4	3.972868674	0.515331478	3.152861295	4.792876053
Nerve Median, Proximal, Right, Proximal	Day 85	High Dose	4	4.196508045	0.135628701	3.980692515	4.412323574
Nerve, Femoral, Distal, Left, Distal	Day 43	Vehicle	2	0	0		
Nerve, Femoral, Distal, Left, Distal	Day 43	Low Dose	4	5.355280756	0.142805146	5.128045902	5.58251561
Nerve, Femoral, Distal, Left, Distal	Day 43	High Dose	4	5.273286203	0.34560559	4.723350586	5.82322182
Nerve, Femoral, Distal, Left, Distal	Day 85	Vehicle	2	0	0		
Nerve, Femoral, Distal, Left, Distal	Day 85	Low Dose	4	4.135144111	0.519820601	3.307993536	4.962294686
Nerve, Femoral, Distal, Left, Distal	Day 85	High Dose	4	4.79475903	0.107482691	4.623730084	4.965787976
Nerve, Femoral, Distal, Left, Proximal	Day 43	Vehicle	2	0	0		
Nerve, Femoral, Distal, Left, Proximal	Day 43	Low Dose	4	5.581222152	0.247484659	5.187418833	5.975025471
Nerve, Femoral, Distal, Left, Proximal	Day 43	High Dose	4	5.221825038	0.407017892	4.574168745	5.869481331
Nerve, Femoral, Distal, Left, Proximal	Day 85	Vehicle	2	0.984521484	1.392323635		

n, mean, standard deviation lower and upper 95% confidence intervals for Day 85 and Day 43 Across Tissue and Group for Log10 AAV2-ITR Target Copies/ug of DNA for Not Normalized Data

Tissue	Timepoint	Group	n	Mean	Std	CI_lower	CI_upper
Nerve, Femoral, Distal, Left, Proximal	Day 85	Low Dose	4	4.348051542	0.793794438	3.084947454	5.61115563
Nerve, Femoral, Distal, Left, Proximal	Day 85	High Dose	4	4.20657294	0.356906491	3.638655069	4.774490811
Nerve, Femoral, Distal, Right, Distal	Day 43	Vehicle	2	0	0		
Nerve, Femoral, Distal, Right, Distal	Day 43	Low Dose	4	5.334767521	0.377755853	4.733673661	5.93586138
Nerve, Femoral, Distal, Right, Distal	Day 43	High Dose	4	5.188219246	0.244856094	4.798598561	5.577839932
Nerve, Femoral, Distal, Right, Distal	Day 85	Vehicle	2	0	0		
Nerve, Femoral, Distal, Right, Distal	Day 85	Low Dose	4	4.206896623	0.561045734	3.314147662	5.099645585
Nerve, Femoral, Distal, Right, Distal	Day 85	High Dose	4	4.355618657	0.516830291	3.533226332	5.178010983
Nerve, Femoral, Distal, Right, Proximal	Day 43	Vehicle	2	0	0		
Nerve, Femoral, Distal, Right, Proximal	Day 43	Low Dose	4	5.511834297	0.565748677	4.611601904	6.412066691
Nerve, Femoral, Distal, Right, Proximal	Day 43	High Dose	4	5.034660148	0.38392345	4.423752265	5.645568031
Nerve, Femoral, Distal, Right, Proximal	Day 85	Vehicle	2	0	0		
Nerve, Femoral, Distal, Right, Proximal	Day 85	Low Dose	4	3.875392515	0.827632749	2.558444123	5.192340907
Nerve, Femoral, Distal, Right, Proximal	Day 85	High Dose	4	4.539269627	0.387861745	3.922095038	5.156444215
Nerve, Femoral, Proximal, Left, Distal	Day 43	Vehicle	2	0	0		
Nerve, Femoral, Proximal, Left, Distal	Day 43	Low Dose	4	5.339287791	0.367850028	4.75395631	5.924619271
Nerve, Femoral, Proximal, Left, Distal	Day 43	High Dose	4	4.869681443	0.402847586	4.228661038	5.510701848
Nerve, Femoral, Proximal, Left, Distal	Day 85	Vehicle	2	0	0		
Nerve, Femoral, Proximal, Left, Distal	Day 85	Low Dose	4	4.057884136	0.637679483	3.043193779	5.072574493
Nerve, Femoral, Proximal, Left, Distal	Day 85	High Dose	4	4.288204853	0.671002887	3.220489524	5.355920183
Nerve, Femoral, Proximal, Left, Proximal	Day 43	Vehicle	2	0	0		
Nerve, Femoral, Proximal, Left, Proximal	Day 43	Low Dose	4	4.945771189	0.584439705	4.015797199	5.875745179
Nerve, Femoral, Proximal, Left, Proximal	Day 43	High Dose	4	4.820138623	0.403764858	4.177658633	5.462618613
Nerve, Femoral, Proximal, Left, Proximal	Day 85	Vehicle	2	0	0		
Nerve, Femoral, Proximal, Left, Proximal	Day 85	Low Dose	4	3.945965805	0.664841694	2.888054308	5.003877302
Nerve, Femoral, Proximal, Left, Proximal	Day 85	High Dose	4	4.907478276	0.830677649	3.585684767	6.229271784
Nerve, Femoral, Proximal, Right, Distal	Day 43	Vehicle	2	1.148091186	1.623646126		
Nerve, Femoral, Proximal, Right, Distal	Day 43	Low Dose	4	5.307506553	0.513584598	4.490278849	6.124734257
Nerve, Femoral, Proximal, Right, Distal	Day 43	High Dose	4	5.18648849	0.393589819	4.560199258	5.812777722
Nerve, Femoral, Proximal, Right, Distal	Day 85	Vehicle	2	1.083599572	1.53244121		
Nerve, Femoral, Proximal, Right, Distal	Day 85	Low Dose	4	3.781703803	0.788833869	2.526493086	5.036914519
Nerve, Femoral, Proximal, Right, Distal	Day 85	High Dose	4	4.157707536	0.163494995	3.897550514	4.417864558
Nerve, Femoral, Proximal, Right, Proximal	Day 43	Vehicle	2	0	0		
Nerve, Femoral, Proximal, Right, Proximal	Day 43	Low Dose	4	5.115924412	0.460209426	4.383628519	5.848220306
Nerve, Femoral, Proximal, Right, Proximal	Day 43	High Dose	4	4.971047078	0.271841461	4.538486651	5.403607505
Nerve, Femoral, Proximal, Right, Proximal	Day 85	Vehicle	2	0	0		
Nerve, Femoral, Proximal, Right, Proximal	Day 85	Low Dose	4	3.840636125	0.806403588	2.557468066	5.123804184
Nerve, Femoral, Proximal, Right, Proximal	Day 85	High Dose	4	4.125603667	0.550572972	3.249519206	5.001688128
Nerve, Optic	Day 43	Vehicle	2	0	0		
Nerve, Optic	Day 43	Low Dose	4	5.618740378	0.257417149	5.20913225	6.028348506
Nerve, Optic	Day 43	High Dose	4	5.599264128	0.775928998	4.364587942	6.833940314
Nerve, Optic	Day 85	Vehicle	2	0	0		
Nerve, Optic	Day 85	Low Dose	4	5.508074808	0.555158699	4.624693433	6.391456182
Nerve, Optic	Day 85	High Dose	4	5.172739159	0.574772023	4.258148609	6.08732971
Nerve, Sciatic, Distal, Left, Distal	Day 43	Vehicle	2	0	0		
Nerve, Sciatic, Distal, Left, Distal	Day 43	Low Dose	4	5.114695488	0.255220723	4.708582364	5.520808612
Nerve, Sciatic, Distal, Left, Distal	Day 43	High Dose	4	5.065300788	0.099870728	4.906384173	5.224217403
Nerve, Sciatic, Distal, Left, Distal	Day 85	Vehicle	2	1.187401658	1.679239529		
Nerve, Sciatic, Distal, Left, Distal	Day 85	Low Dose	4	4.255116179	0.987969384	2.68303642	5.827195938
Nerve, Sciatic, Distal, Left, Distal	Day 85	High Dose	4	4.454937159	0.218516848	4.107228092	4.802646227
Nerve, Sciatic, Distal, Left, Proximal	Day 43	Vehicle	2	0	0		
Nerve, Sciatic, Distal, Left, Proximal	Day 43	Low Dose	4	5.560157197	0.375291362	4.962984892	6.157329501
Nerve, Sciatic, Distal, Left, Proximal	Day 43	High Dose	4	5.362582261	0.171983488	5.088918154	5.636246369
Nerve, Sciatic, Distal, Left, Proximal	Day 85	Vehicle	2	0	0		
Nerve, Sciatic, Distal, Left, Proximal	Day 85	Low Dose	4	4.426201069	1.02311146	2.798202426	6.054199713
Nerve, Sciatic, Distal, Left, Proximal	Day 85	High Dose	4	4.807303887	0.31973232	4.298538417	5.316069358
Nerve, Sciatic, Distal, Right, Distal	Day 43	Vehicle	2	0	0		
Nerve, Sciatic, Distal, Right, Distal	Day 43	Low Dose	4	4.965473942	0.048875625	4.887701916	5.043245968
Nerve, Sciatic, Distal, Right, Distal	Day 43	High Dose	4	5.20251147	0.223015081	4.847644711	5.55737823
Nerve, Sciatic, Distal, Right, Distal	Day 85	Vehicle	2	0	0		
Nerve, Sciatic, Distal, Right, Distal	Day 85	Low Dose	4	4.52358833	0.705530533	3.400931812	5.646244849
Nerve, Sciatic, Distal, Right, Distal	Day 85	High Dose	4	4.54118969	0.266881157	4.116522213	4.965857166
Nerve, Sciatic, Distal, Right, Proximal	Day 43	Vehicle	2	0	0		
Nerve, Sciatic, Distal, Right, Proximal	Day 43	Low Dose	4	5.021680592	0.140003931	4.798903097	5.244458088
Nerve, Sciatic, Distal, Right, Proximal	Day 43	High Dose	4	5.241226087	0.278686782	4.797773226	5.684678947

n, mean, standard deviation lower and upper 95% confidence intervals for Day 85 and Day 43 Across Tissue and Group for Log10 AAV2-ITR Target Copies/ug of DNA for Not Normalized Data

Tissue	Timepoint	Group	n	Mean	Std	CI_lower	CI_upper
Nerve, Sciatic, Distal, Right, Proximal	Day 85	Vehicle	2	0	0		
Nerve, Sciatic, Distal, Right, Proximal	Day 85	Low Dose	4	4.448999995	0.888621995	3.035004103	5.862995888
Nerve, Sciatic, Distal, Right, Proximal	Day 85	High Dose	4	4.486381723	0.170466371	4.215131688	4.757631759
Nerve, Sciatic, Proximal, Left, Distal	Day 43	Vehicle	2	0	0		
Nerve, Sciatic, Proximal, Left, Distal	Day 43	Low Dose	4	5.294020132	0.293173477	4.827515707	5.760524556
Nerve, Sciatic, Proximal, Left, Distal	Day 43	High Dose	4	5.214179593	0.223918193	4.85787578	5.570483405
Nerve, Sciatic, Proximal, Left, Distal	Day 85	Vehicle	2	0	0		
Nerve, Sciatic, Proximal, Left, Distal	Day 85	Low Dose	4	4.434614258	1.072911471	2.727372684	6.141855832
Nerve, Sciatic, Proximal, Left, Distal	Day 85	High Dose	4	4.387646027	0.258166139	3.976846089	4.798445966
Nerve, Sciatic, Proximal, Left, Proximal	Day 43	Vehicle	2	0	0		
Nerve, Sciatic, Proximal, Left, Proximal	Day 43	Low Dose	4	5.37292044	0.337363984	4.836099057	5.909741822
Nerve, Sciatic, Proximal, Left, Proximal	Day 43	High Dose	4	5.3567331	0.257153904	4.947543854	5.765922346
Nerve, Sciatic, Proximal, Left, Proximal	Day 85	Vehicle	2	0	0		
Nerve, Sciatic, Proximal, Left, Proximal	Day 85	Low Dose	4	4.386002797	0.729606956	3.225035316	5.546970277
Nerve, Sciatic, Proximal, Left, Proximal	Day 85	High Dose	4	4.314284412	0.087990958	4.174271162	4.454297662
Nerve, Sciatic, Proximal, Right, Distal	Day 43	Vehicle	2	0	0		
Nerve, Sciatic, Proximal, Right, Distal	Day 43	Low Dose	4	5.433939906	0.192820104	5.127120093	5.740759719
Nerve, Sciatic, Proximal, Right, Distal	Day 43	High Dose	4	5.28978952	0.411643524	4.634772814	5.944806226
Nerve, Sciatic, Proximal, Right, Distal	Day 85	Vehicle	2	1.17498825	1.661684318		
Nerve, Sciatic, Proximal, Right, Distal	Day 85	Low Dose	4	4.275985829	1.033548042	2.631380255	5.920591404
Nerve, Sciatic, Proximal, Right, Distal	Day 85	High Dose	4	4.765677983	0.487648116	3.989721011	5.541634955
Nerve, Sciatic, Proximal, Right, Proximal	Day 43	Vehicle	2	0	0		
Nerve, Sciatic, Proximal, Right, Proximal	Day 43	Low Dose	4	4.931779032	0.537118115	4.077104251	5.786453813
Nerve, Sciatic, Proximal, Right, Proximal	Day 43	High Dose	4	5.346833452	0.276531771	4.906809697	5.786857208
Nerve, Sciatic, Proximal, Right, Proximal	Day 85	Vehicle	2	1.163771416	1.64582132		
Nerve, Sciatic, Proximal, Right, Proximal	Day 85	Low Dose	4	4.279715116	0.943554367	2.778309562	5.78112067
Nerve, Sciatic, Proximal, Right, Proximal	Day 85	High Dose	4	4.585162826	0.249099422	4.188790059	4.981535593
Nerve, Ulnar, Distal, Left, Distal	Day 43	Vehicle	2	0	0		
Nerve, Ulnar, Distal, Left, Distal	Day 43	Low Dose	4	4.711855662	0.385556395	4.0983494	5.325361924
Nerve, Ulnar, Distal, Left, Distal	Day 43	High Dose	4	5.092680902	0.227200793	4.731153739	5.454208064
Nerve, Ulnar, Distal, Left, Distal	Day 85	Vehicle	2	0	0		
Nerve, Ulnar, Distal, Left, Distal	Day 85	Low Dose	4	4.219746293	1.00503202	2.620516073	5.818976513
Nerve, Ulnar, Distal, Left, Distal	Day 85	High Dose	4	5.017361891	0.375511282	4.419839645	5.614884137
Nerve, Ulnar, Distal, Left, Proximal	Day 43	Vehicle	2	0	0		
Nerve, Ulnar, Distal, Left, Proximal	Day 43	Low Dose	4	5.215814607	0.459032127	4.485392059	5.946237156
Nerve, Ulnar, Distal, Left, Proximal	Day 43	High Dose	4	5.255187143	0.37665196	4.655849823	5.854524463
Nerve, Ulnar, Distal, Left, Proximal	Day 85	Vehicle	2	0	0		
Nerve, Ulnar, Distal, Left, Proximal	Day 85	Low Dose	4	4.007732306	0.9499569	2.496138892	5.51932572
Nerve, Ulnar, Distal, Left, Proximal	Day 85	High Dose	4	4.307965097	0.566467989	3.406588118	5.209342077
Nerve, Ulnar, Distal, Right, Distal	Day 43	Vehicle	2	0	0		
Nerve, Ulnar, Distal, Right, Distal	Day 43	Low Dose	4	4.760288269	0.220004769	4.410211587	5.110364952
Nerve, Ulnar, Distal, Right, Distal	Day 43	High Dose	4	5.126576461	0.463199633	4.389522481	5.863630441
Nerve, Ulnar, Distal, Right, Distal	Day 85	Vehicle	2	0	0		
Nerve, Ulnar, Distal, Right, Distal	Day 85	Low Dose	4	4.359892443	0.731538382	3.195851632	5.523933254
Nerve, Ulnar, Distal, Right, Distal	Day 85	High Dose	4	4.54996499	0.42948767	3.866554266	5.233375715
Nerve, Ulnar, Distal, Right, Proximal	Day 43	Vehicle	2	0	0		
Nerve, Ulnar, Distal, Right, Proximal	Day 43	Low Dose	4	4.892276758	0.408051223	4.242976204	5.541577312
Nerve, Ulnar, Distal, Right, Proximal	Day 43	High Dose	4	5.060255699	0.27532851	4.622146599	5.498364799
Nerve, Ulnar, Distal, Right, Proximal	Day 85	Vehicle	2	0	0		
Nerve, Ulnar, Distal, Right, Proximal	Day 85	Low Dose	4	4.107121492	0.660701957	3.055797241	5.158445743
Nerve, Ulnar, Distal, Right, Proximal	Day 85	High Dose	4	4.464270334	0.600423501	3.508862559	5.41967811
Nerve, Ulnar, Proximal, Left, Distal	Day 43	Vehicle	2	0	0		
Nerve, Ulnar, Proximal, Left, Distal	Day 43	Low Dose	4	5.313765624	0.202007799	4.992326138	5.635205111
Nerve, Ulnar, Proximal, Left, Distal	Day 43	High Dose	4	5.262659494	0.24070065	4.879651046	5.645667942
Nerve, Ulnar, Proximal, Left, Distal	Day 85	Vehicle	2	0	0		
Nerve, Ulnar, Proximal, Left, Distal	Day 85	Low Dose	4	4.027721858	1.068535797	2.327442958	5.728000758
Nerve, Ulnar, Proximal, Left, Distal	Day 85	High Dose	4	4.398192856	0.292625093	3.932561032	4.86382468
Nerve, Ulnar, Proximal, Left, Proximal	Day 43	Vehicle	2	0	0		
Nerve, Ulnar, Proximal, Left, Proximal	Day 43	Low Dose	4	4.790694064	0.790920466	3.532163107	6.049225021
Nerve, Ulnar, Proximal, Left, Proximal	Day 43	High Dose	4	4.753884887	0.275069271	4.316188295	5.191581479
Nerve, Ulnar, Proximal, Left, Proximal	Day 85	Vehicle	2	0	0		
Nerve, Ulnar, Proximal, Left, Proximal	Day 85	Low Dose	4	3.847829861	0.328494082	3.325122472	4.370537249
Nerve, Ulnar, Proximal, Left, Proximal	Day 85	High Dose	4	4.474402827	0.628859742	3.473746646	5.475059007
Nerve, Ulnar, Proximal, Right, Distal	Day 43	Vehicle	2	0	0		
Nerve, Ulnar, Proximal, Right, Distal	Day 43	Low Dose	4	5.035392905	0.650212	4.000760516	6.070025294

n, mean, standard deviation lower and upper 95% confidence intervals for Day 85 and Day 43 Across Tissue and Group for Log10 AAV2-ITR Target Copies/ug of DNA for Not Normalized Data

Tissue	Timepoint	Group	n	Mean	Std	CI_lower	CI_upper
Nerve, Ulnar, Proximal, Right, Distal	Day 43	High Dose	4	5.259085348	0.328010068	4.737148133	5.781022563
Nerve, Ulnar, Proximal, Right, Distal	Day 85	Vehicle	2	0	0		
Nerve, Ulnar, Proximal, Right, Distal	Day 85	Low Dose	4	3.940136017	0.425299468	3.263389657	4.616882377
Nerve, Ulnar, Proximal, Right, Distal	Day 85	High Dose	4	4.650655533	0.618603339	3.666319578	5.634991488
Nerve, Ulnar, Proximal, Right, Proximal	Day 43	Vehicle	2	0	0		
Nerve, Ulnar, Proximal, Right, Proximal	Day 43	Low Dose	4	5.106559726	0.605598912	4.142916716	6.070202737
Nerve, Ulnar, Proximal, Right, Proximal	Day 43	High Dose	4	5.171250933	0.455144982	4.4470137	5.895488166
Nerve, Ulnar, Proximal, Right, Proximal	Day 85	Vehicle	2	0	0		
Nerve, Ulnar, Proximal, Right, Proximal	Day 85	Low Dose	4	4.135414656	0.205141596	3.808988598	4.461840713
Nerve, Ulnar, Proximal, Right, Proximal	Day 85	High Dose	4	4.296460222	0.428862042	3.614045012	4.978875433
Ovary	Day 43	Vehicle	1	0			
Ovary	Day 43	Low Dose	2	4.161632289	0.065776381	3.570654951	4.752609627
Ovary	Day 43	High Dose	2	4.879072357	0.778537701	0	11.87395607
Ovary	Day 85	Vehicle	1	0			
Ovary	Day 85	Low Dose	2	5.108504443	0.75873212	0	11.92544208
Ovary	Day 85	High Dose	2	4.124085115	0.033261145	3.825245581	4.422924649
Spinal Cord, Cervical	Day 43	Vehicle	2	1.133832216	1.603480897		
Spinal Cord, Cervical	Day 43	Low Dose	4	7.156607907	0.874680543	5.764795976	8.548419838
Spinal Cord, Cervical	Day 43	High Dose	4	5.655214041	0.597885021	4.703845554	6.606582529
Spinal Cord, Cervical	Day 85	Vehicle	2	0	0		
Spinal Cord, Cervical	Day 85	Low Dose	4	5.408819478	0.39388688	4.782057555	6.0355814
Spinal Cord, Cervical	Day 85	High Dose	4	5.433690709	0.490043537	4.653922086	6.213459331
Spinal Cord, Lumbar	Day 43	Vehicle	2	0	0		
Spinal Cord, Lumbar	Day 43	Low Dose	4	6.887211957	0.488438247	6.10999771	7.664426204
Spinal Cord, Lumbar	Day 43	High Dose	4	6.215120971	0.16735109	5.948828043	6.481413899
Spinal Cord, Lumbar	Day 85	Vehicle	2	0	0		
Spinal Cord, Lumbar	Day 85	Low Dose	4	5.382257611	0.3756662	4.784488856	5.980026367
Spinal Cord, Lumbar	Day 85	High Dose	4	5.719534052	1.216475254	3.783850464	7.65521764
Spinal Cord, Sacral	Day 43	Vehicle	2	0	0		
Spinal Cord, Sacral	Day 43	Low Dose	4	6.949756639	0.767876582	5.727893643	8.171619634
Spinal Cord, Sacral	Day 43	High Dose	4	6.063783663	0.278297088	5.620950893	6.506616432
Spinal Cord, Sacral	Day 85	Vehicle	2	0	0		
Spinal Cord, Sacral	Day 85	Low Dose	4	5.366148411	0.310221036	4.872517516	5.859779306
Spinal Cord, Sacral	Day 85	High Dose	4	5.727021285	0.670957064	4.65937887	6.7946637
Spinal Cord, Spinal Roots	Day 43	Vehicle	2	0	0		
Spinal Cord, Spinal Roots	Day 43	Low Dose	4	6.42062159	0.525837106	5.583897412	7.257345769
Spinal Cord, Spinal Roots	Day 43	High Dose	4	6.573451558	0.833073862	5.247845141	7.899057975
Spinal Cord, Spinal Roots	Day 85	Vehicle	2	0	0		
Spinal Cord, Spinal Roots	Day 85	Low Dose	4	5.473175584	0.68073574	4.389973113	6.556378054
Spinal Cord, Spinal Roots	Day 85	High Dose	4	5.911067251	1.194639459	4.010129285	7.812005217
Spinal Cord, Thoracic	Day 43	Vehicle	2	0	0		
Spinal Cord, Thoracic	Day 43	Low Dose	4	7.13082641	1.102817433	5.375997778	8.885655043
Spinal Cord, Thoracic	Day 43	High Dose	4	6.320487479	0.509548876	5.509681509	7.131293448
Spinal Cord, Thoracic	Day 85	Vehicle	2	0	0		
Spinal Cord, Thoracic	Day 85	Low Dose	4	5.571557341	0.496417679	4.781646036	6.361468645
Spinal Cord, Thoracic	Day 85	High Dose	4	6.011653531	1.188651252	4.120244137	7.903062924
Spleen	Day 43	Vehicle	2	1.325944156	1.875168208		
Spleen	Day 43	Low Dose	4	6.001107178	0.800131718	4.727919062	7.274295293
Spleen	Day 43	High Dose	4	6.222078002	0.043966789	6.15211703	6.292038974
Spleen	Day 85	Vehicle	2	1.131889025	1.60073281		
Spleen	Day 85	Low Dose	4	5.957873024	0.727268483	4.800626575	7.115119473
Spleen	Day 85	High Dose	4	6.019695519	0.300772976	5.541098596	6.498292443
Testis	Day 43	Vehicle	1	0			
Testis	Day 43	Low Dose	2	4.480541933	0.274192546	2.017019646	6.944064221
Testis	Day 43	High Dose	2	4.500774321	0.226023059	2.470037707	6.531510934
Testis	Day 85	Vehicle	1	0			
Testis	Day 85	Low Dose	2	3.990620619	0.001464489	3.977462708	4.003778529
Testis	Day 85	High Dose	2	4.324652129	0.34605393	1.215480925	7.433823332

Results of Evaluating Mean Differences Between Different Tissues Across Timepoint and Group for Log10 AAV2-ITR Target Copies/ug of DNA for Normalized Data

Group	Timepoint	Difference	Mean	StdDev	LowerCLMean	UpperCLMean	DF	tValue	P-Value
Vehicle	Day 43	Brain - Ganglion, Dorsal Root, Cervical	-1.87127E-05	2.64637E-05	-0.00025648	0.000219055	1	-1	0.5
Vehicle	Day 43	Brain - Ganglion, Dorsal Root, Lumbar	0	0	0	0	1		
Vehicle	Day 43	Brain - Ganglion, Dorsal Root, Thoracic	0	0	0	0	1		
Vehicle	Day 43	Brain - Heart	0	0	0	0	1		
Vehicle	Day 43	Brain - Kidney	0	0	0	0	1		
Vehicle	Day 43	Brain - Lung	0	0	0	0	1		
Vehicle	Day 43	Brain - Liver	0	0	0	0	1		
Vehicle	Day 43	Brain - Muscle, Skeletal, Tibialis Anterior Left	0	0	0	0	1		
Vehicle	Day 43	Brain - Muscle, Skeletal, Tibialis Anterior Right	-6.21787E-05	8.7934E-05	-0.000852234	0.000727877	1	-1	0.5
Vehicle	Day 43	Brain - Muscle, Skeletal, Bicep Brachii Left	-0.000143992	0.000203636	-0.001973589	0.001685604	1	-1	0.5
Vehicle	Day 43	Brain - Muscle, Skeletal, Bicep Brachii Right	0	0	0	0	1		
Vehicle	Day 43	Brain - Nerve, Femoral, Distal, Left, Distal	0	0	0	0	1		
Vehicle	Day 43	Brain - Nerve, Femoral, Distal, Left, Proximal	0	0	0	0	1		
Vehicle	Day 43	Brain - Nerve, Femoral, Distal, Right, Distal	0	0	0	0	1		
Vehicle	Day 43	Brain - Nerve, Femoral, Distal, Right, Proximal	0	0	0	0	1		
Vehicle	Day 43	Brain - Nerve, Femoral, Proximal, Left, Distal	0	0	0	0	1		
Vehicle	Day 43	Brain - Nerve, Femoral, Proximal, Left, Proximal	0	0	0	0	1		
Vehicle	Day 43	Brain - Nerve, Femoral, Proximal, Right, Distal	-0.00010276	0.000145325	-0.00140845	0.00120293	1	-1	0.5
Vehicle	Day 43	Brain - Nerve, Femoral, Proximal, Right, Proximal	0	0	0	0	1		
Vehicle	Day 43	Brain - Nerve Median, Distal, Left, Distal	0	0	0	0	1		
Vehicle	Day 43	Brain - Nerve Median, Distal, Left, Proximal	0	0	0	0	1		
Vehicle	Day 43	Brain - Nerve Median, Distal, Right, Distal	0	0	0	0	1		
Vehicle	Day 43	Brain - Nerve Median, Distal, Right, Proximal	0	0	0	0	1		
Vehicle	Day 43	Brain - Nerve Median, Proximal, Left, Distal	0	0	0	0	1		
Vehicle	Day 43	Brain - Nerve Median, Proximal, Left, Proximal	0	0	0	0	1		
Vehicle	Day 43	Brain - Nerve Median, Proximal, Right, Distal	0	0	0	0	1		
Vehicle	Day 43	Brain - Nerve Median, Proximal, Right, Proximal	0	0	0	0	1		
Vehicle	Day 43	Brain - Nerve, Sciatic, Distal, Left, Distal	0	0	0	0	1		
Vehicle	Day 43	Brain - Nerve, Sciatic, Distal, Left, Proximal	0	0	0	0	1		
Vehicle	Day 43	Brain - Nerve, Sciatic, Distal, Right, Distal	0	0	0	0	1		
Vehicle	Day 43	Brain - Nerve, Sciatic, Distal, Right, Proximal	0	0	0	0	1		
Vehicle	Day 43	Brain - Nerve, Sciatic, Proximal, Left, Distal	0	0	0	0	1		
Vehicle	Day 43	Brain - Nerve, Sciatic, Proximal, Left, Proximal	0	0	0	0	1		
Vehicle	Day 43	Brain - Nerve, Sciatic, Proximal, Right, Distal	0	0	0	0	1		
Vehicle	Day 43	Brain - Nerve, Sciatic, Proximal, Right, Proximal	0	0	0	0	1		
Vehicle	Day 43	Brain - Nerve, Ulnar, Distal, Left, Distal	0	0	0	0	1		
Vehicle	Day 43	Brain - Nerve, Ulnar, Distal, Left, Proximal	0	0	0	0	1		
Vehicle	Day 43	Brain - Nerve, Ulnar, Distal, Right, Distal	0	0	0	0	1		
Vehicle	Day 43	Brain - Nerve, Ulnar, Distal, Right, Proximal	0	0	0	0	1		
Vehicle	Day 43	Brain - Nerve, Ulnar, Proximal, Left, Distal	0	0	0	0	1		
Vehicle	Day 43	Brain - Nerve, Ulnar, Proximal, Left, Proximal	0	0	0	0	1		
Vehicle	Day 43	Brain - Nerve, Ulnar, Proximal, Right, Distal	0	0	0	0	1		
Vehicle	Day 43	Brain - Nerve, Ulnar, Proximal, Right, Proximal	0	0	0	0	1		
Vehicle	Day 43	Brain - Nerve, Optic	0	0	0	0	1		
Vehicle	Day 43	Brain - Ovary	0				0		
Vehicle	Day 43	Brain - Spinal Cord, Cervical	-0.0001082	0.000153018	-0.001483016	0.001266615	1	-1	0.5
Vehicle	Day 43	Brain - Spinal Cord, Lumbar	0	0	0	0	1		
Vehicle	Day 43	Brain - Spinal Cord, Sacral	0	0	0	0	1		
Vehicle	Day 43	Brain - Spinal Cord, Spinal Roots	0	0	0	0	1		
Vehicle	Day 43	Brain - Spinal Cord, Thoracic	0	0	0	0	1		
Vehicle	Day 43	Brain - Spleen	-0.000216745	0.000306524	-0.002970753	0.002537263	1	-1	0.5
Vehicle	Day 43	Brain - Testis	0				0		
Vehicle	Day 43	Ganglion, Dorsal Root, Cervical - Ganglion, Dorsal Root, Lumbar	1.87127E-05	2.64637E-05	-0.000219055	0.00025648	1	1	0.5
Vehicle	Day 43	Ganglion, Dorsal Root, Cervical - Ganglion, Dorsal Root, Thoracic	1.87127E-05	2.64637E-05	-0.000219055	0.00025648	1	1	0.5
Vehicle	Day 43	Ganglion, Dorsal Root, Cervical - Heart	1.87127E-05	2.64637E-05	-0.000219055	0.00025648	1	1	0.5
Vehicle	Day 43	Ganglion, Dorsal Root, Cervical - Kidney	1.87127E-05	2.64637E-05	-0.000219055	0.00025648	1	1	0.5
Vehicle	Day 43	Ganglion, Dorsal Root, Cervical - Lung	1.87127E-05	2.64637E-05	-0.000219055	0.00025648	1	1	0.5
Vehicle	Day 43	Ganglion, Dorsal Root, Cervical - Liver	1.87127E-05	2.64637E-05	-0.000219055	0.00025648	1	1	0.5
Vehicle	Day 43	Ganglion, Dorsal Root, Cervical - Muscle, Skeletal, Tibialis Anterior Left	1.87127E-05	2.64637E-05	-0.000219055	0.00025648	1	1	0.5
Vehicle	Day 43	Ganglion, Dorsal Root, Cervical - Muscle, Skeletal, Tibialis Anterior Right	-4.3466E-05	0.000114398	-0.001071289	0.000984357	1	-0.537338104	0.686101863
Vehicle	Day 43	Ganglion, Dorsal Root, Cervical - Muscle, Skeletal, Bicep Brachii Left	-0.00012528	0.000177172	-0.001717109	0.001466549	1	-1	0.5
Vehicle	Day 43	Ganglion, Dorsal Root, Cervical - Muscle, Skeletal, Bicep Brachii Right	1.87127E-05	2.64637E-05	-0.000219055	0.00025648	1	1	0.5
Vehicle	Day 43	Ganglion, Dorsal Root, Cervical - Nerve, Femoral, Distal, Left, Distal	1.87127E-05	2.64637E-05	-0.000219055	0.00025648	1	1	0.5
Vehicle	Day 43	Ganglion, Dorsal Root, Cervical - Nerve, Femoral, Distal, Left, Proximal	1.87127E-05	2.64637E-05	-0.000219055	0.00025648	1	1	0.5
Vehicle	Day 43	Ganglion, Dorsal Root, Cervical - Nerve, Femoral, Distal, Right, Distal	1.87127E-05	2.64637E-05	-0.000219055	0.00025648	1	1	0.5
Vehicle	Day 43	Ganglion, Dorsal Root, Cervical - Nerve, Femoral, Distal, Right, Proximal	1.87127E-05	2.64637E-05	-0.000219055	0.00025648	1	1	0.5
Vehicle	Day 43	Ganglion, Dorsal Root, Cervical - Nerve, Femoral, Proximal, Left, Distal	1.87127E-05	2.64637E-05	-0.000219055	0.00025648	1	1	0.5
Vehicle	Day 43	Ganglion, Dorsal Root, Cervical - Nerve, Femoral, Proximal, Left, Proximal	1.87127E-05	2.64637E-05	-0.000219055	0.00025648	1	1	0.5
Vehicle	Day 43	Ganglion, Dorsal Root, Cervical - Nerve, Femoral, Proximal, Right, Distal	-8.40474E-05	0.000171788	-0.001627505	0.00145941	1	-0.691903116	0.614672438
Vehicle	Day 43	Ganglion, Dorsal Root, Cervical - Nerve, Femoral, Proximal, Right, Proximal	1.87127E-05	2.64637E-05	-0.000219055	0.00025648	1	1	0.5
Vehicle	Day 43	Ganglion, Dorsal Root, Cervical - Nerve Median, Distal, Left, Distal	1.87127E-05	2.64637E-05	-0.000219055	0.00025648	1	1	0.5
Vehicle	Day 43	Ganglion, Dorsal Root, Cervical - Nerve Median, Distal, Left, Proximal	1.87127E-05	2.64637E-05	-0.000219055	0.00025648	1	1	0.5
Vehicle	Day 43	Ganglion, Dorsal Root, Cervical - Nerve Median, Distal, Right, Distal	1.87127E-05	2.64637E-05	-0.000219055	0.00025648	1	1	0.5
Vehicle	Day 43	Ganglion, Dorsal Root, Cervical - Nerve Median, Distal, Right, Proximal	1.87127E-05	2.64637E-05	-0.000219055	0.00025648	1	1	0.5
Vehicle	Day 43	Ganglion, Dorsal Root, Cervical - Nerve Median, Proximal, Left, Distal	1.87127E-05	2.64637E-05	-0.000219055	0.00025648	1	1	0.5
Vehicle	Day 43	Ganglion, Dorsal Root, Cervical - Nerve Median, Proximal, Left, Proximal	1.87127E-05	2.64637E-05	-0.000219055	0.00025648	1	1	0.5
Vehicle	Day 43	Ganglion, Dorsal Root, Cervical - Nerve Median, Proximal, Right, Distal	1.87127E-05	2.64637E-05	-0.000219055	0.00025648	1	1	0.5
Vehicle	Day 43	Ganglion, Dorsal Root, Cervical - Nerve Median, Proximal, Right, Proximal	1.87127E-05	2.64637E-05	-0.000219055	0.00025648	1	1	0.5
Vehicle	Day 43	Ganglion, Dorsal Root, Cervical - Nerve, Sciatic, Distal, Left, Distal	1.87127E-05	2.64637E-05	-0.000219055	0.00025648	1	1	0.5
Vehicle	Day 43	Ganglion, Dorsal Root, Cervical - Nerve, Sciatic, Distal, Left, Proximal	1.87127E-05	2.64637E-05	-0.000219055	0.00025648	1	1	0.5
Vehicle	Day 43	Ganglion, Dorsal Root, Cervical - Nerve, Sciatic, Distal, Right, Distal	1.87127E-05	2.64637E-05	-0.000219055	0.00025648	1	1	0.5
Vehicle	Day 43	Ganglion, Dorsal Root, Cervical - Nerve, Sciatic, Distal, Right, Proximal	1.87127E-05	2.64637E-05	-0.000219055	0.00025648	1	1	0.5
Vehicle	Day 43	Ganglion, Dorsal Root, Cervical - Nerve, Sciatic, Proximal, Left, Distal	1.87127E-05	2.64637E-05	-0.000219055	0.00025648	1	1	0.5
Vehicle	Day 43	Ganglion, Dorsal Root, Cervical - Nerve, Sciatic, Proximal, Left, Proximal	1.87127E-05	2.64637E-05	-0.000219055	0.00025648	1	1	0.5
Vehicle	Day 43	Ganglion, Dorsal Root, Cervical - Nerve, Sciatic, Proximal, Right, Distal	1.87127E-05	2.64637E-05	-0.000219055	0.00025648	1	1	0.5
Vehicle	Day 43	Ganglion, Dorsal Root, Cervical - Nerve, Sciatic, Proximal, Right, Proximal	1.87127E-05	2.64637E-05	-0.000219055	0.00025648	1	1	0.5
Vehicle	Day 43	Ganglion, Dorsal Root, Cervical - Nerve, Ulnar, Distal, Left, Distal	1.87127E-05	2.64637E-05	-0.000219055	0.00025648	1	1	0.5
Vehicle	Day 43	Ganglion, Dorsal Root, Cervical - Nerve, Ulnar, Distal, Left, Proximal	1.87127E-05	2.64637E-05	-0.000219055	0.00025648	1	1	0.5
Vehicle	Day 43	Ganglion, Dorsal Root, Cervical - Nerve, Ulnar, Distal, Right, Distal	1.87127E-05	2.64637E-05	-0.000219055	0.00025648	1	1	0.5
Vehicle	Day 43	Ganglion, Dorsal Root, Cervical - Nerve, Ulnar, Distal, Right, Proximal	1.87127E-05	2.64637E-05	-0.000219055	0.00025648	1	1	0.5
Vehicle	Day 43	Ganglion, Dorsal Root, Cervical - Nerve, Ulnar, Proximal, Left, Distal	1.87127E-05	2.64637E-05	-0.000219055	0.00025648	1	1	0.5
Vehicle	Day 43	Ganglion, Dorsal Root, Cervical - Nerve, Ulnar, Proximal, Left, Proximal	1.87127E-05	2.64637E-05	-0.000219055	0.00025648	1	1	0.5
Vehicle	Day 43	Ganglion, Dorsal Root, Cervical - Nerve, Ulnar, Proximal, Right, Distal	1.87127E-05	2.64637E-05	-0.000219055	0.00025648	1	1	0.5

Results of Evaluating Mean Differences Between Different Tissues Across Timepoint and Group for Log10 AAV2-ITR Target Copies/ug of DNA for Normalized Data

Group	Timepoint	Difference	Mean	StdDev	LowerCLMean	UpperCLMean	DF	tValue	P-Value
Vehicle	Day 43	Ganglion, Dorsal Root, Cervical - Nerve, Ulnar, Proximal, Right, Proximal	1.87127E-05	2.64637E-05	-0.000219055	0.00025648	1	1	0.5
Vehicle	Day 43	Ganglion, Dorsal Root, Cervical - Nerve, Optic	1.87127E-05	2.64637E-05	-0.000219055	0.00025648	1	1	0.5
Vehicle	Day 43	Ganglion, Dorsal Root, Cervical - Ovary	3.74254E-05				0		
Vehicle	Day 43	Ganglion, Dorsal Root, Cervical - Spinal Cord, Cervical	-8.94876E-05	0.000126555	-0.001226536	0.001047561	1	-1	0.5
Vehicle	Day 43	Ganglion, Dorsal Root, Cervical - Spinal Cord, Lumbar	1.87127E-05	2.64637E-05	-0.000219055	0.00025648	1	1	0.5
Vehicle	Day 43	Ganglion, Dorsal Root, Cervical - Spinal Cord, Sacral	1.87127E-05	2.64637E-05	-0.000219055	0.00025648	1	1	0.5
Vehicle	Day 43	Ganglion, Dorsal Root, Cervical - Spinal Cord, Spinal Roots	1.87127E-05	2.64637E-05	-0.000219055	0.00025648	1	1	0.5
Vehicle	Day 43	Ganglion, Dorsal Root, Cervical - Spinal Cord, Thoracic	1.87127E-05	2.64637E-05	-0.000219055	0.00025648	1	1	0.5
Vehicle	Day 43	Ganglion, Dorsal Root, Cervical - Spleen	-0.000198032	0.000332988	-0.003189808	0.002793743	1	-0.841052737	0.554826608
Vehicle	Day 43	Ganglion, Dorsal Root, Cervical - Testis	0				0		
Vehicle	Day 43	Ganglion, Dorsal Root, Lumbar - Ganglion, Dorsal Root, Thoracic	0	0	0	0	1		
Vehicle	Day 43	Ganglion, Dorsal Root, Lumbar - Heart	0	0	0	0	1		
Vehicle	Day 43	Ganglion, Dorsal Root, Lumbar - Kidney	0	0	0	0	1		
Vehicle	Day 43	Ganglion, Dorsal Root, Lumbar - Lung	0	0	0	0	1		
Vehicle	Day 43	Ganglion, Dorsal Root, Lumbar - Liver	0	0	0	0	1		
Vehicle	Day 43	Ganglion, Dorsal Root, Lumbar - Muscle, Skeletal, Tibialis Anterior Left	0	0	0	0	1		
Vehicle	Day 43	Ganglion, Dorsal Root, Lumbar - Muscle, Skeletal, Tibialis Anterior Right	-6.21787E-05	8.7934E-05	-0.000852234	0.000727877	1	-1	0.5
Vehicle	Day 43	Ganglion, Dorsal Root, Lumbar - Muscle, Skeletal, Bicep Brachii Left	-0.000143992	0.000203636	-0.001973589	0.001685604	1	-1	0.5
Vehicle	Day 43	Ganglion, Dorsal Root, Lumbar - Muscle, Skeletal, Bicep Brachii Right	0	0	0	0	1		
Vehicle	Day 43	Ganglion, Dorsal Root, Lumbar - Nerve, Femoral, Distal, Left, Distal	0	0	0	0	1		
Vehicle	Day 43	Ganglion, Dorsal Root, Lumbar - Nerve, Femoral, Distal, Left, Proximal	0	0	0	0	1		
Vehicle	Day 43	Ganglion, Dorsal Root, Lumbar - Nerve, Femoral, Distal, Right, Distal	0	0	0	0	1		
Vehicle	Day 43	Ganglion, Dorsal Root, Lumbar - Nerve, Femoral, Distal, Right, Proximal	0	0	0	0	1		
Vehicle	Day 43	Ganglion, Dorsal Root, Lumbar - Nerve, Femoral, Proximal, Left, Distal	0	0	0	0	1		
Vehicle	Day 43	Ganglion, Dorsal Root, Lumbar - Nerve, Femoral, Proximal, Left, Proximal	0	0	0	0	1		
Vehicle	Day 43	Ganglion, Dorsal Root, Lumbar - Nerve, Femoral, Proximal, Right, Distal	-0.00010276	0.000145325	-0.00140845	0.00120293	1	-1	0.5
Vehicle	Day 43	Ganglion, Dorsal Root, Lumbar - Nerve, Femoral, Proximal, Right, Proximal	0	0	0	0	1		
Vehicle	Day 43	Ganglion, Dorsal Root, Lumbar - Nerve Median, Distal, Left, Distal	0	0	0	0	1		
Vehicle	Day 43	Ganglion, Dorsal Root, Lumbar - Nerve Median, Distal, Left, Proximal	0	0	0	0	1		
Vehicle	Day 43	Ganglion, Dorsal Root, Lumbar - Nerve Median, Distal, Right, Distal	0	0	0	0	1		
Vehicle	Day 43	Ganglion, Dorsal Root, Lumbar - Nerve Median, Distal, Right, Proximal	0	0	0	0	1		
Vehicle	Day 43	Ganglion, Dorsal Root, Lumbar - Nerve Median, Proximal, Left, Distal	0	0	0	0	1		
Vehicle	Day 43	Ganglion, Dorsal Root, Lumbar - Nerve Median, Proximal, Left, Proximal	0	0	0	0	1		
Vehicle	Day 43	Ganglion, Dorsal Root, Lumbar - Nerve Median, Proximal, Right, Distal	0	0	0	0	1		
Vehicle	Day 43	Ganglion, Dorsal Root, Lumbar - Nerve Median, Proximal, Right, Proximal	0	0	0	0	1		
Vehicle	Day 43	Ganglion, Dorsal Root, Lumbar - Nerve, Sciatic, Distal, Left, Distal	0	0	0	0	1		
Vehicle	Day 43	Ganglion, Dorsal Root, Lumbar - Nerve, Sciatic, Distal, Left, Proximal	0	0	0	0	1		
Vehicle	Day 43	Ganglion, Dorsal Root, Lumbar - Nerve, Sciatic, Distal, Right, Distal	0	0	0	0	1		
Vehicle	Day 43	Ganglion, Dorsal Root, Lumbar - Nerve, Sciatic, Distal, Right, Proximal	0	0	0	0	1		
Vehicle	Day 43	Ganglion, Dorsal Root, Lumbar - Nerve, Sciatic, Proximal, Left, Distal	0	0	0	0	1		
Vehicle	Day 43	Ganglion, Dorsal Root, Lumbar - Nerve, Sciatic, Proximal, Left, Proximal	0	0	0	0	1		
Vehicle	Day 43	Ganglion, Dorsal Root, Lumbar - Nerve, Sciatic, Proximal, Right, Distal	0	0	0	0	1		
Vehicle	Day 43	Ganglion, Dorsal Root, Lumbar - Nerve, Sciatic, Proximal, Right, Proximal	0	0	0	0	1		
Vehicle	Day 43	Ganglion, Dorsal Root, Lumbar - Nerve, Ulnar, Distal, Left, Distal	0	0	0	0	1		
Vehicle	Day 43	Ganglion, Dorsal Root, Lumbar - Nerve, Ulnar, Distal, Left, Proximal	0	0	0	0	1		
Vehicle	Day 43	Ganglion, Dorsal Root, Lumbar - Nerve, Ulnar, Distal, Right, Distal	0	0	0	0	1		
Vehicle	Day 43	Ganglion, Dorsal Root, Lumbar - Nerve, Ulnar, Distal, Right, Proximal	0	0	0	0	1		
Vehicle	Day 43	Ganglion, Dorsal Root, Lumbar - Nerve, Ulnar, Proximal, Left, Distal	0	0	0	0	1		
Vehicle	Day 43	Ganglion, Dorsal Root, Lumbar - Nerve, Ulnar, Proximal, Left, Proximal	0	0	0	0	1		
Vehicle	Day 43	Ganglion, Dorsal Root, Lumbar - Nerve, Ulnar, Proximal, Right, Distal	0	0	0	0	1		
Vehicle	Day 43	Ganglion, Dorsal Root, Lumbar - Nerve, Ulnar, Proximal, Right, Proximal	0	0	0	0	1		
Vehicle	Day 43	Ganglion, Dorsal Root, Lumbar - Nerve, Optic	0	0	0	0	1		
Vehicle	Day 43	Ganglion, Dorsal Root, Lumbar - Ovary	0				0		
Vehicle	Day 43	Ganglion, Dorsal Root, Lumbar - Spinal Cord, Cervical	-0.0001082	0.000153018	-0.001483016	0.001266615	1	-1	0.5
Vehicle	Day 43	Ganglion, Dorsal Root, Lumbar - Spinal Cord, Lumbar	0	0	0	0	1		
Vehicle	Day 43	Ganglion, Dorsal Root, Lumbar - Spinal Cord, Sacral	0	0	0	0	1		
Vehicle	Day 43	Ganglion, Dorsal Root, Lumbar - Spinal Cord, Spinal Roots	0	0	0	0	1		
Vehicle	Day 43	Ganglion, Dorsal Root, Lumbar - Spinal Cord, Thoracic	0	0	0	0	1		
Vehicle	Day 43	Ganglion, Dorsal Root, Lumbar - Spleen	-0.000216745	0.000306524	-0.002970753	0.002537263	1	-1	0.5
Vehicle	Day 43	Ganglion, Dorsal Root, Lumbar - Testis	0				0		
Vehicle	Day 43	Ganglion, Dorsal Root, Thoracic - Heart	0	0	0	0	1		
Vehicle	Day 43	Ganglion, Dorsal Root, Thoracic - Kidney	0	0	0	0	1		
Vehicle	Day 43	Ganglion, Dorsal Root, Thoracic - Lung	0	0	0	0	1		
Vehicle	Day 43	Ganglion, Dorsal Root, Thoracic - Liver	0	0	0	0	1		
Vehicle	Day 43	Ganglion, Dorsal Root, Thoracic - Muscle, Skeletal, Tibialis Anterior Left	0	0	0	0	1		
Vehicle	Day 43	Ganglion, Dorsal Root, Thoracic - Muscle, Skeletal, Tibialis Anterior Right	-6.21787E-05	8.7934E-05	-0.000852234	0.000727877	1	-1	0.5
Vehicle	Day 43	Ganglion, Dorsal Root, Thoracic - Muscle, Skeletal, Bicep Brachii Left	-0.000143992	0.000203636	-0.001973589	0.001685604	1	-1	0.5
Vehicle	Day 43	Ganglion, Dorsal Root, Thoracic - Muscle, Skeletal, Bicep Brachii Right	0	0	0	0	1		
Vehicle	Day 43	Ganglion, Dorsal Root, Thoracic - Nerve, Femoral, Distal, Left, Distal	0	0	0	0	1		
Vehicle	Day 43	Ganglion, Dorsal Root, Thoracic - Nerve, Femoral, Distal, Left, Proximal	0	0	0	0	1		
Vehicle	Day 43	Ganglion, Dorsal Root, Thoracic - Nerve, Femoral, Distal, Right, Distal	0	0	0	0	1		
Vehicle	Day 43	Ganglion, Dorsal Root, Thoracic - Nerve, Femoral, Distal, Right, Proximal	0	0	0	0	1		
Vehicle	Day 43	Ganglion, Dorsal Root, Thoracic - Nerve, Femoral, Proximal, Left, Distal	0	0	0	0	1		
Vehicle	Day 43	Ganglion, Dorsal Root, Thoracic - Nerve, Femoral, Proximal, Left, Proximal	0	0	0	0	1		
Vehicle	Day 43	Ganglion, Dorsal Root, Thoracic - Nerve, Femoral, Proximal, Right, Distal	-0.00010276	0.000145325	-0.00140845	0.00120293	1	-1	0.5
Vehicle	Day 43	Ganglion, Dorsal Root, Thoracic - Nerve, Femoral, Proximal, Right, Proximal	0	0	0	0	1		
Vehicle	Day 43	Ganglion, Dorsal Root, Thoracic - Nerve Median, Distal, Left, Distal	0	0	0	0	1		
Vehicle	Day 43	Ganglion, Dorsal Root, Thoracic - Nerve Median, Distal, Left, Proximal	0	0	0	0	1		
Vehicle	Day 43	Ganglion, Dorsal Root, Thoracic - Nerve Median, Distal, Right, Distal	0	0	0	0	1		
Vehicle	Day 43	Ganglion, Dorsal Root, Thoracic - Nerve Median, Distal, Right, Proximal	0	0	0	0	1		
Vehicle	Day 43	Ganglion, Dorsal Root, Thoracic - Nerve Median, Proximal, Left, Distal	0	0	0	0	1		
Vehicle	Day 43	Ganglion, Dorsal Root, Thoracic - Nerve Median, Proximal, Left, Proximal	0	0	0	0	1		
Vehicle	Day 43	Ganglion, Dorsal Root, Thoracic - Nerve Median, Proximal, Right, Distal	0	0	0	0	1		
Vehicle	Day 43	Ganglion, Dorsal Root, Thoracic - Nerve Median, Proximal, Right, Proximal	0	0	0	0	1		
Vehicle	Day 43	Ganglion, Dorsal Root, Thoracic - Nerve, Sciatic, Distal, Left, Distal	0	0	0	0	1		
Vehicle	Day 43	Ganglion, Dorsal Root, Thoracic - Nerve, Sciatic, Distal, Left, Proximal	0	0	0	0	1		
Vehicle	Day 43	Ganglion, Dorsal Root, Thoracic - Nerve, Sciatic, Distal, Right, Distal	0	0	0	0	1		
Vehicle	Day 43	Ganglion, Dorsal Root, Thoracic - Nerve, Sciatic, Distal, Right, Proximal	0	0	0	0	1		
Vehicle	Day 43	Ganglion, Dorsal Root, Thoracic - Nerve, Sciatic, Proximal, Left, Distal	0	0	0	0	1		
Vehicle	Day 43	Ganglion, Dorsal Root, Thoracic - Nerve, Sciatic, Proximal, Left, Proximal	0	0	0	0	1		
Vehicle	Day 43	Ganglion, Dorsal Root, Thoracic - Nerve, Sciatic, Proximal, Right, Distal	0	0	0	0	1		
Vehicle	Day 43	Ganglion, Dorsal Root, Thoracic - Nerve, Sciatic, Proximal, Right, Proximal	0	0	0	0	1		
Vehicle	Day 43	Ganglion, Dorsal Root, Thoracic - Nerve, Ulnar, Distal, Left, Distal	0	0	0	0	1		

Results of Evaluating Mean Differences Between Different Tissues Across Timepoint and Group for Log10 AAV2-ITR Target Copies/ug of DNA for Normalized Data

Group	Timepoint	Difference	Mean	StdDev	LowerCLMean	UpperCLMean	DF	tValue	P-Value
Vehicle	Day 43	Ganglion, Dorsal Root, Thoracic - Nerve, Ulnar, Distal, Left, Proximal	0	0	0	0	1		
Vehicle	Day 43	Ganglion, Dorsal Root, Thoracic - Nerve, Ulnar, Distal, Right, Distal	0	0	0	0	1		
Vehicle	Day 43	Ganglion, Dorsal Root, Thoracic - Nerve, Ulnar, Distal, Right, Proximal	0	0	0	0	1		
Vehicle	Day 43	Ganglion, Dorsal Root, Thoracic - Nerve, Ulnar, Proximal, Left, Distal	0	0	0	0	1		
Vehicle	Day 43	Ganglion, Dorsal Root, Thoracic - Nerve, Ulnar, Proximal, Left, Proximal	0	0	0	0	1		
Vehicle	Day 43	Ganglion, Dorsal Root, Thoracic - Nerve, Ulnar, Proximal, Right, Distal	0	0	0	0	1		
Vehicle	Day 43	Ganglion, Dorsal Root, Thoracic - Nerve, Ulnar, Proximal, Right, Proximal	0	0	0	0	1		
Vehicle	Day 43	Ganglion, Dorsal Root, Thoracic - Nerve, Optic	0	0	0	0	1		
Vehicle	Day 43	Ganglion, Dorsal Root, Thoracic - Ovary	0				0		
Vehicle	Day 43	Ganglion, Dorsal Root, Thoracic - Spinal Cord, Cervical	-0.0001082	0.000153018	-0.001483016	0.001266615	1	-1	0.5
Vehicle	Day 43	Ganglion, Dorsal Root, Thoracic - Spinal Cord, Lumbar	0	0	0	0	1		
Vehicle	Day 43	Ganglion, Dorsal Root, Thoracic - Spinal Cord, Sacral	0	0	0	0	1		
Vehicle	Day 43	Ganglion, Dorsal Root, Thoracic - Spinal Cord, Spinal Roots	0	0	0	0	1		
Vehicle	Day 43	Ganglion, Dorsal Root, Thoracic - Spinal Cord, Thoracic	0	0	0	0	1		
Vehicle	Day 43	Ganglion, Dorsal Root, Thoracic - Spleen	-0.000216745	0.000306524	-0.002970753	0.002537263	1	-1	0.5
Vehicle	Day 43	Ganglion, Dorsal Root, Thoracic - Testis	0				0		
Vehicle	Day 43	Heart - Kidney	0	0	0	0	1		
Vehicle	Day 43	Heart - Lung	0	0	0	0	1		
Vehicle	Day 43	Heart - Liver	0	0	0	0	1		
Vehicle	Day 43	Heart - Muscle, Skeletal, Tibialis Anterior Left	0	0	0	0	1		
Vehicle	Day 43	Heart - Muscle, Skeletal, Tibialis Anterior Right	-6.21787E-05	8.7934E-05	-0.000852234	0.000727877	1	-1	0.5
Vehicle	Day 43	Heart - Muscle, Skeletal, Bicep Brachii Left	-0.000143992	0.000203636	-0.001973589	0.001685604	1	-1	0.5
Vehicle	Day 43	Heart - Muscle, Skeletal, Bicep Brachii Right	0	0	0	0	1		
Vehicle	Day 43	Heart - Nerve, Femoral, Distal, Left, Distal	0	0	0	0	1		
Vehicle	Day 43	Heart - Nerve, Femoral, Distal, Left, Proximal	0	0	0	0	1		
Vehicle	Day 43	Heart - Nerve, Femoral, Distal, Right, Distal	0	0	0	0	1		
Vehicle	Day 43	Heart - Nerve, Femoral, Distal, Right, Proximal	0	0	0	0	1		
Vehicle	Day 43	Heart - Nerve, Femoral, Proximal, Left, Distal	0	0	0	0	1		
Vehicle	Day 43	Heart - Nerve, Femoral, Proximal, Left, Proximal	0	0	0	0	1		
Vehicle	Day 43	Heart - Nerve, Femoral, Proximal, Right, Distal	-0.00010276	0.000145325	-0.00140845	0.00120293	1	-1	0.5
Vehicle	Day 43	Heart - Nerve, Femoral, Proximal, Right, Proximal	0	0	0	0	1		
Vehicle	Day 43	Heart - Nerve Median, Distal, Left, Distal	0	0	0	0	1		
Vehicle	Day 43	Heart - Nerve Median, Distal, Left, Proximal	0	0	0	0	1		
Vehicle	Day 43	Heart - Nerve Median, Distal, Right, Distal	0	0	0	0	1		
Vehicle	Day 43	Heart - Nerve Median, Distal, Right, Proximal	0	0	0	0	1		
Vehicle	Day 43	Heart - Nerve Median, Proximal, Left, Distal	0	0	0	0	1		
Vehicle	Day 43	Heart - Nerve Median, Proximal, Left, Proximal	0	0	0	0	1		
Vehicle	Day 43	Heart - Nerve Median, Proximal, Right, Distal	0	0	0	0	1		
Vehicle	Day 43	Heart - Nerve Median, Proximal, Right, Proximal	0	0	0	0	1		
Vehicle	Day 43	Heart - Nerve, Sciatic, Distal, Left, Distal	0	0	0	0	1		
Vehicle	Day 43	Heart - Nerve, Sciatic, Distal, Left, Proximal	0	0	0	0	1		
Vehicle	Day 43	Heart - Nerve, Sciatic, Distal, Right, Distal	0	0	0	0	1		
Vehicle	Day 43	Heart - Nerve, Sciatic, Distal, Right, Proximal	0	0	0	0	1		
Vehicle	Day 43	Heart - Nerve, Sciatic, Proximal, Left, Distal	0	0	0	0	1		
Vehicle	Day 43	Heart - Nerve, Sciatic, Proximal, Left, Proximal	0	0	0	0	1		
Vehicle	Day 43	Heart - Nerve, Sciatic, Proximal, Right, Distal	0	0	0	0	1		
Vehicle	Day 43	Heart - Nerve, Sciatic, Proximal, Right, Proximal	0	0	0	0	1		
Vehicle	Day 43	Heart - Nerve, Ulnar, Distal, Left, Distal	0	0	0	0	1		
Vehicle	Day 43	Heart - Nerve, Ulnar, Distal, Left, Proximal	0	0	0	0	1		
Vehicle	Day 43	Heart - Nerve, Ulnar, Distal, Right, Distal	0	0	0	0	1		
Vehicle	Day 43	Heart - Nerve, Ulnar, Distal, Right, Proximal	0	0	0	0	1		
Vehicle	Day 43	Heart - Nerve, Ulnar, Proximal, Left, Distal	0	0	0	0	1		
Vehicle	Day 43	Heart - Nerve, Ulnar, Proximal, Left, Proximal	0	0	0	0	1		
Vehicle	Day 43	Heart - Nerve, Ulnar, Proximal, Right, Distal	0	0	0	0	1		
Vehicle	Day 43	Heart - Nerve, Ulnar, Proximal, Right, Proximal	0	0	0	0	1		
Vehicle	Day 43	Heart - Nerve, Optic	0	0	0	0	1		
Vehicle	Day 43	Heart - Ovary	0				0		
Vehicle	Day 43	Heart - Spinal Cord, Cervical	-0.0001082	0.000153018	-0.001483016	0.001266615	1	-1	0.5
Vehicle	Day 43	Heart - Spinal Cord, Lumbar	0	0	0	0	1		
Vehicle	Day 43	Heart - Spinal Cord, Sacral	0	0	0	0	1		
Vehicle	Day 43	Heart - Spinal Cord, Spinal Roots	0	0	0	0	1		
Vehicle	Day 43	Heart - Spinal Cord, Thoracic	0	0	0	0	1		
Vehicle	Day 43	Heart - Spleen	-0.000216745	0.000306524	-0.002970753	0.002537263	1	-1	0.5
Vehicle	Day 43	Heart - Testis	0				0		
Vehicle	Day 43	Kidney - Lung	0	0	0	0	1		
Vehicle	Day 43	Kidney - Liver	0	0	0	0	1		
Vehicle	Day 43	Kidney - Muscle, Skeletal, Tibialis Anterior Left	0	0	0	0	1		
Vehicle	Day 43	Kidney - Muscle, Skeletal, Tibialis Anterior Right	-6.21787E-05	8.7934E-05	-0.000852234	0.000727877	1	-1	0.5
Vehicle	Day 43	Kidney - Muscle, Skeletal, Bicep Brachii Left	-0.000143992	0.000203636	-0.001973589	0.001685604	1	-1	0.5
Vehicle	Day 43	Kidney - Muscle, Skeletal, Bicep Brachii Right	0	0	0	0	1		
Vehicle	Day 43	Kidney - Nerve, Femoral, Distal, Left, Distal	0	0	0	0	1		
Vehicle	Day 43	Kidney - Nerve, Femoral, Distal, Left, Proximal	0	0	0	0	1		
Vehicle	Day 43	Kidney - Nerve, Femoral, Distal, Right, Distal	0	0	0	0	1		
Vehicle	Day 43	Kidney - Nerve, Femoral, Distal, Right, Proximal	0	0	0	0	1		
Vehicle	Day 43	Kidney - Nerve, Femoral, Proximal, Left, Distal	0	0	0	0	1		
Vehicle	Day 43	Kidney - Nerve, Femoral, Proximal, Left, Proximal	0	0	0	0	1		
Vehicle	Day 43	Kidney - Nerve, Femoral, Proximal, Right, Distal	-0.00010276	0.000145325	-0.00140845	0.00120293	1	-1	0.5
Vehicle	Day 43	Kidney - Nerve, Femoral, Proximal, Right, Proximal	0	0	0	0	1		
Vehicle	Day 43	Kidney - Nerve Median, Distal, Left, Distal	0	0	0	0	1		
Vehicle	Day 43	Kidney - Nerve Median, Distal, Left, Proximal	0	0	0	0	1		
Vehicle	Day 43	Kidney - Nerve Median, Distal, Right, Distal	0	0	0	0	1		
Vehicle	Day 43	Kidney - Nerve Median, Distal, Right, Proximal	0	0	0	0	1		
Vehicle	Day 43	Kidney - Nerve Median, Proximal, Left, Distal	0	0	0	0	1		
Vehicle	Day 43	Kidney - Nerve Median, Proximal, Left, Proximal	0	0	0	0	1		
Vehicle	Day 43	Kidney - Nerve Median, Proximal, Right, Distal	0	0	0	0	1		
Vehicle	Day 43	Kidney - Nerve Median, Proximal, Right, Proximal	0	0	0	0	1		
Vehicle	Day 43	Kidney - Nerve, Sciatic, Distal, Left, Distal	0	0	0	0	1		
Vehicle	Day 43	Kidney - Nerve, Sciatic, Distal, Left, Proximal	0	0	0	0	1		
Vehicle	Day 43	Kidney - Nerve, Sciatic, Distal, Right, Distal	0	0	0	0	1		
Vehicle	Day 43	Kidney - Nerve, Sciatic, Distal, Right, Proximal	0	0	0	0	1		
Vehicle	Day 43	Kidney - Nerve, Sciatic, Proximal, Left, Distal	0	0	0	0	1		
Vehicle	Day 43	Kidney - Nerve, Sciatic, Proximal, Left, Proximal	0	0	0	0	1		
Vehicle	Day 43	Kidney - Nerve, Sciatic, Proximal, Right, Distal	0	0	0	0	1		

Results of Evaluating Mean Differences Between Different Tissues Across Timepoint and Group for Log10 AAV2-ITR Target Copies/ug of DNA for Normalized Data

Group	Timepoint	Difference	Mean	StdDev	LowerCLMean	UpperCLMean	DF	tValue	P-Value
Vehicle	Day 43	Kidney - Nerve, Sciatic, Proximal, Right, Proximal	0	0	0	0	1		
Vehicle	Day 43	Kidney - Nerve, Ulnar, Distal, Left, Distal	0	0	0	0	1		
Vehicle	Day 43	Kidney - Nerve, Ulnar, Distal, Left, Proximal	0	0	0	0	1		
Vehicle	Day 43	Kidney - Nerve, Ulnar, Distal, Right, Distal	0	0	0	0	1		
Vehicle	Day 43	Kidney - Nerve, Ulnar, Distal, Right, Proximal	0	0	0	0	1		
Vehicle	Day 43	Kidney - Nerve, Ulnar, Proximal, Left, Distal	0	0	0	0	1		
Vehicle	Day 43	Kidney - Nerve, Ulnar, Proximal, Left, Proximal	0	0	0	0	1		
Vehicle	Day 43	Kidney - Nerve, Ulnar, Proximal, Right, Distal	0	0	0	0	1		
Vehicle	Day 43	Kidney - Nerve, Ulnar, Proximal, Right, Proximal	0	0	0	0	1		
Vehicle	Day 43	Kidney - Nerve, Optic	0	0	0	0	1		
Vehicle	Day 43	Kidney - Ovary	0				0		
Vehicle	Day 43	Kidney - Spinal Cord, Cervical	-0.0001082	0.000153018	-0.001483016	0.001266615	1	-1	0.5
Vehicle	Day 43	Kidney - Spinal Cord, Lumbar	0	0	0	0	1		
Vehicle	Day 43	Kidney - Spinal Cord, Sacral	0	0	0	0	1		
Vehicle	Day 43	Kidney - Spinal Cord, Spinal Roots	0	0	0	0	1		
Vehicle	Day 43	Kidney - Spinal Cord, Thoracic	0	0	0	0	1		
Vehicle	Day 43	Kidney - Spleen	-0.000216745	0.000306524	-0.002970753	0.002537263	1	-1	0.5
Vehicle	Day 43	Kidney - Testis	0				0		
Vehicle	Day 43	Lung - Liver	0	0	0	0	1		
Vehicle	Day 43	Lung - Muscle, Skeletal, Tibialis Anterior Left	0	0	0	0	1		
Vehicle	Day 43	Lung - Muscle, Skeletal, Tibialis Anterior Right	-6.21787E-05	8.7934E-05	-0.000852234	0.000727877	1	-1	0.5
Vehicle	Day 43	Lung - Muscle, Skeletal, Bicep Brachii Left	-0.000143992	0.000203636	-0.001973589	0.001685604	1	-1	0.5
Vehicle	Day 43	Lung - Muscle, Skeletal, Bicep Brachii Right	0	0	0	0	1		
Vehicle	Day 43	Lung - Nerve, Femoral, Distal, Left, Distal	0	0	0	0	1		
Vehicle	Day 43	Lung - Nerve, Femoral, Distal, Left, Proximal	0	0	0	0	1		
Vehicle	Day 43	Lung - Nerve, Femoral, Distal, Right, Distal	0	0	0	0	1		
Vehicle	Day 43	Lung - Nerve, Femoral, Distal, Right, Proximal	0	0	0	0	1		
Vehicle	Day 43	Lung - Nerve, Femoral, Proximal, Left, Distal	0	0	0	0	1		
Vehicle	Day 43	Lung - Nerve, Femoral, Proximal, Left, Proximal	0	0	0	0	1		
Vehicle	Day 43	Lung - Nerve, Femoral, Proximal, Right, Distal	-0.00010276	0.000145325	-0.00140845	0.00120293	1	-1	0.5
Vehicle	Day 43	Lung - Nerve, Femoral, Proximal, Right, Proximal	0	0	0	0	1		
Vehicle	Day 43	Lung - Nerve Median, Distal, Left, Distal	0	0	0	0	1		
Vehicle	Day 43	Lung - Nerve Median, Distal, Left, Proximal	0	0	0	0	1		
Vehicle	Day 43	Lung - Nerve Median, Distal, Right, Distal	0	0	0	0	1		
Vehicle	Day 43	Lung - Nerve Median, Distal, Right, Proximal	0	0	0	0	1		
Vehicle	Day 43	Lung - Nerve Median, Proximal, Left, Distal	0	0	0	0	1		
Vehicle	Day 43	Lung - Nerve Median, Proximal, Left, Proximal	0	0	0	0	1		
Vehicle	Day 43	Lung - Nerve Median, Proximal, Right, Distal	0	0	0	0	1		
Vehicle	Day 43	Lung - Nerve Median, Proximal, Right, Proximal	0	0	0	0	1		
Vehicle	Day 43	Lung - Nerve, Sciatic, Distal, Left, Distal	0	0	0	0	1		
Vehicle	Day 43	Lung - Nerve, Sciatic, Distal, Left, Proximal	0	0	0	0	1		
Vehicle	Day 43	Lung - Nerve, Sciatic, Distal, Right, Distal	0	0	0	0	1		
Vehicle	Day 43	Lung - Nerve, Sciatic, Distal, Right, Proximal	0	0	0	0	1		
Vehicle	Day 43	Lung - Nerve, Sciatic, Proximal, Left, Distal	0	0	0	0	1		
Vehicle	Day 43	Lung - Nerve, Sciatic, Proximal, Left, Proximal	0	0	0	0	1		
Vehicle	Day 43	Lung - Nerve, Sciatic, Proximal, Right, Distal	0	0	0	0	1		
Vehicle	Day 43	Lung - Nerve, Sciatic, Proximal, Right, Proximal	0	0	0	0	1		
Vehicle	Day 43	Lung - Nerve, Ulnar, Distal, Left, Distal	0	0	0	0	1		
Vehicle	Day 43	Lung - Nerve, Ulnar, Distal, Left, Proximal	0	0	0	0	1		
Vehicle	Day 43	Lung - Nerve, Ulnar, Distal, Right, Distal	0	0	0	0	1		
Vehicle	Day 43	Lung - Nerve, Ulnar, Distal, Right, Proximal	0	0	0	0	1		
Vehicle	Day 43	Lung - Nerve, Ulnar, Proximal, Left, Distal	0	0	0	0	1		
Vehicle	Day 43	Lung - Nerve, Ulnar, Proximal, Left, Proximal	0	0	0	0	1		
Vehicle	Day 43	Lung - Nerve, Ulnar, Proximal, Right, Distal	0	0	0	0	1		
Vehicle	Day 43	Lung - Nerve, Ulnar, Proximal, Right, Proximal	0	0	0	0	1		
Vehicle	Day 43	Lung - Nerve, Optic	0	0	0	0	1		
Vehicle	Day 43	Lung - Ovary	0				0		
Vehicle	Day 43	Lung - Spinal Cord, Cervical	-0.0001082	0.000153018	-0.001483016	0.001266615	1	-1	0.5
Vehicle	Day 43	Lung - Spinal Cord, Lumbar	0	0	0	0	1		
Vehicle	Day 43	Lung - Spinal Cord, Sacral	0	0	0	0	1		
Vehicle	Day 43	Lung - Spinal Cord, Spinal Roots	0	0	0	0	1		
Vehicle	Day 43	Lung - Spinal Cord, Thoracic	0	0	0	0	1		
Vehicle	Day 43	Lung - Spleen	-0.000216745	0.000306524	-0.002970753	0.002537263	1	-1	0.5
Vehicle	Day 43	Lung - Testis	0				0		
Vehicle	Day 43	Liver - Muscle, Skeletal, Tibialis Anterior Left	0	0	0	0	1		
Vehicle	Day 43	Liver - Muscle, Skeletal, Tibialis Anterior Right	-6.21787E-05	8.7934E-05	-0.000852234	0.000727877	1	-1	0.5
Vehicle	Day 43	Liver - Muscle, Skeletal, Bicep Brachii Left	-0.000143992	0.000203636	-0.001973589	0.001685604	1	-1	0.5
Vehicle	Day 43	Liver - Muscle, Skeletal, Bicep Brachii Right	0	0	0	0	1		
Vehicle	Day 43	Liver - Nerve, Femoral, Distal, Left, Distal	0	0	0	0	1		
Vehicle	Day 43	Liver - Nerve, Femoral, Distal, Left, Proximal	0	0	0	0	1		
Vehicle	Day 43	Liver - Nerve, Femoral, Distal, Right, Distal	0	0	0	0	1		
Vehicle	Day 43	Liver - Nerve, Femoral, Distal, Right, Proximal	0	0	0	0	1		
Vehicle	Day 43	Liver - Nerve, Femoral, Proximal, Left, Distal	0	0	0	0	1		
Vehicle	Day 43	Liver - Nerve, Femoral, Proximal, Left, Proximal	0	0	0	0	1		
Vehicle	Day 43	Liver - Nerve, Femoral, Proximal, Right, Distal	-0.00010276	0.000145325	-0.00140845	0.00120293	1	-1	0.5
Vehicle	Day 43	Liver - Nerve, Femoral, Proximal, Right, Proximal	0	0	0	0	1		
Vehicle	Day 43	Liver - Nerve Median, Distal, Left, Distal	0	0	0	0	1		
Vehicle	Day 43	Liver - Nerve Median, Distal, Left, Proximal	0	0	0	0	1		
Vehicle	Day 43	Liver - Nerve Median, Distal, Right, Distal	0	0	0	0	1		
Vehicle	Day 43	Liver - Nerve Median, Distal, Right, Proximal	0	0	0	0	1		
Vehicle	Day 43	Liver - Nerve Median, Proximal, Left, Distal	0	0	0	0	1		
Vehicle	Day 43	Liver - Nerve Median, Proximal, Left, Proximal	0	0	0	0	1		
Vehicle	Day 43	Liver - Nerve Median, Proximal, Right, Distal	0	0	0	0	1		
Vehicle	Day 43	Liver - Nerve Median, Proximal, Right, Proximal	0	0	0	0	1		
Vehicle	Day 43	Liver - Nerve, Sciatic, Distal, Left, Distal	0	0	0	0	1		
Vehicle	Day 43	Liver - Nerve, Sciatic, Distal, Left, Proximal	0	0	0	0	1		
Vehicle	Day 43	Liver - Nerve, Sciatic, Distal, Right, Distal	0	0	0	0	1		
Vehicle	Day 43	Liver - Nerve, Sciatic, Distal, Right, Proximal	0	0	0	0	1		
Vehicle	Day 43	Liver - Nerve, Sciatic, Proximal, Left, Distal	0	0	0	0	1		
Vehicle	Day 43	Liver - Nerve, Sciatic, Proximal, Left, Proximal	0	0	0	0	1		
Vehicle	Day 43	Liver - Nerve, Sciatic, Proximal, Right, Distal	0	0	0	0	1		
Vehicle	Day 43	Liver - Nerve, Sciatic, Proximal, Right, Proximal	0	0	0	0	1		
Vehicle	Day 43	Liver - Nerve, Ulnar, Distal, Left, Distal	0	0	0	0	1		

Results of Evaluating Mean Differences Between Different Tissues Across Timepoint and Group for Log10 AAV2-ITR Target Copies/ug of DNA for Normalized Data

Group	Timepoint	Difference	Mean	StdDev	LowerCLMean	UpperCLMean	DF	tValue	P-Value
Vehicle	Day 43	Liver - Nerve, Ulnar, Distal, Left, Proximal	0	0	0	0	1		
Vehicle	Day 43	Liver - Nerve, Ulnar, Distal, Right, Distal	0	0	0	0	1		
Vehicle	Day 43	Liver - Nerve, Ulnar, Distal, Right, Proximal	0	0	0	0	1		
Vehicle	Day 43	Liver - Nerve, Ulnar, Proximal, Left, Distal	0	0	0	0	1		
Vehicle	Day 43	Liver - Nerve, Ulnar, Proximal, Left, Proximal	0	0	0	0	1		
Vehicle	Day 43	Liver - Nerve, Ulnar, Proximal, Right, Distal	0	0	0	0	1		
Vehicle	Day 43	Liver - Nerve, Ulnar, Proximal, Right, Proximal	0	0	0	0	1		
Vehicle	Day 43	Liver - Nerve, Optic	0	0	0	0	1		
Vehicle	Day 43	Liver - Ovary	0				0		
Vehicle	Day 43	Liver - Spinal Cord, Cervical	-0.0001082	0.000153018	-0.001483016	0.001266615	1	-1	0.5
Vehicle	Day 43	Liver - Spinal Cord, Lumbar	0	0	0	0	1		
Vehicle	Day 43	Liver - Spinal Cord, Sacral	0	0	0	0	1		
Vehicle	Day 43	Liver - Spinal Cord, Spinal Roots	0	0	0	0	1		
Vehicle	Day 43	Liver - Spinal Cord, Thoracic	0	0	0	0	1		
Vehicle	Day 43	Liver - Spleen	-0.000216745	0.000306524	-0.002970753	0.002537263	1	-1	0.5
Vehicle	Day 43	Liver - Testis	0				0		
Vehicle	Day 43	Muscle, Skeletal, Tibialis Anterior Left - Muscle, Skeletal, Tibialis Anterior Right	-6.21787E-05	8.7934E-05	-0.000852234	0.000727877	1	-1	0.5
Vehicle	Day 43	Muscle, Skeletal, Tibialis Anterior Left - Muscle, Skeletal, Bicep Brachii Left	-0.000143992	0.000203636	-0.001973589	0.001685604	1	-1	0.5
Vehicle	Day 43	Muscle, Skeletal, Tibialis Anterior Left - Muscle, Skeletal, Bicep Brachii Right	0	0	0	0	1		
Vehicle	Day 43	Muscle, Skeletal, Tibialis Anterior Left - Nerve, Femoral, Distal, Left, Distal	0	0	0	0	1		
Vehicle	Day 43	Muscle, Skeletal, Tibialis Anterior Left - Nerve, Femoral, Distal, Left, Proximal	0	0	0	0	1		
Vehicle	Day 43	Muscle, Skeletal, Tibialis Anterior Left - Nerve, Femoral, Distal, Right, Distal	0	0	0	0	1		
Vehicle	Day 43	Muscle, Skeletal, Tibialis Anterior Left - Nerve, Femoral, Distal, Right, Proximal	0	0	0	0	1		
Vehicle	Day 43	Muscle, Skeletal, Tibialis Anterior Left - Nerve, Femoral, Proximal, Left, Distal	0	0	0	0	1		
Vehicle	Day 43	Muscle, Skeletal, Tibialis Anterior Left - Nerve, Femoral, Proximal, Left, Proximal	0	0	0	0	1		
Vehicle	Day 43	Muscle, Skeletal, Tibialis Anterior Left - Nerve, Femoral, Proximal, Right, Distal	-0.00010276	0.000145325	-0.00140845	0.00120293	1	-1	0.5
Vehicle	Day 43	Muscle, Skeletal, Tibialis Anterior Left - Nerve, Femoral, Proximal, Right, Proximal	0	0	0	0	1		
Vehicle	Day 43	Muscle, Skeletal, Tibialis Anterior Left - Nerve Median, Distal, Left, Distal	0	0	0	0	1		
Vehicle	Day 43	Muscle, Skeletal, Tibialis Anterior Left - Nerve Median, Distal, Left, Proximal	0	0	0	0	1		
Vehicle	Day 43	Muscle, Skeletal, Tibialis Anterior Left - Nerve Median, Distal, Right, Distal	0	0	0	0	1		
Vehicle	Day 43	Muscle, Skeletal, Tibialis Anterior Left - Nerve Median, Distal, Right, Proximal	0	0	0	0	1		
Vehicle	Day 43	Muscle, Skeletal, Tibialis Anterior Left - Nerve Median, Proximal, Left, Distal	0	0	0	0	1		
Vehicle	Day 43	Muscle, Skeletal, Tibialis Anterior Left - Nerve Median, Proximal, Left, Proximal	0	0	0	0	1		
Vehicle	Day 43	Muscle, Skeletal, Tibialis Anterior Left - Nerve Median, Proximal, Right, Distal	0	0	0	0	1		
Vehicle	Day 43	Muscle, Skeletal, Tibialis Anterior Left - Nerve Median, Proximal, Right, Proximal	0	0	0	0	1		
Vehicle	Day 43	Muscle, Skeletal, Tibialis Anterior Left - Nerve, Sciatic, Distal, Left, Distal	0	0	0	0	1		
Vehicle	Day 43	Muscle, Skeletal, Tibialis Anterior Left - Nerve, Sciatic, Distal, Left, Proximal	0	0	0	0	1		
Vehicle	Day 43	Muscle, Skeletal, Tibialis Anterior Left - Nerve, Sciatic, Distal, Right, Distal	0	0	0	0	1		
Vehicle	Day 43	Muscle, Skeletal, Tibialis Anterior Left - Nerve, Sciatic, Distal, Right, Proximal	0	0	0	0	1		
Vehicle	Day 43	Muscle, Skeletal, Tibialis Anterior Left - Nerve, Sciatic, Proximal, Left, Distal	0	0	0	0	1		
Vehicle	Day 43	Muscle, Skeletal, Tibialis Anterior Left - Nerve, Sciatic, Proximal, Left, Proximal	0	0	0	0	1		
Vehicle	Day 43	Muscle, Skeletal, Tibialis Anterior Left - Nerve, Sciatic, Proximal, Right, Distal	0	0	0	0	1		
Vehicle	Day 43	Muscle, Skeletal, Tibialis Anterior Left - Nerve, Sciatic, Proximal, Right, Proximal	0	0	0	0	1		
Vehicle	Day 43	Muscle, Skeletal, Tibialis Anterior Left - Nerve, Ulnar, Distal, Left, Distal	0	0	0	0	1		
Vehicle	Day 43	Muscle, Skeletal, Tibialis Anterior Left - Nerve, Ulnar, Distal, Left, Proximal	0	0	0	0	1		
Vehicle	Day 43	Muscle, Skeletal, Tibialis Anterior Left - Nerve, Ulnar, Distal, Right, Distal	0	0	0	0	1		
Vehicle	Day 43	Muscle, Skeletal, Tibialis Anterior Left - Nerve, Ulnar, Distal, Right, Proximal	0	0	0	0	1		
Vehicle	Day 43	Muscle, Skeletal, Tibialis Anterior Left - Nerve, Ulnar, Proximal, Left, Distal	0	0	0	0	1		
Vehicle	Day 43	Muscle, Skeletal, Tibialis Anterior Left - Nerve, Ulnar, Proximal, Left, Proximal	0	0	0	0	1		
Vehicle	Day 43	Muscle, Skeletal, Tibialis Anterior Left - Nerve, Ulnar, Proximal, Right, Distal	0	0	0	0	1		
Vehicle	Day 43	Muscle, Skeletal, Tibialis Anterior Left - Nerve, Ulnar, Proximal, Right, Proximal	0	0	0	0	1		
Vehicle	Day 43	Muscle, Skeletal, Tibialis Anterior Left - Nerve, Optic	0	0	0	0	1		
Vehicle	Day 43	Muscle, Skeletal, Tibialis Anterior Left - Ovary	0				0		
Vehicle	Day 43	Muscle, Skeletal, Tibialis Anterior Left - Spinal Cord, Cervical	-0.0001082	0.000153018	-0.001483016	0.001266615	1	-1	0.5
Vehicle	Day 43	Muscle, Skeletal, Tibialis Anterior Left - Spinal Cord, Lumbar	0	0	0	0	1		
Vehicle	Day 43	Muscle, Skeletal, Tibialis Anterior Left - Spinal Cord, Sacral	0	0	0	0	1		
Vehicle	Day 43	Muscle, Skeletal, Tibialis Anterior Left - Spinal Cord, Spinal Roots	0	0	0	0	1		
Vehicle	Day 43	Muscle, Skeletal, Tibialis Anterior Left - Spinal Cord, Thoracic	0	0	0	0	1		
Vehicle	Day 43	Muscle, Skeletal, Tibialis Anterior Left - Spleen	-0.000216745	0.000306524	-0.002970753	0.002537263	1	-1	0.5
Vehicle	Day 43	Muscle, Skeletal, Tibialis Anterior Left - Testis	0				0		
Vehicle	Day 43	Muscle, Skeletal, Tibialis Anterior Right - Muscle, Skeletal, Bicep Brachii Left	-8.18136E-05	0.00029157	-0.002701465	0.002537838	1	-0.396823935	0.75950708
Vehicle	Day 43	Muscle, Skeletal, Tibialis Anterior Right - Muscle, Skeletal, Bicep Brachii Right	6.21787E-05	8.7934E-05	-0.000727877	0.000852234	1	1	0.5
Vehicle	Day 43	Muscle, Skeletal, Tibialis Anterior Right - Nerve, Femoral, Distal, Left, Distal	6.21787E-05	8.7934E-05	-0.000727877	0.000852234	1	1	0.5
Vehicle	Day 43	Muscle, Skeletal, Tibialis Anterior Right - Nerve, Femoral, Distal, Left, Proximal	6.21787E-05	8.7934E-05	-0.000727877	0.000852234	1	1	0.5
Vehicle	Day 43	Muscle, Skeletal, Tibialis Anterior Right - Nerve, Femoral, Distal, Right, Distal	6.21787E-05	8.7934E-05	-0.000727877	0.000852234	1	1	0.5
Vehicle	Day 43	Muscle, Skeletal, Tibialis Anterior Right - Nerve, Femoral, Distal, Right, Proximal	6.21787E-05	8.7934E-05	-0.000727877	0.000852234	1	1	0.5
Vehicle	Day 43	Muscle, Skeletal, Tibialis Anterior Right - Nerve, Femoral, Proximal, Left, Distal	6.21787E-05	8.7934E-05	-0.000727877	0.000852234	1	1	0.5
Vehicle	Day 43	Muscle, Skeletal, Tibialis Anterior Right - Nerve, Femoral, Proximal, Left, Proximal	6.21787E-05	8.7934E-05	-0.000727877	0.000852234	1	1	0.5
Vehicle	Day 43	Muscle, Skeletal, Tibialis Anterior Right - Nerve, Femoral, Proximal, Right, Distal	-4.05813E-05	5.73907E-05	-0.000556216	0.000475053	1	-1	0.5
Vehicle	Day 43	Muscle, Skeletal, Tibialis Anterior Right - Nerve, Femoral, Proximal, Right, Proximal	6.21787E-05	8.7934E-05	-0.000727877	0.000852234	1	1	0.5
Vehicle	Day 43	Muscle, Skeletal, Tibialis Anterior Right - Nerve Median, Distal, Left, Distal	6.21787E-05	8.7934E-05	-0.000727877	0.000852234	1	1	0.5
Vehicle	Day 43	Muscle, Skeletal, Tibialis Anterior Right - Nerve Median, Distal, Left, Proximal	6.21787E-05	8.7934E-05	-0.000727877	0.000852234	1	1	0.5
Vehicle	Day 43	Muscle, Skeletal, Tibialis Anterior Right - Nerve Median, Distal, Right, Distal	6.21787E-05	8.7934E-05	-0.000727877	0.000852234	1	1	0.5
Vehicle	Day 43	Muscle, Skeletal, Tibialis Anterior Right - Nerve Median, Distal, Right, Proximal	6.21787E-05	8.7934E-05	-0.000727877	0.000852234	1	1	0.5
Vehicle	Day 43	Muscle, Skeletal, Tibialis Anterior Right - Nerve Median, Proximal, Left, Distal	6.21787E-05	8.7934E-05	-0.000727877	0.000852234	1	1	0.5
Vehicle	Day 43	Muscle, Skeletal, Tibialis Anterior Right - Nerve Median, Proximal, Left, Proximal	6.21787E-05	8.7934E-05	-0.000727877	0.000852234	1	1	0.5
Vehicle	Day 43	Muscle, Skeletal, Tibialis Anterior Right - Nerve Median, Proximal, Right, Distal	6.21787E-05	8.7934E-05	-0.000727877	0.000852234	1	1	0.5
Vehicle	Day 43	Muscle, Skeletal, Tibialis Anterior Right - Nerve Median, Proximal, Right, Proximal	6.21787E-05	8.7934E-05	-0.000727877	0.000852234	1	1	0.5
Vehicle	Day 43	Muscle, Skeletal, Tibialis Anterior Right - Nerve, Sciatic, Distal, Left, Distal	6.21787E-05	8.7934E-05	-0.000727877	0.000852234	1	1	0.5
Vehicle	Day 43	Muscle, Skeletal, Tibialis Anterior Right - Nerve, Sciatic, Distal, Left, Proximal	6.21787E-05	8.7934E-05	-0.000727877	0.000852234	1	1	0.5
Vehicle	Day 43	Muscle, Skeletal, Tibialis Anterior Right - Nerve, Sciatic, Distal, Right, Distal	6.21787E-05	8.7934E-05	-0.000727877	0.000852234	1	1	0.5
Vehicle	Day 43	Muscle, Skeletal, Tibialis Anterior Right - Nerve, Sciatic, Distal, Right, Proximal	6.21787E-05	8.7934E-05	-0.000727877	0.000852234	1	1	0.5
Vehicle	Day 43	Muscle, Skeletal, Tibialis Anterior Right - Nerve, Sciatic, Proximal, Left, Distal	6.21787E-05	8.7934E-05	-0.000727877	0.000852234	1	1	0.5
Vehicle	Day 43	Muscle, Skeletal, Tibialis Anterior Right - Nerve, Sciatic, Proximal, Left, Proximal	6.21787E-05	8.7934E-05	-0.000727877	0.000852234	1	1	0.5
Vehicle	Day 43	Muscle, Skeletal, Tibialis Anterior Right - Nerve, Sciatic, Proximal, Right, Distal	6.21787E-05	8.7934E-05	-0.000727877	0.000852234	1	1	0.5
Vehicle	Day 43	Muscle, Skeletal, Tibialis Anterior Right - Nerve, Sciatic, Proximal, Right, Proximal	6.21787E-05	8.7934E-05	-0.000727877	0.000852234	1	1	0.5
Vehicle	Day 43	Muscle, Skeletal, Tibialis Anterior Right - Nerve, Ulnar, Distal, Left, Distal	6.21787E-05	8.7934E-05	-0.000727877	0.000852234	1	1	0.5
Vehicle	Day 43	Muscle, Skeletal, Tibialis Anterior Right - Nerve, Ulnar, Distal, Left, Proximal	6.21787E-05	8.7934E-05	-0.000727877	0.000852234	1	1	0.5
Vehicle	Day 43	Muscle, Skeletal, Tibialis Anterior Right - Nerve, Ulnar, Distal, Right, Distal	6.21787E-05	8.7934E-05	-0.000727877	0.000852234	1	1	0.5
Vehicle	Day 43	Muscle, Skeletal, Tibialis Anterior Right - Nerve, Ulnar, Distal, Right, Proximal	6.21787E-05	8.7934E-05	-0.000727877	0.000852234	1	1	0.5
Vehicle	Day 43	Muscle, Skeletal, Tibialis Anterior Right - Nerve, Ulnar, Proximal, Left, Distal	6.21787E-05	8.7934E-05	-0.000727877	0.000852234	1	1	0.5
Vehicle	Day 43	Muscle, Skeletal, Tibialis Anterior Right - Nerve, Ulnar, Proximal, Left, Proximal	6.21787E-05	8.7934E-05	-0.000727877	0.000852234	1	1	0.5
Vehicle	Day 43	Muscle, Skeletal, Tibialis Anterior Right - Nerve, Ulnar, Proximal, Right, Distal	6.21787E-05	8.7934E-05	-0.000727877	0.000852234	1	1	0.5

Results of Evaluating Mean Differences Between Different Tissues Across Timepoint and Group for Log10 AAV2-ITR Target Copies/ug of DNA for Normalized Data

Group	Timepoint	Difference	Mean	StdDev	LowerCLMean	UpperCLMean	DF	tValue	P-Value
Vehicle	Day 43	Muscle, Skeletal, Tibialis Anterior Right - Nerve, Ulnar, Proximal, Right, Proximal	6.21787E-05	8.7934E-05	-0.000727877	0.000852234	1	1	0.5
Vehicle	Day 43	Muscle, Skeletal, Tibialis Anterior Right - Nerve, Optic	6.21787E-05	8.7934E-05	-0.000727877	0.000852234	1	1	0.5
Vehicle	Day 43	Muscle, Skeletal, Tibialis Anterior Right - Ovary	0				0		
Vehicle	Day 43	Muscle, Skeletal, Tibialis Anterior Right - Spinal Cord, Cervical	-4.60216E-05	0.000240952	-0.002210893	0.00211885	1	-0.270113024	0.83204877
Vehicle	Day 43	Muscle, Skeletal, Tibialis Anterior Right - Spinal Cord, Lumbar	6.21787E-05	8.7934E-05	-0.000727877	0.000852234	1	1	0.5
Vehicle	Day 43	Muscle, Skeletal, Tibialis Anterior Right - Spinal Cord, Sacral	6.21787E-05	8.7934E-05	-0.000727877	0.000852234	1	1	0.5
Vehicle	Day 43	Muscle, Skeletal, Tibialis Anterior Right - Spinal Cord, Spinal Roots	6.21787E-05	8.7934E-05	-0.000727877	0.000852234	1	1	0.5
Vehicle	Day 43	Muscle, Skeletal, Tibialis Anterior Right - Spinal Cord, Thoracic	6.21787E-05	8.7934E-05	-0.000727877	0.000852234	1	1	0.5
Vehicle	Day 43	Muscle, Skeletal, Tibialis Anterior Right - Spleen	-0.000154566	0.00021859	-0.002118519	0.001809386	1	-1	0.5
Vehicle	Day 43	Muscle, Skeletal, Tibialis Anterior Right - Testis	0.000124357				0		
Vehicle	Day 43	Muscle, Skeletal, Bicep Brachii Left - Muscle, Skeletal, Bicep Brachii Right	0.000143992	0.000203636	-0.001685604	0.001973589	1	1	0.5
Vehicle	Day 43	Muscle, Skeletal, Bicep Brachii Left - Nerve, Femoral, Distal, Left, Distal	0.000143992	0.000203636	-0.001685604	0.001973589	1	1	0.5
Vehicle	Day 43	Muscle, Skeletal, Bicep Brachii Left - Nerve, Femoral, Distal, Left, Proximal	0.000143992	0.000203636	-0.001685604	0.001973589	1	1	0.5
Vehicle	Day 43	Muscle, Skeletal, Bicep Brachii Left - Nerve, Femoral, Distal, Right, Distal	0.000143992	0.000203636	-0.001685604	0.001973589	1	1	0.5
Vehicle	Day 43	Muscle, Skeletal, Bicep Brachii Left - Nerve, Femoral, Distal, Right, Proximal	0.000143992	0.000203636	-0.001685604	0.001973589	1	1	0.5
Vehicle	Day 43	Muscle, Skeletal, Bicep Brachii Left - Nerve, Femoral, Proximal, Left, Distal	0.000143992	0.000203636	-0.001685604	0.001973589	1	1	0.5
Vehicle	Day 43	Muscle, Skeletal, Bicep Brachii Left - Nerve, Femoral, Proximal, Left, Proximal	0.000143992	0.000203636	-0.001685604	0.001973589	1	1	0.5
Vehicle	Day 43	Muscle, Skeletal, Bicep Brachii Left - Nerve, Femoral, Proximal, Right, Distal	4.12323E-05	0.000348961	-0.003094054	0.003176519	1	0.167099816	0.894594807
Vehicle	Day 43	Muscle, Skeletal, Bicep Brachii Left - Nerve, Femoral, Proximal, Right, Proximal	0.000143992	0.000203636	-0.001685604	0.001973589	1	1	0.5
Vehicle	Day 43	Muscle, Skeletal, Bicep Brachii Left - Nerve Median, Distal, Left, Distal	0.000143992	0.000203636	-0.001685604	0.001973589	1	1	0.5
Vehicle	Day 43	Muscle, Skeletal, Bicep Brachii Left - Nerve Median, Distal, Left, Proximal	0.000143992	0.000203636	-0.001685604	0.001973589	1	1	0.5
Vehicle	Day 43	Muscle, Skeletal, Bicep Brachii Left - Nerve Median, Distal, Right, Distal	0.000143992	0.000203636	-0.001685604	0.001973589	1	1	0.5
Vehicle	Day 43	Muscle, Skeletal, Bicep Brachii Left - Nerve Median, Distal, Right, Proximal	0.000143992	0.000203636	-0.001685604	0.001973589	1	1	0.5
Vehicle	Day 43	Muscle, Skeletal, Bicep Brachii Left - Nerve Median, Proximal, Left, Distal	0.000143992	0.000203636	-0.001685604	0.001973589	1	1	0.5
Vehicle	Day 43	Muscle, Skeletal, Bicep Brachii Left - Nerve Median, Proximal, Left, Proximal	0.000143992	0.000203636	-0.001685604	0.001973589	1	1	0.5
Vehicle	Day 43	Muscle, Skeletal, Bicep Brachii Left - Nerve Median, Proximal, Right, Distal	0.000143992	0.000203636	-0.001685604	0.001973589	1	1	0.5
Vehicle	Day 43	Muscle, Skeletal, Bicep Brachii Left - Nerve Median, Proximal, Right, Proximal	0.000143992	0.000203636	-0.001685604	0.001973589	1	1	0.5
Vehicle	Day 43	Muscle, Skeletal, Bicep Brachii Left - Nerve, Sciatic, Distal, Left, Distal	0.000143992	0.000203636	-0.001685604	0.001973589	1	1	0.5
Vehicle	Day 43	Muscle, Skeletal, Bicep Brachii Left - Nerve, Sciatic, Distal, Left, Proximal	0.000143992	0.000203636	-0.001685604	0.001973589	1	1	0.5
Vehicle	Day 43	Muscle, Skeletal, Bicep Brachii Left - Nerve, Sciatic, Distal, Right, Distal	0.000143992	0.000203636	-0.001685604	0.001973589	1	1	0.5
Vehicle	Day 43	Muscle, Skeletal, Bicep Brachii Left - Nerve, Sciatic, Distal, Right, Proximal	0.000143992	0.000203636	-0.001685604	0.001973589	1	1	0.5
Vehicle	Day 43	Muscle, Skeletal, Bicep Brachii Left - Nerve, Sciatic, Proximal, Left, Distal	0.000143992	0.000203636	-0.001685604	0.001973589	1	1	0.5
Vehicle	Day 43	Muscle, Skeletal, Bicep Brachii Left - Nerve, Sciatic, Proximal, Left, Proximal	0.000143992	0.000203636	-0.001685604	0.001973589	1	1	0.5
Vehicle	Day 43	Muscle, Skeletal, Bicep Brachii Left - Nerve, Sciatic, Proximal, Right, Distal	0.000143992	0.000203636	-0.001685604	0.001973589	1	1	0.5
Vehicle	Day 43	Muscle, Skeletal, Bicep Brachii Left - Nerve, Sciatic, Proximal, Right, Proximal	0.000143992	0.000203636	-0.001685604	0.001973589	1	1	0.5
Vehicle	Day 43	Muscle, Skeletal, Bicep Brachii Left - Nerve, Ulnar, Distal, Left, Distal	0.000143992	0.000203636	-0.001685604	0.001973589	1	1	0.5
Vehicle	Day 43	Muscle, Skeletal, Bicep Brachii Left - Nerve, Ulnar, Distal, Left, Proximal	0.000143992	0.000203636	-0.001685604	0.001973589	1	1	0.5
Vehicle	Day 43	Muscle, Skeletal, Bicep Brachii Left - Nerve, Ulnar, Distal, Right, Distal	0.000143992	0.000203636	-0.001685604	0.001973589	1	1	0.5
Vehicle	Day 43	Muscle, Skeletal, Bicep Brachii Left - Nerve, Ulnar, Distal, Right, Proximal	0.000143992	0.000203636	-0.001685604	0.001973589	1	1	0.5
Vehicle	Day 43	Muscle, Skeletal, Bicep Brachii Left - Nerve, Ulnar, Proximal, Left, Distal	0.000143992	0.000203636	-0.001685604	0.001973589	1	1	0.5
Vehicle	Day 43	Muscle, Skeletal, Bicep Brachii Left - Nerve, Ulnar, Proximal, Left, Proximal	0.000143992	0.000203636	-0.001685604	0.001973589	1	1	0.5
Vehicle	Day 43	Muscle, Skeletal, Bicep Brachii Left - Nerve, Ulnar, Proximal, Right, Distal	0.000143992	0.000203636	-0.001685604	0.001973589	1	1	0.5
Vehicle	Day 43	Muscle, Skeletal, Bicep Brachii Left - Nerve, Ulnar, Proximal, Right, Proximal	0.000143992	0.000203636	-0.001685604	0.001973589	1	1	0.5
Vehicle	Day 43	Muscle, Skeletal, Bicep Brachii Left - Nerve, Optic	0.000143992	0.000203636	-0.001685604	0.001973589	1	1	0.5
Vehicle	Day 43	Muscle, Skeletal, Bicep Brachii Left - Ovary	0.000287985				0		
Vehicle	Day 43	Muscle, Skeletal, Bicep Brachii Left - Spinal Cord, Cervical	3.5792E-05	5.06176E-05	-0.000418989	0.000490573	1	1	0.5
Vehicle	Day 43	Muscle, Skeletal, Bicep Brachii Left - Spinal Cord, Lumbar	0.000143992	0.000203636	-0.001685604	0.001973589	1	1	0.5
Vehicle	Day 43	Muscle, Skeletal, Bicep Brachii Left - Spinal Cord, Sacral	0.000143992	0.000203636	-0.001685604	0.001973589	1	1	0.5
Vehicle	Day 43	Muscle, Skeletal, Bicep Brachii Left - Spinal Cord, Spinal Roots	0.000143992	0.000203636	-0.001685604	0.001973589	1	1	0.5
Vehicle	Day 43	Muscle, Skeletal, Bicep Brachii Left - Spinal Cord, Thoracic	0.000143992	0.000203636	-0.001685604	0.001973589	1	1	0.5
Vehicle	Day 43	Muscle, Skeletal, Bicep Brachii Left - Spleen	-7.27528E-05	0.00051016	-0.004656357	0.004510851	1	-0.201677928	0.873307298
Vehicle	Day 43	Muscle, Skeletal, Bicep Brachii Left - Testis	0				0		
Vehicle	Day 43	Muscle, Skeletal, Bicep Brachii Right - Nerve, Femoral, Distal, Left, Distal	0	0	0	0	1		
Vehicle	Day 43	Muscle, Skeletal, Bicep Brachii Right - Nerve, Femoral, Distal, Left, Proximal	0	0	0	0	1		
Vehicle	Day 43	Muscle, Skeletal, Bicep Brachii Right - Nerve, Femoral, Distal, Right, Distal	0	0	0	0	1		
Vehicle	Day 43	Muscle, Skeletal, Bicep Brachii Right - Nerve, Femoral, Distal, Right, Proximal	0	0	0	0	1		
Vehicle	Day 43	Muscle, Skeletal, Bicep Brachii Right - Nerve, Femoral, Proximal, Left, Distal	0	0	0	0	1		
Vehicle	Day 43	Muscle, Skeletal, Bicep Brachii Right - Nerve, Femoral, Proximal, Left, Proximal	0	0	0	0	1		
Vehicle	Day 43	Muscle, Skeletal, Bicep Brachii Right - Nerve, Femoral, Proximal, Right, Distal	-0.00010276	0.000145325	-0.00140845	0.00120293	1	-1	0.5
Vehicle	Day 43	Muscle, Skeletal, Bicep Brachii Right - Nerve, Femoral, Proximal, Right, Proximal	0	0	0	0	1		
Vehicle	Day 43	Muscle, Skeletal, Bicep Brachii Right - Nerve Median, Distal, Left, Distal	0	0	0	0	1		
Vehicle	Day 43	Muscle, Skeletal, Bicep Brachii Right - Nerve Median, Distal, Left, Proximal	0	0	0	0	1		
Vehicle	Day 43	Muscle, Skeletal, Bicep Brachii Right - Nerve Median, Distal, Right, Distal	0	0	0	0	1		
Vehicle	Day 43	Muscle, Skeletal, Bicep Brachii Right - Nerve Median, Distal, Right, Proximal	0	0	0	0	1		
Vehicle	Day 43	Muscle, Skeletal, Bicep Brachii Right - Nerve Median, Proximal, Left, Distal	0	0	0	0	1		
Vehicle	Day 43	Muscle, Skeletal, Bicep Brachii Right - Nerve Median, Proximal, Left, Proximal	0	0	0	0	1		
Vehicle	Day 43	Muscle, Skeletal, Bicep Brachii Right - Nerve Median, Proximal, Right, Distal	0	0	0	0	1		
Vehicle	Day 43	Muscle, Skeletal, Bicep Brachii Right - Nerve Median, Proximal, Right, Proximal	0	0	0	0	1		
Vehicle	Day 43	Muscle, Skeletal, Bicep Brachii Right - Nerve, Sciatic, Distal, Left, Distal	0	0	0	0	1		
Vehicle	Day 43	Muscle, Skeletal, Bicep Brachii Right - Nerve, Sciatic, Distal, Left, Proximal	0	0	0	0	1		
Vehicle	Day 43	Muscle, Skeletal, Bicep Brachii Right - Nerve, Sciatic, Distal, Right, Distal	0	0	0	0	1		
Vehicle	Day 43	Muscle, Skeletal, Bicep Brachii Right - Nerve, Sciatic, Distal, Right, Proximal	0	0	0	0	1		
Vehicle	Day 43	Muscle, Skeletal, Bicep Brachii Right - Nerve, Sciatic, Proximal, Left, Distal	0	0	0	0	1		
Vehicle	Day 43	Muscle, Skeletal, Bicep Brachii Right - Nerve, Sciatic, Proximal, Left, Proximal	0	0	0	0	1		
Vehicle	Day 43	Muscle, Skeletal, Bicep Brachii Right - Nerve, Sciatic, Proximal, Right, Distal	0	0	0	0	1		
Vehicle	Day 43	Muscle, Skeletal, Bicep Brachii Right - Nerve, Sciatic, Proximal, Right, Proximal	0	0	0	0	1		
Vehicle	Day 43	Muscle, Skeletal, Bicep Brachii Right - Nerve, Ulnar, Distal, Left, Distal	0	0	0	0	1		
Vehicle	Day 43	Muscle, Skeletal, Bicep Brachii Right - Nerve, Ulnar, Distal, Left, Proximal	0	0	0	0	1		
Vehicle	Day 43	Muscle, Skeletal, Bicep Brachii Right - Nerve, Ulnar, Distal, Right, Distal	0	0	0	0	1		
Vehicle	Day 43	Muscle, Skeletal, Bicep Brachii Right - Nerve, Ulnar, Distal, Right, Proximal	0	0	0	0	1		
Vehicle	Day 43	Muscle, Skeletal, Bicep Brachii Right - Nerve, Ulnar, Proximal, Left, Distal	0	0	0	0	1		
Vehicle	Day 43	Muscle, Skeletal, Bicep Brachii Right - Nerve, Ulnar, Proximal, Left, Proximal	0	0	0	0	1		
Vehicle	Day 43	Muscle, Skeletal, Bicep Brachii Right - Nerve, Ulnar, Proximal, Right, Distal	0	0	0	0	1		
Vehicle	Day 43	Muscle, Skeletal, Bicep Brachii Right - Nerve, Ulnar, Proximal, Right, Proximal	0	0	0	0	1		
Vehicle	Day 43	Muscle, Skeletal, Bicep Brachii Right - Nerve, Optic	0	0	0	0	1		
Vehicle	Day 43	Muscle, Skeletal, Bicep Brachii Right - Ovary	0				0		
Vehicle	Day 43	Muscle, Skeletal, Bicep Brachii Right - Spinal Cord, Cervical	-0.0001082	0.000153018	-0.001483016	0.001266615	1	-1	0.5
Vehicle	Day 43	Muscle, Skeletal, Bicep Brachii Right - Spinal Cord, Lumbar	0	0	0	0	1		
Vehicle	Day 43	Muscle, Skeletal, Bicep Brachii Right - Spinal Cord, Sacral	0	0	0	0	1		
Vehicle	Day 43	Muscle, Skeletal, Bicep Brachii Right - Spinal Cord, Spinal Roots	0	0	0	0	1		
Vehicle	Day 43	Muscle, Skeletal, Bicep Brachii Right - Spinal Cord, Thoracic	0	0	0	0	1		
Vehicle	Day 43	Muscle, Skeletal, Bicep Brachii Right - Spleen	-0.000216745	0.000306524	-0.002970753	0.002537263	1	-1	0.5
Vehicle	Day 43	Muscle, Skeletal, Bicep Brachii Right - Testis	0				0		

Results of Evaluating Mean Differences Between Different Tissues Across Timepoint and Group for Log10 AAV2-ITR Target Copies/ug of DNA for Normalized Data

Group	Timepoint	Difference	Mean	StdDev	LowerCLMean	UpperCLMean	DF	tValue	P-Value
Vehicle	Day 43	Nerve, Femoral, Distal, Left, Distal - Nerve, Femoral, Distal, Left, Proximal	0	0	0	0	1		
Vehicle	Day 43	Nerve, Femoral, Distal, Left, Distal - Nerve, Femoral, Distal, Right, Distal	0	0	0	0	1		
Vehicle	Day 43	Nerve, Femoral, Distal, Left, Distal - Nerve, Femoral, Distal, Right, Proximal	0	0	0	0	1		
Vehicle	Day 43	Nerve, Femoral, Distal, Left, Distal - Nerve, Femoral, Proximal, Left, Distal	0	0	0	0	1		
Vehicle	Day 43	Nerve, Femoral, Distal, Left, Distal - Nerve, Femoral, Proximal, Left, Proximal	0	0	0	0	1		
Vehicle	Day 43	Nerve, Femoral, Distal, Left, Distal - Nerve, Femoral, Proximal, Right, Distal	-0.00010276	0.000145325	-0.00140845	0.00120293	1	-1	0.5
Vehicle	Day 43	Nerve, Femoral, Distal, Left, Distal - Nerve, Femoral, Proximal, Right, Proximal	0	0	0	0	1		
Vehicle	Day 43	Nerve, Femoral, Distal, Left, Distal - Nerve Median, Distal, Left, Distal	0	0	0	0	1		
Vehicle	Day 43	Nerve, Femoral, Distal, Left, Distal - Nerve Median, Distal, Left, Proximal	0	0	0	0	1		
Vehicle	Day 43	Nerve, Femoral, Distal, Left, Distal - Nerve Median, Distal, Right, Distal	0	0	0	0	1		
Vehicle	Day 43	Nerve, Femoral, Distal, Left, Distal - Nerve Median, Distal, Right, Proximal	0	0	0	0	1		
Vehicle	Day 43	Nerve, Femoral, Distal, Left, Distal - Nerve Median, Proximal, Left, Distal	0	0	0	0	1		
Vehicle	Day 43	Nerve, Femoral, Distal, Left, Distal - Nerve Median, Proximal, Left, Proximal	0	0	0	0	1		
Vehicle	Day 43	Nerve, Femoral, Distal, Left, Distal - Nerve Median, Proximal, Right, Distal	0	0	0	0	1		
Vehicle	Day 43	Nerve, Femoral, Distal, Left, Distal - Nerve Median, Proximal, Right, Proximal	0	0	0	0	1		
Vehicle	Day 43	Nerve, Femoral, Distal, Left, Distal - Nerve, Sciatic, Distal, Left, Distal	0	0	0	0	1		
Vehicle	Day 43	Nerve, Femoral, Distal, Left, Distal - Nerve, Sciatic, Distal, Left, Proximal	0	0	0	0	1		
Vehicle	Day 43	Nerve, Femoral, Distal, Left, Distal - Nerve, Sciatic, Distal, Right, Distal	0	0	0	0	1		
Vehicle	Day 43	Nerve, Femoral, Distal, Left, Distal - Nerve, Sciatic, Distal, Right, Proximal	0	0	0	0	1		
Vehicle	Day 43	Nerve, Femoral, Distal, Left, Distal - Nerve, Sciatic, Proximal, Left, Distal	0	0	0	0	1		
Vehicle	Day 43	Nerve, Femoral, Distal, Left, Distal - Nerve, Sciatic, Proximal, Left, Proximal	0	0	0	0	1		
Vehicle	Day 43	Nerve, Femoral, Distal, Left, Distal - Nerve, Sciatic, Proximal, Right, Distal	0	0	0	0	1		
Vehicle	Day 43	Nerve, Femoral, Distal, Left, Distal - Nerve, Sciatic, Proximal, Right, Proximal	0	0	0	0	1		
Vehicle	Day 43	Nerve, Femoral, Distal, Left, Distal - Nerve, Ulnar, Distal, Left, Distal	0	0	0	0	1		
Vehicle	Day 43	Nerve, Femoral, Distal, Left, Distal - Nerve, Ulnar, Distal, Left, Proximal	0	0	0	0	1		
Vehicle	Day 43	Nerve, Femoral, Distal, Left, Distal - Nerve, Ulnar, Distal, Right, Distal	0	0	0	0	1		
Vehicle	Day 43	Nerve, Femoral, Distal, Left, Distal - Nerve, Ulnar, Distal, Right, Proximal	0	0	0	0	1		
Vehicle	Day 43	Nerve, Femoral, Distal, Left, Distal - Nerve, Ulnar, Proximal, Left, Distal	0	0	0	0	1		
Vehicle	Day 43	Nerve, Femoral, Distal, Left, Distal - Nerve, Ulnar, Proximal, Left, Proximal	0	0	0	0	1		
Vehicle	Day 43	Nerve, Femoral, Distal, Left, Distal - Nerve, Ulnar, Proximal, Right, Distal	0	0	0	0	1		
Vehicle	Day 43	Nerve, Femoral, Distal, Left, Distal - Nerve, Ulnar, Proximal, Right, Proximal	0	0	0	0	1		
Vehicle	Day 43	Nerve, Femoral, Distal, Left, Distal - Nerve, Optic	0	0	0	0	1		
Vehicle	Day 43	Nerve, Femoral, Distal, Left, Distal - Ovary	0				0		
Vehicle	Day 43	Nerve, Femoral, Distal, Left, Distal - Spinal Cord, Cervical	-0.0001082	0.000153018	-0.001483016	0.001266615	1	-1	0.5
Vehicle	Day 43	Nerve, Femoral, Distal, Left, Distal - Spinal Cord, Lumbar	0	0	0	0	1		
Vehicle	Day 43	Nerve, Femoral, Distal, Left, Distal - Spinal Cord, Sacral	0	0	0	0	1		
Vehicle	Day 43	Nerve, Femoral, Distal, Left, Distal - Spinal Cord, Spinal Roots	0	0	0	0	1		
Vehicle	Day 43	Nerve, Femoral, Distal, Left, Distal - Spinal Cord, Thoracic	0	0	0	0	1		
Vehicle	Day 43	Nerve, Femoral, Distal, Left, Distal - Spleen	-0.000216745	0.000306524	-0.002970753	0.002537263	1	-1	0.5
Vehicle	Day 43	Nerve, Femoral, Distal, Left, Distal - Testis	0				0		
Vehicle	Day 43	Nerve, Femoral, Distal, Left, Proximal - Nerve, Femoral, Distal, Right, Distal	0	0	0	0	1		
Vehicle	Day 43	Nerve, Femoral, Distal, Left, Proximal - Nerve, Femoral, Distal, Right, Proximal	0	0	0	0	1		
Vehicle	Day 43	Nerve, Femoral, Distal, Left, Proximal - Nerve, Femoral, Proximal, Left, Distal	0	0	0	0	1		
Vehicle	Day 43	Nerve, Femoral, Distal, Left, Proximal - Nerve, Femoral, Proximal, Left, Proximal	0	0	0	0	1		
Vehicle	Day 43	Nerve, Femoral, Distal, Left, Proximal - Nerve, Femoral, Proximal, Right, Distal	-0.00010276	0.000145325	-0.00140845	0.00120293	1	-1	0.5
Vehicle	Day 43	Nerve, Femoral, Distal, Left, Proximal - Nerve, Femoral, Proximal, Right, Proximal	0	0	0	0	1		
Vehicle	Day 43	Nerve, Femoral, Distal, Left, Proximal - Nerve Median, Distal, Left, Distal	0	0	0	0	1		
Vehicle	Day 43	Nerve, Femoral, Distal, Left, Proximal - Nerve Median, Distal, Left, Proximal	0	0	0	0	1		
Vehicle	Day 43	Nerve, Femoral, Distal, Left, Proximal - Nerve Median, Distal, Right, Distal	0	0	0	0	1		
Vehicle	Day 43	Nerve, Femoral, Distal, Left, Proximal - Nerve Median, Distal, Right, Proximal	0	0	0	0	1		
Vehicle	Day 43	Nerve, Femoral, Distal, Left, Proximal - Nerve Median, Proximal, Left, Distal	0	0	0	0	1		
Vehicle	Day 43	Nerve, Femoral, Distal, Left, Proximal - Nerve Median, Proximal, Left, Proximal	0	0	0	0	1		
Vehicle	Day 43	Nerve, Femoral, Distal, Left, Proximal - Nerve Median, Proximal, Right, Distal	0	0	0	0	1		
Vehicle	Day 43	Nerve, Femoral, Distal, Left, Proximal - Nerve Median, Proximal, Right, Proximal	0	0	0	0	1		
Vehicle	Day 43	Nerve, Femoral, Distal, Left, Proximal - Nerve, Sciatic, Distal, Left, Distal	0	0	0	0	1		
Vehicle	Day 43	Nerve, Femoral, Distal, Left, Proximal - Nerve, Sciatic, Distal, Left, Proximal	0	0	0	0	1		
Vehicle	Day 43	Nerve, Femoral, Distal, Left, Proximal - Nerve, Sciatic, Distal, Right, Distal	0	0	0	0	1		
Vehicle	Day 43	Nerve, Femoral, Distal, Left, Proximal - Nerve, Sciatic, Distal, Right, Proximal	0	0	0	0	1		
Vehicle	Day 43	Nerve, Femoral, Distal, Left, Proximal - Nerve, Sciatic, Proximal, Left, Distal	0	0	0	0	1		
Vehicle	Day 43	Nerve, Femoral, Distal, Left, Proximal - Nerve, Sciatic, Proximal, Left, Proximal	0	0	0	0	1		
Vehicle	Day 43	Nerve, Femoral, Distal, Left, Proximal - Nerve, Sciatic, Proximal, Right, Distal	0	0	0	0	1		
Vehicle	Day 43	Nerve, Femoral, Distal, Left, Proximal - Nerve, Sciatic, Proximal, Right, Proximal	0	0	0	0	1		
Vehicle	Day 43	Nerve, Femoral, Distal, Left, Proximal - Nerve, Ulnar, Distal, Left, Distal	0	0	0	0	1		
Vehicle	Day 43	Nerve, Femoral, Distal, Left, Proximal - Nerve, Ulnar, Distal, Left, Proximal	0	0	0	0	1		
Vehicle	Day 43	Nerve, Femoral, Distal, Left, Proximal - Nerve, Ulnar, Distal, Right, Distal	0	0	0	0	1		
Vehicle	Day 43	Nerve, Femoral, Distal, Left, Proximal - Nerve, Ulnar, Distal, Right, Proximal	0	0	0	0	1		
Vehicle	Day 43	Nerve, Femoral, Distal, Left, Proximal - Nerve, Ulnar, Proximal, Left, Distal	0	0	0	0	1		
Vehicle	Day 43	Nerve, Femoral, Distal, Left, Proximal - Nerve, Ulnar, Proximal, Left, Proximal	0	0	0	0	1		
Vehicle	Day 43	Nerve, Femoral, Distal, Left, Proximal - Nerve, Ulnar, Proximal, Right, Distal	0	0	0	0	1		
Vehicle	Day 43	Nerve, Femoral, Distal, Left, Proximal - Nerve, Ulnar, Proximal, Right, Proximal	0	0	0	0	1		
Vehicle	Day 43	Nerve, Femoral, Distal, Left, Proximal - Nerve, Optic	0	0	0	0	1		
Vehicle	Day 43	Nerve, Femoral, Distal, Left, Proximal - Ovary	0				0		
Vehicle	Day 43	Nerve, Femoral, Distal, Left, Proximal - Spinal Cord, Cervical	-0.0001082	0.000153018	-0.001483016	0.001266615	1	-1	0.5
Vehicle	Day 43	Nerve, Femoral, Distal, Left, Proximal - Spinal Cord, Lumbar	0	0	0	0	1		
Vehicle	Day 43	Nerve, Femoral, Distal, Left, Proximal - Spinal Cord, Sacral	0	0	0	0	1		
Vehicle	Day 43	Nerve, Femoral, Distal, Left, Proximal - Spinal Cord, Spinal Roots	0	0	0	0	1		
Vehicle	Day 43	Nerve, Femoral, Distal, Left, Proximal - Spinal Cord, Thoracic	0	0	0	0	1		
Vehicle	Day 43	Nerve, Femoral, Distal, Left, Proximal - Spleen	-0.000216745	0.000306524	-0.002970753	0.002537263	1	-1	0.5
Vehicle	Day 43	Nerve, Femoral, Distal, Left, Proximal - Testis	0				0		
Vehicle	Day 43	Nerve, Femoral, Distal, Right, Distal - Nerve, Femoral, Distal, Right, Proximal	0	0	0	0	1		
Vehicle	Day 43	Nerve, Femoral, Distal, Right, Distal - Nerve, Femoral, Proximal, Left, Distal	0	0	0	0	1		
Vehicle	Day 43	Nerve, Femoral, Distal, Right, Distal - Nerve, Femoral, Proximal, Left, Proximal	0	0	0	0	1		
Vehicle	Day 43	Nerve, Femoral, Distal, Right, Distal - Nerve, Femoral, Proximal, Right, Distal	-0.00010276	0.000145325	-0.00140845	0.00120293	1	-1	0.5
Vehicle	Day 43	Nerve, Femoral, Distal, Right, Distal - Nerve, Femoral, Proximal, Right, Proximal	0	0	0	0	1		
Vehicle	Day 43	Nerve, Femoral, Distal, Right, Distal - Nerve Median, Distal, Left, Distal	0	0	0	0	1		
Vehicle	Day 43	Nerve, Femoral, Distal, Right, Distal - Nerve Median, Distal, Left, Proximal	0	0	0	0	1		
Vehicle	Day 43	Nerve, Femoral, Distal, Right, Distal - Nerve Median, Distal, Right, Distal	0	0	0	0	1		
Vehicle	Day 43	Nerve, Femoral, Distal, Right, Distal - Nerve Median, Distal, Right, Proximal	0	0	0	0	1		
Vehicle	Day 43	Nerve, Femoral, Distal, Right, Distal - Nerve Median, Proximal, Left, Distal	0	0	0	0	1		
Vehicle	Day 43	Nerve, Femoral, Distal, Right, Distal - Nerve Median, Proximal, Left, Proximal	0	0	0	0	1		
Vehicle	Day 43	Nerve, Femoral, Distal, Right, Distal - Nerve Median, Proximal, Right, Distal	0	0	0	0	1		
Vehicle	Day 43	Nerve, Femoral, Distal, Right, Distal - Nerve Median, Proximal, Right, Proximal	0	0	0	0	1		
Vehicle	Day 43	Nerve, Femoral, Distal, Right, Distal - Nerve, Sciatic, Distal, Left, Distal	0	0	0	0	1		

Results of Evaluating Mean Differences Between Different Tissues Across Timepoint and Group for Log10 AAV2-ITR Target Copies/ug of DNA for Normalized Data

Group	Timepoint	Difference	Mean	StdDev	LowerCLMean	UpperCLMean	DF	tValue	P-Value
Vehicle	Day 43	Nerve, Femoral, Distal, Right, Distal - Nerve, Sciatic, Distal, Left, Proximal	0	0	0	0	1		
Vehicle	Day 43	Nerve, Femoral, Distal, Right, Distal - Nerve, Sciatic, Distal, Right, Distal	0	0	0	0	1		
Vehicle	Day 43	Nerve, Femoral, Distal, Right, Distal - Nerve, Sciatic, Distal, Right, Proximal	0	0	0	0	1		
Vehicle	Day 43	Nerve, Femoral, Distal, Right, Distal - Nerve, Sciatic, Proximal, Left, Distal	0	0	0	0	1		
Vehicle	Day 43	Nerve, Femoral, Distal, Right, Distal - Nerve, Sciatic, Proximal, Left, Proximal	0	0	0	0	1		
Vehicle	Day 43	Nerve, Femoral, Distal, Right, Distal - Nerve, Sciatic, Proximal, Right, Distal	0	0	0	0	1		
Vehicle	Day 43	Nerve, Femoral, Distal, Right, Distal - Nerve, Sciatic, Proximal, Right, Proximal	0	0	0	0	1		
Vehicle	Day 43	Nerve, Femoral, Distal, Right, Distal - Nerve, Ulnar, Distal, Left, Distal	0	0	0	0	1		
Vehicle	Day 43	Nerve, Femoral, Distal, Right, Distal - Nerve, Ulnar, Distal, Left, Proximal	0	0	0	0	1		
Vehicle	Day 43	Nerve, Femoral, Distal, Right, Distal - Nerve, Ulnar, Distal, Right, Distal	0	0	0	0	1		
Vehicle	Day 43	Nerve, Femoral, Distal, Right, Distal - Nerve, Ulnar, Distal, Right, Proximal	0	0	0	0	1		
Vehicle	Day 43	Nerve, Femoral, Distal, Right, Distal - Nerve, Ulnar, Proximal, Left, Distal	0	0	0	0	1		
Vehicle	Day 43	Nerve, Femoral, Distal, Right, Distal - Nerve, Ulnar, Proximal, Left, Proximal	0	0	0	0	1		
Vehicle	Day 43	Nerve, Femoral, Distal, Right, Distal - Nerve, Ulnar, Proximal, Right, Distal	0	0	0	0	1		
Vehicle	Day 43	Nerve, Femoral, Distal, Right, Distal - Nerve, Ulnar, Proximal, Right, Proximal	0	0	0	0	1		
Vehicle	Day 43	Nerve, Femoral, Distal, Right, Distal - Nerve, Optic	0	0	0	0	1		
Vehicle	Day 43	Nerve, Femoral, Distal, Right, Distal - Ovary	0				0		
Vehicle	Day 43	Nerve, Femoral, Distal, Right, Distal - Spinal Cord, Cervical	-0.0001082	0.000153018	-0.001483016	0.001266615	1	-1	0.5
Vehicle	Day 43	Nerve, Femoral, Distal, Right, Distal - Spinal Cord, Lumbar	0	0	0	0	1		
Vehicle	Day 43	Nerve, Femoral, Distal, Right, Distal - Spinal Cord, Sacral	0	0	0	0	1		
Vehicle	Day 43	Nerve, Femoral, Distal, Right, Distal - Spinal Cord, Spinal Roots	0	0	0	0	1		
Vehicle	Day 43	Nerve, Femoral, Distal, Right, Distal - Spinal Cord, Thoracic	0	0	0	0	1		
Vehicle	Day 43	Nerve, Femoral, Distal, Right, Distal - Spleen	-0.000216745	0.000306524	-0.002970753	0.002537263	1	-1	0.5
Vehicle	Day 43	Nerve, Femoral, Distal, Right, Distal - Testis	0				0		
Vehicle	Day 43	Nerve, Femoral, Distal, Right, Proximal - Nerve, Femoral, Proximal, Left, Distal	0	0	0	0	1		
Vehicle	Day 43	Nerve, Femoral, Distal, Right, Proximal - Nerve, Femoral, Proximal, Left, Proximal	0	0	0	0	1		
Vehicle	Day 43	Nerve, Femoral, Distal, Right, Proximal - Nerve, Femoral, Proximal, Right, Distal	-0.00010276	0.000145325	-0.00140845	0.00120293	1	-1	0.5
Vehicle	Day 43	Nerve, Femoral, Distal, Right, Proximal - Nerve, Femoral, Proximal, Right, Proximal	0	0	0	0	1		
Vehicle	Day 43	Nerve, Femoral, Distal, Right, Proximal - Nerve Median, Distal, Left, Distal	0	0	0	0	1		
Vehicle	Day 43	Nerve, Femoral, Distal, Right, Proximal - Nerve Median, Distal, Left, Proximal	0	0	0	0	1		
Vehicle	Day 43	Nerve, Femoral, Distal, Right, Proximal - Nerve Median, Distal, Right, Distal	0	0	0	0	1		
Vehicle	Day 43	Nerve, Femoral, Distal, Right, Proximal - Nerve Median, Distal, Right, Proximal	0	0	0	0	1		
Vehicle	Day 43	Nerve, Femoral, Distal, Right, Proximal - Nerve Median, Proximal, Left, Distal	0	0	0	0	1		
Vehicle	Day 43	Nerve, Femoral, Distal, Right, Proximal - Nerve Median, Proximal, Left, Proximal	0	0	0	0	1		
Vehicle	Day 43	Nerve, Femoral, Distal, Right, Proximal - Nerve Median, Proximal, Right, Distal	0	0	0	0	1		
Vehicle	Day 43	Nerve, Femoral, Distal, Right, Proximal - Nerve Median, Proximal, Right, Proximal	0	0	0	0	1		
Vehicle	Day 43	Nerve, Femoral, Distal, Right, Proximal - Nerve, Sciatic, Distal, Left, Distal	0	0	0	0	1		
Vehicle	Day 43	Nerve, Femoral, Distal, Right, Proximal - Nerve, Sciatic, Distal, Left, Proximal	0	0	0	0	1		
Vehicle	Day 43	Nerve, Femoral, Distal, Right, Proximal - Nerve, Sciatic, Distal, Right, Distal	0	0	0	0	1		
Vehicle	Day 43	Nerve, Femoral, Distal, Right, Proximal - Nerve, Sciatic, Distal, Right, Proximal	0	0	0	0	1		
Vehicle	Day 43	Nerve, Femoral, Distal, Right, Proximal - Nerve, Sciatic, Proximal, Left, Distal	0	0	0	0	1		
Vehicle	Day 43	Nerve, Femoral, Distal, Right, Proximal - Nerve, Sciatic, Proximal, Left, Proximal	0	0	0	0	1		
Vehicle	Day 43	Nerve, Femoral, Distal, Right, Proximal - Nerve, Sciatic, Proximal, Right, Distal	0	0	0	0	1		
Vehicle	Day 43	Nerve, Femoral, Distal, Right, Proximal - Nerve, Sciatic, Proximal, Right, Proximal	0	0	0	0	1		
Vehicle	Day 43	Nerve, Femoral, Distal, Right, Proximal - Nerve, Ulnar, Distal, Left, Distal	0	0	0	0	1		
Vehicle	Day 43	Nerve, Femoral, Distal, Right, Proximal - Nerve, Ulnar, Distal, Left, Proximal	0	0	0	0	1		
Vehicle	Day 43	Nerve, Femoral, Distal, Right, Proximal - Nerve, Ulnar, Distal, Right, Distal	0	0	0	0	1		
Vehicle	Day 43	Nerve, Femoral, Distal, Right, Proximal - Nerve, Ulnar, Distal, Right, Proximal	0	0	0	0	1		
Vehicle	Day 43	Nerve, Femoral, Distal, Right, Proximal - Nerve, Ulnar, Proximal, Left, Distal	0	0	0	0	1		
Vehicle	Day 43	Nerve, Femoral, Distal, Right, Proximal - Nerve, Ulnar, Proximal, Left, Proximal	0	0	0	0	1		
Vehicle	Day 43	Nerve, Femoral, Distal, Right, Proximal - Nerve, Ulnar, Proximal, Right, Distal	0	0	0	0	1		
Vehicle	Day 43	Nerve, Femoral, Distal, Right, Proximal - Nerve, Ulnar, Proximal, Right, Proximal	0	0	0	0	1		
Vehicle	Day 43	Nerve, Femoral, Distal, Right, Proximal - Nerve, Optic	0	0	0	0	1		
Vehicle	Day 43	Nerve, Femoral, Distal, Right, Proximal - Ovary	0				0		
Vehicle	Day 43	Nerve, Femoral, Distal, Right, Proximal - Spinal Cord, Cervical	-0.0001082	0.000153018	-0.001483016	0.001266615	1	-1	0.5
Vehicle	Day 43	Nerve, Femoral, Distal, Right, Proximal - Spinal Cord, Lumbar	0	0	0	0	1		
Vehicle	Day 43	Nerve, Femoral, Distal, Right, Proximal - Spinal Cord, Sacral	0	0	0	0	1		
Vehicle	Day 43	Nerve, Femoral, Distal, Right, Proximal - Spinal Cord, Spinal Roots	0	0	0	0	1		
Vehicle	Day 43	Nerve, Femoral, Distal, Right, Proximal - Spinal Cord, Thoracic	0	0	0	0	1		
Vehicle	Day 43	Nerve, Femoral, Distal, Right, Proximal - Spleen	-0.000216745	0.000306524	-0.002970753	0.002537263	1	-1	0.5
Vehicle	Day 43	Nerve, Femoral, Distal, Right, Proximal - Testis	0				0		
Vehicle	Day 43	Nerve, Femoral, Proximal, Left, Distal - Nerve, Femoral, Proximal, Left, Proximal	0	0	0	0	1		
Vehicle	Day 43	Nerve, Femoral, Proximal, Left, Distal - Nerve, Femoral, Proximal, Right, Distal	-0.00010276	0.000145325	-0.00140845	0.00120293	1	-1	0.5
Vehicle	Day 43	Nerve, Femoral, Proximal, Left, Distal - Nerve, Femoral, Proximal, Right, Proximal	0	0	0	0	1		
Vehicle	Day 43	Nerve, Femoral, Proximal, Left, Distal - Nerve Median, Distal, Left, Distal	0	0	0	0	1		
Vehicle	Day 43	Nerve, Femoral, Proximal, Left, Distal - Nerve Median, Distal, Left, Proximal	0	0	0	0	1		
Vehicle	Day 43	Nerve, Femoral, Proximal, Left, Distal - Nerve Median, Distal, Right, Distal	0	0	0	0	1		
Vehicle	Day 43	Nerve, Femoral, Proximal, Left, Distal - Nerve Median, Distal, Right, Proximal	0	0	0	0	1		
Vehicle	Day 43	Nerve, Femoral, Proximal, Left, Distal - Nerve Median, Proximal, Left, Distal	0	0	0	0	1		
Vehicle	Day 43	Nerve, Femoral, Proximal, Left, Distal - Nerve Median, Proximal, Left, Proximal	0	0	0	0	1		
Vehicle	Day 43	Nerve, Femoral, Proximal, Left, Distal - Nerve Median, Proximal, Right, Distal	0	0	0	0	1		
Vehicle	Day 43	Nerve, Femoral, Proximal, Left, Distal - Nerve Median, Proximal, Right, Proximal	0	0	0	0	1		
Vehicle	Day 43	Nerve, Femoral, Proximal, Left, Distal - Nerve, Sciatic, Distal, Left, Distal	0	0	0	0	1		
Vehicle	Day 43	Nerve, Femoral, Proximal, Left, Distal - Nerve, Sciatic, Distal, Left, Proximal	0	0	0	0	1		
Vehicle	Day 43	Nerve, Femoral, Proximal, Left, Distal - Nerve, Sciatic, Distal, Right, Distal	0	0	0	0	1		
Vehicle	Day 43	Nerve, Femoral, Proximal, Left, Distal - Nerve, Sciatic, Distal, Right, Proximal	0	0	0	0	1		
Vehicle	Day 43	Nerve, Femoral, Proximal, Left, Distal - Nerve, Sciatic, Proximal, Left, Distal	0	0	0	0	1		
Vehicle	Day 43	Nerve, Femoral, Proximal, Left, Distal - Nerve, Sciatic, Proximal, Left, Proximal	0	0	0	0	1		
Vehicle	Day 43	Nerve, Femoral, Proximal, Left, Distal - Nerve, Sciatic, Proximal, Right, Distal	0	0	0	0	1		
Vehicle	Day 43	Nerve, Femoral, Proximal, Left, Distal - Nerve, Sciatic, Proximal, Right, Proximal	0	0	0	0	1		
Vehicle	Day 43	Nerve, Femoral, Proximal, Left, Distal - Nerve, Ulnar, Distal, Left, Distal	0	0	0	0	1		
Vehicle	Day 43	Nerve, Femoral, Proximal, Left, Distal - Nerve, Ulnar, Distal, Left, Proximal	0	0	0	0	1		
Vehicle	Day 43	Nerve, Femoral, Proximal, Left, Distal - Nerve, Ulnar, Distal, Right, Distal	0	0	0	0	1		
Vehicle	Day 43	Nerve, Femoral, Proximal, Left, Distal - Nerve, Ulnar, Distal, Right, Proximal	0	0	0	0	1		
Vehicle	Day 43	Nerve, Femoral, Proximal, Left, Distal - Nerve, Ulnar, Proximal, Left, Distal	0	0	0	0	1		
Vehicle	Day 43	Nerve, Femoral, Proximal, Left, Distal - Nerve, Ulnar, Proximal, Left, Proximal	0	0	0	0	1		
Vehicle	Day 43	Nerve, Femoral, Proximal, Left, Distal - Nerve, Ulnar, Proximal, Right, Distal	0	0	0	0	1		
Vehicle	Day 43	Nerve, Femoral, Proximal, Left, Distal - Nerve, Ulnar, Proximal, Right, Proximal	0	0	0	0	1		
Vehicle	Day 43	Nerve, Femoral, Proximal, Left, Distal - Nerve, Optic	0	0	0	0	1		
Vehicle	Day 43	Nerve, Femoral, Proximal, Left, Distal - Ovary	0				0		
Vehicle	Day 43	Nerve, Femoral, Proximal, Left, Distal - Spinal Cord, Cervical	-0.0001082	0.000153018	-0.001483016	0.001266615	1	-1	0.5
Vehicle	Day 43	Nerve, Femoral, Proximal, Left, Distal - Spinal Cord, Lumbar	0	0	0	0	1		
Vehicle	Day 43	Nerve, Femoral, Proximal, Left, Distal - Spinal Cord, Sacral	0	0	0	0	1		

Results of Evaluating Mean Differences Between Different Tissues Across Timepoint and Group for Log10 AAV2-ITR Target Copies/ug of DNA for Normalized Data

Group	Timepoint	Difference	Mean	StdDev	LowerCLMean	UpperCLMean	DF	tValue	P-Value
Vehicle	Day 43	Nerve, Femoral, Proximal, Left, Distal - Spinal Cord, Spinal Roots	0	0	0	0	1		
Vehicle	Day 43	Nerve, Femoral, Proximal, Left, Distal - Spinal Cord, Thoracic	0	0	0	0	1		
Vehicle	Day 43	Nerve, Femoral, Proximal, Left, Distal - Spleen	-0.000216745	0.000306524	-0.002970753	0.002537263	1	-1	0.5
Vehicle	Day 43	Nerve, Femoral, Proximal, Left, Distal - Testis	0				0		
Vehicle	Day 43	Nerve, Femoral, Proximal, Left, Proximal - Nerve, Femoral, Proximal, Right, Distal	-0.00010276	0.000145325	-0.00140845	0.00120293	1	-1	0.5
Vehicle	Day 43	Nerve, Femoral, Proximal, Left, Proximal - Nerve, Femoral, Proximal, Right, Proximal	0	0	0	0	1		
Vehicle	Day 43	Nerve, Femoral, Proximal, Left, Proximal - Nerve Median, Distal, Left, Distal	0	0	0	0	1		
Vehicle	Day 43	Nerve, Femoral, Proximal, Left, Proximal - Nerve Median, Distal, Left, Proximal	0	0	0	0	1		
Vehicle	Day 43	Nerve, Femoral, Proximal, Left, Proximal - Nerve Median, Distal, Right, Distal	0	0	0	0	1		
Vehicle	Day 43	Nerve, Femoral, Proximal, Left, Proximal - Nerve Median, Distal, Right, Proximal	0	0	0	0	1		
Vehicle	Day 43	Nerve, Femoral, Proximal, Left, Proximal - Nerve Median, Proximal, Left, Distal	0	0	0	0	1		
Vehicle	Day 43	Nerve, Femoral, Proximal, Left, Proximal - Nerve Median, Proximal, Left, Proximal	0	0	0	0	1		
Vehicle	Day 43	Nerve, Femoral, Proximal, Left, Proximal - Nerve Median, Proximal, Right, Distal	0	0	0	0	1		
Vehicle	Day 43	Nerve, Femoral, Proximal, Left, Proximal - Nerve Median, Proximal, Right, Proximal	0	0	0	0	1		
Vehicle	Day 43	Nerve, Femoral, Proximal, Left, Proximal - Nerve, Sciatic, Distal, Left, Distal	0	0	0	0	1		
Vehicle	Day 43	Nerve, Femoral, Proximal, Left, Proximal - Nerve, Sciatic, Distal, Left, Proximal	0	0	0	0	1		
Vehicle	Day 43	Nerve, Femoral, Proximal, Left, Proximal - Nerve, Sciatic, Distal, Right, Distal	0	0	0	0	1		
Vehicle	Day 43	Nerve, Femoral, Proximal, Left, Proximal - Nerve, Sciatic, Distal, Right, Proximal	0	0	0	0	1		
Vehicle	Day 43	Nerve, Femoral, Proximal, Left, Proximal - Nerve, Sciatic, Proximal, Left, Distal	0	0	0	0	1		
Vehicle	Day 43	Nerve, Femoral, Proximal, Left, Proximal - Nerve, Sciatic, Proximal, Left, Proximal	0	0	0	0	1		
Vehicle	Day 43	Nerve, Femoral, Proximal, Left, Proximal - Nerve, Sciatic, Proximal, Right, Distal	0	0	0	0	1		
Vehicle	Day 43	Nerve, Femoral, Proximal, Left, Proximal - Nerve, Sciatic, Proximal, Right, Proximal	0	0	0	0	1		
Vehicle	Day 43	Nerve, Femoral, Proximal, Left, Proximal - Nerve, Ulnar, Distal, Left, Distal	0	0	0	0	1		
Vehicle	Day 43	Nerve, Femoral, Proximal, Left, Proximal - Nerve, Ulnar, Distal, Left, Proximal	0	0	0	0	1		
Vehicle	Day 43	Nerve, Femoral, Proximal, Left, Proximal - Nerve, Ulnar, Distal, Right, Distal	0	0	0	0	1		
Vehicle	Day 43	Nerve, Femoral, Proximal, Left, Proximal - Nerve, Ulnar, Distal, Right, Proximal	0	0	0	0	1		
Vehicle	Day 43	Nerve, Femoral, Proximal, Left, Proximal - Nerve, Ulnar, Proximal, Left, Distal	0	0	0	0	1		
Vehicle	Day 43	Nerve, Femoral, Proximal, Left, Proximal - Nerve, Ulnar, Proximal, Left, Proximal	0	0	0	0	1		
Vehicle	Day 43	Nerve, Femoral, Proximal, Left, Proximal - Nerve, Ulnar, Proximal, Right, Distal	0	0	0	0	1		
Vehicle	Day 43	Nerve, Femoral, Proximal, Left, Proximal - Nerve, Ulnar, Proximal, Right, Proximal	0	0	0	0	1		
Vehicle	Day 43	Nerve, Femoral, Proximal, Left, Proximal - Nerve, Optic	0	0	0	0	1		
Vehicle	Day 43	Nerve, Femoral, Proximal, Left, Proximal - Ovary	0				0		
Vehicle	Day 43	Nerve, Femoral, Proximal, Left, Proximal - Spinal Cord, Cervical	-0.0001082	0.000153018	-0.001483016	0.001266615	1	-1	0.5
Vehicle	Day 43	Nerve, Femoral, Proximal, Left, Proximal - Spinal Cord, Lumbar	0	0	0	0	1		
Vehicle	Day 43	Nerve, Femoral, Proximal, Left, Proximal - Spinal Cord, Sacral	0	0	0	0	1		
Vehicle	Day 43	Nerve, Femoral, Proximal, Left, Proximal - Spinal Cord, Spinal Roots	0	0	0	0	1		
Vehicle	Day 43	Nerve, Femoral, Proximal, Left, Proximal - Spinal Cord, Thoracic	0	0	0	0	1		
Vehicle	Day 43	Nerve, Femoral, Proximal, Left, Proximal - Spleen	-0.000216745	0.000306524	-0.002970753	0.002537263	1	-1	0.5
Vehicle	Day 43	Nerve, Femoral, Proximal, Left, Proximal - Testis	0				0		
Vehicle	Day 43	Nerve, Femoral, Proximal, Right, Distal - Nerve, Femoral, Proximal, Right, Proximal	0.00010276	0.000145325	-0.00120293	0.00140845	1	1	0.5
Vehicle	Day 43	Nerve, Femoral, Proximal, Right, Distal - Nerve Median, Distal, Left, Distal	0.00010276	0.000145325	-0.00120293	0.00140845	1	1	0.5
Vehicle	Day 43	Nerve, Femoral, Proximal, Right, Distal - Nerve Median, Distal, Left, Proximal	0.00010276	0.000145325	-0.00120293	0.00140845	1	1	0.5
Vehicle	Day 43	Nerve, Femoral, Proximal, Right, Distal - Nerve Median, Distal, Right, Distal	0.00010276	0.000145325	-0.00120293	0.00140845	1	1	0.5
Vehicle	Day 43	Nerve, Femoral, Proximal, Right, Distal - Nerve Median, Distal, Right, Proximal	0.00010276	0.000145325	-0.00120293	0.00140845	1	1	0.5
Vehicle	Day 43	Nerve, Femoral, Proximal, Right, Distal - Nerve Median, Proximal, Left, Distal	0.00010276	0.000145325	-0.00120293	0.00140845	1	1	0.5
Vehicle	Day 43	Nerve, Femoral, Proximal, Right, Distal - Nerve Median, Proximal, Left, Proximal	0.00010276	0.000145325	-0.00120293	0.00140845	1	1	0.5
Vehicle	Day 43	Nerve, Femoral, Proximal, Right, Distal - Nerve Median, Proximal, Right, Distal	0.00010276	0.000145325	-0.00120293	0.00140845	1	1	0.5
Vehicle	Day 43	Nerve, Femoral, Proximal, Right, Distal - Nerve Median, Proximal, Right, Proximal	0.00010276	0.000145325	-0.00120293	0.00140845	1	1	0.5
Vehicle	Day 43	Nerve, Femoral, Proximal, Right, Distal - Nerve, Sciatic, Distal, Left, Distal	0.00010276	0.000145325	-0.00120293	0.00140845	1	1	0.5
Vehicle	Day 43	Nerve, Femoral, Proximal, Right, Distal - Nerve, Sciatic, Distal, Left, Proximal	0.00010276	0.000145325	-0.00120293	0.00140845	1	1	0.5
Vehicle	Day 43	Nerve, Femoral, Proximal, Right, Distal - Nerve, Sciatic, Distal, Right, Distal	0.00010276	0.000145325	-0.00120293	0.00140845	1	1	0.5
Vehicle	Day 43	Nerve, Femoral, Proximal, Right, Distal - Nerve, Sciatic, Distal, Right, Proximal	0.00010276	0.000145325	-0.00120293	0.00140845	1	1	0.5
Vehicle	Day 43	Nerve, Femoral, Proximal, Right, Distal - Nerve, Sciatic, Proximal, Left, Distal	0.00010276	0.000145325	-0.00120293	0.00140845	1	1	0.5
Vehicle	Day 43	Nerve, Femoral, Proximal, Right, Distal - Nerve, Sciatic, Proximal, Left, Proximal	0.00010276	0.000145325	-0.00120293	0.00140845	1	1	0.5
Vehicle	Day 43	Nerve, Femoral, Proximal, Right, Distal - Nerve, Sciatic, Proximal, Right, Distal	0.00010276	0.000145325	-0.00120293	0.00140845	1	1	0.5
Vehicle	Day 43	Nerve, Femoral, Proximal, Right, Distal - Nerve, Sciatic, Proximal, Right, Proximal	0.00010276	0.000145325	-0.00120293	0.00140845	1	1	0.5
Vehicle	Day 43	Nerve, Femoral, Proximal, Right, Distal - Nerve, Ulnar, Distal, Left, Distal	0.00010276	0.000145325	-0.00120293	0.00140845	1	1	0.5
Vehicle	Day 43	Nerve, Femoral, Proximal, Right, Distal - Nerve, Ulnar, Distal, Left, Proximal	0.00010276	0.000145325	-0.00120293	0.00140845	1	1	0.5
Vehicle	Day 43	Nerve, Femoral, Proximal, Right, Distal - Nerve, Ulnar, Distal, Right, Distal	0.00010276	0.000145325	-0.00120293	0.00140845	1	1	0.5
Vehicle	Day 43	Nerve, Femoral, Proximal, Right, Distal - Nerve, Ulnar, Distal, Right, Proximal	0.00010276	0.000145325	-0.00120293	0.00140845	1	1	0.5
Vehicle	Day 43	Nerve, Femoral, Proximal, Right, Distal - Nerve, Ulnar, Proximal, Left, Distal	0.00010276	0.000145325	-0.00120293	0.00140845	1	1	0.5
Vehicle	Day 43	Nerve, Femoral, Proximal, Right, Distal - Nerve, Ulnar, Proximal, Left, Proximal	0.00010276	0.000145325	-0.00120293	0.00140845	1	1	0.5
Vehicle	Day 43	Nerve, Femoral, Proximal, Right, Distal - Nerve, Ulnar, Proximal, Right, Distal	0.00010276	0.000145325	-0.00120293	0.00140845	1	1	0.5
Vehicle	Day 43	Nerve, Femoral, Proximal, Right, Distal - Nerve, Ulnar, Proximal, Right, Proximal	0.00010276	0.000145325	-0.00120293	0.00140845	1	1	0.5
Vehicle	Day 43	Nerve, Femoral, Proximal, Right, Distal - Nerve, Optic	0.00010276	0.000145325	-0.00120293	0.00140845	1	1	0.5
Vehicle	Day 43	Nerve, Femoral, Proximal, Right, Distal - Ovary	0				0		
Vehicle	Day 43	Nerve, Femoral, Proximal, Right, Distal - Spinal Cord, Cervical	-5.44027E-06	0.000298343	-0.002685946	0.002675066	1	-0.025788102	0.983586422
Vehicle	Day 43	Nerve, Femoral, Proximal, Right, Distal - Spinal Cord, Lumbar	0.00010276	0.000145325	-0.00120293	0.00140845	1	1	0.5
Vehicle	Day 43	Nerve, Femoral, Proximal, Right, Distal - Spinal Cord, Sacral	0.00010276	0.000145325	-0.00120293	0.00140845	1	1	0.5
Vehicle	Day 43	Nerve, Femoral, Proximal, Right, Distal - Spinal Cord, Spinal Roots	0.00010276	0.000145325	-0.00120293	0.00140845	1	1	0.5
Vehicle	Day 43	Nerve, Femoral, Proximal, Right, Distal - Spinal Cord, Thoracic	0.00010276	0.000145325	-0.00120293	0.00140845	1	1	0.5
Vehicle	Day 43	Nerve, Femoral, Proximal, Right, Distal - Spleen	-0.000113985	0.000161199	-0.001562303	0.001334333	1	-1	0.5
Vehicle	Day 43	Nerve, Femoral, Proximal, Right, Distal - Testis	0.00020552				0		
Vehicle	Day 43	Nerve, Femoral, Proximal, Right, Proximal - Nerve Median, Distal, Left, Distal	0	0	0	0	1		
Vehicle	Day 43	Nerve, Femoral, Proximal, Right, Proximal - Nerve Median, Distal, Left, Proximal	0	0	0	0	1		
Vehicle	Day 43	Nerve, Femoral, Proximal, Right, Proximal - Nerve Median, Distal, Right, Distal	0	0	0	0	1		
Vehicle	Day 43	Nerve, Femoral, Proximal, Right, Proximal - Nerve Median, Distal, Right, Proximal	0	0	0	0	1		
Vehicle	Day 43	Nerve, Femoral, Proximal, Right, Proximal - Nerve Median, Proximal, Left, Distal	0	0	0	0	1		
Vehicle	Day 43	Nerve, Femoral, Proximal, Right, Proximal - Nerve Median, Proximal, Left, Proximal	0	0	0	0	1		
Vehicle	Day 43	Nerve, Femoral, Proximal, Right, Proximal - Nerve Median, Proximal, Right, Distal	0	0	0	0	1		
Vehicle	Day 43	Nerve, Femoral, Proximal, Right, Proximal - Nerve Median, Proximal, Right, Proximal	0	0	0	0	1		
Vehicle	Day 43	Nerve, Femoral, Proximal, Right, Proximal - Nerve, Sciatic, Distal, Left, Distal	0	0	0	0	1		
Vehicle	Day 43	Nerve, Femoral, Proximal, Right, Proximal - Nerve, Sciatic, Distal, Left, Proximal	0	0	0	0	1		
Vehicle	Day 43	Nerve, Femoral, Proximal, Right, Proximal - Nerve, Sciatic, Distal, Right, Distal	0	0	0	0	1		
Vehicle	Day 43	Nerve, Femoral, Proximal, Right, Proximal - Nerve, Sciatic, Distal, Right, Proximal	0	0	0	0	1		
Vehicle	Day 43	Nerve, Femoral, Proximal, Right, Proximal - Nerve, Sciatic, Proximal, Left, Distal	0	0	0	0	1		
Vehicle	Day 43	Nerve, Femoral, Proximal, Right, Proximal - Nerve, Sciatic, Proximal, Left, Proximal	0	0	0	0	1		
Vehicle	Day 43	Nerve, Femoral, Proximal, Right, Proximal - Nerve, Sciatic, Proximal, Right, Distal	0	0	0	0	1		
Vehicle	Day 43	Nerve, Femoral, Proximal, Right, Proximal - Nerve, Sciatic, Proximal, Right, Proximal	0	0	0	0	1		
Vehicle	Day 43	Nerve, Femoral, Proximal, Right, Proximal - Nerve, Ulnar, Distal, Left, Distal	0	0	0	0	1		
Vehicle	Day 43	Nerve, Femoral, Proximal, Right, Proximal - Nerve, Ulnar, Distal, Left, Proximal	0	0	0	0	1		
Vehicle	Day 43	Nerve, Femoral, Proximal, Right, Proximal - Nerve, Ulnar, Distal, Right, Distal	0	0	0	0	1		
Vehicle	Day 43	Nerve, Femoral, Proximal, Right, Proximal - Nerve, Ulnar, Distal, Right, Proximal	0	0	0	0	1		

Results of Evaluating Mean Differences Between Different Tissues Across Timepoint and Group for Log10 AAV2-ITR Target Copies/ug of DNA for Normalized Data

Group	Timepoint	Difference	Mean	StdDev	LowerCLMean	UpperCLMean	DF	tValue	P-Value
Vehicle	Day 43	Nerve, Femoral, Proximal, Right, Proximal - Nerve, Ulnar, Proximal, Left, Distal	0	0	0	0	1		
Vehicle	Day 43	Nerve, Femoral, Proximal, Right, Proximal - Nerve, Ulnar, Proximal, Left, Proximal	0	0	0	0	1		
Vehicle	Day 43	Nerve, Femoral, Proximal, Right, Proximal - Nerve, Ulnar, Proximal, Right, Distal	0	0	0	0	1		
Vehicle	Day 43	Nerve, Femoral, Proximal, Right, Proximal - Nerve, Ulnar, Proximal, Right, Proximal	0	0	0	0	1		
Vehicle	Day 43	Nerve, Femoral, Proximal, Right, Proximal - Nerve, Optic	0	0	0	0	1		
Vehicle	Day 43	Nerve, Femoral, Proximal, Right, Proximal - Ovary	0				0		
Vehicle	Day 43	Nerve, Femoral, Proximal, Right, Proximal - Spinal Cord, Cervical	-0.0001082	0.000153018	-0.001483016	0.001266615	1	-1	0.5
Vehicle	Day 43	Nerve, Femoral, Proximal, Right, Proximal - Spinal Cord, Lumbar	0	0	0	0	1		
Vehicle	Day 43	Nerve, Femoral, Proximal, Right, Proximal - Spinal Cord, Sacral	0	0	0	0	1		
Vehicle	Day 43	Nerve, Femoral, Proximal, Right, Proximal - Spinal Cord, Spinal Roots	0	0	0	0	1		
Vehicle	Day 43	Nerve, Femoral, Proximal, Right, Proximal - Spinal Cord, Thoracic	0	0	0	0	1		
Vehicle	Day 43	Nerve, Femoral, Proximal, Right, Proximal - Spleen	-0.000216745	0.000306524	-0.002970753	0.002537263	1	-1	0.5
Vehicle	Day 43	Nerve, Femoral, Proximal, Right, Proximal - Testis	0				0		
Vehicle	Day 43	Nerve Median, Distal, Left, Distal - Nerve Median, Distal, Left, Proximal	0	0	0	0	1		
Vehicle	Day 43	Nerve Median, Distal, Left, Distal - Nerve Median, Distal, Right, Distal	0	0	0	0	1		
Vehicle	Day 43	Nerve Median, Distal, Left, Distal - Nerve Median, Distal, Right, Proximal	0	0	0	0	1		
Vehicle	Day 43	Nerve Median, Distal, Left, Distal - Nerve Median, Proximal, Left, Distal	0	0	0	0	1		
Vehicle	Day 43	Nerve Median, Distal, Left, Distal - Nerve Median, Proximal, Left, Proximal	0	0	0	0	1		
Vehicle	Day 43	Nerve Median, Distal, Left, Distal - Nerve Median, Proximal, Right, Distal	0	0	0	0	1		
Vehicle	Day 43	Nerve Median, Distal, Left, Distal - Nerve Median, Proximal, Right, Proximal	0	0	0	0	1		
Vehicle	Day 43	Nerve Median, Distal, Left, Distal - Nerve, Sciatic, Distal, Left, Distal	0	0	0	0	1		
Vehicle	Day 43	Nerve Median, Distal, Left, Distal - Nerve, Sciatic, Distal, Left, Proximal	0	0	0	0	1		
Vehicle	Day 43	Nerve Median, Distal, Left, Distal - Nerve, Sciatic, Distal, Right, Distal	0	0	0	0	1		
Vehicle	Day 43	Nerve Median, Distal, Left, Distal - Nerve, Sciatic, Distal, Right, Proximal	0	0	0	0	1		
Vehicle	Day 43	Nerve Median, Distal, Left, Distal - Nerve, Sciatic, Proximal, Left, Distal	0	0	0	0	1		
Vehicle	Day 43	Nerve Median, Distal, Left, Distal - Nerve, Sciatic, Proximal, Left, Proximal	0	0	0	0	1		
Vehicle	Day 43	Nerve Median, Distal, Left, Distal - Nerve, Sciatic, Proximal, Right, Distal	0	0	0	0	1		
Vehicle	Day 43	Nerve Median, Distal, Left, Distal - Nerve, Sciatic, Proximal, Right, Proximal	0	0	0	0	1		
Vehicle	Day 43	Nerve Median, Distal, Left, Distal - Nerve, Ulnar, Distal, Left, Distal	0	0	0	0	1		
Vehicle	Day 43	Nerve Median, Distal, Left, Distal - Nerve, Ulnar, Distal, Left, Proximal	0	0	0	0	1		
Vehicle	Day 43	Nerve Median, Distal, Left, Distal - Nerve, Ulnar, Distal, Right, Distal	0	0	0	0	1		
Vehicle	Day 43	Nerve Median, Distal, Left, Distal - Nerve, Ulnar, Distal, Right, Proximal	0	0	0	0	1		
Vehicle	Day 43	Nerve Median, Distal, Left, Distal - Nerve, Ulnar, Proximal, Left, Distal	0	0	0	0	1		
Vehicle	Day 43	Nerve Median, Distal, Left, Distal - Nerve, Ulnar, Proximal, Left, Proximal	0	0	0	0	1		
Vehicle	Day 43	Nerve Median, Distal, Left, Distal - Nerve, Ulnar, Proximal, Right, Distal	0	0	0	0	1		
Vehicle	Day 43	Nerve Median, Distal, Left, Distal - Nerve, Ulnar, Proximal, Right, Proximal	0	0	0	0	1		
Vehicle	Day 43	Nerve Median, Distal, Left, Distal - Nerve, Optic	0	0	0	0	1		
Vehicle	Day 43	Nerve Median, Distal, Left, Distal - Ovary	0				0		
Vehicle	Day 43	Nerve Median, Distal, Left, Distal - Spinal Cord, Cervical	-0.0001082	0.000153018	-0.001483016	0.001266615	1	-1	0.5
Vehicle	Day 43	Nerve Median, Distal, Left, Distal - Spinal Cord, Lumbar	0	0	0	0	1		
Vehicle	Day 43	Nerve Median, Distal, Left, Distal - Spinal Cord, Sacral	0	0	0	0	1		
Vehicle	Day 43	Nerve Median, Distal, Left, Distal - Spinal Cord, Spinal Roots	0	0	0	0	1		
Vehicle	Day 43	Nerve Median, Distal, Left, Distal - Spinal Cord, Thoracic	0	0	0	0	1		
Vehicle	Day 43	Nerve Median, Distal, Left, Distal - Spleen	-0.000216745	0.000306524	-0.002970753	0.002537263	1	-1	0.5
Vehicle	Day 43	Nerve Median, Distal, Left, Distal - Testis	0				0		
Vehicle	Day 43	Nerve Median, Distal, Left, Proximal - Nerve Median, Distal, Right, Distal	0	0	0	0	1		
Vehicle	Day 43	Nerve Median, Distal, Left, Proximal - Nerve Median, Distal, Right, Proximal	0	0	0	0	1		
Vehicle	Day 43	Nerve Median, Distal, Left, Proximal - Nerve Median, Proximal, Left, Distal	0	0	0	0	1		
Vehicle	Day 43	Nerve Median, Distal, Left, Proximal - Nerve Median, Proximal, Left, Proximal	0	0	0	0	1		
Vehicle	Day 43	Nerve Median, Distal, Left, Proximal - Nerve Median, Proximal, Right, Distal	0	0	0	0	1		
Vehicle	Day 43	Nerve Median, Distal, Left, Proximal - Nerve Median, Proximal, Right, Proximal	0	0	0	0	1		
Vehicle	Day 43	Nerve Median, Distal, Left, Proximal - Nerve, Sciatic, Distal, Left, Distal	0	0	0	0	1		
Vehicle	Day 43	Nerve Median, Distal, Left, Proximal - Nerve, Sciatic, Distal, Left, Proximal	0	0	0	0	1		
Vehicle	Day 43	Nerve Median, Distal, Left, Proximal - Nerve, Sciatic, Distal, Right, Distal	0	0	0	0	1		
Vehicle	Day 43	Nerve Median, Distal, Left, Proximal - Nerve, Sciatic, Distal, Right, Proximal	0	0	0	0	1		
Vehicle	Day 43	Nerve Median, Distal, Left, Proximal - Nerve, Sciatic, Proximal, Left, Distal	0	0	0	0	1		
Vehicle	Day 43	Nerve Median, Distal, Left, Proximal - Nerve, Sciatic, Proximal, Left, Proximal	0	0	0	0	1		
Vehicle	Day 43	Nerve Median, Distal, Left, Proximal - Nerve, Sciatic, Proximal, Right, Distal	0	0	0	0	1		
Vehicle	Day 43	Nerve Median, Distal, Left, Proximal - Nerve, Sciatic, Proximal, Right, Proximal	0	0	0	0	1		
Vehicle	Day 43	Nerve Median, Distal, Left, Proximal - Nerve, Ulnar, Distal, Left, Distal	0	0	0	0	1		
Vehicle	Day 43	Nerve Median, Distal, Left, Proximal - Nerve, Ulnar, Distal, Left, Proximal	0	0	0	0	1		
Vehicle	Day 43	Nerve Median, Distal, Left, Proximal - Nerve, Ulnar, Distal, Right, Distal	0	0	0	0	1		
Vehicle	Day 43	Nerve Median, Distal, Left, Proximal - Nerve, Ulnar, Distal, Right, Proximal	0	0	0	0	1		
Vehicle	Day 43	Nerve Median, Distal, Left, Proximal - Nerve, Ulnar, Proximal, Left, Distal	0	0	0	0	1		
Vehicle	Day 43	Nerve Median, Distal, Left, Proximal - Nerve, Ulnar, Proximal, Left, Proximal	0	0	0	0	1		
Vehicle	Day 43	Nerve Median, Distal, Left, Proximal - Nerve, Ulnar, Proximal, Right, Distal	0	0	0	0	1		
Vehicle	Day 43	Nerve Median, Distal, Left, Proximal - Nerve, Ulnar, Proximal, Right, Proximal	0	0	0	0	1		
Vehicle	Day 43	Nerve Median, Distal, Left, Proximal - Nerve, Optic	0	0	0	0	1		
Vehicle	Day 43	Nerve Median, Distal, Left, Proximal - Ovary	0				0		
Vehicle	Day 43	Nerve Median, Distal, Left, Proximal - Spinal Cord, Cervical	-0.0001082	0.000153018	-0.001483016	0.001266615	1	-1	0.5
Vehicle	Day 43	Nerve Median, Distal, Left, Proximal - Spinal Cord, Lumbar	0	0	0	0	1		
Vehicle	Day 43	Nerve Median, Distal, Left, Proximal - Spinal Cord, Sacral	0	0	0	0	1		
Vehicle	Day 43	Nerve Median, Distal, Left, Proximal - Spinal Cord, Spinal Roots	0	0	0	0	1		
Vehicle	Day 43	Nerve Median, Distal, Left, Proximal - Spinal Cord, Thoracic	0	0	0	0	1		
Vehicle	Day 43	Nerve Median, Distal, Left, Proximal - Spleen	-0.000216745	0.000306524	-0.002970753	0.002537263	1	-1	0.5
Vehicle	Day 43	Nerve Median, Distal, Left, Proximal - Testis	0				0		
Vehicle	Day 43	Nerve Median, Distal, Right, Distal - Nerve Median, Distal, Right, Proximal	0	0	0	0	1		
Vehicle	Day 43	Nerve Median, Distal, Right, Distal - Nerve Median, Proximal, Left, Distal	0	0	0	0	1		
Vehicle	Day 43	Nerve Median, Distal, Right, Distal - Nerve Median, Proximal, Left, Proximal	0	0	0	0	1		
Vehicle	Day 43	Nerve Median, Distal, Right, Distal - Nerve Median, Proximal, Right, Distal	0	0	0	0	1		
Vehicle	Day 43	Nerve Median, Distal, Right, Distal - Nerve Median, Proximal, Right, Proximal	0	0	0	0	1		
Vehicle	Day 43	Nerve Median, Distal, Right, Distal - Nerve, Sciatic, Distal, Left, Distal	0	0	0	0	1		
Vehicle	Day 43	Nerve Median, Distal, Right, Distal - Nerve, Sciatic, Distal, Left, Proximal	0	0	0	0	1		
Vehicle	Day 43	Nerve Median, Distal, Right, Distal - Nerve, Sciatic, Distal, Right, Distal	0	0	0	0	1		
Vehicle	Day 43	Nerve Median, Distal, Right, Distal - Nerve, Sciatic, Distal, Right, Proximal	0	0	0	0	1		
Vehicle	Day 43	Nerve Median, Distal, Right, Distal - Nerve, Sciatic, Proximal, Left, Distal	0	0	0	0	1		
Vehicle	Day 43	Nerve Median, Distal, Right, Distal - Nerve, Sciatic, Proximal, Left, Proximal	0	0	0	0	1		
Vehicle	Day 43	Nerve Median, Distal, Right, Distal - Nerve, Sciatic, Proximal, Right, Distal	0	0	0	0	1		
Vehicle	Day 43	Nerve Median, Distal, Right, Distal - Nerve, Sciatic, Proximal, Right, Proximal	0	0	0	0	1		
Vehicle	Day 43	Nerve Median, Distal, Right, Distal - Nerve, Ulnar, Distal, Left, Distal	0	0	0	0	1		
Vehicle	Day 43	Nerve Median, Distal, Right, Distal - Nerve, Ulnar, Distal, Left, Proximal	0	0	0	0	1		
Vehicle	Day 43	Nerve Median, Distal, Right, Distal - Nerve, Ulnar, Distal, Right, Distal	0	0	0	0	1		
Vehicle	Day 43	Nerve Median, Distal, Right, Distal - Nerve, Ulnar, Distal, Right, Proximal	0	0	0	0	1		

Results of Evaluating Mean Differences Between Different Tissues Across Timepoint and Group for Log10 AAV2-ITR Target Copies/ug of DNA for Normalized Data

Group	Timepoint	Difference	Mean	StdDev	LowerCLMean	UpperCLMean	DF	tValue	P-Value
Vehicle	Day 43	Nerve Median, Distal, Right, Distal - Nerve, Ulnar, Proximal, Left, Distal	0	0	0	0	1		
Vehicle	Day 43	Nerve Median, Distal, Right, Distal - Nerve, Ulnar, Proximal, Left, Proximal	0	0	0	0	1		
Vehicle	Day 43	Nerve Median, Distal, Right, Distal - Nerve, Ulnar, Proximal, Right, Distal	0	0	0	0	1		
Vehicle	Day 43	Nerve Median, Distal, Right, Distal - Nerve, Ulnar, Proximal, Right, Proximal	0	0	0	0	1		
Vehicle	Day 43	Nerve Median, Distal, Right, Distal - Nerve, Optic	0	0	0	0	1		
Vehicle	Day 43	Nerve Median, Distal, Right, Distal - Ovary	0				0		
Vehicle	Day 43	Nerve Median, Distal, Right, Distal - Spinal Cord, Cervical	-0.0001082	0.000153018	-0.001483016	0.001266615	1	-1	0.5
Vehicle	Day 43	Nerve Median, Distal, Right, Distal - Spinal Cord, Lumbar	0	0	0	0	1		
Vehicle	Day 43	Nerve Median, Distal, Right, Distal - Spinal Cord, Sacral	0	0	0	0	1		
Vehicle	Day 43	Nerve Median, Distal, Right, Distal - Spinal Cord, Spinal Roots	0	0	0	0	1		
Vehicle	Day 43	Nerve Median, Distal, Right, Distal - Spinal Cord, Thoracic	0	0	0	0	1		
Vehicle	Day 43	Nerve Median, Distal, Right, Distal - Spleen	-0.000216745	0.000306524	-0.002970753	0.002537263	1	-1	0.5
Vehicle	Day 43	Nerve Median, Distal, Right, Distal - Testis	0				0		
Vehicle	Day 43	Nerve Median, Distal, Right, Proximal - Nerve Median, Proximal, Left, Distal	0	0	0	0	1		
Vehicle	Day 43	Nerve Median, Distal, Right, Proximal - Nerve Median, Proximal, Left, Proximal	0	0	0	0	1		
Vehicle	Day 43	Nerve Median, Distal, Right, Proximal - Nerve Median, Proximal, Right, Distal	0	0	0	0	1		
Vehicle	Day 43	Nerve Median, Distal, Right, Proximal - Nerve Median, Proximal, Right, Proximal	0	0	0	0	1		
Vehicle	Day 43	Nerve Median, Distal, Right, Proximal - Nerve, Sciatic, Distal, Left, Distal	0	0	0	0	1		
Vehicle	Day 43	Nerve Median, Distal, Right, Proximal - Nerve, Sciatic, Distal, Left, Proximal	0	0	0	0	1		
Vehicle	Day 43	Nerve Median, Distal, Right, Proximal - Nerve, Sciatic, Distal, Right, Distal	0	0	0	0	1		
Vehicle	Day 43	Nerve Median, Distal, Right, Proximal - Nerve, Sciatic, Distal, Right, Proximal	0	0	0	0	1		
Vehicle	Day 43	Nerve Median, Distal, Right, Proximal - Nerve, Sciatic, Proximal, Left, Distal	0	0	0	0	1		
Vehicle	Day 43	Nerve Median, Distal, Right, Proximal - Nerve, Sciatic, Proximal, Left, Proximal	0	0	0	0	1		
Vehicle	Day 43	Nerve Median, Distal, Right, Proximal - Nerve, Sciatic, Proximal, Right, Distal	0	0	0	0	1		
Vehicle	Day 43	Nerve Median, Distal, Right, Proximal - Nerve, Sciatic, Proximal, Right, Proximal	0	0	0	0	1		
Vehicle	Day 43	Nerve Median, Distal, Right, Proximal - Nerve, Ulnar, Distal, Left, Distal	0	0	0	0	1		
Vehicle	Day 43	Nerve Median, Distal, Right, Proximal - Nerve, Ulnar, Distal, Left, Proximal	0	0	0	0	1		
Vehicle	Day 43	Nerve Median, Distal, Right, Proximal - Nerve, Ulnar, Distal, Right, Distal	0	0	0	0	1		
Vehicle	Day 43	Nerve Median, Distal, Right, Proximal - Nerve, Ulnar, Distal, Right, Proximal	0	0	0	0	1		
Vehicle	Day 43	Nerve Median, Distal, Right, Proximal - Nerve, Ulnar, Proximal, Left, Distal	0	0	0	0	1		
Vehicle	Day 43	Nerve Median, Distal, Right, Proximal - Nerve, Ulnar, Proximal, Left, Proximal	0	0	0	0	1		
Vehicle	Day 43	Nerve Median, Distal, Right, Proximal - Nerve, Ulnar, Proximal, Right, Distal	0	0	0	0	1		
Vehicle	Day 43	Nerve Median, Distal, Right, Proximal - Nerve, Ulnar, Proximal, Right, Proximal	0	0	0	0	1		
Vehicle	Day 43	Nerve Median, Distal, Right, Proximal - Nerve, Optic	0	0	0	0	1		
Vehicle	Day 43	Nerve Median, Distal, Right, Proximal - Ovary	0				0		
Vehicle	Day 43	Nerve Median, Distal, Right, Proximal - Spinal Cord, Cervical	-0.0001082	0.000153018	-0.001483016	0.001266615	1	-1	0.5
Vehicle	Day 43	Nerve Median, Distal, Right, Proximal - Spinal Cord, Lumbar	0	0	0	0	1		
Vehicle	Day 43	Nerve Median, Distal, Right, Proximal - Spinal Cord, Sacral	0	0	0	0	1		
Vehicle	Day 43	Nerve Median, Distal, Right, Proximal - Spinal Cord, Spinal Roots	0	0	0	0	1		
Vehicle	Day 43	Nerve Median, Distal, Right, Proximal - Spinal Cord, Thoracic	0	0	0	0	1		
Vehicle	Day 43	Nerve Median, Distal, Right, Proximal - Spleen	-0.000216745	0.000306524	-0.002970753	0.002537263	1	-1	0.5
Vehicle	Day 43	Nerve Median, Distal, Right, Proximal - Testis	0				0		
Vehicle	Day 43	Nerve Median, Proximal, Left, Distal - Nerve Median, Proximal, Left, Proximal	0	0	0	0	1		
Vehicle	Day 43	Nerve Median, Proximal, Left, Distal - Nerve Median, Proximal, Right, Distal	0	0	0	0	1		
Vehicle	Day 43	Nerve Median, Proximal, Left, Distal - Nerve Median, Proximal, Right, Proximal	0	0	0	0	1		
Vehicle	Day 43	Nerve Median, Proximal, Left, Distal - Nerve, Sciatic, Distal, Left, Distal	0	0	0	0	1		
Vehicle	Day 43	Nerve Median, Proximal, Left, Distal - Nerve, Sciatic, Distal, Left, Proximal	0	0	0	0	1		
Vehicle	Day 43	Nerve Median, Proximal, Left, Distal - Nerve, Sciatic, Distal, Right, Distal	0	0	0	0	1		
Vehicle	Day 43	Nerve Median, Proximal, Left, Distal - Nerve, Sciatic, Distal, Right, Proximal	0	0	0	0	1		
Vehicle	Day 43	Nerve Median, Proximal, Left, Distal - Nerve, Sciatic, Proximal, Left, Distal	0	0	0	0	1		
Vehicle	Day 43	Nerve Median, Proximal, Left, Distal - Nerve, Sciatic, Proximal, Left, Proximal	0	0	0	0	1		
Vehicle	Day 43	Nerve Median, Proximal, Left, Distal - Nerve, Sciatic, Proximal, Right, Distal	0	0	0	0	1		
Vehicle	Day 43	Nerve Median, Proximal, Left, Distal - Nerve, Sciatic, Proximal, Right, Proximal	0	0	0	0	1		
Vehicle	Day 43	Nerve Median, Proximal, Left, Distal - Nerve, Ulnar, Distal, Left, Distal	0	0	0	0	1		
Vehicle	Day 43	Nerve Median, Proximal, Left, Distal - Nerve, Ulnar, Distal, Left, Proximal	0	0	0	0	1		
Vehicle	Day 43	Nerve Median, Proximal, Left, Distal - Nerve, Ulnar, Distal, Right, Distal	0	0	0	0	1		
Vehicle	Day 43	Nerve Median, Proximal, Left, Distal - Nerve, Ulnar, Distal, Right, Proximal	0	0	0	0	1		
Vehicle	Day 43	Nerve Median, Proximal, Left, Distal - Nerve, Ulnar, Proximal, Left, Distal	0	0	0	0	1		
Vehicle	Day 43	Nerve Median, Proximal, Left, Distal - Nerve, Ulnar, Proximal, Left, Proximal	0	0	0	0	1		
Vehicle	Day 43	Nerve Median, Proximal, Left, Distal - Nerve, Ulnar, Proximal, Right, Distal	0	0	0	0	1		
Vehicle	Day 43	Nerve Median, Proximal, Left, Distal - Nerve, Ulnar, Proximal, Right, Proximal	0	0	0	0	1		
Vehicle	Day 43	Nerve Median, Proximal, Left, Distal - Nerve, Optic	0	0	0	0	1		
Vehicle	Day 43	Nerve Median, Proximal, Left, Distal - Ovary	0				0		
Vehicle	Day 43	Nerve Median, Proximal, Left, Distal - Spinal Cord, Cervical	-0.0001082	0.000153018	-0.001483016	0.001266615	1	-1	0.5
Vehicle	Day 43	Nerve Median, Proximal, Left, Distal - Spinal Cord, Lumbar	0	0	0	0	1		
Vehicle	Day 43	Nerve Median, Proximal, Left, Distal - Spinal Cord, Sacral	0	0	0	0	1		
Vehicle	Day 43	Nerve Median, Proximal, Left, Distal - Spinal Cord, Spinal Roots	0	0	0	0	1		
Vehicle	Day 43	Nerve Median, Proximal, Left, Distal - Spinal Cord, Thoracic	0	0	0	0	1		
Vehicle	Day 43	Nerve Median, Proximal, Left, Distal - Spleen	-0.000216745	0.000306524	-0.002970753	0.002537263	1	-1	0.5
Vehicle	Day 43	Nerve Median, Proximal, Left, Distal - Testis	0				0		
Vehicle	Day 43	Nerve Median, Proximal, Left, Proximal - Nerve Median, Proximal, Right, Distal	0	0	0	0	1		
Vehicle	Day 43	Nerve Median, Proximal, Left, Proximal - Nerve Median, Proximal, Right, Proximal	0	0	0	0	1		
Vehicle	Day 43	Nerve Median, Proximal, Left, Proximal - Nerve, Sciatic, Distal, Left, Distal	0	0	0	0	1		
Vehicle	Day 43	Nerve Median, Proximal, Left, Proximal - Nerve, Sciatic, Distal, Left, Proximal	0	0	0	0	1		
Vehicle	Day 43	Nerve Median, Proximal, Left, Proximal - Nerve, Sciatic, Distal, Right, Distal	0	0	0	0	1		
Vehicle	Day 43	Nerve Median, Proximal, Left, Proximal - Nerve, Sciatic, Distal, Right, Proximal	0	0	0	0	1		
Vehicle	Day 43	Nerve Median, Proximal, Left, Proximal - Nerve, Sciatic, Proximal, Left, Distal	0	0	0	0	1		
Vehicle	Day 43	Nerve Median, Proximal, Left, Proximal - Nerve, Sciatic, Proximal, Left, Proximal	0	0	0	0	1		
Vehicle	Day 43	Nerve Median, Proximal, Left, Proximal - Nerve, Sciatic, Proximal, Right, Distal	0	0	0	0	1		
Vehicle	Day 43	Nerve Median, Proximal, Left, Proximal - Nerve, Sciatic, Proximal, Right, Proximal	0	0	0	0	1		
Vehicle	Day 43	Nerve Median, Proximal, Left, Proximal - Nerve, Ulnar, Distal, Left, Distal	0	0	0	0	1		
Vehicle	Day 43	Nerve Median, Proximal, Left, Proximal - Nerve, Ulnar, Distal, Left, Proximal	0	0	0	0	1		
Vehicle	Day 43	Nerve Median, Proximal, Left, Proximal - Nerve, Ulnar, Distal, Right, Distal	0	0	0	0	1		
Vehicle	Day 43	Nerve Median, Proximal, Left, Proximal - Nerve, Ulnar, Distal, Right, Proximal	0	0	0	0	1		
Vehicle	Day 43	Nerve Median, Proximal, Left, Proximal - Nerve, Ulnar, Proximal, Left, Distal	0	0	0	0	1		
Vehicle	Day 43	Nerve Median, Proximal, Left, Proximal - Nerve, Ulnar, Proximal, Left, Proximal	0	0	0	0	1		
Vehicle	Day 43	Nerve Median, Proximal, Left, Proximal - Nerve, Ulnar, Proximal, Right, Distal	0	0	0	0	1		
Vehicle	Day 43	Nerve Median, Proximal, Left, Proximal - Nerve, Ulnar, Proximal, Right, Proximal	0	0	0	0	1		
Vehicle	Day 43	Nerve Median, Proximal, Left, Proximal - Nerve, Optic	0	0	0	0	1		
Vehicle	Day 43	Nerve Median, Proximal, Left, Proximal - Ovary	0				0		
Vehicle	Day 43	Nerve Median, Proximal, Left, Proximal - Spinal Cord, Cervical	-0.0001082	0.000153018	-0.001483016	0.001266615	1	-1	0.5
Vehicle	Day 43	Nerve Median, Proximal, Left, Proximal - Spinal Cord, Lumbar	0	0	0	0	1		
Vehicle	Day 43	Nerve Median, Proximal, Left, Proximal - Spinal Cord, Sacral	0	0	0	0	1		

Results of Evaluating Mean Differences Between Different Tissues Across Timepoint and Group for Log10 AAV2-ITR Target Copies/ug of DNA for Normalized Data

Group	Timepoint	Difference	Mean	StdDev	LowerCLMean	UpperCLMean	DF	tValue	P-Value
Vehicle	Day 43	Nerve Median, Proximal, Left, Proximal - Spinal Cord, Spinal Roots	0	0	0	0	1		
Vehicle	Day 43	Nerve Median, Proximal, Left, Proximal - Spinal Cord, Thoracic	0	0	0	0	1		
Vehicle	Day 43	Nerve Median, Proximal, Left, Proximal - Spleen	-0.000216745	0.000306524	-0.002970753	0.002537263	1	-1	0.5
Vehicle	Day 43	Nerve Median, Proximal, Left, Proximal - Testis	0				0		
Vehicle	Day 43	Nerve Median, Proximal, Right, Distal - Nerve Median, Proximal, Right, Proximal	0	0	0	0	1		
Vehicle	Day 43	Nerve Median, Proximal, Right, Distal - Nerve, Sciatic, Distal, Left, Distal	0	0	0	0	1		
Vehicle	Day 43	Nerve Median, Proximal, Right, Distal - Nerve, Sciatic, Distal, Left, Proximal	0	0	0	0	1		
Vehicle	Day 43	Nerve Median, Proximal, Right, Distal - Nerve, Sciatic, Distal, Right, Distal	0	0	0	0	1		
Vehicle	Day 43	Nerve Median, Proximal, Right, Distal - Nerve, Sciatic, Distal, Right, Proximal	0	0	0	0	1		
Vehicle	Day 43	Nerve Median, Proximal, Right, Distal - Nerve, Sciatic, Proximal, Left, Distal	0	0	0	0	1		
Vehicle	Day 43	Nerve Median, Proximal, Right, Distal - Nerve, Sciatic, Proximal, Left, Proximal	0	0	0	0	1		
Vehicle	Day 43	Nerve Median, Proximal, Right, Distal - Nerve, Sciatic, Proximal, Right, Distal	0	0	0	0	1		
Vehicle	Day 43	Nerve Median, Proximal, Right, Distal - Nerve, Sciatic, Proximal, Right, Proximal	0	0	0	0	1		
Vehicle	Day 43	Nerve Median, Proximal, Right, Distal - Nerve, Ulnar, Distal, Left, Distal	0	0	0	0	1		
Vehicle	Day 43	Nerve Median, Proximal, Right, Distal - Nerve, Ulnar, Distal, Left, Proximal	0	0	0	0	1		
Vehicle	Day 43	Nerve Median, Proximal, Right, Distal - Nerve, Ulnar, Distal, Right, Distal	0	0	0	0	1		
Vehicle	Day 43	Nerve Median, Proximal, Right, Distal - Nerve, Ulnar, Distal, Right, Proximal	0	0	0	0	1		
Vehicle	Day 43	Nerve Median, Proximal, Right, Distal - Nerve, Ulnar, Proximal, Left, Distal	0	0	0	0	1		
Vehicle	Day 43	Nerve Median, Proximal, Right, Distal - Nerve, Ulnar, Proximal, Left, Proximal	0	0	0	0	1		
Vehicle	Day 43	Nerve Median, Proximal, Right, Distal - Nerve, Ulnar, Proximal, Right, Distal	0	0	0	0	1		
Vehicle	Day 43	Nerve Median, Proximal, Right, Distal - Nerve, Ulnar, Proximal, Right, Proximal	0	0	0	0	1		
Vehicle	Day 43	Nerve Median, Proximal, Right, Distal - Nerve, Optic	0	0	0	0	1		
Vehicle	Day 43	Nerve Median, Proximal, Right, Distal - Ovary	0				0		
Vehicle	Day 43	Nerve Median, Proximal, Right, Distal - Spinal Cord, Cervical	-0.0001082	0.000153018	-0.001483016	0.001266615	1	-1	0.5
Vehicle	Day 43	Nerve Median, Proximal, Right, Distal - Spinal Cord, Lumbar	0	0	0	0	1		
Vehicle	Day 43	Nerve Median, Proximal, Right, Distal - Spinal Cord, Sacral	0	0	0	0	1		
Vehicle	Day 43	Nerve Median, Proximal, Right, Distal - Spinal Cord, Spinal Roots	0	0	0	0	1		
Vehicle	Day 43	Nerve Median, Proximal, Right, Distal - Spinal Cord, Thoracic	0	0	0	0	1		
Vehicle	Day 43	Nerve Median, Proximal, Right, Distal - Spleen	-0.000216745	0.000306524	-0.002970753	0.002537263	1	-1	0.5
Vehicle	Day 43	Nerve Median, Proximal, Right, Distal - Testis	0				0		
Vehicle	Day 43	Nerve Median, Proximal, Right, Proximal - Nerve, Sciatic, Distal, Left, Distal	0	0	0	0	1		
Vehicle	Day 43	Nerve Median, Proximal, Right, Proximal - Nerve, Sciatic, Distal, Left, Proximal	0	0	0	0	1		
Vehicle	Day 43	Nerve Median, Proximal, Right, Proximal - Nerve, Sciatic, Distal, Right, Distal	0	0	0	0	1		
Vehicle	Day 43	Nerve Median, Proximal, Right, Proximal - Nerve, Sciatic, Distal, Right, Proximal	0	0	0	0	1		
Vehicle	Day 43	Nerve Median, Proximal, Right, Proximal - Nerve, Sciatic, Proximal, Left, Distal	0	0	0	0	1		
Vehicle	Day 43	Nerve Median, Proximal, Right, Proximal - Nerve, Sciatic, Proximal, Left, Proximal	0	0	0	0	1		
Vehicle	Day 43	Nerve Median, Proximal, Right, Proximal - Nerve, Sciatic, Proximal, Right, Distal	0	0	0	0	1		
Vehicle	Day 43	Nerve Median, Proximal, Right, Proximal - Nerve, Sciatic, Proximal, Right, Proximal	0	0	0	0	1		
Vehicle	Day 43	Nerve Median, Proximal, Right, Proximal - Nerve, Ulnar, Distal, Left, Distal	0	0	0	0	1		
Vehicle	Day 43	Nerve Median, Proximal, Right, Proximal - Nerve, Ulnar, Distal, Left, Proximal	0	0	0	0	1		
Vehicle	Day 43	Nerve Median, Proximal, Right, Proximal - Nerve, Ulnar, Distal, Right, Distal	0	0	0	0	1		
Vehicle	Day 43	Nerve Median, Proximal, Right, Proximal - Nerve, Ulnar, Distal, Right, Proximal	0	0	0	0	1		
Vehicle	Day 43	Nerve Median, Proximal, Right, Proximal - Nerve, Ulnar, Proximal, Left, Distal	0	0	0	0	1		
Vehicle	Day 43	Nerve Median, Proximal, Right, Proximal - Nerve, Ulnar, Proximal, Left, Proximal	0	0	0	0	1		
Vehicle	Day 43	Nerve Median, Proximal, Right, Proximal - Nerve, Ulnar, Proximal, Right, Distal	0	0	0	0	1		
Vehicle	Day 43	Nerve Median, Proximal, Right, Proximal - Nerve, Ulnar, Proximal, Right, Proximal	0	0	0	0	1		
Vehicle	Day 43	Nerve Median, Proximal, Right, Proximal - Nerve, Optic	0	0	0	0	1		
Vehicle	Day 43	Nerve Median, Proximal, Right, Proximal - Ovary	0				0		
Vehicle	Day 43	Nerve Median, Proximal, Right, Proximal - Spinal Cord, Cervical	-0.0001082	0.000153018	-0.001483016	0.001266615	1	-1	0.5
Vehicle	Day 43	Nerve Median, Proximal, Right, Proximal - Spinal Cord, Lumbar	0	0	0	0	1		
Vehicle	Day 43	Nerve Median, Proximal, Right, Proximal - Spinal Cord, Sacral	0	0	0	0	1		
Vehicle	Day 43	Nerve Median, Proximal, Right, Proximal - Spinal Cord, Spinal Roots	0	0	0	0	1		
Vehicle	Day 43	Nerve Median, Proximal, Right, Proximal - Spinal Cord, Thoracic	0	0	0	0	1		
Vehicle	Day 43	Nerve Median, Proximal, Right, Proximal - Spleen	-0.000216745	0.000306524	-0.002970753	0.002537263	1	-1	0.5
Vehicle	Day 43	Nerve Median, Proximal, Right, Proximal - Testis	0				0		
Vehicle	Day 43	Nerve, Sciatic, Distal, Left, Distal - Nerve, Sciatic, Distal, Left, Proximal	0	0	0	0	1		
Vehicle	Day 43	Nerve, Sciatic, Distal, Left, Distal - Nerve, Sciatic, Distal, Right, Distal	0	0	0	0	1		
Vehicle	Day 43	Nerve, Sciatic, Distal, Left, Distal - Nerve, Sciatic, Distal, Right, Proximal	0	0	0	0	1		
Vehicle	Day 43	Nerve, Sciatic, Distal, Left, Distal - Nerve, Sciatic, Proximal, Left, Distal	0	0	0	0	1		
Vehicle	Day 43	Nerve, Sciatic, Distal, Left, Distal - Nerve, Sciatic, Proximal, Left, Proximal	0	0	0	0	1		
Vehicle	Day 43	Nerve, Sciatic, Distal, Left, Distal - Nerve, Sciatic, Proximal, Right, Distal	0	0	0	0	1		
Vehicle	Day 43	Nerve, Sciatic, Distal, Left, Distal - Nerve, Sciatic, Proximal, Right, Proximal	0	0	0	0	1		
Vehicle	Day 43	Nerve, Sciatic, Distal, Left, Distal - Nerve, Ulnar, Distal, Left, Distal	0	0	0	0	1		
Vehicle	Day 43	Nerve, Sciatic, Distal, Left, Distal - Nerve, Ulnar, Distal, Left, Proximal	0	0	0	0	1		
Vehicle	Day 43	Nerve, Sciatic, Distal, Left, Distal - Nerve, Ulnar, Distal, Right, Distal	0	0	0	0	1		
Vehicle	Day 43	Nerve, Sciatic, Distal, Left, Distal - Nerve, Ulnar, Distal, Right, Proximal	0	0	0	0	1		
Vehicle	Day 43	Nerve, Sciatic, Distal, Left, Distal - Nerve, Ulnar, Proximal, Left, Distal	0	0	0	0	1		
Vehicle	Day 43	Nerve, Sciatic, Distal, Left, Distal - Nerve, Ulnar, Proximal, Left, Proximal	0	0	0	0	1		
Vehicle	Day 43	Nerve, Sciatic, Distal, Left, Distal - Nerve, Ulnar, Proximal, Right, Distal	0	0	0	0	1		
Vehicle	Day 43	Nerve, Sciatic, Distal, Left, Distal - Nerve, Ulnar, Proximal, Right, Proximal	0	0	0	0	1		
Vehicle	Day 43	Nerve, Sciatic, Distal, Left, Distal - Nerve, Optic	0	0	0	0	1		
Vehicle	Day 43	Nerve, Sciatic, Distal, Left, Distal - Ovary	0				0		
Vehicle	Day 43	Nerve, Sciatic, Distal, Left, Distal - Spinal Cord, Cervical	-0.0001082	0.000153018	-0.001483016	0.001266615	1	-1	0.5
Vehicle	Day 43	Nerve, Sciatic, Distal, Left, Distal - Spinal Cord, Lumbar	0	0	0	0	1		
Vehicle	Day 43	Nerve, Sciatic, Distal, Left, Distal - Spinal Cord, Sacral	0	0	0	0	1		
Vehicle	Day 43	Nerve, Sciatic, Distal, Left, Distal - Spinal Cord, Spinal Roots	0	0	0	0	1		
Vehicle	Day 43	Nerve, Sciatic, Distal, Left, Distal - Spinal Cord, Thoracic	0	0	0	0	1		
Vehicle	Day 43	Nerve, Sciatic, Distal, Left, Distal - Spleen	-0.000216745	0.000306524	-0.002970753	0.002537263	1	-1	0.5
Vehicle	Day 43	Nerve, Sciatic, Distal, Left, Distal - Testis	0				0		
Vehicle	Day 43	Nerve, Sciatic, Distal, Left, Proximal - Nerve, Sciatic, Distal, Right, Distal	0	0	0	0	1		
Vehicle	Day 43	Nerve, Sciatic, Distal, Left, Proximal - Nerve, Sciatic, Distal, Right, Proximal	0	0	0	0	1		
Vehicle	Day 43	Nerve, Sciatic, Distal, Left, Proximal - Nerve, Sciatic, Proximal, Left, Distal	0	0	0	0	1		
Vehicle	Day 43	Nerve, Sciatic, Distal, Left, Proximal - Nerve, Sciatic, Proximal, Left, Proximal	0	0	0	0	1		
Vehicle	Day 43	Nerve, Sciatic, Distal, Left, Proximal - Nerve, Sciatic, Proximal, Right, Distal	0	0	0	0	1		
Vehicle	Day 43	Nerve, Sciatic, Distal, Left, Proximal - Nerve, Sciatic, Proximal, Right, Proximal	0	0	0	0	1		
Vehicle	Day 43	Nerve, Sciatic, Distal, Left, Proximal - Nerve, Ulnar, Distal, Left, Distal	0	0	0	0	1		
Vehicle	Day 43	Nerve, Sciatic, Distal, Left, Proximal - Nerve, Ulnar, Distal, Left, Proximal	0	0	0	0	1		
Vehicle	Day 43	Nerve, Sciatic, Distal, Left, Proximal - Nerve, Ulnar, Distal, Right, Distal	0	0	0	0	1		
Vehicle	Day 43	Nerve, Sciatic, Distal, Left, Proximal - Nerve, Ulnar, Distal, Right, Proximal	0	0	0	0	1		
Vehicle	Day 43	Nerve, Sciatic, Distal, Left, Proximal - Nerve, Ulnar, Proximal, Left, Distal	0	0	0	0	1		
Vehicle	Day 43	Nerve, Sciatic, Distal, Left, Proximal - Nerve, Ulnar, Proximal, Left, Proximal	0	0	0	0	1		
Vehicle	Day 43	Nerve, Sciatic, Distal, Left, Proximal - Nerve, Ulnar, Proximal, Right, Distal	0	0	0	0	1		
Vehicle	Day 43	Nerve, Sciatic, Distal, Left, Proximal - Nerve, Ulnar, Proximal, Right, Proximal	0	0	0	0	1		

Results of Evaluating Mean Differences Between Different Tissues Across Timepoint and Group for Log10 AAV2-ITR Target Copies/ug of DNA for Normalized Data

Group	Timepoint	Difference	Mean	StdDev	LowerCLMean	UpperCLMean	DF	tValue	P-Value
Vehicle	Day 43	Nerve, Sciatic, Distal, Left, Proximal - Nerve, Optic	0	0	0	0	1		
Vehicle	Day 43	Nerve, Sciatic, Distal, Left, Proximal - Ovary	0				0		
Vehicle	Day 43	Nerve, Sciatic, Distal, Left, Proximal - Spinal Cord, Cervical	-0.0001082	0.000153018	-0.001483016	0.001266615	1	-1	0.5
Vehicle	Day 43	Nerve, Sciatic, Distal, Left, Proximal - Spinal Cord, Lumbar	0	0	0	0	1		
Vehicle	Day 43	Nerve, Sciatic, Distal, Left, Proximal - Spinal Cord, Sacral	0	0	0	0	1		
Vehicle	Day 43	Nerve, Sciatic, Distal, Left, Proximal - Spinal Cord, Spinal Roots	0	0	0	0	1		
Vehicle	Day 43	Nerve, Sciatic, Distal, Left, Proximal - Spinal Cord, Thoracic	0	0	0	0	1		
Vehicle	Day 43	Nerve, Sciatic, Distal, Left, Proximal - Spleen	-0.000216745	0.000306524	-0.002970753	0.002537263	1	-1	0.5
Vehicle	Day 43	Nerve, Sciatic, Distal, Left, Proximal - Testis	0				0		
Vehicle	Day 43	Nerve, Sciatic, Distal, Right, Distal - Nerve, Sciatic, Distal, Right, Proximal	0	0	0	0	1		
Vehicle	Day 43	Nerve, Sciatic, Distal, Right, Distal - Nerve, Sciatic, Proximal, Left, Distal	0	0	0	0	1		
Vehicle	Day 43	Nerve, Sciatic, Distal, Right, Distal - Nerve, Sciatic, Proximal, Left, Proximal	0	0	0	0	1		
Vehicle	Day 43	Nerve, Sciatic, Distal, Right, Distal - Nerve, Sciatic, Proximal, Right, Distal	0	0	0	0	1		
Vehicle	Day 43	Nerve, Sciatic, Distal, Right, Distal - Nerve, Sciatic, Proximal, Right, Proximal	0	0	0	0	1		
Vehicle	Day 43	Nerve, Sciatic, Distal, Right, Distal - Nerve, Ulnar, Distal, Left, Distal	0	0	0	0	1		
Vehicle	Day 43	Nerve, Sciatic, Distal, Right, Distal - Nerve, Ulnar, Distal, Left, Proximal	0	0	0	0	1		
Vehicle	Day 43	Nerve, Sciatic, Distal, Right, Distal - Nerve, Ulnar, Distal, Right, Distal	0	0	0	0	1		
Vehicle	Day 43	Nerve, Sciatic, Distal, Right, Distal - Nerve, Ulnar, Distal, Right, Proximal	0	0	0	0	1		
Vehicle	Day 43	Nerve, Sciatic, Distal, Right, Distal - Nerve, Ulnar, Proximal, Left, Distal	0	0	0	0	1		
Vehicle	Day 43	Nerve, Sciatic, Distal, Right, Distal - Nerve, Ulnar, Proximal, Left, Proximal	0	0	0	0	1		
Vehicle	Day 43	Nerve, Sciatic, Distal, Right, Distal - Nerve, Ulnar, Proximal, Right, Distal	0	0	0	0	1		
Vehicle	Day 43	Nerve, Sciatic, Distal, Right, Distal - Nerve, Ulnar, Proximal, Right, Proximal	0	0	0	0	1		
Vehicle	Day 43	Nerve, Sciatic, Distal, Right, Distal - Nerve, Optic	0	0	0	0	1		
Vehicle	Day 43	Nerve, Sciatic, Distal, Right, Distal - Ovary	0				0		
Vehicle	Day 43	Nerve, Sciatic, Distal, Right, Distal - Spinal Cord, Cervical	-0.0001082	0.000153018	-0.001483016	0.001266615	1	-1	0.5
Vehicle	Day 43	Nerve, Sciatic, Distal, Right, Distal - Spinal Cord, Lumbar	0	0	0	0	1		
Vehicle	Day 43	Nerve, Sciatic, Distal, Right, Distal - Spinal Cord, Sacral	0	0	0	0	1		
Vehicle	Day 43	Nerve, Sciatic, Distal, Right, Distal - Spinal Cord, Spinal Roots	0	0	0	0	1		
Vehicle	Day 43	Nerve, Sciatic, Distal, Right, Distal - Spinal Cord, Thoracic	0	0	0	0	1		
Vehicle	Day 43	Nerve, Sciatic, Distal, Right, Distal - Spleen	-0.000216745	0.000306524	-0.002970753	0.002537263	1	-1	0.5
Vehicle	Day 43	Nerve, Sciatic, Distal, Right, Distal - Testis	0				0		
Vehicle	Day 43	Nerve, Sciatic, Distal, Right, Proximal - Nerve, Sciatic, Proximal, Left, Distal	0	0	0	0	1		
Vehicle	Day 43	Nerve, Sciatic, Distal, Right, Proximal - Nerve, Sciatic, Proximal, Left, Proximal	0	0	0	0	1		
Vehicle	Day 43	Nerve, Sciatic, Distal, Right, Proximal - Nerve, Sciatic, Proximal, Right, Distal	0	0	0	0	1		
Vehicle	Day 43	Nerve, Sciatic, Distal, Right, Proximal - Nerve, Sciatic, Proximal, Right, Proximal	0	0	0	0	1		
Vehicle	Day 43	Nerve, Sciatic, Distal, Right, Proximal - Nerve, Ulnar, Distal, Left, Distal	0	0	0	0	1		
Vehicle	Day 43	Nerve, Sciatic, Distal, Right, Proximal - Nerve, Ulnar, Distal, Left, Proximal	0	0	0	0	1		
Vehicle	Day 43	Nerve, Sciatic, Distal, Right, Proximal - Nerve, Ulnar, Distal, Right, Distal	0	0	0	0	1		
Vehicle	Day 43	Nerve, Sciatic, Distal, Right, Proximal - Nerve, Ulnar, Distal, Right, Proximal	0	0	0	0	1		
Vehicle	Day 43	Nerve, Sciatic, Distal, Right, Proximal - Nerve, Ulnar, Proximal, Left, Distal	0	0	0	0	1		
Vehicle	Day 43	Nerve, Sciatic, Distal, Right, Proximal - Nerve, Ulnar, Proximal, Left, Proximal	0	0	0	0	1		
Vehicle	Day 43	Nerve, Sciatic, Distal, Right, Proximal - Nerve, Ulnar, Proximal, Right, Distal	0	0	0	0	1		
Vehicle	Day 43	Nerve, Sciatic, Distal, Right, Proximal - Nerve, Ulnar, Proximal, Right, Proximal	0	0	0	0	1		
Vehicle	Day 43	Nerve, Sciatic, Distal, Right, Proximal - Nerve, Optic	0	0	0	0	1		
Vehicle	Day 43	Nerve, Sciatic, Distal, Right, Proximal - Ovary	0				0		
Vehicle	Day 43	Nerve, Sciatic, Distal, Right, Proximal - Spinal Cord, Cervical	-0.0001082	0.000153018	-0.001483016	0.001266615	1	-1	0.5
Vehicle	Day 43	Nerve, Sciatic, Distal, Right, Proximal - Spinal Cord, Lumbar	0	0	0	0	1		
Vehicle	Day 43	Nerve, Sciatic, Distal, Right, Proximal - Spinal Cord, Sacral	0	0	0	0	1		
Vehicle	Day 43	Nerve, Sciatic, Distal, Right, Proximal - Spinal Cord, Spinal Roots	0	0	0	0	1		
Vehicle	Day 43	Nerve, Sciatic, Distal, Right, Proximal - Spinal Cord, Thoracic	0	0	0	0	1		
Vehicle	Day 43	Nerve, Sciatic, Distal, Right, Proximal - Spleen	-0.000216745	0.000306524	-0.002970753	0.002537263	1	-1	0.5
Vehicle	Day 43	Nerve, Sciatic, Distal, Right, Proximal - Testis	0				0		
Vehicle	Day 43	Nerve, Sciatic, Proximal, Left, Distal - Nerve, Sciatic, Proximal, Left, Proximal	0	0	0	0	1		
Vehicle	Day 43	Nerve, Sciatic, Proximal, Left, Distal - Nerve, Sciatic, Proximal, Right, Distal	0	0	0	0	1		
Vehicle	Day 43	Nerve, Sciatic, Proximal, Left, Distal - Nerve, Sciatic, Proximal, Right, Proximal	0	0	0	0	1		
Vehicle	Day 43	Nerve, Sciatic, Proximal, Left, Distal - Nerve, Ulnar, Distal, Left, Distal	0	0	0	0	1		
Vehicle	Day 43	Nerve, Sciatic, Proximal, Left, Distal - Nerve, Ulnar, Distal, Left, Proximal	0	0	0	0	1		
Vehicle	Day 43	Nerve, Sciatic, Proximal, Left, Distal - Nerve, Ulnar, Distal, Right, Distal	0	0	0	0	1		
Vehicle	Day 43	Nerve, Sciatic, Proximal, Left, Distal - Nerve, Ulnar, Distal, Right, Proximal	0	0	0	0	1		
Vehicle	Day 43	Nerve, Sciatic, Proximal, Left, Distal - Nerve, Ulnar, Proximal, Left, Distal	0	0	0	0	1		
Vehicle	Day 43	Nerve, Sciatic, Proximal, Left, Distal - Nerve, Ulnar, Proximal, Left, Proximal	0	0	0	0	1		
Vehicle	Day 43	Nerve, Sciatic, Proximal, Left, Distal - Nerve, Ulnar, Proximal, Right, Distal	0	0	0	0	1		
Vehicle	Day 43	Nerve, Sciatic, Proximal, Left, Distal - Nerve, Ulnar, Proximal, Right, Proximal	0	0	0	0	1		
Vehicle	Day 43	Nerve, Sciatic, Proximal, Left, Distal - Nerve, Optic	0	0	0	0	1		
Vehicle	Day 43	Nerve, Sciatic, Proximal, Left, Distal - Ovary	0				0		
Vehicle	Day 43	Nerve, Sciatic, Proximal, Left, Distal - Spinal Cord, Cervical	-0.0001082	0.000153018	-0.001483016	0.001266615	1	-1	0.5
Vehicle	Day 43	Nerve, Sciatic, Proximal, Left, Distal - Spinal Cord, Lumbar	0	0	0	0	1		
Vehicle	Day 43	Nerve, Sciatic, Proximal, Left, Distal - Spinal Cord, Sacral	0	0	0	0	1		
Vehicle	Day 43	Nerve, Sciatic, Proximal, Left, Distal - Spinal Cord, Spinal Roots	0	0	0	0	1		
Vehicle	Day 43	Nerve, Sciatic, Proximal, Left, Distal - Spinal Cord, Thoracic	0	0	0	0	1		
Vehicle	Day 43	Nerve, Sciatic, Proximal, Left, Distal - Spleen	-0.000216745	0.000306524	-0.002970753	0.002537263	1	-1	0.5
Vehicle	Day 43	Nerve, Sciatic, Proximal, Left, Distal - Testis	0				0		
Vehicle	Day 43	Nerve, Sciatic, Proximal, Left, Proximal - Nerve, Sciatic, Proximal, Right, Distal	0	0	0	0	1		
Vehicle	Day 43	Nerve, Sciatic, Proximal, Left, Proximal - Nerve, Sciatic, Proximal, Right, Proximal	0	0	0	0	1		
Vehicle	Day 43	Nerve, Sciatic, Proximal, Left, Proximal - Nerve, Ulnar, Distal, Left, Distal	0	0	0	0	1		
Vehicle	Day 43	Nerve, Sciatic, Proximal, Left, Proximal - Nerve, Ulnar, Distal, Left, Proximal	0	0	0	0	1		
Vehicle	Day 43	Nerve, Sciatic, Proximal, Left, Proximal - Nerve, Ulnar, Distal, Right, Distal	0	0	0	0	1		
Vehicle	Day 43	Nerve, Sciatic, Proximal, Left, Proximal - Nerve, Ulnar, Distal, Right, Proximal	0	0	0	0	1		
Vehicle	Day 43	Nerve, Sciatic, Proximal, Left, Proximal - Nerve, Ulnar, Proximal, Left, Distal	0	0	0	0	1		
Vehicle	Day 43	Nerve, Sciatic, Proximal, Left, Proximal - Nerve, Ulnar, Proximal, Left, Proximal	0	0	0	0	1		
Vehicle	Day 43	Nerve, Sciatic, Proximal, Left, Proximal - Nerve, Ulnar, Proximal, Right, Distal	0	0	0	0	1		
Vehicle	Day 43	Nerve, Sciatic, Proximal, Left, Proximal - Nerve, Ulnar, Proximal, Right, Proximal	0	0	0	0	1		
Vehicle	Day 43	Nerve, Sciatic, Proximal, Left, Proximal - Nerve, Optic	0	0	0	0	1		
Vehicle	Day 43	Nerve, Sciatic, Proximal, Left, Proximal - Ovary	0				0		
Vehicle	Day 43	Nerve, Sciatic, Proximal, Left, Proximal - Spinal Cord, Cervical	-0.0001082	0.000153018	-0.001483016	0.001266615	1	-1	0.5
Vehicle	Day 43	Nerve, Sciatic, Proximal, Left, Proximal - Spinal Cord, Lumbar	0	0	0	0	1		
Vehicle	Day 43	Nerve, Sciatic, Proximal, Left, Proximal - Spinal Cord, Sacral	0	0	0	0	1		
Vehicle	Day 43	Nerve, Sciatic, Proximal, Left, Proximal - Spinal Cord, Spinal Roots	0	0	0	0	1		
Vehicle	Day 43	Nerve, Sciatic, Proximal, Left, Proximal - Spinal Cord, Thoracic	0	0	0	0	1		
Vehicle	Day 43	Nerve, Sciatic, Proximal, Left, Proximal - Spleen	-0.000216745	0.000306524	-0.002970753	0.002537263	1	-1	0.5
Vehicle	Day 43	Nerve, Sciatic, Proximal, Left, Proximal - Testis	0				0		
Vehicle	Day 43	Nerve, Sciatic, Proximal, Right, Distal - Nerve, Sciatic, Proximal, Right, Proximal	0	0	0	0	1		
Vehicle	Day 43	Nerve, Sciatic, Proximal, Right, Distal - Nerve, Ulnar, Distal, Left, Distal	0	0	0	0	1		

Results of Evaluating Mean Differences Between Different Tissues Across Timepoint and Group for Log10 AAV2-ITR Target Copies/ug of DNA for Normalized Data

Group	Timepoint	Difference	Mean	StdDev	LowerCLMean	UpperCLMean	DF	tValue	P-Value
Vehicle	Day 43	Nerve, Sciatic, Proximal, Right, Distal - Nerve, Ulnar, Distal, Left, Proximal	0	0	0	0	1		
Vehicle	Day 43	Nerve, Sciatic, Proximal, Right, Distal - Nerve, Ulnar, Distal, Right, Distal	0	0	0	0	1		
Vehicle	Day 43	Nerve, Sciatic, Proximal, Right, Distal - Nerve, Ulnar, Distal, Right, Proximal	0	0	0	0	1		
Vehicle	Day 43	Nerve, Sciatic, Proximal, Right, Distal - Nerve, Ulnar, Proximal, Left, Distal	0	0	0	0	1		
Vehicle	Day 43	Nerve, Sciatic, Proximal, Right, Distal - Nerve, Ulnar, Proximal, Left, Proximal	0	0	0	0	1		
Vehicle	Day 43	Nerve, Sciatic, Proximal, Right, Distal - Nerve, Ulnar, Proximal, Right, Distal	0	0	0	0	1		
Vehicle	Day 43	Nerve, Sciatic, Proximal, Right, Distal - Nerve, Ulnar, Proximal, Right, Proximal	0	0	0	0	1		
Vehicle	Day 43	Nerve, Sciatic, Proximal, Right, Distal - Nerve, Optic	0	0	0	0	1		
Vehicle	Day 43	Nerve, Sciatic, Proximal, Right, Distal - Ovary	0				0		
Vehicle	Day 43	Nerve, Sciatic, Proximal, Right, Distal - Spinal Cord, Cervical	-0.0001082	0.000153018	-0.001483016	0.001266615	1	-1	0.5
Vehicle	Day 43	Nerve, Sciatic, Proximal, Right, Distal - Spinal Cord, Lumbar	0	0	0	0	1		
Vehicle	Day 43	Nerve, Sciatic, Proximal, Right, Distal - Spinal Cord, Sacral	0	0	0	0	1		
Vehicle	Day 43	Nerve, Sciatic, Proximal, Right, Distal - Spinal Cord, Spinal Roots	0	0	0	0	1		
Vehicle	Day 43	Nerve, Sciatic, Proximal, Right, Distal - Spinal Cord, Thoracic	0	0	0	0	1		
Vehicle	Day 43	Nerve, Sciatic, Proximal, Right, Distal - Spleen	-0.000216745	0.000306524	-0.002970753	0.002537263	1	-1	0.5
Vehicle	Day 43	Nerve, Sciatic, Proximal, Right, Distal - Testis	0				0		
Vehicle	Day 43	Nerve, Sciatic, Proximal, Right, Proximal - Nerve, Ulnar, Distal, Left, Distal	0	0	0	0	1		
Vehicle	Day 43	Nerve, Sciatic, Proximal, Right, Proximal - Nerve, Ulnar, Distal, Left, Proximal	0	0	0	0	1		
Vehicle	Day 43	Nerve, Sciatic, Proximal, Right, Proximal - Nerve, Ulnar, Distal, Right, Distal	0	0	0	0	1		
Vehicle	Day 43	Nerve, Sciatic, Proximal, Right, Proximal - Nerve, Ulnar, Distal, Right, Proximal	0	0	0	0	1		
Vehicle	Day 43	Nerve, Sciatic, Proximal, Right, Proximal - Nerve, Ulnar, Proximal, Left, Distal	0	0	0	0	1		
Vehicle	Day 43	Nerve, Sciatic, Proximal, Right, Proximal - Nerve, Ulnar, Proximal, Left, Proximal	0	0	0	0	1		
Vehicle	Day 43	Nerve, Sciatic, Proximal, Right, Proximal - Nerve, Ulnar, Proximal, Right, Distal	0	0	0	0	1		
Vehicle	Day 43	Nerve, Sciatic, Proximal, Right, Proximal - Nerve, Ulnar, Proximal, Right, Proximal	0	0	0	0	1		
Vehicle	Day 43	Nerve, Sciatic, Proximal, Right, Proximal - Nerve, Optic	0	0	0	0	1		
Vehicle	Day 43	Nerve, Sciatic, Proximal, Right, Proximal - Ovary	0				0		
Vehicle	Day 43	Nerve, Sciatic, Proximal, Right, Proximal - Spinal Cord, Cervical	-0.0001082	0.000153018	-0.001483016	0.001266615	1	-1	0.5
Vehicle	Day 43	Nerve, Sciatic, Proximal, Right, Proximal - Spinal Cord, Lumbar	0	0	0	0	1		
Vehicle	Day 43	Nerve, Sciatic, Proximal, Right, Proximal - Spinal Cord, Sacral	0	0	0	0	1		
Vehicle	Day 43	Nerve, Sciatic, Proximal, Right, Proximal - Spinal Cord, Spinal Roots	0	0	0	0	1		
Vehicle	Day 43	Nerve, Sciatic, Proximal, Right, Proximal - Spinal Cord, Thoracic	0	0	0	0	1		
Vehicle	Day 43	Nerve, Sciatic, Proximal, Right, Proximal - Spleen	-0.000216745	0.000306524	-0.002970753	0.002537263	1	-1	0.5
Vehicle	Day 43	Nerve, Sciatic, Proximal, Right, Proximal - Testis	0				0		
Vehicle	Day 43	Nerve, Ulnar, Distal, Left, Distal - Nerve, Ulnar, Distal, Left, Proximal	0	0	0	0	1		
Vehicle	Day 43	Nerve, Ulnar, Distal, Left, Distal - Nerve, Ulnar, Distal, Right, Distal	0	0	0	0	1		
Vehicle	Day 43	Nerve, Ulnar, Distal, Left, Distal - Nerve, Ulnar, Distal, Right, Proximal	0	0	0	0	1		
Vehicle	Day 43	Nerve, Ulnar, Distal, Left, Distal - Nerve, Ulnar, Proximal, Left, Distal	0	0	0	0	1		
Vehicle	Day 43	Nerve, Ulnar, Distal, Left, Distal - Nerve, Ulnar, Proximal, Left, Proximal	0	0	0	0	1		
Vehicle	Day 43	Nerve, Ulnar, Distal, Left, Distal - Nerve, Ulnar, Proximal, Right, Distal	0	0	0	0	1		
Vehicle	Day 43	Nerve, Ulnar, Distal, Left, Distal - Nerve, Ulnar, Proximal, Right, Proximal	0	0	0	0	1		
Vehicle	Day 43	Nerve, Ulnar, Distal, Left, Distal - Nerve, Optic	0	0	0	0	1		
Vehicle	Day 43	Nerve, Ulnar, Distal, Left, Distal - Ovary	0				0		
Vehicle	Day 43	Nerve, Ulnar, Distal, Left, Distal - Spinal Cord, Cervical	-0.0001082	0.000153018	-0.001483016	0.001266615	1	-1	0.5
Vehicle	Day 43	Nerve, Ulnar, Distal, Left, Distal - Spinal Cord, Lumbar	0	0	0	0	1		
Vehicle	Day 43	Nerve, Ulnar, Distal, Left, Distal - Spinal Cord, Sacral	0	0	0	0	1		
Vehicle	Day 43	Nerve, Ulnar, Distal, Left, Distal - Spinal Cord, Spinal Roots	0	0	0	0	1		
Vehicle	Day 43	Nerve, Ulnar, Distal, Left, Distal - Spinal Cord, Thoracic	0	0	0	0	1		
Vehicle	Day 43	Nerve, Ulnar, Distal, Left, Distal - Spleen	-0.000216745	0.000306524	-0.002970753	0.002537263	1	-1	0.5
Vehicle	Day 43	Nerve, Ulnar, Distal, Left, Distal - Testis	0				0		
Vehicle	Day 43	Nerve, Ulnar, Distal, Left, Proximal - Nerve, Ulnar, Distal, Right, Distal	0	0	0	0	1		
Vehicle	Day 43	Nerve, Ulnar, Distal, Left, Proximal - Nerve, Ulnar, Distal, Right, Proximal	0	0	0	0	1		
Vehicle	Day 43	Nerve, Ulnar, Distal, Left, Proximal - Nerve, Ulnar, Proximal, Left, Distal	0	0	0	0	1		
Vehicle	Day 43	Nerve, Ulnar, Distal, Left, Proximal - Nerve, Ulnar, Proximal, Left, Proximal	0	0	0	0	1		
Vehicle	Day 43	Nerve, Ulnar, Distal, Left, Proximal - Nerve, Ulnar, Proximal, Right, Distal	0	0	0	0	1		
Vehicle	Day 43	Nerve, Ulnar, Distal, Left, Proximal - Nerve, Ulnar, Proximal, Right, Proximal	0	0	0	0	1		
Vehicle	Day 43	Nerve, Ulnar, Distal, Left, Proximal - Nerve, Optic	0	0	0	0	1		
Vehicle	Day 43	Nerve, Ulnar, Distal, Left, Proximal - Ovary	0				0		
Vehicle	Day 43	Nerve, Ulnar, Distal, Left, Proximal - Spinal Cord, Cervical	-0.0001082	0.000153018	-0.001483016	0.001266615	1	-1	0.5
Vehicle	Day 43	Nerve, Ulnar, Distal, Left, Proximal - Spinal Cord, Lumbar	0	0	0	0	1		
Vehicle	Day 43	Nerve, Ulnar, Distal, Left, Proximal - Spinal Cord, Sacral	0	0	0	0	1		
Vehicle	Day 43	Nerve, Ulnar, Distal, Left, Proximal - Spinal Cord, Spinal Roots	0	0	0	0	1		
Vehicle	Day 43	Nerve, Ulnar, Distal, Left, Proximal - Spinal Cord, Thoracic	0	0	0	0	1		
Vehicle	Day 43	Nerve, Ulnar, Distal, Left, Proximal - Spleen	-0.000216745	0.000306524	-0.002970753	0.002537263	1	-1	0.5
Vehicle	Day 43	Nerve, Ulnar, Distal, Left, Proximal - Testis	0				0		
Vehicle	Day 43	Nerve, Ulnar, Distal, Right, Distal - Nerve, Ulnar, Distal, Right, Proximal	0	0	0	0	1		
Vehicle	Day 43	Nerve, Ulnar, Distal, Right, Distal - Nerve, Ulnar, Proximal, Left, Distal	0	0	0	0	1		
Vehicle	Day 43	Nerve, Ulnar, Distal, Right, Distal - Nerve, Ulnar, Proximal, Left, Proximal	0	0	0	0	1		
Vehicle	Day 43	Nerve, Ulnar, Distal, Right, Distal - Nerve, Ulnar, Proximal, Right, Distal	0	0	0	0	1		
Vehicle	Day 43	Nerve, Ulnar, Distal, Right, Distal - Nerve, Ulnar, Proximal, Right, Proximal	0	0	0	0	1		
Vehicle	Day 43	Nerve, Ulnar, Distal, Right, Distal - Nerve, Optic	0	0	0	0	1		
Vehicle	Day 43	Nerve, Ulnar, Distal, Right, Distal - Ovary	0				0		
Vehicle	Day 43	Nerve, Ulnar, Distal, Right, Distal - Spinal Cord, Cervical	-0.0001082	0.000153018	-0.001483016	0.001266615	1	-1	0.5
Vehicle	Day 43	Nerve, Ulnar, Distal, Right, Distal - Spinal Cord, Lumbar	0	0	0	0	1		
Vehicle	Day 43	Nerve, Ulnar, Distal, Right, Distal - Spinal Cord, Sacral	0	0	0	0	1		
Vehicle	Day 43	Nerve, Ulnar, Distal, Right, Distal - Spinal Cord, Spinal Roots	0	0	0	0	1		
Vehicle	Day 43	Nerve, Ulnar, Distal, Right, Distal - Spinal Cord, Thoracic	0	0	0	0	1		
Vehicle	Day 43	Nerve, Ulnar, Distal, Right, Distal - Spleen	-0.000216745	0.000306524	-0.002970753	0.002537263	1	-1	0.5
Vehicle	Day 43	Nerve, Ulnar, Distal, Right, Distal - Testis	0				0		
Vehicle	Day 43	Nerve, Ulnar, Distal, Right, Proximal - Nerve, Ulnar, Proximal, Left, Distal	0	0	0	0	1		
Vehicle	Day 43	Nerve, Ulnar, Distal, Right, Proximal - Nerve, Ulnar, Proximal, Left, Proximal	0	0	0	0	1		
Vehicle	Day 43	Nerve, Ulnar, Distal, Right, Proximal - Nerve, Ulnar, Proximal, Right, Distal	0	0	0	0	1		
Vehicle	Day 43	Nerve, Ulnar, Distal, Right, Proximal - Nerve, Ulnar, Proximal, Right, Proximal	0	0	0	0	1		
Vehicle	Day 43	Nerve, Ulnar, Distal, Right, Proximal - Nerve, Optic	0	0	0	0	1		
Vehicle	Day 43	Nerve, Ulnar, Distal, Right, Proximal - Ovary	0				0		
Vehicle	Day 43	Nerve, Ulnar, Distal, Right, Proximal - Spinal Cord, Cervical	-0.0001082	0.000153018	-0.001483016	0.001266615	1	-1	0.5
Vehicle	Day 43	Nerve, Ulnar, Distal, Right, Proximal - Spinal Cord, Lumbar	0	0	0	0	1		
Vehicle	Day 43	Nerve, Ulnar, Distal, Right, Proximal - Spinal Cord, Sacral	0	0	0	0	1		
Vehicle	Day 43	Nerve, Ulnar, Distal, Right, Proximal - Spinal Cord, Spinal Roots	0	0	0	0	1		
Vehicle	Day 43	Nerve, Ulnar, Distal, Right, Proximal - Spinal Cord, Thoracic	0	0	0	0	1		
Vehicle	Day 43	Nerve, Ulnar, Distal, Right, Proximal - Spleen	-0.000216745	0.000306524	-0.002970753	0.002537263	1	-1	0.5
Vehicle	Day 43	Nerve, Ulnar, Distal, Right, Proximal - Testis	0				0		
Vehicle	Day 43	Nerve, Ulnar, Proximal, Left, Distal - Nerve, Ulnar, Proximal, Left, Proximal	0	0	0	0	1		
Vehicle	Day 43	Nerve, Ulnar, Proximal, Left, Distal - Nerve, Ulnar, Proximal, Right, Distal	0	0	0	0	1		

Results of Evaluating Mean Differences Between Different Tissues Across Timepoint and Group for Log10 AAV2-ITR Target Copies/ug of DNA for Normalized Data

Group	Timepoint	Difference	Mean	StdDev	LowerCLMean	UpperCLMean	DF	tValue	P-Value
Vehicle	Day 43	Nerve, Ulnar, Proximal, Left, Distal - Nerve, Ulnar, Proximal, Right, Proximal	0	0	0	0	1		
Vehicle	Day 43	Nerve, Ulnar, Proximal, Left, Distal - Nerve, Optic	0	0	0	0	1		
Vehicle	Day 43	Nerve, Ulnar, Proximal, Left, Distal - Ovary	0				0		
Vehicle	Day 43	Nerve, Ulnar, Proximal, Left, Distal - Spinal Cord, Cervical	-0.0001082	0.000153018	-0.001483016	0.001266615	1	-1	0.5
Vehicle	Day 43	Nerve, Ulnar, Proximal, Left, Distal - Spinal Cord, Lumbar	0	0	0	0	1		
Vehicle	Day 43	Nerve, Ulnar, Proximal, Left, Distal - Spinal Cord, Sacral	0	0	0	0	1		
Vehicle	Day 43	Nerve, Ulnar, Proximal, Left, Distal - Spinal Cord, Spinal Roots	0	0	0	0	1		
Vehicle	Day 43	Nerve, Ulnar, Proximal, Left, Distal - Spinal Cord, Thoracic	0	0	0	0	1		
Vehicle	Day 43	Nerve, Ulnar, Proximal, Left, Distal - Spleen	-0.000216745	0.000306524	-0.002970753	0.002537263	1	-1	0.5
Vehicle	Day 43	Nerve, Ulnar, Proximal, Left, Distal - Testis	0				0		
Vehicle	Day 43	Nerve, Ulnar, Proximal, Left, Proximal - Nerve, Ulnar, Proximal, Right, Distal	0	0	0	0	1		
Vehicle	Day 43	Nerve, Ulnar, Proximal, Left, Proximal - Nerve, Ulnar, Proximal, Right, Proximal	0	0	0	0	1		
Vehicle	Day 43	Nerve, Ulnar, Proximal, Left, Proximal - Nerve, Optic	0	0	0	0	1		
Vehicle	Day 43	Nerve, Ulnar, Proximal, Left, Proximal - Ovary	0				0		
Vehicle	Day 43	Nerve, Ulnar, Proximal, Left, Proximal - Spinal Cord, Cervical	-0.0001082	0.000153018	-0.001483016	0.001266615	1	-1	0.5
Vehicle	Day 43	Nerve, Ulnar, Proximal, Left, Proximal - Spinal Cord, Lumbar	0	0	0	0	1		
Vehicle	Day 43	Nerve, Ulnar, Proximal, Left, Proximal - Spinal Cord, Sacral	0	0	0	0	1		
Vehicle	Day 43	Nerve, Ulnar, Proximal, Left, Proximal - Spinal Cord, Spinal Roots	0	0	0	0	1		
Vehicle	Day 43	Nerve, Ulnar, Proximal, Left, Proximal - Spinal Cord, Thoracic	0	0	0	0	1		
Vehicle	Day 43	Nerve, Ulnar, Proximal, Left, Proximal - Spleen	-0.000216745	0.000306524	-0.002970753	0.002537263	1	-1	0.5
Vehicle	Day 43	Nerve, Ulnar, Proximal, Left, Proximal - Testis	0				0		
Vehicle	Day 43	Nerve, Ulnar, Proximal, Right, Distal - Nerve, Ulnar, Proximal, Right, Proximal	0	0	0	0	1		
Vehicle	Day 43	Nerve, Ulnar, Proximal, Right, Distal - Nerve, Optic	0	0	0	0	1		
Vehicle	Day 43	Nerve, Ulnar, Proximal, Right, Distal - Ovary	0				0		
Vehicle	Day 43	Nerve, Ulnar, Proximal, Right, Distal - Spinal Cord, Cervical	-0.0001082	0.000153018	-0.001483016	0.001266615	1	-1	0.5
Vehicle	Day 43	Nerve, Ulnar, Proximal, Right, Distal - Spinal Cord, Lumbar	0	0	0	0	1		
Vehicle	Day 43	Nerve, Ulnar, Proximal, Right, Distal - Spinal Cord, Sacral	0	0	0	0	1		
Vehicle	Day 43	Nerve, Ulnar, Proximal, Right, Distal - Spinal Cord, Spinal Roots	0	0	0	0	1		
Vehicle	Day 43	Nerve, Ulnar, Proximal, Right, Distal - Spinal Cord, Thoracic	0	0	0	0	1		
Vehicle	Day 43	Nerve, Ulnar, Proximal, Right, Distal - Spleen	-0.000216745	0.000306524	-0.002970753	0.002537263	1	-1	0.5
Vehicle	Day 43	Nerve, Ulnar, Proximal, Right, Distal - Testis	0				0		
Vehicle	Day 43	Nerve, Ulnar, Proximal, Right, Proximal - Nerve, Optic	0	0	0	0	1		
Vehicle	Day 43	Nerve, Ulnar, Proximal, Right, Proximal - Ovary	0				0		
Vehicle	Day 43	Nerve, Ulnar, Proximal, Right, Proximal - Spinal Cord, Cervical	-0.0001082	0.000153018	-0.001483016	0.001266615	1	-1	0.5
Vehicle	Day 43	Nerve, Ulnar, Proximal, Right, Proximal - Spinal Cord, Lumbar	0	0	0	0	1		
Vehicle	Day 43	Nerve, Ulnar, Proximal, Right, Proximal - Spinal Cord, Sacral	0	0	0	0	1		
Vehicle	Day 43	Nerve, Ulnar, Proximal, Right, Proximal - Spinal Cord, Spinal Roots	0	0	0	0	1		
Vehicle	Day 43	Nerve, Ulnar, Proximal, Right, Proximal - Spinal Cord, Thoracic	0	0	0	0	1		
Vehicle	Day 43	Nerve, Ulnar, Proximal, Right, Proximal - Spleen	-0.000216745	0.000306524	-0.002970753	0.002537263	1	-1	0.5
Vehicle	Day 43	Nerve, Ulnar, Proximal, Right, Proximal - Testis	0				0		
Vehicle	Day 43	Nerve, Optic - Ovary	0				0		
Vehicle	Day 43	Nerve, Optic - Spinal Cord, Cervical	-0.0001082	0.000153018	-0.001483016	0.001266615	1	-1	0.5
Vehicle	Day 43	Nerve, Optic - Spinal Cord, Lumbar	0	0	0	0	1		
Vehicle	Day 43	Nerve, Optic - Spinal Cord, Sacral	0	0	0	0	1		
Vehicle	Day 43	Nerve, Optic - Spinal Cord, Spinal Roots	0	0	0	0	1		
Vehicle	Day 43	Nerve, Optic - Spinal Cord, Thoracic	0	0	0	0	1		
Vehicle	Day 43	Nerve, Optic - Spleen	-0.000216745	0.000306524	-0.002970753	0.002537263	1	-1	0.5
Vehicle	Day 43	Nerve, Optic - Testis	0				0		
Vehicle	Day 43	Ovary - Spinal Cord, Cervical	-0.000216401				0		
Vehicle	Day 43	Ovary - Spinal Cord, Lumbar	0				0		
Vehicle	Day 43	Ovary - Spinal Cord, Sacral	0				0		
Vehicle	Day 43	Ovary - Spinal Cord, Spinal Roots	0				0		
Vehicle	Day 43	Ovary - Spinal Cord, Thoracic	0				0		
Vehicle	Day 43	Ovary - Spleen	0				0		
Vehicle	Day 43	Ovary - Testis					0		
Vehicle	Day 43	Spinal Cord, Cervical - Spinal Cord, Lumbar	0.0001082	0.000153018	-0.001266615	0.001483016	1	1	0.5
Vehicle	Day 43	Spinal Cord, Cervical - Spinal Cord, Sacral	0.0001082	0.000153018	-0.001266615	0.001483016	1	1	0.5
Vehicle	Day 43	Spinal Cord, Cervical - Spinal Cord, Spinal Roots	0.0001082	0.000153018	-0.001266615	0.001483016	1	1	0.5
Vehicle	Day 43	Spinal Cord, Cervical - Spinal Cord, Thoracic	0.0001082	0.000153018	-0.001266615	0.001483016	1	1	0.5
Vehicle	Day 43	Spinal Cord, Cervical - Spleen	-0.000108545	0.000459542	-0.004237368	0.004020279	1	-0.334040056	0.794762399
Vehicle	Day 43	Spinal Cord, Cervical - Testis	0				0		
Vehicle	Day 43	Spinal Cord, Lumbar - Spinal Cord, Sacral	0	0	0	0	1		
Vehicle	Day 43	Spinal Cord, Lumbar - Spinal Cord, Spinal Roots	0	0	0	0	1		
Vehicle	Day 43	Spinal Cord, Lumbar - Spinal Cord, Thoracic	0	0	0	0	1		
Vehicle	Day 43	Spinal Cord, Lumbar - Spleen	-0.000216745	0.000306524	-0.002970753	0.002537263	1	-1	0.5
Vehicle	Day 43	Spinal Cord, Lumbar - Testis	0				0		
Vehicle	Day 43	Spinal Cord, Sacral - Spinal Cord, Spinal Roots	0	0	0	0	1		
Vehicle	Day 43	Spinal Cord, Sacral - Spinal Cord, Thoracic	0	0	0	0	1		
Vehicle	Day 43	Spinal Cord, Sacral - Spleen	-0.000216745	0.000306524	-0.002970753	0.002537263	1	-1	0.5
Vehicle	Day 43	Spinal Cord, Sacral - Testis	0				0		
Vehicle	Day 43	Spinal Cord, Spinal Roots - Spinal Cord, Thoracic	0	0	0	0	1		
Vehicle	Day 43	Spinal Cord, Spinal Roots - Spleen	-0.000216745	0.000306524	-0.002970753	0.002537263	1	-1	0.5
Vehicle	Day 43	Spinal Cord, Spinal Roots - Testis	0				0		
Vehicle	Day 43	Spinal Cord, Thoracic - Spleen	-0.000216745	0.000306524	-0.002970753	0.002537263	1	-1	0.5
Vehicle	Day 43	Spinal Cord, Thoracic - Testis	0				0		
Vehicle	Day 43	Spleen - Testis	0.00043349				0		
Vehicle	Day 85	Brain - Ganglion, Dorsal Root, Cervical	7.31318E-05	0.000103424	-0.000856096	0.00100236	1	1	0.5
Vehicle	Day 85	Brain - Ganglion, Dorsal Root, Lumbar	-2.74981E-05	3.88882E-05	-0.000376894	0.000321898	1	-1	0.5
Vehicle	Day 85	Brain - Ganglion, Dorsal Root, Thoracic	7.31318E-05	0.000103424	-0.000856096	0.00100236	1	1	0.5
Vehicle	Day 85	Brain - Heart	7.31318E-05	0.000103424	-0.000856096	0.00100236	1	1	0.5
Vehicle	Day 85	Brain - Kidney	7.31318E-05	0.000103424	-0.000856096	0.00100236	1	1	0.5
Vehicle	Day 85	Brain - Lung	7.31318E-05	0.000103424	-0.000856096	0.00100236	1	1	0.5
Vehicle	Day 85	Brain - Liver	-0.000160379	0.00022681	-0.002198188	0.00187743	1	-1	0.5
Vehicle	Day 85	Brain - Muscle, Skeletal, Tibialis Anterior Left	1.77022E-05	2.50347E-05	-0.000207226	0.00024263	1	1	0.5
Vehicle	Day 85	Brain - Muscle, Skeletal, Tibialis Anterior Right	7.31318E-05	0.000103424	-0.000856096	0.00100236	1	1	0.5
Vehicle	Day 85	Brain - Muscle, Skeletal, Bicep Brachii Left	7.31318E-05	0.000103424	-0.000856096	0.00100236	1	1	0.5
Vehicle	Day 85	Brain - Muscle, Skeletal, Bicep Brachii Right	7.31318E-05	0.000103424	-0.000856096	0.00100236	1	1	0.5
Vehicle	Day 85	Brain - Nerve, Femoral, Distal, Left, Distal	7.31318E-05	0.000103424	-0.000856096	0.00100236	1	1	0.5
Vehicle	Day 85	Brain - Nerve, Femoral, Distal, Left, Proximal	2.8499E-05	0.000166544	-0.001467842	0.00152484	1	0.242000174	0.848843956
Vehicle	Day 85	Brain - Nerve, Femoral, Distal, Right, Distal	7.31318E-05	0.000103424	-0.000856096	0.00100236	1	1	0.5
Vehicle	Day 85	Brain - Nerve, Femoral, Distal, Right, Proximal	7.31318E-05	0.000103424	-0.000856096	0.00100236	1	1	0.5
Vehicle	Day 85	Brain - Nerve, Femoral, Proximal, Left, Distal	7.31318E-05	0.000103424	-0.000856096	0.00100236	1	1	0.5
Vehicle	Day 85	Brain - Nerve, Femoral, Proximal, Left, Proximal	7.31318E-05	0.000103424	-0.000856096	0.00100236	1	1	0.5

Results of Evaluating Mean Differences Between Different Tissues Across Timepoint and Group for Log10 AAV2-ITR Target Copies/ug of DNA for Normalized Data

Group	Timepoint	Difference	Mean	StdDev	LowerCLMean	UpperCLMean	DF	tValue	P-Value
Vehicle	Day 85	Brain - Nerve, Femoral, Proximal, Right, Distal	-1.24753E-05	0.000224491	-0.002029444	0.002004494	1	-0.078589909	0.950070735
Vehicle	Day 85	Brain - Nerve, Femoral, Proximal, Right, Proximal	7.31318E-05	0.000103424	-0.000856096	0.00100236	1	1	0.5
Vehicle	Day 85	Brain - Nerve Median, Distal, Left, Distal	7.31318E-05	0.000103424	-0.000856096	0.00100236	1	1	0.5
Vehicle	Day 85	Brain - Nerve Median, Distal, Left, Proximal	7.31318E-05	0.000103424	-0.000856096	0.00100236	1	1	0.5
Vehicle	Day 85	Brain - Nerve Median, Distal, Right, Distal	7.31318E-05	0.000103424	-0.000856096	0.00100236	1	1	0.5
Vehicle	Day 85	Brain - Nerve Median, Distal, Right, Proximal	7.31318E-05	0.000103424	-0.000856096	0.00100236	1	1	0.5
Vehicle	Day 85	Brain - Nerve Median, Proximal, Left, Distal	7.31318E-05	0.000103424	-0.000856096	0.00100236	1	1	0.5
Vehicle	Day 85	Brain - Nerve Median, Proximal, Left, Proximal	2.7299E-05	0.000168241	-0.00148429	0.001538888	1	0.229471545	0.856399954
Vehicle	Day 85	Brain - Nerve Median, Proximal, Right, Distal	7.31318E-05	0.000103424	-0.000856096	0.00100236	1	1	0.5
Vehicle	Day 85	Brain - Nerve Median, Proximal, Right, Proximal	7.31318E-05	0.000103424	-0.000856096	0.00100236	1	1	0.5
Vehicle	Day 85	Brain - Nerve, Sciatic, Distal, Left, Distal	-3.50651E-05	4.95895E-05	-0.000480609	0.000410479	1	-1	0.5
Vehicle	Day 85	Brain - Nerve, Sciatic, Distal, Left, Proximal	7.31318E-05	0.000103424	-0.000856096	0.00100236	1	1	0.5
Vehicle	Day 85	Brain - Nerve, Sciatic, Distal, Right, Distal	7.31318E-05	0.000103424	-0.000856096	0.00100236	1	1	0.5
Vehicle	Day 85	Brain - Nerve, Sciatic, Distal, Right, Proximal	7.31318E-05	0.000103424	-0.000856096	0.00100236	1	1	0.5
Vehicle	Day 85	Brain - Nerve, Sciatic, Proximal, Left, Distal	7.31318E-05	0.000103424	-0.000856096	0.00100236	1	1	0.5
Vehicle	Day 85	Brain - Nerve, Sciatic, Proximal, Left, Proximal	7.31318E-05	0.000103424	-0.000856096	0.00100236	1	1	0.5
Vehicle	Day 85	Brain - Nerve, Sciatic, Proximal, Right, Distal	-9.08613E-05	0.000128497	-0.001245364	0.001063641	1	-1	0.5
Vehicle	Day 85	Brain - Nerve, Sciatic, Proximal, Right, Proximal	-6.53558E-05	0.000299275	-0.002754236	0.002623524	1	-0.308836588	0.809304424
Vehicle	Day 85	Brain - Nerve, Ulnar, Distal, Left, Distal	7.31318E-05	0.000103424	-0.000856096	0.00100236	1	1	0.5
Vehicle	Day 85	Brain - Nerve, Ulnar, Distal, Left, Proximal	7.31318E-05	0.000103424	-0.000856096	0.00100236	1	1	0.5
Vehicle	Day 85	Brain - Nerve, Ulnar, Distal, Right, Distal	7.31318E-05	0.000103424	-0.000856096	0.00100236	1	1	0.5
Vehicle	Day 85	Brain - Nerve, Ulnar, Distal, Right, Proximal	7.31318E-05	0.000103424	-0.000856096	0.00100236	1	1	0.5
Vehicle	Day 85	Brain - Nerve, Ulnar, Proximal, Left, Distal	7.31318E-05	0.000103424	-0.000856096	0.00100236	1	1	0.5
Vehicle	Day 85	Brain - Nerve, Ulnar, Proximal, Left, Proximal	7.31318E-05	0.000103424	-0.000856096	0.00100236	1	1	0.5
Vehicle	Day 85	Brain - Nerve, Ulnar, Proximal, Right, Distal	7.31318E-05	0.000103424	-0.000856096	0.00100236	1	1	0.5
Vehicle	Day 85	Brain - Nerve, Ulnar, Proximal, Right, Proximal	7.31318E-05	0.000103424	-0.000856096	0.00100236	1	1	0.5
Vehicle	Day 85	Brain - Nerve, Optic	7.31318E-05	0.000103424	-0.000856096	0.00100236	1	1	0.5
Vehicle	Day 85	Brain - Ovary	0.000146264				0		
Vehicle	Day 85	Brain - Spinal Cord, Cervical	7.31318E-05	0.000103424	-0.000856096	0.00100236	1	1	0.5
Vehicle	Day 85	Brain - Spinal Cord, Lumbar	7.31318E-05	0.000103424	-0.000856096	0.00100236	1	1	0.5
Vehicle	Day 85	Brain - Spinal Cord, Sacral	7.31318E-05	0.000103424	-0.000856096	0.00100236	1	1	0.5
Vehicle	Day 85	Brain - Spinal Cord, Spinal Roots	7.31318E-05	0.000103424	-0.000856096	0.00100236	1	1	0.5
Vehicle	Day 85	Brain - Spinal Cord, Thoracic	7.31318E-05	0.000103424	-0.000856096	0.00100236	1	1	0.5
Vehicle	Day 85	Brain - Spleen	-5.30806E-05	7.50674E-05	-0.000727534	0.000621373	1	-1	0.5
Vehicle	Day 85	Brain - Testis	0				0		
Vehicle	Day 85	Ganglion, Dorsal Root, Cervical - Ganglion, Dorsal Root, Lumbar	-0.00010063	0.000142312	-0.001379254	0.001177994	1	-1	0.5
Vehicle	Day 85	Ganglion, Dorsal Root, Cervical - Ganglion, Dorsal Root, Thoracic	0	0	0	0	1		
Vehicle	Day 85	Ganglion, Dorsal Root, Cervical - Heart	0	0	0	0	1		
Vehicle	Day 85	Ganglion, Dorsal Root, Cervical - Kidney	0	0	0	0	1		
Vehicle	Day 85	Ganglion, Dorsal Root, Cervical - Lung	0	0	0	0	1		
Vehicle	Day 85	Ganglion, Dorsal Root, Cervical - Liver	-0.000233511	0.000330234	-0.003200548	0.002733526	1	-1	0.5
Vehicle	Day 85	Ganglion, Dorsal Root, Cervical - Muscle, Skeletal, Tibialis Anterior Left	-5.54296E-05	7.83893E-05	-0.000759729	0.00064887	1	-1	0.5
Vehicle	Day 85	Ganglion, Dorsal Root, Cervical - Muscle, Skeletal, Tibialis Anterior Right	0	0	0	0	1		
Vehicle	Day 85	Ganglion, Dorsal Root, Cervical - Muscle, Skeletal, Bicep Brachii Left	0	0	0	0	1		
Vehicle	Day 85	Ganglion, Dorsal Root, Cervical - Muscle, Skeletal, Bicep Brachii Right	0	0	0	0	1		
Vehicle	Day 85	Ganglion, Dorsal Root, Cervical - Nerve, Femoral, Distal, Left, Distal	0	0	0	0	1		
Vehicle	Day 85	Ganglion, Dorsal Root, Cervical - Nerve, Femoral, Distal, Left, Proximal	-4.46328E-05	6.31203E-05	-0.000611746	0.00052248	1	-1	0.5
Vehicle	Day 85	Ganglion, Dorsal Root, Cervical - Nerve, Femoral, Distal, Right, Distal	0	0	0	0	1		
Vehicle	Day 85	Ganglion, Dorsal Root, Cervical - Nerve, Femoral, Distal, Right, Proximal	0	0	0	0	1		
Vehicle	Day 85	Ganglion, Dorsal Root, Cervical - Nerve, Femoral, Proximal, Left, Distal	0	0	0	0	1		
Vehicle	Day 85	Ganglion, Dorsal Root, Cervical - Nerve, Femoral, Proximal, Left, Proximal	0	0	0	0	1		
Vehicle	Day 85	Ganglion, Dorsal Root, Cervical - Nerve, Femoral, Proximal, Right, Distal	-8.56071E-05	0.000121067	-0.001173348	0.001002134	1	-1	0.5
Vehicle	Day 85	Ganglion, Dorsal Root, Cervical - Nerve, Femoral, Proximal, Right, Proximal	0	0	0	0	1		
Vehicle	Day 85	Ganglion, Dorsal Root, Cervical - Nerve Median, Distal, Left, Distal	0	0	0	0	1		
Vehicle	Day 85	Ganglion, Dorsal Root, Cervical - Nerve Median, Distal, Left, Proximal	0	0	0	0	1		
Vehicle	Day 85	Ganglion, Dorsal Root, Cervical - Nerve Median, Distal, Right, Distal	0	0	0	0	1		
Vehicle	Day 85	Ganglion, Dorsal Root, Cervical - Nerve Median, Distal, Right, Proximal	0	0	0	0	1		
Vehicle	Day 85	Ganglion, Dorsal Root, Cervical - Nerve Median, Proximal, Left, Distal	0	0	0	0	1		
Vehicle	Day 85	Ganglion, Dorsal Root, Cervical - Nerve Median, Proximal, Left, Proximal	-4.58328E-05	6.48174E-05	-0.000628194	0.000536528	1	-1	0.5
Vehicle	Day 85	Ganglion, Dorsal Root, Cervical - Nerve Median, Proximal, Right, Distal	0	0	0	0	1		
Vehicle	Day 85	Ganglion, Dorsal Root, Cervical - Nerve Median, Proximal, Right, Proximal	0	0	0	0	1		
Vehicle	Day 85	Ganglion, Dorsal Root, Cervical - Nerve, Sciatic, Distal, Left, Distal	-0.000108197	0.000153014	-0.001482969	0.001266575	1	-1	0.5
Vehicle	Day 85	Ganglion, Dorsal Root, Cervical - Nerve, Sciatic, Distal, Left, Proximal	0	0	0	0	1		
Vehicle	Day 85	Ganglion, Dorsal Root, Cervical - Nerve, Sciatic, Distal, Right, Distal	0	0	0	0	1		
Vehicle	Day 85	Ganglion, Dorsal Root, Cervical - Nerve, Sciatic, Distal, Right, Proximal	0	0	0	0	1		
Vehicle	Day 85	Ganglion, Dorsal Root, Cervical - Nerve, Sciatic, Proximal, Left, Distal	0	0	0	0	1		
Vehicle	Day 85	Ganglion, Dorsal Root, Cervical - Nerve, Sciatic, Proximal, Left, Proximal	0	0	0	0	1		
Vehicle	Day 85	Ganglion, Dorsal Root, Cervical - Nerve, Sciatic, Proximal, Right, Distal	-0.000163993	0.000231921	-0.002247723	0.001919737	1	-1	0.5
Vehicle	Day 85	Ganglion, Dorsal Root, Cervical - Nerve, Sciatic, Proximal, Right, Proximal	-0.000138488	0.000195851	-0.00189814	0.001621165	1	-1	0.5
Vehicle	Day 85	Ganglion, Dorsal Root, Cervical - Nerve, Ulnar, Distal, Left, Distal	0	0	0	0	1		
Vehicle	Day 85	Ganglion, Dorsal Root, Cervical - Nerve, Ulnar, Distal, Left, Proximal	0	0	0	0	1		
Vehicle	Day 85	Ganglion, Dorsal Root, Cervical - Nerve, Ulnar, Distal, Right, Distal	0	0	0	0	1		
Vehicle	Day 85	Ganglion, Dorsal Root, Cervical - Nerve, Ulnar, Distal, Right, Proximal	0	0	0	0	1		
Vehicle	Day 85	Ganglion, Dorsal Root, Cervical - Nerve, Ulnar, Proximal, Left, Distal	0	0	0	0	1		
Vehicle	Day 85	Ganglion, Dorsal Root, Cervical - Nerve, Ulnar, Proximal, Left, Proximal	0	0	0	0	1		
Vehicle	Day 85	Ganglion, Dorsal Root, Cervical - Nerve, Ulnar, Proximal, Right, Distal	0	0	0	0	1		
Vehicle	Day 85	Ganglion, Dorsal Root, Cervical - Nerve, Ulnar, Proximal, Right, Proximal	0	0	0	0	1		
Vehicle	Day 85	Ganglion, Dorsal Root, Cervical - Nerve, Optic	0	0	0	0	1		
Vehicle	Day 85	Ganglion, Dorsal Root, Cervical - Ovary	0				0		
Vehicle	Day 85	Ganglion, Dorsal Root, Cervical - Spinal Cord, Cervical	0	0	0	0	1		
Vehicle	Day 85	Ganglion, Dorsal Root, Cervical - Spinal Cord, Lumbar	0	0	0	0	1		
Vehicle	Day 85	Ganglion, Dorsal Root, Cervical - Spinal Cord, Sacral	0	0	0	0	1		
Vehicle	Day 85	Ganglion, Dorsal Root, Cervical - Spinal Cord, Spinal Roots	0	0	0	0	1		
Vehicle	Day 85	Ganglion, Dorsal Root, Cervical - Spinal Cord, Thoracic	0	0	0	0	1		
Vehicle	Day 85	Ganglion, Dorsal Root, Cervical - Spleen	-0.000126212	0.000178491	-0.001729894	0.001477469	1	-1	0.5
Vehicle	Day 85	Ganglion, Dorsal Root, Cervical - Testis	0				0		
Vehicle	Day 85	Ganglion, Dorsal Root, Lumbar - Ganglion, Dorsal Root, Thoracic	0.00010063	0.000142312	-0.001177994	0.001379254	1	1	0.5
Vehicle	Day 85	Ganglion, Dorsal Root, Lumbar - Heart	0.00010063	0.000142312	-0.001177994	0.001379254	1	1	0.5
Vehicle	Day 85	Ganglion, Dorsal Root, Lumbar - Kidney	0.00010063	0.000142312	-0.001177994	0.001379254	1	1	0.5
Vehicle	Day 85	Ganglion, Dorsal Root, Lumbar - Lung	0.00010063	0.000142312	-0.001177994	0.001379254	1	1	0.5
Vehicle	Day 85	Ganglion, Dorsal Root, Lumbar - Liver	-0.000132881	0.000187922	-0.001821294	0.001555532	1	-1	0.5
Vehicle	Day 85	Ganglion, Dorsal Root, Lumbar - Muscle, Skeletal, Tibialis Anterior Left	4.52003E-05	6.39229E-05	-0.000529124	0.000619525	1	1	0.5
Vehicle	Day 85	Ganglion, Dorsal Root, Lumbar - Muscle, Skeletal, Tibialis Anterior Right	0.00010063	0.000142312	-0.001177994	0.001379254	1	1	0.5

Results of Evaluating Mean Differences Between Different Tissues Across Timepoint and Group for Log10 AAV2-ITR Target Copies/ug of DNA for Normalized Data

Group	Timepoint	Difference	Mean	StdDev	LowerCLMean	UpperCLMean	DF	tValue	P-Value
Vehicle	Day 85	Ganglion, Dorsal Root, Lumbar - Muscle, Skeletal, Bicep Brachii Left	0.00010063	0.000142312	-0.001177994	0.001379254	1	1	0.5
Vehicle	Day 85	Ganglion, Dorsal Root, Lumbar - Muscle, Skeletal, Bicep Brachii Right	0.00010063	0.000142312	-0.001177994	0.001379254	1	1	0.5
Vehicle	Day 85	Ganglion, Dorsal Root, Lumbar - Nerve, Femoral, Distal, Left, Distal	0.00010063	0.000142312	-0.001177994	0.001379254	1	1	0.5
Vehicle	Day 85	Ganglion, Dorsal Root, Lumbar - Nerve, Femoral, Distal, Left, Proximal	5.59971E-05	0.000205432	-0.00178974	0.001901734	1	0.385488835	0.76576562
Vehicle	Day 85	Ganglion, Dorsal Root, Lumbar - Nerve, Femoral, Distal, Right, Distal	0.00010063	0.000142312	-0.001177994	0.001379254	1	1	0.5
Vehicle	Day 85	Ganglion, Dorsal Root, Lumbar - Nerve, Femoral, Distal, Right, Proximal	0.00010063	0.000142312	-0.001177994	0.001379254	1	1	0.5
Vehicle	Day 85	Ganglion, Dorsal Root, Lumbar - Nerve, Femoral, Proximal, Left, Distal	0.00010063	0.000142312	-0.001177994	0.001379254	1	1	0.5
Vehicle	Day 85	Ganglion, Dorsal Root, Lumbar - Nerve, Femoral, Proximal, Left, Proximal	0.00010063	0.000142312	-0.001177994	0.001379254	1	1	0.5
Vehicle	Day 85	Ganglion, Dorsal Root, Lumbar - Nerve, Femoral, Proximal, Right, Distal	1.50228E-05	0.000263379	-0.002351342	0.002381388	1	0.080665024	0.948758
Vehicle	Day 85	Ganglion, Dorsal Root, Lumbar - Nerve, Femoral, Proximal, Right, Proximal	0.00010063	0.000142312	-0.001177994	0.001379254	1	1	0.5
Vehicle	Day 85	Ganglion, Dorsal Root, Lumbar - Nerve Median, Distal, Left, Distal	0.00010063	0.000142312	-0.001177994	0.001379254	1	1	0.5
Vehicle	Day 85	Ganglion, Dorsal Root, Lumbar - Nerve Median, Distal, Left, Proximal	0.00010063	0.000142312	-0.001177994	0.001379254	1	1	0.5
Vehicle	Day 85	Ganglion, Dorsal Root, Lumbar - Nerve Median, Distal, Right, Distal	0.00010063	0.000142312	-0.001177994	0.001379254	1	1	0.5
Vehicle	Day 85	Ganglion, Dorsal Root, Lumbar - Nerve Median, Distal, Right, Proximal	0.00010063	0.000142312	-0.001177994	0.001379254	1	1	0.5
Vehicle	Day 85	Ganglion, Dorsal Root, Lumbar - Nerve Median, Proximal, Left, Distal	0.00010063	0.000142312	-0.001177994	0.001379254	1	1	0.5
Vehicle	Day 85	Ganglion, Dorsal Root, Lumbar - Nerve Median, Proximal, Left, Proximal	5.47971E-05	0.00020713	-0.001806188	0.001915782	1	0.374136748	0.772081443
Vehicle	Day 85	Ganglion, Dorsal Root, Lumbar - Nerve Median, Proximal, Right, Distal	0.00010063	0.000142312	-0.001177994	0.001379254	1	1	0.5
Vehicle	Day 85	Ganglion, Dorsal Root, Lumbar - Nerve Median, Proximal, Right, Proximal	0.00010063	0.000142312	-0.001177994	0.001379254	1	1	0.5
Vehicle	Day 85	Ganglion, Dorsal Root, Lumbar - Nerve, Sciatic, Distal, Left, Distal	-7.56699E-06	1.07013E-05	-0.000103715	8.85807E-05	1	-1	0.5
Vehicle	Day 85	Ganglion, Dorsal Root, Lumbar - Nerve, Sciatic, Distal, Left, Proximal	0.00010063	0.000142312	-0.001177994	0.001379254	1	1	0.5
Vehicle	Day 85	Ganglion, Dorsal Root, Lumbar - Nerve, Sciatic, Distal, Right, Distal	0.00010063	0.000142312	-0.001177994	0.001379254	1	1	0.5
Vehicle	Day 85	Ganglion, Dorsal Root, Lumbar - Nerve, Sciatic, Distal, Right, Proximal	0.00010063	0.000142312	-0.001177994	0.001379254	1	1	0.5
Vehicle	Day 85	Ganglion, Dorsal Root, Lumbar - Nerve, Sciatic, Proximal, Left, Distal	0.00010063	0.000142312	-0.001177994	0.001379254	1	1	0.5
Vehicle	Day 85	Ganglion, Dorsal Root, Lumbar - Nerve, Sciatic, Proximal, Left, Proximal	0.00010063	0.000142312	-0.001177994	0.001379254	1	1	0.5
Vehicle	Day 85	Ganglion, Dorsal Root, Lumbar - Nerve, Sciatic, Proximal, Right, Distal	-6.33632E-05	8.96091E-05	-0.000868469	0.000741743	1	-1	0.5
Vehicle	Day 85	Ganglion, Dorsal Root, Lumbar - Nerve, Sciatic, Proximal, Right, Proximal	-3.78577E-05	0.000338163	-0.003076134	0.003000419	1	-0.158322737	0.900038321
Vehicle	Day 85	Ganglion, Dorsal Root, Lumbar - Nerve, Ulnar, Distal, Left, Distal	0.00010063	0.000142312	-0.001177994	0.001379254	1	1	0.5
Vehicle	Day 85	Ganglion, Dorsal Root, Lumbar - Nerve, Ulnar, Distal, Left, Proximal	0.00010063	0.000142312	-0.001177994	0.001379254	1	1	0.5
Vehicle	Day 85	Ganglion, Dorsal Root, Lumbar - Nerve, Ulnar, Distal, Right, Distal	0.00010063	0.000142312	-0.001177994	0.001379254	1	1	0.5
Vehicle	Day 85	Ganglion, Dorsal Root, Lumbar - Nerve, Ulnar, Distal, Right, Proximal	0.00010063	0.000142312	-0.001177994	0.001379254	1	1	0.5
Vehicle	Day 85	Ganglion, Dorsal Root, Lumbar - Nerve, Ulnar, Proximal, Left, Distal	0.00010063	0.000142312	-0.001177994	0.001379254	1	1	0.5
Vehicle	Day 85	Ganglion, Dorsal Root, Lumbar - Nerve, Ulnar, Proximal, Left, Proximal	0.00010063	0.000142312	-0.001177994	0.001379254	1	1	0.5
Vehicle	Day 85	Ganglion, Dorsal Root, Lumbar - Nerve, Ulnar, Proximal, Right, Distal	0.00010063	0.000142312	-0.001177994	0.001379254	1	1	0.5
Vehicle	Day 85	Ganglion, Dorsal Root, Lumbar - Nerve, Ulnar, Proximal, Right, Proximal	0.00010063	0.000142312	-0.001177994	0.001379254	1	1	0.5
Vehicle	Day 85	Ganglion, Dorsal Root, Lumbar - Nerve, Optic	0.00010063	0.000142312	-0.001177994	0.001379254	1	1	0.5
Vehicle	Day 85	Ganglion, Dorsal Root, Lumbar - Ovary	0.00020126				0		
Vehicle	Day 85	Ganglion, Dorsal Root, Lumbar - Spinal Cord, Cervical	0.00010063	0.000142312	-0.001177994	0.001379254	1	1	0.5
Vehicle	Day 85	Ganglion, Dorsal Root, Lumbar - Spinal Cord, Lumbar	0.00010063	0.000142312	-0.001177994	0.001379254	1	1	0.5
Vehicle	Day 85	Ganglion, Dorsal Root, Lumbar - Spinal Cord, Sacral	0.00010063	0.000142312	-0.001177994	0.001379254	1	1	0.5
Vehicle	Day 85	Ganglion, Dorsal Root, Lumbar - Spinal Cord, Spinal Roots	0.00010063	0.000142312	-0.001177994	0.001379254	1	1	0.5
Vehicle	Day 85	Ganglion, Dorsal Root, Lumbar - Spinal Cord, Thoracic	0.00010063	0.000142312	-0.001177994	0.001379254	1	1	0.5
Vehicle	Day 85	Ganglion, Dorsal Root, Lumbar - Spleen	-2.55826E-05	3.61792E-05	-0.00035064	0.000299475	1	-1	0.5
Vehicle	Day 85	Ganglion, Dorsal Root, Lumbar - Testis	0				0		
Vehicle	Day 85	Ganglion, Dorsal Root, Thoracic - Heart	0	0	0	0	1		
Vehicle	Day 85	Ganglion, Dorsal Root, Thoracic - Kidney	0	0	0	0	1		
Vehicle	Day 85	Ganglion, Dorsal Root, Thoracic - Lung	0	0	0	0	1		
Vehicle	Day 85	Ganglion, Dorsal Root, Thoracic - Liver	-0.000233511	0.000330234	-0.003200548	0.002733526	1	-1	0.5
Vehicle	Day 85	Ganglion, Dorsal Root, Thoracic - Muscle, Skeletal, Tibialis Anterior Left	-5.54296E-05	7.83893E-05	-0.000759729	0.00064887	1	-1	0.5
Vehicle	Day 85	Ganglion, Dorsal Root, Thoracic - Muscle, Skeletal, Tibialis Anterior Right	0	0	0	0	1		
Vehicle	Day 85	Ganglion, Dorsal Root, Thoracic - Muscle, Skeletal, Bicep Brachii Left	0	0	0	0	1		
Vehicle	Day 85	Ganglion, Dorsal Root, Thoracic - Muscle, Skeletal, Bicep Brachii Right	0	0	0	0	1		
Vehicle	Day 85	Ganglion, Dorsal Root, Thoracic - Nerve, Femoral, Distal, Left, Distal	0	0	0	0	1		
Vehicle	Day 85	Ganglion, Dorsal Root, Thoracic - Nerve, Femoral, Distal, Left, Proximal	-4.46328E-05	6.31203E-05	-0.000611746	0.00052248	1	-1	0.5
Vehicle	Day 85	Ganglion, Dorsal Root, Thoracic - Nerve, Femoral, Distal, Right, Distal	0	0	0	0	1		
Vehicle	Day 85	Ganglion, Dorsal Root, Thoracic - Nerve, Femoral, Distal, Right, Proximal	0	0	0	0	1		
Vehicle	Day 85	Ganglion, Dorsal Root, Thoracic - Nerve, Femoral, Proximal, Left, Distal	0	0	0	0	1		
Vehicle	Day 85	Ganglion, Dorsal Root, Thoracic - Nerve, Femoral, Proximal, Left, Proximal	0	0	0	0	1		
Vehicle	Day 85	Ganglion, Dorsal Root, Thoracic - Nerve, Femoral, Proximal, Right, Distal	-8.56071E-05	0.000121067	-0.001173348	0.001002134	1	-1	0.5
Vehicle	Day 85	Ganglion, Dorsal Root, Thoracic - Nerve, Femoral, Proximal, Right, Proximal	0	0	0	0	1		
Vehicle	Day 85	Ganglion, Dorsal Root, Thoracic - Nerve Median, Distal, Left, Distal	0	0	0	0	1		
Vehicle	Day 85	Ganglion, Dorsal Root, Thoracic - Nerve Median, Distal, Left, Proximal	0	0	0	0	1		
Vehicle	Day 85	Ganglion, Dorsal Root, Thoracic - Nerve Median, Distal, Right, Distal	0	0	0	0	1		
Vehicle	Day 85	Ganglion, Dorsal Root, Thoracic - Nerve Median, Distal, Right, Proximal	0	0	0	0	1		
Vehicle	Day 85	Ganglion, Dorsal Root, Thoracic - Nerve Median, Proximal, Left, Distal	0	0	0	0	1		
Vehicle	Day 85	Ganglion, Dorsal Root, Thoracic - Nerve Median, Proximal, Left, Proximal	-4.58328E-05	6.48174E-05	-0.000628194	0.000536528	1	-1	0.5
Vehicle	Day 85	Ganglion, Dorsal Root, Thoracic - Nerve Median, Proximal, Right, Distal	0	0	0	0	1		
Vehicle	Day 85	Ganglion, Dorsal Root, Thoracic - Nerve Median, Proximal, Right, Proximal	0	0	0	0	1		
Vehicle	Day 85	Ganglion, Dorsal Root, Thoracic - Nerve, Sciatic, Distal, Left, Distal	-0.000108197	0.000153014	-0.001482969	0.001266575	1	-1	0.5
Vehicle	Day 85	Ganglion, Dorsal Root, Thoracic - Nerve, Sciatic, Distal, Left, Proximal	0	0	0	0	1		
Vehicle	Day 85	Ganglion, Dorsal Root, Thoracic - Nerve, Sciatic, Distal, Right, Distal	0	0	0	0	1		
Vehicle	Day 85	Ganglion, Dorsal Root, Thoracic - Nerve, Sciatic, Distal, Right, Proximal	0	0	0	0	1		
Vehicle	Day 85	Ganglion, Dorsal Root, Thoracic - Nerve, Sciatic, Proximal, Left, Distal	0	0	0	0	1		
Vehicle	Day 85	Ganglion, Dorsal Root, Thoracic - Nerve, Sciatic, Proximal, Left, Proximal	0	0	0	0	1		
Vehicle	Day 85	Ganglion, Dorsal Root, Thoracic - Nerve, Sciatic, Proximal, Right, Distal	-0.000163993	0.000231921	-0.002247723	0.001919737	1	-1	0.5
Vehicle	Day 85	Ganglion, Dorsal Root, Thoracic - Nerve, Sciatic, Proximal, Right, Proximal	-0.000138488	0.000195851	-0.00189814	0.001621165	1	-1	0.5
Vehicle	Day 85	Ganglion, Dorsal Root, Thoracic - Nerve, Ulnar, Distal, Left, Distal	0	0	0	0	1		
Vehicle	Day 85	Ganglion, Dorsal Root, Thoracic - Nerve, Ulnar, Distal, Left, Proximal	0	0	0	0	1		
Vehicle	Day 85	Ganglion, Dorsal Root, Thoracic - Nerve, Ulnar, Distal, Right, Distal	0	0	0	0	1		
Vehicle	Day 85	Ganglion, Dorsal Root, Thoracic - Nerve, Ulnar, Distal, Right, Proximal	0	0	0	0	1		
Vehicle	Day 85	Ganglion, Dorsal Root, Thoracic - Nerve, Ulnar, Proximal, Left, Distal	0	0	0	0	1		
Vehicle	Day 85	Ganglion, Dorsal Root, Thoracic - Nerve, Ulnar, Proximal, Left, Proximal	0	0	0	0	1		
Vehicle	Day 85	Ganglion, Dorsal Root, Thoracic - Nerve, Ulnar, Proximal, Right, Distal	0	0	0	0	1		
Vehicle	Day 85	Ganglion, Dorsal Root, Thoracic - Nerve, Ulnar, Proximal, Right, Proximal	0	0	0	0	1		
Vehicle	Day 85	Ganglion, Dorsal Root, Thoracic - Nerve, Optic	0	0	0	0	1		
Vehicle	Day 85	Ganglion, Dorsal Root, Thoracic - Ovary	0				0		
Vehicle	Day 85	Ganglion, Dorsal Root, Thoracic - Spinal Cord, Cervical	0	0	0	0	1		
Vehicle	Day 85	Ganglion, Dorsal Root, Thoracic - Spinal Cord, Lumbar	0	0	0	0	1		
Vehicle	Day 85	Ganglion, Dorsal Root, Thoracic - Spinal Cord, Sacral	0	0	0	0	1		
Vehicle	Day 85	Ganglion, Dorsal Root, Thoracic - Spinal Cord, Spinal Roots	0	0	0	0	1		
Vehicle	Day 85	Ganglion, Dorsal Root, Thoracic - Spinal Cord, Thoracic	0	0	0	0	1		
Vehicle	Day 85	Ganglion, Dorsal Root, Thoracic - Spleen	-0.000126212	0.000178491	-0.001729894	0.001477469	1	-1	0.5
Vehicle	Day 85	Ganglion, Dorsal Root, Thoracic - Testis	0				0		
Vehicle	Day 85	Heart - Kidney	0	0	0	0	1		

Results of Evaluating Mean Differences Between Different Tissues Across Timepoint and Group for Log10 AAV2-ITR Target Copies/ug of DNA for Normalized Data

Group	Timepoint	Difference	Mean	StdDev	LowerCLMean	UpperCLMean	DF	tValue	P-Value
Vehicle	Day 85	Heart - Lung	0	0	0	0	1		
Vehicle	Day 85	Heart - Liver	-0.000233511	0.000330234	-0.003200548	0.002733526	1	-1	0.5
Vehicle	Day 85	Heart - Muscle, Skeletal, Tibialis Anterior Left	-5.54296E-05	7.83893E-05	-0.000759729	0.00064887	1	-1	0.5
Vehicle	Day 85	Heart - Muscle, Skeletal, Tibialis Anterior Right	0	0	0	0	1		
Vehicle	Day 85	Heart - Muscle, Skeletal, Bicep Brachii Left	0	0	0	0	1		
Vehicle	Day 85	Heart - Muscle, Skeletal, Bicep Brachii Right	0	0	0	0	1		
Vehicle	Day 85	Heart - Nerve, Femoral, Distal, Left, Distal	0	0	0	0	1		
Vehicle	Day 85	Heart - Nerve, Femoral, Distal, Left, Proximal	-4.46328E-05	6.31203E-05	-0.000611746	0.00052248	1	-1	0.5
Vehicle	Day 85	Heart - Nerve, Femoral, Distal, Right, Distal	0	0	0	0	1		
Vehicle	Day 85	Heart - Nerve, Femoral, Distal, Right, Proximal	0	0	0	0	1		
Vehicle	Day 85	Heart - Nerve, Femoral, Proximal, Left, Distal	0	0	0	0	1		
Vehicle	Day 85	Heart - Nerve, Femoral, Proximal, Left, Proximal	0	0	0	0	1		
Vehicle	Day 85	Heart - Nerve, Femoral, Proximal, Right, Distal	-8.56071E-05	0.000121067	-0.001173348	0.001002134	1	-1	0.5
Vehicle	Day 85	Heart - Nerve, Femoral, Proximal, Right, Proximal	0	0	0	0	1		
Vehicle	Day 85	Heart - Nerve Median, Distal, Left, Distal	0	0	0	0	1		
Vehicle	Day 85	Heart - Nerve Median, Distal, Left, Proximal	0	0	0	0	1		
Vehicle	Day 85	Heart - Nerve Median, Distal, Right, Distal	0	0	0	0	1		
Vehicle	Day 85	Heart - Nerve Median, Distal, Right, Proximal	0	0	0	0	1		
Vehicle	Day 85	Heart - Nerve Median, Proximal, Left, Distal	0	0	0	0	1		
Vehicle	Day 85	Heart - Nerve Median, Proximal, Left, Proximal	-4.58328E-05	6.48174E-05	-0.000628194	0.000536528	1	-1	0.5
Vehicle	Day 85	Heart - Nerve Median, Proximal, Right, Distal	0	0	0	0	1		
Vehicle	Day 85	Heart - Nerve Median, Proximal, Right, Proximal	0	0	0	0	1		
Vehicle	Day 85	Heart - Nerve, Sciatic, Distal, Left, Distal	-0.000108197	0.000153014	-0.001482969	0.001266575	1	-1	0.5
Vehicle	Day 85	Heart - Nerve, Sciatic, Distal, Left, Proximal	0	0	0	0	1		
Vehicle	Day 85	Heart - Nerve, Sciatic, Distal, Right, Distal	0	0	0	0	1		
Vehicle	Day 85	Heart - Nerve, Sciatic, Distal, Right, Proximal	0	0	0	0	1		
Vehicle	Day 85	Heart - Nerve, Sciatic, Proximal, Left, Distal	0	0	0	0	1		
Vehicle	Day 85	Heart - Nerve, Sciatic, Proximal, Left, Proximal	0	0	0	0	1		
Vehicle	Day 85	Heart - Nerve, Sciatic, Proximal, Right, Distal	-0.000163993	0.000231921	-0.002247723	0.001919737	1	-1	0.5
Vehicle	Day 85	Heart - Nerve, Sciatic, Proximal, Right, Proximal	-0.000138488	0.000195851	-0.00189814	0.001621165	1	-1	0.5
Vehicle	Day 85	Heart - Nerve, Ulnar, Distal, Left, Distal	0	0	0	0	1		
Vehicle	Day 85	Heart - Nerve, Ulnar, Distal, Left, Proximal	0	0	0	0	1		
Vehicle	Day 85	Heart - Nerve, Ulnar, Distal, Right, Distal	0	0	0	0	1		
Vehicle	Day 85	Heart - Nerve, Ulnar, Distal, Right, Proximal	0	0	0	0	1		
Vehicle	Day 85	Heart - Nerve, Ulnar, Proximal, Left, Distal	0	0	0	0	1		
Vehicle	Day 85	Heart - Nerve, Ulnar, Proximal, Left, Proximal	0	0	0	0	1		
Vehicle	Day 85	Heart - Nerve, Ulnar, Proximal, Right, Distal	0	0	0	0	1		
Vehicle	Day 85	Heart - Nerve, Ulnar, Proximal, Right, Proximal	0	0	0	0	1		
Vehicle	Day 85	Heart - Nerve, Optic	0	0	0	0	1		
Vehicle	Day 85	Heart - Ovary	0				0		
Vehicle	Day 85	Heart - Spinal Cord, Cervical	0	0	0	0	1		
Vehicle	Day 85	Heart - Spinal Cord, Lumbar	0	0	0	0	1		
Vehicle	Day 85	Heart - Spinal Cord, Sacral	0	0	0	0	1		
Vehicle	Day 85	Heart - Spinal Cord, Spinal Roots	0	0	0	0	1		
Vehicle	Day 85	Heart - Spinal Cord, Thoracic	0	0	0	0	1		
Vehicle	Day 85	Heart - Spleen	-0.000126212	0.000178491	-0.001729894	0.001477469	1	-1	0.5
Vehicle	Day 85	Heart - Testis	0				0		
Vehicle	Day 85	Kidney - Lung	0	0	0	0	1		
Vehicle	Day 85	Kidney - Liver	-0.000233511	0.000330234	-0.003200548	0.002733526	1	-1	0.5
Vehicle	Day 85	Kidney - Muscle, Skeletal, Tibialis Anterior Left	-5.54296E-05	7.83893E-05	-0.000759729	0.00064887	1	-1	0.5
Vehicle	Day 85	Kidney - Muscle, Skeletal, Tibialis Anterior Right	0	0	0	0	1		
Vehicle	Day 85	Kidney - Muscle, Skeletal, Bicep Brachii Left	0	0	0	0	1		
Vehicle	Day 85	Kidney - Muscle, Skeletal, Bicep Brachii Right	0	0	0	0	1		
Vehicle	Day 85	Kidney - Nerve, Femoral, Distal, Left, Distal	0	0	0	0	1		
Vehicle	Day 85	Kidney - Nerve, Femoral, Distal, Left, Proximal	-4.46328E-05	6.31203E-05	-0.000611746	0.00052248	1	-1	0.5
Vehicle	Day 85	Kidney - Nerve, Femoral, Distal, Right, Distal	0	0	0	0	1		
Vehicle	Day 85	Kidney - Nerve, Femoral, Distal, Right, Proximal	0	0	0	0	1		
Vehicle	Day 85	Kidney - Nerve, Femoral, Proximal, Left, Distal	0	0	0	0	1		
Vehicle	Day 85	Kidney - Nerve, Femoral, Proximal, Left, Proximal	0	0	0	0	1		
Vehicle	Day 85	Kidney - Nerve, Femoral, Proximal, Right, Distal	-8.56071E-05	0.000121067	-0.001173348	0.001002134	1	-1	0.5
Vehicle	Day 85	Kidney - Nerve, Femoral, Proximal, Right, Proximal	0	0	0	0	1		
Vehicle	Day 85	Kidney - Nerve Median, Distal, Left, Distal	0	0	0	0	1		
Vehicle	Day 85	Kidney - Nerve Median, Distal, Left, Proximal	0	0	0	0	1		
Vehicle	Day 85	Kidney - Nerve Median, Distal, Right, Distal	0	0	0	0	1		
Vehicle	Day 85	Kidney - Nerve Median, Distal, Right, Proximal	0	0	0	0	1		
Vehicle	Day 85	Kidney - Nerve Median, Proximal, Left, Distal	0	0	0	0	1		
Vehicle	Day 85	Kidney - Nerve Median, Proximal, Left, Proximal	-4.58328E-05	6.48174E-05	-0.000628194	0.000536528	1	-1	0.5
Vehicle	Day 85	Kidney - Nerve Median, Proximal, Right, Distal	0	0	0	0	1		
Vehicle	Day 85	Kidney - Nerve Median, Proximal, Right, Proximal	0	0	0	0	1		
Vehicle	Day 85	Kidney - Nerve, Sciatic, Distal, Left, Distal	-0.000108197	0.000153014	-0.001482969	0.001266575	1	-1	0.5
Vehicle	Day 85	Kidney - Nerve, Sciatic, Distal, Left, Proximal	0	0	0	0	1		
Vehicle	Day 85	Kidney - Nerve, Sciatic, Distal, Right, Distal	0	0	0	0	1		
Vehicle	Day 85	Kidney - Nerve, Sciatic, Distal, Right, Proximal	0	0	0	0	1		
Vehicle	Day 85	Kidney - Nerve, Sciatic, Proximal, Left, Distal	0	0	0	0	1		
Vehicle	Day 85	Kidney - Nerve, Sciatic, Proximal, Left, Proximal	0	0	0	0	1		
Vehicle	Day 85	Kidney - Nerve, Sciatic, Proximal, Right, Distal	-0.000163993	0.000231921	-0.002247723	0.001919737	1	-1	0.5
Vehicle	Day 85	Kidney - Nerve, Sciatic, Proximal, Right, Proximal	-0.000138488	0.000195851	-0.00189814	0.001621165	1	-1	0.5
Vehicle	Day 85	Kidney - Nerve, Ulnar, Distal, Left, Distal	0	0	0	0	1		
Vehicle	Day 85	Kidney - Nerve, Ulnar, Distal, Left, Proximal	0	0	0	0	1		
Vehicle	Day 85	Kidney - Nerve, Ulnar, Distal, Right, Distal	0	0	0	0	1		
Vehicle	Day 85	Kidney - Nerve, Ulnar, Distal, Right, Proximal	0	0	0	0	1		
Vehicle	Day 85	Kidney - Nerve, Ulnar, Proximal, Left, Distal	0	0	0	0	1		
Vehicle	Day 85	Kidney - Nerve, Ulnar, Proximal, Left, Proximal	0	0	0	0	1		
Vehicle	Day 85	Kidney - Nerve, Ulnar, Proximal, Right, Distal	0	0	0	0	1		
Vehicle	Day 85	Kidney - Nerve, Ulnar, Proximal, Right, Proximal	0	0	0	0	1		
Vehicle	Day 85	Kidney - Nerve, Optic	0	0	0	0	1		
Vehicle	Day 85	Kidney - Ovary	0				0		
Vehicle	Day 85	Kidney - Spinal Cord, Cervical	0	0	0	0	1		
Vehicle	Day 85	Kidney - Spinal Cord, Lumbar	0	0	0	0	1		
Vehicle	Day 85	Kidney - Spinal Cord, Sacral	0	0	0	0	1		
Vehicle	Day 85	Kidney - Spinal Cord, Spinal Roots	0	0	0	0	1		
Vehicle	Day 85	Kidney - Spinal Cord, Thoracic	0	0	0	0	1		
Vehicle	Day 85	Kidney - Spleen	-0.000126212	0.000178491	-0.001729894	0.001477469	1	-1	0.5

Results of Evaluating Mean Differences Between Different Tissues Across Timepoint and Group for Log10 AAV2-ITR Target Copies/ug of DNA for Normalized Data

Group	Timepoint	Difference	Mean	StdDev	LowerCLMean	UpperCLMean	DF	tValue	P-Value
Vehicle	Day 85	Kidney - Testis	0				0		
Vehicle	Day 85	Lung - Liver	-0.000233511	0.000330234	-0.003200548	0.002733526	1	-1	0.5
Vehicle	Day 85	Lung - Muscle, Skeletal, Tibialis Anterior Left	-5.54296E-05	7.83893E-05	-0.000759729	0.00064887	1	-1	0.5
Vehicle	Day 85	Lung - Muscle, Skeletal, Tibialis Anterior Right	0	0	0	0	1		
Vehicle	Day 85	Lung - Muscle, Skeletal, Bicep Brachii Left	0	0	0	0	1		
Vehicle	Day 85	Lung - Muscle, Skeletal, Bicep Brachii Right	0	0	0	0	1		
Vehicle	Day 85	Lung - Nerve, Femoral, Distal, Left, Distal	0	0	0	0	1		
Vehicle	Day 85	Lung - Nerve, Femoral, Distal, Left, Proximal	-4.46328E-05	6.31203E-05	-0.000611746	0.00052248	1	-1	0.5
Vehicle	Day 85	Lung - Nerve, Femoral, Distal, Right, Distal	0	0	0	0	1		
Vehicle	Day 85	Lung - Nerve, Femoral, Distal, Right, Proximal	0	0	0	0	1		
Vehicle	Day 85	Lung - Nerve, Femoral, Proximal, Left, Distal	0	0	0	0	1		
Vehicle	Day 85	Lung - Nerve, Femoral, Proximal, Left, Proximal	0	0	0	0	1		
Vehicle	Day 85	Lung - Nerve, Femoral, Proximal, Right, Distal	-8.56071E-05	0.000121067	-0.001173348	0.001002134	1	-1	0.5
Vehicle	Day 85	Lung - Nerve, Femoral, Proximal, Right, Proximal	0	0	0	0	1		
Vehicle	Day 85	Lung - Nerve Median, Distal, Left, Distal	0	0	0	0	1		
Vehicle	Day 85	Lung - Nerve Median, Distal, Left, Proximal	0	0	0	0	1		
Vehicle	Day 85	Lung - Nerve Median, Distal, Right, Distal	0	0	0	0	1		
Vehicle	Day 85	Lung - Nerve Median, Distal, Right, Proximal	0	0	0	0	1		
Vehicle	Day 85	Lung - Nerve Median, Proximal, Left, Distal	0	0	0	0	1		
Vehicle	Day 85	Lung - Nerve Median, Proximal, Left, Proximal	-4.58328E-05	6.48174E-05	-0.000628194	0.000536528	1	-1	0.5
Vehicle	Day 85	Lung - Nerve Median, Proximal, Right, Distal	0	0	0	0	1		
Vehicle	Day 85	Lung - Nerve Median, Proximal, Right, Proximal	0	0	0	0	1		
Vehicle	Day 85	Lung - Nerve, Sciatic, Distal, Left, Distal	-0.000108197	0.000153014	-0.001482969	0.001266575	1	-1	0.5
Vehicle	Day 85	Lung - Nerve, Sciatic, Distal, Left, Proximal	0	0	0	0	1		
Vehicle	Day 85	Lung - Nerve, Sciatic, Distal, Right, Distal	0	0	0	0	1		
Vehicle	Day 85	Lung - Nerve, Sciatic, Distal, Right, Proximal	0	0	0	0	1		
Vehicle	Day 85	Lung - Nerve, Sciatic, Proximal, Left, Distal	0	0	0	0	1		
Vehicle	Day 85	Lung - Nerve, Sciatic, Proximal, Left, Proximal	0	0	0	0	1		
Vehicle	Day 85	Lung - Nerve, Sciatic, Proximal, Right, Distal	-0.000163993	0.000231921	-0.002247723	0.001919737	1	-1	0.5
Vehicle	Day 85	Lung - Nerve, Sciatic, Proximal, Right, Proximal	-0.000138488	0.000195851	-0.00189814	0.001621165	1	-1	0.5
Vehicle	Day 85	Lung - Nerve, Ulnar, Distal, Left, Distal	0	0	0	0	1		
Vehicle	Day 85	Lung - Nerve, Ulnar, Distal, Left, Proximal	0	0	0	0	1		
Vehicle	Day 85	Lung - Nerve, Ulnar, Distal, Right, Distal	0	0	0	0	1		
Vehicle	Day 85	Lung - Nerve, Ulnar, Distal, Right, Proximal	0	0	0	0	1		
Vehicle	Day 85	Lung - Nerve, Ulnar, Proximal, Left, Distal	0	0	0	0	1		
Vehicle	Day 85	Lung - Nerve, Ulnar, Proximal, Left, Proximal	0	0	0	0	1		
Vehicle	Day 85	Lung - Nerve, Ulnar, Proximal, Right, Distal	0	0	0	0	1		
Vehicle	Day 85	Lung - Nerve, Ulnar, Proximal, Right, Proximal	0	0	0	0	1		
Vehicle	Day 85	Lung - Nerve, Optic	0	0	0	0	1		
Vehicle	Day 85	Lung - Ovary	0				0		
Vehicle	Day 85	Lung - Spinal Cord, Cervical	0	0	0	0	1		
Vehicle	Day 85	Lung - Spinal Cord, Lumbar	0	0	0	0	1		
Vehicle	Day 85	Lung - Spinal Cord, Sacral	0	0	0	0	1		
Vehicle	Day 85	Lung - Spinal Cord, Spinal Roots	0	0	0	0	1		
Vehicle	Day 85	Lung - Spinal Cord, Thoracic	0	0	0	0	1		
Vehicle	Day 85	Lung - Spleen	-0.000126212	0.000178491	-0.001729894	0.001477469	1	-1	0.5
Vehicle	Day 85	Lung - Testis	0				0		
Vehicle	Day 85	Liver - Muscle, Skeletal, Tibialis Anterior Left	0.000178081	0.000251845	-0.002084656	0.002440819	1	1	0.5
Vehicle	Day 85	Liver - Muscle, Skeletal, Tibialis Anterior Right	0.000233511	0.000330234	-0.002733526	0.003200548	1	1	0.5
Vehicle	Day 85	Liver - Muscle, Skeletal, Bicep Brachii Left	0.000233511	0.000330234	-0.002733526	0.003200548	1	1	0.5
Vehicle	Day 85	Liver - Muscle, Skeletal, Bicep Brachii Right	0.000233511	0.000330234	-0.002733526	0.003200548	1	1	0.5
Vehicle	Day 85	Liver - Nerve, Femoral, Distal, Left, Distal	0.000233511	0.000330234	-0.002733526	0.003200548	1	1	0.5
Vehicle	Day 85	Liver - Nerve, Femoral, Distal, Left, Proximal	0.000188878	0.000393355	-0.003345272	0.003723028	1	0.679066815	0.620231947
Vehicle	Day 85	Liver - Nerve, Femoral, Distal, Right, Distal	0.000233511	0.000330234	-0.002733526	0.003200548	1	1	0.5
Vehicle	Day 85	Liver - Nerve, Femoral, Distal, Right, Proximal	0.000233511	0.000330234	-0.002733526	0.003200548	1	1	0.5
Vehicle	Day 85	Liver - Nerve, Femoral, Proximal, Left, Distal	0.000233511	0.000330234	-0.002733526	0.003200548	1	1	0.5
Vehicle	Day 85	Liver - Nerve, Femoral, Proximal, Left, Proximal	0.000233511	0.000330234	-0.002733526	0.003200548	1	1	0.5
Vehicle	Day 85	Liver - Nerve, Femoral, Proximal, Right, Distal	0.000147904	0.000451301	-0.003906875	0.004202682	1	0.463476869	0.72370409
Vehicle	Day 85	Liver - Nerve, Femoral, Proximal, Right, Proximal	0.000233511	0.000330234	-0.002733526	0.003200548	1	1	0.5
Vehicle	Day 85	Liver - Nerve Median, Distal, Left, Distal	0.000233511	0.000330234	-0.002733526	0.003200548	1	1	0.5
Vehicle	Day 85	Liver - Nerve Median, Distal, Left, Proximal	0.000233511	0.000330234	-0.002733526	0.003200548	1	1	0.5
Vehicle	Day 85	Liver - Nerve Median, Distal, Right, Distal	0.000233511	0.000330234	-0.002733526	0.003200548	1	1	0.5
Vehicle	Day 85	Liver - Nerve Median, Distal, Right, Proximal	0.000233511	0.000330234	-0.002733526	0.003200548	1	1	0.5
Vehicle	Day 85	Liver - Nerve Median, Proximal, Left, Distal	0.000233511	0.000330234	-0.002733526	0.003200548	1	1	0.5
Vehicle	Day 85	Liver - Nerve Median, Proximal, Left, Proximal	0.000187678	0.000395052	-0.00336172	0.003737076	1	0.6718536	0.623385314
Vehicle	Day 85	Liver - Nerve Median, Proximal, Right, Distal	0.000233511	0.000330234	-0.002733526	0.003200548	1	1	0.5
Vehicle	Day 85	Liver - Nerve Median, Proximal, Right, Proximal	0.000233511	0.000330234	-0.002733526	0.003200548	1	1	0.5
Vehicle	Day 85	Liver - Nerve, Sciatic, Distal, Left, Distal	0.000125314	0.000177221	-0.001466951	0.001717579	1	1	0.5
Vehicle	Day 85	Liver - Nerve, Sciatic, Distal, Left, Proximal	0.000233511	0.000330234	-0.002733526	0.003200548	1	1	0.5
Vehicle	Day 85	Liver - Nerve, Sciatic, Distal, Right, Distal	0.000233511	0.000330234	-0.002733526	0.003200548	1	1	0.5
Vehicle	Day 85	Liver - Nerve, Sciatic, Distal, Right, Proximal	0.000233511	0.000330234	-0.002733526	0.003200548	1	1	0.5
Vehicle	Day 85	Liver - Nerve, Sciatic, Proximal, Left, Distal	0.000233511	0.000330234	-0.002733526	0.003200548	1	1	0.5
Vehicle	Day 85	Liver - Nerve, Sciatic, Proximal, Left, Proximal	0.000233511	0.000330234	-0.002733526	0.003200548	1	1	0.5
Vehicle	Day 85	Liver - Nerve, Sciatic, Proximal, Right, Distal	6.95178E-05	9.8313E-05	-0.000813789	0.000952825	1	1	0.5
Vehicle	Day 85	Liver - Nerve, Sciatic, Proximal, Right, Proximal	9.50232E-05	0.000526085	-0.004631666	0.004821713	1	0.255439842	0.840786536
Vehicle	Day 85	Liver - Nerve, Ulnar, Distal, Left, Distal	0.000233511	0.000330234	-0.002733526	0.003200548	1	1	0.5
Vehicle	Day 85	Liver - Nerve, Ulnar, Distal, Left, Proximal	0.000233511	0.000330234	-0.002733526	0.003200548	1	1	0.5
Vehicle	Day 85	Liver - Nerve, Ulnar, Distal, Right, Distal	0.000233511	0.000330234	-0.002733526	0.003200548	1	1	0.5
Vehicle	Day 85	Liver - Nerve, Ulnar, Distal, Right, Proximal	0.000233511	0.000330234	-0.002733526	0.003200548	1	1	0.5
Vehicle	Day 85	Liver - Nerve, Ulnar, Proximal, Left, Distal	0.000233511	0.000330234	-0.002733526	0.003200548	1	1	0.5
Vehicle	Day 85	Liver - Nerve, Ulnar, Proximal, Left, Proximal	0.000233511	0.000330234	-0.002733526	0.003200548	1	1	0.5
Vehicle	Day 85	Liver - Nerve, Ulnar, Proximal, Right, Distal	0.000233511	0.000330234	-0.002733526	0.003200548	1	1	0.5
Vehicle	Day 85	Liver - Nerve, Ulnar, Proximal, Right, Proximal	0.000233511	0.000330234	-0.002733526	0.003200548	1	1	0.5
Vehicle	Day 85	Liver - Nerve, Optic	0.000233511	0.000330234	-0.002733526	0.003200548	1	1	0.5
Vehicle	Day 85	Liver - Ovary	0.000467022				0		
Vehicle	Day 85	Liver - Spinal Cord, Cervical	0.000233511	0.000330234	-0.002733526	0.003200548	1	1	0.5
Vehicle	Day 85	Liver - Spinal Cord, Lumbar	0.000233511	0.000330234	-0.002733526	0.003200548	1	1	0.5
Vehicle	Day 85	Liver - Spinal Cord, Sacral	0.000233511	0.000330234	-0.002733526	0.003200548	1	1	0.5
Vehicle	Day 85	Liver - Spinal Cord, Spinal Roots	0.000233511	0.000330234	-0.002733526	0.003200548	1	1	0.5
Vehicle	Day 85	Liver - Spinal Cord, Thoracic	0.000233511	0.000330234	-0.002733526	0.003200548	1	1	0.5
Vehicle	Day 85	Liver - Spleen	0.000107298	0.000151743	-0.001256057	0.001470654	1	1	0.5
Vehicle	Day 85	Liver - Testis	0				0		
Vehicle	Day 85	Muscle, Skeletal, Tibialis Anterior Left - Muscle, Skeletal, Tibialis Anterior Right	5.54296E-05	7.83893E-05	-0.00064887	0.000759729	1	1	0.5

Results of Evaluating Mean Differences Between Different Tissues Across Timepoint and Group for Log10 AAV2-ITR Target Copies/ug of DNA for Normalized Data

Group	Timepoint	Difference	Mean	StdDev	LowerCLMean	UpperCLMean	DF	tValue	P-Value
Vehicle	Day 85	Muscle, Skeletal, Tibialis Anterior Left - Muscle, Skeletal, Bicep Brachii Left	5.54296E-05	7.83893E-05	-0.00064887	0.000759729	1	1	0.5
Vehicle	Day 85	Muscle, Skeletal, Tibialis Anterior Left - Muscle, Skeletal, Bicep Brachii Right	5.54296E-05	7.83893E-05	-0.00064887	0.000759729	1	1	0.5
Vehicle	Day 85	Muscle, Skeletal, Tibialis Anterior Left - Nerve, Femoral, Distal, Left, Distal	5.54296E-05	7.83893E-05	-0.00064887	0.000759729	1	1	0.5
Vehicle	Day 85	Muscle, Skeletal, Tibialis Anterior Left - Nerve, Femoral, Distal, Left, Proximal	1.07968E-05	0.00014151	-0.001260616	0.00128221	1	0.107901059	0.931572791
Vehicle	Day 85	Muscle, Skeletal, Tibialis Anterior Left - Nerve, Femoral, Distal, Right, Distal	5.54296E-05	7.83893E-05	-0.00064887	0.000759729	1	1	0.5
Vehicle	Day 85	Muscle, Skeletal, Tibialis Anterior Left - Nerve, Femoral, Distal, Right, Proximal	5.54296E-05	7.83893E-05	-0.00064887	0.000759729	1	1	0.5
Vehicle	Day 85	Muscle, Skeletal, Tibialis Anterior Left - Nerve, Femoral, Proximal, Left, Distal	5.54296E-05	7.83893E-05	-0.00064887	0.000759729	1	1	0.5
Vehicle	Day 85	Muscle, Skeletal, Tibialis Anterior Left - Nerve, Femoral, Proximal, Left, Proximal	5.54296E-05	7.83893E-05	-0.00064887	0.000759729	1	1	0.5
Vehicle	Day 85	Muscle, Skeletal, Tibialis Anterior Left - Nerve, Femoral, Proximal, Right, Distal	-3.01775E-05	0.000199456	-0.001822219	0.001761864	1	-0.21396907	0.865806555
Vehicle	Day 85	Muscle, Skeletal, Tibialis Anterior Left - Nerve, Femoral, Proximal, Right, Proximal	5.54296E-05	7.83893E-05	-0.00064887	0.000759729	1	1	0.5
Vehicle	Day 85	Muscle, Skeletal, Tibialis Anterior Left - Nerve Median, Distal, Left, Distal	5.54296E-05	7.83893E-05	-0.00064887	0.000759729	1	1	0.5
Vehicle	Day 85	Muscle, Skeletal, Tibialis Anterior Left - Nerve Median, Distal, Left, Proximal	5.54296E-05	7.83893E-05	-0.00064887	0.000759729	1	1	0.5
Vehicle	Day 85	Muscle, Skeletal, Tibialis Anterior Left - Nerve Median, Distal, Right, Distal	5.54296E-05	7.83893E-05	-0.00064887	0.000759729	1	1	0.5
Vehicle	Day 85	Muscle, Skeletal, Tibialis Anterior Left - Nerve Median, Distal, Right, Proximal	5.54296E-05	7.83893E-05	-0.00064887	0.000759729	1	1	0.5
Vehicle	Day 85	Muscle, Skeletal, Tibialis Anterior Left - Nerve Median, Proximal, Left, Distal	5.54296E-05	7.83893E-05	-0.00064887	0.000759729	1	1	0.5
Vehicle	Day 85	Muscle, Skeletal, Tibialis Anterior Left - Nerve Median, Proximal, Left, Proximal	9.59678E-06	0.000143207	-0.001277064	0.001296258	1	0.09477143	0.939846297
Vehicle	Day 85	Muscle, Skeletal, Tibialis Anterior Left - Nerve Median, Proximal, Right, Distal	5.54296E-05	7.83893E-05	-0.00064887	0.000759729	1	1	0.5
Vehicle	Day 85	Muscle, Skeletal, Tibialis Anterior Left - Nerve Median, Proximal, Right, Proximal	5.54296E-05	7.83893E-05	-0.00064887	0.000759729	1	1	0.5
Vehicle	Day 85	Muscle, Skeletal, Tibialis Anterior Left - Nerve, Sciatic, Distal, Left, Distal	-5.27673E-05	7.46242E-05	-0.000723239	0.000617705	1	-1	0.5
Vehicle	Day 85	Muscle, Skeletal, Tibialis Anterior Left - Nerve, Sciatic, Distal, Left, Proximal	5.54296E-05	7.83893E-05	-0.00064887	0.000759729	1	1	0.5
Vehicle	Day 85	Muscle, Skeletal, Tibialis Anterior Left - Nerve, Sciatic, Distal, Right, Distal	5.54296E-05	7.83893E-05	-0.00064887	0.000759729	1	1	0.5
Vehicle	Day 85	Muscle, Skeletal, Tibialis Anterior Left - Nerve, Sciatic, Distal, Right, Proximal	5.54296E-05	7.83893E-05	-0.00064887	0.000759729	1	1	0.5
Vehicle	Day 85	Muscle, Skeletal, Tibialis Anterior Left - Nerve, Sciatic, Proximal, Left, Distal	5.54296E-05	7.83893E-05	-0.00064887	0.000759729	1	1	0.5
Vehicle	Day 85	Muscle, Skeletal, Tibialis Anterior Left - Nerve, Sciatic, Proximal, Left, Proximal	5.54296E-05	7.83893E-05	-0.00064887	0.000759729	1	1	0.5
Vehicle	Day 85	Muscle, Skeletal, Tibialis Anterior Left - Nerve, Sciatic, Proximal, Right, Distal	-0.000108564	0.000153532	-0.001487994	0.001270867	1	-1	0.5
Vehicle	Day 85	Muscle, Skeletal, Tibialis Anterior Left - Nerve, Sciatic, Proximal, Right, Proximal	-8.3058E-05	0.00027424	-0.00254701	0.002380894	1	-0.428316959	0.742374758
Vehicle	Day 85	Muscle, Skeletal, Tibialis Anterior Left - Nerve, Ulnar, Distal, Left, Distal	5.54296E-05	7.83893E-05	-0.00064887	0.000759729	1	1	0.5
Vehicle	Day 85	Muscle, Skeletal, Tibialis Anterior Left - Nerve, Ulnar, Distal, Left, Proximal	5.54296E-05	7.83893E-05	-0.00064887	0.000759729	1	1	0.5
Vehicle	Day 85	Muscle, Skeletal, Tibialis Anterior Left - Nerve, Ulnar, Distal, Right, Distal	5.54296E-05	7.83893E-05	-0.00064887	0.000759729	1	1	0.5
Vehicle	Day 85	Muscle, Skeletal, Tibialis Anterior Left - Nerve, Ulnar, Distal, Right, Proximal	5.54296E-05	7.83893E-05	-0.00064887	0.000759729	1	1	0.5
Vehicle	Day 85	Muscle, Skeletal, Tibialis Anterior Left - Nerve, Ulnar, Proximal, Left, Distal	5.54296E-05	7.83893E-05	-0.00064887	0.000759729	1	1	0.5
Vehicle	Day 85	Muscle, Skeletal, Tibialis Anterior Left - Nerve, Ulnar, Proximal, Left, Proximal	5.54296E-05	7.83893E-05	-0.00064887	0.000759729	1	1	0.5
Vehicle	Day 85	Muscle, Skeletal, Tibialis Anterior Left - Nerve, Ulnar, Proximal, Right, Distal	5.54296E-05	7.83893E-05	-0.00064887	0.000759729	1	1	0.5
Vehicle	Day 85	Muscle, Skeletal, Tibialis Anterior Left - Nerve, Ulnar, Proximal, Right, Proximal	5.54296E-05	7.83893E-05	-0.00064887	0.000759729	1	1	0.5
Vehicle	Day 85	Muscle, Skeletal, Tibialis Anterior Left - Nerve, Optic	5.54296E-05	7.83893E-05	-0.00064887	0.000759729	1	1	0.5
Vehicle	Day 85	Muscle, Skeletal, Tibialis Anterior Left - Ovary	0.000110859				0		
Vehicle	Day 85	Muscle, Skeletal, Tibialis Anterior Left - Spinal Cord, Cervical	5.54296E-05	7.83893E-05	-0.00064887	0.000759729	1	1	0.5
Vehicle	Day 85	Muscle, Skeletal, Tibialis Anterior Left - Spinal Cord, Lumbar	5.54296E-05	7.83893E-05	-0.00064887	0.000759729	1	1	0.5
Vehicle	Day 85	Muscle, Skeletal, Tibialis Anterior Left - Spinal Cord, Sacral	5.54296E-05	7.83893E-05	-0.00064887	0.000759729	1	1	0.5
Vehicle	Day 85	Muscle, Skeletal, Tibialis Anterior Left - Spinal Cord, Spinal Roots	5.54296E-05	7.83893E-05	-0.00064887	0.000759729	1	1	0.5
Vehicle	Day 85	Muscle, Skeletal, Tibialis Anterior Left - Spinal Cord, Thoracic	5.54296E-05	7.83893E-05	-0.00064887	0.000759729	1	1	0.5
Vehicle	Day 85	Muscle, Skeletal, Tibialis Anterior Left - Spleen	-7.07829E-05	0.000100102	-0.000970164	0.000828599	1	-1	0.5
Vehicle	Day 85	Muscle, Skeletal, Tibialis Anterior Left - Testis	0				0		
Vehicle	Day 85	Muscle, Skeletal, Tibialis Anterior Right - Muscle, Skeletal, Bicep Brachii Left	0	0	0	0	1		
Vehicle	Day 85	Muscle, Skeletal, Tibialis Anterior Right - Muscle, Skeletal, Bicep Brachii Right	0	0	0	0	1		
Vehicle	Day 85	Muscle, Skeletal, Tibialis Anterior Right - Nerve, Femoral, Distal, Left, Distal	0	0	0	0	1		
Vehicle	Day 85	Muscle, Skeletal, Tibialis Anterior Right - Nerve, Femoral, Distal, Left, Proximal	-4.46328E-05	6.31203E-05	-0.000611746	0.00052248	1	-1	0.5
Vehicle	Day 85	Muscle, Skeletal, Tibialis Anterior Right - Nerve, Femoral, Distal, Right, Distal	0	0	0	0	1		
Vehicle	Day 85	Muscle, Skeletal, Tibialis Anterior Right - Nerve, Femoral, Distal, Right, Proximal	0	0	0	0	1		
Vehicle	Day 85	Muscle, Skeletal, Tibialis Anterior Right - Nerve, Femoral, Proximal, Left, Distal	0	0	0	0	1		
Vehicle	Day 85	Muscle, Skeletal, Tibialis Anterior Right - Nerve, Femoral, Proximal, Left, Proximal	0	0	0	0	1		
Vehicle	Day 85	Muscle, Skeletal, Tibialis Anterior Right - Nerve, Femoral, Proximal, Right, Distal	-8.56071E-05	0.000121067	-0.001173348	0.001002134	1	-1	0.5
Vehicle	Day 85	Muscle, Skeletal, Tibialis Anterior Right - Nerve, Femoral, Proximal, Right, Proximal	0	0	0	0	1		
Vehicle	Day 85	Muscle, Skeletal, Tibialis Anterior Right - Nerve Median, Distal, Left, Distal	0	0	0	0	1		
Vehicle	Day 85	Muscle, Skeletal, Tibialis Anterior Right - Nerve Median, Distal, Left, Proximal	0	0	0	0	1		
Vehicle	Day 85	Muscle, Skeletal, Tibialis Anterior Right - Nerve Median, Distal, Right, Distal	0	0	0	0	1		
Vehicle	Day 85	Muscle, Skeletal, Tibialis Anterior Right - Nerve Median, Distal, Right, Proximal	0	0	0	0	1		
Vehicle	Day 85	Muscle, Skeletal, Tibialis Anterior Right - Nerve Median, Proximal, Left, Distal	0	0	0	0	1		
Vehicle	Day 85	Muscle, Skeletal, Tibialis Anterior Right - Nerve Median, Proximal, Left, Proximal	-4.58328E-05	6.48174E-05	-0.000628194	0.000536528	1	-1	0.5
Vehicle	Day 85	Muscle, Skeletal, Tibialis Anterior Right - Nerve Median, Proximal, Right, Distal	0	0	0	0	1		
Vehicle	Day 85	Muscle, Skeletal, Tibialis Anterior Right - Nerve Median, Proximal, Right, Proximal	0	0	0	0	1		
Vehicle	Day 85	Muscle, Skeletal, Tibialis Anterior Right - Nerve, Sciatic, Distal, Left, Distal	-0.000108197	0.000153014	-0.001482969	0.001266575	1	-1	0.5
Vehicle	Day 85	Muscle, Skeletal, Tibialis Anterior Right - Nerve, Sciatic, Distal, Left, Proximal	0	0	0	0	1		
Vehicle	Day 85	Muscle, Skeletal, Tibialis Anterior Right - Nerve, Sciatic, Distal, Right, Distal	0	0	0	0	1		
Vehicle	Day 85	Muscle, Skeletal, Tibialis Anterior Right - Nerve, Sciatic, Distal, Right, Proximal	0	0	0	0	1		
Vehicle	Day 85	Muscle, Skeletal, Tibialis Anterior Right - Nerve, Sciatic, Proximal, Left, Distal	0	0	0	0	1		
Vehicle	Day 85	Muscle, Skeletal, Tibialis Anterior Right - Nerve, Sciatic, Proximal, Left, Proximal	0	0	0	0	1		
Vehicle	Day 85	Muscle, Skeletal, Tibialis Anterior Right - Nerve, Sciatic, Proximal, Right, Distal	-0.000163993	0.000231921	-0.002247723	0.001919737	1	-1	0.5
Vehicle	Day 85	Muscle, Skeletal, Tibialis Anterior Right - Nerve, Sciatic, Proximal, Right, Proximal	-0.000138488	0.000195851	-0.00189814	0.001621165	1	-1	0.5
Vehicle	Day 85	Muscle, Skeletal, Tibialis Anterior Right - Nerve, Ulnar, Distal, Left, Distal	0	0	0	0	1		
Vehicle	Day 85	Muscle, Skeletal, Tibialis Anterior Right - Nerve, Ulnar, Distal, Left, Proximal	0	0	0	0	1		
Vehicle	Day 85	Muscle, Skeletal, Tibialis Anterior Right - Nerve, Ulnar, Distal, Right, Distal	0	0	0	0	1		
Vehicle	Day 85	Muscle, Skeletal, Tibialis Anterior Right - Nerve, Ulnar, Distal, Right, Proximal	0	0	0	0	1		
Vehicle	Day 85	Muscle, Skeletal, Tibialis Anterior Right - Nerve, Ulnar, Proximal, Left, Distal	0	0	0	0	1		
Vehicle	Day 85	Muscle, Skeletal, Tibialis Anterior Right - Nerve, Ulnar, Proximal, Left, Proximal	0	0	0	0	1		
Vehicle	Day 85	Muscle, Skeletal, Tibialis Anterior Right - Nerve, Ulnar, Proximal, Right, Distal	0	0	0	0	1		
Vehicle	Day 85	Muscle, Skeletal, Tibialis Anterior Right - Nerve, Ulnar, Proximal, Right, Proximal	0	0	0	0	1		
Vehicle	Day 85	Muscle, Skeletal, Tibialis Anterior Right - Nerve, Optic	0	0	0	0	1		
Vehicle	Day 85	Muscle, Skeletal, Tibialis Anterior Right - Ovary	0				0		
Vehicle	Day 85	Muscle, Skeletal, Tibialis Anterior Right - Spinal Cord, Cervical	0	0	0	0	1		
Vehicle	Day 85	Muscle, Skeletal, Tibialis Anterior Right - Spinal Cord, Lumbar	0	0	0	0	1		
Vehicle	Day 85	Muscle, Skeletal, Tibialis Anterior Right - Spinal Cord, Sacral	0	0	0	0	1		
Vehicle	Day 85	Muscle, Skeletal, Tibialis Anterior Right - Spinal Cord, Spinal Roots	0	0	0	0	1		
Vehicle	Day 85	Muscle, Skeletal, Tibialis Anterior Right - Spinal Cord, Thoracic	0	0	0	0	1		
Vehicle	Day 85	Muscle, Skeletal, Tibialis Anterior Right - Spleen	-0.000126212	0.000178491	-0.001729894	0.001477469	1	-1	0.5
Vehicle	Day 85	Muscle, Skeletal, Tibialis Anterior Right - Testis	0				0		
Vehicle	Day 85	Muscle, Skeletal, Bicep Brachii Left - Muscle, Skeletal, Bicep Brachii Right	0	0	0	0	1		
Vehicle	Day 85	Muscle, Skeletal, Bicep Brachii Left - Nerve, Femoral, Distal, Left, Distal	0	0	0	0	1		
Vehicle	Day 85	Muscle, Skeletal, Bicep Brachii Left - Nerve, Femoral, Distal, Left, Proximal	-4.46328E-05	6.31203E-05	-0.000611746	0.00052248	1	-1	0.5
Vehicle	Day 85	Muscle, Skeletal, Bicep Brachii Left - Nerve, Femoral, Distal, Right, Distal	0	0	0	0	1		
Vehicle	Day 85	Muscle, Skeletal, Bicep Brachii Left - Nerve, Femoral, Distal, Right, Proximal	0	0	0	0	1		
Vehicle	Day 85	Muscle, Skeletal, Bicep Brachii Left - Nerve, Femoral, Proximal, Left, Distal	0	0	0	0	1		
Vehicle	Day 85	Muscle, Skeletal, Bicep Brachii Left - Nerve, Femoral, Proximal, Left, Proximal	0	0	0	0	1		

Results of Evaluating Mean Differences Between Different Tissues Across Timepoint and Group for Log10 AAV2-ITR Target Copies/ug of DNA for Normalized Data

Group	Timepoint	Difference	Mean	StdDev	LowerCLMean	UpperCLMean	DF	tValue	P-Value
Vehicle	Day 85	Muscle, Skeletal, Bicep Brachii Left - Nerve, Femoral, Proximal, Right, Distal	-8.56071E-05	0.000121067	-0.001173348	0.001002134	1	-1	0.5
Vehicle	Day 85	Muscle, Skeletal, Bicep Brachii Left - Nerve, Femoral, Proximal, Right, Proximal	0	0	0	0	1		
Vehicle	Day 85	Muscle, Skeletal, Bicep Brachii Left - Nerve Median, Distal, Left, Distal	0	0	0	0	1		
Vehicle	Day 85	Muscle, Skeletal, Bicep Brachii Left - Nerve Median, Distal, Left, Proximal	0	0	0	0	1		
Vehicle	Day 85	Muscle, Skeletal, Bicep Brachii Left - Nerve Median, Distal, Right, Distal	0	0	0	0	1		
Vehicle	Day 85	Muscle, Skeletal, Bicep Brachii Left - Nerve Median, Distal, Right, Proximal	0	0	0	0	1		
Vehicle	Day 85	Muscle, Skeletal, Bicep Brachii Left - Nerve Median, Proximal, Left, Distal	0	0	0	0	1		
Vehicle	Day 85	Muscle, Skeletal, Bicep Brachii Left - Nerve Median, Proximal, Left, Proximal	-4.58328E-05	6.48174E-05	-0.000628194	0.000536528	1	-1	0.5
Vehicle	Day 85	Muscle, Skeletal, Bicep Brachii Left - Nerve Median, Proximal, Right, Distal	0	0	0	0	1		
Vehicle	Day 85	Muscle, Skeletal, Bicep Brachii Left - Nerve Median, Proximal, Right, Proximal	0	0	0	0	1		
Vehicle	Day 85	Muscle, Skeletal, Bicep Brachii Left - Nerve, Sciatic, Distal, Left, Distal	-0.000108197	0.000153014	-0.001482969	0.001266575	1	-1	0.5
Vehicle	Day 85	Muscle, Skeletal, Bicep Brachii Left - Nerve, Sciatic, Distal, Left, Proximal	0	0	0	0	1		
Vehicle	Day 85	Muscle, Skeletal, Bicep Brachii Left - Nerve, Sciatic, Distal, Right, Distal	0	0	0	0	1		
Vehicle	Day 85	Muscle, Skeletal, Bicep Brachii Left - Nerve, Sciatic, Distal, Right, Proximal	0	0	0	0	1		
Vehicle	Day 85	Muscle, Skeletal, Bicep Brachii Left - Nerve, Sciatic, Proximal, Left, Distal	0	0	0	0	1		
Vehicle	Day 85	Muscle, Skeletal, Bicep Brachii Left - Nerve, Sciatic, Proximal, Left, Proximal	0	0	0	0	1		
Vehicle	Day 85	Muscle, Skeletal, Bicep Brachii Left - Nerve, Sciatic, Proximal, Right, Distal	-0.000163993	0.000231921	-0.002247723	0.001919737	1	-1	0.5
Vehicle	Day 85	Muscle, Skeletal, Bicep Brachii Left - Nerve, Sciatic, Proximal, Right, Proximal	-0.000138488	0.000195851	-0.00189814	0.001621165	1	-1	0.5
Vehicle	Day 85	Muscle, Skeletal, Bicep Brachii Left - Nerve, Ulnar, Distal, Left, Distal	0	0	0	0	1		
Vehicle	Day 85	Muscle, Skeletal, Bicep Brachii Left - Nerve, Ulnar, Distal, Left, Proximal	0	0	0	0	1		
Vehicle	Day 85	Muscle, Skeletal, Bicep Brachii Left - Nerve, Ulnar, Distal, Right, Distal	0	0	0	0	1		
Vehicle	Day 85	Muscle, Skeletal, Bicep Brachii Left - Nerve, Ulnar, Distal, Right, Proximal	0	0	0	0	1		
Vehicle	Day 85	Muscle, Skeletal, Bicep Brachii Left - Nerve, Ulnar, Proximal, Left, Distal	0	0	0	0	1		
Vehicle	Day 85	Muscle, Skeletal, Bicep Brachii Left - Nerve, Ulnar, Proximal, Left, Proximal	0	0	0	0	1		
Vehicle	Day 85	Muscle, Skeletal, Bicep Brachii Left - Nerve, Ulnar, Proximal, Right, Distal	0	0	0	0	1		
Vehicle	Day 85	Muscle, Skeletal, Bicep Brachii Left - Nerve, Ulnar, Proximal, Right, Proximal	0	0	0	0	1		
Vehicle	Day 85	Muscle, Skeletal, Bicep Brachii Left - Nerve, Optic	0	0	0	0	1		
Vehicle	Day 85	Muscle, Skeletal, Bicep Brachii Left - Ovary	0				0		
Vehicle	Day 85	Muscle, Skeletal, Bicep Brachii Left - Spinal Cord, Cervical	0	0	0	0	1		
Vehicle	Day 85	Muscle, Skeletal, Bicep Brachii Left - Spinal Cord, Lumbar	0	0	0	0	1		
Vehicle	Day 85	Muscle, Skeletal, Bicep Brachii Left - Spinal Cord, Sacral	0	0	0	0	1		
Vehicle	Day 85	Muscle, Skeletal, Bicep Brachii Left - Spinal Cord, Spinal Roots	0	0	0	0	1		
Vehicle	Day 85	Muscle, Skeletal, Bicep Brachii Left - Spinal Cord, Thoracic	0	0	0	0	1		
Vehicle	Day 85	Muscle, Skeletal, Bicep Brachii Left - Spleen	-0.000126212	0.000178491	-0.001729894	0.001477469	1	-1	0.5
Vehicle	Day 85	Muscle, Skeletal, Bicep Brachii Left - Testis	0				0		
Vehicle	Day 85	Muscle, Skeletal, Bicep Brachii Right - Nerve, Femoral, Distal, Left, Distal	0	0	0	0	1		
Vehicle	Day 85	Muscle, Skeletal, Bicep Brachii Right - Nerve, Femoral, Distal, Left, Proximal	-4.46328E-05	6.31203E-05	-0.000611746	0.00052248	1	-1	0.5
Vehicle	Day 85	Muscle, Skeletal, Bicep Brachii Right - Nerve, Femoral, Distal, Right, Distal	0	0	0	0	1		
Vehicle	Day 85	Muscle, Skeletal, Bicep Brachii Right - Nerve, Femoral, Distal, Right, Proximal	0	0	0	0	1		
Vehicle	Day 85	Muscle, Skeletal, Bicep Brachii Right - Nerve, Femoral, Proximal, Left, Distal	0	0	0	0	1		
Vehicle	Day 85	Muscle, Skeletal, Bicep Brachii Right - Nerve, Femoral, Proximal, Left, Proximal	0	0	0	0	1		
Vehicle	Day 85	Muscle, Skeletal, Bicep Brachii Right - Nerve, Femoral, Proximal, Right, Distal	-8.56071E-05	0.000121067	-0.001173348	0.001002134	1	-1	0.5
Vehicle	Day 85	Muscle, Skeletal, Bicep Brachii Right - Nerve, Femoral, Proximal, Right, Proximal	0	0	0	0	1		
Vehicle	Day 85	Muscle, Skeletal, Bicep Brachii Right - Nerve Median, Distal, Left, Distal	0	0	0	0	1		
Vehicle	Day 85	Muscle, Skeletal, Bicep Brachii Right - Nerve Median, Distal, Left, Proximal	0	0	0	0	1		
Vehicle	Day 85	Muscle, Skeletal, Bicep Brachii Right - Nerve Median, Distal, Right, Distal	0	0	0	0	1		
Vehicle	Day 85	Muscle, Skeletal, Bicep Brachii Right - Nerve Median, Distal, Right, Proximal	0	0	0	0	1		
Vehicle	Day 85	Muscle, Skeletal, Bicep Brachii Right - Nerve Median, Proximal, Left, Distal	0	0	0	0	1		
Vehicle	Day 85	Muscle, Skeletal, Bicep Brachii Right - Nerve Median, Proximal, Left, Proximal	-4.58328E-05	6.48174E-05	-0.000628194	0.000536528	1	-1	0.5
Vehicle	Day 85	Muscle, Skeletal, Bicep Brachii Right - Nerve Median, Proximal, Right, Distal	0	0	0	0	1		
Vehicle	Day 85	Muscle, Skeletal, Bicep Brachii Right - Nerve Median, Proximal, Right, Proximal	0	0	0	0	1		
Vehicle	Day 85	Muscle, Skeletal, Bicep Brachii Right - Nerve, Sciatic, Distal, Left, Distal	-0.000108197	0.000153014	-0.001482969	0.001266575	1	-1	0.5
Vehicle	Day 85	Muscle, Skeletal, Bicep Brachii Right - Nerve, Sciatic, Distal, Left, Proximal	0	0	0	0	1		
Vehicle	Day 85	Muscle, Skeletal, Bicep Brachii Right - Nerve, Sciatic, Distal, Right, Distal	0	0	0	0	1		
Vehicle	Day 85	Muscle, Skeletal, Bicep Brachii Right - Nerve, Sciatic, Distal, Right, Proximal	0	0	0	0	1		
Vehicle	Day 85	Muscle, Skeletal, Bicep Brachii Right - Nerve, Sciatic, Proximal, Left, Distal	0	0	0	0	1		
Vehicle	Day 85	Muscle, Skeletal, Bicep Brachii Right - Nerve, Sciatic, Proximal, Left, Proximal	0	0	0	0	1		
Vehicle	Day 85	Muscle, Skeletal, Bicep Brachii Right - Nerve, Sciatic, Proximal, Right, Distal	-0.000163993	0.000231921	-0.002247723	0.001919737	1	-1	0.5
Vehicle	Day 85	Muscle, Skeletal, Bicep Brachii Right - Nerve, Sciatic, Proximal, Right, Proximal	-0.000138488	0.000195851	-0.00189814	0.001621165	1	-1	0.5
Vehicle	Day 85	Muscle, Skeletal, Bicep Brachii Right - Nerve, Ulnar, Distal, Left, Distal	0	0	0	0	1		
Vehicle	Day 85	Muscle, Skeletal, Bicep Brachii Right - Nerve, Ulnar, Distal, Left, Proximal	0	0	0	0	1		
Vehicle	Day 85	Muscle, Skeletal, Bicep Brachii Right - Nerve, Ulnar, Distal, Right, Distal	0	0	0	0	1		
Vehicle	Day 85	Muscle, Skeletal, Bicep Brachii Right - Nerve, Ulnar, Distal, Right, Proximal	0	0	0	0	1		
Vehicle	Day 85	Muscle, Skeletal, Bicep Brachii Right - Nerve, Ulnar, Proximal, Left, Distal	0	0	0	0	1		
Vehicle	Day 85	Muscle, Skeletal, Bicep Brachii Right - Nerve, Ulnar, Proximal, Left, Proximal	0	0	0	0	1		
Vehicle	Day 85	Muscle, Skeletal, Bicep Brachii Right - Nerve, Ulnar, Proximal, Right, Distal	0	0	0	0	1		
Vehicle	Day 85	Muscle, Skeletal, Bicep Brachii Right - Nerve, Ulnar, Proximal, Right, Proximal	0	0	0	0	1		
Vehicle	Day 85	Muscle, Skeletal, Bicep Brachii Right - Nerve, Optic	0	0	0	0	1		
Vehicle	Day 85	Muscle, Skeletal, Bicep Brachii Right - Ovary	0				0		
Vehicle	Day 85	Muscle, Skeletal, Bicep Brachii Right - Spinal Cord, Cervical	0	0	0	0	1		
Vehicle	Day 85	Muscle, Skeletal, Bicep Brachii Right - Spinal Cord, Lumbar	0	0	0	0	1		
Vehicle	Day 85	Muscle, Skeletal, Bicep Brachii Right - Spinal Cord, Sacral	0	0	0	0	1		
Vehicle	Day 85	Muscle, Skeletal, Bicep Brachii Right - Spinal Cord, Spinal Roots	0	0	0	0	1		
Vehicle	Day 85	Muscle, Skeletal, Bicep Brachii Right - Spinal Cord, Thoracic	0	0	0	0	1		
Vehicle	Day 85	Muscle, Skeletal, Bicep Brachii Right - Spleen	-0.000126212	0.000178491	-0.001729894	0.001477469	1	-1	0.5
Vehicle	Day 85	Muscle, Skeletal, Bicep Brachii Right - Testis	0				0		
Vehicle	Day 85	Nerve, Femoral, Distal, Left, Distal - Nerve, Femoral, Distal, Left, Proximal	-4.46328E-05	6.31203E-05	-0.000611746	0.00052248	1	-1	0.5
Vehicle	Day 85	Nerve, Femoral, Distal, Left, Distal - Nerve, Femoral, Distal, Right, Distal	0	0	0	0	1		
Vehicle	Day 85	Nerve, Femoral, Distal, Left, Distal - Nerve, Femoral, Distal, Right, Proximal	0	0	0	0	1		
Vehicle	Day 85	Nerve, Femoral, Distal, Left, Distal - Nerve, Femoral, Proximal, Left, Distal	0	0	0	0	1		
Vehicle	Day 85	Nerve, Femoral, Distal, Left, Distal - Nerve, Femoral, Proximal, Left, Proximal	0	0	0	0	1		
Vehicle	Day 85	Nerve, Femoral, Distal, Left, Distal - Nerve, Femoral, Proximal, Right, Distal	-8.56071E-05	0.000121067	-0.001173348	0.001002134	1	-1	0.5
Vehicle	Day 85	Nerve, Femoral, Distal, Left, Distal - Nerve, Femoral, Proximal, Right, Proximal	0	0	0	0	1		
Vehicle	Day 85	Nerve, Femoral, Distal, Left, Distal - Nerve Median, Distal, Left, Distal	0	0	0	0	1		
Vehicle	Day 85	Nerve, Femoral, Distal, Left, Distal - Nerve Median, Distal, Left, Proximal	0	0	0	0	1		
Vehicle	Day 85	Nerve, Femoral, Distal, Left, Distal - Nerve Median, Distal, Right, Distal	0	0	0	0	1		
Vehicle	Day 85	Nerve, Femoral, Distal, Left, Distal - Nerve Median, Distal, Right, Proximal	0	0	0	0	1		
Vehicle	Day 85	Nerve, Femoral, Distal, Left, Distal - Nerve Median, Proximal, Left, Distal	0	0	0	0	1		
Vehicle	Day 85	Nerve, Femoral, Distal, Left, Distal - Nerve Median, Proximal, Left, Proximal	-4.58328E-05	6.48174E-05	-0.000628194	0.000536528	1	-1	0.5
Vehicle	Day 85	Nerve, Femoral, Distal, Left, Distal - Nerve Median, Proximal, Right, Distal	0	0	0	0	1		
Vehicle	Day 85	Nerve, Femoral, Distal, Left, Distal - Nerve Median, Proximal, Right, Proximal	0	0	0	0	1		
Vehicle	Day 85	Nerve, Femoral, Distal, Left, Distal - Nerve, Sciatic, Distal, Left, Distal	-0.000108197	0.000153014	-0.001482969	0.001266575	1	-1	0.5
Vehicle	Day 85	Nerve, Femoral, Distal, Left, Distal - Nerve, Sciatic, Distal, Left, Proximal	0	0	0	0	1		

Results of Evaluating Mean Differences Between Different Tissues Across Timepoint and Group for Log10 AAV2-ITR Target Copies/ug of DNA for Normalized Data

Group	Timepoint	Difference	Mean	StdDev	LowerCLMean	UpperCLMean	DF	tValue	P-Value
Vehicle	Day 85	Nerve, Femoral, Distal, Left, Distal - Nerve, Sciatic, Distal, Right, Distal	0	0	0	0	1		
Vehicle	Day 85	Nerve, Femoral, Distal, Left, Distal - Nerve, Sciatic, Distal, Right, Proximal	0	0	0	0	1		
Vehicle	Day 85	Nerve, Femoral, Distal, Left, Distal - Nerve, Sciatic, Proximal, Left, Distal	0	0	0	0	1		
Vehicle	Day 85	Nerve, Femoral, Distal, Left, Distal - Nerve, Sciatic, Proximal, Left, Proximal	0	0	0	0	1		
Vehicle	Day 85	Nerve, Femoral, Distal, Left, Distal - Nerve, Sciatic, Proximal, Right, Distal	-0.000163993	0.000231921	-0.002247723	0.001919737	1	-1	0.5
Vehicle	Day 85	Nerve, Femoral, Distal, Left, Distal - Nerve, Sciatic, Proximal, Right, Proximal	-0.000138488	0.000195851	-0.00189814	0.001621165	1	-1	0.5
Vehicle	Day 85	Nerve, Femoral, Distal, Left, Distal - Nerve, Ulnar, Distal, Left, Distal	0	0	0	0	1		
Vehicle	Day 85	Nerve, Femoral, Distal, Left, Distal - Nerve, Ulnar, Distal, Left, Proximal	0	0	0	0	1		
Vehicle	Day 85	Nerve, Femoral, Distal, Left, Distal - Nerve, Ulnar, Distal, Right, Distal	0	0	0	0	1		
Vehicle	Day 85	Nerve, Femoral, Distal, Left, Distal - Nerve, Ulnar, Distal, Right, Proximal	0	0	0	0	1		
Vehicle	Day 85	Nerve, Femoral, Distal, Left, Distal - Nerve, Ulnar, Proximal, Left, Distal	0	0	0	0	1		
Vehicle	Day 85	Nerve, Femoral, Distal, Left, Distal - Nerve, Ulnar, Proximal, Left, Proximal	0	0	0	0	1		
Vehicle	Day 85	Nerve, Femoral, Distal, Left, Distal - Nerve, Ulnar, Proximal, Right, Distal	0	0	0	0	1		
Vehicle	Day 85	Nerve, Femoral, Distal, Left, Distal - Nerve, Ulnar, Proximal, Right, Proximal	0	0	0	0	1		
Vehicle	Day 85	Nerve, Femoral, Distal, Left, Distal - Nerve, Optic	0	0	0	0	1		
Vehicle	Day 85	Nerve, Femoral, Distal, Left, Distal - Ovary	0				0		
Vehicle	Day 85	Nerve, Femoral, Distal, Left, Distal - Spinal Cord, Cervical	0	0	0	0	1		
Vehicle	Day 85	Nerve, Femoral, Distal, Left, Distal - Spinal Cord, Lumbar	0	0	0	0	1		
Vehicle	Day 85	Nerve, Femoral, Distal, Left, Distal - Spinal Cord, Sacral	0	0	0	0	1		
Vehicle	Day 85	Nerve, Femoral, Distal, Left, Distal - Spinal Cord, Spinal Roots	0	0	0	0	1		
Vehicle	Day 85	Nerve, Femoral, Distal, Left, Distal - Spinal Cord, Thoracic	0	0	0	0	1		
Vehicle	Day 85	Nerve, Femoral, Distal, Left, Distal - Spleen	-0.000126212	0.000178491	-0.001729894	0.001477469	1	-1	0.5
Vehicle	Day 85	Nerve, Femoral, Distal, Left, Distal - Testis	0				0		
Vehicle	Day 85	Nerve, Femoral, Distal, Left, Proximal - Nerve, Femoral, Distal, Right, Distal	4.46328E-05	6.31203E-05	-0.00052248	0.000611746	1	1	0.5
Vehicle	Day 85	Nerve, Femoral, Distal, Left, Proximal - Nerve, Femoral, Distal, Right, Proximal	4.46328E-05	6.31203E-05	-0.00052248	0.000611746	1	1	0.5
Vehicle	Day 85	Nerve, Femoral, Distal, Left, Proximal - Nerve, Femoral, Proximal, Left, Distal	4.46328E-05	6.31203E-05	-0.00052248	0.000611746	1	1	0.5
Vehicle	Day 85	Nerve, Femoral, Distal, Left, Proximal - Nerve, Femoral, Proximal, Left, Proximal	4.46328E-05	6.31203E-05	-0.00052248	0.000611746	1	1	0.5
Vehicle	Day 85	Nerve, Femoral, Distal, Left, Proximal - Nerve, Femoral, Proximal, Right, Distal	-4.09743E-05	5.79464E-05	-0.000561602	0.000479654	1	-1	0.5
Vehicle	Day 85	Nerve, Femoral, Distal, Left, Proximal - Nerve, Femoral, Proximal, Right, Proximal	4.46328E-05	6.31203E-05	-0.00052248	0.000611746	1	1	0.5
Vehicle	Day 85	Nerve, Femoral, Distal, Left, Proximal - Nerve Median, Distal, Left, Distal	4.46328E-05	6.31203E-05	-0.00052248	0.000611746	1	1	0.5
Vehicle	Day 85	Nerve, Femoral, Distal, Left, Proximal - Nerve Median, Distal, Left, Proximal	4.46328E-05	6.31203E-05	-0.00052248	0.000611746	1	1	0.5
Vehicle	Day 85	Nerve, Femoral, Distal, Left, Proximal - Nerve Median, Distal, Right, Distal	4.46328E-05	6.31203E-05	-0.00052248	0.000611746	1	1	0.5
Vehicle	Day 85	Nerve, Femoral, Distal, Left, Proximal - Nerve Median, Distal, Right, Proximal	4.46328E-05	6.31203E-05	-0.00052248	0.000611746	1	1	0.5
Vehicle	Day 85	Nerve, Femoral, Distal, Left, Proximal - Nerve Median, Proximal, Left, Distal	4.46328E-05	6.31203E-05	-0.00052248	0.000611746	1	1	0.5
Vehicle	Day 85	Nerve, Femoral, Distal, Left, Proximal - Nerve Median, Proximal, Left, Proximal	-1.20005E-06	1.69713E-06	-1.64481E-05	1.4048E-05	1	-1	0.5
Vehicle	Day 85	Nerve, Femoral, Distal, Left, Proximal - Nerve Median, Proximal, Right, Distal	4.46328E-05	6.31203E-05	-0.00052248	0.000611746	1	1	0.5
Vehicle	Day 85	Nerve, Femoral, Distal, Left, Proximal - Nerve Median, Proximal, Right, Proximal	4.46328E-05	6.31203E-05	-0.00052248	0.000611746	1	1	0.5
Vehicle	Day 85	Nerve, Femoral, Distal, Left, Proximal - Nerve, Sciatic, Distal, Left, Distal	-6.35641E-05	0.000216134	-0.002005449	0.001878321	1	-0.415914863	0.749076088
Vehicle	Day 85	Nerve, Femoral, Distal, Left, Proximal - Nerve, Sciatic, Distal, Left, Proximal	4.46328E-05	6.31203E-05	-0.00052248	0.000611746	1	1	0.5
Vehicle	Day 85	Nerve, Femoral, Distal, Left, Proximal - Nerve, Sciatic, Distal, Right, Distal	4.46328E-05	6.31203E-05	-0.00052248	0.000611746	1	1	0.5
Vehicle	Day 85	Nerve, Femoral, Distal, Left, Proximal - Nerve, Sciatic, Distal, Right, Proximal	4.46328E-05	6.31203E-05	-0.00052248	0.000611746	1	1	0.5
Vehicle	Day 85	Nerve, Femoral, Distal, Left, Proximal - Nerve, Sciatic, Proximal, Left, Distal	4.46328E-05	6.31203E-05	-0.00052248	0.000611746	1	1	0.5
Vehicle	Day 85	Nerve, Femoral, Distal, Left, Proximal - Nerve, Sciatic, Proximal, Left, Proximal	4.46328E-05	6.31203E-05	-0.00052248	0.000611746	1	1	0.5
Vehicle	Day 85	Nerve, Femoral, Distal, Left, Proximal - Nerve, Sciatic, Proximal, Right, Distal	-0.00011936	0.000295042	-0.002770204	0.002531483	1	-0.572126302	0.669166569
Vehicle	Day 85	Nerve, Femoral, Distal, Left, Proximal - Nerve, Sciatic, Proximal, Right, Proximal	-9.38549E-05	0.000132731	-0.001286394	0.001098684	1	-1	0.5
Vehicle	Day 85	Nerve, Femoral, Distal, Left, Proximal - Nerve, Ulnar, Distal, Left, Distal	4.46328E-05	6.31203E-05	-0.00052248	0.000611746	1	1	0.5
Vehicle	Day 85	Nerve, Femoral, Distal, Left, Proximal - Nerve, Ulnar, Distal, Left, Proximal	4.46328E-05	6.31203E-05	-0.00052248	0.000611746	1	1	0.5
Vehicle	Day 85	Nerve, Femoral, Distal, Left, Proximal - Nerve, Ulnar, Distal, Right, Distal	4.46328E-05	6.31203E-05	-0.00052248	0.000611746	1	1	0.5
Vehicle	Day 85	Nerve, Femoral, Distal, Left, Proximal - Nerve, Ulnar, Distal, Right, Proximal	4.46328E-05	6.31203E-05	-0.00052248	0.000611746	1	1	0.5
Vehicle	Day 85	Nerve, Femoral, Distal, Left, Proximal - Nerve, Ulnar, Proximal, Left, Distal	4.46328E-05	6.31203E-05	-0.00052248	0.000611746	1	1	0.5
Vehicle	Day 85	Nerve, Femoral, Distal, Left, Proximal - Nerve, Ulnar, Proximal, Left, Proximal	4.46328E-05	6.31203E-05	-0.00052248	0.000611746	1	1	0.5
Vehicle	Day 85	Nerve, Femoral, Distal, Left, Proximal - Nerve, Ulnar, Proximal, Right, Distal	4.46328E-05	6.31203E-05	-0.00052248	0.000611746	1	1	0.5
Vehicle	Day 85	Nerve, Femoral, Distal, Left, Proximal - Nerve, Ulnar, Proximal, Right, Proximal	4.46328E-05	6.31203E-05	-0.00052248	0.000611746	1	1	0.5
Vehicle	Day 85	Nerve, Femoral, Distal, Left, Proximal - Nerve, Optic	4.46328E-05	6.31203E-05	-0.00052248	0.000611746	1	1	0.5
Vehicle	Day 85	Nerve, Femoral, Distal, Left, Proximal - Ovary	0				0		
Vehicle	Day 85	Nerve, Femoral, Distal, Left, Proximal - Spinal Cord, Cervical	4.46328E-05	6.31203E-05	-0.00052248	0.000611746	1	1	0.5
Vehicle	Day 85	Nerve, Femoral, Distal, Left, Proximal - Spinal Cord, Lumbar	4.46328E-05	6.31203E-05	-0.00052248	0.000611746	1	1	0.5
Vehicle	Day 85	Nerve, Femoral, Distal, Left, Proximal - Spinal Cord, Sacral	4.46328E-05	6.31203E-05	-0.00052248	0.000611746	1	1	0.5
Vehicle	Day 85	Nerve, Femoral, Distal, Left, Proximal - Spinal Cord, Spinal Roots	4.46328E-05	6.31203E-05	-0.00052248	0.000611746	1	1	0.5
Vehicle	Day 85	Nerve, Femoral, Distal, Left, Proximal - Spinal Cord, Thoracic	4.46328E-05	6.31203E-05	-0.00052248	0.000611746	1	1	0.5
Vehicle	Day 85	Nerve, Femoral, Distal, Left, Proximal - Spleen	-8.15797E-05	0.000241612	-0.002252374	0.002089215	1	-0.477506447	0.716391376
Vehicle	Day 85	Nerve, Femoral, Distal, Left, Proximal - Testis	8.92655E-05				0		
Vehicle	Day 85	Nerve, Femoral, Distal, Right, Distal - Nerve, Femoral, Distal, Right, Proximal	0	0	0	0	1		
Vehicle	Day 85	Nerve, Femoral, Distal, Right, Distal - Nerve, Femoral, Proximal, Left, Distal	0	0	0	0	1		
Vehicle	Day 85	Nerve, Femoral, Distal, Right, Distal - Nerve, Femoral, Proximal, Left, Proximal	0	0	0	0	1		
Vehicle	Day 85	Nerve, Femoral, Distal, Right, Distal - Nerve, Femoral, Proximal, Right, Distal	-8.56071E-05	0.000121067	-0.001173348	0.001002134	1	-1	0.5
Vehicle	Day 85	Nerve, Femoral, Distal, Right, Distal - Nerve, Femoral, Proximal, Right, Proximal	0	0	0	0	1		
Vehicle	Day 85	Nerve, Femoral, Distal, Right, Distal - Nerve Median, Distal, Left, Distal	0	0	0	0	1		
Vehicle	Day 85	Nerve, Femoral, Distal, Right, Distal - Nerve Median, Distal, Left, Proximal	0	0	0	0	1		
Vehicle	Day 85	Nerve, Femoral, Distal, Right, Distal - Nerve Median, Distal, Right, Distal	0	0	0	0	1		
Vehicle	Day 85	Nerve, Femoral, Distal, Right, Distal - Nerve Median, Distal, Right, Proximal	0	0	0	0	1		
Vehicle	Day 85	Nerve, Femoral, Distal, Right, Distal - Nerve Median, Proximal, Left, Distal	0	0	0	0	1		
Vehicle	Day 85	Nerve, Femoral, Distal, Right, Distal - Nerve Median, Proximal, Left, Proximal	-4.58328E-05	6.48174E-05	-0.000628194	0.000536528	1	-1	0.5
Vehicle	Day 85	Nerve, Femoral, Distal, Right, Distal - Nerve Median, Proximal, Right, Distal	0	0	0	0	1		
Vehicle	Day 85	Nerve, Femoral, Distal, Right, Distal - Nerve Median, Proximal, Right, Proximal	0	0	0	0	1		
Vehicle	Day 85	Nerve, Femoral, Distal, Right, Distal - Nerve, Sciatic, Distal, Left, Distal	-0.000108197	0.000153014	-0.001482969	0.001266575	1	-1	0.5
Vehicle	Day 85	Nerve, Femoral, Distal, Right, Distal - Nerve, Sciatic, Distal, Left, Proximal	0	0	0	0	1		
Vehicle	Day 85	Nerve, Femoral, Distal, Right, Distal - Nerve, Sciatic, Distal, Right, Distal	0	0	0	0	1		
Vehicle	Day 85	Nerve, Femoral, Distal, Right, Distal - Nerve, Sciatic, Distal, Right, Proximal	0	0	0	0	1		
Vehicle	Day 85	Nerve, Femoral, Distal, Right, Distal - Nerve, Sciatic, Proximal, Left, Distal	0	0	0	0	1		
Vehicle	Day 85	Nerve, Femoral, Distal, Right, Distal - Nerve, Sciatic, Proximal, Left, Proximal	0	0	0	0	1		
Vehicle	Day 85	Nerve, Femoral, Distal, Right, Distal - Nerve, Sciatic, Proximal, Right, Distal	-0.000163993	0.000231921	-0.002247723	0.001919737	1	-1	0.5
Vehicle	Day 85	Nerve, Femoral, Distal, Right, Distal - Nerve, Sciatic, Proximal, Right, Proximal	-0.000138488	0.000195851	-0.00189814	0.001621165	1	-1	0.5
Vehicle	Day 85	Nerve, Femoral, Distal, Right, Distal - Nerve, Ulnar, Distal, Left, Distal	0	0	0	0	1		
Vehicle	Day 85	Nerve, Femoral, Distal, Right, Distal - Nerve, Ulnar, Distal, Left, Proximal	0	0	0	0	1		
Vehicle	Day 85	Nerve, Femoral, Distal, Right, Distal - Nerve, Ulnar, Distal, Right, Distal	0	0	0	0	1		
Vehicle	Day 85	Nerve, Femoral, Distal, Right, Distal - Nerve, Ulnar, Distal, Right, Proximal	0	0	0	0	1		
Vehicle	Day 85	Nerve, Femoral, Distal, Right, Distal - Nerve, Ulnar, Proximal, Left, Distal	0	0	0	0	1		
Vehicle	Day 85	Nerve, Femoral, Distal, Right, Distal - Nerve, Ulnar, Proximal, Left, Proximal	0	0	0	0	1		
Vehicle	Day 85	Nerve, Femoral, Distal, Right, Distal - Nerve, Ulnar, Proximal, Right, Distal	0	0	0	0	1		
Vehicle	Day 85	Nerve, Femoral, Distal, Right, Distal - Nerve, Ulnar, Proximal, Right, Proximal	0	0	0	0	1		
Vehicle	Day 85	Nerve, Femoral, Distal, Right, Distal - Nerve, Optic	0	0	0	0	1		
Vehicle	Day 85	Nerve, Femoral, Distal, Right, Distal - Ovary	0				0		

Results of Evaluating Mean Differences Between Different Tissues Across Timepoint and Group for Log10 AAV2-ITR Target Copies/ug of DNA for Normalized Data

Group	Timepoint	Difference	Mean	StdDev	LowerCLMean	UpperCLMean	DF	tValue	P-Value
Vehicle	Day 85	Nerve, Femoral, Distal, Right, Distal - Spinal Cord, Cervical	0	0	0	0	1		
Vehicle	Day 85	Nerve, Femoral, Distal, Right, Distal - Spinal Cord, Lumbar	0	0	0	0	1		
Vehicle	Day 85	Nerve, Femoral, Distal, Right, Distal - Spinal Cord, Sacral	0	0	0	0	1		
Vehicle	Day 85	Nerve, Femoral, Distal, Right, Distal - Spinal Cord, Spinal Roots	0	0	0	0	1		
Vehicle	Day 85	Nerve, Femoral, Distal, Right, Distal - Spinal Cord, Thoracic	0	0	0	0	1		
Vehicle	Day 85	Nerve, Femoral, Distal, Right, Distal - Spleen	-0.000126212	0.000178491	-0.001729894	0.001477469	1	-1	0.5
Vehicle	Day 85	Nerve, Femoral, Distal, Right, Distal - Testis	0				0		
Vehicle	Day 85	Nerve, Femoral, Distal, Right, Proximal - Nerve, Femoral, Proximal, Left, Distal	0	0	0	0	1		
Vehicle	Day 85	Nerve, Femoral, Distal, Right, Proximal - Nerve, Femoral, Proximal, Left, Proximal	0	0	0	0	1		
Vehicle	Day 85	Nerve, Femoral, Distal, Right, Proximal - Nerve, Femoral, Proximal, Right, Distal	-8.56071E-05	0.000121067	-0.001173348	0.001002134	1	-1	0.5
Vehicle	Day 85	Nerve, Femoral, Distal, Right, Proximal - Nerve, Femoral, Proximal, Right, Proximal	0	0	0	0	1		
Vehicle	Day 85	Nerve, Femoral, Distal, Right, Proximal - Nerve Median, Distal, Left, Distal	0	0	0	0	1		
Vehicle	Day 85	Nerve, Femoral, Distal, Right, Proximal - Nerve Median, Distal, Left, Proximal	0	0	0	0	1		
Vehicle	Day 85	Nerve, Femoral, Distal, Right, Proximal - Nerve Median, Distal, Right, Distal	0	0	0	0	1		
Vehicle	Day 85	Nerve, Femoral, Distal, Right, Proximal - Nerve Median, Distal, Right, Proximal	0	0	0	0	1		
Vehicle	Day 85	Nerve, Femoral, Distal, Right, Proximal - Nerve Median, Proximal, Left, Distal	0	0	0	0	1		
Vehicle	Day 85	Nerve, Femoral, Distal, Right, Proximal - Nerve Median, Proximal, Left, Proximal	-4.58328E-05	6.48174E-05	-0.000628194	0.000536528	1	-1	0.5
Vehicle	Day 85	Nerve, Femoral, Distal, Right, Proximal - Nerve Median, Proximal, Right, Distal	0	0	0	0	1		
Vehicle	Day 85	Nerve, Femoral, Distal, Right, Proximal - Nerve Median, Proximal, Right, Proximal	0	0	0	0	1		
Vehicle	Day 85	Nerve, Femoral, Distal, Right, Proximal - Nerve, Sciatic, Distal, Left, Distal	-0.000108197	0.000153014	-0.001482969	0.001266575	1	-1	0.5
Vehicle	Day 85	Nerve, Femoral, Distal, Right, Proximal - Nerve, Sciatic, Distal, Left, Proximal	0	0	0	0	1		
Vehicle	Day 85	Nerve, Femoral, Distal, Right, Proximal - Nerve, Sciatic, Distal, Right, Distal	0	0	0	0	1		
Vehicle	Day 85	Nerve, Femoral, Distal, Right, Proximal - Nerve, Sciatic, Distal, Right, Proximal	0	0	0	0	1		
Vehicle	Day 85	Nerve, Femoral, Distal, Right, Proximal - Nerve, Sciatic, Proximal, Left, Distal	0	0	0	0	1		
Vehicle	Day 85	Nerve, Femoral, Distal, Right, Proximal - Nerve, Sciatic, Proximal, Left, Proximal	0	0	0	0	1		
Vehicle	Day 85	Nerve, Femoral, Distal, Right, Proximal - Nerve, Sciatic, Proximal, Right, Distal	-0.000163993	0.000231921	-0.002247723	0.001919737	1	-1	0.5
Vehicle	Day 85	Nerve, Femoral, Distal, Right, Proximal - Nerve, Sciatic, Proximal, Right, Proximal	-0.000138488	0.000195851	-0.00189814	0.001621165	1	-1	0.5
Vehicle	Day 85	Nerve, Femoral, Distal, Right, Proximal - Nerve, Ulnar, Distal, Left, Distal	0	0	0	0	1		
Vehicle	Day 85	Nerve, Femoral, Distal, Right, Proximal - Nerve, Ulnar, Distal, Left, Proximal	0	0	0	0	1		
Vehicle	Day 85	Nerve, Femoral, Distal, Right, Proximal - Nerve, Ulnar, Distal, Right, Distal	0	0	0	0	1		
Vehicle	Day 85	Nerve, Femoral, Distal, Right, Proximal - Nerve, Ulnar, Distal, Right, Proximal	0	0	0	0	1		
Vehicle	Day 85	Nerve, Femoral, Distal, Right, Proximal - Nerve, Ulnar, Proximal, Left, Distal	0	0	0	0	1		
Vehicle	Day 85	Nerve, Femoral, Distal, Right, Proximal - Nerve, Ulnar, Proximal, Left, Proximal	0	0	0	0	1		
Vehicle	Day 85	Nerve, Femoral, Distal, Right, Proximal - Nerve, Ulnar, Proximal, Right, Distal	0	0	0	0	1		
Vehicle	Day 85	Nerve, Femoral, Distal, Right, Proximal - Nerve, Ulnar, Proximal, Right, Proximal	0	0	0	0	1		
Vehicle	Day 85	Nerve, Femoral, Distal, Right, Proximal - Nerve, Optic	0	0	0	0	1		
Vehicle	Day 85	Nerve, Femoral, Distal, Right, Proximal - Ovary	0				0		
Vehicle	Day 85	Nerve, Femoral, Distal, Right, Proximal - Spinal Cord, Cervical	0	0	0	0	1		
Vehicle	Day 85	Nerve, Femoral, Distal, Right, Proximal - Spinal Cord, Lumbar	0	0	0	0	1		
Vehicle	Day 85	Nerve, Femoral, Distal, Right, Proximal - Spinal Cord, Sacral	0	0	0	0	1		
Vehicle	Day 85	Nerve, Femoral, Distal, Right, Proximal - Spinal Cord, Spinal Roots	0	0	0	0	1		
Vehicle	Day 85	Nerve, Femoral, Distal, Right, Proximal - Spinal Cord, Thoracic	0	0	0	0	1		
Vehicle	Day 85	Nerve, Femoral, Distal, Right, Proximal - Spleen	-0.000126212	0.000178491	-0.001729894	0.001477469	1	-1	0.5
Vehicle	Day 85	Nerve, Femoral, Distal, Right, Proximal - Testis	0				0		
Vehicle	Day 85	Nerve, Femoral, Proximal, Left, Distal - Nerve, Femoral, Proximal, Left, Proximal	0	0	0	0	1		
Vehicle	Day 85	Nerve, Femoral, Proximal, Left, Distal - Nerve, Femoral, Proximal, Right, Distal	-8.56071E-05	0.000121067	-0.001173348	0.001002134	1	-1	0.5
Vehicle	Day 85	Nerve, Femoral, Proximal, Left, Distal - Nerve, Femoral, Proximal, Right, Proximal	0	0	0	0	1		
Vehicle	Day 85	Nerve, Femoral, Proximal, Left, Distal - Nerve Median, Distal, Left, Distal	0	0	0	0	1		
Vehicle	Day 85	Nerve, Femoral, Proximal, Left, Distal - Nerve Median, Distal, Left, Proximal	0	0	0	0	1		
Vehicle	Day 85	Nerve, Femoral, Proximal, Left, Distal - Nerve Median, Distal, Right, Distal	0	0	0	0	1		
Vehicle	Day 85	Nerve, Femoral, Proximal, Left, Distal - Nerve Median, Distal, Right, Proximal	0	0	0	0	1		
Vehicle	Day 85	Nerve, Femoral, Proximal, Left, Distal - Nerve Median, Proximal, Left, Distal	0	0	0	0	1		
Vehicle	Day 85	Nerve, Femoral, Proximal, Left, Distal - Nerve Median, Proximal, Left, Proximal	-4.58328E-05	6.48174E-05	-0.000628194	0.000536528	1	-1	0.5
Vehicle	Day 85	Nerve, Femoral, Proximal, Left, Distal - Nerve Median, Proximal, Right, Distal	0	0	0	0	1		
Vehicle	Day 85	Nerve, Femoral, Proximal, Left, Distal - Nerve Median, Proximal, Right, Proximal	0	0	0	0	1		
Vehicle	Day 85	Nerve, Femoral, Proximal, Left, Distal - Nerve, Sciatic, Distal, Left, Distal	-0.000108197	0.000153014	-0.001482969	0.001266575	1	-1	0.5
Vehicle	Day 85	Nerve, Femoral, Proximal, Left, Distal - Nerve, Sciatic, Distal, Left, Proximal	0	0	0	0	1		
Vehicle	Day 85	Nerve, Femoral, Proximal, Left, Distal - Nerve, Sciatic, Distal, Right, Distal	0	0	0	0	1		
Vehicle	Day 85	Nerve, Femoral, Proximal, Left, Distal - Nerve, Sciatic, Distal, Right, Proximal	0	0	0	0	1		
Vehicle	Day 85	Nerve, Femoral, Proximal, Left, Distal - Nerve, Sciatic, Proximal, Left, Distal	0	0	0	0	1		
Vehicle	Day 85	Nerve, Femoral, Proximal, Left, Distal - Nerve, Sciatic, Proximal, Left, Proximal	0	0	0	0	1		
Vehicle	Day 85	Nerve, Femoral, Proximal, Left, Distal - Nerve, Sciatic, Proximal, Right, Distal	-0.000163993	0.000231921	-0.002247723	0.001919737	1	-1	0.5
Vehicle	Day 85	Nerve, Femoral, Proximal, Left, Distal - Nerve, Sciatic, Proximal, Right, Proximal	-0.000138488	0.000195851	-0.00189814	0.001621165	1	-1	0.5
Vehicle	Day 85	Nerve, Femoral, Proximal, Left, Distal - Nerve, Ulnar, Distal, Left, Distal	0	0	0	0	1		
Vehicle	Day 85	Nerve, Femoral, Proximal, Left, Distal - Nerve, Ulnar, Distal, Left, Proximal	0	0	0	0	1		
Vehicle	Day 85	Nerve, Femoral, Proximal, Left, Distal - Nerve, Ulnar, Distal, Right, Distal	0	0	0	0	1		
Vehicle	Day 85	Nerve, Femoral, Proximal, Left, Distal - Nerve, Ulnar, Distal, Right, Proximal	0	0	0	0	1		
Vehicle	Day 85	Nerve, Femoral, Proximal, Left, Distal - Nerve, Ulnar, Proximal, Left, Distal	0	0	0	0	1		
Vehicle	Day 85	Nerve, Femoral, Proximal, Left, Distal - Nerve, Ulnar, Proximal, Left, Proximal	0	0	0	0	1		
Vehicle	Day 85	Nerve, Femoral, Proximal, Left, Distal - Nerve, Ulnar, Proximal, Right, Distal	0	0	0	0	1		
Vehicle	Day 85	Nerve, Femoral, Proximal, Left, Distal - Nerve, Ulnar, Proximal, Right, Proximal	0	0	0	0	1		
Vehicle	Day 85	Nerve, Femoral, Proximal, Left, Distal - Nerve, Optic	0	0	0	0	1		
Vehicle	Day 85	Nerve, Femoral, Proximal, Left, Distal - Ovary	0				0		
Vehicle	Day 85	Nerve, Femoral, Proximal, Left, Distal - Spinal Cord, Cervical	0	0	0	0	1		
Vehicle	Day 85	Nerve, Femoral, Proximal, Left, Distal - Spinal Cord, Lumbar	0	0	0	0	1		
Vehicle	Day 85	Nerve, Femoral, Proximal, Left, Distal - Spinal Cord, Sacral	0	0	0	0	1		
Vehicle	Day 85	Nerve, Femoral, Proximal, Left, Distal - Spinal Cord, Spinal Roots	0	0	0	0	1		
Vehicle	Day 85	Nerve, Femoral, Proximal, Left, Distal - Spinal Cord, Thoracic	0	0	0	0	1		
Vehicle	Day 85	Nerve, Femoral, Proximal, Left, Distal - Spleen	-0.000126212	0.000178491	-0.001729894	0.001477469	1	-1	0.5
Vehicle	Day 85	Nerve, Femoral, Proximal, Left, Distal - Testis	0				0		
Vehicle	Day 85	Nerve, Femoral, Proximal, Left, Proximal - Nerve, Femoral, Proximal, Right, Distal	-8.56071E-05	0.000121067	-0.001173348	0.001002134	1	-1	0.5
Vehicle	Day 85	Nerve, Femoral, Proximal, Left, Proximal - Nerve, Femoral, Proximal, Right, Proximal	0	0	0	0	1		
Vehicle	Day 85	Nerve, Femoral, Proximal, Left, Proximal - Nerve Median, Distal, Left, Distal	0	0	0	0	1		
Vehicle	Day 85	Nerve, Femoral, Proximal, Left, Proximal - Nerve Median, Distal, Left, Proximal	0	0	0	0	1		
Vehicle	Day 85	Nerve, Femoral, Proximal, Left, Proximal - Nerve Median, Distal, Right, Distal	0	0	0	0	1		
Vehicle	Day 85	Nerve, Femoral, Proximal, Left, Proximal - Nerve Median, Distal, Right, Proximal	0	0	0	0	1		
Vehicle	Day 85	Nerve, Femoral, Proximal, Left, Proximal - Nerve Median, Proximal, Left, Distal	0	0	0	0	1		
Vehicle	Day 85	Nerve, Femoral, Proximal, Left, Proximal - Nerve Median, Proximal, Left, Proximal	-4.58328E-05	6.48174E-05	-0.000628194	0.000536528	1	-1	0.5
Vehicle	Day 85	Nerve, Femoral, Proximal, Left, Proximal - Nerve Median, Proximal, Right, Distal	0	0	0	0	1		
Vehicle	Day 85	Nerve, Femoral, Proximal, Left, Proximal - Nerve Median, Proximal, Right, Proximal	0	0	0	0	1		
Vehicle	Day 85	Nerve, Femoral, Proximal, Left, Proximal - Nerve, Sciatic, Distal, Left, Distal	-0.000108197	0.000153014	-0.001482969	0.001266575	1	-1	0.5
Vehicle	Day 85	Nerve, Femoral, Proximal, Left, Proximal - Nerve, Sciatic, Distal, Left, Proximal	0	0	0	0	1		
Vehicle	Day 85	Nerve, Femoral, Proximal, Left, Proximal - Nerve, Sciatic, Distal, Right, Distal	0	0	0	0	1		

Results of Evaluating Mean Differences Between Different Tissues Across Timepoint and Group for Log10 AAV2-ITR Target Copies/ug of DNA for Normalized Data

Group	Timepoint	Difference	Mean	StdDev	LowerCLMean	UpperCLMean	DF	tValue	P-Value
Vehicle	Day 85	Nerve, Femoral, Proximal, Left, Proximal - Nerve, Sciatic, Distal, Right, Proximal	0	0	0	0	1		
Vehicle	Day 85	Nerve, Femoral, Proximal, Left, Proximal - Nerve, Sciatic, Proximal, Left, Distal	0	0	0	0	1		
Vehicle	Day 85	Nerve, Femoral, Proximal, Left, Proximal - Nerve, Sciatic, Proximal, Left, Proximal	0	0	0	0	1		
Vehicle	Day 85	Nerve, Femoral, Proximal, Left, Proximal - Nerve, Sciatic, Proximal, Right, Distal	-0.000163993	0.000231921	-0.002247723	0.001919737	1	-1	0.5
Vehicle	Day 85	Nerve, Femoral, Proximal, Left, Proximal - Nerve, Sciatic, Proximal, Right, Proximal	-0.000138488	0.000195851	-0.00189814	0.001621165	1	-1	0.5
Vehicle	Day 85	Nerve, Femoral, Proximal, Left, Proximal - Nerve, Ulnar, Distal, Left, Distal	0	0	0	0	1		
Vehicle	Day 85	Nerve, Femoral, Proximal, Left, Proximal - Nerve, Ulnar, Distal, Left, Proximal	0	0	0	0	1		
Vehicle	Day 85	Nerve, Femoral, Proximal, Left, Proximal - Nerve, Ulnar, Distal, Right, Distal	0	0	0	0	1		
Vehicle	Day 85	Nerve, Femoral, Proximal, Left, Proximal - Nerve, Ulnar, Distal, Right, Proximal	0	0	0	0	1		
Vehicle	Day 85	Nerve, Femoral, Proximal, Left, Proximal - Nerve, Ulnar, Proximal, Left, Distal	0	0	0	0	1		
Vehicle	Day 85	Nerve, Femoral, Proximal, Left, Proximal - Nerve, Ulnar, Proximal, Left, Proximal	0	0	0	0	1		
Vehicle	Day 85	Nerve, Femoral, Proximal, Left, Proximal - Nerve, Ulnar, Proximal, Right, Distal	0	0	0	0	1		
Vehicle	Day 85	Nerve, Femoral, Proximal, Left, Proximal - Nerve, Ulnar, Proximal, Right, Proximal	0	0	0	0	1		
Vehicle	Day 85	Nerve, Femoral, Proximal, Left, Proximal - Nerve, Optic	0	0	0	0	1		
Vehicle	Day 85	Nerve, Femoral, Proximal, Left, Proximal - Ovary	0				0		
Vehicle	Day 85	Nerve, Femoral, Proximal, Left, Proximal - Spinal Cord, Cervical	0	0	0	0	1		
Vehicle	Day 85	Nerve, Femoral, Proximal, Left, Proximal - Spinal Cord, Lumbar	0	0	0	0	1		
Vehicle	Day 85	Nerve, Femoral, Proximal, Left, Proximal - Spinal Cord, Sacral	0	0	0	0	1		
Vehicle	Day 85	Nerve, Femoral, Proximal, Left, Proximal - Spinal Cord, Spinal Roots	0	0	0	0	1		
Vehicle	Day 85	Nerve, Femoral, Proximal, Left, Proximal - Spinal Cord, Thoracic	0	0	0	0	1		
Vehicle	Day 85	Nerve, Femoral, Proximal, Left, Proximal - Spleen	-0.000126212	0.000178491	-0.001729894	0.001477469	1	-1	0.5
Vehicle	Day 85	Nerve, Femoral, Proximal, Left, Proximal - Testis	0				0		
Vehicle	Day 85	Nerve, Femoral, Proximal, Right, Distal - Nerve, Femoral, Proximal, Right, Proximal	8.56071E-05	0.000121067	-0.001002134	0.001173348	1	1	0.5
Vehicle	Day 85	Nerve, Femoral, Proximal, Right, Distal - Nerve Median, Distal, Left, Distal	8.56071E-05	0.000121067	-0.001002134	0.001173348	1	1	0.5
Vehicle	Day 85	Nerve, Femoral, Proximal, Right, Distal - Nerve Median, Distal, Left, Proximal	8.56071E-05	0.000121067	-0.001002134	0.001173348	1	1	0.5
Vehicle	Day 85	Nerve, Femoral, Proximal, Right, Distal - Nerve Median, Distal, Right, Distal	8.56071E-05	0.000121067	-0.001002134	0.001173348	1	1	0.5
Vehicle	Day 85	Nerve, Femoral, Proximal, Right, Distal - Nerve Median, Distal, Right, Proximal	8.56071E-05	0.000121067	-0.001002134	0.001173348	1	1	0.5
Vehicle	Day 85	Nerve, Femoral, Proximal, Right, Distal - Nerve Median, Proximal, Left, Distal	8.56071E-05	0.000121067	-0.001002134	0.001173348	1	1	0.5
Vehicle	Day 85	Nerve, Femoral, Proximal, Right, Distal - Nerve Median, Proximal, Left, Proximal	3.97743E-05	5.62493E-05	-0.000465606	0.000545154	1	1	0.5
Vehicle	Day 85	Nerve, Femoral, Proximal, Right, Distal - Nerve Median, Proximal, Right, Distal	8.56071E-05	0.000121067	-0.001002134	0.001173348	1	1	0.5
Vehicle	Day 85	Nerve, Femoral, Proximal, Right, Distal - Nerve Median, Proximal, Right, Proximal	8.56071E-05	0.000121067	-0.001002134	0.001173348	1	1	0.5
Vehicle	Day 85	Nerve, Femoral, Proximal, Right, Distal - Nerve, Sciatic, Distal, Left, Distal	-2.25898E-05	0.00027408	-0.002485103	0.002439923	1	-0.116560045	0.926128911
Vehicle	Day 85	Nerve, Femoral, Proximal, Right, Distal - Nerve, Sciatic, Distal, Left, Proximal	8.56071E-05	0.000121067	-0.001002134	0.001173348	1	1	0.5
Vehicle	Day 85	Nerve, Femoral, Proximal, Right, Distal - Nerve, Sciatic, Distal, Right, Distal	8.56071E-05	0.000121067	-0.001002134	0.001173348	1	1	0.5
Vehicle	Day 85	Nerve, Femoral, Proximal, Right, Distal - Nerve, Sciatic, Distal, Right, Proximal	8.56071E-05	0.000121067	-0.001002134	0.001173348	1	1	0.5
Vehicle	Day 85	Nerve, Femoral, Proximal, Right, Distal - Nerve, Sciatic, Proximal, Left, Distal	8.56071E-05	0.000121067	-0.001002134	0.001173348	1	1	0.5
Vehicle	Day 85	Nerve, Femoral, Proximal, Right, Distal - Nerve, Sciatic, Proximal, Left, Proximal	8.56071E-05	0.000121067	-0.001002134	0.001173348	1	1	0.5
Vehicle	Day 85	Nerve, Femoral, Proximal, Right, Distal - Nerve, Sciatic, Proximal, Right, Distal	-7.8386E-05	0.000352988	-0.003249857	0.003093085	1	-0.31404634	0.806281052
Vehicle	Day 85	Nerve, Femoral, Proximal, Right, Distal - Nerve, Sciatic, Proximal, Right, Proximal	-5.28806E-05	7.47844E-05	-0.000724792	0.000619031	1	-1	0.5
Vehicle	Day 85	Nerve, Femoral, Proximal, Right, Distal - Nerve, Ulnar, Distal, Left, Distal	8.56071E-05	0.000121067	-0.001002134	0.001173348	1	1	0.5
Vehicle	Day 85	Nerve, Femoral, Proximal, Right, Distal - Nerve, Ulnar, Distal, Left, Proximal	8.56071E-05	0.000121067	-0.001002134	0.001173348	1	1	0.5
Vehicle	Day 85	Nerve, Femoral, Proximal, Right, Distal - Nerve, Ulnar, Distal, Right, Distal	8.56071E-05	0.000121067	-0.001002134	0.001173348	1	1	0.5
Vehicle	Day 85	Nerve, Femoral, Proximal, Right, Distal - Nerve, Ulnar, Distal, Right, Proximal	8.56071E-05	0.000121067	-0.001002134	0.001173348	1	1	0.5
Vehicle	Day 85	Nerve, Femoral, Proximal, Right, Distal - Nerve, Ulnar, Proximal, Left, Distal	8.56071E-05	0.000121067	-0.001002134	0.001173348	1	1	0.5
Vehicle	Day 85	Nerve, Femoral, Proximal, Right, Distal - Nerve, Ulnar, Proximal, Left, Proximal	8.56071E-05	0.000121067	-0.001002134	0.001173348	1	1	0.5
Vehicle	Day 85	Nerve, Femoral, Proximal, Right, Distal - Nerve, Ulnar, Proximal, Right, Distal	8.56071E-05	0.000121067	-0.001002134	0.001173348	1	1	0.5
Vehicle	Day 85	Nerve, Femoral, Proximal, Right, Distal - Nerve, Ulnar, Proximal, Right, Proximal	8.56071E-05	0.000121067	-0.001002134	0.001173348	1	1	0.5
Vehicle	Day 85	Nerve, Femoral, Proximal, Right, Distal - Nerve, Optic	8.56071E-05	0.000121067	-0.001002134	0.001173348	1	1	0.5
Vehicle	Day 85	Nerve, Femoral, Proximal, Right, Distal - Ovary	0				0		
Vehicle	Day 85	Nerve, Femoral, Proximal, Right, Distal - Spinal Cord, Cervical	8.56071E-05	0.000121067	-0.001002134	0.001173348	1	1	0.5
Vehicle	Day 85	Nerve, Femoral, Proximal, Right, Distal - Spinal Cord, Lumbar	8.56071E-05	0.000121067	-0.001002134	0.001173348	1	1	0.5
Vehicle	Day 85	Nerve, Femoral, Proximal, Right, Distal - Spinal Cord, Sacral	8.56071E-05	0.000121067	-0.001002134	0.001173348	1	1	0.5
Vehicle	Day 85	Nerve, Femoral, Proximal, Right, Distal - Spinal Cord, Spinal Roots	8.56071E-05	0.000121067	-0.001002134	0.001173348	1	1	0.5
Vehicle	Day 85	Nerve, Femoral, Proximal, Right, Distal - Spinal Cord, Thoracic	8.56071E-05	0.000121067	-0.001002134	0.001173348	1	1	0.5
Vehicle	Day 85	Nerve, Femoral, Proximal, Right, Distal - Spleen	-4.06054E-05	0.000299558	-0.002732028	0.002650817	1	-0.191697947	0.879424074
Vehicle	Day 85	Nerve, Femoral, Proximal, Right, Distal - Testis	0.000171214				0		
Vehicle	Day 85	Nerve, Femoral, Proximal, Right, Proximal - Nerve Median, Distal, Left, Distal	0	0	0	0	1		
Vehicle	Day 85	Nerve, Femoral, Proximal, Right, Proximal - Nerve Median, Distal, Left, Proximal	0	0	0	0	1		
Vehicle	Day 85	Nerve, Femoral, Proximal, Right, Proximal - Nerve Median, Distal, Right, Distal	0	0	0	0	1		
Vehicle	Day 85	Nerve, Femoral, Proximal, Right, Proximal - Nerve Median, Distal, Right, Proximal	0	0	0	0	1		
Vehicle	Day 85	Nerve, Femoral, Proximal, Right, Proximal - Nerve Median, Proximal, Left, Distal	0	0	0	0	1		
Vehicle	Day 85	Nerve, Femoral, Proximal, Right, Proximal - Nerve Median, Proximal, Left, Proximal	-4.58328E-05	6.48174E-05	-0.000628194	0.000536528	1	-1	0.5
Vehicle	Day 85	Nerve, Femoral, Proximal, Right, Proximal - Nerve Median, Proximal, Right, Distal	0	0	0	0	1		
Vehicle	Day 85	Nerve, Femoral, Proximal, Right, Proximal - Nerve Median, Proximal, Right, Proximal	0	0	0	0	1		
Vehicle	Day 85	Nerve, Femoral, Proximal, Right, Proximal - Nerve, Sciatic, Distal, Left, Distal	-0.000108197	0.000153014	-0.001482969	0.001266575	1	-1	0.5
Vehicle	Day 85	Nerve, Femoral, Proximal, Right, Proximal - Nerve, Sciatic, Distal, Left, Proximal	0	0	0	0	1		
Vehicle	Day 85	Nerve, Femoral, Proximal, Right, Proximal - Nerve, Sciatic, Distal, Right, Distal	0	0	0	0	1		
Vehicle	Day 85	Nerve, Femoral, Proximal, Right, Proximal - Nerve, Sciatic, Distal, Right, Proximal	0	0	0	0	1		
Vehicle	Day 85	Nerve, Femoral, Proximal, Right, Proximal - Nerve, Sciatic, Proximal, Left, Distal	0	0	0	0	1		
Vehicle	Day 85	Nerve, Femoral, Proximal, Right, Proximal - Nerve, Sciatic, Proximal, Left, Proximal	0	0	0	0	1		
Vehicle	Day 85	Nerve, Femoral, Proximal, Right, Proximal - Nerve, Sciatic, Proximal, Right, Distal	-0.000163993	0.000231921	-0.002247723	0.001919737	1	-1	0.5
Vehicle	Day 85	Nerve, Femoral, Proximal, Right, Proximal - Nerve, Sciatic, Proximal, Right, Proximal	-0.000138488	0.000195851	-0.00189814	0.001621165	1	-1	0.5
Vehicle	Day 85	Nerve, Femoral, Proximal, Right, Proximal - Nerve, Ulnar, Distal, Left, Distal	0	0	0	0	1		
Vehicle	Day 85	Nerve, Femoral, Proximal, Right, Proximal - Nerve, Ulnar, Distal, Left, Proximal	0	0	0	0	1		
Vehicle	Day 85	Nerve, Femoral, Proximal, Right, Proximal - Nerve, Ulnar, Distal, Right, Distal	0	0	0	0	1		
Vehicle	Day 85	Nerve, Femoral, Proximal, Right, Proximal - Nerve, Ulnar, Distal, Right, Proximal	0	0	0	0	1		
Vehicle	Day 85	Nerve, Femoral, Proximal, Right, Proximal - Nerve, Ulnar, Proximal, Left, Distal	0	0	0	0	1		
Vehicle	Day 85	Nerve, Femoral, Proximal, Right, Proximal - Nerve, Ulnar, Proximal, Left, Proximal	0	0	0	0	1		
Vehicle	Day 85	Nerve, Femoral, Proximal, Right, Proximal - Nerve, Ulnar, Proximal, Right, Distal	0	0	0	0	1		
Vehicle	Day 85	Nerve, Femoral, Proximal, Right, Proximal - Nerve, Ulnar, Proximal, Right, Proximal	0	0	0	0	1		
Vehicle	Day 85	Nerve, Femoral, Proximal, Right, Proximal - Nerve, Optic	0	0	0	0	1		
Vehicle	Day 85	Nerve, Femoral, Proximal, Right, Proximal - Ovary	0				0		
Vehicle	Day 85	Nerve, Femoral, Proximal, Right, Proximal - Spinal Cord, Cervical	0	0	0	0	1		
Vehicle	Day 85	Nerve, Femoral, Proximal, Right, Proximal - Spinal Cord, Lumbar	0	0	0	0	1		
Vehicle	Day 85	Nerve, Femoral, Proximal, Right, Proximal - Spinal Cord, Sacral	0	0	0	0	1		
Vehicle	Day 85	Nerve, Femoral, Proximal, Right, Proximal - Spinal Cord, Spinal Roots	0	0	0	0	1		
Vehicle	Day 85	Nerve, Femoral, Proximal, Right, Proximal - Spinal Cord, Thoracic	0	0	0	0	1		
Vehicle	Day 85	Nerve, Femoral, Proximal, Right, Proximal - Spleen	-0.000126212	0.000178491	-0.001729894	0.001477469	1	-1	0.5
Vehicle	Day 85	Nerve, Femoral, Proximal, Right, Proximal - Testis	0				0		
Vehicle	Day 85	Nerve Median, Distal, Left, Distal - Nerve Median, Distal, Left, Proximal	0	0	0	0	1		
Vehicle	Day 85	Nerve Median, Distal, Left, Distal - Nerve Median, Distal, Right, Distal	0	0	0	0	1		
Vehicle	Day 85	Nerve Median, Distal, Left, Distal - Nerve Median, Distal, Right, Proximal	0	0	0	0	1		
Vehicle	Day 85	Nerve Median, Distal, Left, Distal - Nerve Median, Proximal, Left, Distal	0	0	0	0	1		

Results of Evaluating Mean Differences Between Different Tissues Across Timepoint and Group for Log10 AAV2-ITR Target Copies/ug of DNA for Normalized Data

Group	Timepoint	Difference	Mean	StdDev	LowerCLMean	UpperCLMean	DF	tValue	P-Value
Vehicle	Day 85	Nerve Median, Distal, Left, Distal - Nerve Median, Proximal, Left, Proximal	-4.58328E-05	6.48174E-05	-0.000628194	0.000536528	1	-1	0.5
Vehicle	Day 85	Nerve Median, Distal, Left, Distal - Nerve Median, Proximal, Right, Distal	0	0	0	0	1		
Vehicle	Day 85	Nerve Median, Distal, Left, Distal - Nerve Median, Proximal, Right, Proximal	0	0	0	0	1		
Vehicle	Day 85	Nerve Median, Distal, Left, Distal - Nerve, Sciatic, Distal, Left, Distal	-0.000108197	0.000153014	-0.001482969	0.001266575	1	-1	0.5
Vehicle	Day 85	Nerve Median, Distal, Left, Distal - Nerve, Sciatic, Distal, Left, Proximal	0	0	0	0	1		
Vehicle	Day 85	Nerve Median, Distal, Left, Distal - Nerve, Sciatic, Distal, Right, Distal	0	0	0	0	1		
Vehicle	Day 85	Nerve Median, Distal, Left, Distal - Nerve, Sciatic, Distal, Right, Proximal	0	0	0	0	1		
Vehicle	Day 85	Nerve Median, Distal, Left, Distal - Nerve, Sciatic, Proximal, Left, Distal	0	0	0	0	1		
Vehicle	Day 85	Nerve Median, Distal, Left, Distal - Nerve, Sciatic, Proximal, Left, Proximal	0	0	0	0	1		
Vehicle	Day 85	Nerve Median, Distal, Left, Distal - Nerve, Sciatic, Proximal, Right, Distal	-0.000163993	0.000231921	-0.002247723	0.001919737	1	-1	0.5
Vehicle	Day 85	Nerve Median, Distal, Left, Distal - Nerve, Sciatic, Proximal, Right, Proximal	-0.000138488	0.000195851	-0.00189814	0.001621165	1	-1	0.5
Vehicle	Day 85	Nerve Median, Distal, Left, Distal - Nerve, Ulnar, Distal, Left, Distal	0	0	0	0	1		
Vehicle	Day 85	Nerve Median, Distal, Left, Distal - Nerve, Ulnar, Distal, Left, Proximal	0	0	0	0	1		
Vehicle	Day 85	Nerve Median, Distal, Left, Distal - Nerve, Ulnar, Distal, Right, Distal	0	0	0	0	1		
Vehicle	Day 85	Nerve Median, Distal, Left, Distal - Nerve, Ulnar, Distal, Right, Proximal	0	0	0	0	1		
Vehicle	Day 85	Nerve Median, Distal, Left, Distal - Nerve, Ulnar, Proximal, Left, Distal	0	0	0	0	1		
Vehicle	Day 85	Nerve Median, Distal, Left, Distal - Nerve, Ulnar, Proximal, Left, Proximal	0	0	0	0	1		
Vehicle	Day 85	Nerve Median, Distal, Left, Distal - Nerve, Ulnar, Proximal, Right, Distal	0	0	0	0	1		
Vehicle	Day 85	Nerve Median, Distal, Left, Distal - Nerve, Ulnar, Proximal, Right, Proximal	0	0	0	0	1		
Vehicle	Day 85	Nerve Median, Distal, Left, Distal - Nerve, Optic	0	0	0	0	1		
Vehicle	Day 85	Nerve Median, Distal, Left, Distal - Ovary	0				0		
Vehicle	Day 85	Nerve Median, Distal, Left, Distal - Spinal Cord, Cervical	0	0	0	0	1		
Vehicle	Day 85	Nerve Median, Distal, Left, Distal - Spinal Cord, Lumbar	0	0	0	0	1		
Vehicle	Day 85	Nerve Median, Distal, Left, Distal - Spinal Cord, Sacral	0	0	0	0	1		
Vehicle	Day 85	Nerve Median, Distal, Left, Distal - Spinal Cord, Spinal Roots	0	0	0	0	1		
Vehicle	Day 85	Nerve Median, Distal, Left, Distal - Spinal Cord, Thoracic	0	0	0	0	1		
Vehicle	Day 85	Nerve Median, Distal, Left, Distal - Spleen	-0.000126212	0.000178491	-0.001729894	0.001477469	1	-1	0.5
Vehicle	Day 85	Nerve Median, Distal, Left, Distal - Testis	0				0		
Vehicle	Day 85	Nerve Median, Distal, Left, Proximal - Nerve Median, Distal, Right, Distal	0	0	0	0	1		
Vehicle	Day 85	Nerve Median, Distal, Left, Proximal - Nerve Median, Distal, Right, Proximal	0	0	0	0	1		
Vehicle	Day 85	Nerve Median, Distal, Left, Proximal - Nerve Median, Proximal, Left, Distal	0	0	0	0	1		
Vehicle	Day 85	Nerve Median, Distal, Left, Proximal - Nerve Median, Proximal, Left, Proximal	-4.58328E-05	6.48174E-05	-0.000628194	0.000536528	1	-1	0.5
Vehicle	Day 85	Nerve Median, Distal, Left, Proximal - Nerve Median, Proximal, Right, Distal	0	0	0	0	1		
Vehicle	Day 85	Nerve Median, Distal, Left, Proximal - Nerve Median, Proximal, Right, Proximal	0	0	0	0	1		
Vehicle	Day 85	Nerve Median, Distal, Left, Proximal - Nerve, Sciatic, Distal, Left, Distal	-0.000108197	0.000153014	-0.001482969	0.001266575	1	-1	0.5
Vehicle	Day 85	Nerve Median, Distal, Left, Proximal - Nerve, Sciatic, Distal, Left, Proximal	0	0	0	0	1		
Vehicle	Day 85	Nerve Median, Distal, Left, Proximal - Nerve, Sciatic, Distal, Right, Distal	0	0	0	0	1		
Vehicle	Day 85	Nerve Median, Distal, Left, Proximal - Nerve, Sciatic, Distal, Right, Proximal	0	0	0	0	1		
Vehicle	Day 85	Nerve Median, Distal, Left, Proximal - Nerve, Sciatic, Proximal, Left, Distal	0	0	0	0	1		
Vehicle	Day 85	Nerve Median, Distal, Left, Proximal - Nerve, Sciatic, Proximal, Left, Proximal	0	0	0	0	1		
Vehicle	Day 85	Nerve Median, Distal, Left, Proximal - Nerve, Sciatic, Proximal, Right, Distal	-0.000163993	0.000231921	-0.002247723	0.001919737	1	-1	0.5
Vehicle	Day 85	Nerve Median, Distal, Left, Proximal - Nerve, Sciatic, Proximal, Right, Proximal	-0.000138488	0.000195851	-0.00189814	0.001621165	1	-1	0.5
Vehicle	Day 85	Nerve Median, Distal, Left, Proximal - Nerve, Ulnar, Distal, Left, Distal	0	0	0	0	1		
Vehicle	Day 85	Nerve Median, Distal, Left, Proximal - Nerve, Ulnar, Distal, Left, Proximal	0	0	0	0	1		
Vehicle	Day 85	Nerve Median, Distal, Left, Proximal - Nerve, Ulnar, Distal, Right, Distal	0	0	0	0	1		
Vehicle	Day 85	Nerve Median, Distal, Left, Proximal - Nerve, Ulnar, Distal, Right, Proximal	0	0	0	0	1		
Vehicle	Day 85	Nerve Median, Distal, Left, Proximal - Nerve, Ulnar, Proximal, Left, Distal	0	0	0	0	1		
Vehicle	Day 85	Nerve Median, Distal, Left, Proximal - Nerve, Ulnar, Proximal, Left, Proximal	0	0	0	0	1		
Vehicle	Day 85	Nerve Median, Distal, Left, Proximal - Nerve, Ulnar, Proximal, Right, Distal	0	0	0	0	1		
Vehicle	Day 85	Nerve Median, Distal, Left, Proximal - Nerve, Ulnar, Proximal, Right, Proximal	0	0	0	0	1		
Vehicle	Day 85	Nerve Median, Distal, Left, Proximal - Nerve, Optic	0	0	0	0	1		
Vehicle	Day 85	Nerve Median, Distal, Left, Proximal - Ovary	0				0		
Vehicle	Day 85	Nerve Median, Distal, Left, Proximal - Spinal Cord, Cervical	0	0	0	0	1		
Vehicle	Day 85	Nerve Median, Distal, Left, Proximal - Spinal Cord, Lumbar	0	0	0	0	1		
Vehicle	Day 85	Nerve Median, Distal, Left, Proximal - Spinal Cord, Sacral	0	0	0	0	1		
Vehicle	Day 85	Nerve Median, Distal, Left, Proximal - Spinal Cord, Spinal Roots	0	0	0	0	1		
Vehicle	Day 85	Nerve Median, Distal, Left, Proximal - Spinal Cord, Thoracic	0	0	0	0	1		
Vehicle	Day 85	Nerve Median, Distal, Left, Proximal - Spleen	-0.000126212	0.000178491	-0.001729894	0.001477469	1	-1	0.5
Vehicle	Day 85	Nerve Median, Distal, Left, Proximal - Testis	0				0		
Vehicle	Day 85	Nerve Median, Distal, Right, Distal - Nerve Median, Distal, Right, Proximal	0	0	0	0	1		
Vehicle	Day 85	Nerve Median, Distal, Right, Distal - Nerve Median, Proximal, Left, Distal	0	0	0	0	1		
Vehicle	Day 85	Nerve Median, Distal, Right, Distal - Nerve Median, Proximal, Left, Proximal	-4.58328E-05	6.48174E-05	-0.000628194	0.000536528	1	-1	0.5
Vehicle	Day 85	Nerve Median, Distal, Right, Distal - Nerve Median, Proximal, Right, Distal	0	0	0	0	1		
Vehicle	Day 85	Nerve Median, Distal, Right, Distal - Nerve Median, Proximal, Right, Proximal	0	0	0	0	1		
Vehicle	Day 85	Nerve Median, Distal, Right, Distal - Nerve, Sciatic, Distal, Left, Distal	-0.000108197	0.000153014	-0.001482969	0.001266575	1	-1	0.5
Vehicle	Day 85	Nerve Median, Distal, Right, Distal - Nerve, Sciatic, Distal, Left, Proximal	0	0	0	0	1		
Vehicle	Day 85	Nerve Median, Distal, Right, Distal - Nerve, Sciatic, Distal, Right, Distal	0	0	0	0	1		
Vehicle	Day 85	Nerve Median, Distal, Right, Distal - Nerve, Sciatic, Distal, Right, Proximal	0	0	0	0	1		
Vehicle	Day 85	Nerve Median, Distal, Right, Distal - Nerve, Sciatic, Proximal, Left, Distal	0	0	0	0	1		
Vehicle	Day 85	Nerve Median, Distal, Right, Distal - Nerve, Sciatic, Proximal, Left, Proximal	0	0	0	0	1		
Vehicle	Day 85	Nerve Median, Distal, Right, Distal - Nerve, Sciatic, Proximal, Right, Distal	-0.000163993	0.000231921	-0.002247723	0.001919737	1	-1	0.5
Vehicle	Day 85	Nerve Median, Distal, Right, Distal - Nerve, Sciatic, Proximal, Right, Proximal	-0.000138488	0.000195851	-0.00189814	0.001621165	1	-1	0.5
Vehicle	Day 85	Nerve Median, Distal, Right, Distal - Nerve, Ulnar, Distal, Left, Distal	0	0	0	0	1		
Vehicle	Day 85	Nerve Median, Distal, Right, Distal - Nerve, Ulnar, Distal, Left, Proximal	0	0	0	0	1		
Vehicle	Day 85	Nerve Median, Distal, Right, Distal - Nerve, Ulnar, Distal, Right, Distal	0	0	0	0	1		
Vehicle	Day 85	Nerve Median, Distal, Right, Distal - Nerve, Ulnar, Distal, Right, Proximal	0	0	0	0	1		
Vehicle	Day 85	Nerve Median, Distal, Right, Distal - Nerve, Ulnar, Proximal, Left, Distal	0	0	0	0	1		
Vehicle	Day 85	Nerve Median, Distal, Right, Distal - Nerve, Ulnar, Proximal, Left, Proximal	0	0	0	0	1		
Vehicle	Day 85	Nerve Median, Distal, Right, Distal - Nerve, Ulnar, Proximal, Right, Distal	0	0	0	0	1		
Vehicle	Day 85	Nerve Median, Distal, Right, Distal - Nerve, Ulnar, Proximal, Right, Proximal	0	0	0	0	1		
Vehicle	Day 85	Nerve Median, Distal, Right, Distal - Nerve, Optic	0	0	0	0	1		
Vehicle	Day 85	Nerve Median, Distal, Right, Distal - Ovary	0				0		
Vehicle	Day 85	Nerve Median, Distal, Right, Distal - Spinal Cord, Cervical	0	0	0	0	1		
Vehicle	Day 85	Nerve Median, Distal, Right, Distal - Spinal Cord, Lumbar	0	0	0	0	1		
Vehicle	Day 85	Nerve Median, Distal, Right, Distal - Spinal Cord, Sacral	0	0	0	0	1		
Vehicle	Day 85	Nerve Median, Distal, Right, Distal - Spinal Cord, Spinal Roots	0	0	0	0	1		
Vehicle	Day 85	Nerve Median, Distal, Right, Distal - Spinal Cord, Thoracic	0	0	0	0	1		
Vehicle	Day 85	Nerve Median, Distal, Right, Distal - Spleen	-0.000126212	0.000178491	-0.001729894	0.001477469	1	-1	0.5
Vehicle	Day 85	Nerve Median, Distal, Right, Distal - Testis	0				0		
Vehicle	Day 85	Nerve Median, Distal, Right, Proximal - Nerve Median, Proximal, Left, Distal	0	0	0	0	1		
Vehicle	Day 85	Nerve Median, Distal, Right, Proximal - Nerve Median, Proximal, Left, Proximal	-4.58328E-05	6.48174E-05	-0.000628194	0.000536528	1	-1	0.5
Vehicle	Day 85	Nerve Median, Distal, Right, Proximal - Nerve Median, Proximal, Right, Distal	0	0	0	0	1		
Vehicle	Day 85	Nerve Median, Distal, Right, Proximal - Nerve Median, Proximal, Right, Proximal	0	0	0	0	1		

Results of Evaluating Mean Differences Between Different Tissues Across Timepoint and Group for Log10 AAV2-ITR Target Copies/ug of DNA for Normalized Data

Group	Timepoint	Difference	Mean	StdDev	LowerCLMean	UpperCLMean	DF	tValue	P-Value
Vehicle	Day 85	Nerve Median, Distal, Right, Proximal - Nerve, Sciatic, Distal, Left, Distal	-0.000108197	0.000153014	-0.001482969	0.001266575	1	-1	0.5
Vehicle	Day 85	Nerve Median, Distal, Right, Proximal - Nerve, Sciatic, Distal, Left, Proximal	0	0	0	0	1		
Vehicle	Day 85	Nerve Median, Distal, Right, Proximal - Nerve, Sciatic, Distal, Right, Distal	0	0	0	0	1		
Vehicle	Day 85	Nerve Median, Distal, Right, Proximal - Nerve, Sciatic, Distal, Right, Proximal	0	0	0	0	1		
Vehicle	Day 85	Nerve Median, Distal, Right, Proximal - Nerve, Sciatic, Proximal, Left, Distal	0	0	0	0	1		
Vehicle	Day 85	Nerve Median, Distal, Right, Proximal - Nerve, Sciatic, Proximal, Left, Proximal	0	0	0	0	1		
Vehicle	Day 85	Nerve Median, Distal, Right, Proximal - Nerve, Sciatic, Proximal, Right, Distal	-0.000163993	0.000231921	-0.002247723	0.001919737	1	-1	0.5
Vehicle	Day 85	Nerve Median, Distal, Right, Proximal - Nerve, Sciatic, Proximal, Right, Proximal	-0.000138488	0.000195851	-0.00189814	0.001621165	1	-1	0.5
Vehicle	Day 85	Nerve Median, Distal, Right, Proximal - Nerve, Ulnar, Distal, Left, Distal	0	0	0	0	1		
Vehicle	Day 85	Nerve Median, Distal, Right, Proximal - Nerve, Ulnar, Distal, Left, Proximal	0	0	0	0	1		
Vehicle	Day 85	Nerve Median, Distal, Right, Proximal - Nerve, Ulnar, Distal, Right, Distal	0	0	0	0	1		
Vehicle	Day 85	Nerve Median, Distal, Right, Proximal - Nerve, Ulnar, Distal, Right, Proximal	0	0	0	0	1		
Vehicle	Day 85	Nerve Median, Distal, Right, Proximal - Nerve, Ulnar, Proximal, Left, Distal	0	0	0	0	1		
Vehicle	Day 85	Nerve Median, Distal, Right, Proximal - Nerve, Ulnar, Proximal, Left, Proximal	0	0	0	0	1		
Vehicle	Day 85	Nerve Median, Distal, Right, Proximal - Nerve, Ulnar, Proximal, Right, Distal	0	0	0	0	1		
Vehicle	Day 85	Nerve Median, Distal, Right, Proximal - Nerve, Ulnar, Proximal, Right, Proximal	0	0	0	0	1		
Vehicle	Day 85	Nerve Median, Distal, Right, Proximal - Nerve, Optic	0	0	0	0	1		
Vehicle	Day 85	Nerve Median, Distal, Right, Proximal - Ovary	0				0		
Vehicle	Day 85	Nerve Median, Distal, Right, Proximal - Spinal Cord, Cervical	0	0	0	0	1		
Vehicle	Day 85	Nerve Median, Distal, Right, Proximal - Spinal Cord, Lumbar	0	0	0	0	1		
Vehicle	Day 85	Nerve Median, Distal, Right, Proximal - Spinal Cord, Sacral	0	0	0	0	1		
Vehicle	Day 85	Nerve Median, Distal, Right, Proximal - Spinal Cord, Spinal Roots	0	0	0	0	1		
Vehicle	Day 85	Nerve Median, Distal, Right, Proximal - Spinal Cord, Thoracic	0	0	0	0	1		
Vehicle	Day 85	Nerve Median, Distal, Right, Proximal - Spleen	-0.000126212	0.000178491	-0.001729894	0.001477469	1	-1	0.5
Vehicle	Day 85	Nerve Median, Distal, Right, Proximal - Testis	0				0		
Vehicle	Day 85	Nerve Median, Proximal, Left, Distal - Nerve Median, Proximal, Left, Proximal	-4.58328E-05	6.48174E-05	-0.000628194	0.000536528	1	-1	0.5
Vehicle	Day 85	Nerve Median, Proximal, Left, Distal - Nerve Median, Proximal, Right, Distal	0	0	0	0	1		
Vehicle	Day 85	Nerve Median, Proximal, Left, Distal - Nerve Median, Proximal, Right, Proximal	0	0	0	0	1		
Vehicle	Day 85	Nerve Median, Proximal, Left, Distal - Nerve, Sciatic, Distal, Left, Distal	-0.000108197	0.000153014	-0.001482969	0.001266575	1	-1	0.5
Vehicle	Day 85	Nerve Median, Proximal, Left, Distal - Nerve, Sciatic, Distal, Left, Proximal	0	0	0	0	1		
Vehicle	Day 85	Nerve Median, Proximal, Left, Distal - Nerve, Sciatic, Distal, Right, Distal	0	0	0	0	1		
Vehicle	Day 85	Nerve Median, Proximal, Left, Distal - Nerve, Sciatic, Distal, Right, Proximal	0	0	0	0	1		
Vehicle	Day 85	Nerve Median, Proximal, Left, Distal - Nerve, Sciatic, Proximal, Left, Distal	0	0	0	0	1		
Vehicle	Day 85	Nerve Median, Proximal, Left, Distal - Nerve, Sciatic, Proximal, Left, Proximal	0	0	0	0	1		
Vehicle	Day 85	Nerve Median, Proximal, Left, Distal - Nerve, Sciatic, Proximal, Right, Distal	-0.000163993	0.000231921	-0.002247723	0.001919737	1	-1	0.5
Vehicle	Day 85	Nerve Median, Proximal, Left, Distal - Nerve, Sciatic, Proximal, Right, Proximal	-0.000138488	0.000195851	-0.00189814	0.001621165	1	-1	0.5
Vehicle	Day 85	Nerve Median, Proximal, Left, Distal - Nerve, Ulnar, Distal, Left, Distal	0	0	0	0	1		
Vehicle	Day 85	Nerve Median, Proximal, Left, Distal - Nerve, Ulnar, Distal, Left, Proximal	0	0	0	0	1		
Vehicle	Day 85	Nerve Median, Proximal, Left, Distal - Nerve, Ulnar, Distal, Right, Distal	0	0	0	0	1		
Vehicle	Day 85	Nerve Median, Proximal, Left, Distal - Nerve, Ulnar, Distal, Right, Proximal	0	0	0	0	1		
Vehicle	Day 85	Nerve Median, Proximal, Left, Distal - Nerve, Ulnar, Proximal, Left, Distal	0	0	0	0	1		
Vehicle	Day 85	Nerve Median, Proximal, Left, Distal - Nerve, Ulnar, Proximal, Left, Proximal	0	0	0	0	1		
Vehicle	Day 85	Nerve Median, Proximal, Left, Distal - Nerve, Ulnar, Proximal, Right, Distal	0	0	0	0	1		
Vehicle	Day 85	Nerve Median, Proximal, Left, Distal - Nerve, Ulnar, Proximal, Right, Proximal	0	0	0	0	1		
Vehicle	Day 85	Nerve Median, Proximal, Left, Distal - Nerve, Optic	0	0	0	0	1		
Vehicle	Day 85	Nerve Median, Proximal, Left, Distal - Ovary	0				0		
Vehicle	Day 85	Nerve Median, Proximal, Left, Distal - Spinal Cord, Cervical	0	0	0	0	1		
Vehicle	Day 85	Nerve Median, Proximal, Left, Distal - Spinal Cord, Lumbar	0	0	0	0	1		
Vehicle	Day 85	Nerve Median, Proximal, Left, Distal - Spinal Cord, Sacral	0	0	0	0	1		
Vehicle	Day 85	Nerve Median, Proximal, Left, Distal - Spinal Cord, Spinal Roots	0	0	0	0	1		
Vehicle	Day 85	Nerve Median, Proximal, Left, Distal - Spinal Cord, Thoracic	0	0	0	0	1		
Vehicle	Day 85	Nerve Median, Proximal, Left, Distal - Spleen	-0.000126212	0.000178491	-0.001729894	0.001477469	1	-1	0.5
Vehicle	Day 85	Nerve Median, Proximal, Left, Distal - Testis	0				0		
Vehicle	Day 85	Nerve Median, Proximal, Left, Proximal - Nerve Median, Proximal, Right, Distal	4.58328E-05	6.48174E-05	-0.000536528	0.000628194	1	1	0.5
Vehicle	Day 85	Nerve Median, Proximal, Left, Proximal - Nerve Median, Proximal, Right, Proximal	4.58328E-05	6.48174E-05	-0.000536528	0.000628194	1	1	0.5
Vehicle	Day 85	Nerve Median, Proximal, Left, Proximal - Nerve, Sciatic, Distal, Left, Distal	-6.23641E-05	0.000217831	-0.002019497	0.001894769	1	-0.404883417	0.755086569
Vehicle	Day 85	Nerve Median, Proximal, Left, Proximal - Nerve, Sciatic, Distal, Left, Proximal	4.58328E-05	6.48174E-05	-0.000536528	0.000628194	1	1	0.5
Vehicle	Day 85	Nerve Median, Proximal, Left, Proximal - Nerve, Sciatic, Distal, Right, Distal	4.58328E-05	6.48174E-05	-0.000536528	0.000628194	1	1	0.5
Vehicle	Day 85	Nerve Median, Proximal, Left, Proximal - Nerve, Sciatic, Distal, Right, Proximal	4.58328E-05	6.48174E-05	-0.000536528	0.000628194	1	1	0.5
Vehicle	Day 85	Nerve Median, Proximal, Left, Proximal - Nerve, Sciatic, Proximal, Left, Distal	4.58328E-05	6.48174E-05	-0.000536528	0.000628194	1	1	0.5
Vehicle	Day 85	Nerve Median, Proximal, Left, Proximal - Nerve, Sciatic, Proximal, Left, Proximal	4.58328E-05	6.48174E-05	-0.000536528	0.000628194	1	1	0.5
Vehicle	Day 85	Nerve Median, Proximal, Left, Proximal - Nerve, Sciatic, Proximal, Right, Distal	-0.00011816	0.000296739	-0.002784252	0.002547931	1	-0.563134887	0.673495787
Vehicle	Day 85	Nerve Median, Proximal, Left, Proximal - Nerve, Sciatic, Proximal, Right, Proximal	-9.26548E-05	0.000131034	-0.001269946	0.001084636	1	-1	0.5
Vehicle	Day 85	Nerve Median, Proximal, Left, Proximal - Nerve, Ulnar, Distal, Left, Distal	4.58328E-05	6.48174E-05	-0.000536528	0.000628194	1	1	0.5
Vehicle	Day 85	Nerve Median, Proximal, Left, Proximal - Nerve, Ulnar, Distal, Left, Proximal	4.58328E-05	6.48174E-05	-0.000536528	0.000628194	1	1	0.5
Vehicle	Day 85	Nerve Median, Proximal, Left, Proximal - Nerve, Ulnar, Distal, Right, Distal	4.58328E-05	6.48174E-05	-0.000536528	0.000628194	1	1	0.5
Vehicle	Day 85	Nerve Median, Proximal, Left, Proximal - Nerve, Ulnar, Distal, Right, Proximal	4.58328E-05	6.48174E-05	-0.000536528	0.000628194	1	1	0.5
Vehicle	Day 85	Nerve Median, Proximal, Left, Proximal - Nerve, Ulnar, Proximal, Left, Distal	4.58328E-05	6.48174E-05	-0.000536528	0.000628194	1	1	0.5
Vehicle	Day 85	Nerve Median, Proximal, Left, Proximal - Nerve, Ulnar, Proximal, Left, Proximal	4.58328E-05	6.48174E-05	-0.000536528	0.000628194	1	1	0.5
Vehicle	Day 85	Nerve Median, Proximal, Left, Proximal - Nerve, Ulnar, Proximal, Right, Distal	4.58328E-05	6.48174E-05	-0.000536528	0.000628194	1	1	0.5
Vehicle	Day 85	Nerve Median, Proximal, Left, Proximal - Nerve, Ulnar, Proximal, Right, Proximal	4.58328E-05	6.48174E-05	-0.000536528	0.000628194	1	1	0.5
Vehicle	Day 85	Nerve Median, Proximal, Left, Proximal - Nerve, Optic	4.58328E-05	6.48174E-05	-0.000536528	0.000628194	1	1	0.5
Vehicle	Day 85	Nerve Median, Proximal, Left, Proximal - Ovary	0				0		
Vehicle	Day 85	Nerve Median, Proximal, Left, Proximal - Spinal Cord, Cervical	4.58328E-05	6.48174E-05	-0.000536528	0.000628194	1	1	0.5
Vehicle	Day 85	Nerve Median, Proximal, Left, Proximal - Spinal Cord, Lumbar	4.58328E-05	6.48174E-05	-0.000536528	0.000628194	1	1	0.5
Vehicle	Day 85	Nerve Median, Proximal, Left, Proximal - Spinal Cord, Sacral	4.58328E-05	6.48174E-05	-0.000536528	0.000628194	1	1	0.5
Vehicle	Day 85	Nerve Median, Proximal, Left, Proximal - Spinal Cord, Spinal Roots	4.58328E-05	6.48174E-05	-0.000536528	0.000628194	1	1	0.5
Vehicle	Day 85	Nerve Median, Proximal, Left, Proximal - Spinal Cord, Thoracic	4.58328E-05	6.48174E-05	-0.000536528	0.000628194	1	1	0.5
Vehicle	Day 85	Nerve Median, Proximal, Left, Proximal - Spleen	-8.03796E-05	0.000243309	-0.002266422	0.002105663	1	-0.467200536	0.721755482
Vehicle	Day 85	Nerve Median, Proximal, Left, Proximal - Testis	9.16656E-05				0		
Vehicle	Day 85	Nerve Median, Proximal, Right, Distal - Nerve Median, Proximal, Right, Proximal	0	0	0	0	1		
Vehicle	Day 85	Nerve Median, Proximal, Right, Distal - Nerve, Sciatic, Distal, Left, Distal	-0.000108197	0.000153014	-0.001482969	0.001266575	1	-1	0.5
Vehicle	Day 85	Nerve Median, Proximal, Right, Distal - Nerve, Sciatic, Distal, Left, Proximal	0	0	0	0	1		
Vehicle	Day 85	Nerve Median, Proximal, Right, Distal - Nerve, Sciatic, Distal, Right, Distal	0	0	0	0	1		
Vehicle	Day 85	Nerve Median, Proximal, Right, Distal - Nerve, Sciatic, Distal, Right, Proximal	0	0	0	0	1		
Vehicle	Day 85	Nerve Median, Proximal, Right, Distal - Nerve, Sciatic, Proximal, Left, Distal	0	0	0	0	1		
Vehicle	Day 85	Nerve Median, Proximal, Right, Distal - Nerve, Sciatic, Proximal, Left, Proximal	0	0	0	0	1		
Vehicle	Day 85	Nerve Median, Proximal, Right, Distal - Nerve, Sciatic, Proximal, Right, Distal	-0.000163993	0.000231921	-0.002247723	0.001919737	1	-1	0.5
Vehicle	Day 85	Nerve Median, Proximal, Right, Distal - Nerve, Sciatic, Proximal, Right, Proximal	-0.000138488	0.000195851	-0.00189814	0.001621165	1	-1	0.5
Vehicle	Day 85	Nerve Median, Proximal, Right, Distal - Nerve, Ulnar, Distal, Left, Distal	0	0	0	0	1		
Vehicle	Day 85	Nerve Median, Proximal, Right, Distal - Nerve, Ulnar, Distal, Left, Proximal	0	0	0	0	1		
Vehicle	Day 85	Nerve Median, Proximal, Right, Distal - Nerve, Ulnar, Distal, Right, Distal	0	0	0	0	1		
Vehicle	Day 85	Nerve Median, Proximal, Right, Distal - Nerve, Ulnar, Distal, Right, Proximal	0	0	0	0	1		

Results of Evaluating Mean Differences Between Different Tissues Across Timepoint and Group for Log10 AAV2-ITR Target Copies/ug of DNA for Normalized Data

Group	Timepoint	Difference	Mean	StdDev	LowerCLMean	UpperCLMean	DF	tValue	P-Value
Vehicle	Day 85	Nerve Median, Proximal, Right, Distal - Nerve, Ulnar, Proximal, Left, Distal	0	0	0	0	1		
Vehicle	Day 85	Nerve Median, Proximal, Right, Distal - Nerve, Ulnar, Proximal, Left, Proximal	0	0	0	0	1		
Vehicle	Day 85	Nerve Median, Proximal, Right, Distal - Nerve, Ulnar, Proximal, Right, Distal	0	0	0	0	1		
Vehicle	Day 85	Nerve Median, Proximal, Right, Distal - Nerve, Ulnar, Proximal, Right, Proximal	0	0	0	0	1		
Vehicle	Day 85	Nerve Median, Proximal, Right, Distal - Nerve, Optic	0	0	0	0	1		
Vehicle	Day 85	Nerve Median, Proximal, Right, Distal - Ovary	0				0		
Vehicle	Day 85	Nerve Median, Proximal, Right, Distal - Spinal Cord, Cervical	0	0	0	0	1		
Vehicle	Day 85	Nerve Median, Proximal, Right, Distal - Spinal Cord, Lumbar	0	0	0	0	1		
Vehicle	Day 85	Nerve Median, Proximal, Right, Distal - Spinal Cord, Sacral	0	0	0	0	1		
Vehicle	Day 85	Nerve Median, Proximal, Right, Distal - Spinal Cord, Spinal Roots	0	0	0	0	1		
Vehicle	Day 85	Nerve Median, Proximal, Right, Distal - Spinal Cord, Thoracic	0	0	0	0	1		
Vehicle	Day 85	Nerve Median, Proximal, Right, Distal - Spleen	-0.000126212	0.000178491	-0.001729894	0.001477469	1	-1	0.5
Vehicle	Day 85	Nerve Median, Proximal, Right, Distal - Testis	0				0		
Vehicle	Day 85	Nerve Median, Proximal, Right, Proximal - Nerve, Sciatic, Distal, Left, Distal	-0.000108197	0.000153014	-0.001482969	0.001266575	1	-1	0.5
Vehicle	Day 85	Nerve Median, Proximal, Right, Proximal - Nerve, Sciatic, Distal, Left, Proximal	0	0	0	0	1		
Vehicle	Day 85	Nerve Median, Proximal, Right, Proximal - Nerve, Sciatic, Distal, Right, Distal	0	0	0	0	1		
Vehicle	Day 85	Nerve Median, Proximal, Right, Proximal - Nerve, Sciatic, Distal, Right, Proximal	0	0	0	0	1		
Vehicle	Day 85	Nerve Median, Proximal, Right, Proximal - Nerve, Sciatic, Proximal, Left, Distal	0	0	0	0	1		
Vehicle	Day 85	Nerve Median, Proximal, Right, Proximal - Nerve, Sciatic, Proximal, Left, Proximal	0	0	0	0	1		
Vehicle	Day 85	Nerve Median, Proximal, Right, Proximal - Nerve, Sciatic, Proximal, Right, Distal	-0.000163993	0.000231921	-0.002247723	0.001919737	1	-1	0.5
Vehicle	Day 85	Nerve Median, Proximal, Right, Proximal - Nerve, Sciatic, Proximal, Right, Proximal	-0.000138488	0.000195851	-0.00189814	0.001621165	1	-1	0.5
Vehicle	Day 85	Nerve Median, Proximal, Right, Proximal - Nerve, Ulnar, Distal, Left, Distal	0	0	0	0	1		
Vehicle	Day 85	Nerve Median, Proximal, Right, Proximal - Nerve, Ulnar, Distal, Left, Proximal	0	0	0	0	1		
Vehicle	Day 85	Nerve Median, Proximal, Right, Proximal - Nerve, Ulnar, Distal, Right, Distal	0	0	0	0	1		
Vehicle	Day 85	Nerve Median, Proximal, Right, Proximal - Nerve, Ulnar, Distal, Right, Proximal	0	0	0	0	1		
Vehicle	Day 85	Nerve Median, Proximal, Right, Proximal - Nerve, Ulnar, Proximal, Left, Distal	0	0	0	0	1		
Vehicle	Day 85	Nerve Median, Proximal, Right, Proximal - Nerve, Ulnar, Proximal, Left, Proximal	0	0	0	0	1		
Vehicle	Day 85	Nerve Median, Proximal, Right, Proximal - Nerve, Ulnar, Proximal, Right, Distal	0	0	0	0	1		
Vehicle	Day 85	Nerve Median, Proximal, Right, Proximal - Nerve, Ulnar, Proximal, Right, Proximal	0	0	0	0	1		
Vehicle	Day 85	Nerve Median, Proximal, Right, Proximal - Nerve, Optic	0	0	0	0	1		
Vehicle	Day 85	Nerve Median, Proximal, Right, Proximal - Ovary	0				0		
Vehicle	Day 85	Nerve Median, Proximal, Right, Proximal - Spinal Cord, Cervical	0	0	0	0	1		
Vehicle	Day 85	Nerve Median, Proximal, Right, Proximal - Spinal Cord, Lumbar	0	0	0	0	1		
Vehicle	Day 85	Nerve Median, Proximal, Right, Proximal - Spinal Cord, Sacral	0	0	0	0	1		
Vehicle	Day 85	Nerve Median, Proximal, Right, Proximal - Spinal Cord, Spinal Roots	0	0	0	0	1		
Vehicle	Day 85	Nerve Median, Proximal, Right, Proximal - Spinal Cord, Thoracic	0	0	0	0	1		
Vehicle	Day 85	Nerve Median, Proximal, Right, Proximal - Spleen	-0.000126212	0.000178491	-0.001729894	0.001477469	1	-1	0.5
Vehicle	Day 85	Nerve Median, Proximal, Right, Proximal - Testis	0				0		
Vehicle	Day 85	Nerve, Sciatic, Distal, Left, Distal - Nerve, Sciatic, Distal, Left, Proximal	0.000108197	0.000153014	-0.001266575	0.001482969	1	1	0.5
Vehicle	Day 85	Nerve, Sciatic, Distal, Left, Distal - Nerve, Sciatic, Distal, Right, Distal	0.000108197	0.000153014	-0.001266575	0.001482969	1	1	0.5
Vehicle	Day 85	Nerve, Sciatic, Distal, Left, Distal - Nerve, Sciatic, Distal, Right, Proximal	0.000108197	0.000153014	-0.001266575	0.001482969	1	1	0.5
Vehicle	Day 85	Nerve, Sciatic, Distal, Left, Distal - Nerve, Sciatic, Proximal, Left, Distal	0.000108197	0.000153014	-0.001266575	0.001482969	1	1	0.5
Vehicle	Day 85	Nerve, Sciatic, Distal, Left, Distal - Nerve, Sciatic, Proximal, Left, Proximal	0.000108197	0.000153014	-0.001266575	0.001482969	1	1	0.5
Vehicle	Day 85	Nerve, Sciatic, Distal, Left, Distal - Nerve, Sciatic, Proximal, Right, Distal	-5.57962E-05	7.89078E-05	-0.000764755	0.000653162	1	-1	0.5
Vehicle	Day 85	Nerve, Sciatic, Distal, Left, Distal - Nerve, Sciatic, Proximal, Right, Proximal	-3.02908E-05	0.000348865	-0.003164715	0.003104133	1	-0.122791463	0.922217893
Vehicle	Day 85	Nerve, Sciatic, Distal, Left, Distal - Nerve, Ulnar, Distal, Left, Distal	0.000108197	0.000153014	-0.001266575	0.001482969	1	1	0.5
Vehicle	Day 85	Nerve, Sciatic, Distal, Left, Distal - Nerve, Ulnar, Distal, Left, Proximal	0.000108197	0.000153014	-0.001266575	0.001482969	1	1	0.5
Vehicle	Day 85	Nerve, Sciatic, Distal, Left, Distal - Nerve, Ulnar, Distal, Right, Distal	0.000108197	0.000153014	-0.001266575	0.001482969	1	1	0.5
Vehicle	Day 85	Nerve, Sciatic, Distal, Left, Distal - Nerve, Ulnar, Distal, Right, Proximal	0.000108197	0.000153014	-0.001266575	0.001482969	1	1	0.5
Vehicle	Day 85	Nerve, Sciatic, Distal, Left, Distal - Nerve, Ulnar, Proximal, Left, Distal	0.000108197	0.000153014	-0.001266575	0.001482969	1	1	0.5
Vehicle	Day 85	Nerve, Sciatic, Distal, Left, Distal - Nerve, Ulnar, Proximal, Left, Proximal	0.000108197	0.000153014	-0.001266575	0.001482969	1	1	0.5
Vehicle	Day 85	Nerve, Sciatic, Distal, Left, Distal - Nerve, Ulnar, Proximal, Right, Distal	0.000108197	0.000153014	-0.001266575	0.001482969	1	1	0.5
Vehicle	Day 85	Nerve, Sciatic, Distal, Left, Distal - Nerve, Ulnar, Proximal, Right, Proximal	0.000108197	0.000153014	-0.001266575	0.001482969	1	1	0.5
Vehicle	Day 85	Nerve, Sciatic, Distal, Left, Distal - Nerve, Optic	0.000108197	0.000153014	-0.001266575	0.001482969	1	1	0.5
Vehicle	Day 85	Nerve, Sciatic, Distal, Left, Distal - Ovary	0.000216394				0		
Vehicle	Day 85	Nerve, Sciatic, Distal, Left, Distal - Spinal Cord, Cervical	0.000108197	0.000153014	-0.001266575	0.001482969	1	1	0.5
Vehicle	Day 85	Nerve, Sciatic, Distal, Left, Distal - Spinal Cord, Lumbar	0.000108197	0.000153014	-0.001266575	0.001482969	1	1	0.5
Vehicle	Day 85	Nerve, Sciatic, Distal, Left, Distal - Spinal Cord, Sacral	0.000108197	0.000153014	-0.001266575	0.001482969	1	1	0.5
Vehicle	Day 85	Nerve, Sciatic, Distal, Left, Distal - Spinal Cord, Spinal Roots	0.000108197	0.000153014	-0.001266575	0.001482969	1	1	0.5
Vehicle	Day 85	Nerve, Sciatic, Distal, Left, Distal - Spinal Cord, Thoracic	0.000108197	0.000153014	-0.001266575	0.001482969	1	1	0.5
Vehicle	Day 85	Nerve, Sciatic, Distal, Left, Distal - Spleen	-1.80156E-05	2.54779E-05	-0.000246925	0.000210894	1	-1	0.5
Vehicle	Day 85	Nerve, Sciatic, Distal, Left, Distal - Testis	0				0		
Vehicle	Day 85	Nerve, Sciatic, Distal, Left, Proximal - Nerve, Sciatic, Distal, Right, Distal	0	0	0	0	1		
Vehicle	Day 85	Nerve, Sciatic, Distal, Left, Proximal - Nerve, Sciatic, Distal, Right, Proximal	0	0	0	0	1		
Vehicle	Day 85	Nerve, Sciatic, Distal, Left, Proximal - Nerve, Sciatic, Proximal, Left, Distal	0	0	0	0	1		
Vehicle	Day 85	Nerve, Sciatic, Distal, Left, Proximal - Nerve, Sciatic, Proximal, Left, Proximal	0	0	0	0	1		
Vehicle	Day 85	Nerve, Sciatic, Distal, Left, Proximal - Nerve, Sciatic, Proximal, Right, Distal	-0.000163993	0.000231921	-0.002247723	0.001919737	1	-1	0.5
Vehicle	Day 85	Nerve, Sciatic, Distal, Left, Proximal - Nerve, Sciatic, Proximal, Right, Proximal	-0.000138488	0.000195851	-0.00189814	0.001621165	1	-1	0.5
Vehicle	Day 85	Nerve, Sciatic, Distal, Left, Proximal - Nerve, Ulnar, Distal, Left, Distal	0	0	0	0	1		
Vehicle	Day 85	Nerve, Sciatic, Distal, Left, Proximal - Nerve, Ulnar, Distal, Left, Proximal	0	0	0	0	1		
Vehicle	Day 85	Nerve, Sciatic, Distal, Left, Proximal - Nerve, Ulnar, Distal, Right, Distal	0	0	0	0	1		
Vehicle	Day 85	Nerve, Sciatic, Distal, Left, Proximal - Nerve, Ulnar, Distal, Right, Proximal	0	0	0	0	1		
Vehicle	Day 85	Nerve, Sciatic, Distal, Left, Proximal - Nerve, Ulnar, Proximal, Left, Distal	0	0	0	0	1		
Vehicle	Day 85	Nerve, Sciatic, Distal, Left, Proximal - Nerve, Ulnar, Proximal, Left, Proximal	0	0	0	0	1		
Vehicle	Day 85	Nerve, Sciatic, Distal, Left, Proximal - Nerve, Ulnar, Proximal, Right, Distal	0	0	0	0	1		
Vehicle	Day 85	Nerve, Sciatic, Distal, Left, Proximal - Nerve, Ulnar, Proximal, Right, Proximal	0	0	0	0	1		
Vehicle	Day 85	Nerve, Sciatic, Distal, Left, Proximal - Nerve, Optic	0	0	0	0	1		
Vehicle	Day 85	Nerve, Sciatic, Distal, Left, Proximal - Ovary	0				0		
Vehicle	Day 85	Nerve, Sciatic, Distal, Left, Proximal - Spinal Cord, Cervical	0	0	0	0	1		
Vehicle	Day 85	Nerve, Sciatic, Distal, Left, Proximal - Spinal Cord, Lumbar	0	0	0	0	1		
Vehicle	Day 85	Nerve, Sciatic, Distal, Left, Proximal - Spinal Cord, Sacral	0	0	0	0	1		
Vehicle	Day 85	Nerve, Sciatic, Distal, Left, Proximal - Spinal Cord, Spinal Roots	0	0	0	0	1		
Vehicle	Day 85	Nerve, Sciatic, Distal, Left, Proximal - Spinal Cord, Thoracic	0	0	0	0	1		
Vehicle	Day 85	Nerve, Sciatic, Distal, Left, Proximal - Spleen	-0.000126212	0.000178491	-0.001729894	0.001477469	1	-1	0.5
Vehicle	Day 85	Nerve, Sciatic, Distal, Left, Proximal - Testis	0				0		
Vehicle	Day 85	Nerve, Sciatic, Distal, Right, Distal - Nerve, Sciatic, Distal, Right, Proximal	0	0	0	0	1		
Vehicle	Day 85	Nerve, Sciatic, Distal, Right, Distal - Nerve, Sciatic, Proximal, Left, Distal	0	0	0	0	1		
Vehicle	Day 85	Nerve, Sciatic, Distal, Right, Distal - Nerve, Sciatic, Proximal, Left, Proximal	0	0	0	0	1		
Vehicle	Day 85	Nerve, Sciatic, Distal, Right, Distal - Nerve, Sciatic, Proximal, Right, Distal	-0.000163993	0.000231921	-0.002247723	0.001919737	1	-1	0.5
Vehicle	Day 85	Nerve, Sciatic, Distal, Right, Distal - Nerve, Sciatic, Proximal, Right, Proximal	-0.000138488	0.000195851	-0.00189814	0.001621165	1	-1	0.5
Vehicle	Day 85	Nerve, Sciatic, Distal, Right, Distal - Nerve, Ulnar, Distal, Left, Distal	0	0	0	0	1		
Vehicle	Day 85	Nerve, Sciatic, Distal, Right, Distal - Nerve, Ulnar, Distal, Left, Proximal	0	0	0	0	1		
Vehicle	Day 85	Nerve, Sciatic, Distal, Right, Distal - Nerve, Ulnar, Distal, Right, Distal	0	0	0	0	1		

Results of Evaluating Mean Differences Between Different Tissues Across Timepoint and Group for Log10 AAV2-ITR Target Copies/ug of DNA for Normalized Data

Group	Timepoint	Difference	Mean	StdDev	LowerCLMean	UpperCLMean	DF	tValue	P-Value
Vehicle	Day 85	Nerve, Sciatic, Distal, Right, Distal - Nerve, Ulnar, Distal, Right, Proximal	0	0	0	0	1		
Vehicle	Day 85	Nerve, Sciatic, Distal, Right, Distal - Nerve, Ulnar, Proximal, Left, Distal	0	0	0	0	1		
Vehicle	Day 85	Nerve, Sciatic, Distal, Right, Distal - Nerve, Ulnar, Proximal, Left, Proximal	0	0	0	0	1		
Vehicle	Day 85	Nerve, Sciatic, Distal, Right, Distal - Nerve, Ulnar, Proximal, Right, Distal	0	0	0	0	1		
Vehicle	Day 85	Nerve, Sciatic, Distal, Right, Distal - Nerve, Ulnar, Proximal, Right, Proximal	0	0	0	0	1		
Vehicle	Day 85	Nerve, Sciatic, Distal, Right, Distal - Nerve, Optic	0	0	0	0	1		
Vehicle	Day 85	Nerve, Sciatic, Distal, Right, Distal - Ovary	0				0		
Vehicle	Day 85	Nerve, Sciatic, Distal, Right, Distal - Spinal Cord, Cervical	0	0	0	0	1		
Vehicle	Day 85	Nerve, Sciatic, Distal, Right, Distal - Spinal Cord, Lumbar	0	0	0	0	1		
Vehicle	Day 85	Nerve, Sciatic, Distal, Right, Distal - Spinal Cord, Sacral	0	0	0	0	1		
Vehicle	Day 85	Nerve, Sciatic, Distal, Right, Distal - Spinal Cord, Spinal Roots	0	0	0	0	1		
Vehicle	Day 85	Nerve, Sciatic, Distal, Right, Distal - Spinal Cord, Thoracic	0	0	0	0	1		
Vehicle	Day 85	Nerve, Sciatic, Distal, Right, Distal - Spleen	-0.000126212	0.000178491	-0.001729894	0.001477469	1	-1	0.5
Vehicle	Day 85	Nerve, Sciatic, Distal, Right, Distal - Testis	0				0		
Vehicle	Day 85	Nerve, Sciatic, Distal, Right, Proximal - Nerve, Sciatic, Proximal, Left, Distal	0	0	0	0	1		
Vehicle	Day 85	Nerve, Sciatic, Distal, Right, Proximal - Nerve, Sciatic, Proximal, Left, Proximal	0	0	0	0	1		
Vehicle	Day 85	Nerve, Sciatic, Distal, Right, Proximal - Nerve, Sciatic, Proximal, Right, Distal	-0.000163993	0.000231921	-0.002247723	0.001919737	1	-1	0.5
Vehicle	Day 85	Nerve, Sciatic, Distal, Right, Proximal - Nerve, Sciatic, Proximal, Right, Proximal	-0.000138488	0.000195851	-0.00189814	0.001621165	1	-1	0.5
Vehicle	Day 85	Nerve, Sciatic, Distal, Right, Proximal - Nerve, Ulnar, Distal, Left, Distal	0	0	0	0	1		
Vehicle	Day 85	Nerve, Sciatic, Distal, Right, Proximal - Nerve, Ulnar, Distal, Left, Proximal	0	0	0	0	1		
Vehicle	Day 85	Nerve, Sciatic, Distal, Right, Proximal - Nerve, Ulnar, Distal, Right, Distal	0	0	0	0	1		
Vehicle	Day 85	Nerve, Sciatic, Distal, Right, Proximal - Nerve, Ulnar, Distal, Right, Proximal	0	0	0	0	1		
Vehicle	Day 85	Nerve, Sciatic, Distal, Right, Proximal - Nerve, Ulnar, Proximal, Left, Distal	0	0	0	0	1		
Vehicle	Day 85	Nerve, Sciatic, Distal, Right, Proximal - Nerve, Ulnar, Proximal, Left, Proximal	0	0	0	0	1		
Vehicle	Day 85	Nerve, Sciatic, Distal, Right, Proximal - Nerve, Ulnar, Proximal, Right, Distal	0	0	0	0	1		
Vehicle	Day 85	Nerve, Sciatic, Distal, Right, Proximal - Nerve, Ulnar, Proximal, Right, Proximal	0	0	0	0	1		
Vehicle	Day 85	Nerve, Sciatic, Distal, Right, Proximal - Nerve, Optic	0	0	0	0	1		
Vehicle	Day 85	Nerve, Sciatic, Distal, Right, Proximal - Ovary	0				0		
Vehicle	Day 85	Nerve, Sciatic, Distal, Right, Proximal - Spinal Cord, Cervical	0	0	0	0	1		
Vehicle	Day 85	Nerve, Sciatic, Distal, Right, Proximal - Spinal Cord, Lumbar	0	0	0	0	1		
Vehicle	Day 85	Nerve, Sciatic, Distal, Right, Proximal - Spinal Cord, Sacral	0	0	0	0	1		
Vehicle	Day 85	Nerve, Sciatic, Distal, Right, Proximal - Spinal Cord, Spinal Roots	0	0	0	0	1		
Vehicle	Day 85	Nerve, Sciatic, Distal, Right, Proximal - Spinal Cord, Thoracic	0	0	0	0	1		
Vehicle	Day 85	Nerve, Sciatic, Distal, Right, Proximal - Spleen	-0.000126212	0.000178491	-0.001729894	0.001477469	1	-1	0.5
Vehicle	Day 85	Nerve, Sciatic, Distal, Right, Proximal - Testis	0				0		
Vehicle	Day 85	Nerve, Sciatic, Proximal, Left, Distal - Nerve, Sciatic, Proximal, Left, Proximal	0	0	0	0	1		
Vehicle	Day 85	Nerve, Sciatic, Proximal, Left, Distal - Nerve, Sciatic, Proximal, Right, Distal	-0.000163993	0.000231921	-0.002247723	0.001919737	1	-1	0.5
Vehicle	Day 85	Nerve, Sciatic, Proximal, Left, Distal - Nerve, Sciatic, Proximal, Right, Proximal	-0.000138488	0.000195851	-0.00189814	0.001621165	1	-1	0.5
Vehicle	Day 85	Nerve, Sciatic, Proximal, Left, Distal - Nerve, Ulnar, Distal, Left, Distal	0	0	0	0	1		
Vehicle	Day 85	Nerve, Sciatic, Proximal, Left, Distal - Nerve, Ulnar, Distal, Left, Proximal	0	0	0	0	1		
Vehicle	Day 85	Nerve, Sciatic, Proximal, Left, Distal - Nerve, Ulnar, Distal, Right, Distal	0	0	0	0	1		
Vehicle	Day 85	Nerve, Sciatic, Proximal, Left, Distal - Nerve, Ulnar, Distal, Right, Proximal	0	0	0	0	1		
Vehicle	Day 85	Nerve, Sciatic, Proximal, Left, Distal - Nerve, Ulnar, Proximal, Left, Distal	0	0	0	0	1		
Vehicle	Day 85	Nerve, Sciatic, Proximal, Left, Distal - Nerve, Ulnar, Proximal, Left, Proximal	0	0	0	0	1		
Vehicle	Day 85	Nerve, Sciatic, Proximal, Left, Distal - Nerve, Ulnar, Proximal, Right, Distal	0	0	0	0	1		
Vehicle	Day 85	Nerve, Sciatic, Proximal, Left, Distal - Nerve, Ulnar, Proximal, Right, Proximal	0	0	0	0	1		
Vehicle	Day 85	Nerve, Sciatic, Proximal, Left, Distal - Nerve, Optic	0	0	0	0	1		
Vehicle	Day 85	Nerve, Sciatic, Proximal, Left, Distal - Ovary	0				0		
Vehicle	Day 85	Nerve, Sciatic, Proximal, Left, Distal - Spinal Cord, Cervical	0	0	0	0	1		
Vehicle	Day 85	Nerve, Sciatic, Proximal, Left, Distal - Spinal Cord, Lumbar	0	0	0	0	1		
Vehicle	Day 85	Nerve, Sciatic, Proximal, Left, Distal - Spinal Cord, Sacral	0	0	0	0	1		
Vehicle	Day 85	Nerve, Sciatic, Proximal, Left, Distal - Spinal Cord, Spinal Roots	0	0	0	0	1		
Vehicle	Day 85	Nerve, Sciatic, Proximal, Left, Distal - Spinal Cord, Thoracic	0	0	0	0	1		
Vehicle	Day 85	Nerve, Sciatic, Proximal, Left, Distal - Spleen	-0.000126212	0.000178491	-0.001729894	0.001477469	1	-1	0.5
Vehicle	Day 85	Nerve, Sciatic, Proximal, Left, Distal - Testis	0				0		
Vehicle	Day 85	Nerve, Sciatic, Proximal, Left, Proximal - Nerve, Sciatic, Proximal, Right, Distal	-0.000163993	0.000231921	-0.002247723	0.001919737	1	-1	0.5
Vehicle	Day 85	Nerve, Sciatic, Proximal, Left, Proximal - Nerve, Sciatic, Proximal, Right, Proximal	-0.000138488	0.000195851	-0.00189814	0.001621165	1	-1	0.5
Vehicle	Day 85	Nerve, Sciatic, Proximal, Left, Proximal - Nerve, Ulnar, Distal, Left, Distal	0	0	0	0	1		
Vehicle	Day 85	Nerve, Sciatic, Proximal, Left, Proximal - Nerve, Ulnar, Distal, Left, Proximal	0	0	0	0	1		
Vehicle	Day 85	Nerve, Sciatic, Proximal, Left, Proximal - Nerve, Ulnar, Distal, Right, Distal	0	0	0	0	1		
Vehicle	Day 85	Nerve, Sciatic, Proximal, Left, Proximal - Nerve, Ulnar, Distal, Right, Proximal	0	0	0	0	1		
Vehicle	Day 85	Nerve, Sciatic, Proximal, Left, Proximal - Nerve, Ulnar, Proximal, Left, Distal	0	0	0	0	1		
Vehicle	Day 85	Nerve, Sciatic, Proximal, Left, Proximal - Nerve, Ulnar, Proximal, Left, Proximal	0	0	0	0	1		
Vehicle	Day 85	Nerve, Sciatic, Proximal, Left, Proximal - Nerve, Ulnar, Proximal, Right, Distal	0	0	0	0	1		
Vehicle	Day 85	Nerve, Sciatic, Proximal, Left, Proximal - Nerve, Ulnar, Proximal, Right, Proximal	0	0	0	0	1		
Vehicle	Day 85	Nerve, Sciatic, Proximal, Left, Proximal - Nerve, Optic	0	0	0	0	1		
Vehicle	Day 85	Nerve, Sciatic, Proximal, Left, Proximal - Ovary	0				0		
Vehicle	Day 85	Nerve, Sciatic, Proximal, Left, Proximal - Spinal Cord, Cervical	0	0	0	0	1		
Vehicle	Day 85	Nerve, Sciatic, Proximal, Left, Proximal - Spinal Cord, Lumbar	0	0	0	0	1		
Vehicle	Day 85	Nerve, Sciatic, Proximal, Left, Proximal - Spinal Cord, Sacral	0	0	0	0	1		
Vehicle	Day 85	Nerve, Sciatic, Proximal, Left, Proximal - Spinal Cord, Spinal Roots	0	0	0	0	1		
Vehicle	Day 85	Nerve, Sciatic, Proximal, Left, Proximal - Spinal Cord, Thoracic	0	0	0	0	1		
Vehicle	Day 85	Nerve, Sciatic, Proximal, Left, Proximal - Spleen	-0.000126212	0.000178491	-0.001729894	0.001477469	1	-1	0.5
Vehicle	Day 85	Nerve, Sciatic, Proximal, Left, Proximal - Testis	0				0		
Vehicle	Day 85	Nerve, Sciatic, Proximal, Right, Distal - Nerve, Sciatic, Proximal, Right, Proximal	2.55055E-05	0.000427772	-0.003817877	0.003868888	1	0.084320989	0.946446274
Vehicle	Day 85	Nerve, Sciatic, Proximal, Right, Distal - Nerve, Ulnar, Distal, Left, Distal	0.000163993	0.000231921	-0.001919737	0.002247723	1	1	0.5
Vehicle	Day 85	Nerve, Sciatic, Proximal, Right, Distal - Nerve, Ulnar, Distal, Left, Proximal	0.000163993	0.000231921	-0.001919737	0.002247723	1	1	0.5
Vehicle	Day 85	Nerve, Sciatic, Proximal, Right, Distal - Nerve, Ulnar, Distal, Right, Distal	0.000163993	0.000231921	-0.001919737	0.002247723	1	1	0.5
Vehicle	Day 85	Nerve, Sciatic, Proximal, Right, Distal - Nerve, Ulnar, Distal, Right, Proximal	0.000163993	0.000231921	-0.001919737	0.002247723	1	1	0.5
Vehicle	Day 85	Nerve, Sciatic, Proximal, Right, Distal - Nerve, Ulnar, Proximal, Left, Distal	0.000163993	0.000231921	-0.001919737	0.002247723	1	1	0.5
Vehicle	Day 85	Nerve, Sciatic, Proximal, Right, Distal - Nerve, Ulnar, Proximal, Left, Proximal	0.000163993	0.000231921	-0.001919737	0.002247723	1	1	0.5
Vehicle	Day 85	Nerve, Sciatic, Proximal, Right, Distal - Nerve, Ulnar, Proximal, Right, Distal	0.000163993	0.000231921	-0.001919737	0.002247723	1	1	0.5
Vehicle	Day 85	Nerve, Sciatic, Proximal, Right, Distal - Nerve, Ulnar, Proximal, Right, Proximal	0.000163993	0.000231921	-0.001919737	0.002247723	1	1	0.5
Vehicle	Day 85	Nerve, Sciatic, Proximal, Right, Distal - Nerve, Optic	0.000163993	0.000231921	-0.001919737	0.002247723	1	1	0.5
Vehicle	Day 85	Nerve, Sciatic, Proximal, Right, Distal - Ovary	0.000327986				0		
Vehicle	Day 85	Nerve, Sciatic, Proximal, Right, Distal - Spinal Cord, Cervical	0.000163993	0.000231921	-0.001919737	0.002247723	1	1	0.5
Vehicle	Day 85	Nerve, Sciatic, Proximal, Right, Distal - Spinal Cord, Lumbar	0.000163993	0.000231921	-0.001919737	0.002247723	1	1	0.5
Vehicle	Day 85	Nerve, Sciatic, Proximal, Right, Distal - Spinal Cord, Sacral	0.000163993	0.000231921	-0.001919737	0.002247723	1	1	0.5
Vehicle	Day 85	Nerve, Sciatic, Proximal, Right, Distal - Spinal Cord, Spinal Roots	0.000163993	0.000231921	-0.001919737	0.002247723	1	1	0.5
Vehicle	Day 85	Nerve, Sciatic, Proximal, Right, Distal - Spinal Cord, Thoracic	0.000163993	0.000231921	-0.001919737	0.002247723	1	1	0.5
Vehicle	Day 85	Nerve, Sciatic, Proximal, Right, Distal - Spleen	3.77807E-05	5.34299E-05	-0.000442268	0.000517829	1	1	0.5
Vehicle	Day 85	Nerve, Sciatic, Proximal, Right, Distal - Testis	0				0		
Vehicle	Day 85	Nerve, Sciatic, Proximal, Right, Proximal - Nerve, Ulnar, Distal, Left, Distal	0.000138488	0.000195851	-0.001621165	0.00189814	1	1	0.5

Results of Evaluating Mean Differences Between Different Tissues Across Timepoint and Group for Log10 AAV2-ITR Target Copies/ug of DNA for Normalized Data

Group	Timepoint	Difference	Mean	StdDev	LowerCLMean	UpperCLMean	DF	tValue	P-Value
Vehicle	Day 85	Nerve, Sciatic, Proximal, Right, Proximal - Nerve, Ulnar, Distal, Left, Proximal	0.000138488	0.000195851	-0.001621165	0.00189814	1	1	0.5
Vehicle	Day 85	Nerve, Sciatic, Proximal, Right, Proximal - Nerve, Ulnar, Distal, Right, Distal	0.000138488	0.000195851	-0.001621165	0.00189814	1	1	0.5
Vehicle	Day 85	Nerve, Sciatic, Proximal, Right, Proximal - Nerve, Ulnar, Distal, Right, Proximal	0.000138488	0.000195851	-0.001621165	0.00189814	1	1	0.5
Vehicle	Day 85	Nerve, Sciatic, Proximal, Right, Proximal - Nerve, Ulnar, Proximal, Left, Distal	0.000138488	0.000195851	-0.001621165	0.00189814	1	1	0.5
Vehicle	Day 85	Nerve, Sciatic, Proximal, Right, Proximal - Nerve, Ulnar, Proximal, Left, Proximal	0.000138488	0.000195851	-0.001621165	0.00189814	1	1	0.5
Vehicle	Day 85	Nerve, Sciatic, Proximal, Right, Proximal - Nerve, Ulnar, Proximal, Right, Distal	0.000138488	0.000195851	-0.001621165	0.00189814	1	1	0.5
Vehicle	Day 85	Nerve, Sciatic, Proximal, Right, Proximal - Nerve, Ulnar, Proximal, Right, Proximal	0.000138488	0.000195851	-0.001621165	0.00189814	1	1	0.5
Vehicle	Day 85	Nerve, Sciatic, Proximal, Right, Proximal - Nerve, Optic	0.000138488	0.000195851	-0.001621165	0.00189814	1	1	0.5
Vehicle	Day 85	Nerve, Sciatic, Proximal, Right, Proximal - Ovary	0				0		
Vehicle	Day 85	Nerve, Sciatic, Proximal, Right, Proximal - Spinal Cord, Cervical	0.000138488	0.000195851	-0.001621165	0.00189814	1	1	0.5
Vehicle	Day 85	Nerve, Sciatic, Proximal, Right, Proximal - Spinal Cord, Lumbar	0.000138488	0.000195851	-0.001621165	0.00189814	1	1	0.5
Vehicle	Day 85	Nerve, Sciatic, Proximal, Right, Proximal - Spinal Cord, Sacral	0.000138488	0.000195851	-0.001621165	0.00189814	1	1	0.5
Vehicle	Day 85	Nerve, Sciatic, Proximal, Right, Proximal - Spinal Cord, Spinal Roots	0.000138488	0.000195851	-0.001621165	0.00189814	1	1	0.5
Vehicle	Day 85	Nerve, Sciatic, Proximal, Right, Proximal - Spinal Cord, Thoracic	0.000138488	0.000195851	-0.001621165	0.00189814	1	1	0.5
Vehicle	Day 85	Nerve, Sciatic, Proximal, Right, Proximal - Spleen	1.22752E-05	0.000374342	-0.003351059	0.003375609	1	0.046373922	0.97049858
Vehicle	Day 85	Nerve, Sciatic, Proximal, Right, Proximal - Testis	0.000276975				0		
Vehicle	Day 85	Nerve, Ulnar, Distal, Left, Distal - Nerve, Ulnar, Distal, Left, Proximal	0	0	0	0	1		
Vehicle	Day 85	Nerve, Ulnar, Distal, Left, Distal - Nerve, Ulnar, Distal, Right, Distal	0	0	0	0	1		
Vehicle	Day 85	Nerve, Ulnar, Distal, Left, Distal - Nerve, Ulnar, Distal, Right, Proximal	0	0	0	0	1		
Vehicle	Day 85	Nerve, Ulnar, Distal, Left, Distal - Nerve, Ulnar, Proximal, Left, Distal	0	0	0	0	1		
Vehicle	Day 85	Nerve, Ulnar, Distal, Left, Distal - Nerve, Ulnar, Proximal, Left, Proximal	0	0	0	0	1		
Vehicle	Day 85	Nerve, Ulnar, Distal, Left, Distal - Nerve, Ulnar, Proximal, Right, Distal	0	0	0	0	1		
Vehicle	Day 85	Nerve, Ulnar, Distal, Left, Distal - Nerve, Ulnar, Proximal, Right, Proximal	0	0	0	0	1		
Vehicle	Day 85	Nerve, Ulnar, Distal, Left, Distal - Nerve, Optic	0	0	0	0	1		
Vehicle	Day 85	Nerve, Ulnar, Distal, Left, Distal - Ovary	0				0		
Vehicle	Day 85	Nerve, Ulnar, Distal, Left, Distal - Spinal Cord, Cervical	0	0	0	0	1		
Vehicle	Day 85	Nerve, Ulnar, Distal, Left, Distal - Spinal Cord, Lumbar	0	0	0	0	1		
Vehicle	Day 85	Nerve, Ulnar, Distal, Left, Distal - Spinal Cord, Sacral	0	0	0	0	1		
Vehicle	Day 85	Nerve, Ulnar, Distal, Left, Distal - Spinal Cord, Spinal Roots	0	0	0	0	1		
Vehicle	Day 85	Nerve, Ulnar, Distal, Left, Distal - Spinal Cord, Thoracic	0	0	0	0	1		
Vehicle	Day 85	Nerve, Ulnar, Distal, Left, Distal - Spleen	-0.000126212	0.000178491	-0.001729894	0.001477469	1	-1	0.5
Vehicle	Day 85	Nerve, Ulnar, Distal, Left, Distal - Testis	0				0		
Vehicle	Day 85	Nerve, Ulnar, Distal, Left, Proximal - Nerve, Ulnar, Distal, Right, Distal	0	0	0	0	1		
Vehicle	Day 85	Nerve, Ulnar, Distal, Left, Proximal - Nerve, Ulnar, Distal, Right, Proximal	0	0	0	0	1		
Vehicle	Day 85	Nerve, Ulnar, Distal, Left, Proximal - Nerve, Ulnar, Proximal, Left, Distal	0	0	0	0	1		
Vehicle	Day 85	Nerve, Ulnar, Distal, Left, Proximal - Nerve, Ulnar, Proximal, Left, Proximal	0	0	0	0	1		
Vehicle	Day 85	Nerve, Ulnar, Distal, Left, Proximal - Nerve, Ulnar, Proximal, Right, Distal	0	0	0	0	1		
Vehicle	Day 85	Nerve, Ulnar, Distal, Left, Proximal - Nerve, Ulnar, Proximal, Right, Proximal	0	0	0	0	1		
Vehicle	Day 85	Nerve, Ulnar, Distal, Left, Proximal - Nerve, Optic	0	0	0	0	1		
Vehicle	Day 85	Nerve, Ulnar, Distal, Left, Proximal - Ovary	0				0		
Vehicle	Day 85	Nerve, Ulnar, Distal, Left, Proximal - Spinal Cord, Cervical	0	0	0	0	1		
Vehicle	Day 85	Nerve, Ulnar, Distal, Left, Proximal - Spinal Cord, Lumbar	0	0	0	0	1		
Vehicle	Day 85	Nerve, Ulnar, Distal, Left, Proximal - Spinal Cord, Sacral	0	0	0	0	1		
Vehicle	Day 85	Nerve, Ulnar, Distal, Left, Proximal - Spinal Cord, Spinal Roots	0	0	0	0	1		
Vehicle	Day 85	Nerve, Ulnar, Distal, Left, Proximal - Spinal Cord, Thoracic	0	0	0	0	1		
Vehicle	Day 85	Nerve, Ulnar, Distal, Left, Proximal - Spleen	-0.000126212	0.000178491	-0.001729894	0.001477469	1	-1	0.5
Vehicle	Day 85	Nerve, Ulnar, Distal, Left, Proximal - Testis	0				0		
Vehicle	Day 85	Nerve, Ulnar, Distal, Right, Distal - Nerve, Ulnar, Distal, Right, Proximal	0	0	0	0	1		
Vehicle	Day 85	Nerve, Ulnar, Distal, Right, Distal - Nerve, Ulnar, Proximal, Left, Distal	0	0	0	0	1		
Vehicle	Day 85	Nerve, Ulnar, Distal, Right, Distal - Nerve, Ulnar, Proximal, Left, Proximal	0	0	0	0	1		
Vehicle	Day 85	Nerve, Ulnar, Distal, Right, Distal - Nerve, Ulnar, Proximal, Right, Distal	0	0	0	0	1		
Vehicle	Day 85	Nerve, Ulnar, Distal, Right, Distal - Nerve, Ulnar, Proximal, Right, Proximal	0	0	0	0	1		
Vehicle	Day 85	Nerve, Ulnar, Distal, Right, Distal - Nerve, Optic	0	0	0	0	1		
Vehicle	Day 85	Nerve, Ulnar, Distal, Right, Distal - Ovary	0				0		
Vehicle	Day 85	Nerve, Ulnar, Distal, Right, Distal - Spinal Cord, Cervical	0	0	0	0	1		
Vehicle	Day 85	Nerve, Ulnar, Distal, Right, Distal - Spinal Cord, Lumbar	0	0	0	0	1		
Vehicle	Day 85	Nerve, Ulnar, Distal, Right, Distal - Spinal Cord, Sacral	0	0	0	0	1		
Vehicle	Day 85	Nerve, Ulnar, Distal, Right, Distal - Spinal Cord, Spinal Roots	0	0	0	0	1		
Vehicle	Day 85	Nerve, Ulnar, Distal, Right, Distal - Spinal Cord, Thoracic	0	0	0	0	1		
Vehicle	Day 85	Nerve, Ulnar, Distal, Right, Distal - Spleen	-0.000126212	0.000178491	-0.001729894	0.001477469	1	-1	0.5
Vehicle	Day 85	Nerve, Ulnar, Distal, Right, Distal - Testis	0				0		
Vehicle	Day 85	Nerve, Ulnar, Distal, Right, Proximal - Nerve, Ulnar, Proximal, Left, Distal	0	0	0	0	1		
Vehicle	Day 85	Nerve, Ulnar, Distal, Right, Proximal - Nerve, Ulnar, Proximal, Left, Proximal	0	0	0	0	1		
Vehicle	Day 85	Nerve, Ulnar, Distal, Right, Proximal - Nerve, Ulnar, Proximal, Right, Distal	0	0	0	0	1		
Vehicle	Day 85	Nerve, Ulnar, Distal, Right, Proximal - Nerve, Ulnar, Proximal, Right, Proximal	0	0	0	0	1		
Vehicle	Day 85	Nerve, Ulnar, Distal, Right, Proximal - Nerve, Optic	0	0	0	0	1		
Vehicle	Day 85	Nerve, Ulnar, Distal, Right, Proximal - Ovary	0				0		
Vehicle	Day 85	Nerve, Ulnar, Distal, Right, Proximal - Spinal Cord, Cervical	0	0	0	0	1		
Vehicle	Day 85	Nerve, Ulnar, Distal, Right, Proximal - Spinal Cord, Lumbar	0	0	0	0	1		
Vehicle	Day 85	Nerve, Ulnar, Distal, Right, Proximal - Spinal Cord, Sacral	0	0	0	0	1		
Vehicle	Day 85	Nerve, Ulnar, Distal, Right, Proximal - Spinal Cord, Spinal Roots	0	0	0	0	1		
Vehicle	Day 85	Nerve, Ulnar, Distal, Right, Proximal - Spinal Cord, Thoracic	0	0	0	0	1		
Vehicle	Day 85	Nerve, Ulnar, Distal, Right, Proximal - Spleen	-0.000126212	0.000178491	-0.001729894	0.001477469	1	-1	0.5
Vehicle	Day 85	Nerve, Ulnar, Distal, Right, Proximal - Testis	0				0		
Vehicle	Day 85	Nerve, Ulnar, Proximal, Left, Distal - Nerve, Ulnar, Proximal, Left, Proximal	0	0	0	0	1		
Vehicle	Day 85	Nerve, Ulnar, Proximal, Left, Distal - Nerve, Ulnar, Proximal, Right, Distal	0	0	0	0	1		
Vehicle	Day 85	Nerve, Ulnar, Proximal, Left, Distal - Nerve, Ulnar, Proximal, Right, Proximal	0	0	0	0	1		
Vehicle	Day 85	Nerve, Ulnar, Proximal, Left, Distal - Nerve, Optic	0	0	0	0	1		
Vehicle	Day 85	Nerve, Ulnar, Proximal, Left, Distal - Ovary	0				0		
Vehicle	Day 85	Nerve, Ulnar, Proximal, Left, Distal - Spinal Cord, Cervical	0	0	0	0	1		
Vehicle	Day 85	Nerve, Ulnar, Proximal, Left, Distal - Spinal Cord, Lumbar	0	0	0	0	1		
Vehicle	Day 85	Nerve, Ulnar, Proximal, Left, Distal - Spinal Cord, Sacral	0	0	0	0	1		
Vehicle	Day 85	Nerve, Ulnar, Proximal, Left, Distal - Spinal Cord, Spinal Roots	0	0	0	0	1		
Vehicle	Day 85	Nerve, Ulnar, Proximal, Left, Distal - Spinal Cord, Thoracic	0	0	0	0	1		
Vehicle	Day 85	Nerve, Ulnar, Proximal, Left, Distal - Spleen	-0.000126212	0.000178491	-0.001729894	0.001477469	1	-1	0.5
Vehicle	Day 85	Nerve, Ulnar, Proximal, Left, Distal - Testis	0				0		
Vehicle	Day 85	Nerve, Ulnar, Proximal, Left, Proximal - Nerve, Ulnar, Proximal, Right, Distal	0	0	0	0	1		
Vehicle	Day 85	Nerve, Ulnar, Proximal, Left, Proximal - Nerve, Ulnar, Proximal, Right, Proximal	0	0	0	0	1		
Vehicle	Day 85	Nerve, Ulnar, Proximal, Left, Proximal - Nerve, Optic	0	0	0	0	1		
Vehicle	Day 85	Nerve, Ulnar, Proximal, Left, Proximal - Ovary	0				0		
Vehicle	Day 85	Nerve, Ulnar, Proximal, Left, Proximal - Spinal Cord, Cervical	0	0	0	0	1		
Vehicle	Day 85	Nerve, Ulnar, Proximal, Left, Proximal - Spinal Cord, Lumbar	0	0	0	0	1		
Vehicle	Day 85	Nerve, Ulnar, Proximal, Left, Proximal - Spinal Cord, Sacral	0	0	0	0	1		

Results of Evaluating Mean Differences Between Different Tissues Across Timepoint and Group for Log10 AAV2-ITR Target Copies/ug of DNA for Normalized Data

Group	Timepoint	Difference	Mean	StdDev	LowerCLMean	UpperCLMean	DF	tValue	P-Value
Vehicle	Day 85	Nerve, Ulnar, Proximal, Left, Proximal - Spinal Cord, Spinal Roots	0	0	0	0	1		
Vehicle	Day 85	Nerve, Ulnar, Proximal, Left, Proximal - Spinal Cord, Thoracic	0	0	0	0	1		
Vehicle	Day 85	Nerve, Ulnar, Proximal, Left, Proximal - Spleen	-0.000126212	0.000178491	-0.001729894	0.001477469	1	-1	0.5
Vehicle	Day 85	Nerve, Ulnar, Proximal, Left, Proximal - Testis	0				0		
Vehicle	Day 85	Nerve, Ulnar, Proximal, Right, Distal - Nerve, Ulnar, Proximal, Right, Proximal	0	0	0	0	1		
Vehicle	Day 85	Nerve, Ulnar, Proximal, Right, Distal - Nerve, Optic	0	0	0	0	1		
Vehicle	Day 85	Nerve, Ulnar, Proximal, Right, Distal - Ovary	0				0		
Vehicle	Day 85	Nerve, Ulnar, Proximal, Right, Distal - Spinal Cord, Cervical	0	0	0	0	1		
Vehicle	Day 85	Nerve, Ulnar, Proximal, Right, Distal - Spinal Cord, Lumbar	0	0	0	0	1		
Vehicle	Day 85	Nerve, Ulnar, Proximal, Right, Distal - Spinal Cord, Sacral	0	0	0	0	1		
Vehicle	Day 85	Nerve, Ulnar, Proximal, Right, Distal - Spinal Cord, Spinal Roots	0	0	0	0	1		
Vehicle	Day 85	Nerve, Ulnar, Proximal, Right, Distal - Spinal Cord, Thoracic	0	0	0	0	1		
Vehicle	Day 85	Nerve, Ulnar, Proximal, Right, Distal - Spleen	-0.000126212	0.000178491	-0.001729894	0.001477469	1	-1	0.5
Vehicle	Day 85	Nerve, Ulnar, Proximal, Right, Distal - Testis	0				0		
Vehicle	Day 85	Nerve, Ulnar, Proximal, Right, Proximal - Nerve, Optic	0	0	0	0	1		
Vehicle	Day 85	Nerve, Ulnar, Proximal, Right, Proximal - Ovary	0				0		
Vehicle	Day 85	Nerve, Ulnar, Proximal, Right, Proximal - Spinal Cord, Cervical	0	0	0	0	1		
Vehicle	Day 85	Nerve, Ulnar, Proximal, Right, Proximal - Spinal Cord, Lumbar	0	0	0	0	1		
Vehicle	Day 85	Nerve, Ulnar, Proximal, Right, Proximal - Spinal Cord, Sacral	0	0	0	0	1		
Vehicle	Day 85	Nerve, Ulnar, Proximal, Right, Proximal - Spinal Cord, Spinal Roots	0	0	0	0	1		
Vehicle	Day 85	Nerve, Ulnar, Proximal, Right, Proximal - Spinal Cord, Thoracic	0	0	0	0	1		
Vehicle	Day 85	Nerve, Ulnar, Proximal, Right, Proximal - Spleen	-0.000126212	0.000178491	-0.001729894	0.001477469	1	-1	0.5
Vehicle	Day 85	Nerve, Ulnar, Proximal, Right, Proximal - Testis	0				0		
Vehicle	Day 85	Nerve, Optic - Ovary	0				0		
Vehicle	Day 85	Nerve, Optic - Spinal Cord, Cervical	0	0	0	0	1		
Vehicle	Day 85	Nerve, Optic - Spinal Cord, Lumbar	0	0	0	0	1		
Vehicle	Day 85	Nerve, Optic - Spinal Cord, Sacral	0	0	0	0	1		
Vehicle	Day 85	Nerve, Optic - Spinal Cord, Spinal Roots	0	0	0	0	1		
Vehicle	Day 85	Nerve, Optic - Spinal Cord, Thoracic	0	0	0	0	1		
Vehicle	Day 85	Nerve, Optic - Spleen	-0.000126212	0.000178491	-0.001729894	0.001477469	1	-1	0.5
Vehicle	Day 85	Nerve, Optic - Testis	0				0		
Vehicle	Day 85	Ovary - Spinal Cord, Cervical	0				0		
Vehicle	Day 85	Ovary - Spinal Cord, Lumbar	0				0		
Vehicle	Day 85	Ovary - Spinal Cord, Sacral	0				0		
Vehicle	Day 85	Ovary - Spinal Cord, Spinal Roots	0				0		
Vehicle	Day 85	Ovary - Spinal Cord, Thoracic	0				0		
Vehicle	Day 85	Ovary - Spleen	-0.000252425				0		
Vehicle	Day 85	Ovary - Testis					0		
Vehicle	Day 85	Spinal Cord, Cervical - Spinal Cord, Lumbar	0	0	0	0	1		
Vehicle	Day 85	Spinal Cord, Cervical - Spinal Cord, Sacral	0	0	0	0	1		
Vehicle	Day 85	Spinal Cord, Cervical - Spinal Cord, Spinal Roots	0	0	0	0	1		
Vehicle	Day 85	Spinal Cord, Cervical - Spinal Cord, Thoracic	0	0	0	0	1		
Vehicle	Day 85	Spinal Cord, Cervical - Spleen	-0.000126212	0.000178491	-0.001729894	0.001477469	1	-1	0.5
Vehicle	Day 85	Spinal Cord, Cervical - Testis	0				0		
Vehicle	Day 85	Spinal Cord, Lumbar - Spinal Cord, Sacral	0	0	0	0	1		
Vehicle	Day 85	Spinal Cord, Lumbar - Spinal Cord, Spinal Roots	0	0	0	0	1		
Vehicle	Day 85	Spinal Cord, Lumbar - Spinal Cord, Thoracic	0	0	0	0	1		
Vehicle	Day 85	Spinal Cord, Lumbar - Spleen	-0.000126212	0.000178491	-0.001729894	0.001477469	1	-1	0.5
Vehicle	Day 85	Spinal Cord, Lumbar - Testis	0				0		
Vehicle	Day 85	Spinal Cord, Sacral - Spinal Cord, Spinal Roots	0	0	0	0	1		
Vehicle	Day 85	Spinal Cord, Sacral - Spinal Cord, Thoracic	0	0	0	0	1		
Vehicle	Day 85	Spinal Cord, Sacral - Spleen	-0.000126212	0.000178491	-0.001729894	0.001477469	1	-1	0.5
Vehicle	Day 85	Spinal Cord, Sacral - Testis	0				0		
Vehicle	Day 85	Spinal Cord, Spinal Roots - Spinal Cord, Thoracic	0	0	0	0	1		
Vehicle	Day 85	Spinal Cord, Spinal Roots - Spleen	-0.000126212	0.000178491	-0.001729894	0.001477469	1	-1	0.5
Vehicle	Day 85	Spinal Cord, Spinal Roots - Testis	0				0		
Vehicle	Day 85	Spinal Cord, Thoracic - Spleen	-0.000126212	0.000178491	-0.001729894	0.001477469	1	-1	0.5
Vehicle	Day 85	Spinal Cord, Thoracic - Testis	0				0		
Vehicle	Day 85	Spleen - Testis	0				0		
Low Dose	Day 43	Brain - Ganglion, Dorsal Root, Cervical	-1.438735723	0.628309024	-2.438515588	-0.438955858	3	-4.579707339	0.019544239
Low Dose	Day 43	Brain - Ganglion, Dorsal Root, Lumbar	-0.934219772	0.890844825	-2.351752683	0.483313139	3	-2.097379355	0.126881437
Low Dose	Day 43	Brain - Ganglion, Dorsal Root, Thoracic	-0.989686596	0.39876678	-1.62421353	-0.355159663	3	-4.963736426	0.015702461
Low Dose	Day 43	Brain - Heart	0.316757443	0.509631124	-0.494179401	1.127694286	3	1.243085156	0.302135512
Low Dose	Day 43	Brain - Kidney	0.37776265	0.506837779	-0.428729358	1.184254658	3	1.490664927	0.232836595
Low Dose	Day 43	Brain - Lung	0.408783812	0.410810277	-0.244907012	1.062474636	3	1.990134302	0.140665568
Low Dose	Day 43	Brain - Liver	-0.630371034	1.154872184	-2.468030392	1.207288323	3	-1.091672383	0.35480523
Low Dose	Day 43	Brain - Muscle, Skeletal, Tibialis Anterior Left	0.433004701	0.471246328	-0.316853366	1.182862769	3	1.837700055	0.16341154
Low Dose	Day 43	Brain - Muscle, Skeletal, Tibialis Anterior Right	0.421139916	0.467867588	-0.323341822	1.165621654	3	1.800252579	0.169637019
Low Dose	Day 43	Brain - Muscle, Skeletal, Bicep Brachii Left	0.443728896	0.452109119	-0.275677602	1.163135395	3	1.962928317	0.144439003
Low Dose	Day 43	Brain - Muscle, Skeletal, Bicep Brachii Right	0.424430951	0.479878591	-0.339162973	1.188024876	3	1.768909717	0.175058441
Low Dose	Day 43	Brain - Nerve, Femoral, Distal, Left, Distal	0.237019308	0.491083716	-0.544404471	1.018443087	3	0.965290845	0.405604816
Low Dose	Day 43	Brain - Nerve, Femoral, Distal, Left, Proximal	0.157850544	0.511244597	-0.655653696	0.971354784	3	0.617514767	0.580601601
Low Dose	Day 43	Brain - Nerve, Femoral, Distal, Right, Distal	0.226376278	0.449731122	-0.489246295	0.941998851	3	1.006718313	0.38823354
Low Dose	Day 43	Brain - Nerve, Femoral, Distal, Right, Proximal	0.174160372	0.582249015	-0.752327741	1.100648485	3	0.598233291	0.59183719
Low Dose	Day 43	Brain - Nerve, Femoral, Proximal, Left, Distal	0.252990767	0.523183791	-0.579511394	1.085492928	3	0.967120051	0.404822676
Low Dose	Day 43	Brain - Nerve, Femoral, Proximal, Left, Proximal	0.324844354	0.559989795	-0.566224373	1.215913081	3	1.16017955	0.329916623
Low Dose	Day 43	Brain - Nerve, Femoral, Proximal, Right, Distal	0.254704642	0.486781472	-0.519873307	1.02928259	3	1.046484536	0.372224185
Low Dose	Day 43	Brain - Nerve, Femoral, Proximal, Right, Proximal	0.312394442	0.540340006	-0.547407086	1.172195971	3	1.156288406	0.331282428
Low Dose	Day 43	Brain - Nerve Median, Distal, Left, Distal	0.379577574	0.42899166	-0.303043887	1.062199035	3	1.769626822	0.174932197
Low Dose	Day 43	Brain - Nerve Median, Distal, Left, Proximal	0.311486271	0.525273248	-0.524340683	1.147313226	3	1.185997087	0.320997238
Low Dose	Day 43	Brain - Nerve Median, Distal, Right, Distal	0.419648886	0.429851766	-0.264341196	1.103638968	3	1.952528381	0.14591281
Low Dose	Day 43	Brain - Nerve Median, Distal, Right, Proximal	0.351522251	0.427665534	-0.328989048	1.03203355	3	1.643911999	0.198740686
Low Dose	Day 43	Brain - Nerve Median, Proximal, Left, Distal	0.369568984	0.431491249	-0.317029881	1.056167849	3	1.712984841	0.185232841
Low Dose	Day 43	Brain - Nerve Median, Proximal, Left, Proximal	0.316172217	0.541280519	-0.545125878	1.177470311	3	1.168237929	0.327106094
Low Dose	Day 43	Brain - Nerve Median, Proximal, Right, Distal	0.222290407	0.189332171	-0.078979327	0.523560141	3	2.348152518	0.100474253
Low Dose	Day 43	Brain - Nerve Median, Proximal, Right, Proximal	0.287713686	0.54473206	-0.57907658	1.154503953	3	1.056349378	0.368352003
Low Dose	Day 43	Brain - Nerve, Sciatic, Distal, Left, Distal	0.343664829	0.456913815	-0.383387012	1.070716671	3	1.504287321	0.229557278
Low Dose	Day 43	Brain - Nerve, Sciatic, Distal, Left, Proximal	0.157910904	0.591907466	-0.78394596	1.099767767	3	0.533566183	0.630643059
Low Dose	Day 43	Brain - Nerve, Sciatic, Distal, Right, Distal	0.393099756	0.443882853	-0.313216917	1.09941643	3	1.77118694	0.174657905
Low Dose	Day 43	Brain - Nerve, Sciatic, Distal, Right, Proximal	0.338450065	0.488787619	-0.439320111	1.116220241	3	1.384855312	0.260115082
Low Dose	Day 43	Brain - Nerve, Sciatic, Proximal, Left, Distal	0.294178898	0.516669838	-0.52795811	1.116315907	3	1.138750036	0.33750918
Low Dose	Day 43	Brain - Nerve, Sciatic, Proximal, Left, Proximal	0.261360986	0.519574291	-0.565397656	1.088119627	3	1.006058192	0.388504757
Low Dose	Day 43	Brain - Nerve, Sciatic, Proximal, Right, Distal	0.243664175	0.468706884	-0.502153071	0.98948142	3	1.039729447	0.374898339

Results of Evaluating Mean Differences Between Different Tissues Across Timepoint and Group for Log10 AAV2-ITR Target Copies/ug of DNA for Normalized Data

Group	Timepoint	Difference	Mean	StdDev	LowerCLMean	UpperCLMean	DF	tValue	P-Value
Low Dose	Day 43	Brain - Nerve, Sciatic, Proximal, Right, Proximal	0.361558304	0.399348739	-0.273894655	0.997011264	3	1.810739682	0.167866392
Low Dose	Day 43	Brain - Nerve, Ulnar, Distal, Left, Distal	0.423715819	0.447698098	-0.28867176	1.136103398	3	1.892864058	0.154716528
Low Dose	Day 43	Brain - Nerve, Ulnar, Distal, Left, Proximal	0.282616509	0.525721658	-0.553923964	1.119156982	3	1.075156425	0.361077699
Low Dose	Day 43	Brain - Nerve, Ulnar, Distal, Right, Distal	0.417499071	0.439085193	-0.281183454	1.116181596	3	1.901676838	0.153377878
Low Dose	Day 43	Brain - Nerve, Ulnar, Distal, Right, Proximal	0.381531153	0.413460168	-0.276376239	1.039438545	3	1.845552161	0.162139905
Low Dose	Day 43	Brain - Nerve, Ulnar, Proximal, Left, Distal	0.292813314	0.428786083	-0.38948103	0.975107657	3	1.365778065	0.265390191
Low Dose	Day 43	Brain - Nerve, Ulnar, Proximal, Left, Proximal	0.344745439	0.486851385	-0.429943757	1.119434635	3	1.416224538	0.251683921
Low Dose	Day 43	Brain - Nerve, Ulnar, Proximal, Right, Distal	0.308246851	0.484101836	-0.462067199	1.078560901	3	1.273479372	0.292562538
Low Dose	Day 43	Brain - Nerve, Ulnar, Proximal, Right, Proximal	0.300828904	0.563627757	-0.596028633	1.19768644	3	1.067473699	0.36403224
Low Dose	Day 43	Brain - Nerve, Optic	0.106843973	0.33941255	-0.433237135	0.64692508	3	0.629581744	0.573649846
Low Dose	Day 43	Brain - Ovary	0.283818755	0.131584499	-0.89842106	1.466058569	1	3.05036183	0.201674376
Low Dose	Day 43	Brain - Spinal Cord, Cervical	-1.180700492	0.754071326	-2.380596245	0.019195262	3	-3.131535307	0.052003569
Low Dose	Day 43	Brain - Spinal Cord, Lumbar	-0.856796638	0.671041832	-1.924573938	0.210980662	3	-2.553631076	0.08367737
Low Dose	Day 43	Brain - Spinal Cord, Sacral	-0.973450746	0.781184639	-2.21648983	0.269588338	3	-2.492242416	0.08830901
Low Dose	Day 43	Brain - Spinal Cord, Spinal Roots	-0.449830386	0.607691891	-1.416803793	0.517143022	3	-1.480455448	0.235327982
Low Dose	Day 43	Brain - Spinal Cord, Thoracic	-1.270715965	1.299468605	-3.338460495	0.797028566	3	-1.955747079	0.1454548
Low Dose	Day 43	Brain - Spleen	-0.263210726	0.582363855	-1.189881575	0.663460122	3	-0.90393909	0.432659068
Low Dose	Day 43	Brain - Testis	0.637525176	0.673750497	-5.41588287	6.690933222	1	1.338176008	0.408558245
Low Dose	Day 43	Ganglion, Dorsal Root, Cervical - Ganglion, Dorsal Root, Lumbar	0.504515951	0.389970562	-0.116014237	1.125046139	3	2.587456591	0.081252855
Low Dose	Day 43	Ganglion, Dorsal Root, Cervical - Ganglion, Dorsal Root, Thoracic	0.449049127	0.263533917	0.029707856	0.868390398	3	3.407903856	0.042215566
Low Dose	Day 43	Ganglion, Dorsal Root, Cervical - Heart	1.755493166	0.663582699	0.699585011	2.811401321	3	5.290955197	0.013172221
Low Dose	Day 43	Ganglion, Dorsal Root, Cervical - Kidney	1.816498373	0.71217322	0.683271856	2.94972489	3	5.101282445	0.014567775
Low Dose	Day 43	Ganglion, Dorsal Root, Cervical - Lung	1.847519535	0.652890187	0.808625553	2.886413517	3	5.659510809	0.010923322
Low Dose	Day 43	Ganglion, Dorsal Root, Cervical - Liver	0.808364689	1.188433285	-1.082697869	2.699427246	3	1.360387157	0.266901455
Low Dose	Day 43	Ganglion, Dorsal Root, Cervical - Muscle, Skeletal, Tibialis Anterior Left	1.871740424	0.702261129	0.754286256	2.989194593	3	5.330610925	0.012902769
Low Dose	Day 43	Ganglion, Dorsal Root, Cervical - Muscle, Skeletal, Tibialis Anterior Right	1.859875639	0.649052998	0.827087481	2.892663797	3	5.73104398	0.010546399
Low Dose	Day 43	Ganglion, Dorsal Root, Cervical - Muscle, Skeletal, Bicep Brachii Left	1.88246462	0.662862978	0.827701702	2.937227538	3	5.679800144	0.010814645
Low Dose	Day 43	Ganglion, Dorsal Root, Cervical - Muscle, Skeletal, Bicep Brachii Right	1.863166674	0.709457037	0.734262211	2.992071137	3	5.252373511	0.013441472
Low Dose	Day 43	Ganglion, Dorsal Root, Cervical - Nerve, Femoral, Distal, Left, Distal	1.675755031	0.693467772	0.572293056	2.779217005	3	4.83297162	0.016890283
Low Dose	Day 43	Ganglion, Dorsal Root, Cervical - Nerve, Femoral, Distal, Left, Proximal	1.596586267	0.733650279	0.429184957	2.763987577	3	4.352445063	0.022404452
Low Dose	Day 43	Ganglion, Dorsal Root, Cervical - Nerve, Femoral, Distal, Right, Distal	1.665112001	0.694503627	0.560001751	2.770222252	3	4.795113911	0.017255869
Low Dose	Day 43	Ganglion, Dorsal Root, Cervical - Nerve, Femoral, Distal, Right, Proximal	1.612896095	0.905798323	0.171568831	3.054223359	3	3.561269774	0.037785981
Low Dose	Day 43	Ganglion, Dorsal Root, Cervical - Nerve, Femoral, Proximal, Left, Distal	1.69172649	0.765864715	0.473064824	2.910388157	3	4.417820686	0.021529043
Low Dose	Day 43	Ganglion, Dorsal Root, Cervical - Nerve, Femoral, Proximal, Left, Proximal	1.763580077	0.872935829	0.374544375	3.152615779	3	4.040572098	0.027277433
Low Dose	Day 43	Ganglion, Dorsal Root, Cervical - Nerve, Femoral, Proximal, Right, Distal	1.693440365	0.795282955	0.427967714	2.958913016	3	4.258711579	0.023741659
Low Dose	Day 43	Ganglion, Dorsal Root, Cervical - Nerve, Femoral, Proximal, Right, Proximal	1.751130165	0.793688076	0.488195324	3.014065007	3	4.412640732	0.021596772
Low Dose	Day 43	Ganglion, Dorsal Root, Cervical - Nerve Median, Distal, Left, Distal	1.818313297	0.674757994	0.744622755	2.892003839	3	5.38952725	0.012515589
Low Dose	Day 43	Ganglion, Dorsal Root, Cervical - Nerve Median, Distal, Left, Proximal	1.750221994	0.891002031	0.332438934	3.168005055	3	3.928659944	0.029356124
Low Dose	Day 43	Ganglion, Dorsal Root, Cervical - Nerve Median, Distal, Right, Distal	1.858384609	0.687322738	0.764700755	2.952068463	3	5.407604045	0.012399843
Low Dose	Day 43	Ganglion, Dorsal Root, Cervical - Nerve Median, Distal, Right, Proximal	1.790257974	0.723996252	0.638218377	2.942297572	3	4.945489623	0.015861534
Low Dose	Day 43	Ganglion, Dorsal Root, Cervical - Nerve Median, Proximal, Left, Distal	1.808304707	0.743166042	0.625761696	2.990847719	3	4.866489065	0.016574976
Low Dose	Day 43	Ganglion, Dorsal Root, Cervical - Nerve Median, Proximal, Left, Proximal	1.75490794	0.810053288	0.465932392	3.043883487	3	4.332820976	0.022676188
Low Dose	Day 43	Ganglion, Dorsal Root, Cervical - Nerve Median, Proximal, Right, Distal	1.66102613	0.718380787	0.51792199	2.80413027	3	4.624361232	0.019038868
Low Dose	Day 43	Ganglion, Dorsal Root, Cervical - Nerve Median, Proximal, Right, Proximal	1.72644941	0.750710145	0.531902046	2.920996773	3	4.59951	0.019318
Low Dose	Day 43	Ganglion, Dorsal Root, Cervical - Nerve, Sciatic, Distal, Left, Distal	1.782400553	0.685467459	0.691668862	2.873132243	3	5.200540244	0.013814627
Low Dose	Day 43	Ganglion, Dorsal Root, Cervical - Nerve, Sciatic, Distal, Left, Proximal	1.596646627	0.751573559	0.400725379	2.792567874	3	4.248810001	0.023888877
Low Dose	Day 43	Ganglion, Dorsal Root, Cervical - Nerve, Sciatic, Distal, Right, Distal	1.83183548	0.667829268	0.769170087	2.894500872	3	5.485939501	0.011914126
Low Dose	Day 43	Ganglion, Dorsal Root, Cervical - Nerve, Sciatic, Distal, Right, Proximal	1.777185788	0.628129765	0.777691164	2.776680413	3	5.658658094	0.010927921
Low Dose	Day 43	Ganglion, Dorsal Root, Cervical - Nerve, Sciatic, Proximal, Left, Distal	1.732914621	0.769911373	0.507813819	2.958015424	3	4.501595071	0.020470902
Low Dose	Day 43	Ganglion, Dorsal Root, Cervical - Nerve, Sciatic, Proximal, Left, Proximal	1.700096709	0.788619033	0.445227846	2.954965572	3	4.311579201	0.022975149
Low Dose	Day 43	Ganglion, Dorsal Root, Cervical - Nerve, Sciatic, Proximal, Right, Distal	1.682399898	0.61383712	0.70564806	2.659151735	3	5.481584097	0.011940471
Low Dose	Day 43	Ganglion, Dorsal Root, Cervical - Nerve, Sciatic, Proximal, Right, Proximal	1.800294028	0.656778859	0.755212302	2.845375753	3	5.482192382	0.011936787
Low Dose	Day 43	Ganglion, Dorsal Root, Cervical - Nerve, Ulnar, Distal, Left, Distal	1.862451542	0.71230999	0.729007394	2.995895691	3	5.229328713	0.013605729
Low Dose	Day 43	Ganglion, Dorsal Root, Cervical - Nerve, Ulnar, Distal, Left, Proximal	1.721352232	0.850423082	0.368139334	3.07456513	3	4.048225566	0.027142298
Low Dose	Day 43	Ganglion, Dorsal Root, Cervical - Nerve, Ulnar, Distal, Right, Distal	1.856234794	0.680698481	0.773091611	2.939377977	3	5.453911964	0.012109651
Low Dose	Day 43	Ganglion, Dorsal Root, Cervical - Nerve, Ulnar, Distal, Right, Proximal	1.820266876	0.715233184	0.682171275	2.958362478	3	5.089995592	0.014656777
Low Dose	Day 43	Ganglion, Dorsal Root, Cervical - Nerve, Ulnar, Proximal, Left, Distal	1.731549037	0.730793899	0.568692865	2.894405209	3	4.738816345	0.017818786
Low Dose	Day 43	Ganglion, Dorsal Root, Cervical - Nerve, Ulnar, Proximal, Left, Proximal	1.783481162	0.821994136	0.475505062	3.091457263	3	4.339401181	0.022584599
Low Dose	Day 43	Ganglion, Dorsal Root, Cervical - Nerve, Ulnar, Proximal, Right, Distal	1.746982574	0.818173635	0.445085744	3.048879405	3	4.270444561	0.023568726
Low Dose	Day 43	Ganglion, Dorsal Root, Cervical - Nerve, Ulnar, Proximal, Right, Proximal	1.739564627	0.868719843	0.357237499	3.121891754	3	4.004892119	0.027918984
Low Dose	Day 43	Ganglion, Dorsal Root, Cervical - Nerve, Optic	1.545579696	0.703690959	0.42585035	2.665309041	3	4.392779747	0.02185903
Low Dose	Day 43	Ganglion, Dorsal Root, Cervical - Ovary	2.24723086	0.09998164	1.348931467	3.145530253	1	31.78647967	0.020021401
Low Dose	Day 43	Ganglion, Dorsal Root, Cervical - Spinal Cord, Cervical	0.258035232	0.213112286	-0.081073973	0.597144436	3	2.421589444	0.09403497
Low Dose	Day 43	Ganglion, Dorsal Root, Cervical - Spinal Cord, Lumbar	0.581939085	0.333681894	0.05097673	1.11290144	3	3.487987185	0.039824671
Low Dose	Day 43	Ganglion, Dorsal Root, Cervical - Spinal Cord, Sacral	0.465284977	0.260754653	0.050366137	0.880203818	3	3.568756859	0.03758526
Low Dose	Day 43	Ganglion, Dorsal Root, Cervical - Spinal Cord, Spinal Roots	0.988905338	0.323564338	0.474042272	1.503768403	3	6.112573126	0.008799543
Low Dose	Day 43	Ganglion, Dorsal Root, Cervical - Spinal Cord, Thoracic	0.168019759	0.758720337	-1.039273608	1.375313125	3	0.442903005	0.687826014
Low Dose	Day 43	Ganglion, Dorsal Root, Cervical - Spleen	1.175524997	1.12456007	-0.613901024	2.964951017	3	2.090639758	0.127699036
Low Dose	Day 43	Ganglion, Dorsal Root, Cervical - Testis	1.551584517	0.960415321	-7.077404782	10.18057382	1	2.284711437	0.262651247
Low Dose	Day 43	Ganglion, Dorsal Root, Lumbar - Ganglion, Dorsal Root, Thoracic	-0.055466824	0.602703157	-1.014502042	0.903568394	3	-0.184060175	0.865704797
Low Dose	Day 43	Ganglion, Dorsal Root, Lumbar - Heart	1.250977215	0.698455981	0.139577887	2.362376542	3	3.582121851	0.03723033
Low Dose	Day 43	Ganglion, Dorsal Root, Lumbar - Kidney	1.311982422	0.743123879	0.129506501	2.494458343	3	3.530992503	0.038611764
Low Dose	Day 43	Ganglion, Dorsal Root, Lumbar - Lung	1.343003584	0.73486319	0.173672261	2.512334907	3	3.655111867	0.035365671
Low Dose	Day 43	Ganglion, Dorsal Root, Lumbar - Liver	0.303848738	1.041629215	-1.353615785	1.961313261	3	0.583410552	0.600580384
Low Dose	Day 43	Ganglion, Dorsal Root, Lumbar - Muscle, Skeletal, Tibialis Anterior Left	1.367224473	0.75723621	0.162292685	2.572156262	3	3.611091113	0.036475571
Low Dose	Day 43	Ganglion, Dorsal Root, Lumbar - Muscle, Skeletal, Tibialis Anterior Right	1.355359688	0.717120869	0.214260357	2.496459019	3	3.780003472	0.03244337
Low Dose	Day 43	Ganglion, Dorsal Root, Lumbar - Muscle, Skeletal, Bicep Brachii Left	1.377948669	0.723263775	0.227074604	2.528822733	3	3.810362737	0.031780193
Low Dose	Day 43	Ganglion, Dorsal Root, Lumbar - Muscle, Skeletal, Bicep Brachii Right	1.358650723	0.761610153	0.146759014	2.570542433	3	3.567837738	0.037609828
Low Dose	Day 43	Ganglion, Dorsal Root, Lumbar - Nerve, Femoral, Distal, Left, Distal	1.17123908	0.771634252	-0.056603208	2.399081368	3	3.035736364	0.056055946
Low Dose	Day 43	Ganglion, Dorsal Root, Lumbar - Nerve, Femoral, Distal, Left, Proximal	1.092070317	0.800874065	-0.182299037	2.36643967	3	2.727196109	0.072110736
Low Dose	Day 43	Ganglion, Dorsal Root, Lumbar - Nerve, Femoral, Distal, Right, Distal	1.16059605	0.743652495	-0.022721018	2.343913118	3	3.12133976	0.05241691
Low Dose	Day 43	Ganglion, Dorsal Root, Lumbar - Nerve, Femoral, Distal, Right, Proximal	1.108380144	0.940942112	-0.38886873	2.605629019	3	2.355894438	0.099770631
Low Dose	Day 43	Ganglion, Dorsal Root, Lumbar - Nerve, Femoral, Proximal, Left, Distal	1.18721054	0.791539089	-0.072304786	2.446725865	3	2.999752142	0.057680275
Low Dose	Day 43	Ganglion, Dorsal Root, Lumbar - Nerve, Femoral, Proximal, Left, Proximal	1.259064126	0.918252119	-0.202079906	2.720208158	3	2.742305953	0.071200343
Low Dose	Day 43	Ganglion, Dorsal Root, Lumbar - Nerve, Femoral, Proximal, Right, Distal	1.188924414	0.848724517	-0.161585688	2.539434516	3	2.80167331	0.067758769
Low Dose	Day 43	Ganglion, Dorsal Root, Lumbar - Nerve, Femoral, Proximal, Right, Proximal	1.246614215	0.821256215	-0.06018769	2.553416119	3	3.03587161	0.05604995
Low Dose	Day 43	Ganglion, Dorsal Root, Lumbar - Nerve Median, Distal, Left, Distal	1.313797346	0.741946352	0.133195133	2.494399559	3	3.541488797	0.038322905
Low Dose	Day 43	Ganglion, Dorsal Root, Lumbar - Nerve Median, Distal, Left, Proximal	1.245706044	0.962569328	-0.285956557	2.777368644	3	2.588293659	0.081193961
Low Dose	Day 43	Ganglion, Dorsal Root, Lumbar - Nerve Median, Distal, Right, Distal	1.353868658	0.764617014	0.137192363	2.570544954	3	3.541298803	0.038328109
Low Dose	Day 43	Ganglion, Dorsal Root, Lumbar - Nerve Median, Distal, Right, Proximal	1.285742023	0.800558179	0.011875314	2.559608733	3	3.21211389	0.048877315
Low Dose	Day 43	Ganglion, Dorsal Root, Lumbar - Nerve Median, Proximal, Left, Distal	1.303788756	0.823479597	-0.006551045	2.614128557	3	3.166535663	0.05061552
Low Dose	Day 43	Ganglion, Dorsal Root, Lumbar - Nerve Median, Proximal, Left, Proximal	1.250391989	0.871234293	-0.13593619	2.636720167	3	2.870392038	0.064029782
Low Dose	Day 43	Ganglion, Dorsal Root, Lumbar - Nerve Median, Proximal, Right, Distal	1.156510179	0.908744199	-0.28950463	2.602524988	3	2.545293122	0.084288633

Results of Evaluating Mean Differences Between Different Tissues Across Timepoint and Group for Log10 AAV2-ITR Target Copies/ug of DNA for Normalized Data

Group	Timepoint	Difference	Mean	StdDev	LowerCLMean	UpperCLMean	DF	tValue	P-Value
Low Dose	Day 43	Ganglion, Dorsal Root, Lumbar - Nerve Median, Proximal, Right, Proximal	1.221933459	0.760249529	0.012206807	2.43166011	3	3.214558938	0.048786216
Low Dose	Day 43	Ganglion, Dorsal Root, Lumbar - Nerve, Sciatic, Distal, Left, Distal	1.277884602	0.739558456	0.101082064	2.45468714	3	3.455804179	0.040764244
Low Dose	Day 43	Ganglion, Dorsal Root, Lumbar - Nerve, Sciatic, Distal, Left, Proximal	1.092130676	0.726568228	-0.064001511	2.248262862	3	3.006271493	0.057381649
Low Dose	Day 43	Ganglion, Dorsal Root, Lumbar - Nerve, Sciatic, Distal, Right, Distal	1.327319529	0.737293041	0.154121772	2.500517285	3	3.600520975	0.036748679
Low Dose	Day 43	Ganglion, Dorsal Root, Lumbar - Nerve, Sciatic, Distal, Right, Proximal	1.272669838	0.688551328	0.177031022	2.368308653	3	3.696659306	0.034357442
Low Dose	Day 43	Ganglion, Dorsal Root, Lumbar - Nerve, Sciatic, Proximal, Left, Distal	1.22839867	0.814842187	-0.068197083	2.524994423	3	3.015059089	0.056982189
Low Dose	Day 43	Ganglion, Dorsal Root, Lumbar - Nerve, Sciatic, Proximal, Left, Proximal	1.195580758	0.826034036	-0.118823725	2.509985241	3	2.894749384	0.062769783
Low Dose	Day 43	Ganglion, Dorsal Root, Lumbar - Nerve, Sciatic, Proximal, Right, Distal	1.177883947	0.670003153	0.111759417	2.244008477	3	3.516054936	0.039027653
Low Dose	Day 43	Ganglion, Dorsal Root, Lumbar - Nerve, Sciatic, Proximal, Right, Proximal	1.295778077	0.741262734	0.116263652	2.475292501	3	3.496137111	0.039591124
Low Dose	Day 43	Ganglion, Dorsal Root, Lumbar - Nerve, Ulnar, Distal, Left, Distal	1.357935592	0.78246306	0.112862254	2.603008929	3	3.470925749	0.040319299
Low Dose	Day 43	Ganglion, Dorsal Root, Lumbar - Nerve, Ulnar, Distal, Left, Proximal	1.216836281	0.916875449	-0.242117161	2.675789723	3	2.65431097	0.076710544
Low Dose	Day 43	Ganglion, Dorsal Root, Lumbar - Nerve, Ulnar, Distal, Right, Distal	1.351718843	0.759893126	0.142559307	2.560878379	3	3.557655141	0.037883374
Low Dose	Day 43	Ganglion, Dorsal Root, Lumbar - Nerve, Ulnar, Distal, Right, Proximal	1.315750925	0.80793044	0.030153304	2.601348547	3	3.257089622	0.047235406
Low Dose	Day 43	Ganglion, Dorsal Root, Lumbar - Nerve, Ulnar, Proximal, Left, Distal	1.227033086	0.819750877	-0.077373489	2.53143966	3	2.993673128	0.05796049
Low Dose	Day 43	Ganglion, Dorsal Root, Lumbar - Nerve, Ulnar, Proximal, Left, Proximal	1.278965211	0.893022925	-0.142033542	2.699963965	3	2.864350233	0.064347201
Low Dose	Day 43	Ganglion, Dorsal Root, Lumbar - Nerve, Ulnar, Proximal, Right, Distal	1.242466623	0.884212493	-0.164512767	2.649446014	3	2.810334922	0.067274105
Low Dose	Day 43	Ganglion, Dorsal Root, Lumbar - Nerve, Ulnar, Proximal, Right, Proximal	1.235048676	0.906807101	-0.207883779	2.67798113	3	2.723950163	0.072308191
Low Dose	Day 43	Ganglion, Dorsal Root, Lumbar - Nerve, Optic	1.041063745	0.838783696	-0.293628292	2.375755781	3	2.482317552	0.089087069
Low Dose	Day 43	Ganglion, Dorsal Root, Lumbar - Ovary	1.962376529	0.267954057	-0.44509516	4.369848219	1	10.35707214	0.061277211
Low Dose	Day 43	Ganglion, Dorsal Root, Lumbar - Spinal Cord, Cervical	-0.246480719	0.219717849	-0.596100847	0.103139408	3	-2.243611258	0.110590652
Low Dose	Day 43	Ganglion, Dorsal Root, Lumbar - Spinal Cord, Lumbar	0.077423134	0.310894749	-0.417279789	0.572126057	3	0.498066527	0.652659738
Low Dose	Day 43	Ganglion, Dorsal Root, Lumbar - Spinal Cord, Sacral	-0.039230974	0.351632582	-0.598756879	0.520294932	3	-0.223136169	0.837759632
Low Dose	Day 43	Ganglion, Dorsal Root, Lumbar - Spinal Cord, Spinal Roots	0.484389387	0.386214082	-0.130163402	1.098942176	3	2.50839837	0.087060198
Low Dose	Day 43	Ganglion, Dorsal Root, Lumbar - Spinal Cord, Thoracic	-0.336496192	0.451053267	-1.054222594	0.38123021	3	-1.492046358	0.232501714
Low Dose	Day 43	Ganglion, Dorsal Root, Lumbar - Spleen	0.671009046	1.255499339	-1.326770571	2.668788663	3	1.068911826	0.363477395
Low Dose	Day 43	Ganglion, Dorsal Root, Lumbar - Testis	0.827406946	0.602854791	-4.589028457	6.243842349	1	1.940981711	0.302862987
Low Dose	Day 43	Ganglion, Dorsal Root, Thoracic - Heart	1.306444039	0.557412954	0.419475641	2.193412437	3	4.687526652	0.018352577
Low Dose	Day 43	Ganglion, Dorsal Root, Thoracic - Kidney	1.367449246	0.601999751	0.409533304	2.325365188	3	4.54302263	0.019972511
Low Dose	Day 43	Ganglion, Dorsal Root, Thoracic - Lung	1.398470408	0.51750707	0.575001177	2.22193964	3	5.404642714	0.012418709
Low Dose	Day 43	Ganglion, Dorsal Root, Thoracic - Liver	0.359315562	1.153559774	-1.476255458	2.194886582	3	0.622968259	0.577452213
Low Dose	Day 43	Ganglion, Dorsal Root, Thoracic - Muscle, Skeletal, Tibialis Anterior Left	1.422691298	0.576328666	0.505623781	2.339758814	3	4.937083239	0.015935526
Low Dose	Day 43	Ganglion, Dorsal Root, Thoracic - Muscle, Skeletal, Tibialis Anterior Right	1.410826512	0.521715091	0.58066138	2.240991645	3	5.408417489	0.012394668
Low Dose	Day 43	Ganglion, Dorsal Root, Thoracic - Muscle, Skeletal, Bicep Brachii Left	1.433415493	0.540239043	0.57377462	2.293056366	3	5.306597187	0.013065067
Low Dose	Day 43	Ganglion, Dorsal Root, Thoracic - Muscle, Skeletal, Bicep Brachii Right	1.414117548	0.584073314	0.484726567	2.343508528	3	4.842260424	0.016802125
Low Dose	Day 43	Ganglion, Dorsal Root, Thoracic - Nerve, Femoral, Distal, Left, Distal	1.226705904	0.551831504	0.348618839	2.104792969	3	4.445943721	0.021166089
Low Dose	Day 43	Ganglion, Dorsal Root, Thoracic - Nerve, Femoral, Distal, Left, Proximal	1.147537141	0.593144665	0.203711617	2.091362664	3	3.869333094	0.030540946
Low Dose	Day 43	Ganglion, Dorsal Root, Thoracic - Nerve, Femoral, Distal, Right, Distal	1.216062874	0.582307374	0.289481899	2.14264385	3	4.176704363	0.024997317
Low Dose	Day 43	Ganglion, Dorsal Root, Thoracic - Nerve, Femoral, Distal, Right, Proximal	1.163846969	0.769409271	-0.060454878	2.388148815	3	3.025300089	0.056521057
Low Dose	Day 43	Ganglion, Dorsal Root, Thoracic - Nerve, Femoral, Proximal, Left, Distal	1.242677364	0.653134972	0.203393874	2.281960853	3	3.805269712	0.031890225
Low Dose	Day 43	Ganglion, Dorsal Root, Thoracic - Nerve, Femoral, Proximal, Left, Proximal	1.314530951	0.73146535	0.150606351	2.47845555	3	3.59423984	0.036912207
Low Dose	Day 43	Ganglion, Dorsal Root, Thoracic - Nerve, Femoral, Proximal, Right, Distal	1.244391238	0.660799531	0.192911725	2.295870751	3	3.766319978	0.032748077
Low Dose	Day 43	Ganglion, Dorsal Root, Thoracic - Nerve, Femoral, Proximal, Right, Proximal	1.302081039	0.674073831	0.229479152	2.374682926	3	3.863318759	0.030664462
Low Dose	Day 43	Ganglion, Dorsal Root, Thoracic - Nerve Median, Distal, Left, Distal	1.36926417	0.547859155	0.497497998	2.241030342	3	4.998599212	0.015404265
Low Dose	Day 43	Ganglion, Dorsal Root, Thoracic - Nerve Median, Distal, Left, Proximal	1.301172868	0.731458585	0.137259032	2.465086704	3	3.55774857	0.037880853
Low Dose	Day 43	Ganglion, Dorsal Root, Thoracic - Nerve Median, Distal, Right, Distal	1.409335482	0.548493614	0.536559745	2.28211122	3	5.138931235	0.014275943
Low Dose	Day 43	Ganglion, Dorsal Root, Thoracic - Nerve Median, Distal, Right, Proximal	1.341208848	0.582115993	0.414932402	2.267485293	3	4.608046724	0.019221519
Low Dose	Day 43	Ganglion, Dorsal Root, Thoracic - Nerve Median, Proximal, Left, Distal	1.35925558	0.595297539	0.412004354	2.306506807	3	4.566642703	0.019695382
Low Dose	Day 43	Ganglion, Dorsal Root, Thoracic - Nerve Median, Proximal, Left, Proximal	1.305858813	0.663579833	0.249955219	2.361762407	3	3.93580018	0.029217535
Low Dose	Day 43	Ganglion, Dorsal Root, Thoracic - Nerve Median, Proximal, Right, Distal	1.211977003	0.51447391	0.393334206	2.0306198	3	4.711519786	0.018100322
Low Dose	Day 43	Ganglion, Dorsal Root, Thoracic - Nerve Median, Proximal, Right, Proximal	1.277400283	0.650828392	0.241787077	2.313013489	3	3.925459608	0.029418515
Low Dose	Day 43	Ganglion, Dorsal Root, Thoracic - Nerve, Sciatic, Distal, Left, Distal	1.333351426	0.564862134	0.434529721	2.232173131	3	4.720980028	0.018002099
Low Dose	Day 43	Ganglion, Dorsal Root, Thoracic - Nerve, Sciatic, Distal, Left, Proximal	1.1475975	0.677635745	0.069327814	2.225867186	3	3.387063062	0.042867514
Low Dose	Day 43	Ganglion, Dorsal Root, Thoracic - Nerve, Sciatic, Distal, Right, Distal	1.382786353	0.537256116	0.527891982	2.237680723	3	5.1475872	0.014209928
Low Dose	Day 43	Ganglion, Dorsal Root, Thoracic - Nerve, Sciatic, Distal, Right, Proximal	1.328136662	0.510684342	0.515523913	2.14074941	3	5.201399585	0.013808331
Low Dose	Day 43	Ganglion, Dorsal Root, Thoracic - Nerve, Sciatic, Proximal, Left, Distal	1.283865495	0.640148321	0.265246665	2.302484325	3	4.011150078	0.027805067
Low Dose	Day 43	Ganglion, Dorsal Root, Thoracic - Nerve, Sciatic, Proximal, Left, Proximal	1.251047582	0.663533704	0.19521739	2.306877775	3	3.770863709	0.032646491
Low Dose	Day 43	Ganglion, Dorsal Root, Thoracic - Nerve, Sciatic, Proximal, Right, Distal	1.233350771	0.502825355	0.433243424	2.033458118	3	4.905682491	0.016215919
Low Dose	Day 43	Ganglion, Dorsal Root, Thoracic - Nerve, Sciatic, Proximal, Right, Proximal	1.351244901	0.520412335	0.523152744	2.179337057	3	5.19297799	0.013870194
Low Dose	Day 43	Ganglion, Dorsal Root, Thoracic - Nerve, Ulnar, Distal, Left, Distal	1.413402416	0.574483588	0.49927083	2.327534002	3	4.920601545	0.016081905
Low Dose	Day 43	Ganglion, Dorsal Root, Thoracic - Nerve, Ulnar, Distal, Left, Proximal	1.272303105	0.69669941	0.163698873	2.380907337	3	3.652373138	0.035433453
Low Dose	Day 43	Ganglion, Dorsal Root, Thoracic - Nerve, Ulnar, Distal, Right, Distal	1.407185667	0.540375484	0.547327686	2.267043648	3	5.208177312	0.013758804
Low Dose	Day 43	Ganglion, Dorsal Root, Thoracic - Nerve, Ulnar, Distal, Right, Proximal	1.37121775	0.561275227	0.478103614	2.264331886	3	4.886079713	0.016394224
Low Dose	Day 43	Ganglion, Dorsal Root, Thoracic - Nerve, Ulnar, Proximal, Left, Distal	1.28249991	0.576778946	0.364715897	2.200283923	3	4.447110693	0.0211512
Low Dose	Day 43	Ganglion, Dorsal Root, Thoracic - Nerve, Ulnar, Proximal, Left, Proximal	1.334432036	0.669742085	0.268722923	2.400141148	3	3.984913192	0.028286713
Low Dose	Day 43	Ganglion, Dorsal Root, Thoracic - Nerve, Ulnar, Proximal, Right, Distal	1.297933448	0.670971044	0.230268788	2.365598108	3	3.868821044	0.030551437
Low Dose	Day 43	Ganglion, Dorsal Root, Thoracic - Nerve, Ulnar, Proximal, Right, Proximal	1.2905155	0.732990658	0.124163794	2.456867206	3	3.521233144	0.038882838
Low Dose	Day 43	Ganglion, Dorsal Root, Thoracic - Nerve, Optic	1.096530569	0.522653708	0.264871889	1.928189249	3	4.196011826	0.024694115
Low Dose	Day 43	Ganglion, Dorsal Root, Thoracic - Ovary	1.596051381	0.2887442	-0.998212332	4.190315093	1	7.81715271	0.080998906
Low Dose	Day 43	Ganglion, Dorsal Root, Thoracic - Spinal Cord, Cervical	-0.191013895	0.448279147	-0.904326052	0.522298262	3	-0.852209595	0.456726262
Low Dose	Day 43	Ganglion, Dorsal Root, Thoracic - Spinal Cord, Lumbar	0.132889958	0.410652012	-0.52054903	0.786328947	3	0.647214452	0.563603131
Low Dose	Day 43	Ganglion, Dorsal Root, Thoracic - Spinal Cord, Sacral	0.016235851	0.41392842	-0.642416635	0.674888336	3	0.078447624	0.942411462
Low Dose	Day 43	Ganglion, Dorsal Root, Thoracic - Spinal Cord, Spinal Roots	0.539856211	0.348973982	-0.015439269	1.09515169	3	3.093962525	0.053547491
Low Dose	Day 43	Ganglion, Dorsal Root, Thoracic - Spinal Cord, Thoracic	-0.281029368	1.009023652	-1.886611164	1.324552428	3	-0.557032271	0.616364094
Low Dose	Day 43	Ganglion, Dorsal Root, Thoracic - Spleen	0.72647587	0.924171149	-0.74408666	2.1970384	3	1.572167386	0.213957732
Low Dose	Day 43	Ganglion, Dorsal Root, Thoracic - Testis	1.304665743	0.864018463	-6.458232149	9.067563636	1	2.135458984	0.278810143
Low Dose	Day 43	Heart - Kidney	0.061005207	0.08005534	-0.066380703	0.188391118	3	1.524075906	0.224883661
Low Dose	Day 43	Heart - Lung	0.092026369	0.107818254	-0.079536533	0.263589272	3	1.707064724	0.186348845
Low Dose	Day 43	Heart - Liver	-0.947128477	0.65614828	-1.991206812	0.096949858	3	-2.886934267	0.063170654
Low Dose	Day 43	Heart - Muscle, Skeletal, Tibialis Anterior Left	0.116247259	0.078183813	-0.008160636	0.240655153	3	2.973691187	0.058893684
Low Dose	Day 43	Heart - Muscle, Skeletal, Tibialis Anterior Right	0.104382473	0.063470856	0.003386178	0.205378768	3	3.289146552	0.04610756
Low Dose	Day 43	Heart - Muscle, Skeletal, Bicep Brachii Left	0.126971454	0.06965476	0.016135187	0.237807721	3	3.645736597	0.035598394
Low Dose	Day 43	Heart - Muscle, Skeletal, Bicep Brachii Right	0.107673509	0.076609136	-0.014228723	0.22957574	3	2.810983492	0.067237988
Low Dose	Day 43	Heart - Nerve, Femoral, Distal, Left, Distal	-0.079738135	0.126242853	-0.280618686	0.121142416	3	-1.263249884	0.295748801
Low Dose	Day 43	Heart - Nerve, Femoral, Distal, Left, Proximal	-0.158906898	0.127692574	-0.362094278	0.044280481	3	-2.488898041	0.088570258
Low Dose	Day 43	Heart - Nerve, Femoral, Distal, Right, Distal	-0.090381165	0.172949392	-0.365582241	0.184819912	3	-1.045174704	0.372741271
Low Dose	Day 43	Heart - Nerve, Femoral, Distal, Right, Proximal	-0.14259707	0.265301109	-0.564750337	0.279556196	3	-1.074982846	0.361144194
Low Dose	Day 43	Heart - Nerve, Femoral, Proximal, Left, Distal	-0.063766675	0.146350313	-0.296642682	0.169109331	3	-0.871425199	0.447650762
Low Dose	Day 43	Heart - Nerve, Femoral, Proximal, Left, Proximal	0.008086911	0.227243473	-0.353508164	0.369681987	3	0.071173983	0.947738444
Low Dose	Day 43	Heart - Nerve, Femoral, Proximal, Right, Distal	-0.062052801	0.191084767	-0.366111306	0.242005704	3	-0.649479308	0.562322304
Low Dose	Day 43	Heart - Nerve, Femoral, Proximal, Right, Proximal	-0.004363	0.149863675	-0.242829549	0.234103548	3	-0.058226255	0.957229792
Low Dose	Day 43	Heart - Nerve Median, Distal, Left, Distal	0.062820131	0.118111896	-0.125122253	0.250762515	3	1.06373927	0.36547687
Low Dose	Day 43	Heart - Nerve Median, Distal, Left, Proximal	-0.005271171	0.2708453	-0.436246484	0.425704142	3	-0.038923853	0.971396503
Low Dose	Day 43	Heart - Nerve Median, Distal, Right, Distal	0.102891443	0.104336398	-0.063131048	0.268913935	3	1.972302007	0.143125592

Results of Evaluating Mean Differences Between Different Tissues Across Timepoint and Group for Log10 AAV2-ITR Target Copies/ug of DNA for Normalized Data

Group	Timepoint	Difference	Mean	StdDev	LowerCLMean	UpperCLMean	DF	tValue	P-Value
Low Dose	Day 43	Heart - Nerve Median, Distal, Right, Proximal	0.034764809	0.147559542	-0.200035351	0.269564968	3	0.471197025	0.669647235
Low Dose	Day 43	Heart - Nerve Median, Proximal, Left, Distal	0.052811541	0.158759767	-0.199810675	0.305433758	3	0.665301324	0.553436085
Low Dose	Day 43	Heart - Nerve Median, Proximal, Left, Proximal	-0.000585226	0.179613326	-0.286390109	0.285219657	3	-0.006516511	0.995209724
Low Dose	Day 43	Heart - Nerve Median, Proximal, Right, Distal	-0.094467036	0.393481092	-0.72058326	0.531649189	3	-0.480160483	0.663949988
Low Dose	Day 43	Heart - Nerve Median, Proximal, Right, Proximal	-0.029043756	0.132877498	-0.240481508	0.182393995	3	-0.437150858	0.691557261
Low Dose	Day 43	Heart - Nerve, Sciatic, Distal, Left, Distal	0.026907387	0.096335275	-0.126383533	0.180198307	3	0.558619608	0.615406243
Low Dose	Day 43	Heart - Nerve, Sciatic, Distal, Left, Proximal	-0.158846539	0.185017866	-0.45325125	0.135558172	3	-1.71709406	0.184462682
Low Dose	Day 43	Heart - Nerve, Sciatic, Distal, Right, Distal	0.076342314	0.075420742	-0.043668917	0.196353544	3	2.024438154	0.136071971
Low Dose	Day 43	Heart - Nerve, Sciatic, Distal, Right, Proximal	0.021692623	0.072674913	-0.093949381	0.137334627	3	0.596976917	0.592574711
Low Dose	Day 43	Heart - Nerve, Sciatic, Proximal, Left, Distal	-0.022578544	0.120425282	-0.214202042	0.169044953	3	-0.374980136	0.732615483
Low Dose	Day 43	Heart - Nerve, Sciatic, Proximal, Left, Proximal	-0.055396457	0.154154562	-0.300690765	0.189897852	3	-0.718713166	0.524238845
Low Dose	Day 43	Heart - Nerve, Sciatic, Proximal, Right, Distal	-0.073093268	0.054950771	-0.160532207	0.014345671	3	-2.660318197	0.076317938
Low Dose	Day 43	Heart - Nerve, Sciatic, Proximal, Right, Proximal	0.044800862	0.128519024	-0.159701585	0.249303308	3	0.697186462	0.535856971
Low Dose	Day 43	Heart - Nerve, Ulnar, Distal, Left, Distal	0.106958377	0.108634688	-0.065903654	0.279820408	3	1.969138557	0.143567269
Low Dose	Day 43	Heart - Nerve, Ulnar, Distal, Left, Proximal	-0.034140934	0.219216367	-0.382963093	0.314681225	3	-0.311481613	0.775825523
Low Dose	Day 43	Heart - Nerve, Ulnar, Distal, Right, Distal	0.100741628	0.091756924	-0.045264113	0.24674737	3	2.195837091	0.115621082
Low Dose	Day 43	Heart - Nerve, Ulnar, Distal, Right, Proximal	0.064773711	0.145145037	-0.166184433	0.295731854	3	0.892537724	0.437864084
Low Dose	Day 43	Heart - Nerve, Ulnar, Proximal, Left, Distal	-0.023944129	0.146249369	-0.256659512	0.208771253	3	-0.327442494	0.764852032
Low Dose	Day 43	Heart - Nerve, Ulnar, Proximal, Left, Proximal	0.027987997	0.205652101	-0.299250388	0.355226381	3	0.2721878	0.803135947
Low Dose	Day 43	Heart - Nerve, Ulnar, Proximal, Right, Distal	-0.008510591	0.207207283	-0.338223618	0.321202435	3	-0.082145679	0.939704653
Low Dose	Day 43	Heart - Nerve, Ulnar, Proximal, Right, Proximal	-0.015928539	0.222687286	-0.370273705	0.338416627	3	-0.143057463	0.895313064
Low Dose	Day 43	Heart - Nerve, Optic	-0.20991347	0.218115771	-0.556984334	0.137157394	3	-1.924789476	0.149930838
Low Dose	Day 43	Heart - Ovary	0.215631648	0.012215662	0.105878276	0.32538502	1	24.96378753	0.025488103
Low Dose	Day 43	Heart - Spinal Cord, Cervical	-1.497457934	0.696881754	-2.606352316	-0.388563552	3	-4.297595467	0.023174744
Low Dose	Day 43	Heart - Spinal Cord, Lumbar	-1.173554081	0.441501203	-1.876081017	-0.471027144	3	-5.316198789	0.012999856
Low Dose	Day 43	Heart - Spinal Cord, Sacral	-1.290208188	0.672053859	-2.359595849	-0.220820528	3	-3.83959759	0.031157923
Low Dose	Day 43	Heart - Spinal Cord, Spinal Roots	-0.766587828	0.408137438	-1.416025568	-0.117150088	3	-3.756518063	0.032968605
Low Dose	Day 43	Heart - Spinal Cord, Thoracic	-1.587473407	1.125773157	-3.37882972	0.203882905	3	-2.820236736	0.066725323
Low Dose	Day 43	Heart - Spleen	-0.579968169	0.601157312	-1.536543603	0.376607265	3	-1.929505496	0.149238659
Low Dose	Day 43	Heart - Testis	0.072197398	0.041332934	-0.299164281	0.443559077	1	2.470246587	0.244877587
Low Dose	Day 43	Kidney - Lung	0.031021162	0.100379988	-0.128705799	0.190748123	3	0.618074631	0.580277701
Low Dose	Day 43	Kidney - Liver	-1.008133684	0.667829948	-2.07080016	0.054532792	3	-3.0191329	0.056798189
Low Dose	Day 43	Kidney - Muscle, Skeletal, Tibialis Anterior Left	0.055242051	0.048219038	-0.021485198	0.131969301	3	2.291296293	0.105830312
Low Dose	Day 43	Kidney - Muscle, Skeletal, Tibialis Anterior Right	0.043377266	0.120516278	-0.148391025	0.235145557	3	0.719857378	0.52362697
Low Dose	Day 43	Kidney - Muscle, Skeletal, Bicep Brachii Left	0.065966247	0.065665192	-0.038521728	0.170454221	3	2.009169366	0.13809439
Low Dose	Day 43	Kidney - Muscle, Skeletal, Bicep Brachii Right	0.046668301	0.047127907	-0.028322716	0.121659319	3	1.980495372	0.141989055
Low Dose	Day 43	Kidney - Nerve, Femoral, Distal, Left, Distal	-0.140743342	0.175085866	-0.419344026	0.137857341	3	-1.6077065	0.206262758
Low Dose	Day 43	Kidney - Nerve, Femoral, Distal, Left, Proximal	-0.219912106	0.159740366	-0.474094674	0.034270462	3	-2.753369256	0.0705428
Low Dose	Day 43	Kidney - Nerve, Femoral, Distal, Right, Distal	-0.151386372	0.113351493	-0.331753892	0.028981148	3	-2.671096212	0.075619745
Low Dose	Day 43	Kidney - Nerve, Femoral, Distal, Right, Proximal	-0.203602278	0.202442657	-0.525733721	0.118529166	3	-2.011456285	0.137789228
Low Dose	Day 43	Kidney - Nerve, Femoral, Proximal, Left, Distal	-0.124771883	0.067051934	-0.231466472	-0.018077293	3	-3.721649035	0.033768694
Low Dose	Day 43	Kidney - Nerve, Femoral, Proximal, Left, Proximal	-0.052918296	0.177407342	-0.335212965	0.229376374	3	-0.596573911	0.592811424
Low Dose	Day 43	Kidney - Nerve, Femoral, Proximal, Right, Distal	-0.123058008	0.119508969	-0.313223447	0.06710743	3	-2.059393691	0.131572406
Low Dose	Day 43	Kidney - Nerve, Femoral, Proximal, Right, Proximal	-0.065368208	0.08292153	-0.197314866	0.066578451	3	-1.576628108	0.212974541
Low Dose	Day 43	Kidney - Nerve Median, Distal, Left, Distal	0.001814924	0.079908095	-0.125336688	0.128966535	3	0.045425281	0.966622943
Low Dose	Day 43	Kidney - Nerve Median, Distal, Left, Proximal	-0.066276379	0.222678311	-0.420607262	0.288054505	3	-0.595265685	0.593580304
Low Dose	Day 43	Kidney - Nerve Median, Distal, Right, Distal	0.041886236	0.087837678	-0.097883111	0.181655584	3	0.953719107	0.410585345
Low Dose	Day 43	Kidney - Nerve Median, Distal, Right, Proximal	-0.026240399	0.099420252	-0.184440205	0.131959408	3	-0.527868281	0.634143472
Low Dose	Day 43	Kidney - Nerve Median, Proximal, Left, Distal	-0.008193666	0.113555174	-0.188885288	0.172497956	3	-0.144311626	0.894403681
Low Dose	Day 43	Kidney - Nerve Median, Proximal, Left, Proximal	-0.061590433	0.178628741	-0.345828622	0.222647755	3	-0.689591531	0.540004223
Low Dose	Day 43	Kidney - Nerve Median, Proximal, Right, Distal	-0.155472243	0.368513881	-0.741860063	0.430915576	3	-0.843779576	0.460758502
Low Dose	Day 43	Kidney - Nerve Median, Proximal, Right, Proximal	-0.090048964	0.060431182	-0.186208459	0.006110532	3	-2.980215218	0.058586941
Low Dose	Day 43	Kidney - Nerve, Sciatic, Distal, Left, Distal	-0.03409782	0.050850447	-0.115012228	0.046816587	3	-1.341102107	0.27238313
Low Dose	Day 43	Kidney - Nerve, Sciatic, Distal, Left, Proximal	-0.219851746	0.130252813	-0.427113038	-0.012590454	3	-3.375769644	0.043226145
Low Dose	Day 43	Kidney - Nerve, Sciatic, Distal, Right, Distal	0.015337106	0.080050139	-0.112040528	0.142714741	3	0.383187504	0.727121196
Low Dose	Day 43	Kidney - Nerve, Sciatic, Distal, Right, Proximal	-0.039312585	0.146678428	-0.272710696	0.194085526	3	-0.536037713	0.629128734
Low Dose	Day 43	Kidney - Nerve, Sciatic, Proximal, Left, Distal	-0.083583752	0.082208313	-0.214395523	0.04722802	3	-2.033462272	0.134893147
Low Dose	Day 43	Kidney - Nerve, Sciatic, Proximal, Left, Proximal	-0.116401664	0.084690926	-0.251163827	0.018360499	3	-2.748857971	0.070810011
Low Dose	Day 43	Kidney - Nerve, Sciatic, Proximal, Right, Distal	-0.134098475	0.114289892	-0.315959197	0.047762246	3	-2.346637547	0.100612644
Low Dose	Day 43	Kidney - Nerve, Sciatic, Proximal, Right, Proximal	-0.016204346	0.107818566	-0.187767744	0.155359053	3	-0.300585439	0.783357683
Low Dose	Day 43	Kidney - Nerve, Ulnar, Distal, Left, Distal	0.045953169	0.078270115	-0.078592049	0.170498388	3	1.174220061	0.325035321
Low Dose	Day 43	Kidney - Nerve, Ulnar, Distal, Left, Proximal	-0.095146141	0.185258478	-0.389933721	0.199641438	3	-1.027171788	0.379918714
Low Dose	Day 43	Kidney - Nerve, Ulnar, Distal, Right, Distal	0.039736421	0.102255465	-0.122974842	0.202447684	3	0.777198972	0.493698385
Low Dose	Day 43	Kidney - Nerve, Ulnar, Distal, Right, Proximal	0.003768503	0.125343057	-0.19568027	0.203217277	3	0.060131024	0.95583285
Low Dose	Day 43	Kidney - Nerve, Ulnar, Proximal, Left, Distal	-0.084949336	0.126354257	-0.286007156	0.116108484	3	-1.344621671	0.271373864
Low Dose	Day 43	Kidney - Nerve, Ulnar, Proximal, Left, Proximal	-0.033017211	0.157081518	-0.282968959	0.216934538	3	-0.420383138	0.702500832
Low Dose	Day 43	Kidney - Nerve, Ulnar, Proximal, Right, Distal	-0.069515799	0.147872248	-0.304813544	0.165781946	3	-0.940214267	0.416469425
Low Dose	Day 43	Kidney - Nerve, Ulnar, Proximal, Right, Proximal	-0.076933746	0.165845596	-0.340831099	0.186963606	3	-0.927775571	0.421957493
Low Dose	Day 43	Kidney - Nerve, Optic	-0.270918677	0.209866512	-0.60486313	0.063025776	3	-2.58181903	0.081650872
Low Dose	Day 43	Kidney - Ovary	0.107069003	0.109378341	-0.875656402	1.089794408	1	1.384354841	0.39825301
Low Dose	Day 43	Kidney - Spinal Cord, Cervical	-1.558463141	0.73737847	-2.731796834	-0.385129449	3	-4.227037284	0.024216768
Low Dose	Day 43	Kidney - Spinal Cord, Lumbar	-1.234559288	0.503976862	-2.03649894	-0.432619636	3	-4.89926971	0.01627397
Low Dose	Day 43	Kidney - Spinal Cord, Sacral	-1.351213396	0.738966249	-2.5270736	-0.175353192	3	-3.657036835	0.035318128
Low Dose	Day 43	Kidney - Spinal Cord, Spinal Roots	-0.827593035	0.474741335	-1.583012439	-0.072173632	3	-3.486500858	0.039867453
Low Dose	Day 43	Kidney - Spinal Cord, Thoracic	-1.648478615	1.159823987	-3.494017396	0.197060167	3	-2.842635836	0.065504354
Low Dose	Day 43	Kidney - Spleen	-0.640973376	0.545335172	-1.508723328	0.226776575	3	-2.350750179	0.100237495
Low Dose	Day 43	Kidney - Testis	0.058749628	0.068520188	-0.556879836	0.674379092	1	1.212555353	0.439028441
Low Dose	Day 43	Lung - Liver	-1.039154846	0.752958367	-2.237279633	0.15896994	3	-2.760192044	0.070141049
Low Dose	Day 43	Lung - Muscle, Skeletal, Tibialis Anterior Left	0.024220889	0.066008929	-0.080814047	0.129255826	3	0.733867054	0.516181681
Low Dose	Day 43	Lung - Muscle, Skeletal, Tibialis Anterior Right	0.012356104	0.094599184	-0.138172309	0.162884516	3	0.261230662	0.81082175
Low Dose	Day 43	Lung - Muscle, Skeletal, Bicep Brachii Left	0.034945084	0.041869681	-0.031678921	0.10156909	3	1.669231005	0.193663884
Low Dose	Day 43	Lung - Muscle, Skeletal, Bicep Brachii Right	0.015647139	0.075747555	-0.104884123	0.136178402	3	0.413139125	0.707259019
Low Dose	Day 43	Lung - Nerve, Femoral, Distal, Left, Distal	-0.171764504	0.156180141	-0.420281961	0.076752952	3	-2.199569075	0.115218347
Low Dose	Day 43	Lung - Nerve, Femoral, Distal, Left, Proximal	-0.250933268	0.160402141	-0.506168868	0.004302333	3	-3.128801972	0.052113978
Low Dose	Day 43	Lung - Nerve, Femoral, Distal, Right, Distal	-0.182407534	0.118484007	-0.370942029	0.006126961	3	-3.079023725	0.054177369
Low Dose	Day 43	Lung - Nerve, Femoral, Distal, Right, Proximal	-0.23462344	0.25586226	-0.641757392	0.172510512	3	-1.833982391	0.164017635
Low Dose	Day 43	Lung - Nerve, Femoral, Proximal, Left, Distal	-0.155793045	0.142386821	-0.382362251	0.070776162	3	-2.188307081	0.116438837
Low Dose	Day 43	Lung - Nerve, Femoral, Proximal, Left, Proximal	-0.083939458	0.223243337	-0.439169425	0.271290509	3	-0.751999669	0.506673043
Low Dose	Day 43	Lung - Nerve, Femoral, Proximal, Right, Distal	-0.15407917	0.1507174	-0.393904187	0.085745846	3	-2.044610248	0.133453527
Low Dose	Day 43	Lung - Nerve, Femoral, Proximal, Right, Proximal	-0.09638937	0.157901099	-0.347645254	0.154866515	3	-1.220882822	0.309332448
Low Dose	Day 43	Lung - Nerve Median, Distal, Left, Distal	-0.029206238	0.044212817	-0.099558697	0.04114622	3	-1.32116612	0.278175432
Low Dose	Day 43	Lung - Nerve Median, Distal, Left, Proximal	-0.097297541	0.24156609	-0.481683096	0.287088014	3	-0.805556283	0.479431298
Low Dose	Day 43	Lung - Nerve Median, Distal, Right, Distal	0.010865074	0.037900957	-0.049443806	0.071173954	3	0.573340362	0.606572257

Results of Evaluating Mean Differences Between Different Tissues Across Timepoint and Group for Log10 AAV2-ITR Target Copies/ug of DNA for Normalized Data

Group	Timepoint	Difference	Mean	StdDev	LowerCLMean	UpperCLMean	DF	tValue	P-Value
Low Dose	Day 43	Lung - Nerve Median, Distal, Right, Proximal	-0.057261561	0.075220755	-0.176954567	0.062431446	3	-1.522493654	0.225253471
Low Dose	Day 43	Lung - Nerve Median, Proximal, Left, Distal	-0.039214828	0.09321833	-0.187545993	0.109116337	3	-0.841354439	0.461924235
Low Dose	Day 43	Lung - Nerve Median, Proximal, Left, Proximal	-0.092611595	0.199242532	-0.409650925	0.224427734	3	-0.929636807	0.421132106
Low Dose	Day 43	Lung - Nerve Median, Proximal, Right, Distal	-0.186493405	0.286814206	-0.642878811	0.269892001	3	-1.300447474	0.284332952
Low Dose	Day 43	Lung - Nerve Median, Proximal, Right, Proximal	-0.121070126	0.152145184	-0.363167064	0.121026813	3	-1.59150783	0.209731118
Low Dose	Day 43	Lung - Nerve, Sciatic, Distal, Left, Distal	-0.065118983	0.056852623	-0.155584193	0.025346228	3	-2.290799577	0.105878608
Low Dose	Day 43	Lung - Nerve, Sciatic, Distal, Left, Proximal	-0.250872908	0.213466071	-0.590545063	0.088799246	3	-2.350471035	0.100262905
Low Dose	Day 43	Lung - Nerve, Sciatic, Distal, Right, Distal	-0.015684056	0.038941423	-0.07764855	0.046280438	3	-0.805520413	0.479449122
Low Dose	Day 43	Lung - Nerve, Sciatic, Distal, Right, Proximal	-0.070333747	0.128864891	-0.275386544	0.134719051	3	-1.091588971	0.354836639
Low Dose	Day 43	Lung - Nerve, Sciatic, Proximal, Left, Distal	-0.114604914	0.131829513	-0.324375087	0.095165259	3	-1.738683719	0.180475798
Low Dose	Day 43	Lung - Nerve, Sciatic, Proximal, Left, Proximal	-0.147422826	0.146068885	-0.379851018	0.085005366	3	-2.018538389	0.136849245
Low Dose	Day 43	Lung - Nerve, Sciatic, Proximal, Right, Distal	-0.165119637	0.09170653	-0.311045192	-0.019194083	3	-3.601044258	0.036735098
Low Dose	Day 43	Lung - Nerve, Sciatic, Proximal, Right, Proximal	-0.047225508	0.02551208	-0.087820921	-0.006630094	3	-3.702207479	0.034225601
Low Dose	Day 43	Lung - Nerve, Ulnar, Distal, Left, Distal	0.014932007	0.061977759	-0.083688437	0.113552452	3	0.48185051	0.662879176
Low Dose	Day 43	Lung - Nerve, Ulnar, Distal, Left, Proximal	-0.126167303	0.207033209	-0.455603339	0.203268733	3	-1.218812224	0.310012554
Low Dose	Day 43	Lung - Nerve, Ulnar, Distal, Right, Distal	0.008715259	0.058090274	-0.08371933	0.101149847	3	0.300059144	0.783722311
Low Dose	Day 43	Lung - Nerve, Ulnar, Distal, Right, Proximal	-0.027252659	0.075975578	-0.148146757	0.093641439	3	-0.717405769	0.524938684
Low Dose	Day 43	Lung - Nerve, Ulnar, Proximal, Left, Distal	-0.115970498	0.09132191	-0.261284035	0.029343039	3	-2.539817639	0.084693034
Low Dose	Day 43	Lung - Nerve, Ulnar, Proximal, Left, Proximal	-0.064038373	0.170777373	-0.335783283	0.207706537	3	-0.749963204	0.507733761
Low Dose	Day 43	Lung - Nerve, Ulnar, Proximal, Right, Distal	-0.100536961	0.16636746	-0.365264715	0.164190793	3	-1.208613281	0.313384898
Low Dose	Day 43	Lung - Nerve, Ulnar, Proximal, Right, Proximal	-0.107954908	0.219458598	-0.45716251	0.241252693	3	-0.983829384	0.397742921
Low Dose	Day 43	Lung - Nerve, Optic	-0.301939839	0.13214109	-0.512205801	-0.091673878	3	-4.569961389	0.019656845
Low Dose	Day 43	Lung - Ovary	0.05214365	0.028475922	-0.203702362	0.307989661	1	2.589635395	0.234603383
Low Dose	Day 43	Lung - Spinal Cord, Cervical	-1.589484304	0.701846751	-2.706279103	-0.472689504	3	-4.529434101	0.020134235
Low Dose	Day 43	Lung - Spinal Cord, Lumbar	-1.26558045	0.482786148	-2.033800947	-0.497359953	3	-5.242820053	0.01350925
Low Dose	Day 43	Lung - Spinal Cord, Sacral	-1.382234558	0.699312933	-2.494997487	-0.269471628	3	-3.95312168	0.028884812
Low Dose	Day 43	Lung - Spinal Cord, Spinal Roots	-0.858614198	0.440729587	-1.559913321	-0.157315074	3	-3.896331096	0.029994292
Low Dose	Day 43	Lung - Spinal Cord, Thoracic	-1.679499777	1.162147109	-3.528735163	0.16973561	3	-2.890339379	0.062995599
Low Dose	Day 43	Lung - Spleen	-0.671994538	0.532446033	-1.519234993	0.175245917	3	-2.524178965	0.085861254
Low Dose	Day 43	Lung - Testis	0.051632657	0.077758939	-0.647003695	0.75026901	1	0.939050928	0.520003917
Low Dose	Day 43	Liver - Muscle, Skeletal, Tibialis Anterior Left	1.063375736	0.692255014	-0.03815647	2.164907942	3	3.072208114	0.05446784
Low Dose	Day 43	Liver - Muscle, Skeletal, Tibialis Anterior Right	1.05151095	0.692075126	-0.049735014	2.152756915	3	3.038719093	0.055923899
Low Dose	Day 43	Liver - Muscle, Skeletal, Bicep Brachii Left	1.074099931	0.713615049	-0.061420857	2.209620719	3	3.010306278	0.057197804
Low Dose	Day 43	Liver - Muscle, Skeletal, Bicep Brachii Right	1.054801986	0.682813955	-0.031707389	2.14131136	3	3.089573602	0.053731584
Low Dose	Day 43	Liver - Nerve, Femoral, Distal, Left, Distal	0.867390342	0.672636951	-0.202925147	1.937705831	3	2.579074318	0.08184552
Low Dose	Day 43	Liver - Nerve, Femoral, Distal, Left, Proximal	0.788221579	0.648757433	-0.244096269	1.820539426	3	2.429942345	0.093334837
Low Dose	Day 43	Liver - Nerve, Femoral, Distal, Right, Distal	0.856747313	0.765411565	-0.361193291	2.074687916	3	2.238657872	0.111099814
Low Dose	Day 43	Liver - Nerve, Femoral, Distal, Right, Proximal	0.804531407	0.658566001	-0.243394062	1.852456876	3	2.443282541	0.092229825
Low Dose	Day 43	Liver - Nerve, Femoral, Proximal, Left, Distal	0.883361802	0.67321663	-0.187876086	1.95459969	3	2.624301785	0.078709626
Low Dose	Day 43	Liver - Nerve, Femoral, Proximal, Left, Proximal	0.955215389	0.647745844	-0.075492796	1.985923573	3	2.949352426	0.060055949
Low Dose	Day 43	Liver - Nerve, Femoral, Proximal, Right, Distal	0.885075676	0.718003083	-0.257427454	2.027578806	3	2.465381268	0.090434275
Low Dose	Day 43	Liver - Nerve, Femoral, Proximal, Right, Proximal	0.942765477	0.645994313	-0.08515563	1.970686584	3	2.918804262	0.061555707
Low Dose	Day 43	Liver - Nerve Median, Distal, Left, Distal	1.009948608	0.746796174	-0.178370754	2.198267971	3	2.704750354	0.07348996
Low Dose	Day 43	Liver - Nerve Median, Distal, Left, Proximal	0.941857306	0.707814609	-0.184433688	2.0681483	3	2.661310726	0.07625331
Low Dose	Day 43	Liver - Nerve Median, Distal, Right, Distal	1.050019921	0.730557023	-0.112459329	2.21249917	3	2.874573476	0.063811246
Low Dose	Day 43	Liver - Nerve Median, Distal, Right, Proximal	0.981893286	0.74635729	-0.205727714	2.169514286	3	2.631161506	0.07824704
Low Dose	Day 43	Liver - Nerve Median, Proximal, Left, Distal	0.999940019	0.742336994	-0.181283793	2.18116383	3	2.694032568	0.074160071
Low Dose	Day 43	Liver - Nerve Median, Proximal, Left, Proximal	0.946543251	0.630945849	-0.057432391	1.950518893	3	3.000394576	0.057650761
Low Dose	Day 43	Liver - Nerve Median, Proximal, Right, Distal	0.852661441	1.01563191	-0.763435568	2.468758451	3	1.679075722	0.191729583
Low Dose	Day 43	Liver - Nerve Median, Proximal, Right, Proximal	0.918084721	0.652778616	-0.120631726	1.956801168	3	2.812851705	0.067134087
Low Dose	Day 43	Liver - Nerve, Sciatic, Distal, Left, Distal	0.974035864	0.717697638	-0.167981234	2.116052962	3	2.714334875	0.072897052
Low Dose	Day 43	Liver - Nerve, Sciatic, Distal, Left, Proximal	0.788281938	0.648266781	-0.243255174	1.81981905	3	2.431967704	0.093166033
Low Dose	Day 43	Liver - Nerve, Sciatic, Distal, Right, Distal	1.023470791	0.716222852	-0.116199594	2.163141176	3	2.857967427	0.064684673
Low Dose	Day 43	Liver - Nerve, Sciatic, Distal, Right, Proximal	0.9688211	0.680133886	-0.113423686	2.051065886	3	2.848912897	0.065167202
Low Dose	Day 43	Liver - Nerve, Sciatic, Proximal, Left, Distal	0.924549933	0.649819571	-0.109458013	1.958557879	3	2.845558904	0.065347081
Low Dose	Day 43	Liver - Nerve, Sciatic, Proximal, Left, Proximal	0.89173202	0.668459841	-0.171936755	1.955400796	3	2.668019724	0.075818229
Low Dose	Day 43	Liver - Nerve, Sciatic, Proximal, Right, Distal	0.874035209	0.703880039	-0.245995006	1.994065424	3	2.483477753	0.088995683
Low Dose	Day 43	Liver - Nerve, Sciatic, Proximal, Right, Proximal	0.991929339	0.769414381	-0.232380638	2.216239315	3	2.57840083	0.08189337
Low Dose	Day 43	Liver - Nerve, Ulnar, Distal, Left, Distal	1.054086854	0.714992293	-0.083625437	2.191799145	3	2.948526478	0.060095893
Low Dose	Day 43	Liver - Nerve, Ulnar, Distal, Left, Proximal	0.912987543	0.670873958	-0.154522631	1.980497717	3	2.721785613	0.072440237
Low Dose	Day 43	Liver - Nerve, Ulnar, Distal, Right, Distal	1.047870105	0.716639576	-0.09246338	2.188203591	3	2.924399212	0.061277546
Low Dose	Day 43	Liver - Nerve, Ulnar, Distal, Right, Proximal	1.011902188	0.747675383	-0.177816193	2.201620568	3	2.706795516	0.073362944
Low Dose	Day 43	Liver - Nerve, Ulnar, Proximal, Left, Distal	0.923184348	0.733559412	-0.244072372	2.090441068	3	2.516999532	0.086404188
Low Dose	Day 43	Liver - Nerve, Ulnar, Proximal, Left, Proximal	0.975116474	0.70836384	-0.15204847	2.102281417	3	2.753151468	0.070555671
Low Dose	Day 43	Liver - Nerve, Ulnar, Proximal, Right, Distal	0.938617886	0.717915348	-0.203745638	2.080981409	3	2.614842789	0.079353047
Low Dose	Day 43	Liver - Nerve, Ulnar, Proximal, Right, Proximal	0.931199938	0.646318798	-0.097237497	1.959637373	3	2.881549914	0.063448703
Low Dose	Day 43	Liver - Nerve, Optic	0.737215007	0.823733527	-0.573528852	2.047958866	3	1.789935661	0.171399944
Low Dose	Day 43	Liver - Ovary	1.379305776	0.002358191	1.358118271	1.400493281	1	827.1734471	0.000769632
Low Dose	Day 43	Liver - Spinal Cord, Cervical	-0.550329457	1.162892951	-2.400751645	1.300092731	3	-0.946483434	0.413728333
Low Dose	Day 43	Liver - Spinal Cord, Lumbar	-0.226425604	0.862978261	-1.599616593	1.146765385	3	-0.524753899	0.636062185
Low Dose	Day 43	Liver - Spinal Cord, Sacral	-0.343079711	1.072156486	-2.049119936	1.362960513	3	-0.639980666	0.567708714
Low Dose	Day 43	Liver - Spinal Cord, Spinal Roots	0.180540649	0.870696476	-1.204931743	1.566013041	3	0.414703984	0.706229619
Low Dose	Day 43	Liver - Spinal Cord, Thoracic	-0.64034493	1.370066136	-2.820425887	1.539736027	3	-0.934764991	0.418865591
Low Dose	Day 43	Liver - Spleen	0.367160308	1.003742259	-1.230017614	1.964338231	3	0.731582844	0.517389734
Low Dose	Day 43	Liver - Testis	0.802780224	1.092011977	-9.008558121	10.61411857	1	1.039643066	0.48762804
Low Dose	Day 43	Muscle, Skeletal, Tibialis Anterior Left - Muscle, Skeletal, Tibialis Anterior Right	-0.011864785	0.09013204	-0.155284975	0.131555404	3	-0.263275643	0.809385101
Low Dose	Day 43	Muscle, Skeletal, Tibialis Anterior Left - Muscle, Skeletal, Bicep Brachii Left	0.010724195	0.03985598	-0.052695563	0.074143954	3	0.53814736	0.627838066
Low Dose	Day 43	Muscle, Skeletal, Tibialis Anterior Left - Muscle, Skeletal, Bicep Brachii Right	-0.00857375	0.010447674	-0.025198331	0.008050831	3	-1.641274435	0.19927813
Low Dose	Day 43	Muscle, Skeletal, Tibialis Anterior Left - Nerve, Femoral, Distal, Left, Distal	-0.195985394	0.140771764	-0.419984684	0.028013897	3	-2.784441812	0.068735961
Low Dose	Day 43	Muscle, Skeletal, Tibialis Anterior Left - Nerve, Femoral, Distal, Left, Proximal	-0.275154157	0.128560001	-0.479721807	-0.070586507	3	-4.280556231	0.023420993
Low Dose	Day 43	Muscle, Skeletal, Tibialis Anterior Left - Nerve, Femoral, Distal, Right, Distal	-0.206628423	0.12514456	-0.405761345	-0.007495501	3	-3.302235793	0.045656863
Low Dose	Day 43	Muscle, Skeletal, Tibialis Anterior Left - Nerve, Femoral, Distal, Right, Proximal	-0.258844329	0.206922682	-0.588104492	0.070415834	3	-2.50184587	0.087564061
Low Dose	Day 43	Muscle, Skeletal, Tibialis Anterior Left - Nerve, Femoral, Proximal, Left, Distal	-0.180013934	0.095355328	-0.331745539	-0.028282328	3	-3.775645005	0.032540029
Low Dose	Day 43	Muscle, Skeletal, Tibialis Anterior Left - Nerve, Femoral, Proximal, Left, Proximal	-0.108160347	0.172125749	-0.382050824	0.16573013	3	-1.256759638	0.297789037
Low Dose	Day 43	Muscle, Skeletal, Tibialis Anterior Left - Nerve, Femoral, Proximal, Right, Distal	-0.17830006	0.118493081	-0.366848994	0.010248874	3	-3.009459425	0.057236329
Low Dose	Day 43	Muscle, Skeletal, Tibialis Anterior Left - Nerve, Femoral, Proximal, Right, Proximal	-0.120610259	0.099469135	-0.27888785	0.037667332	3	-2.425079067	0.093741696
Low Dose	Day 43	Muscle, Skeletal, Tibialis Anterior Left - Nerve Median, Distal, Left, Distal	-0.053427127	0.06325207	-0.154075286	0.047221031	3	-1.689340045	0.189736105
Low Dose	Day 43	Muscle, Skeletal, Tibialis Anterior Left - Nerve Median, Distal, Left, Proximal	-0.12151843	0.205833671	-0.449045733	0.206008873	3	-1.180743939	0.322792114
Low Dose	Day 43	Muscle, Skeletal, Tibialis Anterior Left - Nerve Median, Distal, Right, Distal	-0.013355815	0.042816195	-0.081485935	0.054774305	3	-0.623867453	0.576934139
Low Dose	Day 43	Muscle, Skeletal, Tibialis Anterior Left - Nerve Median, Distal, Right, Proximal	-0.08148245	0.071089933	-0.194602398	0.031637498	3	-2.292376608	0.105725365
Low Dose	Day 43	Muscle, Skeletal, Tibialis Anterior Left - Nerve Median, Proximal, Left, Distal	-0.063435717	0.081760326	-0.193534641	0.066663207	3	-1.551748142	0.218523242
Low Dose	Day 43	Muscle, Skeletal, Tibialis Anterior Left - Nerve Median, Proximal, Left, Proximal	-0.116832485	0.154151244	-0.362121513	0.128456544	3	-1.515816305	0.226821532
Low Dose	Day 43	Muscle, Skeletal, Tibialis Anterior Left - Nerve Median, Proximal, Right, Distal	-0.210714294	0.334823448	-0.743493117	0.322064529	3	-1.258659125	0.297190419

Results of Evaluating Mean Differences Between Different Tissues Across Timepoint and Group for Log10 AAV2-ITR Target Copies/ug of DNA for Normalized Data

Group	Timepoint	Difference	Mean	StdDev	LowerCLMean	UpperCLMean	DF	tValue	P-Value
Low Dose	Day 43	Muscle, Skeletal, Tibialis Anterior Left - Nerve Median, Proximal, Right, Proximal	-0.145291015	0.104195687	-0.311089604	0.020507574	3	-2.788810545	0.068486561
Low Dose	Day 43	Muscle, Skeletal, Tibialis Anterior Left - Nerve, Sciatic, Distal, Left, Distal	-0.089339872	0.04267164	-0.157239973	-0.02143977	3	-4.187318421	0.024830044
Low Dose	Day 43	Muscle, Skeletal, Tibialis Anterior Left - Nerve, Sciatic, Distal, Left, Proximal	-0.275093798	0.177169769	-0.557010436	0.00682284	3	-3.105425936	0.053070391
Low Dose	Day 43	Muscle, Skeletal, Tibialis Anterior Left - Nerve, Sciatic, Distal, Right, Distal	-0.039904945	0.040117513	-0.103740861	0.023930971	3	-1.989402709	0.140765509
Low Dose	Day 43	Muscle, Skeletal, Tibialis Anterior Left - Nerve, Sciatic, Distal, Right, Proximal	-0.094554636	0.125256349	-0.293865438	0.104756166	3	-1.509777937	0.228249893
Low Dose	Day 43	Muscle, Skeletal, Tibialis Anterior Left - Nerve, Sciatic, Proximal, Left, Distal	-0.138825803	0.07102393	-0.251840725	-0.025810881	3	-3.909268395	0.029736792
Low Dose	Day 43	Muscle, Skeletal, Tibialis Anterior Left - Nerve, Sciatic, Proximal, Left, Proximal	-0.171643715	0.093321035	-0.320138308	-0.023149123	3	-3.678564313	0.034791971
Low Dose	Day 43	Muscle, Skeletal, Tibialis Anterior Left - Nerve, Sciatic, Proximal, Right, Distal	-0.189340527	0.099893045	-0.348292653	-0.0303884	3	-3.790865041	0.032204086
Low Dose	Day 43	Muscle, Skeletal, Tibialis Anterior Left - Nerve, Sciatic, Proximal, Right, Proximal	-0.071446397	0.079045942	-0.19722613	0.054333336	3	-1.807718269	0.168374328
Low Dose	Day 43	Muscle, Skeletal, Tibialis Anterior Left - Nerve, Ulnar, Distal, Left, Distal	-0.009288882	0.034839356	-0.064726073	0.046148309	3	-0.533240726	0.63084265
Low Dose	Day 43	Muscle, Skeletal, Tibialis Anterior Left - Nerve, Ulnar, Distal, Left, Proximal	-0.150388192	0.164503645	-0.412150202	0.111373817	3	-1.828387352	0.164934712
Low Dose	Day 43	Muscle, Skeletal, Tibialis Anterior Left - Nerve, Ulnar, Distal, Right, Distal	-0.01550563	0.055794184	-0.104286628	0.073275368	3	-0.555815287	0.617099156
Low Dose	Day 43	Muscle, Skeletal, Tibialis Anterior Left - Nerve, Ulnar, Distal, Right, Proximal	-0.051473548	0.080419355	-0.179438687	0.076491591	3	-1.280128353	0.2905107
Low Dose	Day 43	Muscle, Skeletal, Tibialis Anterior Left - Nerve, Ulnar, Proximal, Left, Distal	-0.140191388	0.082255578	-0.271078367	-0.009304408	3	-3.408677972	0.042191593
Low Dose	Day 43	Muscle, Skeletal, Tibialis Anterior Left - Nerve, Ulnar, Proximal, Left, Proximal	-0.088259262	0.13630886	-0.305157076	0.128638552	3	-1.294989362	0.285978792
Low Dose	Day 43	Muscle, Skeletal, Tibialis Anterior Left - Nerve, Ulnar, Proximal, Right, Distal	-0.12475785	0.13377855	-0.337629377	0.088113677	3	-1.86513981	0.159017417
Low Dose	Day 43	Muscle, Skeletal, Tibialis Anterior Left - Nerve, Ulnar, Proximal, Right, Proximal	-0.132175798	0.166902099	-0.397754283	0.133402687	3	-1.583872199	0.211388569
Low Dose	Day 43	Muscle, Skeletal, Tibialis Anterior Left - Nerve, Optic	-0.326160729	0.164370429	-0.587710761	-0.064610696	3	-3.968605917	0.028591493
Low Dose	Day 43	Muscle, Skeletal, Tibialis Anterior Left - Ovary	0.033428121	0.042210681	-0.345819797	0.41267604	1	1.11996542	0.464013095
Low Dose	Day 43	Muscle, Skeletal, Tibialis Anterior Left - Spinal Cord, Cervical	-1.613705193	0.741460762	-2.793534725	-0.433875661	3	-4.352772997	0.022399947
Low Dose	Day 43	Muscle, Skeletal, Tibialis Anterior Left - Spinal Cord, Lumbar	-1.289801339	0.506008599	-2.094973937	-0.484628742	3	-5.097942379	0.014594039
Low Dose	Day 43	Muscle, Skeletal, Tibialis Anterior Left - Spinal Cord, Sacral	-1.406455447	0.731774317	-2.570871683	-0.242039211	3	-3.843959577	0.031066422
Low Dose	Day 43	Muscle, Skeletal, Tibialis Anterior Left - Spinal Cord, Spinal Roots	-0.882835087	0.469140343	-1.629342062	-0.136328112	3	-3.763628947	0.032808432
Low Dose	Day 43	Muscle, Skeletal, Tibialis Anterior Left - Spinal Cord, Thoracic	-1.703720666	1.181079378	-3.583081517	0.175640186	3	-2.885023136	0.06326917
Low Dose	Day 43	Muscle, Skeletal, Tibialis Anterior Left - Spleen	-0.696215427	0.525596912	-1.532557403	0.140126548	3	-2.64923713	0.077044095
Low Dose	Day 43	Muscle, Skeletal, Tibialis Anterior Left - Testis	0.021906407	0.035208563	-0.294429981	0.338242795	1	0.879909188	0.540612836
Low Dose	Day 43	Muscle, Skeletal, Tibialis Anterior Right - Muscle, Skeletal, Bicep Brachii Left	0.022588981	0.077699473	-0.10104822	0.146226182	3	0.581444885	0.601746686
Low Dose	Day 43	Muscle, Skeletal, Tibialis Anterior Right - Muscle, Skeletal, Bicep Brachii Right	0.003291035	0.09046285	-0.140655546	0.147237617	3	0.072759932	0.94657662
Low Dose	Day 43	Muscle, Skeletal, Tibialis Anterior Right - Nerve, Femoral, Distal, Left, Distal	-0.184120608	0.078904197	-0.309674793	-0.058566424	3	-4.666940823	0.01857266
Low Dose	Day 43	Muscle, Skeletal, Tibialis Anterior Right - Nerve, Femoral, Distal, Left, Proximal	-0.263289371	0.096867113	-0.417426564	-0.109152179	3	-5.436094141	0.012220246
Low Dose	Day 43	Muscle, Skeletal, Tibialis Anterior Right - Nerve, Femoral, Distal, Right, Distal	-0.194763638	0.197268252	-0.508661448	0.119134172	3	-1.974607019	0.142804776
Low Dose	Day 43	Muscle, Skeletal, Tibialis Anterior Right - Nerve, Femoral, Distal, Right, Proximal	-0.246979544	0.282276237	-0.696144027	0.20218494	3	-1.749913819	0.17844094
Low Dose	Day 43	Muscle, Skeletal, Tibialis Anterior Right - Nerve, Femoral, Proximal, Left, Distal	-0.168149148	0.181915123	-0.457616703	0.121318406	3	-1.848654978	0.161640573
Low Dose	Day 43	Muscle, Skeletal, Tibialis Anterior Right - Nerve, Femoral, Proximal, Left, Proximal	-0.096295562	0.236033144	-0.471876965	0.279285842	3	-0.81594949	0.474290675
Low Dose	Day 43	Muscle, Skeletal, Tibialis Anterior Right - Nerve, Femoral, Proximal, Right, Distal	-0.166435274	0.205721962	-0.493784824	0.160914275	3	-1.618060341	0.204079506
Low Dose	Day 43	Muscle, Skeletal, Tibialis Anterior Right - Nerve, Femoral, Proximal, Right, Proximal	-0.108745473	0.179465698	-0.394315447	0.176824501	3	-1.211880315	0.312300567
Low Dose	Day 43	Muscle, Skeletal, Tibialis Anterior Right - Nerve Median, Distal, Left, Distal	-0.041562342	0.126012602	-0.242076512	0.158951828	3	-0.659653738	0.556595609
Low Dose	Day 43	Muscle, Skeletal, Tibialis Anterior Right - Nerve Median, Distal, Left, Proximal	-0.109653644	0.265966715	-0.532866039	0.31355875	3	-0.824566672	0.470064404
Low Dose	Day 43	Muscle, Skeletal, Tibialis Anterior Right - Nerve Median, Distal, Right, Distal	-0.00149103	0.090727861	-0.145859303	0.142877244	3	-0.032868177	0.975844231
Low Dose	Day 43	Muscle, Skeletal, Tibialis Anterior Right - Nerve Median, Distal, Right, Proximal	-0.069617665	0.14478457	-0.300002224	0.160766895	3	-0.961672431	0.407156138
Low Dose	Day 43	Muscle, Skeletal, Tibialis Anterior Right - Nerve Median, Proximal, Left, Distal	-0.051570932	0.150771672	-0.291482307	0.188340443	3	-0.684093121	0.543022308
Low Dose	Day 43	Muscle, Skeletal, Tibialis Anterior Right - Nerve Median, Proximal, Left, Proximal	-0.104967699	0.164816247	-0.367227128	0.157291729	3	-1.273754269	0.292477407
Low Dose	Day 43	Muscle, Skeletal, Tibialis Anterior Right - Nerve Median, Proximal, Right, Distal	-0.198849509	0.360105109	-0.771857096	0.374158078	3	-1.104397044	0.35004566
Low Dose	Day 43	Muscle, Skeletal, Tibialis Anterior Right - Nerve Median, Proximal, Right, Proximal	-0.133426229	0.180227775	-0.420208837	0.153356378	3	-1.480640037	0.235282679
Low Dose	Day 43	Muscle, Skeletal, Tibialis Anterior Right - Nerve, Sciatic, Distal, Left, Distal	-0.077475086	0.115383318	-0.261075693	0.10612552	3	-1.342916597	0.271862313
Low Dose	Day 43	Muscle, Skeletal, Tibialis Anterior Right - Nerve, Sciatic, Distal, Left, Proximal	-0.263229012	0.241603063	-0.647673399	0.121215375	3	-2.179020492	0.117456932
Low Dose	Day 43	Muscle, Skeletal, Tibialis Anterior Right - Nerve, Sciatic, Distal, Right, Distal	-0.028040159	0.064711686	-0.131010893	0.074930574	3	-0.8666181	0.449906088
Low Dose	Day 43	Muscle, Skeletal, Tibialis Anterior Right - Nerve, Sciatic, Distal, Right, Proximal	-0.08268985	0.040710872	-0.147469932	-0.017909769	3	-4.062298201	0.026896064
Low Dose	Day 43	Muscle, Skeletal, Tibialis Anterior Right - Nerve, Sciatic, Proximal, Left, Distal	-0.126961018	0.133325312	-0.339111341	0.085189306	3	-1.904529837	0.152947409
Low Dose	Day 43	Muscle, Skeletal, Tibialis Anterior Right - Nerve, Sciatic, Proximal, Left, Proximal	-0.15977893	0.177833814	-0.442752212	0.123194352	3	-1.796946559	0.170199659
Low Dose	Day 43	Muscle, Skeletal, Tibialis Anterior Right - Nerve, Sciatic, Proximal, Right, Distal	-0.177475741	0.055686381	-0.2660852	-0.088866282	3	-6.374116505	0.00781643
Low Dose	Day 43	Muscle, Skeletal, Tibialis Anterior Right - Nerve, Sciatic, Proximal, Right, Proximal	-0.059581611	0.120067347	-0.250635554	0.131472331	3	-0.992469856	0.394127646
Low Dose	Day 43	Muscle, Skeletal, Tibialis Anterior Right - Nerve, Ulnar, Distal, Left, Distal	0.002575904	0.102999412	-0.161319145	0.166470952	3	0.05001783	0.963252059
Low Dose	Day 43	Muscle, Skeletal, Tibialis Anterior Right - Nerve, Ulnar, Distal, Left, Proximal	-0.138523407	0.211700645	-0.475386375	0.198339561	3	-1.308672506	0.281871517
Low Dose	Day 43	Muscle, Skeletal, Tibialis Anterior Right - Nerve, Ulnar, Distal, Right, Distal	-0.003640845	0.060503178	-0.099914902	0.092633212	3	-0.120352186	0.911812024
Low Dose	Day 43	Muscle, Skeletal, Tibialis Anterior Right - Nerve, Ulnar, Distal, Right, Proximal	-0.039608763	0.119591847	-0.229906079	0.150688553	3	-0.662399044	0.555058039
Low Dose	Day 43	Muscle, Skeletal, Tibialis Anterior Right - Nerve, Ulnar, Proximal, Left, Distal	-0.128326602	0.121494542	-0.32165153	0.064998326	3	-2.112466953	0.125073604
Low Dose	Day 43	Muscle, Skeletal, Tibialis Anterior Right - Nerve, Ulnar, Proximal, Left, Proximal	-0.076394477	0.201385424	-0.396843626	0.244054673	3	-0.758689233	0.503201514
Low Dose	Day 43	Muscle, Skeletal, Tibialis Anterior Right - Nerve, Ulnar, Proximal, Right, Distal	-0.112893065	0.209840783	-0.446796576	0.221010447	3	-1.075987835	0.360759369
Low Dose	Day 43	Muscle, Skeletal, Tibialis Anterior Right - Nerve, Ulnar, Proximal, Right, Proximal	-0.120311012	0.23776657	-0.498650683	0.258028658	3	-1.012009488	0.386066094
Low Dose	Day 43	Muscle, Skeletal, Tibialis Anterior Right - Nerve, Optic	-0.314295943	0.175007938	-0.592772625	-0.035819261	3	-3.591790721	0.036976221
Low Dose	Day 43	Muscle, Skeletal, Tibialis Anterior Right - Ovary	0.090610644	0.078066755	-0.610791323	0.792012611	1	1.641451616	0.348338639
Low Dose	Day 43	Muscle, Skeletal, Tibialis Anterior Right - Spinal Cord, Cervical	-1.601840407	0.70184803	-2.718637242	-0.485043573	3	-4.564636045	0.019718731
Low Dose	Day 43	Muscle, Skeletal, Tibialis Anterior Right - Spinal Cord, Lumbar	-1.277936554	0.444948933	-1.985949598	-0.56992351	3	-5.744194263	0.01047897
Low Dose	Day 43	Muscle, Skeletal, Tibialis Anterior Right - Spinal Cord, Sacral	-1.394590662	0.660352557	-2.445358939	-0.343822384	3	-4.223776062	0.02426638
Low Dose	Day 43	Muscle, Skeletal, Tibialis Anterior Right - Spinal Cord, Spinal Roots	-0.870970301	0.40041208	-1.508115274	-0.233825328	3	-4.350369751	0.022432989
Low Dose	Day 43	Muscle, Skeletal, Tibialis Anterior Right - Spinal Cord, Thoracic	-1.69185588	1.152633706	-3.52595332	0.142241559	3	-2.935634923	0.060723682
Low Dose	Day 43	Muscle, Skeletal, Tibialis Anterior Right - Spleen	-0.684350642	0.58586489	-1.61659242	0.247891136	3	-2.336206362	0.101571836
Low Dose	Day 43	Muscle, Skeletal, Tibialis Anterior Right - Testis	-0.011546545	0.005899871	-0.06455478	0.041461691	1	-2.767735178	0.220723381
Low Dose	Day 43	Muscle, Skeletal, Bicep Brachii Left - Muscle, Skeletal, Bicep Brachii Right	-0.019297945	0.048318482	-0.096183433	0.057587542	3	-0.798781107	0.48280791
Low Dose	Day 43	Muscle, Skeletal, Bicep Brachii Left - Nerve, Femoral, Distal, Left, Distal	-0.206709589	0.145612256	-0.438411183	0.024992005	3	-2.83917843	0.065690987
Low Dose	Day 43	Muscle, Skeletal, Bicep Brachii Left - Nerve, Femoral, Distal, Left, Proximal	-0.285878352	0.145569834	-0.517512442	-0.054244262	3	-3.927714207	0.029374544
Low Dose	Day 43	Muscle, Skeletal, Bicep Brachii Left - Nerve, Femoral, Distal, Right, Distal	-0.217352618	0.119641839	-0.407729482	-0.026975755	3	-3.633388136	0.035907917
Low Dose	Day 43	Muscle, Skeletal, Bicep Brachii Left - Nerve, Femoral, Distal, Right, Proximal	-0.269568524	0.244746302	-0.659014507	0.119877458	3	-2.202840427	0.114866705
Low Dose	Day 43	Muscle, Skeletal, Bicep Brachii Left - Nerve, Femoral, Proximal, Left, Distal	-0.190738129	0.121073166	-0.383392554	0.001916296	3	-3.150791133	0.05123406
Low Dose	Day 43	Muscle, Skeletal, Bicep Brachii Left - Nerve, Femoral, Proximal, Left, Proximal	-0.118884542	0.211576199	-0.455549489	0.217780404	3	-1.123798829	0.342909606
Low Dose	Day 43	Muscle, Skeletal, Bicep Brachii Left - Nerve, Femoral, Proximal, Right, Distal	-0.189024255	0.147719291	-0.424078612	0.046030102	3	-2.559235874	0.08326954
Low Dose	Day 43	Muscle, Skeletal, Bicep Brachii Left - Nerve, Femoral, Proximal, Right, Proximal	-0.131334454	0.135209337	-0.346482682	0.083813773	3	-1.942683205	0.147324285
Low Dose	Day 43	Muscle, Skeletal, Bicep Brachii Left - Nerve Median, Distal, Left, Distal	-0.064151323	0.051355049	-0.145868665	0.01756602	3	-2.49834531	0.087834707
Low Dose	Day 43	Muscle, Skeletal, Bicep Brachii Left - Nerve Median, Distal, Left, Proximal	-0.132242625	0.241889771	-0.517143229	0.252657979	3	-1.093412298	0.354150691
Low Dose	Day 43	Muscle, Skeletal, Bicep Brachii Left - Nerve Median, Distal, Right, Distal	-0.02408001	0.046269761	-0.097705525	0.049545505	3	-1.040853027	0.374452266
Low Dose	Day 43	Muscle, Skeletal, Bicep Brachii Left - Nerve Median, Distal, Right, Proximal	-0.092206645	0.087280693	-0.231089705	0.046676415	3	-2.112876094	0.125025009
Low Dose	Day 43	Muscle, Skeletal, Bicep Brachii Left - Nerve Median, Proximal, Left, Distal	-0.074159912	0.104420396	-0.240316064	0.091996239	3	-1.420410479	0.250581295
Low Dose	Day 43	Muscle, Skeletal, Bicep Brachii Left - Nerve Median, Proximal, Left, Proximal	-0.12755668	0.185764458	-0.423149386	0.168036026	3	-1.373316312	0.263292233
Low Dose	Day 43	Muscle, Skeletal, Bicep Brachii Left - Nerve Median, Proximal, Right, Distal	-0.22143849	0.32750838	-0.742577407	0.299700428	3	-1.352261517	0.269196701
Low Dose	Day 43	Muscle, Skeletal, Bicep Brachii Left - Nerve Median, Proximal, Right, Proximal	-0.15601521	0.122750073	-0.351337969	0.039307549	3	-2.541997828	0.084531727
Low Dose	Day 43	Muscle, Skeletal, Bicep Brachii Left - Nerve, Sciatic, Distal, Left, Distal	-0.100064067	0.039317672	-0.162627257	-0.037500877	3	-5.090030124	0.014656504
Low Dose	Day 43	Muscle, Skeletal, Bicep Brachii Left - Nerve, Sciatic, Distal, Left, Proximal	-0.285817993	0.184244562	-0.578992206	0.007356221	3	-3.102593523	0.053187775
Low Dose	Day 43	Muscle, Skeletal, Bicep Brachii Left - Nerve, Sciatic, Distal, Right, Distal	-0.05062914	0.024305812	-0.089305112	-0.011953169	3	-4.166010924	0.025167314
Low Dose	Day 43	Muscle, Skeletal, Bicep Brachii Left - Nerve, Sciatic, Distal, Right, Proximal	-0.105278831	0.10927841	-0.279165167	0.068607505	3	-1.926800202	0.149635262
Low Dose	Day 43	Muscle, Skeletal, Bicep Brachii Left - Nerve, Sciatic, Proximal, Left, Distal	-0.149549998	0.110444245	-0.325291437	0.026191441	3	-2.708153762	0.07327874
Low Dose	Day 43	Muscle, Skeletal, Bicep Brachii Left - Nerve, Sciatic, Proximal, Left, Proximal	-0.182367911	0.128894118	-0.387467216	0.022731395	3	-2.829732078	0.066204299
Low Dose	Day 43	Muscle, Skeletal, Bicep Brachii Left - Nerve, Sciatic, Proximal, Right, Distal	-0.200064722	0.069816027	-0.3111576	-0.088971844	3	-5.731197574	0.010545608

Results of Evaluating Mean Differences Between Different Tissues Across Timepoint and Group for Log10 AAV2-ITR Target Copies/ug of DNA for Normalized Data

Group	Timepoint	Difference	Mean	StdDev	LowerCLMean	UpperCLMean	DF	tValue	P-Value
Low Dose	Day 43	Muscle, Skeletal, Bicep Brachii Left - Nerve, Sciatic, Proximal, Right, Proximal	-0.082170592	0.059541526	-0.176914447	0.012573263	3	-2.760110372	0.070145842
Low Dose	Day 43	Muscle, Skeletal, Bicep Brachii Left - Nerve, Ulnar, Distal, Left, Distal	-0.020013077	0.059253432	-0.114298509	0.074272355	3	-0.675507786	0.547761045
Low Dose	Day 43	Muscle, Skeletal, Bicep Brachii Left - Nerve, Ulnar, Distal, Left, Proximal	-0.161112388	0.201525987	-0.481785205	0.159560429	3	-1.598924185	0.208135146
Low Dose	Day 43	Muscle, Skeletal, Bicep Brachii Left - Nerve, Ulnar, Distal, Right, Distal	-0.026229826	0.057099081	-0.117087206	0.064627555	3	-0.918747724	0.425981986
Low Dose	Day 43	Muscle, Skeletal, Bicep Brachii Left - Nerve, Ulnar, Distal, Right, Proximal	-0.062197743	0.093931047	-0.211663	0.087267513	3	-1.324327692	0.277248239
Low Dose	Day 43	Muscle, Skeletal, Bicep Brachii Left - Nerve, Ulnar, Proximal, Left, Distal	-0.150915583	0.102498941	-0.314014271	0.012183105	3	-2.94472472	0.060280181
Low Dose	Day 43	Muscle, Skeletal, Bicep Brachii Left - Nerve, Ulnar, Proximal, Left, Proximal	-0.098983457	0.171226151	-0.371442472	0.173475558	3	-1.156172197	0.331323305
Low Dose	Day 43	Muscle, Skeletal, Bicep Brachii Left - Nerve, Ulnar, Proximal, Right, Distal	-0.135482045	0.167139967	-0.401439031	0.130474941	3	-1.621180708	0.203426623
Low Dose	Day 43	Muscle, Skeletal, Bicep Brachii Left - Nerve, Ulnar, Proximal, Right, Proximal	-0.142899993	0.205978214	-0.470657295	0.184857309	3	-1.387525317	0.259385784
Low Dose	Day 43	Muscle, Skeletal, Bicep Brachii Left - Nerve, Optic	-0.336884924	0.165516736	-0.600258987	-0.073510861	3	-4.070705249	0.026750335
Low Dose	Day 43	Muscle, Skeletal, Bicep Brachii Left - Ovary	0.040404706	0.04330697	-0.34869298	0.429502392	1	1.319438498	0.412871345
Low Dose	Day 43	Muscle, Skeletal, Bicep Brachii Left - Spinal Cord, Cervical	-1.624429388	0.702893715	-2.742890141	-0.505968635	3	-4.622119544	0.019063831
Low Dose	Day 43	Muscle, Skeletal, Bicep Brachii Left - Spinal Cord, Lumbar	-1.300525535	0.472283284	-2.05203363	-0.549017439	3	-5.507396006	0.01178544
Low Dose	Day 43	Muscle, Skeletal, Bicep Brachii Left - Spinal Cord, Sacral	-1.417179642	0.696883135	-2.526076221	-0.308283064	3	-4.067194546	0.026811066
Low Dose	Day 43	Muscle, Skeletal, Bicep Brachii Left - Spinal Cord, Spinal Roots	-0.893559282	0.434832193	-1.585474336	-0.201644228	3	-4.109903984	0.026084131
Low Dose	Day 43	Muscle, Skeletal, Bicep Brachii Left - Spinal Cord, Thoracic	-1.714444861	1.149016807	-3.542787007	0.113897285	3	-2.984194576	0.058400825
Low Dose	Day 43	Muscle, Skeletal, Bicep Brachii Left - Spleen	-0.706939623	0.55140051	-1.584340881	0.170461636	3	-2.564160204	0.08291324
Low Dose	Day 43	Muscle, Skeletal, Bicep Brachii Left - Testis	-0.006518568	0.024045342	-0.222557392	0.209520255	1	-0.383385999	0.766931952
Low Dose	Day 43	Muscle, Skeletal, Bicep Brachii Right - Nerve, Femoral, Distal, Left, Distal	-0.187411644	0.137007285	-0.405420808	0.030597521	3	-2.735790916	0.071591124
Low Dose	Day 43	Muscle, Skeletal, Bicep Brachii Right - Nerve, Femoral, Distal, Left, Proximal	-0.266580407	0.12219572	-0.461021065	-0.072139748	3	-4.363170941	0.022257709
Low Dose	Day 43	Muscle, Skeletal, Bicep Brachii Right - Nerve, Femoral, Distal, Right, Distal	-0.198054673	0.132594092	-0.409041462	0.012932116	3	-2.987383079	0.058252233
Low Dose	Day 43	Muscle, Skeletal, Bicep Brachii Right - Nerve, Femoral, Distal, Right, Proximal	-0.250270579	0.20158851	-0.571042883	0.070501725	3	-2.482984566	0.089034516
Low Dose	Day 43	Muscle, Skeletal, Bicep Brachii Right - Nerve, Femoral, Proximal, Left, Distal	-0.171440184	0.093937533	-0.320915761	-0.021964607	3	-3.650089134	0.035490108
Low Dose	Day 43	Muscle, Skeletal, Bicep Brachii Right - Nerve, Femoral, Proximal, Left, Proximal	-0.099586597	0.165441895	-0.36284157	0.163668376	3	-1.20388608	0.314960671
Low Dose	Day 43	Muscle, Skeletal, Bicep Brachii Right - Nerve, Femoral, Proximal, Right, Distal	-0.16972631	0.118469371	-0.358237516	0.018784897	3	-2.865319664	0.064296138
Low Dose	Day 43	Muscle, Skeletal, Bicep Brachii Right - Nerve, Femoral, Proximal, Right, Proximal	-0.112036509	0.093712404	-0.261153856	0.037080838	3	-2.391071068	0.096648072
Low Dose	Day 43	Muscle, Skeletal, Bicep Brachii Right - Nerve Median, Distal, Left, Distal	-0.044853377	0.07325853	-0.161424046	0.071717291	3	-1.224523004	0.308140505
Low Dose	Day 43	Muscle, Skeletal, Bicep Brachii Right - Nerve Median, Distal, Left, Proximal	-0.11294468	0.201490849	-0.433561584	0.207672224	3	-1.12108992	0.34389723
Low Dose	Day 43	Muscle, Skeletal, Bicep Brachii Right - Nerve Median, Distal, Right, Distal	-0.004782065	0.050335117	-0.084876468	0.075312338	3	-0.1900091	0.861431948
Low Dose	Day 43	Muscle, Skeletal, Bicep Brachii Right - Nerve Median, Distal, Right, Proximal	-0.0729087	0.076760519	-0.195051816	0.049234416	3	-1.89964061	0.153685974
Low Dose	Day 43	Muscle, Skeletal, Bicep Brachii Right - Nerve Median, Proximal, Left, Distal	-0.054861967	0.084769657	-0.189749409	0.080025474	3	-1.294377465	0.286163924
Low Dose	Day 43	Muscle, Skeletal, Bicep Brachii Right - Nerve Median, Proximal, Left, Proximal	-0.108258735	0.145876793	-0.340381264	0.123863795	3	-1.484248903	0.234398887
Low Dose	Day 43	Muscle, Skeletal, Bicep Brachii Right - Nerve Median, Proximal, Right, Distal	-0.202140544	0.34251666	-0.747160985	0.342879896	3	-1.180325325	0.322935581
Low Dose	Day 43	Muscle, Skeletal, Bicep Brachii Right - Nerve Median, Proximal, Right, Proximal	-0.136717265	0.102413816	-0.2996805	0.02624597	3	-2.669898853	0.075696918
Low Dose	Day 43	Muscle, Skeletal, Bicep Brachii Right - Nerve, Sciatic, Distal, Left, Distal	-0.080766122	0.051498793	-0.162712193	0.00117995	3	-3.136621934	0.051798886
Low Dose	Day 43	Muscle, Skeletal, Bicep Brachii Right - Nerve, Sciatic, Distal, Left, Proximal	-0.266520048	0.176853457	-0.547933363	0.014893268	3	-3.014021347	0.05702918
Low Dose	Day 43	Muscle, Skeletal, Bicep Brachii Right - Nerve, Sciatic, Distal, Right, Distal	-0.031331195	0.047132783	-0.10632997	0.043667581	3	-1.329486306	0.27574237
Low Dose	Day 43	Muscle, Skeletal, Bicep Brachii Right - Nerve, Sciatic, Distal, Right, Proximal	-0.085980886	0.124945336	-0.284796797	0.112835026	3	-1.376296044	0.262467838
Low Dose	Day 43	Muscle, Skeletal, Bicep Brachii Right - Nerve, Sciatic, Proximal, Left, Distal	-0.130252053	0.062199121	-0.229224735	-0.031279371	3	-4.18822806	0.024815776
Low Dose	Day 43	Muscle, Skeletal, Bicep Brachii Right - Nerve, Sciatic, Proximal, Left, Proximal	-0.163069965	0.089198038	-0.305003949	-0.021135981	3	-3.656357657	0.035334893
Low Dose	Day 43	Muscle, Skeletal, Bicep Brachii Right - Nerve, Sciatic, Proximal, Right, Distal	-0.180766777	0.103512342	-0.345478012	-0.016055541	3	-3.492661312	0.039690514
Low Dose	Day 43	Muscle, Skeletal, Bicep Brachii Right - Nerve, Sciatic, Proximal, Right, Proximal	-0.062872647	0.089276853	-0.204932042	0.079186749	3	-1.408487072	0.253735861
Low Dose	Day 43	Muscle, Skeletal, Bicep Brachii Right - Nerve, Ulnar, Distal, Left, Distal	-0.000715132	0.03881969	-0.062485921	0.061055657	3	-0.036843771	0.97292412
Low Dose	Day 43	Muscle, Skeletal, Bicep Brachii Right - Nerve, Ulnar, Distal, Left, Proximal	-0.141814442	0.158243607	-0.393615333	0.109986449	3	-1.792356041	0.17098447
Low Dose	Day 43	Muscle, Skeletal, Bicep Brachii Right - Nerve, Ulnar, Distal, Right, Distal	-0.00693188	0.058703977	-0.100343007	0.086479247	3	-0.236163908	0.828510988
Low Dose	Day 43	Muscle, Skeletal, Bicep Brachii Right - Nerve, Ulnar, Distal, Right, Proximal	-0.042899798	0.083950978	-0.176484538	0.090684942	3	-1.022020211	0.381996811
Low Dose	Day 43	Muscle, Skeletal, Bicep Brachii Right - Nerve, Ulnar, Proximal, Left, Distal	-0.131617638	0.083320407	-0.264198999	0.000963724	3	-3.159313351	0.050898079
Low Dose	Day 43	Muscle, Skeletal, Bicep Brachii Right - Nerve, Ulnar, Proximal, Left, Proximal	-0.079685512	0.133007848	-0.291330679	0.131959655	3	-1.198207672	0.316864209
Low Dose	Day 43	Muscle, Skeletal, Bicep Brachii Right - Nerve, Ulnar, Proximal, Right, Distal	-0.1161841	0.1319021	-0.326069775	0.093701575	3	-1.761671731	0.176338531
Low Dose	Day 43	Muscle, Skeletal, Bicep Brachii Right - Nerve, Ulnar, Proximal, Right, Proximal	-0.123602048	0.160594112	-0.379143117	0.131939022	3	-1.539309829	0.221357212
Low Dose	Day 43	Muscle, Skeletal, Bicep Brachii Right - Nerve, Optic	-0.317586979	0.169117275	-0.586690302	-0.048483655	3	-3.755819484	0.032984394
Low Dose	Day 43	Muscle, Skeletal, Bicep Brachii Right - Ovary	0.043044282	0.037259501	-0.291719053	0.377807616	1	1.633779443	0.349665243
Low Dose	Day 43	Muscle, Skeletal, Bicep Brachii Right - Spinal Cord, Cervical	-1.605131443	0.748209218	-2.795699274	-0.414563611	3	-4.290595206	0.023275504
Low Dose	Day 43	Muscle, Skeletal, Bicep Brachii Right - Spinal Cord, Lumbar	-1.281227589	0.509530003	-2.092003527	-0.470451652	3	-5.029056512	0.015149762
Low Dose	Day 43	Muscle, Skeletal, Bicep Brachii Right - Spinal Cord, Sacral	-1.397881697	0.735819402	-2.568734566	-0.227028828	3	-3.799523885	0.032014948
Low Dose	Day 43	Muscle, Skeletal, Bicep Brachii Right - Spinal Cord, Spinal Roots	-0.874261337	0.472952217	-1.626833854	-0.121688819	3	-3.697038752	0.034348404
Low Dose	Day 43	Muscle, Skeletal, Bicep Brachii Right - Spinal Cord, Thoracic	-1.695146916	1.185408706	-3.581396695	0.191102863	3	-2.860021032	0.064575854
Low Dose	Day 43	Muscle, Skeletal, Bicep Brachii Right - Spleen	-0.687641677	0.525562802	-1.523929376	0.148646021	3	-2.616782144	0.0792206
Low Dose	Day 43	Muscle, Skeletal, Bicep Brachii Right - Testis	0.029437747	0.052488639	-0.44215396	0.501029454	1	0.793148044	0.573115291
Low Dose	Day 43	Nerve, Femoral, Distal, Left, Distal - Nerve, Femoral, Distal, Left, Proximal	-0.079168763	0.045185266	-0.151068604	-0.007268923	3	-3.50418493	0.039362213
Low Dose	Day 43	Nerve, Femoral, Distal, Left, Distal - Nerve, Femoral, Distal, Right, Distal	-0.01064303	0.261570486	-0.426860043	0.405573984	3	-0.081377909	0.940266537
Low Dose	Day 43	Nerve, Femoral, Distal, Left, Distal - Nerve, Femoral, Distal, Right, Proximal	-0.062858936	0.287030217	-0.519588063	0.393870191	3	-0.437995248	0.691008791
Low Dose	Day 43	Nerve, Femoral, Distal, Left, Distal - Nerve, Femoral, Proximal, Left, Distal	0.01597146	0.226030066	-0.343692814	0.375635733	3	0.14132155	0.896572114
Low Dose	Day 43	Nerve, Femoral, Distal, Left, Distal - Nerve, Femoral, Proximal, Left, Proximal	0.087825046	0.230882982	-0.2795613	0.455211393	3	0.760775399	0.502122925
Low Dose	Day 43	Nerve, Femoral, Distal, Left, Distal - Nerve, Femoral, Proximal, Right, Distal	0.017685334	0.236689689	-0.358940779	0.394311447	3	0.149438988	0.890688163
Low Dose	Day 43	Nerve, Femoral, Distal, Left, Distal - Nerve, Femoral, Proximal, Right, Proximal	0.075375135	0.207252351	-0.254409605	0.405159874	3	0.727375436	0.519620894
Low Dose	Day 43	Nerve, Femoral, Distal, Left, Distal - Nerve Median, Distal, Left, Distal	0.142558266	0.188162846	-0.156850811	0.441967343	3	1.515264773	0.226951586
Low Dose	Day 43	Nerve, Femoral, Distal, Left, Distal - Nerve Median, Distal, Left, Proximal	0.074466964	0.254501257	-0.330501329	0.479435256	3	0.585199182	0.599520514
Low Dose	Day 43	Nerve, Femoral, Distal, Left, Distal - Nerve Median, Distal, Right, Distal	0.182629578	0.138650501	-0.037994309	0.403253466	3	2.634387568	0.078030651
Low Dose	Day 43	Nerve, Femoral, Distal, Left, Distal - Nerve Median, Distal, Right, Proximal	0.114502944	0.184909301	-0.179729017	0.408734904	3	1.238476842	0.303614999
Low Dose	Day 43	Nerve, Femoral, Distal, Left, Distal - Nerve Median, Proximal, Left, Distal	0.132549676	0.179288815	-0.152738837	0.41783819	3	1.478616235	0.235779893
Low Dose	Day 43	Nerve, Femoral, Distal, Left, Distal - Nerve Median, Proximal, Left, Proximal	0.079152909	0.124047054	-0.118233635	0.276539453	3	1.276175555	0.291728697
Low Dose	Day 43	Nerve, Femoral, Distal, Left, Distal - Nerve Median, Proximal, Right, Distal	-0.014728901	0.383438532	-0.62486517	0.595407369	3	-0.076825356	0.943599223
Low Dose	Day 43	Nerve, Femoral, Distal, Left, Distal - Nerve Median, Proximal, Right, Proximal	0.050694379	0.230339214	-0.315826711	0.417215468	3	0.440171502	0.689596385
Low Dose	Day 43	Nerve, Femoral, Distal, Left, Distal - Nerve, Sciatic, Distal, Left, Distal	0.106645522	0.177440091	-0.175701259	0.388992303	3	1.20204539	0.315576434
Low Dose	Day 43	Nerve, Femoral, Distal, Left, Distal - Nerve, Sciatic, Distal, Left, Proximal	-0.079108404	0.299178725	-0.555168517	0.396951709	3	-0.528837097	0.633547386
Low Dose	Day 43	Nerve, Femoral, Distal, Left, Distal - Nerve, Sciatic, Distal, Right, Distal	0.156080449	0.12623627	-0.044789627	0.356950525	3	2.472830486	0.089838672
Low Dose	Day 43	Nerve, Femoral, Distal, Left, Distal - Nerve, Sciatic, Distal, Right, Proximal	0.101430758	0.087669881	-0.038071587	0.240933102	3	2.313924834	0.103658141
Low Dose	Day 43	Nerve, Femoral, Distal, Left, Distal - Nerve, Sciatic, Proximal, Left, Distal	0.057159591	0.149014793	-0.179956197	0.294275379	3	0.767166663	0.498830415
Low Dose	Day 43	Nerve, Femoral, Distal, Left, Distal - Nerve, Sciatic, Proximal, Left, Proximal	0.024341678	0.206878432	-0.304848073	0.353531429	3	0.235323498	0.829106509
Low Dose	Day 43	Nerve, Femoral, Distal, Left, Distal - Nerve, Sciatic, Proximal, Right, Distal	0.006644867	0.132938819	-0.204890459	0.218180193	3	0.099968798	0.926675134
Low Dose	Day 43	Nerve, Femoral, Distal, Left, Distal - Nerve, Sciatic, Proximal, Right, Proximal	0.124538997	0.180140557	-0.162104829	0.411182822	3	1.382686926	0.260708982
Low Dose	Day 43	Nerve, Femoral, Distal, Left, Distal - Nerve, Ulnar, Distal, Left, Distal	0.186696512	0.142371133	-0.039847731	0.413240754	3	2.622673686	0.078819913
Low Dose	Day 43	Nerve, Femoral, Distal, Left, Distal - Nerve, Ulnar, Distal, Left, Proximal	0.045597201	0.192721628	-0.261065915	0.352260317	3	0.473192363	0.668376379
Low Dose	Day 43	Nerve, Femoral, Distal, Left, Distal - Nerve, Ulnar, Distal, Right, Distal	0.180479763	0.102480205	0.017410888	0.343548639	3	3.522236567	0.038854856
Low Dose	Day 43	Nerve, Femoral, Distal, Left, Distal - Nerve, Ulnar, Distal, Right, Proximal	0.144511845	0.142837721	-0.082774842	0.371798533	3	2.023440936	0.136202984
Low Dose	Day 43	Nerve, Femoral, Distal, Left, Distal - Nerve, Ulnar, Proximal, Left, Distal	0.055794006	0.135586498	-0.159954369	0.271542381	3	0.823002389	0.470829185
Low Dose	Day 43	Nerve, Femoral, Distal, Left, Distal - Nerve, Ulnar, Proximal, Left, Proximal	0.107726131	0.203575301	-0.216207601	0.431659864	3	1.058341859	0.36757465
Low Dose	Day 43	Nerve, Femoral, Distal, Left, Distal - Nerve, Ulnar, Proximal, Right, Distal	0.071227543	0.223659907	-0.284665279	0.427120366	3	0.636927238	0.569448422
Low Dose	Day 43	Nerve, Femoral, Distal, Left, Distal - Nerve, Ulnar, Proximal, Right, Proximal	0.063809596	0.24094756	-0.319591741	0.447210933	3	0.529655463	0.633044158
Low Dose	Day 43	Nerve, Femoral, Distal, Left, Distal - Nerve, Optic	-0.130175335	0.182668464	-0.420841625	0.160490954	3	-1.425263367	0.249309511
Low Dose	Day 43	Nerve, Femoral, Distal, Left, Distal - Ovary	0.258888098	0.192974572	-1.474919645	1.992695842	1	1.897260634	0.30880695
Low Dose	Day 43	Nerve, Femoral, Distal, Left, Distal - Spinal Cord, Cervical	-1.417719799	0.759236567	-2.625834603	-0.209604996	3	-3.73459304	0.033468814

Results of Evaluating Mean Differences Between Different Tissues Across Timepoint and Group for Log10 AAV2-ITR Target Copies/ug of DNA for Normalized Data

Group	Timepoint	Difference	Mean	StdDev	LowerCLMean	UpperCLMean	DF	tValue	P-Value
Low Dose	Day 43	Nerve, Femoral, Distal, Left, Distal - Spinal Cord, Lumbar	-1.093815946	0.486110677	-1.867326509	-0.320305382	3	-4.500275343	0.020487043
Low Dose	Day 43	Nerve, Femoral, Distal, Left, Distal - Spinal Cord, Sacral	-1.210470053	0.687946729	-2.305146817	-0.11579329	3	-3.519080771	0.038942948
Low Dose	Day 43	Nerve, Femoral, Distal, Left, Distal - Spinal Cord, Spinal Roots	-0.686849693	0.43551285	-1.379847823	0.006148437	3	-3.154210919	0.051098903
Low Dose	Day 43	Nerve, Femoral, Distal, Left, Distal - Spinal Cord, Thoracic	-1.507735272	1.211422611	-3.435378979	0.419908434	3	-2.489197838	0.088546801
Low Dose	Day 43	Nerve, Femoral, Distal, Left, Distal - Spleen	-0.500230034	0.575531332	-1.416028814	0.415568747	3	-1.738324245	0.180541371
Low Dose	Day 43	Nerve, Femoral, Distal, Left, Distal - Testis	0.188417217	0.060956604	-0.359256137	0.736090572	1	4.37134237	0.143171278
Low Dose	Day 43	Nerve, Femoral, Distal, Left, Proximal - Nerve, Femoral, Distal, Right, Distal	0.068525734	0.253120336	-0.334245206	0.471296673	3	0.541447873	0.625822408
Low Dose	Day 43	Nerve, Femoral, Distal, Left, Proximal - Nerve, Femoral, Distal, Right, Proximal	0.016309828	0.246801501	-0.376406435	0.40902609	3	0.1321696	0.90321664
Low Dose	Day 43	Nerve, Femoral, Distal, Left, Proximal - Nerve, Femoral, Proximal, Left, Distal	0.095140223	0.20162351	-0.225687774	0.41596822	3	0.943741362	0.414925209
Low Dose	Day 43	Nerve, Femoral, Distal, Left, Proximal - Nerve, Femoral, Proximal, Left, Proximal	0.16699381	0.189432172	-0.134435049	0.468422668	3	1.763098715	0.176085311
Low Dose	Day 43	Nerve, Femoral, Distal, Left, Proximal - Nerve, Femoral, Proximal, Right, Distal	0.096854097	0.209627711	-0.23671037	0.430418565	3	0.924058148	0.423610453
Low Dose	Day 43	Nerve, Femoral, Distal, Left, Proximal - Nerve, Femoral, Proximal, Right, Proximal	0.154543898	0.175448559	-0.124633911	0.433721707	3	1.761700401	0.176333439
Low Dose	Day 43	Nerve, Femoral, Distal, Left, Proximal - Nerve Median, Distal, Left, Distal	0.221727029	0.184145454	-0.07128948	0.514743539	3	2.408172721	0.095173014
Low Dose	Day 43	Nerve, Femoral, Distal, Left, Proximal - Nerve Median, Distal, Left, Proximal	0.153635727	0.217077996	-0.191783806	0.49905526	3	1.415488719	0.251878285
Low Dose	Day 43	Nerve, Femoral, Distal, Left, Proximal - Nerve Median, Distal, Right, Distal	0.261798342	0.133842205	0.048825526	0.474771157	3	3.912044662	0.029681905
Low Dose	Day 43	Nerve, Femoral, Distal, Left, Proximal - Nerve Median, Distal, Right, Proximal	0.193671707	0.171068165	-0.078535918	0.465879332	3	2.264263567	0.108497873
Low Dose	Day 43	Nerve, Femoral, Distal, Left, Proximal - Nerve Median, Proximal, Left, Distal	0.21171844	0.161539334	-0.045326688	0.468763568	3	2.621261764	0.07891571
Low Dose	Day 43	Nerve, Femoral, Distal, Left, Proximal - Nerve Median, Proximal, Left, Proximal	0.158321672	0.080452404	0.030303944	0.286339401	3	3.935784727	0.029217835
Low Dose	Day 43	Nerve, Femoral, Distal, Left, Proximal - Nerve Median, Proximal, Right, Distal	0.064439863	0.38896454	-0.554489519	0.683369244	3	0.331340551	0.762183093
Low Dose	Day 43	Nerve, Femoral, Distal, Left, Proximal - Nerve Median, Proximal, Right, Proximal	0.129863142	0.208916633	-0.202569842	0.462296126	3	1.243205388	0.302097012
Low Dose	Day 43	Nerve, Femoral, Distal, Left, Proximal - Nerve, Sciatic, Distal, Left, Distal	0.185814285	0.169915639	-0.084559414	0.456187984	3	2.187135758	0.116566665
Low Dose	Day 43	Nerve, Femoral, Distal, Left, Proximal - Nerve, Sciatic, Distal, Left, Proximal	6.03593E-05	0.281500847	-0.447870305	0.447991024	3	0.000428839	0.999684758
Low Dose	Day 43	Nerve, Femoral, Distal, Left, Proximal - Nerve, Sciatic, Distal, Right, Distal	0.235249212	0.127071397	0.033050263	0.437448161	3	3.702630446	0.034215577
Low Dose	Day 43	Nerve, Femoral, Distal, Left, Proximal - Nerve, Sciatic, Distal, Right, Proximal	0.180599521	0.114360848	-0.001374108	0.36257315	3	3.158415207	0.050933357
Low Dose	Day 43	Nerve, Femoral, Distal, Left, Proximal - Nerve, Sciatic, Proximal, Left, Distal	0.136328354	0.116178679	-0.04853785	0.321194558	3	2.346873883	0.10059104
Low Dose	Day 43	Nerve, Femoral, Distal, Left, Proximal - Nerve, Sciatic, Proximal, Left, Proximal	0.103510442	0.176606676	-0.17751019	0.384531073	3	1.17221437	0.325728131
Low Dose	Day 43	Nerve, Femoral, Distal, Left, Proximal - Nerve, Sciatic, Proximal, Right, Distal	0.08581363	0.149938891	-0.152772605	0.324399865	3	1.144648059	0.335402202
Low Dose	Day 43	Nerve, Femoral, Distal, Left, Proximal - Nerve, Sciatic, Proximal, Right, Proximal	0.20370776	0.182235489	-0.08626957	0.49368509	3	2.235654108	0.111409948
Low Dose	Day 43	Nerve, Femoral, Distal, Left, Proximal - Nerve, Ulnar, Distal, Left, Distal	0.265865275	0.12907598	0.060476587	0.471253963	3	4.119515885	0.025924046
Low Dose	Day 43	Nerve, Femoral, Distal, Left, Proximal - Nerve, Ulnar, Distal, Left, Proximal	0.124765965	0.152920291	-0.118564343	0.368096272	3	1.631777753	0.201226816
Low Dose	Day 43	Nerve, Femoral, Distal, Left, Proximal - Nerve, Ulnar, Distal, Right, Distal	0.259648527	0.102777854	0.096106026	0.423191027	3	5.052616248	0.014956643
Low Dose	Day 43	Nerve, Femoral, Distal, Left, Proximal - Nerve, Ulnar, Distal, Right, Proximal	0.223680609	0.13338988	0.011427544	0.435933674	3	3.353786794	0.043935223
Low Dose	Day 43	Nerve, Femoral, Distal, Left, Proximal - Nerve, Ulnar, Proximal, Left, Distal	0.134962769	0.120871602	-0.057370922	0.32729646	3	2.233159276	0.111668329
Low Dose	Day 43	Nerve, Femoral, Distal, Left, Proximal - Nerve, Ulnar, Proximal, Left, Proximal	0.186894895	0.170486858	-0.084387741	0.458177531	3	2.192484473	0.11598432
Low Dose	Day 43	Nerve, Femoral, Distal, Left, Proximal - Nerve, Ulnar, Proximal, Right, Distal	0.150396307	0.192711113	-0.156250078	0.457042691	3	1.560847266	0.216475551
Low Dose	Day 43	Nerve, Femoral, Distal, Left, Proximal - Nerve, Ulnar, Proximal, Right, Proximal	0.142978359	0.200659307	-0.176315376	0.462272095	3	1.425085746	0.249355937
Low Dose	Day 43	Nerve, Femoral, Distal, Left, Proximal - Nerve, Optic	-0.051006572	0.185713583	-0.346518325	0.244505182	3	-0.549303619	0.621042424
Low Dose	Day 43	Nerve, Femoral, Distal, Left, Proximal - Ovary	0.319406946	0.161619785	-1.132689212	1.771503105	1	2.794890705	0.218744369
Low Dose	Day 43	Nerve, Femoral, Distal, Left, Proximal - Spinal Cord, Cervical	-1.338551036	0.793255249	-2.600797154	-0.076304918	3	-3.374830579	0.043256137
Low Dose	Day 43	Nerve, Femoral, Distal, Left, Proximal - Spinal Cord, Lumbar	-1.014647182	0.521020183	-1.843706561	-0.185587804	3	-3.894847898	0.030023996
Low Dose	Day 43	Nerve, Femoral, Distal, Left, Proximal - Spinal Cord, Sacral	-1.13130129	0.728949346	-2.291222366	0.028619786	3	-3.103922918	0.05313264
Low Dose	Day 43	Nerve, Femoral, Distal, Left, Proximal - Spinal Cord, Spinal Roots	-0.60768093	0.474163762	-1.362181286	0.146819426	3	-2.563169009	0.082984807
Low Dose	Day 43	Nerve, Femoral, Distal, Left, Proximal - Spinal Cord, Thoracic	-1.428566509	1.236710827	-3.39644941	0.539316392	3	-2.310267651	0.10400552
Low Dose	Day 43	Nerve, Femoral, Distal, Left, Proximal - Spleen	-0.421061271	0.547887047	-1.292871824	0.450749283	3	-1.537036778	0.221879493
Low Dose	Day 43	Nerve, Femoral, Distal, Left, Proximal - Testis	0.286235896	0.122199764	-0.811685424	1.384157215	1	3.312597935	0.186643368
Low Dose	Day 43	Nerve, Femoral, Distal, Right, Distal - Nerve, Femoral, Distal, Right, Proximal	-0.052215906	0.236796091	-0.429011328	0.324579516	3	-0.44102	0.689046161
Low Dose	Day 43	Nerve, Femoral, Distal, Right, Distal - Nerve, Femoral, Proximal, Left, Distal	0.026614489	0.105271674	-0.140896236	0.194125215	3	0.505634387	0.647924931
Low Dose	Day 43	Nerve, Femoral, Distal, Right, Distal - Nerve, Femoral, Proximal, Left, Proximal	0.098468076	0.232879707	-0.272095506	0.469031658	3	0.845656131	0.459858227
Low Dose	Day 43	Nerve, Femoral, Distal, Right, Distal - Nerve, Femoral, Proximal, Right, Distal	0.028328363	0.123691094	-0.168491769	0.225148496	3	0.458050171	0.678057568
Low Dose	Day 43	Nerve, Femoral, Distal, Right, Distal - Nerve, Femoral, Proximal, Right, Proximal	0.086018164	0.148746835	-0.150671243	0.322707571	3	1.156571359	0.331182919
Low Dose	Day 43	Nerve, Femoral, Distal, Right, Distal - Nerve Median, Distal, Left, Distal	0.153201296	0.076308388	0.031777623	0.274624969	3	4.015319958	0.027729493
Low Dose	Day 43	Nerve, Femoral, Distal, Right, Distal - Nerve Median, Distal, Left, Proximal	0.085109993	0.256987177	-0.323813953	0.494033939	3	0.662367626	0.555075617
Low Dose	Day 43	Nerve, Femoral, Distal, Right, Distal - Nerve Median, Distal, Right, Distal	0.193272608	0.12890106	-0.011837743	0.398382959	3	2.998774528	0.057725223
Low Dose	Day 43	Nerve, Femoral, Distal, Right, Distal - Nerve Median, Distal, Right, Proximal	0.125145973	0.106003779	-0.043529694	0.29382164	3	2.361160602	0.099295438
Low Dose	Day 43	Nerve, Femoral, Distal, Right, Distal - Nerve Median, Proximal, Left, Distal	0.143192706	0.130827839	-0.06498358	0.351368992	3	2.18902502	0.11636057
Low Dose	Day 43	Nerve, Femoral, Distal, Right, Distal - Nerve Median, Proximal, Left, Proximal	0.089795938	0.267365634	-0.335642448	0.515234325	3	0.671708904	0.549868054
Low Dose	Day 43	Nerve, Femoral, Distal, Right, Distal - Nerve Median, Proximal, Right, Distal	-0.004085871	0.308527868	-0.495022558	0.486850816	3	-0.026486237	0.980532864
Low Dose	Day 43	Nerve, Femoral, Distal, Right, Distal - Nerve Median, Proximal, Right, Proximal	0.061337408	0.113606277	-0.11943553	0.242110347	3	1.079824284	0.359294016
Low Dose	Day 43	Nerve, Femoral, Distal, Right, Distal - Nerve, Sciatic, Distal, Left, Distal	0.117288551	0.085041278	-0.018031099	0.252608201	3	2.758391093	0.070246819
Low Dose	Day 43	Nerve, Femoral, Distal, Right, Distal - Nerve, Sciatic, Distal, Left, Proximal	-0.068465374	0.144207891	-0.297932309	0.16100156	3	-0.949537146	0.412399179
Low Dose	Day 43	Nerve, Femoral, Distal, Right, Distal - Nerve, Sciatic, Distal, Right, Distal	0.166723478	0.136887536	-0.051095138	0.384542094	3	2.435919054	0.092837778
Low Dose	Day 43	Nerve, Femoral, Distal, Right, Distal - Nerve, Sciatic, Distal, Right, Proximal	0.112073787	0.226153164	-0.247786364	0.471933939	3	0.991131719	0.394685508
[truncated: 2,280,622 more chars]
